# A goldilocks computational protocol for inhibitor discovery targeting DNA damage responses including replication-repair functions

**DOI:** 10.3389/fmolb.2024.1442267

**Published:** 2024-11-28

**Authors:** Davide Moiani, John A. Tainer

**Affiliations:** ^1^ Department of Molecular and Cellular Oncology, University of Texas MD Anderson Cancer Center, Houston, TX, United States; ^2^ Department of Cancer Biology, University of Texas MD Anderson Cancer Center, Houston, TX, United States; ^3^ Moiani Research Inc., Missouri City, TX, United States; ^4^ Biophysics and Integrated Bioimaging, Lawrence Berkeley National Laboratory, Berkeley, CA, United States

**Keywords:** cancer, DNA repair, DNA damage response, DNA replication, precision oncology, computational pipeline, computational docking, *in silico* testing

## Abstract

While many researchers can design knockdown and knockout methodologies to remove a gene product, this is mainly untrue for new chemical inhibitor designs that empower multifunctional DNA Damage Response (DDR) networks. Here, we present a robust Goldilocks (GL) computational discovery protocol to efficiently innovate inhibitor tools and preclinical drug candidates for cellular and structural biologists without requiring extensive virtual screen (VS) and chemical synthesis expertise. By computationally targeting DDR replication and repair proteins, we exemplify the identification of DDR target sites and compounds to probe cancer biology. Our GL pipeline integrates experimental and predicted structures to efficiently discover leads, allowing early-structure and early-testing (ESET) experiments by many laboratories. By employing an efficient VS protocol to examine protein-protein interfaces (PPIs) and allosteric interactions, we identify ligand binding sites beyond active sites, leveraging *in silico* advances for molecular docking and modeling to screen PPIs and multiple targets. A diverse 3,174 compound ESET library combines Diamond Light Source DSI-poised, Protein Data Bank fragments, and FDA-approved drugs to span relevant chemotypes and facilitate downstream hit evaluation efficiency for academic laboratories. Two VS per library and multiple ranked ligand binding poses enable target testing for several DDR targets. This GL library and protocol can thus strategically probe multiple DDR network targets and identify readily available compounds for early structural and activity testing to overcome bottlenecks that can limit timely breakthrough drug discoveries. By testing accessible compounds to dissect multi-functional DDRs and suggesting inhibitor mechanisms from initial docking, the GL approach may enable more groups to help accelerate discovery, suggest new sites and compounds for challenging targets including emerging biothreats and advance cancer biology for future precision medicine clinical trials.

## 1 Introduction

Cost and efficiency bottlenecks can limit breakthrough drug discoveries and divert efforts toward repurposing or improving existing cancer drugs (Weth et al., 2024). Yet, cancer heterogeneity and fast mutation rates make therapeutic resistance a major medical challenge, which compels oncology researchers to seek innovative solutions for new precision chemotherapeutics. As part of these efforts, the DNA Damage Response (DDR) (Pilie et al., 2019; Voutsadakis and Digklia, 2023) involving DNA repair pathways (Voutsadakis and Digklia, 2023; Wang et al., 2021) combined with their interface to DNA replication and transcription (Ye et al., 2024) have emerged as important targets for precision cancer therapy. However, these dynamic networks have numerous different potential protein targets (Brownlie et al., 2023; Choi et al., 2022; Groelly et al., 2023; da Costa et al., 2023; Helleday and Rudd, 2022; Hopkins et al., 2022; Saxena et al., 2024). So, enabling a protocol to successfully evaluate multiple targets and inhibitors may advance actionable mechanistic knowledge of cancer biology and help overcome resistance.

Furthermore, advances in large-scale production of recombinant proteins and their complexes mean that sample availability is often no longer rate-limiting (Gradia et al., 2017). These observations suggest that efficient assessment capable of examining multiple targets with accessible pre-clinical inhibitors that can be structurally tested and validated in cancer cells and models may be a superior strategy to choosing and focusing on a single candidate. Thus, we reason that providing more laboratories with robust, practical computational methods to initiate early structural and activity testing for new chemical inhibitor design may empower multifunctional DDR networks where inhibitors may show different phenotypes than knockout or knockdown strategies that completely remove a multifunction protein component.

To efficiently identify novel inhibitor tools and target sites for early structure and activity testing to help meet cancer resistance challenges, here we present the development and testing for an *in silico* Goldilocks’ (GL) protocol. Compared to canonical methods that rely on extensive HT screening of large compound libraries, GL protocol leverages a compact curated virtual screening (VS) compound library and a straightforward docking protocol with Glide as exemplary accessible docking software. GL novelty includes efficient ESET library use, combining Diamond Light Source DSI-poised, Protein Data Bank fragments, and FDA-approved drugs. ESET compounds were chosen to span relevant chemotypes and be suitable for both structure determinations and cell testing. This GL approach may provide a compact library and a focused and manageable screening process, facilitating early structure and cell testing without requiring extensive synthetic chemistry resources.

GL methodology can harness advanced *in silico* techniques for molecular docking and molecular modeling to explore protein-protein interfaces (PPIs) (Lu et al., 2020; Scott et al., 2016; Tsuihiji et al., 2022) including allosteric interactions and active sites (Nussinov and Tsai, 2013). By looking beyond targeting these protein regions and transition state analogs, researchers can likely explore both larger target sites and greater opportunities to reduce cross-reactivity (Moiani et al., 2018). Thus, the GL protocol aims to efficiently and computationally examine potential inhibitor binding sites and identify ligands suitable for experimental structural and biological testing to ultimately enable the modulation of protein functions within DDR pathways in predetermined ways.

The GL protocol presented here aims to offer three general potential advantages: 1) reduce the need for an extensive experimental screening and medicinal chemistry effort for preclinical target testing, 2) efficiently obtain VS hits suitable for focused experimental tests, and 3) going wider on interactome targets rather than deeper to harness the power of defined genome networks and the many PDB and AF3 structures. The presented GL protocol seamlessly incorporates improving technologies for accurate structure prediction of fold and biomolecular interactions (Abramson et al., 2024) and experimental results. GL results should aid efficient applications of experimental structural advances in X-ray scattering (Rosenberg et al., 2022), Small-Angle Neutron Scattering (SANS) (Adhikari et al., 2023), mass spectrometry (Filandr et al., 2024; Syed et al., 2023), crystallographic fragment screening (Wilson et al., 2021), and cryo-electron microscopy (Duan et al., 2024; Cebi et al., 2024), which can enable experimental measures of flexibility and ligand-binding site interactions.

Despite its smaller size, GL results suggest that the ESET library can identify high-quality hits with strong binding affinities and favorable binding modes. There are certainly limitations for targeting PPIs by FDA-approved compounds. Yet, we found that FDA-approved novobiocin can function as a non-competitive allosteric inhibitor for PolQ helicase, which is now in cancer clinical trials (Syed et al., 2023; Zhou et al., 2021). These and other findings support the inclusion of FDA-approved compounds in our library, as we suggest that “off-target” effects are more common than widely recognized and may uncover valuable therapeutic interactions. Our findings indicate that the ESET library achieves effective enrichment rates, underscoring its efficiency and practicality. Additionally, the ESET library yields a manageable number of false positives that our docking and scoring protocols can efficiently filter out to improve hit quality and facilitate subsequent experimental validations. By providing multiple hits and an accessible chemical library, GL enables early structural and cell testing to offer a viable alternative for researchers with limited resources, broadening access to effective drug discovery methodologies. Notably, the streamlined computational approach and compact library can accommodate multiple individual candidate proteins to infer potential impacts on functional pathways and networks.

Computationally evaluating multiple targets, allostery, and protein-protein Interfaces (PPIs) may help identify optimal targets to control pathway selection, reduce resistance, and encompass potential metabolic-epigenetic axes (Shibata et al., 2014). Specifically, we tested the GL protocol to examine emerging cancer targets within the DDR (O’Connor, 2015), including its interface with the immune response (Lee et al., 2024; Ye et al., 2024; Yuan et al., 2022; Ye et al., 2021a; Hou et al., 2020; Brosey et al., 2017), and replication (Syed and Tainer, 2018; Ye et al., 2024; Longo et al., 2023). These results suggest target sites and chemotypes for tool inhibitors and potential precision cancer drugs to control phenotypes and create synthetic lethality in cancer cells (Setton et al., 2021). In fact, this GL protocol generally empowers rapid initial inhibitor identification making it suitable for timely coordinated responses to address emerging pathogens and biothreats as well as cancer. The GL pipeline thus aims to identify chemotypes that can bind to selected sites, laying the groundwork for efficient experimental validation and optimization with structural biophysics and cytology. Ultimately, the GL protocol, coupled with subsequent experimental validation cycles, aspires to empower the design of mechanism-based interventions by many oncology and cancer biology researchers to accelerate the identification of novel agents for lower toxicity and higher precision cancer therapy.

## 2 Materials and methods

This section describes the GL approach, encompassing virtual chemical libraries, target preparation, molecular docking, energy evaluation, molecule selection, binding mode analysis, and chemical analysis. Systematic GL methodology provides the computational platform to identify compounds for efficient biophysical and cytological testing to discover and develop chemical tools and potential cancer therapeutics targeting DDR and Repair pathways.

### 2.1 Virtual chemical libraries

Description and Preparation: A compact virtual chemical library comprising diverse small molecules was compiled to span chemotypes and structural diversity and be suitable for biophysical and cytological testing. GL protocol employs three chemical libraries and the Ligprep utility of the Schrödinger package to generate the virtual assembly for virtual screening (Sastry et al., 2013; Schrodinger, 2017; Lu et al., 2021). We term the combined library as the Early Structure-Early Testing (ESET) library, after the Egyptian goddess of healing.

Library I is the DSI-poised developed at Diamond Light Source, United Kingdom (Diamond) and Structural Genomic Consortium, United Kingdom (SGC) in the frame of one of the iNEXT Joint Research Activities constituted by 860 fragments described in the original paper (Cox et al., 2016). Library II contains 203 PDB ligands extracted and filtered from the Protein Data Bank (Revillo Imbernon et al., 2023). Library III is the LH1 (LH1 is the commercial code used by TargetMol for this library selection) FDA-approved drug from the AD part of the Tainer group GLAD library (Moiani et al., 2021). It comprises 2,111 approved FDA drugs, natural products, and chemicals in pre-clinical and clinical studies, as updated in 2020 (Zhu et al., 2021). All three libraries were independently prepared for computational studies using the Moiani et al., 2021 protocol (Moiani et al., 2021).

A novel aspect of our GL protocol comes from the Early Structure-Early Testing (ESET) library, a compact virtual chemical library curated to span chemotypes and structural diversity suitable for biophysical and cytological testing without requiring extensive high-throughput screening resources. The ESET library’s design facilitates the early identification of high-potential compounds, enabling structure and cell testing at an early stage. This approach contrasts with traditional methods that require large libraries and extensive synthetic chemistry, making it accessible to researchers with limited resources.

### 2.2 Target selection and preparation for *in silico* studies

Target Identification: The concept of synthetic lethality comes from functional defects within the DNA Damage Response (DDR) and Repair pathways that are lethal in combination with cancer cell defects. This notion suggests targeting functional defects in pathways or networks rather than focusing on a single protein. Thus, multiple DDR proteins were selected as the focus of this study, including Poly (ADP-ribose) polymerase (PARP) (Zandarashvili et al., 2020), Poly (ADP-ribose) glycohydrolase (PARG) (Houl et al., 2019), Base Excision Repair (BER) proteins (Bacolla et al., 2021; Tsutakawa et al., 2013; Tubbs et al., 2009; Hitomi et al., 2007), and Nucleotide Excision Repair (NER) proteins (Bralic et al., 2023; Yu et al., 2023; Tsutakawa et al., 2020a; Fan et al., 2008), Double-Strand Break Repair (DSBR) proteins (e.g., MRE11-Rad50-Nbs1 complex) (Williams et al., 2008; Rotheneder et al., 2023), and DNA polymerase theta (Pol θ) (Zhou et al., 2021; Syed et al., 2023).

Target Preparation: The three-dimensional structures of selected targets were obtained from experimental databases. Targets without available experimental structures can use homology modeling by AlphaFold 2 or AlphaFold 3. These structures were prepared by removing water molecules and adding hydrogen atoms to ensure proper protonation states. In selected docked complexes, subsequent examination of bound water, atomic hydrophilicity, and protein surface topography may guide chemical optimizations as the addition or removal of a water molecule can inhibit reactions (Kuhn et al., 1992).

### 2.3 PDB structure selection and docking grid generation

For *in silico* analysis, we selected an exemplary series of available protein database (PDB) structures from the Protein structure database (https://www.rcsb.org/), mainly obtained by X-ray, with some using cryo-EM. To prepare for VS, we optimized target structures *in silico* by selecting high-resolution structures from experimental databases and ensuring they were suitable for computational analysis through the Protein Preparation Workflow in the Schrödinger suite (Schrödinger) (Moiani et al., 2021; Moiani et al., 2018).

We selected at least one relevant structure for a protein or complex involved in a DDR pathway of interest. We generated Grid for Docking runs aimed at protein-protein interaction domains, allosteric domains, active sites, and protein-nucleic acid interaction domains.

For PARP1, we chose PDB 7ONR, resolution 2.05 Å (Johannes et al., 2021); for PARG, we used the series of three PDBs presented in the previous methodology development (Moiani et al., 2021): 4B1H A, 4B1H B, resolution 2 Å, 6OAK, resolution 1.7 Å (Houl et al., 2019).

For the base-excision repair (BER) pathway, we selected UDG first for its relevance, as its activity is required to initiate the base-excision repair pathway and remove uracil from DNA (Parikh et al., 2000a; Putnam et al., 1999; Mol et al., 1995b), and second, because we have been studying its structural biology for two decades (Nguyen et al., 2021; Slupphaug et al., 1996; Mol et al., 1995a; Parikh et al., 2000b). The PDB selected for the *in silico* study is 6VBA, with a resolution of 1.8 Å (Nguyen et al., 2021).

For the nucleotide excision repair (NER) pathway, we selected XPD as a pivotal helicase and the lynchpin of the NER complex (Tsutakawa et al., 2020b) that we evaluated previously (Fan et al., 2008; Mui et al., 2011; Tsutakawa et al., 2021; Bralic et al., 2023; Yu et al., 2023). The PDB selected for the *in silico* study is 6RO4 solved at 3.5 Å resolution by cryo-EM (Kokic et al., 2019).

For the DNA double-strand break repair (DSBR) pathway, we evaluated two targets in the signal transduction cascade after our recent publication (Ye et al., 2021b), specifically the interface of Mre11-Nbs1 for the MRN complex and the GRB2 adaptor plastic dimerization site. The region selected for targeting the MRE11 dimer was at the NBS1 contact with the selected PDB code 8BAH solved at 4.13 Å resolution by cryo-EM (Rotheneder et al., 2023). The GRB2 structure selected was the dimeric structure solved at 3.1 Å resolution in PDB code 1GRI (Maignan et al., 1995).

For the cancer-relevant alternative end joining (Atl-EJ) break repair pathway, we selected POLQ (DNA Polymerase Theta), which is a cancer target undergoing clinical trials (Syed et al., 2023; Zhou et al., 2021). For this target, we organized the *in silico* study for two different protein sites: the putative contact of Novobiocin (Hambo Guo, 2023), where Nucleotide binds during polymerase activity, and an allosteric site of the protein. We used two structures: PDB ID 5AGA for the helicase domain at 2.9 Å resolution and PDB ID 8E24 for the catalytic domain at 2.34 Å resolution (Newman et al., 2015; Bubenik et al., 2022).

We generated a docking grid for each target structure with the generation function of the receptor grid in the Schrödinger suite. The grid box was sized to ensure coverage of the entire binding site, with dimensions typically set to 20 × 20 × 20 Å, centered on the binding pocket (Moiani et al., 2021). Table 1 shows the residues and position of the Grid generated for each structure.

**TABLE 1:** List of VS Targets and Grids. All the protein targets (left column) are listed with structure PDB ID (center column), and domain or amino acid are for VS grid (right column).

Target	PDB	ID GRID
PARP1	7ONR	H862
PARG	4B1H A	Y795, F875, F902
PARG	4B1H B	Y795, F875, F902
PARG	6OAK	Y795, F875, F902
UDG	6VBA	Q144
XPD	6RO4	S506, T540
MRE11	8BAH	E92
GRB2	1GRI	R207
POLQ	5AGA	F422, V147, S148
POLQ	8E24	I2362, C2386

### 2.4 Two VS approach and molecular docking parameters

Docking Protocols: Molecular docking studies were conducted using Glide, which is included in the Schrödinger drug discovery suite and employs well-established protocols and parameters (Yang et al., 2021). For each target, both XP (extra precision) and SP (standard precision) docking runs were performed.

Our GL approach combines two virtual screening runs per the target’s Grid. We repeated our approach for each chemical library (see Results). The first VS run in the Virtual Screening workflow section of Glide, in the Docking section, enables the first three parameters: *Enhance planarity of conjugated pi groups, Use Epik state penalties for docking write interaction scores for residues within 12 Å of the grid center.* The scaling factor is 0.8, and the partial charge cutoff is 0.15. At the same time, we enabled Glide HTVS, Glide SP (option generates up to three compounds per state), and Glide XP; we also enabled postprocessing with Prime MM-GBSA. The second VS differed from the previous one because we disabled the Dock with Glide HTVS and Dock with Glide XP capabilities in the same Docking section. The combination of two virtual screening runs per chemical library, and target’s Grid gave us informative analyses for the site evaluated for ligand binding. The first VS (VS1) docking provided a single top-ranked binding pose, while the second VS (VS2) generated multiple ligand binding poses. VS2 was used to refine the initial and only hit from VS1, providing a comprehensive view of potential binding modes. Alternative methods like MD simulations (see protocol in (Moiani et al., 2009), PDBs of VS1 complexes for exploring MD simulations are available in supplementary info) and binding pocket analysis tools can complement this approach. A cutoff of 10% was applied for the second VS results to select the most promising binding poses for further energy ranking analysis.

Energy Evaluation: The binding energies of the selected results of the second VS binding poses were evaluated using appropriate scoring functions and MM-GBSA energetic analysis to assess the strength of ligand-protein interactions, as presented in Moiani et al. (2021).

### 2.5 Ranking top molecules for structural biology techniques

Based on the docking results and energy evaluations, the top-performing molecules, which imply reasonable binding affinity and favorable binding modes, are proposed for future investigation by structural biology techniques and *in vitro* binding assays. Selecting favorable binding modes involved assessing the docking scores, binding affinities, and interaction profiles. Molecules with strong binding affinities and favorable interaction profiles could be prioritized for further investigation. These criteria were used to imply selected compounds with the potential for effective binding and inhibition of the target proteins. The calibration process involved re-ranking based on docking scores and MM-GBSA binding energies. The MM-GBSA calculation is an energy evaluation of complexes at 0 nanoseconds of MD simulation; such energy profile can be extended during validation with MD simulations and calculated at each time to monitor *in silico* binding affinity. The discussion paragraph explains the reasons for the top group and their chemo-diversity. The optimal result from the VS1 was combined with a selection of results from the VS2. Future studies should involve structural biology to reveal density or putative ligand binding and *in vitro* binding assays to confirm binding affinities in the lower μM range, followed by functional assays to test the activity of top-ranked ligands.

### 2.6 Graphical representation combines VS1-VS2 results

The VS1 ranks ligands and gives a single result, representing the absolute minimum in the binding landscape. The addition of the ten percent cutoff of results of VS2 as density reveals the local minimum around the absolute and the possible binding landscape of the same ligand in different conformations or other ligands, effectively probing the site. As shown in the Results, the overlap of all these poses with our graphical analyses outlines the binding surface we employ to optimize with further medicinal chemistry efforts. Allosteric interaction domains and transient protein-protein interfaces during the catalytic mechanism of studied enzymes and complex enzymes can include small or large regions, so this graphical analysis can help define the size and type of chemical tools to be designed and improved.

### 2.7 Binding mode evaluation

Binding modes were scrutinized to assess their potential to interfere with Protein-Protein Interaction (PPI) surfaces and allosteric regions within target proteins to suggest ligands that may disrupt complexes and conformations to regulate protein function. For proteins in macromolecular complexes, the best results aim to define the interaction of the ligand binding mode with proper complex assembly and functional conformational changes during catalytic mechanisms. DNA repair complexes are typically plastic with flexible domains and modular interfaces that can show vulnerabilities during conformational changes (Chinnam et al., 2024a). Binding mode evaluation involved analyzing protein-ligand interactions, conformational changes, and functional impacts using structural and biochemical data. This analysis of the population of specific chemotypes binding specific interfaces can help elucidate mechanisms or other allosteric interactions unknown before.

To validate the protocol, we assessed the enrichment of active compounds during docking, as shown in the PARG and PARP1 examples. These results demonstrate that the docking and ESET library achieves adequate enrichment of hits with a manageable number of false positives. This observation supports using the ESET library for efficient and practical drug discovery.

Our approach aims to detect potential ligand binding modes that interfere with functional mechanisms. The same overlap can be made with nucleic substrates (DNA, RNA, or DNA/RNA hybrid) or other substrates involved in the catalytic functions of our protein targets.

### 2.8 Chemical analysis to suggest chemical optimizations

To suggest further optimization efforts, selected molecules’ chemical properties and interactions were analyzed for hydrogen bonding, hydrophobic interactions, and electrostatic interactions. 2D interactive maps were plotted for the single result of VS1 for each target and library.

### 2.9 Focus library generation guidance

Based on the chemical analysis and binding modes results, the GL approach can suggest design-focused libraries of compounds for each target for commercial or medicinal chemistry collaborators to probe and optimize ligand design and chemical optimization for subsequent studies.

## 3 Results

**FIGURE 1:**
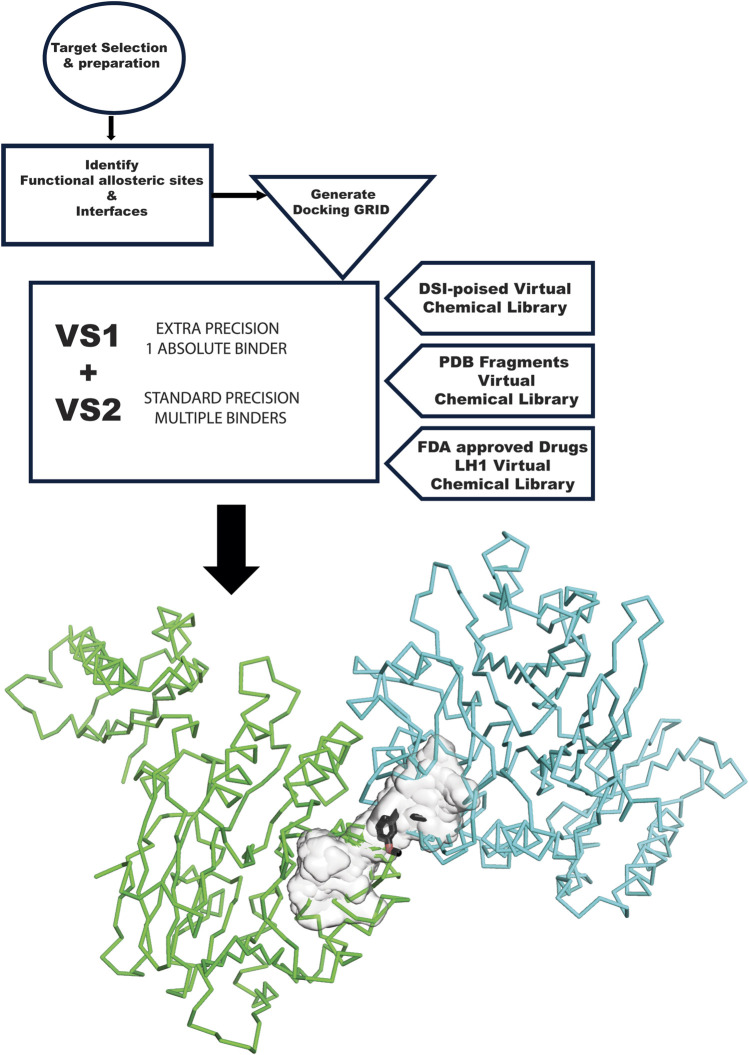
GL VS flow chart. The flow chart starts at the top left, with the target selection and appropriate structure. The structure needs to be prepared for *in silico* experiments. The next step is analyzing the target and the surface of contact with other proteins involved in its complexes and another allosteric site relevant to the mechanism. These sites become the center of the GRID generation. VS1 and VS2 are run with the three libraries used in this study: DSI Poised, PDB Fragments, and FDA-approved drugs LH1. Results are then graphically plotted and evaluated.

The GL computational methodology flow chart (Figure 1) outlines a practical VS protocol that may be assessable to many groups. We illustrate the application of the GL protocol by presenting results for each target in graphic representation, showing the overlap of VS1 and VS2 for each library. The complete chemical characterization of ligands and their ranking are fully described in the Supplementary information for each protein target. The VS1 result provides a ranking of ligands and includes 2D interactive maps. Employing two VS allows users to test if the VS1 single result shows a unique chemotype among targets or if we may have a screening artifact or promiscuous binder to consider as a false positive. VS2 results probing the site are shown as a surface, including the VS1 result (the top binder). Details of each molecule, ranking, and *in silico* affinity are tabulated in Supplementary information.

### 3.1 Targeting PARP1 and PARG *PARylation* and *dePARylation*

**FIGURE 2:**
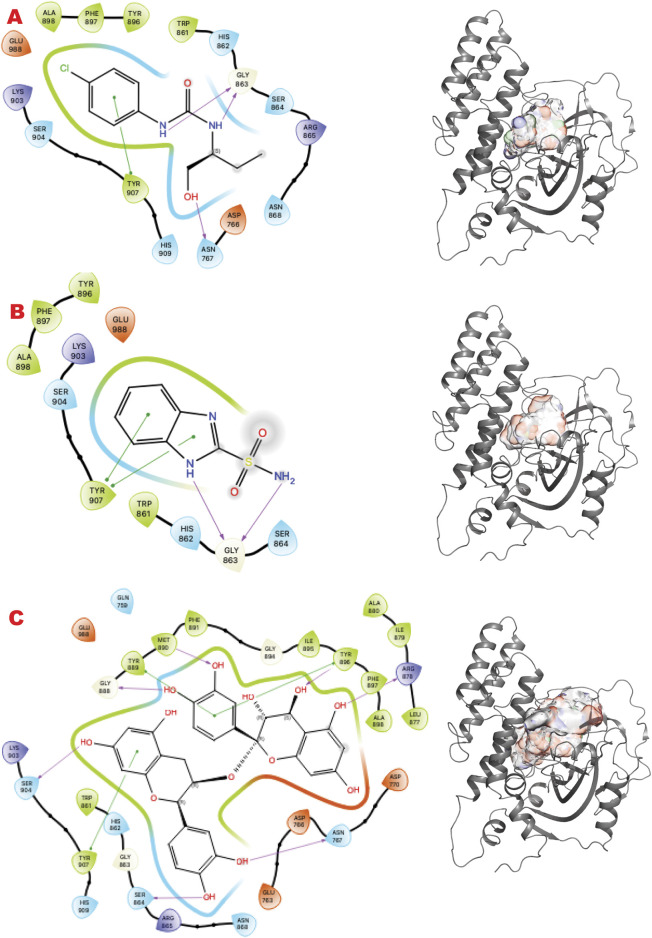
PARP1 VS targeting results. **(A)** VS1 result, from DSI-poised library, from left 2D ligand interactive map of VS1 result, compound 1-(4-chlorophenyl)-3-(1-hydroxybutan-2-yl)urea, right VS2+VS1 results electrostatic maps from DSI-poised library targeting PARP1 catalytic domain; **(B)** VS1 result, from PDB Fragment library, left 2D ligand interactive map of VS1 result, compound 1H-Benzo [d]imidazole-2-sulfonamide-1, right VS2+VS1 results electrostatic maps from PDB Fragment library targeting PARP1 catalytic domain; **(C)** VS1 result from FDA approved drugs library, left 2D ligand interactive map of VS1 result, compound Proanthocyanidins, right VS2+VS1 results electrostatic maps from FDA approved drugs library targeting PARP1 catalytic domain.

PARP1 has been successfully targeted with four approved drugs, and new-generation drugs are in clinical trials. Our *in silico* results re-explore chemotypes for binding to the active site area (Figure 2). Specifically, we start with the 2D interactive map of the VS1 unique binder, analyzing the type of chemo-group and interaction with specific residue around the aimed grid. On the right side, the surface generated by the multiple VS2 results, which includes the VS1 result, shows where the minimum energy binder can grow the unique binder in multiple directions. Such a view has been repeated for the results of the three ESET component libraries (see the supplementary information for details on VS2 targeting PARP1).

**FIGURE 3:**
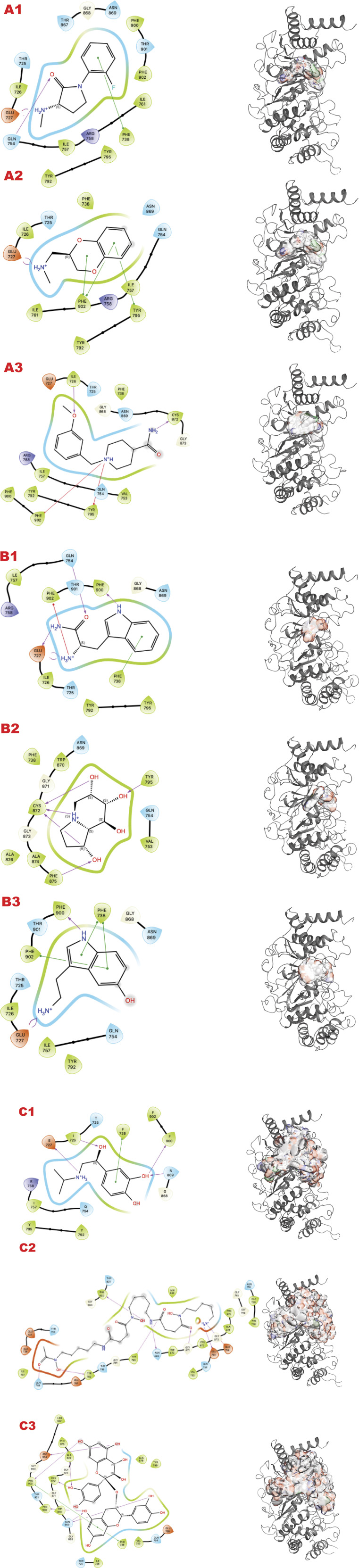
PARG VS targeting results for two structures: 4B1H_A, 4B1H_B, 6OAK. Panel A: DSI-poised library results, Panel B: PDB: PDB Fragment library results, Panel C: LH1 FDA-approved drugs library results. **(A1)** VS1 result, from DSI-poised library, from left 2D ligand interactive map of VS1 results, compound: 1-(2-fluorophenyl)-3-(methylamino)pyrrolidin-2-one hydrochloride; right VS2+VS1 results in electrostatic maps targeting PARG catalytic 4B1H_A domain; **(A2)** VS1 result, from DSI-poised library, from left 2D ligand interactive map of VS1 results, compound: [(2,3-dihydro-1,4-benzodioxin-2-yl)methyl](methyl)amine; right VS2+VS1 results in electrostatic maps targeting PARG catalytic 4B1H_B domain; **(A3)** VS1 result, from DSI-poised library, from left 2D ligand interactive map of VS1 results, compound: 1-[(3-methoxyphenyl)methyl]piperidine-4-carboxamide; right VS2+VS1 results in electrostatic maps targeting PARG catalytic 6OAK domain; **(B1)** VS1 result, from PDB-fragments library, from left 2D ligand interactive map of VS1 results, compound: L-tryptophanamide-2; right VS2+VS1 results in electrostatic maps targeting PARG catalytic 4B1H_A domain; **(B2)** VS1 result, from PDB-fragments library, from left 2D ligand interactive map of VS1 results, compound: indolizidine-1,6,7,8-tetrol; right VS2+VS1 results in electrostatic maps targeting PARG catalytic 4B1H_B domain; **(B3)** VS1 result, from PDB-fragments library, from left 2D ligand interactive map of VS1 results, compound: Serotonin; right VS2+VS1 results in electrostatic maps targeting PARG catalytic 6OAK domain; **(C1)** VS1 result, from LH1 FDA-approved drugs library, from left 2D ligand interactive map of VS1 results, compound: Isoprenaline hydrochloride; right VS2 results in electrostatic maps targeting PARG catalytic 4B1H_A domain; **(C2)** VS1 result, from LH1 FDA-approved drugs library, left 2D ligand interactive map of VS1 results, compound: Deferoxamine Mesylate; right VS2+VS1 results in electrostatic maps targeting PARG catalytic 4B1H_B domain; **(C3)** VS1 result, from LH1 FDA-approved drugs library, from left 2D ligand interactive map of VS1 results, compound: Proanthocyanidins; right VS2+VS1 results in electrostatic maps targeting PARG catalytic 6OAK domain.

In the human genome, the Glycohydrolase PARG is the only homolog of its class for dePARylation, and its singularity makes it attractive for specificity. PARG is an emerging target with a few compounds in early-stage clinical trials, and compounds form our group in preclinical testing (Brosey et al., 2021; Houl et al., 2019; Moiani et al., 2021). Here, we demonstrate our methodology for improving chemical scaffolds for toxicity or other aspects, although toxicity testing was not performed in this study. In Figure 3 (panels A, B, and C), we present respectively the results for PARG structure 4B1H_A (panel A), 4B1H_B (panel B), and 6OAK (panel C). In this case, we used three structures for the same target, focusing on a conformational change of an aromatic residue in the catalytic domain and the effect on the *in silico* results (the reasons were explained in (Moiani et al., 2021)). The three panels show results for the three different libraries (A) DSI-poised (B) PDB fragments, (C) LH1 FDA-approved drugs. Each panel has three rows: 1) representing the result for PARG structure 4B1H_A, 2) for PARG structure 4B1H_B, and 3) for PARG structure 6OAK. Collectively, these unveil the complexity of examining allosteric and transient conformational change and how to feed the *in silico* screening. Considering the same protein target, the same region of GRID, and just changing the orientation of one amino acid, which is crucial for the mechanism, the results of the three panels are different for the same libraries and protocols.

We analyzed the enrichment of active compounds for PARP1 and PARG, demonstrating the protocol’s effectiveness in identifying high-potential hits. Specifically, our findings highlight the manageable false positive rates and the quality of hits suitable for further experimental validation.

#### 3.1.1 Analysis of enrichment of active compounds for PARP1 and PARG

We analyzed the PDB fragment results for all three PARG target structures and PARP1 to validate the enrichment. We observed that results of PARG_4B1H_B, PARG_6OAK, and PARP1 showed at least thirty percent or more of the results are redundant with the compound octahydroindolizine-1,6,7,8-tetrol (except for PARG_4B1H_A where this compound has not been found due to a conformational block in the binding site). We identify such compound as false a positive for a molecular hit, though we consider his chemotype as a hint in the probing mode. In fact, this similarity to the sugar moiety helps analyze binding modes for putative rational substrates with sugar moieties that are part of the polynucleotide. Analyzing the top specific hit for PARG and PARP1, we found two clear families of rational binders: tryptophan-related compounds (Melatonin and Serotonin) and tyrosine-related compounds (Tyrosinamide and Resveratrol). Those compounds are related to specific neurotransmitters, Serotonin, and Dopamine, which are involved in the Circadian clock (Korshunov et al., 2017; Daut and Fonken, 2019; Morin, 1999), as well as the PARP1 (Yang et al., 2023). The rational enrichment of specific metabolites in the results from 200 down to 5 helps to probe the site, elucidate possible binders, and exclude others (Groocock et al., 2014).

### 3.2 UDG BER initiator

Targeting UDG is relevant to controlling the BER pathway in coordination with the APE1 endonuclease and is crucial in immunology and cancer immunotherapy (Mol et al., 2000; Parikh et al., 2000a; Slupphaug et al., 1996). Our group defined inhibition mediated by promiscuous ligand, ATA (Nguyen et al., 2021), and its implications. Here, we revisit the UDG site where the uracil binds and releases apurinic bases containing DNA to understand and find better and more specific chemical binders.

**FIGURE 4:**
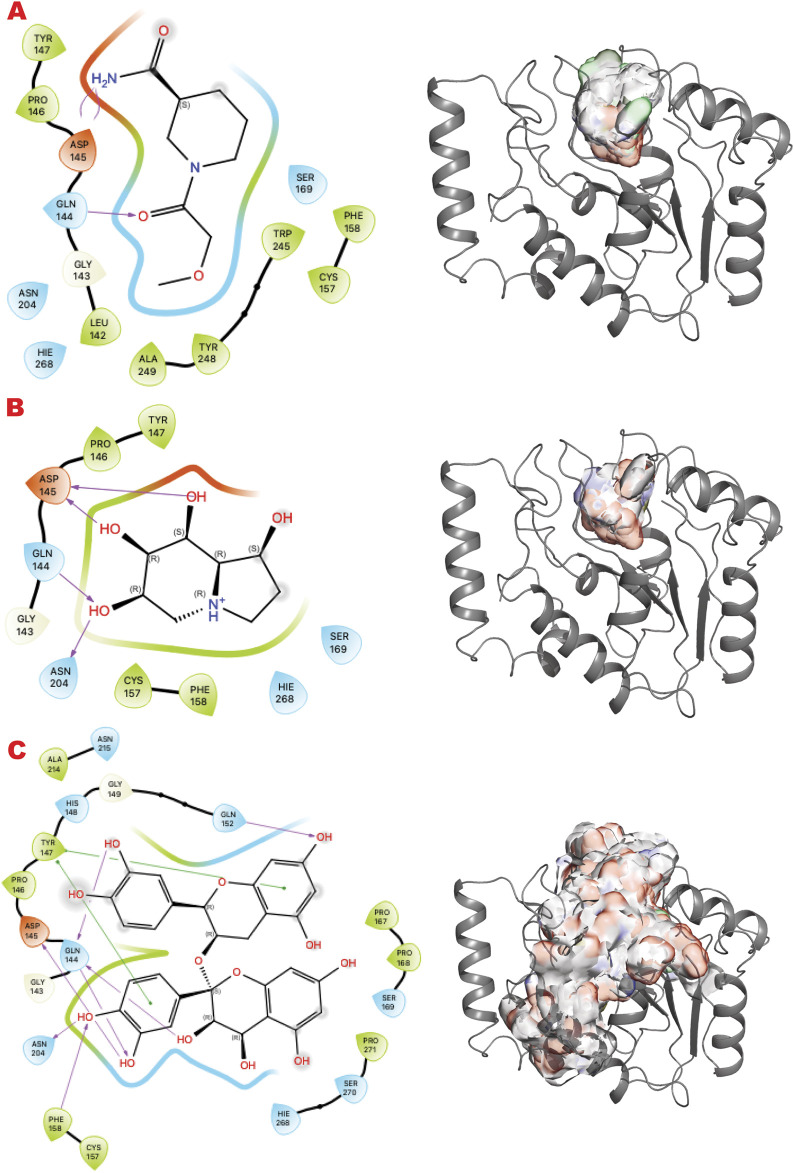
UDG VS targeting results. **(A)** VS1 result, from DSI-poised library, from left 2D ligand interactive map of VS1 result, compound 1-(2-methoxyacetyl)piperidine-3-carboxamide, right VS2+VS1 results electrostatic maps from DSI-poised library targeting UDG catalytic domain; **(B)** VS1 result, from PDB-fragments library, from left 2D ligand interactive map of VS1 result, compound octahydroindolizine-1,6,7,8-tetrol, right VS2+VS1 results electrostatic maps from PDB-fragments library targeting UDG catalytic domain; **(C)** VS1 result, from LH1 FDA-approved drugs library, from left 2D ligand interactive map of VS1 result, compound Proanthocyanidins, right VS2+VS1 results electrostatic maps from LH1 FDA-approved drugs library targeting UDG catalytic domain.

Figure 4 shows the results of the GL *in silico* methods applied in three rows. In the same order of representation for VS1 2D, and VS2 surfaces that include VS1 absolute binder, a clear binding mode in the uracil recognizing pocket emerges for all three libraries: a polar fragment with no specific orientation. This explains the previously characterized ATA, a polar ligand with three equal units that bind without specificity. In this case, directionality could be given by mimicking the ssDNA or dsDNA binding mode of the substrate of the UDG. Such observations may help to further design binders for more specificity and better functionality in targeting the enzyme’s role in initiating the BER pathway.

### 3.3 Targeting XPD as a pivotal NER enzyme

**FIGURE 5:**
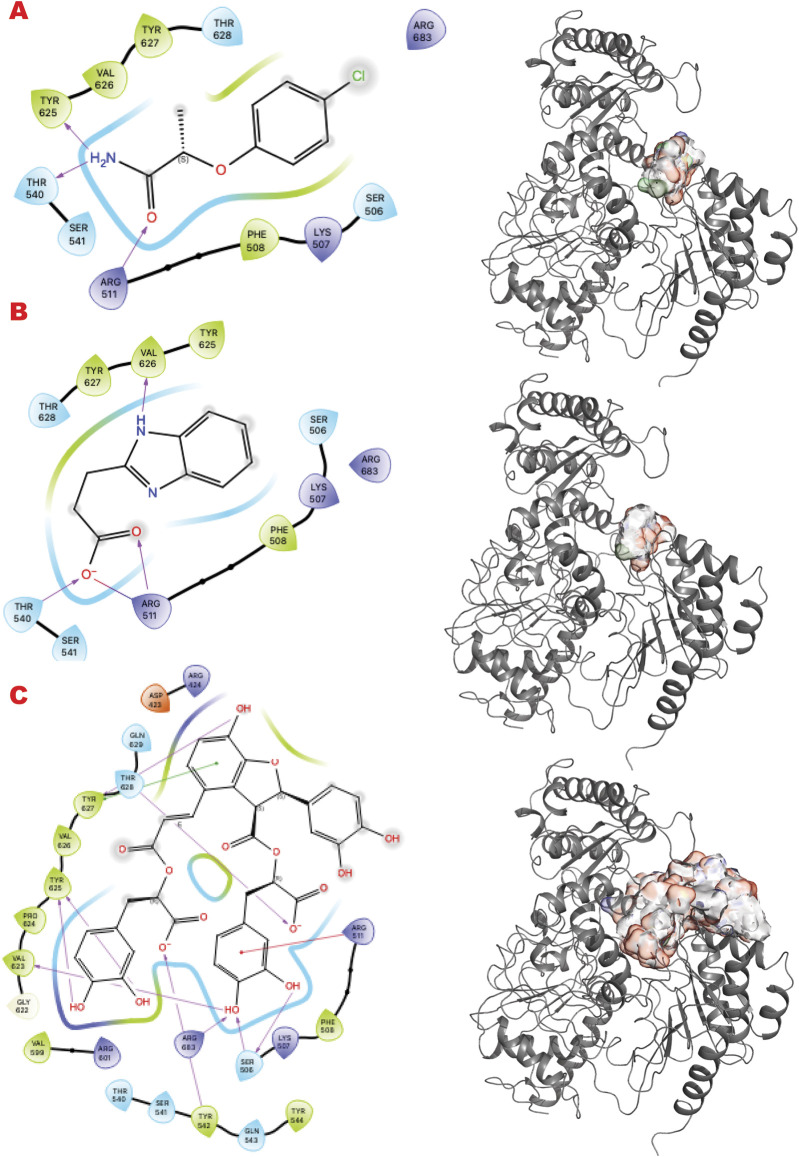
XPD VS targeting results. **(A)** VS1 result, from DSI-poised library, from left 2D ligand interactive map of VS1 result, compound 2-(4-chlorophenoxy)propenamide, right VS2+VS1 results electrostatic maps from DSI-poised library targeting XPD helicase domain; **(B)** VS1 result, from PDB-fragments library, from left 2D ligand interactive map of VS1 result, compound Procodazole, right VS2+VS1 results electrostatic maps from PDB-fragments library targeting XPD helicase domain; **(C)** VS1 result, from LH1 FDA-approved drugs library, from left 2D ligand interactive map of VS1 result, compound Salvianolic Acid B, right VS2+VS1 results electrostatic maps from LH1 FDA-approved drugs library targeting XPD helicase domain.

XPD and its helicase activity are essential in the initial damage verification step of the TFIH complex (Bralic et al., 2023). Targeting the narrowest point where DNA binds first may suppress TFIIH activity and the NER pathway. Figure 5 shows the results of the *in silico* methods applied in three rows. Specifically, the first row represents the results of the DSI Poised library targeting XPD helicase: (left) the result of VS1 shown using 2D ligand interaction map, (right) VS2 results that include VS1 absolute binder, and the sum of the electrostatic surface of all ligands evaluated represents such results. In the same order, the second row shows the results of the PDB fragments library targeting the XPD helicase domain. In the same order, the third row shows the LH1 FDA-approved drug library results targeting the XPD helicase domain. The site targeted is involved in DNA binding.

Interestingly, the VS1 binder in the first row mimics a tyrosine and a phosphotyrosine and may also reveal a phosphotyrosyl DNA-adduct binding site. Learning from top binders’ chemotype can suggest future ligand customization for better preclinical studies.

### 3.4 Targeting MRE11 dimer and NBS1 interfaces to control DSB repair

MRE11, part of the MRE11-RAD50-NBS1 (MRN) complex, participates in multiple DNA Repair pathways (Syed and Tainer, 2018). Here, we sought to target the MRE11-NBS1 interface that regulates the symmetric/asymmetric dimeric conformation of MRE11-RAD50, which is crucial for mechanism and pathway selection (Shibata et al., 2014; Moiani et al., 2018).

**FIGURE 6:**
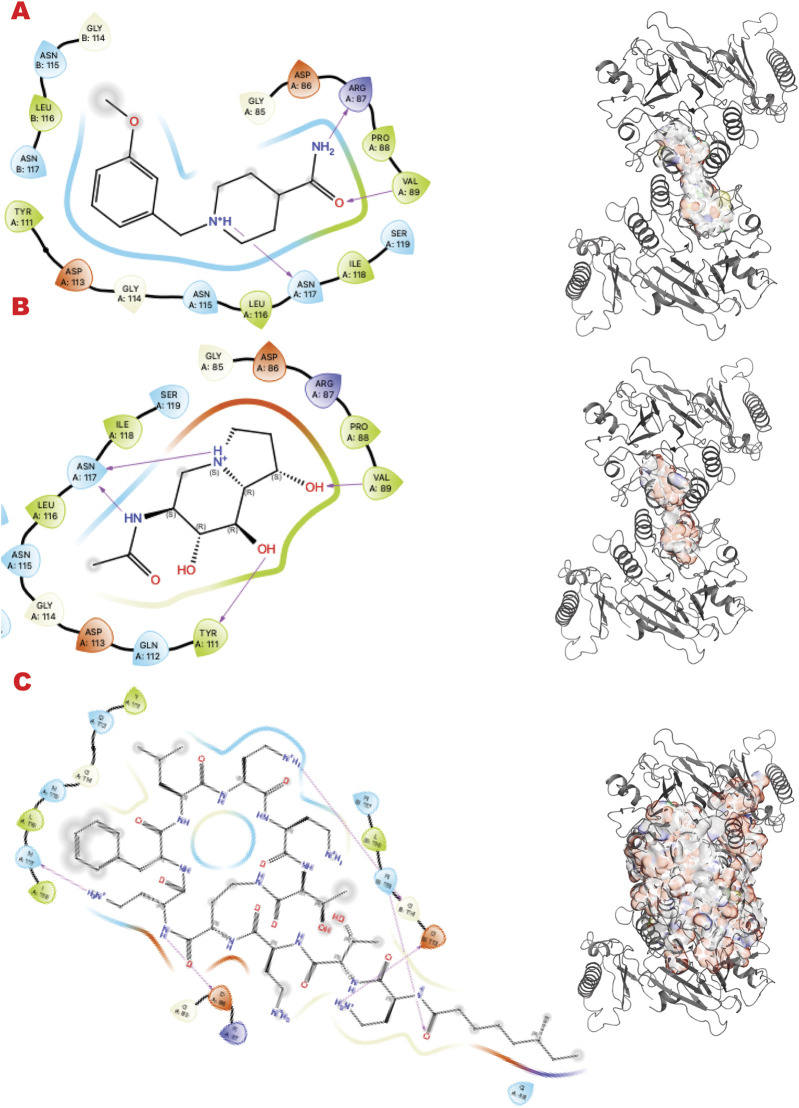
MRE11 dimer VS targeting results. **(A)** VS1 result, from DSI-poised library, from left 2D ligand interactive map of VS1 result, compound 1-[(3-methoxyphenyl)methyl]piperidine-4-carboxamide, right VS2+VS1 results electrostatic maps from DSI-poised library targeting MRE11 dimer; **(B)** VS1 result, from PDB-fragments library, from left 2D ligand interactive map of VS1 result, compound 6-acetamido-6-Deoxy-Castanospermine, right VS2+VS1 results electrostatic maps from PDB-fragments library targeting MRE11 dimer; **(C)** VS1 result, from LH1 FDA-approved drugs library, from left 2D ligand interactive map of VS1 result, compound Polymyxin B sulfate, right VS2+VS1 results electrostatic maps from LH1 FDA-approved drugs library targeting MRE11 dimer.

Figure 6 shows the results of the *in silico* methods applied in three rows. Specifically, from the VS1 results of the three libraries, the chemistry that should outcompete the NBS1 binding has some characteristics already explored by previous ligands from the PFM analogs. The DSI-poised top binder is a chemotype that combines an aromatic cycle and a non-aromatic heterocycle connected by carbon, which gives flexibility to the two units. The complex FDA drug that is indeed a cyclopeptide has a termination as ILE, as has been explored in the MRE11 endonuclease inhibitors. All observed dockings are at the interface of the MRE11 dimer and may impact symmetric or asymmetric dimerization.

### 3.5 Stabilizing inactive GRB2 adaptor to control DNA break repair

GRB2 is a plastic adapter of signal transduction that is active as a monomer and inactive as a dimer (Ye et al., 2021b; Ye et al., 2024; Ahmed et al., 2015). Although GRB2 has been studied for decades, attempts to find small molecules to control its activity have yet to reach clinical evaluation. Its participation in signaling in the DDR pathway connected to the MRN complex recently suggested new ways to modulate its functions and activities. Here, our results identify sites and chemotypes for potential modulators.

**FIGURE 7:**
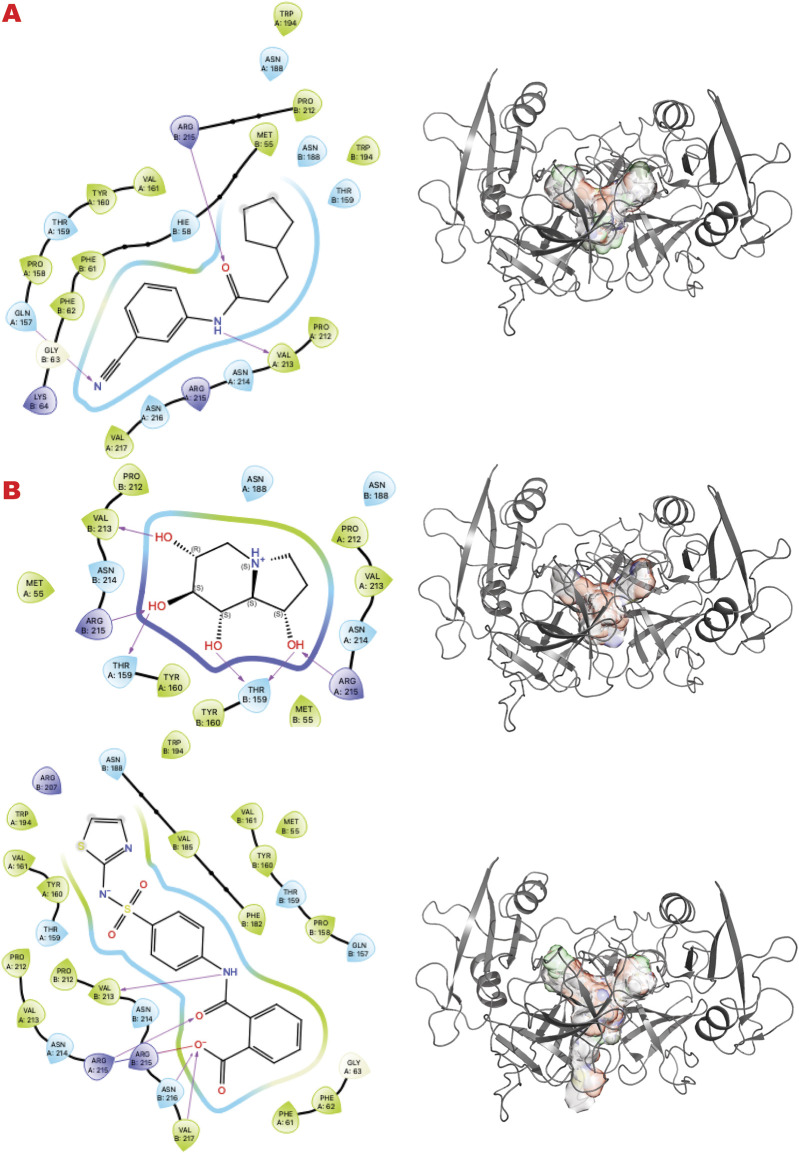
GRB2 dimer VS targeting results. **(A)** VS1 result, from DSI-poised library, from left 2D ligand interactive map of VS1 result, compound N-(3-cyanophenyl)-3-cyclopentylpropanamide, right VS2+VS1 results electrostatic maps from DSI-poised library targeting GRB2 dimer; **(B)** VS1 result, from PDB-fragments library, from left 2D ligand interactive map of VS1 result, compound octahydroindolizine-1,6,7,8-tetrol, right VS2+VS1 results electrostatic maps from PDB-fragments library targeting GRB2 dimer; **(C)** VS1 result, from LH1 FDA-approved drugs library, from left 2D ligand interactive map of VS1 result, compound Phthalylsulfathiazole, right VS2+VS1 results electrostatic maps from LH1 FDA-approved drugs library targeting GRB2 dimer.

Figure 7 shows the results of the GL *in silico* methods applied in three rows. Specifically, the cavity created internally by such dimerization only allows a specific chemical and sterical configuration, as the VS1 ligands show. A combination of multi-fragment chemicals, such as three aromatic connected in the results of the FDA-approved drug or an aromatic bridged to a nonaromatic ring by a urea moiety, can usually be nucleotides or polynucleotides or vitamin-based cofactors.

### 3.6 Controlling cancer dependency on microhomology end-joining by targeting POLQ helicase-polymerase

**FIGURE 8:**
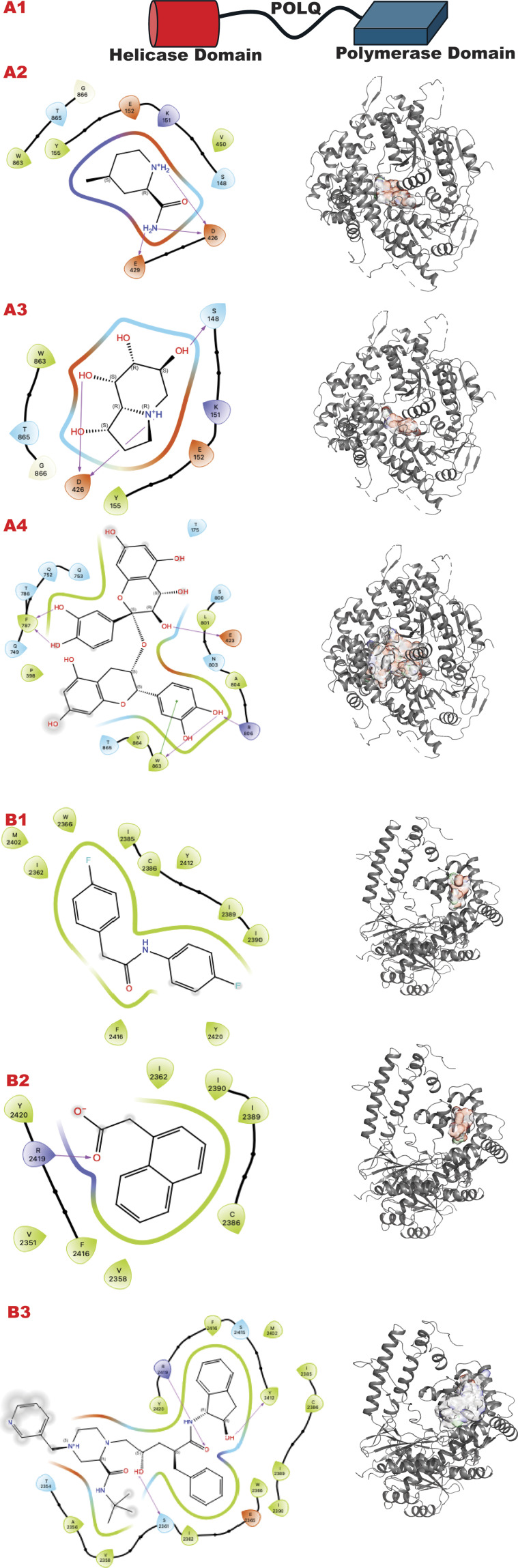
POLQ VS targeting results for its two different functional domains. Panel A. **(A1)** Schematic representation of POLQ and its functional domains; **(A2)** VS1 result, from DSI-poised library, from left 2D ligand interactive map of VS1 result, compound 4-methylpiperidine-2-carboxamide, right VS2+VS1 results electrostatic maps from DSI-poised library targeting POLQ helicase domain; **(A3)** VS1 result, from PDB-fragments library, from left 2D ligand interactive map of VS1 result, compound octahydroindolizine-1,6,7,8-tetrol, right VS2+VS1 results electrostatic maps from PDB-fragments library targeting POLQ helicase domain; **(A4)** VS1 result, from LH1 FDA-approved drugs library, from left 2D ligand interactive map of VS1 result, compound Proanthocyanidins, right VS2+VS1 results electrostatic maps from LH1 FDA-approved drugs library targeting POLQ helicase domain. Panel B. **(B1)** VS1 result, from DSI-poised library, from left 2D ligand interactive map of VS1 result, compound N,2-bis(4-fluorophenyl)acetamide, right VS2+VS1 results electrostatic maps from DSI-poised library targeting POLQ allosteric domain; **(B2)** VS1 result, from PDB-fragments library, from left 2D ligand interactive map of VS1 result, compound2-(1-naphthyl)acetic acid, right VS2+VS1 results electrostatic maps from PDB-fragments library targeting POLQ allosteric domain; **(B3)** VS1 result, from LH1 FDA-approved drugs library, from left 2D ligand interactive map of VS1 result, compound Indinavir sulfate, right VS2+VS1 results electrostatic maps from LH1 FDA-approved drugs library targeting POLQ allosteric domain.

The POLQ enzyme comprises three principal domains, as shown in Figure 8 panel A: the Helicase domain, a central linker domain, and the polymerase domain. PolQ acts in Alt-EJ and replication fork restart (Syed et al., 2023). Our strategy aims at two targets using the GL *in silico* methodology. A) The helicase domain where Novobiocin (Newman et al., 2015; Syed et al., 2023; Zhou et al., 2021) is implicated to bind as an allosteric inhibitor of ATPase activation. This site still lacks a high-resolution structure in literature but evidently blocks DNA binding and ATPase stimulation. B) An allosteric site in the polymerase domain (Bubenik et al., 2022).

Figure 8 (panels A and B) shows the results of the GL silico methods applied to the two grids of POLQ as seen in the scheme in A1: specifically, panel A shows results for the POLQ helicase domain, and panel B for the POLQ allosteric domain. Figure 8, panels A and B show a clear difference in chemotypes targeting the two domains. Specifically, panel A shows a chemotype that we have similarly explored in another target while aiming at a region where polynucleotides bind, which could help us control the function of the helicase domain of POLQ. The other chemotypes targeting the allosteric site behind the ATPase region show clear chemotypes with aromatic rings or two condensed rings that can properly bind the flexible site’s hydrophobic site. An analysis of the surfaces of the VS2 can help to understand the area where this ligand can be expanded, making them more specific for the target and targeted region.

## 4 Discussion

There is a significant recent shift towards embracing computational technologies in academia and pharma. Furthermore, the computational timeline (2–12 months) is favorable compared to longer standard times for gene-to-lead discovery (4–6 years) (Sadybekov and Katritch, 2023). We therefore developed and evaluated an efficient computational approach for a cancer hallmark and potential Achilles’ heel for therapy: genomic instability that depends upon DNA damage responses in the context of stresses from non-B-DNA, replication, transcription, and mitochondrial dysfunction including changes to the epigenetic-metabolic regulatory axis plus the development of apoptotic resistance and metastasis (Bacolla et al., 2019; Bacolla et al., 2016; Das et al., 2024; Singh et al., 2020; Provasek et al., 2024; Fu et al., 2024; Tsutakawa et al., 2020a; Tsutakawa et al., 2017; Tsutakawa et al., 2011). These DDR targets tend to be hard-to-drug, dynamic proteins, and multi-functional complexes that would benefit from approaches that address both these challenges and the opportunities from advances in both protein preparation (Gradia et al., 2017) and structural biology (Fraser and Murcko, 2024; Dyer et al., 2014; Kuhlbrandt, 2014). What may be critical bottlenecks in structure-based drug design for DDR?.

For academic research or biotech teams interested in obtaining inhibitor tools for actionable knowledge of biology and mechanisms for preclinical drug research, three main problem areas may be chemical synthesis, structure-based optimizations, and biological testing. For example, although a vast library size and thousands of virtual hits may be the most pragmatic area to optimize, doing so may not improve success if it comes at the cost of making the other areas more difficult. While larger libraries can yield more hits, our compact library approach focuses on quality (its small size aids accurate curation) and efficiency, aiming to identify high-potential compounds suitable for early structural and activity testing and allowing for further development.

The docking score improves with library size, reflecting that libraries are still far smaller than parameter space. As a result, molecular libraries for virtual screening have increased from 3.5 million to over 29 billion compounds. Yet, for huge libraries, hits chosen from among the top-ranking molecules can become dominated by artifacts and incorrect tautomerization (Lyu et al., 2023). Even ignoring this problem, superior initial hits from vast libraries come at the cost of doing more chemistry, challenging curation, structural biology, and biological assays to distinguish biologically valuable hits. The resources of most research teams generally cannot be arbitrarily expanded to curate huge libraries, synthesize vast numbers of hits, process the many synthesized hit structures, or do thousands of biological assays in multiple animals or cell types. We find that even relatively compact academic libraries can be sufficient for identifying binders with the potential to control allostery and provide ligands for X-ray structures for further optimizations, pending experimental verification (Brosey et al., 2024).

Furthermore, we know that flexibility impacts binding even for protein-antibody recognition (Tainer et al., 1984), that stable inactive complexes can divert functional pathways (Shibata et al., 2014; Tubbs et al., 2009), and that expanding flexible inhibitor pockets can improve specificity and affinity for otherwise undruggable targets (Garcin et al., 2008) What is the most valuable *in silico* approach for preclinical drug discovery involving DDR? We argue that it may be a protocol facilitating the validation of multiple target sites with knowledge of mechanism and biological activity supported by structures and tool compounds to probe target sites and to dissect multi-functionality.

Preclinical biological assays are best done initially in cell assays and later in animal models–these are usually restricted by practicality to selected compounds, so thousands of hits may not prove valuable for cancer biology. What is potentially more valuable is a strategy and method to identify a limited set of hits suitable for biological testing and optimization. In this way, initial hits may serve as tools to test the target site and the biology of cells. We suggest that our compact ESET library and practical GL may facilitate initial hit identification for subsequent validation in cell-based assays, providing a foundation for future animal studies.

We reason that VS could better enable researchers to efficiently harness advanced and integrative structural techniques, including NMR, X-ray Crystallography, X-ray scattering, mass spectrometry, and Cryo-EM for pharmacologically relevant targets. To do this, starting from compact libraries that may allow early structures without new chemical synthesis for compounds suitable for in-cell and potential future animal testing may be optimal. Furthermore, structural and biological advances encourage looking beyond single targets toward pathways and networks where efficient VS protocols may identify tool compounds to test multiple or pivotal targets for cancer biology and medicine more efficiently than giant library approaches. The *in silico* GL gambit, therefore, involves employing a compact chemical library exemplified by the ESET combined library employed here and two VS levels, providing a ranking of hits (VS1) and a probing of binding sites (VS2). Coupling such compact chemical libraries that are accessible without synthesis to an efficient GL *in silico* pipeline providing an objective ranking of hits and probing of sites may facilitate structure-early processes to interrogate multiple targets and biological networks to test biological outcomes, not enzymes and find pivotal targets. The ESET library comprises fragments and FDA-approved compounds for diverse chemotypes. Fragments provide starting points for optimization, while FDA-approved compounds may offer immediate potential for cell-based testing.

Actionable cancer biology is intrinsically hard to achieve due to the complexity of cells and tissues and the amount of heterogeneity and noise in biological systems. The GL protocol targets may allow researchers to probe multiple proteins to test potential impacts on broader pathways and networks, potentially aiding in understanding complex biological systems.

The GL methodology, therefore, proposes an exemplary test of a new pragmatic computational approach and pipeline. The GL approach uses the compact ESET library to probe novel interfaces and allosteric interactions that inform biology and mechanisms for DDR targets in tumor-relevant cellular pathways, such as seen for PolQ, whose importance is greater in cancer and radiation therapy resistance than in normal cells (Dutta et al., 2017). Thus, The GL methodology aims to facilitate preclinical drug discovery for target and tool development to probe cancer biology and the mechanism for undruggable proteins and multifunctional complexes (out of our protein targets, only PARP1 is a clinically validated target).

### 4.1 Targeting PARylation and dePARylation

Dynamically formed and removed PARylation is a critical post-translational modification controlling the DDR signaling and repair initiation (Han and Tainer, 2002; Houl et al., 2019). Analyzing the PARP1 results, we find that only niraparib of the approved drugs (olaparib, rucaparib, talazoparib, niraparib) was ranked in our top 200 results representing a top 10 percent cutoff. To consider these *in silico* results compared to experimental binding for the specific VS2 of the LH1 FDA-approved drugs for targeting PARP1 catalytic domain, we analyzed 100 percent of results involving ∼ 17000 poses with unique chemotypes or repeated with a difference conformation. Performing this comprehensive analysis of all binding results revealed all four approved drugs in multiple conformational poses, specifically olaparib (4 poses), Rucaparib (12 poses), niraparib (5 poses), and talazoparib (21 poses). The ranking was niraparib best, rucaparib second, olaparib third, and talazoparib fourth.

**FIGURE 9:**
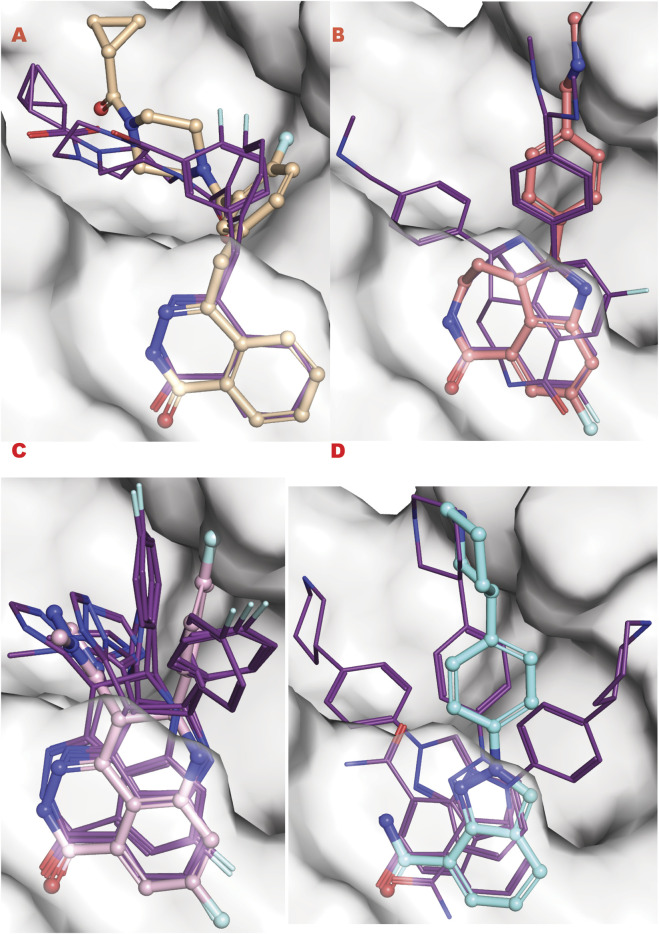
PARP1 approved FDA drugs VS results superposed onto crystallographic results. **(A)** Olaparib from crystal structure PDB ID 5DS3 (ball and sticks) superposed to PARP1 structure targeted by VS2 (PDB ID 7ONR) with best Olaparib *in silico* results bold lines; **(B)** Rucaparib from crystal structure PDB ID 6VKK (ball and sticks) superposed to PARP1 structure targeted by VS2 (PDB ID 7ONR) with best Olaparib *in silico* results bold lines; **(C)** Talazoparib from crystal structure PDB ID 7KK3 (ball and sticks) superposed to PARP1 structure targeted by VS2 (PDB ID 7ONR) with best Olaparib *in silico* results bold lines; **(D)** Niraparib from crystal structure PDB ID 7KK5 (ball and sticks) superposed to PARP1 structure targeted by VS2 (PDB ID 7ONR) with best Olaparib *in silico* results bold lines.

To better understand this behavior versus our VS1 result (which ranks ligands) and other better-ranked ligands, we examined all these poses superimposed with the crystal structure of each ligand bound to the PARP1 catalytic domain (PDB ID: 5DS3 (Olaparib) (Dawicki-McKenna et al., 2015), 6VKK (Rucaparib) (Zandarashvili et al., 2020), 7KK3 (Talazoparib) (Ryan et al., 2021), 7KK5 (Niraparib) (Ryan et al., 2021) (Figure 9). For the four drugs, the representation clearly matches the *in silico* calculation for the multi-aromatic unit in the center of the active site. Yet, for all drugs, the other chemical moiety shows multiple conformations around the crystal structure coordinate, yielding low energy binding poses (see Supplementary information for the complete tabulation of VS2 results). Notably, GL docking fits the approved drugs in multiple poses, with only some close to crystal structure as the energetic minimum. We expect the multiple pose representation partly reflects limitations in adequately assessing entropic impacts in our silico energy evaluations. While crystal structures provide valuable details, they may not always reflect the most biologically relevant solution conformations (Chinnam et al., 2024b), which can impact docking evaluations. Also, different assembly states can be seen in the presence of protein partners as seen for proliferation proteins such as CKS and CKD2 and repair protein complexes (Parge et al., 1993; Bourne et al., 1996; Morris et al., 2002; Hammel et al., 2010). Furthermore, computational predictions are optimized to predict crystal structural conformations, as shown by comprehensive X-ray scattering analyses (Hura et al., 2019). We reason that the correct GL docking of larger drug-sized compounds can have more error than smaller chemical ligands that may better satisfy our predominantly enthalpic energetic evaluation. This notion argues in favor of fitting smaller compounds and chemotypes to reduce multiple pose interaction volumes.

We thus consider our VS2 top results that probe sites as relevant, along with the approved drugs that may rank below them, as seen here. Interestingly, new fragments binding in the catalytic site for our FDA-approved Anna Karenina principle states that one must be successful in all three arenas to avoid failure. This means that although a huge library size and thousands of virtual hits may be the most pragmatic arena to optimize, doing so may come at the cost of making the other areas more difficult.

Our top binder is proanthocyanidin, a natural polyphenol compound with cardiovascular properties. The promiscuity of compounds like tannin and flavonoids provides helpful results for *in silico* screening that may also reflect the biological factor that metabolites, RNA, and other small molecules provide regulation and balance in cells and tissues (Das et al., 2024). To optimize *in silico* preclinical hits, added structure-based and biology-based testing is necessary: this is the premise of the early-structure-early-testing ESET combined library and GL protocol. Furthermore, to propose new strategic ligands for DDR *in vitro* analysis, GL methodology may help find optimal natural compounds that can inform target and biological mechanisms. Such compounds are valuable for having optimal solubility, cell permeability, and low toxicity. Most PARP1-approved drugs after treatment involve resistance mechanisms (Noordermeer and van Attikum, 2019), prompting efforts to find other targets in the BRCA-PARP network, such as MRE11. GRB2, ASCC1, and PARG (Brosey et al., 2021; Houl et al., 2019; Ye et al., 2021b; Ye et al., 2024; Chinnam et al., 2024a).

When we explored potential inhibitors for the deParylation enzyme PARG, we observed that gallotanin was an initially potent inhibitor. This compound is also part of the same class as proanthocyanidin. In GL *in silico* methodology, such compounds populate the top rankings due to the size and type of chemo group involved in the interactions (multiple hydroxyls). The advantage of these compounds is the lower toxicity. They often have limited specificity and potency but can provide useful entry points for structure-based optimizations. Notably, the PARG macrodomain is an important enzymatic domain for many pathogenic RNA viruses, so these analyses are also advancing insights into inhibitors against emerging RNA viral pathogens and biothreats.

### 4.2 Targeting DNA base excision and transferase activities

BER is the critical DNA repair pathway that deals with most DNA base damage, and uracil DNA glycosylase (UDG) acts in DDR and its interface of replication by removing uracil incorporated into DNA from cytidine deamination or misincorporation in duplex DNA and at replication forks (Parikh et al., 2000a). Although a DNA-mimicking protein inhibitor exists for humans and *E. coli* UDG, chemical inhibitors would be highly valuable (Putnam et al., 1999; Mol et al., 1995a). Let’s analyze the top VS1 results targeting the UDG DNA binding active site (Figure 4). These suggest a mimicry of the uracil base from analyzing the top compound of the DSI poised library (Figure 4A). If we observe the top binder from the PDB fragment, the compound octahydroindolizine-1,6,7,8-tetrol-61, which may be a false positive for high recurrence, represents the binding of a sugar moiety like pentose of hexose. One could choose a pentose connected to the uracil ring for this site. It is a lower binder than the uracil product moiety that can remain bound after the enzyme releases the abasic-containing DNA product. The top result for the FDA approval again shows another possible false positive, the Proanthocyanidin. Still, this compound reach mimics the broader binding of a polynucleotide (double or single strands) as the previous target mimicked the binding of a poly-ADP-ribose. A deep analysis of the listed local minimums in specific regions of the targeting Grid from the VS2 run identifies several potential ways to improve the VS1 scaffold and make a compound more specific for the selected target.

We observe analogous chemical pattern results for the XPD DNA binding channel target site. The helicase XPD is an ATP-driven ssDNA transferase that binds and processes ssDNA in the TFIIH complex for the NER pathway; the site we target is the narrowest point where DNA binds first (Yu et al., 2023; Bralic et al., 2023; Yan et al., 2019). Similar pinch points in replication-repair nucleases EXO5, WRN, FEN1, and XPG suggest possible analogous targeting (Hambarde et al., 2021; Tsutakawa et al., 2020a; Tsutakawa et al., 2017). Furthermore, the plasticity and flexibility of these regions resemble the BER and PARG enzymes analyzed in terms of partial pre-organization for binding and recognition of polynucleotides or polyADPribose. As we can see in Figure 5A, the DSI top result is a tyrosine or phosphotyrosine mimic compound, which could lead to the classification of the site as potentially phospho-tyrosine-DNA adduct or just tyrosine phosphorylation or dephosphorylation site connect to DNA Repair mechanism proceeding. The PDB fragment 5B hit again resembles a nucleotide or aromatic chemotype in agreement with the other result. The FDA-approved top result (5C) is again a high molecular weight compound that is very hydrophilic, Desmopressin, a modification of the hormone vasopressin (antidiuretic). The compound has two aromatic amino acid sites, PHE-TYR, adjacent to the top binder of the other fragments for this target.

### 4.3 Targeting the critical adapter GRB2

GRB2 acts in the RAS-Map Tyr kinase signaling pathway for proliferation. It has recently been discovered and characterized for interactions with MRE11 nuclease (MRN complex) as well as the RAD51 recombinase (Ye et al., 2024; Ye et al., 2021b). GRB2 exemplifies a class of adaptors that link cell proliferation to DDR. As such, targeting GRB2 opens a new way to control DDR at the signal transduction level (Ye et al., 2021b). MRE11 and GRB2 have a dimeric form; MRE11 dimerizes at dsDNA binding in active form, while GRB2 dimer is inactive. The *in silico* methodology runs against the dimer structure of both with two different aims: 1) targeting the MRE11-NBS1 interface should reveal the chemistry behind the organization of MRE11 as a symmetrical or asymmetrical dimer induced by NBS1 with repercussions on its activity, 2) targeting GRB2 dimer aims to inhibit GRB2 function and MRE11 interface. Identifying tool compounds for these two ways to interfere with DSBRs should help to test their ability to target tumor cells (Ye et al., 2021b; Ye et al., 2024).

**FIGURE 10:**
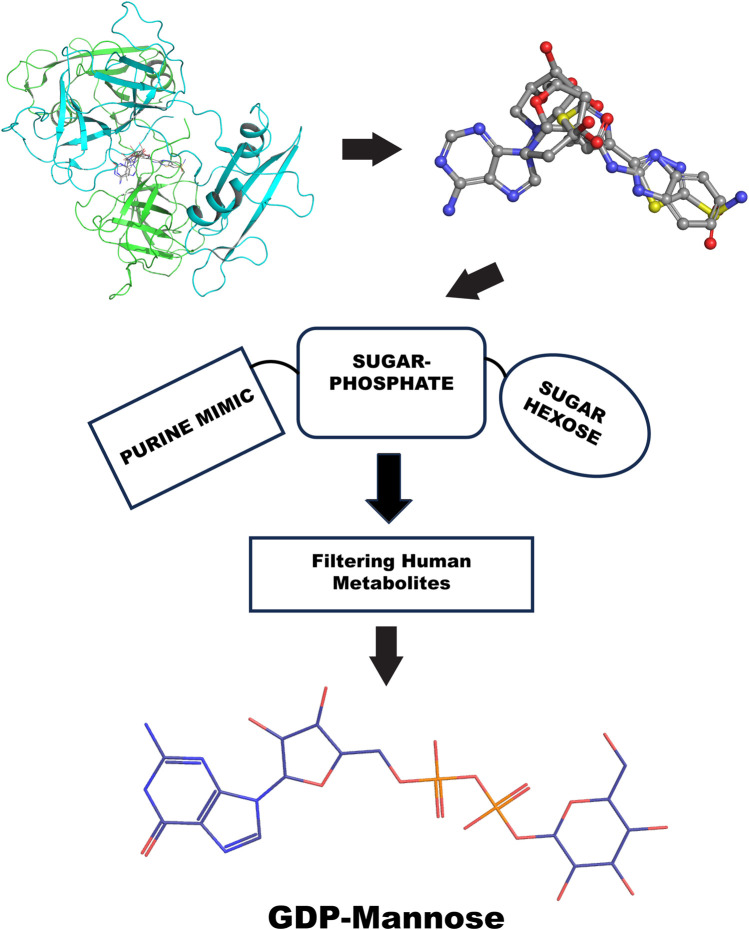
GRB2 potential substrate binding flow chart. The top left shows the top VS2 results of the PDB fragment library targeting the GRB2 dimer interface; the top right superposition of fragments results. The center represents the chemotype scheme of the potential substrate, followed by a screening process through the human metabolome; at the bottom is the potential result, represented by a 2D chemical sketch of GDP-mannose.

Furthermore, the GRB2 analyses suggest a new way to probe substrate binding. The dimeric structure of GRB2 has been challenged as a natural solution dimer structure (Maignan et al., 1995). The plasticity of this adapter in solution is high due to its adaptivity to signal transduction substrate. It is intrinsic in its molecular organization as three domains connected by flexible linkers. We reason that the X-ray crystal structure contains relevant information on the GRB2 inactive form that merits interpretation of what is entrapped in its homodimeric interface. Therefore, we provide a chemical interpretation for combining top results from the PDB fragments library. This illustration shows how a more significant representation can be developed to understand a potential natural substrate that may govern such an inactive form. We present the superposition of top results from the PDB fragment library and how to merge the chemotype to envision a potential substrate binder to keep such inactive dimer form in the cell (Figure 10). In the center, we have the octahydroindolizine-1,6,7,8-tetrol-61 (a false positive) representing sugar (pentose or hexose) and phosphate mimic binding. On the left side, we have a purine base mimic (Adenosyl or Guanosyl). On the ending right side, we have a 6-ring binding with polarity. In this initial evaluation of a possible binding chemotype, the combination hints at a putative substrate like the GDP-mannose related to the GRB2 cellular environment. The next step might be to search a human metabolite database, e.g., Human Metabolome (https://hmdb.ca/).

### 4.4 Targeting pol Q polymerase and helicase sites

The PolQ helicase-polymerase is central to the minor DSBR pathway ATL-EJ, which becomes important for many cancer cells and therapeutic resistance (Eckelmann et al., 2020; Dutta et al., 2017; Syed et al., 2023; Zhou et al., 2021). Targeting the different helicase and polymerase sites of PolQ’s distinct catalytic processes to control a specific cellular and molecular pathway is attractive but challenging. For POLQ we target the helicase site at the DNA binding site where putative Novobiocin binds (Hambo Guo, 2023) and an allosteric site near the Polymerase catalytic site.

Dissecting multiple functions of the same protein with two different chemicals has been proven possible (Shibata et al., 2014), so it can also be applied to polymerase. Here, we observe that the GL results show different chemotypes from the two domain-targeted results (Figure 8, panels A, B). Specifically, the helicase domain site is predisposed to interact with groups, as shown by the top fragment VS1, which ranks ligands. This is typically mimicking polynucleotide binding. The allosteric site allows more hydrophobic binding driven by a double aromatic ring-connected motif. Also, the FDA-approved results confirm the hydrophilicity versus hydrophobicity of the two sites. One challenge will be in the experimental evaluation of the combination of best affinity molecules and results in normal versus tumor cells where PolQ is more highly expressed.

In the VS2 targeting that probes the helicase domain site, novobiocin sodium, which is active against POLQ (Zhou et al., 2021), is ranked within the first 35 compounds (without energy re-ranking); we use energy re-ranking to calibrate novobiocin on top of the list, and we plotted the binding site with 2D map in the supplementary information (Supplementary Figure S1). The experimental validation of this site comes from mass spectrometry (Syed et al., 2023), which is consistent with a BioRkiv preprint (Hambo Guo, 2023). With the energy re-ranking ratio 0.7 (docking score) plus 0.3 (0.1 MMBSA delta G bind), Novobiocin ranked in the top 25 FDA-approved drug compounds.

### 4.5 Limitations of this study

This is a manuscript on the GL computational approach and ESET library. The GL approach thus provides both target probing (to select promising points in pathway networks) and tool inhibitors suitable for optimization due to the chemistry selected for the ESET library. The computational results identify testable tool inhibitors, but these are supposed to be considered hypotheses until tested experimentally. However, experimental tests are noted for earlier versions of the GL protocol in several cited papers. This cited published work with multiple related efforts suggests that the reported GL-type approach can generate inhibitor tools for several systems as validated by both affinity measures and crystal structures. Notably, validation will require new experimental support for newly discovered inhibitors. Furthermore, crystal structures may not always reflect the most biologically relevant solution conformations (Chinnam et al., 2024a). However, they are still a gold standard in drug design and the best starting point for docking and evaluating the VS hits proposed here.

## 5 Future perspectives

Significant advances in the structural biochemistry of DNA repair and replication networks promote an ongoing renaissance in biophysics and molecular biology stimulated by their interface with cancer biology (Zhang et al., 2021). In the context of these experimental and computational advances, we suggest that there remains a lag in novel drug development. Furthermore, we reason that added novelty could come from better enabling structural and biological researchers to employ their knowledge to identify and test tool inhibitors probing fundamental mechanisms and new targets. These observations motivated us to develop an enabling practical GL protocol with two VS protocols (VS1 for ligand ranking and VS2 for site probing) combined with an ESET compact chemical library that may harness more biological expertise to stimulate increasing future breakthroughs. To our knowledge, this is the only VS study targeting multiple DDR pathways and targets that supports its efficiency and ability to consider networks rather than single targets.

Currently, many groups can design knockdown and knockout methodologies to remove a gene product, but relatively few can design the new chemical inhibitor tools needed to dissect multifunctional DDR networks. Large chemical libraries will continue to identify leads for structure-based and biology-based optimizations. However, an alternative GL strategy is to provide a compact library of cell-accessible compounds for efficient VS for early structures and early testing (ESET) on multiple targets. Based upon the exemplary GL VS results presented here, a practical path to obtain promising hits involves the following steps as also supported by independent observations (Zhao, 2024): 1) test a diverse set of fragments that have less flexibility and torsional variation to identify favorable subsite interactions, 2) focus on well-ranked poses for relatively rigid and higher molecular weight compounds, and 3) select compounds available without chemical synthesis and suitable to complement VS rankings with subsequent experimental affinity measurements, activity assays, and structure determinations.

Complex DDR systems challenge canonical approaches that typically focus on a single target (deep VS efforts) due to the efforts required for large libraries and subsequent steps. Here, the notion presented is that the simple, robust GL protocol lets researchers test multiple proteins (go wider for pre-clinical searchers) compared to larger library screens that yield more hits that need more time for each target. We, therefore, envision that the GL approach will allow more researchers to test networks and activities by targeting allostery, interfaces, and active sites. The GL protocol thereby provides an efficient compass to guide biology-driven inhibitor tool development by many groups. Currently, *in silico* researchers often seek to discover functional ligands by running calculations against well-established systems already of interest to Pharma and Biotech teams. The presented GL protocol aims to leverage a basic science approach enabling the evaluation of new experimental and modeled structures and interactions by developing inhibitors that can probe mechanisms and biological differences separating pathways and interactions networks in normal and cancer cells. For example, researchers can apply the ESET library and GL protocol to find inhibitor tools to probe cancer impacts for point mutations and for functionality-implicated variants of unknown significance in pockets and channels revealed by cancer genome sequencing and experimental structures or AlphaFold modeling (Longo et al., 2023; Tsutakawa et al., 2021; Fan et al., 2008; Tsutakawa et al., 2020a). We anticipate that the collective methods and results presented here, which unveil the GL protocol, compact ESET chemical library, and exemplary DDR target sites and compounds, may allow more researchers to probe mechanisms and advance chemical tools for actionable knowledge to develop novel future therapeutic inventions. Viral biothreats are another representative emerging area where the ability to efficiently test multiple targets and dissect network interactions is critically important. Thus, GL-enabled inhibitor results may help address the missing novelty in drug development that is key to precision oncology and likely also to biopreparedness and efficient response capabilities.

## Data Availability

The datasets presented in this study can be found in online repositories. The names of the repository/repositories and accession number(s) can be found in the article/Supplementary Material.
