# Supplementary material for: A goldilocks computational protocol for inhibitor discovery targeting DNA damage responses including replication-repair functions
Source: Front Mol Biosci. 2024 Nov 28;11:1442267. doi: 10.3389/fmolb.2024.1442267 (PMC11635304; doi:10.3389/fmolb.2024.1442267)
Supplement: Supplementary file 1 [file Supplementary_Info.pdf › Supplementary_Info.pdf]

Supplementary Info

Figure S1.

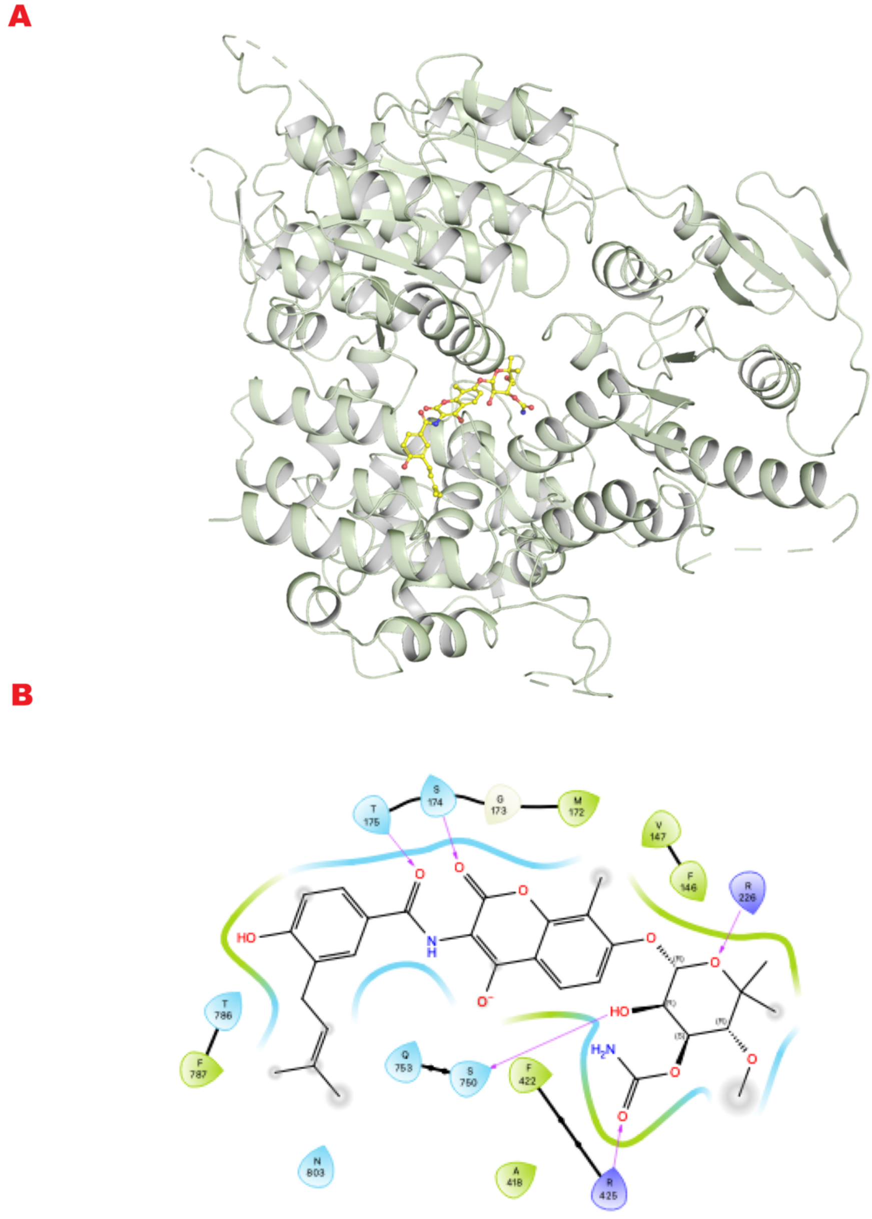

Figure S1. Novobiocin docked to POLQ helicase domain and 2D intercative map of interaction from in silico pose.

Table S1. VS1 and VS2 for DSI-poised targeting PARP1

compound	MW	Catalog ID	Energy in silico
VS1			
DSi PoisedLibrary_1680186639697.662	242.082	Z123852286	-7.5748
VS2			
DSi PoisedLibrary_1680186639697.103	248.321	Z54748186	-7.5388
DSi PoisedLibrary_1680186639697.719	203.069	Z332370018	-7.4484
DSi PoisedLibrary_1680186639697.662	242.082	Z123852286	-7.3369
DSi PoisedLibrary_1680186639697.718	251.280	Z55222357	-7.3026
DSi PoisedLibrary_1680186639697.146	203.106	Z68639747	-6.8719
DSi PoisedLibrary_1680186639697.63	299.729	Z1004825200	-6.8537
DSi PoisedLibrary_1680186639697.388	222.117	Z46169164	-6.8445
DSi PoisedLibrary_1680186639697.103	248.321	Z54748186	-6.7559
DSi PoisedLibrary_1680186639697.45	224.095	Z235449082	-6.7547
DSi PoisedLibrary_1680186639697.718	251.280	Z55222357	-6.7189
DSi PoisedLibrary_1680186639697.45	224.095	Z235449082	-6.7037
DSi PoisedLibrary_1680186639697.121	233.095	Z26333434	-6.6869
DSi PoisedLibrary_1680186639697.36	239.131	Z1470724953	-6.6869
DSi PoisedLibrary_1680186639697.388	222.117	Z46169164	-6.6839
DSi PoisedLibrary_1680186639697.662	242.082	Z123852286	-6.6649
DSi PoisedLibrary_1680186639697.662	242.082	Z123852286	-6.6605
DSi PoisedLibrary_1680186639697.388	222.117	Z46169164	-6.6592
DSi PoisedLibrary_1680186639697.718	251.280	Z55222357	-6.6547
DSi PoisedLibrary_1680186639697.662	242.082	Z123852286	-6.651
DSi PoisedLibrary_1680186639697.705	211.076	Z1127662991	-6.6125
DSi PoisedLibrary_1680186639697.674	233.178	Z54508609	-6.6088
DSi PoisedLibrary_1680186639697.36	239.131	Z1470724953	-6.5879
DSi PoisedLibrary_1680186639697.674	233.178	Z54508609	-6.5803
DSi PoisedLibrary_1680186639697.63	299.729	Z1004825200	-6.5711
DSi PoisedLibrary_1680186639697.536	232.278	Z413792090	-6.5683
DSi PoisedLibrary_1680186639697.457	226.091	Z26333448	-6.5512
DSi PoisedLibrary_1680186639697.436	248.228	Z44586758	-6.5381
DSi PoisedLibrary_1680186639697.396	243.090	Z68195082	-6.5348
DSi PoisedLibrary_1680186639697.396	243.090	Z68195082	-6.5325
DSi PoisedLibrary_1680186639697.399	238.326	Z32399948	-6.5228
DSi PoisedLibrary_1680186639697.137	190.111	Z285141302	-6.5106
DSi PoisedLibrary_1680186639697.769	219.137	Z1509441355	-6.4969
DSi PoisedLibrary_1680186639697.662	242.082	Z123852286	-6.4929
DSi PoisedLibrary_1680186639697.136	234.226	Z44584202	-6.4758
DSi PoisedLibrary_1680186639697.59	223.112	Z198194394	-6.4702
DSi PoisedLibrary_1680186639697.396	243.090	Z68195082	-6.4696

DSi PoisedLibrary_1680186639697.456	199.111	Z992240004	-6.4604
DSi PoisedLibrary_1680186639697.719	203.069	Z332370018	-6.441
DSi PoisedLibrary_1680186639697.628	240.106	Z124039706	-6.427
DSi PoisedLibrary_1680186639697.487	247.293	Z33545544	-6.4161
DSi PoisedLibrary_1680186639697.59	223.112	Z198194394	-6.409
DSi PoisedLibrary_1680186639697.58	207.269	Z1331830630	-6.4088
DSi PoisedLibrary_1680186639697.125	238.087	Z275165822	-6.4033
DSi PoisedLibrary_1680186639697.36	239.131	Z1470724953	-6.4004
DSi PoisedLibrary_1680186639697.61	237.646	Z56259166	-6.3939
DSi PoisedLibrary_1680186639697.657	244.264	Z44609285	-6.3794
DSi PoisedLibrary_1680186639697.259	225.268	Z79432418	-6.358
DSi PoisedLibrary_1680186639697.234	210.046	Z119990900	-6.332
DSi PoisedLibrary_1680186639697.58	207.269	Z1331830630	-6.3304
DSi PoisedLibrary_1680186639697.692	212.095	Z198195774	-6.3282
DSi PoisedLibrary_1680186639697.232	204.126	Z1509140850	-6.3135
DSi PoisedLibrary_1680186639697.63	299.729	Z1004825200	-6.3112
DSi PoisedLibrary_1680186639697.402	237.127	Z57299529	-6.3098
DSi PoisedLibrary_1680186639697.232	204.126	Z1509140850	-6.3095
DSi PoisedLibrary_1680186639697.749	179.095	Z33452106	-6.2994
DSi PoisedLibrary_1680186639697.67	229.110	Z56943440	-6.2966
DSi PoisedLibrary_1680186639697.541	244.078	Z1267773765	-6.2927
DSi PoisedLibrary_1680186639697.674	233.178	Z54508609	-6.2922
DSi PoisedLibrary_1680186639697.769	219.137	Z1509441355	-6.2909
DSi PoisedLibrary_1680186639697.739	247.240	Z27805986	-6.2832
DSi PoisedLibrary_1680186639697.628	240.106	Z124039706	-6.283
DSi PoisedLibrary_1680186639697.383	245.085	Z19735192	-6.2822
DSi PoisedLibrary_1680186639697.234	210.046	Z119990900	-6.2791
DSi PoisedLibrary_1680186639697.674	233.178	Z54508609	-6.2698
DSi PoisedLibrary_1680186639697.19	219.101	Z111529496	-6.2696
DSi PoisedLibrary_1680186639697.541	244.078	Z1267773765	-6.2669
DSi PoisedLibrary_1680186639697.585	232.321	Z123935312	-6.2609
DSi PoisedLibrary_1680186639697.36	239.131	Z1470724953	-6.246
DSi PoisedLibrary_1680186639697.63	299.729	Z1004825200	-6.2459
DSi PoisedLibrary_1680186639697.803	175.075	Z383202616	-6.2431
DSi PoisedLibrary_1680186639697.45	224.095	Z235449082	-6.2307
DSi PoisedLibrary_1680186639697.724	227.250	Z30802768	-6.2298
DSi PoisedLibrary_1680186639697.378	206.142	Z332382842	-6.2285
DSi PoisedLibrary_1680186639697.58	207.269	Z1331830630	-6.2275
DSi PoisedLibrary_1680186639697.63	299.729	Z1004825200	-6.225
DSi PoisedLibrary_1680186639697.294	217.122	Z26781943	-6.2238
DSi PoisedLibrary_1680186639697.813	219.207	Z1994237215	-6.2192
DSi PoisedLibrary_1680186639697.217	170.106	Z220816104	-6.2153
DSi PoisedLibrary_1680186639697.136	234.226	Z44584202	-6.2109

DSi PoisedLibrary_1680186639697.217	170.106	Z220816104	-6.2107
DSi PoisedLibrary_1680186639697.749	179.095	Z33452106	-6.2078
DSi PoisedLibrary_1680186639697.719	203.069	Z332370018	-6.2041
DSi PoisedLibrary_1680186639697.536	232.278	Z413792090	-6.1998
DSi PoisedLibrary_1680186639697.249	222.267	Z466628048	-6.1944
DSi PoisedLibrary_1680186639697.234	210.046	Z119990900	-6.1921
DSi PoisedLibrary_1680186639697.368	192.101	Z111634612	-6.1887
DSi PoisedLibrary_1680186639697.368	192.101	Z111634612	-6.1807
DSi PoisedLibrary_1680186639697.246	228.126	Z44095027	-6.1742
DSi PoisedLibrary_1680186639697.351	241.085	Z26781964	-6.1621
DSi PoisedLibrary_1680186639697.63	299.729	Z1004825200	-6.1573
DSi PoisedLibrary_1680186639697.436	248.228	Z44586758	-6.1569
DSi PoisedLibrary_1680186639697.11	199.040	Z19727416	-6.1501
DSi PoisedLibrary_1680186639697.678	223.229	Z198194396	-6.1388
DSi PoisedLibrary_1680186639697.777	267.127	Z352356442	-6.1351
DSi PoisedLibrary_1680186639697.643	253.098	Z381033368	-6.1255
DSi PoisedLibrary_1680186639697.378	206.142	Z332382842	-6.1251
DSi PoisedLibrary_1680186639697.98	204.101	Z131516158	-6.1244
DSi PoisedLibrary_1680186639697.778	245.085	Z1250129028	-6.1052
DSi PoisedLibrary_1680186639697.172	204.126	Z1206890404	-6.1009
DSi PoisedLibrary_1680186639697.683	231.051	Z57111868	-6.0922
DSi PoisedLibrary_1680186639697.809	404.399	Z3015133857	-6.0692
DSi PoisedLibrary_1680186639697.570	231.294	Z29077827	-6.0653
DSi PoisedLibrary_1680186639697.125	238.087	Z275165822	-6.0381
DSi PoisedLibrary_1680186639697.605	198.056	Z1696091761	-6.0331
DSi PoisedLibrary_1680186639697.424	217.122	Z26781952	-6.0297
DSi PoisedLibrary_1680186639697.754	165.090	Z1324853681	-6.0164
DSi PoisedLibrary_1680186639697.769	219.137	Z1509441355	-6.0141
DSi PoisedLibrary_1680186639697.553	179.095	Z56960248	-6.0087
DSi PoisedLibrary_1680186639697.803	175.075	Z383202616	-6.0078
DSi PoisedLibrary_1680186639697.399	238.326	Z32399948	-5.9977
DSi PoisedLibrary_1680186639697.294	217.122	Z26781943	-5.996
DSi PoisedLibrary_1680186639697.777	267.127	Z352356442	-5.9952
DSi PoisedLibrary_1680186639697.503	242.316	Z223388508	-5.9842
DSi PoisedLibrary_1680186639697.503	242.316	Z223388508	-5.9806
DSi PoisedLibrary_1680186639697.547	246.080	Z55290386	-5.9782
DSi PoisedLibrary_1680186639697.59	223.112	Z198194394	-5.9759
DSi PoisedLibrary_1680186639697.810	244.101	Z606937774	-5.9749
DSi PoisedLibrary_1680186639697.602	153.059	Z1198169663	-5.9675
DSi PoisedLibrary_1680186639697.217	170.106	Z220816104	-5.9544
DSi PoisedLibrary_1680186639697.266	206.106	Z29177127	-5.947
DSi PoisedLibrary_1680186639697.137	190.111	Z285141302	-5.9324
DSi PoisedLibrary_1680186639697.137	190.111	Z285141302	-5.9231

DSi PoisedLibrary_1680186639697.828	236.023	Z277969624	-5.9212
DSi PoisedLibrary_1680186639697.424	217.122	Z26781952	-5.9165
DSi PoisedLibrary_1680186639697.789	210.248	Z1929757385	-5.9164
DSi PoisedLibrary_1680186639697.570	231.294	Z29077827	-5.9103
DSi PoisedLibrary_1680186639697.236	226.078	Z1333043510	-5.8969
DSi PoisedLibrary_1680186639697.639	159.090	Z979770898	-5.8864
DSi PoisedLibrary_1680186639697.806	175.056	Z1262633486	-5.8835
DSi PoisedLibrary_1680186639697.777	267.127	Z352356442	-5.8714
DSi PoisedLibrary_1680186639697.403	189.115	Z28870646	-5.8646
DSi PoisedLibrary_1680186639697.541	244.078	Z1267773765	-5.8388
DSi PoisedLibrary_1680186639697.137	190.111	Z285141302	-5.8325
DSi PoisedLibrary_1680186639697.378	206.142	Z332382842	-5.828
DSi PoisedLibrary_1680186639697.208	189.077	Z56960214	-5.8215
DSi PoisedLibrary_1680186639697.424	217.122	Z26781952	-5.8203
DSi PoisedLibrary_1680186639697.432	277.243	Z364577298	-5.8191
DSi PoisedLibrary_1680186639697.260	171.101	Z90122368	-5.8125
DSi PoisedLibrary_1680186639697.254	175.075	Z1618027593	-5.8113
DSi PoisedLibrary_1680186639697.477	147.068	Z1203730757	-5.8099
DSi PoisedLibrary_1680186639697.645	162.091	Z234898257	-5.7979
DSi PoisedLibrary_1680186639697.68	203.106	Z26251905	-5.7976
DSi PoisedLibrary_1680186639697.236	226.078	Z1333043510	-5.7973
DSi PoisedLibrary_1680186639697.309	209.217	Z1730522163	-5.796
DSi PoisedLibrary_1680186639697.403	189.115	Z28870646	-5.7708
DSi PoisedLibrary_1680186639697.254	175.075	Z1618027593	-5.7451
DSi PoisedLibrary_1680186639697.432	277.243	Z364577298	-5.7438
DSi PoisedLibrary_1680186639697.503	242.316	Z223388508	-5.7346
DSi PoisedLibrary_1680186639697.602	153.059	Z1198169663	-5.6409
DSi PoisedLibrary_1680186639697.260	171.101	Z90122368	-5.6367
DSi PoisedLibrary_1680186639697.28	265.111	Z85893853	-5.6177
DSi PoisedLibrary_1680186639697.217	170.106	Z220816104	-5.5968
DSi PoisedLibrary_1680186639697.28	265.111	Z85893853	-5.508
DSi PoisedLibrary_1680186639697.217	170.106	Z220816104	-5.5052
DSi PoisedLibrary_1680186639697.28	265.111	Z85893853	-5.4774
DSi PoisedLibrary_1680186639697.562	163.081	Z1198316457	-5.4553
DSi PoisedLibrary_1680186639697.817	173.167	Z1250093582	-4.9666
DSi PoisedLibrary_1680186639697.817	173.167	Z1250093582	-4.9308

Table S2. VS1 and VS2 for PDB fragment library targeting PARP1

Compound	PUBCHEM_MW	Energy in silico
VS1		
1H-Benzo[d]imidazole-2-sulfonamide	197.220	-5.8791
VS2		

Proflavine	209.250	-6.9278
octahydroindolizine-1,6,7,8-tetrol	189.210	-6.7111
octahydroindolizine-1,6,7,8-tetrol	189.210	-6.668
(2S,3R,5S)-5-(6-aminopurin-9-yl)-2-(hydroxymethyl)oxolan-3-ol	251.240	-6.5825
octahydroindolizine-1,6,7,8-tetrol	189.210	-6.5145
octahydroindolizine-1,6,7,8-tetrol	189.210	-6.4968
(2S,3R,5S)-5-(6-aminopurin-9-yl)-2-(hydroxymethyl)oxolan-3-ol	251.240	-6.4839
Melatonin	232.280	-6.4695
octahydroindolizine-1,6,7,8-tetrol	189.210	-6.451
octahydroindolizine-1,6,7,8-tetrol	189.210	-6.4434
octahydroindolizine-1,6,7,8-tetrol	189.210	-6.4323
1H-Benzo[d]imidazole-2-sulfonamide	197.220	-6.4034
Tyramine	137.180	-6.38
octahydroindolizine-1,6,7,8-tetrol	189.210	-6.3615
octahydroindolizine-1,6,7,8-tetrol	189.210	-6.3523
2-amino-6-chloro purine	169.570	-6.326
4'-Hydroxyflavanone	240.250	-6.3196
4'-Hydroxyflavanone	240.250	-6.3079
octahydroindolizine-1,6,7,8-tetrol	189.210	-6.3072
Cordycepin	251.240	-6.3017
4'-Hydroxyflavanone	240.250	-6.2999
Thiabendazole	201.250	-6.2138
1H-Benzo[d]imidazole-2-sulfonamide	197.220	-6.1752
4'-Hydroxyflavanone	240.250	-6.1543
Resveratrol	228.240	-6.1504
Resveratrol	228.240	-6.1327
Cytosine deoxyribonucleoside		-6.1262
Serotonin	176.210	-6.1245
Thiabendazole	201.250	-6.0859
1H-Indazole	118.140	-6.0792
Resveratrol	228.240	-6.0565
Serotonin	176.210	-6.0174
6-Hydroxy-2-benzothiazolesulfonamide	230.300	-5.9866
Dimedazol	146.190	-5.9099
7-Aminoindazole	133.150	-5.9045
Difunisal	250.200	-5.7229
6-methyltetrahydropterin	181.200	-5.6869
6-methyltetrahydropterin	181.200	-5.6282
(1H-Indol-3-yl)acetic acid	175.180	-5.2675

Table S3. VS1 and VS2 for FDA approved LH1 targeting PARP1

compound	MolWt	Name	Energy in silico
VS1			
MOlport_LH1_Approved_FDA_library.maegz:605	594.520	Proanthocyanidins	-11.9696
VS2			
MOlport_LH1_Approved_FDA_library.maegz:110	444.480	Mitoxantrone	-9.7899
MOlport_LH1_Approved_FDA_library.maegz:1976	517.400	Mitoxantrone hydrochloride	-9.7887
MOlport_LH1_Approved_FDA_library.maegz:110	444.480	Mitoxantrone	-9.681
MOlport_LH1_Approved_FDA_library.maegz:1976	517.400	Mitoxantrone hydrochloride	-9.6795
MOlport_LH1_Approved_FDA_library.maegz:605	594.520	Proanthocyanidins	-9.2854
MOlport_LH1_Approved_FDA_library.maegz:605	594.520	Proanthocyanidins	-9.1062
MOlport_LH1_Approved_FDA_library.maegz:605	594.520	Proanthocyanidins	-9.0315
MOlport_LH1_Approved_FDA_library.maegz:110	444.480	Mitoxantrone	-8.9159
MOlport_LH1_Approved_FDA_library.maegz:1976	517.400	Mitoxantrone hydrochloride	-8.9156
MOlport_LH1_Approved_FDA_library.maegz:605	594.520	Proanthocyanidins	-8.8949
MOlport_LH1_Approved_FDA_library.maegz:605	594.520	Proanthocyanidins	-8.8942
MOlport_LH1_Approved_FDA_library.maegz:605	594.520	Proanthocyanidins	-8.8788
MOlport_LH1_Approved_FDA_library.maegz:605	594.520	Proanthocyanidins	-8.8631
MOlport_LH1_Approved_FDA_library.maegz:605	594.520	Proanthocyanidins	-8.8348
MOlport_LH1_Approved_FDA_library.maegz:605	594.520	Proanthocyanidins	-8.7844
MOlport_LH1_Approved_FDA_library.maegz:605	594.520	Proanthocyanidins	-8.7774
MOlport_LH1_Approved_FDA_library.maegz:605	594.520	Proanthocyanidins	-8.7405
MOlport_LH1_Approved_FDA_library.maegz:1580	610.520	Rutin	-8.6593
MOlport_LH1_Approved_FDA_library.maegz:605	594.520	Proanthocyanidins	-8.5661
MOlport_LH1_Approved_FDA_library.maegz:605	594.520	Proanthocyanidins	-8.5432
MOlport_LH1_Approved_FDA_library.maegz:605	594.520	Proanthocyanidins	-8.4863
MOlport_LH1_Approved_FDA_library.maegz:605	594.520	Proanthocyanidins	-8.47
MOlport_LH1_Approved_FDA_library.maegz:1428	527.590	Dequalinium chloride	-8.4372
MOlport_LH1_Approved_FDA_library.maegz:605	594.520	Proanthocyanidins	-8.4251
MOlport_LH1_Approved_FDA_library.maegz:605	594.520	Proanthocyanidins	-8.3777
MOlport_LH1_Approved_FDA_library.maegz:605	594.520	Proanthocyanidins	-8.3694
MOlport_LH1_Approved_FDA_library.maegz:1580	610.520	Rutin	-8.3457
MOlport_LH1_Approved_FDA_library.maegz:265	711.870	Indinavir sulfate	-8.3416
MOlport_LH1_Approved_FDA_library.maegz:605	594.520	Proanthocyanidins	-8.3367
MOlport_LH1_Approved_FDA_library.maegz:1918	624.590	Methyl hesperidin	-8.2999
MOlport_LH1_Approved_FDA_library.maegz:265	711.870	Indinavir sulfate	-8.2891
MOlport_LH1_Approved_FDA_library.maegz:1597	396.580	Casanthranol	-8.2871
MOlport_LH1_Approved_FDA_library.maegz:605	594.520	Proanthocyanidins	-8.2778
MOlport_LH1_Approved_FDA_library.maegz:605	594.520	Proanthocyanidins	-8.2722
MOlport_LH1_Approved_FDA_library.maegz:1918	624.590	Methyl hesperidin	-8.2646
MOlport_LH1_Approved_FDA_library.maegz:265	711.870	Indinavir sulfate	-8.2477
MOlport_LH1_Approved_FDA_library.maegz:605	594.520	Proanthocyanidins	-8.2372
MOlport_LH1_Approved_FDA_library.maegz:1597	396.580	Casanthranol	-8.2216

MOlport_LH1_Approved_FDA_library.maegz:989	510.040	Raloxifene hydrochloride	-8.2189
MOlport_LH1_Approved_FDA_library.maegz:605	594.520	Proanthocyanidins	-8.2175
MOlport_LH1_Approved_FDA_library.maegz:265	711.870	Indinavir sulfate	-8.2127
MOlport_LH1_Approved_FDA_library.maegz:1450	433.950	Hydroxychloroquine sulfate	-8.2026
MOlport_LH1_Approved_FDA_library.maegz:605	594.520	Proanthocyanidins	-8.1884
MOlport_LH1_Approved_FDA_library.maegz:605	594.520	Proanthocyanidins	-8.1846
MOlport_LH1_Approved_FDA_library.maegz:1597	396.580	Casanthranol	-8.1562
MOlport_LH1_Approved_FDA_library.maegz:1918	624.590	Methyl hesperidin	-8.1424
MOlport_LH1_Approved_FDA_library.maegz:451	814.840	Isavuconazonium sulfate	-8.1237
MOlport_LH1_Approved_FDA_library.maegz:605	594.520	Proanthocyanidins	-8.1167
MOlport_LH1_Approved_FDA_library.maegz:1597	396.580	Casanthranol	-8.1052
MOlport_LH1_Approved_FDA_library.maegz:1428	527.590	Dequalinium chloride	-8.1012
MOlport_LH1_Approved_FDA_library.maegz:605	594.520	Proanthocyanidins	-8.0904
MOlport_LH1_Approved_FDA_library.maegz:265	711.870	Indinavir sulfate	-8.0762
MOlport_LH1_Approved_FDA_library.maegz:1318	713.710	Paromomycin Sulfate	-8.0383
MOlport_LH1_Approved_FDA_library.maegz:605	594.520	Proanthocyanidins	-8.0323
MOlport_LH1_Approved_FDA_library.maegz:1597	396.580	Casanthranol	-8.0262
MOlport_LH1_Approved_FDA_library.maegz:605	594.520	Proanthocyanidins	-8.025
MOlport_LH1_Approved_FDA_library.maegz:1597	396.580	Casanthranol	-8.0161
MOlport_LH1_Approved_FDA_library.maegz:1597	396.580	Casanthranol	-7.989
MOlport_LH1_Approved_FDA_library.maegz:1597	396.580	Casanthranol	-7.9815
MOlport_LH1_Approved_FDA_library.maegz:605	594.520	Proanthocyanidins	-7.953
MOlport_LH1_Approved_FDA_library.maegz:265	711.870	Indinavir sulfate	-7.9495
MOlport_LH1_Approved_FDA_library.maegz:265	711.870	Indinavir sulfate	-7.9178
MOlport_LH1_Approved_FDA_library.maegz:256	663.890	Nelfinavir Mesylate	-7.9011
MOlport_LH1_Approved_FDA_library.maegz:605	594.520	Proanthocyanidins	-7.8984
MOlport_LH1_Approved_FDA_library.maegz:716	635.510	Tenofovir Disoproxil Fumarate	-7.882
MOlport_LH1_Approved_FDA_library.maegz:605	594.520	Proanthocyanidins	-7.8658
MOlport_LH1_Approved_FDA_library.maegz:268	500.550	Travoprost	-7.8457
MOlport_LH1_Approved_FDA_library.maegz:605	594.520	Proanthocyanidins	-7.8446
MOlport_LH1_Approved_FDA_library.maegz:1318	713.710	Paromomycin Sulfate	-7.8431
MOlport_LH1_Approved_FDA_library.maegz:1597	396.580	Casanthranol	-7.8419
MOlport_LH1_Approved_FDA_library.maegz:605	594.520	Proanthocyanidins	-7.8289
MOlport_LH1_Approved_FDA_library.maegz:623	418.390	Aloin	-7.8205
MOlport_LH1_Approved_FDA_library.maegz:1318	713.710	Paromomycin Sulfate	-7.8166
MOlport_LH1_Approved_FDA_library.maegz:605	594.520	Proanthocyanidins	-7.8138
MOlport_LH1_Approved_FDA_library.maegz:1450	433.950	Hydroxychloroquine sulfate	-7.8017
MOlport_LH1_Approved_FDA_library.maegz:864	388.440	Ilaprazole sodium	-7.7874
MOlport_LH1_Approved_FDA_library.maegz:605	594.520	Proanthocyanidins	-7.7833
MOlport_LH1_Approved_FDA_library.maegz:605	594.520	Proanthocyanidins	-7.7805
MOlport_LH1_Approved_FDA_library.maegz:268	500.550	Travoprost	-7.776
MOlport_LH1_Approved_FDA_library.maegz:136	518.410	Fenticonazole Nitrate	-7.7731

MOlport_LH1_Approved_FDA_library.maegz:292	507.430	Fupentixol Dihydrochloride	-7.7723
MOlport_LH1_Approved_FDA_library.maegz:93	500.780	Sertaconazole nitrate	-7.7722
MOlport_LH1_Approved_FDA_library.maegz:1597	396.580	Casanthranol	-7.7662
MOlport_LH1_Approved_FDA_library.maegz:605	594.520	Proanthocyanidins	-7.7614
MOlport_LH1_Approved_FDA_library.maegz:1597	396.580	Casanthranol	-7.7607
MOlport_LH1_Approved_FDA_library.maegz:1597	396.580	Casanthranol	-7.7562
MOlport_LH1_Approved_FDA_library.maegz:605	594.520	Proanthocyanidins	-7.7561
MOlport_LH1_Approved_FDA_library.maegz:2003	579.980	Epirubicin hydrochloride	-7.7561
MOlport_LH1_Approved_FDA_library.maegz:605	594.520	Proanthocyanidins	-7.7477
MOlport_LH1_Approved_FDA_library.maegz:256	663.890	Nelfinavir Mesylate	-7.735
MOlport_LH1_Approved_FDA_library.maegz:265	711.870	Indinavir sulfate	-7.7258
MOlport_LH1_Approved_FDA_library.maegz:1318	713.710	Paromomycin Sulfate	-7.7154
MOlport_LH1_Approved_FDA_library.maegz:2058	510.440	Fluphenazine hydrochloride	-7.7073
MOlport_LH1_Approved_FDA_library.maegz:1597	396.580	Casanthranol	-7.7022
MOlport_LH1_Approved_FDA_library.maegz:268	500.550	Travoprost	-7.6933
MOlport_LH1_Approved_FDA_library.maegz:268	500.550	Travoprost	-7.6911
MOlport_LH1_Approved_FDA_library.maegz:1451	908.870	Neomycin sulfate	-7.6884
MOlport_LH1_Approved_FDA_library.maegz:605	594.520	Proanthocyanidins	-7.6865
MOlport_LH1_Approved_FDA_library.maegz:605	594.520	Proanthocyanidins	-7.6861
MOlport_LH1_Approved_FDA_library.maegz:825	712.220	Neomycin Sulphate B	-7.6811
MOlport_LH1_Approved_FDA_library.maegz:1318	713.710	Paromomycin Sulfate	-7.6788
MOlport_LH1_Approved_FDA_library.maegz:268	500.550	Travoprost	-7.6722
MOlport_LH1_Approved_FDA_library.maegz:1807	473.400	Clofazimine	-7.6685
MOlport_LH1_Approved_FDA_library.maegz:1597	396.580	Casanthranol	-7.6618
MOlport_LH1_Approved_FDA_library.maegz:623	418.390	Aloin	-7.6578
MOlport_LH1_Approved_FDA_library.maegz:1597	396.580	Casanthranol	-7.6531
MOlport_LH1_Approved_FDA_library.maegz:1674	369.360	Lansoprazole	-7.6479
MOlport_LH1_Approved_FDA_library.maegz:674	564.550	Aclidinium bromide	-7.6474
MOlport_LH1_Approved_FDA_library.maegz:1597	396.580	Casanthranol	-7.6457
MOlport_LH1_Approved_FDA_library.maegz:265	711.870	Indinavir sulfate	-7.6419
MOlport_LH1_Approved_FDA_library.maegz:136	518.410	Fenticonazole Nitrate	-7.6363
MOlport_LH1_Approved_FDA_library.maegz:268	500.550	Travoprost	-7.63
MOlport_LH1_Approved_FDA_library.maegz:1597	396.580	Casanthranol	-7.626
MOlport_LH1_Approved_FDA_library.maegz:1597	396.580	Casanthranol	-7.6199
MOlport_LH1_Approved_FDA_library.maegz:265	711.870	Indinavir sulfate	-7.6185
MOlport_LH1_Approved_FDA_library.maegz:93	500.780	Sertaconazole nitrate	-7.6153
MOlport_LH1_Approved_FDA_library.maegz:268	500.550	Travoprost	-7.6043
MOlport_LH1_Approved_FDA_library.maegz:864	388.440	Ilaprazole sodium	-7.5915
MOlport_LH1_Approved_FDA_library.maegz:2049	943.470	Lapatinib ditosylate monohydrate	-7.5851
MOlport_LH1_Approved_FDA_library.maegz:265	711.870	Indinavir sulfate	-7.5846
MOlport_LH1_Approved_FDA_library.maegz:210	925.460	Lapatinib Ditosylate	-7.5824
MOlport_LH1_Approved_FDA_library.maegz:2050	581.060	Lapatinib	-7.5824

MOlport_LH1_Approved_FDA_library.maegz:1597	396.580	Casanthranol	-7.5823
MOlport_LH1_Approved_FDA_library.maegz:465	582.970	Ivosidenib	-7.5786
MOlport_LH1_Approved_FDA_library.maegz:2003	579.980	Epirubicin hydrochloride	-7.5683
MOlport_LH1_Approved_FDA_library.maegz:1393	579.990	Doxorubicin hydrochloride	-7.5631
MOlport_LH1_Approved_FDA_library.maegz:605	594.520	Proanthocyanidins	-7.561
MOlport_LH1_Approved_FDA_library.maegz:268	500.550	Travoprost	-7.5599
MOlport_LH1_Approved_FDA_library.maegz:265	711.870	Indinavir sulfate	-7.5575
MOlport_LH1_Approved_FDA_library.maegz:1597	396.580	Casanthranol	-7.5546
MOlport_LH1_Approved_FDA_library.maegz:1318	713.710	Paromomycin Sulfate	-7.5538
MOlport_LH1_Approved_FDA_library.maegz:790	730.710	Dihydrostreptomycin sulfate	-7.5518
MOlport_LH1_Approved_FDA_library.maegz:1597	396.580	Casanthranol	-7.551
MOlport_LH1_Approved_FDA_library.maegz:1451	908.870	Neomycin sulfate	-7.5451
MOlport_LH1_Approved_FDA_library.maegz:268	500.550	Travoprost	-7.544
MOlport_LH1_Approved_FDA_library.maegz:1807	473.400	Clofazimine	-7.5424
MOlport_LH1_Approved_FDA_library.maegz:265	711.870	Indinavir sulfate	-7.5412
MOlport_LH1_Approved_FDA_library.maegz:825	712.220	Neomycin Sulphate B	-7.5409
MOlport_LH1_Approved_FDA_library.maegz:605	594.520	Proanthocyanidins	-7.5388
MOlport_LH1_Approved_FDA_library.maegz:268	500.550	Travoprost	-7.5334
MOlport_LH1_Approved_FDA_library.maegz:458	595.710	Osimertinib mesylate	-7.5325
MOlport_LH1_Approved_FDA_library.maegz:1013	1219.600	Aliskiren hemifumarate	-7.5299
MOlport_LH1_Approved_FDA_library.maegz:710	499.610	Osimertinib	-7.5292
MOlport_LH1_Approved_FDA_library.maegz:1836	479.150	Miconazole nitrate	-7.5251
MOlport_LH1_Approved_FDA_library.maegz:268	500.550	Travoprost	-7.525
MOlport_LH1_Approved_FDA_library.maegz:436	480.360	Anlotinib Dihydrochloride	-7.5196
MOlport_LH1_Approved_FDA_library.maegz:479	407.440	Anlotinib	-7.5196
MOlport_LH1_Approved_FDA_library.maegz:635	367.400	Esomeprazole Sodium	-7.5173
MOlport_LH1_Approved_FDA_library.maegz:1950	472.540	Linagliptin	-7.5149
MOlport_LH1_Approved_FDA_library.maegz:1318	713.710	Paromomycin Sulfate	-7.506
MOlport_LH1_Approved_FDA_library.maegz:1597	396.580	Casanthranol	-7.504
MOlport_LH1_Approved_FDA_library.maegz:636	713.120	Esomeprazole Magnesium	-7.5017
MOlport_LH1_Approved_FDA_library.maegz:268	500.550	Travoprost	-7.4951
MOlport_LH1_Approved_FDA_library.maegz:1597	396.580	Casanthranol	-7.4939
MOlport_LH1_Approved_FDA_library.maegz:674	564.550	Aclidinium bromide	-7.4897
MOlport_LH1_Approved_FDA_library.maegz:1451	908.870	Neomycin sulfate	-7.4884
MOlport_LH1_Approved_FDA_library.maegz:605	594.520	Proanthocyanidins	-7.4872
MOlport_LH1_Approved_FDA_library.maegz:1597	396.580	Casanthranol	-7.4857
MOlport_LH1_Approved_FDA_library.maegz:825	712.220	Neomycin Sulphate B	-7.4841
MOlport_LH1_Approved_FDA_library.maegz:1597	396.580	Casanthranol	-7.4838
MOlport_LH1_Approved_FDA_library.maegz:1597	396.580	Casanthranol	-7.482
MOlport_LH1_Approved_FDA_library.maegz:1597	396.580	Casanthranol	-7.4694
MOlport_LH1_Approved_FDA_library.maegz:1318	713.710	Paromomycin Sulfate	-7.4686
MOlport_LH1_Approved_FDA_library.maegz:2065	1457.380	Streptomycin sulfate	-7.4515
MOlport_LH1_Approved_FDA_library.maegz:268	500.550	Travoprost	-7.4498

MOlport_LH1_Approved_FDA_library.maegz:268	500.550	Travoprost	-7.4482
MOlport_LH1_Approved_FDA_library.maegz:93	500.780	Sertaconazole nitrate	-7.4436
MOlport_LH1_Approved_FDA_library.maegz:1451	908.870	Neomycin sulfate	-7.4385
MOlport_LH1_Approved_FDA_library.maegz:97	315.470	Rotigotine	-7.4379
MOlport_LH1_Approved_FDA_library.maegz:292	507.430	Fupentixol Dihydrochloride	-7.436
MOlport_LH1_Approved_FDA_library.maegz:1597	396.580	Casanthranol	-7.4342
MOlport_LH1_Approved_FDA_library.maegz:93	500.780	Sertaconazole nitrate	-7.4324
MOlport_LH1_Approved_FDA_library.maegz:268	500.550	Travoprost	-7.4302
MOlport_LH1_Approved_FDA_library.maegz:265	711.870	Indinavir sulfate	-7.4271
MOlport_LH1_Approved_FDA_library.maegz:825	712.220	Neomycin Sulphate B	-7.4243
MOlport_LH1_Approved_FDA_library.maegz:265	711.870	Indinavir sulfate	-7.4236
MOlport_LH1_Approved_FDA_library.maegz:605	594.520	Proanthocyanidins	-7.423
MOlport_LH1_Approved_FDA_library.maegz:1597	396.580	Casanthranol	-7.4225
MOlport_LH1_Approved_FDA_library.maegz:1597	396.580	Casanthranol	-7.4207
MOlport_LH1_Approved_FDA_library.maegz:268	500.550	Travoprost	-7.4189
MOlport_LH1_Approved_FDA_library.maegz:1948	683.620	Manidipine dihydrochloride	-7.4038
MOlport_LH1_Approved_FDA_library.maegz:1597	396.580	Casanthranol	-7.4033
MOlport_LH1_Approved_FDA_library.maegz:112	610.700	Manidipine	-7.4023
MOlport_LH1_Approved_FDA_library.maegz:930	408.330	Trazodone hydrochloride	-7.3973
MOlport_LH1_Approved_FDA_library.maegz:1597	396.580	Casanthranol	-7.3964
MOlport_LH1_Approved_FDA_library.maegz:265	711.870	Indinavir sulfate	-7.3913
MOlport_LH1_Approved_FDA_library.maegz:716	635.510	Tenofovir Disoproxil Fumarate	-7.3911
MOlport_LH1_Approved_FDA_library.maegz:1450	433.950	Hydroxychloroquine sulfate	-7.3906
MOlport_LH1_Approved_FDA_library.maegz:605	594.520	Proanthocyanidins	-7.3848
MOlport_LH1_Approved_FDA_library.maegz:268	500.550	Travoprost	-7.3839
MOlport_LH1_Approved_FDA_library.maegz:731	557.210	Pixantrone dimaleate	-7.38
MOlport_LH1_Approved_FDA_library.maegz:1506	608.540	Diosmin	-7.3781
MOlport_LH1_Approved_FDA_library.maegz:2003	579.980	Epirubicin hydrochloride	-7.3742
MOlport_LH1_Approved_FDA_library.maegz:1450	433.950	Hydroxychloroquine sulfate	-7.3708
MOlport_LH1_Approved_FDA_library.maegz:17	398.440	Ademetionine	-7.3704
MOlport_LH1_Approved_FDA_library.maegz:1318	713.710	Paromomycin Sulfate	-7.3688
MOlport_LH1_Approved_FDA_library.maegz:2049	943.470	Lapatinib ditosylate monohydrate	-7.3661
MOlport_LH1_Approved_FDA_library.maegz:210	925.460	Lapatinib Ditosylate	-7.3622
MOlport_LH1_Approved_FDA_library.maegz:2050	581.060	Lapatinib	-7.3622
MOlport_LH1_Approved_FDA_library.maegz:153	341.870	Dapoxetine HCl	-7.3617
MOlport_LH1_Approved_FDA_library.maegz:1947	515.870	Chloroquine diphosphate	-7.3488
MOlport_LH1_Approved_FDA_library.maegz:2058	510.440	Fluphenazine hydrochloride	-7.3481
MOlport_LH1_Approved_FDA_library.maegz:825	712.220	Neomycin Sulphate B	-7.3323
MOlport_LH1_Approved_FDA_library.maegz:1597	396.580	Casanthranol	-7.3275
MOlport_LH1_Approved_FDA_library.maegz:1451	908.870	Neomycin sulfate	-7.3246
MOlport_LH1_Approved_FDA_library.maegz:1450	433.950	Hydroxychloroquine sulfate	-7.322

MOlport_LH1_Approved_FDA_library.maegz:1021	563.990	Daunorubicin hydrochloride	-7.32
MOlport_LH1_Approved_FDA_library.maegz:1597	396.580	Casanthranol	-7.3193
MOlport_LH1_Approved_FDA_library.maegz:458	595.710	Osimertinib mesylate	-7.3173
MOlport_LH1_Approved_FDA_library.maegz:710	499.610	Osimertinib	-7.3149
MOlport_LH1_Approved_FDA_library.maegz:710	499.610	Osimertinib	-7.3134
MOlport_LH1_Approved_FDA_library.maegz:1597	396.580	Casanthranol	-7.3125
MOlport_LH1_Approved_FDA_library.maegz:458	595.710	Osimertinib mesylate	-7.3119
MOlport_LH1_Approved_FDA_library.maegz:1597	396.580	Casanthranol	-7.3105
MOlport_LH1_Approved_FDA_library.maegz:1451	908.870	Neomycin sulfate	-7.3065
MOlport_LH1_Approved_FDA_library.maegz:989	510.040	Raloxifene hydrochloride	-7.3062
MOlport_LH1_Approved_FDA_library.maegz:265	711.870	Indinavir sulfate	-7.3053
MOlport_LH1_Approved_FDA_library.maegz:1597	396.580	Casanthranol	-7.3039
MOlport_LH1_Approved_FDA_library.maegz:825	712.220	Neomycin Sulphate B	-7.3028
MOlport_LH1_Approved_FDA_library.maegz:605	594.520	Proanthocyanidins	-7.3023
MOlport_LH1_Approved_FDA_library.maegz:1451	908.870	Neomycin sulfate	-7.3008
MOlport_LH1_Approved_FDA_library.maegz:825	712.220	Neomycin Sulphate B	-7.2981
MOlport_LH1_Approved_FDA_library.maegz:1451	908.870	Neomycin sulfate	-7.2949
MOlport_LH1_Approved_FDA_library.maegz:265	711.870	Indinavir sulfate	-7.291
MOlport_LH1_Approved_FDA_library.maegz:2058	510.440	Fluphenazine hydrochloride	-7.2907
MOlport_LH1_Approved_FDA_library.maegz:825	712.220	Neomycin Sulphate B	-7.2898
MOlport_LH1_Approved_FDA_library.maegz:636	713.120	Esomeprazole Magnesium	-7.2881
MOlport_LH1_Approved_FDA_library.maegz:268	500.550	Travoprost	-7.2854
MOlport_LH1_Approved_FDA_library.maegz:189	312.370	Tofacitinib	-7.282
MOlport_LH1_Approved_FDA_library.maegz:726	504.490	Tofacitinib Citrate	-7.282
MOlport_LH1_Approved_FDA_library.maegz:268	500.550	Travoprost	-7.2801
MOlport_LH1_Approved_FDA_library.maegz:1947	515.870	Chloroquine diphosphate	-7.2794
MOlport_LH1_Approved_FDA_library.maegz:1277	332.870	Palonosetron hydrochloride	-7.2761
MOlport_LH1_Approved_FDA_library.maegz:605	594.520	Proanthocyanidins	-7.2724
MOlport_LH1_Approved_FDA_library.maegz:268	500.550	Travoprost	-7.2724
MOlport_LH1_Approved_FDA_library.maegz:54	473.980	Pazopanib HCl	-7.2701
MOlport_LH1_Approved_FDA_library.maegz:2029	437.520	Pazopanib	-7.2683
MOlport_LH1_Approved_FDA_library.maegz:268	500.550	Travoprost	-7.2666
MOlport_LH1_Approved_FDA_library.maegz:1597	396.580	Casanthranol	-7.2624
MOlport_LH1_Approved_FDA_library.maegz:2065	1457.380	Streptomycin sulfate	-7.2623
MOlport_LH1_Approved_FDA_library.maegz:757	447.880	Elvitegravir	-7.2619
MOlport_LH1_Approved_FDA_library.maegz:1597	396.580	Casanthranol	-7.2617
MOlport_LH1_Approved_FDA_library.maegz:605	594.520	Proanthocyanidins	-7.2597
MOlport_LH1_Approved_FDA_library.maegz:1597	396.580	Casanthranol	-7.2554
MOlport_LH1_Approved_FDA_library.maegz:1450	433.950	Hydroxychloroquine sulfate	-7.2478
MOlport_LH1_Approved_FDA_library.maegz:989	510.040	Raloxifene hydrochloride	-7.2449
MOlport_LH1_Approved_FDA_library.maegz:1597	396.580	Casanthranol	-7.2429
MOlport_LH1_Approved_FDA_library.maegz:268	500.550	Travoprost	-7.2417
MOlport_LH1_Approved_FDA_library.maegz:136	518.410	Fenticonazole Nitrate	-7.2402

MOlport_LH1_Approved_FDA_library.maegz:1451	908.870	Neomycin sulfate	-7.2391
MOlport_LH1_Approved_FDA_library.maegz:268	500.550	Travoprost	-7.2385
MOlport_LH1_Approved_FDA_library.maegz:1950	472.540	Linagliptin	-7.2372
MOlport_LH1_Approved_FDA_library.maegz:877	420.430	Aminophylline	-7.2362
MOlport_LH1_Approved_FDA_library.maegz:265	711.870	Indinavir sulfate	-7.235
MOlport_LH1_Approved_FDA_library.maegz:623	418.390	Aloin	-7.2331
MOlport_LH1_Approved_FDA_library.maegz:1228	515.990	Nicardipine hydrochloride	-7.2318
MOlport_LH1_Approved_FDA_library.maegz:825	712.220	Neomycin Sulphate B	-7.2309
MOlport_LH1_Approved_FDA_library.maegz:112	610.700	Manidipine	-7.2277
MOlport_LH1_Approved_FDA_library.maegz:1948	683.620	Manidipine dihydrochloride	-7.2274
MOlport_LH1_Approved_FDA_library.maegz:265	711.870	Indinavir sulfate	-7.2221
MOlport_LH1_Approved_FDA_library.maegz:825	712.220	Neomycin Sulphate B	-7.2193
MOlport_LH1_Approved_FDA_library.maegz:1451	908.870	Neomycin sulfate	-7.2191
MOlport_LH1_Approved_FDA_library.maegz:1396	585.600	Amikacin	-7.2178
MOlport_LH1_Approved_FDA_library.maegz:2065	1457.380	Streptomycin sulfate	-7.2178
MOlport_LH1_Approved_FDA_library.maegz:731	557.210	Pixantrone dimaleate	-7.2154
MOlport_LH1_Approved_FDA_library.maegz:265	711.870	Indinavir sulfate	-7.2141
MOlport_LH1_Approved_FDA_library.maegz:265	711.870	Indinavir sulfate	-7.2118
MOlport_LH1_Approved_FDA_library.maegz:1973	432.370	Pantoprazole Sodium Hydrate	-7.2114
MOlport_LH1_Approved_FDA_library.maegz:268	500.550	Travoprost	-7.2102
MOlport_LH1_Approved_FDA_library.maegz:1597	396.580	Casanthranol	-7.2068
MOlport_LH1_Approved_FDA_library.maegz:1950	472.540	Linagliptin	-7.2043
MOlport_LH1_Approved_FDA_library.maegz:1580	610.520	Rutin	-7.1938
MOlport_LH1_Approved_FDA_library.maegz:1391	527.520	Hygromycin B	-7.1931
MOlport_LH1_Approved_FDA_library.maegz:1082	474.790	Butoconazole nitrate	-7.1841
MOlport_LH1_Approved_FDA_library.maegz:1597	396.580	Casanthranol	-7.1832
MOlport_LH1_Approved_FDA_library.maegz:707	464.590	Tozasertib	-7.179
MOlport_LH1_Approved_FDA_library.maegz:1597	396.580	Casanthranol	-7.1762
MOlport_LH1_Approved_FDA_library.maegz:1337	473.560	Nafronyl oxalate	-7.1736
MOlport_LH1_Approved_FDA_library.maegz:605	594.520	Proanthocyanidins	-7.1708
MOlport_LH1_Approved_FDA_library.maegz:528	766.860	Sultamicillin Tosylate	-7.1683
MOlport_LH1_Approved_FDA_library.maegz:1251	400.490	Ifenprodil Tartrate	-7.1648
MOlport_LH1_Approved_FDA_library.maegz:951	409.430	Ezetimibe	-7.1611
MOlport_LH1_Approved_FDA_library.maegz:92	482.440	Silymarin	-7.1601
MOlport_LH1_Approved_FDA_library.maegz:531	683.680	Amikacin Sulfate Salt	-7.1591
MOlport_LH1_Approved_FDA_library.maegz:605	594.520	Proanthocyanidins	-7.154
MOlport_LH1_Approved_FDA_library.maegz:1582	582.580	Kanamycin sulfate	-7.1483
MOlport_LH1_Approved_FDA_library.maegz:268	500.550	Travoprost	-7.1439
MOlport_LH1_Approved_FDA_library.maegz:1879	695.050	Meclocycline sulfosalicylate salt	-7.1426
MOlport_LH1_Approved_FDA_library.maegz:92	482.440	Silymarin	-7.1337
MOlport_LH1_Approved_FDA_library.maegz:1597	396.580	Casanthranol	-7.133

MOlport_LH1_Approved_FDA_library.maegz:268	500.550	Travoprost	-7.1318
MOlport_LH1_Approved_FDA_library.maegz:1981	530.450	Bosutinib	-7.1261
MOlport_LH1_Approved_FDA_library.maegz:268	500.550	Travoprost	-7.1248
MOlport_LH1_Approved_FDA_library.maegz:1879	695.050	Meclocycline sulfosalicylate salt	-7.1216
MOlport_LH1_Approved_FDA_library.maegz:268	500.550	Travoprost	-7.1215
MOlport_LH1_Approved_FDA_library.maegz:265	711.870	Indinavir sulfate	-7.1207
MOlport_LH1_Approved_FDA_library.maegz:731	557.210	Pixantrone dimaleate	-7.11
MOlport_LH1_Approved_FDA_library.maegz:92	482.440	Silymarin	-7.1075
MOlport_LH1_Approved_FDA_library.maegz:363	426.570	Perospirone	-7.1065
MOlport_LH1_Approved_FDA_library.maegz:1391	527.520	Hygromycin B	-7.1002
MOlport_LH1_Approved_FDA_library.maegz:2058	510.440	Fluphenazine hydrochloride	-7.0996
MOlport_LH1_Approved_FDA_library.maegz:1048	365.860	Ondansetron hydrochloride	-7.0986
MOlport_LH1_Approved_FDA_library.maegz:92	482.440	Silymarin	-7.0939
MOlport_LH1_Approved_FDA_library.maegz:605	594.520	Proanthocyanidins	-7.0935
MOlport_LH1_Approved_FDA_library.maegz:268	500.550	Travoprost	-7.092
MOlport_LH1_Approved_FDA_library.maegz:1318	713.710	Paromomycin Sulfate	-7.0911
MOlport_LH1_Approved_FDA_library.maegz:1597	396.580	Casanthranol	-7.0884
MOlport_LH1_Approved_FDA_library.maegz:674	564.550	Aclidinium bromide	-7.0878
MOlport_LH1_Approved_FDA_library.maegz:605	594.520	Proanthocyanidins	-7.0802
MOlport_LH1_Approved_FDA_library.maegz:1517	341.440	Propafenone	-7.0786
MOlport_LH1_Approved_FDA_library.maegz:1048	365.860	Ondansetron hydrochloride	-7.0742
MOlport_LH1_Approved_FDA_library.maegz:1597	396.580	Casanthranol	-7.0724
MOlport_LH1_Approved_FDA_library.maegz:363	426.570	Perospirone	-7.072
MOlport_LH1_Approved_FDA_library.maegz:757	447.880	Elvitegravir	-7.0719
MOlport_LH1_Approved_FDA_library.maegz:755	483.950	Avanafil	-7.0672
MOlport_LH1_Approved_FDA_library.maegz:268	500.550	Travoprost	-7.0647
MOlport_LH1_Approved_FDA_library.maegz:1597	396.580	Casanthranol	-7.061
MOlport_LH1_Approved_FDA_library.maegz:92	482.440	Silymarin	-7.0596
MOlport_LH1_Approved_FDA_library.maegz:864	388.440	Ilaprazole sodium	-7.0583
MOlport_LH1_Approved_FDA_library.maegz:1597	396.580	Casanthranol	-7.0536
MOlport_LH1_Approved_FDA_library.maegz:268	500.550	Travoprost	-7.0528
MOlport_LH1_Approved_FDA_library.maegz:268	500.550	Travoprost	-7.0515
MOlport_LH1_Approved_FDA_library.maegz:1318	713.710	Paromomycin Sulfate	-7.0514
MOlport_LH1_Approved_FDA_library.maegz:1391	527.520	Hygromycin B	-7.0393
MOlport_LH1_Approved_FDA_library.maegz:312	768.765	Cefotiam Hexetil Hydrochloride	-7.0388
MOlport_LH1_Approved_FDA_library.maegz:1318	713.710	Paromomycin Sulfate	-7.0302
MOlport_LH1_Approved_FDA_library.maegz:1403	448.400	Propantheline bromide	-7.0284
MOlport_LH1_Approved_FDA_library.maegz:926	628.800	Lopinavir	-7.0281
MOlport_LH1_Approved_FDA_library.maegz:1597	396.580	Casanthranol	-7.0264
MOlport_LH1_Approved_FDA_library.maegz:1927	463.430	Eletriptan HBr	-7.0255
MOlport_LH1_Approved_FDA_library.maegz:605	594.520	Proanthocyanidins	-7.0235

MOlport_LH1_Approved_FDA_library.maegz:1251	400.490	Ifenprodil Tartrate	-7.0227
MOlport_LH1_Approved_FDA_library.maegz:268	500.550	Travoprost	-7.0222
MOlport_LH1_Approved_FDA_library.maegz:605	594.520	Proanthocyanidins	-7.0187
MOlport_LH1_Approved_FDA_library.maegz:268	500.550	Travoprost	-7.0187
MOlport_LH1_Approved_FDA_library.maegz:265	711.870	Indinavir sulfate	-7.0175
MOlport_LH1_Approved_FDA_library.maegz:823	417.810	Pexidartinib	-7.0175
MOlport_LH1_Approved_FDA_library.maegz:1597	396.580	Casanthranol	-7.016
MOlport_LH1_Approved_FDA_library.maegz:1517	341.440	Propafenone	-7.0129
MOlport_LH1_Approved_FDA_library.maegz:605	594.520	Proanthocyanidins	-7.0128
MOlport_LH1_Approved_FDA_library.maegz:265	711.870	Indinavir sulfate	-7.0126
MOlport_LH1_Approved_FDA_library.maegz:608	270.240	Aloe-emodin	-7.0121
MOlport_LH1_Approved_FDA_library.maegz:733	539.580	Nafamostat mesylate	-7.0074
MOlport_LH1_Approved_FDA_library.maegz:268	500.550	Travoprost	-7.0057
MOlport_LH1_Approved_FDA_library.maegz:312	768.765	Cefotiam Hexetil Hydrochloride	-7.0051
MOlport_LH1_Approved_FDA_library.maegz:1391	527.520	Hygromycin B	-6.9969
MOlport_LH1_Approved_FDA_library.maegz:268	500.550	Travoprost	-6.9962
MOlport_LH1_Approved_FDA_library.maegz:210	925.460	Lapatinib Ditosylate	-6.9933
MOlport_LH1_Approved_FDA_library.maegz:2050	581.060	Lapatinib	-6.9933
MOlport_LH1_Approved_FDA_library.maegz:1751	406.470	Carvedilol	-6.9912
MOlport_LH1_Approved_FDA_library.maegz:268	500.550	Travoprost	-6.9911
MOlport_LH1_Approved_FDA_library.maegz:1839	522.490	Carvedilol phosphate	-6.9897
MOlport_LH1_Approved_FDA_library.maegz:2049	943.470	Lapatinib ditosylate monohydrate	-6.9897
MOlport_LH1_Approved_FDA_library.maegz:268	500.550	Travoprost	-6.9878
MOlport_LH1_Approved_FDA_library.maegz:742	349.430	Panobinostat	-6.9876
MOlport_LH1_Approved_FDA_library.maegz:268	500.550	Travoprost	-6.9871
MOlport_LH1_Approved_FDA_library.maegz:825	712.220	Neomycin Sulphate B	-6.9858
MOlport_LH1_Approved_FDA_library.maegz:268	500.550	Travoprost	-6.9843
MOlport_LH1_Approved_FDA_library.maegz:2093	375.870	Haloperidol	-6.9836
MOlport_LH1_Approved_FDA_library.maegz:1251	400.490	Ifenprodil Tartrate	-6.9833
MOlport_LH1_Approved_FDA_library.maegz:268	500.550	Travoprost	-6.9831
MOlport_LH1_Approved_FDA_library.maegz:1597	396.580	Casanthranol	-6.9769
MOlport_LH1_Approved_FDA_library.maegz:268	500.550	Travoprost	-6.9753
MOlport_LH1_Approved_FDA_library.maegz:265	711.870	Indinavir sulfate	-6.9747
MOlport_LH1_Approved_FDA_library.maegz:825	712.220	Neomycin Sulphate B	-6.9737
MOlport_LH1_Approved_FDA_library.maegz:1201	467.510	Tobramycin	-6.9732
MOlport_LH1_Approved_FDA_library.maegz:825	712.220	Neomycin Sulphate B	-6.9731
MOlport_LH1_Approved_FDA_library.maegz:1451	908.870	Neomycin sulfate	-6.9718
MOlport_LH1_Approved_FDA_library.maegz:605	594.520	Proanthocyanidins	-6.9706
MOlport_LH1_Approved_FDA_library.maegz:268	500.550	Travoprost	-6.9706
MOlport_LH1_Approved_FDA_library.maegz:265	711.870	Indinavir sulfate	-6.9701
MOlport_LH1_Approved_FDA_library.maegz:1020	391.470	Rizatriptan benzoate	-6.9682

MOlport_LH1_Approved_FDA_library.maegz:1337	473.560	Nafronyl oxalate	-6.9676
MOlport_LH1_Approved_FDA_library.maegz:1597	396.580	Casanthranol	-6.9666
MOlport_LH1_Approved_FDA_library.maegz:605	594.520	Proanthocyanidins	-6.9656
MOlport_LH1_Approved_FDA_library.maegz:268	500.550	Travoprost	-6.9638
MOlport_LH1_Approved_FDA_library.maegz:1225	1385.430	Sisomicin Sulfate	-6.9634
MOlport_LH1_Approved_FDA_library.maegz:1251	400.490	Ifenprodil Tartrate	-6.9632
MOlport_LH1_Approved_FDA_library.maegz:1451	908.870	Neomycin sulfate	-6.9609
MOlport_LH1_Approved_FDA_library.maegz:605	594.520	Proanthocyanidins	-6.9592
MOlport_LH1_Approved_FDA_library.maegz:1451	908.870	Neomycin sulfate	-6.9591
MOlport_LH1_Approved_FDA_library.maegz:808	270.240	Apigenin	-6.9575
MOlport_LH1_Approved_FDA_library.maegz:605	594.520	Proanthocyanidins	-6.9573
MOlport_LH1_Approved_FDA_library.maegz:1597	396.580	Casanthranol	-6.957
MOlport_LH1_Approved_FDA_library.maegz:1597	396.580	Casanthranol	-6.9568
MOlport_LH1_Approved_FDA_library.maegz:607	464.380	Hyperoside	-6.9566
MOlport_LH1_Approved_FDA_library.maegz:612	416.380	Puerarin	-6.9563
MOlport_LH1_Approved_FDA_library.maegz:92	482.440	Silymarin	-6.956
MOlport_LH1_Approved_FDA_library.maegz:823	417.810	Pexidartinib	-6.956
MOlport_LH1_Approved_FDA_library.maegz:757	447.880	Elvitegravir	-6.9512
MOlport_LH1_Approved_FDA_library.maegz:1317	523.970	Penfluridol	-6.9485
MOlport_LH1_Approved_FDA_library.maegz:1761	516.570	Proflavine Hemisulfate	-6.9479
MOlport_LH1_Approved_FDA_library.maegz:1597	396.580	Casanthranol	-6.9464
MOlport_LH1_Approved_FDA_library.maegz:1337	473.560	Nafronyl oxalate	-6.9464
MOlport_LH1_Approved_FDA_library.maegz:1979	508.630	Argatroban	-6.9463
MOlport_LH1_Approved_FDA_library.maegz:268	500.550	Travoprost	-6.9462
MOlport_LH1_Approved_FDA_library.maegz:1839	522.490	Carvedilol phosphate	-6.9425
MOlport_LH1_Approved_FDA_library.maegz:1751	406.470	Carvedilol	-6.9404
MOlport_LH1_Approved_FDA_library.maegz:136	518.410	Fenticonazole Nitrate	-6.9401
MOlport_LH1_Approved_FDA_library.maegz:265	711.870	Indinavir sulfate	-6.9388
MOlport_LH1_Approved_FDA_library.maegz:605	594.520	Proanthocyanidins	-6.9378
MOlport_LH1_Approved_FDA_library.maegz:1337	473.560	Nafronyl oxalate	-6.9343
MOlport_LH1_Approved_FDA_library.maegz:825	712.220	Neomycin Sulphate B	-6.934
MOlport_LH1_Approved_FDA_library.maegz:1317	523.970	Penfluridol	-6.9327
MOlport_LH1_Approved_FDA_library.maegz:605	594.520	Proanthocyanidins	-6.9315
MOlport_LH1_Approved_FDA_library.maegz:772	437.470	Isavuconazole	-6.9284
MOlport_LH1_Approved_FDA_library.maegz:268	500.550	Travoprost	-6.9237
MOlport_LH1_Approved_FDA_library.maegz:1378	720.780	Netilmicin sulfate	-6.9201
MOlport_LH1_Approved_FDA_library.maegz:1451	908.870	Neomycin sulfate	-6.9165
MOlport_LH1_Approved_FDA_library.maegz:1079	752.760	Capastat sulfate	-6.9124
MOlport_LH1_Approved_FDA_library.maegz:265	711.870	Indinavir sulfate	-6.9033
MOlport_LH1_Approved_FDA_library.maegz:451	814.840	Isavuconazonium sulfate	-6.903
MOlport_LH1_Approved_FDA_library.maegz:1721	342.690	Sertraline hydrochloride	-6.8955
MOlport_LH1_Approved_FDA_library.maegz:58	578.590	Netupitant	-6.8949
MOlport_LH1_Approved_FDA_library.maegz:930	408.330	Trazodone hydrochloride	-6.8948

MOlport_LH1_Approved_FDA_library.maegz:1223	480.430	Trifluoperazine dihydrochloride	-6.8937
MOlport_LH1_Approved_FDA_library.maegz:268	500.550	Travoprost	-6.8912
MOlport_LH1_Approved_FDA_library.maegz:268	500.550	Travoprost	-6.8895
MOlport_LH1_Approved_FDA_library.maegz:755	483.950	Avanafil	-6.888
MOlport_LH1_Approved_FDA_library.maegz:857	444.520	Canagliflozin	-6.8863
MOlport_LH1_Approved_FDA_library.maegz:268	500.550	Travoprost	-6.8845
MOlport_LH1_Approved_FDA_library.maegz:92	482.440	Silymarin	-6.8836
MOlport_LH1_Approved_FDA_library.maegz:268	500.550	Travoprost	-6.8801
MOlport_LH1_Approved_FDA_library.maegz:1582	582.580	Kanamycin sulfate	-6.8791
MOlport_LH1_Approved_FDA_library.maegz:1597	396.580	Casanthranol	-6.8753
MOlport_LH1_Approved_FDA_library.maegz:1020	391.470	Rizatriptan benzoate	-6.8748
MOlport_LH1_Approved_FDA_library.maegz:92	482.440	Silymarin	-6.8739
MOlport_LH1_Approved_FDA_library.maegz:1597	396.580	Casanthranol	-6.8728
MOlport_LH1_Approved_FDA_library.maegz:605	594.520	Proanthocyanidins	-6.8717
MOlport_LH1_Approved_FDA_library.maegz:265	711.870	Indinavir sulfate	-6.8712
MOlport_LH1_Approved_FDA_library.maegz:1597	396.580	Casanthranol	-6.8705
MOlport_LH1_Approved_FDA_library.maegz:1597	396.580	Casanthranol	-6.8689
MOlport_LH1_Approved_FDA_library.maegz:1318	713.710	Paromomycin Sulfate	-6.8688
MOlport_LH1_Approved_FDA_library.maegz:1597	396.580	Casanthranol	-6.8673
MOlport_LH1_Approved_FDA_library.maegz:21	534.500	Tenofovir alafenamide fumarate	-6.8648
MOlport_LH1_Approved_FDA_library.maegz:1597	396.580	Casanthranol	-6.8607
MOlport_LH1_Approved_FDA_library.maegz:1684	580.530	Naringin	-6.8602
MOlport_LH1_Approved_FDA_library.maegz:605	594.520	Proanthocyanidins	-6.8591
MOlport_LH1_Approved_FDA_library.maegz:1597	396.580	Casanthranol	-6.8587
MOlport_LH1_Approved_FDA_library.maegz:268	500.550	Travoprost	-6.8585
MOlport_LH1_Approved_FDA_library.maegz:1048	365.860	Ondansetron hydrochloride	-6.8551
MOlport_LH1_Approved_FDA_library.maegz:521	320.390	Niraparib	-6.8548
MOlport_LH1_Approved_FDA_library.maegz:384	326.400	Ajmaline	-6.8543
MOlport_LH1_Approved_FDA_library.maegz:17	398.440	Ademetionine	-6.8514
MOlport_LH1_Approved_FDA_library.maegz:265	711.870	Indinavir sulfate	-6.8511
MOlport_LH1_Approved_FDA_library.maegz:59	492.590	Niraparib tosylate	-6.8503
MOlport_LH1_Approved_FDA_library.maegz:268	500.550	Travoprost	-6.8493
MOlport_LH1_Approved_FDA_library.maegz:265	711.870	Indinavir sulfate	-6.848
MOlport_LH1_Approved_FDA_library.maegz:1493	460.440	Oxytetracycline	-6.8477
MOlport_LH1_Approved_FDA_library.maegz:268	500.550	Travoprost	-6.8476
MOlport_LH1_Approved_FDA_library.maegz:605	594.520	Proanthocyanidins	-6.8462
MOlport_LH1_Approved_FDA_library.maegz:1451	908.870	Neomycin sulfate	-6.8462
MOlport_LH1_Approved_FDA_library.maegz:825	712.220	Neomycin Sulphate B	-6.8452
MOlport_LH1_Approved_FDA_library.maegz:1597	396.580	Casanthranol	-6.8448
MOlport_LH1_Approved_FDA_library.maegz:1251	400.490	Ifenprodil Tartrate	-6.8306
MOlport_LH1_Approved_FDA_library.maegz:1721	342.690	Sertraline hydrochloride	-6.8297

MOlport_LH1_Approved_FDA_library.maegz:605	594.520	Proanthocyanidins	-6.8295
MOlport_LH1_Approved_FDA_library.maegz:265	711.870	Indinavir sulfate	-6.829
MOlport_LH1_Approved_FDA_library.maegz:1358	247.720	Isoprenaline hydrochloride	-6.8239
MOlport_LH1_Approved_FDA_library.maegz:1597	396.580	Casanthranol	-6.8234
MOlport_LH1_Approved_FDA_library.maegz:1684	580.530	Naringin	-6.823
MOlport_LH1_Approved_FDA_library.maegz:2058	510.440	Fluphenazine hydrochloride	-6.8194
MOlport_LH1_Approved_FDA_library.maegz:1597	396.580	Casanthranol	-6.8178
MOlport_LH1_Approved_FDA_library.maegz:689	505.050	Ivabradine hydrochloride	-6.8157
MOlport_LH1_Approved_FDA_library.maegz:268	500.550	Travoprost	-6.8147
MOlport_LH1_Approved_FDA_library.maegz:825	712.220	Neomycin Sulphate B	-6.8134
MOlport_LH1_Approved_FDA_library.maegz:1146	273.710	Carprofen	-6.8132
MOlport_LH1_Approved_FDA_library.maegz:1597	396.580	Casanthranol	-6.809
MOlport_LH1_Approved_FDA_library.maegz:1082	474.790	Butoconazole nitrate	-6.8085
MOlport_LH1_Approved_FDA_library.maegz:825	712.220	Neomycin Sulphate B	-6.8078
MOlport_LH1_Approved_FDA_library.maegz:755	483.950	Avanafil	-6.8073
MOlport_LH1_Approved_FDA_library.maegz:1451	908.870	Neomycin sulfate	-6.8065
MOlport_LH1_Approved_FDA_library.maegz:2068	377.520	Famprofazone	-6.8056
MOlport_LH1_Approved_FDA_library.maegz:612	416.380	Puerarin	-6.8055
MOlport_LH1_Approved_FDA_library.maegz:1597	396.580	Casanthranol	-6.8015
MOlport_LH1_Approved_FDA_library.maegz:1973	432.370	Pantoprazole Sodium Hydrate	-6.7974
MOlport_LH1_Approved_FDA_library.maegz:265	711.870	Indinavir sulfate	-6.7924
MOlport_LH1_Approved_FDA_library.maegz:237	306.370	S-Ruxolitinib	-6.791
MOlport_LH1_Approved_FDA_library.maegz:9	497.520	Levomefolate calcium	-6.7887
MOlport_LH1_Approved_FDA_library.maegz:857	444.520	Canagliflozin	-6.7846
MOlport_LH1_Approved_FDA_library.maegz:333	612.660	Cefpirome sulfate	-6.7816
MOlport_LH1_Approved_FDA_library.maegz:1451	908.870	Neomycin sulfate	-6.7815
MOlport_LH1_Approved_FDA_library.maegz:265	711.870	Indinavir sulfate	-6.7772
MOlport_LH1_Approved_FDA_library.maegz:1493	460.440	Oxytetracycline	-6.7721
MOlport_LH1_Approved_FDA_library.maegz:1229	552.550	Ribostamycin sulfate	-6.7708
MOlport_LH1_Approved_FDA_library.maegz:70	543.460	Bitopertin	-6.7703
MOlport_LH1_Approved_FDA_library.maegz:1009	720.960	Ritonavir	-6.7702
MOlport_LH1_Approved_FDA_library.maegz:465	582.970	Ivosidenib	-6.7694
MOlport_LH1_Approved_FDA_library.maegz:1396	585.600	Amikacin	-6.7627
MOlport_LH1_Approved_FDA_library.maegz:268	500.550	Travoprost	-6.7597
MOlport_LH1_Approved_FDA_library.maegz:605	594.520	Proanthocyanidins	-6.7596
MOlport_LH1_Approved_FDA_library.maegz:265	711.870	Indinavir sulfate	-6.7592
MOlport_LH1_Approved_FDA_library.maegz:265	711.870	Indinavir sulfate	-6.7583
MOlport_LH1_Approved_FDA_library.maegz:531	683.680	Amikacin Sulfate Salt	-6.7581
MOlport_LH1_Approved_FDA_library.maegz:1228	515.990	Nicardipine hydrochloride	-6.7578
MOlport_LH1_Approved_FDA_library.maegz:1948	683.620	Manidipine dihydrochloride	-6.7548
MOlport_LH1_Approved_FDA_library.maegz:268	500.550	Travoprost	-6.7548
MOlport_LH1_Approved_FDA_library.maegz:1392	369.360	R-(+)-Lansoprazole	-6.7546

MOlport_LH1_Approved_FDA_library.maegz:1674	369.360	Lansoprazole	-6.7546
MOlport_LH1_Approved_FDA_library.maegz:112	610.700	Manidipine	-6.7527
MOlport_LH1_Approved_FDA_library.maegz:268	500.550	Travoprost	-6.7486
MOlport_LH1_Approved_FDA_library.maegz:17	398.440	Ademetionine	-6.748
MOlport_LH1_Approved_FDA_library.maegz:268	500.550	Travoprost	-6.7468
MOlport_LH1_Approved_FDA_library.maegz:20	575.700	Pyrvinium pamoate	-6.7458
MOlport_LH1_Approved_FDA_library.maegz:1318	713.710	Paromomycin Sulfate	-6.7451
MOlport_LH1_Approved_FDA_library.maegz:70	543.460	Bitopertin	-6.7381
MOlport_LH1_Approved_FDA_library.maegz:605	594.520	Proanthocyanidins	-6.7255
MOlport_LH1_Approved_FDA_library.maegz:268	500.550	Travoprost	-6.7241
MOlport_LH1_Approved_FDA_library.maegz:1391	527.520	Hygromycin B	-6.7241
MOlport_LH1_Approved_FDA_library.maegz:1391	527.520	Hygromycin B	-6.7208
MOlport_LH1_Approved_FDA_library.maegz:2093	375.870	Haloperidol	-6.7183
MOlport_LH1_Approved_FDA_library.maegz:265	711.870	Indinavir sulfate	-6.7174
MOlport_LH1_Approved_FDA_library.maegz:1201	467.510	Tobramycin	-6.716
MOlport_LH1_Approved_FDA_library.maegz:1597	396.580	Casanthranol	-6.7135
MOlport_LH1_Approved_FDA_library.maegz:605	594.520	Proanthocyanidins	-6.7128
MOlport_LH1_Approved_FDA_library.maegz:92	482.440	Silymarin	-6.7125
MOlport_LH1_Approved_FDA_library.maegz:2002	230.670	Diazoxide	-6.7109
MOlport_LH1_Approved_FDA_library.maegz:891	482.440	Silibinin	-6.7103
MOlport_LH1_Approved_FDA_library.maegz:582	372.410	Arctigenin	-6.7103
MOlport_LH1_Approved_FDA_library.maegz:1794	337.350	Linezolid	-6.7071
MOlport_LH1_Approved_FDA_library.maegz:654	555.500	Bedaquiline	-6.6921
MOlport_LH1_Approved_FDA_library.maegz:268	500.550	Travoprost	-6.6908
MOlport_LH1_Approved_FDA_library.maegz:1597	396.580	Casanthranol	-6.6877
MOlport_LH1_Approved_FDA_library.maegz:265	711.870	Indinavir sulfate	-6.6875
MOlport_LH1_Approved_FDA_library.maegz:256	663.890	Nelfinavir Mesylate	-6.6868
MOlport_LH1_Approved_FDA_library.maegz:612	416.380	Puerarin	-6.676
MOlport_LH1_Approved_FDA_library.maegz:1804	430.370	Bicalutamide	-6.6741
MOlport_LH1_Approved_FDA_library.maegz:265	711.870	Indinavir sulfate	-6.6726
MOlport_LH1_Approved_FDA_library.maegz:1317	523.970	Penfluridol	-6.6722
MOlport_LH1_Approved_FDA_library.maegz:1684	580.530	Naringin	-6.6717
MOlport_LH1_Approved_FDA_library.maegz:1747	345.790	Fluoxetine hydrochloride	-6.6714
MOlport_LH1_Approved_FDA_library.maegz:1748	309.330	Fluoxetine	-6.6714
MOlport_LH1_Approved_FDA_library.maegz:265	711.870	Indinavir sulfate	-6.6685
MOlport_LH1_Approved_FDA_library.maegz:1009	720.960	Ritonavir	-6.6652
MOlport_LH1_Approved_FDA_library.maegz:265	711.870	Indinavir sulfate	-6.6627
MOlport_LH1_Approved_FDA_library.maegz:9	497.520	Levomefolate calcium	-6.6603
MOlport_LH1_Approved_FDA_library.maegz:607	464.380	Hyperoside	-6.6599
MOlport_LH1_Approved_FDA_library.maegz:608	270.240	Aloe-emodin	-6.659
MOlport_LH1_Approved_FDA_library.maegz:265	711.870	Indinavir sulfate	-6.6577
MOlport_LH1_Approved_FDA_library.maegz:905	321.330	Famciclovir	-6.6522
MOlport_LH1_Approved_FDA_library.maegz:825	712.220	Neomycin Sulphate B	-6.6503

MOlport_LH1_Approved_FDA_library.maegz:243	411.470	Entospletinib	-6.649
MOlport_LH1_Approved_FDA_library.maegz:265	711.870	Indinavir sulfate	-6.6485
MOlport_LH1_Approved_FDA_library.maegz:1493	460.440	Oxytetracycline	-6.6461
MOlport_LH1_Approved_FDA_library.maegz:452	330.290	Jaceosidin	-6.6452
MOlport_LH1_Approved_FDA_library.maegz:1396	585.600	Amikacin	-6.6448
MOlport_LH1_Approved_FDA_library.maegz:742	349.430	Panobinostat	-6.6438
MOlport_LH1_Approved_FDA_library.maegz:453	446.550	Erdafitinib	-6.6424
MOlport_LH1_Approved_FDA_library.maegz:192	638.820	Lonafarnib	-6.6408
MOlport_LH1_Approved_FDA_library.maegz:265	711.870	Indinavir sulfate	-6.6407
MOlport_LH1_Approved_FDA_library.maegz:362	427.410	Cariprazine	-6.6395
MOlport_LH1_Approved_FDA_library.maegz:857	444.520	Canagliflozin	-6.6394
MOlport_LH1_Approved_FDA_library.maegz:951	409.430	Ezetimibe	-6.6385
MOlport_LH1_Approved_FDA_library.maegz:1597	396.580	Casanthranol	-6.6383
MOlport_LH1_Approved_FDA_library.maegz:531	683.680	Amikacin Sulfate Salt	-6.6335
MOlport_LH1_Approved_FDA_library.maegz:605	594.520	Proanthocyanidins	-6.63
MOlport_LH1_Approved_FDA_library.maegz:1582	582.580	Kanamycin sulfate	-6.6269
MOlport_LH1_Approved_FDA_library.maegz:605	594.520	Proanthocyanidins	-6.6238
MOlport_LH1_Approved_FDA_library.maegz:1148	208.260	Bendazol	-6.6235
MOlport_LH1_Approved_FDA_library.maegz:1032	365.840	Indapamide	-6.6223
MOlport_LH1_Approved_FDA_library.maegz:1146	273.710	Carprofen	-6.6123
MOlport_LH1_Approved_FDA_library.maegz:265	711.870	Indinavir sulfate	-6.6063
MOlport_LH1_Approved_FDA_library.maegz:366	389.900	Cyclothiazide	-6.606
MOlport_LH1_Approved_FDA_library.maegz:951	409.430	Ezetimibe	-6.6049
MOlport_LH1_Approved_FDA_library.maegz:769	469.550	Almotriptan Malate	-6.6029
MOlport_LH1_Approved_FDA_library.maegz:265	711.870	Indinavir sulfate	-6.6005
MOlport_LH1_Approved_FDA_library.maegz:777	520.500	VX661	-6.5998
MOlport_LH1_Approved_FDA_library.maegz:905	321.330	Famciclovir	-6.5992
MOlport_LH1_Approved_FDA_library.maegz:718	393.870	Cinacalcet hydrochloride	-6.5961
MOlport_LH1_Approved_FDA_library.maegz:268	500.550	Travoprost	-6.586
MOlport_LH1_Approved_FDA_library.maegz:265	711.870	Indinavir sulfate	-6.5722
MOlport_LH1_Approved_FDA_library.maegz:268	500.550	Travoprost	-6.5683
MOlport_LH1_Approved_FDA_library.maegz:687	415.570	Bimatoprost	-6.563
MOlport_LH1_Approved_FDA_library.maegz:808	270.240	Apigenin	-6.563
MOlport_LH1_Approved_FDA_library.maegz:268	500.550	Travoprost	-6.5629
MOlport_LH1_Approved_FDA_library.maegz:268	500.550	Travoprost	-6.5604
MOlport_LH1_Approved_FDA_library.maegz:1597	396.580	Casanthranol	-6.5601
MOlport_LH1_Approved_FDA_library.maegz:1032	365.840	Indapamide	-6.5478
MOlport_LH1_Approved_FDA_library.maegz:605	594.520	Proanthocyanidins	-6.5445
MOlport_LH1_Approved_FDA_library.maegz:643	679.560	Thymopentin	-6.5444
MOlport_LH1_Approved_FDA_library.maegz:605	594.520	Proanthocyanidins	-6.5381
MOlport_LH1_Approved_FDA_library.maegz:268	500.550	Travoprost	-6.537
MOlport_LH1_Approved_FDA_library.maegz:1181	441.530	Vilazodone	-6.5326
MOlport_LH1_Approved_FDA_library.maegz:268	500.550	Travoprost	-6.5312

MOlport_LH1_Approved_FDA_library.maegz:605	594.520	Proanthocyanidins	-6.5282
MOlport_LH1_Approved_FDA_library.maegz:701	238.190	Rufinamide	-6.5213
MOlport_LH1_Approved_FDA_library.maegz:605	594.520	Proanthocyanidins	-6.5209
MOlport_LH1_Approved_FDA_library.maegz:268	500.550	Travoprost	-6.5182
MOlport_LH1_Approved_FDA_library.maegz:770	398.430	Omarigliptin	-6.5167
MOlport_LH1_Approved_FDA_library.maegz:9	497.520	Levomefolate calcium	-6.512
MOlport_LH1_Approved_FDA_library.maegz:268	500.550	Travoprost	-6.5088
MOlport_LH1_Approved_FDA_library.maegz:969	288.390	Estriol	-6.5063
MOlport_LH1_Approved_FDA_library.maegz:1597	396.580	Casanthranol	-6.4947
MOlport_LH1_Approved_FDA_library.maegz:969	288.390	Estriol	-6.4902
MOlport_LH1_Approved_FDA_library.maegz:605	594.520	Proanthocyanidins	-6.4892
MOlport_LH1_Approved_FDA_library.maegz:605	594.520	Proanthocyanidins	-6.4874
MOlport_LH1_Approved_FDA_library.maegz:2006	582.650	Azelnidipine	-6.4865
MOlport_LH1_Approved_FDA_library.maegz:1967	396.580	Depofemin	-6.4635
MOlport_LH1_Approved_FDA_library.maegz:1597	396.580	Casanthranol	-6.4623
MOlport_LH1_Approved_FDA_library.maegz:268	500.550	Travoprost	-6.4613
MOlport_LH1_Approved_FDA_library.maegz:1597	396.580	Casanthranol	-6.4569
MOlport_LH1_Approved_FDA_library.maegz:265	711.870	Indinavir sulfate	-6.4561
MOlport_LH1_Approved_FDA_library.maegz:9	497.520	Levomefolate calcium	-6.4448
MOlport_LH1_Approved_FDA_library.maegz:162	511.500	Calcium Levofolinate	-6.4393
MOlport_LH1_Approved_FDA_library.maegz:1378	720.780	Netilmicin sulfate	-6.4327
MOlport_LH1_Approved_FDA_library.maegz:1681	244.240	Piceatannol	-6.4293
MOlport_LH1_Approved_FDA_library.maegz:874	512.900	Doxycycline hyclate	-6.4285
MOlport_LH1_Approved_FDA_library.maegz:229	434.540	Ribociclib	-6.4276
MOlport_LH1_Approved_FDA_library.maegz:1013	1219.600	Aliskiren hemifumarate	-6.4254
MOlport_LH1_Approved_FDA_library.maegz:265	711.870	Indinavir sulfate	-6.4214
MOlport_LH1_Approved_FDA_library.maegz:1681	244.240	Piceatannol	-6.4201
MOlport_LH1_Approved_FDA_library.maegz:605	594.520	Proanthocyanidins	-6.4127
MOlport_LH1_Approved_FDA_library.maegz:605	594.520	Proanthocyanidins	-6.4042
MOlport_LH1_Approved_FDA_library.maegz:1058	415.820	Acemetacin	-6.3836
MOlport_LH1_Approved_FDA_library.maegz:605	594.520	Proanthocyanidins	-6.3813
MOlport_LH1_Approved_FDA_library.maegz:9	497.520	Levomefolate calcium	-6.373
MOlport_LH1_Approved_FDA_library.maegz:1746	209.250	Minoxidil	-6.3659
MOlport_LH1_Approved_FDA_library.maegz:66	401.860	Fenoldopam mesylate	-6.365
MOlport_LH1_Approved_FDA_library.maegz:265	711.870	Indinavir sulfate	-6.3599
MOlport_LH1_Approved_FDA_library.maegz:231	571.550	Baloxavir marboxil	-6.3564
MOlport_LH1_Approved_FDA_library.maegz:1039	586.650	Fosinopril sodium	-6.3511
MOlport_LH1_Approved_FDA_library.maegz:268	500.550	Travoprost	-6.3457
MOlport_LH1_Approved_FDA_library.maegz:268	500.550	Travoprost	-6.3337
MOlport_LH1_Approved_FDA_library.maegz:265	711.870	Indinavir sulfate	-6.3311
MOlport_LH1_Approved_FDA_library.maegz:605	594.520	Proanthocyanidins	-6.3208
MOlport_LH1_Approved_FDA_library.maegz:268	500.550	Travoprost	-6.3166
MOlport_LH1_Approved_FDA_library.maegz:605	594.520	Proanthocyanidins	-6.3099

MOlport_LH1_Approved_FDA_library.maegz:757	447.880	Elvitegravir	-6.3039
MOlport_LH1_Approved_FDA_library.maegz:1675	446.520	Pravastatin sodium	-6.2992
MOlport_LH1_Approved_FDA_library.maegz:9	497.520	Levomefolate calcium	-6.2924
MOlport_LH1_Approved_FDA_library.maegz:740	404.450	Ipragliflozin	-6.2749
MOlport_LH1_Approved_FDA_library.maegz:757	447.880	Elvitegravir	-6.2637
MOlport_LH1_Approved_FDA_library.maegz:1316	421.830	Sarafloxacin hydrochloride	-6.2533
MOlport_LH1_Approved_FDA_library.maegz:265	711.870	Indinavir sulfate	-6.2441
MOlport_LH1_Approved_FDA_library.maegz:1225	1385.430	Sisomicin Sulfate	-6.2311
MOlport_LH1_Approved_FDA_library.maegz:1071	297.740	Hydrochlorothiazide	-6.2221
MOlport_LH1_Approved_FDA_library.maegz:605	594.520	Proanthocyanidins	-6.22
MOlport_LH1_Approved_FDA_library.maegz:277	461.460	Prulifloxacin	-6.2104
MOlport_LH1_Approved_FDA_library.maegz:687	415.570	Bimatoprost	-6.2031
MOlport_LH1_Approved_FDA_library.maegz:605	594.520	Proanthocyanidins	-6.202
MOlport_LH1_Approved_FDA_library.maegz:66	401.860	Fenoldopam mesylate	-6.2014
MOlport_LH1_Approved_FDA_library.maegz:233	679.850	Telaprevir	-6.2005
MOlport_LH1_Approved_FDA_library.maegz:9	497.520	Levomefolate calcium	-6.1905
MOlport_LH1_Approved_FDA_library.maegz:880	481.540	Rosuvastatin	-6.1792
MOlport_LH1_Approved_FDA_library.maegz:772	437.470	Isavuconazole	-6.1715
MOlport_LH1_Approved_FDA_library.maegz:265	711.870	Indinavir sulfate	-6.1654
MOlport_LH1_Approved_FDA_library.maegz:1683	238.290	10,11-Dihydrocarbamazepine	-6.1525
MOlport_LH1_Approved_FDA_library.maegz:312	768.765	Cefotiam Hexetil Hydrochloride	-6.1493
MOlport_LH1_Approved_FDA_library.maegz:70	543.460	Bitopertin	-6.1409
MOlport_LH1_Approved_FDA_library.maegz:1305	182.180	Dacarbazine	-6.1398
MOlport_LH1_Approved_FDA_library.maegz:1675	446.520	Pravastatin sodium	-6.1259
MOlport_LH1_Approved_FDA_library.maegz:1974	256.090	Anagrelide	-6.1161
MOlport_LH1_Approved_FDA_library.maegz:173	292.550	Anagrelide HCl	-6.1158
MOlport_LH1_Approved_FDA_library.maegz:265	711.870	Indinavir sulfate	-6.1128
MOlport_LH1_Approved_FDA_library.maegz:746	555.550	Rociletinib	-6.0988
MOlport_LH1_Approved_FDA_library.maegz:161	645.670	Cefoperazone	-6.0885
MOlport_LH1_Approved_FDA_library.maegz:66	401.860	Fenoldopam mesylate	-6.0817
MOlport_LH1_Approved_FDA_library.maegz:122	667.680	Isepamicin sulfate	-6.0612
MOlport_LH1_Approved_FDA_library.maegz:777	520.500	VX661	-6.0311
MOlport_LH1_Approved_FDA_library.maegz:605	594.520	Proanthocyanidins	-6.0186
MOlport_LH1_Approved_FDA_library.maegz:265	711.870	Indinavir sulfate	-6.0037
MOlport_LH1_Approved_FDA_library.maegz:361	767.890	Pentagastrin	-5.9075
MOlport_LH1_Approved_FDA_library.maegz:265	711.870	Indinavir sulfate	-5.9
MOlport_LH1_Approved_FDA_library.maegz:605	594.520	Proanthocyanidins	-5.8973
MOlport_LH1_Approved_FDA_library.maegz:338	519.680	Boceprevir	-5.887
MOlport_LH1_Approved_FDA_library.maegz:265	711.870	Indinavir sulfate	-5.7866
MOlport_LH1_Approved_FDA_library.maegz:2006	582.650	Azelnidipine	-5.779
MOlport_LH1_Approved_FDA_library.maegz:629	718.590	Salvianolic acid B	-5.7205

MOlport_LH1_Approved_FDA_library.maegz:757	447.880	Elvitegravir	-5.6634
MOlport_LH1_Approved_FDA_library.maegz:522	765.890	Paritaprevir	-5.6248
MOlport_LH1_Approved_FDA_library.maegz:122	667.680	Isepamicin sulfate	-5.5984
MOlport_LH1_Approved_FDA_library.maegz:265	711.870	Indinavir sulfate	-5.5619
MOlport_LH1_Approved_FDA_library.maegz:746	555.550	Rociletinib	-5.4438
MOlport_LH1_Approved_FDA_library.maegz:531	683.680	Amikacin Sulfate Salt	-5.4257
MOlport_LH1_Approved_FDA_library.maegz:1396	585.600	Amikacin	-5.3323
MOlport_LH1_Approved_FDA_library.maegz:265	711.870	Indinavir sulfate	-5.3258
MOlport_LH1_Approved_FDA_library.maegz:265	711.870	Indinavir sulfate	-5.3181
MOlport_LH1_Approved_FDA_library.maegz:265	711.870	Indinavir sulfate	-5.0565
MOlport_LH1_Approved_FDA_library.maegz:629	718.590	Salvianolic acid B	-4.9541
MOlport_LH1_Approved_FDA_library.maegz:522	765.890	Paritaprevir	-4.8745

Table S4. VS1 and VS2 for DSI-poised targeting PARG_4B1H_A

Entry Name	MW	Catalog ID	Energy in silico
VS1			
DSi PoisedLibrary_1680186639697.541	244.078	Z1267773765	-7.7018
VS2			
DSi PoisedLibrary_1680186639697.351	241.085	Z26781964	-7.5373
DSi PoisedLibrary_1680186639697.541	244.078	Z1267773765	-7.393
DSi PoisedLibrary_1680186639697.260	171.101	Z90122368	-7.2341
DSi PoisedLibrary_1680186639697.541	244.078	Z1267773765	-7.2328
DSi PoisedLibrary_1680186639697.351	241.085	Z26781964	-7.1781
DSi PoisedLibrary_1680186639697.696	166.099	Z1255402624	-7.1623
DSi PoisedLibrary_1680186639697.678	223.229	Z198194396	-7.1408
DSi PoisedLibrary_1680186639697.294	217.122	Z26781943	-7.1154
DSi PoisedLibrary_1680186639697.541	244.078	Z1267773765	-7.0947
DSi PoisedLibrary_1680186639697.137	190.111	Z285141302	-7.0912
DSi PoisedLibrary_1680186639697.657	244.264	Z44609285	-7.0674
DSi PoisedLibrary_1680186639697.260	171.101	Z90122368	-7.049
DSi PoisedLibrary_1680186639697.230	165.115	Z104473836	-7.0132
DSi PoisedLibrary_1680186639697.103	248.321	Z54748186	-6.9977
DSi PoisedLibrary_1680186639697.812	195.101	Z57627041	-6.9848
DSi PoisedLibrary_1680186639697.63	299.729	Z1004825200	-6.9663
DSi PoisedLibrary_1680186639697.44	205.041	Z363993198	-6.9487
DSi PoisedLibrary_1680186639697.487	247.293	Z33545544	-6.9409
DSi PoisedLibrary_1680186639697.424	217.122	Z26781952	-6.9193
DSi PoisedLibrary_1680186639697.260	171.101	Z90122368	-6.917
DSi PoisedLibrary_1680186639697.186	193.085	Z198195770	-6.8936
DSi PoisedLibrary_1680186639697.437	248.116	Z32327641	-6.8868
DSi PoisedLibrary_1680186639697.230	165.115	Z104473836	-6.8665
DSi PoisedLibrary_1680186639697.424	217.122	Z26781952	-6.809

DSi PoisedLibrary_1680186639697.424	217.122	Z26781952	-6.8059
DSi PoisedLibrary_1680186639697.678	223.229	Z198194396	-6.8055
DSi PoisedLibrary_1680186639697.137	190.111	Z285141302	-6.8011
DSi PoisedLibrary_1680186639697.295	240.103	Z1457921102	-6.773
DSi PoisedLibrary_1680186639697.553	179.095	Z56960248	-6.7531
DSi PoisedLibrary_1680186639697.245	241.110	Z33452549	-6.7442
DSi PoisedLibrary_1680186639697.15	228.333	Z131833926	-6.7406
DSi PoisedLibrary_1680186639697.509	239.106	Z1587220559	-6.7282
DSi PoisedLibrary_1680186639697.803	175.075	Z383202616	-6.7261
DSi PoisedLibrary_1680186639697.103	248.321	Z54748186	-6.7239
DSi PoisedLibrary_1680186639697.261	170.106	Z32399802	-6.7087
DSi PoisedLibrary_1680186639697.294	217.122	Z26781943	-6.7085
DSi PoisedLibrary_1680186639697.19	219.101	Z111529496	-6.6832
DSi PoisedLibrary_1680186639697.591	244.119	Z56792776	-6.682
DSi PoisedLibrary_1680186639697.19	219.101	Z111529496	-6.6743
DSi PoisedLibrary_1680186639697.687	204.126	Z381408780	-6.6716
DSi PoisedLibrary_1680186639697.503	242.316	Z223388508	-6.6679
DSi PoisedLibrary_1680186639697.645	162.091	Z234898257	-6.6672
DSi PoisedLibrary_1680186639697.803	175.075	Z383202616	-6.6588
DSi PoisedLibrary_1680186639697.137	190.111	Z285141302	-6.6394
DSi PoisedLibrary_1680186639697.19	219.101	Z111529496	-6.6142
DSi PoisedLibrary_1680186639697.607	186.137	Z317064236	-6.6069
DSi PoisedLibrary_1680186639697.330	233.142	Z419995480	-6.5981
DSi PoisedLibrary_1680186639697.503	242.316	Z223388508	-6.5951
DSi PoisedLibrary_1680186639697.63	299.729	Z1004825200	-6.5934
DSi PoisedLibrary_1680186639697.714	243.057	Z53860899	-6.5795
DSi PoisedLibrary_1680186639697.295	240.103	Z1457921102	-6.5542
DSi PoisedLibrary_1680186639697.209	163.100	Z270979606	-6.5357
DSi PoisedLibrary_1680186639697.541	244.078	Z1267773765	-6.5325
DSi PoisedLibrary_1680186639697.788	186.049	Z55671900	-6.532
DSi PoisedLibrary_1680186639697.301	247.268	Z1203107138	-6.521
DSi PoisedLibrary_1680186639697.788	186.049	Z55671900	-6.519
DSi PoisedLibrary_1680186639697.159	260.090	Z1818332938	-6.5186
DSi PoisedLibrary_1680186639697.60	186.049	Z53836105	-6.5177
DSi PoisedLibrary_1680186639697.230	165.115	Z104473836	-6.5117
DSi PoisedLibrary_1680186639697.28	265.111	Z85893853	-6.5002
DSi PoisedLibrary_1680186639697.11	199.040	Z19727416	-6.4942
DSi PoisedLibrary_1680186639697.261	170.106	Z32399802	-6.4922
DSi PoisedLibrary_1680186639697.11	199.040	Z19727416	-6.4856
DSi PoisedLibrary_1680186639697.344	212.264	Z1796224493	-6.4799
DSi PoisedLibrary_1680186639697.310	180.051	Z802540802	-6.4735
DSi PoisedLibrary_1680186639697.378	206.142	Z332382842	-6.4716
DSi PoisedLibrary_1680186639697.541	244.078	Z1267773765	-6.4668

DSi PoisedLibrary_1680186639697.336	194.025	Z56837087	-6.4607
DSi PoisedLibrary_1680186639697.696	166.099	Z1255402624	-6.4533
DSi PoisedLibrary_1680186639697.45	224.095	Z235449082	-6.4525
DSi PoisedLibrary_1680186639697.823	191.069	Z26794305	-6.4514
DSi PoisedLibrary_1680186639697.378	206.142	Z332382842	-6.4501
DSi PoisedLibrary_1680186639697.462	209.087	Z1318254096	-6.4481
DSi PoisedLibrary_1680186639697.750	176.095	Z1192341021	-6.4476
DSi PoisedLibrary_1680186639697.714	243.057	Z53860899	-6.4403
DSi PoisedLibrary_1680186639697.215	160.100	Z54750053	-6.4401
DSi PoisedLibrary_1680186639697.417	203.106	Z1416571195	-6.4365
DSi PoisedLibrary_1680186639697.553	179.095	Z56960248	-6.4153
DSi PoisedLibrary_1680186639697.595	230.266	Z275181224	-6.4132
DSi PoisedLibrary_1680186639697.378	206.142	Z332382842	-6.4089
DSi PoisedLibrary_1680186639697.788	186.049	Z55671900	-6.4086
DSi PoisedLibrary_1680186639697.278	241.010	Z1551999220	-6.3963
DSi PoisedLibrary_1680186639697.645	162.091	Z234898257	-6.3942
DSi PoisedLibrary_1680186639697.261	170.106	Z32399802	-6.3867
DSi PoisedLibrary_1680186639697.717	192.163	Z86129854	-6.3862
DSi PoisedLibrary_1680186639697.30	220.067	Z1134990241	-6.3834
DSi PoisedLibrary_1680186639697.88	224.098	Z1101755952	-6.3776
DSi PoisedLibrary_1680186639697.88	224.098	Z1101755952	-6.3699
DSi PoisedLibrary_1680186639697.602	153.059	Z1198169663	-6.3653
DSi PoisedLibrary_1680186639697.663	228.057	Z30932204	-6.3627
DSi PoisedLibrary_1680186639697.576	194.058	Z56900769	-6.3472
DSi PoisedLibrary_1680186639697.137	190.111	Z285141302	-6.3444
DSi PoisedLibrary_1680186639697.432	277.243	Z364577298	-6.3444
DSi PoisedLibrary_1680186639697.435	244.068	Z56953052	-6.3233
DSi PoisedLibrary_1680186639697.234	210.046	Z119990900	-6.3232
DSi PoisedLibrary_1680186639697.749	179.095	Z33452106	-6.3148
DSi PoisedLibrary_1680186639697.15	228.333	Z131833926	-6.3144
DSi PoisedLibrary_1680186639697.351	241.085	Z26781964	-6.3043
DSi PoisedLibrary_1680186639697.310	180.051	Z802540802	-6.3027
DSi PoisedLibrary_1680186639697.539	159.090	Z1436457631	-6.3019
DSi PoisedLibrary_1680186639697.172	204.126	Z1206890404	-6.3013
DSi PoisedLibrary_1680186639697.209	163.100	Z270979606	-6.3006
DSi PoisedLibrary_1680186639697.553	179.095	Z56960248	-6.2909
DSi PoisedLibrary_1680186639697.750	176.095	Z1192341021	-6.2843
DSi PoisedLibrary_1680186639697.812	195.101	Z57627041	-6.2776
DSi PoisedLibrary_1680186639697.151	190.074	Z19739650	-6.2737
DSi PoisedLibrary_1680186639697.437	248.116	Z32327641	-6.2715
DSi PoisedLibrary_1680186639697.605	198.056	Z1696091761	-6.2707
DSi PoisedLibrary_1680186639697.698	180.126	Z1008185878	-6.2685
DSi PoisedLibrary_1680186639697.576	194.058	Z56900769	-6.2678

DSi PoisedLibrary_1680186639697.608	213.249	Z2272040604	-6.2635
DSi PoisedLibrary_1680186639697.98	204.101	Z131516158	-6.263
DSi PoisedLibrary_1680186639697.162	137.048	Z19684186	-6.2616
DSi PoisedLibrary_1680186639697.208	189.077	Z56960214	-6.247
DSi PoisedLibrary_1680186639697.344	212.264	Z1796224493	-6.2462
DSi PoisedLibrary_1680186639697.687	204.126	Z381408780	-6.2461
DSi PoisedLibrary_1680186639697.750	176.095	Z1192341021	-6.2416
DSi PoisedLibrary_1680186639697.399	238.326	Z32399948	-6.2376
DSi PoisedLibrary_1680186639697.457	226.091	Z26333448	-6.2316
DSi PoisedLibrary_1680186639697.399	238.326	Z32399948	-6.2259
DSi PoisedLibrary_1680186639697.607	186.137	Z317064236	-6.2166
DSi PoisedLibrary_1680186639697.789	210.248	Z1929757385	-6.2134
DSi PoisedLibrary_1680186639697.607	186.137	Z317064236	-6.2016
DSi PoisedLibrary_1680186639697.550	217.057	Z45497619	-6.1972
DSi PoisedLibrary_1680186639697.217	170.106	Z220816104	-6.1708
DSi PoisedLibrary_1680186639697.749	179.095	Z33452106	-6.1702
DSi PoisedLibrary_1680186639697.789	210.248	Z1929757385	-6.1697
DSi PoisedLibrary_1680186639697.731	150.079	Z1203252645	-6.1655
DSi PoisedLibrary_1680186639697.2	227.163	Z44603686	-6.1567
DSi PoisedLibrary_1680186639697.344	212.264	Z1796224493	-6.1419
DSi PoisedLibrary_1680186639697.398	205.085	Z1162778919	-6.1366
DSi PoisedLibrary_1680186639697.562	163.081	Z1198316457	-6.0739
DSi PoisedLibrary_1680186639697.398	205.085	Z1162778919	-6.0474
DSi PoisedLibrary_1680186639697.789	210.248	Z1929757385	-6.0406
DSi PoisedLibrary_1680186639697.717	192.163	Z86129854	-6.0317
DSi PoisedLibrary_1680186639697.526	170.142	Z317064394	-6.0118
DSi PoisedLibrary_1680186639697.431	205.256	Z1401276297	-6.0013
DSi PoisedLibrary_1680186639697.760	175.042	Z608065044	-5.9852
DSi PoisedLibrary_1680186639697.802	179.062	Z425757818	-5.9499
DSi PoisedLibrary_1680186639697.217	170.106	Z220816104	-5.9103
DSi PoisedLibrary_1680186639697.403	189.115	Z28870646	-5.8559
DSi PoisedLibrary_1680186639697.562	163.081	Z1198316457	-5.8422
DSi PoisedLibrary_1680186639697.605	198.056	Z1696091761	-5.7132
DSi PoisedLibrary_1680186639697.246	228.126	Z44095027	-5.6106
DSi PoisedLibrary_1680186639697.817	173.167	Z1250093582	-5.3623

Table S5. VS1 and VS2 for PDB fragment library targeting PARG_4B1H_A

Compound	MW	Energy in silico
VS1		
L-tryptophanamide	203.106	-8.2228
VS2		
Umbelliferone	162.032	-8.1233

L-tryptophanamide	203.106	-7.8392
6-Hydroxy-2-benzothiazolesulfonamide	229.982	-7.5327
Tyramine	137.084	-7.527
Tyramine	137.084	-7.5261
Tryptamine	160.100	-7.3575
Umbelliferone	162.032	-7.2874
Tryptamine	160.100	-7.2428
tyrosinamide	180.090	-7.1318
5-Fluorouridine	262.060	-7.104
Thymidine	242.090	-7.0201
Cytosine deoxyribonucleoside		-7.0118
Cytosine deoxyribonucleoside		-7.0075
Thymidine	242.090	-6.9623
Thymidine	242.090	-6.8487
L-tryptophanamide	203.106	-6.6795
4'-Hydroxyacetophenone	136.052	-6.6259
1H-Benzo[d]imidazole-2-sulfonamide	197.026	-6.5775
(1H-Indol-3-yl)acetic acid	175.063	-6.5645
N-(2-aminoethyl)isoquinoline-5-sulfonamide	251.073	-6.5554
2-Amino-1H-benzo[d]imidazol-5-ol	149.059	-6.552
nabumetone	228.115	-6.5427
5-methoxyindole-acetate	205.074	-6.5416
(1H-Indol-3-yl)acetic acid	175.063	-6.5374
4'-Hydroxyacetophenone	136.052	-6.53
5-methoxyindole-acetate	205.074	-6.4309
Serotonin	176.095	-6.401
2-(carbamoylamino)-3-phenylpropanoic acid	208.085	-6.3704
1H-Indazole	118.053	-6.3189
3-Carboxyindole	161.048	-6.2739
(1H-Indol-3-yl)acetic acid	175.063	-6.0752

Table S6. VS1 and VS2 for LH1 FDA Approved drugs targeting PARG_4B1H_A

Compound	Name	MolWt	Energy in silico
VS1			
MOlport_LH1_Approved_FDA_library.maegz:1358	Isoprenaline hydrochloride	247.720	-11.2592
VS2			
MOlport_LH1_Approved_FDA_library.maegz:361	Pentagastrin	767.890	-9.9514
MOlport_LH1_Approved_FDA_library.maegz:361	Pentagastrin	767.890	-9.4803
MOlport_LH1_Approved_FDA_library.maegz:434	Goserelin acetate	1329.460	-9.2869
MOlport_LH1_Approved_FDA_library.maegz:361	Pentagastrin	767.890	-8.7934
MOlport_LH1_Approved_FDA_library.maegz:1181	Vilazodone	441.530	-8.6698

MOlport_LH1_Approved_FDA_library.maegz:92	Silymarin	482.440	-8.6252
MOlport_LH1_Approved_FDA_library.maegz:92	Silymarin	482.440	-8.568
MOlport_LH1_Approved_FDA_library.maegz:930	Trazodone hydrochloride	408.330	-8.5315
MOlport_LH1_Approved_FDA_library.maegz:449	Lifitegrast	615.480	-8.4915
MOlport_LH1_Approved_FDA_library.maegz:92	Silymarin	482.440	-8.3736
MOlport_LH1_Approved_FDA_library.maegz:92	Silymarin	482.440	-8.3522
MOlport_LH1_Approved_FDA_library.maegz:265	Indinavir sulfate	711.870	-8.2696
MOlport_LH1_Approved_FDA_library.maegz:92	Silymarin	482.440	-8.2642
MOlport_LH1_Approved_FDA_library.maegz:451	Isavuconazonium sulfate	814.840	-8.2423
MOlport_LH1_Approved_FDA_library.maegz:92	Silymarin	482.440	-8.2307
MOlport_LH1_Approved_FDA_library.maegz:268	Travoprost	500.550	-8.1778
MOlport_LH1_Approved_FDA_library.maegz:847	Carfilzomib	719.910	-8.1521
MOlport_LH1_Approved_FDA_library.maegz:268	Travoprost	500.550	-8.1516
MOlport_LH1_Approved_FDA_library.maegz:237	S-Ruxolitinib	306.370	-8.1376
MOlport_LH1_Approved_FDA_library.maegz:576	Scopoletin	192.170	-8.1098
MOlport_LH1_Approved_FDA_library.maegz:268	Travoprost	500.550	-8.0637
MOlport_LH1_Approved_FDA_library.maegz:434	Goserelin acetate	1329.460	-8.0394
MOlport_LH1_Approved_FDA_library.maegz:706	Mocetinostat	396.440	-8.0197
MOlport_LH1_Approved_FDA_library.maegz:289	Cabozantinib hydrochloride	537.960	-8.0045
MOlport_LH1_Approved_FDA_library.maegz:653	Cabozantinib	501.500	-8.0045
MOlport_LH1_Approved_FDA_library.maegz:846	Cabozantinib Malate	635.590	-8.0045
MOlport_LH1_Approved_FDA_library.maegz:268	Travoprost	500.550	-8.0017
MOlport_LH1_Approved_FDA_library.maegz:237	S-Ruxolitinib	306.370	-7.9943
MOlport_LH1_Approved_FDA_library.maegz:547	Ruxolitinib (INCB-18424) phosphate	404.360	-7.9751
MOlport_LH1_Approved_FDA_library.maegz:843	Ruxolitinib	306.370	-7.9751
MOlport_LH1_Approved_FDA_library.maegz:407	Tucidinostat	390.410	-7.9434
MOlport_LH1_Approved_FDA_library.maegz:265	Indinavir sulfate	711.870	-7.9094
MOlport_LH1_Approved_FDA_library.maegz:268	Travoprost	500.550	-7.9083
MOlport_LH1_Approved_FDA_library.maegz:92	Silymarin	482.440	-7.881
MOlport_LH1_Approved_FDA_library.maegz:547	Ruxolitinib (INCB-18424) phosphate	404.360	-7.8809
MOlport_LH1_Approved_FDA_library.maegz:843	Ruxolitinib	306.370	-7.8809
MOlport_LH1_Approved_FDA_library.maegz:1358	Isoprenaline hydrochloride	247.720	-7.8512
MOlport_LH1_Approved_FDA_library.maegz:847	Carfilzomib	719.910	-7.8406
MOlport_LH1_Approved_FDA_library.maegz:265	Indinavir sulfate	711.870	-7.8325
MOlport_LH1_Approved_FDA_library.maegz:265	Indinavir sulfate	711.870	-7.8211
MOlport_LH1_Approved_FDA_library.maegz:750	Baricitinib?Phosphate	469.410	-7.8199
MOlport_LH1_Approved_FDA_library.maegz:711	Baricitinib	371.420	-7.8184
MOlport_LH1_Approved_FDA_library.maegz:711	Baricitinib	371.420	-7.8153
MOlport_LH1_Approved_FDA_library.maegz:750	Baricitinib?Phosphate	469.410	-7.8153
MOlport_LH1_Approved_FDA_library.maegz:547	Ruxolitinib (INCB-18424) phosphate	404.360	-7.8135

MOlport_LH1_Approved_FDA_library.maegz:843	Ruxolitinib	306.370	-7.8135
MOlport_LH1_Approved_FDA_library.maegz:576	Scopoletin	192.170	-7.8056
MOlport_LH1_Approved_FDA_library.maegz:268	Travoprost	500.550	-7.7915
MOlport_LH1_Approved_FDA_library.maegz:265	Indinavir sulfate	711.870	-7.7596
MOlport_LH1_Approved_FDA_library.maegz:706	Mocetinostat	396.440	-7.7458
MOlport_LH1_Approved_FDA_library.maegz:92	Silymarin	482.440	-7.7378
MOlport_LH1_Approved_FDA_library.maegz:1019	Telbivudine	242.230	-7.7354
MOlport_LH1_Approved_FDA_library.maegz:289	Cabozantinib hydrochloride	537.960	-7.7284
MOlport_LH1_Approved_FDA_library.maegz:653	Cabozantinib	501.500	-7.7284
MOlport_LH1_Approved_FDA_library.maegz:846	Cabozantinib Malate	635.590	-7.7284
MOlport_LH1_Approved_FDA_library.maegz:289	Cabozantinib hydrochloride	537.960	-7.7222
MOlport_LH1_Approved_FDA_library.maegz:653	Cabozantinib	501.500	-7.7222
MOlport_LH1_Approved_FDA_library.maegz:846	Cabozantinib Malate	635.590	-7.7222
MOlport_LH1_Approved_FDA_library.maegz:268	Travoprost	500.550	-7.7196
MOlport_LH1_Approved_FDA_library.maegz:759	Dolutegravir sodium	441.360	-7.7189
MOlport_LH1_Approved_FDA_library.maegz:92	Silymarin	482.440	-7.7173
MOlport_LH1_Approved_FDA_library.maegz:451	Isavuconazonium sulfate	814.840	-7.7089
MOlport_LH1_Approved_FDA_library.maegz:265	Indinavir sulfate	711.870	-7.6962
MOlport_LH1_Approved_FDA_library.maegz:913	Deferoxamine Mesylate	656.790	-7.6813
MOlport_LH1_Approved_FDA_library.maegz:986	Itopride hydrochloride	394.900	-7.6718
MOlport_LH1_Approved_FDA_library.maegz:165	Bisacodyl	361.390	-7.6467
MOlport_LH1_Approved_FDA_library.maegz:268	Travoprost	500.550	-7.6416
MOlport_LH1_Approved_FDA_library.maegz:268	Travoprost	500.550	-7.6292
MOlport_LH1_Approved_FDA_library.maegz:125	Imidapril HCl	441.910	-7.6265
MOlport_LH1_Approved_FDA_library.maegz:1322	Polymyxin B sulfate	1301.560	-7.6147
MOlport_LH1_Approved_FDA_library.maegz:161	Cefoperazone	645.670	-7.6145
MOlport_LH1_Approved_FDA_library.maegz:265	Indinavir sulfate	711.870	-7.6098
MOlport_LH1_Approved_FDA_library.maegz:750	Baricitinib?Phosphate	469.410	-7.6086
MOlport_LH1_Approved_FDA_library.maegz:711	Baricitinib	371.420	-7.608
MOlport_LH1_Approved_FDA_library.maegz:687	Bimatoprost	415.570	-7.6046
MOlport_LH1_Approved_FDA_library.maegz:265	Indinavir sulfate	711.870	-7.6002
MOlport_LH1_Approved_FDA_library.maegz:9	Levomefolate calcium	497.520	-7.5963
MOlport_LH1_Approved_FDA_library.maegz:265	Indinavir sulfate	711.870	-7.5928
MOlport_LH1_Approved_FDA_library.maegz:315	Desmopressin	1069.220	-7.5911
MOlport_LH1_Approved_FDA_library.maegz:265	Indinavir sulfate	711.870	-7.5907
MOlport_LH1_Approved_FDA_library.maegz:265	Indinavir sulfate	711.870	-7.5821
MOlport_LH1_Approved_FDA_library.maegz:1942	Meptazinol hydrochloride	269.810	-7.5746
MOlport_LH1_Approved_FDA_library.maegz:930	Trazodone hydrochloride	408.330	-7.5678
MOlport_LH1_Approved_FDA_library.maegz:268	Travoprost	500.550	-7.5566
MOlport_LH1_Approved_FDA_library.maegz:913	Deferoxamine Mesylate	656.790	-7.5495
MOlport_LH1_Approved_FDA_library.maegz:213	Vilazodone HCl	477.990	-7.5467
MOlport_LH1_Approved_FDA_library.maegz:1804	Bicalutamide	430.370	-7.5448
MOlport_LH1_Approved_FDA_library.maegz:265	Indinavir sulfate	711.870	-7.5444

MOlport_LH1_Approved_FDA_library.maegz:1804	Bicalutamide	430.370	-7.5422
MOlport_LH1_Approved_FDA_library.maegz:2046	Domperidone	425.920	-7.5324
MOlport_LH1_Approved_FDA_library.maegz:407	Tucidinostat	390.410	-7.5317
MOlport_LH1_Approved_FDA_library.maegz:161	Cefoperazone	645.670	-7.5271
MOlport_LH1_Approved_FDA_library.maegz:930	Trazodone hydrochloride	408.330	-7.5267
MOlport_LH1_Approved_FDA_library.maegz:1181	Vilazodone	441.530	-7.5252
MOlport_LH1_Approved_FDA_library.maegz:1067	Deferasirox	373.360	-7.4967
MOlport_LH1_Approved_FDA_library.maegz:265	Indinavir sulfate	711.870	-7.4845
MOlport_LH1_Approved_FDA_library.maegz:847	Carfilzomib	719.910	-7.4807
MOlport_LH1_Approved_FDA_library.maegz:213	Vilazodone HCl	477.990	-7.4784
MOlport_LH1_Approved_FDA_library.maegz:515	Etilefrine hydrochloride	217.690	-7.4756
MOlport_LH1_Approved_FDA_library.maegz:582	Arctigenin	372.410	-7.4625
MOlport_LH1_Approved_FDA_library.maegz:265	Indinavir sulfate	711.870	-7.4591
MOlport_LH1_Approved_FDA_library.maegz:268	Travoprost	500.550	-7.4494
MOlport_LH1_Approved_FDA_library.maegz:2093	Haloperidol	375.870	-7.4422
MOlport_LH1_Approved_FDA_library.maegz:265	Indinavir sulfate	711.870	-7.4417
MOlport_LH1_Approved_FDA_library.maegz:593	Fraxetin	208.170	-7.4404
MOlport_LH1_Approved_FDA_library.maegz:92	Silymarin	482.440	-7.4363
MOlport_LH1_Approved_FDA_library.maegz:863	Empagliflozin	450.910	-7.4346
MOlport_LH1_Approved_FDA_library.maegz:17	Ademetionine	398.440	-7.429
MOlport_LH1_Approved_FDA_library.maegz:1322	Polymyxin B sulfate	1301.560	-7.4279
MOlport_LH1_Approved_FDA_library.maegz:913	Deferoxamine Mesylate	656.790	-7.4186
MOlport_LH1_Approved_FDA_library.maegz:1694	Tioxolone	168.170	-7.4132
MOlport_LH1_Approved_FDA_library.maegz:2093	Haloperidol	375.870	-7.4127
MOlport_LH1_Approved_FDA_library.maegz:92	Silymarin	482.440	-7.41
MOlport_LH1_Approved_FDA_library.maegz:1417	Droperidol	379.430	-7.4078
MOlport_LH1_Approved_FDA_library.maegz:1174	Synephrine	167.210	-7.4061
MOlport_LH1_Approved_FDA_library.maegz:1174	Synephrine	167.210	-7.4036
MOlport_LH1_Approved_FDA_library.maegz:759	Dolutegravir sodium	441.360	-7.4006
MOlport_LH1_Approved_FDA_library.maegz:268	Travoprost	500.550	-7.3985
MOlport_LH1_Approved_FDA_library.maegz:245	Anidulafungin	1140.240	-7.394
MOlport_LH1_Approved_FDA_library.maegz:2047	Lafutidine	431.550	-7.3939
MOlport_LH1_Approved_FDA_library.maegz:1485	Pyrithioxin	368.470	-7.3905
MOlport_LH1_Approved_FDA_library.maegz:593	Fraxetin	208.170	-7.388
MOlport_LH1_Approved_FDA_library.maegz:268	Travoprost	500.550	-7.3869
MOlport_LH1_Approved_FDA_library.maegz:268	Travoprost	500.550	-7.385
MOlport_LH1_Approved_FDA_library.maegz:315	Desmopressin	1069.220	-7.3821
MOlport_LH1_Approved_FDA_library.maegz:268	Travoprost	500.550	-7.3738
MOlport_LH1_Approved_FDA_library.maegz:1804	Bicalutamide	430.370	-7.3716
MOlport_LH1_Approved_FDA_library.maegz:265	Indinavir sulfate	711.870	-7.363
MOlport_LH1_Approved_FDA_library.maegz:406	Bictegravir	449.380	-7.3579
MOlport_LH1_Approved_FDA_library.maegz:158	Clevudine	260.220	-7.355
MOlport_LH1_Approved_FDA_library.maegz:265	Indinavir sulfate	711.870	-7.3524

MOlport_LH1_Approved_FDA_library.maegz:265	Indinavir sulfate	711.870	-7.3502
MOlport_LH1_Approved_FDA_library.maegz:265	Indinavir sulfate	711.870	-7.3494
MOlport_LH1_Approved_FDA_library.maegz:213	Vilazodone HCl	477.990	-7.3459
MOlport_LH1_Approved_FDA_library.maegz:161	Cefoperazone	645.670	-7.3455
MOlport_LH1_Approved_FDA_library.maegz:1092	Trifluridine	296.200	-7.3438
MOlport_LH1_Approved_FDA_library.maegz:1322	Polymyxin B sulfate	1301.560	-7.3434
MOlport_LH1_Approved_FDA_library.maegz:687	Bimatoprost	415.570	-7.341
MOlport_LH1_Approved_FDA_library.maegz:312	Cefotiam Hexetil Hydrochloride	768.765	-7.3385
MOlport_LH1_Approved_FDA_library.maegz:923	Alprostadil	354.480	-7.332
MOlport_LH1_Approved_FDA_library.maegz:417	Edoxudine	256.260	-7.3298
MOlport_LH1_Approved_FDA_library.maegz:317	Prostaglandin E2 (PGE2)	352.470	-7.3247
MOlport_LH1_Approved_FDA_library.maegz:268	Travoprost	500.550	-7.3116
MOlport_LH1_Approved_FDA_library.maegz:266	Edrophonium chloride	201.690	-7.3115
MOlport_LH1_Approved_FDA_library.maegz:268	Travoprost	500.550	-7.3058
MOlport_LH1_Approved_FDA_library.maegz:1631	Isoliquiritigenin	256.250	-7.3038
MOlport_LH1_Approved_FDA_library.maegz:1631	Isoliquiritigenin	256.250	-7.3035
MOlport_LH1_Approved_FDA_library.maegz:907	Dopamine hydrochloride	189.640	-7.3029
MOlport_LH1_Approved_FDA_library.maegz:268	Travoprost	500.550	-7.3026
MOlport_LH1_Approved_FDA_library.maegz:1681	Piceatannol	244.240	-7.3022
MOlport_LH1_Approved_FDA_library.maegz:1092	Trifluridine	296.200	-7.2983
MOlport_LH1_Approved_FDA_library.maegz:1174	Synephrine	167.210	-7.298
MOlport_LH1_Approved_FDA_library.maegz:265	Indinavir sulfate	711.870	-7.2974
MOlport_LH1_Approved_FDA_library.maegz:265	Indinavir sulfate	711.870	-7.2973
MOlport_LH1_Approved_FDA_library.maegz:1346	DL-Adrenaline Hydrochloride	219.670	-7.2943
MOlport_LH1_Approved_FDA_library.maegz:18	Avatrombopag	649.650	-7.2907
MOlport_LH1_Approved_FDA_library.maegz:1092	Trifluridine	296.200	-7.2844
MOlport_LH1_Approved_FDA_library.maegz:1631	Isoliquiritigenin	256.250	-7.2824
MOlport_LH1_Approved_FDA_library.maegz:17	Ademetionine	398.440	-7.2803
MOlport_LH1_Approved_FDA_library.maegz:268	Travoprost	500.550	-7.2793
MOlport_LH1_Approved_FDA_library.maegz:17	Ademetionine	398.440	-7.277
MOlport_LH1_Approved_FDA_library.maegz:417	Edoxudine	256.260	-7.2745
MOlport_LH1_Approved_FDA_library.maegz:200	Lomitapide	692.710	-7.2737
MOlport_LH1_Approved_FDA_library.maegz:265	Indinavir sulfate	711.870	-7.2718
MOlport_LH1_Approved_FDA_library.maegz:265	Indinavir sulfate	711.870	-7.27
MOlport_LH1_Approved_FDA_library.maegz:265	Indinavir sulfate	711.870	-7.269
MOlport_LH1_Approved_FDA_library.maegz:1067	Deferasirox	373.360	-7.2681
MOlport_LH1_Approved_FDA_library.maegz:268	Travoprost	500.550	-7.2463
MOlport_LH1_Approved_FDA_library.maegz:268	Travoprost	500.550	-7.2456
MOlport_LH1_Approved_FDA_library.maegz:268	Travoprost	500.550	-7.2387
MOlport_LH1_Approved_FDA_library.maegz:812	Fulvestrant	606.770	-7.2366
MOlport_LH1_Approved_FDA_library.maegz:675	Etofibrate	363.790	-7.2206
MOlport_LH1_Approved_FDA_library.maegz:237	S-Ruxolitinib	306.370	-7.2199

MOlport_LH1_Approved_FDA_library.maegz:763	Darunavir	547.660	-7.2149
MOlport_LH1_Approved_FDA_library.maegz:488	Darunavir?Ethanolate	593.740	-7.2146
MOlport_LH1_Approved_FDA_library.maegz:1853	Fipexide	388.850	-7.2145
MOlport_LH1_Approved_FDA_library.maegz:1174	Synephrine	167.210	-7.213
MOlport_LH1_Approved_FDA_library.maegz:406	Bictegravir	449.380	-7.2112
MOlport_LH1_Approved_FDA_library.maegz:265	Indinavir sulfate	711.870	-7.2098
MOlport_LH1_Approved_FDA_library.maegz:266	Edrophonium chloride	201.690	-7.1994
MOlport_LH1_Approved_FDA_library.maegz:434	Goserelin acetate	1329.460	-7.1955
MOlport_LH1_Approved_FDA_library.maegz:1253	Rivaroxaban	435.880	-7.1941
MOlport_LH1_Approved_FDA_library.maegz:759	Dolutegravir sodium	441.360	-7.1892
MOlport_LH1_Approved_FDA_library.maegz:2093	Haloperidol	375.870	-7.1892
MOlport_LH1_Approved_FDA_library.maegz:265	Indinavir sulfate	711.870	-7.1878
MOlport_LH1_Approved_FDA_library.maegz:268	Travoprost	500.550	-7.1867
MOlport_LH1_Approved_FDA_library.maegz:1760	L-Tryptophan	204.230	-7.1823
MOlport_LH1_Approved_FDA_library.maegz:868	Apixaban	459.500	-7.1792
MOlport_LH1_Approved_FDA_library.maegz:230	Dolutegravir	419.380	-7.1775
MOlport_LH1_Approved_FDA_library.maegz:682	Pimozide	461.550	-7.1679
MOlport_LH1_Approved_FDA_library.maegz:1322	Polymyxin B sulfate	1301.560	-7.1675
MOlport_LH1_Approved_FDA_library.maegz:266	Edrophonium chloride	201.690	-7.1608
MOlport_LH1_Approved_FDA_library.maegz:417	Edoxudine	256.260	-7.159
MOlport_LH1_Approved_FDA_library.maegz:265	Indinavir sulfate	711.870	-7.1573
MOlport_LH1_Approved_FDA_library.maegz:1358	Isoprenaline hydrochloride	247.720	-7.1566
MOlport_LH1_Approved_FDA_library.maegz:1174	Synephrine	167.210	-7.1501
MOlport_LH1_Approved_FDA_library.maegz:1560	Acetohexamide	324.400	-7.148
MOlport_LH1_Approved_FDA_library.maegz:870	Rabeprazole Related Compound E	343.440	-7.1462
MOlport_LH1_Approved_FDA_library.maegz:576	Scopoletin	192.170	-7.142
MOlport_LH1_Approved_FDA_library.maegz:1751	Carvedilol	406.470	-7.1335
MOlport_LH1_Approved_FDA_library.maegz:1839	Carvedilol phosphate	522.490	-7.1332
MOlport_LH1_Approved_FDA_library.maegz:1346	DL-Adrenaline Hydrochloride	219.670	-7.1331
MOlport_LH1_Approved_FDA_library.maegz:759	Dolutegravir sodium	441.360	-7.1246
MOlport_LH1_Approved_FDA_library.maegz:268	Travoprost	500.550	-7.1208
MOlport_LH1_Approved_FDA_library.maegz:265	Indinavir sulfate	711.870	-7.1208
MOlport_LH1_Approved_FDA_library.maegz:268	Travoprost	500.550	-7.1184
MOlport_LH1_Approved_FDA_library.maegz:2093	Haloperidol	375.870	-7.1179
MOlport_LH1_Approved_FDA_library.maegz:230	Dolutegravir	419.380	-7.1136
MOlport_LH1_Approved_FDA_library.maegz:682	Pimozide	461.550	-7.1128
MOlport_LH1_Approved_FDA_library.maegz:603	Daphnetin	178.140	-7.105
MOlport_LH1_Approved_FDA_library.maegz:907	Dopamine hydrochloride	189.640	-7.1004
MOlport_LH1_Approved_FDA_library.maegz:868	Apixaban	459.500	-7.1002
MOlport_LH1_Approved_FDA_library.maegz:1358	Isoprenaline hydrochloride	247.720	-7.093
MOlport_LH1_Approved_FDA_library.maegz:268	Travoprost	500.550	-7.092
MOlport_LH1_Approved_FDA_library.maegz:265	Indinavir sulfate	711.870	-7.0909

MOlport_LH1_Approved_FDA_library.maegz:515	Etilefrine hydrochloride	217.690	-7.0853
MOlport_LH1_Approved_FDA_library.maegz:268	Travoprost	500.550	-7.083
MOlport_LH1_Approved_FDA_library.maegz:926	Lopinavir	628.800	-7.0806
MOlport_LH1_Approved_FDA_library.maegz:265	Indinavir sulfate	711.870	-7.0806
MOlport_LH1_Approved_FDA_library.maegz:268	Travoprost	500.550	-7.0799
MOlport_LH1_Approved_FDA_library.maegz:515	Etilefrine hydrochloride	217.690	-7.0717
MOlport_LH1_Approved_FDA_library.maegz:1992	Cefpiramide acid	612.640	-7.0682
MOlport_LH1_Approved_FDA_library.maegz:686	Fingolimod hydrochloride	343.930	-7.0671
MOlport_LH1_Approved_FDA_library.maegz:1895	Mepiroxol	125.130	-7.0668
MOlport_LH1_Approved_FDA_library.maegz:268	Travoprost	500.550	-7.0667
MOlport_LH1_Approved_FDA_library.maegz:200	Lomitapide	692.710	-7.0662
MOlport_LH1_Approved_FDA_library.maegz:716	Tenofovir Disoproxil Fumarate	635.510	-7.0654
MOlport_LH1_Approved_FDA_library.maegz:1346	DL-Adrenaline Hydrochloride	219.670	-7.0619
MOlport_LH1_Approved_FDA_library.maegz:4	Temocapil	476.600	-7.0591
MOlport_LH1_Approved_FDA_library.maegz:17	Ademetionine	398.440	-7.0555
MOlport_LH1_Approved_FDA_library.maegz:1931	Pioglitazone	356.440	-7.0521
MOlport_LH1_Approved_FDA_library.maegz:315	Desmopressin	1069.220	-7.0518
MOlport_LH1_Approved_FDA_library.maegz:245	Anidulafungin	1140.240	-7.0504
MOlport_LH1_Approved_FDA_library.maegz:1560	Acetohexamide	324.400	-7.0489
MOlport_LH1_Approved_FDA_library.maegz:330	Ertugliflozin	436.880	-7.0477
MOlport_LH1_Approved_FDA_library.maegz:17	Ademetionine	398.440	-7.0474
MOlport_LH1_Approved_FDA_library.maegz:812	Fulvestrant	606.770	-7.0467
MOlport_LH1_Approved_FDA_library.maegz:265	Indinavir sulfate	711.870	-7.0374
MOlport_LH1_Approved_FDA_library.maegz:1441	Floxuridine	246.190	-7.0335
MOlport_LH1_Approved_FDA_library.maegz:1020	Rizatriptan benzoate	391.470	-7.0306
MOlport_LH1_Approved_FDA_library.maegz:1804	Bicalutamide	430.370	-7.027
MOlport_LH1_Approved_FDA_library.maegz:126	Ibutilide Fumarate	885.230	-7.027
MOlport_LH1_Approved_FDA_library.maegz:944	Doxifluridine	246.190	-7.0253
MOlport_LH1_Approved_FDA_library.maegz:1259	Temozolomide	194.150	-7.0157
MOlport_LH1_Approved_FDA_library.maegz:158	Clevudine	260.220	-7.0147
MOlport_LH1_Approved_FDA_library.maegz:268	Travoprost	500.550	-7.009
MOlport_LH1_Approved_FDA_library.maegz:437	Afobazole hydrochloride	343.870	-7.0022
MOlport_LH1_Approved_FDA_library.maegz:158	Clevudine	260.220	-7.0008
MOlport_LH1_Approved_FDA_library.maegz:265	Indinavir sulfate	711.870	-7.0006
MOlport_LH1_Approved_FDA_library.maegz:687	Bimatoprost	415.570	-6.9962
MOlport_LH1_Approved_FDA_library.maegz:1804	Bicalutamide	430.370	-6.9951
MOlport_LH1_Approved_FDA_library.maegz:1009	Ritonavir	720.960	-6.9891
MOlport_LH1_Approved_FDA_library.maegz:1474	Ambroxol	378.100	-6.9881
MOlport_LH1_Approved_FDA_library.maegz:211	Ambroxol hydrochloride	414.560	-6.9872
MOlport_LH1_Approved_FDA_library.maegz:1019	Telbivudine	242.230	-6.9783
MOlport_LH1_Approved_FDA_library.maegz:317	Prostaglandin E2 (PGE2)	352.470	-6.9754
MOlport_LH1_Approved_FDA_library.maegz:1760	L-Tryptophan	204.230	-6.9754

MOlport_LH1_Approved_FDA_library.maegz:245	Anidulafungin	1140.240	-6.9727
MOlport_LH1_Approved_FDA_library.maegz:1635	Brivudine	333.140	-6.9712
MOlport_LH1_Approved_FDA_library.maegz:1240	Sulpiride	341.430	-6.9646
MOlport_LH1_Approved_FDA_library.maegz:1560	Acetohexamide	324.400	-6.9637
MOlport_LH1_Approved_FDA_library.maegz:1931	Pioglitazone	356.440	-6.9594
MOlport_LH1_Approved_FDA_library.maegz:1240	Sulpiride	341.430	-6.9551
MOlport_LH1_Approved_FDA_library.maegz:1232	Levosulpiride	341.430	-6.9542
MOlport_LH1_Approved_FDA_library.maegz:686	Fingolimod hydrochloride	343.930	-6.9536
MOlport_LH1_Approved_FDA_library.maegz:19	Revefenacin	597.750	-6.9524
MOlport_LH1_Approved_FDA_library.maegz:265	Indinavir sulfate	711.870	-6.9464
MOlport_LH1_Approved_FDA_library.maegz:39	Norepinephrine	169.180	-6.9447
MOlport_LH1_Approved_FDA_library.maegz:1513	Noradrenaline bitartrate	319.260	-6.9447
MOlport_LH1_Approved_FDA_library.maegz:1180	Nepafenac	254.280	-6.9446
MOlport_LH1_Approved_FDA_library.maegz:1938	Ranolazine dihydrochloride	500.460	-6.9443
MOlport_LH1_Approved_FDA_library.maegz:987	Tegaserod maleate	417.460	-6.9436
MOlport_LH1_Approved_FDA_library.maegz:515	Etilefrine hydrochloride	217.690	-6.9411
MOlport_LH1_Approved_FDA_library.maegz:100	Ranolazine	427.540	-6.9407
MOlport_LH1_Approved_FDA_library.maegz:268	Travoprost	500.550	-6.937
MOlport_LH1_Approved_FDA_library.maegz:1322	Polymyxin B sulfate	1301.560	-6.9286
MOlport_LH1_Approved_FDA_library.maegz:15	Teriflunomide	270.210	-6.928
MOlport_LH1_Approved_FDA_library.maegz:1112	Stavudine	224.210	-6.9278
MOlport_LH1_Approved_FDA_library.maegz:265	Indinavir sulfate	711.870	-6.922
MOlport_LH1_Approved_FDA_library.maegz:265	Indinavir sulfate	711.870	-6.9214
MOlport_LH1_Approved_FDA_library.maegz:265	Indinavir sulfate	711.870	-6.9203
MOlport_LH1_Approved_FDA_library.maegz:1635	Brivudine	333.140	-6.9184
MOlport_LH1_Approved_FDA_library.maegz:546	Teriflunomide	270.210	-6.9181
MOlport_LH1_Approved_FDA_library.maegz:245	Anidulafungin	1140.240	-6.9128
MOlport_LH1_Approved_FDA_library.maegz:303	Cephapirin Sodium	445.450	-6.9119
MOlport_LH1_Approved_FDA_library.maegz:268	Travoprost	500.550	-6.9112
MOlport_LH1_Approved_FDA_library.maegz:125	Imidapril HCl	441.910	-6.902
MOlport_LH1_Approved_FDA_library.maegz:863	Empagliflozin	450.910	-6.902
MOlport_LH1_Approved_FDA_library.maegz:265	Indinavir sulfate	711.870	-6.9002
MOlport_LH1_Approved_FDA_library.maegz:268	Travoprost	500.550	-6.8938
MOlport_LH1_Approved_FDA_library.maegz:1694	Tioxolone	168.170	-6.8793
MOlport_LH1_Approved_FDA_library.maegz:107	Naratriptan	371.930	-6.8793
MOlport_LH1_Approved_FDA_library.maegz:265	Indinavir sulfate	711.870	-6.8786
MOlport_LH1_Approved_FDA_library.maegz:588	Apremilast	460.500	-6.8772
MOlport_LH1_Approved_FDA_library.maegz:1346	DL-Adrenaline Hydrochloride	219.670	-6.8696
MOlport_LH1_Approved_FDA_library.maegz:265	Indinavir sulfate	711.870	-6.8687
MOlport_LH1_Approved_FDA_library.maegz:1760	L-Tryptophan	204.230	-6.8619
MOlport_LH1_Approved_FDA_library.maegz:434	Goserelin acetate	1329.460	-6.8591
MOlport_LH1_Approved_FDA_library.maegz:310	Zofenopril (calcium)	897.170	-6.8558
MOlport_LH1_Approved_FDA_library.maegz:1804	Bicalutamide	430.370	-6.8547

MOlport_LH1_Approved_FDA_library.maegz:1240	Sulpiride	341.430	-6.854
MOlport_LH1_Approved_FDA_library.maegz:1232	Levosulpiride	341.430	-6.8528
MOlport_LH1_Approved_FDA_library.maegz:265	Indinavir sulfate	711.870	-6.8492
MOlport_LH1_Approved_FDA_library.maegz:588	Apremilast	460.500	-6.8486
MOlport_LH1_Approved_FDA_library.maegz:265	Indinavir sulfate	711.870	-6.8481
MOlport_LH1_Approved_FDA_library.maegz:265	Indinavir sulfate	711.870	-6.8467
MOlport_LH1_Approved_FDA_library.maegz:265	Indinavir sulfate	711.870	-6.8464
MOlport_LH1_Approved_FDA_library.maegz:1843	Aniracetam	219.240	-6.845
MOlport_LH1_Approved_FDA_library.maegz:265	Indinavir sulfate	711.870	-6.8424
MOlport_LH1_Approved_FDA_library.maegz:811	Carbidopa Hydrate	244.240	-6.8371
MOlport_LH1_Approved_FDA_library.maegz:1174	Synephrine	167.210	-6.8357
MOlport_LH1_Approved_FDA_library.maegz:1358	Isoprenaline hydrochloride	247.720	-6.8331
MOlport_LH1_Approved_FDA_library.maegz:17	Ademetionine	398.440	-6.8249
MOlport_LH1_Approved_FDA_library.maegz:1322	Polymyxin B sulfate	1301.560	-6.823
MOlport_LH1_Approved_FDA_library.maegz:312	Cefotiam Hexetil Hydrochloride	768.765	-6.8127
MOlport_LH1_Approved_FDA_library.maegz:265	Indinavir sulfate	711.870	-6.8114
MOlport_LH1_Approved_FDA_library.maegz:141	Enalapril Maleate	492.520	-6.8084
MOlport_LH1_Approved_FDA_library.maegz:265	Indinavir sulfate	711.870	-6.8062
MOlport_LH1_Approved_FDA_library.maegz:15	Teriflunomide	270.210	-6.7925
MOlport_LH1_Approved_FDA_library.maegz:546	Teriflunomide	270.210	-6.7919
MOlport_LH1_Approved_FDA_library.maegz:811	Carbidopa Hydrate	244.240	-6.7892
MOlport_LH1_Approved_FDA_library.maegz:141	Enalapril Maleate	492.520	-6.7885
MOlport_LH1_Approved_FDA_library.maegz:1785	Hydroxyquinoline	145.160	-6.7879
MOlport_LH1_Approved_FDA_library.maegz:317	Prostaglandin E2 (PGE2)	352.470	-6.7848
MOlport_LH1_Approved_FDA_library.maegz:1322	Polymyxin B sulfate	1301.560	-6.7796
MOlport_LH1_Approved_FDA_library.maegz:1346	DL-Adrenaline Hydrochloride	219.670	-6.7734
MOlport_LH1_Approved_FDA_library.maegz:283	MD 69276	207.230	-6.7711
MOlport_LH1_Approved_FDA_library.maegz:268	Travoprost	500.550	-6.747
MOlport_LH1_Approved_FDA_library.maegz:1240	Sulpiride	341.430	-6.7442
MOlport_LH1_Approved_FDA_library.maegz:1490	Chloroxylenol	156.610	-6.7414
MOlport_LH1_Approved_FDA_library.maegz:1346	DL-Adrenaline Hydrochloride	219.670	-6.7402
MOlport_LH1_Approved_FDA_library.maegz:1490	Chloroxylenol	156.610	-6.7399
MOlport_LH1_Approved_FDA_library.maegz:1432	Novobiocin Sodium	634.610	-6.7338
MOlport_LH1_Approved_FDA_library.maegz:17	Ademetionine	398.440	-6.7323
MOlport_LH1_Approved_FDA_library.maegz:923	Alprostadil	354.480	-6.7284
MOlport_LH1_Approved_FDA_library.maegz:265	Indinavir sulfate	711.870	-6.7266
MOlport_LH1_Approved_FDA_library.maegz:265	Indinavir sulfate	711.870	-6.7176
MOlport_LH1_Approved_FDA_library.maegz:1283	Bupropion hydrochloride	276.210	-6.7172
MOlport_LH1_Approved_FDA_library.maegz:1895	Mepiroxol	125.130	-6.7129
MOlport_LH1_Approved_FDA_library.maegz:303	Cephapirin Sodium	445.450	-6.7063
MOlport_LH1_Approved_FDA_library.maegz:268	Travoprost	500.550	-6.7025
MOlport_LH1_Approved_FDA_library.maegz:842	Ibrutinib	440.500	-6.6992

MOlport_LH1_Approved_FDA_library.maegz:1396	Amikacin	585.600	-6.6945
MOlport_LH1_Approved_FDA_library.maegz:725	Bortezomib	384.240	-6.6932
MOlport_LH1_Approved_FDA_library.maegz:531	Amikacin Sulfate Salt	683.680	-6.6925
MOlport_LH1_Approved_FDA_library.maegz:487	Tavaborole	151.930	-6.6849
MOlport_LH1_Approved_FDA_library.maegz:1441	Floxuridine	246.190	-6.679
MOlport_LH1_Approved_FDA_library.maegz:1594	Coumarin	146.140	-6.6776
MOlport_LH1_Approved_FDA_library.maegz:1569	Diflunisal	250.200	-6.6739
MOlport_LH1_Approved_FDA_library.maegz:225	Emtricitabine	247.250	-6.664
MOlport_LH1_Approved_FDA_library.maegz:742	Panobinostat	349.430	-6.6557
MOlport_LH1_Approved_FDA_library.maegz:740	Ipragliflozin	404.450	-6.6521
MOlport_LH1_Approved_FDA_library.maegz:848	Micafungin sodium	1292.260	-6.6457
MOlport_LH1_Approved_FDA_library.maegz:811	Carbidopa Hydrate	244.240	-6.6453
MOlport_LH1_Approved_FDA_library.maegz:1506	Diosmin	608.540	-6.6438
MOlport_LH1_Approved_FDA_library.maegz:904	Ronidazole	200.150	-6.6407
MOlport_LH1_Approved_FDA_library.maegz:1101	Zidovudine	267.240	-6.6404
MOlport_LH1_Approved_FDA_library.maegz:812	Fulvestrant	606.770	-6.6323
MOlport_LH1_Approved_FDA_library.maegz:17	Ademetionine	398.440	-6.6318
MOlport_LH1_Approved_FDA_library.maegz:17	Ademetionine	398.440	-6.6301
MOlport_LH1_Approved_FDA_library.maegz:742	Panobinostat	349.430	-6.6271
MOlport_LH1_Approved_FDA_library.maegz:329	Butylphthalide	190.240	-6.6264
MOlport_LH1_Approved_FDA_library.maegz:9	Levomefolate calcium	497.520	-6.6216
MOlport_LH1_Approved_FDA_library.maegz:742	Panobinostat	349.430	-6.6048
MOlport_LH1_Approved_FDA_library.maegz:443	Pentostatin	268.270	-6.6023
MOlport_LH1_Approved_FDA_library.maegz:1712	Tyrosine	181.190	-6.595
MOlport_LH1_Approved_FDA_library.maegz:265	Indinavir sulfate	711.870	-6.5857
MOlport_LH1_Approved_FDA_library.maegz:9	Levomefolate calcium	497.520	-6.5814
MOlport_LH1_Approved_FDA_library.maegz:265	Indinavir sulfate	711.870	-6.5801
MOlport_LH1_Approved_FDA_library.maegz:953	Cyclocytidine hydrochloride	261.660	-6.5769
MOlport_LH1_Approved_FDA_library.maegz:265	Indinavir sulfate	711.870	-6.5685
MOlport_LH1_Approved_FDA_library.maegz:219	Pemetrexed Disodium Hydrate	516.420	-6.5651
MOlport_LH1_Approved_FDA_library.maegz:1952	Pemetrexed acid	427.410	-6.5632
MOlport_LH1_Approved_FDA_library.maegz:1951	Pemetrexed disodium	471.370	-6.563
MOlport_LH1_Approved_FDA_library.maegz:1724	Zileuton	236.290	-6.5629
MOlport_LH1_Approved_FDA_library.maegz:1101	Zidovudine	267.240	-6.5579
MOlport_LH1_Approved_FDA_library.maegz:1322	Polymyxin B sulfate	1301.560	-6.5519
MOlport_LH1_Approved_FDA_library.maegz:848	Micafungin sodium	1292.260	-6.5434
MOlport_LH1_Approved_FDA_library.maegz:1269	Ethinamide	166.250	-6.5334
MOlport_LH1_Approved_FDA_library.maegz:754	Ebastine	469.660	-6.5285
MOlport_LH1_Approved_FDA_library.maegz:1694	Tioxolone	168.170	-6.5206
MOlport_LH1_Approved_FDA_library.maegz:629	Salvianolic acid B	718.590	-6.5192
MOlport_LH1_Approved_FDA_library.maegz:368	Pipobroman	356.060	-6.5002
MOlport_LH1_Approved_FDA_library.maegz:1020	Rizatriptan benzoate	391.470	-6.4884

MOlport_LH1_Approved_FDA_library.maegz:265	Indinavir sulfate	711.870	-6.4724
MOlport_LH1_Approved_FDA_library.maegz:1746	Minoxidil	209.250	-6.4699
MOlport_LH1_Approved_FDA_library.maegz:806	Scopolamine HBr trihydrate	438.310	-6.4629
MOlport_LH1_Approved_FDA_library.maegz:265	Indinavir sulfate	711.870	-6.4515
MOlport_LH1_Approved_FDA_library.maegz:1007	Gliclazide	323.420	-6.4402
MOlport_LH1_Approved_FDA_library.maegz:923	Alprostadil	354.480	-6.4332
MOlport_LH1_Approved_FDA_library.maegz:169	Bepotastine Besilate	547.060	-6.418
MOlport_LH1_Approved_FDA_library.maegz:791	Serotonin hydrochloride	212.680	-6.4156
MOlport_LH1_Approved_FDA_library.maegz:665	Felypressin	1040.220	-6.4082
MOlport_LH1_Approved_FDA_library.maegz:791	Serotonin hydrochloride	212.680	-6.4071
MOlport_LH1_Approved_FDA_library.maegz:1724	Zileuton	236.290	-6.3957
MOlport_LH1_Approved_FDA_library.maegz:1199	Nabumetone	228.290	-6.3774
MOlport_LH1_Approved_FDA_library.maegz:1683	10,11-Dihydrocarbamazepine	238.290	-6.3686
MOlport_LH1_Approved_FDA_library.maegz:953	Cyclocytidine hydrochloride	261.660	-6.361
MOlport_LH1_Approved_FDA_library.maegz:1466	Docusate sodium	444.560	-6.3581
MOlport_LH1_Approved_FDA_library.maegz:596	Paeonol	166.170	-6.3571
MOlport_LH1_Approved_FDA_library.maegz:1942	Meptazinol hydrochloride	269.810	-6.3489
MOlport_LH1_Approved_FDA_library.maegz:1712	Tyrosine	181.190	-6.3321
MOlport_LH1_Approved_FDA_library.maegz:263	Calcium dobesilate	418.410	-6.3313
MOlport_LH1_Approved_FDA_library.maegz:864	Ilaprazole sodium	388.440	-6.3299
MOlport_LH1_Approved_FDA_library.maegz:2110	Ethamsylate	263.310	-6.3284
MOlport_LH1_Approved_FDA_library.maegz:1712	Tyrosine	181.190	-6.3211
MOlport_LH1_Approved_FDA_library.maegz:629	Salvianolic acid B	718.590	-6.3196
MOlport_LH1_Approved_FDA_library.maegz:1322	Polymyxin B sulfate	1301.560	-6.3131
MOlport_LH1_Approved_FDA_library.maegz:1942	Meptazinol hydrochloride	269.810	-6.2804
MOlport_LH1_Approved_FDA_library.maegz:626	5-Acetylsalicylic acid	180.160	-6.2797
MOlport_LH1_Approved_FDA_library.maegz:2110	Ethamsylate	263.310	-6.268
MOlport_LH1_Approved_FDA_library.maegz:586	Psoralen	186.170	-6.2669
MOlport_LH1_Approved_FDA_library.maegz:368	Pipobroman	356.060	-6.2666
MOlport_LH1_Approved_FDA_library.maegz:263	Calcium dobesilate	418.410	-6.2643
MOlport_LH1_Approved_FDA_library.maegz:1724	Zileuton	236.290	-6.2625
MOlport_LH1_Approved_FDA_library.maegz:1259	Temozolomide	194.150	-6.2564
MOlport_LH1_Approved_FDA_library.maegz:1414	Methoxamine hydrochloride	247.720	-6.2416
MOlport_LH1_Approved_FDA_library.maegz:1942	Meptazinol hydrochloride	269.810	-6.2373
MOlport_LH1_Approved_FDA_library.maegz:1251	Ifenprodil Tartrate	400.490	-6.2116
MOlport_LH1_Approved_FDA_library.maegz:1687	Protocatechuic acid	154.120	-6.1784
MOlport_LH1_Approved_FDA_library.maegz:1004	Mitiglinide calcium	704.910	-6.1745
MOlport_LH1_Approved_FDA_library.maegz:665	Felypressin	1040.220	-6.1524
MOlport_LH1_Approved_FDA_library.maegz:1735	Loxoprofen	246.300	-6.0972
MOlport_LH1_Approved_FDA_library.maegz:1873	Flurbiprofen	244.260	-6.0628
MOlport_LH1_Approved_FDA_library.maegz:1414	Methoxamine hydrochloride	247.720	-6.0483
MOlport_LH1_Approved_FDA_library.maegz:234	Desvenlafaxine succinate hydrate	399.480	-6.0256

MOlport_LH1_Approved_FDA_library.maegz:963	Desvenlafaxine	263.380	-6.0256
MOlport_LH1_Approved_FDA_library.maegz:1735	Loxoprofen	246.300	-6.0185
MOlport_LH1_Approved_FDA_library.maegz:1949	Levetiracetam	170.210	-5.9682
MOlport_LH1_Approved_FDA_library.maegz:1949	Levetiracetam	170.210	-5.9392
MOlport_LH1_Approved_FDA_library.maegz:1045	Ciclopirox	207.270	-5.9168
MOlport_LH1_Approved_FDA_library.maegz:1310	Ciclopirox ethanolamine	268.350	-5.9168
MOlport_LH1_Approved_FDA_library.maegz:970	Telmisartan	514.620	-5.9112
MOlport_LH1_Approved_FDA_library.maegz:1352	Baclofen	213.660	-5.8469
MOlport_LH1_Approved_FDA_library.maegz:184	(R)-baclofen	213.660	-5.8439
MOlport_LH1_Approved_FDA_library.maegz:284	N-Acetyl-L-tyrosine	223.230	-5.7849
MOlport_LH1_Approved_FDA_library.maegz:284	N-Acetyl-L-tyrosine	223.230	-5.7421
MOlport_LH1_Approved_FDA_library.maegz:284	N-Acetyl-L-tyrosine	223.230	-5.734
MOlport_LH1_Approved_FDA_library.maegz:1899	Methscopolamine	398.290	-5.7109
MOlport_LH1_Approved_FDA_library.maegz:1132	Cefalexine EP IMpurity B	214.240	-4.9993

Table S7. VS1 and VS2 for DSI-poised targeting PARG_4B1H_B

Compound	Catalog ID	MW	Energy in silico
VS1			
DSi PoisedLibrary_1680186639697.553	Z56960248	179.095	-6.3918
VS2			
DSi PoisedLibrary_1680186639697.63	Z1004825200	299.729	-7.119
DSi PoisedLibrary_1680186639697.63	Z1004825200	299.729	-7.0015
DSi PoisedLibrary_1680186639697.63	Z1004825200	299.729	-6.3605
DSi PoisedLibrary_1680186639697.301	Z1203107138	247.268	-6.1555
DSi PoisedLibrary_1680186639697.643	Z381033368	253.098	-6.1082
DSi PoisedLibrary_1680186639697.68	Z26251905	203.106	-6.1036
DSi PoisedLibrary_1680186639697.553	Z56960248	179.095	-6.0823
DSi PoisedLibrary_1680186639697.595	Z275181224	230.266	-6.0533
DSi PoisedLibrary_1680186639697.396	Z68195082	243.090	-5.9944
DSi PoisedLibrary_1680186639697.510	Z32400357	246.305	-5.9839
DSi PoisedLibrary_1680186639697.510	Z32400357	246.305	-5.968
DSi PoisedLibrary_1680186639697.36	Z1470724953	239.131	-5.9651
DSi PoisedLibrary_1680186639697.739	Z27805986	247.240	-5.9514
DSi PoisedLibrary_1680186639697.769	Z1509441355	219.137	-5.9182
DSi PoisedLibrary_1680186639697.541	Z1267773765	244.078	-5.9035
DSi PoisedLibrary_1680186639697.63	Z1004825200	299.729	-5.895
DSi PoisedLibrary_1680186639697.59	Z198194394	223.112	-5.8293
DSi PoisedLibrary_1680186639697.236	Z1333043510	226.078	-5.8083
DSi PoisedLibrary_1680186639697.115	Z1449748885	216.067	-5.7986
DSi PoisedLibrary_1680186639697.301	Z1203107138	247.268	-5.7905
DSi PoisedLibrary_1680186639697.125	Z275165822	238.087	-5.7891
DSi PoisedLibrary_1680186639697.719	Z332370018	203.069	-5.7846

DSi PoisedLibrary_1680186639697.718	Z55222357	251.280	-5.7753
DSi PoisedLibrary_1680186639697.487	Z33545544	247.293	-5.7621
DSi PoisedLibrary_1680186639697.36	Z1470724953	239.131	-5.7618
DSi PoisedLibrary_1680186639697.236	Z1333043510	226.078	-5.7359
DSi PoisedLibrary_1680186639697.487	Z33545544	247.293	-5.7233
DSi PoisedLibrary_1680186639697.433	Z1741959530	197.105	-5.7213
DSi PoisedLibrary_1680186639697.63	Z1004825200	299.729	-5.7186
DSi PoisedLibrary_1680186639697.780	Z18839017	247.157	-5.7155
DSi PoisedLibrary_1680186639697.769	Z1509441355	219.137	-5.7121
DSi PoisedLibrary_1680186639697.36	Z1470724953	239.131	-5.712
DSi PoisedLibrary_1680186639697.605	Z1696091761	198.056	-5.7064
DSi PoisedLibrary_1680186639697.378	Z332382842	206.142	-5.6998
DSi PoisedLibrary_1680186639697.526	Z317064394	170.142	-5.6945
DSi PoisedLibrary_1680186639697.378	Z332382842	206.142	-5.6914
DSi PoisedLibrary_1680186639697.137	Z285141302	190.111	-5.6869
DSi PoisedLibrary_1680186639697.428	Z90713255	232.301	-5.6845
DSi PoisedLibrary_1680186639697.456	Z992240004	199.111	-5.6822
DSi PoisedLibrary_1680186639697.825	Z19735067	223.101	-5.6732
DSi PoisedLibrary_1680186639697.378	Z332382842	206.142	-5.6697
DSi PoisedLibrary_1680186639697.396	Z68195082	243.090	-5.6655
DSi PoisedLibrary_1680186639697.336	Z56837087	194.025	-5.6654
DSi PoisedLibrary_1680186639697.635	Z90664455	142.111	-5.6596
DSi PoisedLibrary_1680186639697.59	Z198194394	223.112	-5.6504
DSi PoisedLibrary_1680186639697.172	Z1206890404	204.126	-5.6492
DSi PoisedLibrary_1680186639697.45	Z235449082	224.095	-5.6446
DSi PoisedLibrary_1680186639697.99	Z53834613	243.261	-5.632
DSi PoisedLibrary_1680186639697.236	Z1333043510	226.078	-5.6268
DSi PoisedLibrary_1680186639697.547	Z55290386	246.080	-5.6252
DSi PoisedLibrary_1680186639697.329	Z123929036	235.088	-5.6228
DSi PoisedLibrary_1680186639697.526	Z317064394	170.142	-5.607
DSi PoisedLibrary_1680186639697.294	Z26781943	217.122	-5.6066
DSi PoisedLibrary_1680186639697.750	Z1192341021	176.095	-5.6019
DSi PoisedLibrary_1680186639697.309	Z1730522163	209.217	-5.5994
DSi PoisedLibrary_1680186639697.713	Z57102475	167.095	-5.5939
DSi PoisedLibrary_1680186639697.47	Z1216837642	142.111	-5.5905
DSi PoisedLibrary_1680186639697.547	Z55290386	246.080	-5.5888
DSi PoisedLibrary_1680186639697.313	Z28429411	245.340	-5.5716
DSi PoisedLibrary_1680186639697.217	Z220816104	170.106	-5.5506
DSi PoisedLibrary_1680186639697.741	Z1295863442	189.026	-5.5505
DSi PoisedLibrary_1680186639697.553	Z56960248	179.095	-5.5494
DSi PoisedLibrary_1680186639697.769	Z1509441355	219.137	-5.5459
DSi PoisedLibrary_1680186639697.419	Z57160355	235.078	-5.5368
DSi PoisedLibrary_1680186639697.709	Z111782404	242.094	-5.5365

DSi PoisedLibrary_1680186639697.812	Z57627041	195.101	-5.5355
DSi PoisedLibrary_1680186639697.606	Z57472297	224.116	-5.5341
DSi PoisedLibrary_1680186639697.351	Z26781964	241.085	-5.5327
DSi PoisedLibrary_1680186639697.586	Z415636694	240.103	-5.5256
DSi PoisedLibrary_1680186639697.97	Z2692078340	223.088	-5.5199
DSi PoisedLibrary_1680186639697.789	Z1929757385	210.248	-5.5032
DSi PoisedLibrary_1680186639697.351	Z26781964	241.085	-5.5005
DSi PoisedLibrary_1680186639697.773	Z193091370	192.057	-5.4987
DSi PoisedLibrary_1680186639697.68	Z26251905	203.106	-5.4956
DSi PoisedLibrary_1680186639697.142	Z1757321532	244.109	-5.4951
DSi PoisedLibrary_1680186639697.719	Z332370018	203.069	-5.4751
DSi PoisedLibrary_1680186639697.114	Z1758466000	229.105	-5.4744
DSi PoisedLibrary_1680186639697.789	Z1929757385	210.248	-5.4742
DSi PoisedLibrary_1680186639697.665	Z53116498	203.106	-5.4621
DSi PoisedLibrary_1680186639697.2	Z44603686	227.163	-5.457
DSi PoisedLibrary_1680186639697.623	Z56963343	155.050	-5.4468
DSi PoisedLibrary_1680186639697.378	Z332382842	206.142	-5.4424
DSi PoisedLibrary_1680186639697.254	Z1618027593	175.075	-5.4401
DSi PoisedLibrary_1680186639697.713	Z57102475	167.095	-5.4392
DSi PoisedLibrary_1680186639697.789	Z1929757385	210.248	-5.4387
DSi PoisedLibrary_1680186639697.812	Z57627041	195.101	-5.4382
DSi PoisedLibrary_1680186639697.234	Z119990900	210.046	-5.4366
DSi PoisedLibrary_1680186639697.466	Z33486130	221.080	-5.4326
DSi PoisedLibrary_1680186639697.261	Z32399802	170.106	-5.4185
DSi PoisedLibrary_1680186639697.402	Z57299529	237.127	-5.4155
DSi PoisedLibrary_1680186639697.259	Z79432418	225.268	-5.4143
DSi PoisedLibrary_1680186639697.254	Z1618027593	175.075	-5.4068
DSi PoisedLibrary_1680186639697.45	Z235449082	224.095	-5.4041
DSi PoisedLibrary_1680186639697.45	Z235449082	224.095	-5.4024
DSi PoisedLibrary_1680186639697.719	Z332370018	203.069	-5.3873
DSi PoisedLibrary_1680186639697.713	Z57102475	167.095	-5.3869
DSi PoisedLibrary_1680186639697.526	Z317064394	170.142	-5.382
DSi PoisedLibrary_1680186639697.699	Z1493056027	178.078	-5.3783
DSi PoisedLibrary_1680186639697.750	Z1192341021	176.095	-5.376
DSi PoisedLibrary_1680186639697.607	Z317064236	186.137	-5.3757
DSi PoisedLibrary_1680186639697.586	Z415636694	240.103	-5.3637
DSi PoisedLibrary_1680186639697.254	Z1618027593	175.075	-5.363
DSi PoisedLibrary_1680186639697.750	Z1192341021	176.095	-5.3617
DSi PoisedLibrary_1680186639697.734	Z1265813904	207.201	-5.3546
DSi PoisedLibrary_1680186639697.208	Z56960214	189.077	-5.3495
DSi PoisedLibrary_1680186639697.553	Z56960248	179.095	-5.3408
DSi PoisedLibrary_1680186639697.276	Z169506466	191.056	-5.3385
DSi PoisedLibrary_1680186639697.261	Z32399802	170.106	-5.3357

DSi PoisedLibrary_1680186639697.553	Z56960248	179.095	-5.3345
DSi PoisedLibrary_1680186639697.547	Z55290386	246.080	-5.3272
DSi PoisedLibrary_1680186639697.560	Z133716556	227.062	-5.323
DSi PoisedLibrary_1680186639697.321	Z1272517105	227.109	-5.3199
DSi PoisedLibrary_1680186639697.503	Z223388508	242.316	-5.3124
DSi PoisedLibrary_1680186639697.259	Z79432418	225.268	-5.3086
DSi PoisedLibrary_1680186639697.68	Z26251905	203.106	-5.2925
DSi PoisedLibrary_1680186639697.181	Z285140852	184.158	-5.2723
DSi PoisedLibrary_1680186639697.665	Z53116498	203.106	-5.2596
DSi PoisedLibrary_1680186639697.687	Z381408780	204.126	-5.2564
DSi PoisedLibrary_1680186639697.689	Z111810692	223.001	-5.2558
DSi PoisedLibrary_1680186639697.98	Z131516158	204.101	-5.2547
DSi PoisedLibrary_1680186639697.639	Z979770898	159.090	-5.2274
DSi PoisedLibrary_1680186639697.603	Z1020729274	170.142	-5.2143
DSi PoisedLibrary_1680186639697.803	Z383202616	175.075	-5.1619
DSi PoisedLibrary_1680186639697.261	Z32399802	170.106	-5.1592
DSi PoisedLibrary_1680186639697.217	Z220816104	170.106	-5.1498
DSi PoisedLibrary_1680186639697.639	Z979770898	159.090	-5.1473
DSi PoisedLibrary_1680186639697.773	Z193091370	192.057	-5.1468
DSi PoisedLibrary_1680186639697.278	Z1551999220	241.010	-5.1411
DSi PoisedLibrary_1680186639697.699	Z1493056027	178.078	-5.1353
DSi PoisedLibrary_1680186639697.754	Z1324853681	165.090	-5.1269
DSi PoisedLibrary_1680186639697.812	Z57627041	195.101	-5.1262
DSi PoisedLibrary_1680186639697.605	Z1696091761	198.056	-5.1208
DSi PoisedLibrary_1680186639697.295	Z1457921102	240.103	-5.1123
DSi PoisedLibrary_1680186639697.114	Z1758466000	229.105	-5.1
DSi PoisedLibrary_1680186639697.592	Z1267800292	174.079	-5.0995
DSi PoisedLibrary_1680186639697.754	Z1324853681	165.090	-5.0939
DSi PoisedLibrary_1680186639697.645	Z234898257	162.091	-5.0786
DSi PoisedLibrary_1680186639697.600	Z1973465508	244.109	-5.0746
DSi PoisedLibrary_1680186639697.85	Z1443544073	189.057	-5.0666
DSi PoisedLibrary_1680186639697.234	Z119990900	210.046	-5.0123
DSi PoisedLibrary_1680186639697.817	Z1250093582	173.167	-5.0102
DSi PoisedLibrary_1680186639697.635	Z90664455	142.111	-5.0094
DSi PoisedLibrary_1680186639697.699	Z1493056027	178.078	-4.9159

Table S8. VS1 and VS2 for PDB fragment library targeting PARG_4B1H_B

compound	MW	Energy in silico
VS1		
octahydroindolizine-1,6,7,8-tetrol	189.1	-6.2916
Vs2		

octahydroindolizine-1,6,7,8-tetrol	189.100	-6.72
tyrosinamide	180.090	-6.4455
tyrosinamide	180.090	-6.4414
octahydroindolizine-1,6,7,8-tetrol	189.100	-6.281
tyrosinamide	180.090	-6.2763
1-butyl-2-(hydroxymethyl)piperidine-3,4,5-triol	219.147	-6.2334
1-butyl-2-(hydroxymethyl)piperidine-3,4,5-triol	219.147	-6.2008
octahydroindolizine-1,6,7,8-tetrol	189.100	-6.1809
octahydroindolizine-1,6,7,8-tetrol	189.100	-6.158
octahydroindolizine-1,6,7,8-tetrol	189.100	-6.1333
Proflavine	209.095	-6.1294
1-butyl-2-(hydroxymethyl)piperidine-3,4,5-triol	219.147	-6.0607
octahydroindolizine-1,6,7,8-tetrol	189.100	-6.0581
octahydroindolizine-1,6,7,8-tetrol	189.100	-6.0367
Resveratrol	228.079	-6.0068
Thioguanine	167.027	-5.9491
octahydroindolizine-1,6,7,8-tetrol	189.100	-5.9133
Proflavine	209.095	-5.9083
octahydroindolizine-1,6,7,8-tetrol	189.100	-5.9023
octahydroindolizine-1,6,7,8-tetrol	189.100	-5.901
L-tryptophanamide	203.106	-5.8723
Serotonin	176.095	-5.8688
octahydroindolizine-1,6,7,8-tetrol	189.100	-5.8624
octahydroindolizine-1,6,7,8-tetrol	189.100	-5.8608
Arabinosylcytosine		-5.8605
octahydroindolizine-1,6,7,8-tetrol	189.100	-5.8566
1-butyl-2-(hydroxymethyl)piperidine-3,4,5-triol	219.147	-5.7974
Arabinosylcytosine		-5.7821
octahydroindolizine-1,6,7,8-tetrol	189.100	-5.751
2-Naphthimidamide	170.084	-5.731
L-tryptophanamide	203.106	-5.6926
octahydroindolizine-1,6,7,8-tetrol	189.100	-5.6914
Histamine	111.080	-5.6868
L-tryptophanamide	203.106	-5.6815
octahydroindolizine-1,6,7,8-tetrol	189.100	-5.6553
Serotonin	176.095	-5.6421
octahydroindolizine-1,6,7,8-tetrol	189.100	-5.6236
Tryptamine	160.100	-5.6229
Arabinosylcytosine		-5.6228
6-methyltetrahydropterin	181.096	-5.5999
octahydroindolizine-1,6,7,8-tetrol	189.100	-5.5418
octahydroindolizine-1,6,7,8-tetrol	189.100	-5.5415
octahydroindolizine-1,6,7,8-tetrol	189.100	-5.496

Tryptamine	160.100	-5.4519
octahydroindolizine-1,6,7,8-tetrol	189.100	-5.4504
2-Naphthimidamide	170.084	-5.4305
Tryptamine	160.100	-5.4293
6-Hydroxy-2-benzothiazolesulfonamide	229.982	-5.3769
Histamine	111.080	-5.3633
2-Phenylethylamine	157.066	-5.2902
Histamine	111.080	-5.1931
1H-Benzo[d]imidazole-2-sulfonamide	197.026	-5.1721
2-Amino-1H-benzo[d]imidazol-5-ol	149.059	-5.1628
6-methyltetrahydropterin	181.096	-5.1327
Tranexamic Acid	157.110	-5.0933
Histamine	111.080	-5.0772
3,4,5-trihydroxycyclohex-2-ene-1-carboxylic acid	174.053	-5.0744
Tranexamic Acid	157.110	-5.0565
3' epi gemcitabine (impurity)	263.072	-5.0124
Tranexamic Acid	157.110	-4.8931
Tranexamic Acid	157.110	-4.662
Tranexamic Acid	157.110	-4.5587

Table S9. VS1 and VS2 for LH1 FDA Approved drugs targeting PARG_4B1H_B

Name	MolWt	Energy in silico
Deferoxamine Mesylate	656.790	-11.012
Goserelin acetate	1329.460	-9.3418
Ledipasvir	889.020	-9.0092
Ledipasvir	889.020	-8.8973
Ledipasvir	889.020	-8.843
Polymyxin B sulfate	1301.560	-8.679
Posaconazole	700.780	-8.6302
Goserelin acetate	1329.460	-8.6105
Posaconazole	700.780	-8.4276
Polymyxin B sulfate	1301.560	-8.2536
ABT199	868.440	-8.2509
Goserelin acetate	1329.460	-8.2505
ABT199	868.440	-8.2207
Goserelin acetate	1329.460	-8.1947
Deferoxamine Mesylate	656.790	-8.1934
Goserelin acetate	1329.460	-8.1462

Atracurium besylate	1243.490	-8.1439
Anidulafungin	1140.240	-8.1259
Anidulafungin	1140.240	-8.0232
ABT199	868.440	-7.9944
Atracurium besylate	1243.490	-7.9366
Desmopressin	1069.220	-7.9335
Posaconazole	700.780	-7.933
Atracurium besylate	1243.490	-7.8925
ABT199	868.440	-7.8823
Carbetocin	988.160	-7.8767
Irinotecan hydrochloride trihydrate	677.180	-7.848
Lapatinib Ditosylate	925.460	-7.8441
Lapatinib	581.060	-7.8441
Irinotecan	586.680	-7.8429
Lapatinib ditosylate monohydrate	943.470	-7.8299
Irinotecan hydrochloride trihydrate	677.180	-7.8264
Irinotecan	586.680	-7.8222
Octenidine Dihydrochloride	623.820	-7.8019
Anidulafungin	1140.240	-7.7866
Polymyxin B sulfate	1301.560	-7.7623
Carbetocin	988.160	-7.7534
Irinotecan hydrochloride trihydrate	677.180	-7.7529
Irinotecan	586.680	-7.7514
Carbetocin	988.160	-7.7238
Darolutamide	398.850	-7.7168
Darolutamide	398.850	-7.6969
Deferoxamine Mesylate	656.790	-7.6917
Felypressin	1040.220	-7.6673
Darolutamide	398.850	-7.655
Anidulafungin	1140.240	-7.6532
Desmopressin	1069.220	-7.6454
Lapatinib ditosylate monohydrate	943.470	-7.6416
Lapatinib Ditosylate	925.460	-7.6404
Lapatinib	581.060	-7.6404
Deferoxamine Mesylate	656.790	-7.6283
Desmopressin	1069.220	-7.6191
Polymyxin B sulfate	1301.560	-7.6179
Troxerutin	742.670	-7.5976
Isavuconazonium sulfate	814.840	-7.5613
Troxerutin	742.670	-7.5526
Ombitasvir	894.110	-7.5507
Atracurium besylate	1243.490	-7.546
Ritonavir	720.960	-7.541

Troxerutin	742.670	-7.5284
VX661	520.500	-7.5196
Diosmin	608.540	-7.5081
Travoprost	500.550	-7.5079
Ledipasvir	889.020	-7.4833
VX661	520.500	-7.4764
Ledipasvir	889.020	-7.473
Goserelin acetate	1329.460	-7.4536
Isavuconazonium sulfate	814.840	-7.4438
VX661	520.500	-7.43
Anidulafungin	1140.240	-7.411
Sophoricoside	432.390	-7.4083
Indinavir sulfate	711.870	-7.4032
Mitoxantrone hydrochloride	517.400	-7.3949
Mitoxantrone	444.480	-7.3925
ABT199	868.440	-7.3806
Polymyxin B sulfate	1301.560	-7.3772
Felypressin	1040.220	-7.3729
Darolutamide	398.850	-7.3696
Odanacatib	525.560	-7.3685
Capastat sulfate	752.760	-7.3648
Atracurium besylate	1243.490	-7.3646
Travoprost	500.550	-7.3568
Amikacin	585.600	-7.3494
Amikacin Sulfate Salt	683.680	-7.3348
Octenidine Dihydrochloride	623.820	-7.3338
Salvianolic acid B	718.590	-7.3329
Revefenacin	597.750	-7.3263
Thymopentin	679.560	-7.3256
Dequalinium chloride	527.590	-7.325
Trazodone hydrochloride	408.330	-7.312
Goserelin acetate	1329.460	-7.3113
Anidulafungin	1140.240	-7.3055
Thymopentin	679.560	-7.295
Capastat sulfate	752.760	-7.2932
Isavuconazonium sulfate	814.840	-7.283
Polymyxin B sulfate	1301.560	-7.2497
Mitoxantrone	444.480	-7.2445
Mitoxantrone hydrochloride	517.400	-7.2433
Mitoxantrone hydrochloride	517.400	-7.2423
Mitoxantrone	444.480	-7.2402
Indinavir sulfate	711.870	-7.2342
Goserelin acetate	1329.460	-7.2274

Pirarubicin	627.640	-7.2204
Pentagastrin	767.890	-7.2168
Verteporfin	718.790	-7.2047
Lapatinib Ditosylate	925.460	-7.1973
Lapatinib	581.060	-7.1973
Capastat sulfate	752.760	-7.1972
Indinavir sulfate	711.870	-7.1957
Lapatinib ditosylate monohydrate	943.470	-7.1943
Darolutamide	398.850	-7.1915
Thymopentin	679.560	-7.1429
Cefotiam Hexetil Hydrochloride	768.765	-7.1352
Capastat sulfate	752.760	-7.1309
Pirarubicin	627.640	-7.1267
Atracurium besylate	1243.490	-7.0998
Capastat sulfate	752.760	-7.094
Ledipasvir	889.020	-7.082
Revefenacin	597.750	-7.0799
Hesperidin	610.560	-7.0794
Indinavir sulfate	711.870	-7.0674
Capastat sulfate	752.760	-7.0496
Anidulafungin	1140.240	-7.0444
Ledipasvir	889.020	-7.0402
Topotecan	421.450	-7.0379
Vilazodone	441.530	-7.0281
Indinavir sulfate	711.870	-7.0263
Felypressin	1040.220	-7.0193
Capastat sulfate	752.760	-7.0057
Travoprost	500.550	-7.0056
Nelfinavir Mesylate	663.890	-6.9889
Indinavir sulfate	711.870	-6.9847
Indinavir sulfate	711.870	-6.9732
Indinavir sulfate	711.870	-6.9528
Ademetionine	398.440	-6.9363
Nelfinavir Mesylate	663.890	-6.9276
Capastat sulfate	752.760	-6.9268
Fedratinib	524.680	-6.9178
Felypressin	1040.220	-6.9171
Isavuconazonium sulfate	814.840	-6.9108
Hydroxy Camptothecine	364.350	-6.9106
10-Hydroxycampothecin	364.350	-6.9079
Darolutamide	398.850	-6.9037
Verteporfin	718.790	-6.902
Indinavir sulfate	711.870	-6.9017

Pirarubicin	627.640	-6.8914
Trazodone hydrochloride	408.330	-6.8886
Palbociclib Isethionate	573.660	-6.8847
Palbociclib hydrochloride	483.990	-6.8847
Carfilzomib	719.910	-6.8826
Indinavir sulfate	711.870	-6.8819
Palbociclib	447.540	-6.8793
Sanguinarine chloride	367.780	-6.8687
Panobinostat	349.430	-6.8657
Ademetionine	398.440	-6.8597
Hesperidin	610.560	-6.8456
Indinavir sulfate	711.870	-6.8322
Topotecan	421.450	-6.8304
Ademetionine	398.440	-6.8285
Levomefolate calcium	497.520	-6.8209
Isavuconazonium sulfate	814.840	-6.8195
Felypressin	1040.220	-6.8095
Imatinib	493.600	-6.8088
Imatinib Mesylate	589.710	-6.8088
Odanacatib	525.560	-6.8062
Ombitasvir	894.110	-6.8051
10-Hydroxycampothecin	364.350	-6.803
Hydroxy Camptothecine	364.350	-6.7985
D-Pantethine	554.720	-6.7925
Canagliflozin	444.520	-6.7923
Atracurium besylate	1243.490	-6.7917
10-Hydroxycampothecin	364.350	-6.7901
Hydroxy Camptothecine	364.350	-6.7889
Esomeprazole Sodium	367.400	-6.7882
Hydroxy Camptothecine	364.350	-6.7876
10-Hydroxycampothecin	364.350	-6.787
10-Hydroxycamptothecin	364.360	-6.787
ABT199	868.440	-6.782
Travoprost	500.550	-6.7708
Topotecan	421.450	-6.7681
Cefpodoxime proxetil	557.600	-6.7643
Indinavir sulfate	711.870	-6.7622
Indinavir sulfate	711.870	-6.7617
Esomeprazole Sodium	367.400	-6.7602
Fulvestrant	606.770	-6.7479
Trazodone hydrochloride	408.330	-6.7463
Indinavir sulfate	711.870	-6.7404
Carfilzomib	719.910	-6.7374

Thymopentin	679.560	-6.7327
Trazodone hydrochloride	408.330	-6.7281
10-Hydroxycampothecin	364.350	-6.7268
10-Hydroxycamptothecin	364.360	-6.7268
Hydroxy Camptothecine	364.350	-6.7253
Travoprost	500.550	-6.7145
Atracurium besylate	1243.490	-6.7122
Clofazimine	473.400	-6.7063
Hydroxy Camptothecine	364.350	-6.7054
10-Hydroxycampothecin	364.350	-6.703
10-Hydroxycamptothecin	364.360	-6.703
Levomefolate calcium	497.520	-6.687
Tenofovir alafenamide fumarate	534.500	-6.6821
Thymopentin	679.560	-6.6789
Indinavir sulfate	711.870	-6.6777
Sanguinarine chloride	367.780	-6.6736
Indinavir sulfate	711.870	-6.6691
Indinavir sulfate	711.870	-6.6637
Tigecycline	585.650	-6.6621
Indinavir sulfate	711.870	-6.6561
Nelfinavir Mesylate	663.890	-6.6558
Vilazodone HCl	477.990	-6.6541
Indinavir sulfate	711.870	-6.6521
Micafungin sodium	1292.260	-6.6472
Ademetionine	398.440	-6.6417
Diosmin	608.540	-6.6403
Volasertib	618.810	-6.6401
Tucidinostat	390.410	-6.6344
Salvianolic acid B	718.590	-6.6308
Capastat sulfate	752.760	-6.63
Lomitapide	692.710	-6.63
Indinavir sulfate	711.870	-6.6293
Indinavir sulfate	711.870	-6.6263
Nelfinavir Mesylate	663.890	-6.6248
Levomefolate calcium	497.520	-6.6222
Bicalutamide	430.370	-6.6169
Trazodone hydrochloride	408.330	-6.6129
Diosmin	608.540	-6.6083
Cefminox Sodium	563.530	-6.5984
Ademetionine	398.440	-6.5902
Sanguinarine chloride	367.780	-6.5896
Esomeprazole Sodium	367.400	-6.5793
Palbociclib	447.540	-6.5783

Palbociclib Isethionate	573.660	-6.5765
Palbociclib hydrochloride	483.990	-6.5765
Ademetionine	398.440	-6.5739
Indinavir sulfate	711.870	-6.5722
Berberine hydrogen sulphate	433.430	-6.5693
Berberine	336.360	-6.5693
Norepinephrine	169.180	-6.5638
Noradrenaline bitartrate	319.260	-6.5638
Linagliptin	472.540	-6.5595
Linezolid	337.350	-6.5482
Amikacin Sulfate Salt	683.680	-6.5448
Amikacin	585.600	-6.5419
Ilaprazole sodium	388.440	-6.5405
Pixantrone dimaleate	557.210	-6.5378
Cefmetazole sodium	493.520	-6.5368
Revefenacin	597.750	-6.5326
Amikacin	585.600	-6.5308
Lafutidine	431.550	-6.5303
Esomeprazole Magnesium	713.120	-6.5283
Amikacin Sulfate Salt	683.680	-6.527
Casanthranol	396.580	-6.5258
Isavuconazonium sulfate	814.840	-6.5233
Adefovir dipivoxil	501.470	-6.5181
Cefminox Sodium	563.530	-6.5092
Anidulafungin	1140.240	-6.5059
Esomeprazole Magnesium	713.120	-6.4986
Fedratinib	524.680	-6.4951
Travoprost	500.550	-6.4906
Indinavir sulfate	711.870	-6.4904
Nelfinavir Mesylate	663.890	-6.4836
Dofetilide	441.560	-6.4822
Indinavir sulfate	711.870	-6.4774
Sennoside A	862.740	-6.4774
Hesperidin	610.560	-6.4741
Sennoside A	862.740	-6.4703
Odanacatib	525.560	-6.469
DL-Adrenaline Hydrochloride	219.670	-6.469
Levomefolate calcium	497.520	-6.4674
Casanthranol	396.580	-6.4673
Vilazodone	441.530	-6.4647
Bicalutamide	430.370	-6.463
Indinavir sulfate	711.870	-6.4619
bremelanotide	1085.220	-6.455

Levomefolate calcium	497.520	-6.4531
Casanthranol	396.580	-6.4475
Casanthranol	396.580	-6.4448
Travoprost	500.550	-6.4364
Indinavir sulfate	711.870	-6.4333
Casanthranol	396.580	-6.4331
Diprophylline	254.250	-6.4321
Relugolix	623.630	-6.431
Tucidinostat	390.410	-6.4308
Amphotericin B	924.080	-6.4253
Casanthranol	396.580	-6.424
Casanthranol	396.580	-6.4182
Casanthranol	396.580	-6.4171
Casanthranol	396.580	-6.4153
bremelanotide	1085.220	-6.4149
Dofetilide	441.560	-6.4098
Polymyxin B sulfate	1301.560	-6.4083
Indinavir sulfate	711.870	-6.4054
Cabazitaxel	835.930	-6.4048
Vilazodone HCl	477.990	-6.4032
Roflumilast N-oxide	419.210	-6.4007
Anidulafungin	1140.240	-6.3971
Nafronyl oxalate	473.560	-6.3811
DL-Adrenaline Hydrochloride	219.670	-6.3798
Casanthranol	396.580	-6.3796
Casanthranol	396.580	-6.3758
Indinavir sulfate	711.870	-6.369
Dabigatran	471.510	-6.3684
Sennoside A	862.740	-6.3682
bremelanotide	1085.220	-6.3673
Levomefolate calcium	497.520	-6.3642
Fexofenadine hydrochloride	538.120	-6.364
Levomefolate calcium	497.520	-6.3623
Neomycin sulfate	908.870	-6.3612
Nortriptyline hydrochloride	299.840	-6.3602
Indinavir sulfate	711.870	-6.3588
Proanthocyanidins	594.520	-6.3544
Methyl hesperidin	624.590	-6.3433
Pixantrone dimaleate	557.210	-6.342
Sitagliptin	407.320	-6.3413
Carvedilol	406.470	-6.3371
Carvedilol phosphate	522.490	-6.337
Carvedilol phosphate	522.490	-6.3353

Neomycin Sulphate B	712.220	-6.3329
Indinavir sulfate	711.870	-6.3323
Cefotiam Hexetil Hydrochloride	768.765	-6.3241
Anagrelide HCl	292.550	-6.3193
Anagrelide	256.090	-6.3187
Indinavir sulfate	711.870	-6.318
Aloe-emodin	270.240	-6.3147
Capastat sulfate	752.760	-6.3137
Flurbiprofen Axetil	330.360	-6.31
Ipragliflozin	404.450	-6.3099
Ilaprazole sodium	388.440	-6.3033
Polymyxin B sulfate	1301.560	-6.3001
Norepinephrine	169.180	-6.2968
Noradrenaline bitartrate	319.260	-6.2968
Larotrectinib	428.440	-6.2963
Aloe-emodin	270.240	-6.2949
Larotrectinib sulfate	526.510	-6.2915
Travoprost	500.550	-6.2902
Trazodone hydrochloride	408.330	-6.2896
Ertapenem sodium	497.502	-6.2859
Ademetionine	398.440	-6.2853
Vilazodone	441.530	-6.2792
Micafungin sodium	1292.260	-6.278
Casanthranol	396.580	-6.2778
Pixantrone dimaleate	557.210	-6.2771
Casanthranol	396.580	-6.2755
Tedizolid Phosphate	450.320	-6.2724
Tedizolid Phosphate	450.320	-6.2722
Polymyxin B sulfate	1301.560	-6.2717
Travoprost	500.550	-6.2706
Amphotericin B	924.080	-6.2694
tafamidis meglumine	503.330	-6.2691
Cefminox Sodium	563.530	-6.2654
Thymopentin	679.560	-6.2609
Mefloquine hydrochloride	414.770	-6.2586
Probucol	516.840	-6.2581
Promazine hydrochloride	320.880	-6.256
Indinavir sulfate	711.870	-6.2544
Olopatadine hydrochloride	373.870	-6.2544
Anidulafungin	1140.240	-6.2509
Dopamine hydrochloride	189.640	-6.25
Levomefolate calcium	497.520	-6.2489
Riociguat	422.420	-6.2472

Benzthiazide	431.940	-6.2446
Tirofiban hydrochloride monohydrate	495.070	-6.239
Casanthranol	396.580	-6.2318
Casanthranol	396.580	-6.2304
Ipragliflozin	404.450	-6.2294
Tirofiban hydrochloride monohydrate	495.070	-6.227
Isepamicin sulfate	667.680	-6.213
Travoprost	500.550	-6.2015
bremelanotide	1085.220	-6.1995
Folic acid	441.400	-6.199
Tedizolid Phosphate	450.320	-6.1984
Dorzolamide hydrochloride	360.900	-6.1931
Roflumilast N-oxide	419.210	-6.1891
Ademetionine	398.440	-6.1865
Aloe-emodin	270.240	-6.1853
Tedizolid Phosphate	450.320	-6.1833
Troxipide	294.350	-6.1822
Proflavine Hemisulfate	516.570	-6.1816
Travoprost	500.550	-6.1803
Carvedilol	406.470	-6.1792
Proanthocyanidins	594.520	-6.1762
Riociguat	422.420	-6.171
Diprophylline	254.250	-6.1696
Indinavir sulfate	711.870	-6.1566
Folic acid	441.400	-6.1551
Salvianolic acid B	718.590	-6.1515
Desloratadine	310.820	-6.151
Lifitegrast	615.480	-6.1502
Indinavir sulfate	711.870	-6.1488
Promazine hydrochloride	320.880	-6.1439
Tobramycin	467.510	-6.1433
Proanthocyanidins	594.520	-6.1427
Casanthranol	396.580	-6.136
Ilaprazole sodium	388.440	-6.1329
Sophoricoside	432.390	-6.1255
Casanthranol	396.580	-6.1213
Felypressin	1040.220	-6.1203
Proanthocyanidins	594.520	-6.111
Casanthranol	396.580	-6.1109
Olopatadine hydrochloride	373.870	-6.1072
Sumatriptan succinate	413.490	-6.1008
Naftifine hydrochloride	323.860	-6.0958
Hygromycin B	527.520	-6.0903

bremelanotide	1085.220	-6.0889
Naratriptan	371.930	-6.0848
Ombitasvir	894.110	-6.0786
Proanthocyanidins	594.520	-6.0706
Fexofenadine hydrochloride	538.120	-6.0604
Casanthranol	396.580	-6.0599
Zofenopril (calcium)	897.170	-6.0592
Levomefolate calcium	497.520	-6.0557
Hygromycin B	527.520	-6.0518
Doxepin hydrochloride	315.840	-6.0467
Sultamicillin Tosylate	766.860	-6.0286
Promethazine	284.420	-6.0251
Pentostatin	268.270	-6.0157
Dichlorphenamide	305.160	-6.011
Avatrombopag	649.650	-6.0097
Pentagastrin	767.890	-6.009
Methyl hesperidin	624.590	-6.0066
Tenofovir alafenamide fumarate	534.500	-6.0018
Sennoside A	862.740	-6.0004
Pemetrexed acid	427.410	-5.9979
Novobiocin Sodium	634.610	-5.9964
Pemetrexed disodium	471.370	-5.9949
Pemetrexed Disodium Hydrate	516.420	-5.9913
Sennoside A	862.740	-5.9905
Ademetionine	398.440	-5.9868
Tobramycin	467.510	-5.9866
Pioglitazone	356.440	-5.9742
Quinapril hydrochloride	474.980	-5.9708
Olopatadine hydrochloride	373.870	-5.9691
Dopamine hydrochloride	189.640	-5.9632
Pioglitazone	356.440	-5.9591
Polymyxin B sulfate	1301.560	-5.9492
Indinavir sulfate	711.870	-5.9492
R-(+)-Lansoprazole	369.360	-5.9455
Lansoprazole	369.360	-5.9455
Pentagastrin	767.890	-5.9444
Pentostatin	268.270	-5.9428
Cyclocytidine hydrochloride	261.660	-5.9378
Levomefolate calcium	497.520	-5.9335
Doxofylline	266.260	-5.9236
Ademetionine	398.440	-5.9177
Sumatriptan succinate	413.490	-5.9123
Casanthranol	396.580	-5.9048

Micafungin sodium	1292.260	-5.9042
Micafungin sodium	1292.260	-5.9024
Pemetrexed disodium	471.370	-5.897
tafamidis meglumine	503.330	-5.895
Micafungin sodium	1292.260	-5.8934
Dichlorphenamide	305.160	-5.8913
Pemetrexed acid	427.410	-5.8906
Pemetrexed Disodium Hydrate	516.420	-5.8889
Sennoside A	862.740	-5.8846
Cephapirin Sodium	445.450	-5.8776
Serotonin hydrochloride	212.680	-5.8737
Folic acid	441.400	-5.8595
Bicalutamide	430.370	-5.85
Neomycin sulfate	908.870	-5.8472
Oxiracetam	158.160	-5.8406
Neomycin Sulphate B	712.220	-5.8373
Daphnetin	178.140	-5.8161
Itraconazole	705.630	-5.8151
5-Azacytidine	244.200	-5.8122
Levomefolate calcium	497.520	-5.8033
Levomefolate calcium	497.520	-5.7973
Dichlorphenamide	305.160	-5.783
Chlorpropamide	276.740	-5.7769
Pentostatin	268.270	-5.7642
4-Methylumbelliferone	176.170	-5.7506
Tobramycin	467.510	-5.7479
Hematoporphyrin dihydrochloride	671.610	-5.7078
Lesinurad	404.280	-5.7078
Hematoporphyrin dihydrochloride	671.610	-5.707
Albendazole Oxide	281.330	-5.7046
Histamine 2HCl	184.070	-5.6961
Histamine	111.150	-5.6961
Glucosamine hydrochloride	215.630	-5.6878
Hematoporphyrin dihydrochloride	671.610	-5.6849
Promethazine	284.420	-5.6741
Serotonin hydrochloride	212.680	-5.6388
Aminophylline	420.430	-5.627
Hematoporphyrin dihydrochloride	671.610	-5.6104
Methylthiouracil	142.180	-5.6076
Glucosamine hydrochloride	215.630	-5.6036
Glucosamine hydrochloride	215.630	-5.5978
Glucosamine hydrochloride	215.630	-5.584
Propylthiouracil	170.240	-5.5701

Mepiroxol	125.130	-5.5525
Carbidopa Hydrate	244.240	-5.5391
Levomefolate calcium	497.520	-5.5066
Levomefolate calcium	497.520	-5.5013
Dacarbazine	182.180	-5.4698
Levomefolate calcium	497.520	-5.4694
Carbazochrome	236.230	-5.4327
Cefoxitin sodium	449.430	-5.4208
Hematoporphyrin dihydrochloride	671.610	-5.3754
Pemetrexed acid	427.410	-5.3709
Histamine 2HCl	184.070	-5.3669
Histamine	111.150	-5.3669
Carbazochrome sodium sulfonate	376.320	-5.3611
Micafungin sodium	1292.260	-5.33
Amifostine	214.220	-5.1241
Amifostine	214.220	-4.9501

Table S10. VS1 and VS2 for DSI-poised targeting PARG_6OAK

compound	MW	Catalog ID	Energy in silico
VS1			
DSi PoisedLibrary_1680186639697.103	248.321	Z54748186	-6.8256
VS2			
DSi PoisedLibrary_1680186639697.777	267.127	Z352356442	-7.0978
DSi PoisedLibrary_1680186639697.254	175.075	Z1618027593	-6.9957
DSi PoisedLibrary_1680186639697.63	299.729	Z1004825200	-6.9588
DSi PoisedLibrary_1680186639697.63	299.729	Z1004825200	-6.9224
DSi PoisedLibrary_1680186639697.777	267.127	Z352356442	-6.9124
DSi PoisedLibrary_1680186639697.63	299.729	Z1004825200	-6.9083
DSi PoisedLibrary_1680186639697.63	299.729	Z1004825200	-6.809
DSi PoisedLibrary_1680186639697.246	228.126	Z44095027	-6.7407
DSi PoisedLibrary_1680186639697.103	248.321	Z54748186	-6.7304
DSi PoisedLibrary_1680186639697.254	175.075	Z1618027593	-6.7281
DSi PoisedLibrary_1680186639697.638	247.121	Z31432226	-6.72
DSi PoisedLibrary_1680186639697.68	203.106	Z26251905	-6.7176
DSi PoisedLibrary_1680186639697.448	247.677	Z57515803	-6.7014
DSi PoisedLibrary_1680186639697.59	223.112	Z198194394	-6.6978
DSi PoisedLibrary_1680186639697.638	247.121	Z31432226	-6.687
DSi PoisedLibrary_1680186639697.574	229.278	Z57715447	-6.6518
DSi PoisedLibrary_1680186639697.601	205.041	Z1020823294	-6.6391
DSi PoisedLibrary_1680186639697.103	248.321	Z54748186	-6.6383
DSi PoisedLibrary_1680186639697.769	219.137	Z1509441355	-6.6056

DSi PoisedLibrary_1680186639697.712	193.110	Z235361235	-6.6042
DSi PoisedLibrary_1680186639697.777	267.127	Z352356442	-6.602
DSi PoisedLibrary_1680186639697.306	246.237	Z1619958679	-6.5985
DSi PoisedLibrary_1680186639697.574	229.278	Z57715447	-6.5904
DSi PoisedLibrary_1680186639697.240	247.132	Z2472938267	-6.5798
DSi PoisedLibrary_1680186639697.511	214.237	Z1689442171	-6.5767
DSi PoisedLibrary_1680186639697.683	231.051	Z57111868	-6.5762
DSi PoisedLibrary_1680186639697.63	299.729	Z1004825200	-6.5739
DSi PoisedLibrary_1680186639697.806	175.056	Z1262633486	-6.5712
DSi PoisedLibrary_1680186639697.126	239.131	Z1342868616	-6.5632
DSi PoisedLibrary_1680186639697.602	153.059	Z1198169663	-6.5537
DSi PoisedLibrary_1680186639697.750	176.095	Z1192341021	-6.5508
DSi PoisedLibrary_1680186639697.777	267.127	Z352356442	-6.5485
DSi PoisedLibrary_1680186639697.541	244.078	Z1267773765	-6.5479
DSi PoisedLibrary_1680186639697.522	248.116	Z274555794	-6.5447
DSi PoisedLibrary_1680186639697.29	272.746	Z1457279522	-6.5431
DSi PoisedLibrary_1680186639697.605	198.056	Z1696091761	-6.5364
DSi PoisedLibrary_1680186639697.503	242.316	Z223388508	-6.5293
DSi PoisedLibrary_1680186639697.44	205.041	Z363993198	-6.5258
DSi PoisedLibrary_1680186639697.306	246.237	Z1619958679	-6.5225
DSi PoisedLibrary_1680186639697.306	246.237	Z1619958679	-6.5194
DSi PoisedLibrary_1680186639697.816	220.101	Z1220452176	-6.5042
DSi PoisedLibrary_1680186639697.260	171.101	Z90122368	-6.5037
DSi PoisedLibrary_1680186639697.687	204.126	Z381408780	-6.4888
DSi PoisedLibrary_1680186639697.36	239.131	Z1470724953	-6.4797
DSi PoisedLibrary_1680186639697.483	227.259	Z89307993	-6.4771
DSi PoisedLibrary_1680186639697.442	187.111	Z316121102	-6.4638
DSi PoisedLibrary_1680186639697.511	214.237	Z1689442171	-6.4581
DSi PoisedLibrary_1680186639697.36	239.131	Z1470724953	-6.4529
DSi PoisedLibrary_1680186639697.638	247.121	Z31432226	-6.4452
DSi PoisedLibrary_1680186639697.531	207.174	Z444860982	-6.4215
DSi PoisedLibrary_1680186639697.531	207.174	Z444860982	-6.403
DSi PoisedLibrary_1680186639697.433	197.105	Z1741959530	-6.3964
DSi PoisedLibrary_1680186639697.806	175.056	Z1262633486	-6.3939
DSi PoisedLibrary_1680186639697.230	165.115	Z104473836	-6.3924
DSi PoisedLibrary_1680186639697.63	299.729	Z1004825200	-6.3893
DSi PoisedLibrary_1680186639697.645	162.091	Z234898257	-6.3792
DSi PoisedLibrary_1680186639697.683	231.051	Z57111868	-6.3665
DSi PoisedLibrary_1680186639697.351	241.085	Z26781964	-6.3635
DSi PoisedLibrary_1680186639697.816	220.101	Z1220452176	-6.3538
DSi PoisedLibrary_1680186639697.29	272.746	Z1457279522	-6.3515
DSi PoisedLibrary_1680186639697.806	175.056	Z1262633486	-6.3509
DSi PoisedLibrary_1680186639697.57	230.038	Z594274330	-6.3467

DSi PoisedLibrary_1680186639697.276	191.056	Z169506466	-6.3362
DSi PoisedLibrary_1680186639697.121	233.095	Z26333434	-6.3319
DSi PoisedLibrary_1680186639697.483	227.259	Z89307993	-6.3202
DSi PoisedLibrary_1680186639697.261	170.106	Z32399802	-6.3086
DSi PoisedLibrary_1680186639697.349	205.102	Z445191014	-6.3059
DSi PoisedLibrary_1680186639697.531	207.174	Z444860982	-6.2777
DSi PoisedLibrary_1680186639697.309	209.217	Z1730522163	-6.2757
DSi PoisedLibrary_1680186639697.246	228.126	Z44095027	-6.2596
DSi PoisedLibrary_1680186639697.754	165.090	Z1324853681	-6.2535
DSi PoisedLibrary_1680186639697.541	244.078	Z1267773765	-6.2516
DSi PoisedLibrary_1680186639697.712	193.110	Z235361235	-6.2439
DSi PoisedLibrary_1680186639697.602	153.059	Z1198169663	-6.2427
DSi PoisedLibrary_1680186639697.236	226.078	Z1333043510	-6.2341
DSi PoisedLibrary_1680186639697.172	204.126	Z1206890404	-6.2243
DSi PoisedLibrary_1680186639697.602	153.059	Z1198169663	-6.2139
DSi PoisedLibrary_1680186639697.754	165.090	Z1324853681	-6.2124
DSi PoisedLibrary_1680186639697.59	223.112	Z198194394	-6.2036
DSi PoisedLibrary_1680186639697.696	166.099	Z1255402624	-6.1982
DSi PoisedLibrary_1680186639697.153	210.256	Z2033637875	-6.1965
DSi PoisedLibrary_1680186639697.57	230.038	Z594274330	-6.1884
DSi PoisedLibrary_1680186639697.829	191.086	Z431546706	-6.183
DSi PoisedLibrary_1680186639697.270	234.251	Z1545313172	-6.1784
DSi PoisedLibrary_1680186639697.208	189.077	Z56960214	-6.1724
DSi PoisedLibrary_1680186639697.78	199.051	Z906021418	-6.1614
DSi PoisedLibrary_1680186639697.713	167.095	Z57102475	-6.1586
DSi PoisedLibrary_1680186639697.278	241.010	Z1551999220	-6.1565
DSi PoisedLibrary_1680186639697.812	195.101	Z57627041	-6.1522
DSi PoisedLibrary_1680186639697.736	191.031	Z1222424326	-6.1496
DSi PoisedLibrary_1680186639697.466	221.080	Z33486130	-6.1485
DSi PoisedLibrary_1680186639697.315	201.067	Z1266933824	-6.1473
DSi PoisedLibrary_1680186639697.153	210.256	Z2033637875	-6.1462
DSi PoisedLibrary_1680186639697.754	165.090	Z1324853681	-6.1417
DSi PoisedLibrary_1680186639697.11	199.040	Z19727416	-6.1403
DSi PoisedLibrary_1680186639697.31	214.041	Z45656995	-6.1392
DSi PoisedLibrary_1680186639697.803	175.075	Z383202616	-6.1391
DSi PoisedLibrary_1680186639697.605	198.056	Z1696091761	-6.1391
DSi PoisedLibrary_1680186639697.713	167.095	Z57102475	-6.1323
DSi PoisedLibrary_1680186639697.399	238.326	Z32399948	-6.1257
DSi PoisedLibrary_1680186639697.812	195.101	Z57627041	-6.1191
DSi PoisedLibrary_1680186639697.645	162.091	Z234898257	-6.118
DSi PoisedLibrary_1680186639697.788	186.049	Z55671900	-6.1031
DSi PoisedLibrary_1680186639697.186	193.085	Z198195770	-6.1022
DSi PoisedLibrary_1680186639697.217	170.106	Z220816104	-6.0995

DSi PoisedLibrary_1680186639697.510	246.305	Z32400357	-6.0928
DSi PoisedLibrary_1680186639697.829	191.086	Z431546706	-6.0917
DSi PoisedLibrary_1680186639697.605	198.056	Z1696091761	-6.079
DSi PoisedLibrary_1680186639697.678	223.229	Z198194396	-6.0763
DSi PoisedLibrary_1680186639697.731	150.079	Z1203252645	-6.0737
DSi PoisedLibrary_1680186639697.236	226.078	Z1333043510	-6.0718
DSi PoisedLibrary_1680186639697.719	203.069	Z332370018	-6.0667
DSi PoisedLibrary_1680186639697.349	205.102	Z445191014	-6.0646
DSi PoisedLibrary_1680186639697.788	186.049	Z55671900	-6.0551
DSi PoisedLibrary_1680186639697.270	234.251	Z1545313172	-6.0547
DSi PoisedLibrary_1680186639697.217	170.106	Z220816104	-6.0501
DSi PoisedLibrary_1680186639697.230	165.115	Z104473836	-6.0488
DSi PoisedLibrary_1680186639697.718	251.280	Z55222357	-6.0389
DSi PoisedLibrary_1680186639697.78	199.051	Z906021418	-6.0316
DSi PoisedLibrary_1680186639697.750	176.095	Z1192341021	-6.0288
DSi PoisedLibrary_1680186639697.696	166.099	Z1255402624	-6.0248
DSi PoisedLibrary_1680186639697.736	191.031	Z1222424326	-6.0171
DSi PoisedLibrary_1680186639697.261	170.106	Z32399802	-6.0145
DSi PoisedLibrary_1680186639697.378	206.142	Z332382842	-6.0129
DSi PoisedLibrary_1680186639697.553	179.095	Z56960248	-6.0116
DSi PoisedLibrary_1680186639697.679	223.011	Z385450668	-6.006
DSi PoisedLibrary_1680186639697.348	177.090	Z1198172663	-6.0023
DSi PoisedLibrary_1680186639697.348	177.090	Z1198172663	-5.9888
DSi PoisedLibrary_1680186639697.617	139.075	Z432057220	-5.9863
DSi PoisedLibrary_1680186639697.31	214.041	Z45656995	-5.9855
DSi PoisedLibrary_1680186639697.754	165.090	Z1324853681	-5.9616
DSi PoisedLibrary_1680186639697.209	163.100	Z270979606	-5.9598
DSi PoisedLibrary_1680186639697.617	139.075	Z432057220	-5.9591
DSi PoisedLibrary_1680186639697.665	203.106	Z53116498	-5.9496
DSi PoisedLibrary_1680186639697.539	159.090	Z1436457631	-5.9394
DSi PoisedLibrary_1680186639697.2	227.163	Z44603686	-5.9376
DSi PoisedLibrary_1680186639697.165	149.084	Z52214433	-5.9273
DSi PoisedLibrary_1680186639697.260	171.101	Z90122368	-5.9223
DSi PoisedLibrary_1680186639697.477	147.068	Z1203730757	-5.904
DSi PoisedLibrary_1680186639697.803	175.075	Z383202616	-5.8985
DSi PoisedLibrary_1680186639697.754	165.090	Z1324853681	-5.8925
DSi PoisedLibrary_1680186639697.2	227.163	Z44603686	-5.8591
DSi PoisedLibrary_1680186639697.645	162.091	Z234898257	-5.8294
DSi PoisedLibrary_1680186639697.217	170.106	Z220816104	-5.8129
DSi PoisedLibrary_1680186639697.217	170.106	Z220816104	-5.8104
DSi PoisedLibrary_1680186639697.562	163.081	Z1198316457	-5.7904
DSi PoisedLibrary_1680186639697.803	175.075	Z383202616	-5.7288
DSi PoisedLibrary_1680186639697.802	179.062	Z425757818	-5.6783

DSi PoisedLibrary_1680186639697.599	142.111	Z439698382	-5.5533
DSi PoisedLibrary_1680186639697.817	173.167	Z1250093582	-5.4177

Table S11. VS1 and VS2 for PDB fragment library targeting PARG_6OAK

compound	WT	Energy in silico
VS1		
Serotonin-2	176.095	-7.4398
VS2		
Tryptamine-2	160.100	-7.4329
Umbelliferone-1	162.032	-6.8401
Umbelliferone-1	162.032	-6.8099
Caffeine-1	194.080	-6.801
4'-Hydroxyflavanone-2	240.079	-6.7829
4'-Hydroxyflavanone-2	240.079	-6.7539
Caffeine-1	194.080	-6.7431
2'-deoxyadenosine-1	251.102	-6.7295
N-(2-aminoethyl)isoquinoline-5-sulfonamide-2	251.073	-6.6981
Serotonin-2	176.095	-6.6724
2'-deoxyadenosine-1	251.102	-6.6556
4'-Hydroxyflavanone-1	240.079	-6.5918
Caffeine-1	194.080	-6.5892
4-(Imidazol-1-yl)phenol-1	160.064	-6.5878
octahydroindolizine-1,6,7,8-tetrol-49	189.100	-6.5671
2-Naphthimidamide-2	170.084	-6.5328
octahydroindolizine-1,6,7,8-tetrol-49	189.100	-6.519
4'-Hydroxyflavanone-2	240.079	-6.4924
octahydroindolizine-1,6,7,8-tetrol-53	189.100	-6.4831
octahydroindolizine-1,6,7,8-tetrol-35	189.100	-6.4755
octahydroindolizine-1,6,7,8-tetrol-49	189.100	-6.4718
octahydroindolizine-1,6,7,8-tetrol-53	189.100	-6.4691
octahydroindolizine-1,6,7,8-tetrol-41	189.100	-6.4295
octahydroindolizine-1,6,7,8-tetrol-41	189.100	-6.4253
2-Naphthimidamide-2	170.084	-6.4246
9-Ethyladenine-1	163.086	-6.4123
octahydroindolizine-1,6,7,8-tetrol-53	189.100	-6.4049
octahydroindolizine-1,6,7,8-tetrol-43	189.100	-6.4049
6-Benzylaminopurine-2	225.101	-6.4038
Serotonin-2	176.095	-6.3907
N-(2-aminoethyl)isoquinoline-5-sulfonamide-2	251.073	-6.3838
octahydroindolizine-1,6,7,8-tetrol-55	189.100	-6.3305

octahydroindolizine-1,6,7,8-tetrol-41	189.100	-6.3255
6-Benzylaminopurine-2	225.101	-6.3198
octahydroindolizine-1,6,7,8-tetrol-40	189.100	-6.3022
Difunisal-2	250.044	-6.2775
octahydroindolizine-1,6,7,8-tetrol-44	189.100	-6.2696
Cordycepin-1	251.102	-6.2654
Isopentenyladenine-2	203.117	-6.2407
octahydroindolizine-1,6,7,8-tetrol-34	189.100	-6.2175
tyrosinamide-2	180.090	-6.2074
octahydroindolizine-1,6,7,8-tetrol-63	189.100	-6.1775
N-(2-aminoethyl)isoquinoline-5-sulfonamide-2	251.073	-6.1744
octahydroindolizine-1,6,7,8-tetrol-63	189.100	-6.1738
Difunisal-2	250.044	-6.1058
tyrosinamide-2	180.090	-6.0304
5-Carboxy-8-hydroxyquinoline-2	189.043	-6.0263
5-Hydroxyindole-3-acetic acid-2	191.058	-6.0238
Piracetam-1	142.074	-6.0088
5-Carboxy-8-hydroxyquinoline-2	189.043	-6.0055
tyrosinamide-2	180.090	-5.953
Piracetam-1	142.074	-5.9152
Serotonin-2	176.095	-5.8236
(1H-Indol-3-yl)acetic acid-2	175.063	-5.8012
5-Hydroxyindole-3-acetic acid-2	191.058	-5.6904
(1H-Indol-3-yl)acetic acid-2	175.063	-5.6232

Table S12. VS1 and VS2 for LH1 FDA approved drugs targeting PARG_6OAK

compound	Name	MolWt	Energy in silico
VS1			
MOlport_LH1_Approved_FDA_library.maegz:605	Proanthocyanidins	594.520	-12.1682
VS2			
MOlport_LH1_Approved_FDA_library.maegz:605	Proanthocyanidins	594.520	-8.87
MOlport_LH1_Approved_FDA_library.maegz:711	Baricitinib	371.420	-8.8557
MOlport_LH1_Approved_FDA_library.maegz:750	Baricitinib?Phosphate	469.410	-8.8557
MOlport_LH1_Approved_FDA_library.maegz:605	Proanthocyanidins	594.520	-8.7537
MOlport_LH1_Approved_FDA_library.maegz:434	Goserelin acetate	1329.460	-8.6933
MOlport_LH1_Approved_FDA_library.maegz:605	Proanthocyanidins	594.520	-8.5999
MOlport_LH1_Approved_FDA_library.maegz:172	Atracurium besylate	1243.490	-8.5449
MOlport_LH1_Approved_FDA_library.maegz:172	Atracurium besylate	1243.490	-8.5294
MOlport_LH1_Approved_FDA_library.maegz:1000	Darifenacin HBr	505.450	-8.4645
MOlport_LH1_Approved_FDA_library.maegz:1379	Hesperidin	610.560	-8.4057
MOlport_LH1_Approved_FDA_library.maegz:1379	Hesperidin	610.560	-8.3732

MOlport_LH1_Approved_FDA_library.maegz:92	Silymarin	482.440	-8.3414
MOlport_LH1_Approved_FDA_library.maegz:172	Atracurium besylate	1243.490	-8.2899
MOlport_LH1_Approved_FDA_library.maegz:315	Desmopressin	1069.220	-8.2843
MOlport_LH1_Approved_FDA_library.maegz:361	Pentagastrin	767.890	-8.2825
MOlport_LH1_Approved_FDA_library.maegz:268	Travoprost	500.550	-8.265
MOlport_LH1_Approved_FDA_library.maegz:1322	Polymyxin B sulfate	1301.560	-8.2615
MOlport_LH1_Approved_FDA_library.maegz:1398	Itraconazole	705.630	-8.2089
MOlport_LH1_Approved_FDA_library.maegz:1684	Naringin	580.530	-8.2088
MOlport_LH1_Approved_FDA_library.maegz:315	Desmopressin	1069.220	-8.1615
MOlport_LH1_Approved_FDA_library.maegz:717	Tenofovir alafenamide	476.470	-8.1389
MOlport_LH1_Approved_FDA_library.maegz:268	Travoprost	500.550	-8.136
MOlport_LH1_Approved_FDA_library.maegz:1322	Polymyxin B sulfate	1301.560	-8.1083
MOlport_LH1_Approved_FDA_library.maegz:19	Revefenacin	597.750	-8.0906
MOlport_LH1_Approved_FDA_library.maegz:237	S-Ruxolitinib	306.370	-8.0453
MOlport_LH1_Approved_FDA_library.maegz:1379	Hesperidin	610.560	-8.043
MOlport_LH1_Approved_FDA_library.maegz:711	Baricitinib	371.420	-7.9892
MOlport_LH1_Approved_FDA_library.maegz:750	Baricitinib?Phosphate	469.410	-7.9892
MOlport_LH1_Approved_FDA_library.maegz:465	Ivosidenib	582.970	-7.9768
MOlport_LH1_Approved_FDA_library.maegz:659	Dienogest	311.420	-7.9744
MOlport_LH1_Approved_FDA_library.maegz:465	Ivosidenib	582.970	-7.9585
MOlport_LH1_Approved_FDA_library.maegz:268	Travoprost	500.550	-7.9505
MOlport_LH1_Approved_FDA_library.maegz:822	ABT199	868.440	-7.9491
MOlport_LH1_Approved_FDA_library.maegz:268	Travoprost	500.550	-7.932
MOlport_LH1_Approved_FDA_library.maegz:1684	Naringin	580.530	-7.9095
MOlport_LH1_Approved_FDA_library.maegz:92	Silymarin	482.440	-7.905
MOlport_LH1_Approved_FDA_library.maegz:268	Travoprost	500.550	-7.9045
MOlport_LH1_Approved_FDA_library.maegz:237	S-Ruxolitinib	306.370	-7.9011
MOlport_LH1_Approved_FDA_library.maegz:268	Travoprost	500.550	-7.8963
MOlport_LH1_Approved_FDA_library.maegz:822	ABT199	868.440	-7.8961
MOlport_LH1_Approved_FDA_library.maegz:21	Tenofovir alafenamide fumarate	534.500	-7.8887
MOlport_LH1_Approved_FDA_library.maegz:268	Travoprost	500.550	-7.8886
MOlport_LH1_Approved_FDA_library.maegz:547	Ruxolitinib (INCB-18424) phosphate	404.360	-7.8819
MOlport_LH1_Approved_FDA_library.maegz:843	Ruxolitinib	306.370	-7.8819
MOlport_LH1_Approved_FDA_library.maegz:547	Ruxolitinib (INCB-18424) phosphate	404.360	-7.8799
MOlport_LH1_Approved_FDA_library.maegz:843	Ruxolitinib	306.370	-7.8799
MOlport_LH1_Approved_FDA_library.maegz:1009	Ritonavir	720.960	-7.8791
MOlport_LH1_Approved_FDA_library.maegz:363	Perospirone	426.570	-7.8694
MOlport_LH1_Approved_FDA_library.maegz:1684	Naringin	580.530	-7.868
MOlport_LH1_Approved_FDA_library.maegz:451	Isavuconazonium sulfate	814.840	-7.8578
MOlport_LH1_Approved_FDA_library.maegz:1009	Ritonavir	720.960	-7.8524

MOlport_LH1_Approved_FDA_library.maegz:92	Silymarin	482.440	-7.8497
MOlport_LH1_Approved_FDA_library.maegz:451	Isavuconazonium sulfate	814.840	-7.8405
MOlport_LH1_Approved_FDA_library.maegz:92	Silymarin	482.440	-7.8305
MOlport_LH1_Approved_FDA_library.maegz:361	Pentagastrin	767.890	-7.8283
MOlport_LH1_Approved_FDA_library.maegz:172	Atracurium besylate	1243.490	-7.7979
MOlport_LH1_Approved_FDA_library.maegz:659	Dienogest	311.420	-7.7874
MOlport_LH1_Approved_FDA_library.maegz:848	Micafungin sodium	1292.260	-7.7856
MOlport_LH1_Approved_FDA_library.maegz:659	Dienogest	311.420	-7.7843
MOlport_LH1_Approved_FDA_library.maegz:1398	Itraconazole	705.630	-7.7694
MOlport_LH1_Approved_FDA_library.maegz:265	Indinavir sulfate	711.870	-7.7659
MOlport_LH1_Approved_FDA_library.maegz:451	Isavuconazonium sulfate	814.840	-7.7613
MOlport_LH1_Approved_FDA_library.maegz:930	Trazodone hydrochloride	408.330	-7.7606
MOlport_LH1_Approved_FDA_library.maegz:2093	Haloperidol	375.870	-7.755
MOlport_LH1_Approved_FDA_library.maegz:750	Baricitinib?Phosphate	469.410	-7.7436
MOlport_LH1_Approved_FDA_library.maegz:711	Baricitinib	371.420	-7.743
MOlport_LH1_Approved_FDA_library.maegz:268	Travoprost	500.550	-7.7366
MOlport_LH1_Approved_FDA_library.maegz:172	Atracurium besylate	1243.490	-7.7267
MOlport_LH1_Approved_FDA_library.maegz:265	Indinavir sulfate	711.870	-7.7179
MOlport_LH1_Approved_FDA_library.maegz:92	Silymarin	482.440	-7.7166
MOlport_LH1_Approved_FDA_library.maegz:92	Silymarin	482.440	-7.7116
MOlport_LH1_Approved_FDA_library.maegz:714	Luliconazole	354.280	-7.6999
MOlport_LH1_Approved_FDA_library.maegz:268	Travoprost	500.550	-7.6976
MOlport_LH1_Approved_FDA_library.maegz:1398	Itraconazole	705.630	-7.6937
MOlport_LH1_Approved_FDA_library.maegz:1597	Casanthranol	396.580	-7.6859
MOlport_LH1_Approved_FDA_library.maegz:1597	Casanthranol	396.580	-7.684
MOlport_LH1_Approved_FDA_library.maegz:1322	Polymyxin B sulfate	1301.560	-7.6767
MOlport_LH1_Approved_FDA_library.maegz:1417	Droperidol	379.430	-7.6697
MOlport_LH1_Approved_FDA_library.maegz:213	Vilazodone HCl	477.990	-7.6631
MOlport_LH1_Approved_FDA_library.maegz:265	Indinavir sulfate	711.870	-7.6588
MOlport_LH1_Approved_FDA_library.maegz:102	Pirarubicin	627.640	-7.6566
MOlport_LH1_Approved_FDA_library.maegz:547	Ruxolitinib (INCB-18424) phosphate	404.360	-7.6521
MOlport_LH1_Approved_FDA_library.maegz:843	Ruxolitinib	306.370	-7.6521
MOlport_LH1_Approved_FDA_library.maegz:265	Indinavir sulfate	711.870	-7.6444
MOlport_LH1_Approved_FDA_library.maegz:1597	Casanthranol	396.580	-7.6324
MOlport_LH1_Approved_FDA_library.maegz:265	Indinavir sulfate	711.870	-7.6304
MOlport_LH1_Approved_FDA_library.maegz:1597	Casanthranol	396.580	-7.6266
MOlport_LH1_Approved_FDA_library.maegz:268	Travoprost	500.550	-7.62
MOlport_LH1_Approved_FDA_library.maegz:237	S-Ruxolitinib	306.370	-7.6193
MOlport_LH1_Approved_FDA_library.maegz:1597	Casanthranol	396.580	-7.6171
MOlport_LH1_Approved_FDA_library.maegz:268	Travoprost	500.550	-7.6114
MOlport_LH1_Approved_FDA_library.maegz:1597	Casanthranol	396.580	-7.6107
MOlport_LH1_Approved_FDA_library.maegz:911	Ciprofloxacin	331.340	-7.61

MOlport_LH1_Approved_FDA_library.maegz:1901	Ciprofloxacin hydrochloride	367.800	-7.61
MOlport_LH1_Approved_FDA_library.maegz:92	Silymarin	482.440	-7.6098
MOlport_LH1_Approved_FDA_library.maegz:361	Pentagastrin	767.890	-7.5944
MOlport_LH1_Approved_FDA_library.maegz:92	Silymarin	482.440	-7.5918
MOlport_LH1_Approved_FDA_library.maegz:666	Etonogestrel	324.460	-7.5888
MOlport_LH1_Approved_FDA_library.maegz:1398	Itraconazole	705.630	-7.5881
MOlport_LH1_Approved_FDA_library.maegz:1181	Vilazodone	441.530	-7.5869
MOlport_LH1_Approved_FDA_library.maegz:897	Pentamidine isethionate	592.680	-7.581
MOlport_LH1_Approved_FDA_library.maegz:92	Silymarin	482.440	-7.5751
MOlport_LH1_Approved_FDA_library.maegz:168	Besifloxacin HCl	430.300	-7.563
MOlport_LH1_Approved_FDA_library.maegz:1009	Ritonavir	720.960	-7.5589
MOlport_LH1_Approved_FDA_library.maegz:92	Silymarin	482.440	-7.5586
MOlport_LH1_Approved_FDA_library.maegz:1398	Itraconazole	705.630	-7.5564
MOlport_LH1_Approved_FDA_library.maegz:2058	Fluphenazine hydrochloride	510.440	-7.5542
MOlport_LH1_Approved_FDA_library.maegz:1597	Casanthranol	396.580	-7.5515
MOlport_LH1_Approved_FDA_library.maegz:56	Darolutamide	398.850	-7.5488
MOlport_LH1_Approved_FDA_library.maegz:161	Cefoperazone	645.670	-7.5485
MOlport_LH1_Approved_FDA_library.maegz:32	Tenofovir Disoproxil	519.440	-7.5463
MOlport_LH1_Approved_FDA_library.maegz:1398	Itraconazole	705.630	-7.5338
MOlport_LH1_Approved_FDA_library.maegz:58	Netupitant	578.590	-7.532
MOlport_LH1_Approved_FDA_library.maegz:1181	Vilazodone	441.530	-7.5302
MOlport_LH1_Approved_FDA_library.maegz:848	Micafungin sodium	1292.260	-7.5187
MOlport_LH1_Approved_FDA_library.maegz:22	Gestrinone	308.410	-7.5142
MOlport_LH1_Approved_FDA_library.maegz:265	Indinavir sulfate	711.870	-7.5099
MOlport_LH1_Approved_FDA_library.maegz:265	Indinavir sulfate	711.870	-7.5094
MOlport_LH1_Approved_FDA_library.maegz:1012	Hydroxy Camptothecine	364.350	-7.5037
MOlport_LH1_Approved_FDA_library.maegz:348	10-Hydroxycampothecin	364.350	-7.5034
MOlport_LH1_Approved_FDA_library.maegz:1337	Nafronyl oxalate	473.560	-7.4959
MOlport_LH1_Approved_FDA_library.maegz:1597	Casanthranol	396.580	-7.49
MOlport_LH1_Approved_FDA_library.maegz:268	Travoprost	500.550	-7.4896
MOlport_LH1_Approved_FDA_library.maegz:1012	Hydroxy Camptothecine	364.350	-7.4804
MOlport_LH1_Approved_FDA_library.maegz:348	10-Hydroxycampothecin	364.350	-7.4801
MOlport_LH1_Approved_FDA_library.maegz:618	10-Hydroxycamptothecin	364.360	-7.4801
MOlport_LH1_Approved_FDA_library.maegz:2068	Famprofazone	377.520	-7.4758
MOlport_LH1_Approved_FDA_library.maegz:451	Isavuconazonium sulfate	814.840	-7.4746
MOlport_LH1_Approved_FDA_library.maegz:245	Anidulafungin	1140.240	-7.4738
MOlport_LH1_Approved_FDA_library.maegz:56	Darolutamide	398.850	-7.472
MOlport_LH1_Approved_FDA_library.maegz:256	Nelfinavir Mesylate	663.890	-7.4693
MOlport_LH1_Approved_FDA_library.maegz:161	Cefoperazone	645.670	-7.4654
MOlport_LH1_Approved_FDA_library.maegz:585	Tanshinone IIA sulfonate sodium	396.390	-7.4514
MOlport_LH1_Approved_FDA_library.maegz:1839	Carvedilol phosphate	522.490	-7.449
MOlport_LH1_Approved_FDA_library.maegz:1918	Methyl hesperidin	624.590	-7.4466

MOlport_LH1_Approved_FDA_library.maegz:1751	Carvedilol	406.470	-7.4448
MOlport_LH1_Approved_FDA_library.maegz:1597	Casanthranol	396.580	-7.4358
MOlport_LH1_Approved_FDA_library.maegz:1337	Nafronyl oxalate	473.560	-7.4267
MOlport_LH1_Approved_FDA_library.maegz:1450	Hydroxychloroquine sulfate	433.950	-7.4238
MOlport_LH1_Approved_FDA_library.maegz:233	Telaprevir	679.850	-7.4222
MOlport_LH1_Approved_FDA_library.maegz:588	Apremilast	460.500	-7.4217
MOlport_LH1_Approved_FDA_library.maegz:265	Indinavir sulfate	711.870	-7.42
MOlport_LH1_Approved_FDA_library.maegz:2068	Famprofazone	377.520	-7.4158
MOlport_LH1_Approved_FDA_library.maegz:1174	Synephrine	167.210	-7.4123
MOlport_LH1_Approved_FDA_library.maegz:265	Indinavir sulfate	711.870	-7.4122
MOlport_LH1_Approved_FDA_library.maegz:1174	Synephrine	167.210	-7.4081
MOlport_LH1_Approved_FDA_library.maegz:1575	Antrapurol	240.220	-7.408
MOlport_LH1_Approved_FDA_library.maegz:22	Gestrinone	308.410	-7.4073
MOlport_LH1_Approved_FDA_library.maegz:1575	Antrapurol	240.220	-7.405
MOlport_LH1_Approved_FDA_library.maegz:1012	Hydroxy Camptothecine	364.350	-7.4039
MOlport_LH1_Approved_FDA_library.maegz:348	10-Hydroxycampothecin	364.350	-7.403
MOlport_LH1_Approved_FDA_library.maegz:618	10-Hydroxycamptothecin	364.360	-7.403
MOlport_LH1_Approved_FDA_library.maegz:1597	Casanthranol	396.580	-7.4009
MOlport_LH1_Approved_FDA_library.maegz:1450	Hydroxychloroquine sulfate	433.950	-7.4
MOlport_LH1_Approved_FDA_library.maegz:1450	Hydroxychloroquine sulfate	433.950	-7.3982
MOlport_LH1_Approved_FDA_library.maegz:348	10-Hydroxycampothecin	364.350	-7.397
MOlport_LH1_Approved_FDA_library.maegz:618	10-Hydroxycamptothecin	364.360	-7.397
MOlport_LH1_Approved_FDA_library.maegz:1597	Casanthranol	396.580	-7.3969
MOlport_LH1_Approved_FDA_library.maegz:1012	Hydroxy Camptothecine	364.350	-7.3958
MOlport_LH1_Approved_FDA_library.maegz:265	Indinavir sulfate	711.870	-7.3945
MOlport_LH1_Approved_FDA_library.maegz:348	10-Hydroxycampothecin	364.350	-7.3871
MOlport_LH1_Approved_FDA_library.maegz:857	Canagliflozin	444.520	-7.3855
MOlport_LH1_Approved_FDA_library.maegz:1012	Hydroxy Camptothecine	364.350	-7.3841
MOlport_LH1_Approved_FDA_library.maegz:848	Micafungin sodium	1292.260	-7.3788
MOlport_LH1_Approved_FDA_library.maegz:265	Indinavir sulfate	711.870	-7.3784
MOlport_LH1_Approved_FDA_library.maegz:714	Luliconazole	354.280	-7.3692
MOlport_LH1_Approved_FDA_library.maegz:848	Micafungin sodium	1292.260	-7.3636
MOlport_LH1_Approved_FDA_library.maegz:265	Indinavir sulfate	711.870	-7.3586
MOlport_LH1_Approved_FDA_library.maegz:1349	Anthralin	226.230	-7.358
MOlport_LH1_Approved_FDA_library.maegz:2000	Sanguinarine chloride	367.780	-7.3559
MOlport_LH1_Approved_FDA_library.maegz:1428	Dequalinium chloride	527.590	-7.3543
MOlport_LH1_Approved_FDA_library.maegz:17	Ademetionine	398.440	-7.3489
MOlport_LH1_Approved_FDA_library.maegz:605	Proanthocyanidins	594.520	-7.3424
MOlport_LH1_Approved_FDA_library.maegz:245	Anidulafungin	1140.240	-7.3421
MOlport_LH1_Approved_FDA_library.maegz:265	Indinavir sulfate	711.870	-7.3317
MOlport_LH1_Approved_FDA_library.maegz:1597	Casanthranol	396.580	-7.3305
MOlport_LH1_Approved_FDA_library.maegz:1059	Nadifloxacin	360.390	-7.3301
MOlport_LH1_Approved_FDA_library.maegz:434	Goserelin acetate	1329.460	-7.3266

MOlport_LH1_Approved_FDA_library.maegz:268	Travoprost	500.550	-7.3249
MOlport_LH1_Approved_FDA_library.maegz:1018	Norethindrone	298.420	-7.3197
MOlport_LH1_Approved_FDA_library.maegz:22	Gestrinone	308.410	-7.3148
MOlport_LH1_Approved_FDA_library.maegz:1226	Butenafine hydrochloride	353.930	-7.3126
MOlport_LH1_Approved_FDA_library.maegz:733	Nafamostat mesylate	539.580	-7.312
MOlport_LH1_Approved_FDA_library.maegz:410	Roflumilast N-oxide	419.210	-7.3111
MOlport_LH1_Approved_FDA_library.maegz:1794	Linezolid	337.350	-7.3108
MOlport_LH1_Approved_FDA_library.maegz:265	Indinavir sulfate	711.870	-7.3063
MOlport_LH1_Approved_FDA_library.maegz:993	Naftifine hydrochloride	323.860	-7.3042
MOlport_LH1_Approved_FDA_library.maegz:265	Indinavir sulfate	711.870	-7.2996
MOlport_LH1_Approved_FDA_library.maegz:1349	Anthralin	226.230	-7.2992
MOlport_LH1_Approved_FDA_library.maegz:1794	Linezolid	337.350	-7.2958
MOlport_LH1_Approved_FDA_library.maegz:9	Levomefolate calcium	497.520	-7.2817
MOlport_LH1_Approved_FDA_library.maegz:1794	Linezolid	337.350	-7.2793
MOlport_LH1_Approved_FDA_library.maegz:1522	Mefloquine hydrochloride	414.770	-7.2768
MOlport_LH1_Approved_FDA_library.maegz:1751	Carvedilol	406.470	-7.2723
MOlport_LH1_Approved_FDA_library.maegz:1839	Carvedilol phosphate	522.490	-7.2702
MOlport_LH1_Approved_FDA_library.maegz:666	Etonogestrel	324.460	-7.2666
MOlport_LH1_Approved_FDA_library.maegz:911	Ciprofloxacin	331.340	-7.2648
MOlport_LH1_Approved_FDA_library.maegz:1901	Ciprofloxacin hydrochloride	367.800	-7.2648
MOlport_LH1_Approved_FDA_library.maegz:56	Darolutamide	398.850	-7.2646
MOlport_LH1_Approved_FDA_library.maegz:1322	Polymyxin B sulfate	1301.560	-7.2627
MOlport_LH1_Approved_FDA_library.maegz:92	Silymarin	482.440	-7.2614
MOlport_LH1_Approved_FDA_library.maegz:9	Levomefolate calcium	497.520	-7.2593
MOlport_LH1_Approved_FDA_library.maegz:1597	Casanthranol	396.580	-7.2589
MOlport_LH1_Approved_FDA_library.maegz:21	Tenofovir alafenamide fumarate	534.500	-7.2575
MOlport_LH1_Approved_FDA_library.maegz:245	Anidulafungin	1140.240	-7.2572
MOlport_LH1_Approved_FDA_library.maegz:2058	Fluphenazine hydrochloride	510.440	-7.2489
MOlport_LH1_Approved_FDA_library.maegz:1253	Rivaroxaban	435.880	-7.2481
MOlport_LH1_Approved_FDA_library.maegz:92	Silymarin	482.440	-7.2464
MOlport_LH1_Approved_FDA_library.maegz:1597	Casanthranol	396.580	-7.2449
MOlport_LH1_Approved_FDA_library.maegz:1597	Casanthranol	396.580	-7.2428
MOlport_LH1_Approved_FDA_library.maegz:1067	Deferasirox	373.360	-7.2416
MOlport_LH1_Approved_FDA_library.maegz:1181	Vilazodone	441.530	-7.2395
MOlport_LH1_Approved_FDA_library.maegz:810	Eburnalritardo	294.390	-7.2347
MOlport_LH1_Approved_FDA_library.maegz:265	Indinavir sulfate	711.870	-7.2345
MOlport_LH1_Approved_FDA_library.maegz:17	Ademetionine	398.440	-7.2319
MOlport_LH1_Approved_FDA_library.maegz:733	Nafamostat mesylate	539.580	-7.2163
MOlport_LH1_Approved_FDA_library.maegz:92	Silymarin	482.440	-7.2158
MOlport_LH1_Approved_FDA_library.maegz:969	Estriol	288.390	-7.2138
MOlport_LH1_Approved_FDA_library.maegz:666	Etonogestrel	324.460	-7.2092
MOlport_LH1_Approved_FDA_library.maegz:1450	Hydroxychloroquine sulfate	433.950	-7.2079

MOlport_LH1_Approved_FDA_library.maegz:1337	Nafronyl oxalate	473.560	-7.2021
MOlport_LH1_Approved_FDA_library.maegz:17	Ademetionine	398.440	-7.2015
MOlport_LH1_Approved_FDA_library.maegz:969	Estriol	288.390	-7.2
MOlport_LH1_Approved_FDA_library.maegz:4	Temocapil	476.600	-7.195
MOlport_LH1_Approved_FDA_library.maegz:92	Silymarin	482.440	-7.1948
MOlport_LH1_Approved_FDA_library.maegz:1400	Aquacrine	270.370	-7.1948
MOlport_LH1_Approved_FDA_library.maegz:265	Indinavir sulfate	711.870	-7.1939
MOlport_LH1_Approved_FDA_library.maegz:1337	Nafronyl oxalate	473.560	-7.1925
MOlport_LH1_Approved_FDA_library.maegz:17	Ademetionine	398.440	-7.1801
MOlport_LH1_Approved_FDA_library.maegz:1517	Propafenone	341.440	-7.1797
MOlport_LH1_Approved_FDA_library.maegz:265	Indinavir sulfate	711.870	-7.1796
MOlport_LH1_Approved_FDA_library.maegz:265	Indinavir sulfate	711.870	-7.1794
MOlport_LH1_Approved_FDA_library.maegz:2056	Pentoxifylline	278.310	-7.1788
MOlport_LH1_Approved_FDA_library.maegz:9	Levomefolate calcium	497.520	-7.1776
MOlport_LH1_Approved_FDA_library.maegz:1839	Carvedilol phosphate	522.490	-7.1688
MOlport_LH1_Approved_FDA_library.maegz:1751	Carvedilol	406.470	-7.1658
MOlport_LH1_Approved_FDA_library.maegz:1018	Norethindrone	298.420	-7.1648
MOlport_LH1_Approved_FDA_library.maegz:1773	Pefloxacin Mesylate	429.460	-7.1636
MOlport_LH1_Approved_FDA_library.maegz:1772	Pefloxacin Mesylate Dihydrate	465.490	-7.1633
MOlport_LH1_Approved_FDA_library.maegz:92	Silymarin	482.440	-7.1596
MOlport_LH1_Approved_FDA_library.maegz:363	Perospirone	426.570	-7.1578
MOlport_LH1_Approved_FDA_library.maegz:265	Indinavir sulfate	711.870	-7.1568
MOlport_LH1_Approved_FDA_library.maegz:259	Larotrectinib	428.440	-7.1567
MOlport_LH1_Approved_FDA_library.maegz:9	Levomefolate calcium	497.520	-7.1533
MOlport_LH1_Approved_FDA_library.maegz:61	Larotrectinib sulfate	526.510	-7.1528
MOlport_LH1_Approved_FDA_library.maegz:1322	Polymyxin B sulfate	1301.560	-7.1525
MOlport_LH1_Approved_FDA_library.maegz:605	Proanthocyanidins	594.520	-7.1516
MOlport_LH1_Approved_FDA_library.maegz:434	Goserelin acetate	1329.460	-7.1477
MOlport_LH1_Approved_FDA_library.maegz:265	Indinavir sulfate	711.870	-7.1456
MOlport_LH1_Approved_FDA_library.maegz:1597	Casanthranol	396.580	-7.144
MOlport_LH1_Approved_FDA_library.maegz:847	Carfilzomib	719.910	-7.1416
MOlport_LH1_Approved_FDA_library.maegz:969	Estriol	288.390	-7.1414
MOlport_LH1_Approved_FDA_library.maegz:924	Lomefloxacin hydrochloride	387.810	-7.1405
MOlport_LH1_Approved_FDA_library.maegz:1076	Lomefloxacin	351.350	-7.1405
MOlport_LH1_Approved_FDA_library.maegz:268	Travoprost	500.550	-7.1351
MOlport_LH1_Approved_FDA_library.maegz:265	Indinavir sulfate	711.870	-7.1255
MOlport_LH1_Approved_FDA_library.maegz:863	Empagliflozin	450.910	-7.125
MOlport_LH1_Approved_FDA_library.maegz:348	10-Hydroxycampothecin	364.350	-7.1196
MOlport_LH1_Approved_FDA_library.maegz:1012	Hydroxy Camptothecine	364.350	-7.1193
MOlport_LH1_Approved_FDA_library.maegz:143	Donepezil HCl	415.960	-7.117
MOlport_LH1_Approved_FDA_library.maegz:41	Donepezil	379.500	-7.1161
MOlport_LH1_Approved_FDA_library.maegz:268	Travoprost	500.550	-7.1153

MOlport_LH1_Approved_FDA_library.maegz:265	Indinavir sulfate	711.870	-7.1082
MOlport_LH1_Approved_FDA_library.maegz:1751	Carvedilol	406.470	-7.1078
MOlport_LH1_Approved_FDA_library.maegz:1839	Carvedilol phosphate	522.490	-7.1057
MOlport_LH1_Approved_FDA_library.maegz:436	Anlotinib Dihydrochloride	480.360	-7.1037
MOlport_LH1_Approved_FDA_library.maegz:479	Anlotinib	407.440	-7.1037
MOlport_LH1_Approved_FDA_library.maegz:717	Tenofovir alafenamide	476.470	-7.0999
MOlport_LH1_Approved_FDA_library.maegz:268	Travoprost	500.550	-7.0956
MOlport_LH1_Approved_FDA_library.maegz:92	Silymarin	482.440	-7.0856
MOlport_LH1_Approved_FDA_library.maegz:265	Indinavir sulfate	711.870	-7.0852
MOlport_LH1_Approved_FDA_library.maegz:810	Eburnalritardo	294.390	-7.0851
MOlport_LH1_Approved_FDA_library.maegz:265	Indinavir sulfate	711.870	-7.0836
MOlport_LH1_Approved_FDA_library.maegz:1159	Norfloxacin	319.340	-7.0785
MOlport_LH1_Approved_FDA_library.maegz:877	Aminophylline	420.430	-7.0764
MOlport_LH1_Approved_FDA_library.maegz:17	Ademetionine	398.440	-7.0703
MOlport_LH1_Approved_FDA_library.maegz:2058	Fluphenazine hydrochloride	510.440	-7.0703
MOlport_LH1_Approved_FDA_library.maegz:1918	Methyl hesperidin	624.590	-7.0696
MOlport_LH1_Approved_FDA_library.maegz:287	Nestoron	370.480	-7.0691
MOlport_LH1_Approved_FDA_library.maegz:924	Lomefloxacin hydrochloride	387.810	-7.0665
MOlport_LH1_Approved_FDA_library.maegz:1076	Lomefloxacin	351.350	-7.0665
MOlport_LH1_Approved_FDA_library.maegz:268	Travoprost	500.550	-7.063
MOlport_LH1_Approved_FDA_library.maegz:1597	Casanthranol	396.580	-7.0571
MOlport_LH1_Approved_FDA_library.maegz:265	Indinavir sulfate	711.870	-7.0565
MOlport_LH1_Approved_FDA_library.maegz:434	Goserelin acetate	1329.460	-7.0524
MOlport_LH1_Approved_FDA_library.maegz:1059	Nadifloxacin	360.390	-7.0511
MOlport_LH1_Approved_FDA_library.maegz:265	Indinavir sulfate	711.870	-7.0401
MOlport_LH1_Approved_FDA_library.maegz:911	Ciprofloxacin	331.340	-7.0355
MOlport_LH1_Approved_FDA_library.maegz:1901	Ciprofloxacin hydrochloride	367.800	-7.0355
MOlport_LH1_Approved_FDA_library.maegz:1575	Antrapurol	240.220	-7.0329
MOlport_LH1_Approved_FDA_library.maegz:1120	4-Methylumbelliferone	176.170	-7.0316
MOlport_LH1_Approved_FDA_library.maegz:1194	Levonorgestrel	312.450	-7.0283
MOlport_LH1_Approved_FDA_library.maegz:2000	Sanguinarine chloride	367.780	-7.0282
MOlport_LH1_Approved_FDA_library.maegz:1511	Dinitolmide	225.160	-7.0256
MOlport_LH1_Approved_FDA_library.maegz:1942	Meptazinol hydrochloride	269.810	-7.0235
MOlport_LH1_Approved_FDA_library.maegz:1092	Trifluridine	296.200	-7.0203
MOlport_LH1_Approved_FDA_library.maegz:1059	Nadifloxacin	360.390	-7.0179
MOlport_LH1_Approved_FDA_library.maegz:1183	Danofloxacin mesylate	453.480	-7.0173
MOlport_LH1_Approved_FDA_library.maegz:434	Goserelin acetate	1329.460	-7.0168
MOlport_LH1_Approved_FDA_library.maegz:1183	Danofloxacin mesylate	453.480	-7.0165
MOlport_LH1_Approved_FDA_library.maegz:848	Micafungin sodium	1292.260	-7.0131
MOlport_LH1_Approved_FDA_library.maegz:17	Ademetionine	398.440	-7.0124
MOlport_LH1_Approved_FDA_library.maegz:1597	Casanthranol	396.580	-7.0113
MOlport_LH1_Approved_FDA_library.maegz:17	Ademetionine	398.440	-7.0092
MOlport_LH1_Approved_FDA_library.maegz:1120	4-Methylumbelliferone	176.170	-7.0079

MOlport_LH1_Approved_FDA_library.maegz:161	Cefoperazone	645.670	-7.0078
MOlport_LH1_Approved_FDA_library.maegz:1085	Praziquantel	312.420	-7.0039
MOlport_LH1_Approved_FDA_library.maegz:265	Indinavir sulfate	711.870	-7.0028
MOlport_LH1_Approved_FDA_library.maegz:2011	Balofloxacin	389.420	-7.0024
MOlport_LH1_Approved_FDA_library.maegz:1085	Praziquantel	312.420	-6.9971
MOlport_LH1_Approved_FDA_library.maegz:268	Travoprost	500.550	-6.9962
MOlport_LH1_Approved_FDA_library.maegz:601	Baicalein	270.240	-6.9955
MOlport_LH1_Approved_FDA_library.maegz:1158	Carmofur	257.270	-6.9951
MOlport_LH1_Approved_FDA_library.maegz:863	Empagliflozin	450.910	-6.995
MOlport_LH1_Approved_FDA_library.maegz:1868	Lactitol monohydrate	362.330	-6.9935
MOlport_LH1_Approved_FDA_library.maegz:265	Indinavir sulfate	711.870	-6.9932
MOlport_LH1_Approved_FDA_library.maegz:369	Dolasetron	324.380	-6.9924
MOlport_LH1_Approved_FDA_library.maegz:733	Nafamostat mesylate	539.580	-6.9872
MOlport_LH1_Approved_FDA_library.maegz:1597	Casanthranol	396.580	-6.9825
MOlport_LH1_Approved_FDA_library.maegz:1085	Praziquantel	312.420	-6.967
MOlport_LH1_Approved_FDA_library.maegz:268	Travoprost	500.550	-6.9644
MOlport_LH1_Approved_FDA_library.maegz:17	Ademetionine	398.440	-6.9638
MOlport_LH1_Approved_FDA_library.maegz:1636	Enoxacin	320.320	-6.9637
MOlport_LH1_Approved_FDA_library.maegz:1637	Enoxacin Sesquihydrate	347.340	-6.9637
MOlport_LH1_Approved_FDA_library.maegz:1597	Casanthranol	396.580	-6.9544
MOlport_LH1_Approved_FDA_library.maegz:877	Aminophylline	420.430	-6.9533
MOlport_LH1_Approved_FDA_library.maegz:936	Hydralazine hydrochloride	196.640	-6.9511
MOlport_LH1_Approved_FDA_library.maegz:576	Scopoletin	192.170	-6.9511
MOlport_LH1_Approved_FDA_library.maegz:17	Ademetionine	398.440	-6.9474
MOlport_LH1_Approved_FDA_library.maegz:417	Edoxudine	256.260	-6.9441
MOlport_LH1_Approved_FDA_library.maegz:1865	Triflupromazine hydrochloride	388.880	-6.9422
MOlport_LH1_Approved_FDA_library.maegz:265	Indinavir sulfate	711.870	-6.937
MOlport_LH1_Approved_FDA_library.maegz:821	Ixazomib	361.030	-6.9308
MOlport_LH1_Approved_FDA_library.maegz:379	Trioxsalen	228.250	-6.9268
MOlport_LH1_Approved_FDA_library.maegz:17	Ademetionine	398.440	-6.926
MOlport_LH1_Approved_FDA_library.maegz:1349	Anthralin	226.230	-6.9243
MOlport_LH1_Approved_FDA_library.maegz:1934	Azelastine hydrochloride	418.360	-6.9172
MOlport_LH1_Approved_FDA_library.maegz:1120	4-Methylumbelliferone	176.170	-6.9164
MOlport_LH1_Approved_FDA_library.maegz:213	Vilazodone HCl	477.990	-6.9157
MOlport_LH1_Approved_FDA_library.maegz:1020	Rizatriptan benzoate	391.470	-6.9115
MOlport_LH1_Approved_FDA_library.maegz:1722	Doxofylline	266.260	-6.9113
MOlport_LH1_Approved_FDA_library.maegz:17	Ademetionine	398.440	-6.9039
MOlport_LH1_Approved_FDA_library.maegz:911	Ciprofloxacin	331.340	-6.9024
MOlport_LH1_Approved_FDA_library.maegz:1901	Ciprofloxacin hydrochloride	367.800	-6.9024
MOlport_LH1_Approved_FDA_library.maegz:1322	Polymyxin B sulfate	1301.560	-6.9005
MOlport_LH1_Approved_FDA_library.maegz:269	Topotecan	421.450	-6.8945
MOlport_LH1_Approved_FDA_library.maegz:434	Goserelin acetate	1329.460	-6.8926

MOlport_LH1_Approved_FDA_library.maegz:1804	Bicalutamide	430.370	-6.8882
MOlport_LH1_Approved_FDA_library.maegz:434	Goserelin acetate	1329.460	-6.8873
MOlport_LH1_Approved_FDA_library.maegz:1020	Rizatriptan benzoate	391.470	-6.8841
MOlport_LH1_Approved_FDA_library.maegz:1560	Acetohexamide	324.400	-6.8756
MOlport_LH1_Approved_FDA_library.maegz:256	Nelfinavir Mesylate	663.890	-6.8704
MOlport_LH1_Approved_FDA_library.maegz:315	Desmopressin	1069.220	-6.8682
MOlport_LH1_Approved_FDA_library.maegz:605	Proanthocyanidins	594.520	-6.8617
MOlport_LH1_Approved_FDA_library.maegz:1601	Promazine hydrochloride	320.880	-6.8602
MOlport_LH1_Approved_FDA_library.maegz:837	Dinaciclib	396.490	-6.8567
MOlport_LH1_Approved_FDA_library.maegz:1635	Brivudine	333.140	-6.8506
MOlport_LH1_Approved_FDA_library.maegz:455	Lasmiditan	377.140	-6.8459
MOlport_LH1_Approved_FDA_library.maegz:265	Indinavir sulfate	711.870	-6.8454
MOlport_LH1_Approved_FDA_library.maegz:1951	Pemetrexed disodium	471.370	-6.8442
MOlport_LH1_Approved_FDA_library.maegz:1092	Trifluridine	296.200	-6.8434
MOlport_LH1_Approved_FDA_library.maegz:219	Pemetrexed Disodium Hydrate	516.420	-6.8424
MOlport_LH1_Approved_FDA_library.maegz:1952	Pemetrexed acid	427.410	-6.8423
MOlport_LH1_Approved_FDA_library.maegz:9	Levomefolate calcium	497.520	-6.842
MOlport_LH1_Approved_FDA_library.maegz:265	Indinavir sulfate	711.870	-6.8367
MOlport_LH1_Approved_FDA_library.maegz:48	Riociguat	422.420	-6.8366
MOlport_LH1_Approved_FDA_library.maegz:1159	Norfloxacin	319.340	-6.8327
MOlport_LH1_Approved_FDA_library.maegz:17	Ademetionine	398.440	-6.8295
MOlport_LH1_Approved_FDA_library.maegz:1092	Trifluridine	296.200	-6.8255
MOlport_LH1_Approved_FDA_library.maegz:629	Salvianolic acid B	718.590	-6.8229
MOlport_LH1_Approved_FDA_library.maegz:9	Levomefolate calcium	497.520	-6.8218
MOlport_LH1_Approved_FDA_library.maegz:605	Proanthocyanidins	594.520	-6.8191
MOlport_LH1_Approved_FDA_library.maegz:166	Biapenem	350.390	-6.8141
MOlport_LH1_Approved_FDA_library.maegz:1749	Menadione	172.180	-6.8131
MOlport_LH1_Approved_FDA_library.maegz:1749	Menadione	172.180	-6.8084
MOlport_LH1_Approved_FDA_library.maegz:848	Micafungin sodium	1292.260	-6.8024
MOlport_LH1_Approved_FDA_library.maegz:548	Eprazinone dihydrochloride	453.440	-6.8024
MOlport_LH1_Approved_FDA_library.maegz:605	Proanthocyanidins	594.520	-6.802
MOlport_LH1_Approved_FDA_library.maegz:663	Menadiol Diacetate	258.270	-6.7999
MOlport_LH1_Approved_FDA_library.maegz:1522	Mefloquine hydrochloride	414.770	-6.7869
MOlport_LH1_Approved_FDA_library.maegz:1322	Polymyxin B sulfate	1301.560	-6.7792
MOlport_LH1_Approved_FDA_library.maegz:465	Ivosidenib	582.970	-6.7758
MOlport_LH1_Approved_FDA_library.maegz:1214	Oxolinic acid	261.240	-6.7669
MOlport_LH1_Approved_FDA_library.maegz:58	Netupitant	578.590	-6.7658
MOlport_LH1_Approved_FDA_library.maegz:1746	Minoxidil	209.250	-6.7524
MOlport_LH1_Approved_FDA_library.maegz:1358	Isoprenaline hydrochloride	247.720	-6.7495
MOlport_LH1_Approved_FDA_library.maegz:1018	Norethindrone	298.420	-6.747
MOlport_LH1_Approved_FDA_library.maegz:269	Topotecan	421.450	-6.7463
MOlport_LH1_Approved_FDA_library.maegz:926	Lopinavir	628.800	-6.7424

MOlport_LH1_Approved_FDA_library.maegz:1522	Mefloquine hydrochloride	414.770	-6.7357
MOlport_LH1_Approved_FDA_library.maegz:924	Lomefloxacin hydrochloride	387.810	-6.7269
MOlport_LH1_Approved_FDA_library.maegz:1076	Lomefloxacin	351.350	-6.7269
MOlport_LH1_Approved_FDA_library.maegz:232	Fidaxomicin	1058.040	-6.6947
MOlport_LH1_Approved_FDA_library.maegz:1085	Praziquantel	312.420	-6.6894
MOlport_LH1_Approved_FDA_library.maegz:169	Bepotastine Besilate	547.060	-6.689
MOlport_LH1_Approved_FDA_library.maegz:17	Ademetionine	398.440	-6.6847
MOlport_LH1_Approved_FDA_library.maegz:576	Scopoletin	192.170	-6.684
MOlport_LH1_Approved_FDA_library.maegz:791	Serotonin hydrochloride	212.680	-6.6788
MOlport_LH1_Approved_FDA_library.maegz:1760	L-Tryptophan	204.230	-6.6691
MOlport_LH1_Approved_FDA_library.maegz:443	Pentostatin	268.270	-6.666
MOlport_LH1_Approved_FDA_library.maegz:1036	Gemifloxacin?mesylate	485.490	-6.665
MOlport_LH1_Approved_FDA_library.maegz:17	Ademetionine	398.440	-6.6618
MOlport_LH1_Approved_FDA_library.maegz:404	Stiripentol	234.290	-6.6564
MOlport_LH1_Approved_FDA_library.maegz:1746	Minoxidil	209.250	-6.6525
MOlport_LH1_Approved_FDA_library.maegz:19	Revefenacin	597.750	-6.6439
MOlport_LH1_Approved_FDA_library.maegz:434	Goserelin acetate	1329.460	-6.6438
MOlport_LH1_Approved_FDA_library.maegz:17	Ademetionine	398.440	-6.6343
MOlport_LH1_Approved_FDA_library.maegz:1700	Dermofongin	179.600	-6.6238
MOlport_LH1_Approved_FDA_library.maegz:1174	Synephrine	167.210	-6.6205
MOlport_LH1_Approved_FDA_library.maegz:1064	Candesartan	440.470	-6.6196
MOlport_LH1_Approved_FDA_library.maegz:1174	Synephrine	167.210	-6.6119
MOlport_LH1_Approved_FDA_library.maegz:1795	Alkomide	200.580	-6.5966
MOlport_LH1_Approved_FDA_library.maegz:1754	Promethazine	284.420	-6.5958
MOlport_LH1_Approved_FDA_library.maegz:287	Nestoron	370.480	-6.5675
MOlport_LH1_Approved_FDA_library.maegz:605	Proanthocyanidins	594.520	-6.5585
MOlport_LH1_Approved_FDA_library.maegz:586	Psoralen	186.170	-6.5541
MOlport_LH1_Approved_FDA_library.maegz:1036	Gemifloxacin?mesylate	485.490	-6.5529
MOlport_LH1_Approved_FDA_library.maegz:643	Thymopentin	679.560	-6.5339
MOlport_LH1_Approved_FDA_library.maegz:924	Lomefloxacin hydrochloride	387.810	-6.5303
MOlport_LH1_Approved_FDA_library.maegz:1076	Lomefloxacin	351.350	-6.5303
MOlport_LH1_Approved_FDA_library.maegz:1795	Alkomide	200.580	-6.5295
MOlport_LH1_Approved_FDA_library.maegz:185	Sitafloxacin Hydrate	436.840	-6.4812
MOlport_LH1_Approved_FDA_library.maegz:1597	Casanthranol	396.580	-6.4771
MOlport_LH1_Approved_FDA_library.maegz:1059	Nadifloxacin	360.390	-6.4717
MOlport_LH1_Approved_FDA_library.maegz:598	Nicotine	162.230	-6.4544
MOlport_LH1_Approved_FDA_library.maegz:1159	Norfloxacin	319.340	-6.4506
MOlport_LH1_Approved_FDA_library.maegz:307	Olprinone	250.260	-6.4439
MOlport_LH1_Approved_FDA_library.maegz:306	Nicotine Ditartrate	462.410	-6.44
MOlport_LH1_Approved_FDA_library.maegz:1636	Enoxacin	320.320	-6.425
MOlport_LH1_Approved_FDA_library.maegz:1637	Enoxacin Sesquihydrate	347.340	-6.4244
MOlport_LH1_Approved_FDA_library.maegz:1059	Nadifloxacin	360.390	-6.4223
MOlport_LH1_Approved_FDA_library.maegz:39	Norepinephrine	169.180	-6.4138

MOlport_LH1_Approved_FDA_library.maegz:1513	Noradrenaline bitartrate	319.260	-6.4138
MOlport_LH1_Approved_FDA_library.maegz:1895	Mepiroxol	125.130	-6.4085
MOlport_LH1_Approved_FDA_library.maegz:1414	Methoxamine hydrochloride	247.720	-6.4041
MOlport_LH1_Approved_FDA_library.maegz:219	Pemetrexed Disodium Hydrate	516.420	-6.3994
MOlport_LH1_Approved_FDA_library.maegz:877	Aminophylline	420.430	-6.3976
MOlport_LH1_Approved_FDA_library.maegz:643	Thymopentin	679.560	-6.3974
MOlport_LH1_Approved_FDA_library.maegz:1059	Nadifloxacin	360.390	-6.3966
MOlport_LH1_Approved_FDA_library.maegz:1952	Pemetrexed acid	427.410	-6.3885
MOlport_LH1_Approved_FDA_library.maegz:1951	Pemetrexed disodium	471.370	-6.3871
MOlport_LH1_Approved_FDA_library.maegz:791	Serotonin hydrochloride	212.680	-6.3857
MOlport_LH1_Approved_FDA_library.maegz:1059	Nadifloxacin	360.390	-6.3597
MOlport_LH1_Approved_FDA_library.maegz:1045	Ciclopirox	207.270	-6.3481
MOlport_LH1_Approved_FDA_library.maegz:1310	Ciclopirox ethanolamine	268.350	-6.3481
MOlport_LH1_Approved_FDA_library.maegz:38	Dronedarone	556.760	-6.3413
MOlport_LH1_Approved_FDA_library.maegz:1344	Dronedarone hydrochloride	593.220	-6.3404
MOlport_LH1_Approved_FDA_library.maegz:1530	Carbazochrome sodium sulfonate	376.320	-6.3385
MOlport_LH1_Approved_FDA_library.maegz:232	Fidaxomicin	1058.040	-6.338
MOlport_LH1_Approved_FDA_library.maegz:907	Dopamine hydrochloride	189.640	-6.3254
MOlport_LH1_Approved_FDA_library.maegz:1263	Diclazuril	407.640	-6.3091
MOlport_LH1_Approved_FDA_library.maegz:643	Thymopentin	679.560	-6.3077
MOlport_LH1_Approved_FDA_library.maegz:1322	Polymyxin B sulfate	1301.560	-6.2995
MOlport_LH1_Approved_FDA_library.maegz:1895	Mepiroxol	125.130	-6.2983
MOlport_LH1_Approved_FDA_library.maegz:1059	Nadifloxacin	360.390	-6.2885
MOlport_LH1_Approved_FDA_library.maegz:219	Pemetrexed Disodium Hydrate	516.420	-6.2785
MOlport_LH1_Approved_FDA_library.maegz:1569	Diflunisal	250.200	-6.2763
MOlport_LH1_Approved_FDA_library.maegz:1951	Pemetrexed disodium	471.370	-6.2749
MOlport_LH1_Approved_FDA_library.maegz:1952	Pemetrexed acid	427.410	-6.2745
MOlport_LH1_Approved_FDA_library.maegz:516	Cefminox Sodium	563.530	-6.2607
MOlport_LH1_Approved_FDA_library.maegz:1322	Polymyxin B sulfate	1301.560	-6.248
MOlport_LH1_Approved_FDA_library.maegz:1647	Triflusal	248.160	-6.2471
MOlport_LH1_Approved_FDA_library.maegz:1322	Polymyxin B sulfate	1301.560	-6.2268
MOlport_LH1_Approved_FDA_library.maegz:66	Fenoldopam mesylate	401.860	-6.2247
MOlport_LH1_Approved_FDA_library.maegz:1263	Diclazuril	407.640	-6.2106
MOlport_LH1_Approved_FDA_library.maegz:1064	Candesartan	440.470	-6.146
MOlport_LH1_Approved_FDA_library.maegz:926	Lopinavir	628.800	-6.1405
MOlport_LH1_Approved_FDA_library.maegz:1303	Cephalothin sodium	418.400	-6.058
MOlport_LH1_Approved_FDA_library.maegz:1647	Triflusal	248.160	-6.051
MOlport_LH1_Approved_FDA_library.maegz:309	Elagolix sodium	653.570	-6.037
MOlport_LH1_Approved_FDA_library.maegz:1949	Levetiracetam	170.210	-5.9829
MOlport_LH1_Approved_FDA_library.maegz:1132	Cefalexine EP IMpurity B	214.240	-5.6941

MOlport_LH1_Approved_FDA_library.maegz:166	Biapenem	350.390	-5.6757

Table S13. VS1 and VS2 for DSI-poised targeting UDG

Compound	Catalog ID	MW	Energy in silico
VS1			
DSi PoisedLibrary_1680186639697.774	Z32279730	200.116	-4.4729
VS2			
DSi PoisedLibrary_1680186639697.377	Z165170770	214.041	-6.112
DSi PoisedLibrary_1680186639697.858	Z27682767	228.057	-5.9304
DSi PoisedLibrary_1680186639697.741	Z1295863442	189.026	-5.8939
DSi PoisedLibrary_1680186639697.726	Z123856654	243.068	-5.8798
DSi PoisedLibrary_1680186639697.700	Z18197050	215.025	-5.8493
DSi PoisedLibrary_1680186639697.726	Z123856654	243.068	-5.837
DSi PoisedLibrary_1680186639697.774	Z32279730	200.116	-5.7475
DSi PoisedLibrary_1680186639697.726	Z123856654	243.068	-5.739
DSi PoisedLibrary_1680186639697.663	Z30932204	228.057	-5.7194
DSi PoisedLibrary_1680186639697.605	Z1696091761	198.056	-5.7099
DSi PoisedLibrary_1680186639697.812	Z57627041	195.101	-5.6761
DSi PoisedLibrary_1680186639697.236	Z1333043510	226.078	-5.6609
DSi PoisedLibrary_1680186639697.639	Z979770898	159.090	-5.6255
DSi PoisedLibrary_1680186639697.401	Z57101343	225.249	-5.596
DSi PoisedLibrary_1680186639697.777	Z352356442	267.127	-5.5618
DSi PoisedLibrary_1680186639697.59	Z198194394	223.112	-5.5487
DSi PoisedLibrary_1680186639697.777	Z352356442	267.127	-5.5454
DSi PoisedLibrary_1680186639697.663	Z30932204	228.057	-5.5343
DSi PoisedLibrary_1680186639697.817	Z1250093582	173.167	-5.5264
DSi PoisedLibrary_1680186639697.848	Z359331368	230.036	-5.519
DSi PoisedLibrary_1680186639697.377	Z165170770	214.041	-5.5159
DSi PoisedLibrary_1680186639697.63	Z1004825200	299.729	-5.5088
DSi PoisedLibrary_1680186639697.35	Z952656810	152.095	-5.4966
DSi PoisedLibrary_1680186639697.741	Z1295863442	189.026	-5.4441
DSi PoisedLibrary_1680186639697.341	Z982117526	244.077	-5.4221
DSi PoisedLibrary_1680186639697.806	Z1262633486	175.056	-5.4085
DSi PoisedLibrary_1680186639697.341	Z982117526	244.077	-5.3888
DSi PoisedLibrary_1680186639697.85	Z1443544073	189.057	-5.3752
DSi PoisedLibrary_1680186639697.696	Z1255402624	166.099	-5.3582
DSi PoisedLibrary_1680186639697.663	Z30932204	228.057	-5.3531
DSi PoisedLibrary_1680186639697.452	Z982117894	209.036	-5.3529
DSi PoisedLibrary_1680186639697.528	Z239095910	199.168	-5.3525
DSi PoisedLibrary_1680186639697.754	Z1324853681	165.090	-5.3447

DSi PoisedLibrary_1680186639697.11	Z19727416	199.040	-5.3409
DSi PoisedLibrary_1680186639697.59	Z198194394	223.112	-5.3255
DSi PoisedLibrary_1680186639697.59	Z198194394	223.112	-5.3229
DSi PoisedLibrary_1680186639697.812	Z57627041	195.101	-5.292
DSi PoisedLibrary_1680186639697.31	Z45656995	214.041	-5.2917
DSi PoisedLibrary_1680186639697.812	Z57627041	195.101	-5.2892
DSi PoisedLibrary_1680186639697.639	Z979770898	159.090	-5.2881
DSi PoisedLibrary_1680186639697.269	Z2072621991	219.228	-5.2861
DSi PoisedLibrary_1680186639697.261	Z32399802	170.106	-5.2819
DSi PoisedLibrary_1680186639697.777	Z352356442	267.127	-5.2804
DSi PoisedLibrary_1680186639697.269	Z2072621991	219.228	-5.2756
DSi PoisedLibrary_1680186639697.31	Z45656995	214.041	-5.2633
DSi PoisedLibrary_1680186639697.433	Z1741959530	197.105	-5.2632
DSi PoisedLibrary_1680186639697.582	Z2092555279	210.248	-5.2622
DSi PoisedLibrary_1680186639697.736	Z1222424326	191.031	-5.26
DSi PoisedLibrary_1680186639697.639	Z979770898	159.090	-5.2581
DSi PoisedLibrary_1680186639697.399	Z32399948	238.326	-5.2453
DSi PoisedLibrary_1680186639697.217	Z220816104	170.106	-5.2298
DSi PoisedLibrary_1680186639697.68	Z26251905	203.106	-5.2232
DSi PoisedLibrary_1680186639697.68	Z26251905	203.106	-5.222
DSi PoisedLibrary_1680186639697.601	Z1020823294	205.041	-5.2173
DSi PoisedLibrary_1680186639697.76	Z1639162606	203.042	-5.2168
DSi PoisedLibrary_1680186639697.736	Z1222424326	191.031	-5.2153
DSi PoisedLibrary_1680186639697.858	Z27682767	228.057	-5.2107
DSi PoisedLibrary_1680186639697.63	Z1004825200	299.729	-5.2064
DSi PoisedLibrary_1680186639697.617	Z432057220	139.075	-5.1959
DSi PoisedLibrary_1680186639697.802	Z425757818	179.062	-5.1949
DSi PoisedLibrary_1680186639697.713	Z57102475	167.095	-5.1943
DSi PoisedLibrary_1680186639697.714	Z53860899	243.057	-5.1909
DSi PoisedLibrary_1680186639697.234	Z119990900	210.046	-5.1889
DSi PoisedLibrary_1680186639697.403	Z28870646	189.115	-5.1816
DSi PoisedLibrary_1680186639697.651	Z1270393711	156.053	-5.1744
DSi PoisedLibrary_1680186639697.754	Z1324853681	165.090	-5.1682
DSi PoisedLibrary_1680186639697.760	Z608065044	175.042	-5.153
DSi PoisedLibrary_1680186639697.840	Z2074076908	219.228	-5.1488
DSi PoisedLibrary_1680186639697.302	Z190780124	206.241	-5.141
DSi PoisedLibrary_1680186639697.399	Z32399948	238.326	-5.1386
DSi PoisedLibrary_1680186639697.503	Z223388508	242.316	-5.1375
DSi PoisedLibrary_1680186639697.639	Z979770898	159.090	-5.1336
DSi PoisedLibrary_1680186639697.431	Z1401276297	205.256	-5.12
DSi PoisedLibrary_1680186639697.31	Z45656995	214.041	-5.1195
DSi PoisedLibrary_1680186639697.791	Z33546965	208.085	-5.108
DSi PoisedLibrary_1680186639697.817	Z1250093582	173.167	-5.106

DSi PoisedLibrary_1680186639697.759	Z45527714	204.996	-5.103
DSi PoisedLibrary_1680186639697.635	Z90664455	142.111	-5.102
DSi PoisedLibrary_1680186639697.449	Z56813508	166.111	-5.0986
DSi PoisedLibrary_1680186639697.804	Z1491215378	214.054	-5.0975
DSi PoisedLibrary_1680186639697.466	Z33486130	221.080	-5.0963
DSi PoisedLibrary_1680186639697.605	Z1696091761	198.056	-5.0923
DSi PoisedLibrary_1680186639697.803	Z383202616	175.075	-5.0891
DSi PoisedLibrary_1680186639697.806	Z1262633486	175.056	-5.0891
DSi PoisedLibrary_1680186639697.560	Z133716556	227.062	-5.0884
DSi PoisedLibrary_1680186639697.217	Z220816104	170.106	-5.0853
DSi PoisedLibrary_1680186639697.840	Z2074076908	219.228	-5.0648
DSi PoisedLibrary_1680186639697.378	Z332382842	206.142	-5.0502
DSi PoisedLibrary_1680186639697.425	Z401437758	203.069	-5.0386
DSi PoisedLibrary_1680186639697.446	Z1147230594	201.100	-5.0375
DSi PoisedLibrary_1680186639697.804	Z1491215378	214.054	-5.0304
DSi PoisedLibrary_1680186639697.468	Z2049876225	187.084	-5.0265
DSi PoisedLibrary_1680186639697.269	Z2072621991	219.228	-5.0258
DSi PoisedLibrary_1680186639697.64	Z287484230	167.106	-5.0228
DSi PoisedLibrary_1680186639697.217	Z220816104	170.106	-5.0208
DSi PoisedLibrary_1680186639697.209	Z270979606	163.100	-5.0207
DSi PoisedLibrary_1680186639697.803	Z383202616	175.075	-5.0139
DSi PoisedLibrary_1680186639697.260	Z90122368	171.101	-5.0052
DSi PoisedLibrary_1680186639697.256	Z166645986	142.111	-5.0004
DSi PoisedLibrary_1680186639697.535	Z2153384824	181.085	-4.9923
DSi PoisedLibrary_1680186639697.817	Z1250093582	173.167	-4.9911
DSi PoisedLibrary_1680186639697.433	Z1741959530	197.105	-4.9905
DSi PoisedLibrary_1680186639697.769	Z1509441355	219.137	-4.9896
DSi PoisedLibrary_1680186639697.713	Z57102475	167.095	-4.9709
DSi PoisedLibrary_1680186639697.827	Z1758466556	212.144	-4.9681
DSi PoisedLibrary_1680186639697.602	Z1198169663	153.059	-4.9637
DSi PoisedLibrary_1680186639697.217	Z220816104	170.106	-4.9615
DSi PoisedLibrary_1680186639697.446	Z1147230594	201.100	-4.9546
DSi PoisedLibrary_1680186639697.800	Z133632670	207.057	-4.9461
DSi PoisedLibrary_1680186639697.160	Z385440544	171.090	-4.9452
DSi PoisedLibrary_1680186639697.468	Z2049876225	187.084	-4.9306
DSi PoisedLibrary_1680186639697.261	Z32399802	170.106	-4.9236
DSi PoisedLibrary_1680186639697.85	Z1443544073	189.057	-4.9175
DSi PoisedLibrary_1680186639697.804	Z1491215378	214.054	-4.9139
DSi PoisedLibrary_1680186639697.31	Z45656995	214.041	-4.9077
DSi PoisedLibrary_1680186639697.562	Z1198316457	163.081	-4.9004
DSi PoisedLibrary_1680186639697.617	Z432057220	139.075	-4.9001
DSi PoisedLibrary_1680186639697.254	Z1618027593	175.075	-4.8871
DSi PoisedLibrary_1680186639697.217	Z220816104	170.106	-4.8845

DSi PoisedLibrary_1680186639697.635	Z90664455	142.111	-4.8673
DSi PoisedLibrary_1680186639697.160	Z385440544	171.090	-4.8644
DSi PoisedLibrary_1680186639697.28	Z85893853	265.111	-4.8585
DSi PoisedLibrary_1680186639697.234	Z119990900	210.046	-4.8379
DSi PoisedLibrary_1680186639697.146	Z68639747	203.106	-4.8305
DSi PoisedLibrary_1680186639697.31	Z45656995	214.041	-4.8267
DSi PoisedLibrary_1680186639697.256	Z166645986	142.111	-4.8253
DSi PoisedLibrary_1680186639697.446	Z1147230594	201.100	-4.8206
DSi PoisedLibrary_1680186639697.773	Z193091370	192.057	-4.8144
DSi PoisedLibrary_1680186639697.446	Z1147230594	201.100	-4.8068
DSi PoisedLibrary_1680186639697.677	Z56978034	170.038	-4.8047
DSi PoisedLibrary_1680186639697.217	Z220816104	170.106	-4.7467
DSi PoisedLibrary_1680186639697.446	Z1147230594	201.100	-4.7337
DSi PoisedLibrary_1680186639697.54	Z751811134	186.046	-4.7235
DSi PoisedLibrary_1680186639697.493	Z1486018170	203.999	-4.7217
DSi PoisedLibrary_1680186639697.468	Z2049876225	187.084	-4.7055
DSi PoisedLibrary_1680186639697.137	Z285141302	190.111	-4.6816
DSi PoisedLibrary_1680186639697.746	Z1171217431	143.058	-4.6736
DSi PoisedLibrary_1680186639697.260	Z90122368	171.101	-4.6721
DSi PoisedLibrary_1680186639697.535	Z2153384824	181.085	-4.6343
DSi PoisedLibrary_1680186639697.535	Z2153384824	181.085	-4.627
DSi PoisedLibrary_1680186639697.468	Z2049876225	187.084	-4.6048
DSi PoisedLibrary_1680186639697.443	Z65532537	184.054	-4.602
DSi PoisedLibrary_1680186639697.160	Z385440544	171.090	-4.5882
DSi PoisedLibrary_1680186639697.580	Z1198317053	191.069	-4.581
DSi PoisedLibrary_1680186639697.468	Z2049876225	187.084	-4.5651
DSi PoisedLibrary_1680186639697.800	Z133632670	207.057	-4.5648
DSi PoisedLibrary_1680186639697.94	Z133622412	221.072	-4.5448
DSi PoisedLibrary_1680186639697.189	Z1230013388	196.085	-4.5431
DSi PoisedLibrary_1680186639697.746	Z1171217431	143.058	-4.4845
DSi PoisedLibrary_1680186639697.800	Z133632670	207.057	-4.4234
DSi PoisedLibrary_1680186639697.160	Z385440544	171.090	-4.4054
DSi PoisedLibrary_1680186639697.580	Z1198317053	191.069	-4.3815
DSi PoisedLibrary_1680186639697.243	Z951183688	157.074	-4.359
DSi PoisedLibrary_1680186639697.580	Z1198317053	191.069	-4.3288
DSi PoisedLibrary_1680186639697.746	Z1171217431	143.058	-4.3238

Table S14. VS1 and VS2 for PDB fragment library targeting UDG

Compound	WT	Energy in silico
VS1		
octahydroindolizine-1,6,7,8-tetrol	189.100	-6.6525
VS2		

octahydroindolizine-1,6,7,8-tetrol	189.100	-6.4978
octahydroindolizine-1,6,7,8-tetrol	189.100	-6.1754
Arabinosylcytosine		-6.0948
3,4,5-trihydroxycyclohex-2-ene-1-carboxylic acid	174.053	-6.0256
6-Hydroxy-2-benzothiazolesulfonamide	229.982	-5.9949
3,4,5-trihydroxycyclohex-2-ene-1-carboxylic acid	174.053	-5.9398
3,4,5-trihydroxycyclohex-2-ene-1-carboxylic acid	174.053	-5.9377
octahydroindolizine-1,6,7,8-tetrol	189.100	-5.8966
octahydroindolizine-1,6,7,8-tetrol	189.100	-5.8803
3,4,5-trihydroxycyclohex-2-ene-1-carboxylic acid	174.053	-5.8779
Umbelliferone	162.032	-5.8768
octahydroindolizine-1,6,7,8-tetrol	189.100	-5.8447
Phloroglucinol	126.032	-5.8374
Phloroglucinol	126.032	-5.8367
Phloroglucinol	126.032	-5.8321
Acetazolamide	221.988	-5.8096
4'-Hydroxyflavanone	240.079	-5.7592
octahydroindolizine-1,6,7,8-tetrol	189.100	-5.7528
4'-Hydroxyflavanone	240.079	-5.7526
tyrosinamide	180.090	-5.7051
Piracetam	142.074	-5.6469
octahydroindolizine-1,6,7,8-tetrol	189.100	-5.6394
3,4,5-trihydroxycyclohex-2-ene-1-carboxylic acid	174.053	-5.6333
L-tryptophanamide	203.106	-5.6249
octahydroindolizine-1,6,7,8-tetrol	189.100	-5.618
6-Hydroxy-2-benzothiazolesulfonamide	229.982	-5.6031
3,4,5-trihydroxycyclohex-2-ene-1-carboxylic acid	174.053	-5.5959
L-tryptophanamide	203.106	-5.516
Resorcinol	110.037	-5.4351
3,4,5-trihydroxycyclohex-2-ene-1-carboxylic acid	174.053	-5.434
Tranexamic Acid	157.110	-5.3845
5-Hydroxyindole-3-acetic acid	191.058	-5.3703
3,4,5-trihydroxycyclohex-2-ene-1-carboxylic acid	174.053	-5.3656
2-Methyl-4-carboxy-5-hydroxy-3,4,5,6-tetrahydropyrimidine	158.069	-5.3216
3,4-Diaminobenzotrifluoride	176.056	-5.292
Piracetam	142.074	-5.2447
3,4,5-trihydroxycyclohex-2-ene-1-carboxylic acid	174.053	-5.2357
3,4,5-trihydroxycyclohex-2-ene-1-carboxylic acid	174.053	-5.1874
Tranexamic Acid	157.110	-5.1678
6-methyltetrahydropterin	181.096	-5.1242
6-methyltetrahydropterin	181.096	-5.08
Tranexamic Acid	157.110	-5.0627
Procodazole	190.074	-4.9897

2-Succinylbenzoate	222.053	-4.6214
2-Succinylbenzoate	222.053	-4.2913
N-Acetyl-L-glutamic acid	189.064	-4.1653

Table S15. VS1 and VS2 for LH1 FDA approved drugs targeting UDG

Compound	Drug Name	MolWt	Energy In silico
VS1			
MOlport_LH1_Approved_FDA_library.maegz:605-16	Proanthocyanidins	594.520	-9.3468
VS2			
MOlport_LH1_Approved_FDA_library.maegz:665-2	Felypressin	1040.220	-7.1877
MOlport_LH1_Approved_FDA_library.maegz:434-5	Goserelin acetate	1329.460	-7.0795
MOlport_LH1_Approved_FDA_library.maegz:1580-1	Rutin	610.520	-7.0535
MOlport_LH1_Approved_FDA_library.maegz:1596-1	Troxerutin	742.670	-7.0304
MOlport_LH1_Approved_FDA_library.maegz:629-2	Salvianolic acid B	718.590	-6.9819
MOlport_LH1_Approved_FDA_library.maegz:1684-1	Naringin	580.530	-6.9556
MOlport_LH1_Approved_FDA_library.maegz:1684-1	Naringin	580.530	-6.9375
MOlport_LH1_Approved_FDA_library.maegz:665-2	Felypressin	1040.220	-6.9257
MOlport_LH1_Approved_FDA_library.maegz:1596-1	Troxerutin	742.670	-6.9228
MOlport_LH1_Approved_FDA_library.maegz:629-2	Salvianolic acid B	718.590	-6.9119
MOlport_LH1_Approved_FDA_library.maegz:1596-1	Troxerutin	742.670	-6.904
MOlport_LH1_Approved_FDA_library.maegz:245-3	Anidulafungin	1140.240	-6.8418
MOlport_LH1_Approved_FDA_library.maegz:361-2	Pentagastrin	767.890	-6.8369
MOlport_LH1_Approved_FDA_library.maegz:245-3	Anidulafungin	1140.240	-6.802
MOlport_LH1_Approved_FDA_library.maegz:629-2	Salvianolic acid B	718.590	-6.7963
MOlport_LH1_Approved_FDA_library.maegz:1580-1	Rutin	610.520	-6.7643
MOlport_LH1_Approved_FDA_library.maegz:1580-1	Rutin	610.520	-6.7196
MOlport_LH1_Approved_FDA_library.maegz:605-16	Proanthocyanidins	594.520	-6.7171
MOlport_LH1_Approved_FDA_library.maegz:1931-3	Pioglitazone	356.440	-6.6238
MOlport_LH1_Approved_FDA_library.maegz:837-1	Dinaciclib	396.490	-6.6079
MOlport_LH1_Approved_FDA_library.maegz:1868-1	Lactitol monohydrate	362.330	-6.6044
MOlport_LH1_Approved_FDA_library.maegz:268-14	Travoprost	500.550	-6.6014
MOlport_LH1_Approved_FDA_library.maegz:335-3	Ertapenem sodium	497.502	-6.5666
MOlport_LH1_Approved_FDA_library.maegz:268-16	Travoprost	500.550	-6.5636
MOlport_LH1_Approved_FDA_library.maegz:605-23	Proanthocyanidins	594.520	-6.5314
MOlport_LH1_Approved_FDA_library.maegz:192-1	Lonafarnib	638.820	-6.4909
MOlport_LH1_Approved_FDA_library.maegz:1931-3	Pioglitazone	356.440	-6.4873
MOlport_LH1_Approved_FDA_library.maegz:605-10	Proanthocyanidins	594.520	-6.485
MOlport_LH1_Approved_FDA_library.maegz:605-14	Proanthocyanidins	594.520	-6.4535
MOlport_LH1_Approved_FDA_library.maegz:268-22	Travoprost	500.550	-6.4392
MOlport_LH1_Approved_FDA_library.maegz:605-32	Proanthocyanidins	594.520	-6.4387
MOlport_LH1_Approved_FDA_library.maegz:434-5	Goserelin acetate	1329.460	-6.4351

MOlport_LH1_Approved_FDA_library.maegz:638-1	Regadenoson	390.350	-6.4335
MOlport_LH1_Approved_FDA_library.maegz:268-14	Travoprost	500.550	-6.4312
MOlport_LH1_Approved_FDA_library.maegz:605-10	Proanthocyanidins	594.520	-6.4255
MOlport_LH1_Approved_FDA_library.maegz:1597-3	Casanthranol	396.580	-6.4212
MOlport_LH1_Approved_FDA_library.maegz:268-20	Travoprost	500.550	-6.4031
MOlport_LH1_Approved_FDA_library.maegz:848-4	Micafungin sodium	1292.260	-6.3967
MOlport_LH1_Approved_FDA_library.maegz:605-19	Proanthocyanidins	594.520	-6.3462
MOlport_LH1_Approved_FDA_library.maegz:847-1	Carfilzomib	719.910	-6.3274
MOlport_LH1_Approved_FDA_library.maegz:192-1	Lonafarnib	638.820	-6.3203
MOlport_LH1_Approved_FDA_library.maegz:605-16	Proanthocyanidins	594.520	-6.3161
MOlport_LH1_Approved_FDA_library.maegz:1597-23	Casanthranol	396.580	-6.3113
MOlport_LH1_Approved_FDA_library.maegz:268-9	Travoprost	500.550	-6.3108
MOlport_LH1_Approved_FDA_library.maegz:268-7	Travoprost	500.550	-6.2907
MOlport_LH1_Approved_FDA_library.maegz:605-14	Proanthocyanidins	594.520	-6.2902
MOlport_LH1_Approved_FDA_library.maegz:1439-3	Sennoside A	862.740	-6.2862
MOlport_LH1_Approved_FDA_library.maegz:1597-23	Casanthranol	396.580	-6.2828
MOlport_LH1_Approved_FDA_library.maegz:92-4	Silymarin	482.440	-6.2808
MOlport_LH1_Approved_FDA_library.maegz:1506-1	Diosmin	608.540	-6.2767
MOlport_LH1_Approved_FDA_library.maegz:1377-4	Doripenem Hydrate	438.520	-6.2724
MOlport_LH1_Approved_FDA_library.maegz:335-3	Ertapenem sodium	497.502	-6.2711
MOlport_LH1_Approved_FDA_library.maegz:208-2	Trelagliptin	357.380	-6.2704
MOlport_LH1_Approved_FDA_library.maegz:774-2	Trelagliptin succinate	475.470	-6.2683
MOlport_LH1_Approved_FDA_library.maegz:1060-4	Mupirocin	500.640	-6.2675
MOlport_LH1_Approved_FDA_library.maegz:268-22	Travoprost	500.550	-6.2591
MOlport_LH1_Approved_FDA_library.maegz:268-14	Travoprost	500.550	-6.2585
MOlport_LH1_Approved_FDA_library.maegz:605-27	Proanthocyanidins	594.520	-6.2324
MOlport_LH1_Approved_FDA_library.maegz:9-41	Levomefolate calcium	497.520	-6.2296
MOlport_LH1_Approved_FDA_library.maegz:926-1	Lopinavir	628.800	-6.2286
MOlport_LH1_Approved_FDA_library.maegz:1439-3	Sennoside A	862.740	-6.2284
MOlport_LH1_Approved_FDA_library.maegz:926-1	Lopinavir	628.800	-6.2222
MOlport_LH1_Approved_FDA_library.maegz:1377-4	Doripenem Hydrate	438.520	-6.2217
MOlport_LH1_Approved_FDA_library.maegz:268-22	Travoprost	500.550	-6.2172
MOlport_LH1_Approved_FDA_library.maegz:605-18	Proanthocyanidins	594.520	-6.2105
MOlport_LH1_Approved_FDA_library.maegz:605-21	Proanthocyanidins	594.520	-6.2087
MOlport_LH1_Approved_FDA_library.maegz:848-8	Micafungin sodium	1292.260	-6.2069
MOlport_LH1_Approved_FDA_library.maegz:593-1	Fraxetin	208.170	-6.202
MOlport_LH1_Approved_FDA_library.maegz:1377-3	Doripenem Hydrate	438.520	-6.2015
MOlport_LH1_Approved_FDA_library.maegz:605-1	Proanthocyanidins	594.520	-6.1786
MOlport_LH1_Approved_FDA_library.maegz:1432-1	Novobiocin Sodium	634.610	-6.1739
MOlport_LH1_Approved_FDA_library.maegz:1895-1	Mepiroxol	125.130	-6.1703
MOlport_LH1_Approved_FDA_library.maegz:605-32	Proanthocyanidins	594.520	-6.1682
MOlport_LH1_Approved_FDA_library.maegz:1299-2	Oxiracetam	158.160	-6.1622
MOlport_LH1_Approved_FDA_library.maegz:245-8	Anidulafungin	1140.240	-6.1592

MOlport_LH1_Approved_FDA_library.maegz:295-2	Glecaprevir	838.870	-6.1551
MOlport_LH1_Approved_FDA_library.maegz:774-2	Trelagliptin succinate	475.470	-6.1434
MOlport_LH1_Approved_FDA_library.maegz:208-2	Trelagliptin	357.380	-6.1428
MOlport_LH1_Approved_FDA_library.maegz:404-2	Stiripentol	234.290	-6.1405
MOlport_LH1_Approved_FDA_library.maegz:605-32	Proanthocyanidins	594.520	-6.1295
MOlport_LH1_Approved_FDA_library.maegz:1377-3	Doripenem Hydrate	438.520	-6.1286
MOlport_LH1_Approved_FDA_library.maegz:643-5	Thymopentin	679.560	-6.1255
MOlport_LH1_Approved_FDA_library.maegz:265-59	Indinavir sulfate	711.870	-6.1209
MOlport_LH1_Approved_FDA_library.maegz:452-1	Jaceosidin	330.290	-6.1149
MOlport_LH1_Approved_FDA_library.maegz:1377-4	Doripenem Hydrate	438.520	-6.1145
MOlport_LH1_Approved_FDA_library.maegz:245-3	Anidulafungin	1140.240	-6.1106
MOlport_LH1_Approved_FDA_library.maegz:865-1	Torezolid	370.340	-6.1087
MOlport_LH1_Approved_FDA_library.maegz:605-18	Proanthocyanidins	594.520	-6.1053
MOlport_LH1_Approved_FDA_library.maegz:1439-3	Sennoside A	862.740	-6.0986
MOlport_LH1_Approved_FDA_library.maegz:605-20	Proanthocyanidins	594.520	-6.0962
MOlport_LH1_Approved_FDA_library.maegz:848-7	Micafungin sodium	1292.260	-6.0946
MOlport_LH1_Approved_FDA_library.maegz:245-8	Anidulafungin	1140.240	-6.0943
MOlport_LH1_Approved_FDA_library.maegz:1597-23	Casanthranol	396.580	-6.0775
MOlport_LH1_Approved_FDA_library.maegz:605-14	Proanthocyanidins	594.520	-6.0516
MOlport_LH1_Approved_FDA_library.maegz:268-21	Travoprost	500.550	-6.0476
MOlport_LH1_Approved_FDA_library.maegz:268-17	Travoprost	500.550	-6.0461
MOlport_LH1_Approved_FDA_library.maegz:1895-1	Mepiroxol	125.130	-6.0428
MOlport_LH1_Approved_FDA_library.maegz:1681-1	Piceatannol	244.240	-6.0406
MOlport_LH1_Approved_FDA_library.maegz:452-1	Jaceosidin	330.290	-6.0374
MOlport_LH1_Approved_FDA_library.maegz:605-6	Proanthocyanidins	594.520	-6.0358
MOlport_LH1_Approved_FDA_library.maegz:848-7	Micafungin sodium	1292.260	-6.0329
MOlport_LH1_Approved_FDA_library.maegz:1681-1	Piceatannol	244.240	-6.0234
MOlport_LH1_Approved_FDA_library.maegz:605-3	Proanthocyanidins	594.520	-6.021
MOlport_LH1_Approved_FDA_library.maegz:1009-1	Ritonavir	720.960	-6.013
MOlport_LH1_Approved_FDA_library.maegz:802-1	2-Aminobenzenesulfonamide	172.210	-6.0077
MOlport_LH1_Approved_FDA_library.maegz:1120-1	4-Methylumbelliferone	176.170	-6.0072
MOlport_LH1_Approved_FDA_library.maegz:865-1	Torezolid	370.340	-6.0062
MOlport_LH1_Approved_FDA_library.maegz:605-24	Proanthocyanidins	594.520	-6.0034
MOlport_LH1_Approved_FDA_library.maegz:1032-2	Indapamide	365.840	-5.9942
MOlport_LH1_Approved_FDA_library.maegz:847-1	Carfilzomib	719.910	-5.9931
MOlport_LH1_Approved_FDA_library.maegz:2004-1	Sulfanilamide	172.200	-5.9766
MOlport_LH1_Approved_FDA_library.maegz:605-13	Proanthocyanidins	594.520	-5.9689
MOlport_LH1_Approved_FDA_library.maegz:1895-1	Mepiroxol	125.130	-5.9379
MOlport_LH1_Approved_FDA_library.maegz:1597-3	Casanthranol	396.580	-5.9376
MOlport_LH1_Approved_FDA_library.maegz:1322-15	Polymyxin B sulfate	1301.560	-5.933
MOlport_LH1_Approved_FDA_library.maegz:452-1	Jaceosidin	330.290	-5.9285
MOlport_LH1_Approved_FDA_library.maegz:576-1	Scopoletin	192.170	-5.9275

MOlport_LH1_Approved_FDA_library.maegz:603-1	Daphnetin	178.140	-5.9247
MOlport_LH1_Approved_FDA_library.maegz:812-2	Fulvestrant	606.770	-5.9211
MOlport_LH1_Approved_FDA_library.maegz:2004-1	Sulfanilamide	172.200	-5.9166
MOlport_LH1_Approved_FDA_library.maegz:605-24	Proanthocyanidins	594.520	-5.9123
MOlport_LH1_Approved_FDA_library.maegz:1751-3	Carvedilol	406.470	-5.9055
MOlport_LH1_Approved_FDA_library.maegz:256-2	Nelfinavir Mesylate	663.890	-5.9048
MOlport_LH1_Approved_FDA_library.maegz:1839-3	Carvedilol phosphate	522.490	-5.904
MOlport_LH1_Approved_FDA_library.maegz:1439-4	Sennoside A	862.740	-5.9021
MOlport_LH1_Approved_FDA_library.maegz:1775-1	Inositol	180.160	-5.9016
MOlport_LH1_Approved_FDA_library.maegz:847-1	Carfilzomib	719.910	-5.8983
MOlport_LH1_Approved_FDA_library.maegz:605-1	Proanthocyanidins	594.520	-5.8929
MOlport_LH1_Approved_FDA_library.maegz:9-37	Levomefolate calcium	497.520	-5.8905
MOlport_LH1_Approved_FDA_library.maegz:605-18	Proanthocyanidins	594.520	-5.8826
MOlport_LH1_Approved_FDA_library.maegz:192-1	Lonafarnib	638.820	-5.8764
MOlport_LH1_Approved_FDA_library.maegz:1597-3	Casanthranol	396.580	-5.8702
MOlport_LH1_Approved_FDA_library.maegz:605-21	Proanthocyanidins	594.520	-5.8569
MOlport_LH1_Approved_FDA_library.maegz:665-3	Felypressin	1040.220	-5.8467
MOlport_LH1_Approved_FDA_library.maegz:317-2	Prostaglandin E2 (PGE2)	352.470	-5.8461
MOlport_LH1_Approved_FDA_library.maegz:605-7	Proanthocyanidins	594.520	-5.8416
MOlport_LH1_Approved_FDA_library.maegz:605-23	Proanthocyanidins	594.520	-5.8371
MOlport_LH1_Approved_FDA_library.maegz:1868-1	Lactitol monohydrate	362.330	-5.8282
MOlport_LH1_Approved_FDA_library.maegz:1597-8	Casanthranol	396.580	-5.8275
MOlport_LH1_Approved_FDA_library.maegz:43-1	Tubercidin	266.250	-5.8165
MOlport_LH1_Approved_FDA_library.maegz:149-1	Dichlorphenamide	305.160	-5.8087
MOlport_LH1_Approved_FDA_library.maegz:1529-1	Adenosine	267.240	-5.8072
MOlport_LH1_Approved_FDA_library.maegz:605-21	Proanthocyanidins	594.520	-5.8064
MOlport_LH1_Approved_FDA_library.maegz:1868-1	Lactitol monohydrate	362.330	-5.8033
MOlport_LH1_Approved_FDA_library.maegz:1597-1	Casanthranol	396.580	-5.7968
MOlport_LH1_Approved_FDA_library.maegz:1023-2	Decitabine	228.210	-5.7963
MOlport_LH1_Approved_FDA_library.maegz:161-2	Cefoperazone	645.670	-5.7934
MOlport_LH1_Approved_FDA_library.maegz:1529-1	Adenosine	267.240	-5.7813
MOlport_LH1_Approved_FDA_library.maegz:802-1	2-Aminobenzenesulfonamide	172.210	-5.7715
MOlport_LH1_Approved_FDA_library.maegz:605-1	Proanthocyanidins	594.520	-5.7702
MOlport_LH1_Approved_FDA_library.maegz:822-3	ABT199	868.440	-5.7655
MOlport_LH1_Approved_FDA_library.maegz:1206-1	Hydrocortisone acetate	404.500	-5.7648
MOlport_LH1_Approved_FDA_library.maegz:268-10	Travoprost	500.550	-5.7619
MOlport_LH1_Approved_FDA_library.maegz:1259-1	Temozolomide	194.150	-5.7613
MOlport_LH1_Approved_FDA_library.maegz:848-8	Micafungin sodium	1292.260	-5.7611
MOlport_LH1_Approved_FDA_library.maegz:1059-3	Nadifloxacin	360.390	-5.7598
MOlport_LH1_Approved_FDA_library.maegz:1564-1	Acetazolamide	222.250	-5.7563
MOlport_LH1_Approved_FDA_library.maegz:1299-1	Oxiracetam	158.160	-5.7536
MOlport_LH1_Approved_FDA_library.maegz:496-1	Eslicarbazepine Acetate	296.320	-5.7461

MOlport_LH1_Approved_FDA_library.maegz:1931-7	Pioglitazone	356.440	-5.7348
MOlport_LH1_Approved_FDA_library.maegz:1931-3	Pioglitazone	356.440	-5.7266
MOlport_LH1_Approved_FDA_library.maegz:605-13	Proanthocyanidins	594.520	-5.725
MOlport_LH1_Approved_FDA_library.maegz:245-8	Anidulafungin	1140.240	-5.7203
MOlport_LH1_Approved_FDA_library.maegz:404-2	Stiripentol	234.290	-5.7118
MOlport_LH1_Approved_FDA_library.maegz:1439-4	Sennoside A	862.740	-5.7073
MOlport_LH1_Approved_FDA_library.maegz:1496-1	Diprophylline	254.250	-5.7028
MOlport_LH1_Approved_FDA_library.maegz:149-1	Dichlorphenamide	305.160	-5.7012
MOlport_LH1_Approved_FDA_library.maegz:22-1	Gestrinone	308.410	-5.6976
MOlport_LH1_Approved_FDA_library.maegz:1049-1	AICAR (Acadesine)	258.240	-5.6972
MOlport_LH1_Approved_FDA_library.maegz:573-1	Gastrodin	286.280	-5.6889
MOlport_LH1_Approved_FDA_library.maegz:268-32	Travoprost	500.550	-5.6855
MOlport_LH1_Approved_FDA_library.maegz:516-3	Cefminox Sodium	563.530	-5.6796
MOlport_LH1_Approved_FDA_library.maegz:314-3	Ceforanide	519.550	-5.6795
MOlport_LH1_Approved_FDA_library.maegz:1439-4	Sennoside A	862.740	-5.6736
MOlport_LH1_Approved_FDA_library.maegz:1153-2	Nitrofurantoin	238.160	-5.6729
MOlport_LH1_Approved_FDA_library.maegz:774-2	Trelagliptin succinate	475.470	-5.6721
MOlport_LH1_Approved_FDA_library.maegz:295-2	Glecaprevir	838.870	-5.6716
MOlport_LH1_Approved_FDA_library.maegz:208-2	Trelagliptin	357.380	-5.6706
MOlport_LH1_Approved_FDA_library.maegz:624-2	Glycyrrhizin	822.930	-5.6705
MOlport_LH1_Approved_FDA_library.maegz:1690-2	Diammonium Glycyrrhizinate	857.000	-5.6705
MOlport_LH1_Approved_FDA_library.maegz:1500-1	D(+)-Glucose	180.160	-5.6681
MOlport_LH1_Approved_FDA_library.maegz:9-40	Levomefolate calcium	497.520	-5.6661
MOlport_LH1_Approved_FDA_library.maegz:573-1	Gastrodin	286.280	-5.6618
MOlport_LH1_Approved_FDA_library.maegz:848-8	Micafungin sodium	1292.260	-5.6564
MOlport_LH1_Approved_FDA_library.maegz:22-1	Gestrinone	308.410	-5.6544
MOlport_LH1_Approved_FDA_library.maegz:1771-1	Piracetam	142.160	-5.6448
MOlport_LH1_Approved_FDA_library.maegz:1008-2	Nifuratel	285.280	-5.638
MOlport_LH1_Approved_FDA_library.maegz:1949-1	Levetiracetam	170.210	-5.6379
MOlport_LH1_Approved_FDA_library.maegz:1775-1	Inositol	180.160	-5.633
MOlport_LH1_Approved_FDA_library.maegz:687-1	Bimatoprost	415.570	-5.6328
MOlport_LH1_Approved_FDA_library.maegz:587-1	Glucosamine hydrochloride	215.630	-5.6299
MOlport_LH1_Approved_FDA_library.maegz:1870-1	Clofarabine	303.680	-5.6265
MOlport_LH1_Approved_FDA_library.maegz:434-5	Goserelin acetate	1329.460	-5.6226
MOlport_LH1_Approved_FDA_library.maegz:877-3	Aminophylline	420.430	-5.6218
MOlport_LH1_Approved_FDA_library.maegz:1992-2	Cefpiramide acid	612.640	-5.6211
MOlport_LH1_Approved_FDA_library.maegz:1293-1	Taurocholic acid sodium salt hydrate	537.680	-5.6195
MOlport_LH1_Approved_FDA_library.maegz:1931-4	Pioglitazone	356.440	-5.6192
MOlport_LH1_Approved_FDA_library.maegz:359-2	Hydrocortisone 21-hemisuccinate	462.530	-5.61
MOlport_LH1_Approved_FDA_library.maegz:605-7	Proanthocyanidins	594.520	-5.608

MOlport_LH1_Approved_FDA_library.maegz:1665-1	Ribavirin	244.200	-5.6054
MOlport_LH1_Approved_FDA_library.maegz:695-3	Zanamivir	332.310	-5.604
MOlport_LH1_Approved_FDA_library.maegz:807-1	Scopolamine N-oxide HBr	400.260	-5.604
MOlport_LH1_Approved_FDA_library.maegz:43-1	Tubercidin	266.250	-5.6027
MOlport_LH1_Approved_FDA_library.maegz:404-2	Stiripentol	234.290	-5.6009
MOlport_LH1_Approved_FDA_library.maegz:1018-1	Norethindrone	298.420	-5.5979
MOlport_LH1_Approved_FDA_library.maegz:829-3	MLN9708	517.120	-5.5939
MOlport_LH1_Approved_FDA_library.maegz:1358-4	Isoprenaline hydrochloride	247.720	-5.586
MOlport_LH1_Approved_FDA_library.maegz:318-1	Ethoxzolamide	258.320	-5.5852
MOlport_LH1_Approved_FDA_library.maegz:1005-3	Miglitol	207.220	-5.5834
MOlport_LH1_Approved_FDA_library.maegz:1597-28	Casanthranol	396.580	-5.5806
MOlport_LH1_Approved_FDA_library.maegz:1821-2	Cefadroxil	363.390	-5.5797
MOlport_LH1_Approved_FDA_library.maegz:1821-2	Cefadroxil	363.390	-5.579
MOlport_LH1_Approved_FDA_library.maegz:1500-1	D(+)-Glucose	180.160	-5.5783
MOlport_LH1_Approved_FDA_library.maegz:808-1	Apigenin	270.240	-5.5758
MOlport_LH1_Approved_FDA_library.maegz:1694-1	Tioxolone	168.170	-5.5668
MOlport_LH1_Approved_FDA_library.maegz:443-2	Pentostatin	268.270	-5.5648
MOlport_LH1_Approved_FDA_library.maegz:9-40	Levomefolate calcium	497.520	-5.5621
MOlport_LH1_Approved_FDA_library.maegz:9-40	Levomefolate calcium	497.520	-5.5551
MOlport_LH1_Approved_FDA_library.maegz:1942-5	Meptazinol hydrochloride	269.810	-5.555
MOlport_LH1_Approved_FDA_library.maegz:980-1	Prednisolone	360.440	-5.5525
MOlport_LH1_Approved_FDA_library.maegz:1146-3	Carprofen	273.710	-5.542
MOlport_LH1_Approved_FDA_library.maegz:817-2	Dorzolamide hydrochloride	360.900	-5.5397
MOlport_LH1_Approved_FDA_library.maegz:619-3	Kasugamycin hydrochloride	415.830	-5.5363
MOlport_LH1_Approved_FDA_library.maegz:1470-4	Allantoin	158.120	-5.5258
MOlport_LH1_Approved_FDA_library.maegz:1213-2	5,5-Dimethyloxazolidine-2,4-dione	129.120	-5.5243
MOlport_LH1_Approved_FDA_library.maegz:1377-3	Doripenem Hydrate	438.520	-5.5235
MOlport_LH1_Approved_FDA_library.maegz:1842-3	Rosiglitazone	357.430	-5.5218
MOlport_LH1_Approved_FDA_library.maegz:1190-1	Abacavir	286.340	-5.5199
MOlport_LH1_Approved_FDA_library.maegz:182-1	Abacavir sulfate	335.350	-5.5196
MOlport_LH1_Approved_FDA_library.maegz:969-1	Estriol	288.390	-5.5172
MOlport_LH1_Approved_FDA_library.maegz:1013-2	Aliskiren hemifumarate	1219.600	-5.5171
MOlport_LH1_Approved_FDA_library.maegz:1194-1	Levonorgestrel	312.450	-5.5163
MOlport_LH1_Approved_FDA_library.maegz:1496-2	Diprophylline	254.250	-5.5158
MOlport_LH1_Approved_FDA_library.maegz:666-1	Etonogestrel	324.460	-5.5145
MOlport_LH1_Approved_FDA_library.maegz:605-23	Proanthocyanidins	594.520	-5.5118
MOlport_LH1_Approved_FDA_library.maegz:817-2	Dorzolamide hydrochloride	360.900	-5.5089
MOlport_LH1_Approved_FDA_library.maegz:627-1	Scoparone	206.200	-5.5074
MOlport_LH1_Approved_FDA_library.maegz:9-44	Levomefolate calcium	497.520	-5.5043
MOlport_LH1_Approved_FDA_library.maegz:644-1	Norgestrel	312.450	-5.5037
MOlport_LH1_Approved_FDA_library.maegz:808-1	Apigenin	270.240	-5.5009

MOlport_LH1_Approved_FDA_library.maegz:605-29	Proanthocyanidins	594.520	-5.4986
MOlport_LH1_Approved_FDA_library.maegz:1259-1	Temozolomide	194.150	-5.4914
MOlport_LH1_Approved_FDA_library.maegz:1842-3	Rosiglitazone	357.430	-5.4905
MOlport_LH1_Approved_FDA_library.maegz:162-1	Calcium Levofolinate	511.500	-5.4873
MOlport_LH1_Approved_FDA_library.maegz:1597-12	Casanthranol	396.580	-5.4871
MOlport_LH1_Approved_FDA_library.maegz:1597-29	Casanthranol	396.580	-5.4858
MOlport_LH1_Approved_FDA_library.maegz:1610-1	Furazolidone	225.160	-5.4856
MOlport_LH1_Approved_FDA_library.maegz:829-3	MLN9708	517.120	-5.4802
MOlport_LH1_Approved_FDA_library.maegz:9-41	Levomefolate calcium	497.520	-5.4651
MOlport_LH1_Approved_FDA_library.maegz:173-1	Anagrelide HCl	292.550	-5.4585
MOlport_LH1_Approved_FDA_library.maegz:1974-1	Anagrelide	256.090	-5.4585
MOlport_LH1_Approved_FDA_library.maegz:307-2	Olprinone	250.260	-5.4568
MOlport_LH1_Approved_FDA_library.maegz:234-4	Desvenlafaxine succinate hydrate	399.480	-5.4429
MOlport_LH1_Approved_FDA_library.maegz:963-4	Desvenlafaxine	263.380	-5.4429
MOlport_LH1_Approved_FDA_library.maegz:295-2	Glecaprevir	838.870	-5.4423
MOlport_LH1_Approved_FDA_library.maegz:1647-2	Triflusal	248.160	-5.4341
MOlport_LH1_Approved_FDA_library.maegz:2063-3	Folic acid	441.400	-5.4252
MOlport_LH1_Approved_FDA_library.maegz:149-1	Dichlorphenamide	305.160	-5.4198
MOlport_LH1_Approved_FDA_library.maegz:201-2	Bosentan	551.610	-5.4099
MOlport_LH1_Approved_FDA_library.maegz:441-1	Dantrolene sodium hemiheptahydrate	399.290	-5.4099
MOlport_LH1_Approved_FDA_library.maegz:162-1	Calcium Levofolinate	511.500	-5.4097
MOlport_LH1_Approved_FDA_library.maegz:361-2	Pentagastrin	767.890	-5.407
MOlport_LH1_Approved_FDA_library.maegz:265-48	Indinavir sulfate	711.870	-5.3939
MOlport_LH1_Approved_FDA_library.maegz:576-1	Scopoletin	192.170	-5.3892
MOlport_LH1_Approved_FDA_library.maegz:1120-1	4-Methylumbelliferone	176.170	-5.384
MOlport_LH1_Approved_FDA_library.maegz:318-1	Ethoxzolamide	258.320	-5.3828
MOlport_LH1_Approved_FDA_library.maegz:1990-2	Brinzolamide	383.510	-5.3817
MOlport_LH1_Approved_FDA_library.maegz:817-2	Dorzolamide hydrochloride	360.900	-5.3789
MOlport_LH1_Approved_FDA_library.maegz:1671-2	Chlorothiazide	295.720	-5.3706
MOlport_LH1_Approved_FDA_library.maegz:314-3	Ceforanide	519.550	-5.3639
MOlport_LH1_Approved_FDA_library.maegz:829-4	MLN9708	517.120	-5.3554
MOlport_LH1_Approved_FDA_library.maegz:1597-29	Casanthranol	396.580	-5.3485
MOlport_LH1_Approved_FDA_library.maegz:1470-4	Allantoin	158.120	-5.3453
MOlport_LH1_Approved_FDA_library.maegz:1249-4	Mizoribine	259.220	-5.3414
MOlport_LH1_Approved_FDA_library.maegz:74-3	Zaltoprofen	298.360	-5.3384
MOlport_LH1_Approved_FDA_library.maegz:1281-2	Albendazole	265.330	-5.3347
MOlport_LH1_Approved_FDA_library.maegz:554-5	5-hydroxytryptophan	220.230	-5.3265
MOlport_LH1_Approved_FDA_library.maegz:1387-2	Mafenide hydrochloride	222.690	-5.3238
MOlport_LH1_Approved_FDA_library.maegz:603-1	Daphnetin	178.140	-5.3066
MOlport_LH1_Approved_FDA_library.maegz:1974-1	Anagrelide	256.090	-5.3062
MOlport_LH1_Approved_FDA_library.maegz:173-1	Anagrelide HCl	292.550	-5.3059

MOlport_LH1_Approved_FDA_library.maegz:1757-1	Salicoside	286.280	-5.3027
MOlport_LH1_Approved_FDA_library.maegz:318-1	Ethoxzolamide	258.320	-5.3023
MOlport_LH1_Approved_FDA_library.maegz:624-2	Glycyrrhizin	822.930	-5.2983
MOlport_LH1_Approved_FDA_library.maegz:1690-2	Diammonium Glycyrrhizinate	857.000	-5.2983
MOlport_LH1_Approved_FDA_library.maegz:307-2	Olprinone	250.260	-5.2974
MOlport_LH1_Approved_FDA_library.maegz:877-3	Aminophylline	420.430	-5.286
MOlport_LH1_Approved_FDA_library.maegz:1775-1	Inositol	180.160	-5.2802
MOlport_LH1_Approved_FDA_library.maegz:1765-1	Dicurone	176.120	-5.2735
MOlport_LH1_Approved_FDA_library.maegz:643-3	Thymopentin	679.560	-5.2597
MOlport_LH1_Approved_FDA_library.maegz:1975-3	Pranoprofen	255.280	-5.2587
MOlport_LH1_Approved_FDA_library.maegz:1500-1	D(+)-Glucose	180.160	-5.2576
MOlport_LH1_Approved_FDA_library.maegz:791-2	Serotonin hydrochloride	212.680	-5.2503
MOlport_LH1_Approved_FDA_library.maegz:1771-1	Piracetam	142.160	-5.2435
MOlport_LH1_Approved_FDA_library.maegz:315-2	Desmopressin	1069.220	-5.2239
MOlport_LH1_Approved_FDA_library.maegz:532-2	Carbendazim	191.190	-5.2225
MOlport_LH1_Approved_FDA_library.maegz:1530-2	Carbazochrome sodium sulfonate	376.320	-5.2224
MOlport_LH1_Approved_FDA_library.maegz:1194-1	Levonorgestrel	312.450	-5.2053
MOlport_LH1_Approved_FDA_library.maegz:1530-2	Carbazochrome sodium sulfonate	376.320	-5.1967
MOlport_LH1_Approved_FDA_library.maegz:1952-2	Pemetrexed acid	427.410	-5.1955
MOlport_LH1_Approved_FDA_library.maegz:9-44	Levomefolate calcium	497.520	-5.1914
MOlport_LH1_Approved_FDA_library.maegz:1213-2	5,5-Dimethyloxazolidine-2,4-dione	129.120	-5.1904
MOlport_LH1_Approved_FDA_library.maegz:1951-2	Pemetrexed disodium	471.370	-5.1869
MOlport_LH1_Approved_FDA_library.maegz:86-3	Suprofen	260.310	-5.1839
MOlport_LH1_Approved_FDA_library.maegz:219-2	Pemetrexed Disodium Hydrate	516.420	-5.1788
MOlport_LH1_Approved_FDA_library.maegz:284-2	N-Acetyl-L-tyrosine	223.230	-5.1541
MOlport_LH1_Approved_FDA_library.maegz:284-2	N-Acetyl-L-tyrosine	223.230	-5.1484
MOlport_LH1_Approved_FDA_library.maegz:466-3	Carbazochrome	236.230	-5.1376
MOlport_LH1_Approved_FDA_library.maegz:270-4	DL-Menthol	156.260	-5.1307
MOlport_LH1_Approved_FDA_library.maegz:1404-2	Amoxicillin trihydrate	419.450	-5.1168
MOlport_LH1_Approved_FDA_library.maegz:1405-2	Amoxicillin	365.400	-5.1168
MOlport_LH1_Approved_FDA_library.maegz:1856-1	Citiolone	159.210	-5.1143
MOlport_LH1_Approved_FDA_library.maegz:1470-3	Allantoin	158.120	-5.1096
MOlport_LH1_Approved_FDA_library.maegz:2059-2	Nicotinic acid N-oxide	139.110	-5.1069
MOlport_LH1_Approved_FDA_library.maegz:466-4	Carbazochrome	236.230	-5.1046
MOlport_LH1_Approved_FDA_library.maegz:1765-2	Dicurone	176.120	-5.0997
MOlport_LH1_Approved_FDA_library.maegz:500-2	Delapril?Hydrochloride	489.010	-5.0955
MOlport_LH1_Approved_FDA_library.maegz:791-2	Serotonin hydrochloride	212.680	-5.0101
MOlport_LH1_Approved_FDA_library.maegz:1937-1	Prednisolone phosphate sodium	484.390	-5.0046

MOlport_LH1_Approved_FDA_library.maegz:961-1	Naproxen sodium	252.240	-4.9933
MOlport_LH1_Approved_FDA_library.maegz:962-2	Naproxen	230.270	-4.9933
MOlport_LH1_Approved_FDA_library.maegz:946-2	Rimantadine hydrochloride	215.760	-4.9919
MOlport_LH1_Approved_FDA_library.maegz:1387-2	Mafenide hydrochloride	222.690	-4.9857
MOlport_LH1_Approved_FDA_library.maegz:262-2	Menadione sodium bisulfite	276.240	-4.9834
MOlport_LH1_Approved_FDA_library.maegz:1093-3	Fosfosal	218.100	-4.9804
MOlport_LH1_Approved_FDA_library.maegz:536-1	Povidone-iodine	364.950	-4.9685
MOlport_LH1_Approved_FDA_library.maegz:1354-2	Ticarcillin disodium	428.390	-4.9644
MOlport_LH1_Approved_FDA_library.maegz:1217-2	Pamabrom	348.200	-4.962
MOlport_LH1_Approved_FDA_library.maegz:1352-4	Baclofen	213.660	-4.9554
MOlport_LH1_Approved_FDA_library.maegz:1146-3	Carprofen	273.710	-4.9307
MOlport_LH1_Approved_FDA_library.maegz:2059-2	Nicotinic acid N-oxide	139.110	-4.9004
MOlport_LH1_Approved_FDA_library.maegz:156-4	Clinofibrate	468.580	-4.8935
MOlport_LH1_Approved_FDA_library.maegz:284-2	N-Acetyl-L-tyrosine	223.230	-4.892
MOlport_LH1_Approved_FDA_library.maegz:1271-2	Acipimox	154.130	-4.8882
MOlport_LH1_Approved_FDA_library.maegz:1975-3	Pranoprofen	255.280	-4.8768
MOlport_LH1_Approved_FDA_library.maegz:1470-3	Allantoin	158.120	-4.8674
MOlport_LH1_Approved_FDA_library.maegz:665-3	Felypressin	1040.220	-4.8651
MOlport_LH1_Approved_FDA_library.maegz:1124-3	Acetylleucine	173.210	-4.8426
MOlport_LH1_Approved_FDA_library.maegz:1975-4	Pranoprofen	255.280	-4.8388
MOlport_LH1_Approved_FDA_library.maegz:1093-3	Fosfosal	218.100	-4.8291
MOlport_LH1_Approved_FDA_library.maegz:1124-3	Acetylleucine	173.210	-4.8289
MOlport_LH1_Approved_FDA_library.maegz:361-2	Pentagastrin	767.890	-4.8281
MOlport_LH1_Approved_FDA_library.maegz:263-1	Calcium dobesilate	418.410	-4.8217
MOlport_LH1_Approved_FDA_library.maegz:2110-2	Ethamsylate	263.310	-4.8212
MOlport_LH1_Approved_FDA_library.maegz:829-3	MLN9708	517.120	-4.819
MOlport_LH1_Approved_FDA_library.maegz:910-1	Strontium ranelate	513.490	-4.8096
MOlport_LH1_Approved_FDA_library.maegz:130-2	Gabapentin HCl	207.700	-4.7978
MOlport_LH1_Approved_FDA_library.maegz:1650-2	Gabapentin	171.240	-4.7978
MOlport_LH1_Approved_FDA_library.maegz:1452-2	Amstat	157.210	-4.7826
MOlport_LH1_Approved_FDA_library.maegz:2110-2	Ethamsylate	263.310	-4.7636
MOlport_LH1_Approved_FDA_library.maegz:263-1	Calcium dobesilate	418.410	-4.7629
MOlport_LH1_Approved_FDA_library.maegz:1093-3	Fosfosal	218.100	-4.7456
MOlport_LH1_Approved_FDA_library.maegz:73-6	Zoledronic Acid	272.090	-4.7421
MOlport_LH1_Approved_FDA_library.maegz:1452-2	Amstat	157.210	-4.729
MOlport_LH1_Approved_FDA_library.maegz:73-6	Zoledronic Acid	272.090	-4.6068
MOlport_LH1_Approved_FDA_library.maegz:625-4	Sodium Demethylcantharidate	230.140	-4.5951
MOlport_LH1_Approved_FDA_library.maegz:1124-3	Acetylleucine	173.210	-4.5833
MOlport_LH1_Approved_FDA_library.maegz:910-1	Strontium ranelate	513.490	-4.4676
MOlport_LH1_Approved_FDA_library.maegz:179-5	Alendronate sodium hydrate	325.120	-4.4403

MOlport_LH1_Approved_FDA_library.maegz:73-3	Zoledronic Acid	272.090	-4.4392
MOlport_LH1_Approved_FDA_library.maegz:677-2	Succimer	182.220	-4.3763
MOlport_LH1_Approved_FDA_library.maegz:625-3	Sodium Demethylcantharidate	230.140	-4.3703
MOlport_LH1_Approved_FDA_library.maegz:1135-3	Pamidronate disodium salt	279.030	-4.363
MOlport_LH1_Approved_FDA_library.maegz:1373-5	Risedronate Sodium	305.090	-4.3451
MOlport_LH1_Approved_FDA_library.maegz:1607-3	Carbaglu	190.150	-4.3395
MOlport_LH1_Approved_FDA_library.maegz:625-2	Sodium Demethylcantharidate	230.140	-4.3375
MOlport_LH1_Approved_FDA_library.maegz:1805-4	Risedronic Acid	283.110	-4.3274
MOlport_LH1_Approved_FDA_library.maegz:1135-4	Pamidronate disodium salt	279.030	-4.2888
MOlport_LH1_Approved_FDA_library.maegz:179-5	Alendronate sodium hydrate	325.120	-4.2524
MOlport_LH1_Approved_FDA_library.maegz:625-4	Sodium Demethylcantharidate	230.140	-4.2341
MOlport_LH1_Approved_FDA_library.maegz:625-2	Sodium Demethylcantharidate	230.140	-4.2215
MOlport_LH1_Approved_FDA_library.maegz:625-1	Sodium Demethylcantharidate	230.140	-4.1806
MOlport_LH1_Approved_FDA_library.maegz:625-1	Sodium Demethylcantharidate	230.140	-4.1507
MOlport_LH1_Approved_FDA_library.maegz:1135-3	Pamidronate disodium salt	279.030	-4.1264
MOlport_LH1_Approved_FDA_library.maegz:677-2	Succimer	182.220	-4.1262
MOlport_LH1_Approved_FDA_library.maegz:73-3	Zoledronic Acid	272.090	-4.1165
MOlport_LH1_Approved_FDA_library.maegz:625-1	Sodium Demethylcantharidate	230.140	-4.022
MOlport_LH1_Approved_FDA_library.maegz:1373-5	Risedronate Sodium	305.090	-4.0194
MOlport_LH1_Approved_FDA_library.maegz:1805-4	Risedronic Acid	283.110	-4.0098
MOlport_LH1_Approved_FDA_library.maegz:1135-4	Pamidronate disodium salt	279.030	-3.9706
MOlport_LH1_Approved_FDA_library.maegz:677-2	Succimer	182.220	-3.947
MOlport_LH1_Approved_FDA_library.maegz:1135-3	Pamidronate disodium salt	279.030	-3.9186
MOlport_LH1_Approved_FDA_library.maegz:625-3	Sodium Demethylcantharidate	230.140	-3.9074
MOlport_LH1_Approved_FDA_library.maegz:1135-4	Pamidronate disodium salt	279.030	-3.8751
MOlport_LH1_Approved_FDA_library.maegz:1861-3	Etidronate	206.030	-3.8242
MOlport_LH1_Approved_FDA_library.maegz:1233-3	Sodium etidronate	249.990	-3.8218
MOlport_LH1_Approved_FDA_library.maegz:1233-2	Sodium etidronate	249.990	-3.7385
MOlport_LH1_Approved_FDA_library.maegz:1861-4	Etidronate	206.030	-3.737
MOlport_LH1_Approved_FDA_library.maegz:1233-3	Sodium etidronate	249.990	-3.6676
MOlport_LH1_Approved_FDA_library.maegz:1861-3	Etidronate	206.030	-3.6646
MOlport_LH1_Approved_FDA_library.maegz:1233-3	Sodium etidronate	249.990	-3.4723
MOlport_LH1_Approved_FDA_library.maegz:1861-3	Etidronate	206.030	-3.4702
MOlport_LH1_Approved_FDA_library.maegz:469-1	Gastrodenol	704.470	-3.0224

Table S16. VS1 and VS2 for DSI-poised targeting XPD

Compound	Catalog ID	MW	Energy in silico
VS1			
DSi PoisedLibrary_1680186639697.11	Z19727416	199.040	-4.3478
VS2			
DSi PoisedLibrary_1680186639697.773	Z193091370	192.057	-5.4688
DSi PoisedLibrary_1680186639697.11	Z19727416	199.040	-5.4652
DSi PoisedLibrary_1680186639697.509	Z1587220559	239.106	-5.4311
DSi PoisedLibrary_1680186639697.11	Z19727416	199.040	-5.3246
DSi PoisedLibrary_1680186639697.633	Z18769001	190.074	-5.1818
DSi PoisedLibrary_1680186639697.63	Z1004825200	299.729	-5.1454
DSi PoisedLibrary_1680186639697.803	Z383202616	175.075	-5.1415
DSi PoisedLibrary_1680186639697.773	Z193091370	192.057	-5.1401
DSi PoisedLibrary_1680186639697.63	Z1004825200	299.729	-5.0955
DSi PoisedLibrary_1680186639697.509	Z1587220559	239.106	-5.0535
DSi PoisedLibrary_1680186639697.209	Z270979606	163.100	-5.039
DSi PoisedLibrary_1680186639697.633	Z18769001	190.074	-5.0227
DSi PoisedLibrary_1680186639697.63	Z1004825200	299.729	-5.0048
DSi PoisedLibrary_1680186639697.68	Z26251905	203.106	-4.992
DSi PoisedLibrary_1680186639697.11	Z19727416	199.040	-4.9618
DSi PoisedLibrary_1680186639697.234	Z119990900	210.046	-4.9497
DSi PoisedLibrary_1680186639697.11	Z19727416	199.040	-4.9451
DSi PoisedLibrary_1680186639697.168	Z2255081886	173.059	-4.9314
DSi PoisedLibrary_1680186639697.754	Z1324853681	165.090	-4.9241
DSi PoisedLibrary_1680186639697.59	Z198194394	223.112	-4.9191
DSi PoisedLibrary_1680186639697.382	Z56923284	248.062	-4.9145
DSi PoisedLibrary_1680186639697.812	Z57627041	195.101	-4.9131
DSi PoisedLibrary_1680186639697.217	Z220816104	170.106	-4.8978
DSi PoisedLibrary_1680186639697.754	Z1324853681	165.090	-4.8962
DSi PoisedLibrary_1680186639697.64	Z287484230	167.106	-4.8927
DSi PoisedLibrary_1680186639697.137	Z285141302	190.111	-4.8903
DSi PoisedLibrary_1680186639697.52	Z954606858	199.067	-4.886
DSi PoisedLibrary_1680186639697.137	Z285141302	190.111	-4.8812
DSi PoisedLibrary_1680186639697.217	Z220816104	170.106	-4.8785
DSi PoisedLibrary_1680186639697.639	Z979770898	159.090	-4.8757
DSi PoisedLibrary_1680186639697.828	Z277969624	236.023	-4.8677
DSi PoisedLibrary_1680186639697.374	Z1446981563	233.328	-4.8628
DSi PoisedLibrary_1680186639697.52	Z954606858	199.067	-4.8596
DSi PoisedLibrary_1680186639697.657	Z44609285	244.264	-4.8584
DSi PoisedLibrary_1680186639697.741	Z1295863442	189.026	-4.8423
DSi PoisedLibrary_1680186639697.562	Z1198316457	163.081	-4.8255

DSi PoisedLibrary_1680186639697.11	Z19727416	199.040	-4.8182
DSi PoisedLibrary_1680186639697.547	Z55290386	246.080	-4.815
DSi PoisedLibrary_1680186639697.151	Z19739650	190.074	-4.8125
DSi PoisedLibrary_1680186639697.47	Z1216837642	142.111	-4.8118
DSi PoisedLibrary_1680186639697.633	Z18769001	190.074	-4.8082
DSi PoisedLibrary_1680186639697.11	Z19727416	199.040	-4.8059
DSi PoisedLibrary_1680186639697.85	Z1443544073	189.057	-4.805
DSi PoisedLibrary_1680186639697.382	Z56923284	248.062	-4.8045
DSi PoisedLibrary_1680186639697.278	Z1551999220	241.010	-4.796
DSi PoisedLibrary_1680186639697.278	Z1551999220	241.010	-4.7944
DSi PoisedLibrary_1680186639697.153	Z2033637875	210.256	-4.7848
DSi PoisedLibrary_1680186639697.217	Z220816104	170.106	-4.7772
DSi PoisedLibrary_1680186639697.802	Z425757818	179.062	-4.774
DSi PoisedLibrary_1680186639697.696	Z1255402624	166.099	-4.7713
DSi PoisedLibrary_1680186639697.136	Z44584202	234.226	-4.7605
DSi PoisedLibrary_1680186639697.146	Z68639747	203.106	-4.7565
DSi PoisedLibrary_1680186639697.503	Z223388508	242.316	-4.7557
DSi PoisedLibrary_1680186639697.436	Z44586758	248.228	-4.7526
DSi PoisedLibrary_1680186639697.773	Z193091370	192.057	-4.7525
DSi PoisedLibrary_1680186639697.278	Z1551999220	241.010	-4.7521
DSi PoisedLibrary_1680186639697.205	Z371866204	237.057	-4.747
DSi PoisedLibrary_1680186639697.278	Z1551999220	241.010	-4.7439
DSi PoisedLibrary_1680186639697.94	Z133622412	221.072	-4.7407
DSi PoisedLibrary_1680186639697.236	Z1333043510	226.078	-4.7335
DSi PoisedLibrary_1680186639697.639	Z979770898	159.090	-4.7331
DSi PoisedLibrary_1680186639697.260	Z90122368	171.101	-4.7286
DSi PoisedLibrary_1680186639697.696	Z1255402624	166.099	-4.7255
DSi PoisedLibrary_1680186639697.850	Z2120167298	217.057	-4.7253
DSi PoisedLibrary_1680186639697.151	Z19739650	190.074	-4.7067
DSi PoisedLibrary_1680186639697.374	Z1446981563	233.328	-4.702
DSi PoisedLibrary_1680186639697.85	Z1443544073	189.057	-4.7011
DSi PoisedLibrary_1680186639697.731	Z1203252645	150.079	-4.696
DSi PoisedLibrary_1680186639697.759	Z45527714	204.996	-4.6927
DSi PoisedLibrary_1680186639697.575	Z104492884	249.057	-4.6923
DSi PoisedLibrary_1680186639697.467	Z822382694	200.051	-4.6918
DSi PoisedLibrary_1680186639697.84	Z2237072016	164.070	-4.6914
DSi PoisedLibrary_1680186639697.260	Z90122368	171.101	-4.6913
DSi PoisedLibrary_1680186639697.278	Z1551999220	241.010	-4.6899
DSi PoisedLibrary_1680186639697.382	Z56923284	248.062	-4.6892
DSi PoisedLibrary_1680186639697.430	Z134785326	235.099	-4.6833
DSi PoisedLibrary_1680186639697.754	Z1324853681	165.090	-4.6786
DSi PoisedLibrary_1680186639697.639	Z979770898	159.090	-4.6782
DSi PoisedLibrary_1680186639697.639	Z979770898	159.090	-4.676

DSi PoisedLibrary_1680186639697.85	Z1443544073	189.057	-4.671
DSi PoisedLibrary_1680186639697.334	Z414477942	235.099	-4.6656
DSi PoisedLibrary_1680186639697.759	Z45527714	204.996	-4.6646
DSi PoisedLibrary_1680186639697.257	Z336080990	175.111	-4.6604
DSi PoisedLibrary_1680186639697.823	Z26794305	191.069	-4.6601
DSi PoisedLibrary_1680186639697.258	Z1503180352	191.095	-4.6586
DSi PoisedLibrary_1680186639697.430	Z134785326	235.099	-4.6565
DSi PoisedLibrary_1680186639697.750	Z1192341021	176.095	-4.6502
DSi PoisedLibrary_1680186639697.35	Z952656810	152.095	-4.6489
DSi PoisedLibrary_1680186639697.78	Z906021418	199.051	-4.6462
DSi PoisedLibrary_1680186639697.165	Z52214433	149.084	-4.6431
DSi PoisedLibrary_1680186639697.234	Z119990900	210.046	-4.6383
DSi PoisedLibrary_1680186639697.679	Z385450668	223.011	-4.6373
DSi PoisedLibrary_1680186639697.854	Z438096750	234.104	-4.6356
DSi PoisedLibrary_1680186639697.696	Z1255402624	166.099	-4.6356
DSi PoisedLibrary_1680186639697.665	Z53116498	203.106	-4.6224
DSi PoisedLibrary_1680186639697.165	Z52214433	149.084	-4.6198
DSi PoisedLibrary_1680186639697.550	Z45497619	217.057	-4.6172
DSi PoisedLibrary_1680186639697.261	Z32399802	170.106	-4.6088
DSi PoisedLibrary_1680186639697.52	Z954606858	199.067	-4.6068
DSi PoisedLibrary_1680186639697.639	Z979770898	159.090	-4.6
DSi PoisedLibrary_1680186639697.430	Z134785326	235.099	-4.5968
DSi PoisedLibrary_1680186639697.334	Z414477942	235.099	-4.588
DSi PoisedLibrary_1680186639697.217	Z220816104	170.106	-4.5866
DSi PoisedLibrary_1680186639697.209	Z270979606	163.100	-4.5849
DSi PoisedLibrary_1680186639697.374	Z1446981563	233.328	-4.5822
DSi PoisedLibrary_1680186639697.773	Z193091370	192.057	-4.5799
DSi PoisedLibrary_1680186639697.261	Z32399802	170.106	-4.5751
DSi PoisedLibrary_1680186639697.411	Z56921372	182.051	-4.5682
DSi PoisedLibrary_1680186639697.261	Z32399802	170.106	-4.5662
DSi PoisedLibrary_1680186639697.719	Z332370018	203.069	-4.5652
DSi PoisedLibrary_1680186639697.514	Z818928496	191.098	-4.559
DSi PoisedLibrary_1680186639697.234	Z119990900	210.046	-4.5533
DSi PoisedLibrary_1680186639697.695	Z31432009	212.152	-4.5526
DSi PoisedLibrary_1680186639697.151	Z19739650	190.074	-4.5446
DSi PoisedLibrary_1680186639697.812	Z57627041	195.101	-4.5338
DSi PoisedLibrary_1680186639697.639	Z979770898	159.090	-4.5227
DSi PoisedLibrary_1680186639697.773	Z193091370	192.057	-4.519
DSi PoisedLibrary_1680186639697.848	Z359331368	230.036	-4.5185
DSi PoisedLibrary_1680186639697.260	Z90122368	171.101	-4.5144
DSi PoisedLibrary_1680186639697.773	Z193091370	192.057	-4.5138
DSi PoisedLibrary_1680186639697.374	Z1446981563	233.328	-4.5121
DSi PoisedLibrary_1680186639697.217	Z220816104	170.106	-4.5102

DSi PoisedLibrary_1680186639697.759	Z45527714	204.996	-4.5032
DSi PoisedLibrary_1680186639697.560	Z133716556	227.062	-4.502
DSi PoisedLibrary_1680186639697.103	Z54748186	248.321	-4.5005
DSi PoisedLibrary_1680186639697.374	Z1446981563	233.328	-4.4954
DSi PoisedLibrary_1680186639697.700	Z18197050	215.025	-4.491
DSi PoisedLibrary_1680186639697.398	Z1162778919	205.085	-4.4904
DSi PoisedLibrary_1680186639697.746	Z1171217431	143.058	-4.4892
DSi PoisedLibrary_1680186639697.754	Z1324853681	165.090	-4.4891
DSi PoisedLibrary_1680186639697.398	Z1162778919	205.085	-4.4856
DSi PoisedLibrary_1680186639697.550	Z45497619	217.057	-4.4737
DSi PoisedLibrary_1680186639697.562	Z1198316457	163.081	-4.4722
DSi PoisedLibrary_1680186639697.168	Z2255081886	173.059	-4.4649
DSi PoisedLibrary_1680186639697.800	Z133632670	207.057	-4.4625
DSi PoisedLibrary_1680186639697.514	Z818928496	191.098	-4.4616
DSi PoisedLibrary_1680186639697.806	Z1262633486	175.056	-4.4548
DSi PoisedLibrary_1680186639697.695	Z31432009	212.152	-4.4483
DSi PoisedLibrary_1680186639697.541	Z1267773765	244.078	-4.4464
DSi PoisedLibrary_1680186639697.645	Z234898257	162.091	-4.4421
DSi PoisedLibrary_1680186639697.605	Z1696091761	198.056	-4.4315
DSi PoisedLibrary_1680186639697.137	Z285141302	190.111	-4.4295
DSi PoisedLibrary_1680186639697.817	Z1250093582	173.167	-4.4125
DSi PoisedLibrary_1680186639697.576	Z56900769	194.058	-4.4097
DSi PoisedLibrary_1680186639697.535	Z2153384824	181.085	-4.3894
DSi PoisedLibrary_1680186639697.468	Z2049876225	187.084	-4.3819
DSi PoisedLibrary_1680186639697.278	Z1551999220	241.010	-4.3754
DSi PoisedLibrary_1680186639697.217	Z220816104	170.106	-4.3511
DSi PoisedLibrary_1680186639697.677	Z56978034	170.038	-4.348
DSi PoisedLibrary_1680186639697.562	Z1198316457	163.081	-4.3471
DSi PoisedLibrary_1680186639697.806	Z1262633486	175.056	-4.345
DSi PoisedLibrary_1680186639697.580	Z1198317053	191.069	-4.3438
DSi PoisedLibrary_1680186639697.47	Z1216837642	142.111	-4.3419
DSi PoisedLibrary_1680186639697.804	Z1491215378	214.054	-4.3354
DSi PoisedLibrary_1680186639697.617	Z432057220	139.075	-4.3282
DSi PoisedLibrary_1680186639697.541	Z1267773765	244.078	-4.3193
DSi PoisedLibrary_1680186639697.160	Z385440544	171.090	-4.319
DSi PoisedLibrary_1680186639697.468	Z2049876225	187.084	-4.3051
DSi PoisedLibrary_1680186639697.804	Z1491215378	214.054	-4.2968
DSi PoisedLibrary_1680186639697.135	Z1198221039	163.063	-4.2849
DSi PoisedLibrary_1680186639697.806	Z1262633486	175.056	-4.2799
DSi PoisedLibrary_1680186639697.146	Z68639747	203.106	-4.2755
DSi PoisedLibrary_1680186639697.535	Z2153384824	181.085	-4.2581
DSi PoisedLibrary_1680186639697.468	Z2049876225	187.084	-4.2504
DSi PoisedLibrary_1680186639697.468	Z2049876225	187.084	-4.2438

DSi PoisedLibrary_1680186639697.817	Z1250093582	173.167	-4.2292
DSi PoisedLibrary_1680186639697.468	Z2049876225	187.084	-4.2192
DSi PoisedLibrary_1680186639697.800	Z133632670	207.057	-4.2054
DSi PoisedLibrary_1680186639697.860	Z1216861874	161.029	-4.1742
DSi PoisedLibrary_1680186639697.47	Z1216837642	142.111	-4.1302
DSi PoisedLibrary_1680186639697.468	Z2049876225	187.084	-4.0972
DSi PoisedLibrary_1680186639697.800	Z133632670	207.057	-4.0891
DSi PoisedLibrary_1680186639697.47	Z1216837642	142.111	-4.0517

Table S17. VS1 and VS2 for PDB fragment library targeting XPD

Compound	PUBCHEM_EXACT_MASS	Energy in silico
VS1		
Procodazole	190.074	-5.3499
VS2		
L-tryptophanamide	203.106	-6.0208
L-tryptophanamide	203.106	-5.4057
L-tryptophanamide	203.106	-5.3584
2-(carbamoylamino)-3-phenylpropanoic acid	208.085	-5.3226
Thymidine	242.090	-5.2837
Procodazole	190.074	-5.2042
octahydroindolizine-1,6,7,8-tetrol	189.100	-5.203
Thymidine	242.090	-5.1721
L-tryptophanamide	203.106	-5.1425
3,4,5-trihydroxycyclohex-2-ene-1-carboxylic acid	174.053	-5.1346
1H-Benzo[d]imidazole-2-sulfonamide	197.026	-5.1025
3,4,5-trihydroxycyclohex-2-ene-1-carboxylic acid	174.053	-5.0767
Neopterine	253.081	-5.073
3,4,5-trihydroxycyclohex-2-ene-1-carboxylic acid	174.053	-5.0539
octahydroindolizine-1,6,7,8-tetrol	189.100	-5.0491
octahydroindolizine-1,6,7,8-tetrol	189.100	-5.0425
6-Hydroxy-2-benzothiazolesulfonamide	229.982	-4.9395
Piracetam	142.074	-4.8787
3,4,5-trihydroxycyclohex-2-ene-1-carboxylic acid	174.053	-4.8252
Resorcinol	110.037	-4.8217
Resorcinol	110.037	-4.8194
Procodazole	190.074	-4.8126
octahydroindolizine-1,6,7,8-tetrol	189.100	-4.7965
6-methyltetrahydropterin	181.096	-4.7827
Acetylglutamine	188.080	-4.7759
Piracetam	142.074	-4.758
Tetrahjydrobiopterin	241.117	-4.7439

Phloroglucinol	126.032	-4.7146
Phloroglucinol	126.032	-4.7145
Phloroglucinol	126.032	-4.7131
6-methyltetrahydropterin	181.096	-4.6524
chlorzoxazone	168.993	-4.6365
2-Amino-4-hydroxy-6-(hydroxymethyl)-7,8-dihydropteridine	195.076	-4.6358
Guanine	151.049	-4.6267
3,4,5-trihydroxycyclohex-2-ene-1-carboxylic acid	174.053	-4.591
Cytosine deoxyribonucleoside		-4.5285
6-methyltetrahydropterin	181.096	-4.5044
3,4,5-trihydroxycyclohex-2-ene-1-carboxylic acid	174.053	-4.4598
1H-Pyrrole-2-carboxylate		-4.2326

Table S18. VS1 and VS2 for LH1 FDA approved drugs targeting XPD

Drug Name	MolWt	Energy in silico
VS1		
Salvianolic acid B	718.590	-9.4796
VS2		
Pentagastrin	767.890	-6.9032
Pentagastrin	767.890	-6.7057
Desmopressin	1069.220	-6.6637
Indinavir sulfate	711.870	-6.6491
Indinavir sulfate	711.870	-6.5823
Troxerutin	742.670	-6.5747
Indinavir sulfate	711.870	-6.5135
Desmopressin	1069.220	-6.4979
Felypressin	1040.220	-6.3448
Racecadotril	385.490	-6.3129
Asunaprevir	748.290	-6.2978
Bimatoprost	415.570	-6.2756
Indinavir sulfate	711.870	-6.2594
Asunaprevir	748.290	-6.2379
Indinavir sulfate	711.870	-6.2065
Salvianolic acid B	718.590	-6.2062
Bimatoprost	415.570	-6.2004
Ezetimibe	409.430	-6.1708
Indinavir sulfate	711.870	-6.1669
Proanthocyanidins	594.520	-6.1629
Felypressin	1040.220	-6.162
Indinavir sulfate	711.870	-6.1499
Indinavir sulfate	711.870	-6.1461

Indinavir sulfate	711.870	-6.1373
Ceforanide	519.550	-6.1369
Indinavir sulfate	711.870	-6.1168
Proanthocyanidins	594.520	-6.0888
Indinavir sulfate	711.870	-6.0793
Carfilzomib	719.910	-6.0756
Proanthocyanidins	594.520	-6.0676
Indinavir sulfate	711.870	-6.0642
Indinavir sulfate	711.870	-6.0372
Travoprost	500.550	-6.0299
Indinavir sulfate	711.870	-6.0298
Indinavir sulfate	711.870	-6.0268
Proanthocyanidins	594.520	-6.0192
Indinavir sulfate	711.870	-5.9938
Ezetimibe	409.430	-5.9673
Indinavir sulfate	711.870	-5.937
Citicoline	488.320	-5.9309
Travoprost	500.550	-5.9271
Citicoline sodium	510.310	-5.9252
Indinavir sulfate	711.870	-5.913
Indinavir sulfate	711.870	-5.9087
Indinavir sulfate	711.870	-5.8851
Travoprost	500.550	-5.872
Proanthocyanidins	594.520	-5.8687
Proanthocyanidins	594.520	-5.8679
Salvianolic acid B	718.590	-5.85
Indinavir sulfate	711.870	-5.8499
Azilsartan Medoxomil	568.530	-5.8466
Pentagastrin	767.890	-5.8423
Indinavir sulfate	711.870	-5.8323
Indinavir sulfate	711.870	-5.8249
Indinavir sulfate	711.870	-5.8215
Indinavir sulfate	711.870	-5.8195
Levomefolate calcium	497.520	-5.8011
Indinavir sulfate	711.870	-5.7949
Casanthranol	396.580	-5.7852
Indinavir sulfate	711.870	-5.7791
Proanthocyanidins	594.520	-5.7761
Travoprost	500.550	-5.7633
Proanthocyanidins	594.520	-5.7541
Indinavir sulfate	711.870	-5.7509
Lapatinib ditosylate monohydrate	943.470	-5.7425
Proanthocyanidins	594.520	-5.7425

Lapatinib Ditosylate	925.460	-5.7386
Lapatinib	581.060	-5.7386
Casanthranol	396.580	-5.7372
Troxerutin	742.670	-5.7245
Silymarin	482.440	-5.7149
Casanthranol	396.580	-5.7134
Cefpiramide acid	612.640	-5.7026
Proanthocyanidins	594.520	-5.7019
Proanthocyanidins	594.520	-5.6977
Cefpiramide acid	612.640	-5.6945
Proanthocyanidins	594.520	-5.6883
Indinavir sulfate	711.870	-5.674
Gliquidone	527.630	-5.6706
Casanthranol	396.580	-5.6582
Casanthranol	396.580	-5.658
Isavuconazonium sulfate	814.840	-5.6565
Ticagrelor	522.570	-5.6533
Ilaprazole sodium	388.440	-5.6349
Ezetimibe	409.430	-5.6208
Travoprost	500.550	-5.6121
Indinavir sulfate	711.870	-5.6091
Isavuconazonium sulfate	814.840	-5.6089
Indinavir sulfate	711.870	-5.6046
VX661	520.500	-5.603
Proanthocyanidins	594.520	-5.6008
Lapatinib ditosylate monohydrate	943.470	-5.5903
Lapatinib Ditosylate	925.460	-5.5897
Lapatinib	581.060	-5.5897
Ceforanide	519.550	-5.5781
Cefonicid sodium	586.530	-5.5633
Indinavir sulfate	711.870	-5.5625
Casanthranol	396.580	-5.5586
Asunaprevir	748.290	-5.5571
Silymarin	482.440	-5.5547
Esomeprazole Magnesium	713.120	-5.552
Indinavir sulfate	711.870	-5.5511
Aloin	418.390	-5.5454
Fenoldopam mesylate	401.860	-5.5424
Deferoxamine Mesylate	656.790	-5.5356
Travoprost	500.550	-5.532
Thymopentin	679.560	-5.5314
Indinavir sulfate	711.870	-5.5296
Esomeprazole Magnesium	713.120	-5.5282

Epirubicin hydrochloride	579.980	-5.5222
Silymarin	482.440	-5.5164
Indinavir sulfate	711.870	-5.5055
Zonisamide	212.230	-5.5025
Casanthranol	396.580	-5.4998
Fenoldopam mesylate	401.860	-5.4995
Indinavir sulfate	711.870	-5.4978
Casanthranol	396.580	-5.4968
Tedizolid Phosphate	450.320	-5.4959
Atracurium besylate	1243.490	-5.4837
Travoprost	500.550	-5.4783
Thymopentin	679.560	-5.4733
Indinavir sulfate	711.870	-5.4716
Indinavir sulfate	711.870	-5.4632
Sennoside A	862.740	-5.4631
Hydroquinidine	326.430	-5.4625
Travoprost	500.550	-5.4601
Isoprenaline hydrochloride	247.720	-5.458
Naloxegol oxalate	741.830	-5.4571
Proanthocyanidins	594.520	-5.4526
Naloxegol oxalate	741.830	-5.4502
Isoprenaline hydrochloride	247.720	-5.4401
Allantoin	158.120	-5.4365
Norepinephrine	169.180	-5.4064
Noradrenaline bitartrate	319.260	-5.4064
Avatrombopag	649.650	-5.4063
DL-Adrenaline Hydrochloride	219.670	-5.4043
Naloxegol oxalate	741.830	-5.4012
Sivelestat	434.460	-5.397
Loxeen	391.520	-5.3953
Indinavir sulfate	711.870	-5.3773
Casanthranol	396.580	-5.3745
Diphenidol hydrochloride	345.910	-5.3688
Isoprenaline hydrochloride	247.720	-5.3655
Methyl hesperidin	624.590	-5.3605
Isoprenaline hydrochloride	247.720	-5.3541
Tedizolid Phosphate	450.320	-5.353
Desvenlafaxine succinate hydrate	399.480	-5.3475
Desvenlafaxine	263.380	-5.3475
Casanthranol	396.580	-5.3468
Bepotastine Besilate	547.060	-5.3383
Proanthocyanidins	594.520	-5.3364
Nafronyl oxalate	473.560	-5.3317

DL-Adrenaline Hydrochloride	219.670	-5.3306
Indinavir sulfate	711.870	-5.33
Aminophylline	420.430	-5.3274
Hydroquinidine	326.430	-5.3208
Indinavir sulfate	711.870	-5.3162
Isavuconazonium sulfate	814.840	-5.3152
Prostaglandin E2 (PGE2)	352.470	-5.3151
Felypressin	1040.220	-5.314
Methyl hesperidin	624.590	-5.311
Roxadustat	352.340	-5.3004
Isoprenaline hydrochloride	247.720	-5.2995
Desvenlafaxine succinate hydrate	399.480	-5.2961
Desvenlafaxine	263.380	-5.2961
Hesperidin	610.560	-5.2958
Felbamate	238.240	-5.2935
Thymopentin	679.560	-5.2911
Epirubicin hydrochloride	579.980	-5.2901
Travoprost	500.550	-5.2848
Citicoline	488.320	-5.2814
Citicoline sodium	510.310	-5.2766
Calcium folinate	511.510	-5.2719
Casanthranol	396.580	-5.2717
N-Acetyl-L-tyrosine	223.230	-5.267
Docusate sodium	444.560	-5.266
Sapropterin Hydrochloride	314.170	-5.2601
Doripenem Hydrate	438.520	-5.2577
Meptazinol hydrochloride	269.810	-5.2489
Dipivefrin hydrochloride	387.900	-5.2485
N-Acetyl-L-tyrosine	223.230	-5.2463
Proanthocyanidins	594.520	-5.2447
Droxidopa	213.190	-5.2424
Allantoin	158.120	-5.2421
Lafutidine	431.550	-5.2392
MLN9708	517.120	-5.2339
Casanthranol	396.580	-5.2303
Roxadustat	352.340	-5.2302
Citicoline	488.320	-5.2253
Indinavir sulfate	711.870	-5.2218
Citicoline sodium	510.310	-5.2187
Lisinopril dihydrate	441.520	-5.2183
Polymyxin B sulfate	1301.560	-5.2128
Lafutidine	431.550	-5.2096
Loxeen	391.520	-5.2087

Levomefolate calcium	497.520	-5.2084
Pioglitazone	356.440	-5.2059
AICAR (Acadesine)	258.240	-5.1981
Carprofen	273.710	-5.1837
Indinavir sulfate	711.870	-5.1824
DL-trihexyphenidyl hydrochloride	337.930	-5.1793
Loxeen	391.520	-5.1764
Casanthranol	396.580	-5.1681
Casanthranol	396.580	-5.166
Sennoside A	862.740	-5.1576
Sapropterin Hydrochloride	314.170	-5.1535
Ticarcillin disodium	428.390	-5.1499
Casanthranol	396.580	-5.148
Phenytoin sodium	274.250	-5.148
Phenytoin	252.270	-5.1456
Meptazinol hydrochloride	269.810	-5.1439
Rebamipide	370.790	-5.1405
Hesperidin	610.560	-5.1403
Mepiroxol	125.130	-5.1359
Allantoin	158.120	-5.1349
Carprofen	273.710	-5.1332
Darolutamide	398.850	-5.1331
Sulpiride	341.430	-5.1307
Ticarcillin disodium	428.390	-5.1298
Isoprenaline hydrochloride	247.720	-5.1258
Carprofen	273.710	-5.1208
Alcaftadine	307.390	-5.1198
Ademetionine	398.440	-5.1163
Roxadustat	352.340	-5.1161
Folinic Acid Calcium Salt Pentahydrate	601.580	-5.1159
Atracurium besylate	1243.490	-5.1151
Iguratimod	374.370	-5.1151
Esomeprazole Sodium	367.400	-5.115
Fostamatinib	580.460	-5.1072
R788 Disodium	624.420	-5.1018
Dichlorphenamide	305.160	-5.101
Carbenicillin disodium	422.360	-5.0946
Carprofen	273.710	-5.092
DL-Adrenaline Hydrochloride	219.670	-5.0859
Mefloquine hydrochloride	414.770	-5.0808
Levomefolate calcium	497.520	-5.0802
Levomefolate calcium	497.520	-5.0801
Allantoin	158.120	-5.0769

Felbamate	238.240	-5.0723
Levetiracetam	170.210	-5.0692
Scopoletin	192.170	-5.0658
Sapropterin Hydrochloride	314.170	-5.0644
Indinavir sulfate	711.870	-5.0557
Synephrine	167.210	-5.0534
Salicoside	286.280	-5.0531
Synephrine	167.210	-5.0508
Pemetrexed acid	427.410	-5.0497
Proanthocyanidins	594.520	-5.0442
Fraxetin	208.170	-5.0429
Nalmefene hydrochloride	375.890	-5.0429
Raltitrexed	458.490	-5.0427
Ilaprazole sodium	388.440	-5.0406
Panobinostat	349.430	-5.0389
Desvenlafaxine succinate hydrate	399.480	-5.0348
Desvenlafaxine	263.380	-5.0348
Esomeprazole Magnesium	713.120	-5.0333
Floxuridine	246.190	-5.0325
Zileuton	236.290	-5.0302
Mepiroxol	125.130	-5.0295
Synephrine	167.210	-5.0234
Indinavir sulfate	711.870	-5.0203
Zileuton	236.290	-5.0154
Carbendazim	191.190	-5.0116
Sitagliptin	407.320	-5.001
Indinavir sulfate	711.870	-4.9933
Casanthranol	396.580	-4.98
Dichlorphenamide	305.160	-4.9731
Pemetrexed Disodium Hydrate	516.420	-4.9699
10,11-Dihydrocarbamazepine	238.290	-4.9563
Naratriptan	371.930	-4.9555
AICAR (Acadesine)	258.240	-4.9522
Omeprazole	345.420	-4.9496
Levomefolate calcium	497.520	-4.9457
Proanthocyanidins	594.520	-4.9454
Nalmefene hydrochloride	375.890	-4.9434
10,11-Dihydrocarbamazepine	238.290	-4.9419
Panobinostat	349.430	-4.9386
Lisinopril dihydrate	441.520	-4.9362
Ticarcillin disodium	428.390	-4.9355
AICAR (Acadesine)	258.240	-4.9344
Cefadroxil	363.390	-4.93

Droxidopa	213.190	-4.923
Meptazinol hydrochloride	269.810	-4.9226
Pranoprofen	255.280	-4.9171
L-Tryptophan	204.230	-4.9164
Ronidazole	200.150	-4.9152
Droxidopa	213.190	-4.9099
Fludarabine	285.230	-4.9088
Mycophenolic acid	320.340	-4.9063
4-Methylumbelliferone	176.170	-4.8969
Chlortalidone	338.770	-4.8945
Indinavir sulfate	711.870	-4.8896
Ethamsylate	263.310	-4.8787
Piracetam	142.160	-4.8787
Calcium dobesilate	418.410	-4.8777
Norepinephrine	169.180	-4.8571
Noradrenaline bitartrate	319.260	-4.8571
Pranoprofen	255.280	-4.8564
4-Methylumbelliferone	176.170	-4.8512
Allantoin	158.120	-4.8452
Levetiracetam	170.210	-4.8417
Biapenem	350.390	-4.8408
Pemetrexed Disodium Hydrate	516.420	-4.8393
Meptazinol hydrochloride	269.810	-4.8379
Calcium folinate	511.510	-4.8367
Pranoprofen	255.280	-4.8354
Fenoprofen calcium dihydrate	558.630	-4.8352
Tioxolone	168.170	-4.8349
Doripenem Hydrate	438.520	-4.8335
Hydrochlorothiazide	297.740	-4.8325
Pranoprofen	255.280	-4.8303
Mepiroxol	125.130	-4.8293
Tioxolone	168.170	-4.8252
Mafenide hydrochloride	222.690	-4.8243
Pemetrexed acid	427.410	-4.8222
4-Methylumbelliferone	176.170	-4.8185
Ademetionine	398.440	-4.8122
Aminoglutethimide	232.280	-4.8104
Sitagliptin	407.320	-4.7997
Norepinephrine	169.180	-4.7971
Noradrenaline bitartrate	319.260	-4.7971
Pranoprofen	255.280	-4.7961
Sulfanilylurea	215.230	-4.796
Latamoxef sodium	564.440	-4.7909

Zaltoprofen	298.360	-4.7902
Capastat sulfate	752.760	-4.7855
DL-Menthol	156.260	-4.7839
Esomeprazole Sodium	367.400	-4.78
MLN9708	517.120	-4.7673
Levetiracetam	170.210	-4.7663
Piracetam	142.160	-4.7556
Pranoprofen	255.280	-4.7511
Mitiglinide calcium	704.910	-4.7506
Anthralin	226.230	-4.7482
Anthralin	226.230	-4.7459
Biapenem	350.390	-4.7459
Gallic acid	170.120	-4.7437
Mafenide hydrochloride	222.690	-4.7436
Pemetrexed disodium	471.370	-4.7377
L-Hydroxyprolin	131.130	-4.727
Levodopa	197.190	-4.7216
Cefprozil hydrate	407.440	-4.7184
Mycophenolic acid	320.340	-4.7133
DL-Menthol	156.260	-4.7106
Zileuton	236.290	-4.707
Chloroxylenol	156.610	-4.6904
Protocatechuic acid	154.120	-4.6819
Chloroxylenol	156.610	-4.6746
MLN9708	517.120	-4.6722
Delta-Gluconolactone	178.140	-4.6619
Oxiracetam	158.160	-4.6591
N-Acetyl-L-tyrosine	223.230	-4.6528
Flopropione	182.180	-4.6505
Zonisamide	212.230	-4.6485
Rebamipide	370.790	-4.6475
Delta-Gluconolactone	178.140	-4.6472
Dermofongin	179.600	-4.6456
Sulfamethazine sodium salt	300.310	-4.6449
L-5-Hydroxytryptophan	220.220	-4.6412
Chlorzoxazone	169.570	-4.641
Clofibric acid	214.650	-4.6358
Mafenide hydrochloride	222.690	-4.6348
Sulfanilamide	172.200	-4.6315
Flopropione	182.180	-4.631
Carbidopa Hydrate	244.240	-4.6063
Mandelic acid	152.140	-4.6045
Tyrosine	181.190	-4.5803

Serotonin hydrochloride	212.680	-4.5744
L-Hydroxyprolin	131.130	-4.5725
Clevudine	260.220	-4.5655
Piracetam	142.160	-4.5601
Meticrane	275.340	-4.5594
Dicurone	176.120	-4.5544
Dicurone	176.120	-4.5446
L-5-Hydroxytryptophan	220.220	-4.5341
Sulfanilamide	172.200	-4.5151
Dicurone	176.120	-4.504
Chromocarbe	190.150	-4.5022
Patchouli alcohol	222.200	-4.4982
Nicotinic acid N-oxide	139.110	-4.4955
Carbazochrome	236.230	-4.4599
5-hydroxytryptophan	220.230	-4.455
Amstat	157.210	-4.4522
Pemetrexed disodium	471.370	-4.4439
Dicurone	176.120	-4.4351
Carbazochrome sodium sulfonate	376.320	-4.4188
L-Hydroxyprolin	131.130	-4.4067
Histamine 2HCl	184.070	-4.4051
Histamine	111.150	-4.4051
Chromocarbe	190.150	-4.3922
Acipimox	154.130	-4.3779
Pasiniazid	290.280	-4.3705
Acipimox	154.130	-4.3588
Allantoin	158.120	-4.3522
Calcium dobesilate	418.410	-4.3458
Ethamsylate	263.310	-4.344
Nicotinic acid N-oxide	139.110	-4.3352
L-Histidine monohydrochloride monohydrat	209.630	-4.2631
histidine	155.150	-4.2631
Bismuth subsalicylat	362.090	-4.2574
Salicylic acid	138.120	-4.2571
Amstat	157.210	-4.2553
Carbaglu	190.150	-4.2215
Acipimox	154.130	-4.2109
Hydroxyurea	76.060	-4.1613
Dacarbazine	182.180	-3.9744
Prednisolone phosphate sodium	484.390	-3.9665
Deoxycholic acid	392.570	-3.9149
Hematoporphyrin dihydrochloride	671.610	-3.3402

Table S19. VS1 and VS2 for DSI-poised targeting MRE11 dimer

Compound	Catalog ID	MW	Energy in silico
VS1			
DSI_poised_library_2023.maegz:103	Z54748186	248.321	-3.643
VS2			
DSI_poised_library_2023.maegz:593	Z90503851	246.173	-4.377
DSI_poised_library_2023.maegz:718	Z55222357	251.280	-3.899
DSI_poised_library_2023.maegz:718	Z55222357	251.280	-3.878
DSI_poised_library_2023.maegz:593	Z90503851	246.173	-3.853
DSI_poised_library_2023.maegz:488	Z137811222	246.348	-3.818
DSI_poised_library_2023.maegz:513	Z56618096	240.163	-3.806
DSI_poised_library_2023.maegz:488	Z137811222	246.348	-3.801
DSI_poised_library_2023.maegz:772	Z2217052426	205.147	-3.799
DSI_poised_library_2023.maegz:772	Z2217052426	205.147	-3.765
DSI_poised_library_2023.maegz:172	Z1206890404	204.126	-3.757
DSI_poised_library_2023.maegz:103	Z54748186	248.321	-3.735
DSI_poised_library_2023.maegz:116	Z1891775331	261.070	-3.641
DSI_poised_library_2023.maegz:103	Z54748186	248.321	-3.628
DSI_poised_library_2023.maegz:585	Z123935312	232.321	-3.625
DSI_poised_library_2023.maegz:176	Z265541128	211.132	-3.617
DSI_poised_library_2023.maegz:103	Z54748186	248.321	-3.569
DSI_poised_library_2023.maegz:197	Z221421630	219.126	-3.55
DSI_poised_library_2023.maegz:15	Z131833926	228.333	-3.531
DSI_poised_library_2023.maegz:705	Z1127662991	211.076	-3.523
DSI_poised_library_2023.maegz:29	Z1457279522	272.746	-3.471
DSI_poised_library_2023.maegz:772	Z2217052426	205.147	-3.457
DSI_poised_library_2023.maegz:772	Z2217052426	205.147	-3.445
DSI_poised_library_2023.maegz:772	Z2217052426	205.147	-3.443
DSI_poised_library_2023.maegz:197	Z221421630	219.126	-3.441
DSI_poised_library_2023.maegz:29	Z1457279522	272.746	-3.437
DSI_poised_library_2023.maegz:169	Z905434478	197.087	-3.431
DSI_poised_library_2023.maegz:705	Z1127662991	211.076	-3.431
DSI_poised_library_2023.maegz:169	Z905434478	197.087	-3.423
DSI_poised_library_2023.maegz:513	Z56618096	240.163	-3.423
DSI_poised_library_2023.maegz:772	Z2217052426	205.147	-3.389
DSI_poised_library_2023.maegz:541	Z1267773765	244.078	-3.385
DSI_poised_library_2023.maegz:197	Z221421630	219.126	-3.373
DSI_poised_library_2023.maegz:36	Z1470724953	239.131	-3.344
DSI_poised_library_2023.maegz:116	Z1891775331	261.070	-3.342
DSI_poised_library_2023.maegz:593	Z90503851	246.173	-3.341
DSI_poised_library_2023.maegz:772	Z2217052426	205.147	-3.338
DSI_poised_library_2023.maegz:268	Z241832786	197.153	-3.327

DSI_poised_library_2023.maegz:772	Z2217052426	205.147	-3.324
DSI_poised_library_2023.maegz:772	Z2217052426	205.147	-3.323
DSI_poised_library_2023.maegz:172	Z1206890404	204.126	-3.314
DSI_poised_library_2023.maegz:265	Z1209494589	200.278	-3.309
DSI_poised_library_2023.maegz:772	Z2217052426	205.147	-3.29
DSI_poised_library_2023.maegz:754	Z1324853681	165.090	-3.288
DSI_poised_library_2023.maegz:754	Z1324853681	165.090	-3.285
DSI_poised_library_2023.maegz:541	Z1267773765	244.078	-3.261
DSI_poised_library_2023.maegz:541	Z1267773765	244.078	-3.255
DSI_poised_library_2023.maegz:430	Z134785326	235.099	-3.225
DSI_poised_library_2023.maegz:172	Z1206890404	204.126	-3.224
DSI_poised_library_2023.maegz:541	Z1267773765	244.078	-3.221
DSI_poised_library_2023.maegz:169	Z905434478	197.087	-3.219
DSI_poised_library_2023.maegz:456	Z992240004	199.111	-3.216
DSI_poised_library_2023.maegz:564	Z54846870	200.116	-3.208
DSI_poised_library_2023.maegz:36	Z1470724953	239.131	-3.201
DSI_poised_library_2023.maegz:772	Z2217052426	205.147	-3.152
DSI_poised_library_2023.maegz:512	Z871834402	225.130	-3.147
DSI_poised_library_2023.maegz:435	Z56953052	244.068	-3.138
DSI_poised_library_2023.maegz:390	Z274575916	192.137	-3.121
DSI_poised_library_2023.maegz:79	Z86130391	205.147	-3.115
DSI_poised_library_2023.maegz:543	Z1259042855	198.094	-3.109
DSI_poised_library_2023.maegz:68	Z26251905	203.106	-3.082
DSI_poised_library_2023.maegz:628	Z124039706	240.106	-3.077
DSI_poised_library_2023.maegz:116	Z1891775331	261.070	-3.077
DSI_poised_library_2023.maegz:15	Z131833926	228.333	-3.074
DSI_poised_library_2023.maegz:808	Z803064328	282.101	-3.071
DSI_poised_library_2023.maegz:274	Z57821475	237.128	-3.057
DSI_poised_library_2023.maegz:754	Z1324853681	165.090	-3.05
DSI_poised_library_2023.maegz:426	Z56781951	129.115	-3.042
DSI_poised_library_2023.maegz:292	Z1868351290	207.174	-3.036
DSI_poised_library_2023.maegz:384	Z1428159350	224.083	-3.034
DSI_poised_library_2023.maegz:15	Z131833926	228.333	-3.033
DSI_poised_library_2023.maegz:729	Z991506900	191.142	-3.028
DSI_poised_library_2023.maegz:355	Z1142623285	171.174	-3.024
DSI_poised_library_2023.maegz:47	Z1216837642	142.111	-3.019
DSI_poised_library_2023.maegz:808	Z803064328	282.101	-3.017
DSI_poised_library_2023.maegz:115	Z1449748885	216.067	-3.011
DSI_poised_library_2023.maegz:627	Z1262246195	232.059	-2.999
DSI_poised_library_2023.maegz:729	Z991506900	191.142	-2.986
DSI_poised_library_2023.maegz:418	Z477328060	142.111	-2.977
DSI_poised_library_2023.maegz:103	Z54748186	248.321	-2.977
DSI_poised_library_2023.maegz:384	Z1428159350	224.083	-2.976

DSI_poised_library_2023.maegz:740	Z57329001	215.111	-2.948
DSI_poised_library_2023.maegz:15	Z131833926	228.333	-2.929
DSI_poised_library_2023.maegz:426	Z56781951	129.115	-2.92
DSI_poised_library_2023.maegz:47	Z1216837642	142.111	-2.914
DSI_poised_library_2023.maegz:512	Z871834402	225.130	-2.911
DSI_poised_library_2023.maegz:68	Z26251905	203.106	-2.897
DSI_poised_library_2023.maegz:772	Z2217052426	205.147	-2.893
DSI_poised_library_2023.maegz:485	Z57326966	204.072	-2.89
DSI_poised_library_2023.maegz:295	Z1457921102	240.103	-2.881
DSI_poised_library_2023.maegz:754	Z1324853681	165.090	-2.875
DSI_poised_library_2023.maegz:384	Z1428159350	224.083	-2.855
DSI_poised_library_2023.maegz:390	Z274575916	192.137	-2.853
DSI_poised_library_2023.maegz:256	Z166645986	142.111	-2.85
DSI_poised_library_2023.maegz:418	Z477328060	142.111	-2.842
DSI_poised_library_2023.maegz:256	Z166645986	142.111	-2.842
DSI_poised_library_2023.maegz:115	Z1449748885	216.067	-2.836
DSI_poised_library_2023.maegz:827	Z1758466556	212.144	-2.814
DSI_poised_library_2023.maegz:246	Z44095027	228.126	-2.811
DSI_poised_library_2023.maegz:501	Z228587394	194.117	-2.805
DSI_poised_library_2023.maegz:433	Z1741959530	197.105	-2.8
DSI_poised_library_2023.maegz:268	Z241832786	197.153	-2.8
DSI_poised_library_2023.maegz:348	Z1198172663	177.090	-2.782
DSI_poised_library_2023.maegz:92	Z85956652	234.033	-2.777
DSI_poised_library_2023.maegz:614	Z439694282	158.106	-2.776
DSI_poised_library_2023.maegz:68	Z26251905	203.106	-2.77
DSI_poised_library_2023.maegz:553	Z56960248	179.095	-2.763
DSI_poised_library_2023.maegz:827	Z1758466556	212.144	-2.763
DSI_poised_library_2023.maegz:355	Z1142623285	171.174	-2.759
DSI_poised_library_2023.maegz:268	Z241832786	197.153	-2.743
DSI_poised_library_2023.maegz:512	Z871834402	225.130	-2.742
DSI_poised_library_2023.maegz:501	Z228587394	194.117	-2.737
DSI_poised_library_2023.maegz:256	Z166645986	142.111	-2.736
DSI_poised_library_2023.maegz:418	Z477328060	142.111	-2.73
DSI_poised_library_2023.maegz:126	Z1342868616	239.131	-2.718
DSI_poised_library_2023.maegz:426	Z56781951	129.115	-2.716
DSI_poised_library_2023.maegz:47	Z1216837642	142.111	-2.711
DSI_poised_library_2023.maegz:355	Z1142623285	171.174	-2.692
DSI_poised_library_2023.maegz:753	Z992569480	192.137	-2.684
DSI_poised_library_2023.maegz:635	Z90664455	142.111	-2.68
DSI_poised_library_2023.maegz:348	Z1198172663	177.090	-2.679
DSI_poised_library_2023.maegz:652	Z235341991	230.082	-2.663
DSI_poised_library_2023.maegz:217	Z220816104	170.106	-2.659
DSI_poised_library_2023.maegz:332	Z1562155982	206.119	-2.657

DSI_poised_library_2023.maegz:282	Z1888396049	211.064	-2.656
DSI_poised_library_2023.maegz:563	Z2737076969	241.067	-2.636
DSI_poised_library_2023.maegz:627	Z1262246195	232.059	-2.619
DSI_poised_library_2023.maegz:754	Z1324853681	165.090	-2.618
DSI_poised_library_2023.maegz:142	Z1757321532	244.109	-2.611
DSI_poised_library_2023.maegz:603	Z1020729274	170.142	-2.604
DSI_poised_library_2023.maegz:599	Z439698382	142.111	-2.599
DSI_poised_library_2023.maegz:433	Z1741959530	197.105	-2.571
DSI_poised_library_2023.maegz:426	Z56781951	129.115	-2.56
DSI_poised_library_2023.maegz:650	Z854097264	144.090	-2.558
DSI_poised_library_2023.maegz:343	Z1741966630	142.147	-2.55
DSI_poised_library_2023.maegz:217	Z220816104	170.106	-2.534
DSI_poised_library_2023.maegz:433	Z1741959530	197.105	-2.529
DSI_poised_library_2023.maegz:355	Z1142623285	171.174	-2.525
DSI_poised_library_2023.maegz:501	Z228587394	194.117	-2.522
DSI_poised_library_2023.maegz:827	Z1758466556	212.144	-2.499
DSI_poised_library_2023.maegz:553	Z56960248	179.095	-2.481
DSI_poised_library_2023.maegz:47	Z1216837642	142.111	-2.473
DSI_poised_library_2023.maegz:284	Z431555038	144.126	-2.467
DSI_poised_library_2023.maegz:614	Z439694282	158.106	-2.459
DSI_poised_library_2023.maegz:599	Z439698382	142.111	-2.44
DSI_poised_library_2023.maegz:754	Z1324853681	165.090	-2.427
DSI_poised_library_2023.maegz:650	Z854097264	144.090	-2.416
DSI_poised_library_2023.maegz:343	Z1741966630	142.147	-2.416
DSI_poised_library_2023.maegz:782	Z513800486	150.116	-2.415
DSI_poised_library_2023.maegz:47	Z1216837642	142.111	-2.408
DSI_poised_library_2023.maegz:713	Z57102475	167.095	-2.405
DSI_poised_library_2023.maegz:635	Z90664455	142.111	-2.403
DSI_poised_library_2023.maegz:666	Z166605480	189.072	-2.387
DSI_poised_library_2023.maegz:635	Z90664455	142.111	-2.384
DSI_poised_library_2023.maegz:808	Z803064328	282.101	-2.378
DSI_poised_library_2023.maegz:343	Z1741966630	142.147	-2.371
DSI_poised_library_2023.maegz:614	Z439694282	158.106	-2.371
DSI_poised_library_2023.maegz:208	Z56960214	189.077	-2.359
DSI_poised_library_2023.maegz:512	Z871834402	225.130	-2.358
DSI_poised_library_2023.maegz:500	Z271007874	144.126	-2.328
DSI_poised_library_2023.maegz:343	Z1741966630	142.147	-2.328
DSI_poised_library_2023.maegz:783	Z425662036	139.111	-2.325
DSI_poised_library_2023.maegz:713	Z57102475	167.095	-2.319
DSI_poised_library_2023.maegz:713	Z57102475	167.095	-2.309
DSI_poised_library_2023.maegz:527	Z364013242	156.126	-2.291
DSI_poised_library_2023.maegz:645	Z234898257	162.091	-2.282
DSI_poised_library_2023.maegz:486	Z57390007	122.084	-2.262

DSI_poised_library_2023.maegz:650	Z854097264	144.090	-2.252
DSI_poised_library_2023.maegz:527	Z364013242	156.126	-2.252
DSI_poised_library_2023.maegz:795	Z426041412	236.039	-2.235
DSI_poised_library_2023.maegz:7	Z1013740522	191.098	-2.23
DSI_poised_library_2023.maegz:527	Z364013242	156.126	-2.23
DSI_poised_library_2023.maegz:500	Z271007874	144.126	-2.183
DSI_poised_library_2023.maegz:500	Z271007874	144.126	-2.171
DSI_poised_library_2023.maegz:355	Z1142623285	171.174	-2.151
DSI_poised_library_2023.maegz:300	Z2574937229	249.092	-2.146
DSI_poised_library_2023.maegz:500	Z271007874	144.126	-2.145
DSI_poised_library_2023.maegz:500	Z271007874	144.126	-2.139
DSI_poised_library_2023.maegz:7	Z1013740522	191.098	-2.135
DSI_poised_library_2023.maegz:154	Z285141528	192.093	-2.131
DSI_poised_library_2023.maegz:47	Z1216837642	142.111	-2.118
DSI_poised_library_2023.maegz:526	Z317064394	170.142	-2.118
DSI_poised_library_2023.maegz:699	Z1493056027	178.078	-2.11
DSI_poised_library_2023.maegz:804	Z1491215378	214.054	-2.103
DSI_poised_library_2023.maegz:500	Z271007874	144.126	-2.101
DSI_poised_library_2023.maegz:699	Z1493056027	178.078	-2.094
DSI_poised_library_2023.maegz:650	Z854097264	144.090	-2.081
DSI_poised_library_2023.maegz:804	Z1491215378	214.054	-2.074
DSI_poised_library_2023.maegz:650	Z854097264	144.090	-2.046
DSI_poised_library_2023.maegz:219	Z270760338	157.122	-2.041
DSI_poised_library_2023.maegz:47	Z1216837642	142.111	-2.037
DSI_poised_library_2023.maegz:512	Z871834402	225.130	-2.037
DSI_poised_library_2023.maegz:47	Z1216837642	142.111	-2.008
DSI_poised_library_2023.maegz:782	Z513800486	150.116	-2.002
DSI_poised_library_2023.maegz:804	Z1491215378	214.054	-2.001
DSI_poised_library_2023.maegz:795	Z426041412	236.039	-1.997
DSI_poised_library_2023.maegz:486	Z57390007	122.084	-1.98
DSI_poised_library_2023.maegz:47	Z1216837642	142.111	-1.959
DSI_poised_library_2023.maegz:486	Z57390007	122.084	-1.956
DSI_poised_library_2023.maegz:512	Z871834402	225.130	-1.954
DSI_poised_library_2023.maegz:7	Z1013740522	191.098	-1.937
DSI_poised_library_2023.maegz:645	Z234898257	162.091	-1.892
DSI_poised_library_2023.maegz:154	Z285141528	192.093	-1.885
DSI_poised_library_2023.maegz:47	Z1216837642	142.111	-1.883
DSI_poised_library_2023.maegz:47	Z1216837642	142.111	-1.881
DSI_poised_library_2023.maegz:355	Z1142623285	171.174	-1.872
DSI_poised_library_2023.maegz:47	Z1216837642	142.111	-1.872
DSI_poised_library_2023.maegz:782	Z513800486	150.116	-1.849
DSI_poised_library_2023.maegz:154	Z285141528	192.093	-1.848
DSI_poised_library_2023.maegz:198	Z1272684692	199.043	-1.81

DSI_poised_library_2023.maegz:526	Z317064394	170.142	-1.742
DSI_poised_library_2023.maegz:645	Z234898257	162.091	-1.731
DSI_poised_library_2023.maegz:276	Z169506466	191.056	-1.639
DSI_poised_library_2023.maegz:699	Z1493056027	178.078	-1.578

Table S20. VS1 and VS2 for PDB fragment library targeting MRE11 dimer

Compound	MW	Energy in silico
VS1		
6-Acetamido-6-Deoxy-Castanospermine	230.127	-6.2164
VS2		
6-Acetamido-6-Deoxy-Castanospermine	230.127	-6.4068
L-tryptophanamide	203.106	-6.364
octahydroindolizine-1,6,7,8-tetrol	189.100	-6.1916
6-Acetamido-6-Deoxy-Castanospermine	230.127	-6.1817
octahydroindolizine-1,6,7,8-tetrol	189.100	-6.1401
octahydroindolizine-1,6,7,8-tetrol	189.100	-6.075
octahydroindolizine-1,6,7,8-tetrol	189.100	-6.0528
octahydroindolizine-1,6,7,8-tetrol	189.100	-6.043
octahydroindolizine-1,6,7,8-tetrol	189.100	-6.0272
octahydroindolizine-1,6,7,8-tetrol	189.100	-6.0144
octahydroindolizine-1,6,7,8-tetrol	189.100	-5.9927
octahydroindolizine-1,6,7,8-tetrol	189.100	-5.9716
1-Deoxy-L-rhamnojirimycin	147.090	-5.9668
octahydroindolizine-1,6,7,8-tetrol	189.100	-5.9541
octahydroindolizine-1,6,7,8-tetrol	189.100	-5.9275
octahydroindolizine-1,6,7,8-tetrol	189.100	-5.8854
1-Deoxy-L-rhamnojirimycin	147.090	-5.8662
octahydroindolizine-1,6,7,8-tetrol	189.100	-5.8645
L-tryptophanamide	203.106	-5.851
octahydroindolizine-1,6,7,8-tetrol	189.100	-5.8499
L-tryptophanamide	203.106	-5.8444
octahydroindolizine-1,6,7,8-tetrol	189.100	-5.8439
octahydroindolizine-1,6,7,8-tetrol	189.100	-5.8165
octahydroindolizine-1,6,7,8-tetrol	189.100	-5.8125
octahydroindolizine-1,6,7,8-tetrol	189.100	-5.8121
octahydroindolizine-1,6,7,8-tetrol	189.100	-5.8057
octahydroindolizine-1,6,7,8-tetrol	189.100	-5.8042
octahydroindolizine-1,6,7,8-tetrol	189.100	-5.804
octahydroindolizine-1,6,7,8-tetrol	189.100	-5.7877
Tryptamine	160.100	-5.7815
octahydroindolizine-1,6,7,8-tetrol	189.100	-5.7809

octahydroindolizine-1,6,7,8-tetrol	189.100	-5.7804
octahydroindolizine-1,6,7,8-tetrol	189.100	-5.7791
octahydroindolizine-1,6,7,8-tetrol	189.100	-5.7574
octahydroindolizine-1,6,7,8-tetrol	189.100	-5.7537
octahydroindolizine-1,6,7,8-tetrol	189.100	-5.7488
octahydroindolizine-1,6,7,8-tetrol	189.100	-5.7414
octahydroindolizine-1,6,7,8-tetrol	189.100	-5.7318
Migalastat	163.084	-5.7165
octahydroindolizine-1,6,7,8-tetrol	189.100	-5.7165
6-Acetamido-6-Deoxy-Castanospermine	230.127	-5.7141
octahydroindolizine-1,6,7,8-tetrol	189.100	-5.6961
octahydroindolizine-1,6,7,8-tetrol	189.100	-5.695
octahydroindolizine-1,6,7,8-tetrol	189.100	-5.6928
octahydroindolizine-1,6,7,8-tetrol	189.100	-5.679
1-Deoxy-L-rhamnojirimycin	147.090	-5.6744
octahydroindolizine-1,6,7,8-tetrol	189.100	-5.6707
1-Deoxy-L-rhamnojirimycin	147.090	-5.6636
6-Acetamido-6-Deoxy-Castanospermine	230.127	-5.6573
Migalastat	163.084	-5.6531
octahydroindolizine-1,6,7,8-tetrol	189.100	-5.652
octahydroindolizine-1,6,7,8-tetrol	189.100	-5.6388
octahydroindolizine-1,6,7,8-tetrol	189.100	-5.6366
Migalastat	163.084	-5.6351
Serotonin	176.095	-5.6338
octahydroindolizine-1,6,7,8-tetrol	189.100	-5.6324
octahydroindolizine-1,6,7,8-tetrol	189.100	-5.6309
octahydroindolizine-1,6,7,8-tetrol	189.100	-5.6275
octahydroindolizine-1,6,7,8-tetrol	189.100	-5.6084
1-Deoxy-L-rhamnojirimycin	147.090	-5.6083
octahydroindolizine-1,6,7,8-tetrol	189.100	-5.5992
octahydroindolizine-1,6,7,8-tetrol	189.100	-5.599
octahydroindolizine-1,6,7,8-tetrol	189.100	-5.5963
octahydroindolizine-1,6,7,8-tetrol	189.100	-5.5948
octahydroindolizine-1,6,7,8-tetrol	189.100	-5.5938
octahydroindolizine-1,6,7,8-tetrol	189.100	-5.5918
1-Deoxy-L-rhamnojirimycin	147.090	-5.5887
1-Deoxy-L-rhamnojirimycin	147.090	-5.581
octahydroindolizine-1,6,7,8-tetrol	189.100	-5.578
octahydroindolizine-1,6,7,8-tetrol	189.100	-5.5759
1-Deoxy-L-rhamnojirimycin	147.090	-5.567
1-Deoxy-L-rhamnojirimycin	147.090	-5.5661
2-Acetamido-1,2-Dideoxynojirmycin	204.111	-5.5598
octahydroindolizine-1,6,7,8-tetrol	189.100	-5.5558

1-Deoxy-L-rhamnojirimycin	147.090	-5.5537
octahydroindolizine-1,6,7,8-tetrol	189.100	-5.545
octahydroindolizine-1,6,7,8-tetrol	189.100	-5.5443
octahydroindolizine-1,6,7,8-tetrol	189.100	-5.542
1-Deoxy-L-rhamnojirimycin	147.090	-5.5419
1-Deoxy-L-rhamnojirimycin	147.090	-5.5261
1-butyl-2-(hydroxymethyl)piperidine-3,4,5-triol	219.147	-5.5253
1-Deoxy-L-rhamnojirimycin	147.090	-5.5237
1-butyl-2-(hydroxymethyl)piperidine-3,4,5-triol	219.147	-5.5236
2-Acetamido-1,2-Dideoxynojirmycin	204.111	-5.5213
1-Deoxy-L-rhamnojirimycin	147.090	-5.5153
Serotonin	176.095	-5.5073
octahydroindolizine-1,6,7,8-tetrol	189.100	-5.5054
1-butyl-2-(hydroxymethyl)piperidine-3,4,5-triol	219.147	-5.4998
MANNOImidazole	200.080	-5.4971
1-Deoxymannojirimycin	163.084	-5.4921
octahydroindolizine-1,6,7,8-tetrol	189.100	-5.4853
1-Deoxy-L-rhamnojirimycin	147.090	-5.4835
1-butyl-2-(hydroxymethyl)piperidine-3,4,5-triol	219.147	-5.4821
1-Deoxy-L-rhamnojirimycin	147.090	-5.479
octahydroindolizine-1,6,7,8-tetrol	189.100	-5.4761
octahydroindolizine-1,6,7,8-tetrol	189.100	-5.4733
N-(2-aminoethyl)isoquinoline-5-sulfonamide	251.073	-5.4681
octahydroindolizine-1,6,7,8-tetrol	189.100	-5.4647
octahydroindolizine-1,6,7,8-tetrol	189.100	-5.4621
octahydroindolizine-1,6,7,8-tetrol	189.100	-5.4548
1-Deoxy-L-rhamnojirimycin	147.090	-5.4525
1-Deoxy-L-rhamnojirimycin	147.090	-5.4491
octahydroindolizine-1,6,7,8-tetrol	189.100	-5.449
octahydroindolizine-1,6,7,8-tetrol	189.100	-5.4451
octahydroindolizine-1,6,7,8-tetrol	189.100	-5.443
1-Deoxy-L-rhamnojirimycin	147.090	-5.4423
1-butyl-2-(hydroxymethyl)piperidine-3,4,5-triol	219.147	-5.4416
octahydroindolizine-1,6,7,8-tetrol	189.100	-5.4391
octahydroindolizine-1,6,7,8-tetrol	189.100	-5.439
octahydroindolizine-1,6,7,8-tetrol	189.100	-5.4362
octahydroindolizine-1,6,7,8-tetrol	189.100	-5.4351
1-butyl-2-(hydroxymethyl)piperidine-3,4,5-triol	219.147	-5.4221
1-Deoxy-L-rhamnojirimycin	147.090	-5.4209
1-Deoxymannojirimycin	163.084	-5.4139
octahydroindolizine-1,6,7,8-tetrol	189.100	-5.3934
tyrosinamide	180.090	-5.3906
tyrosinamide	180.090	-5.3895

octahydroindolizine-1,6,7,8-tetrol	189.100	-5.3892
6-methyltetrahydropterin	181.096	-5.3876
1-Deoxy-L-rhamnojirimycin	147.090	-5.3864
1-butyl-2-(hydroxymethyl)piperidine-3,4,5-triol	219.147	-5.3829
1-Deoxy-L-rhamnojirimycin	147.090	-5.3818
1-Deoxy-L-rhamnojirimycin	147.090	-5.3807
1-Deoxy-L-rhamnojirimycin	147.090	-5.3789
2-Acetamido-1,2-Dideoxynojirmycin	204.111	-5.3741
octahydroindolizine-1,6,7,8-tetrol	189.100	-5.3708
octahydroindolizine-1,6,7,8-tetrol	189.100	-5.3671
1-Deoxy-L-rhamnojirimycin	147.090	-5.3574
1-Deoxy-L-rhamnojirimycin	147.090	-5.3567
octahydroindolizine-1,6,7,8-tetrol	189.100	-5.3526
1-Deoxy-L-rhamnojirimycin	147.090	-5.3523
octahydroindolizine-1,6,7,8-tetrol	189.100	-5.3498
1-butyl-2-(hydroxymethyl)piperidine-3,4,5-triol	219.147	-5.3495
1-Deoxy-L-rhamnojirimycin	147.090	-5.3437
1-butyl-2-(hydroxymethyl)piperidine-3,4,5-triol	219.147	-5.3427
1-Deoxy-L-rhamnojirimycin	147.090	-5.3366
octahydroindolizine-1,6,7,8-tetrol	189.100	-5.3312
1-Deoxy-L-rhamnojirimycin	147.090	-5.326
octahydroindolizine-1,6,7,8-tetrol	189.100	-5.3257
1-Deoxy-L-rhamnojirimycin	147.090	-5.3255
tyrosinamide	180.090	-5.3228
octahydroindolizine-1,6,7,8-tetrol	189.100	-5.3197
octahydroindolizine-1,6,7,8-tetrol	189.100	-5.3187
Serotonin	176.095	-5.3164
1-Deoxymannojirimycin	163.084	-5.3143
octahydroindolizine-1,6,7,8-tetrol	189.100	-5.3141
Resveratrol	228.079	-5.3032
1-butyl-2-(hydroxymethyl)piperidine-3,4,5-triol	219.147	-5.296
octahydroindolizine-1,6,7,8-tetrol	189.100	-5.2943
octahydroindolizine-1,6,7,8-tetrol	189.100	-5.2939
1-butyl-2-(hydroxymethyl)piperidine-3,4,5-triol	219.147	-5.2937
octahydroindolizine-1,6,7,8-tetrol	189.100	-5.2845
1-Deoxy-L-rhamnojirimycin	147.090	-5.2835
N-(2-aminoethyl)isoquinoline-5-sulfonamide	251.073	-5.2774
1-Deoxy-L-rhamnojirimycin	147.090	-5.2771
1-butyl-2-(hydroxymethyl)piperidine-3,4,5-triol	219.147	-5.2763
octahydroindolizine-1,6,7,8-tetrol	189.100	-5.2575
6-Acetamido-6-Deoxy-Castanospermine	230.127	-5.2545
1-Deoxy-L-rhamnojirimycin	147.090	-5.2495
6-methyltetrahydropterin	181.096	-5.2421

octahydroindolizine-1,6,7,8-tetrol	189.100	-5.2399
6-methyltetrahydropterin	181.096	-5.2395
octahydroindolizine-1,6,7,8-tetrol	189.100	-5.2289
Histamine	111.080	-5.223
1-Deoxy-L-rhamnojirimycin	147.090	-5.1829
octahydroindolizine-1,6,7,8-tetrol	189.100	-5.1825
1-Deoxy-L-rhamnojirimycin	147.090	-5.1744
octahydroindolizine-1,6,7,8-tetrol	189.100	-5.1728
Tryptamine	160.100	-5.1692
1-Deoxy-L-rhamnojirimycin	147.090	-5.1679
octahydroindolizine-1,6,7,8-tetrol	189.100	-5.1659
1-Deoxy-L-rhamnojirimycin	147.090	-5.1615
2-Naphthimidamide	170.084	-5.1451
Thioguanine	167.027	-5.1352
octahydroindolizine-1,6,7,8-tetrol	189.100	-5.131
1-Deoxy-L-rhamnojirimycin	147.090	-5.1254
1-Deoxy-L-rhamnojirimycin	147.090	-5.1205
6-methyltetrahydropterin	181.096	-5.1108
Isofagomine	147.090	-5.0952
Histamine	111.080	-5.094
Histamine	111.080	-5.0828
1-Deoxy-L-rhamnojirimycin	147.090	-5.0514
Histamine	111.080	-5.0264
1-Deoxy-L-rhamnojirimycin	147.090	-5.0215
Tryptamine	160.100	-5.0103
N-(2-aminoethyl)isoquinoline-5-sulfonamide	251.073	-5.0097
1-Deoxy-L-rhamnojirimycin	147.090	-5.0036
Tyramine	137.084	-4.9549
Tyramine	137.084	-4.9313
Histamine	111.080	-4.8465
2-Naphthimidamide	170.084	-4.8433
Histamine	111.080	-4.7986

Table S21. VS1 and VS2 for LH1 FDA Approved drugs targeting MRE11 dimer

Drug Name	MolWt	Energy in silico
VS1		
Polymyxin B sulfate	1301.560	-11.2277
VS2		
Polymyxin B sulfate	1301.560	-9.4069
Revefenacin	597.750	-8.6705
Revefenacin	597.750	-8.3133

Revefenacin	597.750	-8.1184
Polymyxin B sulfate	1301.560	-8.1147
Felypressin	1040.220	-7.9943
Felypressin	1040.220	-7.9333
Polymyxin B sulfate	1301.560	-7.9229
Revefenacin	597.750	-7.9201
Revefenacin	597.750	-7.9197
Revefenacin	597.750	-7.875
Desmopressin	1069.220	-7.851
Polymyxin B sulfate	1301.560	-7.8265
Isavuconazonium sulfate	814.840	-7.7549
Felypressin	1040.220	-7.747
Revefenacin	597.750	-7.7426
Revefenacin	597.750	-7.6922
Polymyxin B sulfate	1301.560	-7.68
Polymyxin B sulfate	1301.560	-7.6227
Revefenacin	597.750	-7.5655
Polymyxin B sulfate	1301.560	-7.5531
Polymyxin B sulfate	1301.560	-7.5431
Polymyxin B sulfate	1301.560	-7.4458
Goserelin acetate	1329.460	-7.4437
Revefenacin	597.750	-7.4358
Daclatasvir dihydrochloride	811.800	-7.4053
Polymyxin B sulfate	1301.560	-7.3747
Revefenacin	597.750	-7.3555
Daclatasvir dihydrochloride	811.800	-7.3466
Polymyxin B sulfate	1301.560	-7.3463
Goserelin acetate	1329.460	-7.3288
Revefenacin	597.750	-7.2717
Revefenacin	597.750	-7.2478
Goserelin acetate	1329.460	-7.2241
Elbasvir	882.020	-7.2126
Hematoporphyrin dihydrochloride	671.610	-7.2059
Lafutidine	431.550	-7.1644
Atracurium besylate	1243.490	-7.1634
Ledipasvir acetone	947.080	-7.1575
Esomeprazole Magnesium	713.120	-7.1561
Esomeprazole Sodium	367.400	-7.148
Polymyxin B sulfate	1301.560	-7.1383
Goserelin acetate	1329.460	-7.1232
Goserelin acetate	1329.460	-7.1103
Deferoxamine Mesylate	656.790	-7.1062
Daclatasvir dihydrochloride	811.800	-7.0992

Daclatasvir dihydrochloride	811.800	-7.0897
Isavuconazonium sulfate	814.840	-7.0863
Esomeprazole Sodium	367.400	-7.0854
Daclatasvir dihydrochloride	811.800	-7.0783
Elbasvir	882.020	-7.0712
Vancomycin hydrochloride	1485.710	-7.0559
Mitoxantrone hydrochloride	517.400	-7.0152
Mitoxantrone	444.480	-7.0134
Elbasvir	882.020	-7.0029
Atracurium besylate	1243.490	-6.9962
Desmopressin	1069.220	-6.9687
Carbetocin	988.160	-6.9451
Pentamidine isethionate	592.680	-6.9409
Esomeprazole Magnesium	713.120	-6.9348
Ademetionine	398.440	-6.9241
Daclatasvir	738.880	-6.9152
Mitoxantrone hydrochloride	517.400	-6.9114
Mitoxantrone	444.480	-6.9096
Tilorone dihydrochloride	483.470	-6.8782
Travoprost	500.550	-6.8727
Ademetionine	398.440	-6.8711
Desmopressin	1069.220	-6.8707
Tilorone dihydrochloride	483.470	-6.8705
Pentamidine isethionate	592.680	-6.8587
Carfilzomib	719.910	-6.8431
Esomeprazole Sodium	367.400	-6.8419
Capastat sulfate	752.760	-6.84
Indinavir sulfate	711.870	-6.8384
Ademetionine	398.440	-6.8358
Ledipasvir acetone	947.080	-6.823
Capastat sulfate	752.760	-6.7992
Capastat sulfate	752.760	-6.7977
Omarigliptin	398.430	-6.7925
Ademetionine	398.440	-6.7778
Indinavir sulfate	711.870	-6.7736
Felypressin	1040.220	-6.7525
Mitoxantrone	444.480	-6.6793
Mitoxantrone hydrochloride	517.400	-6.6787
Indinavir sulfate	711.870	-6.6665
Indinavir sulfate	711.870	-6.6664
Troxerutin	742.670	-6.6639
Esomeprazole Magnesium	713.120	-6.6622
Capastat sulfate	752.760	-6.651

Diminazene Aceturate	515.520	-6.6469
Tilorone dihydrochloride	483.470	-6.6411
Ademetionine	398.440	-6.6335
Isavuconazonium sulfate	814.840	-6.6307
Isavuconazonium sulfate	814.840	-6.6243
Ledipasvir acetone	947.080	-6.6146
Lapatinib ditosylate monohydrate	943.470	-6.6117
Isavuconazonium sulfate	814.840	-6.6094
Lapatinib Ditosylate	925.460	-6.6093
Lapatinib	581.060	-6.6093
Carbetocin	988.160	-6.6062
Sisomicin Sulfate	1385.430	-6.6061
Capastat sulfate	752.760	-6.6033
Sisomicin Sulfate	1385.430	-6.5842
Capastat sulfate	752.760	-6.5793
Daclatasvir	738.880	-6.5781
Polymyxin B sulfate	1301.560	-6.5754
Lafutidine	431.550	-6.5707
Elbasvir	882.020	-6.5642
Indinavir sulfate	711.870	-6.559
Capastat sulfate	752.760	-6.553
Sisomicin Sulfate	1385.430	-6.541
Indinavir sulfate	711.870	-6.5408
Indinavir sulfate	711.870	-6.5288
Indinavir sulfate	711.870	-6.5278
Omarigliptin	398.430	-6.5247
Deferoxamine Mesylate	656.790	-6.5167
Goserelin acetate	1329.460	-6.5138
Ademetionine	398.440	-6.509
Amphotericin B	924.080	-6.5043
Ademetionine	398.440	-6.5037
Mebeverine hydrochloride	466.010	-6.4956
Felypressin	1040.220	-6.492
Indinavir sulfate	711.870	-6.4806
Travoprost	500.550	-6.4727
Micafungin sodium	1292.260	-6.4713
Amphotericin B	924.080	-6.4601
Isavuconazonium sulfate	814.840	-6.4573
Omarigliptin	398.430	-6.4547
Naringin	580.530	-6.4535
Ledipasvir acetone	947.080	-6.4426
Polymyxin B sulfate	1301.560	-6.4406
Capastat sulfate	752.760	-6.4353

Isavuconazonium sulfate	814.840	-6.4315
Anidulafungin	1140.240	-6.4314
Pentamidine isethionate	592.680	-6.4182
Ademetionine	398.440	-6.4177
Carvedilol	406.470	-6.4172
Amphotericin B	924.080	-6.4148
Carvedilol phosphate	522.490	-6.4139
Ademetionine	398.440	-6.3986
Travoprost	500.550	-6.3962
Daclatasvir dihydrochloride	811.800	-6.3915
Indinavir sulfate	711.870	-6.388
Troxerutin	742.670	-6.3852
Rebeprazole sodium	381.420	-6.3803
Omarigliptin	398.430	-6.3708
Indinavir sulfate	711.870	-6.3643
Rebeprazole sodium	381.420	-6.3637
Indinavir sulfate	711.870	-6.3626
Indinavir sulfate	711.870	-6.3557
Polymyxin B sulfate	1301.560	-6.3489
Tobramycin	467.510	-6.3353
Haloperidol	375.870	-6.3339
bremelanotide	1085.220	-6.3337
Indinavir sulfate	711.870	-6.3302
Dihydrostreptomycin sulfate	730.710	-6.3253
Capastat sulfate	752.760	-6.3247
bremelanotide	1085.220	-6.3204
Capastat sulfate	752.760	-6.3121
Atracurium besylate	1243.490	-6.3044
Lafutidine	431.550	-6.2995
Rebeprazole sodium	381.420	-6.295
Deferoxamine Mesylate	656.790	-6.289
Polymyxin B sulfate	1301.560	-6.2864
Lafutidine	431.550	-6.2801
Revefenacin	597.750	-6.278
Isavuconazonium sulfate	814.840	-6.2732
Polymyxin B sulfate	1301.560	-6.2721
Isepamicin sulfate	667.680	-6.2713
Mebeverine hydrochloride	466.010	-6.2701
Mebeverine hydrochloride	466.010	-6.2677
Lafutidine	431.550	-6.2637
Hydroxychloroquine sulfate	433.950	-6.2541
Isepamicin sulfate	667.680	-6.2519
Daclatasvir	738.880	-6.2472

Troxerutin	742.670	-6.2447
Daclatasvir	738.880	-6.2352
Daclatasvir	738.880	-6.2328
Daclatasvir dihydrochloride	811.800	-6.2291
bremelanotide	1085.220	-6.2172
Indinavir sulfate	711.870	-6.2107
Mebeverine hydrochloride	466.010	-6.2096
Travoprost	500.550	-6.2093
Paromomycin Sulfate	713.710	-6.2065
Travoprost	500.550	-6.1973
Aliskiren hemifumarate	1219.600	-6.1922
Pantoprazole Sodium Hydrate	432.370	-6.1891
Haloperidol	375.870	-6.1879
Ilaprazole sodium	388.440	-6.1876
Indinavir sulfate	711.870	-6.1868
Felypressin	1040.220	-6.1811
Thymopentin	679.560	-6.1786
Capastat sulfate	752.760	-6.1765
Streptomycin sulfate	1457.380	-6.1752
Nifekalant hydrochloride	441.910	-6.163
Ademetionine	398.440	-6.1616
Travoprost	500.550	-6.1575
Dequalinium chloride	527.590	-6.157
Ademetionine	398.440	-6.1436
Tobramycin	467.510	-6.142
Indinavir sulfate	711.870	-6.133
Posaconazole	700.780	-6.1314
Ademetionine	398.440	-6.1296
Vilazodone HCl	477.990	-6.1232
Isavuconazonium sulfate	814.840	-6.1229
Indinavir sulfate	711.870	-6.1222
Streptomycin sulfate	1457.380	-6.1222
Isavuconazonium sulfate	814.840	-6.1167
Ademetionine	398.440	-6.1164
Isavuconazonium sulfate	814.840	-6.1137
Fipexide	388.850	-6.1095
Esomeprazole Magnesium	713.120	-6.1043
Proanthocyanidins	594.520	-6.1024
Travoprost	500.550	-6.1019
Trazodone hydrochloride	408.330	-6.101
Darifenacin HBr	505.450	-6.0989
Thymopentin	679.560	-6.0981
Micafungin sodium	1292.260	-6.0968

(S)-crizotinib	450.340	-6.0909
Ademetionine	398.440	-6.0852
Indinavir sulfate	711.870	-6.0798
Darifenacin HBr	505.450	-6.0791
Ethacridine lactate	343.380	-6.0786
Polymyxin B sulfate	1301.560	-6.071
Spectinomycin dihydrochloride	405.270	-6.0689
Ademetionine	398.440	-6.0672
Ademetionine	398.440	-6.0663
Travoprost	500.550	-6.0614
Indinavir sulfate	711.870	-6.0612
Indinavir sulfate	711.870	-6.0593
Daclatasvir	738.880	-6.0577
(S)-crizotinib	450.340	-6.0533
Indinavir sulfate	711.870	-6.052
Ranolazine	427.540	-6.0505
Ranolazine dihydrochloride	500.460	-6.0505
Levomefolate calcium	497.520	-6.0495
Fipexide	388.850	-6.0495
Ademetionine	398.440	-6.043
Polymyxin B sulfate	1301.560	-6.0369
Mebeverine hydrochloride	466.010	-6.0368
Indinavir sulfate	711.870	-6.0342
Ademetionine	398.440	-6.0341
Palbociclib Isethionate	573.660	-6.0275
Palbociclib hydrochloride	483.990	-6.0275
Travoprost	500.550	-6.0272
Palbociclib	447.540	-6.0236
Vilazodone	441.530	-6.0221
Isavuconazonium sulfate	814.840	-6.0213
Indinavir sulfate	711.870	-6.0195
Rotigotine	315.470	-6.0165
Capastat sulfate	752.760	-6.0151
Ademetionine	398.440	-6.0122
Pixantrone dimaleate	557.210	-6.0114
Esomeprazole Sodium	367.400	-6.0079
Omeprazole	345.420	-6.0079
Ethacridine lactate monohydrate	361.400	-6.0075
Esomeprazole Magnesium	713.120	-6.0075
Indinavir sulfate	711.870	-6.0017
Lansoprazole	369.360	-5.9993
Cefotiam Hexetil Hydrochloride	768.765	-5.9989
Ademetionine	398.440	-5.9948

Hygromycin B	527.520	-5.9924
Travoprost	500.550	-5.9911
Lapatinib Ditosylate	925.460	-5.9899
Lapatinib	581.060	-5.9899
Lapatinib ditosylate monohydrate	943.470	-5.989
Relugolix	623.630	-5.9888
Mebeverine hydrochloride	466.010	-5.9874
Levomefolate calcium	497.520	-5.9865
Proanthocyanidins	594.520	-5.9846
Plerixafor 8HCl (AMD3100 8HCl)	794.460	-5.984
Plerixafor	502.780	-5.984
Crizotinib	450.340	-5.9836
Crizotinib	450.340	-5.9823
Ademetionine	398.440	-5.9792
Indinavir sulfate	711.870	-5.9687
Tobramycin	467.510	-5.9657
(-)-Sparteine sulfate pentahydrate	422.540	-5.9615
Travoprost	500.550	-5.9608
Indinavir sulfate	711.870	-5.9593
Capastat sulfate	752.760	-5.9584
Dacarbazine	182.180	-5.9578
Isavuconazonium sulfate	814.840	-5.9578
Indinavir sulfate	711.870	-5.9577
Relugolix	623.630	-5.9564
Donepezil HCl	415.960	-5.9558
Penfluridol	523.970	-5.9558
Hygromycin B	527.520	-5.9553
Donepezil	379.500	-5.9549
Capastat sulfate	752.760	-5.9538
Pentagastrin	767.890	-5.9464
Lapatinib Ditosylate	925.460	-5.9458
Lapatinib	581.060	-5.9458
Lapatinib ditosylate monohydrate	943.470	-5.9452
Prucalopride	367.870	-5.9441
Chloroquine diphosphate	515.870	-5.9426
Indinavir sulfate	711.870	-5.9413
Famprofazone	377.520	-5.9374
Fendiline hydrochloride	351.910	-5.9371
Ademetionine	398.440	-5.937
Netilmicin sulfate	720.780	-5.9361
Indinavir sulfate	711.870	-5.936
Spectinomycin dihydrochloride	405.270	-5.9359
Silymarin	482.440	-5.9349

Relugolix	623.630	-5.9336
Vilazodone HCl	477.990	-5.9289
Indinavir sulfate	711.870	-5.9284
Tobramycin	467.510	-5.9265
Spectinomycin dihydrochloride	405.270	-5.9253
Dacarbazine	182.180	-5.9202
Raloxifene hydrochloride	510.040	-5.9179
Saxagliptin hydrate	333.430	-5.9162
Miglitol	207.220	-5.9152
Indinavir sulfate	711.870	-5.9119
Capastat sulfate	752.760	-5.909
(S)-crizotinib	450.340	-5.9061
Gentamycin sulfate	1506.800	-5.906
Goserelin acetate	1329.460	-5.906
Proanthocyanidins	594.520	-5.9059
Pantoprazole Sodium Hydrate	432.370	-5.9028
Indinavir sulfate	711.870	-5.9018
Polymyxin B sulfate	1301.560	-5.9005
Penfluridol	523.970	-5.9
Indinavir sulfate	711.870	-5.8985
Amikacin	585.600	-5.8982
Gentamycin sulfate	1506.800	-5.8934
Diminazene Aceturate	515.520	-5.8932
Dacarbazine	182.180	-5.8907
Ademetionine	398.440	-5.8874
Anlotinib Dihydrochloride	480.360	-5.886
Anlotinib	407.440	-5.886
Haloperidol	375.870	-5.8829
Penfluridol	523.970	-5.8815
Fendiline hydrochloride	351.910	-5.8803
Indinavir sulfate	711.870	-5.8791
Proanthocyanidins	594.520	-5.8789
Ademetionine	398.440	-5.8759
Thymopentin	679.560	-5.8753
Micafungin sodium	1292.260	-5.8634
Gentamycin sulfate	1506.800	-5.8633
Indinavir sulfate	711.870	-5.8619
Thymopentin	679.560	-5.8615
Hygromycin B	527.520	-5.8608
Indinavir sulfate	711.870	-5.8589
Pixantrone dimaleate	557.210	-5.8507
Indinavir sulfate	711.870	-5.8504
Crizotinib	450.340	-5.8488

Amikacin Sulfate Salt	683.680	-5.847
Indinavir sulfate	711.870	-5.8433
Dofetilide	441.560	-5.8396
Capastat sulfate	752.760	-5.8391
Silymarin	482.440	-5.8387
Travoprost	500.550	-5.8377
Isoprenaline hydrochloride	247.720	-5.8348
Fipexide	388.850	-5.8317
Indinavir sulfate	711.870	-5.8313
Roxatidine Acetate hydrochloride	384.900	-5.8303
Indinavir sulfate	711.870	-5.8295
Travoprost	500.550	-5.8224
Hygromycin B	527.520	-5.8204
Ademetionine	398.440	-5.818
Carvedilol	406.470	-5.817
Carvedilol phosphate	522.490	-5.8164
Amikacin	585.600	-5.8141
Miglitol	207.220	-5.8129
Silymarin	482.440	-5.8124
Ademetionine	398.440	-5.8122
Esomeprazole Sodium	367.400	-5.8074
Omeprazole	345.420	-5.8074
Dacarbazine	182.180	-5.8048
Brinzolamide	383.510	-5.804
Carvedilol phosphate	522.490	-5.8029
Penfluridol	523.970	-5.8017
Carvedilol	406.470	-5.8014
Dihydrostreptomycin sulfate	730.710	-5.7994
Ethacridine lactate	343.380	-5.7966
Dacarbazine	182.180	-5.7941
Hygromycin B	527.520	-5.7939
Anidulafungin	1140.240	-5.7935
Silymarin	482.440	-5.7913
Travoprost	500.550	-5.7908
Norepinephrine	169.180	-5.7868
Noradrenaline bitartrate	319.260	-5.7868
Ademetionine	398.440	-5.7863
Clindamycin HCl	461.440	-5.7851
Cinepazide maleate	533.570	-5.785
Tobramycin	467.510	-5.7844
Indinavir sulfate	711.870	-5.7839
Dofetilide	441.560	-5.7836
Ademetionine	398.440	-5.7833

Amprolium Hydrochloride	315.240	-5.7818
Pixantrone dimaleate	557.210	-5.7785
Famprofazone	377.520	-5.7785
Ethacridine lactate	343.380	-5.778
Indinavir sulfate	711.870	-5.7774
Ifenprodil Tartrate	400.490	-5.7761
Ademetionine	398.440	-5.7755
Amikacin Sulfate Salt	683.680	-5.7716
Hygromycin B	527.520	-5.7701
Atracurium besylate	1243.490	-5.7696
Tobramycin	467.510	-5.7694
Levomefolate calcium	497.520	-5.7677
Omarigliptin	398.430	-5.766
Donepezil HCl	415.960	-5.7633
Donepezil	379.500	-5.7627
Esomeprazole Sodium	367.400	-5.7619
Omeprazole	345.420	-5.7619
Thymopentin	679.560	-5.7599
Polymyxin B sulfate	1301.560	-5.7585
Aliskiren hemifumarate	1219.600	-5.7567
Indinavir sulfate	711.870	-5.7558
Paromomycin Sulfate	713.710	-5.7558
Travoprost	500.550	-5.753
Reserpine	608.680	-5.7495
Volasertib	618.810	-5.7459
Netilmicin sulfate	720.780	-5.7452
Nelfinavir Mesylate	663.890	-5.7445
Isavuconazonium sulfate	814.840	-5.7428
Ifenprodil Tartrate	400.490	-5.7422
Isepamicin sulfate	667.680	-5.7413
Hydroxychloroquine sulfate	433.950	-5.7397
Ademetionine	398.440	-5.7376
Levomefolate calcium	497.520	-5.7375
Plerixafor 8HCl (AMD3100 8HCl)	794.460	-5.7299
Plerixafor	502.780	-5.7299
Indinavir sulfate	711.870	-5.7298
Hydroxychloroquine sulfate	433.950	-5.7297
Ethacridine lactate monohydrate	361.400	-5.7258
Norepinephrine	169.180	-5.7242
Noradrenaline bitartrate	319.260	-5.7242
Isoprenaline hydrochloride	247.720	-5.722
Proanthocyanidins	594.520	-5.7216
Travoprost	500.550	-5.7203

Proanthocyanidins	594.520	-5.7197
Tobramycin	467.510	-5.7168
Indinavir sulfate	711.870	-5.7165
Carbetocin	988.160	-5.7159
Indinavir sulfate	711.870	-5.7144
Silymarin	482.440	-5.7137
Plerixafor 8HCl (AMD3100 8HCl)	794.460	-5.713
Plerixafor	502.780	-5.713
Omarigliptin	398.430	-5.7105
Hygromycin B	527.520	-5.7071
Proanthocyanidins	594.520	-5.7038
Ethacridine lactate monohydrate	361.400	-5.7018
Levomefolate calcium	497.520	-5.6986
Hydroxychloroquine sulfate	433.950	-5.6962
Proanthocyanidins	594.520	-5.6959
Diminazene Aceturate	515.520	-5.6956
Hydroxychloroquine sulfate	433.950	-5.6956
Goserelin acetate	1329.460	-5.695
Plerixafor 8HCl (AMD3100 8HCl)	794.460	-5.6929
Plerixafor	502.780	-5.6929
Trazodone hydrochloride	408.330	-5.6925
Synephrine	167.210	-5.6908
Donepezil	379.500	-5.689
Donepezil HCl	415.960	-5.6881
(-)-Sparteine sulfate pentahydrate	422.540	-5.6778
Levomefolate calcium	497.520	-5.6749
Silymarin	482.440	-5.6746
Tobramycin	467.510	-5.6743
Famprofazone	377.520	-5.6736
Ifenprodil Tartrate	400.490	-5.6731
Indinavir sulfate	711.870	-5.672
Famprofazone	377.520	-5.6661
Darifenacin HBr	505.450	-5.6603
(-)-Sparteine sulfate pentahydrate	422.540	-5.6574
Indinavir sulfate	711.870	-5.6564
Cefotiam Hexetil Hydrochloride	768.765	-5.6537
Ademetionine	398.440	-5.6519
Miglitol	207.220	-5.6471
Micafungin sodium	1292.260	-5.6443
Indinavir sulfate	711.870	-5.6415
Ademetionine	398.440	-5.6373
Isoprenaline hydrochloride	247.720	-5.6365
Micafungin sodium	1292.260	-5.636

Serotonin hydrochloride	212.680	-5.6332
Sitagliptin	407.320	-5.6317
Mafenide hydrochloride	222.690	-5.629
Travoprost	500.550	-5.6274
Hematoporphyrin dihydrochloride	671.610	-5.6268
Neomycin Sulphate B	712.220	-5.6225
DL-Adrenaline Hydrochloride	219.670	-5.6219
Dihydrostreptomycin sulfate	730.710	-5.619
Diphenidol hydrochloride	345.910	-5.6189
Donepezil	379.500	-5.6178
Neomycin sulfate	908.870	-5.6163
Donepezil HCl	415.960	-5.616
Vancomycin hydrochloride	1485.710	-5.6125
Isoprenaline hydrochloride	247.720	-5.6117
Netilmicin sulfate	720.780	-5.61
Donepezil	379.500	-5.6096
Donepezil HCl	415.960	-5.6096
Atracurium besylate	1243.490	-5.6055
Mafenide hydrochloride	222.690	-5.6013
Isavuconazonium sulfate	814.840	-5.599
Dolasetron	324.380	-5.5965
Hydroxychloroquine sulfate	433.950	-5.595
Levomefolate calcium	497.520	-5.5896
Anidulafungin	1140.240	-5.5853
Rebeprazole sodium	381.420	-5.5847
Troxipide	294.350	-5.5837
Donepezil HCl	415.960	-5.5802
Quinidine hydrochloride	378.890	-5.5793
Atracurium besylate	1243.490	-5.5789
Ademetionine	398.440	-5.5784
Donepezil	379.500	-5.5772
Haloperidol	375.870	-5.5742
Atracurium besylate	1243.490	-5.5739
Synephrine	167.210	-5.5738
Neomycin sulfate	908.870	-5.5733
Chloroquine diphosphate	515.870	-5.5724
Naftifine hydrochloride	323.860	-5.5678
Ademetionine	398.440	-5.5658
Ifenprodil Tartrate	400.490	-5.5596
Pirarubicin	627.640	-5.5567
Plerixafor 8HCl (AMD3100 8HCl)	794.460	-5.556
Plerixafor	502.780	-5.556
Diperodon hydrochloride	433.930	-5.5531

Niraparib tosylate	492.590	-5.5524
Niraparib	320.390	-5.5518
Proanthocyanidins	594.520	-5.5516
Ademetionine	398.440	-5.5442
Neomycin Sulphate B	712.220	-5.5417
Ilaprazole sodium	388.440	-5.5385
Indinavir sulfate	711.870	-5.5354
Silymarin	482.440	-5.5343
Ademetionine	398.440	-5.5323
Proanthocyanidins	594.520	-5.5308
Reserpine	608.680	-5.5296
Eletriptan HBr	463.430	-5.5291
Famprofazone	377.520	-5.5281
LY2835219	602.700	-5.5132
Tobramycin	467.510	-5.5131
Netilmicin sulfate	720.780	-5.5117
Norepinephrine	169.180	-5.5113
Noradrenaline bitartrate	319.260	-5.5113
Levomefolate calcium	497.520	-5.5075
Serotonin hydrochloride	212.680	-5.5052
Levomefolate calcium	497.520	-5.5034
Tacrine hydrochloride hydrate	234.720	-5.5013
Indinavir sulfate	711.870	-5.4983
Cyclovirobuxin D	402.660	-5.4953
Quinidine hydrochloride	378.890	-5.4953
Silymarin	482.440	-5.4946
Indinavir sulfate	711.870	-5.4897
Levomefolate calcium	497.520	-5.4894
Sitagliptin	407.320	-5.4881
Ivabradine hydrochloride	505.050	-5.4856
Dofetilide	441.560	-5.485
Levomefolate calcium	497.520	-5.4825
Levomefolate calcium	497.520	-5.4823
Amikacin	585.600	-5.4782
Amikacin Sulfate Salt	683.680	-5.4735
Proanthocyanidins	594.520	-5.4713
Ifenprodil Tartrate	400.490	-5.4675
Gastrodin	286.280	-5.4662
Etilefrine hydrochloride	217.690	-5.4661
Ademetionine	398.440	-5.4588
Naratriptan	371.930	-5.4573
Proanthocyanidins	594.520	-5.4532
Neomycin sulfate	908.870	-5.4508

Brinzolamide	383.510	-5.4484
Nafamostat mesylate	539.580	-5.4441
Proanthocyanidins	594.520	-5.4439
Boceprevir	519.680	-5.4428
Levomefolate calcium	497.520	-5.44
Cinacalcet hydrochloride	393.870	-5.4369
Levomefolate calcium	497.520	-5.4368
Tobramycin	467.510	-5.4315
Levomefolate calcium	497.520	-5.4306
Casanthranol	396.580	-5.4303
(-)-Sparteine sulfate pentahydrate	422.540	-5.4297
Indinavir sulfate	711.870	-5.4247
Troxipide	294.350	-5.4202
Ademetionine	398.440	-5.4187
Plerixafor 8HCl (AMD3100 8HCl)	794.460	-5.4179
Plerixafor	502.780	-5.4179
Vincristine sulfate	923.040	-5.417
Fendiline hydrochloride	351.910	-5.4137
Proanthocyanidins	594.520	-5.4117
Nafamostat mesylate	539.580	-5.411
Tobramycin	467.510	-5.4106
Ilaprazole sodium	388.440	-5.4047
Sennoside A	862.740	-5.4001
Kanamycin sulfate	582.580	-5.3962
Hygromycin B	527.520	-5.3944
Sennoside A	862.740	-5.3938
(-)-Sparteine sulfate pentahydrate	422.540	-5.3926
Piceatannol	244.240	-5.3922
Casanthranol	396.580	-5.3906
Oseltamivir phosphate	410.400	-5.3833
Rizatriptan benzoate	391.470	-5.383
Proanthocyanidins	594.520	-5.3814
Naftifine hydrochloride	323.860	-5.3787
Cinacalcet hydrochloride	393.870	-5.3734
Indinavir sulfate	711.870	-5.3723
Diphenidol hydrochloride	345.910	-5.3705
Indinavir sulfate	711.870	-5.3681
Indinavir sulfate	711.870	-5.3635
Capastat sulfate	752.760	-5.3598
Levomefolate calcium	497.520	-5.3587
Neomycin sulfate	908.870	-5.3542
Levomefolate calcium	497.520	-5.3527
Kanamycin sulfate	582.580	-5.3479

Neomycin Sulphate B	712.220	-5.342
Famprofazone	377.520	-5.3386
Paromomycin Sulfate	713.710	-5.3354
Valbenazine	418.570	-5.3325
Troxipide	294.350	-5.327
Vilazodone HCl	477.990	-5.3263
Omeprazole	345.420	-5.3255
Cefotiam Hexetil Hydrochloride	768.765	-5.3229
Serotonin hydrochloride	212.680	-5.3212
Etilefrine hydrochloride	217.690	-5.3181
Neomycin Sulphate B	712.220	-5.3178
Paromomycin Sulfate	713.710	-5.3083
Proanthocyanidins	594.520	-5.3075
Indinavir sulfate	711.870	-5.3043
Aloe-emodin	270.240	-5.3043
Lincomycin hydrochloride monohydrate	461.010	-5.3002
Lincomycin hydrochloride	443.000	-5.2975
Casanthranol	396.580	-5.2958
Vilazodone	441.530	-5.2946
Oseltamivir phosphate	410.400	-5.2928
Hematoporphyrin dihydrochloride	671.610	-5.2906
Netilmicin sulfate	720.780	-5.2852
Naratriptan	371.930	-5.2813
Glucosamine hydrochloride	215.630	-5.2761
Kanamycin sulfate	582.580	-5.2761
Naftifine hydrochloride	323.860	-5.2746
Proanthocyanidins	594.520	-5.2725
Isoprenaline hydrochloride	247.720	-5.2705
Glucosamine hydrochloride	215.630	-5.2693
Paromomycin Sulfate	713.710	-5.2675
Bimatoprost	415.570	-5.2649
Netilmicin sulfate	720.780	-5.257
(-)-Sparteine sulfate pentahydrate	422.540	-5.2417
Hematoporphyrin dihydrochloride	671.610	-5.2412
Dorzolamide hydrochloride	360.900	-5.2402
Bimatoprost	415.570	-5.2377
Gastrodin	286.280	-5.2357
Histamine 2HCl	184.070	-5.2344
Histamine	111.150	-5.2344
Etilefrine hydrochloride	217.690	-5.2327
Aminacrine	194.230	-5.2309
Indinavir sulfate	711.870	-5.2297
Cefotiam Hexetil Hydrochloride	768.765	-5.2217

L-Hydroxyprolin	131.130	-5.2184
Naltrexone hydrochloride	377.860	-5.2174
Glucosamine hydrochloride	215.630	-5.1959
Oxiracetam	158.160	-5.195
Casanthranol	396.580	-5.19
Glucosamine hydrochloride	215.630	-5.1774
Methoxamine hydrochloride	247.720	-5.1597
Nalmefene hydrochloride	375.890	-5.1553
DL-Adrenaline Hydrochloride	219.670	-5.1464
Naratriptan	371.930	-5.1423
(-)-Sparteine sulfate pentahydrate	422.540	-5.1422
Methoxamine hydrochloride	247.720	-5.1403
Vancomycin hydrochloride	1485.710	-5.1357
Miglitol	207.220	-5.1344
(-)-Sparteine sulfate pentahydrate	422.540	-5.1342
D-Pantethine	554.720	-5.1316
Pipemidic acid	303.320	-5.1221
Histamine 2HCl	184.070	-5.1051
Histamine	111.150	-5.1051
Naftifine hydrochloride	323.860	-5.1039
L-Hydroxyprolin	131.130	-5.102
Dorzolamide hydrochloride	360.900	-5.0861
Dopamine hydrochloride	189.640	-5.0823
Naftifine hydrochloride	323.860	-5.0761
(-)-Sparteine sulfate pentahydrate	422.540	-5.0718
(-)-Sparteine sulfate pentahydrate	422.540	-5.0707
solriamfetol	194.230	-5.0703
Niraparib	320.390	-5.064
Ademetionine	398.440	-5.0619
Niraparib tosylate	492.590	-5.0598
Histamine 2HCl	184.070	-5.0551
Histamine	111.150	-5.0551
L-5-Hydroxytryptophan	220.220	-5.0485
Capastat sulfate	752.760	-5.0457
Histamine 2HCl	184.070	-5.0354
Histamine	111.150	-5.0354
Proflavine Hemisulfate	516.570	-4.9973
Vancomycin hydrochloride	1485.710	-4.9919
Mafenide hydrochloride	222.690	-4.9872
Mepiroxol	125.130	-4.9564
Lupetidin	113.200	-4.9168
Tranylcypromine (2-PCPA) hydrochloride	169.660	-4.9162
Hematoporphyrin dihydrochloride	671.610	-4.8998

Glucosamine hydrochloride	215.630	-4.8995
(R)-baclofen	213.660	-4.8959
Baclofen	213.660	-4.8959
Sennoside A	862.740	-4.8933
Baclofen	213.660	-4.8877
Histamine 2HCl	184.070	-4.8545
Histamine	111.150	-4.8545
Dopamine hydrochloride	189.640	-4.8301
Histamine 2HCl	184.070	-4.8063
Histamine	111.150	-4.8063
Ribavirin	244.200	-4.7783
Sennoside A	862.740	-4.7221
L-Hydroxyprolin	131.130	-4.7187
Hematoporphyrin dihydrochloride	671.610	-4.7059
Oxaliplatin	397.290	-4.6906
(-)-Sparteine sulfate pentahydrate	422.540	-4.6787
Hematoporphyrin dihydrochloride	671.610	-4.5661
Hematoporphyrin dihydrochloride	671.610	-4.4796
Hematoporphyrin dihydrochloride	671.610	-4.351
Hematoporphyrin dihydrochloride	671.610	-4.2788
Pamidronate disodium salt	279.030	-4.2233

Table S22. VS1 and VS2 for DSI-poised targeting GRB2 dimer

Compound	MW	Catalog ID	Energy in silico
VS1			
DSI_poised_library_2023.maegz:27	242.142	Z26548228	-6.5
VS2			
DSI_poised_library_2023.maegz:245	241.110	Z33452549	-6.9703
DSI_poised_library_2023.maegz:63	299.729	Z1004825200	-6.8837
DSI_poised_library_2023.maegz:547	246.080	Z55290386	-6.8652
DSI_poised_library_2023.maegz:473	249.289	Z1152242726	-6.8173
DSI_poised_library_2023.maegz:271	246.173	Z44585920	-6.8047
DSI_poised_library_2023.maegz:27	242.142	Z26548228	-6.7531
DSI_poised_library_2023.maegz:566	233.085	Z26769872	-6.7396
DSI_poised_library_2023.maegz:271	246.173	Z44585920	-6.7131
DSI_poised_library_2023.maegz:792	242.106	Z728882494	-6.709
DSI_poised_library_2023.maegz:240	247.132	Z2472938267	-6.7038
DSI_poised_library_2023.maegz:792	242.106	Z728882494	-6.6864
DSI_poised_library_2023.maegz:184	238.087	Z46169171	-6.6518
DSI_poised_library_2023.maegz:207	247.168	Z111507846	-6.641
DSI_poised_library_2023.maegz:245	241.110	Z33452549	-6.6356

DSI_poised_library_2023.maegz:402	237.127	Z57299529	-6.6242
DSI_poised_library_2023.maegz:473	249.289	Z1152242726	-6.6198
DSI_poised_library_2023.maegz:102	248.301	Z19750454	-6.5919
DSI_poised_library_2023.maegz:405	213.090	Z44592329	-6.5903
DSI_poised_library_2023.maegz:473	249.289	Z1152242726	-6.5752
DSI_poised_library_2023.maegz:240	247.132	Z2472938267	-6.5625
DSI_poised_library_2023.maegz:509	239.106	Z1587220559	-6.5383
DSI_poised_library_2023.maegz:509	239.106	Z1587220559	-6.531
DSI_poised_library_2023.maegz:792	242.106	Z728882494	-6.5107
DSI_poised_library_2023.maegz:566	233.085	Z26769872	-6.5024
DSI_poised_library_2023.maegz:536	232.278	Z413792090	-6.5005
DSI_poised_library_2023.maegz:739	247.240	Z27805986	-6.4954
DSI_poised_library_2023.maegz:102	248.301	Z19750454	-6.4879
DSI_poised_library_2023.maegz:63	299.729	Z1004825200	-6.4652
DSI_poised_library_2023.maegz:102	248.301	Z19750454	-6.461
DSI_poised_library_2023.maegz:644	243.261	Z321318226	-6.4608
DSI_poised_library_2023.maegz:839	248.301	Z62645406	-6.4455
DSI_poised_library_2023.maegz:313	245.340	Z28429411	-6.4429
DSI_poised_library_2023.maegz:383	245.085	Z19735192	-6.4383
DSI_poised_library_2023.maegz:99	243.261	Z53834613	-6.4265
DSI_poised_library_2023.maegz:583	231.294	Z69091635	-6.4233
DSI_poised_library_2023.maegz:136	234.226	Z44584202	-6.4175
DSI_poised_library_2023.maegz:536	232.278	Z413792090	-6.4125
DSI_poised_library_2023.maegz:405	213.090	Z44592329	-6.3906
DSI_poised_library_2023.maegz:313	245.340	Z28429411	-6.3845
DSI_poised_library_2023.maegz:509	239.106	Z1587220559	-6.3813
DSI_poised_library_2023.maegz:402	237.127	Z57299529	-6.3796
DSI_poised_library_2023.maegz:240	247.132	Z2472938267	-6.3775
DSI_poised_library_2023.maegz:402	237.127	Z57299529	-6.3765
DSI_poised_library_2023.maegz:463	243.221	Z57292378	-6.373
DSI_poised_library_2023.maegz:547	246.080	Z55290386	-6.369
DSI_poised_library_2023.maegz:99	243.261	Z53834613	-6.3673
DSI_poised_library_2023.maegz:217	170.106	Z220816104	-6.3659
DSI_poised_library_2023.maegz:712	193.110	Z235361235	-6.3628
DSI_poised_library_2023.maegz:87	241.288	Z1693429442	-6.3614
DSI_poised_library_2023.maegz:810	244.101	Z606937774	-6.3269
DSI_poised_library_2023.maegz:547	246.080	Z55290386	-6.3121
DSI_poised_library_2023.maegz:383	245.085	Z19735192	-6.3064
DSI_poised_library_2023.maegz:463	243.221	Z57292378	-6.3053
DSI_poised_library_2023.maegz:505	236.310	Z86949053	-6.2861
DSI_poised_library_2023.maegz:107	194.106	Z1563512128	-6.2728
DSI_poised_library_2023.maegz:403	189.115	Z28870646	-6.2611
DSI_poised_library_2023.maegz:719	203.069	Z332370018	-6.2584

DSI_poised_library_2023.maegz:184	238.087	Z46169171	-6.2558
DSI_poised_library_2023.maegz:739	247.240	Z27805986	-6.2544
DSI_poised_library_2023.maegz:778	245.085	Z1250129028	-6.2515
DSI_poised_library_2023.maegz:201	249.119	Z19751622	-6.2258
DSI_poised_library_2023.maegz:708	241.110	Z33452282	-6.2169
DSI_poised_library_2023.maegz:87	241.288	Z1693429442	-6.2152
DSI_poised_library_2023.maegz:505	236.310	Z86949053	-6.2053
DSI_poised_library_2023.maegz:23	226.295	Z1881545321	-6.2048
DSI_poised_library_2023.maegz:405	213.090	Z44592329	-6.1979
DSI_poised_library_2023.maegz:207	247.168	Z111507846	-6.1792
DSI_poised_library_2023.maegz:778	245.085	Z1250129028	-6.1738
DSI_poised_library_2023.maegz:566	233.085	Z26769872	-6.1714
DSI_poised_library_2023.maegz:59	223.112	Z198194394	-6.1693
DSI_poised_library_2023.maegz:403	189.115	Z28870646	-6.1601
DSI_poised_library_2023.maegz:270	234.251	Z1545313172	-6.1554
DSI_poised_library_2023.maegz:136	234.226	Z44584202	-6.1478
DSI_poised_library_2023.maegz:351	241.085	Z26781964	-6.1369
DSI_poised_library_2023.maegz:448	247.677	Z57515803	-6.1355
DSI_poised_library_2023.maegz:663	228.057	Z30932204	-6.132
DSI_poised_library_2023.maegz:662	242.082	Z123852286	-6.1319
DSI_poised_library_2023.maegz:291	237.119	Z29191465	-6.131
DSI_poised_library_2023.maegz:61	237.646	Z56259166	-6.1304
DSI_poised_library_2023.maegz:270	234.251	Z1545313172	-6.1212
DSI_poised_library_2023.maegz:207	247.168	Z111507846	-6.1159
DSI_poised_library_2023.maegz:771	239.225	Z1614545742	-6.1146
DSI_poised_library_2023.maegz:301	247.268	Z1203107138	-6.0833
DSI_poised_library_2023.maegz:436	248.228	Z44586758	-6.0788
DSI_poised_library_2023.maegz:136	234.226	Z44584202	-6.0663
DSI_poised_library_2023.maegz:633	190.074	Z18769001	-6.0562
DSI_poised_library_2023.maegz:463	243.221	Z57292378	-6.0529
DSI_poised_library_2023.maegz:436	248.228	Z44586758	-6.0496
DSI_poised_library_2023.maegz:67	229.110	Z56943440	-6.0442
DSI_poised_library_2023.maegz:802	179.062	Z425757818	-6.0353
DSI_poised_library_2023.maegz:175	209.105	Z1889787519	-6.0226
DSI_poised_library_2023.maegz:582	210.248	Z2092555279	-6.0121
DSI_poised_library_2023.maegz:718	251.280	Z55222357	-6.0077
DSI_poised_library_2023.maegz:383	245.085	Z19735192	-6.0073
DSI_poised_library_2023.maegz:63	299.729	Z1004825200	-5.9809
DSI_poised_library_2023.maegz:696	166.099	Z1255402624	-5.9782
DSI_poised_library_2023.maegz:712	193.110	Z235361235	-5.9706
DSI_poised_library_2023.maegz:59	223.112	Z198194394	-5.9618
DSI_poised_library_2023.maegz:21	241.245	Z1190787729	-5.9604
DSI_poised_library_2023.maegz:540	248.278	Z1086293874	-5.9592

DSI_poised_library_2023.maegz:274	237.128	Z57821475	-5.9546
DSI_poised_library_2023.maegz:104	235.137	Z31484539	-5.9487
DSI_poised_library_2023.maegz:696	166.099	Z1255402624	-5.9389
DSI_poised_library_2023.maegz:61	237.646	Z56259166	-5.9387
DSI_poised_library_2023.maegz:386	233.142	Z19735904	-5.9348
DSI_poised_library_2023.maegz:754	165.090	Z1324853681	-5.9197
DSI_poised_library_2023.maegz:662	242.082	Z123852286	-5.9151
DSI_poised_library_2023.maegz:613	213.130	Z1461982627	-5.9119
DSI_poised_library_2023.maegz:312	245.080	Z277116890	-5.9044
DSI_poised_library_2023.maegz:509	239.106	Z1587220559	-5.8978
DSI_poised_library_2023.maegz:63	299.729	Z1004825200	-5.8942
DSI_poised_library_2023.maegz:705	211.076	Z1127662991	-5.8816
DSI_poised_library_2023.maegz:754	165.090	Z1324853681	-5.8726
DSI_poised_library_2023.maegz:270	234.251	Z1545313172	-5.8695
DSI_poised_library_2023.maegz:683	231.051	Z57111868	-5.8683
DSI_poised_library_2023.maegz:696	166.099	Z1255402624	-5.8618
DSI_poised_library_2023.maegz:704	274.996	Z352704742	-5.8543
DSI_poised_library_2023.maegz:582	210.248	Z2092555279	-5.8387
DSI_poised_library_2023.maegz:217	170.106	Z220816104	-5.8321
DSI_poised_library_2023.maegz:719	203.069	Z332370018	-5.8237
DSI_poised_library_2023.maegz:104	235.137	Z31484539	-5.8224
DSI_poised_library_2023.maegz:802	179.062	Z425757818	-5.8194
DSI_poised_library_2023.maegz:137	190.111	Z285141302	-5.8171
DSI_poised_library_2023.maegz:358	218.295	Z31792168	-5.8094
DSI_poised_library_2023.maegz:771	239.225	Z1614545742	-5.8082
DSI_poised_library_2023.maegz:11	199.040	Z19727416	-5.8054
DSI_poised_library_2023.maegz:754	165.090	Z1324853681	-5.7942
DSI_poised_library_2023.maegz:754	165.090	Z1324853681	-5.7917
DSI_poised_library_2023.maegz:633	190.074	Z18769001	-5.7778
DSI_poised_library_2023.maegz:769	219.137	Z1509441355	-5.7728
DSI_poised_library_2023.maegz:67	229.110	Z56943440	-5.7677
DSI_poised_library_2023.maegz:312	245.080	Z277116890	-5.7613
DSI_poised_library_2023.maegz:254	175.075	Z1618027593	-5.75
DSI_poised_library_2023.maegz:51	239.698	Z1545312521	-5.7484
DSI_poised_library_2023.maegz:67	229.110	Z56943440	-5.7476
DSI_poised_library_2023.maegz:851	239.276	Z56983806	-5.7432
DSI_poised_library_2023.maegz:78	199.051	Z906021418	-5.7303
DSI_poised_library_2023.maegz:260	171.101	Z90122368	-5.7199
DSI_poised_library_2023.maegz:137	190.111	Z285141302	-5.6876
DSI_poised_library_2023.maegz:137	190.111	Z285141302	-5.6792
DSI_poised_library_2023.maegz:725	209.328	Z1275599911	-5.6744
DSI_poised_library_2023.maegz:217	170.106	Z220816104	-5.6588
DSI_poised_library_2023.maegz:260	171.101	Z90122368	-5.6431

DSI_poised_library_2023.maegz:260	171.101	Z90122368	-5.6324
DSI_poised_library_2023.maegz:812	195.101	Z57627041	-5.6311
DSI_poised_library_2023.maegz:773	192.057	Z193091370	-5.6283
DSI_poised_library_2023.maegz:85	189.057	Z1443544073	-5.6185
DSI_poised_library_2023.maegz:27	242.142	Z26548228	-5.6184
DSI_poised_library_2023.maegz:754	165.090	Z1324853681	-5.601
DSI_poised_library_2023.maegz:217	170.106	Z220816104	-5.601
DSI_poised_library_2023.maegz:217	170.106	Z220816104	-5.5963
DSI_poised_library_2023.maegz:773	192.057	Z193091370	-5.5895
DSI_poised_library_2023.maegz:773	192.057	Z193091370	-5.5674
DSI_poised_library_2023.maegz:773	192.057	Z193091370	-5.543
DSI_poised_library_2023.maegz:806	175.056	Z1262633486	-5.5382
DSI_poised_library_2023.maegz:261	170.106	Z32399802	-5.519
DSI_poised_library_2023.maegz:773	192.057	Z193091370	-5.5027
DSI_poised_library_2023.maegz:312	245.080	Z277116890	-5.457
DSI_poised_library_2023.maegz:769	219.137	Z1509441355	-5.4561
DSI_poised_library_2023.maegz:172	204.126	Z1206890404	-5.4492
DSI_poised_library_2023.maegz:323	174.116	Z1137725943	-5.4475
DSI_poised_library_2023.maegz:217	170.106	Z220816104	-5.4066
DSI_poised_library_2023.maegz:773	192.057	Z193091370	-5.3464
DSI_poised_library_2023.maegz:399	238.326	Z32399948	-5.2912
DSI_poised_library_2023.maegz:374	233.328	Z1446981563	-5.2824
DSI_poised_library_2023.maegz:817	173.167	Z1250093582	-5.2787
DSI_poised_library_2023.maegz:817	173.167	Z1250093582	-5.2132

Table S23. VS1 and VS2 for PDB fragment library targeting GRB2 dimer

Compound	MW	Energy in silico
VS1		
octahydroindolizine-1,6,7,8-tetrol	189.100	-8.387
VS2		
octahydroindolizine-1,6,7,8-tetrol	189.100	-7.2851
octahydroindolizine-1,6,7,8-tetrol	189.100	-7.0913
octahydroindolizine-1,6,7,8-tetrol	189.100	-6.9214
octahydroindolizine-1,6,7,8-tetrol	189.100	-6.8794
octahydroindolizine-1,6,7,8-tetrol	189.100	-6.8761
octahydroindolizine-1,6,7,8-tetrol	189.100	-6.8587
octahydroindolizine-1,6,7,8-tetrol	189.100	-6.85
octahydroindolizine-1,6,7,8-tetrol	189.100	-6.8459
octahydroindolizine-1,6,7,8-tetrol	189.100	-6.7903
octahydroindolizine-1,6,7,8-tetrol	189.100	-6.7264
octahydroindolizine-1,6,7,8-tetrol	189.100	-6.695
octahydroindolizine-1,6,7,8-tetrol	189.100	-6.5895
octahydroindolizine-1,6,7,8-tetrol	189.100	-6.5858

octahydroindolizine-1,6,7,8-tetrol	189.100	-6.5642
octahydroindolizine-1,6,7,8-tetrol	189.100	-6.5598
octahydroindolizine-1,6,7,8-tetrol	189.100	-6.5172
octahydroindolizine-1,6,7,8-tetrol	189.100	-6.5168
octahydroindolizine-1,6,7,8-tetrol	189.100	-6.5113
octahydroindolizine-1,6,7,8-tetrol	189.100	-6.4905
octahydroindolizine-1,6,7,8-tetrol	189.100	-6.4668
octahydroindolizine-1,6,7,8-tetrol	189.100	-6.459
octahydroindolizine-1,6,7,8-tetrol	189.100	-6.4577
1-butyl-2-(hydroxymethyl)piperidine-3,4,5-triol	219.147	-6.4453
octahydroindolizine-1,6,7,8-tetrol	189.100	-6.4159
octahydroindolizine-1,6,7,8-tetrol	189.100	-6.4101
octahydroindolizine-1,6,7,8-tetrol	189.100	-6.4085
octahydroindolizine-1,6,7,8-tetrol	189.100	-6.406
octahydroindolizine-1,6,7,8-tetrol	189.100	-6.4049
octahydroindolizine-1,6,7,8-tetrol	189.100	-6.4039
octahydroindolizine-1,6,7,8-tetrol	189.100	-6.4036
1-butyl-2-(hydroxymethyl)piperidine-3,4,5-triol	219.147	-6.4016
Cytosine deoxyribonucleoside		-6.3963
octahydroindolizine-1,6,7,8-tetrol	189.100	-6.3729
octahydroindolizine-1,6,7,8-tetrol	189.100	-6.3242
octahydroindolizine-1,6,7,8-tetrol	189.100	-6.3082
1-butyl-2-(hydroxymethyl)piperidine-3,4,5-triol	219.147	-6.3033
octahydroindolizine-1,6,7,8-tetrol	189.100	-6.2742
1, 3-Diphenylurea	212.095	-6.2702
octahydroindolizine-1,6,7,8-tetrol	189.100	-6.2683
octahydroindolizine-1,6,7,8-tetrol	189.100	-6.268
octahydroindolizine-1,6,7,8-tetrol	189.100	-6.2674
octahydroindolizine-1,6,7,8-tetrol	189.100	-6.2642
octahydroindolizine-1,6,7,8-tetrol	189.100	-6.2616
octahydroindolizine-1,6,7,8-tetrol	189.100	-6.2507
octahydroindolizine-1,6,7,8-tetrol	189.100	-6.249
Cordycepin	251.102	-6.2391
octahydroindolizine-1,6,7,8-tetrol	189.100	-6.2364
octahydroindolizine-1,6,7,8-tetrol	189.100	-6.2321
octahydroindolizine-1,6,7,8-tetrol	189.100	-6.2289
octahydroindolizine-1,6,7,8-tetrol	189.100	-6.2222
octahydroindolizine-1,6,7,8-tetrol	189.100	-6.2214
octahydroindolizine-1,6,7,8-tetrol	189.100	-6.1957
Acetazolamide	221.988	-6.1881
octahydroindolizine-1,6,7,8-tetrol	189.100	-6.186
1-butyl-2-(hydroxymethyl)piperidine-3,4,5-triol	219.147	-6.1857
octahydroindolizine-1,6,7,8-tetrol	189.100	-6.1848

1-butyl-2-(hydroxymethyl)piperidine-3,4,5-triol	219.147	-6.1846
Cytosine deoxyribonucleoside		-6.1605
octahydroindolizine-1,6,7,8-tetrol	189.100	-6.1565
octahydroindolizine-1,6,7,8-tetrol	189.100	-6.1526
1, 3-Diphenylurea	212.095	-6.1444
octahydroindolizine-1,6,7,8-tetrol	189.100	-6.1439
1-butyl-2-(hydroxymethyl)piperidine-3,4,5-triol	219.147	-6.121
Arabinosylcytosine		-6.0991
1-butyl-2-(hydroxymethyl)piperidine-3,4,5-triol	219.147	-6.0703
Thiabendazole	201.036	-6.0659
1-butyl-2-(hydroxymethyl)piperidine-3,4,5-triol	219.147	-6.0525
octahydroindolizine-1,6,7,8-tetrol	189.100	-6.0465
octahydroindolizine-1,6,7,8-tetrol	189.100	-6.0455
octahydroindolizine-1,6,7,8-tetrol	189.100	-6.0362
Arabinosylcytosine		-6.0313
octahydroindolizine-1,6,7,8-tetrol	189.100	-6.0243
octahydroindolizine-1,6,7,8-tetrol	189.100	-6.004
octahydroindolizine-1,6,7,8-tetrol	189.100	-5.9824
octahydroindolizine-1,6,7,8-tetrol	189.100	-5.9471
Thiabendazole	201.036	-5.9389
octahydroindolizine-1,6,7,8-tetrol	189.100	-5.9305
octahydroindolizine-1,6,7,8-tetrol	189.100	-5.9107
1-butyl-2-(hydroxymethyl)piperidine-3,4,5-triol	219.147	-5.8852
Arabinosylcytosine		-5.8797
Resveratrol	228.079	-5.8766
Umbelliferone	162.032	-5.8749
2-Amino-1H-benzo[d]imidazol-5-ol	149.059	-5.8738
Melatonin	232.121	-5.8689
L-tryptophanamide	203.106	-5.8152
octahydroindolizine-1,6,7,8-tetrol	189.100	-5.7993
3,4,5-trihydroxycyclohex-2-ene-1-carboxylic acid	174.053	-5.7817
3,4,5-trihydroxycyclohex-2-ene-1-carboxylic acid	174.053	-5.7743
1-butyl-2-(hydroxymethyl)piperidine-3,4,5-triol	219.147	-5.6917
Arabinosylcytosine		-5.6222
Serotonin	176.095	-5.6141
Piracetam	142.074	-5.6074
3,4,5-trihydroxycyclohex-2-ene-1-carboxylic acid	174.053	-5.5517
2-(3-Hydroxypicolinamido)Acetic Acid	196.048	-5.5227
2-(carbamoylamino)-3-phenylpropanoic acid	208.085	-5.4738
5-Hydroxyindole-3-acetic acid	191.058	-5.4725
5-Hydroxyindole-3-acetic acid	191.058	-5.4284
5-Hydroxyindole-3-acetic acid	191.058	-5.3322
1-Naphthylacetic acid	186.068	-5.1718

3,4,5-trihydroxycyclohex-2-ene-1-carboxylic acid	174.053	-5.1336

Table S24. VS1 and VS2 for LH1 FDA Approved drugs targeting GRB2 dimer

Drug Name	MolWt	Energy in silico
VS1		
Phthalylsulfathiazole	403.430	-7.023
VS2		
Diperodon hydrochloride	433.930	-8.9545
Diperodon hydrochloride	433.930	-8.6151
Mitoxantrone hydrochloride	517.400	-8.6066
Mitoxantrone	444.480	-8.6054
Diperodon hydrochloride	433.930	-8.0991
Diperodon hydrochloride	433.930	-7.8768
Diperodon hydrochloride	433.930	-7.5501
R-(+)-Lansoprazole	369.360	-7.3867
Lansoprazole	369.360	-7.3867
Diperodon hydrochloride	433.930	-7.2781
Diperodon hydrochloride	433.930	-7.2226
Phthalylsulfathiazole	403.430	-7.1942
Flupirtine maleate	420.390	-7.0422
Diperodon hydrochloride	433.930	-7.0241
Nifenazone	308.340	-6.9426
Flupirtine maleate	420.390	-6.911
Travoprost	500.550	-6.7809
Diperodon hydrochloride	433.930	-6.7766
Nifenazone	308.340	-6.7722
Naftifine hydrochloride	323.860	-6.7282
Naftifine hydrochloride	323.860	-6.7239
Furaltadone hydrochloride	360.750	-6.7196
Albendazole	265.330	-6.6615
Travoprost	500.550	-6.6456
Mepiroxol	125.130	-6.6245
Omeprazole	345.420	-6.6039
Dinaciclib	396.490	-6.6029
Flupirtine maleate	420.390	-6.5904
Terbinafine	291.430	-6.5804
Terbinafine	291.430	-6.5759
Pramiracetam	269.380	-6.5675
Lansoprazole	369.360	-6.5436
Methocarbamol	241.240	-6.5409

Nifenazone	308.340	-6.5179
Felbamate	238.240	-6.4881
Terbinafine	291.430	-6.4787
Travoprost	500.550	-6.4617
Naftifine hydrochloride	323.860	-6.4448
Lansoprazole	369.360	-6.4351
Furaltadone hydrochloride	360.750	-6.4206
Naftifine hydrochloride	323.860	-6.4155
Travoprost	500.550	-6.4092
Terbinafine	291.430	-6.4006
Dinaciclib	396.490	-6.3907
Omeprazole	345.420	-6.3868
Phthalylsulfathiazole	403.430	-6.3713
Methocarbamol	241.240	-6.3675
Phthalylsulfathiazole	403.430	-6.3671
Tasimelteon	245.320	-6.3637
Travoprost	500.550	-6.3532
Terbinafine	291.430	-6.3466
Mitoxantrone	444.480	-6.3289
Mitoxantrone hydrochloride	517.400	-6.3265
Omeprazole	345.420	-6.323
Suplatast tosilate	499.640	-6.323
Furaltadone hydrochloride	360.750	-6.3221
Alprostadil	354.480	-6.3173
Oxiracetam	158.160	-6.3107
Furaltadone hydrochloride	360.750	-6.3014
Racecadotril	385.490	-6.2872
Mitoxantrone hydrochloride	517.400	-6.2737
Mitoxantrone	444.480	-6.2722
Phthalylsulfacetamide	362.360	-6.2593
Pramiracetam	269.380	-6.2412
Oxiracetam	158.160	-6.24
Phthalylsulfacetamide	362.360	-6.2219
Anisomycin	265.300	-6.2183
Tenofovir Disoproxil	519.440	-6.2083
Adenosine	267.240	-6.1989
Tubercidin	266.250	-6.1987
Travoprost	500.550	-6.188
Isoprenaline hydrochloride	247.720	-6.186
R-(+)-Lansoprazole	369.360	-6.1839
Lansoprazole	369.360	-6.1839
Phthalylsulfathiazole	403.430	-6.1749
R-(+)-Lansoprazole	369.360	-6.1709

Lansoprazole	369.360	-6.1709
Travoprost	500.550	-6.1643
Naftifine hydrochloride	323.860	-6.1591
Felbamate	238.240	-6.1576
Propafenone	341.440	-6.1515
Nifuroxazide	275.220	-6.1489
Acetazolamide	222.250	-6.142
Racecadotril	385.490	-6.1359
Piperine	285.350	-6.1338
Esomeprazole Sodium	367.400	-6.1327
Piceatannol	244.240	-6.1211
Isocarboxazid	231.250	-6.1203
Nifuratel	285.280	-6.1129
Tenofovir Disoproxil	519.440	-6.1026
Omeprazole sulfide	329.420	-6.1005
Salicoside	286.280	-6.0868
Cyclandelate	276.380	-6.085
Adenosine	267.240	-6.0829
Pemirolast potassium	266.310	-6.0711
Rabeprazole Related Compound E	343.440	-6.0698
Naftifine hydrochloride	323.860	-6.0623
Nifuratel	285.280	-6.0595
Tenofovir Disoproxil	519.440	-6.051
Nicarbazin	426.380	-6.0507
Ramelteon	259.350	-6.0479
Albendazole	265.330	-6.0457
Oxiracetam	158.160	-6.0431
Daphnetin	178.140	-6.042
Alprostadil	354.480	-6.0415
Pantoprazole Sodium Hydrate	432.370	-6.0408
Norepinephrine	169.180	-6.0366
Noradrenaline bitartrate	319.260	-6.0366
Nitazoxanide	307.280	-6.034
Nifuratel	285.280	-6.0307
Daphnetin	178.140	-6.0292
Succinylsulfathiazole	355.390	-6.0278
Dibucaine hydrochloride	379.920	-6.0274
Dibucaine	343.460	-6.0274
Succinylsulfathiazole	355.390	-6.0244
Mepiroxol	125.130	-6.0175
Daphnetin	178.140	-6.0169
Pramiracetam	269.380	-6.0078

Albendazole Oxide	281.330	-6.0004
Etilefrine hydrochloride	217.690	-6.0002
Alprostadil	354.480	-5.9934
Camylofin	393.400	-5.9858
Propafenone	341.440	-5.9827
Retinol	286.450	-5.9754
Meloxicam	351.400	-5.9634
Triclabendazole	359.660	-5.9585
Sulfamethoxypyridazine	280.300	-5.958
Mepiroxol	125.130	-5.9431
Methocarbamol	241.240	-5.9429
Retinol	286.450	-5.9423
Pemirolast potassium	266.310	-5.9356
Terbinafine	291.430	-5.9338
Tasimelteon	245.320	-5.9276
10,11-Dihydrocarbamazepine	238.290	-5.9272
Suplatast tosilate	499.640	-5.9254
Isocarboxazid	231.250	-5.922
Benzydamine hydrochloride	345.870	-5.9182
Racecadotril	385.490	-5.9168
Butylphthalide	190.240	-5.9113
Oxiracetam	158.160	-5.9086
Piperine	285.350	-5.9081
Rabeprazole Related Compound E	343.440	-5.9079
Dicurone	176.120	-5.907
Racecadotril	385.490	-5.8985
Dibucaine hydrochloride	379.920	-5.8834
Dibucaine	343.460	-5.8834
Dicurone	176.120	-5.8743
Lafutidine	431.550	-5.8722
Piceatannol	244.240	-5.8601
Methocarbamol	241.240	-5.8561
Salicoside	286.280	-5.8541
Norepinephrine	169.180	-5.8532
Noradrenaline bitartrate	319.260	-5.8532
Nafronyl oxalate	473.560	-5.8517
Sulfachloropyridazine	284.720	-5.8509
Milrinone	211.220	-5.8463
Fraxetin	208.170	-5.8343
Travoprost	500.550	-5.8342
Etilefrine hydrochloride	217.690	-5.8309
ETC1002	344.490	-5.8255

Propafenone	341.440	-5.8216
Piceatannol	244.240	-5.8211
Upadacitinib	380.370	-5.8165
Zileuton	236.290	-5.8119
Succinylsulfathiazole	355.390	-5.8005
Pexidartinib	417.810	-5.7869
Tioxolone	168.170	-5.7794
4-Methylumbelliferone	176.170	-5.7752
Dicurone	176.120	-5.774
Agomelatine	243.300	-5.7663
Delta-Gluconolactone	178.140	-5.762
Sulfachloropyridazine	284.720	-5.757
Travoprost	500.550	-5.7513
Miglitol	207.220	-5.751
4-Aminoantipyrine	203.240	-5.7477
Terbinafine hydrochloride	327.890	-5.7429
Gemcitabine HCl	299.660	-5.742
Gemcitabine	263.200	-5.742
Norepinephrine	169.180	-5.7345
Noradrenaline bitartrate	319.260	-5.7345
Albendazole	265.330	-5.7329
Aloe-emodin	270.240	-5.7328
Meloxicam	351.400	-5.7315
Aspartame	294.300	-5.7234
Dermofongin	179.600	-5.7231
Tioxolone	168.170	-5.7126
Chloroxylenol	156.610	-5.7075
DL-Adrenaline Hydrochloride	219.670	-5.7018
Chloroxylenol	156.610	-5.6995
AICAR (Acadesine)	258.240	-5.6987
Fraxetin	208.170	-5.6914
Stiripentol	234.290	-5.6861
Cyclandelate	276.380	-5.6724
Milrinone	211.220	-5.6701
Ornidazole	219.630	-5.6662
Piperine	285.350	-5.6657
Dinaciclib	396.490	-5.656
Oxiracetam	158.160	-5.6555
Rufinamide	238.190	-5.6528
Furazolidone	225.160	-5.6489
Aminophylline	420.430	-5.6482
(+)-Pilocarpine hydrochloride	244.720	-5.6482
Pilocarpine nitrate	271.270	-5.6479

Rufinamide	238.190	-5.6405
Upadacitinib	380.370	-5.6192
Serotonin hydrochloride	212.680	-5.6177
Succinylsulfathiazole	355.390	-5.6163
Travoprost	500.550	-5.6138
Piracetam	142.160	-5.6074
Carbazochrome	236.230	-5.6064
Dermofongin	179.600	-5.6046
DL-Adrenaline Hydrochloride	219.670	-5.5997
Terbinafine hydrochloride	327.890	-5.5966
ETC1002	344.490	-5.5959
Suplatast tosilate	499.640	-5.592
Edrophonium chloride	201.690	-5.5892
Omeprazole sulfide	329.420	-5.5863
Stiripentol	234.290	-5.5854
Dicurone	176.120	-5.5846
Bendazol	208.260	-5.5793
Cefalexine EP IMpurity B	214.240	-5.5736
Tioxolone	168.170	-5.5619
Pantoprazole Sodium Hydrate	432.370	-5.5579
Milrinone	211.220	-5.5523
Chlormezanone	273.730	-5.5424
Phthalylsulfacetamide	362.360	-5.5381
Isoprenaline hydrochloride	247.720	-5.5375
Oxiracetam	158.160	-5.5353
Terbinafine hydrochloride	327.890	-5.5336
Sulfameter	280.310	-5.5307
Diazoxide	230.670	-5.524
Ribavirin	244.200	-5.5228
Leflunomide	270.210	-5.522
Suplatast tosilate	499.640	-5.519
Levetiracetam	170.210	-5.5182
4-Aminoantipyrine	203.240	-5.5039
AICAR (Acadesine)	258.240	-5.5026
Dermofongin	179.600	-5.4978
Dinitolmide	225.160	-5.4957
Isoprenaline hydrochloride	247.720	-5.4902
L-5-Hydroxytryptophan	220.220	-5.4828
4-Methylumbelliferone	176.170	-5.4817
Naphazoline hydrochloride	246.740	-5.4705
Esomeprazole Magnesium	713.120	-5.4703
Eslicarbazepine Acetate	296.320	-5.4688
Serotonin hydrochloride	212.680	-5.4664

Citiolone	159.210	-5.4643
Serotonin hydrochloride	212.680	-5.4562
Chrysophanic Acid	254.240	-5.4552
Synephrine	167.210	-5.4501
Travoprost	500.550	-5.4483
Imidapril HCl	441.910	-5.4421
Propylthiouracil	170.240	-5.4364
ETC1002	344.490	-5.4347
Piracetam	142.160	-5.434
Hydroxychloroquine sulfate	433.950	-5.4322
Succinylsulfathiazole	355.390	-5.4283
Aminophylline	420.430	-5.4255
Chlormezanone	273.730	-5.4234
Cyclandelate	276.380	-5.4229
Pantoprazole Sodium Hydrate	432.370	-5.4194
Tubercidin	266.250	-5.4174
Milrinone	211.220	-5.415
Tubercidin	266.250	-5.408
Rimantadine hydrochloride	215.760	-5.3984
Propylthiouracil	170.240	-5.3982
Rufinamide	238.190	-5.3962
Dinitolmide	225.160	-5.3908
Piracetam	142.160	-5.3871
Mafenide hydrochloride	222.690	-5.3584
Benzyl alcohol	108.140	-5.3584
Flutamide	276.210	-5.3535
L-5-Hydroxytryptophan	220.220	-5.3473
Diprophylline	254.250	-5.3452
Pexidartinib	417.810	-5.3444
Propylthiouracil	170.240	-5.3336
Zonisamide	212.230	-5.3333
Zonisamide	212.230	-5.3275
Hydroxychloroquine sulfate	433.950	-5.327
Gallic acid	170.120	-5.3259
L-5-Hydroxytryptophan	220.220	-5.3245
Dichlorphenamide	305.160	-5.3199
Esomeprazole Magnesium	713.120	-5.3177
Etofenamate	369.340	-5.2967
Nicotinic acid N-oxide	139.110	-5.2914
Tizoxanide	265.250	-5.2873
Meptazinol hydrochloride	269.810	-5.2825
Triflusal	248.160	-5.2772
Travoprost	500.550	-5.2734

Lansoprazole	369.360	-5.2726
Esomeprazole Sodium	367.400	-5.267
Carbamazepine	236.270	-5.2638
Memantine hydrochloride	215.760	-5.256
Ambroxol hydrochloride	414.560	-5.2529
Ambroxol	378.100	-5.2529
Ixazomib	361.030	-5.2507
Flopropione	182.180	-5.2503
Tretinoin	300.440	-5.243
L-Tryptophan	204.230	-5.2308
Aminacrine	194.230	-5.2295
10,11-Dihydrocarbamazepine	238.290	-5.2244
Oxcarbazepine	252.270	-5.2223
Ademetionine	398.440	-5.2171
Meticrane	275.340	-5.217
Acitretin	326.430	-5.2136
cystine	240.290	-5.2117
Carbazochrome	236.230	-5.2108
Rebeprazole sodium	381.420	-5.2067
Butenafine hydrochloride	353.930	-5.1987
Tizoxanide	265.250	-5.1957
Esomeprazole Magnesium	713.120	-5.1851
Butoconazole nitrate	474.790	-5.1765
Tenofovir Disoproxil Fumarate	635.510	-5.1658
Isoliquiritigenin	256.250	-5.1562
Travoprost	500.550	-5.1534
Baricitinib	371.420	-5.1393
Baricitinib?Phosphate	469.410	-5.1393
Bortezomib	384.240	-5.1298
Baricitinib	371.420	-5.1256
Baricitinib?Phosphate	469.410	-5.1256
Mafenide hydrochloride	222.690	-5.1145
Carbazochrome	236.230	-5.0879
Bicalutamide	430.370	-5.0772
Terbinafine hydrochloride	327.890	-5.0742
Memantine hydrochloride	215.760	-5.067
Ciclopirox	207.270	-5.0648
Ciclopirox ethanolamine	268.350	-5.0648
10,11-Dihydrocarbamazepine	238.290	-5.0569
Spiculisporic Acid	328.410	-5.0565
Enalaprilat Dihydrate	348.400	-5.0516
Esomeprazole Magnesium	713.120	-5.05
Nafronyl oxalate	473.560	-5.0292

Levetiracetam	170.210	-5.0257
Penicillin V potassium salt	388.480	-5.0098
Cyclocytidine hydrochloride	261.660	-5.0095
Travoprost	500.550	-5.0053
Ademetionine	398.440	-5.0011
Bicalutamide	430.370	-4.9947
Butenafine hydrochloride	353.930	-4.9859
Mupirocin	500.640	-4.9686
Esomeprazole Magnesium	713.120	-4.9614
Cyclocytidine hydrochloride	261.660	-4.9577
Tenofovir Disoproxil	519.440	-4.9573
Ademetionine	398.440	-4.9494
Rimantadine hydrochloride	215.760	-4.9175
Hydroxychloroquine sulfate	433.950	-4.911
Methoxamine hydrochloride	247.720	-4.9104
Biapenem	350.390	-4.9088
Lubiprostone	390.460	-4.8928
Ademetionine	398.440	-4.8916
Isoprinosine	1115.230	-4.8817
Inosine	268.230	-4.8817
L-Hydroxyprolin	131.130	-4.8799
Rabeprazole Related Compound E	343.440	-4.8676
Tizoxanide	265.250	-4.8653
Lubiprostone	390.460	-4.8649
Spiculisporic Acid	328.410	-4.8553
(S)-crizotinib	450.340	-4.8545
Hydroxychloroquine sulfate	433.950	-4.8379
Ademetionine	398.440	-4.823
Bicalutamide	430.370	-4.8166
Dorzolamide hydrochloride	360.900	-4.7961
Gliclazide	323.420	-4.7767
Nafronyl oxalate	473.560	-4.7607
Imidapril HCl	441.910	-4.7447
Dorzolamide hydrochloride	360.900	-4.7416
Enalapril Maleate	492.520	-4.7361
Biapenem	350.390	-4.7154
Istradefylline	384.430	-4.6779
Esomeprazole Sodium	367.400	-4.6426
Spiculisporic Acid	328.410	-4.637
Travoprost	500.550	-4.6208
Ademetionine	398.440	-4.5996
Cinacalcet hydrochloride	393.870	-4.5779

Biapenem	350.390	-4.5409
Rebeprazole sodium	381.420	-4.5112
Lubiprostone	390.460	-4.435
Ademetionine	398.440	-4.4258
Ademetionine	398.440	-4.3818
Procaine penicillin G	570.700	-4.2331
Ademetionine	398.440	-4.2215
Benzylpenicillin potassium	372.480	-4.1929
Penicillin G sodium salt	356.370	-4.1851
Travoprost	500.550	-4.1839
Lumiracoxib	293.720	-4.1747
Ramipril	416.510	-3.9278
Ademetionine	398.440	-3.7953
Ademetionine	398.440	-3.6653
Roxadustat	352.340	-3.3712

Table S25. VS1 and VS2 for DSI-poised targeting POLQ helicase site

Compound	MW	Catalog ID	Energy in silico
VS1			
DSI_poised_library_2023.maegz:47	142.111	Z1216837642	-3.7405
VS2			
DSI_poised_library_2023.maegz:643	253.098	Z381033368	-5.5864
DSI_poised_library_2023.maegz:63	299.729	Z1004825200	-5.5171
DSI_poised_library_2023.maegz:643	253.098	Z381033368	-5.4332
DSI_poised_library_2023.maegz:643	253.098	Z381033368	-5.4313
DSI_poised_library_2023.maegz:283	205.133	Z56791867	-5.3605
DSI_poised_library_2023.maegz:63	299.729	Z1004825200	-5.3537
DSI_poised_library_2023.maegz:600	244.109	Z1973465508	-5.2839
DSI_poised_library_2023.maegz:274	237.128	Z57821475	-5.2227
DSI_poised_library_2023.maegz:274	237.128	Z57821475	-5.1963
DSI_poised_library_2023.maegz:752	246.259	Z29634868	-5.1703
DSI_poised_library_2023.maegz:368	192.101	Z111634612	-5.1594
DSI_poised_library_2023.maegz:403	189.115	Z28870646	-5.1518
DSI_poised_library_2023.maegz:283	205.133	Z56791867	-5.1493
DSI_poised_library_2023.maegz:47	142.111	Z1216837642	-5.1252
DSI_poised_library_2023.maegz:542	207.083	Z31222641	-5.1233
DSI_poised_library_2023.maegz:466	221.080	Z33486130	-5.1105
DSI_poised_library_2023.maegz:11	199.040	Z19727416	-5.0892
DSI_poised_library_2023.maegz:63	299.729	Z1004825200	-5.0881
DSI_poised_library_2023.maegz:403	189.115	Z28870646	-5.083
DSI_poised_library_2023.maegz:827	212.144	Z1758466556	-5.0678

DSI_poised_library_2023.maegz:274	237.128	Z57821475	-5.063
DSI_poised_library_2023.maegz:47	142.111	Z1216837642	-5.0595
DSI_poised_library_2023.maegz:114	229.105	Z1758466000	-5.0532
DSI_poised_library_2023.maegz:553	179.095	Z56960248	-5.0261
DSI_poised_library_2023.maegz:119	262.054	Z1491353358	-5.0245
DSI_poised_library_2023.maegz:570	231.294	Z29077827	-5.0029
DSI_poised_library_2023.maegz:299	216.119	Z2239077906	-4.9973
DSI_poised_library_2023.maegz:63	299.729	Z1004825200	-4.9847
DSI_poised_library_2023.maegz:92	234.033	Z85956652	-4.9616
DSI_poised_library_2023.maegz:645	162.091	Z234898257	-4.9555
DSI_poised_library_2023.maegz:542	207.083	Z31222641	-4.9461
DSI_poised_library_2023.maegz:553	179.095	Z56960248	-4.9395
DSI_poised_library_2023.maegz:600	244.109	Z1973465508	-4.934
DSI_poised_library_2023.maegz:600	244.109	Z1973465508	-4.9266
DSI_poised_library_2023.maegz:115	216.067	Z1449748885	-4.9258
DSI_poised_library_2023.maegz:553	179.095	Z56960248	-4.9195
DSI_poised_library_2023.maegz:115	216.067	Z1449748885	-4.9078
DSI_poised_library_2023.maegz:63	299.729	Z1004825200	-4.8854
DSI_poised_library_2023.maegz:645	162.091	Z234898257	-4.8757
DSI_poised_library_2023.maegz:671	238.112	Z192955056	-4.8757
DSI_poised_library_2023.maegz:553	179.095	Z56960248	-4.8745
DSI_poised_library_2023.maegz:645	162.091	Z234898257	-4.8718
DSI_poised_library_2023.maegz:250	239.119	Z316901672	-4.869
DSI_poised_library_2023.maegz:589	290.062	Z3074074344	-4.8561
DSI_poised_library_2023.maegz:151	190.074	Z19739650	-4.8554
DSI_poised_library_2023.maegz:718	251.280	Z55222357	-4.8481
DSI_poised_library_2023.maegz:114	229.105	Z1758466000	-4.8452
DSI_poised_library_2023.maegz:250	239.119	Z316901672	-4.8435
DSI_poised_library_2023.maegz:699	178.078	Z1493056027	-4.8346
DSI_poised_library_2023.maegz:650	144.090	Z854097264	-4.8275
DSI_poised_library_2023.maegz:115	216.067	Z1449748885	-4.8206
DSI_poised_library_2023.maegz:299	216.119	Z2239077906	-4.8198
DSI_poised_library_2023.maegz:142	244.109	Z1757321532	-4.8119
DSI_poised_library_2023.maegz:741	189.026	Z1295863442	-4.8024
DSI_poised_library_2023.maegz:635	142.111	Z90664455	-4.7981
DSI_poised_library_2023.maegz:256	142.111	Z166645986	-4.7918
DSI_poised_library_2023.maegz:236	226.078	Z1333043510	-4.786
DSI_poised_library_2023.maegz:47	142.111	Z1216837642	-4.7747
DSI_poised_library_2023.maegz:28	265.111	Z85893853	-4.7717
DSI_poised_library_2023.maegz:114	229.105	Z1758466000	-4.7679
DSI_poised_library_2023.maegz:545	197.049	Z364321922	-4.7652
DSI_poised_library_2023.maegz:98	204.101	Z131516158	-4.7567
DSI_poised_library_2023.maegz:812	195.101	Z57627041	-4.7523

DSI_poised_library_2023.maegz:804	214.054	Z1491215378	-4.7494
DSI_poised_library_2023.maegz:209	163.100	Z270979606	-4.7476
DSI_poised_library_2023.maegz:47	142.111	Z1216837642	-4.7422
DSI_poised_library_2023.maegz:713	167.095	Z57102475	-4.7321
DSI_poised_library_2023.maegz:679	223.011	Z385450668	-4.7255
DSI_poised_library_2023.maegz:637	196.132	Z1742054999	-4.7247
DSI_poised_library_2023.maegz:56	142.147	Z271005810	-4.7225
DSI_poised_library_2023.maegz:564	200.116	Z54846870	-4.7223
DSI_poised_library_2023.maegz:699	178.078	Z1493056027	-4.7148
DSI_poised_library_2023.maegz:635	142.111	Z90664455	-4.7135
DSI_poised_library_2023.maegz:828	236.023	Z277969624	-4.7125
DSI_poised_library_2023.maegz:47	142.111	Z1216837642	-4.7075
DSI_poised_library_2023.maegz:388	222.117	Z46169164	-4.6957
DSI_poised_library_2023.maegz:142	244.109	Z1757321532	-4.6912
DSI_poised_library_2023.maegz:795	236.039	Z426041412	-4.6837
DSI_poised_library_2023.maegz:466	221.080	Z33486130	-4.6793
DSI_poised_library_2023.maegz:418	142.111	Z477328060	-4.6754
DSI_poised_library_2023.maegz:500	144.126	Z271007874	-4.6751
DSI_poised_library_2023.maegz:154	192.093	Z285141528	-4.671
DSI_poised_library_2023.maegz:545	197.049	Z364321922	-4.67
DSI_poised_library_2023.maegz:666	189.072	Z166605480	-4.6699
DSI_poised_library_2023.maegz:804	214.054	Z1491215378	-4.6647
DSI_poised_library_2023.maegz:433	197.105	Z1741959530	-4.663
DSI_poised_library_2023.maegz:47	142.111	Z1216837642	-4.6524
DSI_poised_library_2023.maegz:47	142.111	Z1216837642	-4.6507
DSI_poised_library_2023.maegz:378	206.142	Z332382842	-4.6499
DSI_poised_library_2023.maegz:528	199.168	Z239095910	-4.647
DSI_poised_library_2023.maegz:795	236.039	Z426041412	-4.6446
DSI_poised_library_2023.maegz:713	167.095	Z57102475	-4.6266
DSI_poised_library_2023.maegz:47	142.111	Z1216837642	-4.6251
DSI_poised_library_2023.maegz:261	170.106	Z32399802	-4.6164
DSI_poised_library_2023.maegz:576	194.058	Z56900769	-4.614
DSI_poised_library_2023.maegz:351	241.085	Z26781964	-4.6139
DSI_poised_library_2023.maegz:68	203.106	Z26251905	-4.6119
DSI_poised_library_2023.maegz:411	182.051	Z56921372	-4.6101
DSI_poised_library_2023.maegz:217	170.106	Z220816104	-4.5937
DSI_poised_library_2023.maegz:256	142.111	Z166645986	-4.5905
DSI_poised_library_2023.maegz:56	142.147	Z271005810	-4.588
DSI_poised_library_2023.maegz:96	197.049	Z364328788	-4.5846
DSI_poised_library_2023.maegz:56	142.147	Z271005810	-4.584
DSI_poised_library_2023.maegz:719	203.069	Z332370018	-4.579
DSI_poised_library_2023.maegz:47	142.111	Z1216837642	-4.5748
DSI_poised_library_2023.maegz:47	142.111	Z1216837642	-4.5738

DSI_poised_library_2023.maegz:600	244.109	Z1973465508	-4.5726
DSI_poised_library_2023.maegz:736	191.031	Z1222424326	-4.5678
DSI_poised_library_2023.maegz:256	142.111	Z166645986	-4.5635
DSI_poised_library_2023.maegz:804	214.054	Z1491215378	-4.5634
DSI_poised_library_2023.maegz:234	210.046	Z119990900	-4.5625
DSI_poised_library_2023.maegz:773	192.057	Z193091370	-4.5585
DSI_poised_library_2023.maegz:713	167.095	Z57102475	-4.5489
DSI_poised_library_2023.maegz:802	179.062	Z425757818	-4.5433
DSI_poised_library_2023.maegz:773	192.057	Z193091370	-4.5429
DSI_poised_library_2023.maegz:47	142.111	Z1216837642	-4.5428
DSI_poised_library_2023.maegz:430	235.099	Z134785326	-4.5395
DSI_poised_library_2023.maegz:144	177.054	Z1509191464	-4.5376
DSI_poised_library_2023.maegz:754	165.090	Z1324853681	-4.5297
DSI_poised_library_2023.maegz:773	192.057	Z193091370	-4.5065
DSI_poised_library_2023.maegz:500	144.126	Z271007874	-4.505
DSI_poised_library_2023.maegz:198	199.043	Z1272684692	-4.502
DSI_poised_library_2023.maegz:621	142.147	Z271005826	-4.5006
DSI_poised_library_2023.maegz:564	200.116	Z54846870	-4.5001
DSI_poised_library_2023.maegz:623	155.050	Z56963343	-4.4536
DSI_poised_library_2023.maegz:635	142.111	Z90664455	-4.4505
DSI_poised_library_2023.maegz:460	163.067	Z53825479	-4.4243
DSI_poised_library_2023.maegz:527	156.126	Z364013242	-4.4237
DSI_poised_library_2023.maegz:7	191.098	Z1013740522	-4.4046
DSI_poised_library_2023.maegz:639	159.090	Z979770898	-4.3794
DSI_poised_library_2023.maegz:639	159.090	Z979770898	-4.3735
DSI_poised_library_2023.maegz:621	142.147	Z271005826	-4.3511
DSI_poised_library_2023.maegz:468	187.084	Z2049876225	-4.3409
DSI_poised_library_2023.maegz:198	199.043	Z1272684692	-4.3317
DSI_poised_library_2023.maegz:418	142.111	Z477328060	-4.3252
DSI_poised_library_2023.maegz:433	197.105	Z1741959530	-4.3213
DSI_poised_library_2023.maegz:512	225.130	Z871834402	-4.3106
DSI_poised_library_2023.maegz:198	199.043	Z1272684692	-4.2866
DSI_poised_library_2023.maegz:500	144.126	Z271007874	-4.2855
DSI_poised_library_2023.maegz:746	143.058	Z1171217431	-4.2228
DSI_poised_library_2023.maegz:217	170.106	Z220816104	-4.2085
DSI_poised_library_2023.maegz:639	159.090	Z979770898	-4.2072
DSI_poised_library_2023.maegz:208	189.077	Z56960214	-4.1633
DSI_poised_library_2023.maegz:208	189.077	Z56960214	-4.1578
DSI_poised_library_2023.maegz:782	150.116	Z513800486	-4.1432
DSI_poised_library_2023.maegz:564	200.116	Z54846870	-4.1162
DSI_poised_library_2023.maegz:699	178.078	Z1493056027	-3.9949
DSI_poised_library_2023.maegz:486	122.084	Z57390007	-3.9858
DSI_poised_library_2023.maegz:56	142.147	Z271005810	-3.9378

DSI_poised_library_2023.maegz:418	142.111	Z477328060	-3.9101
DSI_poised_library_2023.maegz:343	142.147	Z1741966630	-3.8241
DSI_poised_library_2023.maegz:343	142.147	Z1741966630	-3.7218
DSI_poised_library_2023.maegz:355	171.174	Z1142623285	-3.6941
DSI_poised_library_2023.maegz:12	145.110	Z734147462	-3.5628
DSI_poised_library_2023.maegz:176	211.132	Z265541128	-3.4717

Table S26. VS1 and VS2 for PDB fragment library targeting POLQ helicase site

Compound	MW	Energy in silico
VS1		
octahydroindolizine-1,6,7,8-tetrol	189.210	-5.5616
VS2		
octahydroindolizine-1,6,7,8-tetrol	189.210	-6.0061
octahydroindolizine-1,6,7,8-tetrol	189.210	-5.8401
octahydroindolizine-1,6,7,8-tetrol	189.210	-5.6458
octahydroindolizine-1,6,7,8-tetrol	189.210	-5.6048
1-Deoxy-L-rhamnojirimycin	147.170	-5.5572
octahydroindolizine-1,6,7,8-tetrol	189.210	-5.5359
octahydroindolizine-1,6,7,8-tetrol	189.210	-5.5015
N-(2-aminoethyl)isoquinoline-5-sulfonamide	251.310	-5.4976
octahydroindolizine-1,6,7,8-tetrol	189.210	-5.4671
octahydroindolizine-1,6,7,8-tetrol	189.210	-5.4644
octahydroindolizine-1,6,7,8-tetrol	189.210	-5.4609
octahydroindolizine-1,6,7,8-tetrol	189.210	-5.4515
Migalastat	163.170	-5.3931
1-Deoxy-L-rhamnojirimycin	147.170	-5.3902
octahydroindolizine-1,6,7,8-tetrol	189.210	-5.3763
1-Deoxy-L-rhamnojirimycin	147.170	-5.3316
N-(2-aminoethyl)isoquinoline-5-sulfonamide	251.310	-5.3315
octahydroindolizine-1,6,7,8-tetrol	189.210	-5.3303
octahydroindolizine-1,6,7,8-tetrol	189.210	-5.3213
octahydroindolizine-1,6,7,8-tetrol	189.210	-5.3151
Migalastat	163.170	-5.2837
Caffeine	194.190	-5.2481
Migalastat	163.170	-5.2477
Arabinosylcytosine		-5.2448
octahydroindolizine-1,6,7,8-tetrol	189.210	-5.2429
1-Deoxy-L-rhamnojirimycin	147.170	-5.2357
1-Deoxymannojirimycin	163.170	-5.2307
1-Deoxy-L-rhamnojirimycin	147.170	-5.2262

octahydroindolizine-1,6,7,8-tetrol	189.210	-5.2159
octahydroindolizine-1,6,7,8-tetrol	189.210	-5.2051
Tranexamic Acid	157.210	-5.2039
1-Deoxy-L-rhamnojirimycin	147.170	-5.1987
octahydroindolizine-1,6,7,8-tetrol	189.210	-5.1976
1-Deoxy-L-rhamnojirimycin	147.170	-5.1974
octahydroindolizine-1,6,7,8-tetrol	189.210	-5.1915
1-Deoxy-L-rhamnojirimycin	147.170	-5.1834
N-(2-aminoethyl)isoquinoline-5-sulfonamide	251.310	-5.1741
1-Deoxy-L-rhamnojirimycin	147.170	-5.1735
1-Deoxy-L-rhamnojirimycin	147.170	-5.1733
octahydroindolizine-1,6,7,8-tetrol	189.210	-5.1729
octahydroindolizine-1,6,7,8-tetrol	189.210	-5.171
octahydroindolizine-1,6,7,8-tetrol	189.210	-5.1704
octahydroindolizine-1,6,7,8-tetrol	189.210	-5.1511
octahydroindolizine-1,6,7,8-tetrol	189.210	-5.1473
octahydroindolizine-1,6,7,8-tetrol	189.210	-5.1469
octahydroindolizine-1,6,7,8-tetrol	189.210	-5.1277
1-Deoxy-L-rhamnojirimycin	147.170	-5.1266
Difunisal	250.200	-5.1214
octahydroindolizine-1,6,7,8-tetrol	189.210	-5.1173
octahydroindolizine-1,6,7,8-tetrol	189.210	-5.1156
1-Deoxy-L-rhamnojirimycin	147.170	-5.1037
Arabinosylcytosine		-5.0989
1-Deoxy-L-rhamnojirimycin	147.170	-5.0982
octahydroindolizine-1,6,7,8-tetrol	189.210	-5.0918
1-Deoxy-L-rhamnojirimycin	147.170	-5.078
octahydroindolizine-1,6,7,8-tetrol	189.210	-5.0763
L-tryptophanamide	203.240	-5.0729
octahydroindolizine-1,6,7,8-tetrol	189.210	-5.0307
octahydroindolizine-1,6,7,8-tetrol	189.210	-5.0287
octahydroindolizine-1,6,7,8-tetrol	189.210	-5.0177
octahydroindolizine-1,6,7,8-tetrol	189.210	-5.0113
2-Acetamido-1,2-Dideoxynojirmycin	204.220	-5.0013
octahydroindolizine-1,6,7,8-tetrol	189.210	-4.9997
Arabinosylcytosine		-4.9992
octahydroindolizine-1,6,7,8-tetrol	189.210	-4.9917
octahydroindolizine-1,6,7,8-tetrol	189.210	-4.9915
octahydroindolizine-1,6,7,8-tetrol	189.210	-4.9894
2-Naphthimidamide	170.210	-4.9842
1-Deoxymannojirimycin	163.170	-4.9838
octahydroindolizine-1,6,7,8-tetrol	189.210	-4.9832
1-Deoxy-L-rhamnojirimycin	147.170	-4.9777

1-Deoxymannojirimycin	163.170	-4.9768
1-Deoxy-L-rhamnojirimycin	147.170	-4.9645
octahydroindolizine-1,6,7,8-tetrol	189.210	-4.9526
octahydroindolizine-1,6,7,8-tetrol	189.210	-4.9415
octahydroindolizine-1,6,7,8-tetrol	189.210	-4.9357
octahydroindolizine-1,6,7,8-tetrol	189.210	-4.9344
octahydroindolizine-1,6,7,8-tetrol	189.210	-4.9284
octahydroindolizine-1,6,7,8-tetrol	189.210	-4.9153
1-Deoxy-L-rhamnojirimycin	147.170	-4.9133
1-Deoxy-L-rhamnojirimycin	147.170	-4.907
octahydroindolizine-1,6,7,8-tetrol	189.210	-4.9037
1-Deoxy-L-rhamnojirimycin	147.170	-4.9013
1-Deoxy-L-rhamnojirimycin	147.170	-4.8982
1-Deoxy-L-rhamnojirimycin	147.170	-4.8724
1-Deoxy-L-rhamnojirimycin	147.170	-4.8711
1-Deoxy-L-rhamnojirimycin	147.170	-4.865
Tranexamic Acid	157.210	-4.8645
1-Deoxy-L-rhamnojirimycin	147.170	-4.8645
octahydroindolizine-1,6,7,8-tetrol	189.210	-4.8584
1-Deoxy-L-rhamnojirimycin	147.170	-4.8462
octahydroindolizine-1,6,7,8-tetrol	189.210	-4.8428
octahydroindolizine-1,6,7,8-tetrol	189.210	-4.8405
Histamine	111.150	-4.8253
Thiophene-3-carboxamidine		-4.824
octahydroindolizine-1,6,7,8-tetrol	189.210	-4.8015
1-Deoxy-L-rhamnojirimycin	147.170	-4.7918
Thioguanine	167.190	-4.774
1-Deoxy-L-rhamnojirimycin	147.170	-4.7698
octahydroindolizine-1,6,7,8-tetrol	189.210	-4.7672
octahydroindolizine-1,6,7,8-tetrol	189.210	-4.7669
Tranexamic Acid	157.210	-4.759
octahydroindolizine-1,6,7,8-tetrol	189.210	-4.7447
octahydroindolizine-1,6,7,8-tetrol	189.210	-4.7427
octahydroindolizine-1,6,7,8-tetrol	189.210	-4.7425
octahydroindolizine-1,6,7,8-tetrol	189.210	-4.7386
octahydroindolizine-1,6,7,8-tetrol	189.210	-4.7369
1-Deoxy-L-rhamnojirimycin	147.170	-4.7196
octahydroindolizine-1,6,7,8-tetrol	189.210	-4.7165
octahydroindolizine-1,6,7,8-tetrol	189.210	-4.7156
1-Deoxy-L-rhamnojirimycin	147.170	-4.7154
1-Deoxy-L-rhamnojirimycin	147.170	-4.7068
Tranexamic Acid	157.210	-4.7064
1-Deoxy-L-rhamnojirimycin	147.170	-4.7014

1-Deoxy-L-rhamnojirimycin	147.170	-4.7005
octahydroindolizine-1,6,7,8-tetrol	189.210	-4.6928
6-Acetamido-6-Deoxy-Castanospermine	230.260	-4.687
1-Deoxy-L-rhamnojirimycin	147.170	-4.6423
octahydroindolizine-1,6,7,8-tetrol	189.210	-4.5955
1-Deoxy-L-rhamnojirimycin	147.170	-4.5657
1H-Benzo[d]imidazole-2-sulfonamide	197.220	-4.5656
Tranexamic Acid	157.210	-4.5572
octahydroindolizine-1,6,7,8-tetrol	189.210	-4.5558
1-Deoxy-L-rhamnojirimycin	147.170	-4.5423
octahydroindolizine-1,6,7,8-tetrol	189.210	-4.5359
Tranexamic Acid	157.210	-4.5244
octahydroindolizine-1,6,7,8-tetrol	189.210	-4.501
6-Acetamido-6-Deoxy-Castanospermine	230.260	-4.4992
Tryptamine	160.220	-4.4907
octahydroindolizine-1,6,7,8-tetrol	189.210	-4.4083
octahydroindolizine-1,6,7,8-tetrol	189.210	-4.4081
3,4,5-trihydroxycyclohex-2-ene-1-carboxylic acid	174.150	-4.3927
1-Deoxy-L-rhamnojirimycin	147.170	-4.3624
2-(carbamoylamino)-3-phenylpropanoic acid	208.210	-4.2849
2-Acetamido-1,2-Dideoxynojirmycin	204.220	-4.1896
octahydroindolizine-1,6,7,8-tetrol	189.210	-3.9737

Table S27. VS1 and VS2 for LH1 FDA Approved drugs targeting POLQ helicase site

Drug Name	MolWt	Energy in silico
VS1		
Proanthocyanidins	594.520	-9.6747
VS2		
Lopinavir	628.800	-8.3099
Pentagastrin	767.890	-8.1946
Troxerutin	742.670	-8.0476
Thymopentin	679.560	-7.8233
Indinavir sulfate	711.870	-7.6869
Velpatasvir	883.020	-7.6723
Salvianolic acid B	718.590	-7.5763
Lifitegrast	615.480	-7.5276
Salvianolic acid B	718.590	-7.4921
Velpatasvir	883.020	-7.4696
ABT199	868.440	-7.4338
Indinavir sulfate	711.870	-7.4121

ABT199	868.440	-7.3899
Thymopentin	679.560	-7.3517
Velpatasvir	883.020	-7.2977
Lopinavir	628.800	-7.2943
Atracurium besylate	1243.490	-7.2359
Indinavir sulfate	711.870	-7.2346
Indinavir sulfate	711.870	-7.2116
Indinavir sulfate	711.870	-7.2009
ABT199	868.440	-7.1883
Lifitegrast	615.480	-7.1255
Troxerutin	742.670	-7.1041
Lifitegrast	615.480	-7.083
Proanthocyanidins	594.520	-7.0793
Troxerutin	742.670	-7.078
Lapatinib ditosylate monohydrate	943.470	-7.0696
Lapatinib Ditosylate	925.460	-7.0687
Lapatinib	581.060	-7.0687
Lapatinib ditosylate monohydrate	943.470	-7.0575
Lapatinib Ditosylate	925.460	-7.0542
Lapatinib	581.060	-7.0542
Grazoprevir	766.900	-7.0392
Atracurium besylate	1243.490	-7.0308
ABT199	868.440	-7.0133
Isavuconazonium sulfate	814.840	-7.0087
Salvianolic acid B	718.590	-7.007
Lapatinib ditosylate monohydrate	943.470	-6.9981
Lapatinib Ditosylate	925.460	-6.9972
Lapatinib	581.060	-6.9972
Proanthocyanidins	594.520	-6.9887
ABT199	868.440	-6.9826
Indinavir sulfate	711.870	-6.9681
Indinavir sulfate	711.870	-6.9119
Cefotiam Hexetil Hydrochloride	768.765	-6.9113
Indinavir sulfate	711.870	-6.9091
Elbasvir	882.020	-6.9075
Asunaprevir	748.290	-6.8875
Daclatasvir	738.880	-6.8664
Elbasvir	882.020	-6.847
Elbasvir	882.020	-6.845
Daclatasvir	738.880	-6.8322
Indinavir sulfate	711.870	-6.8097
Atracurium besylate	1243.490	-6.8066
Indinavir sulfate	711.870	-6.7963

Deferoxamine Mesylate	656.790	-6.782
Grazoprevir	766.900	-6.7758
Proanthocyanidins	594.520	-6.772
Levomefolate calcium	497.520	-6.7606
Levomefolate calcium	497.520	-6.7566
Novobiocin Sodium	634.610	-6.7414
Isavuconazonium sulfate	814.840	-6.7316
Indinavir sulfate	711.870	-6.7267
Elbasvir	882.020	-6.7262
Cefotiam Hexetil Hydrochloride	768.765	-6.725
Indinavir sulfate	711.870	-6.7187
Atracurium besylate	1243.490	-6.6957
Levomefolate calcium	497.520	-6.6702
Indinavir sulfate	711.870	-6.6677
Bosentan	551.610	-6.6631
Pentagastrin	767.890	-6.6616
Indinavir sulfate	711.870	-6.6546
Levomefolate calcium	497.520	-6.6496
ABT199	868.440	-6.6443
Indinavir sulfate	711.870	-6.6301
Deferoxamine Mesylate	656.790	-6.627
Levomefolate calcium	497.520	-6.6157
Indinavir sulfate	711.870	-6.6107
Velpatasvir	883.020	-6.6079
Elbasvir	882.020	-6.6063
Atracurium besylate	1243.490	-6.6007
Casanthranol	396.580	-6.5936
Indinavir sulfate	711.870	-6.5908
Methyl hesperidin	624.590	-6.5866
Mitoxantrone	444.480	-6.5771
Mitoxantrone hydrochloride	517.400	-6.5759
Isavuconazonium sulfate	814.840	-6.5746
Indinavir sulfate	711.870	-6.5737
Velpatasvir	883.020	-6.5552
Thymopentin	679.560	-6.5416
Travoprost	500.550	-6.5413
Velpatasvir	883.020	-6.5361
Fulvestrant	606.770	-6.5325
Cefoperazone	645.670	-6.5299
Indinavir sulfate	711.870	-6.5226
Proanthocyanidins	594.520	-6.5115
Hesperidin	610.560	-6.4974
Mitoxantrone hydrochloride	517.400	-6.4946

Isavuconazonium sulfate	814.840	-6.4942
Mitoxantrone	444.480	-6.491
Elbasvir	882.020	-6.4853
Atazanavir	704.870	-6.48
Atazanavir sulfate	802.930	-6.48
Indinavir sulfate	711.870	-6.459
Casanthranol	396.580	-6.4583
Grazoprevir	766.900	-6.4501
Indinavir sulfate	711.870	-6.4471
Reserpine	608.680	-6.4363
Levomefolate calcium	497.520	-6.4354
Ademetionine	398.440	-6.431
Haloperidol	375.870	-6.4291
Levomefolate calcium	497.520	-6.4286
Isavuconazonium sulfate	814.840	-6.4263
Cefoperazone	645.670	-6.4261
Mitoxantrone hydrochloride	517.400	-6.4261
Mitoxantrone	444.480	-6.4255
Indinavir sulfate	711.870	-6.4233
Levomefolate calcium	497.520	-6.4213
Casanthranol	396.580	-6.4194
Ertapenem sodium	497.502	-6.4187
Levomefolate calcium	497.520	-6.4174
Tanshinone IIA sulfonate sodium	396.390	-6.4159
Indinavir sulfate	711.870	-6.4141
Elbasvir	882.020	-6.4132
Indinavir sulfate	711.870	-6.4033
Nelfinavir Mesylate	663.890	-6.4024
Travoprost	500.550	-6.3982
Inositol nicotinate	810.730	-6.3911
Indinavir sulfate	711.870	-6.3895
Velpatasvir	883.020	-6.385
Fexofenadine hydrochloride	538.120	-6.3801
Proanthocyanidins	594.520	-6.3671
Indinavir sulfate	711.870	-6.3659
Telaprevir	679.850	-6.3577
Velpatasvir	883.020	-6.349
Indinavir sulfate	711.870	-6.3464
Capastat sulfate	752.760	-6.342
Cefoperazone	645.670	-6.3416
Pazopanib	437.520	-6.3397
Pazopanib HCl	473.980	-6.3394
Indinavir sulfate	711.870	-6.3308

Daclatasvir	738.880	-6.3265
Indinavir sulfate	711.870	-6.326
Indinavir sulfate	711.870	-6.323
Velpatasvir	883.020	-6.3163
Indinavir sulfate	711.870	-6.3069
Proanthocyanidins	594.520	-6.302
Indinavir sulfate	711.870	-6.2965
Nelfinavir Mesylate	663.890	-6.2881
Cefpiramide acid	612.640	-6.2872
Nelfinavir Mesylate	663.890	-6.2871
Indinavir sulfate	711.870	-6.2837
Travoprost	500.550	-6.2836
Ledipasvir acetone	947.080	-6.2799
Indinavir sulfate	711.870	-6.2781
Indinavir sulfate	711.870	-6.2764
Doxorubicin hydrochloride	579.990	-6.2748
Nelfinavir Mesylate	663.890	-6.2745
Indinavir sulfate	711.870	-6.2695
Isavuconazonium sulfate	814.840	-6.2694
Dabigatran etexilate	627.730	-6.2688
Thymopentin	679.560	-6.2676
Indinavir sulfate	711.870	-6.2673
Tenofovir Disoproxil	519.440	-6.2654
Deferoxamine Mesylate	656.790	-6.2618
Levomefolate calcium	497.520	-6.2534
Pentagastrin	767.890	-6.2461
Indinavir sulfate	711.870	-6.2418
Travoprost	500.550	-6.2411
Posaconazole	700.780	-6.2403
Epirubicin hydrochloride	579.980	-6.2396
Silymarin	482.440	-6.2391
Nelfinavir Mesylate	663.890	-6.2388
Indinavir sulfate	711.870	-6.2378
Travoprost	500.550	-6.2314
Proanthocyanidins	594.520	-6.2295
Daclatasvir dihydrochloride	811.800	-6.2284
Capastat sulfate	752.760	-6.2256
Itraconazole	705.630	-6.2236
Isavuconazonium sulfate	814.840	-6.219
Proanthocyanidins	594.520	-6.2103
Casanthranol	396.580	-6.2049
Proanthocyanidins	594.520	-6.1986
Indinavir sulfate	711.870	-6.1958

Proanthocyanidins	594.520	-6.1909
Trazodone hydrochloride	408.330	-6.1893
Proanthocyanidins	594.520	-6.1884
Indinavir sulfate	711.870	-6.1833
Fexofenadine hydrochloride	538.120	-6.1828
Levomefolate calcium	497.520	-6.1803
Levomefolate calcium	497.520	-6.1735
Anlotinib Dihydrochloride	480.360	-6.1725
Anlotinib	407.440	-6.1725
Bosentan	551.610	-6.1718
Travoprost	500.550	-6.1672
Travoprost	500.550	-6.1641
Indinavir sulfate	711.870	-6.1639
Ertapenem sodium	497.502	-6.1637
Indinavir sulfate	711.870	-6.1618
Elagolix sodium	653.570	-6.1608
Ledipasvir acetone	947.080	-6.1603
Indinavir sulfate	711.870	-6.1597
Bimatoprost	415.570	-6.1564
Indinavir sulfate	711.870	-6.1487
Ertapenem sodium	497.502	-6.1472
Silymarin	482.440	-6.1437
Indinavir sulfate	711.870	-6.1396
Cefotiam hydrochloride	562.080	-6.1376
Proanthocyanidins	594.520	-6.1357
Proanthocyanidins	594.520	-6.1334
Tozasertib	464.590	-6.1309
Indinavir sulfate	711.870	-6.129
Adefovir dipivoxil	501.470	-6.1284
Imidapril HCl	441.910	-6.1231
Velpatasvir	883.020	-6.123
Palbociclib Isethionate	573.660	-6.1202
Palbociclib hydrochloride	483.990	-6.1202
Palbociclib	447.540	-6.1202
Indinavir sulfate	711.870	-6.1169
Indinavir sulfate	711.870	-6.1114
Indinavir sulfate	711.870	-6.1107
Epirubicin hydrochloride	579.980	-6.1078
Adefovir dipivoxil	501.470	-6.1017
Thymopentin	679.560	-6.0973
Posaconazole	700.780	-6.097
Irinotecan	586.680	-6.0959
Indinavir sulfate	711.870	-6.0958

Telaprevir	679.850	-6.0957
Irinotecan hydrochloride trihydrate	677.180	-6.0926
Levomefolate calcium	497.520	-6.0885
Casanthranol	396.580	-6.0757
Indinavir sulfate	711.870	-6.0737
Cefpiramide acid	612.640	-6.0709
Ledipasvir acetone	947.080	-6.0701
Cefotiam hydrochloride	562.080	-6.0654
Haloperidol	375.870	-6.0648
Indinavir sulfate	711.870	-6.0633
Indinavir sulfate	711.870	-6.061
Cefotiam hydrochloride	562.080	-6.061
Indinavir sulfate	711.870	-6.0564
Indinavir sulfate	711.870	-6.0558
Indinavir sulfate	711.870	-6.0548
Pixantrone dimaleate	557.210	-6.0539
Casanthranol	396.580	-6.0432
Indinavir sulfate	711.870	-6.0372
Amikacin	585.600	-6.0324
Indinavir sulfate	711.870	-6.0305
Indinavir sulfate	711.870	-6.0276
Indinavir sulfate	711.870	-6.0271
Proanthocyanidins	594.520	-6.0231
Casanthranol	396.580	-6.0167
Ivosidenib	582.970	-6.0031
Velpatasvir	883.020	-5.9979
Indinavir sulfate	711.870	-5.9849
Proanthocyanidins	594.520	-5.9818
Travoprost	500.550	-5.9816
Tanshinone IIA sulfonate sodium	396.390	-5.9811
Indinavir sulfate	711.870	-5.9802
Indinavir sulfate	711.870	-5.9744
Dapagliflozin ((2S)-1,2-propanediol, hydrate)	502.980	-5.9701
Aliskiren hemifumarate	1219.600	-5.9697
Dapagliflozin	408.870	-5.9671
Indinavir sulfate	711.870	-5.9662
Indinavir sulfate	711.870	-5.9638
Indinavir sulfate	711.870	-5.9447
Levomefolate calcium	497.520	-5.9442
Cefpiramide acid	612.640	-5.9428
Posaconazole	700.780	-5.9423
Itraconazole	705.630	-5.9412
Capastat sulfate	752.760	-5.9332

Tanshinone I	276.290	-5.9277
Indinavir sulfate	711.870	-5.9272
Pentamidine isethionate	592.680	-5.9252
Amikacin Sulfate Salt	683.680	-5.9224
Felypressin	1040.220	-5.9204
Azelnidipine	582.650	-5.916
Casanthranol	396.580	-5.9157
Indinavir sulfate	711.870	-5.9066
Argatroban	508.630	-5.9062
Imidapril HCl	441.910	-5.9045
Methyl hesperidin	624.590	-5.9012
Indinavir sulfate	711.870	-5.9002
Capastat sulfate	752.760	-5.8968
Itraconazole	705.630	-5.8939
Casanthranol	396.580	-5.8914
Casanthranol	396.580	-5.8898
Casanthranol	396.580	-5.8884
Proanthocyanidins	594.520	-5.8879
Proanthocyanidins	594.520	-5.8861
Tenofovir Disoproxil Fumarate	635.510	-5.8771
Gliquidone	527.630	-5.8728
Amikacin	585.600	-5.8715
Epirubicin hydrochloride	579.980	-5.8715
Hesperidin	610.560	-5.867
Amikacin	585.600	-5.8592
Cefotiam Hexetil Hydrochloride	768.765	-5.8576
Thymopentin	679.560	-5.8552
Indinavir sulfate	711.870	-5.852
Amikacin Sulfate Salt	683.680	-5.8515
Famprofazone	377.520	-5.8457
Casanthranol	396.580	-5.8385
Itraconazole	705.630	-5.8338
Amikacin Sulfate Salt	683.680	-5.8321
Travoprost	500.550	-5.8311
Ipragliflozin	404.450	-5.8293
Ademetionine	398.440	-5.8287
Empagliflozin	450.910	-5.8271
Indinavir sulfate	711.870	-5.8252
Elagolix sodium	653.570	-5.8245
Odanacatib	525.560	-5.8203
Indinavir sulfate	711.870	-5.8107
Fruquintinib	393.390	-5.8104
D-Pantethine	554.720	-5.8081

Indinavir sulfate	711.870	-5.8045
Levomefolate calcium	497.520	-5.8005
Proanthocyanidins	594.520	-5.8004
Propafenone	341.440	-5.7878
Indinavir sulfate	711.870	-5.7856
Pazopanib HCl	473.980	-5.7843
Pazopanib	437.520	-5.7834
Dofetilide	441.560	-5.7831
Indinavir sulfate	711.870	-5.783
Indinavir sulfate	711.870	-5.7825
Casanthranol	396.580	-5.7816
Cefsulodine sodium	554.520	-5.7765
Ademetionine	398.440	-5.7728
Travoprost	500.550	-5.7706
Cefoselis Sulfate	620.640	-5.7691
Proanthocyanidins	594.520	-5.7612
Temocapil	476.600	-5.7605
Elagolix sodium	653.570	-5.7552
Proanthocyanidins	594.520	-5.7551
Proanthocyanidins	594.520	-5.7478
Propafenone	341.440	-5.7456
Silibinin	482.440	-5.7447
Temocapil	476.600	-5.7416
Sparfloxacin	392.400	-5.739
Cinoxacin	262.220	-5.7386
Indinavir sulfate	711.870	-5.7357
Indinavir sulfate	711.870	-5.7343
Ivosidenib	582.970	-5.7336
Bictegravir	449.380	-5.7333
Casanthranol	396.580	-5.7329
Indinavir sulfate	711.870	-5.7327
Oxolinic acid	261.240	-5.7319
Treprostinil Sodium	412.500	-5.7264
Puerarin	416.380	-5.7254
Indinavir sulfate	711.870	-5.7252
Metolazone	365.830	-5.7227
Bictegravir	449.380	-5.7223
Tenofovir alafenamide	476.470	-5.7222
Doripenem Hydrate	438.520	-5.7189
Casanthranol	396.580	-5.7152
Indinavir sulfate	711.870	-5.7141
Cefotetan	575.620	-5.7123
Daclatasvir dihydrochloride	811.800	-5.7107

Flumazenil	303.290	-5.7088
Levomefolate calcium	497.520	-5.6991
Cefsulodine sodium	554.520	-5.696
Proanthocyanidins	594.520	-5.6949
Levomefolate calcium	497.520	-5.6864
Biapenem	350.390	-5.6822
Capastat sulfate	752.760	-5.6817
Casanthranol	396.580	-5.6814
Proanthocyanidins	594.520	-5.6778
Indinavir sulfate	711.870	-5.6777
Levofloxacin	361.370	-5.6772
Levofloxacin Hemihydrate	370.380	-5.6772
Treprostinil Sodium	412.500	-5.6772
Ofloxacin	361.370	-5.6757
Odanacatib	525.560	-5.6742
Indinavir sulfate	711.870	-5.6702
Fupentixol Dihydrochloride	507.430	-5.6664
Vemurafenib	489.920	-5.6657
Indinavir sulfate	711.870	-5.6562
Indinavir sulfate	711.870	-5.6538
Capastat sulfate	752.760	-5.6521
Diosmin	608.540	-5.6408
Ledipasvir acetone	947.080	-5.6401
Odanacatib	525.560	-5.6396
Proanthocyanidins	594.520	-5.6313
Levomefolate calcium	497.520	-5.6249
Ivosidenib	582.970	-5.6215
Levomefolate calcium	497.520	-5.6173
Rutin	610.520	-5.6135
Cefpirome sulfate	612.660	-5.6113
Ademetionine	398.440	-5.6063
Latamoxef sodium	564.440	-5.5944
Ofloxacin	361.370	-5.5931
Tenofovir alafenamide	476.470	-5.5927
Proanthocyanidins	594.520	-5.5891
Indinavir sulfate	711.870	-5.5851
Olprinone	250.260	-5.5841
Meticrane	275.340	-5.5798
Ademetionine	398.440	-5.5718
Indinavir sulfate	711.870	-5.5605
Trioxsalen	228.250	-5.5584
Folic acid	441.400	-5.5524
Levomefolate calcium	497.520	-5.5496

Indinavir sulfate	711.870	-5.5489
Ertapenem sodium	497.502	-5.5458
Levomefolate calcium	497.520	-5.5452
Latamoxef sodium	564.440	-5.5442
Cefotiam Hexetil Hydrochloride	768.765	-5.5432
Isepamicin sulfate	667.680	-5.5409
Cinoxacin	262.220	-5.5393
Biapenem	350.390	-5.5375
Felypressin	1040.220	-5.5361
Nalidixic acid sodium salt	254.220	-5.5348
Nalidixic acid	232.240	-5.5348
Itraconazole	705.630	-5.5272
Doripenem Hydrate	438.520	-5.5268
Spectinomycin dihydrochloride	405.270	-5.5259
Neomycin sulfate	908.870	-5.5179
Argatroban	508.630	-5.5179
Cefonicid sodium	586.530	-5.5123
Latamoxef sodium	564.440	-5.5107
Indinavir sulfate	711.870	-5.5081
Oxolinic acid	261.240	-5.5076
Enzalutamide	464.440	-5.5076
R788 Disodium	624.420	-5.5073
8-Methoxypsoralen	216.190	-5.5015
Levomefolate calcium	497.520	-5.5007
Argatroban	508.630	-5.4993
Eburnalritardo	294.390	-5.4908
Proanthocyanidins	594.520	-5.4876
Neomycin sulfate	908.870	-5.4841
Fostamatinib	580.460	-5.4812
Neomycin Sulphate B	712.220	-5.4786
Neomycin Sulphate B	712.220	-5.4769
Esomeprazole Sodium	367.400	-5.4763
Levomefolate calcium	497.520	-5.4742
Isavuconazonium sulfate	814.840	-5.4623
Indinavir sulfate	711.870	-5.4594
Esomeprazole Sodium	367.400	-5.4592
Elagolix sodium	653.570	-5.4541
Atazanavir	704.870	-5.4534
Atazanavir sulfate	802.930	-5.4534
Propafenone	341.440	-5.4486
Argatroban	508.630	-5.4482
Scoparone	206.200	-5.444
Lonidamine	321.170	-5.438

Norepinephrine	169.180	-5.4377
Noradrenaline bitartrate	319.260	-5.4377
Proanthocyanidins	594.520	-5.4353
Sanguinarine chloride	367.780	-5.4297
Norepinephrine	169.180	-5.428
Noradrenaline bitartrate	319.260	-5.428
Nalidixic acid sodium salt	254.220	-5.4259
Nalidixic acid	232.240	-5.4259
Dipivefrin hydrochloride	387.900	-5.4176
Levomefolate calcium	497.520	-5.4175
Proanthocyanidins	594.520	-5.4169
Capastat sulfate	752.760	-5.4049
Ramatroban	416.470	-5.397
Folic acid	441.400	-5.3952
Proanthocyanidins	594.520	-5.3947
Doripenem Hydrate	438.520	-5.3747
Biapenem	350.390	-5.3637
Trapidil	205.260	-5.3605
Latamoxef sodium	564.440	-5.3587
Oxcarbazepine	252.270	-5.3529
Glucosamine hydrochloride	215.630	-5.3493
Methotrexate	454.440	-5.3387
Fosinopril sodium	586.650	-5.3372
Fupentixol Dihydrochloride	507.430	-5.337
Moexipril HCl	535.030	-5.3299
Reserpine	608.680	-5.3289
Biapenem	350.390	-5.3242
Levomefolate calcium	497.520	-5.3197
Indinavir sulfate	711.870	-5.3142
Cefoxitin sodium	449.430	-5.3099
Isavuconazonium sulfate	814.840	-5.3023
Indinavir sulfate	711.870	-5.3018
Anagrelide HCl	292.550	-5.299
Anagrelide	256.090	-5.299
Norepinephrine	169.180	-5.294
Noradrenaline bitartrate	319.260	-5.294
Asunaprevir	748.290	-5.2803
Telaprevir	679.850	-5.2702
Ramatroban	416.470	-5.2691
Isepamicin sulfate	667.680	-5.2682
Indinavir sulfate	711.870	-5.2569
Atazanavir	704.870	-5.2416
Atazanavir sulfate	802.930	-5.2416

Levomefolate calcium	497.520	-5.2347
Menadione sodium bisulfite	276.240	-5.2187
Glucosamine hydrochloride	215.630	-5.2173
Mepiroxol	125.130	-5.2166
Kasugamycin hydrochloride	415.830	-5.2085
Indinavir sulfate	711.870	-5.2077
Amstat	157.210	-5.2036
Bestatin hydrochloride	344.840	-5.2
Ubenimex	308.370	-5.2
Isepamicin sulfate	667.680	-5.1964
Proanthocyanidins	594.520	-5.191
Lopinavir	628.800	-5.1772
Carbazochrome sodium sulfonate	376.320	-5.1494
Neomycin Sulphate B	712.220	-5.145
Diflunisal	250.200	-5.1184
Neomycin sulfate	908.870	-5.1174
Saxagliptin hydrate	333.430	-5.1134
Glucosamine hydrochloride	215.630	-5.108
Argatroban	508.630	-5.1038
Spectinomycin dihydrochloride	405.270	-5.09
Hydroxychloroquine sulfate	433.950	-5.0684
Saxagliptin hydrate	333.430	-5.0628
5-Azacytidine	244.200	-5.058
Indinavir sulfate	711.870	-5.0485
Amifostine	214.220	-5.0475
Proanthocyanidins	594.520	-5.0413
Neomycin Sulphate B	712.220	-5.0367
Neomycin sulfate	908.870	-5.0362
Decitabine	228.210	-5.0309
Doripenem Hydrate	438.520	-5.0288
Amifostine	214.220	-5.0245
Doripenem Hydrate	438.520	-5.014
Dacarbazine	182.180	-5.0104
Sennoside A	862.740	-4.989
Esomeprazole Magnesium	713.120	-4.9816
Fosinopril sodium	586.650	-4.9762
Nicotinic acid N-oxide	139.110	-4.9678
Spectinomycin dihydrochloride	405.270	-4.9558
Felypressin	1040.220	-4.9356
Geniposidic Acid	374.340	-4.8872
L-5-Hydroxytryptophan	220.220	-4.8843
Dacarbazine	182.180	-4.8841
Acipimox	154.130	-4.8736

Pyrantel pamoate	594.690	-4.8711
Amstat	157.210	-4.8651
Geniposidic Acid	374.340	-4.856
Decitabine	228.210	-4.8449
Dacarbazine	182.180	-4.84
Fosinopril sodium	586.650	-4.8234
Histamine 2HCl	184.070	-4.8223
Histamine	111.150	-4.8223
Prednisolone phosphate sodium	484.390	-4.8223
L-5-Hydroxytryptophan	220.220	-4.8093
Dacarbazine	182.180	-4.7507
Ceforanide	519.550	-4.7163
Pemetrexed acid	427.410	-4.6696
Pemetrexed disodium	471.370	-4.6663
Pemetrexed Disodium Hydrate	516.420	-4.6651
Strontium ranelate	513.490	-4.5898
Spectinomycin dihydrochloride	405.270	-4.5763
Amstat	157.210	-4.5539
Dacarbazine	182.180	-4.5162
Amifostine	214.220	-4.3852

Table S28. VS1 and VS2 for DSI-poised targeting POLQ allosteric site

Compound	MW	Catalog ID	Energy in silico
VS1			
DSI_poised_library_2023.maegz:739	247.240	Z27805986	-7.4617
VS2			
DSI_poised_library_2023.maegz:591	244.119	Z56792776	-7.265
DSI_poised_library_2023.maegz:739	247.240	Z27805986	-7.2521
DSI_poised_library_2023.maegz:777	267.127	Z352356442	-7.2517
DSI_poised_library_2023.maegz:36	239.131	Z1470724953	-7.2093
DSI_poised_library_2023.maegz:739	247.240	Z27805986	-7.2034
DSI_poised_library_2023.maegz:777	267.127	Z352356442	-7.1872
DSI_poised_library_2023.maegz:559	252.126	Z332725774	-7.1691
DSI_poised_library_2023.maegz:624	248.132	Z30620520	-7.1304
DSI_poised_library_2023.maegz:761	252.126	Z234898223	-7.1231
DSI_poised_library_2023.maegz:591	244.119	Z56792776	-7.1146
DSI_poised_library_2023.maegz:36	239.131	Z1470724953	-7.11
DSI_poised_library_2023.maegz:674	233.178	Z54508609	-7.0603
DSI_poised_library_2023.maegz:513	240.163	Z56618096	-7.0537
DSI_poised_library_2023.maegz:740	215.111	Z57329001	-7.0374
DSI_poised_library_2023.maegz:674	233.178	Z54508609	-7.0371

DSI_poised_library_2023.maegz:780	247.157	Z18839017	-7.0316
DSI_poised_library_2023.maegz:36	239.131	Z1470724953	-7.0191
DSI_poised_library_2023.maegz:628	240.106	Z124039706	-7.0119
DSI_poised_library_2023.maegz:36	239.131	Z1470724953	-7.0037
DSI_poised_library_2023.maegz:427	229.090	Z27749656	-6.9993
DSI_poised_library_2023.maegz:740	215.111	Z57329001	-6.9806
DSI_poised_library_2023.maegz:125	238.087	Z275165822	-6.9783
DSI_poised_library_2023.maegz:125	238.087	Z275165822	-6.9696
DSI_poised_library_2023.maegz:383	245.085	Z19735192	-6.9631
DSI_poised_library_2023.maegz:503	242.316	Z223388508	-6.9512
DSI_poised_library_2023.maegz:591	244.119	Z56792776	-6.9485
DSI_poised_library_2023.maegz:125	238.087	Z275165822	-6.9331
DSI_poised_library_2023.maegz:121	233.095	Z26333434	-6.9279
DSI_poised_library_2023.maegz:513	240.163	Z56618096	-6.9045
DSI_poised_library_2023.maegz:63	299.729	Z1004825200	-6.8949
DSI_poised_library_2023.maegz:740	215.111	Z57329001	-6.8948
DSI_poised_library_2023.maegz:674	233.178	Z54508609	-6.8933
DSI_poised_library_2023.maegz:36	239.131	Z1470724953	-6.8738
DSI_poised_library_2023.maegz:638	247.121	Z31432226	-6.8723
DSI_poised_library_2023.maegz:780	247.157	Z18839017	-6.8661
DSI_poised_library_2023.maegz:32	248.095	Z453319206	-6.8345
DSI_poised_library_2023.maegz:427	229.090	Z27749656	-6.8182
DSI_poised_library_2023.maegz:316	248.152	Z208334100	-6.803
DSI_poised_library_2023.maegz:777	267.127	Z352356442	-6.7899
DSI_poised_library_2023.maegz:436	248.228	Z44586758	-6.7716
DSI_poised_library_2023.maegz:300	249.092	Z2574937229	-6.7604
DSI_poised_library_2023.maegz:674	233.178	Z54508609	-6.7582
DSI_poised_library_2023.maegz:104	235.137	Z31484539	-6.7533
DSI_poised_library_2023.maegz:422	246.328	Z57080305	-6.7467
DSI_poised_library_2023.maegz:483	227.259	Z89307993	-6.7347
DSI_poised_library_2023.maegz:655	215.291	Z54756666	-6.7247
DSI_poised_library_2023.maegz:63	299.729	Z1004825200	-6.7173
DSI_poised_library_2023.maegz:777	267.127	Z352356442	-6.7171
DSI_poised_library_2023.maegz:674	233.178	Z54508609	-6.7156
DSI_poised_library_2023.maegz:245	241.110	Z33452549	-6.7021
DSI_poised_library_2023.maegz:778	245.085	Z1250129028	-6.6995
DSI_poised_library_2023.maegz:22	245.241	Z168902372	-6.6994
DSI_poised_library_2023.maegz:825	223.101	Z19735067	-6.6987
DSI_poised_library_2023.maegz:674	233.178	Z54508609	-6.6902
DSI_poised_library_2023.maegz:457	226.091	Z26333448	-6.6811
DSI_poised_library_2023.maegz:383	245.085	Z19735192	-6.6794
DSI_poised_library_2023.maegz:313	245.340	Z28429411	-6.6754
DSI_poised_library_2023.maegz:291	237.119	Z29191465	-6.6734

DSI_poised_library_2023.maegz:63	299.729	Z1004825200	-6.6725
DSI_poised_library_2023.maegz:62	243.301	Z32665176	-6.654
DSI_poised_library_2023.maegz:657	244.264	Z44609285	-6.6511
DSI_poised_library_2023.maegz:761	252.126	Z234898223	-6.6451
DSI_poised_library_2023.maegz:22	245.241	Z168902372	-6.6358
DSI_poised_library_2023.maegz:780	247.157	Z18839017	-6.6346
DSI_poised_library_2023.maegz:201	249.119	Z19751622	-6.6335
DSI_poised_library_2023.maegz:400	223.101	Z1627772104	-6.6329
DSI_poised_library_2023.maegz:457	226.091	Z26333448	-6.632
DSI_poised_library_2023.maegz:657	244.264	Z44609285	-6.6257
DSI_poised_library_2023.maegz:48	242.106	Z58982727	-6.6255
DSI_poised_library_2023.maegz:671	238.112	Z192955056	-6.6047
DSI_poised_library_2023.maegz:121	233.095	Z26333434	-6.6044
DSI_poised_library_2023.maegz:769	219.137	Z1509441355	-6.5959
DSI_poised_library_2023.maegz:825	223.101	Z19735067	-6.593
DSI_poised_library_2023.maegz:844	249.136	Z17497990	-6.5897
DSI_poised_library_2023.maegz:126	239.131	Z1342868616	-6.5848
DSI_poised_library_2023.maegz:345	240.300	Z85525355	-6.5834
DSI_poised_library_2023.maegz:547	246.080	Z55290386	-6.5784
DSI_poised_library_2023.maegz:488	246.348	Z137811222	-6.5773
DSI_poised_library_2023.maegz:381	245.360	Z419884046	-6.5742
DSI_poised_library_2023.maegz:136	234.226	Z44584202	-6.5723
DSI_poised_library_2023.maegz:62	243.301	Z32665176	-6.5674
DSI_poised_library_2023.maegz:560	227.062	Z133716556	-6.5654
DSI_poised_library_2023.maegz:503	242.316	Z223388508	-6.5637
DSI_poised_library_2023.maegz:136	234.226	Z44584202	-6.5619
DSI_poised_library_2023.maegz:36	239.131	Z1470724953	-6.5619
DSI_poised_library_2023.maegz:769	219.137	Z1509441355	-6.5612
DSI_poised_library_2023.maegz:22	245.241	Z168902372	-6.5571
DSI_poised_library_2023.maegz:563	241.067	Z2737076969	-6.5514
DSI_poised_library_2023.maegz:643	253.098	Z381033368	-6.5508
DSI_poised_library_2023.maegz:522	248.116	Z274555794	-6.5468
DSI_poised_library_2023.maegz:316	248.152	Z208334100	-6.5399
DSI_poised_library_2023.maegz:522	248.116	Z274555794	-6.5352
DSI_poised_library_2023.maegz:593	246.173	Z90503851	-6.5332
DSI_poised_library_2023.maegz:541	244.078	Z1267773765	-6.5265
DSI_poised_library_2023.maegz:777	267.127	Z352356442	-6.5243
DSI_poised_library_2023.maegz:506	222.153	Z334942570	-6.5153
DSI_poised_library_2023.maegz:104	235.137	Z31484539	-6.5132
DSI_poised_library_2023.maegz:739	247.240	Z27805986	-6.5116
DSI_poised_library_2023.maegz:59	223.112	Z198194394	-6.5095
DSI_poised_library_2023.maegz:777	267.127	Z352356442	-6.507
DSI_poised_library_2023.maegz:172	204.126	Z1206890404	-6.5062

DSI_poised_library_2023.maegz:436	248.228	Z44586758	-6.5059
DSI_poised_library_2023.maegz:316	248.152	Z208334100	-6.5044
DSI_poised_library_2023.maegz:59	223.112	Z198194394	-6.5043
DSI_poised_library_2023.maegz:547	246.080	Z55290386	-6.5036
DSI_poised_library_2023.maegz:851	239.276	Z56983806	-6.5008
DSI_poised_library_2023.maegz:338	298.115	Z227950824	-6.4745
DSI_poised_library_2023.maegz:330	233.142	Z419995480	-6.4717
DSI_poised_library_2023.maegz:593	246.173	Z90503851	-6.4711
DSI_poised_library_2023.maegz:643	253.098	Z381033368	-6.4697
DSI_poised_library_2023.maegz:30	220.067	Z1134990241	-6.4686
DSI_poised_library_2023.maegz:470	245.178	Z30162334	-6.4677
DSI_poised_library_2023.maegz:483	227.259	Z89307993	-6.4657
DSI_poised_library_2023.maegz:22	245.241	Z168902372	-6.4654
DSI_poised_library_2023.maegz:59	223.112	Z198194394	-6.4637
DSI_poised_library_2023.maegz:777	267.127	Z352356442	-6.4575
DSI_poised_library_2023.maegz:15	228.333	Z131833926	-6.451
DSI_poised_library_2023.maegz:769	219.137	Z1509441355	-6.439
DSI_poised_library_2023.maegz:121	233.095	Z26333434	-6.4385
DSI_poised_library_2023.maegz:402	237.127	Z57299529	-6.4368
DSI_poised_library_2023.maegz:63	299.729	Z1004825200	-6.4357
DSI_poised_library_2023.maegz:337	201.127	Z168801722	-6.4198
DSI_poised_library_2023.maegz:809	404.399	Z3015133857	-6.4182
DSI_poised_library_2023.maegz:574	229.278	Z57715447	-6.4176
DSI_poised_library_2023.maegz:643	253.098	Z381033368	-6.4173
DSI_poised_library_2023.maegz:381	245.360	Z419884046	-6.4099
DSI_poised_library_2023.maegz:470	245.178	Z30162334	-6.4087
DSI_poised_library_2023.maegz:227	224.083	Z1220645147	-6.4006
DSI_poised_library_2023.maegz:309	209.217	Z1730522163	-6.3918
DSI_poised_library_2023.maegz:337	201.127	Z168801722	-6.3847
DSI_poised_library_2023.maegz:470	245.178	Z30162334	-6.3828
DSI_poised_library_2023.maegz:62	243.301	Z32665176	-6.3821
DSI_poised_library_2023.maegz:563	241.067	Z2737076969	-6.3817
DSI_poised_library_2023.maegz:541	244.078	Z1267773765	-6.3801
DSI_poised_library_2023.maegz:824	220.067	Z56767623	-6.3771
DSI_poised_library_2023.maegz:593	246.173	Z90503851	-6.3717
DSI_poised_library_2023.maegz:22	245.241	Z168902372	-6.3651
DSI_poised_library_2023.maegz:419	235.078	Z57160355	-6.3603
DSI_poised_library_2023.maegz:718	251.280	Z55222357	-6.3468
DSI_poised_library_2023.maegz:278	241.010	Z1551999220	-6.3027
DSI_poised_library_2023.maegz:208	189.077	Z56960214	-6.2922
DSI_poised_library_2023.maegz:330	233.142	Z419995480	-6.2887
DSI_poised_library_2023.maegz:291	237.119	Z29191465	-6.2803
DSI_poised_library_2023.maegz:28	265.111	Z85893853	-6.274

DSI_poised_library_2023.maegz:725	209.328	Z1275599911	-6.2452
DSI_poised_library_2023.maegz:378	206.142	Z332382842	-6.2318
DSI_poised_library_2023.maegz:504	247.157	Z31480458	-6.2235
DSI_poised_library_2023.maegz:208	189.077	Z56960214	-6.2027
DSI_poised_library_2023.maegz:503	242.316	Z223388508	-6.164
DSI_poised_library_2023.maegz:750	176.095	Z1192341021	-6.1329
DSI_poised_library_2023.maegz:274	237.128	Z57821475	-6.1013
DSI_poised_library_2023.maegz:706	225.173	Z31721798	-6.1003
DSI_poised_library_2023.maegz:432	277.243	Z364577298	-6.0988
DSI_poised_library_2023.maegz:68	203.106	Z26251905	-6.0316
DSI_poised_library_2023.maegz:741	189.026	Z1295863442	-6.0012
DSI_poised_library_2023.maegz:303	210.017	Z54628157	-5.9711
DSI_poised_library_2023.maegz:399	238.326	Z32399948	-5.9398
DSI_poised_library_2023.maegz:337	201.127	Z168801722	-5.9064

Table S29. VS1 and VS2 for PDB fragment library targeting POLQ allosteric site

Compound	Entry Name	MW	Energy in silico
VS1	8E24_clean.1		
1-Naphthylacetic acid	NLA.1	186.068	-2.804
VS2	8E24_clean.1		
nabumetone	NBO.1	228.115	-5.196
nabumetone	NBO.1	228.115	-4.895
[1-(4-fluorophenyl)-5-methyl-4-pyrazolyl]methanol	FMQ.1	206.086	-4.769
1, 3-Diphenylurea	BSU.1	212.095	-4.724
tolfenamic acid	TLF.1	261.056	-4.671
tolfenamic acid	TLF.1	261.056	-4.663
4'-Hydroxyflavanone	DFL.1	240.079	-4.642
4'-Hydroxyflavanone	DFL.1	240.079	-4.638
Mefenamic acid	ID8.1		-4.628
Mefenamic acid	ID8.1		-4.601
[1-(4-fluorophenyl)-5-methyl-4-pyrazolyl]methanol	FMQ.1	206.086	-4.484
4'-Hydroxyflavanone	DFL.1	240.079	-4.47
4'-Hydroxyflavanone	DFL.1	240.079	-4.469
5-Bromo-2-(piperidin-3-ylamino)benzoic acid	AJD.1		-4.05
4'-Hydroxyflavanone	DFL.1	240.079	-4.045
octahydroindolizine-1,6,7,8-tetrol	CTS.1	189.100	-4.04
L-tryptophanamide	LTN.1	203.106	-4.038
octahydroindolizine-1,6,7,8-tetrol	CTS.1	189.100	-3.924
5'_deoxy_adenosine	5AD.1	251.102	-3.913
Caffeine	CFF.1	194.080	-3.857

Coumarin	COU.1	146.037	-3.829
Coumarin	COU.1	146.037	-3.563
N-(4-Sulfamoylphenethyl)acetamide	M25.1	242.073	-3.556
Difunisal	1FL.1	250.044	-3.437
0-phenantroline	PHN.1		-3.263
Difunisal	1FL.1	250.044	-3.25
Difunisal	1FL.1	250.044	-3.235
5-Chlorobenzo[b]thiophene-3-acetic acid	3N2.1	225.986	-3.206
0-phenantroline	PHN.1		-3.201
6-Hydroxy-2-benzothiazolesulfonamide	ZEC.1	229.982	-3.158
2-Naphthimidamide	2UP.1	170.084	-3.087
1-Naphthylacetic acid	NLA.1	186.068	-2.976
3,4-Diaminobenzotrifluoride	X0V.1	176.056	-2.885
3,4-Diaminobenzotrifluoride	X0V.1	176.056	-2.871
3,4-Diaminobenzotrifluoride	X0V.1	176.056	-2.824
5-Chlorobenzo[b]thiophene-3-acetic acid	3N2.1	225.986	-2.636
1-Naphthylacetic acid	NLA.1	186.068	-2.463

Table S30. VS1 and VS2 for LH1 FDA Approved drugs targeting POLQ allosteric site

Drug Name	MolWt	Energy in silico
VS1		
Indinavir sulfate	711.870	-8.9587
VS2		
Fluoxetine hydrochloride	345.790	-8.3074
Fluoxetine	309.330	-8.3074
Indinavir sulfate	711.870	-8.2275
Indinavir sulfate	711.870	-8.0615
Dinaciclib	396.490	-7.9696
Bifonazole	310.390	-7.9493
Bifonazole	310.390	-7.9174
Talniflumate	414.330	-7.9061
Indinavir sulfate	711.870	-7.8977
Fluoxetine hydrochloride	345.790	-7.8398
Fluoxetine	309.330	-7.8398
Fluoxetine hydrochloride	345.790	-7.8287
Fluoxetine	309.330	-7.8287
Indinavir sulfate	711.870	-7.8141
Indinavir sulfate	711.870	-7.8139
Indinavir sulfate	711.870	-7.8119
Butenafine hydrochloride	353.930	-7.8111
Indinavir sulfate	711.870	-7.8067

Fluphenazine hydrochloride	510.440	-7.791
Talniflumate	414.330	-7.7856
Fluoxetine hydrochloride	345.790	-7.7728
Fluoxetine	309.330	-7.7728
Indinavir sulfate	711.870	-7.7363
Fluoxetine hydrochloride	345.790	-7.7278
Fluoxetine	309.330	-7.7278
Indinavir sulfate	711.870	-7.7173
Indinavir sulfate	711.870	-7.7033
Indinavir sulfate	711.870	-7.7009
Talniflumate	414.330	-7.6983
Indinavir sulfate	711.870	-7.6841
Indinavir sulfate	711.870	-7.6768
Indinavir sulfate	711.870	-7.6524
Propafenone	341.440	-7.6422
Indinavir sulfate	711.870	-7.619
Triflupromazine hydrochloride	388.880	-7.611
Lomitapide	692.710	-7.6009
Indinavir sulfate	711.870	-7.5949
Indinavir sulfate	711.870	-7.5937
Triflupromazine hydrochloride	388.880	-7.5926
Indinavir sulfate	711.870	-7.5795
Indinavir sulfate	711.870	-7.5693
Fluphenazine hydrochloride	510.440	-7.5649
voxelotor	337.370	-7.5635
Ondansetron hydrochloride	365.860	-7.5563
Fluphenazine hydrochloride	510.440	-7.5338
Glafenine Hydrochloride	409.260	-7.5005
Glafenine	372.800	-7.5005
Indinavir sulfate	711.870	-7.5002
Indinavir sulfate	711.870	-7.4983
Indinavir sulfate	711.870	-7.4835
Indinavir sulfate	711.870	-7.4754
Glafenine Hydrochloride	409.260	-7.4679
Glafenine	372.800	-7.4679
Indinavir sulfate	711.870	-7.4559
Indinavir sulfate	711.870	-7.4489
Indinavir sulfate	711.870	-7.4474
Triflupromazine hydrochloride	388.880	-7.4305
Indinavir sulfate	711.870	-7.4269
Indinavir sulfate	711.870	-7.4188
Clomipramine hydrochloride	351.310	-7.4178
Glafenine Hydrochloride	409.260	-7.4072

Glafenine	372.800	-7.4072
Indinavir sulfate	711.870	-7.4013
Indinavir sulfate	711.870	-7.3961
Indinavir sulfate	711.870	-7.3895
Tropicamide	284.360	-7.3892
Indinavir sulfate	711.870	-7.3872
Indinavir sulfate	711.870	-7.3843
Naftifine hydrochloride	323.860	-7.375
Indinavir sulfate	711.870	-7.3658
Glafenine Hydrochloride	409.260	-7.3584
Glafenine	372.800	-7.3584
Zaltoprofen	298.360	-7.3537
Indinavir sulfate	711.870	-7.3435
Indinavir sulfate	711.870	-7.3404
Azelastine hydrochloride	418.360	-7.3373
Indinavir sulfate	711.870	-7.3322
Indinavir sulfate	711.870	-7.3269
Butenafine hydrochloride	353.930	-7.3227
Chlorpromazine hydrochloride	355.320	-7.3195
Indinavir sulfate	711.870	-7.3122
Azelastine hydrochloride	418.360	-7.3015
Indinavir sulfate	711.870	-7.2985
Butenafine hydrochloride	353.930	-7.2951
Praziquantel	312.420	-7.2929
Indinavir sulfate	711.870	-7.2841
Indinavir sulfate	711.870	-7.2787
Indinavir sulfate	711.870	-7.277
Sertaconazole nitrate	500.780	-7.2737
Fluoxetine hydrochloride	345.790	-7.2621
Fluoxetine	309.330	-7.2621
Chrysophanic Acid	254.240	-7.2596
Glafenine Hydrochloride	409.260	-7.2561
Glafenine	372.800	-7.2561
Ezetimibe	409.430	-7.2456
Indinavir sulfate	711.870	-7.2425
Indinavir sulfate	711.870	-7.2266
Indinavir sulfate	711.870	-7.2186
Seratrodast	354.440	-7.1962
Indinavir sulfate	711.870	-7.1935
Indinavir sulfate	711.870	-7.181
Indinavir sulfate	711.870	-7.1798
Flurbiprofen Axetil	330.360	-7.1769
Ruxolitinib (INCB-18424) phosphate	404.360	-7.1766

Ruxolitinib	306.370	-7.1766
Ondansetron hydrochloride	365.860	-7.1733
Racecadotril	385.490	-7.1728
Benzydamine hydrochloride	345.870	-7.1727
Darolutamide	398.850	-7.1679
Fluphenazine hydrochloride	510.440	-7.1614
Zaltoprofen	298.360	-7.1613
Indinavir sulfate	711.870	-7.1579
Indinavir sulfate	711.870	-7.1526
Ondansetron hydrochloride	365.860	-7.1511
Azelastine hydrochloride	418.360	-7.1459
Indinavir sulfate	711.870	-7.1439
Fupentixol Dihydrochloride	507.430	-7.1427
Indinavir sulfate	711.870	-7.1339
Darolutamide	398.850	-7.1323
Indinavir sulfate	711.870	-7.1314
Indinavir sulfate	711.870	-7.1261
Indinavir sulfate	711.870	-7.1123
Tropicamide	284.360	-7.1096
Indinavir sulfate	711.870	-7.1065
Indinavir sulfate	711.870	-7.1021
Indinavir sulfate	711.870	-7.1009
Praziquantel	312.420	-7.1002
Fipronil	437.150	-7.0832
Eletriptan HBr	463.430	-7.0762
Propiverine hydrochloride	403.940	-7.0756
Isoconazole nitrate	479.140	-7.0714
Isavuconazonium sulfate	814.840	-7.0711
Protriptyline hydrochloride	299.840	-7.0695
Lonidamine	321.170	-7.0691
Ataluren	284.240	-7.0688
Indinavir sulfate	711.870	-7.0673
Indinavir sulfate	711.870	-7.0655
Benzydamine hydrochloride	345.870	-7.0642
Indinavir sulfate	711.870	-7.064
Enasidenib	473.380	-7.0532
Indinavir sulfate	711.870	-7.0532
Bitopertin	543.460	-7.0478
Azelastine hydrochloride	418.360	-7.0472
Bifonazole	310.390	-7.041
Trifluoperazine dihydrochloride	480.430	-7.0379
Darolutamide	398.850	-7.0378
Indinavir sulfate	711.870	-7.0369

Glafenine Hydrochloride	409.260	-7.035
Glafenine	372.800	-7.035
Naftifine hydrochloride	323.860	-7.0295
Gestrinone	308.410	-7.0281
Azatadine Maleate	522.550	-7.0274
Chlorprothixene hydrochloride	352.320	-7.0272
Chlorprothixene	315.860	-7.0272
Desloratadine	310.820	-7.026
Indinavir sulfate	711.870	-7.0209
Ticlopidine hydrochloride	300.250	-7.0209
Indinavir sulfate	711.870	-7.0174
Indinavir sulfate	711.870	-7.0107
Amoxapine	313.790	-7.0097
Indinavir sulfate	711.870	-7.0094
Mefloquine hydrochloride	414.770	-7.0093
Dacomitinib	469.940	-7.0031
Naftifine hydrochloride	323.860	-7.0016
Mebhydroline	276.380	-7.001
Bitopertin	543.460	-7.0009
Mebhydrolin napadisylate	420.520	-7.0004
Glafenine Hydrochloride	409.260	-6.9983
Glafenine	372.800	-6.9983
Glafenine Hydrochloride	409.260	-6.9977
Glafenine	372.800	-6.9977
Anthralin	226.230	-6.9951
Clomipramine hydrochloride	351.310	-6.9948
Indinavir sulfate	711.870	-6.993
Propiverine hydrochloride	403.940	-6.9927
Darolutamide	398.850	-6.9869
Bifonazole	310.390	-6.9739
Rupatadine Fumarate	532.030	-6.9714
Glafenine Hydrochloride	409.260	-6.9681
Glafenine	372.800	-6.9681
Indinavir sulfate	711.870	-6.9665
Ticlopidine hydrochloride	300.250	-6.9655
Empagliflozin	450.910	-6.9627
Eletriptan HBr	463.430	-6.9588
Chlorpromazine hydrochloride	355.320	-6.9572
Trimebutine maleate	503.550	-6.9562
Trimebutine	387.470	-6.9562
Indinavir sulfate	711.870	-6.9542
Tetrahydropalmatine hydrochloride	391.890	-6.9473
Bendazac L-lysine	428.480	-6.9463

Sertaconazole nitrate	500.780	-6.9453
Carbamazepine	236.270	-6.9339
Chrysophanic Acid	254.240	-6.9307
Naftifine hydrochloride	323.860	-6.9294
Enasidenib	473.380	-6.9285
Flufenamic acid	281.230	-6.9273
Mebhydrolin napadisylate	420.520	-6.9248
Indinavir sulfate	711.870	-6.9247
Mebhydroline	276.380	-6.9233
Fipronil	437.150	-6.9231
Escitalopram Oxalate	414.430	-6.9171
Citalopram HBr	405.310	-6.9171
Indinavir sulfate	711.870	-6.9149
Ezetimibe	409.430	-6.9069
Nefopam hydrochloride	289.800	-6.9055
Nafronyl oxalate	473.560	-6.9026
Fipronil	437.150	-6.9024
Doxepin hydrochloride	315.840	-6.9013
Lercanidipine	611.730	-6.9012
Lercanidipine hydrochloride	648.190	-6.9009
Roxatidine Acetate hydrochloride	384.900	-6.8991
Indinavir sulfate	711.870	-6.8913
Safinamide mesylate	398.450	-6.891
Anthralin	226.230	-6.8865
Diltiazem hydrochloride	450.980	-6.8856
Indinavir sulfate	711.870	-6.884
10,11-Dihydrocarbamazepine	238.290	-6.8836
Trimipramine maleate	410.510	-6.8797
Chlorprothixene hydrochloride	352.320	-6.8791
Chlorprothixene	315.860	-6.8791
Indinavir sulfate	711.870	-6.8778
Indinavir sulfate	711.870	-6.8755
Indinavir sulfate	711.870	-6.8748
Ramosetron	279.340	-6.8731
Casanthranol	396.580	-6.8709
Choline Fenofibrate	421.910	-6.8703
Trifluoperazine dihydrochloride	480.430	-6.8703
(S)-(+)-Clopidogrel hydrogen sulfate	419.900	-6.8693
Casanthranol	396.580	-6.8682
Nelfinavir Mesylate	663.890	-6.8682
Donepezil	379.500	-6.8648
Donepezil HCl	415.960	-6.8639
Doxepin hydrochloride	315.840	-6.8589

R788 Disodium	624.420	-6.8555
Trelagliptin succinate	475.470	-6.8536
Trelagliptin	357.380	-6.8533
Lonidamine	321.170	-6.8479
Indapamide	365.840	-6.8443
Fostamatinib	580.460	-6.8414
Chlorpheniramine maleate	390.870	-6.8408
Dexchlorpheniramine Maleate	390.860	-6.8372
Naftifine hydrochloride	323.860	-6.8323
Ruxolitinib (INCB-18424) phosphate	404.360	-6.826
Ruxolitinib	306.370	-6.826
Brompheniramine hydrogen maleate	435.310	-6.8221
Atomoxetine hydrochloride	291.820	-6.8123
Indinavir sulfate	711.870	-6.8113
L-Hyoscyamine	289.380	-6.8108
Atropine sulfate	676.820	-6.8072
Atropine sulfate monohydrate	694.830	-6.8072
Atropine	289.370	-6.8072
Nortriptyline hydrochloride	299.840	-6.8055
Rizatriptan benzoate	391.470	-6.8052
Trifluoperazine dihydrochloride	480.430	-6.8004
Fipronil	437.150	-6.7988
Alosetron hydrochloride	330.810	-6.7921
Promazine hydrochloride	320.880	-6.7919
Entrectinib	560.640	-6.7869
Sertaconazole nitrate	500.780	-6.7802
Flunixin meglumin	491.460	-6.7774
Promazine hydrochloride	320.880	-6.7711
Indinavir sulfate	711.870	-6.7703
Crisaborole	251.050	-6.7695
Aloe-emodin	270.240	-6.7658
Leflunomide	270.210	-6.7646
Indinavir sulfate	711.870	-6.7642
Indinavir sulfate	711.870	-6.7593
Entrectinib	560.640	-6.7567
Mefloquine hydrochloride	414.770	-6.7539
Phenprocoumon	280.320	-6.7528
Promethazine	284.420	-6.7508
Dinaciclib	396.490	-6.7484
Flurbiprofen Axetil	330.360	-6.7468
Rotigotine	315.470	-6.7457
Flurbiprofen Axetil	330.360	-6.7412

Indinavir sulfate	711.870	-6.7412
Pioglitazone	356.440	-6.7393
Promazine hydrochloride	320.880	-6.7372
Meptazinol hydrochloride	269.810	-6.7366
Nabumetone	228.290	-6.7352
Seratrodast	354.440	-6.7352
Eperisone hydrochloride	295.850	-6.7332
Meptazinol hydrochloride	269.810	-6.7312
Cyclandelate	276.380	-6.731
Trimeprazine tartrate	746.980	-6.7207
Oxcarbazepine	252.270	-6.7152
Indinavir sulfate	711.870	-6.7139
Terbinafine	291.430	-6.7138
Alosetron hydrochloride	330.810	-6.7046
Zileuton	236.290	-6.7032
Leflunomide	270.210	-6.702
Meptazinol hydrochloride	269.810	-6.6945
Casanthranol	396.580	-6.6939
Sertaconazole nitrate	500.780	-6.6939
Norethindrone	298.420	-6.6926
Chloroquine diphosphate	515.870	-6.692
Atomoxetine hydrochloride	291.820	-6.692
Ataluren	284.240	-6.6786
Miconazole nitrate	479.150	-6.678
Asenapine Maleate	401.840	-6.6762
Eletriptan HBr	463.430	-6.6755
Pizotifen	295.440	-6.6746
Pizotifen Malate	429.530	-6.6746
Antrapurol	240.220	-6.6714
Flufenamic acid	281.230	-6.6698
Carbamazepine	236.270	-6.6667
Ondansetron hydrochloride	365.860	-6.6588
Antrapurol	240.220	-6.6566
Antrapurol	240.220	-6.6557
Benzyl benzoate	212.240	-6.6529
Nortriptyline hydrochloride	299.840	-6.6505
Nabumetone	228.290	-6.6467
Naftifine hydrochloride	323.860	-6.6462
Anthralin	226.230	-6.6451
Ramosetron	279.340	-6.6433
Olprinone	250.260	-6.6418
Ondansetron hydrochloride	365.860	-6.6355
Talniflumate	414.330	-6.6307

Flunixin meglumin	491.460	-6.6291
Meptazinol hydrochloride	269.810	-6.6281
Cyclothiazide	389.900	-6.6205
Brompheniramine hydrogen maleate	435.310	-6.6201
Choline Fenofibrate	421.910	-6.6189
Racecadotril	385.490	-6.6069
Indinavir sulfate	711.870	-6.6062
Alosetron hydrochloride	330.810	-6.6051
Diphenhydramine hydrochloride	291.820	-6.5972
Dimenhydrinate	469.960	-6.5972
Flurbiprofen Axetil	330.360	-6.5946
Leflunomide	270.210	-6.5911
Maprotiline hydrochloride	313.860	-6.5908
Bephenium Hydroxynaphthoate	443.530	-6.5882
Eletriptan HBr	463.430	-6.5854
Indinavir sulfate	711.870	-6.578
Niflumic acid	282.220	-6.576
Terbinafine hydrochloride	327.890	-6.5741
Dapoxetine HCl	341.870	-6.5731
Indinavir sulfate	711.870	-6.5681
Benazepril hydrochloride	460.950	-6.5663
Norethindrone	298.420	-6.5651
Phenprocoumon	280.320	-6.5648
Eletriptan HBr	463.430	-6.561
Bromfenac sodium	356.150	-6.5606
Nepafenac	254.280	-6.5578
Paroxetine hydrochloride	365.830	-6.5562
Eperisone hydrochloride	295.850	-6.5514
voxelotor	337.370	-6.551
Topiroxostat	248.240	-6.5499
Boceprevir	519.680	-6.5346
Zileuton	236.290	-6.5341
Paroxetine hydrochloride	365.830	-6.5339
Mestranol	310.430	-6.5333
Meptazinol hydrochloride	269.810	-6.5317
Bendazol	208.260	-6.5302
Fostamatinib	580.460	-6.5274
Bendazol	208.260	-6.5273
Levomilnacipran	246.340	-6.5261
Olopatadine hydrochloride	373.870	-6.5254
Ketotifen fumarate	425.500	-6.5244
Indinavir sulfate	711.870	-6.5204

Flurbiprofen Axetil	330.360	-6.5183
Indinavir sulfate	711.870	-6.5126
Bendazac L-lysine	428.480	-6.5125
R788 Disodium	624.420	-6.5073
Indinavir sulfate	711.870	-6.5044
Benzthiazide	431.940	-6.4953
Flurbiprofen Axetil	330.360	-6.4919
Fipronil	437.150	-6.4914
Ondansetron hydrochloride	365.860	-6.4813
Phenprocoumon	280.320	-6.4792
Cyclothiazide	389.900	-6.4716
Alosetron hydrochloride	330.810	-6.4701
Indinavir sulfate	711.870	-6.4594
8-Methoxypsoralen	216.190	-6.4593
Catharanthine	336.420	-6.4571
Boceprevir	519.680	-6.4495
Eburnalritardo	294.390	-6.4446
Gestrinone	308.410	-6.4434
Losartan	422.910	-6.4412
Losartan potassium	461.010	-6.4405
Eburnalritardo	294.390	-6.4376
Indinavir sulfate	711.870	-6.4349
Praziquantel	312.420	-6.4221
Indinavir sulfate	711.870	-6.4196
Praziquantel	312.420	-6.4094
Tropisetron hydrochloride	320.820	-6.4071
Phenprocoumon	280.320	-6.3981
Diflunisal	250.200	-6.3912
Loxoprofen	246.300	-6.3632
Gestrinone	308.410	-6.3598
Indinavir sulfate	711.870	-6.3505
Liranaftate	328.430	-6.3501
Zileuton	236.290	-6.3405
Praziquantel	312.420	-6.3297
Amlexanox	298.290	-6.3273
Valbenazine	418.570	-6.3238
Levomilnacipran	246.340	-6.3186
Phenylindione	222.250	-6.3137
Diflunisal	250.200	-6.3134
Indinavir sulfate	711.870	-6.2959
Cefaclor monohydrate	385.820	-6.2932
Olprinone	250.260	-6.29
Valbenazine	418.570	-6.2875

Desloratadine	310.820	-6.278
Diflunisal	250.200	-6.2769
Psoralen	186.170	-6.2611
Pitavastatin calcium	880.980	-6.2453
Phenprocoumon	280.320	-6.2284
Milnacipran hydrochloride	282.810	-6.1958
Bendamustine hydrochloride	394.720	-6.1932
Levomilnacipran	246.340	-6.1901
Cephalexin	347.390	-6.189
Citalopram HBr	405.310	-6.1881
Oxcarbazepine	252.270	-6.1818
Zileuton	236.290	-6.1734
Norgestimate	369.500	-6.1714
Scopolamine butylbromide	440.370	-6.1547
Levonorgestrel	312.450	-6.1498
Norgestimate	369.500	-6.1479
Valbenazine	418.570	-6.143
Warfarin sodium	330.310	-6.1247
Pipemidic acid	303.320	-6.1186
Saxagliptin hydrate	333.430	-6.1048
Bosentan	551.610	-6.0993
Cephalexin	347.390	-6.0912
Levomilnacipran	246.340	-6.0852
Valbenazine	418.570	-6.0807
Levomilnacipran	246.340	-6.07
Ifenprodil Tartrate	400.490	-6.0644
Ifenprodil Tartrate	400.490	-6.0544
Levonorgestrel	312.450	-6.0281
Valbenazine	418.570	-5.8956
Enalaprilat Dihydrate	348.400	-5.8478
Mitiglinide calcium	704.910	-5.8044
Exemestane	296.400	-5.7214
Ipratropium Bromide	412.370	-5.5638
Ipratropium bromide monohydrate	430.380	-5.5638
Fosinopril sodium	586.650	-5.5111
Ipratropium Bromide	412.370	-5.4669
Ipratropium bromide monohydrate	430.380	-5.4669
Fosinopril sodium	586.650	-5.3064
Hydrocortisone butyrate	432.550	-5.1468

Table S31. VS2 for LH1 FDA Approved drugs targeting PARP1 100% cutoff

CAS	Drug Name	In silico Energy
4852-22-6	Proanthocyanidins	4852-22-6
4852-22-6	Proanthocyanidins	4852-22-6
157810-81-6	Indinavir sulfate	157810-81-7
4852-22-6	Proanthocyanidins	4852-22-6
4852-22-6	Proanthocyanidins	4852-22-6
157810-81-6	Indinavir sulfate	157810-81-6
157810-81-6	Indinavir sulfate	157810-81-6
4852-22-6	Proanthocyanidins	4852-22-6
4852-22-6	Proanthocyanidins	4852-22-6
16679-58-6	Desmopressin	16679-58-7
8024-48-4	Casanthranol	8024-48-5
4852-22-6	Proanthocyanidins	4852-22-6
4852-22-6	Proanthocyanidins	4852-22-6
4852-22-6	Proanthocyanidins	4852-22-6
4852-22-6	Proanthocyanidins	4852-22-6
4852-22-6	Proanthocyanidins	4852-22-6
4852-22-6	Proanthocyanidins	4852-22-6
4852-22-6	Proanthocyanidins	4852-22-6
8024-48-4	Casanthranol	8024-48-5
115939-25-8	Salvianolic acid B	115939-25-9
153-18-4	Rutin	153-18-5
8024-48-4	Casanthranol	8024-48-5
4852-22-6	Proanthocyanidins	4852-22-6
4852-22-6	Proanthocyanidins	4852-22-6
4852-22-6	Proanthocyanidins	4852-22-6
8024-48-4	Casanthranol	8024-48-5
146-56-5	Fluphenazine hydrochloride	146-56-6
8024-48-4	Casanthranol	8024-48-5
4852-22-6	Proanthocyanidins	4852-22-7
8024-48-4	Casanthranol	8024-48-5
4852-22-6	Proanthocyanidins	4852-22-6
4852-22-6	Proanthocyanidins	4852-22-6
115939-25-8	Salvianolic acid B	115939-25-9
4852-22-6	Proanthocyanidins	4852-22-6
4852-22-6	Proanthocyanidins	4852-22-6
8024-48-4	Casanthranol	8024-48-5
1985606-14-1	Baloxavir marboxil	1985606-14-2
157810-81-6	Indinavir sulfate	157810-81-7
123524-52-7	Azelnidipine	123524-52-8
8024-48-4	Casanthranol	8024-48-5

1985606-14-1	Baloxavir marboxil	1985606-14-2
8024-48-4	Casanthranol	8024-48-5
1985606-14-1	Baloxavir marboxil	1985606-14-2
4852-22-6	Proanthocyanidins	4852-22-6
4852-22-6	Proanthocyanidins	4852-22-6
4852-22-6	Proanthocyanidins	4852-22-6
4852-22-6	Proanthocyanidins	4852-22-6
157810-81-6	Indinavir sulfate	157810-81-7
8024-48-4	Casanthranol	8024-48-5
25332-39-2	Trazodone hydrochloride	25332-39-3
155206-00-1	Bimatoprost	155206-00-2
115939-25-8	Salvianolic acid B	115939-25-9
146-56-5	Fluphenazine hydrochloride	146-56-6
149022-22-0	Amikacin Sulfate Salt	149022-22-1
65271-80-9	Mitoxantrone	65271-80-10
70476-82-3	Mitoxantrone hydrochloride	70476-82-4
4852-22-6	Proanthocyanidins	4852-22-7
65271-80-9	Mitoxantrone	65271-80-10
70476-82-3	Mitoxantrone hydrochloride	70476-82-4
4852-22-6	Proanthocyanidins	4852-22-7
157810-81-6	Indinavir sulfate	157810-81-7
130656-51-8	Nifekalant hydrochloride	130656-51-9
157810-81-6	Indinavir sulfate	157810-81-7
8024-48-4	Casanthranol	8024-48-5
157283-68-6	Travoprost	157283-68-7
4852-22-6	Proanthocyanidins	4852-22-7
72956-09-3	Carvedilol	72956-09-4
610309-89-2	Carvedilol phosphate	610309-89-3
155206-00-1	Bimatoprost	155206-00-2
157283-68-6	Travoprost	157283-68-7
2030-63-9	Clofazimine	2030-63-10
72956-09-3	Carvedilol	72956-09-4
610309-89-2	Carvedilol phosphate	610309-89-3
4852-22-6	Proanthocyanidins	4852-22-7
157283-68-6	Travoprost	157283-68-7
8024-48-4	Casanthranol	8024-48-5
4852-22-6	Proanthocyanidins	4852-22-7
153-18-4	Rutin	153-18-5
8024-48-4	Casanthranol	8024-48-4
8024-48-4	Casanthranol	8024-48-4
157283-68-6	Travoprost	157283-68-7

37517-28-5	Amikacin	37517-28-6
157283-68-6	Travoprost	157283-68-7
157810-81-6	Indinavir sulfate	157810-81-7
4852-22-6	Proanthocyanidins	4852-22-7
8024-48-4	Casanthranol	8024-48-5
4852-22-6	Proanthocyanidins	4852-22-7
82640-04-8	Raloxifene hydrochloride	82640-04-9
4852-22-6	Proanthocyanidins	4852-22-7
8024-48-4	Casanthranol	8024-48-4
8024-48-4	Casanthranol	8024-48-4
65271-80-9	Mitoxantrone	65271-80-10
4852-22-6	Proanthocyanidins	4852-22-7
70476-82-3	Mitoxantrone hydrochloride	70476-82-4
182431-12-5	Lomitapide	182431-12-6
8024-48-4	Casanthranol	8024-48-5
25332-39-2	Trazodone hydrochloride	25332-39-3
4852-22-6	Proanthocyanidins	4852-22-7
8024-48-4	Casanthranol	8024-48-5
4852-22-6	Proanthocyanidins	4852-22-7
8024-48-4	Casanthranol	8024-48-5
1297538-32-9	Darolutamide	1297538-32-10
157283-68-6	Travoprost	157283-68-7
146-56-5	Fluphenazine hydrochloride	146-56-6
4852-22-6	Proanthocyanidins	4852-22-7
72956-09-3	Carvedilol	72956-09-4
610309-89-2	Carvedilol phosphate	610309-89-3
8024-48-4	Casanthranol	8024-48-4
8024-48-4	Casanthranol	8024-48-4
157283-68-6	Travoprost	157283-68-7
157810-81-6	Indinavir sulfate	157810-81-7
155206-00-1	Bimatoprost	155206-00-2
29908-03-0	Ademetionine	29908-03-1
155213-67-5	Ritonavir	155213-67-6
8024-48-4	Casanthranol	8024-48-4
8024-48-4	Casanthranol	8024-48-4
157810-81-6	Indinavir sulfate	157810-81-7
4852-22-6	Proanthocyanidins	4852-22-7
157810-81-6	Indinavir sulfate	157810-81-7
4852-22-6	Proanthocyanidins	4852-22-7
157810-81-6	Indinavir sulfate	157810-81-7
64872-77-1	Butoconazole nitrate	64872-77-2

1297538-32-9	Darolutamide	1297538-32-10
4852-22-6	Proanthocyanidins	4852-22-6
4852-22-6	Proanthocyanidins	4852-22-6
159989-65-8	Nelfinavir Mesylate	159989-65-9
8024-48-4	Casanthranol	8024-48-5
4852-22-6	Proanthocyanidins	4852-22-6
4852-22-6	Proanthocyanidins	4852-22-6
4852-22-6	Proanthocyanidins	4852-22-6
193275-84-2	Lonafarnib	193275-84-3
8024-48-4	Casanthranol	8024-48-5
157283-68-6	Travoprost	157283-68-7
29908-03-0	Ademetionine	29908-03-1
157283-68-6	Travoprost	157283-68-7
8024-48-4	Casanthranol	8024-48-5
157810-81-6	Indinavir sulfate	157810-81-7
8024-48-4	Casanthranol	8024-48-5
29908-03-0	Ademetionine	29908-03-1
157283-68-6	Travoprost	157283-68-7
193275-84-2	Lonafarnib	193275-84-3
4852-22-6	Proanthocyanidins	4852-22-7
157283-68-6	Travoprost	157283-68-7
155213-67-5	Ritonavir	155213-67-6
779353-01-4	Dinaciclib	779353-01-5
8024-48-4	Casanthranol	8024-48-5
4852-22-6	Proanthocyanidins	4852-22-7
157283-68-6	Travoprost	157283-68-7
157810-81-6	Indinavir sulfate	157810-81-7
29908-03-0	Ademetionine	29908-03-1
157283-68-6	Travoprost	157283-68-7
29908-03-0	Ademetionine	29908-03-1
157810-81-6	Indinavir sulfate	157810-81-7
29908-03-0	Ademetionine	29908-03-1
157810-81-6	Indinavir sulfate	157810-81-7
4852-22-6	Proanthocyanidins	4852-22-7
157810-81-6	Indinavir sulfate	157810-81-7
8024-48-4	Casanthranol	8024-48-4
8024-48-4	Casanthranol	8024-48-4
65666-07-1	Silymarin	65666-07-2
182431-12-5	Lomitapide	182431-12-6
8024-48-4	Casanthranol	8024-48-5
157810-81-6	Indinavir sulfate	157810-81-7
8024-48-4	Casanthranol	8024-48-5

1415-73-2	Aloin	1415-73-3
481-72-1	Aloe-emodin	481-72-2
8024-48-4	Casanthranol	8024-48-5
157810-81-6	Indinavir sulfate	157810-81-6
157810-81-6	Indinavir sulfate	157810-81-6
159989-65-8	Nelfinavir Mesylate	159989-65-9
157810-81-6	Indinavir sulfate	157810-81-7
8024-48-4	Casanthranol	8024-48-5
100427-26-7	Lercanidipine	100427-26-8
99755-59-6	Rotigotine	99755-59-7
157283-68-6	Travoprost	157283-68-7
132866-11-6	Lercanidipine hydrochloride	132866-11-7
4852-22-6	Proanthocyanidins	4852-22-7
8024-48-4	Casanthranol	8024-48-5
29908-03-0	Ademetionine	29908-03-1
8024-48-4	Casanthranol	8024-48-5
72956-09-3	Carvedilol	72956-09-4
1029044-16-3	Pexidartinib	1029044-16-4
157810-81-6	Indinavir sulfate	157810-81-7
72956-09-3	Carvedilol	72956-09-4
1190307-88-0	Sofosbuvir	1190307-88-1
610309-89-2	Carvedilol phosphate	610309-89-3
286930-03-8	Fesoterodine fumarate	286930-03-9
157810-81-6	Indinavir sulfate	157810-81-6
157810-81-6	Indinavir sulfate	157810-81-6
4852-22-6	Proanthocyanidins	4852-22-7
610309-89-2	Carvedilol phosphate	610309-89-3
8024-48-4	Casanthranol	8024-48-4
8024-48-4	Casanthranol	8024-48-4
4852-22-6	Proanthocyanidins	4852-22-7
159989-65-8	Nelfinavir Mesylate	159989-65-9
29908-03-0	Ademetionine	29908-03-1
1297538-32-9	Darolutamide	1297538-32-10
157810-81-6	Indinavir sulfate	157810-81-7
157283-68-6	Travoprost	157283-68-7
157810-81-6	Indinavir sulfate	157810-81-7
842133-18-0	Canagliflozin	842133-18-1
8024-48-4	Casanthranol	8024-48-5
157810-81-6	Indinavir sulfate	157810-81-7
4852-22-6	Proanthocyanidins	4852-22-7

60925-61-3	Ceforanide	60925-61-4
161973-10-0	Esomeprazole Magnesium	161973-10-1
155213-67-5	Ritonavir	155213-67-6
29908-03-0	Ademetionine	29908-03-1
163222-33-1	Ezetimibe	163222-33-2
157283-68-6	Travoprost	157283-68-7
8024-48-4	Casanthranol	8024-48-4
8024-48-4	Casanthranol	8024-48-4
157810-81-6	Indinavir sulfate	157810-81-7
8024-48-4	Casanthranol	8024-48-5
1985606-14-1	Baloxavir marboxil	1985606-14-2
520-26-3	Hesperidin	520-26-4
157283-68-6	Travoprost	157283-68-7
157810-81-6	Indinavir sulfate	157810-81-6
157810-81-6	Indinavir sulfate	157810-81-6
8024-48-4	Casanthranol	8024-48-5
159989-65-8	Nelfinavir Mesylate	159989-65-9
26864-56-2	Penfluridol	26864-56-3
1985606-14-1	Baloxavir marboxil	1985606-14-2
4852-22-6	Proanthocyanidins	4852-22-7
157283-68-6	Travoprost	157283-68-7
123318-82-1	Clofarabine	123318-82-2
4852-22-6	Proanthocyanidins	4852-22-7
142340-99-6	Adefovir dipivoxil	142340-99-7
8024-48-4	Casanthranol	8024-48-5
4852-22-6	Proanthocyanidins	4852-22-7
132866-11-6	Lercanidipine hydrochloride	132866-11-7
8024-48-4	Casanthranol	8024-48-5
100427-26-7	Lercanidipine	100427-26-8
157810-81-6	Indinavir sulfate	157810-81-7
81110-73-8	Racecadotril	81110-73-9
2030-63-9	Clofazimine	2030-63-10
4852-22-6	Proanthocyanidins	4852-22-7
157283-68-6	Travoprost	157283-68-7
159989-65-8	Nelfinavir Mesylate	159989-65-9
157283-68-6	Travoprost	157283-68-6
157283-68-6	Travoprost	157283-68-6
4852-22-6	Proanthocyanidins	4852-22-7
157810-81-6	Indinavir sulfate	157810-81-7
842133-18-0	Canagliflozin	842133-18-1
138-52-3	Salicoside	138-52-4

157810-81-6	Indinavir sulfate	157810-81-7
10236-47-2	Naringin	10236-47-3
1098-60-8	Triflupromazine hydrochloride	1098-60-9
8024-48-4	Casanthranol	8024-48-5
50-27-1	Estriol	50-27-2
4852-22-6	Proanthocyanidins	4852-22-7
65666-07-1	Silymarin	65666-07-2
157283-68-6	Travoprost	157283-68-7
25316-40-9	Doxorubicin hydrochloride	25316-40-10
157283-68-6	Travoprost	157283-68-7
150915-41-6	Perospirone	150915-41-7
161973-10-0	Esomeprazole Magnesium	161973-10-1
157283-68-6	Travoprost	157283-68-7
130656-51-8	Nifekalant hydrochloride	130656-51-9
157283-68-6	Travoprost	157283-68-7
157810-81-6	Indinavir sulfate	157810-81-7
157283-68-6	Travoprost	157283-68-6
157283-68-6	Travoprost	157283-68-6
65666-07-1	Silymarin	65666-07-2
3200-06-4	Nafronyl oxalate	3200-06-5
138-52-3	Salicoside	138-52-4
50-27-1	Estriol	50-27-2
1415-73-2	Aloin	1415-73-3
99592-39-9	Sertaconazole nitrate	99592-39-10
150915-41-6	Perospirone	150915-41-7
8024-48-4	Casanthranol	8024-48-5
2413-38-9	Fupentixol Dihydrochloride	2413-38-10
157283-68-6	Travoprost	157283-68-7
8024-48-4	Casanthranol	8024-48-4
8024-48-4	Casanthranol	8024-48-4
157810-81-6	Indinavir sulfate	157810-81-7
404950-80-7	Panobinostat	404950-80-8
4852-22-6	Proanthocyanidins	4852-22-7
157810-81-6	Indinavir sulfate	157810-81-7
8024-48-4	Casanthranol	8024-48-5
15176-29-1	Edoxudine	15176-29-2
157810-81-6	Indinavir sulfate	157810-81-7
29908-03-0	Ademetionine	29908-03-1
39562-70-4	Nitrendipine	39562-70-5
146-56-5	Fluphenazine hydrochloride	146-56-6
157283-68-6	Travoprost	157283-68-7
317-34-0	Aminophylline	317-34-1
138-52-3	Salicoside	138-52-4

157810-81-6	Indinavir sulfate	157810-81-7
157283-68-6	Travoprost	157283-68-7
8024-48-4	Casanthranol	8024-48-5
157283-68-6	Travoprost	157283-68-7
130656-51-8	Nifekalant hydrochloride	130656-51-9
8024-48-4	Casanthranol	8024-48-4
8024-48-4	Casanthranol	8024-48-4
157810-81-6	Indinavir sulfate	157810-81-7
53910-25-1	Pentostatin	53910-25-2
404950-80-7	Panobinostat	404950-80-8
157810-81-6	Indinavir sulfate	157810-81-7
8024-48-4	Casanthranol	8024-48-5
130656-51-8	Nifekalant hydrochloride	130656-51-9
53910-25-1	Pentostatin	53910-25-2
1297538-32-9	Darolutamide	1297538-32-10
65666-07-1	Silymarin	65666-07-2
8024-48-4	Casanthranol	8024-48-5
1190307-88-0	Sofosbuvir	1190307-88-1
625115-55-1	Riociguat	625115-55-2
65666-07-1	Silymarin	65666-07-2
4852-22-6	Proanthocyanidins	4852-22-7
157283-68-6	Travoprost	157283-68-7
157810-81-6	Indinavir sulfate	157810-81-7
157283-68-6	Travoprost	157283-68-7
70-00-8	Trifluridine	70-00-9
157283-68-6	Travoprost	157283-68-7
8024-48-4	Casanthranol	8024-48-5
65666-07-1	Silymarin	65666-07-2
4852-22-6	Proanthocyanidins	4852-22-7
73151-29-8	Fenticonazole Nitrate	73151-29-9
379270-37-8	Tenofovir alafenamide	379270-37-9
842133-18-0	Canagliflozin	842133-18-1
4852-22-6	Proanthocyanidins	4852-22-7
1446502-11-9	Enasidenib	1446502-11-10
481-72-1	Aloe-emodin	481-72-2
161973-10-0	Esomeprazole Magnesium	161973-10-1
142340-99-6	Adefovir dipivoxil	142340-99-7
25316-40-9	Doxorubicin hydrochloride	25316-40-10
73151-29-8	Fenticonazole Nitrate	73151-29-9
50-27-1	Estriol	50-27-2

1038915-73-9	Niraparib tosylate	1038915-73-10
29216-28-2	Mequitazine	29216-28-3
1038915-60-4	Niraparib	1038915-60-5
8024-48-4	Casanthranol	8024-48-4
8024-48-4	Casanthranol	8024-48-4
5490-27-7	Dihydrostreptomycin sulfate	5490-27-8
864070-44-0	Empagliflozin	864070-44-1
100427-26-7	Lercanidipine	100427-26-8
157283-68-6	Travoprost	157283-68-7
29908-03-0	Ademetionine	29908-03-1
132866-11-6	Lercanidipine hydrochloride	132866-11-7
4852-22-6	Proanthocyanidins	4852-22-7
5490-27-7	Dihydrostreptomycin sulfate	5490-27-8
157810-81-6	Indinavir sulfate	157810-81-7
157283-68-6	Travoprost	157283-68-7
157810-81-6	Indinavir sulfate	157810-81-7
379270-37-8	Tenofovir alafenamide	379270-37-9
132866-11-6	Lercanidipine hydrochloride	132866-11-7
100427-26-7	Lercanidipine	100427-26-8
404950-80-7	Panobinostat	404950-80-8
193275-84-2	Lonafarnib	193275-84-3
157283-68-6	Travoprost	157283-68-7
864070-44-0	Empagliflozin	864070-44-1
481-72-1	Aloe-emodin	481-72-2
157283-68-6	Travoprost	157283-68-7
157810-81-6	Indinavir sulfate	157810-81-7
64872-77-1	Butoconazole nitrate	64872-77-2
161973-10-0	Esomeprazole Magnesium	161973-10-1
73151-29-8	Fenticonazole Nitrate	73151-29-9
8024-48-4	Casanthranol	8024-48-5
130656-51-8	Nifekalant hydrochloride	130656-51-9
157810-81-6	Indinavir sulfate	157810-81-7
82640-04-8	Raloxifene hydrochloride	82640-04-9
157810-81-6	Indinavir sulfate	157810-81-7
8024-48-4	Casanthranol	8024-48-4
8024-48-4	Casanthranol	8024-48-4
8024-48-4	Casanthranol	8024-48-4
99755-59-6	Rotigotine	99755-59-7
1098-60-8	Triflupromazine hydrochloride	1098-60-9
163222-33-1	Ezetimibe	163222-33-2
65666-07-1	Silymarin	65666-07-1

65666-07-1	Silymarin	65666-07-1
19237-84-4	Prazosin hydrochloride	19237-84-5
117-10-2	Antrapurol	117-10-3
3200-06-4	Nafronyl oxalate	3200-06-5
26864-56-2	Penfluridol	26864-56-3
157810-81-6	Indinavir sulfate	157810-81-6
157810-81-6	Indinavir sulfate	157810-81-6
8024-48-4	Casanthranol	8024-48-5
82640-04-8	Raloxifene hydrochloride	82640-04-9
8024-48-4	Casanthranol	8024-48-5
65666-07-1	Silymarin	65666-07-2
140462-76-6	Olopatadine hydrochloride	140462-76-7
130656-51-8	Nifekalant hydrochloride	130656-51-9
864070-44-0	Empagliflozin	-7.0788
157283-68-6	Travoprost	-7.0779
128794-94-5	Mycophenolate Mofetil	-7.0765
8024-48-4	Casanthranol	-7.0754
73151-29-8	Fenticonazole Nitrate	-7.0753
157283-68-6	Travoprost	-7.0729
53910-25-1	Pentostatin	-7.0708
8024-48-4	Casanthranol	-7.0674
7083-71-8	(+)-Emetine dihydrochloride hydrate	-7.0633
157810-81-6	Indinavir sulfate	-7.0623
157283-68-6	Travoprost	-7.0613
26864-56-2	Penfluridol	-7.0596
161796-78-7	Esomeprazole Sodium	-7.0589
16679-58-6	Desmopressin	-7.0549
90357-06-5	Bicalutamide	-7.0549
167465-36-3	Zosuquidar 3HCl	-7.0523
65666-07-1	Silymarin	-7.0521
1029044-16-3	Pexidartinib	-7.0509
761423-87-4	Ipragliflozin	-7.0482
26864-56-2	Penfluridol	-7.0482
8024-48-4	Casanthranol	-7.0442
8024-48-4	Casanthranol	-7.044
115956-12-2	Dolasetron	-7.0418
157810-81-6	Indinavir sulfate	-7.0415
157283-68-6	Travoprost	-7.0413
4852-22-6	Proanthocyanidins	-7.0409
3681-99-0	Puerarin	-7.0397
157283-68-6	Travoprost	-7.0394
99755-59-6	Rotigotine	-7.0371

1392275-56-7	Tenofovir alafenamide fumarate	-7.0353
157810-81-6	Indinavir sulfate	-7.0349
157810-81-6	Indinavir sulfate	-7.0341
8024-48-4	Casanthranol	-7.0337
72956-09-3	Carvedilol	-7.0297
65666-07-1	Silymarin	-7.0287
72956-09-3	Carvedilol	-7.0287
100643-71-8	Desloratadine	-7.0283
54063-53-5	Propafenone	-7.0278
157283-68-6	Travoprost	-7.0271
157810-81-6	Indinavir sulfate	-7.027
157283-68-6	Travoprost	-7.0269
610309-89-2	Carvedilol phosphate	-7.0263
610309-89-2	Carvedilol phosphate	-7.0258
496775-62-3	Eltrombopag Olamine	-7.0246
630420-16-5	Asunaprevir	-7.0241
75507-68-5	Flupirtine maleate	-7.0228
761423-87-4	Ipragliflozin	-7.0184
39562-70-4	Nitrendipine	-7.0184
100643-71-8	Desloratadine	-7.0164
99755-59-6	Rotigotine	-7.0156
74050-98-9	Ketanserin	-7.0155
157810-81-6	Indinavir sulfate	-7.0142
1985606-14-1	Baloxavir marboxil	-7.0137
157810-81-6	Indinavir sulfate	-7.0107
16816-67-4	D-Pantethine	-7.0105
72956-09-3	Carvedilol	-7.0102
4852-22-6	Proanthocyanidins	-7.0096
610309-89-2	Carvedilol phosphate	-7.009
8024-48-4	Casanthranol	-7.0077
164579-32-2	Pantoprazole Sodium Hydrate	-7.0071
8024-48-4	Casanthranol	-7.007
159989-65-8	Nelfinavir Mesylate	-7.0058
157810-81-6	Indinavir sulfate	-7.0052
630420-16-5	Asunaprevir	-7.004
8024-48-4	Casanthranol	-7.0022
1392275-56-7	Tenofovir alafenamide fumarate	-7.0013
4146-30-9	Neomycin Sulphate B	-7.001
522-51-0	Dequalinium chloride	-7.0002
103639-04-9	Ondansetron hydrochloride	-6.9997

157810-81-6	Indinavir sulfate	-6.999
157810-81-6	Indinavir sulfate	-6.9979
1415-73-2	Aloin	-6.9966
192725-17-0	Lopinavir	-6.9953
157283-68-6	Travoprost	-6.9948
1405-10-3	Neomycin sulfate	-6.9943
24168-96-5	Isoconazole nitrate	-6.9934
157810-81-6	Indinavir sulfate	-6.9932
157283-68-6	Travoprost	-6.9909
81110-73-8	Racecadotril	-6.9891
481-74-3	Chrysophanic Acid	-6.9887
4146-30-9	Neomycin Sulphate B	-6.9873
23210-58-4	Ifenprodil Tartrate	-6.9871
157283-68-6	Travoprost	-6.987
1405-10-3	Neomycin sulfate	-6.9866
1415-73-2	Aloin	-6.9863
4852-22-6	Proanthocyanidins	-6.9818
161796-78-7	Esomeprazole Sodium	-6.9803
157810-81-6	Indinavir sulfate	-6.9801
157810-81-6	Indinavir sulfate	-6.9801
23210-58-4	Ifenprodil Tartrate	-6.9797
157283-68-6	Travoprost	-6.9791
269055-15-4	Etravirine	-6.9789
864750-70-9	Revefenacin	-6.9774
157810-81-6	Indinavir sulfate	-6.972
56390-09-1	Epirubicin hydrochloride	-6.972
53-60-1	Promazine hydrochloride	-6.9709
1029044-16-3	Pexidartinib	-6.9704
8024-48-4	Casanthranol	-6.9698
486460-32-6	Sitagliptin	-6.9697
4880-88-0	Eburnalritardo	-6.9686
52-86-8	Haloperidol	-6.9684
99755-59-6	Rotigotine	-6.9683
157283-68-6	Travoprost	-6.9673
8024-48-4	Casanthranol	-6.967
520-27-4	Diosmin	-6.9659
157810-81-6	Indinavir sulfate	-6.9649
111902-57-9	Temocapil	-6.9647
761423-87-4	Ipragliflozin	-6.9645
99755-59-6	Rotigotine	-6.9637
65666-07-1	Silymarin	-6.9633
117-10-2	Antrapurol	-6.9622

157810-81-6	Indinavir sulfate	-6.9611
167465-36-3	Zosuquidar 3HCl	-6.9609
117-10-2	Antrapurol	-6.9608
157283-68-6	Travoprost	-6.9589
29908-03-0	Ademetionine	-6.9585
157283-68-6	Travoprost	-6.9579
16679-58-6	Desmopressin	-6.9578
779353-01-4	Dinaciclib	-6.956
65666-07-1	Silymarin	-6.9556
58579-51-4	Anagrelide HCl	-6.9542
68475-42-3	Anagrelide	-6.9539
157283-68-6	Travoprost	-6.9522
8024-48-4	Casanthranol	-6.9519
157810-81-6	Indinavir sulfate	-6.9515
101827-46-7	Butenafine hydrochloride	-6.9494
81110-73-8	Racecadotril	-6.9484
7083-71-8	(+)-Emetine dihydrochloride hydrate	-6.9455
29908-03-0	Ademetionine	-6.9453
157810-81-6	Indinavir sulfate	-6.9452
157810-81-6	Indinavir sulfate	-6.9448
157810-81-6	Indinavir sulfate	-6.9446
157283-68-6	Travoprost	-6.9411
26864-56-2	Penfluridol	-6.9402
29216-28-2	Mequitazine	-6.9385
73151-29-8	Fenticonazole Nitrate	-6.9382
2753-45-9	Mebeverine hydrochloride	-6.9351
157810-81-6	Indinavir sulfate	-6.9349
111902-57-9	Temocapil	-6.9341
2753-45-9	Mebeverine hydrochloride	-6.9322
58-61-7	Adenosine	-6.932
832720-36-2	Elagolix sodium	-6.932
4852-22-6	Proanthocyanidins	-6.9316
364-98-7	Diazoxide	-6.931
157810-81-6	Indinavir sulfate	-6.9308
865759-25-7	Trelagliptin	-6.9306
23210-58-4	Ifenprodil Tartrate	-6.9298
1029877-94-8	Trelagliptin succinate	-6.9297
6493-05-6	Pentoxifylline	-6.9295
157810-81-6	Indinavir sulfate	-6.9274
1985606-14-1	Baloxavir marboxil	-6.9274
3681-99-0	Puerarin	-6.9268

29908-03-0	Ademetionine	-6.9264
142340-99-6	Adefovir dipivoxil	-6.925
1985606-14-1	Baloxavir marboxil	-6.9234
8024-48-4	Casanthranol	-6.9223
103639-04-9	Ondansetron hydrochloride	-6.9217
23210-58-4	Ifenprodil Tartrate	-6.9207
625115-55-1	Riociguat	-6.9201
128794-94-5	Mycophenolate Mofetil	-6.9198
19356-17-3	Calcifediol	-6.9197
157810-81-6	Indinavir sulfate	-6.9189
23210-58-4	Ifenprodil Tartrate	-6.9188
81110-73-8	Racecadotril	-6.9187
4146-30-9	Neomycin Sulphate B	-6.9168
8024-48-4	Casanthranol	-6.9168
56390-09-1	Epirubicin hydrochloride	-6.9163
79307-93-0	Azelastine hydrochloride	-6.915
10236-47-2	Naringin	-6.9145
65666-07-1	Silymarin	-6.9145
520-27-4	Diosmin	-6.9139
1405-10-3	Neomycin sulfate	-6.9136
135729-62-3	Palonosetron hydrochloride	-6.9135
8024-48-4	Casanthranol	-6.9135
146-56-5	Fluphenazine hydrochloride	-6.9134
7083-71-8	(+)-Emetine dihydrochloride hydrate	-6.9129
157283-68-6	Travoprost	-6.9125
53-16-7	Aquacrine	-6.9123
29908-03-0	Ademetionine	-6.9105
1025504-45-3	Valbenazine	-6.91
157283-68-6	Travoprost	-6.9097
461432-26-8	Dapagliflozin	-6.9095
960404-48-2	Dapagliflozin ((2S)-1,2-propanediol, hydrate)	-6.9077
1263-89-4	Paromomycin Sulfate	-6.9071
99592-39-9	Sertaconazole nitrate	-6.9059
8024-48-4	Casanthranol	-6.9056
482-36-0	Hyperoside	-6.9043
90357-06-5	Bicalutamide	-6.9042
60-87-7	Promethazine	-6.9037
157283-68-6	Travoprost	-6.9032
52-86-8	Haloperidol	-6.902
65666-07-1	Silymarin	-6.9003
8024-48-4	Casanthranol	-6.8996

157283-68-6	Travoprost	-6.8984
151533-22-1	Levomefolate calcium	-6.8975
1297538-32-9	Darolutamide	-6.897
65666-07-1	Silymarin	-6.8938
103577-45-3	Lansoprazole	-6.892
157283-68-6	Travoprost	-6.8915
1029044-16-3	Pexidartinib	-6.8901
146-56-5	Fluphenazine hydrochloride	-6.8896
1098-60-8	Triflupromazine hydrochloride	-6.8893
157283-68-6	Travoprost	-6.8893
4852-22-6	Proanthocyanidins	-6.8891
157810-81-6	Indinavir sulfate	-6.8884
157283-68-6	Travoprost	-6.888
150915-41-6	Perospirone	-6.8868
388082-77-7	Lapatinib Ditosylate	-6.8854
231277-92-2	Lapatinib	-6.8854
379270-37-8	Tenofovir alafenamide	-6.885
388082-78-8	Lapatinib ditosylate monohydrate	-6.8819
157810-81-6	Indinavir sulfate	-6.8809
62499-27-8	Gastrodin	-6.8806
132203-70-4	Cilnidipine	-6.8794
5490-27-7	Dihydrostreptomycin sulfate	-6.8778
157283-68-6	Travoprost	-6.8759
274693-27-5	Ticagrelor	-6.8755
23210-58-4	Ifenprodil Tartrate	-6.8754
24168-96-5	Isoconazole nitrate	-6.8747
157810-81-6	Indinavir sulfate	-6.8742
157283-68-6	Travoprost	-6.874
72956-09-3	Carvedilol	-6.8735
610309-89-2	Carvedilol phosphate	-6.8735
23210-58-4	Ifenprodil Tartrate	-6.8722
3810-74-0	Streptomycin sulfate	-6.8722
151533-22-1	Levomefolate calcium	-6.8721
28721-07-5	Oxcarbazepine	-6.8709
1811-28-5	Proflavine Hemisulfate	-6.8707
60-87-7	Promethazine	-6.8702
60-87-7	Promethazine	-6.8692
29216-28-2	Mequitazine	-6.8644
2413-38-9	Fupentixol Dihydrochloride	-6.864
8024-48-4	Casanthranol	-6.8636
111902-57-9	Temocapil	-6.8632

960404-48-2	Dapagliflozin ((2S)-1,2-propanediol, hydrate)	-6.8627
461432-26-8	Dapagliflozin	-6.8626
157283-68-6	Travoprost	-6.8611
22832-87-7	Miconazole nitrate	-6.8589
79307-93-0	Azelastine hydrochloride	-6.8574
202138-50-9	Tenofovir Disoproxil Fumarate	-6.8573
157810-81-6	Indinavir sulfate	-6.8566
101827-46-7	Butenafine hydrochloride	-6.856
157810-81-6	Indinavir sulfate	-6.8552
481-74-3	Chrysophanic Acid	-6.8549
8024-48-4	Casanthranol	-6.8543
4852-22-6	Proanthocyanidins	-6.8532
157283-68-6	Travoprost	-6.8524
74050-98-9	Ketanserin	-6.8504
58-61-7	Adenosine	-6.8486
23210-58-4	Ifenprodil Tartrate	-6.8483
2753-45-9	Mebeverine hydrochloride	-6.8461
161796-78-7	Esomeprazole Sodium	-6.8458
73590-58-6	Omeprazole	-6.8458
157810-81-6	Indinavir sulfate	-6.8457
65666-07-1	Silymarin	-6.845
8024-48-4	Casanthranol	-6.8444
26864-56-2	Penfluridol	-6.8425
52-86-8	Haloperidol	-6.8386
298-57-7	Stugeron	-6.8369
157810-81-6	Indinavir sulfate	-6.8351
69-33-0	Tubercidin	-6.8344
29908-03-0	Ademetionine	-6.833
286930-03-8	Fesoterodine fumarate	-6.833
1415-73-2	Aloin	-6.8305
8024-48-4	Casanthranol	-6.8291
157810-81-6	Indinavir sulfate	-6.8269
4852-22-6	Proanthocyanidins	-6.8253
29908-03-0	Ademetionine	-6.8238
157810-81-6	Indinavir sulfate	-6.8236
4330-99-8	Trimeprazine tartrate	-6.8231
635702-64-6	Pazopanib HCl	-6.8215
88889-14-9	Fosinopril sodium	-6.8205
274693-27-5	Ticagrelor	-6.8199
444731-52-6	Pazopanib	-6.8194
161796-78-7	Esomeprazole Sodium	-6.8188
1190307-88-0	Sofosbuvir	-6.8186

1223405-08-0	Larotrectinib sulfate	-6.816
364782-34-3	Cinacalcet hydrochloride	-6.8152
10236-47-2	Naringin	-6.8148
65666-07-1	Silymarin	-6.8147
1223403-58-4	Larotrectinib	-6.8139
3200-06-4	Nafronyl oxalate	-6.8137
157810-81-6	Indinavir sulfate	-6.8119
479-18-5	Diprophylline	-6.8114
4852-22-6	Proanthocyanidins	-6.8102
157810-81-6	Indinavir sulfate	-6.8097
118288-08-7	Lafutidine	-6.8094
103639-04-9	Ondansetron hydrochloride	-6.8062
129453-61-8	Fulvestrant	-6.8052
140462-76-6	Olopatadine hydrochloride	-6.8048
157810-81-6	Indinavir sulfate	-6.8047
128794-94-5	Mycophenolate Mofetil	-6.8033
2753-45-9	Mebeverine hydrochloride	-6.8024
639089-54-6	Tozasertib	-6.802
29908-03-0	Ademetionine	-6.8017
1617-90-9	Vincamine	-6.8004
129938-20-1	Dapoxetine HCl	-6.8002
364782-34-3	Cinacalcet hydrochloride	-6.8
95635-55-5	Ranolazine	-6.7991
95635-56-6	Ranolazine dihydrochloride	-6.7991
894-71-3	Nortriptyline hydrochloride	-6.7968
85650-56-2	Asenapine Maleate	-6.7963
520-26-3	Hesperidin	-6.7946
1143-38-0	Anthralin	-6.7939
29908-03-0	Ademetionine	-6.7936
1392275-56-7	Tenofovir alafenamide fumarate	-6.7912
4852-22-6	Proanthocyanidins	-6.7894
88889-14-9	Fosinopril sodium	-6.7879
138530-94-6	R-(+)-Lansoprazole	-6.7876
103577-45-3	Lansoprazole	-6.7876
53-60-1	Promazine hydrochloride	-6.7876
934660-93-2	Cobimetinib	-6.7869
65473-14-5	Naftifine hydrochloride	-6.7853
482-36-0	Hyperoside	-6.7839
65666-07-1	Silymarin	-6.7828
1953-04-4	Galanthamine HBr	-6.7818

1405-10-3	Neomycin sulfate	-6.781
52-86-8	Haloperidol	-6.7794
8024-48-4	Casanthranol	-6.7791
90357-06-5	Bicalutamide	-6.7775
2030-63-9	Clofazimine	-6.7769
157283-68-6	Travoprost	-6.7762
8024-48-4	Casanthranol	-6.7742
60-87-7	Promethazine	-6.7738
129938-20-1	Dapoxetine HCl	-6.7736
1985606-14-1	Baloxavir marboxil	-6.7732
137281-23-3	Pemetrexed acid	-6.7729
1374640-70-6	Rociletinib	-6.7727
520-26-3	Hesperidin	-6.771
157810-81-6	Indinavir sulfate	-6.7708
4852-22-6	Proanthocyanidins	-6.7707
151533-22-1	Levomefolate calcium	-6.7697
4852-22-6	Proanthocyanidins	-6.7681
4852-22-6	Proanthocyanidins	-6.7679
157810-81-6	Indinavir sulfate	-6.7675
157810-81-6	Indinavir sulfate	-6.7663
177834-92-3	Eletriptan HBr	-6.7654
58-61-7	Adenosine	-6.7617
157810-81-6	Indinavir sulfate	-6.7617
1025504-45-3	Valbenazine	-6.7609
157283-68-6	Travoprost	-6.7592
1029044-16-3	Pexidartinib	-6.7591
357166-30-4	Pemetrexed Disodium Hydrate	-6.7577
286930-03-8	Fesoterodine fumarate	-6.7561
157810-81-6	Indinavir sulfate	-6.755
157283-68-6	Travoprost	-6.7541
22888-70-6	Silibinin	-6.7521
95635-55-5	Ranolazine	-6.7514
95635-56-6	Ranolazine dihydrochloride	-6.7514
779353-01-4	Dinaciclib	-6.7503
157810-81-6	Indinavir sulfate	-6.7496
3200-06-4	Nafronyl oxalate	-6.7495
157810-81-6	Indinavir sulfate	-6.7492
54527-84-3	Nicardipine hydrochloride	-6.7487
486460-32-6	Sitagliptin	-6.7483
64872-77-1	Butoconazole nitrate	-6.748

157283-68-6	Travoprost	-6.7478
4146-30-9	Neomycin Sulphate B	-6.7473
1263-89-4	Paromomycin Sulfate	-6.7473
150399-23-8	Pemetrexed disodium	-6.7466
157283-68-6	Travoprost	-6.7461
1263-89-4	Paromomycin Sulfate	-6.746
668270-12-0	Linagliptin	-6.746
14028-44-5	Amoxapine	-6.7438
8024-48-4	Casanthranol	-6.7427
1415-73-2	Aloin	-6.7425
65666-07-1	Silymarin	-6.7416
65666-07-1	Silymarin	-6.7414
1405-10-3	Neomycin sulfate	-6.7411
157283-68-6	Travoprost	-6.741
153-98-0	Serotonin hydrochloride	-6.7407
4330-99-8	Trimeprazine tartrate	-6.7405
65666-07-1	Silymarin	-6.7388
1421373-66-1	Osimertinib mesylate	-6.7384
1201902-80-8	MLN9708	-6.7377
157810-81-6	Indinavir sulfate	-6.7368
157810-81-6	Indinavir sulfate	-6.736
698387-09-6	Neratinib	-6.7356
103577-45-3	Lansoprazole	-6.7354
1421373-65-0	Osimertinib	-6.7351
4852-22-6	Proanthocyanidins	-6.7345
934660-93-2	Cobimetinib	-6.7338
29908-03-0	Ademetionine	-6.7316
8024-48-4	Casanthranol	-6.731
56211-40-6	Torasemide	-6.7302
157810-81-6	Indinavir sulfate	-6.7301
95635-55-5	Ranolazine	-6.7301
64872-77-1	Butoconazole nitrate	-6.7291
167221-71-8	Clevidipine butyrate	-6.7286
1223405-08-0	Larotrectinib sulfate	-6.7275
164579-32-2	Pantoprazole Sodium Hydrate	-6.7275
726169-73-9	Mocetinostat	-6.7269
101827-46-7	Butenafine hydrochloride	-6.7267
157810-81-6	Indinavir sulfate	-6.7263
1223403-58-4	Larotrectinib	-6.7263

2753-45-9	Mebeverine hydrochloride	-6.7255
7770-78-7	Arctigenin	-6.7242
95635-56-6	Ranolazine dihydrochloride	-6.7241
62499-27-8	Gastrodin	-6.722
79307-93-0	Azelastine hydrochloride	-6.7206
19356-17-3	Calcifediol	-6.7199
29908-03-0	Ademetionine	-6.7198
196597-26-9	Ramelteon	-6.7196
747-36-4	Hydroxychloroquine sulfate	-6.7195
60-87-7	Promethazine	-6.7194
198904-31-3	Atazanavir	-6.7194
229975-97-7	Atazanavir sulfate	-6.7194
177834-92-3	Eletriptan HBr	-6.7179
2413-38-9	Fupentixol Dihydrochloride	-6.7174
23210-58-4	Ifenprodil Tartrate	-6.7169
60-87-7	Promethazine	-6.7167
23210-58-4	Ifenprodil Tartrate	-6.7146
479-18-5	Diprophylline	-6.7136
65666-07-1	Silymarin	-6.7126
8024-48-4	Casanthranol	-6.7118
73590-85-9	Omeprazole sulfide	-6.7113
1201902-80-8	MLN9708	-6.7105
65666-07-1	Silymarin	-6.7098
73590-85-9	Omeprazole sulfide	-6.7084
201341-05-1	Tenofovir Disoproxil	-6.7075
151533-22-1	Levomefolate calcium	-6.7073
298-46-4	Carbamazepine	-6.7067
917389-32-3	Letermovir	-6.7067
917389-32-3	Letermovir	-6.7064
4146-30-9	Neomycin Sulphate B	-6.7057
8024-48-4	Casanthranol	-6.7046
29908-03-0	Ademetionine	-6.7046
859-18-7	Lincomycin hydrochloride	-6.7039
668270-12-0	Linagliptin	-6.7037
29216-28-2	Mequitazine	-6.7033
7179-49-9	Lincomycin hydrochloride monohydrate	-6.7021
99592-39-9	Sertaconazole nitrate	-6.702
1263-89-4	Paromomycin Sulfate	-6.7009
65666-07-1	Silymarin	-6.7007
23210-58-4	Ifenprodil Tartrate	-6.7006
1310726-60-3	Upadacitinib	-6.7005

99011-02-6	Imiquimod	-6.7003
37517-28-5	Amikacin	-6.7002
157283-68-6	Travoprost	-6.6995
26807-65-8	Indapamide	-6.6969
151533-22-1	Levomefolate calcium	-6.6961
4880-88-0	Eburnalritardo	-6.6958
138530-94-6	R-(+)-Lansoprazole	-6.6941
103577-45-3	Lansoprazole	-6.6941
25332-39-2	Trazodone hydrochloride	-6.6939
4852-22-6	Proanthocyanidins	-6.6934
747-36-4	Hydroxychloroquine sulfate	-6.6912
59-30-3	Folic acid	-6.6902
157810-81-6	Indinavir sulfate	-6.6901
5534-95-2	Pentagastrin	-6.6891
832720-36-2	Elagolix sodium	-6.689
446-72-0	Genistein	-6.6884
11013-97-1	Methyl hesperidin	-6.6882
446-72-0	Genistein	-6.6876
149022-22-0	Amikacin Sulfate Salt	-6.6874
157810-81-6	Indinavir sulfate	-6.6874
4330-99-8	Trimeprazine tartrate	-6.6858
55268-74-1	Praziquantel	-6.6843
6493-05-6	Pentoxifylline	-6.6841
151533-22-1	Levomefolate calcium	-6.684
4880-88-0	Eburnalritardo	-6.6836
93957-55-2	Fluvastatin sodium salt	-6.6829
24168-96-5	Isoconazole nitrate	-6.6817
7083-71-8	(+)-Emetine dihydrochloride hydrate	-6.6811
481-74-3	Chrysophanic Acid	-6.681
157283-68-6	Travoprost	-6.6805
3200-06-4	Nafronyl oxalate	-6.6792
29908-03-0	Ademetionine	-6.677
3681-99-0	Puerarin	-6.6757
157283-68-6	Travoprost	-6.6748
1029044-16-3	Pexidartinib	-6.6721
482-36-0	Hyperoside	-6.672
479-18-5	Diprophylline	-6.6713
65666-07-1	Silymarin	-6.6685
25316-40-9	Doxorubicin hydrochloride	-6.6681
157810-81-6	Indinavir sulfate	-6.668
157810-81-6	Indinavir sulfate	-6.6675
65666-07-1	Silymarin	-6.6674

25332-39-2	Trazodone hydrochloride	-6.6651
157810-81-6	Indinavir sulfate	-6.6648
1263-89-4	Paromomycin Sulfate	-6.6619
81131-70-6	Pravastatin sodium	-6.6616
1229-29-4	Doxepin hydrochloride	-6.6608
4852-22-6	Proanthocyanidins	-6.6607
111902-57-9	Temocapil	-6.6591
79307-93-0	Azelastine hydrochloride	-6.6585
99592-39-9	Sertaconazole nitrate	-6.6584
157810-81-6	Indinavir sulfate	-6.6582
22888-70-6	Silibinin	-6.6539
65666-07-1	Silymarin	-6.6538
65666-07-1	Silymarin	-6.6512
157810-81-6	Indinavir sulfate	-6.6505
64872-77-1	Butoconazole nitrate	-6.6498
1435-55-8	Hydroquinidine	-6.6481
1421373-66-1	Osimertinib mesylate	-6.6477
289480-64-4	Treprostinil Sodium	-6.645
864750-70-9	Revefenacin	-6.6442
1421373-65-0	Osimertinib	-6.6441
4852-22-6	Proanthocyanidins	-6.6424
161715-24-8	Tebipenem Pivoxil	-6.6405
190786-44-8	Bepotastine Besilate	-6.64
8024-48-4	Casanthranol	-6.638
2753-45-9	Mebeverine hydrochloride	-6.6368
103577-45-3	Lansoprazole	-6.6367
157283-68-6	Travoprost	-6.6366
51773-92-3	Mefloquine hydrochloride	-6.6366
51264-14-3	Amsacrine	-6.6366
5534-95-2	Pentagastrin	-6.6347
23210-58-4	Ifenprodil Tartrate	-6.6323
157810-81-6	Indinavir sulfate	-6.632
117977-21-6	Rabeprazole Related Compound E	-6.6313
157283-68-6	Travoprost	-6.63
88150-42-9	Amlodipine	-6.6277
157810-81-6	Indinavir sulfate	-6.6272
864750-70-9	Revefenacin	-6.6268
8024-48-4	Casanthranol	-6.6251
832720-36-2	Elagolix sodium	-6.6243
157810-81-6	Indinavir sulfate	-6.6225

1297538-32-9	Darolutamide	-6.6223
4330-99-8	Trimeprazine tartrate	-6.6207
2753-45-9	Mebeverine hydrochloride	-6.6207
157810-81-6	Indinavir sulfate	-6.62
29908-03-0	Ademetionine	-6.6198
859-18-7	Lincomycin hydrochloride	-6.6185
7179-49-9	Lincomycin hydrochloride monohydrate	-6.6185
120410-24-4	Biapenem	-6.6184
1405-10-3	Neomycin sulfate	-6.6176
1985606-14-1	Baloxavir marboxil	-6.6175
192725-17-0	Lopinavir	-6.6173
56390-09-1	Epirubicin hydrochloride	-6.6173
157810-81-6	Indinavir sulfate	-6.616
32222-06-3	Calcitriol	-6.6152
1297538-32-9	Darolutamide	-6.6149
37517-28-5	Amikacin	-6.6146
1229-29-4	Doxepin hydrochloride	-6.6136
865759-25-7	Trelagliptin	-6.6135
4330-99-8	Trimeprazine tartrate	-6.6131
1029877-94-8	Trelagliptin succinate	-6.6126
124937-52-6	Tolterodine tartrate	-6.6126
151533-22-1	Levomefolate calcium	-6.6124
151533-22-1	Levomefolate calcium	-6.6122
364782-34-3	Cinacalcet hydrochloride	-6.6102
479-18-5	Diprophylline	-6.6093
105628-07-7	Fasudil hydrochloride	-6.609
486460-32-6	Sitagliptin	-6.608
202189-78-4	Bilastine	-6.6077
172152-50-0	Ilaprazole sodium	-6.6072
157810-81-6	Indinavir sulfate	-6.6068
58-32-2	Dypyridamole	-6.6064
103639-04-9	Ondansetron hydrochloride	-6.6062
132866-11-6	Lercanidipine hydrochloride	-6.606
151767-02-1	Montelukast sodium	-6.6057
4146-30-9	Neomycin Sulphate B	-6.6049
100427-26-7	Lercanidipine	-6.6048
3200-06-4	Nafronyl oxalate	-6.6039
53-60-1	Promazine hydrochloride	-6.6037
157283-68-6	Travoprost	-6.6029
157283-68-6	Travoprost	-6.6026

91-33-8	Benzthiazide	-6.6024
149022-22-0	Amikacin Sulfate Salt	-6.6017
136434-34-9	Duloxetine hydrochloride	-6.6009
3200-06-4	Nafronyl oxalate	-6.6004
149022-22-0	Amikacin Sulfate Salt	-6.6
1985606-14-1	Baloxavir marboxil	-6.5961
69-33-0	Tubercidin	-6.5948
161973-10-0	Esomeprazole Magnesium	-6.5939
117976-90-6	Rebeprazole sodium	-6.5936
83435-67-0	Delapril?Hydrochloride	-6.5927
157810-81-6	Indinavir sulfate	-6.5926
6398-98-7	Amodiaquine hydrochloride	-6.5923
29908-03-0	Ademetionine	-6.5921
157283-68-6	Travoprost	-6.592
1310726-60-3	Upadacitinib	-6.5901
29908-03-0	Ademetionine	-6.5894
7770-78-7	Arctigenin	-6.5887
520-27-4	Diosmin	-6.5886
157810-81-6	Indinavir sulfate	-6.5876
129453-61-8	Fulvestrant	-6.587
726169-73-9	Mocetinostat	-6.5858
22881-35-2	Famprofazone	-6.5855
143388-64-1	Naratriptan	-6.5853
894-71-3	Nortriptyline hydrochloride	-6.583
4852-22-6	Proanthocyanidins	-6.5826
81131-70-6	Pravastatin sodium	-6.5823
157810-81-6	Indinavir sulfate	-6.5814
167465-36-3	Zosuquidar 3HCl	-6.5799
832720-36-2	Elagolix sodium	-6.5795
54063-53-5	Propafenone	-6.579
161796-78-7	Esomeprazole Sodium	-6.5782
73590-58-6	Omeprazole	-6.5782
157810-81-6	Indinavir sulfate	-6.5781
5534-95-2	Pentagastrin	-6.5768
144675-97-8	Pixantrone dimaleate	-6.5762
65666-07-1	Silymarin	-6.5758
29908-03-0	Ademetionine	-6.5754
4852-22-6	Proanthocyanidins	-6.5729
8024-48-4	Casanthranol	-6.5727
4852-22-6	Proanthocyanidins	-6.5726
2753-45-9	Mebeverine hydrochloride	-6.5722

105826-92-4	Tropisetron hydrochloride	-6.571
135729-62-3	Palonosetron hydrochloride	-6.5705
73-22-3	L-Tryptophan	-6.5702
144675-97-8	Pixantrone dimaleate	-6.57
236395-14-5	Eslicarbazepine Acetate	-6.5676
894-71-3	Nortriptyline hydrochloride	-6.5673
157810-81-6	Indinavir sulfate	-6.567
609799-22-6	Tasimelteon	-6.5657
202825-46-5	Safinamide mesylate	-6.5645
157810-81-6	Indinavir sulfate	-6.5643
100643-71-8	Desloratadine	-6.5642
19237-84-4	Prazosin hydrochloride	-6.564
157810-81-6	Indinavir sulfate	-6.5634
163222-33-1	Ezetimibe	-6.5632
196597-26-9	Ramelteon	-6.563
4880-88-0	Eburnalritardo	-6.5626
164579-32-2	Pantoprazole Sodium Hydrate	-6.5621
73-22-3	L-Tryptophan	-6.5611
100299-08-9	Pemirolast potassium	-6.5603
202825-46-5	Safinamide mesylate	-6.5597
88889-14-9	Fosinopril sodium	-6.5593
117977-21-6	Rabeprazole Related Compound E	-6.5589
79559-97-0	Sertraline hydrochloride	-6.5587
157810-81-6	Indinavir sulfate	-6.5587
869113-09-7	Umeclidinium bromide	-6.5575
70-00-8	Trifluridine	-6.5569
85650-56-2	Asenapine Maleate	-6.556
3200-06-4	Nafronyl oxalate	-6.5558
4852-22-6	Proanthocyanidins	-6.5556
29908-03-0	Ademetionine	-6.5555
23210-58-4	Ifenprodil Tartrate	-6.5551
157283-68-6	Travoprost	-6.5549
179474-81-8	Prucalopride	-6.5546
157810-81-6	Indinavir sulfate	-6.554
157810-81-6	Indinavir sulfate	-6.5539
51264-14-3	Amsacrine	-6.5538
120511-73-1	Anastrozole	-6.5537
3810-74-0	Streptomycin sulfate	-6.5537
65473-14-5	Naftifine hydrochloride	-6.5535
157810-81-6	Indinavir sulfate	-6.5532
73151-29-8	Fenticonazole Nitrate	-6.553
610309-89-2	Carvedilol phosphate	-6.5527
72956-09-3	Carvedilol	-6.5524

151533-22-1	Levomefolate calcium	-6.5518
24168-96-5	Isoconazole nitrate	-6.5515
64872-77-1	Butoconazole nitrate	-6.5498
4852-22-6	Proanthocyanidins	-6.5493
122320-73-4	Rosiglitazone	-6.5485
73590-85-9	Omeprazole sulfide	-6.5481
1346242-81-6	Erdafitinib	-6.548
70-00-8	Trifluridine	-6.5467
441798-33-0	Macitentan	-6.5467
524-81-2	Mebhydroline	-6.5465
19237-84-4	Prazosin hydrochloride	-6.5453
157283-68-6	Travoprost	-6.5451
65666-07-1	Silymarin	-6.545
6153-33-9	Mebhydrolin napadisylate	-6.545
22881-35-2	Famprofazone	-6.5442
157810-81-6	Indinavir sulfate	-6.5441
157810-81-6	Indinavir sulfate	-6.5438
157810-81-6	Indinavir sulfate	-6.5433
763113-22-0	Olaparib	-6.5409
91503-79-6	Flurbiprofen Axetil	-6.5406
15676-16-1	Sulpiride	-6.5404
157283-68-6	Travoprost	-6.5394
161973-10-0	Esomeprazole Magnesium	-6.5389
157283-68-6	Travoprost	-6.5385
22881-35-2	Famprofazone	-6.5381
609799-22-6	Tasimelteon	-6.538
100299-08-9	Pemirolast potassium	-6.538
1297538-32-9	Darolutamide	-6.5379
122647-32-9	Ibutilide Fumarate	-6.5378
537-12-2	Diperodon hydrochloride	-6.5374
726169-73-9	Mocetinostat	-6.537
157810-81-6	Indinavir sulfate	-6.5369
157810-81-6	Indinavir sulfate	-6.5364
73590-85-9	Omeprazole sulfide	-6.5362
206658-92-6	Tacrine hydrochloride hydrate	-6.5342
3200-06-4	Nafronyl oxalate	-6.5326
60925-61-3	Ceforanide	-6.5319
69-09-0	Chlorpromazine hydrochloride	-6.5302
7083-71-8	(+)-Emetine dihydrochloride hydrate	-6.5301
161796-78-7	Esomeprazole Sodium	-6.5297
73590-58-6	Omeprazole	-6.5297

24168-96-5	Isoconazole nitrate	-6.5285
123318-82-1	Clofarabine	-6.5284
123318-82-1	Clofarabine	-6.5265
157810-81-6	Indinavir sulfate	-6.5264
698387-09-6	Neratinib	-6.5258
1187595-84-1	Baricitinib?Phosphate	-6.5253
1223405-08-0	Larotrectinib sulfate	-6.5251
1187594-09-7	Baricitinib	-6.525
8024-48-4	Casanthranol	-6.5235
5490-27-7	Dihydrostreptomycin sulfate	-6.5223
610309-89-2	Carvedilol phosphate	-6.5219
868540-17-4	Carfilzomib	-6.5215
29908-03-0	Ademetionine	-6.5213
172152-50-0	Ilaprazole sodium	-6.5204
202825-46-5	Safinamide mesylate	-6.5195
143388-64-1	Naratriptan	-6.5194
313348-27-5	Regadenoson	-6.5192
64019-93-8	Dipivefrin hydrochloride	-6.5184
117977-21-6	Rabeprazole Related Compound E	-6.5183
72956-09-3	Carvedilol	-6.5171
81110-73-8	Racecadotril	-6.5169
153773-82-1	Ertapenem sodium	-6.5165
330784-47-9	Avanafil	-6.5161
122320-73-4	Rosiglitazone	-6.5159
522-51-0	Dequalinium chloride	-6.5158
845614-11-1	Bitopertin	-6.5153
157810-81-6	Indinavir sulfate	-6.5145
25332-39-2	Trazodone hydrochloride	-6.5139
14028-44-5	Amoxapine	-6.5136
15176-29-1	Edoxudine	-6.5116
T5164	Cabozantinib hydrochloride (849217-68-1(free base))	-6.5111
849217-68-1	Cabozantinib	-6.5111
1140909-48-3	Cabozantinib Malate	-6.5111
116649-85-5	Ramatroban	-6.5101
65666-07-1	Silymarin	-6.5094
65473-14-5	Naftifine hydrochloride	-6.509
202189-78-4	Bilastine	-6.5089
1263-89-4	Paromomycin Sulfate	-6.5084
29216-28-2	Mequitazine	-6.5083
132203-70-4	Cilnidipine	-6.5078

157810-81-6	Indinavir sulfate	-6.5077
64019-93-8	Dipivefrin hydrochloride	-6.5071
157810-81-6	Indinavir sulfate	-6.5064
138112-76-2	Agomelatine	-6.5053
29908-03-0	Ademetionine	-6.5047
456-59-7	Cyclandelate	-6.5047
157810-81-6	Indinavir sulfate	-6.5045
157810-81-6	Indinavir sulfate	-6.5041
37517-28-5	Amikacin	-6.5041
136434-34-9	Duloxetine hydrochloride	-6.504
149022-22-0	Amikacin Sulfate Salt	-6.5037
67656-30-8	10-Hydroxycampothecin	-6.5036
64439-81-2	Hydroxy Camptothecine	-6.503
157810-81-6	Indinavir sulfate	-6.5022
81025-04-9	Lactitol monohydrate	-6.5017
81938-43-4	Zofenopril (calcium)	-6.5005
81131-70-6	Pravastatin sodium	-6.5003
111902-57-9	Temocapil	-6.5002
157283-68-6	Travoprost	-6.5002
202138-50-9	Tenofovir Disoproxil Fumarate	-6.5002
151533-22-1	Levomefolate calcium	-6.4991
117977-21-6	Rabeprazole Related Compound E	-6.4982
172152-50-0	Ilaprazole sodium	-6.4981
157810-81-6	Indinavir sulfate	-6.4976
79559-97-0	Sertraline hydrochloride	-6.4974
4880-88-0	Eburnalritardo	-6.4962
444731-52-6	Pazopanib	-6.4957
635702-64-6	Pazopanib HCl	-6.4951
119637-67-1	Moguisteine	-6.4941
1223403-58-4	Larotrectinib	-6.4921
120511-73-1	Anastrozole	-6.4914
4852-22-6	Proanthocyanidins	-6.4914
456-59-7	Cyclandelate	-6.4912
34161-24-5	Fipexide	-6.4912
1137608-69-5	Telotristat Etiprate	-6.4912
23210-58-4	Ifenprodil Tartrate	-6.4912
157810-81-6	Indinavir sulfate	-6.49
61318-91-0	Sulconazole Nitrate	-6.4896
6153-33-9	Mebhydrolin napadisylate	-6.4855
60628-96-8	Bifonazole	-6.4854
29908-03-0	Ademetionine	-6.4853

157810-81-6	Indinavir sulfate	-6.4843
157810-81-6	Indinavir sulfate	-6.4834
30544-47-9	Etofenamate	-6.4832
479-18-5	Diprophylline	-6.4825
524-81-2	Mebhydroline	-6.4822
52-86-8	Haloperidol	-6.4821
73-48-3	Bendroflumethiazide	-6.482
101827-46-7	Butenafine hydrochloride	-6.4816
53251-94-8	Pinaverium bromide	-6.4809
126-27-2	Oxethazaine	-6.4795
202189-78-4	Bilastine	-6.4786
85650-56-2	Asenapine Maleate	-6.4776
190786-44-8	Bepotastine Besilate	-6.4764
65666-07-1	Silymarin	-6.4764
84-02-6	Prochlorperazine Maleate	-6.4757
157810-81-6	Indinavir sulfate	-6.4741
29216-28-2	Mequitazine	-6.474
T5164	Cabozantinib hydrochloride (849217-68-1(free base))	-6.4731
849217-68-1	Cabozantinib	-6.4731
1140909-48-3	Cabozantinib Malate	-6.4731
625115-55-1	Riociguat	-6.473
133099-07-7	Darifenacin HBr	-6.4728
137281-23-3	Pemetrexed acid	-6.4715
179324-69-7	Bortezomib	-6.4705
150399-23-8	Pemetrexed disodium	-6.4697
1143-38-0	Anthralin	-6.4691
132-69-4	Benzydamine hydrochloride	-6.4689
65666-07-1	Silymarin	-6.4688
3810-74-0	Streptomycin sulfate	-6.4677
357166-30-4	Pemetrexed Disodium Hydrate	-6.4676
444731-52-6	Pazopanib	-6.4649
119637-67-1	Moguisteine	-6.4647
635702-64-6	Pazopanib HCl	-6.464
8024-48-4	Casanthranol	-6.4638
157810-81-6	Indinavir sulfate	-6.4634
122647-32-9	Ibutilide Fumarate	-6.463
151533-22-1	Levomefolate calcium	-6.4619
68099-86-5	Bepridil hydrochloride	-6.4612
60925-61-3	Ceforanide	-6.4602
111902-57-9	Temocapil	-6.4598
15176-29-1	Edoxudine	-6.4591
123447-62-1	Prulifloxacin	-6.4572

1263-89-4	Paromomycin Sulfate	-6.4566
157810-81-6	Indinavir sulfate	-6.4557
1025967-78-5	Lifitegrast	-6.4556
165800-03-3	Linezolid	-6.4551
868540-17-4	Carfilzomib	-6.4534
302962-49-8	Dasatinib	-6.4532
157810-81-6	Indinavir sulfate	-6.453
863127-77-9	Dasatinib monohydrate	-6.4529
111902-57-9	Temocapil	-6.4524
621-72-7	Bendazol	-6.4512
26807-65-8	Indapamide	-6.4512
456-59-7	Cyclandelate	-6.4487
151533-22-1	Levomefolate calcium	-6.4486
157283-68-6	Travoprost	-6.4484
4880-88-0	Eburnalritardo	-6.4477
90-33-5	4-Methylumbelliferone	-6.4473
93793-83-0	Roxatidine Acetate hydrochloride	-6.4469
28721-07-5	Oxcarbazepine	-6.4458
486-35-1	Daphnetin	-6.4453
151533-22-1	Levomefolate calcium	-6.4448
151533-22-1	Levomefolate calcium	-6.4448
27833-64-3	Loxapine succinate salt	-6.4447
53251-94-8	Pinaverium bromide	-6.4445
1143-38-0	Anthralin	-6.4434
149022-22-0	Amikacin Sulfate Salt	-6.4433
22832-87-7	Miconazole nitrate	-6.4427
99592-39-9	Sertaconazole nitrate	-6.4391
120410-24-4	Biapenem	-6.439
161973-10-0	Esomeprazole Magnesium	-6.4388
1269440-17-6	Encorafenib	-6.438
1137608-69-5	Telotristat Etiprate	-6.4379
82586-55-8	Quinapril hydrochloride	-6.4373
157810-81-6	Indinavir sulfate	-6.4372
313348-27-5	Regadenoson	-6.4365
157810-81-6	Indinavir sulfate	-6.4363
496775-62-3	Eltrombopag Olamine	-6.4347
157810-81-6	Indinavir sulfate	-6.4343
58579-51-4	Anagrelide HCl	-6.4341
68475-42-3	Anagrelide	-6.4341
157810-81-6	Indinavir sulfate	-6.4335

157283-68-6	Travoprost	-6.4325
485-49-4	(+)-Bicuculline	-6.4325
1405-10-3	Neomycin sulfate	-6.4321
4146-30-9	Neomycin Sulphate B	-6.4317
59263-76-2	Meptazinol hydrochloride	-6.4308
1446502-11-9	Enasidenib	-6.4302
122852-69-1	Alosetron hydrochloride	-6.4299
6398-98-7	Amodiaquine hydrochloride	-6.4298
84-02-6	Prochlorperazine Maleate	-6.4295
23210-58-4	Ifenprodil Tartrate	-6.4295
1260141-27-2	Vonoprazan fumarate	-6.4295
37693-01-9	Clofoctol	-6.4294
17388-39-5	Swertiamarin	-6.4289
157810-81-6	Indinavir sulfate	-6.4282
1435-55-8	Hydroquinidine	-6.4278
52-86-8	Haloperidol	-6.4262
75507-68-5	Flupirtine maleate	-6.4261
133099-07-7	Darifenacin HBr	-6.426
79559-97-0	Sertraline hydrochloride	-6.4257
73590-85-9	Omeprazole sulfide	-6.4254
6202-23-9	Cyclobenzaprine hydrochloride	-6.4254
122647-32-9	Ibutilide Fumarate	-6.4247
56-69-9	5-hydroxytryptophan	-6.424
157810-81-6	Indinavir sulfate	-6.4234
485-49-4	(+)-Bicuculline	-6.4222
202189-78-4	Bilastine	-6.422
157810-81-6	Indinavir sulfate	-6.4213
23210-58-4	Ifenprodil Tartrate	-6.4211
172152-50-0	Ilaprazole sodium	-6.4194
157810-81-6	Indinavir sulfate	-6.4193
132-69-4	Benzydamine hydrochloride	-6.419
79307-93-0	Azelastine hydrochloride	-6.4189
144675-97-8	Pixantrone dimaleate	-6.4179
84-02-6	Prochlorperazine Maleate	-6.4169
10402-53-6	Eprazinone dihydrochloride	-6.4168
274693-27-5	Ticagrelor	-6.4166
2413-38-9	Fupentixol Dihydrochloride	-6.4165
65666-07-1	Silymarin	-6.4164
157810-81-6	Indinavir sulfate	-6.4159
161715-24-8	Tebipenem Pivoxil	-6.4159

1187594-09-7	Baricitinib	-6.4158
1187595-84-1	Baricitinib?Phosphate	-6.4158
116649-85-5	Ramatroban	-6.4155
126-27-2	Oxethazaine	-6.4152
59-30-3	Folic acid	-6.4126
112809-51-5	Letrozole	-6.4109
3200-06-4	Nafronyl oxalate	-6.4107
1187594-09-7	Baricitinib	-6.4106
1187595-84-1	Baricitinib?Phosphate	-6.4106
574-84-5	Fraxetin	-6.4104
136434-34-9	Duloxetine hydrochloride	-6.4103
157810-81-6	Indinavir sulfate	-6.4102
10402-53-6	Eprazinone dihydrochloride	-6.4101
202189-78-4	Bilastine	-6.4096
50-91-9	Floxuridine	-6.4091
151533-22-1	Levomefolate calcium	-6.4083
747-36-4	Hydroxychloroquine sulfate	-6.4083
65473-14-5	Naftifine hydrochloride	-6.4079
93957-55-2	Fluvastatin sodium salt	-6.4073
869113-09-7	Umeclidinium bromide	-6.4072
85650-56-2	Asenapine Maleate	-6.4065
7085-55-4	Troxerutin	-6.4054
111902-57-9	Temocapil	-6.405
90-45-9	Aminacrine	-6.4043
103639-04-9	Ondansetron hydrochloride	-6.4042
37517-28-5	Amikacin	-6.4037
2413-38-9	Fupentixol Dihydrochloride	-6.4036
6469-93-8	Chlorprothixene hydrochloride	-6.4032
113-59-7	Chlorprothixene	-6.4032
5534-95-2	Pentagastrin	-6.4029
157810-81-6	Indinavir sulfate	-6.4026
108929-04-0	Epinastine HCl	-6.4021
29908-03-0	Ademetionine	-6.4021
67227-57-0	Fenoldopam mesylate	-6.4012
574-84-5	Fraxetin	-6.4003
88889-14-9	Fosinopril sodium	-6.4002
157810-81-6	Indinavir sulfate	-6.4002
67227-57-0	Fenoldopam mesylate	-6.3987
1617-90-9	Vincamine	-6.3984
868540-17-4	Carfilzomib	-6.3974

130-16-5	Dermofongin	-6.397
17388-39-5	Swertiamarin	-6.3963
157810-81-6	Indinavir sulfate	-6.3944
13636-18-5	Fendiline hydrochloride	-6.3941
50-14-6	Vitamin D2	-6.3932
112665-43-7	Seratrodast	-6.3924
161715-24-8	Tebipenem Pivoxil	-6.3922
3200-06-4	Nafronyl oxalate	-6.392
73590-85-9	Omeprazole sulfide	-6.392
7770-78-7	Arctigenin	-6.3914
30123-17-2	Tianeptine sodium	-6.3905
763113-22-0	Olaparib	-6.3905
72509-76-3	Felodipine	-6.3899
112665-43-7	Seratrodast	-6.3896
167933-07-5	Flibanserin	-6.3894
95635-56-6	Ranolazine dihydrochloride	-6.3893
23210-58-4	Ifenprodil Tartrate	-6.3886
28721-07-5	Oxcarbazepine	-6.3879
941685-37-6	S-Ruxolitinib	-6.3874
138112-76-2	Agomelatine	-6.3846
58-28-6	Desipramine hydrochloride	-6.384
2627-69-2	AICAR (Acadesine)	-6.3836
122647-32-9	Ibutilide Fumarate	-6.3835
79307-93-0	Azelastine hydrochloride	-6.3831
31637-97-5	Etofibrate	-6.3827
843663-66-1	Bedaquiline	-6.3823
23210-58-4	Ifenprodil Tartrate	-6.3812
86780-90-7	Aranidipine	-6.3812
25775-90-0	Zucapsaicin	-6.3811
103639-04-9	Ondansetron hydrochloride	-6.3801
111406-87-2	Zileuton	-6.3788
91-33-8	Benzthiazide	-6.3779
167465-36-3	Zosuquidar 3HCl	-6.3775
164579-32-2	Pantoprazole Sodium Hydrate	-6.3762
111902-57-9	Temocapil	-6.376
122852-69-1	Alosetron hydrochloride	-6.3751
95635-55-5	Ranolazine	-6.3749
29908-03-0	Ademetionine	-6.373
90-45-9	Aminacrine	-6.3726
65473-14-5	Naftifine hydrochloride	-6.3716
1435-55-8	Hydroquinidine	-6.3698
178307-42-1	Revaprazan hydrochloride	-6.3693
475-67-2	S-Isocorydine(+)	-6.3686

T4284	(S)-Amlodipine Besylate (103129-82-4(free base))	-6.3683
88150-42-9	Amlodipine	-6.3683
129938-20-1	Dapoxetine HCl	-6.3682
157810-81-6	Indinavir sulfate	-6.3681
50-91-9	Floxuridine	-6.3669
132-69-4	Benzydamine hydrochloride	-6.3668
50-63-5	Chloroquine diphosphate	-6.3665
138530-94-6	R-(+)-Lansoprazole	-6.3664
103577-45-3	Lansoprazole	-6.3664
79307-93-0	Azelastine hydrochloride	-6.3657
99592-39-9	Sertaconazole nitrate	-6.3656
4852-22-6	Proanthocyanidins	-6.3655
446-72-0	Genistein	-6.3648
65666-07-1	Silymarin	-6.364
111902-57-9	Temocapil	-6.3639
115956-12-2	Dolasetron	-6.3629
1405-10-3	Neomycin sulfate	-6.3629
440-17-5	Trifluoperazine dihydrochloride	-6.3629
17321-77-6	Clomipramine hydrochloride	-6.3619
122647-32-9	Ibutilide Fumarate	-6.3618
456-59-7	Cyclandelate	-6.3613
50-14-6	Vitamin D2	-6.3603
10083-24-6	Piceatannol	-6.36
65928-58-7	Dienogest	-6.3596
1454846-35-5	Lorlatinib	-6.3591
1263-89-4	Paromomycin Sulfate	-6.3576
161715-24-8	Tebipenem Pivoxil	-6.3575
132866-11-6	Lercanidipine hydrochloride	-6.3574
100427-26-7	Lercanidipine	-6.3571
132539-06-1	Olanzapine	-6.3558
157810-81-6	Indinavir sulfate	-6.3548
54063-53-5	Propafenone	-6.3544
27848-84-6	Nicergoline	-6.3538
124937-52-6	Tolterodine tartrate	-6.3534
157810-81-6	Indinavir sulfate	-6.3533
635728-49-3	Darunavir?Ethanolate	-6.353
206361-99-1	Darunavir	-6.3527
61318-91-0	Sulconazole Nitrate	-6.3527
73590-58-6	Omeprazole	-6.3521
67656-30-8	10-Hydroxycampothecin	-6.3519
19685-09-7	10-Hydroxycamptothecin	-6.3519
64-86-8	Colchicine	-6.3515

163252-36-6	Clevudine	-6.3513
1985606-14-1	Baloxavir marboxil	-6.3513
37517-28-5	Amikacin	-6.3493
4146-30-9	Neomycin Sulphate B	-6.3492
64439-81-2	Hydroxy Camptothecine	-6.3492
568-73-0	Tanshinone I	-6.3486
54063-53-5	Propafenone	-6.3482
441798-33-0	Macitentan	-6.348
78246-49-8	Paroxetine hydrochloride	-6.3475
330784-47-9	Avanafil	-6.3471
80433-71-2	Calcium Levofolinate	-6.3458
88889-14-9	Fosinopril sodium	-6.3456
151533-22-1	Levomefolate calcium	-6.3453
26807-65-8	Indapamide	-6.3451
157810-81-6	Indinavir sulfate	-6.3446
157283-68-6	Travoprost	-6.3445
151533-22-1	Levomefolate calcium	-6.344
177834-92-3	Eletriptan HBr	-6.3437
2062-78-4	Pimozide	-6.3429
65513-72-6	Glafenine Hydrochloride	-6.3419
3820-67-5	Glafenine	-6.3419
65666-07-1	Silymarin	-6.3418
29908-03-0	Ademetionine	-6.3417
4852-22-6	Proanthocyanidins	-6.3417
11018-89-6	Ouabain octahydrate	-6.3411
157810-81-6	Indinavir sulfate	-6.3407
80-49-9	Homatropine Methylbromide	-6.3404
7083-71-8	(+)-Emetine dihydrochloride hydrate	-6.3404
65666-07-1	Silymarin	-6.3393
915087-33-1	Enzalutamide	-6.3393
23210-58-4	Ifenprodil Tartrate	-6.339
56-69-9	5-hydroxytryptophan	-6.3389
3717-88-2	Flavoxate hydrochloride	-6.3379
6402-23-9	Ethacridine lactate monohydrate	-6.3377
65666-07-1	Silymarin	-6.3374
1263-89-4	Paromomycin Sulfate	-6.3373
111406-87-2	Zileuton	-6.336
157810-81-6	Indinavir sulfate	-6.3359
130-16-5	Dermofongin	-6.3349
1837-57-6	Ethacridine lactate	-6.3344
65666-07-1	Silymarin	-6.3342
23541-50-6	Daunorubicin hydrochloride	-6.334

129453-61-8	Fulvestrant	-6.3339
89396-94-1	Imidapril HCl	-6.3335
61318-91-0	Sulconazole Nitrate	-6.3318
1092939-17-7	Ruxolitinib (INCB-18424) phosphate	-6.3313
941678-49-5	Ruxolitinib	-6.3313
206658-92-6	Tacrine hydrochloride hydrate	-6.3306
73151-29-8	Fenticonazole Nitrate	-6.3306
61-73-4	Methylene Blue	-6.3301
7220-79-3	Methylene Blue trihydrate	-6.3301
55268-74-1	Praziquantel	-6.3299
14028-44-5	Amoxapine	-6.3294
747-36-4	Hydroxychloroquine sulfate	-6.3286
1617-90-9	Vincamine	-6.3283
157810-81-6	Indinavir sulfate	-6.3275
157810-81-6	Indinavir sulfate	-6.327
88889-14-9	Fosinopril sodium	-6.3267
17560-51-9	Metolazone	-6.326
133099-07-7	Darifenacin HBr	-6.3247
157810-81-6	Indinavir sulfate	-6.3247
105628-07-7	Fasudil hydrochloride	-6.3244
67227-57-0	Fenoldopam mesylate	-6.3236
105826-92-4	Tropisetron hydrochloride	-6.3235
163521-12-8	Vilazodone	-6.3222
78628-80-5	Terbinafine hydrochloride	-6.3217
163521-08-2	Vilazodone HCl	-6.3217
440-17-5	Trifluoperazine dihydrochloride	-6.3211
5490-27-7	Dihydrostreptomycin sulfate	-6.3206
961-29-5	Isoliquiritigenin	-6.3196
67814-76-0	Isepamicin sulfate	-6.3192
22888-70-6	Silibinin	-6.3187
90-45-9	Aminacrine	-6.3178
157810-81-6	Indinavir sulfate	-6.3172
4852-22-6	Proanthocyanidins	-6.317
440-17-5	Trifluoperazine dihydrochloride	-6.317
167221-71-8	Clevidipine butyrate	-6.3167
122852-69-1	Alosetron hydrochloride	-6.3162
1617-90-9	Vincamine	-6.3159
116649-85-5	Ramatroban	-6.3151
24168-96-5	Isoconazole nitrate	-6.315
69558-55-0	Thymopentin	-6.3149
82-02-0	Khellin	-6.3141
22881-35-2	Famprofazone	-6.3138

946075-13-4	Isavuconazonium sulfate	-6.3138
5578-73-4	Sanguinarine chloride	-6.3128
65928-58-7	Dienogest	-6.3126
64439-81-2	Hydroxy Camptothecine	-6.3118
163521-08-2	Vilazodone HCl	-6.3109
29908-03-0	Ademetionine	-6.3106
17388-39-5	Swertiamarin	-6.3099
157810-81-6	Indinavir sulfate	-6.3098
56296-78-7	Fluoxetine hydrochloride	-6.3097
54910-89-3	Fluoxetine	-6.3097
67656-30-8	10-Hydroxycampothecin	-6.3094
19685-09-7	10-Hydroxycamptothecin	-6.3094
574-84-5	Fraxetin	-6.3085
65473-14-5	Naftifine hydrochloride	-6.3082
23210-58-4	Ifenprodil Tartrate	-6.3072
104227-87-4	Famciclovir	-6.3062
157810-81-6	Indinavir sulfate	-6.306
3564-73-6	10,11-Dihydrocarbamazepine	-6.3054
161973-10-0	Esomeprazole Magnesium	-6.3048
2627-69-2	AICAR (Acadesine)	-6.3042
157810-81-6	Indinavir sulfate	-6.3026
1229-29-4	Doxepin hydrochloride	-6.302
4852-22-6	Proanthocyanidins	-6.3019
630420-16-5	Asunaprevir	-6.3018
1260141-27-2	Vonoprazan fumarate	-6.3018
668270-12-0	Linagliptin	-6.3004
89796-99-6	Aceclofenac	-6.3002
117977-21-6	Rabeprazole Related Compound E	-6.3002
1446502-11-9	Enasidenib	-6.2999
747-36-4	Hydroxychloroquine sulfate	-6.2985
24168-96-5	Isoconazole nitrate	-6.2979
83435-67-0	Delapril?Hydrochloride	-6.2978
69-09-0	Chlorpromazine hydrochloride	-6.2977
65513-72-6	Glafenine Hydrochloride	-6.2974
3820-67-5	Glafenine	-6.2974
86780-90-7	Aranidipine	-6.297
58579-51-4	Anagrelide HCl	-6.296
68475-42-3	Anagrelide	-6.296
69-33-0	Tubercidin	-6.2952
67-03-8	Thiamine hydrochloride	-6.2933
609799-22-6	Tasimelteon	-6.2931

157810-81-6	Indinavir sulfate	-6.2927
61-73-4	Methylene Blue	-6.2927
7220-79-3	Methylene Blue trihydrate	-6.2927
394730-60-0	Boceprevir	-6.2924
29908-03-0	Ademetionine	-6.2923
100299-08-9	Pemirolast potassium	-6.2912
111902-57-9	Temocapil	-6.29
24168-96-5	Isoconazole nitrate	-6.2885
67227-57-0	Fenoldopam mesylate	-6.288
143491-57-0	Emtricitabine	-6.2869
108929-04-0	Epinastine HCl	-6.2869
99011-02-6	Imiquimod	-6.2858
49763-96-4	Stiripentol	-6.2853
187164-19-8	Luliconazole	-6.2848
30516-87-1	Zidovudine	-6.2846
99592-39-9	Sertaconazole nitrate	-6.2842
13636-18-5	Fendiline hydrochloride	-6.2839
865759-25-7	Trelagliptin	-6.2836
157810-81-6	Indinavir sulfate	-6.2835
1029877-94-8	Trelagliptin succinate	-6.283
106730-54-5	Olprinone	-6.2829
79794-75-5	Loratadine	-6.2823
157810-81-6	Indinavir sulfate	-6.2813
111902-57-9	Temocapil	-6.2811
3759-92-0	Furaltadone hydrochloride	-6.2805
65666-07-1	Silymarin	-6.2797
120410-24-4	Biapenem	-6.2797
36791-04-5	Ribavirin	-6.2796
64872-77-1	Butoconazole nitrate	-6.2794
22832-87-7	Miconazole nitrate	-6.279
50-63-5	Chloroquine diphosphate	-6.2786
120410-24-4	Biapenem	-6.2782
51803-78-2	Nimesulide	-6.2778
54527-84-3	Nicardipine hydrochloride	-6.2777
869113-09-7	Umeclidinium bromide	-6.2772
706782-28-7	Pimavanserin tartrate	-6.2767
706779-91-1	Pimavanserin	-6.2767
157810-81-6	Indinavir sulfate	-6.2764
157810-81-6	Indinavir sulfate	-6.2764
1201902-80-8	MLN9708	-6.2757
122320-73-4	Rosiglitazone	-6.2751

29908-03-0	Ademetionine	-6.2748
60925-61-3	Ceforanide	-6.2745
53-16-7	Aquacrine	-6.2744
1448347-49-6	Ivosidenib	-6.274
157810-81-6	Indinavir sulfate	-6.2739
118288-08-7	Lafutidine	-6.2738
22881-35-2	Famprofazone	-6.2733
25316-40-9	Doxorubicin hydrochloride	-6.2733
83915-83-7	Lisinopril dihydrate	-6.2731
206658-92-6	Tacrine hydrochloride hydrate	-6.2731
1421373-65-0	Osimertinib	-6.2726
22881-35-2	Famprofazone	-6.2726
170105-16-5	Imidafenacin	-6.2723
1092939-17-7	Ruxolitinib (INCB-18424) phosphate	-6.2715
941678-49-5	Ruxolitinib	-6.2715
6024-85-7	Tetrahydropalmatine hydrochloride	-6.2712
154361-50-9	Capecitabine	-6.2706
76095-16-4	Enalapril Maleate	-6.2704
172152-50-0	Ilaprazole sodium	-6.2693
25316-40-9	Doxorubicin hydrochloride	-6.2692
33286-22-5	Diltiazem hydrochloride	-6.2692
74050-98-9	Ketanserin	-6.2691
101827-46-7	Butenafine hydrochloride	-6.2689
17321-77-6	Clomipramine hydrochloride	-6.2683
906093-29-6	Teneligliptin hydrobromide	-6.2681
85650-56-2	Asenapine Maleate	-6.2678
6153-33-9	Mebhydrolin napadisylate	-6.2676
6398-98-7	Amodiaquine hydrochloride	-6.2674
111406-87-2	Zileuton	-6.2672
54063-53-5	Propafenone	-6.267
53251-94-8	Pinaverium bromide	-6.2669
39562-70-4	Nitrendipine	-6.2668
187164-19-8	Luliconazole	-6.266
524-81-2	Mebhydroline	-6.2658
30544-47-9	Etofenamate	-6.2644
1421373-66-1	Osimertinib mesylate	-6.2642
147526-32-7	Pitavastatin calcium	-6.2635
524-81-2	Mebhydroline	-6.2625
120511-73-1	Anastrozole	-6.2624
13636-18-5	Fendiline hydrochloride	-6.2624

6153-33-9	Mebhydrolin napadisylate	-6.2622
1229208-44-9	Entospletinib	-6.2622
1454846-35-5	Lorlatinib	-6.2622
479-18-5	Diprophylline	-6.2614
146-56-5	Fluphenazine hydrochloride	-6.2608
21462-39-5	Clindamycin HCl	-6.2602
111902-57-9	Temocapil	-6.2601
58-27-5	Menadione	-6.2599
133099-07-7	Darifenacin HBr	-6.2599
25332-39-2	Trazodone hydrochloride	-6.2599
150915-41-6	Perospirone	-6.2584
808118-40-3	Roxadustat	-6.2578
140462-76-6	Olopatadine hydrochloride	-6.2576
157810-81-6	Indinavir sulfate	-6.2575
13636-18-5	Fendiline hydrochloride	-6.2571
39562-70-4	Nitrendipine	-6.2569
95635-55-5	Ranolazine	-6.2569
157810-81-6	Indinavir sulfate	-6.2566
157283-68-6	Travoprost	-6.2563
155270-99-8	Istradefylline	-6.2563
26807-65-8	Indapamide	-6.2555
95635-56-6	Ranolazine dihydrochloride	-6.2554
157810-81-6	Indinavir sulfate	-6.2551
157810-81-6	Indinavir sulfate	-6.254
90357-06-5	Bicalutamide	-6.2537
61318-91-0	Sulconazole Nitrate	-6.2527
157810-81-6	Indinavir sulfate	-6.2527
4330-99-8	Trimeprazine tartrate	-6.2525
79307-93-0	Azelastine hydrochloride	-6.2522
1152311-62-0	VX661	-6.2518
108612-45-9	Mizolastine	-6.2507
148849-67-6	Ivabradine hydrochloride	-6.2507
2259-96-3	Cyclothiazide	-6.2487
157810-81-6	Indinavir sulfate	-6.2486
99291-25-5	Levodropropizine	-6.2476
603139-19-1	Odanacatib	-6.2473
112665-43-7	Seratrodast	-6.2468
26807-65-8	Indapamide	-6.2465
29908-03-0	Ademetionine	-6.2458
25913-34-2	Tinoridine hydrochloride	-6.2457

157810-81-6	Indinavir sulfate	-6.2452
7179-49-9	Lincomycin hydrochloride monohydrate	-6.2441
68-89-3	Dipyron	-6.2437
5907-38-0	Analgin	-6.2437
859-18-7	Lincomycin hydrochloride	-6.2435
63372-86-1	Metamizole magnesium	-6.2434
105628-07-7	Fasudil hydrochloride	-6.2432
27833-64-3	Loxapine succinate salt	-6.2425
58-28-6	Desipramine hydrochloride	-6.2413
59263-76-2	Meptazinol hydrochloride	-6.241
51773-92-3	Mefloquine hydrochloride	-6.2409
157810-81-6	Indinavir sulfate	-6.2403
65666-07-1	Silymarin	-6.2401
4146-30-9	Neomycin Sulphate B	-6.2399
13636-18-5	Fendiline hydrochloride	-6.2392
93957-55-2	Fluvastatin sodium salt	-6.2384
485-49-4	(+)-Bicuculline	-6.2383
111902-57-9	Temocapil	-6.2381
84057-95-4	Ropivacaine	-6.2377
98717-15-8	Ropivacaine hydrochloride	-6.2377
62893-19-0	Cefoperazone	-6.2362
58-28-6	Desipramine hydrochloride	-6.236
73816-42-9	Meclocycline sulfosalicylate salt	-6.2341
53885-35-1	Ticlopidine hydrochloride	-6.2337
23210-58-4	Ifenprodil Tartrate	-6.2336
50-63-5	Chloroquine diphosphate	-6.2333
198904-31-3	Atazanavir	-6.2329
229975-97-7	Atazanavir sulfate	-6.2329
67-03-8	Thiamine hydrochloride	-6.2328
29908-03-0	Ademetionine	-6.2322
178307-42-1	Revaprazan hydrochloride	-6.2314
2413-38-9	Fupentixol Dihydrochloride	-6.2314
108929-04-0	Epinastine HCl	-6.2314
2062-78-4	Pimozide	-6.2311
123447-62-1	Prulifloxacin	-6.2298
845614-11-1	Bitopertin	-6.2296
456-59-7	Cyclandelate	-6.228
456-59-7	Cyclandelate	-6.2275
68786-66-3	Triclabendazole	-6.2274
73151-29-8	Fenticonazole Nitrate	-6.2273
29908-03-0	Ademetionine	-6.2273
313348-27-5	Regadenoson	-6.2269
1811-28-5	Proflavine Hemisulfate	-6.2264

491-67-8	Baicalein	-6.2256
126544-47-6	Ciclesonide	-6.2255
68786-66-3	Triclabendazole	-6.2251
53885-35-1	Ticlopidine hydrochloride	-6.2249
568-73-0	Tanshinone I	-6.2249
1092939-17-7	Ruxolitinib (INCB-18424) phosphate	-6.2247
941678-49-5	Ruxolitinib	-6.2247
747-36-4	Hydroxychloroquine sulfate	-6.2247
621-72-7	Bendazol	-6.224
7085-55-4	Troxerutin	-6.2234
88889-14-9	Fosinopril sodium	-6.2232
91503-79-6	Flurbiprofen Axetil	-6.223
202138-50-9	Tenofovir Disoproxil Fumarate	-6.2222
1405-10-3	Neomycin sulfate	-6.222
30516-87-1	Zidovudine	-6.2216
808118-40-3	Roxadustat	-6.2208
151533-22-1	Levomefolate calcium	-6.2207
1263-89-4	Paromomycin Sulfate	-6.2192
38304-91-5	Minoxidil	-6.2189
157810-81-6	Indinavir sulfate	-6.2189
23210-58-4	Ifenprodil Tartrate	-6.2184
832720-36-2	Elagolix sodium	-6.2173
157810-81-6	Indinavir sulfate	-6.2171
73-48-3	Bendroflumethiazide	-6.2159
167465-36-3	Zosuquidar 3HCl	-6.2156
143491-57-0	Emtricitabine	-6.2144
106308-44-5	Rufinamide	-6.214
69-81-8	Carbazochrome	-6.2139
89396-94-1	Imidapril HCl	-6.213
68099-86-5	Bepridil hydrochloride	-6.2127
941685-37-6	S-Ruxolitinib	-6.2124
219861-08-2	Escitalopram Oxalate	-6.2117
59729-32-7	Citalopram HBr	-6.2117
1084-65-7	Meticrane	-6.2116
90-33-5	4-Methylumbelliferone	-6.2112
91503-79-6	Flurbiprofen Axetil	-6.2111
65928-58-7	Dienogest	-6.2107
99592-39-9	Sertaconazole nitrate	-6.2091
537-12-2	Diperodon hydrochloride	-6.209
154361-50-9	Capecitabine	-6.2083
103628-48-4	Sumatriptan succinate	-6.2079
524-81-2	Mebhydroline	-6.2078

6153-33-9	Mebhydrolin napadisylate	-6.2078
132036-88-5	Ramosetron	-6.2077
1098-97-1	Pyrithioxin	-6.2068
5534-95-2	Pentagastrin	-6.2048
157810-81-6	Indinavir sulfate	-6.2044
14984-68-0	Cloperastine hydrochloride	-6.2035
808118-40-3	Roxadustat	-6.2029
111902-57-9	Temocapil	-6.2024
58-32-2	Dypyridamole	-6.2024
55268-74-1	Praziquantel	-6.2021
117976-90-6	Rebeprazole sodium	-6.2019
6202-23-9	Cyclobenzaprine hydrochloride	-6.2014
120014-06-4	Donepezil	-6.2012
107007-99-8	Granisetron hydrochloride	-6.2009
82-02-0	Khellin	-6.2007
906093-29-6	Teneligliptin hydrobromide	-6.2007
120011-70-3	Donepezil HCl	-6.2003
856867-55-5	Tedizolid Phosphate	-6.2002
89796-99-6	Aceclofenac	-6.1999
170105-16-5	Imidafenacin	-6.1995
190786-44-8	Bepotastine Besilate	-6.1994
157810-81-6	Indinavir sulfate	-6.1988
123524-52-7	Azelnidipine	-6.1986
163521-08-2	Vilazodone HCl	-6.1986
147816-24-8	Cefcapene Pivoxil Hydrochloride	-6.1983
117976-90-6	Rebeprazole sodium	-6.1968
52549-17-4	Pranoprofen	-6.1967
132036-88-5	Ramosetron	-6.1964
157283-68-6	Travoprost	-6.1963
51264-14-3	Amsacrine	-6.1962
52645-53-1	Permethrin	-6.196
4394-00-7	Niflumic acid	-6.1959
1297538-32-9	Darolutamide	-6.1959
26807-65-8	Indapamide	-6.1945
157810-81-6	Indinavir sulfate	-6.1918
79794-75-5	Loratadine	-6.1912
6493-05-6	Pentoxifylline	-6.1907
522-51-0	Dequalinium chloride	-6.1904
87233-61-2	Emedastine	-6.189
177834-92-3	Eletriptan HBr	-6.189
29908-03-0	Ademetionine	-6.1889
269055-15-4	Etravirine	-6.1885

101827-46-7	Butenafine hydrochloride	-6.1881
73590-85-9	Omeprazole sulfide	-6.1866
69-09-0	Chlorpromazine hydrochloride	-6.1864
25913-34-2	Tinoridine hydrochloride	-6.1857
99592-39-9	Sertaconazole nitrate	-6.1853
364622-82-2	Doripenem Hydrate	-6.1831
66085-59-4	Nimodipine	-6.183
53885-35-1	Ticlopidine hydrochloride	-6.1819
120202-66-6	(S)-(+)-Clopidogrel hydrogen sulfate	-6.1791
29908-03-0	Ademetionine	-6.1789
117977-21-6	Rabeprazole Related Compound E	-6.1781
64439-81-2	Hydroxy Camptothecine	-6.178
84-02-6	Prochlorperazine Maleate	-6.1779
906093-29-6	Teneligliptin hydrobromide	-6.1778
70797-11-4	Cefpiramide acid	-6.1774
79307-93-0	Azelastine hydrochloride	-6.1772
61-73-4	Methylene Blue	-6.177
7220-79-3	Methylene Blue trihydrate	-6.177
148849-67-6	Ivabradine hydrochloride	-6.1763
202825-46-5	Safinamide mesylate	-6.1755
157283-68-6	Travoprost	-6.1754
698387-09-6	Neratinib	-6.1753
22161-81-5	(S)-(+)-Ketoprofen	-6.175
22071-15-4	Ketoprofen	-6.175
1210344-57-2	Ertugliflozin	-6.1744
157810-81-6	Indinavir sulfate	-6.1743
856866-72-3	Torezolid	-6.1739
67656-30-8	10-Hydroxycampothecin	-6.1738
34140-59-5	Trimebutine maleate	-6.1738
39133-31-8	Trimebutine	-6.1738
747-36-4	Hydroxychloroquine sulfate	-6.1737
82-02-0	Khellin	-6.1733
157810-81-6	Indinavir sulfate	-6.1732
84057-95-4	Ropivacaine	-6.173
98717-15-8	Ropivacaine hydrochloride	-6.173
61318-91-0	Sulconazole Nitrate	-6.1714
108929-04-0	Epinastine HCl	-6.1713
1152311-62-0	VX661	-6.1709
115256-11-6	Dofetilide	-6.1707
157810-81-6	Indinavir sulfate	-6.1704
99291-25-5	Levodropropizine	-6.17

29218-27-7	MD 69276	-6.17
3564-73-6	10,11-Dihydrocarbamazepine	-6.1699
1201902-80-8	MLN9708	-6.1696
157810-81-6	Indinavir sulfate	-6.1694
202825-46-5	Safinamide mesylate	-6.169
120410-24-4	Biapenem	-6.1687
65666-07-1	Silymarin	-6.1687
1269440-17-6	Encorafenib	-6.1667
132203-70-4	Cilnidipine	-6.1666
24280-93-1	Mycophenolic acid	-6.1662
147526-32-7	Pitavastatin calcium	-6.166
3963-95-9	Methacycline hydrochloride	-6.1658
73590-58-6	Omeprazole	-6.1653
157283-68-6	Travoprost	-6.1651
56-69-9	5-hydroxytryptophan	-6.165
287714-41-4	Rosuvastatin	-6.1649
198904-31-3	Atazanavir	-6.1641
229975-97-7	Atazanavir sulfate	-6.1641
141-94-6	Hexetidine	-6.1637
51-52-5	Propylthiouracil	-6.1633
850876-88-9	Danoprevir	-6.1632
119637-67-1	Moguisteine	-6.1628
6402-23-9	Ethacridine lactate monohydrate	-6.1623
6556-11-2	Inositol nicotinate	-6.1618
313-06-4	Depofemin	-6.1613
146-56-5	Fluphenazine hydrochloride	-6.161
148849-67-6	Ivabradine hydrochloride	-6.1599
25775-90-0	Zucapsaicin	-6.1596
130-16-5	Dermofongin	-6.1591
23210-58-4	Ifenprodil Tartrate	-6.1585
91-33-8	Benzthiazide	-6.158
1837-57-6	Ethacridine lactate	-6.1578
52645-53-1	Permethrin	-6.1569
59263-76-2	Meptazinol hydrochloride	-6.1566
157810-81-6	Indinavir sulfate	-6.1565
34161-24-5	Fipexide	-6.1555
69-81-8	Carbazochrome	-6.155
24168-96-5	Isoconazole nitrate	-6.1545
1405-10-3	Neomycin sulfate	-6.1536
298-46-4	Carbamazepine	-6.1528
4146-30-9	Neomycin Sulphate B	-6.152

111406-87-2	Zileuton	-6.152
99592-39-9	Sertaconazole nitrate	-6.1516
4852-22-6	Proanthocyanidins	-6.1514
132539-06-1	Olanzapine	-6.151
697761-98-1	Elvitegravir	-6.151
4360-12-7	Ajmaline	-6.1508
878143-33-0	Dalasetron Mesylate Hydrate	-6.1507
65513-72-6	Glafenine Hydrochloride	-6.1492
3820-67-5	Glafenine	-6.1492
50-34-0	Propantheline bromide	-6.1491
13636-18-5	Fendiline hydrochloride	-6.1489
91503-79-6	Flurbiprofen Axetil	-6.1489
164579-32-2	Pantoprazole Sodium Hydrate	-6.1485
54-30-8	Camylofin	-6.1484
54-30-8	Camylofin	-6.1476
456-59-7	Cyclandelate	-6.1475
1616493-44-7	Tucidinostat	-6.1471
7085-55-4	Troxerutin	-6.1465
487-41-2	Forsythin	-6.1461
169590-42-5	Celecoxib	-6.1459
123524-52-7	Azelnidipine	-6.1459
440-17-5	Trifluoperazine dihydrochloride	-6.1457
60-93-5	Quinine dihydrochloride	-6.145
130-95-0	Quinine	-6.145
13614-98-7	Minocycline hydrochloride	-6.1444
79307-93-0	Azelastine hydrochloride	-6.144
89796-99-6	Aceclofenac	-6.1431
4852-22-6	Proanthocyanidins	-6.1428
157283-68-6	Travoprost	-6.1427
23541-50-6	Daunorubicin hydrochloride	-6.1425
178307-42-1	Revaprazan hydrochloride	-6.142
137281-23-3	Pemetrexed acid	-6.1418
524-81-2	Mebhydroline	-6.1418
3810-74-0	Streptomycin sulfate	-6.1415
6153-33-9	Mebhydrolin napadisylate	-6.1412
17321-77-6	Clomipramine hydrochloride	-6.1392
25913-34-2	Tinoridine hydrochloride	-6.1392
856866-72-3	Torezolid	-6.1389
29218-27-7	MD 69276	-6.1386
57381-26-7	Irsogladine	-6.1385
57381-26-7	Irsogladine	-6.1378
115956-12-2	Dolasetron	-6.1378

150399-23-8	Pemetrexed disodium	-6.1365
42540-40-9	Cefamandole nafate	-6.1363
99011-02-6	Imiquimod	-6.136
81-27-6	Sennoside A	-6.1355
14984-68-0	Cloperastine hydrochloride	-6.1355
38304-91-5	Minoxidil	-6.1349
155270-99-8	Istradefylline	-6.1349
111902-57-9	Temocapil	-6.1349
157810-81-6	Indinavir sulfate	-6.1343
747-36-4	Hydroxychloroquine sulfate	-6.1343
357166-30-4	Pemetrexed Disodium Hydrate	-6.1338
1210344-57-2	Ertugliflozin	-6.1334
108612-45-9	Mizolastine	-6.1327
520-36-5	Apigenin	-6.1324
29908-03-0	Ademetionine	-6.1323
157810-81-6	Indinavir sulfate	-6.1321
90357-06-5	Bicalutamide	-6.1319
108612-45-9	Mizolastine	-6.1315
1297538-32-9	Darolutamide	-6.1312
202825-46-5	Safinamide mesylate	-6.1309
3424-98-4	Telbivudine	-6.1306
157810-81-6	Indinavir sulfate	-6.1296
1617-90-9	Vincamine	-6.1287
6024-85-7	Tetrahydropalmatine hydrochloride	-6.1286
4852-22-6	Proanthocyanidins	-6.1285
108929-04-0	Epinastine HCl	-6.1285
1025504-45-3	Valbenazine	-6.1284
123447-62-1	Prulifloxacin	-6.1278
832720-36-2	Elagolix sodium	-6.1275
960203-27-4	Vortioxetine HBr	-6.127
508233-74-7	Vortioxetine	-6.127
122320-73-4	Rosiglitazone	-6.1267
906093-29-6	Teneligliptin hydrobromide	-6.1256
18085-97-7	Jaceosidin	-6.1254
157810-81-6	Indinavir sulfate	-6.125
111902-57-9	Temocapil	-6.1244
3564-73-6	10,11-Dihydrocarbamazepine	-6.1232
747-36-4	Hydroxychloroquine sulfate	-6.1232
91-33-8	Benzthiazide	-6.1227
633-65-8	Berberine hydrochloride	-6.1227

274901-16-5	Vildagliptin	-6.1226
151767-02-1	Montelukast sodium	-6.1223
73590-85-9	Omeprazole sulfide	-6.1221
65666-07-1	Silymarin	-6.1218
157810-81-6	Indinavir sulfate	-6.1214
14984-68-0	Cloperastine hydrochloride	-6.1206
152-95-4	Sophoricoside	-6.1198
30123-17-2	Tianeptine sodium	-6.1197
24729-96-2	Clindamycin phosphate	-6.1197
6402-23-9	Ethacridine lactate monohydrate	-6.1184
2753-45-9	Mebeverine hydrochloride	-6.1182
2062-78-4	Pimozide	-6.1178
6469-93-8	Chlorprothixene hydrochloride	-6.1177
113-59-7	Chlorprothixene	-6.1177
1405-10-3	Neomycin sulfate	-6.1173
23210-58-4	Ifenprodil Tartrate	-6.117
521-78-8	Trimipramine maleate	-6.117
157283-68-6	Travoprost	-6.1167
52645-53-1	Permethrin	-6.1166
394730-60-0	Boceprevir	-6.1159
85650-56-2	Asenapine Maleate	-6.1159
60925-61-3	Ceforanide	-6.1146
152-43-2	Quinestrol	-6.1126
4146-30-9	Neomycin Sulphate B	-6.1124
120011-70-3	Donepezil HCl	-6.1124
86541-74-4	Benazepril hydrochloride	-6.1123
16320-04-0	Gestrinone	-6.1119
181183-52-8	Almotriptan Malate	-6.1118
84-02-6	Prochlorperazine Maleate	-6.1116
120014-06-4	Donepezil	-6.1112
808118-40-3	Roxadustat	-6.111
32222-06-3	Calcitriol	-6.1109
1837-57-6	Ethacridine lactate	-6.1106
120011-70-3	Donepezil HCl	-6.1106
66898-62-2	Talniflumate	-6.1096
22071-15-4	Ketoprofen	-6.1094
54063-53-5	Propafenone	-6.1094
115256-11-6	Dofetilide	-6.1093
1405-10-3	Neomycin sulfate	-6.1092
84371-65-3	Mifepristone	-6.1087
37693-01-9	Clofoctol	-6.1087
633-66-9	Berberine hydrogen sulphate	-6.1086
2086-83-1	Berberine	-6.1086

120014-06-4	Donepezil	-6.1085
111902-57-9	Temocapil	-6.1078
4146-30-9	Neomycin Sulphate B	-6.1068
461432-26-8	Dapagliflozin	-6.1067
173334-58-2	Aliskiren hemifumarate	-6.1064
161814-49-9	Amprenavir	-6.1062
55268-74-1	Praziquantel	-6.1058
149709-62-6	AHU377	-6.1056
1369773-39-6	AHU377 calcium salt	-6.1056
960404-48-2	Dapagliflozin ((2S)-1,2-propanediol, hydrate)	-6.1046
29767-20-2	Teniposide	-6.1046
22494-42-4	Diflunisal	-6.1044
440-17-5	Trifluoperazine dihydrochloride	-6.1042
112665-43-7	Seratrodast	-6.104
42924-53-8	Nabumetone	-6.1038
3963-95-9	Methacycline hydrochloride	-6.1037
1811-28-5	Proflavine Hemisulfate	-6.1029
157810-81-6	Indinavir sulfate	-6.1027
173334-58-2	Aliskiren hemifumarate	-6.1025
313-06-4	Depofemin	-6.1024
1025504-45-3	Valbenazine	-6.1023
27262-48-2	Levobupivacaine HCl	-6.1022
27262-47-1	Levobupivacaine	-6.1022
18010-40-7	Bupivacaine hydrochloride	-6.1022
157810-81-6	Indinavir sulfate	-6.1019
72496-41-4	Pirarubicin	-6.1019
157810-81-6	Indinavir sulfate	-6.1013
633-65-8	Berberine hydrochloride	-6.1003
1297538-32-9	Darolutamide	-6.1001
111025-46-8	Pioglitazone	-6.1
3810-74-0	Streptomycin sulfate	-6.0998
610309-89-2	Carvedilol phosphate	-6.0998
152-43-2	Quinestrol	-6.0993
49763-96-4	Stiripentol	-6.0982
132866-11-6	Lercanidipine hydrochloride	-6.0979
479-92-5	Propyphenazone (4-Isopropylantipyrine)	-6.0979
51773-92-3	Mefloquine hydrochloride	-6.0977
130693-82-2	Dorzolamide hydrochloride	-6.0975
64439-81-2	Hydroxy Camptothecine	-6.0972
88671-89-0	Myclobutanil	-6.0971

100427-26-7	Lercanidipine	-6.097
67656-30-8	10-Hydroxycampothecin	-6.0969
99592-39-9	Sertaconazole nitrate	-6.0964
72956-09-3	Carvedilol	-6.0962
29218-27-7	MD 69276	-6.096
388082-77-7	Lapatinib Ditosylate	-6.0958
231277-92-2	Lapatinib	-6.0958
52549-17-4	Pranoprofen	-6.0945
56839-43-1	Eperisone hydrochloride	-6.0942
1616493-44-7	Tucidinostat	-6.0942
157810-81-6	Indinavir sulfate	-6.094
187164-19-8	Luliconazole	-6.0937
6202-23-9	Cyclobenzaprine hydrochloride	-6.0926
946075-13-4	Isavuconazonium sulfate	-6.0924
50-63-5	Chloroquine diphosphate	-6.0923
30123-17-2	Tianeptine sodium	-6.0916
485-49-4	(+)-Bicuculline	-6.091
73-48-3	Bendroflumethiazide	-6.0909
4350-09-8	L-5-Hydroxytryptophan	-6.0908
6398-98-7	Amodiaquine hydrochloride	-6.0905
388082-78-8	Lapatinib ditosylate monohydrate	-6.0901
1051375-16-6	Dolutegravir	-6.09
14605-22-2	Tauroursodeoxycholic Acid	-6.0895
485-49-4	(+)-Bicuculline	-6.0894
518-28-5	Podophyllotoxin	-6.0893
4852-22-6	Proanthocyanidins	-6.0893
122111-03-9	Gemcitabine HCl	-6.0878
95058-81-4	Gemcitabine	-6.0878
90357-06-5	Bicalutamide	-6.0878
219989-84-1	Ixabepilone	-6.0878
53797-35-6	Ribostamycin sulfate	-6.0877
68786-66-3	Triclabendazole	-6.0875
65666-07-1	Silymarin	-6.0873
121032-29-9	Nelarabine	-6.087
6469-93-8	Chlorprothixene hydrochloride	-6.0866
113-59-7	Chlorprothixene	-6.0866
122647-32-9	Ibutilide Fumarate	-6.0866
13311-84-7	Flutamide	-6.0864
51-41-2	Norepinephrine	-6.0863
69815-49-2	Noradrenaline bitartrate	-6.0863
91503-79-6	Flurbiprofen Axetil	-6.0861

59263-76-2	Meptazinol hydrochloride	-6.0857
960203-27-4	Vortioxetine HBr	-6.0857
508233-74-7	Vortioxetine	-6.0857
52645-53-1	Permethrin	-6.0852
912-60-7	Noscapine hydrochloride	-6.0851
402957-28-2	Telaprevir	-6.0843
165800-03-3	Linezolid	-6.0842
24280-93-1	Mycophenolic acid	-6.0835
79794-75-5	Loratadine	-6.0822
23672-07-3	Levosulpiride	-6.0815
2753-45-9	Mebeverine hydrochloride	-6.0813
83881-52-1	Cetirizine dihydrochloride	-6.0807
15676-16-1	Sulpiride	-6.0803
119637-67-1	Moguisteine	-6.0793
79307-93-0	Azelastine hydrochloride	-6.079
51803-78-2	Nimesulide	-6.0787
99592-39-9	Sertaconazole nitrate	-6.0786
14919-77-8	Benserazide hydrochloride	-6.0786
178307-42-1	Revaprazan hydrochloride	-6.0779
53251-94-8	Pinaverium bromide	-6.0776
633-66-9	Berberine hydrogen sulphate	-6.0776
2086-83-1	Berberine	-6.0776
956104-40-8	Apalutamide	-6.0774
456-59-7	Cyclandelate	-6.0773
22881-35-2	Famprofazone	-6.0773
4852-22-6	Proanthocyanidins	-6.0765
53885-35-1	Ticlopidine hydrochloride	-6.0764
73-22-3	L-Tryptophan	-6.076
132866-11-6	Lercanidipine hydrochloride	-6.0753
603-50-9	Bisacodyl	-6.0753
157810-81-6	Indinavir sulfate	-6.075
50-14-6	Vitamin D2	-6.0749
1354744-91-4	Naloxegol oxalate	-6.0748
157810-81-6	Indinavir sulfate	-6.0743
479-92-5	Propyphenazone (4-Isopropylantipyrine)	-6.0742
79307-93-0	Azelastine hydrochloride	-6.0741
1025504-45-3	Valbenazine	-6.0739
521-78-8	Trimipramine maleate	-6.0738
122320-73-4	Rosiglitazone	-6.0736
30544-47-9	Etofenamate	-6.0731
3810-74-0	Streptomycin sulfate	-6.0726

171596-29-5	Tadalafil	-6.0722
4991-65-5	Tioxolone	-6.072
29908-03-0	Ademetionine	-6.0718
157810-81-6	Indinavir sulfate	-6.0717
610798-31-7	Icotinib	-6.0716
59-05-2	Methotrexate	-6.071
145202-66-0	Rizatriptan benzoate	-6.0708
100427-26-7	Lercanidipine	-6.0705
90-33-5	4-Methylumbelliferone	-6.0704
157810-81-6	Indinavir sulfate	-6.0701
22881-35-2	Famprofazone	-6.0699
608141-41-9	Apremilast	-6.0699
111902-57-9	Temocapil	-6.0695
117976-90-6	Rebeprazole sodium	-6.0694
486-35-1	Daphnetin	-6.0693
29908-03-0	Ademetionine	-6.0689
157283-68-6	Travoprost	-6.0689
88-04-0	Chloroxylenol	-6.0687
15676-16-1	Sulpiride	-6.0675
363-24-6	Prostaglandin E2 (PGE2)	-6.0675
88889-14-9	Fosinopril sodium	-6.0669
23672-07-3	Levosulpiride	-6.0666
13614-98-7	Minocycline hydrochloride	-6.0654
878672-00-5	Lesinurad	-6.0651
364622-82-2	Doripenem Hydrate	-6.0648
101477-54-7	Lomerizine hydrochloride	-6.0648
60628-96-8	Bifonazole	-6.0647
147536-97-8	Bosentan	-6.0644
1038915-60-4	Niraparib	-6.0626
81-27-6	Sennoside A	-6.0626
147127-20-6	Tenofovir	-6.0619
34161-24-5	Fipexide	-6.0617
138112-76-2	Agomelatine	-6.0613
568-73-0	Tanshinone I	-6.0612
1038915-73-9	Niraparib tosylate	-6.0608
32222-06-3	Calcitriol	-6.0602
111902-57-9	Temocapil	-6.0602
4852-22-6	Proanthocyanidins	-6.0601
91296-87-6	Sarafloxacin hydrochloride	-6.0595
58-15-1	Aminophenazone	-6.0594
73816-42-9	Meclocycline sulfosalicylate salt	-6.0593

827022-33-3	Palbociclib Isethionate	-6.0593
827022-32-2	Palbociclib hydrochloride	-6.0593
698387-09-6	Neratinib	-6.0591
99291-25-5	Levodropropizine	-6.0589
60628-96-8	Bifonazole	-6.0588
88-04-0	Chloroxylenol	-6.0584
56296-78-7	Fluoxetine hydrochloride	-6.0582
54910-89-3	Fluoxetine	-6.0582
941685-37-6	S-Ruxolitinib	-6.0582
571190-30-2	Palbociclib	-6.0578
530-78-9	Flufenamic acid	-6.0576
89226-50-6	Manidipine	-6.0572
89226-75-5	Manidipine dihydrochloride	-6.0572
117976-90-6	Rebeprazole sodium	-6.057
53251-94-8	Pinaverium bromide	-6.0569
169590-42-5	Celecoxib	-6.0563
157810-81-6	Indinavir sulfate	-6.0561
31282-04-9	Hygromycin B	-6.0559
148849-67-6	Ivabradine hydrochloride	-6.0549
147536-97-8	Bosentan	-6.0543
56296-78-7	Fluoxetine hydrochloride	-6.0539
54910-89-3	Fluoxetine	-6.0539
55268-74-1	Praziquantel	-6.0538
61718-82-9	Fluvoxamine maleate	-6.0533
14984-68-0	Cloperastine hydrochloride	-6.0531
74050-98-9	Ketanserin	-6.0529
87233-61-2	Emedastine	-6.0522
456-59-7	Cyclandelate	-6.0517
151767-02-1	Montelukast sodium	-6.0504
111025-46-8	Pioglitazone	-6.05
91161-71-6	Terbinafine	-6.0498
491833-29-5	Eliglustat	-6.0494
917389-32-3	Letermovir	-6.0488
53251-94-8	Pinaverium bromide	-6.0487
118288-08-7	Lafutidine	-6.0481
219989-84-1	Ixabepilone	-6.048
405165-61-9	Besifloxacin HCl	-6.0479
133099-07-7	Darifenacin HBr	-6.0479
184475-35-2	Gefitinib	-6.0478
91161-71-6	Terbinafine	-6.0477
59729-32-7	Citalopram HBr	-6.0476
24280-93-1	Mycophenolic acid	-6.0474
59263-76-2	Meptazinol hydrochloride	-6.0466

29908-03-0	Ademetionine	-6.0454
1229208-44-9	Entospletinib	-6.0446
1263-89-4	Paromomycin Sulfate	-6.0446
157810-81-6	Indinavir sulfate	-6.0444
66309-69-1	Cefotiam hydrochloride	-6.0442
87233-61-2	Emedastine	-6.0439
1084-65-7	Meticrane	-6.0437
1098-97-1	Pyrithioxin	-6.043
832720-36-2	Elagolix sodium	-6.0429
167221-71-8	Clevidipine butyrate	-6.0422
123524-52-7	Azelnidipine	-6.0421
4852-22-6	Proanthocyanidins	-6.0419
115956-12-2	Dolasetron	-6.0418
111902-57-9	Temocapil	-6.0417
107007-99-8	Granisetron hydrochloride	-6.0401
126544-47-6	Ciclesonide	-6.0401
15318-45-3	Thiamphenicol	-6.04
2413-38-9	Fupentixol Dihydrochloride	-6.0395
27833-64-3	Loxapine succinate salt	-6.0394
51022-69-6	Amcinonide	-6.0392
330784-47-9	Avanafil	-6.039
241479-67-4	Isavuconazole	-6.0389
90729-43-4	Ebastine	-6.0389
157810-81-6	Indinavir sulfate	-6.0386
76095-16-4	Enalapril Maleate	-6.0371
190786-44-8	Bepotastine Besilate	-6.0364
76095-16-4	Enalapril Maleate	-6.0361
30123-17-2	Tianeptine sodium	-6.0357
64872-77-1	Butoconazole nitrate	-6.0357
152-95-4	Sophoricoside	-6.0352
167465-36-3	Zosuquidar 3HCl	-6.0352
29908-03-0	Ademetionine	-6.0351
1263-89-4	Paromomycin Sulfate	-6.0346
157810-81-6	Indinavir sulfate	-6.0346
178307-42-1	Revaprazan hydrochloride	-6.0341
86541-74-4	Benazepril hydrochloride	-6.0339
108929-04-0	Epinastine HCl	-6.0333
132539-06-1	Olanzapine	-6.0333
832720-36-2	Elagolix sodium	-6.0331
22494-42-4	Diflunisal	-6.0325
10083-24-6	Piceatannol	-6.0325
91503-79-6	Flurbiprofen Axetil	-6.0322

164579-32-2	Pantoprazole Sodium Hydrate	-6.032
1025504-45-3	Valbenazine	-6.032
88671-89-0	Myclobutanil	-6.0317
50-34-0	Propantheline bromide	-6.0313
4852-22-6	Proanthocyanidins	-6.0312
147127-20-6	Tenofovir	-6.0311
151533-22-1	Levomefolate calcium	-6.0311
55268-74-1	Praziquantel	-6.0308
706782-28-7	Pimavanserin tartrate	-6.0303
706779-91-1	Pimavanserin	-6.0303
56-69-9	5-hydroxytryptophan	-6.03
4852-22-6	Proanthocyanidins	-6.0297
157810-81-6	Indinavir sulfate	-6.0293
58-27-5	Menadione	-6.029
157810-81-6	Indinavir sulfate	-6.0288
15318-45-3	Thiamphenicol	-6.0287
73816-42-9	Meclocycline sulfosalicylate salt	-6.0287
863-61-6	Menaquinone-4	-6.0273
11032-49-8	Menatetrenone	-6.0273
24168-96-5	Isoconazole nitrate	-6.0271
520-36-5	Apigenin	-6.0268
845614-11-1	Bitopertin	-6.026
486-35-1	Daphnetin	-6.0253
3717-88-2	Flavoxate hydrochloride	-6.0251
62499-27-8	Gastrodin	-6.0248
120014-06-4	Donepezil	-6.0248
4360-12-7	Ajmaline	-6.0243
443913-73-3	Vandetanib	-6.0242
603139-19-1	Odanacatib	-6.0241
61-12-1	Dibucaine hydrochloride	-6.0237
85-79-0	Dibucaine	-6.0237
550-99-2	Naphazoline hydrochloride	-6.0236
120011-70-3	Donepezil HCl	-6.0233
65513-72-6	Glafenine Hydrochloride	-6.0232
3820-67-5	Glafenine	-6.0232
10347-81-6	Maprotiline hydrochloride	-6.0231
81110-73-8	Racecadotril	-6.0219
23672-07-3	Levosulpiride	-6.0218
15676-16-1	Sulpiride	-6.0218
537-12-2	Diperodon hydrochloride	-6.0218
179474-81-8	Prucalopride	-6.0217
22071-15-4	Ketoprofen	-6.0216

1617-90-9	Vincamine	-6.0215
42924-53-8	Nabumetone	-6.0214
81919-14-4	Bendazac L-lysine	-6.0211
134678-17-4	Lamivudine	-6.0203
150915-41-6	Perospirone	-6.0202
1446321-46-5	voxelotor	-6.0201
16595-80-5	Levamisole hydrochloride	-6.02
209216-23-9	Entecavir hydrate	-6.0199
1405-20-5	Polymyxin B sulfate	-6.0199
15676-16-1	Sulpiride	-6.0198
1201902-80-8	MLN9708	-6.0192
142217-69-4	Entecavir	-6.0181
69975-86-6	Doxofylline	-6.0172
157810-81-6	Indinavir sulfate	-6.0171
21679-14-1	Fludarabine	-6.0149
56296-78-7	Fluoxetine hydrochloride	-6.0149
54910-89-3	Fluoxetine	-6.0149
13614-98-7	Minocycline hydrochloride	-6.0148
101827-46-7	Butenafine hydrochloride	-6.0146
839712-12-8	Cariprazine	-6.0138
5490-27-7	Dihydrostreptomycin sulfate	-6.0129
157810-81-6	Indinavir sulfate	-6.0117
53885-35-1	Ticlopidine hydrochloride	-6.0114
89226-75-5	Manidipine dihydrochloride	-6.0112
68786-66-3	Triclabendazole	-6.0107
201341-05-1	Tenofovir Disoproxil	-6.0089
52645-53-1	Permethrin	-6.0082
157810-81-6	Indinavir sulfate	-6.008
64872-77-1	Butoconazole nitrate	-6.008
16320-04-0	Gestrinone	-6.0078
89226-50-6	Manidipine	-6.0076
1297538-32-9	Darolutamide	-6.0068
23541-50-6	Daunorubicin hydrochloride	-6.0068
88150-42-9	Amlodipine	-6.0055
82586-55-8	Quinapril hydrochloride	-6.0053
61618-27-7	Amfenac Sodium Monohydrate	-6.0049
388082-78-8	Lapatinib ditosylate monohydrate	-6.0047
1360460-82-7	Anlotinib Dihydrochloride	-6.0041
1058156-90-3	Anlotinib	-6.0041

67227-57-0	Fenoldopam mesylate	-6.004
23672-07-3	Levosulpiride	-6.0038
220991-20-8	Lumiracoxib	-6.0036
161814-49-9	Amprenavir	-6.0034
15676-16-1	Sulpiride	-6.0032
157810-81-6	Indinavir sulfate	-6.0031
22457-89-2	Benfotiamine	-6.0028
912-60-7	Noscapine hydrochloride	-6.0025
706782-28-7	Pimavanserin tartrate	-6.0025
706779-91-1	Pimavanserin	-6.0025
120-08-1	Scoparone	-6.0023
1201902-80-8	MLN9708	-6.002
107007-99-8	Granisetron hydrochloride	-6.0009
388082-77-7	Lapatinib Ditosylate	-6.0005
231277-92-2	Lapatinib	-6.0005
10347-81-6	Maprotiline hydrochloride	-6.0002
202189-78-4	Bilastine	-5.9995
80-35-3	Sulfamethoxypyridazine	-5.9993
67227-57-0	Fenoldopam mesylate	-5.9991
320-67-2	5-Azacytidine	-5.9986
19428-14-9	Benproperine phosphate	-5.9983
487-41-2	Forsythin	-5.9982
54527-84-3	Nicardipine hydrochloride	-5.9976
30516-87-1	Zidovudine	-5.997
111902-57-9	Temocapil	-5.9962
80-49-9	Homatropine Methylbromide	-5.9961
2192-20-3	Hydroxyzine dihydrochloride	-5.9953
10246-75-0	Hydroxyzine Pamoate	-5.9947
93793-83-0	Roxatidine Acetate hydrochloride	-5.9938
91296-87-6	Sarafloxacin hydrochloride	-5.9936
157810-81-6	Indinavir sulfate	-5.9936
496775-61-2	Eltrombopag	-5.9926
73590-85-9	Omeprazole sulfide	-5.9926
5534-95-2	Pentagastrin	-5.9915
123524-52-7	Azelnidipine	-5.9912
394730-60-0	Boceprevir	-5.991
87233-61-2	Emedastine	-5.9904
960203-27-4	Vortioxetine HBr	-5.9897
51-52-5	Propylthiouracil	-5.9894
508233-74-7	Vortioxetine	-5.9891
477600-75-2	Tofacitinib	-5.9889
22881-35-2	Famprofazone	-5.9887

540737-29-9	Tofacitinib Citrate	-5.9886
946075-13-4	Isavuconazonium sulfate	-5.9883
877399-52-5	Crizotinib	-5.9874
1051375-19-9	Dolutegravir sodium	-5.9873
157810-81-6	Indinavir sulfate	-5.9873
491-67-8	Baicalein	-5.9871
67227-57-0	Fenoldopam mesylate	-5.987
236395-14-5	Eslicarbazepine Acetate	-5.9862
132866-11-6	Lercanidipine hydrochloride	-5.986
100427-26-7	Lercanidipine	-5.9854
73590-85-9	Omeprazole sulfide	-5.9853
2468-21-5	Catharanthine	-5.9852
120014-06-4	Donepezil	-5.985
152-95-4	Sophoricoside	-5.9849
157810-81-6	Indinavir sulfate	-5.9849
120011-70-3	Donepezil HCl	-5.9847
112809-51-5	Letrozole	-5.9846
1289023-67-1	BMS927711	-5.9844
157810-81-6	Indinavir sulfate	-5.9842
62-31-7	Dopamine hydrochloride	-5.9839
49763-96-4	Stiripentol	-5.9838
1953-04-4	Galanthamine HBr	-5.9834
479-92-5	Propyphenazone (4-Isopropylantipyrine)	-5.9833
151533-22-1	Levomefolate calcium	-5.983
59729-32-7	Citalopram HBr	-5.9819
65666-07-1	Silymarin	-5.9817
1098-97-1	Pyrithioxin	-5.9812
298-46-4	Carbamazepine	-5.9811
78628-80-5	Terbinafine hydrochloride	-5.9811
72509-76-3	Felodipine	-5.9811
10347-81-6	Maprotiline hydrochloride	-5.9809
24390-14-5	Doxycycline hyclate	-5.9809
918504-65-1	Vemurafenib	-5.9806
313-06-4	Depofemin	-5.9796
1953-04-4	Galanthamine HBr	-5.9795
67227-57-0	Fenoldopam mesylate	-5.9791
120011-70-3	Donepezil HCl	-5.979
42540-40-9	Cefamandole nafate	-5.9783
84057-95-4	Ropivacaine	-5.9781
98717-15-8	Ropivacaine hydrochloride	-5.9781
120014-06-4	Donepezil	-5.9779

120014-06-4	Donepezil	-5.9778
157810-81-6	Indinavir sulfate	-5.9778
148-24-3	Hydroxyquinoline	-5.9777
73590-85-9	Omeprazole sulfide	-5.9775
122647-32-9	Ibutilide Fumarate	-5.9773
3818-50-6	Bephenium Hydroxynaphthoate	-5.9771
123524-52-7	Azelnidipine	-5.9765
170105-16-5	Imidafenacin	-5.976
843663-66-1	Bedaquiline	-5.976
67-03-8	Thiamine hydrochloride	-5.9756
1225-55-4	Protriptyline hydrochloride	-5.9754
120011-70-3	Donepezil HCl	-5.9752
150683-30-0	Tolvaptan	-5.975
29342-05-0	Ciclopirox	-5.9737
41621-49-2	Ciclopirox ethanolamine	-5.9737
1722-62-9	Mepivacaine hydrochloride	-5.9732
16320-04-0	Gestrinone	-5.9729
59263-76-2	Meptazinol hydrochloride	-5.9723
6138-79-0	Triprolidine hydrochloride	-5.972
3978-86-7	Azatadine Maleate	-5.9719
81938-43-4	Zofenopril (calcium)	-5.9713
137-88-2	Amprolium Hydrochloride	-5.9711
24390-14-5	Doxycycline hyclate	-5.9709
456-59-7	Cyclandelate	-5.9709
3963-95-9	Methacycline hydrochloride	-5.9706
196597-26-9	Ramelteon	-5.9702
220991-20-8	Lumiracoxib	-5.9696
22832-87-7	Miconazole nitrate	-5.968
145202-66-0	Rizatriptan benzoate	-5.9679
111902-57-9	Temocapil	-5.9675
3094-09-5	Doxifluridine	-5.9674
878143-33-0	Dalasetron Mesylate Hydrate	-5.9671
58-27-5	Menadione	-5.9668
485-49-4	(+)-Bicuculline	-5.9667
157810-81-6	Indinavir sulfate	-5.966
58186-27-9	Idebenone	-5.966
298-81-7	8-Methoxypsoralen	-5.9659
56-69-9	5-hydroxytryptophan	-5.9658
10402-53-6	Eprazinone dihydrochloride	-5.9652
111902-57-9	Temocapil	-5.965
22881-35-2	Famprofazone	-5.9647
475-67-2	S-Isocorydine(+)	-5.9646
6556-11-2	Inositol nicotinate	-5.9644

1374356-45-2	(S)-crizotinib	-5.9642
18085-97-7	Jaceosidin	-5.9639
83-07-8	4-Aminoantipyrine	-5.9633
606-04-2	Pamabrom	-5.963
110221-44-8	Temocapril HCl	-5.9626
1025504-45-3	Valbenazine	-5.9624
157810-81-6	Indinavir sulfate	-5.9624
29218-27-7	MD 69276	-5.9617
320-67-2	5-Azacytidine	-5.9612
78628-80-5	Terbinafine hydrochloride	-5.9612
864750-70-9	Revefenacin	-5.9606
113-92-8	Chlorpheniramine maleate	-5.9604
122647-32-9	Ibutilide Fumarate	-5.9601
491-67-8	Baicalein	-5.96
89396-94-1	Imidapril HCl	-5.9596
90729-43-4	Ebastine	-5.9588
54527-84-3	Nicardipine hydrochloride	-5.9587
92-61-5	Scopoletin	-5.9584
475-67-2	S-Isocorydine(+)	-5.9584
111902-57-9	Temocapil	-5.9577
1229208-44-9	Entospletinib	-5.957
157810-81-6	Indinavir sulfate	-5.9568
148849-67-6	Ivabradine hydrochloride	-5.9566
106730-54-5	Olprinone	-5.9564
27848-84-6	Nicergoline	-5.9558
161973-10-0	Esomeprazole Magnesium	-5.9554
61318-91-0	Sulconazole Nitrate	-5.9553
69975-86-6	Doxofylline	-5.9549
163252-36-6	Clevudine	-5.9546
111902-57-9	Temocapil	-5.9545
6398-98-7	Amodiaquine hydrochloride	-5.9542
56211-40-6	Torasemide	-5.9537
219989-84-1	Ixabepilone	-5.9536
21679-14-1	Fludarabine	-5.9527
101477-54-7	Lomerizine hydrochloride	-5.9527
23672-07-3	Levosulpiride	-5.9521
15676-16-1	Sulpiride	-5.9521
538-71-6	Domiphen Bromide	-5.9521
143491-57-0	Emtricitabine	-5.9517
120-97-8	Dichlorphenamide	-5.9515

298-81-7	8-Methoxypsoralen	-5.9514
88889-14-9	Fosinopril sodium	-5.9513
76095-16-4	Enalapril Maleate	-5.9511
112665-43-7	Seratrodast	-5.9501
943-17-9	Etilefrine hydrochloride	-5.9494
164579-32-2	Pantoprazole Sodium Hydrate	-5.9491
141-94-6	Hexetidine	-5.949
3978-86-7	Azatadine Maleate	-5.9486
59263-76-2	Meptazinol hydrochloride	-5.9486
172152-50-0	Ilaprazole sodium	-5.9484
1104-22-9	Meclizine dihydrochloride	-5.9475
1098-97-1	Pyrithioxin	-5.9471
74863-84-6	Argatroban	-5.9471
133099-07-7	Darifenacin HBr	-5.9469
73590-58-6	Omeprazole	-5.9464
30123-17-2	Tianeptine sodium	-5.9458
137-58-6	Xylocaine	-5.9456
241479-67-4	Isavuconazole	-5.9453
113-92-8	Chlorpheniramine maleate	-5.9453
6108-05-0	Lidocaine Hydrochloride hydrate	-5.945
73-78-9	Lidocaine hydrochloride	-5.945
4991-65-5	Tioxolone	-5.945
856866-72-3	Torezolid	-5.945
475-67-2	S-Isocorydine(+)	-5.9448
89226-50-6	Manidipine	-5.9447
2753-45-9	Mebeverine hydrochloride	-5.9443
89226-75-5	Manidipine dihydrochloride	-5.9441
148-24-3	Hydroxyquinoline	-5.9438
129453-61-8	Fulvestrant	-5.9435
78281-72-8	Nepafenac	-5.9424
912-60-7	Noscapine hydrochloride	-5.9424
88-04-0	Chloroxylenol	-5.9419
745-65-3	Alprostadil	-5.9418
65666-07-1	Silymarin	-5.9418
146-56-5	Fluphenazine hydrochloride	-5.9417
1263-89-4	Paromomycin Sulfate	-5.9406
157810-81-6	Indinavir sulfate	-5.9402
81-27-6	Sennoside A	-5.9401
1263-89-4	Paromomycin Sulfate	-5.9396
29908-03-0	Ademetionine	-5.9389
93793-83-0	Roxatidine Acetate hydrochloride	-5.9378
4991-65-5	Tioxolone	-5.9377
87233-61-2	Emedastine	-5.9377

132203-70-4	Cilnidipine	-5.9372
11013-97-1	Methyl hesperidin	-5.9367
1405-10-3	Neomycin sulfate	-5.936
3963-95-9	Methacycline hydrochloride	-5.9351
747-36-4	Hydroxychloroquine sulfate	-5.9342
181183-52-8	Almotriptan Malate	-5.9338
88671-89-0	Myclobutanil	-5.9334
105826-92-4	Tropisetron hydrochloride	-5.9334
112885-42-4	Mosapride Citrate	-5.9329
34161-24-5	Fipexide	-5.9323
1025504-45-3	Valbenazine	-5.9322
108612-45-9	Mizolastine	-5.9312
1152311-62-0	VX661	-5.931
1225-55-4	Protriptyline hydrochloride	-5.9307
4429-63-4	Tabersonine	-5.9304
603-50-9	Bisacodyl	-5.9303
496775-62-3	Eltrombopag Olamine	-5.9299
4146-30-9	Neomycin Sulphate B	-5.9289
24168-96-5	Isoconazole nitrate	-5.9286
1163-36-6	Clemizole hydrochloride	-5.9285
7759-35-5	Nestoron	-5.9284
157810-81-6	Indinavir sulfate	-5.9283
60628-96-8	Bifonazole	-5.9278
161715-24-8	Tebipenem Pivoxil	-5.9277
111406-87-2	Zileuton	-5.9274
1051375-16-6	Dolutegravir	-5.9273
1195765-45-7	Dabrafenib	-5.9273
4146-30-9	Neomycin Sulphate B	-5.9272
157810-81-6	Indinavir sulfate	-5.927
456-59-7	Cyclandelate	-5.9269
78628-80-5	Terbinafine hydrochloride	-5.9268
330784-47-9	Avanafil	-5.9266
88150-42-9	Amlodipine	-5.9262
56839-43-1	Eperisone hydrochloride	-5.9242
132036-88-5	Ramosetron	-5.9238
177834-92-3	Eletriptan HBr	-5.9237
51022-69-6	Amcinonide	-5.9235
1405-10-3	Neomycin sulfate	-5.9234
104227-87-4	Famciclovir	-5.9232
157810-81-6	Indinavir sulfate	-5.9232

520-36-5	Apigenin	-5.9229
120410-24-4	Biapenem	-5.9226
41340-25-4	Etodolac	-5.9223
22832-87-7	Miconazole nitrate	-5.922
147536-97-8	Bosentan	-5.9219
475-67-2	S-Isocorydine(+)	-5.9218
56839-43-1	Eperisone hydrochloride	-5.9218
19428-14-9	Benproperine phosphate	-5.9217
241479-67-4	Isavuconazole	-5.9216
4350-09-8	L-5-Hydroxytryptophan	-5.9215
157810-81-6	Indinavir sulfate	-5.9214
79-57-2	Oxytetracycline	-5.9213
946075-13-4	Isavuconazonium sulfate	-5.921
14698-29-4	Oxolinic acid	-5.9204
918504-65-1	Vemurafenib	-5.9201
10405-02-4	Trospium chloride	-5.9199
69-81-8	Carbazochrome	-5.9198
61718-82-9	Fluvoxamine maleate	-5.9195
157283-68-6	Travoprost	-5.919
2627-69-2	AICAR (Acadesine)	-5.9188
120-97-8	Dichlorphenamide	-5.918
1084-65-7	Meticrane	-5.9179
52549-17-4	Pranoprofen	-5.9174
75695-93-1	Isradipine	-5.9172
21679-14-1	Fludarabine	-5.9168
608141-41-9	Apremilast	-5.9162
943-17-9	Etilefrine hydrochloride	-5.9157
132866-11-6	Lercanidipine hydrochloride	-5.9151
59263-76-2	Meptazinol hydrochloride	-5.9145
120011-70-3	Donepezil HCl	-5.9143
138452-21-8	Fexofenadine hydrochloride	-5.9142
485-49-4	(+)-Bicuculline	-5.914
68786-66-3	Triclabendazole	-5.9137
120014-06-4	Donepezil	-5.9134
179474-81-8	Prucalopride	-5.9125
54-30-8	Camylofin	-5.9122
15307-79-6	Diclofenac sodium	-5.912
5578-73-4	Sanguinarine chloride	-5.9119
65666-07-1	Silymarin	-5.9109
53910-25-1	Pentostatin	-5.9105
88678-31-3	Liranaftate	-5.9101
138-37-4	Mafenide hydrochloride	-5.9097
4852-22-6	Proanthocyanidins	-5.9094

15307-86-5	Diclofenac	-5.9093
15307-81-0	Diclofenac Potassium	-5.909
78213-16-8	Diclofenac diethylamine	-5.909
145202-66-0	Rizatriptan benzoate	-5.9089
157810-81-6	Indinavir sulfate	-5.9087
15826-37-6	Sodium cromoglycate	-5.9086
123663-49-0	Iguratimod	-5.9085
386750-22-7	Desvenlafaxine succinate hydrate	-5.9082
93413-62-8	Desvenlafaxine	-5.9082
65513-72-6	Glafenine Hydrochloride	-5.9081
3820-67-5	Glafenine	-5.9081
103628-48-4	Sumatriptan succinate	-5.9079
386750-22-7	Desvenlafaxine succinate hydrate	-5.9077
93413-62-8	Desvenlafaxine	-5.9077
58186-27-9	Idebenone	-5.9074
157810-81-6	Indinavir sulfate	-5.9069
84680-54-6	Enalaprilat Dihydrate	-5.9065
157810-81-6	Indinavir sulfate	-5.9061
6138-79-0	Triprolidine hydrochloride	-5.9053
64872-77-1	Butoconazole nitrate	-5.9053
16595-80-5	Levamisole hydrochloride	-5.9043
52152-93-9	Cefsulodine sodium	-5.9032
6066-49-5	Butylphthalide	-5.9029
124937-52-6	Tolterodine tartrate	-5.9029
10238-21-8	Glyburide	-5.9018
3902-71-4	Trioxsalen	-5.9016
150322-43-3	Prasugrel	-5.9015
153773-82-1	Ertapenem sodium	-5.9014
157810-81-6	Indinavir sulfate	-5.9012
95635-56-6	Ranolazine dihydrochloride	-5.9011
63-45-6	Primaquine diphosphate	-5.9009
178307-42-1	Revaprazan hydrochloride	-5.9008
122852-69-1	Alosetron hydrochloride	-5.9004
82248-59-7	Atomoxetine hydrochloride	-5.9004
380843-75-4	Bosutinib	-5.8997
153-98-0	Serotonin hydrochloride	-5.8995
104227-87-4	Famciclovir	-5.899
1195765-45-7	Dabrafenib	-5.8987
189279-58-1	Delafloxacin	-5.8984
181695-72-7	Valdecoxib	-5.8981
95635-55-5	Ranolazine	-5.8981
2413-38-9	Fupentixol Dihydrochloride	-5.8976

118288-08-7	Lafutidine	-5.8975
3978-86-7	Azatadine Maleate	-5.8971
42461-84-7	Flunixin meglumin	-5.8965
171228-49-2	Posaconazole	-5.8965
6138-79-0	Triprolidine hydrochloride	-5.8962
863127-77-9	Dasatinib monohydrate	-5.8958
84057-84-1	Lamotrigine	-5.8949
111025-46-8	Pioglitazone	-5.8949
521-78-8	Trimipramine maleate	-5.8947
132036-88-5	Ramosetron	-5.8946
101-31-5	L-Hyoscyamine	-5.8944
59-05-2	Methotrexate	-5.894
154-69-8	Tripelennamine hydrochloride	-5.8939
29218-27-7	MD 69276	-5.8939
2468-21-5	Catharanthine	-5.8939
3505-38-2	Carbinoxamine Maleate Salt	-5.8937
36791-04-5	Ribavirin	-5.8934
57808-66-9	Domperidone	-5.8928
548-73-2	Droperidol	-5.8927
55-48-1	Atropine sulfate	-5.8926
5908-99-6	Atropine sulfate monohydrate	-5.8926
51-55-8	Atropine	-5.8926
69558-55-0	Thymopentin	-5.8925
51-30-9	Isoprenaline hydrochloride	-5.8922
182431-12-5	Lomitapide	-5.8922
521-78-8	Trimipramine maleate	-5.892
4852-22-6	Proanthocyanidins	-5.892
6968-72-5	Mepiroxol	-5.8915
289480-64-4	Treprostinil Sodium	-5.8911
2062-78-4	Pimozide	-5.891
84625-61-6	Itraconazole	-5.8909
10605-21-7	Carbendazim	-5.8906
206361-99-1	Darunavir	-5.8904
635728-49-3	Darunavir?Ethanolate	-5.8901
157810-81-6	Indinavir sulfate	-5.89
147127-20-6	Tenofovir	-5.8899
697761-98-1	Elvitegravir	-5.8889
10083-24-6	Piceatannol	-5.8889
302962-49-8	Dasatinib	-5.8889
64-75-5	Tetracycline hydrochloride	-5.8887
126544-47-6	Ciclesonide	-5.8882
52-21-1	Prednisolone-21-acetate	-5.8882
52645-53-1	Permethrin	-5.8879

633-66-9	Berberine hydrogen sulphate	-5.8876
2086-83-1	Berberine	-5.8876
980-71-2	Brompheniramine hydrogen maleate	-5.8876
209216-23-9	Entecavir hydrate	-5.8874
63-45-6	Primaquine diphosphate	-5.8874
151533-22-1	Levomefolate calcium	-5.8873
329-63-5	DL-Adrenaline Hydrochloride	-5.8871
142217-69-4	Entecavir	-5.8865
122111-03-9	Gemcitabine HCl	-5.8862
95058-81-4	Gemcitabine	-5.8862
537-12-2	Diperodon hydrochloride	-5.8862
3011-89-0	Alkomide	-5.8862
68786-66-3	Triclabendazole	-5.8854
14976-57-9	Clemastine fumarate	-5.8853
2468-21-5	Catharanthine	-5.8851
69659-80-9	Tanshinone IIA sulfonate sodium	-5.885
3424-98-4	Telbivudine	-5.8849
179474-81-8	Prucalopride	-5.8849
1098-97-1	Pyrithioxin	-5.8843
157810-81-6	Indinavir sulfate	-5.884
10212-25-6	Cyclocytidine hydrochloride	-5.8838
6398-98-7	Amodiaquine hydrochloride	-5.8838
51-30-9	Isoprenaline hydrochloride	-5.8836
83105-70-8	Sultamicillin Tosylate	-5.8834
70797-11-4	Cefpiramide acid	-5.883
2438-32-6	Dexchlorpheniramine Maleate	-5.8826
60-54-8	Tetracycline	-5.8824
1454846-35-5	Lorlatinib	-5.8823
60628-96-8	Bifonazole	-5.8822
747-36-4	Hydroxychloroquine sulfate	-5.8821
83435-67-0	Delapril?Hydrochloride	-5.8813
80-35-3	Sulfamethoxypyridazine	-5.8794
60-54-8	Tetracycline	-5.879
56390-09-1	Epirubicin hydrochloride	-5.8778
2192-20-3	Hydroxyzine dihydrochloride	-5.8775
14976-57-9	Clemastine fumarate	-5.8775
15307-79-6	Diclofenac sodium	-5.8772
56-59-7	Felypressin	-5.8769
10246-75-0	Hydroxyzine Pamoate	-5.8769
150915-41-6	Perospirone	-5.8767
54527-84-3	Nicardipine hydrochloride	-5.8763
61718-82-9	Fluvoxamine maleate	-5.8761

57-37-4	Benactyzine hydrochloride	-5.8761
23327-57-3	Nefopam hydrochloride	-5.8758
15307-86-5	Diclofenac	-5.8754
120011-70-3	Donepezil HCl	-5.8753
15307-81-0	Diclofenac Potassium	-5.8751
78213-16-8	Diclofenac diethylamine	-5.8751
120014-06-4	Donepezil	-5.8723
29908-03-0	Ademetionine	-5.8722
157810-81-6	Indinavir sulfate	-5.8717
236395-14-5	Eslicarbazepine Acetate	-5.8717
53-16-7	Aquacrine	-5.871
148-24-3	Hydroxyquinoline	-5.8703
49763-96-4	Stiripentol	-5.8702
22881-35-2	Famprofazone	-5.8702
56296-78-7	Fluoxetine hydrochloride	-5.8693
54910-89-3	Fluoxetine	-5.8693
105826-92-4	Tropisetron hydrochloride	-5.8693
289480-64-4	Treprostinil Sodium	-5.8688
74050-98-9	Ketanserin	-5.8686
1263-89-4	Paromomycin Sulfate	-5.8684
1104-22-9	Meclizine dihydrochloride	-5.8684
151533-22-1	Levomefolate calcium	-5.8682
127294-70-6	Balofloxacin	-5.8676
143388-64-1	Naratriptan	-5.8669
87333-19-5	Ramipril	-5.8668
150683-30-0	Tolvaptan	-5.8666
124858-35-1	Nadifloxacin	-5.8655
150322-43-3	Prasugrel	-5.8655
53251-94-8	Pinaverium bromide	-5.8655
157810-81-6	Indinavir sulfate	-5.8654
25913-34-2	Tinoridine hydrochloride	-5.8654
123524-52-7	Azelnidipine	-5.8653
113-92-8	Chlorpheniramine maleate	-5.8652
526-08-9	Sulfaphenazole	-5.8651
2203-97-6	Hydrocortisone 21-hemisuccinate	-5.8647
87233-61-2	Emedastine	-5.8644
124-94-7	Triamcinolone	-5.8644
149-64-4	Scopolamine butylbromide	-5.8632
80-49-9	Homatropine Methylbromide	-5.863
132-20-7	Pheniramine maleate	-5.863
573-20-6	Menadiol Diacetate	-5.8622
157810-81-6	Indinavir sulfate	-5.8622

1260141-27-2	Vonoprazan fumarate	-5.8621
132036-88-5	Ramosetron	-5.8617
88889-14-9	Fosinopril sodium	-5.8611
487-41-2	Forsythin	-5.8611
91161-71-6	Terbinafine	-5.8611
29767-20-2	Teniposide	-5.8611
53797-35-6	Ribostamycin sulfate	-5.8609
111025-46-8	Pioglitazone	-5.8604
3902-71-4	Trioxsalen	-5.8601
201341-05-1	Tenofovir Disoproxil	-5.8596
82752-99-6	Nefazodone hydrochloride	-5.8593
167465-36-3	Zosuquidar 3HCl	-5.8591
122647-32-9	Ibutilide Fumarate	-5.8589
3424-98-4	Telbivudine	-5.8587
93479-97-1	Glimepiride	-5.8577
167933-07-5	Flibanserin	-5.8576
54527-84-3	Nicardipine hydrochloride	-5.8576
10402-53-6	Eprazinone dihydrochloride	-5.8574
3166-62-9	Methylbenactyzine Bromide	-5.8571
147221-93-0	Delavirdine (mesylate)	-5.8571
123663-49-0	Iguratimod	-5.8568
22832-87-7	Miconazole nitrate	-5.8567
65473-14-5	Naftifine hydrochloride	-5.856
82752-99-6	Nefazodone hydrochloride	-5.8557
87333-19-5	Ramipril	-5.8557
69975-86-6	Doxofylline	-5.8556
1405-10-3	Neomycin sulfate	-5.8546
167465-36-3	Zosuquidar 3HCl	-5.8543
54965-21-8	Albendazole	-5.8539
138452-21-8	Fexofenadine hydrochloride	-5.8538
68291-97-4	Zonisamide	-5.8537
137-88-2	Amprolium Hydrochloride	-5.8534
16595-80-5	Levamisole hydrochloride	-5.8531
157810-81-6	Indinavir sulfate	-5.8528
4146-30-9	Neomycin Sulphate B	-5.8527
106730-54-5	Olprinone	-5.8525
27262-48-2	Levobupivacaine HCl	-5.8522
27262-47-1	Levobupivacaine	-5.8522
18010-40-7	Bupivacaine hydrochloride	-5.8522
157810-81-6	Indinavir sulfate	-5.8521
120-08-1	Scoparone	-5.8515
50370-12-2	Cefadroxil	-5.8512

21462-39-5	Clindamycin HCl	-5.8512
30123-17-2	Tianeptine sodium	-5.851
17696-69-4	Hematoporphyrin dihydrochloride	-5.8508
118288-08-7	Lafutidine	-5.8507
877399-52-5	Crizotinib	-5.8506
91296-87-6	Sarafloxacin hydrochloride	-5.8504
190786-44-8	Bepotastine Besilate	-5.8504
56390-09-1	Epirubicin hydrochloride	-5.8499
938-73-8	2-Ethoxybenzamide	-5.8495
1435-55-8	Hydroquinidine	-5.8489
61318-91-0	Sulconazole Nitrate	-5.8489
201341-05-1	Tenofovir Disoproxil	-5.8482
1310726-60-3	Upadacitinib	-5.8481
137975-06-5	Mozavaptan	-5.8479
123447-62-1	Prulifloxacin	-5.8472
52152-93-9	Cefsulodine sodium	-5.847
59263-76-2	Meptazinol hydrochloride	-5.8459
157810-81-6	Indinavir sulfate	-5.8458
103890-78-4	Lacidipine	-5.8453
440-17-5	Trifluoperazine dihydrochloride	-5.8452
145202-66-0	Rizatriptan benzoate	-5.8445
18683-91-5	Ambroxol	-5.8441
80-49-9	Homatropine Methylbromide	-5.8436
17696-69-4	Hematoporphyrin dihydrochloride	-5.8434
66-97-7	Psoralen	-5.8432
23327-57-3	Nefopam hydrochloride	-5.8431
91503-79-6	Flurbiprofen Axetil	-5.843
76801-85-9	Azathramycin	-5.842
298-57-7	Stugeron	-5.8416
53910-25-1	Pentostatin	-5.8416
59729-37-2	Fexinidazole	-5.8415
14222-60-7	Protionamide	-5.8408
550-99-2	Naphazoline hydrochloride	-5.8408
37693-01-9	Clofoctol	-5.8407
1263-89-4	Paromomycin Sulfate	-5.8397
135729-62-3	Palonosetron hydrochloride	-5.8396
14984-68-0	Cloperastine hydrochloride	-5.8394
83915-83-7	Lisinopril dihydrate	-5.839
23828-92-4	Ambroxol hydrochloride	-5.8375
81025-04-9	Lactitol monohydrate	-5.8367
33005-95-7	Tiaprofenic acid	-5.8361
157810-81-6	Indinavir sulfate	-5.836

1981-58-4	Sulfamethazine sodium salt	-5.8359
157810-81-6	Indinavir sulfate	-5.8358
536-33-4	Ethinamide	-5.8351
382-67-2	Desoximetasone	-5.8351
2438-32-6	Dexchlorpheniramine Maleate	-5.8348
1354744-91-4	Naloxegol oxalate	-5.8348
64872-77-1	Butoconazole nitrate	-5.8347
148-01-6	Dinitolmide	-5.8345
2062-78-4	Pimozide	-5.8345
76095-16-4	Enalapril Maleate	-5.8343
80-35-3	Sulfamethoxypyridazine	-5.8342
58-93-5	Hydrochlorothiazide	-5.8339
548-73-2	Droperidol	-5.8337
T4284	(S)-Amlodipine Besylate (103129-82-4(free base))	-5.8335
88150-42-9	Amlodipine	-5.8335
51803-78-2	Nimesulide	-5.833
3505-38-2	Carbinoxamine Maleate Salt	-5.8329
52645-53-1	Permethrin	-5.8328
100427-26-7	Lercanidipine	-5.8326
184475-35-2	Gefitinib	-5.8325
2438-32-6	Dexchlorpheniramine Maleate	-5.832
60628-96-8	Bifonazole	-5.8313
83435-67-0	Delapril?Hydrochloride	-5.8305
157810-81-6	Indinavir sulfate	-5.8303
78246-49-8	Paroxetine hydrochloride	-5.8301
491833-29-5	Eliglustat	-5.8301
87333-19-5	Ramipril	-5.8297
53251-94-8	Pinaverium bromide	-5.8296
154-69-8	Tripelennamine hydrochloride	-5.8295
1446321-46-5	voxelotor	-5.829
111406-87-2	Zileuton	-5.8289
121-33-5	Vanillin	-5.8289
157810-81-6	Indinavir sulfate	-5.8285
3963-95-9	Methacycline hydrochloride	-5.8285
23327-57-3	Nefopam hydrochloride	-5.8281
157810-81-6	Indinavir sulfate	-5.8279
878143-33-0	Dalasetron Mesylate Hydrate	-5.8271
122647-32-9	Ibutilide Fumarate	-5.8271
149-64-4	Scopolamine butylbromide	-5.8269
832720-36-2	Elagolix sodium	-5.8265
382-67-2	Desoximetasone	-5.8265

938-73-8	2-Ethoxybenzamide	-5.8263
60628-96-8	Bifonazole	-5.8258
83881-52-1	Cetirizine dihydrochloride	-5.8257
745-65-3	Alprostadil	-5.8252
123663-49-0	Iguratimod	-5.8251
29908-03-0	Ademetionine	-5.8246
2468-21-5	Catharanthine	-5.8245
745-65-3	Alprostadil	-5.8241
147526-32-7	Pitavastatin calcium	-5.824
83435-67-0	Delapril?Hydrochloride	-5.8236
66085-59-4	Nimodipine	-5.8236
17560-51-9	Metolazone	-5.8232
61318-91-0	Sulconazole Nitrate	-5.8228
64019-93-8	Dipivefrin hydrochloride	-5.8219
943-17-9	Etilefrine hydrochloride	-5.8217
157810-81-6	Indinavir sulfate	-5.8217
163680-77-1	Pazufloxacin Mesylate	-5.8216
61-12-1	Dibucaine hydrochloride	-5.8215
85-79-0	Dibucaine	-5.8215
149709-62-6	AHU377	-5.8214
1369773-39-6	AHU377 calcium salt	-5.8214
84-02-6	Prochlorperazine Maleate	-5.8213
58186-27-9	Idebenone	-5.8205
85622-93-1	Temozolomide	-5.8198
78628-80-5	Terbinafine hydrochloride	-5.8197
100427-26-7	Lercanidipine	-5.8195
906673-24-3	Crisaborole	-5.8191
4360-12-7	Ajmaline	-5.8191
174671-46-6	Tavaborole	-5.8188
538-71-6	Domiphen Bromide	-5.8186
394730-60-0	Boceprevir	-5.8181
132866-11-6	Lercanidipine hydrochloride	-5.818
178307-42-1	Revaprazan hydrochloride	-5.8174
537-12-2	Diperodon hydrochloride	-5.8156
130018-77-8	Levocetirizine	-5.8146
3505-38-2	Carbinoxamine Maleate Salt	-5.8138
3902-71-4	Trioxsalen	-5.8137
167933-07-5	Flibanserin	-5.8136
83881-52-1	Cetirizine dihydrochloride	-5.8131
27848-84-6	Nicergoline	-5.813
57808-66-9	Domperidone	-5.8129
150322-43-3	Prasugrel	-5.8123

29608-49-9	Almitrine dimesylate	-5.8122
107007-99-8	Granisetron hydrochloride	-5.8119
441798-33-0	Macitentan	-5.8117
182349-12-8	Rupatadine Fumarate	-5.8113
79-57-2	Oxytetracycline	-5.811
15676-16-1	Sulpiride	-5.8097
28657-80-9	Cinoxacin	-5.8096
157810-81-6	Indinavir sulfate	-5.8094
329-63-5	DL-Adrenaline Hydrochloride	-5.8093
63612-50-0	Nilutamide	-5.8093
127294-70-6	Balofloxacin	-5.8091
111974-72-2	Quetiapine Fumarate	-5.809
80-49-9	Homatropine Methylbromide	-5.8088
150322-43-3	Prasugrel	-5.8087
24729-96-2	Clindamycin phosphate	-5.8086
906673-24-3	Crisaborole	-5.8083
839712-12-8	Cariprazine	-5.8083
22457-89-2	Benfotiamine	-5.8083
550-99-2	Naphazoline hydrochloride	-5.8079
79-57-2	Oxytetracycline	-5.8079
42924-53-8	Nabumetone	-5.8074
157810-81-6	Indinavir sulfate	-5.8072
53910-25-1	Pentostatin	-5.8071
912-60-7	Noscapine hydrochloride	-5.8068
19428-14-9	Benproperine phosphate	-5.8054
157810-81-6	Indinavir sulfate	-5.8054
29218-27-7	MD 69276	-5.8052
84680-54-6	Enalaprilat Dihydrate	-5.8045
1374640-70-6	Rociletinib	-5.8044
171596-29-5	Tadalafil	-5.8041
157810-81-6	Indinavir sulfate	-5.8037
58-93-5	Hydrochlorothiazide	-5.803
107753-78-6	Zafirlukast	-5.803
154-69-8	Tripelennamine hydrochloride	-5.8025
122111-03-9	Gemcitabine HCl	-5.802
95058-81-4	Gemcitabine	-5.802
2468-21-5	Catharanthine	-5.802
1263-89-4	Paromomycin Sulfate	-5.8015
548-73-2	Droperidol	-5.8014
106730-54-5	Olprinone	-5.801
23327-57-3	Nefopam hydrochloride	-5.801
113-92-8	Chlorpheniramine maleate	-5.8004

65513-72-6	Glafenine Hydrochloride	-5.8001
3820-67-5	Glafenine	-5.8001
1435-55-8	Hydroquinidine	-5.7998
66085-59-4	Nimodipine	-5.7995
111025-46-8	Pioglitazone	-5.7993
808118-40-3	Roxadustat	-5.7992
329-63-5	DL-Adrenaline Hydrochloride	-5.7989
29908-03-0	Ademetionine	-5.7988
39562-70-4	Nitrendipine	-5.7982
74863-84-6	Argatroban	-5.798
66085-59-4	Nimodipine	-5.798
59729-32-7	Citalopram HBr	-5.798
63372-86-1	Metamizole magnesium	-5.7977
320345-99-1	Aclidinium bromide	-5.7972
99011-02-6	Imiquimod	-5.7969
5578-73-4	Sanguinarine chloride	-5.7967
60628-96-8	Bifonazole	-5.7966
32854-75-4	Lappaconitine	-5.7965
148849-67-6	Ivabradine hydrochloride	-5.7964
3505-38-2	Carbinoxamine Maleate Salt	-5.7962
130773-02-3	Neticonazole Hydrochloride	-5.7959
68302-57-8	Amlexanox	-5.7957
55-48-1	Atropine sulfate	-5.7956
5908-99-6	Atropine sulfate monohydrate	-5.7956
51-55-8	Atropine	-5.7956
51-52-5	Propylthiouracil	-5.7951
22373-78-0	Monensin sodium salt	-5.7949
111025-46-8	Pioglitazone	-5.7948
1374640-70-6	Rociletinib	-5.7943
386750-22-7	Desvenlafaxine succinate hydrate	-5.7936
93413-62-8	Desvenlafaxine	-5.7936
36791-04-5	Ribavirin	-5.7935
68-89-3	Dipyron	-5.7932
5907-38-0	Analgin	-5.7932
74050-98-9	Ketanserin	-5.7928
29908-03-0	Ademetionine	-5.7927
67227-57-0	Fenoldopam mesylate	-5.7923
2259-96-3	Cyclothiazide	-5.7921
73590-85-9	Omeprazole sulfide	-5.7921
89226-75-5	Manidipine dihydrochloride	-5.7913
52549-17-4	Pranoprofen	-5.7912
201341-05-1	Tenofovir Disoproxil	-5.791

111902-57-9	Temocapil	-5.7908
89-25-8	Edaravone	-5.7908
29608-49-9	Almitrine dimesylate	-5.7907
66898-62-2	Talniflumate	-5.7906
16773-42-5	Ornidazole	-5.7904
59263-76-2	Meptazinol hydrochloride	-5.7897
37517-28-5	Amikacin	-5.7895
536-33-4	Ethinamide	-5.7893
165800-03-3	Linezolid	-5.7893
147221-93-0	Delavirdine (mesylate)	-5.7891
112665-43-7	Seratrodast	-5.7888
99011-02-6	Imiquimod	-5.7887
112809-51-5	Letrozole	-5.7883
6066-49-5	Butylphthalide	-5.7882
363-24-6	Prostaglandin E2 (PGE2)	-5.7879
89226-50-6	Manidipine	-5.7876
89226-75-5	Manidipine dihydrochloride	-5.7876
14698-29-4	Oxolinic acid	-5.7875
25451-15-4	Felbamate	-5.7872
938-73-8	2-Ethoxybenzamide	-5.7869
75330-75-5	Lovastatin	-5.7869
1354744-91-4	Naloxegol oxalate	-5.7863
51-30-9	Isoprenaline hydrochloride	-5.786
1263-89-4	Paromomycin Sulfate	-5.7859
67814-76-0	Isepamicin sulfate	-5.7855
67227-57-0	Fenoldopam mesylate	-5.7855
89-25-8	Edaravone	-5.7855
123663-49-0	Iguratimod	-5.7854
182349-12-8	Rupatadine Fumarate	-5.7848
329-63-5	DL-Adrenaline Hydrochloride	-5.7847
106308-44-5	Rufinamide	-5.7846
201341-05-1	Tenofovir Disoproxil	-5.7846
111902-57-9	Temocapil	-5.7844
89226-50-6	Manidipine	-5.7844
10246-75-0	Hydroxyzine Pamoate	-5.7842
61318-91-0	Sulconazole Nitrate	-5.7841
317-34-0	Aminophylline	-5.7838
2192-20-3	Hydroxyzine dihydrochloride	-5.7836
4852-22-6	Proanthocyanidins	-5.7835
25451-15-4	Felbamate	-5.7833
61318-91-0	Sulconazole Nitrate	-5.7831
2468-21-5	Catharanthine	-5.783

122320-73-4	Rosiglitazone	-5.7825
64-86-8	Colchicine	-5.7822
84625-61-6	Itraconazole	-5.7818
6170-42-9	Chloropyramine hydrochloride	-5.7816
157810-81-6	Indinavir sulfate	-5.7808
87333-19-5	Ramipril	-5.7805
60628-96-8	Bifonazole	-5.7803
34580-14-8	Ketotifen fumarate	-5.7802
189279-58-1	Delafloxacin	-5.7801
29908-03-0	Ademetionine	-5.7797
57808-66-9	Domperidone	-5.7794
111025-46-8	Pioglitazone	-5.7791
2203-97-6	Hydrocortisone 21-hemisuccinate	-5.779
163253-35-8	Sitafloxacin Hydrate	-5.7787
59263-76-2	Meptazinol hydrochloride	-5.7787
34580-14-8	Ketotifen fumarate	-5.7785
610798-31-7	Icotinib	-5.778
149022-22-0	Amikacin Sulfate Salt	-5.777
51803-78-2	Nimesulide	-5.7767
1163-36-6	Clemizole hydrochloride	-5.7764
32986-56-4	Tobramycin	-5.7761
329-63-5	DL-Adrenaline Hydrochloride	-5.776
58-15-1	Aminophenazone	-5.7752
157810-81-6	Indinavir sulfate	-5.7751
11018-89-6	Ouabain octahydrate	-5.7748
7681-76-7	Ronidazole	-5.7744
181695-72-7	Valdecoxib	-5.7737
50-63-5	Chloroquine diphosphate	-5.7736
127-71-9	Sulfabenzamide	-5.7733
124858-35-1	Nadifloxacin	-5.7732
56296-78-7	Fluoxetine hydrochloride	-5.7728
54910-89-3	Fluoxetine	-5.7728
72-33-3	Mestranol	-5.7728
29908-03-0	Ademetionine	-5.7726
31282-04-9	Hygromycin B	-5.7724
91503-79-6	Flurbiprofen Axetil	-5.7718
122852-69-1	Alosetron hydrochloride	-5.7715
4350-09-8	L-5-Hydroxytryptophan	-5.7711
122647-32-9	Ibutilide Fumarate	-5.771
62893-19-0	Cefoperazone	-5.7709
187235-37-6	Pretomanid	-5.7707
129722-12-9	Aripiprazole	-5.7704
91503-79-6	Flurbiprofen Axetil	-5.7703

78281-72-8	Nepafenac	-5.77
137-88-2	Amprolium Hydrochloride	-5.7692
129-06-6	Warfarin sodium	-5.7687
65513-72-6	Glafenine Hydrochloride	-5.7669
3820-67-5	Glafenine	-5.7669
91-33-8	Benzthiazide	-5.7667
31282-04-9	Hygromycin B	-5.7666
7491-74-9	Piracetam	-5.7665
980-71-2	Brompheniramine hydrogen maleate	-5.7665
1210344-57-2	Ertugliflozin	-5.7665
83-07-8	4-Aminoantipyrine	-5.7663
832720-36-2	Elagolix sodium	-5.7654
19237-84-4	Prazosin hydrochloride	-5.765
66898-62-2	Talniflumate	-5.7645
111902-57-9	Temocapil	-5.7642
14984-68-0	Cloperastine hydrochloride	-5.7636
456-59-7	Cyclandelate	-5.7629
89396-94-1	Imidapril HCl	-5.7627
11018-89-6	Ouabain octahydrate	-5.7624
10212-25-6	Cyclocytidine hydrochloride	-5.7613
157810-81-6	Indinavir sulfate	-5.7606
17696-69-4	Hematoporphyrin dihydrochloride	-5.7603
138-14-7	Deferoxamine Mesylate	-5.7597
3963-95-9	Methacycline hydrochloride	-5.7596
59338-87-3	Alizapride hydrochloride	-5.7596
41354-29-4	Cyproheptadine hydrochloride	-5.7595
57381-26-7	Irsogladine	-5.759
14919-77-8	Benserazide hydrochloride	-5.7589
10246-75-0	Hydroxyzine Pamoate	-5.7586
27262-48-2	Levobupivacaine HCl	-5.7582
27262-47-1	Levobupivacaine	-5.7582
18010-40-7	Bupivacaine hydrochloride	-5.7582
69-81-8	Carbazochrome	-5.7577
755037-03-7	Regorafenib	-5.7573
27848-84-6	Nicergoline	-5.7573
54965-21-8	Albendazole	-5.7572
2192-20-3	Hydroxyzine dihydrochloride	-5.7571
6024-85-7	Tetrahydropalmatine hydrochloride	-5.7569
15676-16-1	Sulpiride	-5.7558
192725-17-0	Lopinavir	-5.7542
3011-89-0	Alkomide	-5.7537
67227-57-0	Fenoldopam mesylate	-5.7536

49763-96-4	Stiripentol	-5.7532
82410-32-0	Ganciclovir	-5.7531
137-88-2	Amprolium Hydrochloride	-5.752
1446321-46-5	voxelotor	-5.7515
129453-61-8	Fulvestrant	-5.7514
53746-45-5	Fenoprofen calcium dihydrate	-5.7514
33005-95-7	Tiaprofenic acid	-5.7512
452-35-7	Ethoxzolamide	-5.7511
456-59-7	Cyclandelate	-5.751
51-30-9	Isoprenaline hydrochloride	-5.751
129453-61-8	Fulvestrant	-5.7509
99011-02-6	Imiquimod	-5.7508
74536-44-0	Metadoxine	-5.7502
30751-05-4	Troxipide	-5.7502
41340-25-4	Etodolac	-5.7492
119637-67-1	Moguisteine	-5.7492
317-34-0	Aminophylline	-5.7486
3200-06-4	Nafronyl oxalate	-5.7479
879085-55-9	Vismodegib	-5.7478
2152-44-5	Betamethasone 17-valerate	-5.7475
521-78-8	Trimipramine maleate	-5.7472
130773-02-3	Neticonazole Hydrochloride	-5.7471
485-49-4	(+)-Bicuculline	-5.7468
39562-70-4	Nitrendipine	-5.7468
980-71-2	Brompheniramine hydrogen maleate	-5.7465
157810-81-6	Indinavir sulfate	-5.7464
130773-02-3	Neticonazole Hydrochloride	-5.7464
721-50-6	Prilocaine	-5.746
91503-79-6	Flurbiprofen Axetil	-5.746
16816-67-4	D-Pantethine	-5.7458
1786-81-8	Propitocaine hydrochloride	-5.7457
26095-59-0	Otilonium bromide	-5.7454
14976-57-9	Clemastine fumarate	-5.7452
82410-32-0	Ganciclovir	-5.745
68291-97-4	Zonisamide	-5.7449
2062-78-4	Pimozide	-5.7449
5630-53-5	Tibolone	-5.7448
91296-87-6	Sarafloxacin hydrochloride	-5.7448
63-45-6	Primaquine diphosphate	-5.7446
58-56-0	Pyridoxine hydrochloride	-5.7445
65-23-6	Pyridoxine	-5.7445
136310-93-5	Tiotropium bromide	-5.7443

912-60-7	Noscapine hydrochloride	-5.744
30484-77-6	Flunarizine dihydrochloride	-5.7439
11013-97-1	Methyl hesperidin	-5.7427
1098-97-1	Pyrithioxin	-5.7421
82752-99-6	Nefazodone hydrochloride	-5.7421
14976-57-9	Clemastine fumarate	-5.7415
163521-12-8	Vilazodone	-5.7413
29908-03-0	Ademetionine	-5.7404
1263-89-4	Paromomycin Sulfate	-5.7401
75507-68-5	Flupirtine maleate	-5.7397
151533-22-1	Levomefolate calcium	-5.7394
63612-50-0	Nilutamide	-5.7394
128794-94-5	Mycophenolate Mofetil	-5.7388
81919-14-4	Bendazac L-lysine	-5.7376
182349-12-8	Rupatadine Fumarate	-5.7376
105826-92-4	Tropisetron hydrochloride	-5.7373
111974-72-2	Quetiapine Fumarate	-5.7363
51022-69-6	Amcinonide	-5.7361
67656-30-8	10-Hydroxycampothecin	-5.7356
19685-09-7	10-Hydroxycamptothecin	-5.7356
157810-81-6	Indinavir sulfate	-5.7355
91-64-5	Coumarin	-5.7354
64439-81-2	Hydroxy Camptothecine	-5.7353
78628-80-5	Terbinafine hydrochloride	-5.7348
138452-21-8	Fexofenadine hydrochloride	-5.7347
26159-34-2	Naproxen sodium	-5.7339
22204-53-1	Naproxen	-5.7339
169590-42-5	Celecoxib	-5.7337
64-73-3	Demeclocycline hydrochloride	-5.7336
69712-56-7	Cefotetan	-5.7335
80-32-0	Sulfachloropyridazine	-5.7334
1051375-19-9	Dolutegravir sodium	-5.7333
6968-72-5	Mepiroxol	-5.7332
19428-14-9	Benproperine phosphate	-5.7331
65513-72-6	Glafenine Hydrochloride	-5.7329
3820-67-5	Glafenine	-5.7329
51-30-9	Isoprenaline hydrochloride	-5.7323
147221-93-0	Delavirdine (mesylate)	-5.7318
171596-29-5	Tadalafil	-5.7315
83105-70-8	Sultamicillin Tosylate	-5.7315
946075-13-4	Isavuconazonium sulfate	-5.731
23327-57-3	Nefopam hydrochloride	-5.7309

1032900-25-6	LDK378	-5.7306
103628-48-4	Sumatriptan succinate	-5.7299
912-60-7	Noscapine hydrochloride	-5.7287
608141-41-9	Apremilast	-5.7286
136470-78-5	Abacavir	-5.7282
84057-84-1	Lamotrigine	-5.7278
188062-50-2	Abacavir sulfate	-5.7276
3505-38-2	Carbinoxamine Maleate Salt	-5.7273
181183-52-8	Almotriptan Malate	-5.7272
207844-01-7	Mitiglinide calcium	-5.7272
65513-72-6	Glafenine Hydrochloride	-5.7272
3820-67-5	Glafenine	-5.7272
19237-84-4	Prazosin hydrochloride	-5.727
341-69-5	Orphenadrine hydrochloride	-5.727
161715-24-8	Tebipenem Pivoxil	-5.7259
363-24-6	Prostaglandin E2 (PGE2)	-5.7259
485-49-4	(+)-Bicuculline	-5.7259
61318-91-0	Sulconazole Nitrate	-5.7258
132-18-3	Diphenylpyraline hydrochloride	-5.7257
93479-97-1	Glimepiride	-5.7257
76095-16-4	Enalapril Maleate	-5.7254
269055-15-4	Etravirine	-5.7251
164579-32-2	Pantoprazole Sodium Hydrate	-5.7249
145202-66-0	Rizatriptan benzoate	-5.7241
23327-57-3	Nefopam hydrochloride	-5.724
132203-70-4	Cilnidipine	-5.7233
54-35-3	Procaine penicillin G	-5.7231
52152-93-9	Cefsulodine sodium	-5.7231
19356-17-3	Calcifediol	-5.7226
747-36-4	Hydroxychloroquine sulfate	-5.7223
202409-33-4	Etoricoxib	-5.722
91-85-0	Thonzylamine	-5.7219
1263-89-4	Paromomycin Sulfate	-5.7211
321-30-2	Adenine sulfate	-5.7211
56-75-7	Chloramphenicol	-5.7206
73-24-5	Adenine	-5.7202
149709-62-6	AHU377	-5.7193
1369773-39-6	AHU377 calcium salt	-5.7193
83881-52-1	Cetirizine dihydrochloride	-5.7191
573-20-6	Menadiol Diacetate	-5.7189
62-68-0	Proadifen hydrochloride	-5.7187

1269440-17-6	Encorafenib	-5.7184
81919-14-4	Bendazac L-lysine	-5.7181
175865-59-5	Valganciclovir hydrochloride	-5.7179
69-81-8	Carbazochrome	-5.7175
15318-45-3	Thiamphenicol	-5.7175
72432-03-2	Miglitol	-5.7171
167465-36-3	Zosuquidar 3HCl	-5.717
61-12-1	Dibucaine hydrochloride	-5.7167
85-79-0	Dibucaine	-5.7167
211914-51-1	Dabigatran	-5.7161
87333-19-5	Ramipril	-5.716
552-41-0	Paeonol	-5.716
138-37-4	Mafenide hydrochloride	-5.7159
27848-84-6	Nicergoline	-5.7159
141-94-6	Hexetidine	-5.7158
443913-73-3	Vandetanib	-5.7158
141-94-6	Hexetidine	-5.7157
17696-69-4	Hematoporphyrin dihydrochloride	-5.7157
111902-57-9	Temocapil	-5.715
375815-87-5	Varenicline Tartrate	-5.715
110221-44-8	Temocapril HCl	-5.7147
1374356-45-2	(S)-crizotinib	-5.7147
178307-42-1	Revaprazan hydrochloride	-5.7141
119637-67-1	Moguisteine	-5.714
173352-39-1	Afobazole hydrochloride	-5.7127
62893-19-0	Cefoperazone	-5.7126
23828-92-4	Ambroxol hydrochloride	-5.7122
18683-91-5	Ambroxol	-5.7119
52645-53-1	Permethrin	-5.7119
51012-33-0	Tiapride hydrochloride	-5.7113
148-01-6	Dinitolmide	-5.7109
157810-81-6	Indinavir sulfate	-5.7108
10246-75-0	Hydroxyzine Pamoate	-5.7103
943-17-9	Etilefrine hydrochloride	-5.7097
2192-20-3	Hydroxyzine dihydrochloride	-5.7097
132-98-9	Penicillin V potassium salt	-5.7096
184475-35-2	Gefitinib	-5.709
86541-74-4	Benazepril hydrochloride	-5.7083
29908-03-0	Ademetionine	-5.7077
157283-68-6	Travoprost	-5.707

1360460-82-7	Anlotinib Dihydrochloride	-5.7068
1058156-90-3	Anlotinib	-5.7068
72-33-3	Mestranol	-5.7067
1225-55-4	Protriptyline hydrochloride	-5.7065
452-35-7	Ethoxzolamide	-5.7063
89226-75-5	Manidipine dihydrochloride	-5.7058
18010-40-7	Bupivacaine hydrochloride	-5.705
89226-50-6	Manidipine	-5.7049
980-71-2	Brompheniramine hydrogen maleate	-5.7044
140-64-7	Pentamidine isethionate	-5.7044
113-92-8	Chlorpheniramine maleate	-5.7043
17696-69-4	Hematoporphyrin dihydrochloride	-5.7038
82248-59-7	Atomoxetine hydrochloride	-5.7034
164579-32-2	Pantoprazole Sodium Hydrate	-5.7031
52645-53-1	Permethrin	-5.7028
72432-10-1	Aniracetam	-5.7027
56-75-7	Chloramphenicol	-5.7027
127294-70-6	Balofloxacin	-5.7023
173334-58-2	Aliskiren hemifumarate	-5.702
171599-83-0	Sildenafil citrate	-5.702
5104-49-4	Flurbiprofen	-5.7017
1263-89-4	Paromomycin Sulfate	-5.7017
915087-33-1	Enzalutamide	-5.7016
151533-22-1	Levomefolate calcium	-5.7014
60925-61-3	Ceforanide	-5.7013
3056-17-5	Stavudine	-5.7007
1072833-77-2	Ixazomib	-5.7004
83915-83-7	Lisinopril dihydrate	-5.7003
66-97-7	Psoralen	-5.7001
1163-36-6	Clemizole hydrochloride	-5.6999
31282-04-9	Hygromycin B	-5.6988
145202-66-0	Rizatriptan benzoate	-5.6983
856867-55-5	Tedizolid Phosphate	-5.6978
496775-61-2	Eltrombopag	-5.6969
73-48-3	Bendroflumethiazide	-5.6962
161715-24-8	Tebipenem Pivoxil	-5.6961
946075-13-4	Isavuconazonium sulfate	-5.6959
912273-65-5	Edoxaban	-5.6957
3505-38-2	Carbinoxamine Maleate Salt	-5.6955
906093-29-6	Teneligliptin hydrobromide	-5.6948

52-21-1	Prednisolone-21-acetate	-5.6947
3759-92-0	Furaltadone hydrochloride	-5.6943
57808-66-9	Domperidone	-5.693
68302-57-8	Amlexanox	-5.6928
61318-91-0	Sulconazole Nitrate	-5.6927
92-61-5	Scopoletin	-5.6921
456-59-7	Cyclandelate	-5.6921
3543-75-7	Bendamustine hydrochloride	-5.6919
879085-55-9	Vismodegib	-5.6917
78281-72-8	Nepafenac	-5.6912
74536-44-0	Metadoxine	-5.6905
202189-78-4	Bilastine	-5.6899
58-56-0	Pyridoxine hydrochloride	-5.6896
65-23-6	Pyridoxine	-5.6896
1263-89-4	Paromomycin Sulfate	-5.6894
603139-19-1	Odanacatib	-5.6882
10040-45-6	Sodium Picosulfate	-5.6875
54-30-8	Camylofin	-5.6875
163253-35-8	Sitafloxacin Hydrate	-5.6873
105826-92-4	Tropisetron hydrochloride	-5.6872
178307-42-1	Revaprazan hydrochloride	-5.6868
123040-16-4	Azasetron hydrochloride	-5.6867
23327-57-3	Nefopam hydrochloride	-5.6865
53885-35-1	Ticlopidine hydrochloride	-5.6863
137-88-2	Amprolium Hydrochloride	-5.6846
78628-80-5	Terbinafine hydrochloride	-5.6844
2259-96-3	Cyclothiazide	-5.6843
91-85-0	Thonzylamine	-5.6843
906673-24-3	Crisaborole	-5.6831
873054-44-5	Ivacaftor	-5.6825
82248-59-7	Atomoxetine hydrochloride	-5.6823
82586-55-8	Quinapril hydrochloride	-5.6821
132-20-7	Pheniramine maleate	-5.6819
57-37-4	Benactyzine hydrochloride	-5.6819
485-49-4	(+)-Bicuculline	-5.6817
68-22-4	Norethindrone	-5.6817
76801-85-9	Azathramycin	-5.6814
532-11-6	Anethole trithione	-5.6812
402957-28-2	Telaprevir	-5.6807
53746-45-5	Fenoprofen calcium dihydrate	-5.6803
41354-29-4	Cyproheptadine hydrochloride	-5.679
133-67-5	Trichlormethiazide	-5.6789
202409-33-4	Etoricoxib	-5.6785

17696-69-4	Hematoporphyrin dihydrochloride	-5.6784
149-64-4	Scopolamine butylbromide	-5.6781
878143-33-0	Dalasetron Mesylate Hydrate	-5.6779
24168-96-5	Isoconazole nitrate	-5.6772
859-18-7	Lincomycin hydrochloride	-5.6771
29908-03-0	Ademetionine	-5.6769
75695-93-1	Isradipine	-5.6767
65391-42-6	Bestatin hydrochloride	-5.6764
58970-76-6	Ubenimex	-5.6764
49763-96-4	Stiripentol	-5.6762
7179-49-9	Lincomycin hydrochloride monohydrate	-5.6762
360-70-3	Nandrolone decanoate	-5.6761
14976-57-9	Clemastine fumarate	-5.6761
1616493-44-7	Tucidinostat	-5.6759
27262-48-2	Levobupivacaine HCl	-5.6758
27262-47-1	Levobupivacaine	-5.6758
18010-40-7	Bupivacaine hydrochloride	-5.6758
26159-34-2	Naproxen sodium	-5.6757
22204-53-1	Naproxen	-5.6757
72432-03-2	Miglitol	-5.6757
22832-87-7	Miconazole nitrate	-5.6754
78415-72-2	Milrinone	-5.6753
153773-82-1	Ertapenem sodium	-5.675
157810-81-6	Indinavir sulfate	-5.6749
133-67-5	Trichlormethiazide	-5.6746
80433-71-2	Calcium Levofolinate	-5.6741
75695-93-1	Isradipine	-5.6741
945667-22-1	Saxagliptin hydrate	-5.6738
69712-56-7	Cefotetan	-5.6738
2139-47-1	Nifenazone	-5.6738
66085-59-4	Nimodipine	-5.6737
22881-35-2	Famprofazone	-5.6733
93479-97-1	Glimepiride	-5.6729
443913-73-3	Vandetanib	-5.6729
375815-87-5	Varenicline Tartrate	-5.6728
219861-08-2	Escitalopram Oxalate	-5.6725
59729-32-7	Citalopram HBr	-5.6725
111025-46-8	Pioglitazone	-5.6725
6024-85-7	Tetrahydropalmatine hydrochloride	-5.6724
797-63-7	Levonorgestrel	-5.6713
30484-77-6	Flunarizine dihydrochloride	-5.6712
68-22-4	Norethindrone	-5.6707

179474-81-8	Prucalopride	-5.6702
3759-92-0	Furaltadone hydrochloride	-5.67
71675-85-9	Amisulpride	-5.6699
1025504-45-3	Valbenazine	-5.6699
90729-43-4	Ebastine	-5.6698
3737-09-5	Disopyramide	-5.6698
83881-52-1	Cetirizine dihydrochloride	-5.6697
386750-22-7	Desvenlafaxine succinate hydrate	-5.6687
93413-62-8	Desvenlafaxine	-5.6687
603-50-9	Bisacodyl	-5.6683
70356-03-5	Cefaclor monohydrate	-5.6679
113-98-4	Benzylpenicillin potassium	-5.6678
153773-82-1	Ertapenem sodium	-5.667
69-57-8	Penicillin G sodium salt	-5.6669
6066-49-5	Butylphthalide	-5.6666
287714-41-4	Rosuvastatin	-5.6663
1051375-16-6	Dolutegravir	-5.6652
190786-44-8	Bepotastine Besilate	-5.6652
274901-16-5	Vildagliptin	-5.6649
189279-58-1	Delafloxacin	-5.6638
63-45-6	Primaquine diphosphate	-5.6632
121032-29-9	Nelarabine	-5.6631
394730-60-0	Boceprevir	-5.663
100-51-6	Benzyl alcohol	-5.6627
426-13-1	Fluorometholone	-5.6624
219861-08-2	Escitalopram Oxalate	-5.6622
59729-32-7	Citalopram HBr	-5.6622
121032-29-9	Nelarabine	-5.6621
366789-02-8	Rivaroxaban	-5.6616
123447-62-1	Prulifloxacin	-5.6615
1051375-19-9	Dolutegravir sodium	-5.6613
84057-95-4	Ropivacaine	-5.6597
98717-15-8	Ropivacaine hydrochloride	-5.6597
17440-83-4	Amiloride hydrochloride dihydrate	-5.6595
304-84-7	Ethamivan	-5.6594
100427-26-7	Lercanidipine	-5.6592
6151-40-2	Quinidine hydrochloride	-5.6587
132866-11-6	Lercanidipine hydrochloride	-5.6583
51012-33-0	Tiapride hydrochloride	-5.6581
618385-01-6	Vorapaxar	-5.6578

1051375-16-6	Dolutegravir	-5.6576
14919-77-8	Benserazide hydrochloride	-5.6573
2413-38-9	Fupentixol Dihydrochloride	-5.6573
202409-33-4	Etoricoxib	-5.657
31637-97-5	Etofibrate	-5.6569
128794-94-5	Mycophenolate Mofetil	-5.6566
174671-46-6	Tavaborole	-5.6564
1508-75-4	Tropicamide	-5.6562
1360460-82-7	Anlotinib Dihydrochloride	-5.6558
1058156-90-3	Anlotinib	-5.6558
157810-81-6	Indinavir sulfate	-5.6547
738-70-5	Trimethoprim	-5.6546
15574-96-6	Pizotifen	-5.6539
5189-11-7	Pizotifen Malate	-5.6539
60-54-8	Tetracycline	-5.6534
74863-84-6	Argatroban	-5.653
178307-42-1	Revaprazan hydrochloride	-5.6529
1435-55-8	Hydroquinidine	-5.6523
14976-57-9	Clemastine fumarate	-5.6523
641571-10-0	Nilotinib	-5.6521
536-33-4	Ethinamide	-5.6517
14919-77-8	Benserazide hydrochloride	-5.6516
23327-57-3	Nefopam hydrochloride	-5.6515
41354-29-4	Cyproheptadine hydrochloride	-5.6513
797-63-7	Levonorgestrel	-5.6512
1269440-17-6	Encorafenib	-5.6511
111025-46-8	Pioglitazone	-5.6509
51803-78-2	Nimesulide	-5.6508
28657-80-9	Cinoxacin	-5.6506
1405-10-3	Neomycin sulfate	-5.6502
89-25-8	Edaravone	-5.65
87333-19-5	Ramipril	-5.6497
56-75-7	Chloramphenicol	-5.6496
22862-76-6	Anisomycin	-5.6494
78628-80-5	Terbinafine hydrochloride	-5.6494
1405-10-3	Neomycin sulfate	-5.6493
4146-30-9	Neomycin Sulphate B	-5.6492
120-51-4	Benzyl benzoate	-5.649
1163-36-6	Clemizole hydrochloride	-5.6488
34140-59-5	Trimebutine maleate	-5.6487

39133-31-8	Trimebutine	-5.6487
151533-22-1	Levomefolate calcium	-5.6486
15574-96-6	Pizotifen	-5.6486
5189-11-7	Pizotifen Malate	-5.6483
153-98-0	Serotonin hydrochloride	-5.6482
5104-49-4	Flurbiprofen	-5.6481
7759-35-5	Nestoron	-5.648
63372-86-1	Metamizole magnesium	-5.6473
130-37-0	Menadione sodium bisulfite	-5.6473
912273-65-5	Edoxaban	-5.6472
62-31-7	Dopamine hydrochloride	-5.647
21736-83-4	Spectinomycin dihydrochloride	-5.6469
33286-22-5	Diltiazem hydrochloride	-5.6469
705260-08-8	Vorapaxar sulfate	-5.6467
68-89-3	Dipyron	-5.6467
5907-38-0	Analgin	-5.6467
17560-51-9	Metolazone	-5.6463
4146-30-9	Neomycin Sulphate B	-5.6461
56-04-2	Methylthiouracil	-5.6454
3644-61-9	Tolperisone hydrochloride	-5.6451
4146-30-9	Neomycin Sulphate B	-5.645
162359-56-0	Fingolimod hydrochloride	-5.6449
120011-70-3	Donepezil HCl	-5.6445
548-66-3	Drofenine hydrochloride	-5.6442
73151-29-8	Fenticonazole Nitrate	-5.6441
34140-59-5	Trimebutine maleate	-5.6436
39133-31-8	Trimebutine	-5.6436
3644-61-9	Tolperisone hydrochloride	-5.6434
120014-06-4	Donepezil	-5.6433
15686-71-2	Cephalexin	-5.643
618385-01-6	Vorapaxar	-5.643
651-06-9	Sulfameter	-5.6425
573-20-6	Menadiol Diacetate	-5.642
850876-88-9	Danoprevir	-5.6419
1195765-45-7	Dabrafenib	-5.6419
10405-02-4	Trospium chloride	-5.6413
84057-95-4	Ropivacaine	-5.6411
98717-15-8	Ropivacaine hydrochloride	-5.6411
18085-97-7	Jaceosidin	-5.6409
912-60-7	Noscapine hydrochloride	-5.6409
22994-85-0	Benznidazole	-5.6409
101-31-5	L-Hyoscyamine	-5.6401

705260-08-8	Vorapaxar sulfate	-5.64
59263-76-2	Meptazinol hydrochloride	-5.6398
25913-34-2	Tinoridine hydrochloride	-5.6398
74863-84-6	Argatroban	-5.6397
66-84-2	Glucosamine hydrochloride	-5.6396
639089-54-6	Tozasertib	-5.6394
912273-65-5	Edoxaban	-5.6392
24729-96-2	Clindamycin phosphate	-5.639
118288-08-7	Lafutidine	-5.6389
61-16-5	Methoxamine hydrochloride	-5.6387
639089-54-6	Tozasertib	-5.6387
53251-94-8	Pinaverium bromide	-5.6381
163252-36-6	Clevudine	-5.638
1405-10-3	Neomycin sulfate	-5.6376
918504-65-1	Vemurafenib	-5.6371
58-32-2	Dypyridamole	-5.6371
17696-69-4	Hematoporphyrin dihydrochloride	-5.637
61270-78-8	Cefonicid sodium	-5.6362
55-48-1	Atropine sulfate	-5.6362
5908-99-6	Atropine sulfate monohydrate	-5.6362
51-55-8	Atropine	-5.6362
28657-80-9	Cinoxacin	-5.6362
4146-30-9	Neomycin Sulphate B	-5.6362
1195765-45-7	Dabrafenib	-5.6357
33005-95-7	Tiaprofenic acid	-5.6355
T4284	(S)-Amlodipine Besylate (103129-82-4(free base))	-5.6349
88150-42-9	Amlodipine	-5.6349
27262-48-2	Levobupivacaine HCl	-5.6347
27262-47-1	Levobupivacaine	-5.6347
18010-40-7	Bupivacaine hydrochloride	-5.6347
341-69-5	Orphenadrine hydrochloride	-5.6341
89226-75-5	Manidipine dihydrochloride	-5.6341
89226-50-6	Manidipine	-5.6335
808118-40-3	Roxadustat	-5.6328
64-75-5	Tetracycline hydrochloride	-5.6324
33386-08-2	Buspirone hydrochloride	-5.6323
163521-12-8	Vilazodone	-5.6323
73-48-3	Bendroflumethiazide	-5.6318
21736-83-4	Spectinomycin dihydrochloride	-5.6317
163521-12-8	Vilazodone	-5.6317
202189-78-4	Bilastine	-5.6317
439239-90-4	Lasmiditan	-5.6314

7512-17-6	N-Acetyl-D-Glucosamine	-5.631
25389-94-0	Kanamycin sulfate	-5.6308
73816-42-9	Meclocycline sulfosalicylate salt	-5.6307
111902-57-9	Temocapil	-5.6302
187235-37-6	Pretomanid	-5.6299
4394-00-7	Niflumic acid	-5.6299
1360460-82-7	Anlotinib Dihydrochloride	-5.6299
1058156-90-3	Anlotinib	-5.6299
179474-81-8	Prucalopride	-5.6299
202189-78-4	Bilastine	-5.6293
1405-10-3	Neomycin sulfate	-5.6292
532-03-6	Methocarbamol	-5.6289
54556-98-8	Propiverine hydrochloride	-5.6286
386750-22-7	Desvenlafaxine succinate hydrate	-5.6285
93413-62-8	Desvenlafaxine	-5.6285
147127-20-6	Tenofovir	-5.628
74103-07-4	Ketorolac tromethamine salt	-5.6278
57-37-4	Benactyzine hydrochloride	-5.6278
54556-98-8	Propiverine hydrochloride	-5.6271
161973-10-0	Esomeprazole Magnesium	-5.6266
31677-93-7	Bupropion hydrochloride	-5.6262
93479-97-1	Glimepiride	-5.6258
103639-04-9	Ondansetron hydrochloride	-5.6253
123524-52-7	Azelnidipine	-5.6252
1374640-70-6	Rociletinib	-5.6249
123663-49-0	Iguratimod	-5.624
2259-96-3	Cyclothiazide	-5.624
22254-24-6	Ipratropium Bromide	-5.6238
66985-17-9	Ipratropium bromide monohydrate	-5.6238
132-20-7	Pheniramine maleate	-5.6238
68099-86-5	Bepridil hydrochloride	-5.6238
375815-87-5	Varenicline Tartrate	-5.6234
83-67-0	Theobromine	-5.6229
2259-96-3	Cyclothiazide	-5.6223
122892-31-3	Itopride hydrochloride	-5.6217
4146-30-9	Neomycin Sulphate B	-5.6213
82586-55-8	Quinapril hydrochloride	-5.6211
123447-62-1	Prulifloxacin	-5.6209
3644-61-9	Tolperisone hydrochloride	-5.6208
123948-87-8	Topotecan	-5.6206

161973-10-0	Esomeprazole Magnesium	-5.6198
106308-44-5	Rufinamide	-5.6196
128794-94-5	Mycophenolate Mofetil	-5.6196
50-34-0	Propantheline bromide	-5.6193
85622-93-1	Temozolomide	-5.6192
60282-87-3	Gestodene	-5.619
51-30-9	Isoprenaline hydrochloride	-5.6186
179324-69-7	Bortezomib	-5.6182
24390-14-5	Doxycycline hyclate	-5.6171
477600-75-2	Tofacitinib	-5.6167
540737-29-9	Tofacitinib Citrate	-5.6167
320-67-2	5-Azacytidine	-5.6163
56211-40-6	Torasemide	-5.6163
84-80-0	Vitamin K1	-5.616
74863-84-6	Argatroban	-5.6158
58-93-5	Hydrochlorothiazide	-5.6145
1025504-45-3	Valbenazine	-5.6142
17696-69-4	Hematoporphyrin dihydrochloride	-5.614
518-28-5	Podophyllotoxin	-5.6139
22862-76-6	Anisomycin	-5.6138
341-69-5	Orphenadrine hydrochloride	-5.6137
54965-21-8	Albendazole	-5.6129
2753-45-9	Mebeverine hydrochloride	-5.6127
17696-69-4	Hematoporphyrin dihydrochloride	-5.6125
298-81-7	8-Methoxypsoralen	-5.612
22494-42-4	Diflunisal	-5.6112
157810-81-6	Indinavir sulfate	-5.6112
61-68-7	Mefenamic acid	-5.6109
6856-31-1	Loxeen	-5.6109
50-42-0	Adiphenine hydrochloride	-5.6101
1104-22-9	Meclizine dihydrochloride	-5.6098
53885-35-1	Ticlopidine hydrochloride	-5.6095
548-62-9	Crystal Violet	-5.6091
112885-42-4	Mosapride Citrate	-5.6087
1405-10-3	Neomycin sulfate	-5.6086
93106-60-6	Enrofloxacin	-5.6086
4146-30-9	Neomycin Sulphate B	-5.6084
91832-40-5	Cefdinir	-5.608
3963-95-9	Methacycline hydrochloride	-5.608
18010-40-7	Bupivacaine hydrochloride	-5.608
125-02-0	Prednisolone phosphate sodium	-5.6078
103-16-2	Monobenzone	-5.6077

179324-69-7	Bortezomib	-5.6076
58-15-1	Aminophenazone	-5.6074
456-59-7	Cyclandelate	-5.607
7327-87-9	Dihydralazine sulphate	-5.6069
1405-10-3	Neomycin sulfate	-5.6067
29608-49-9	Almitrine dimesylate	-5.6065
14222-60-7	Protionamide	-5.6062
122852-69-1	Alosetron hydrochloride	-5.6057
132036-88-5	Ramosetron	-5.6054
153-18-4	Rutin	-5.6053
360-70-3	Nandrolone decanoate	-5.6045
153-18-4	Rutin	-5.6041
6066-49-5	Butylphthalide	-5.6041
56839-43-1	Eperisone hydrochloride	-5.6034
31677-93-7	Bupropion hydrochloride	-5.6033
17696-69-4	Hematoporphyrin dihydrochloride	-5.6032
6385-02-0	Meclofenamate Sodium	-5.6021
6108-05-0	Lidocaine Hydrochloride hydrate	-5.6019
73-78-9	Lidocaine hydrochloride	-5.6019
157810-81-6	Indinavir sulfate	-5.6011
137-58-6	Xylocaine	-5.6004
827022-33-3	Palbociclib Isethionate	-5.5997
827022-32-2	Palbociclib hydrochloride	-5.5997
3717-88-2	Flavoxate hydrochloride	-5.5997
149-64-4	Scopolamine butylbromide	-5.5994
69558-55-0	Thymopentin	-5.5987
93106-60-6	Enrofloxacin	-5.5986
50-91-9	Floxuridine	-5.5986
103639-04-9	Ondansetron hydrochloride	-5.5978
104-29-0	Chlorphenesin	-5.5973
69-81-8	Carbazochrome	-5.5972
151533-22-1	Levomefolate calcium	-5.5972
571190-30-2	Palbociclib	-5.597
1226781-44-7	Omarigliptin	-5.5968
287714-41-4	Rosuvastatin	-5.5965
320345-99-1	Aclidinium bromide	-5.5965
72509-76-3	Felodipine	-5.5963
26159-34-2	Naproxen sodium	-5.5959
22204-53-1	Naproxen	-5.5959
83105-70-8	Sultamicillin Tosylate	-5.5956
879085-55-9	Vismodegib	-5.5954
103-90-2	Acetaminophen	-5.5951

84680-54-6	Enalaprilat Dihydrate	-5.595
2196-13-6	Thioisonicotinamide	-5.595
3963-95-9	Methacycline hydrochloride	-5.5949
136-77-6	Hexylresorcinol	-5.5947
83435-67-0	Delapril?Hydrochloride	-5.5945
52645-53-1	Permethrin	-5.5944
738-70-5	Trimethoprim	-5.5941
34580-14-8	Ketotifen fumarate	-5.5933
912-60-7	Noscapine hydrochloride	-5.5933
124858-35-1	Nadifloxacin	-5.5932
87233-61-2	Emedastine	-5.5928
84-80-0	Vitamin K1	-5.5922
130-40-5	Flavin mononucleotide	-5.5914
150683-30-0	Tolvaptan	-5.5911
50-24-8	Prednisolone	-5.5905
843663-66-1	Bedaquiline	-5.5903
147-24-0	Diphenhydramine hydrochloride	-5.5901
41294-56-8	Alfacalcidol	-5.5896
6151-40-2	Quinidine hydrochloride	-5.5895
83-07-8	4-Aminoantipyrine	-5.5894
523-87-5	Dimenhydrinate	-5.5892
38304-91-5	Minoxidil	-5.5891
54048-10-1	Etonogestrel	-5.5889
52645-53-1	Permethrin	-5.5888
103-16-2	Monobenzone	-5.5885
320345-99-1	Aclidinium bromide	-5.5885
100-51-6	Benzyl alcohol	-5.588
841301-32-4	Amenamevir	-5.5875
6533-00-2	Norgestrel	-5.5874
22373-78-0	Monensin sodium salt	-5.5871
150322-43-3	Prasugrel	-5.587
152459-95-5	Imatinib	-5.5863
220127-57-1	Imatinib Mesylate	-5.5863
426-13-1	Fluorometholone	-5.5862
71675-85-9	Amisulpride	-5.586
132810-10-7	Blonanserin	-5.586
79660-72-3	Fleroxacin	-5.586
4146-30-9	Neomycin Sulphate B	-5.5857
2078-54-8	Propofol	-5.5854
292135-78-5	Roflumilast N-oxide	-5.585
117976-90-6	Rebeprazole sodium	-5.5848
62613-82-5	Oxiracetam	-5.5848
6024-85-7	Tetrahydropalmatine hydrochloride	-5.5848

25913-34-2	Tinoridine hydrochloride	-5.5847
24729-96-2	Clindamycin phosphate	-5.5846
32986-56-4	Tobramycin	-5.5846
95635-55-5	Ranolazine	-5.5845
95635-56-6	Ranolazine dihydrochloride	-5.5845
69-81-8	Carbazochrome	-5.584
31430-15-6	Flubendazole	-5.584
178307-42-1	Revaprazan hydrochloride	-5.5836
71675-85-9	Amisulpride	-5.5833
292135-78-5	Roflumilast N-oxide	-5.5833
122647-32-9	Ibutilide Fumarate	-5.5831
1360460-82-7	Anlotinib Dihydrochloride	-5.5824
1058156-90-3	Anlotinib	-5.5824
120-51-4	Benzyl benzoate	-5.5823
850876-88-9	Danoprevir	-5.5822
85622-93-1	Temozolomide	-5.5817
2078-54-8	Propofol	-5.581
39809-25-1	Penciclovir	-5.5804
73816-42-9	Meclocycline sulfosalicylate salt	-5.5802
66085-59-4	Nimodipine	-5.5801
186826-86-8	Moxifloxacin hydrochloride	-5.58
90729-43-4	Ebastine	-5.5798
1104-22-9	Meclizine dihydrochloride	-5.5791
73-48-3	Bendroflumethiazide	-5.5783
4852-22-6	Proanthocyanidins	-5.5781
124858-35-1	Nadifloxacin	-5.5778
5104-49-4	Flurbiprofen	-5.5776
4429-63-4	Tabersonine	-5.5775
554-92-7	Trimethobenzamide hydrochloride	-5.5774
68099-86-5	Bepridil hydrochloride	-5.5772
11018-89-6	Ouabain octahydrate	-5.577
863127-77-9	Dasatinib monohydrate	-5.5769
33286-22-5	Diltiazem hydrochloride	-5.5768
161715-24-8	Tebipenem Pivoxil	-5.5766
148-01-6	Dinitolmide	-5.5763
56390-09-1	Epirubicin hydrochloride	-5.576
89396-94-1	Imidapril HCl	-5.5754
130773-02-3	Neticonazole Hydrochloride	-5.5753
100-51-6	Benzyl alcohol	-5.5751
1104-22-9	Meclizine dihydrochloride	-5.5745
633-65-8	Berberine hydrochloride	-5.5745

1722-62-9	Mepivacaine hydrochloride	-5.5744
99300-78-4	Venlafaxine hydrochloride	-5.5743
175865-59-5	Valganciclovir hydrochloride	-5.5741
31282-04-9	Hygromycin B	-5.5741
4429-63-4	Tabersonine	-5.574
111974-72-2	Quetiapine Fumarate	-5.5736
120-08-1	Scoparone	-5.573
1405-10-3	Neomycin sulfate	-5.573
15686-71-2	Cephalexin	-5.5729
2078-54-8	Propofol	-5.5728
62-68-0	Proadifen hydrochloride	-5.5728
64-75-5	Tetracycline hydrochloride	-5.5727
946075-13-4	Isavuconazonium sulfate	-5.5724
80-49-9	Homatropine Methylbromide	-5.5719
864750-70-9	Revefenacin	-5.5712
839712-12-8	Cariprazine	-5.5711
75695-93-1	Isradipine	-5.5706
864750-70-9	Revefenacin	-5.5703
90-80-2	Delta-Gluconolactone	-5.5702
138890-62-7	Brinzolamide	-5.5699
382-67-2	Desoximetasone	-5.5698
120410-24-4	Biapenem	-5.5697
72509-76-3	Felodipine	-5.5694
31430-15-6	Flubendazole	-5.5692
120014-06-4	Donepezil	-5.569
51012-33-0	Tiapride hydrochloride	-5.5689
136310-93-5	Tiotropium bromide	-5.5687
120011-70-3	Donepezil HCl	-5.5681
25451-15-4	Felbamate	-5.5671
35212-22-7	Ipriflavone	-5.5669
66-97-7	Psoralen	-5.5662
123040-16-4	Azasetron hydrochloride	-5.5652
111902-57-9	Temocapil	-5.5647
72496-41-4	Pirarubicin	-5.5647
5630-53-5	Tibolone	-5.5642
31677-93-7	Bupropion hydrochloride	-5.5637
110221-44-8	Temocapril HCl	-5.5635
91503-79-6	Flurbiprofen Axetil	-5.5634
5189-11-7	Pizotifen Malate	-5.5632
873054-44-5	Ivacaftor	-5.5629
651-06-9	Sulfameter	-5.5628
914462-92-3	Rolapitant hydrochloride	-5.5627
15574-96-6	Pizotifen	-5.5626

78628-80-5	Terbinafine hydrochloride	-5.5625
6004-24-6	Cetylpyridinium chloride monohydrate	-5.5621
157810-81-6	Indinavir sulfate	-5.5619
80-77-3	Chlormezanone	-5.5617
2353-33-5	Decitabine	-5.5616
115256-11-6	Dofetilide	-5.5613
1163-36-6	Clemizole hydrochloride	-5.5612
123040-16-4	Azasetron hydrochloride	-5.5611
112885-42-4	Mosapride Citrate	-5.5602
31677-93-7	Bupropion hydrochloride	-5.5595
3094-09-5	Doxifluridine	-5.5595
207844-01-7	Mitiglinide calcium	-5.5594
101827-46-7	Butenafine hydrochloride	-5.5592
106730-54-5	Olprinone	-5.5585
832720-36-2	Elagolix sodium	-5.558
1025967-78-5	Lifitegrast	-5.5571
38083-17-9	Climbazole	-5.5568
178429-62-4	solriamfetol	-5.5565
53716-49-7	Carprofen	-5.5563
526-08-9	Sulfaphenazole	-5.5562
64872-77-1	Butoconazole nitrate	-5.5556
5104-49-4	Flurbiprofen	-5.5549
69-81-8	Carbazochrome	-5.5542
55-48-1	Atropine sulfate	-5.5535
5908-99-6	Atropine sulfate monohydrate	-5.5535
51-55-8	Atropine	-5.5535
161973-10-0	Esomeprazole Magnesium	-5.5535
164579-32-2	Pantoprazole Sodium Hydrate	-5.5533
26864-56-2	Penfluridol	-5.5532
15622-65-8	Molindone hydrochloride	-5.5532
101-31-5	L-Hyoscyamine	-5.5532
1354744-91-4	Naloxegol oxalate	-5.5532
140-64-7	Pentamidine isethionate	-5.553
69-81-8	Carbazochrome	-5.5529
122320-73-4	Rosiglitazone	-5.5526
37091-65-9	Azlocillin sodium	-5.5521
157810-81-6	Indinavir sulfate	-5.552
341-69-5	Orphenadrine hydrochloride	-5.5512
1722-62-9	Mepivacaine hydrochloride	-5.5507
70356-03-5	Cefaclor monohydrate	-5.5506
55-48-1	Atropine sulfate	-5.5503

5908-99-6	Atropine sulfate monohydrate	-5.5503
51-55-8	Atropine	-5.5503
6066-49-5	Butylphthalide	-5.5496
124858-35-1	Nadifloxacin	-5.5495
72-33-3	Mestranol	-5.5495
5104-49-4	Flurbiprofen	-5.5493
111902-57-9	Temocapil	-5.5491
95233-18-4	Atovaquone	-5.5487
162359-56-0	Fingolimod hydrochloride	-5.5487
175865-59-5	Valganciclovir hydrochloride	-5.5486
1146-99-2	chlophenadione	-5.5479
304-20-1	Hydralazine hydrochloride	-5.5478
115256-11-6	Dofetilide	-5.5476
1405-10-3	Neomycin sulfate	-5.5474
136310-93-5	Tiotropium bromide	-5.5471
122852-69-1	Alosetron hydrochloride	-5.5468
151533-22-1	Levomefolate calcium	-5.5462
6066-49-5	Butylphthalide	-5.5461
71675-85-9	Amisulpride	-5.546
11018-89-6	Ouabain octahydrate	-5.5459
130773-02-3	Neticonazole Hydrochloride	-5.5458
1051375-19-9	Dolutegravir sodium	-5.5457
53885-35-1	Ticlopidine hydrochloride	-5.5454
177834-92-3	Eletriptan HBr	-5.5449
83-43-2	Methylprednisolone	-5.5439
83881-52-1	Cetirizine dihydrochloride	-5.5433
19408-46-9	Kasugamycin hydrochloride	-5.5432
1744-22-5	Riluzole	-5.5432
95789-30-3	Cefotiam Hexetil Hydrochloride	-5.5432
17696-69-4	Hematoporphyrin dihydrochloride	-5.5431
6151-40-2	Quinidine hydrochloride	-5.543
68-22-4	Norethindrone	-5.543
721-50-6	Prilocaine	-5.5427
1786-81-8	Propitocaine hydrochloride	-5.5427
121123-17-9	Cefprozil hydrate	-5.5425
773-76-2	Capitrol	-5.5423
130656-51-8	Nifekalant hydrochloride	-5.5422
122320-73-4	Rosiglitazone	-5.5421
59-33-6	Pyrilamine maleate	-5.5416
88671-89-0	Myclobutanil	-5.5412
54527-84-3	Nicardipine hydrochloride	-5.5411
87-89-8	Inositol	-5.5404

184475-35-2	Gefitinib	-5.5402
147084-10-4	Alcaftadine	-5.5401
1098-97-1	Pyrithioxin	-5.54
50847-11-5	Ibudilast	-5.5399
4146-30-9	Neomycin Sulphate B	-5.5398
128607-22-7	Ospemifene	-5.5395
827022-33-3	Palbociclib Isethionate	-5.5392
827022-32-2	Palbociclib hydrochloride	-5.5392
65513-72-6	Glafenine Hydrochloride	-5.5389
3820-67-5	Glafenine	-5.5389
3485-62-9	Clidinium bromide	-5.5386
804-30-8	Fursultiamine	-5.5385
99300-78-4	Venlafaxine hydrochloride	-5.5383
73816-42-9	Meclocycline sulfosalicylate salt	-5.5383
63-45-6	Primaquine diphosphate	-5.5379
157810-81-6	Indinavir sulfate	-5.5379
980-71-2	Brompheniramine hydrogen maleate	-5.5373
681492-22-8	Delamanid	-5.5365
14222-60-7	Protionamide	-5.5355
698387-09-6	Neratinib	-5.5355
571190-30-2	Palbociclib	-5.5347
79660-72-3	Fleroxacin	-5.5343
138452-21-8	Fexofenadine hydrochloride	-5.5338
242478-38-2	Solifenacin succinate	-5.5337
111902-57-9	Temocapil	-5.5332
402957-28-2	Telaprevir	-5.5324
341-69-5	Orphenadrine hydrochloride	-5.5323
389-08-2	Nalidixic acid	-5.5323
912-60-7	Noscapine hydrochloride	-5.5323
104-29-0	Chlorphenesin	-5.5319
36330-85-5	Fenbufen	-5.5317
3374-05-8	Nalidixic acid sodium salt	-5.5317
125-02-0	Prednisolone phosphate sodium	-5.5314
59729-37-2	Fexinidazole	-5.5313
3166-62-9	Methylbenactyzine Bromide	-5.5313
52-21-1	Prednisolone-21-acetate	-5.5309
7481-89-2	Zalcitabine	-5.5307
29342-05-0	Ciclopirox	-5.5307
41621-49-2	Ciclopirox ethanolamine	-5.5307
56839-43-1	Eperisone hydrochloride	-5.5305
121-33-5	Vanillin	-5.5299
755037-03-7	Regorafenib	-5.5292
132036-88-5	Ramosetron	-5.5291

150683-30-0	Tolvaptan	-5.5289
1508-75-4	Tropicamide	-5.5284
29908-03-0	Ademetionine	-5.5282
956104-40-8	Apalutamide	-5.5278
1953-04-4	Galanthamine HBr	-5.5276
130-40-5	Flavin mononucleotide	-5.5276
52549-17-4	Pranoprofen	-5.5272
138452-21-8	Fexofenadine hydrochloride	-5.5267
150683-30-0	Tolvaptan	-5.5266
78613-38-4	Amorolfine hydrochloride	-5.5264
70458-95-6	Pefloxacin Mesylate	-5.5262
22254-24-6	Ipratropium Bromide	-5.5261
66985-17-9	Ipratropium bromide monohydrate	-5.5261
74711-43-6	Zaltoprofen	-5.5259
26864-56-2	Penfluridol	-5.5255
149676-40-4	Pefloxacin Mesylate Dihydrate	-5.525
83-43-2	Methylprednisolone	-5.525
906093-29-6	Teneligliptin hydrobromide	-5.5245
78246-49-8	Paroxetine hydrochloride	-5.5244
304-20-1	Hydralazine hydrochloride	-5.5244
6004-24-6	Cetylpyridinium chloride monohydrate	-5.5243
1134-47-0	Baclofen	-5.5237
88889-14-9	Fosinopril sodium	-5.5235
161973-10-0	Esomeprazole Magnesium	-5.5234
153-18-4	Rutin	-5.5234
61-16-5	Methoxamine hydrochloride	-5.5233
6170-42-9	Chloropyramine hydrochloride	-5.5228
386750-22-7	Desvenlafaxine succinate hydrate	-5.5228
93413-62-8	Desvenlafaxine	-5.5228
130773-02-3	Neticonazole Hydrochloride	-5.5223
859-18-7	Lincomycin hydrochloride	-5.5219
151533-22-1	Levomefolate calcium	-5.5214
91503-79-6	Flurbiprofen Axetil	-5.5214
69308-37-8	(R)-baclofen	-5.5213
115256-11-6	Dofetilide	-5.521
3200-06-4	Nafronyl oxalate	-5.521
161973-10-0	Esomeprazole Magnesium	-5.5203
69-81-8	Carbazochrome	-5.5202
91161-71-6	Terbinafine	-5.5198
54556-98-8	Propiverine hydrochloride	-5.5194
548-66-3	Drofenine hydrochloride	-5.519
120068-37-3	Fipronil	-5.5187
7179-49-9	Lincomycin hydrochloride monohydrate	-5.5186

89226-75-5	Manidipine dihydrochloride	-5.518
554-92-7	Trimethobenzamide hydrochloride	-5.5177
83105-70-8	Sultamicillin Tosylate	-5.5173
31677-93-7	Bupropion hydrochloride	-5.5169
171228-49-2	Posaconazole	-5.5167
134678-17-4	Lamivudine	-5.5163
1289023-67-1	BMS927711	-5.5163
968-81-0	Acetohexamide	-5.5162
140-40-9	Nithiamide	-5.5158
456-59-7	Cyclandelate	-5.5155
24316-19-6	Cephalotaxine	-5.5154
302962-49-8	Dasatinib	-5.5154
577778-58-6	Topiroxostat	-5.5149
33564-30-6	Cefoxitin sodium	-5.5148
238750-77-1	Tosedostat	-5.5147
132-98-9	Penicillin V potassium salt	-5.5146
127373-66-4	Sivelestat	-5.5145
94-07-5	Synephrine	-5.5145
89226-50-6	Manidipine	-5.5144
112885-42-4	Mosapride Citrate	-5.5142
14919-77-8	Benserazide hydrochloride	-5.5141
173352-39-1	Afobazole hydrochloride	-5.5135
69304-47-8	Brivudine	-5.5133
59-33-6	Pyrilamine maleate	-5.5129
64-72-2	Chlortetracycline hydrochloride	-5.5129
99300-78-4	Venlafaxine hydrochloride	-5.5126
356-12-7	Fluocinonide	-5.5125
94-07-5	Synephrine	-5.5124
173352-39-1	Afobazole hydrochloride	-5.5123
101-31-5	L-Hyoscyamine	-5.5121
10212-25-6	Cyclocytidine hydrochloride	-5.512
1953-04-4	Galanthamine HBr	-5.5119
55-48-1	Atropine sulfate	-5.51
5908-99-6	Atropine sulfate monohydrate	-5.51
51-55-8	Atropine	-5.51
24390-14-5	Doxycycline hyclate	-5.5097
1263-89-4	Paromomycin Sulfate	-5.5094
31282-04-9	Hygromycin B	-5.5076
583-03-9	Fenipentol	-5.5076
1195765-45-7	Dabrafenib	-5.5074
79660-72-3	Fleroxacin	-5.5068

120014-06-4	Donepezil	-5.5067
120011-70-3	Donepezil HCl	-5.5067
66898-62-2	Talniflumate	-5.5067
66-84-2	Glucosamine hydrochloride	-5.5066
112811-59-3	Gatifloxacin	-5.5064
171599-83-0	Sildenafil citrate	-5.5062
24729-96-2	Clindamycin phosphate	-5.5061
157810-81-6	Indinavir sulfate	-5.506
59-92-7	Levodopa	-5.506
53716-49-7	Carprofen	-5.506
123447-62-1	Prulifloxacin	-5.5059
60-80-0	Antipyrine	-5.5055
65391-42-6	Bestatin hydrochloride	-5.5052
58970-76-6	Ubenimex	-5.5052
81938-43-4	Zofenopril (calcium)	-5.5052
41340-25-4	Etodolac	-5.5049
81025-04-9	Lactitol monohydrate	-5.5048
2353-33-5	Decitabine	-5.5044
1448347-49-6	Ivosidenib	-5.5043
144-83-2	Sulfapyridine	-5.5041
107007-99-8	Granisetron hydrochloride	-5.5039
74711-43-6	Zaltoprofen	-5.503
66309-69-1	Cefotiam hydrochloride	-5.5029
175865-59-5	Valganciclovir hydrochloride	-5.5029
132-20-7	Pheniramine maleate	-5.5028
57808-66-9	Domperidone	-5.5027
155270-99-8	Istradefylline	-5.5023
14919-77-8	Benserazide hydrochloride	-5.5019
58947-95-8	DL-trihexyphenidyl hydrochloride	-5.5019
101477-54-7	Lomerizine hydrochloride	-5.5017
150-76-5	4-Methoxyphenol	-5.5014
127-71-9	Sulfabenzamide	-5.5009
130-37-0	Menadione sodium bisulfite	-5.5008
91-33-8	Benzthiazide	-5.5004
130-37-0	Menadione sodium bisulfite	-5.5004
93479-97-1	Glimepiride	-5.5003
103-90-2	Acetaminophen	-5.5001
112885-42-4	Mosapride Citrate	-5.5
152-43-2	Quinestrol	-5.4998
22254-24-6	Ipratropium Bromide	-5.4995
66985-17-9	Ipratropium bromide monohydrate	-5.4995
1722-62-9	Mepivacaine hydrochloride	-5.4994

5104-49-4	Flurbiprofen	-5.4993
1490-04-6	DL-Menthol	-5.499
55-48-1	Atropine sulfate	-5.4985
5908-99-6	Atropine sulfate monohydrate	-5.4985
51-55-8	Atropine	-5.4985
54556-98-8	Propiverine hydrochloride	-5.4982
22832-87-7	Miconazole nitrate	-5.4982
136-77-6	Hexylresorcinol	-5.4978
27741-01-1	Geniposidic Acid	-5.4978
6106-81-6	Scopolamine N-oxide HBr	-5.4976
53746-45-5	Fenoprofen calcium dihydrate	-5.4974
67227-57-0	Fenoldopam mesylate	-5.4973
98-92-0	Nicotinamide	-5.4971
14919-77-8	Benserazide hydrochloride	-5.497
1108743-60-7	Entrectinib	-5.4967
138890-62-7	Brinzolamide	-5.4966
149-91-7	Gallic acid	-5.4961
202189-78-4	Bilastine	-5.4961
132-20-7	Pheniramine maleate	-5.4959
50370-12-2	Cefadroxil	-5.4957
91-64-5	Coumarin	-5.4955
56-69-9	5-hydroxytryptophan	-5.4953
521-78-8	Trimipramine maleate	-5.495
87233-61-2	Emedastine	-5.495
28395-03-1	Bumetanide	-5.4949
149-91-7	Gallic acid	-5.4948
1490-04-6	DL-Menthol	-5.4938
57808-65-8	Closantel	-5.4937
51-41-2	Norepinephrine	-5.4936
69815-49-2	Noradrenaline bitartrate	-5.4936
53746-45-5	Fenoprofen calcium dihydrate	-5.4935
964-52-3	Moxisylyte hydrochloride	-5.4929
5728-52-9	4-Biphenylacetic acid	-5.4926
121-33-5	Vanillin	-5.4926
491833-29-5	Eliglustat	-5.4926
29908-03-0	Ademetionine	-5.4917
138452-21-8	Fexofenadine hydrochloride	-5.4917
150683-30-0	Tolvaptan	-5.491
14919-77-8	Benserazide hydrochloride	-5.4908
59-30-3	Folic acid	-5.4902
3737-09-5	Disopyramide	-5.4901
92-61-5	Scopoletin	-5.4893

24729-96-2	Clindamycin phosphate	-5.4893
12650-69-0	Mupirocin	-5.4891
66898-62-2	Talniflumate	-5.4889
155-41-9	Methscopolamine	-5.4887
62-68-0	Proadifen hydrochloride	-5.4883
112885-42-4	Mosapride Citrate	-5.4877
7481-89-2	Zalcitabine	-5.4875
3011-89-0	Alkomide	-5.4871
841301-32-4	Amenamevir	-5.487
94-07-5	Synephrine	-5.4865
1722-62-9	Mepivacaine hydrochloride	-5.4865
1490-04-6	DL-Menthol	-5.4864
618385-01-6	Vorapaxar	-5.4863
3759-92-0	Furaltadone hydrochloride	-5.4862
1051375-16-6	Dolutegravir	-5.486
27848-84-6	Nicergoline	-5.4857
537-12-2	Diperodon hydrochloride	-5.4856
72496-41-4	Pirarubicin	-5.4853
38821-49-7	Carbidopa Hydrate	-5.485
21736-83-4	Spectinomycin dihydrochloride	-5.485
120202-66-6	(S)-(+)-Clopidogrel hydrogen sulfate	-5.4849
878143-33-0	Dalasetron Mesylate Hydrate	-5.4846
651-06-9	Sulfameter	-5.4845
3543-75-7	Bendamustine hydrochloride	-5.4845
120068-37-3	Fipronil	-5.4839
22832-87-7	Miconazole nitrate	-5.4839
1051375-19-9	Dolutegravir sodium	-5.4839
583-03-9	Fenipentol	-5.4838
1163-36-6	Clemizole hydrochloride	-5.4838
1025967-78-5	Lifitegrast	-5.4837
500-64-1	L-KAWAIN	-5.4826
13710-19-5	Tolfenamic Acid	-5.4826
915087-33-1	Enzalutamide	-5.4825
155-41-9	Methscopolamine	-5.4823
23979-41-1	(R)-Naproxen	-5.4818
4852-22-6	Proanthocyanidins	-5.4817
3254-89-5	Diphenidol hydrochloride	-5.4815
202189-78-4	Bilastine	-5.4815
112885-42-4	Mosapride Citrate	-5.4812
120068-37-3	Fipronil	-5.4807

154229-18-2	Abiraterone Acetate	-5.4806
1025504-45-3	Valbenazine	-5.4805
946075-13-4	Isavuconazonium sulfate	-5.4805
41340-25-4	Etodolac	-5.4803
292135-78-5	Roflumilast N-oxide	-5.4803
52549-17-4	Pranoprofen	-5.4802
1051375-19-9	Dolutegravir sodium	-5.4801
456-59-7	Cyclandelate	-5.4801
83-43-2	Methylprednisolone	-5.48
532-03-6	Methocarbamol	-5.4796
914462-92-3	Rolapitant hydrochloride	-5.4789
91161-71-6	Terbinafine	-5.4787
7491-74-9	Piracetam	-5.4786
304-20-1	Hydralazine hydrochloride	-5.4779
1104-22-9	Meclizine dihydrochloride	-5.4778
1072833-77-2	Ixazomib	-5.4777
2259-96-3	Cyclothiazide	-5.4776
126544-47-6	Ciclesonide	-5.4772
184475-35-2	Gefitinib	-5.4758
173352-39-1	Afobazole hydrochloride	-5.4756
22254-24-6	Ipratropium Bromide	-5.4752
66985-17-9	Ipratropium bromide monohydrate	-5.4752
62-31-7	Dopamine hydrochloride	-5.4743
124832-27-5	Valacyclovir hydrochloride	-5.4737
3094-09-5	Doxifluridine	-5.4737
94-07-5	Synephrine	-5.4729
53797-35-6	Ribostamycin sulfate	-5.4728
14698-29-4	Oxolinic acid	-5.4727
167465-36-3	Zosuquidar 3HCl	-5.4725
73151-29-8	Fenticonazole Nitrate	-5.4725
3963-95-9	Methacycline hydrochloride	-5.4723
26864-56-2	Penfluridol	-5.4723
4852-22-6	Proanthocyanidins	-5.4722
943-17-9	Etilefrine hydrochloride	-5.4722
173352-39-1	Afobazole hydrochloride	-5.472
30123-17-2	Tianeptine sodium	-5.4719
90729-43-4	Ebastine	-5.4718
364-98-7	Diazoxide	-5.4716
797-63-7	Levonorgestrel	-5.4716
64953-12-4	Latamoxef sodium	-5.4715

147-24-0	Diphenhydramine hydrochloride	-5.4709
523-87-5	Dimenhydrinate	-5.4706
141-94-6	Hexetidine	-5.4702
52205-73-9	Estramustine phosphate sodium	-5.4701
4852-22-6	Proanthocyanidins	-5.4697
4852-22-6	Proanthocyanidins	-5.4697
1405-37-4	Capastat sulfate	-5.4693
705260-08-8	Vorapaxar sulfate	-5.469
123441-03-2	Rivastigmine	-5.4688
53716-49-7	Carprofen	-5.4687
129101-54-8	Rivastigmine tartrate	-5.4682
58-46-8	Tetrabenazine	-5.4681
22994-85-0	Benznidazole	-5.4681
27591-69-1	Tilorone dihydrochloride	-5.4681
52-21-1	Prednisolone-21-acetate	-5.468
50-24-8	Prednisolone	-5.4677
532-11-6	Anethole trithione	-5.4677
87233-61-2	Emedastine	-5.4674
6620-60-6	Proglumide	-5.4674
85721-33-1	Ciprofloxacin	-5.4673
93107-08-5	Ciprofloxacin hydrochloride	-5.4673
89778-26-7	Toremifene	-5.4672
55-48-1	Atropine sulfate	-5.4668
5908-99-6	Atropine sulfate monohydrate	-5.4668
51-55-8	Atropine	-5.4668
89778-27-8	Toremifene citrate	-5.4666
832720-36-2	Elagolix sodium	-5.4663
52645-53-1	Permethrin	-5.4661
132-17-2	Benztropine mesylate	-5.4659
191217-81-9	Pramipexole 2HCl Monohydrate	-5.4658
1072833-77-2	Ixazomib	-5.4655
110221-44-8	Temocapril HCl	-5.4651
2196-13-6	Thioisonicotinamide	-5.4641
84371-65-3	Mifepristone	-5.4635
30652-11-0	Deferidone	-5.4634
1405-10-3	Neomycin sulfate	-5.4633
1163-36-6	Clemizole hydrochloride	-5.4631
112885-42-4	Mosapride Citrate	-5.4627
29767-20-2	Teniposide	-5.4626
107007-99-8	Granisetron hydrochloride	-5.4623
111902-57-9	Temocapil	-5.4618
50370-12-2	Cefadroxil	-5.4617

2139-47-1	Nifenazone	-5.4617
775304-57-9	Ataluren	-5.4616
15421-84-8	Trapidil	-5.4613
21736-83-4	Spectinomycin dihydrochloride	-5.4608
122320-73-4	Rosiglitazone	-5.4607
738-70-5	Trimethoprim	-5.4603
161973-10-0	Esomeprazole Magnesium	-5.46
63-45-6	Primaquine diphosphate	-5.4598
548-66-3	Drofenine hydrochloride	-5.4598
90729-43-4	Ebastine	-5.4596
157810-81-6	Indinavir sulfate	-5.4594
24316-19-6	Cephalotaxine	-5.4592
63612-50-0	Nilutamide	-5.4589
90-80-2	Delta-Gluconolactone	-5.4582
132810-10-7	Blonanserin	-5.4581
83-67-0	Theobromine	-5.4577
129-06-6	Warfarin sodium	-5.4573
456-59-7	Cyclandelate	-5.4569
129938-20-1	Dapoxetine HCl	-5.4563
153-18-4	Rutin	-5.4559
53797-35-6	Ribostamycin sulfate	-5.4555
61618-27-7	Amfenac Sodium Monohydrate	-5.4549
19428-14-9	Benproperine phosphate	-5.4547
91161-71-6	Terbinafine	-5.4546
112885-42-4	Mosapride Citrate	-5.4546
69004-03-1	Toltrazuril	-5.4545
123447-62-1	Prulifloxacin	-5.4544
60282-87-3	Gestodene	-5.4543
4146-30-9	Neomycin Sulphate B	-5.4542
618385-01-6	Vorapaxar	-5.454
618385-01-6	Vorapaxar	-5.4539
71-81-8	Isopropamide Iodide	-5.4532
1847-24-1	Flucloxacillin sodium	-5.4524
6968-72-5	Mepiroxol	-5.4522
60-18-4	Tyrosine	-5.4522
138452-21-8	Fexofenadine hydrochloride	-5.4519
5633-20-5	Oxybutynin	-5.4515
1508-65-2	Oxybutynin hydrochloride	-5.4515
6533-68-2	Scopolamine HBr trihydrate	-5.4509
577778-58-6	Topiroxostat	-5.4508
477600-75-2	Tofacitinib	-5.4505
540737-29-9	Tofacitinib Citrate	-5.4505
23327-57-3	Nefopam hydrochloride	-5.45

60-80-0	Antipyrine	-5.4499
19237-84-4	Prazosin hydrochloride	-5.4499
554-92-7	Trimethobenzamide hydrochloride	-5.4497
67392-87-4	Drospirenone	-5.4497
82419-36-1	Ofloxacin	-5.4496
133-67-5	Trichlormethiazide	-5.4495
54048-10-1	Etonogestrel	-5.449
101-31-5	L-Hyoscyamine	-5.449
74103-07-4	Ketorolac tromethamine salt	-5.4486
3374-05-8	Nalidixic acid sodium salt	-5.4485
389-08-2	Nalidixic acid	-5.4485
55-48-1	Atropine sulfate	-5.4484
5908-99-6	Atropine sulfate monohydrate	-5.4484
51-55-8	Atropine	-5.4484
96847-55-1	Levomilnacipran	-5.4482
51037-30-0	Acipimox	-5.4481
82586-55-8	Quinapril hydrochloride	-5.4481
901119-35-5	Fostamatinib	-5.4475
83881-52-1	Cetirizine dihydrochloride	-5.4473
914462-92-3	Rolapitant hydrochloride	-5.447
132-18-3	Diphenylpyraline hydrochloride	-5.447
2557-49-5	Diflorasone	-5.4466
50264-69-2	Lonidamine	-5.4462
92636-39-0	Cefminox Sodium	-5.4459
50-42-0	Adiphenine hydrochloride	-5.4458
863-61-6	Menaquinone-4	-5.4458
11032-49-8	Menatetrenone	-5.4458
10402-53-6	Eprazinone dihydrochloride	-5.4445
7327-87-9	Dihydralazine sulphate	-5.4444
53251-94-8	Pinaverium bromide	-5.4444
32854-75-4	Lappaconitine	-5.4441
26095-59-0	Otilonium bromide	-5.4437
322-79-2	Triflusal	-5.4432
147127-20-6	Tenofovir	-5.4426
90-22-2	Valethamate bromide	-5.4426
189279-58-1	Delafloxacin	-5.4425
59-05-2	Methotrexate	-5.4424
6856-31-1	Loxeen	-5.4423
86541-74-4	Benazepril hydrochloride	-5.4422
89226-50-6	Manidipine	-5.4422
621-72-7	Bendazol	-5.4422
98-92-0	Nicotinamide	-5.4421
30751-05-4	Troxipide	-5.4415

89226-75-5	Manidipine dihydrochloride	-5.4407
1051375-19-9	Dolutegravir sodium	-5.4405
150322-43-3	Prasugrel	-5.4405
13412-64-1	Dicloxacillin sodium monohydrate	-5.4405
548-66-3	Drofenine hydrochloride	-5.4404
33125-97-2	Etomidate	-5.4402
59338-87-3	Alizapride hydrochloride	-5.4401
136-77-6	Hexylresorcinol	-5.44
50-03-3	Hydrocortisone acetate	-5.44
178307-42-1	Revaprazan hydrochloride	-5.4399
125-02-0	Prednisolone phosphate sodium	-5.4398
23979-41-1	(R)-Naproxen	-5.4395
183319-69-9	Erlotinib hydrochloride	-5.4393
183321-74-6	Erlotinib	-5.4393
58947-95-8	DL-trihexyphenidyl hydrochloride	-5.4389
38083-17-9	Climbazole	-5.4383
58-94-6	Chlorothiazide	-5.4376
6170-42-9	Chloropyramine hydrochloride	-5.4374
17560-51-9	Metolazone	-5.4373
201530-41-8	Deferasirox	-5.4369
61-16-5	Methoxamine hydrochloride	-5.4368
749-02-0	Spiperone	-5.4368
95789-30-3	Cefotiam Hexetil Hydrochloride	-5.4368
68291-97-4	Zonisamide	-5.4363
87-89-8	Inositol	-5.4362
86541-74-4	Benazepril hydrochloride	-5.4362
175865-59-5	Valganciclovir hydrochloride	-5.436
55981-09-4	Nitazoxanide	-5.4357
53-86-1	Indometacin	-5.4354
1025687-58-4	R788 Disodium	-5.4346
611-75-6	Bromhexine hydrochloride	-5.4346
164579-32-2	Pantoprazole Sodium Hydrate	-5.4343
53-86-1	Indometacin	-5.434
60628-96-8	Bifonazole	-5.4336
133-67-5	Trichlormethiazide	-5.4326
15622-65-8	Molindone hydrochloride	-5.4326
91161-71-6	Terbinafine	-5.4325
705260-08-8	Vorapaxar sulfate	-5.4317
641571-10-0	Nilotinib	-5.4315
52-21-1	Prednisolone-21-acetate	-5.4311
149-64-4	Scopolamine butylbromide	-5.4311

36330-85-5	Fenbufen	-5.4306
189279-58-1	Delafloxacin	-5.4305
72-14-0	Sulfathiazole	-5.4298
31430-15-6	Flubendazole	-5.4292
1949-20-8	Oxolamine citrate	-5.4291
3644-61-9	Tolperisone hydrochloride	-5.4285
98079-52-8	Lomefloxacin hydrochloride	-5.4284
98079-51-7	Lomefloxacin	-5.4284
17696-69-4	Hematoporphyrin dihydrochloride	-5.4282
10402-53-6	Eprazinone dihydrochloride	-5.4281
111902-57-9	Temocapil	-5.4279
3818-50-6	Bephenium Hydroxynaphthoate	-5.4279
1448347-49-6	Ivosidenib	-5.4275
1218779-75-9	Apatinib mesylate	-5.4273
811803-05-1	Apatinib	-5.4273
841301-32-4	Amenamevir	-5.4266
134308-13-7	Tolcapone	-5.4264
54048-10-1	Etonogestrel	-5.4262
50847-11-5	Ibudilast	-5.4261
88671-89-0	Myclobutanil	-5.426
58-46-8	Tetrabenazine	-5.426
804-30-8	Fursultiamine	-5.4254
101-31-5	L-Hyoscyamine	-5.4253
78613-38-4	Amorolfine hydrochloride	-5.4251
57-68-1	Sulfamethazine	-5.4249
132-20-7	Pheniramine maleate	-5.4249
36330-85-5	Fenbufen	-5.4247
55-48-1	Atropine sulfate	-5.4241
5908-99-6	Atropine sulfate monohydrate	-5.4241
51-55-8	Atropine	-5.4241
18010-40-7	Bupivacaine hydrochloride	-5.4237
946075-13-4	Isavuconazonium sulfate	-5.4228
51460-26-5	Carbazochrome sodium sulfonate	-5.4228
456-59-7	Cyclandelate	-5.4223
15421-84-8	Trapidil	-5.422
1346242-81-6	Erdafitinib	-5.4219
3963-95-9	Methacycline hydrochloride	-5.4218
100-55-0	Roniacol	-5.4216
618385-01-6	Vorapaxar	-5.4215
141625-93-6	Dronedarone hydrochloride	-5.421

2353-33-5	Decitabine	-5.4205
141626-36-0	Dronedarone	-5.4204
116-38-1	Edrophonium chloride	-5.4204
5370-01-4	Mexiletine hydrochloride	-5.4203
106730-54-5	Olprinone	-5.42
32986-56-4	Tobramycin	-5.4196
6024-85-7	Tetrahydropalmatine hydrochloride	-5.4193
T5164	Cabozantinib hydrochloride (849217-68-1(free base))	-5.419
849217-68-1	Cabozantinib	-5.419
1140909-48-3	Cabozantinib Malate	-5.419
137-88-2	Amprolium Hydrochloride	-5.4187
60-93-5	Quinine dihydrochloride	-5.4187
130-95-0	Quinine	-5.4187
70-70-2	Vanatone	-5.418
3858-89-7	Chloroprocaine HCl	-5.4178
1032900-25-6	LDK378	-5.4175
23327-57-3	Nefopam hydrochloride	-5.4173
98769-84-7	Reboxetine mesylate	-5.4172
706782-28-7	Pimavanserin tartrate	-5.417
706779-91-1	Pimavanserin	-5.417
23651-95-8	Droxidopa	-5.4169
31637-97-5	Etofibrate	-5.4166
24729-96-2	Clindamycin phosphate	-5.4164
80-49-9	Homatropine Methylbromide	-5.4164
162359-56-0	Fingolimod hydrochloride	-5.4161
5728-52-9	4-Biphenylacetic acid	-5.4159
53746-45-5	Fenoprofen calcium dihydrate	-5.4159
238750-77-1	Tosedostat	-5.4157
1098-97-1	Pyrithioxin	-5.4153
17696-69-4	Hematoporphyrin dihydrochloride	-5.4153
115956-12-2	Dolasetron	-5.4153
153559-49-0	Bexarotene	-5.4145
14605-22-2	Tauroursodeoxycholic Acid	-5.4138
22071-15-4	Ketoprofen	-5.4128
5370-01-4	Mexiletine hydrochloride	-5.4126
3963-95-9	Methacycline hydrochloride	-5.4126
6024-85-7	Tetrahydropalmatine hydrochloride	-5.4126
81840-15-5	Vesnarinone	-5.4124
69-81-8	Carbazochrome	-5.4123
57-96-5	Sulfinpyrazone	-5.4123
22994-85-0	Benznidazole	-5.4118

873054-44-5	Ivacaftor	-5.4118
56839-43-1	Eperisone hydrochloride	-5.4117
2508-72-7	Antazoline hydrochloride	-5.4113
548-73-2	Droperidol	-5.4112
50-99-7	D(+)-Glucose	-5.4112
103639-04-9	Ondansetron hydrochloride	-5.4112
57-68-1	Sulfamethazine	-5.4109
1722-62-9	Mepivacaine hydrochloride	-5.4106
2192-20-3	Hydroxyzine dihydrochloride	-5.4099
111902-57-9	Temocapil	-5.4098
151533-22-1	Levomefolate calcium	-5.4096
69712-56-7	Cefotetan	-5.4095
366789-02-8	Rivaroxaban	-5.4094
5370-01-4	Mexiletine hydrochloride	-5.4093
167465-36-3	Zosuquidar 3HCl	-5.4092
122852-69-1	Alosetron hydrochloride	-5.409
136790-76-6	Lubiprostone	-5.4089
15307-81-0	Diclofenac Potassium	-5.4087
78213-16-8	Diclofenac diethylamine	-5.4087
15307-79-6	Diclofenac sodium	-5.4084
15307-86-5	Diclofenac	-5.4081
59-33-6	Pyrilamine maleate	-5.4081
129-06-6	Warfarin sodium	-5.4081
175865-59-5	Valganciclovir hydrochloride	-5.408
132-17-2	Benztropine mesylate	-5.4077
130-37-0	Menadione sodium bisulfite	-5.4076
3206-73-3	Thioctamide	-5.4075
606143-52-6	Selumetinib	-5.4074
54-30-8	Camylofin	-5.4073
68099-86-5	Bepridil hydrochloride	-5.4072
81-27-6	Sennoside A	-5.4071
856867-55-5	Tedizolid Phosphate	-5.4069
15421-84-8	Trapidil	-5.4069
33125-97-2	Etomidate	-5.4068
426-13-1	Fluorometholone	-5.4067
388082-77-7	Lapatinib Ditosylate	-5.4063
231277-92-2	Lapatinib	-5.4063
518048-05-0	Raltegravir	-5.4062
10605-21-7	Carbendazim	-5.406
7512-17-6	N-Acetyl-D-Glucosamine	-5.4053
10402-53-6	Eprazinone dihydrochloride	-5.4052
738-70-5	Trimethoprim	-5.4052
101831-37-2	Diclazuril	-5.4049

123663-49-0	Iguratimod	-5.4048
24356-60-3	Cephapirin Sodium	-5.4046
518-28-5	Podophyllotoxin	-5.4046
1454846-35-5	Lorlatinib	-5.4039
151533-22-1	Levomefolate calcium	-5.4036
388082-78-8	Lapatinib ditosylate monohydrate	-5.4033
132-18-3	Diphenylpyraline hydrochloride	-5.4031
439239-90-4	Lasmiditan	-5.403
30652-11-0	Deferidone	-5.4024
878672-00-5	Lesinurad	-5.4021
14605-22-2	Tauroursodeoxycholic Acid	-5.4016
23651-95-8	Droxidopa	-5.4014
877399-52-5	Crizotinib	-5.4013
3546-41-6	Pyrvinium pamoate	-5.4006
121123-17-9	Cefprozil hydrate	-5.4006
456-59-7	Cyclandelate	-5.4001
10246-75-0	Hydroxyzine Pamoate	-5.3997
189279-58-1	Delafloxacin	-5.3996
98079-52-8	Lomefloxacin hydrochloride	-5.3994
98079-51-7	Lomefloxacin	-5.3994
125-02-0	Prednisolone phosphate sodium	-5.3993
55-48-1	Atropine sulfate	-5.399
5908-99-6	Atropine sulfate monohydrate	-5.399
51-55-8	Atropine	-5.399
150-76-5	4-Methoxyphenol	-5.3987
99755-59-6	Rotigotine	-5.3986
126-27-2	Oxethazaine	-5.3985
74711-43-6	Zaltoprofen	-5.3983
18010-40-7	Bupivacaine hydrochloride	-5.3982
7512-17-6	N-Acetyl-D-Glucosamine	-5.3982
70458-96-7	Norfloxacin	-5.3981
103639-04-9	Ondansetron hydrochloride	-5.3978
90098-04-7	Rebamipide	-5.3977
114-07-8	Erythromycin	-5.3977
132810-10-7	Blonanserin	-5.3972
94-07-5	Synephrine	-5.3969
72-80-0	Chloquinan	-5.3956
64-73-3	Demeclocycline hydrochloride	-5.3955
6024-85-7	Tetrahydropalmatine hydrochloride	-5.3954
133-67-5	Trichlormethiazide	-5.3952
987-78-0	Citicoline	-5.3952
125-02-0	Prednisolone phosphate sodium	-5.3947

78415-72-2	Milrinone	-5.3943
1981-58-4	Sulfamethazine sodium salt	-5.3943
33818-15-4	Citicoline sodium	-5.3935
157810-81-6	Indinavir sulfate	-5.3933
101477-54-7	Lomerizine hydrochloride	-5.3922
38083-17-9	Climbazole	-5.392
10402-53-6	Eprazinone dihydrochloride	-5.392
115256-11-6	Dofetilide	-5.3917
136236-51-6	Rasagiline	-5.3913
64-72-2	Chlortetracycline hydrochloride	-5.3913
89778-27-8	Toremifene citrate	-5.3906
73151-29-8	Fenticonazole Nitrate	-5.3906
161735-79-1	Rasagiline Mesylate	-5.3904
64-72-2	Chlortetracycline hydrochloride	-5.3899
144-83-2	Sulfapyridine	-5.3898
124858-35-1	Nadifloxacin	-5.3897
583-03-9	Fenipentol	-5.3891
89778-26-7	Toremifene	-5.3888
172152-50-0	Ilaprazole sodium	-5.3883
76-90-4	Mepenzolate Bromide	-5.3882
1134-47-0	Baclofen	-5.388
1508-75-4	Tropicamide	-5.3879
32854-75-4	Lappaconitine	-5.3878
1269440-17-6	Encorafenib	-5.3877
3644-61-9	Tolperisone hydrochloride	-5.3876
83915-83-7	Lisinopril dihydrate	-5.3873
24316-19-6	Cephalotaxine	-5.3873
863-61-6	Menaquinone-4	-5.3872
11032-49-8	Menatetrenone	-5.3872
7481-89-2	Zalcitabine	-5.3868
95233-18-4	Atovaquone	-5.3867
297-76-7	Ethynodiol diacetate	-5.3862
773-76-2	Capitrol	-5.3861
31282-04-9	Hygromycin B	-5.3858
405165-61-9	Besifloxacin HCl	-5.3857
111902-57-9	Temocapil	-5.3849
50-02-2	Dexamethasone	-5.3842
3546-41-6	Pyrvinium pamoate	-5.3841
3963-95-9	Methacycline hydrochloride	-5.3841
341-69-5	Orphenadrine hydrochloride	-5.3839
2259-96-3	Cyclothiazide	-5.3839
827022-33-3	Palbociclib Isethionate	-5.3836

827022-32-2	Palbociclib hydrochloride	-5.3836
30652-11-0	Deferidone	-5.3834
571190-30-2	Palbociclib	-5.3833
25316-40-9	Doxorubicin hydrochloride	-5.3831
90-22-2	Valethamate bromide	-5.3827
57-96-5	Sulfinpyrazone	-5.3825
74863-84-6	Argatroban	-5.3818
56-47-3	Deoxycorticosterone acetate	-5.3812
220991-20-8	Lumiracoxib	-5.3811
577-11-7	Docusate sodium	-5.381
22832-87-7	Miconazole nitrate	-5.381
645-05-6	Altretamine	-5.3809
101827-46-7	Butenafine hydrochloride	-5.3807
7681-76-7	Ronidazole	-5.3807
496775-61-2	Eltrombopag	-5.3806
3206-73-3	Thioctamide	-5.3806
59277-89-3	Aciclovir	-5.3802
6106-81-6	Scopolamine N-oxide HBr	-5.3801
174671-46-6	Tavaborole	-5.38
1744-22-5	Riluzole	-5.3798
149676-40-4	Pefloxacin Mesylate Dihydrate	-5.3797
70458-95-6	Pefloxacin Mesylate	-5.3797
144143-96-4	Eprosartan Mesylate	-5.3794
5560-59-8	Alverine citrate	-5.3793
1374640-70-6	Rociletinib	-5.3788
2921-88-2	Clorpyrifos	-5.3787
29908-03-0	Ademetionine	-5.3784
54527-84-3	Nicardipine hydrochloride	-5.3782
91161-71-6	Terbinafine	-5.3782
1110813-31-4	Dacomitinib	-5.3778
86541-74-4	Benazepril hydrochloride	-5.3776
456-59-7	Cyclandelate	-5.3773
120202-66-6	(S)-(+)-Clopidogrel hydrogen sulfate	-5.3773
520-85-4	Medroxyprogesterone	-5.3768
111902-57-9	Temocapil	-5.3761
101831-37-2	Diclazuril	-5.3761
157810-81-6	Indinavir sulfate	-5.3758
159989-65-8	Nelfinavir Mesylate	-5.3757
151533-22-1	Levomefolate calcium	-5.3752
946075-13-4	Isavuconazonium sulfate	-5.3752
705260-08-8	Vorapaxar sulfate	-5.3751

93-14-1	Guaifenesin	-5.375
459868-92-9	Rucaparib Phosphate	-5.3731
283173-50-2	Rucaparib	-5.3731
3737-09-5	Disopyramide	-5.3731
7083-71-8	(+)-Emetine dihydrochloride hydrate	-5.3731
378-44-9	Betamethasone	-5.3728
157810-81-6	Indinavir sulfate	-5.3728
912273-65-5	Edoxaban	-5.3727
173352-39-1	Afobazole hydrochloride	-5.3725
3380-34-5	Triclosan	-5.3723
3166-62-9	Methylbenactyzine Bromide	-5.3719
27262-48-2	Levobupivacaine HCl	-5.3711
27262-47-1	Levobupivacaine	-5.3711
18010-40-7	Bupivacaine hydrochloride	-5.3711
475-67-2	S-Isocorydine(+)	-5.371
111902-57-9	Temocapil	-5.371
41340-25-4	Etodolac	-5.3707
111025-46-8	Pioglitazone	-5.3706
17896-21-8	Citiolone	-5.3703
136236-51-6	Rasagiline	-5.3701
73-48-3	Bendroflumethiazide	-5.3696
60-80-0	Antipyrine	-5.3694
144060-53-7	Febuxostat	-5.3686
69558-55-0	Thymopentin	-5.3685
127-71-9	Sulfabenzamide	-5.3685
10405-02-4	Trospium chloride	-5.3684
144143-96-4	Eprosartan Mesylate	-5.3677
85721-33-1	Ciprofloxacin	-5.3673
93107-08-5	Ciprofloxacin hydrochloride	-5.3673
1263-89-4	Paromomycin Sulfate	-5.3673
162401-32-3	Roflumilast	-5.3668
405165-61-9	Besifloxacin HCl	-5.3667
114-07-8	Erythromycin	-5.3666
23651-95-8	Droxidopa	-5.3666
161735-79-1	Rasagiline Mesylate	-5.3659
15622-65-8	Molindone hydrochloride	-5.3654
62613-82-5	Oxiracetam	-5.3653
274901-16-5	Vildagliptin	-5.365
2295-58-1	Flopropione	-5.3647
75695-93-1	Isradipine	-5.364
538-71-6	Domiphen Bromide	-5.3634
52205-73-9	Estramustine phosphate sodium	-5.3634
68099-86-5	Bepridil hydrochloride	-5.363

964-52-3	Moxisylyte hydrochloride	-5.363
130656-51-8	Nifekalant hydrochloride	-5.363
17737-65-4	Clonixin	-5.3626
389139-89-3	Omadacycline	-5.3626
164650-44-6	Efinaconazole	-5.3617
59937-28-9	Malotilate	-5.3613
38083-17-9	Climbazole	-5.3612
157810-81-6	Indinavir sulfate	-5.3611
91296-87-6	Sarafloxacin hydrochloride	-5.3597
76-90-4	Mepenzolate Bromide	-5.3591
10246-75-0	Hydroxyzine Pamoate	-5.359
364622-82-2	Doripenem Hydrate	-5.3586
38083-17-9	Climbazole	-5.3586
134308-13-7	Tolcapone	-5.3584
681492-22-8	Delamanid	-5.3581
127-47-9	Retinyl acetate	-5.3581
141-94-6	Hexetidine	-5.3578
3380-34-5	Triclosan	-5.3575
151533-22-1	Levomefolate calcium	-5.3568
98769-84-7	Reboxetine mesylate	-5.3565
329-63-5	DL-Adrenaline Hydrochloride	-5.3557
2192-20-3	Hydroxyzine dihydrochloride	-5.3557
51-41-2	Norepinephrine	-5.3551
69815-49-2	Noradrenaline bitartrate	-5.3551
67-20-9	Nitrofurantoin	-5.3543
128607-22-7	Ospemifene	-5.353
120202-66-6	(S)-(+)-Clopidogrel hydrogen sulfate	-5.3523
946075-13-4	Isavuconazonium sulfate	-5.3521
863127-77-9	Dasatinib monohydrate	-5.3519
302962-49-8	Dasatinib	-5.3519
83-12-5	Phenylindione	-5.3514
76801-85-9	Azathramycin	-5.3514
124858-35-1	Nadifloxacin	-5.3514
10238-21-8	Glyburide	-5.3514
82640-04-8	Raloxifene hydrochloride	-5.3512
22368-21-4	Eupatilin	-5.3509
53-43-0	Dehydroepiandrosterone	-5.3501
520-85-4	Medroxyprogesterone	-5.3501
210353-53-0	Gemifloxacin?mesylate	-5.3495
4429-63-4	Tabersonine	-5.3493
10246-75-0	Hydroxyzine Pamoate	-5.3485
554-92-7	Trimethobenzamide hydrochloride	-5.3485
61825-94-3	Oxaliplatin	-5.3484

55-48-1	Atropine sulfate	-5.348
5908-99-6	Atropine sulfate monohydrate	-5.348
51-55-8	Atropine	-5.348
3206-73-3	Thioctamide	-5.3479
35189-28-7	Norgestimate	-5.3476
2192-20-3	Hydroxyzine dihydrochloride	-5.3467
28657-80-9	Cinoxacin	-5.3466
70458-96-7	Norfloxacin	-5.3462
60282-87-3	Gestodene	-5.3452
50924-49-7	Mizoribine	-5.345
53746-45-5	Fenoprofen calcium dihydrate	-5.3441
64019-93-8	Dipivefrin hydrochloride	-5.3441
74050-98-9	Ketanserin	-5.3436
80-49-9	Homatropine Methylbromide	-5.3435
15622-65-8	Molindone hydrochloride	-5.3431
31677-93-7	Bupropion hydrochloride	-5.3431
102767-28-2	Levetiracetam	-5.3431
2508-72-7	Antazoline hydrochloride	-5.343
3306-62-5	2-Aminobenzenesulfonamide	-5.3427
54-30-8	Camylofin	-5.3425
69308-37-8	(R)-baclofen	-5.3422
5003-48-5	Benorilate	-5.3416
548-73-2	Droperidol	-5.3416
33005-95-7	Tiaprofenic acid	-5.3415
204255-11-8	Oseltamivir phosphate	-5.3413
76-90-4	Mepenzolate Bromide	-5.3409
186826-86-8	Moxifloxacin hydrochloride	-5.3408
755037-03-7	Regorafenib	-5.3405
7681-76-7	Ronidazole	-5.3398
138890-62-7	Brinzolamide	-5.3398
90-22-2	Valethamate bromide	-5.3393
1134-47-0	Baclofen	-5.3389
13609-67-1	Hydrocortisone butyrate	-5.3389
532-03-6	Methocarbamol	-5.3377
3737-09-5	Disopyramide	-5.3375
10402-53-6	Eprazinone dihydrochloride	-5.3372
697761-98-1	Elvitegravir	-5.3371
93-14-1	Guaifenesin	-5.3365
1374356-45-2	(S)-crizotinib	-5.3365
10405-02-4	Trospium chloride	-5.3365
106730-54-5	Olprinone	-5.3364
120-51-4	Benzyl benzoate	-5.3363

4852-22-6	Proanthocyanidins	-5.3359
526-08-9	Sulfaphenazole	-5.3346
63-74-1	Sulfanilamide	-5.3342
129938-20-1	Dapoxetine HCl	-5.3341
878672-00-5	Lesinurad	-5.3337
554-92-7	Trimethobenzamide hydrochloride	-5.3334
132-18-3	Diphenylpyraline hydrochloride	-5.3333
1134-47-0	Baclofen	-5.3331
5370-01-4	Mexiletine hydrochloride	-5.3326
51-05-8	Procaine hydrochloride	-5.3325
59-46-1	Procaine	-5.3325
81840-15-5	Vesnarinone	-5.3317
22832-87-7	Miconazole nitrate	-5.3311
140-40-9	Nithiamide	-5.3302
72-14-0	Sulfathiazole	-5.3294
322-79-2	Triflusal	-5.3292
13609-67-1	Hydrocortisone butyrate	-5.3292
123447-62-1	Prulifloxacin	-5.3291
91832-40-5	Cefdinir	-5.3287
66-84-2	Glucosamine hydrochloride	-5.3276
304-20-1	Hydralazine hydrochloride	-5.3267
178307-42-1	Revaprazan hydrochloride	-5.3267
78755-81-4	Flumazenil	-5.3264
50-24-8	Prednisolone	-5.3261
123040-16-4	Azasetron hydrochloride	-5.326
51-24-1	Tiratricol	-5.3259
98079-52-8	Lomefloxacin hydrochloride	-5.3247
98079-51-7	Lomefloxacin	-5.3247
242478-38-2	Solifenacin succinate	-5.3244
60-56-0	Methimazole	-5.324
532-03-6	Methocarbamol	-5.3239
22254-24-6	Ipratropium Bromide	-5.3236
66985-17-9	Ipratropium bromide monohydrate	-5.3236
123040-16-4	Azasetron hydrochloride	-5.3234
70356-03-5	Cefaclor monohydrate	-5.3233
173352-39-1	Afobazole hydrochloride	-5.3233
159989-65-8	Nelfinavir Mesylate	-5.323
124858-35-1	Nadifloxacin	-5.3228
500287-72-9	Rilpivirine	-5.3228
129-06-6	Warfarin sodium	-5.3225
173352-39-1	Afobazole hydrochloride	-5.3218
763113-22-0	Olaparib	-5.3215
3543-75-7	Bendamustine hydrochloride	-5.3207

122852-69-1	Alosetron hydrochloride	-5.3205
72-80-0	Chloquinan	-5.3199
58-46-8	Tetrabenazine	-5.3199
124858-35-1	Nadifloxacin	-5.3198
52-21-1	Prednisolone-21-acetate	-5.3198
91-64-5	Coumarin	-5.319
446-86-6	Azathioprine	-5.3189
968-81-0	Acetohexamide	-5.3187
10605-21-7	Carbendazim	-5.3187
59-92-7	Levodopa	-5.3185
50-99-7	D(+)-Glucose	-5.3182
58-56-0	Pyridoxine hydrochloride	-5.3181
65-23-6	Pyridoxine	-5.3181
15676-16-1	Sulpiride	-5.3179
74536-44-0	Metadoxine	-5.3178
738-70-5	Trimethoprim	-5.3178
446-86-6	Azathioprine	-5.3176
23672-07-3	Levosulpiride	-5.3176
108612-45-9	Mizolastine	-5.3176
163521-12-8	Vilazodone	-5.3175
113-92-8	Chlorpheniramine maleate	-5.3174
3366-95-8	Secnidazole	-5.3172
111902-57-9	Temocapil	-5.3169
61-16-5	Methoxamine hydrochloride	-5.3168
94-07-5	Synephrine	-5.3166
108929-04-0	Epinastine HCl	-5.3164
68-26-8	Retinol	-5.3158
206361-99-1	Darunavir	-5.315
17696-69-4	Hematoporphyrin dihydrochloride	-5.3149
641571-10-0	Nilotinib	-5.3147
635728-49-3	Darunavir?Ethanolate	-5.3147
28657-80-9	Cinoxacin	-5.3141
747-36-4	Hydroxychloroquine sulfate	-5.314
22161-81-5	(S)-(+)-Ketoprofen	-5.3139
22071-15-4	Ketoprofen	-5.3139
456-59-7	Cyclandelate	-5.3139
64-86-8	Colchicine	-5.3135
100-55-0	Roniacol	-5.3133
452-35-7	Ethoxzolamide	-5.3133
82419-36-1	Ofloxacin	-5.3132
120-47-8	Ethylparaben	-5.3131
28395-03-1	Bumetanide	-5.3129
73590-85-9	Omeprazole sulfide	-5.3128

15826-37-6	Sodium cromoglycate	-5.3128
84-80-0	Vitamin K1	-5.3127
116-38-1	Edrophonium chloride	-5.3125
64019-93-8	Dipivefrin hydrochloride	-5.3124
961-29-5	Isoliquiritigenin	-5.3117
22457-89-2	Benfotiamine	-5.3117
13609-67-1	Hydrocortisone butyrate	-5.3117
50-23-7	Hydrocortisone	-5.3115
70-70-2	Vanatone	-5.3109
69004-03-1	Toltrazuril	-5.3098
82410-32-0	Ganciclovir	-5.3098
51803-78-2	Nimesulide	-5.3098
126544-47-6	Ciclesonide	-5.3096
99-50-3	Protocatechuic acid	-5.3095
30123-17-2	Tianeptine sodium	-5.3095
113-38-2	17-Beta-Estradiol-3,17-Dipropionate	-5.3092
15826-37-6	Sodium cromoglycate	-5.3091
132810-10-7	Blonanserin	-5.3089
2259-96-3	Cyclothiazide	-5.3088
53716-49-7	Carprofen	-5.3085
84625-61-6	Itraconazole	-5.3083
183319-69-9	Erlotinib hydrochloride	-5.3081
183321-74-6	Erlotinib	-5.3081
3366-95-8	Secnidazole	-5.308
161814-49-9	Amprenavir	-5.3075
124-94-7	Triamcinolone	-5.3075
129-06-6	Warfarin sodium	-5.3073
84294-96-2	Enoxacin Sesquihydrate	-5.307
130-37-0	Menadione sodium bisulfite	-5.3066
1197953-54-0	Brigatinib	-5.3066
26328-04-1	Cinepazide maleate	-5.3066
80-77-3	Chlormezanone	-5.3063
3818-50-6	Bephenium Hydroxynaphthoate	-5.306
149-91-7	Gallic acid	-5.3058
74011-58-8	Enoxacin	-5.3058
80-49-9	Homatropine Methylbromide	-5.3056
124858-35-1	Nadifloxacin	-5.3056
1226781-44-7	Omarigliptin	-5.3055
21462-39-5	Clindamycin HCl	-5.305
2259-96-3	Cyclothiazide	-5.3046
79-57-2	Oxytetracycline	-5.3046

5560-59-8	Alverine citrate	-5.3046
103-16-2	Monobenzone	-5.3043
13110-96-8	5-Acetylsalicylic acid	-5.3043
583-03-9	Fenipentol	-5.3043
1490-04-6	DL-Menthol	-5.3042
22881-35-2	Famprofazone	-5.3039
515-64-0	Sulfisomidine	-5.3036
6856-31-1	Loxeen	-5.3035
1104-22-9	Meclizine dihydrochloride	-5.3034
147084-10-4	Alcaftadine	-5.303
639089-54-6	Tozasertib	-5.3029
1163-36-6	Clemizole hydrochloride	-5.3024
912-60-7	Noscapine hydrochloride	-5.3016
698387-09-6	Neratinib	-5.3016
4880-88-0	Eburnalritardo	-5.3009
23828-92-4	Ambroxol hydrochloride	-5.3008
58947-95-8	DL-trihexyphenidyl hydrochloride	-5.3004
1986-47-6	Tranylcypromine (2-PCPA) hydrochloride	-5.3002
1354744-91-4	Naloxegol oxalate	-5.3002
157810-81-6	Indinavir sulfate	-5.3001
908-54-3	Diminazene Aceturate	-5.2999
157810-81-6	Indinavir sulfate	-5.2999
18683-91-5	Ambroxol	-5.2999
85721-33-1	Ciprofloxacin	-5.2997
93107-08-5	Ciprofloxacin hydrochloride	-5.2997
38083-17-9	Climbazole	-5.2993
22457-89-2	Benfotiamine	-5.299
946075-13-4	Isavuconazonium sulfate	-5.2986
113-38-2	17-Beta-Estradiol-3,17-Dipropionate	-5.2982
93106-60-6	Enrofloxacin	-5.2982
50-63-5	Chloroquine diphosphate	-5.2978
30123-17-2	Tianeptine sodium	-5.2976
26095-59-0	Otilonium bromide	-5.2972
1218779-75-9	Apatinib mesylate	-5.2971
811803-05-1	Apatinib	-5.2971
154229-18-2	Abiraterone Acetate	-5.297
1104-22-9	Meclizine dihydrochloride	-5.2969
54556-98-8	Propiverine hydrochloride	-5.2965
87848-99-5	Acrivastine	-5.2963
435-97-2	Phenprocoumon	-5.2963
172152-50-0	Ilaprazole sodium	-5.2956

40828-46-4	Suprofen	-5.2954
4852-22-6	Proanthocyanidins	-5.295
532-03-6	Methocarbamol	-5.2949
6620-60-6	Proglumide	-5.2948
548-62-9	Crystal Violet	-5.2937
1134-47-0	Baclofen	-5.2935
112885-42-4	Mosapride Citrate	-5.2932
23327-57-3	Nefopam hydrochloride	-5.2928
78967-07-4	Mofezolac	-5.2927
189188-57-6	Tegaserod maleate	-5.2926
70-70-2	Vanatone	-5.2921
187227-45-8	Oseltamivir acid	-5.2919
151533-22-1	Levomefolate calcium	-5.2917
19408-46-9	Kasugamycin hydrochloride	-5.2916
36703-88-5	Isoprinosine	-5.2914
58-63-9	Inosine	-5.2914
548-62-9	Crystal Violet	-5.2911
65391-42-6	Bestatin hydrochloride	-5.2911
58970-76-6	Ubenimex	-5.2911
6153-33-9	Mebhydrolin napadisylate	-5.2902
69308-37-8	(R)-baclofen	-5.2899
111902-57-9	Temocapil	-5.2898
6533-68-2	Scopolamine HBr trihydrate	-5.2896
298-57-7	Stugeron	-5.2894
524-81-2	Mebhydroline	-5.2893
137-58-6	Xylocaine	-5.2891
127373-66-4	Sivelestat	-5.2891
1953-04-4	Galanthamine HBr	-5.2891
1722-62-9	Mepivacaine hydrochloride	-5.2891
1508-75-4	Tropicamide	-5.2889
19387-91-8	Tinidazole	-5.2885
6108-05-0	Lidocaine Hydrochloride hydrate	-5.2882
73-78-9	Lidocaine hydrochloride	-5.2882
191217-81-9	Pramipexole 2HCl Monohydrate	-5.2875
90-22-2	Valethamate bromide	-5.2873
611-75-6	Bromhexine hydrochloride	-5.287
5633-20-5	Oxybutynin	-5.287
1508-65-2	Oxybutynin hydrochloride	-5.287
27591-69-1	Tilorone dihydrochloride	-5.2864
86780-90-7	Aranidipine	-5.2859
161735-79-1	Rasagiline Mesylate	-5.2858
4360-12-7	Ajmaline	-5.2856
132-18-3	Diphenylpyraline hydrochloride	-5.2855

89396-94-1	Imidapril HCl	-5.2855
175865-59-5	Valganciclovir hydrochloride	-5.2854
172152-50-0	Ilaprazole sodium	-5.285
73963-72-1	Cilostazol	-5.2849
562-10-7	Doxylamine succinate	-5.2847
4852-22-6	Proanthocyanidins	-5.2843
32854-75-4	Lappaconitine	-5.2842
775304-57-9	Ataluren	-5.284
912-60-7	Noscapine hydrochloride	-5.2838
1744-22-5	Riluzole	-5.2827
68767-14-6	Loxoprofen	-5.2826
72-14-0	Sulfathiazole	-5.2826
1490-04-6	DL-Menthol	-5.2824
10402-53-6	Eprazinone dihydrochloride	-5.2824
3737-09-5	Disopyramide	-5.2822
141-94-6	Hexetidine	-5.2821
164650-44-6	Efinaconazole	-5.2814
856867-55-5	Tedizolid Phosphate	-5.2809
1104-22-9	Meclizine dihydrochloride	-5.2809
93106-60-6	Enrofloxacin	-5.2807
101831-37-2	Diclazuril	-5.2806
4852-22-6	Proanthocyanidins	-5.2802
125-02-0	Prednisolone phosphate sodium	-5.2798
127373-66-4	Sivelestat	-5.2797
101831-37-2	Diclazuril	-5.2797
2921-88-2	Clorpyrifos	-5.2797
912-60-7	Noscapine hydrochloride	-5.2796
78415-72-2	Milrinone	-5.2792
51-60-5	Hodostin	-5.2789
901119-35-5	Fostamatinib	-5.2787
157810-81-6	Indinavir sulfate	-5.2786
51-24-1	Tiratricol	-5.2785
80-32-0	Sulfachloropyridazine	-5.2783
178429-62-4	solriamfetol	-5.2777
99300-78-4	Venlafaxine hydrochloride	-5.2768
2259-96-3	Cyclothiazide	-5.2767
74536-44-0	Metadoxine	-5.2766
58-56-0	Pyridoxine hydrochloride	-5.2766
65-23-6	Pyridoxine	-5.2766
1025687-58-4	R788 Disodium	-5.2762
926037-48-1	Radotinib	-5.2761
356-12-7	Fluocinonide	-5.276

88671-89-0	Myclobutanil	-5.2759
943-17-9	Etilefrine hydrochloride	-5.2754
1098-97-1	Pyrithioxin	-5.2754
136236-51-6	Rasagiline	-5.2753
60-18-4	Tyrosine	-5.2751
134678-17-4	Lamivudine	-5.2749
79794-75-5	Loratadine	-5.2745
12650-69-0	Mupirocin	-5.2744
84057-84-1	Lamotrigine	-5.2742
1360460-82-7	Anlotinib Dihydrochloride	-5.2741
1058156-90-3	Anlotinib	-5.2741
167465-36-3	Zosuquidar 3HCl	-5.2736
69-81-8	Carbazochrome	-5.2735
614-39-1	Procainamide hydrochloride	-5.2734
167221-71-8	Clevidipine butyrate	-5.2733
132203-70-4	Cilnidipine	-5.2732
112885-42-4	Mosapride Citrate	-5.273
189188-57-6	Tegaserod maleate	-5.2727
114-80-7	Neostigmine bromide	-5.2726
60-18-4	Tyrosine	-5.2723
439081-18-2	Afatinib	-5.2721
500-64-1	L-KAWAIN	-5.2717
162401-32-3	Roflumilast	-5.2709
30484-77-6	Flunarizine dihydrochloride	-5.2706
1263-89-4	Paromomycin Sulfate	-5.2705
3254-89-5	Diphenidol hydrochloride	-5.2705
13110-96-8	5-Acetylsalicylic acid	-5.2703
850140-73-7	Afatinib Dimaleate	-5.2703
64-73-3	Demeclocycline hydrochloride	-5.27
530-78-9	Flufenamic acid	-5.2695
3374-05-8	Nalidixic acid sodium salt	-5.2692
389-08-2	Nalidixic acid	-5.2692
2196-13-6	Thioisonicotinamide	-5.2692
64019-93-8	Dipivefrin hydrochloride	-5.269
364782-34-3	Cinacalcet hydrochloride	-5.2689
25655-41-8	Povidone-iodine	-5.2688
163253-35-8	Sitafloxacin Hydrate	-5.2682
60-93-5	Quinine dihydrochloride	-5.2682
130-95-0	Quinine	-5.2682
25913-34-2	Tinoridine hydrochloride	-5.2675
74011-58-8	Enoxacin	-5.2674

456-59-7	Cyclandelate	-5.2673
439239-90-4	Lasmiditan	-5.2668
60628-96-8	Bifonazole	-5.2666
100-55-0	Roniacol	-5.2663
84294-96-2	Enoxacin Sesquihydrate	-5.2662
1194506-26-7	Fruquintinib	-5.2662
50-42-0	Adiphenine hydrochloride	-5.2659
33342-05-1	Gliquidone	-5.2657
123040-16-4	Azasetron hydrochloride	-5.2656
189188-57-6	Tegaserod maleate	-5.2655
120-97-8	Dichlorphenamide	-5.2654
50-02-2	Dexamethasone	-5.2653
59729-37-2	Fexinidazole	-5.2652
124832-27-5	Valacyclovir hydrochloride	-5.2652
76-90-4	Mepenzolate Bromide	-5.2652
57808-65-8	Closantel	-5.265
53164-05-9	Acemetacin	-5.2647
112811-59-3	Gatifloxacin	-5.2645
152-47-6	Sulfalen	-5.2643
82752-99-6	Nefazodone hydrochloride	-5.2642
936727-05-8	Lumacaftor	-5.2641
1490-04-6	DL-Menthol	-5.2639
606143-52-6	Selumetinib	-5.2635
22373-78-0	Monensin sodium salt	-5.2632
548-73-2	Droperidol	-5.2624
5003-48-5	Benorilate	-5.2622
149676-40-4	Pefloxacin Mesylate Dihydrate	-5.2617
161796-78-7	Esomeprazole Sodium	-5.2615
3306-62-5	2-Aminobenzenesulfonamide	-5.2612
53716-49-7	Carprofen	-5.2611
70458-95-6	Pefloxacin Mesylate	-5.2611
405165-61-9	Besifloxacin HCl	-5.261
56-04-2	Methylthiouracil	-5.2608
532-03-6	Methocarbamol	-5.2607
532-11-6	Anethole trithione	-5.2606
87-89-8	Inositol	-5.2604
985-13-7	Ethaverine hydrochloride	-5.2604
61-16-5	Methoxamine hydrochloride	-5.2599
2192-20-3	Hydroxyzine dihydrochloride	-5.2596
10246-75-0	Hydroxyzine Pamoate	-5.2593
1490-04-6	DL-Menthol	-5.2592
93106-60-6	Enrofloxacin	-5.2592

129497-78-5	Verteporfin	-5.259
62613-82-5	Oxiracetam	-5.2589
178307-42-1	Revaprazan hydrochloride	-5.2584
54-85-3	Isoniazid	-5.2582
101152-94-7	Milnacipran hydrochloride	-5.2575
150322-43-3	Prasugrel	-5.2571
1134-47-0	Baclofen	-5.2569
96847-55-1	Levomilnacipran	-5.2566
577-11-7	Docusate sodium	-5.2564
124858-35-1	Nadifloxacin	-5.2564
1098-97-1	Pyrithioxin	-5.2558
29908-03-0	Ademetionine	-5.2555
15686-71-2	Cephalexin	-5.2548
33419-42-0	Etoposide	-5.2548
4429-63-4	Tabersonine	-5.2547
129-06-6	Warfarin sodium	-5.2545
1104-22-9	Meclizine dihydrochloride	-5.2544
3697-42-5	Chlorhexidine hydrochloride	-5.2543
56-95-1	Chlorhexidine Acetate	-5.2543
1490-04-6	DL-Menthol	-5.2543
82419-36-1	Ofloxacin	-5.2542
946075-13-4	Isavuconazonium sulfate	-5.254
1218779-75-9	Apatinib mesylate	-5.2532
811803-05-1	Apatinib	-5.2532
24729-96-2	Clindamycin phosphate	-5.2527
96847-55-1	Levomilnacipran	-5.2527
29908-03-0	Ademetionine	-5.2525
54556-98-8	Propiverine hydrochloride	-5.2523
94-62-2	Piperine	-5.2517
1722-62-9	Mepivacaine hydrochloride	-5.2515
313-67-7	Aristolochic Acid	-5.2513
5370-01-4	Mexiletine hydrochloride	-5.2511
57-96-5	Sulfinpyrazone	-5.2509
103890-78-4	Lacidipine	-5.2508
74711-43-6	Zaltoprofen	-5.2508
54029-12-8	Albendazole Oxide	-5.2503
50847-11-5	Ibudilast	-5.2502
112885-42-4	Mosapride Citrate	-5.2494
38083-17-9	Climbazole	-5.2488
87848-99-5	Acrivastine	-5.2485
4852-22-6	Proanthocyanidins	-5.2484
150915-40-5	Tirofiban hydrochloride monohydrate	-5.2478

37091-65-9	Azlocillin sodium	-5.2476
936727-05-8	Lumacaftor	-5.2475
122320-73-4	Rosiglitazone	-5.2474
103639-04-9	Ondansetron hydrochloride	-5.2471
13609-67-1	Hydrocortisone butyrate	-5.2462
22373-78-0	Monensin sodium salt	-5.2459
19428-14-9	Benproperine phosphate	-5.2458
985-13-7	Ethaverine hydrochloride	-5.2458
98769-84-7	Reboxetine mesylate	-5.2458
65513-72-6	Glafenine Hydrochloride	-5.2457
3820-67-5	Glafenine	-5.2457
1230487-00-9	Siponimod	-5.2455
70458-96-7	Norfloxacin	-5.2455
97240-79-4	Topiramate	-5.2454
388082-77-7	Lapatinib Ditosylate	-5.2454
231277-92-2	Lapatinib	-5.2454
87233-61-2	Emedastine	-5.2452
98079-52-8	Lomefloxacin hydrochloride	-5.2451
98079-51-7	Lomefloxacin	-5.2451
154361-50-9	Capecitabine	-5.245
59-92-7	Levodopa	-5.2449
3366-95-8	Secnidazole	-5.2448
220620-09-7	Tigecycline	-5.2448
52214-84-3	Ciprofibrate	-5.2448
297-76-7	Ethynodiol diacetate	-5.2446
25389-94-0	Kanamycin sulfate	-5.2445
74711-43-6	Zaltoprofen	-5.2443
439239-90-4	Lasmiditan	-5.2441
51-03-6	Piperonyl butoxide	-5.2441
1949-20-8	Oxolamine citrate	-5.2441
3254-89-5	Diphenidol hydrochloride	-5.244
15676-16-1	Sulpiride	-5.2436
111974-72-2	Quetiapine Fumarate	-5.2434
645-05-6	Altretamine	-5.2433
500-64-1	L-KAWAIN	-5.2432
164579-32-2	Pantoprazole Sodium Hydrate	-5.2429
6160-12-9	(-)-Sparteine sulfate pentahydrate	-5.2426
520-85-4	Medroxyprogesterone	-5.2423
111902-57-9	Temocapil	-5.242
912-60-7	Noscapine hydrochloride	-5.2419
2508-72-7	Antazoline hydrochloride	-5.2416
148-72-1	Pilocarpine nitrate	-5.2415

61-16-5	Methoxamine hydrochloride	-5.2411
54-71-7	(+)-Pilocarpine hydrochloride	-5.2409
5630-53-5	Tibolone	-5.2407
388082-78-8	Lapatinib ditosylate monohydrate	-5.2406
4852-22-6	Proanthocyanidins	-5.2404
1108743-60-7	Entrectinib	-5.2404
116-38-1	Edrophonium chloride	-5.2403
65513-72-6	Glafenine Hydrochloride	-5.2402
3820-67-5	Glafenine	-5.2402
210353-53-0	Gemifloxacin?mesylate	-5.24
34140-59-5	Trimebutine maleate	-5.2395
39133-31-8	Trimebutine	-5.2395
111902-57-9	Temocapil	-5.2394
946075-13-4	Isavuconazonium sulfate	-5.2386
80-77-3	Chlormezanone	-5.2385
164579-32-2	Pantoprazole Sodium Hydrate	-5.2382
136470-78-5	Abacavir	-5.2377
120-47-8	Ethylparaben	-5.2364
29908-03-0	Ademetionine	-5.2364
313-67-7	Aristolochic Acid	-5.2363
827022-33-3	Palbociclib Isethionate	-5.2363
827022-32-2	Palbociclib hydrochloride	-5.2363
389-08-2	Nalidixic acid	-5.2361
3366-95-8	Secnidazole	-5.2361
99-50-3	Protocatechuic acid	-5.2359
29908-03-0	Ademetionine	-5.2359
3374-05-8	Nalidixic acid sodium salt	-5.2358
188062-50-2	Abacavir sulfate	-5.2356
40828-46-4	Suprofen	-5.2355
10405-02-4	Trospium chloride	-5.2352
67392-87-4	Drospirenone	-5.2344
864750-70-9	Revefenacin	-5.234
56391-57-2	Netilmicin sulfate	-5.234
204255-11-8	Oseltamivir phosphate	-5.2339
33419-42-0	Etoposide	-5.2338
17696-69-4	Hematoporphyrin dihydrochloride	-5.2336
3374-05-8	Nalidixic acid sodium salt	-5.2335
389-08-2	Nalidixic acid	-5.2335
147526-32-7	Pitavastatin calcium	-5.2325
1163-36-6	Clemizole hydrochloride	-5.2321
112885-42-4	Mosapride Citrate	-5.2316
571190-30-2	Palbociclib	-5.2315

673-06-3	D-Phenylalanine	-5.2315
103639-04-9	Ondansetron hydrochloride	-5.2312
191217-81-9	Pramipexole 2HCl Monohydrate	-5.2309
33342-05-1	Gliquidone	-5.2309
89-25-8	Edaravone	-5.2307
697761-98-1	Elvitegravir	-5.2307
871038-72-1	Raltegravir potassium	-5.2307
54-35-3	Procaine penicillin G	-5.2304
4146-30-9	Neomycin Sulphate B	-5.2292
7491-74-9	Piracetam	-5.229
1981-58-4	Sulfamethazine sodium salt	-5.2288
61-16-5	Methoxamine hydrochloride	-5.2287
130-37-0	Menadione sodium bisulfite	-5.2286
439081-18-2	Afatinib	-5.2284
154229-18-2	Abiraterone Acetate	-5.2283
123447-62-1	Prulifloxacin	-5.2283
29908-03-0	Ademetionine	-5.2281
164650-44-6	Efinaconazole	-5.2279
551-92-8	Dimetridazole	-5.2278
111902-57-9	Temocapil	-5.2277
93-14-1	Guaifenesin	-5.2276
54-31-9	Furosemide	-5.2271
204255-11-8	Oseltamivir phosphate	-5.227
129-06-6	Warfarin sodium	-5.2267
132-60-5	Cinchophen	-5.2267
1722-62-9	Mepivacaine hydrochloride	-5.2264
53797-35-6	Ribostamycin sulfate	-5.2263
562-10-7	Doxylamine succinate	-5.2262
151533-22-1	Levomefolate calcium	-5.2251
99300-78-4	Venlafaxine hydrochloride	-5.225
129722-12-9	Aripiprazole	-5.2248
850140-73-7	Afatinib Dimaleate	-5.2245
934660-93-2	Cobimetinib	-5.2242
122320-73-4	Rosiglitazone	-5.2241
2139-47-1	Nifenazone	-5.2238
980-71-2	Brompheniramine hydrogen maleate	-5.2238
21462-39-5	Clindamycin HCl	-5.2237
111025-46-8	Pioglitazone	-5.2234
946075-13-4	Isavuconazonium sulfate	-5.2232
302962-49-8	Dasatinib	-5.2215
141-94-6	Hexetidine	-5.2214
1230487-00-9	Siponimod	-5.2213

54-31-9	Furosemide	-5.2212
27741-01-1	Geniposidic Acid	-5.2201
1454846-35-5	Lorlatinib	-5.2201
75706-12-6	Leflunomide	-5.2199
22254-24-6	Ipratropium Bromide	-5.2198
66985-17-9	Ipratropium bromide monohydrate	-5.2198
132036-88-5	Ramosetron	-5.2197
54527-84-3	Nicardipine hydrochloride	-5.2194
161796-78-7	Esomeprazole Sodium	-5.2194
73590-58-6	Omeprazole	-5.2194
5633-20-5	Oxybutynin	-5.2193
1508-65-2	Oxybutynin hydrochloride	-5.2193
26833-87-4	Homoharringtonine	-5.2182
74011-58-8	Enoxacin	-5.2181
71610-00-9	Cephalomannine	-5.2181
130656-51-8	Nifekalant hydrochloride	-5.2179
84294-96-2	Enoxacin Sesquihydrate	-5.2178
1508-75-4	Tropicamide	-5.2176
130-26-7	Clioquinol	-5.2175
946075-13-4	Isavuconazonium sulfate	-5.2174
863127-77-9	Dasatinib monohydrate	-5.217
83105-70-8	Sultamicillin Tosylate	-5.2163
124832-27-5	Valacyclovir hydrochloride	-5.2158
1949-20-8	Oxolamine citrate	-5.2152
115956-12-2	Dolasetron	-5.2151
130693-82-2	Dorzolamide hydrochloride	-5.2146
130-40-5	Flavin mononucleotide	-5.2146
6620-60-6	Proglumide	-5.2145
T5164	Cabozantinib hydrochloride (849217-68-1(free base))	-5.2143
849217-68-1	Cabozantinib	-5.2143
1140909-48-3	Cabozantinib Malate	-5.2143
101477-54-7	Lomerizine hydrochloride	-5.2142
59-87-0	Furacilin	-5.2138
14919-77-8	Benserazide hydrochloride	-5.2137
1257044-40-8	ABT199	-5.2137
147084-10-4	Alcaftadine	-5.2127
128-13-2	Ursodeoxycholic acid	-5.2126
10605-21-7	Carbendazim	-5.2122
16816-67-4	D-Pantethine	-5.2114
435-97-2	Phenprocoumon	-5.2114

13171-25-0	Trimetazidine dihydrochloride	-5.2111
985-13-7	Ethaverine hydrochloride	-5.2102
111974-72-2	Quetiapine Fumarate	-5.21
108929-04-0	Epinastine HCl	-5.2099
88678-31-3	Liranaftate	-5.2098
5728-52-9	4-Biphenylacetic acid	-5.2095
112885-42-4	Mosapride Citrate	-5.2095
14919-77-8	Benserazide hydrochloride	-5.2091
57808-65-8	Closantel	-5.209
69558-55-0	Thymopentin	-5.209
59-63-2	Isocarboxazid	-5.2089
69004-03-1	Toltrazuril	-5.2086
103577-45-3	Lansoprazole	-5.2085
345909-26-4	Taurocholic acid sodium salt hydrate	-5.2084
1104-22-9	Meclizine dihydrochloride	-5.2074
95789-30-3	Cefotiam Hexetil Hydrochloride	-5.2074
51940-44-4	Pipemidic acid	-5.2073
161796-78-7	Esomeprazole Sodium	-5.2071
73590-58-6	Omeprazole	-5.2071
32449-92-6	Dicurone	-5.207
164579-32-2	Pantoprazole Sodium Hydrate	-5.207
113-98-4	Benzylpenicillin potassium	-5.207
446-86-6	Azathioprine	-5.207
163680-77-1	Pazufloxacin Mesylate	-5.2067
99-50-3	Protocatechuic acid	-5.2063
18883-66-4	Streptozocin	-5.2061
4880-88-0	Eburnalritardo	-5.2059
54527-84-3	Nicardipine hydrochloride	-5.2057
3485-62-9	Clidinium bromide	-5.2056
178429-62-4	solriamfetol	-5.2053
157810-81-6	Indinavir sulfate	-5.2048
178307-42-1	Revaprazan hydrochloride	-5.2046
152-47-6	Sulfalen	-5.2045
1032900-25-6	LDK378	-5.2044
69655-05-6	Didanosine	-5.2043
238750-77-1	Tosedostat	-5.2042
706782-28-7	Pimavanserin tartrate	-5.2038
706779-91-1	Pimavanserin	-5.2038
548-73-2	Droperidol	-5.2038
1490-04-6	DL-Menthol	-5.2037
1405-37-4	Capastat sulfate	-5.2037
66898-62-2	Talniflumate	-5.2035

29908-03-0	Ademetionine	-5.2032
2066-89-9	Pasiniazid	-5.2029
28657-80-9	Cinoxacin	-5.2029
25655-41-8	Povidone-iodine	-5.2027
38821-49-7	Carbidopa Hydrate	-5.2027
69-57-8	Penicillin G sodium salt	-5.2025
1263-89-4	Paromomycin Sulfate	-5.2024
1194506-26-7	Fruquintinib	-5.2021
4852-22-6	Proanthocyanidins	-5.2021
3200-06-4	Nafronyl oxalate	-5.202
14698-29-4	Oxolinic acid	-5.2018
141-90-2	Deracil	-5.2014
220620-09-7	Tigecycline	-5.2008
1986-47-6	Tranylcypromine (2-PCPA) hydrochloride	-5.2004
138452-21-8	Fexofenadine hydrochloride	-5.2002
210353-53-0	Gemifloxacin?mesylate	-5.2002
90-80-2	Delta-Gluconolactone	-5.2
16676-29-2	Naltrexone hydrochloride	-5.2
123447-62-1	Prulifloxacin	-5.1997
151533-22-1	Levomefolate calcium	-5.1996
29908-03-0	Ademetionine	-5.1994
164579-32-2	Pantoprazole Sodium Hydrate	-5.1987
82752-99-6	Nefazodone hydrochloride	-5.1984
132810-10-7	Blonanserin	-5.1983
4093-35-0	Bromopride	-5.1975
29908-03-0	Ademetionine	-5.1968
51-03-6	Piperonyl butoxide	-5.1965
17440-83-4	Amiloride hydrochloride dihydrate	-5.1961
98-96-4	Pyrazinamide	-5.1959
606143-89-9	Binimetinib	-5.1954
150-76-5	4-Methoxyphenol	-5.1947
58-46-8	Tetrabenazine	-5.1947
61-16-5	Methoxamine hydrochloride	-5.1947
2259-96-3	Cyclothiazide	-5.1943
141-94-6	Hexetidine	-5.1942
33386-08-2	Buspirone hydrochloride	-5.1937
17696-69-4	Hematoporphyrin dihydrochloride	-5.1932
775304-57-9	Ataluren	-5.193
3737-09-5	Disopyramide	-5.1926
53716-49-7	Carprofen	-5.1923
112811-59-3	Gatifloxacin	-5.1922
29908-03-0	Ademetionine	-5.1922

87848-99-5	Acrivastine	-5.1919
101152-94-7	Milnacipran hydrochloride	-5.1915
96847-55-1	Levomilnacipran	-5.1912
162401-32-3	Roflumilast	-5.1911
24316-19-6	Cephalotaxine	-5.1911
132-17-2	Benztropine mesylate	-5.191
2295-58-1	Flopropione	-5.1909
1786-81-8	Propitocaine hydrochloride	-5.1908
1110813-31-4	Dacomitinib	-5.1906
1194506-26-7	Fruquintinib	-5.1901
721-50-6	Prilocaine	-5.1899
111902-57-9	Temocapil	-5.1898
140-64-7	Pentamidine isethionate	-5.1898
157810-81-6	Indinavir sulfate	-5.1893
92636-39-0	Cefminox Sodium	-5.1893
35212-22-7	Ipriflavone	-5.1892
15622-65-8	Molindone hydrochloride	-5.1891
67392-87-4	Drospirenone	-5.1884
112811-59-3	Gatifloxacin	-5.1881
485-49-4	(+)-Bicuculline	-5.188
149-64-4	Scopolamine butylbromide	-5.188
23979-41-1	(R)-Naproxen	-5.1872
72509-76-3	Felodipine	-5.187
38083-17-9	Climbazole	-5.1864
523-87-5	Dimenhydrinate	-5.1859
147-24-0	Diphenhydramine hydrochloride	-5.1853
946075-13-4	Isavuconazonium sulfate	-5.1853
67-45-8	Furazolidone	-5.1851
123447-62-1	Prulifloxacin	-5.1851
4852-22-6	Proanthocyanidins	-5.1848
1156-19-0	Tolazamide	-5.1847
130693-82-2	Dorzolamide hydrochloride	-5.1844
72432-03-2	Miglitol	-5.184
16773-42-5	Ornidazole	-5.1838
70458-96-7	Norfloxacin	-5.1835
67-20-9	Nitrofurantoin	-5.1833
118288-08-7	Lafutidine	-5.1832
136310-93-5	Tiotropium bromide	-5.1829
150399-21-6	Balsalazide disodium salt dihydrate	-5.1827
29908-03-0	Ademetionine	-5.1826
439081-18-2	Afatinib	-5.1825

157810-81-6	Indinavir sulfate	-5.1821
22199-08-2	Silver sulfadiazine	-5.1814
92134-98-0	Fosphenytoin sodium	-5.1811
187235-37-6	Pretomanid	-5.181
35189-28-7	Norgestimate	-5.1808
2259-96-3	Cyclothiazide	-5.1806
132-18-3	Diphenylpyraline hydrochloride	-5.1803
57808-65-8	Closantel	-5.1802
850140-73-7	Afatinib Dimaleate	-5.1801
57808-66-9	Domperidone	-5.1798
968-81-0	Acetohexamide	-5.1798
1289023-67-1	BMS927711	-5.1798
83-88-5	Riboflavin	-5.1797
4852-22-6	Proanthocyanidins	-5.1794
360-70-3	Nandrolone decanoate	-5.1792
30123-17-2	Tianeptine sodium	-5.1788
30484-77-6	Flunarizine dihydrochloride	-5.1786
120-47-8	Ethylparaben	-5.1784
435-97-2	Phenprocoumon	-5.1783
2259-96-3	Cyclothiazide	-5.1783
58947-95-8	DL-trihexyphenidyl hydrochloride	-5.1783
53251-94-8	Pinaverium bromide	-5.1777
242478-38-2	Solifenacin succinate	-5.1775
548-66-3	Drofenine hydrochloride	-5.1773
59338-87-3	Alizapride hydrochloride	-5.1771
6024-85-7	Tetrahydropalmatine hydrochloride	-5.1771
69004-03-1	Toltrazuril	-5.1771
4852-22-6	Proanthocyanidins	-5.1769
961-29-5	Isoliquiritigenin	-5.1768
59-30-3	Folic acid	-5.1767
24729-96-2	Clindamycin phosphate	-5.1766
175865-59-5	Valganciclovir hydrochloride	-5.1766
856676-23-8	Choline Fenofibrate	-5.1765
131707-23-8	Arbidol hydrochloride	-5.1761
389139-89-3	Omadacycline	-5.1756
157810-81-6	Indinavir sulfate	-5.1755
101831-37-2	Diclazuril	-5.1753
4940-39-0	Chromocarbe	-5.1752
141-90-2	Deracil	-5.1749
2468-21-5	Catharanthine	-5.1749
157810-81-6	Indinavir sulfate	-5.174
55-56-1	Chlorhexidine	-5.174

59277-89-3	Aciclovir	-5.1736
70356-03-5	Cefaclor monohydrate	-5.1733
1195765-45-7	Dabrafenib	-5.1733
122320-73-4	Rosiglitazone	-5.173
141-94-6	Hexetidine	-5.1725
123524-52-7	Azelnidipine	-5.1722
122841-12-7	Cefoselis Sulfate	-5.1716
10402-53-6	Eprazinone dihydrochloride	-5.1715
161796-78-7	Esomeprazole Sodium	-5.1715
50-03-3	Hydrocortisone acetate	-5.1708
147221-93-0	Delavirdine (mesylate)	-5.1705
27164-46-1	Cefazolin sodium salt	-5.1699
103628-48-4	Sumatriptan succinate	-5.1697
61422-45-5	Carmofur	-5.1694
1354744-91-4	Naloxegol oxalate	-5.1694
132-17-2	Benztropine mesylate	-5.1693
154229-19-3	Abiraterone	-5.1687
73816-42-9	Meclocycline sulfosalicylate salt	-5.1683
108929-04-0	Epinastine HCl	-5.1683
173352-39-1	Afobazole hydrochloride	-5.1683
1211441-98-3	Ribociclib	-5.168
186826-86-8	Moxifloxacin hydrochloride	-5.1675
552-41-0	Paeonol	-5.1674
13110-96-8	5-Acetylsalicylic acid	-5.1668
518048-05-0	Raltegravir	-5.1661
4852-22-6	Proanthocyanidins	-5.1658
58-46-8	Tetrabenazine	-5.1656
1269440-17-6	Encorafenib	-5.1655
89778-27-8	Toremifene citrate	-5.1654
79-57-2	Oxytetracycline	-5.1651
864750-70-9	Revefenacin	-5.1637
52205-73-9	Estramustine phosphate sodium	-5.1636
4852-22-6	Proanthocyanidins	-5.1635
94055-76-2	Suplatast tosilate	-5.1632
89778-26-7	Toremifene	-5.163
24729-96-2	Clindamycin phosphate	-5.1628
24390-14-5	Doxycycline hyclate	-5.1627
55079-83-9	Acitretin	-5.1626
59-87-0	Furacilin	-5.1621

1211441-98-3	Ribociclib	-5.1619
3200-06-4	Nafronyl oxalate	-5.1617
6620-60-6	Proglumide	-5.1612
123171-59-5	Cefepime Dihydrochloride Monohydrate	-5.161
6035-45-6	Folinic Acid Calcium Salt Pentahydrate	-5.1608
113-38-2	17-Beta-Estradiol-3,17-Dipropionate	-5.1607
164579-32-2	Pantoprazole Sodium Hydrate	-5.1605
1231929-97-7	Abemaciclib	-5.1605
93-14-1	Guaifenesin	-5.1605
7512-17-6	N-Acetyl-D-Glucosamine	-5.1604
127294-70-6	Balofloxacin	-5.1598
51-15-0	Pralidoxime Chloride	-5.1598
82159-09-9	Epalrestat	-5.1596
614-39-1	Procainamide hydrochloride	-5.1595
1490-04-6	DL-Menthol	-5.1593
242478-38-2	Solifenacin succinate	-5.159
38083-17-9	Climbazole	-5.159
4880-88-0	Eburnalritardo	-5.1587
154229-19-3	Abiraterone	-5.1584
79794-75-5	Loratadine	-5.1584
50-23-7	Hydrocortisone	-5.1583
551-92-8	Dimetridazole	-5.1582
2192-20-3	Hydroxyzine dihydrochloride	-5.1579
594839-88-0	Tafamidis	-5.1577
1230487-00-9	Siponimod	-5.1571
63675-72-9	Nisoldipine	-5.1569
175865-59-5	Valganciclovir hydrochloride	-5.1569
87233-61-2	Emedastine	-5.1569
138-37-4	Mafenide hydrochloride	-5.1568
59-47-2	Mephenesin	-5.1568
10246-75-0	Hydroxyzine Pamoate	-5.1567
6809-52-5	Teprenone	-5.1566
31282-04-9	Hygromycin B	-5.1565
926037-48-1	Radotinib	-5.1563
22199-08-2	Silver sulfadiazine	-5.1561
78613-38-4	Amorolfine hydrochloride	-5.1556
33564-30-6	Cefoxitin sodium	-5.1554
80-49-9	Homatropine Methylbromide	-5.155
68813-55-8	Oxantel Pamoate	-5.1549
164579-32-2	Pantoprazole Sodium Hydrate	-5.1547

141-94-6	Hexetidine	-5.1543
173352-39-1	Afobazole hydrochloride	-5.1538
112887-68-0	Raltitrexed	-5.1535
70356-03-5	Cefaclor monohydrate	-5.1531
107868-30-4	Exemestane	-5.1531
956104-40-8	Apalutamide	-5.153
6556-11-2	Inositol nicotinate	-5.153
1370468-36-2	Elbasvir	-5.1528
80-77-3	Chlormezanone	-5.1528
100986-85-4	Levofloxacin	-5.1527
138199-71-0	Levofloxacin Hemihydrate	-5.1527
95789-30-3	Cefotiam Hexetil Hydrochloride	-5.152
150915-40-5	Tirofiban hydrochloride monohydrate	-5.1518
82419-36-1	Ofloxacin	-5.1516
59338-87-3	Alizapride hydrochloride	-5.1514
485-49-4	(+)-Bicuculline	-5.1508
548-66-3	Drofenine hydrochloride	-5.1506
3685-84-5	Meclofenoxate hydrochloride	-5.1504
577-11-7	Docusate sodium	-5.1501
446-86-6	Azathioprine	-5.1498
163521-08-2	Vilazodone HCl	-5.1496
34031-32-8	Auranofin	-5.1495
2921-88-2	Clorpyrifos	-5.1494
56211-40-6	Torasemide	-5.1493
964-52-3	Moxisylyte hydrochloride	-5.1492
133-67-5	Trichlormethiazide	-5.1488
157810-81-6	Indinavir sulfate	-5.1486
74103-07-4	Ketorolac tromethamine salt	-5.1484
59338-87-3	Alizapride hydrochloride	-5.1484
93479-97-1	Glimepiride	-5.1481
118292-40-3	Tazarotene	-5.1478
51-03-6	Piperonyl butoxide	-5.1477
304-20-1	Hydralazine hydrochloride	-5.1468
58-46-8	Tetrabenazine	-5.1468
150322-43-3	Prasugrel	-5.1464
50264-69-2	Lonidamine	-5.1462
104-29-0	Chlorphenesin	-5.146
42461-84-7	Flunixin meglumin	-5.1457
157810-81-6	Indinavir sulfate	-5.1457
123447-62-1	Prulifloxacin	-5.1455
111025-46-8	Pioglitazone	-5.1452
62613-82-5	Oxiracetam	-5.1449

871038-72-1	Raltegravir potassium	-5.1446
150399-21-6	Balsalazide disodium salt dihydrate	-5.1442
67814-76-0	Isepamicin sulfate	-5.1442
50-04-4	Cortisone acetate	-5.1442
524-81-2	Mebhydroline	-5.1436
24356-60-3	Cephapirin Sodium	-5.1432
6153-33-9	Mebhydrolin napadisylate	-5.143
4360-12-7	Ajmaline	-5.1427
207844-01-7	Mitiglinide calcium	-5.1426
108929-04-0	Epinastine HCl	-5.1422
1300-94-3	Amylmetacresol	-5.142
132-17-2	Benztropine mesylate	-5.142
29908-03-0	Ademetionine	-5.142
3858-89-7	Chloroprocaine HCl	-5.1418
5370-01-4	Mexiletine hydrochloride	-5.1417
123447-62-1	Prulifloxacin	-5.1413
304-84-7	Ethamivan	-5.1409
439239-90-4	Lasmiditan	-5.1408
611-75-6	Bromhexine hydrochloride	-5.1406
51460-26-5	Carbazochrome sodium sulfonate	-5.1405
435-97-2	Phenprocoumon	-5.1398
321-30-2	Adenine sulfate	-5.1389
33386-08-2	Buspirone hydrochloride	-5.1389
54029-12-8	Albendazole Oxide	-5.1388
526-18-1	Osalmid	-5.1388
4852-22-6	Proanthocyanidins	-5.1387
24729-96-2	Clindamycin phosphate	-5.1386
16773-42-5	Ornidazole	-5.1385
73-24-5	Adenine	-5.1383
515-64-0	Sulfisomidine	-5.1383
16773-42-5	Ornidazole	-5.1381
112811-59-3	Gatifloxacin	-5.1376
562-10-7	Doxylamine succinate	-5.1376
33386-08-2	Buspirone hydrochloride	-5.1373
2468-21-5	Catharanthine	-5.1371
73963-72-1	Cilostazol	-5.1371
55981-09-4	Nitazoxanide	-5.137
13614-98-7	Minocycline hydrochloride	-5.1368
155-41-9	Methscopolamine	-5.1366
71-81-8	Isopropamide Iodide	-5.1357
94055-76-2	Suplatast tosilate	-5.1356
3200-06-4	Nafronyl oxalate	-5.1354
78613-38-4	Amorolfine hydrochloride	-5.1348

116-43-8	Succinylsulfathiazole	-5.1348
596-51-0	Glycopyrrolate	-5.1347
6620-60-6	Proglumide	-5.1345
122841-12-7	Cefoselis Sulfate	-5.1339
101-26-8	Mestinon	-5.1338
54-96-6	Amifampridine	-5.1336
34140-59-5	Trimebutine maleate	-5.1336
39133-31-8	Trimebutine	-5.1336
65473-14-5	Naftifine hydrochloride	-5.1334
56211-40-6	Torasemide	-5.1328
435-97-2	Phenprocoumon	-5.1327
6160-12-9	(-)-Sparteine sulfate pentahydrate	-5.1322
2468-21-5	Catharanthine	-5.1322
127-79-7	Sulfamerazine	-5.1321
53251-94-8	Pinaverium bromide	-5.1321
302-79-4	Tretinoin	-5.1319
29908-03-0	Ademetionine	-5.1318
72509-76-3	Felodipine	-5.1317
35212-22-7	Ipriflavone	-5.1313
24316-19-6	Cephalotaxine	-5.1313
125-02-0	Prednisolone phosphate sodium	-5.1311
157810-81-6	Indinavir sulfate	-5.131
22457-89-2	Benfotiamine	-5.1308
53797-35-6	Ribostamycin sulfate	-5.1306
34140-59-5	Trimebutine maleate	-5.1304
39133-31-8	Trimebutine	-5.1304
56391-57-2	Netilmicin sulfate	-5.1303
106730-54-5	Olprinone	-5.1301
127-69-5	Sulfisoxazole	-5.1299
32449-92-6	Dicurone	-5.1294
60628-96-8	Bifonazole	-5.1291
1231929-97-7	Abemaciclib	-5.1289
305-03-3	Chloroambucil	-5.1288
172152-50-0	Ilaprazole sodium	-5.1287
1454846-35-5	Lorlatinib	-5.128
3374-05-8	Nalidixic acid sodium salt	-5.1278
389-08-2	Nalidixic acid	-5.1278
24316-19-6	Cephalotaxine	-5.1277
6106-81-6	Scopolamine N-oxide HBr	-5.1274
577-11-7	Docusate sodium	-5.1274
90098-04-7	Rebamipide	-5.1272

25913-34-2	Tinoridine hydrochloride	-5.1271
123524-52-7	Azelnidipine	-5.127
1722-62-9	Mepivacaine hydrochloride	-5.1264
577-11-7	Docusate sodium	-5.126
697761-98-1	Elvitegravir	-5.1259
59937-28-9	Malotilate	-5.1257
112887-68-0	Raltitrexed	-5.1251
25775-90-0	Zucapsaicin	-5.1251
51-48-9	L-Thyroxine	-5.1251
178307-42-1	Revaprazan hydrochloride	-5.1246
84611-23-4	Erdosteine	-5.1241
6024-85-7	Tetrahydropalmatine hydrochloride	-5.1239
96847-55-1	Levomilnacipran	-5.1238
37517-28-5	Amikacin	-5.1235
32854-75-4	Lappaconitine	-5.1234
22368-21-4	Eupatilin	-5.1233
15676-16-1	Sulpiride	-5.1231
388082-77-7	Lapatinib Ditosylate	-5.1228
231277-92-2	Lapatinib	-5.1228
435-97-2	Phenprocoumon	-5.1226
51-15-0	Pralidoxime Chloride	-5.1224
394730-60-0	Boceprevir	-5.1223
965-52-6	Nifuroxazide	-5.1217
82419-36-1	Ofloxacin	-5.1217
10040-45-6	Sodium Picosulfate	-5.1209
136310-93-5	Tiotropium bromide	-5.1208
577-11-7	Docusate sodium	-5.1204
72432-10-1	Aniracetam	-5.12
583-03-9	Fenipentol	-5.1199
78247-49-1	Potassium guaiacolsulfonate	-5.1198
83-67-0	Theobromine	-5.1197
58-14-0	Pyrimethamine	-5.1192
91-85-0	Thonzylamine	-5.119
79-57-2	Oxytetracycline	-5.1187
74711-43-6	Zaltoprofen	-5.1187
57808-65-8	Closantel	-5.1184
123447-62-1	Prulifloxacin	-5.1181
435-97-2	Phenprocoumon	-5.1174
577778-58-6	Topiroxostat	-5.1169
364-62-5	5-Chloro-2-methoxyprocainamide	-5.1164
7232-21-5	Metoclopramide hydrochloride	-5.1164
721-50-6	Prilocaine	-5.1163
122-11-2	Sulfadimethoxine	-5.116

364-62-5	5-Chloro-2-methoxyprocainamide	-5.1153
7232-21-5	Metoclopramide hydrochloride	-5.1153
34140-59-5	Trimebutine maleate	-5.1152
39133-31-8	Trimebutine	-5.1152
167465-36-3	Zosuquidar 3HCl	-5.1152
84680-54-6	Enalaprilat Dihydrate	-5.1151
435-97-2	Phenprocoumon	-5.1151
98753-19-6	Cefpirome sulfate	-5.1149
890-38-0	2'-Deoxyinosine	-5.1148
4852-22-6	Proanthocyanidins	-5.1146
15622-65-8	Molindone hydrochloride	-5.1142
92134-98-0	Fosphenytoin sodium	-5.1139
1786-81-8	Propitocaine hydrochloride	-5.1139
389139-89-3	Omadacycline	-5.1135
1211441-98-3	Ribociclib	-5.1135
94055-76-2	Suplatast tosilate	-5.113
106730-54-5	Olprinone	-5.1128
53-03-2	Prednisone	-5.1127
23674-86-4	Difluprednate	-5.1126
38821-49-7	Carbidopa Hydrate	-5.112
119478-55-6	Danofloxacin mesylate	-5.1119
56796-39-5	Cefmetazole sodium	-5.1115
144143-96-4	Eprosartan Mesylate	-5.111
19387-91-8	Tinidazole	-5.1108
136470-78-5	Abacavir	-5.1105
52-86-8	Haloperidol	-5.1101
804-30-8	Fursultiamine	-5.11
364622-82-2	Doripenem Hydrate	-5.1099
71675-85-9	Amisulpride	-5.1098
388082-78-8	Lapatinib ditosylate monohydrate	-5.1096
3206-73-3	Thioctamide	-5.1093
157810-81-6	Indinavir sulfate	-5.1088
4852-22-6	Proanthocyanidins	-5.1088
53797-35-6	Ribostamycin sulfate	-5.1087
773-76-2	Capitrol	-5.1087
13614-98-7	Minocycline hydrochloride	-5.1087
52-86-8	Haloperidol	-5.1086
188062-50-2	Abacavir sulfate	-5.1084
78247-49-1	Potassium guaiacolsulfonate	-5.108
150322-43-3	Prasugrel	-5.1078
73816-42-9	Meclocycline sulfosalicylate salt	-5.1077
302962-49-8	Dasatinib	-5.1071

2152-44-5	Betamethasone 17-valerate	-5.1071
562-10-7	Doxylamine succinate	-5.1067
1108743-60-7	Entrectinib	-5.1067
101831-37-2	Diclazuril	-5.1065
65473-14-5	Naftifine hydrochloride	-5.1064
330784-47-9	Avanafil	-5.1064
83-88-5	Riboflavin	-5.1063
863127-77-9	Dasatinib monohydrate	-5.1059
3644-61-9	Tolperisone hydrochloride	-5.1056
141625-93-6	Dronedarone hydrochloride	-5.1056
4852-22-6	Proanthocyanidins	-5.1054
100986-85-4	Levofloxacin	-5.1046
138199-71-0	Levofloxacin Hemihydrate	-5.1046
721-50-6	Prilocaine	-5.1046
167465-36-3	Zosuquidar 3HCl	-5.1041
6160-12-9	(-)-Sparteine sulfate pentahydrate	-5.1034
882-09-7	Clofibric acid	-5.1031
82419-36-1	Ofloxacin	-5.1031
1226781-44-7	Omarigliptin	-5.1028
104-29-0	Chlorphenesin	-5.1027
141626-36-0	Dronedarone	-5.1026
1786-81-8	Propitocaine hydrochloride	-5.1025
29908-03-0	Ademetionine	-5.1023
150399-21-6	Balsalazide disodium salt dihydrate	-5.102
364622-82-2	Doripenem Hydrate	-5.1019
106730-54-5	Olprinone	-5.1015
172152-50-0	Ilaprazole sodium	-5.1011
4342-03-4	Dacarbazine	-5.101
2447-57-6	Sulfadoxine	-5.1009
55981-09-4	Nitazoxanide	-5.1008
53885-35-1	Ticlopidine hydrochloride	-5.1008
58947-95-8	DL-trihexyphenidyl hydrochloride	-5.1008
54029-12-8	Albendazole Oxide	-5.1007
186826-86-8	Moxifloxacin hydrochloride	-5.1007
6533-00-2	Norgestrel	-5.099
65666-07-1	Silymarin	-5.0987
111902-57-9	Temocapil	-5.0986
21187-98-4	Gliclazide	-5.098
132036-88-5	Ramosetron	-5.0973
596-51-0	Glycopyrrolate	-5.0973
552-41-0	Paeonol	-5.0972

4852-22-6	Proanthocyanidins	-5.097
57-67-0	Sulfaguanidine	-5.0965
475086-01-2	Selexipag	-5.0958
611-75-6	Bromhexine hydrochloride	-5.0957
93479-97-1	Glimepiride	-5.0951
110221-44-8	Temocapril HCl	-5.0947
22862-76-6	Anisomycin	-5.0946
59338-87-3	Alizapride hydrochloride	-5.0945
22368-21-4	Eupatilin	-5.0944
2066-89-9	Pasiniazid	-5.0943
1346242-81-6	Erdafitinib	-5.094
3200-06-4	Nafronyl oxalate	-5.094
610798-31-7	Icotinib	-5.0934
25655-41-8	Povidone-iodine	-5.0929
177036-94-1	Ambrisentan	-5.0928
364782-34-3	Cinacalcet hydrochloride	-5.0924
61-16-5	Methoxamine hydrochloride	-5.0923
58895-64-0	Nalmefene hydrochloride	-5.0923
6533-00-2	Norgestrel	-5.0922
320-67-2	5-Azacytidine	-5.092
40828-46-4	Suprofen	-5.0919
562-10-7	Doxylamine succinate	-5.0917
69004-03-1	Toltrazuril	-5.0917
161735-79-1	Rasagiline Mesylate	-5.0911
2259-96-3	Cyclothiazide	-5.091
50-03-3	Hydrocortisone acetate	-5.0907
123447-62-1	Prulifloxacin	-5.0907
1177-87-3	Dexamethason acetate	-5.0905
747-36-4	Hydroxychloroquine sulfate	-5.0905
23256-30-6	Nifurtimox	-5.0902
6153-33-9	Mebhydrolin napadisylate	-5.0898
136236-51-6	Rasagiline	-5.0896
95789-30-3	Cefotiam Hexetil Hydrochloride	-5.0893
62613-82-5	Oxiracetam	-5.0892
297-76-7	Ethynodiol diacetate	-5.0887
138452-21-8	Fexofenadine hydrochloride	-5.0886
524-81-2	Mebhydroline	-5.0886
24729-96-2	Clindamycin phosphate	-5.0885
25389-94-0	Kanamycin sulfate	-5.0885
55079-83-9	Acitretin	-5.0884
150322-43-3	Prasugrel	-5.0884
606143-52-6	Selumetinib	-5.0883

53-43-0	Dehydroepiandrosterone	-5.0881
161796-78-7	Esomeprazole Sodium	-5.0878
10246-75-0	Hydroxyzine Pamoate	-5.0872
5593-20-4	Betamethasone dipropionate	-5.0866
2192-20-3	Hydroxyzine dihydrochloride	-5.0866
38083-17-9	Climbazole	-5.0865
435-97-2	Phenprocoumon	-5.0861
54029-12-8	Albendazole Oxide	-5.0861
132-98-9	Penicillin V potassium salt	-5.0856
139110-80-8	Zanamivir	-5.0856
2259-96-3	Cyclothiazide	-5.0855
201530-41-8	Deferasirox	-5.0855
738-70-5	Trimethoprim	-5.0853
1300-94-3	Amylmetacresol	-5.0852
189188-57-6	Tegaserod maleate	-5.0851
66357-59-3	Ranitidine Hydrochloride	-5.085
66-76-2	Dicumarol	-5.0847
167221-71-8	Clevidipine butyrate	-5.0847
51-35-4	L-Hydroxyprolin	-5.0843
85721-33-1	Ciprofloxacin	-5.0843
93107-08-5	Ciprofloxacin hydrochloride	-5.0843
22252-43-3	Cefalexine EP IMpurity B	-5.084
26833-87-4	Homoharringtonine	-5.0839
552-94-3	Sasapyrine	-5.0832
30123-17-2	Tianeptine sodium	-5.0823
79794-75-5	Loratadine	-5.082
53-03-2	Prednisone	-5.0818
128794-94-5	Mycophenolate Mofetil	-5.0809
1051375-16-6	Dolutegravir	-5.0809
1490-04-6	DL-Menthol	-5.0806
1490-04-6	DL-Menthol	-5.0805
3380-34-5	Triclosan	-5.0805
31677-93-7	Bupropion hydrochloride	-5.0799
82586-52-5	Moexipril HCl	-5.0792
157810-81-6	Indinavir sulfate	-5.0792
56-69-9	5-hydroxytryptophan	-5.0778
3200-06-4	Nafronyl oxalate	-5.0774
24729-96-2	Clindamycin phosphate	-5.0773
4936-47-4	Nifuratel	-5.0772
95233-18-4	Atovaquone	-5.0769
127-56-0	Sulfacetamide sodium	-5.0768
6209-17-2	Sulfacetamide sodium salt hydrate	-5.0768

645-43-2	Guanethidine Monosulfate	-5.0755
32795-44-1	Acecainide	-5.0748
1492-18-8	Calcium folinate	-5.0747
496-67-3	Bromisoval	-5.0746
144-82-1	Sulfamethizole	-5.0744
39809-25-1	Penciclovir	-5.0741
54527-84-3	Nicardipine hydrochloride	-5.0741
6106-81-6	Scopolamine N-oxide HBr	-5.0738
82159-09-9	Epalrestat	-5.0736
141-94-6	Hexetidine	-5.0733
3200-06-4	Nafronyl oxalate	-5.0733
40828-46-4	Suprofen	-5.0732
57-67-0	Sulfaguanidine	-5.0729
562-10-7	Doxylamine succinate	-5.0722
611-75-6	Bromhexine hydrochloride	-5.072
27164-46-1	Cefazolin sodium salt	-5.0718
128-13-2	Ursodeoxycholic acid	-5.0716
1490-04-6	DL-Menthol	-5.0715
132-17-2	Benztropine mesylate	-5.0713
747-36-4	Hydroxychloroquine sulfate	-5.071
14028-44-5	Amoxapine	-5.0706
3200-06-4	Nafronyl oxalate	-5.0705
2557-49-5	Diflorasone	-5.0701
141626-36-0	Dronedarone	-5.0694
33125-97-2	Etomidate	-5.0689
1025504-45-3	Valbenazine	-5.0688
198904-31-3	Atazanavir	-5.0684
229975-97-7	Atazanavir sulfate	-5.0684
1263-89-4	Paromomycin Sulfate	-5.0683
59-47-2	Mephenesin	-5.068
95635-55-5	Ranolazine	-5.0679
95635-56-6	Ranolazine dihydrochloride	-5.0679
19408-46-9	Kasugamycin hydrochloride	-5.0677
6533-68-2	Scopolamine HBr trihydrate	-5.0674
39809-25-1	Penciclovir	-5.0671
82640-04-8	Raloxifene hydrochloride	-5.0671
54-91-1	Pipobroman	-5.0668
53-06-5	Cortisone	-5.0664
50-04-4	Cortisone acetate	-5.0662
985-13-7	Ethaverine hydrochloride	-5.0662
645-05-6	Altretamine	-5.0652
84625-61-6	Itraconazole	-5.0651

90-22-2	Valethamate bromide	-5.065
78247-49-1	Potassium guaiacolsulfonate	-5.0646
118288-08-7	Lafutidine	-5.0642
118288-08-7	Lafutidine	-5.0642
3200-06-4	Nafronyl oxalate	-5.0642
18883-66-4	Streptozocin	-5.0638
596-51-0	Glycopyrrolate	-5.0636
4852-22-6	Proanthocyanidins	-5.0636
71610-00-9	Cephalomannine	-5.0627
82586-55-8	Quinapril hydrochloride	-5.0626
64953-12-4	Latamoxef sodium	-5.0625
29908-03-0	Ademetionine	-5.0624
101-26-8	Mestinon	-5.0617
56-94-0	Demecarium bromide	-5.0613
75695-93-1	Isradipine	-5.0608
91161-71-6	Terbinafine	-5.0606
141625-93-6	Dronedarone hydrochloride	-5.0604
804-30-8	Fursultiamine	-5.0598
5003-48-5	Benorilate	-5.0596
58-71-9	Cephalothin sodium	-5.0588
1025687-58-4	R788 Disodium	-5.0583
901119-35-5	Fostamatinib	-5.058
57808-65-8	Closantel	-5.058
22457-89-2	Benfotiamine	-5.0572
59338-87-3	Alizapride hydrochloride	-5.057
4852-22-6	Proanthocyanidins	-5.0565
53797-35-6	Ribostamycin sulfate	-5.056
88889-14-9	Fosinopril sodium	-5.0558
184475-35-2	Gefitinib	-5.0557
94-62-2	Piperine	-5.0551
101477-54-7	Lomerizine hydrochloride	-5.0551
526-08-9	Sulfaphenazole	-5.055
242478-38-2	Solifenacin succinate	-5.0548
537-55-3	N-Acetyl-L-tyrosine	-5.0547
61-16-5	Methoxamine hydrochloride	-5.0547
116-43-8	Succinylsulfathiazole	-5.054
78613-38-4	Amorolfine hydrochloride	-5.0537
3858-89-7	Chloroprocaine HCl	-5.0537
518048-05-0	Raltegravir	-5.0533
138844-81-2	Ibandronate sodium	-5.053
459868-92-9	Rucaparib Phosphate	-5.0529
283173-50-2	Rucaparib	-5.0529

2447-57-6	Sulfadoxine	-5.0522
58-14-0	Pyrimethamine	-5.0522
118292-40-3	Tazarotene	-5.0518
61336-70-7	Amoxicillin trihydrate	-5.0517
26787-78-0	Amoxicillin	-5.0517
132810-10-7	Blonanserin	-5.0516
1263-89-4	Paromomycin Sulfate	-5.0515
167221-71-8	Clevidipine butyrate	-5.0513
130-26-7	Clioquinol	-5.0512
141-94-6	Hexetidine	-5.0511
84057-95-4	Ropivacaine	-5.051
98717-15-8	Ropivacaine hydrochloride	-5.051
65666-07-1	Silymarin	-5.0503
127-79-7	Sulfamerazine	-5.05
53164-05-9	Acemetacin	-5.05
10402-53-6	Eprazinone dihydrochloride	-5.0497
104-29-0	Chlorphenesin	-5.0496
606143-89-9	Binimetinib	-5.0492
14919-77-8	Benserazide hydrochloride	-5.0492
1508-75-4	Tropicamide	-5.0491
4394-00-7	Niflumic acid	-5.0488
548-73-2	Droperidol	-5.0484
101-26-8	Mestinon	-5.0482
1986-47-6	Tranylcypromine (2-PCPA) hydrochloride	-5.048
50264-69-2	Lonidamine	-5.0479
551-92-8	Dimetridazole	-5.0475
61-16-5	Methoxamine hydrochloride	-5.0472
1051375-16-6	Dolutegravir	-5.0469
60-56-0	Methimazole	-5.0466
220620-09-7	Tigecycline	-5.046
58-15-1	Aminophenazone	-5.0459
554-57-4	Methazolamide	-5.0455
78613-38-4	Amorolfine hydrochloride	-5.0453
167465-36-3	Zosuquidar 3HCl	-5.0452
52214-84-3	Ciprofibrate	-5.0449
22832-87-7	Miconazole nitrate	-5.0449
119478-55-6	Danofloxacin mesylate	-5.0446
1226781-44-7	Omarigliptin	-5.0442
141-94-6	Hexetidine	-5.0439
70458-96-7	Norfloxacin	-5.0435
143388-64-1	Naratriptan	-5.0435

97682-44-5	Irinotecan	-5.0434
501-68-8	Beclamide	-5.0431
94055-76-2	Suplatast tosilate	-5.0431
129722-12-9	Aripiprazole	-5.0429
15622-65-8	Molindone hydrochloride	-5.0428
65666-07-1	Silymarin	-5.042
1691249-45-2	zanubrutinib	-5.0418
149676-40-4	Pefloxacin Mesylate Dihydrate	-5.0417
14028-44-5	Amoxapine	-5.0416
10238-21-8	Glyburide	-5.0414
1226781-44-7	Omarigliptin	-5.0412
136572-09-3	Irinotecan hydrochloride trihydrate	-5.041
15622-65-8	Molindone hydrochloride	-5.041
164579-32-2	Pantoprazole Sodium Hydrate	-5.0409
54-31-9	Furosemide	-5.0408
163253-35-8	Sitafloxacin Hydrate	-5.0407
150322-43-3	Prasugrel	-5.0407
96847-55-1	Levomilnacipran	-5.0404
22888-70-6	Silibinin	-5.0404
22881-35-2	Famprofazone	-5.0403
98-92-0	Nicotinamide	-5.0403
106730-54-5	Olprinone	-5.04
19387-91-8	Tinidazole	-5.0399
6160-12-9	(-)-Sparteine sulfate pentahydrate	-5.0394
58-94-6	Chlorothiazide	-5.0393
70458-95-6	Pefloxacin Mesylate	-5.0393
22204-24-6	Pyrantel pamoate	-5.039
2259-96-3	Cyclothiazide	-5.039
1220-83-3	Sulfamonomethoxine	-5.0388
161796-78-7	Esomeprazole Sodium	-5.0386
73590-58-6	Omeprazole	-5.0386
4852-22-6	Proanthocyanidins	-5.0382
747-36-4	Hydroxychloroquine sulfate	-5.0382
985-13-7	Ethaverine hydrochloride	-5.0382
139110-80-8	Zanamivir	-5.0379
1354744-91-4	Naloxegol oxalate	-5.0369
80-32-0	Sulfachloropyridazine	-5.0369
33564-30-6	Cefoxitin sodium	-5.0368
104-29-0	Chlorphenesin	-5.0367
133-67-5	Trichlormethiazide	-5.0366

871700-17-3	Trametinib	-5.0365
18010-40-7	Bupivacaine hydrochloride	-5.0362
51-35-4	L-Hydroxyprolin	-5.0358
138530-94-6	R-(+)-Lansoprazole	-5.0358
103577-45-3	Lansoprazole	-5.0358
78755-81-4	Flumazenil	-5.0356
25913-34-2	Tinoridine hydrochloride	-5.0355
56-53-1	Diethylstilbestrol	-5.035
946075-13-4	Isavuconazonium sulfate	-5.035
603-50-9	Bisacodyl	-5.0344
5633-20-5	Oxybutynin	-5.0341
1508-65-2	Oxybutynin hydrochloride	-5.0341
139110-80-8	Zanamivir	-5.0336
59-47-2	Mephenesin	-5.0331
68767-14-6	Loxoprofen	-5.033
380843-75-4	Bosutinib	-5.033
53797-35-6	Ribostamycin sulfate	-5.0328
34140-59-5	Trimebutine maleate	-5.0322
39133-31-8	Trimebutine	-5.0322
1953-04-4	Galanthamine HBr	-5.0311
183319-69-9	Erlotinib hydrochloride	-5.0309
183321-74-6	Erlotinib	-5.0309
100986-85-4	Levofloxacin	-5.0309
138199-71-0	Levofloxacin Hemihydrate	-5.0309
583-03-9	Fenipentol	-5.0307
82419-36-1	Ofloxacin	-5.0306
202189-78-4	Bilastine	-5.0303
163680-77-1	Pazufloxacin Mesylate	-5.0303
84371-65-3	Mifepristone	-5.0302
705260-08-8	Vorapaxar sulfate	-5.03
95789-30-3	Cefotiam Hexetil Hydrochloride	-5.03
5593-20-4	Betamethasone dipropionate	-5.0296
4697-14-7	Ticarcillin disodium	-5.0293
129-06-6	Warfarin sodium	-5.0293
56391-57-2	Netilmicin sulfate	-5.0292
187227-45-8	Oseltamivir acid	-5.029
50370-12-2	Cefadroxil	-5.0289
102767-28-2	Levetiracetam	-5.0287
435-97-2	Phenprocoumon	-5.0287
127294-70-6	Balofloxacin	-5.0285
136310-93-5	Tiotropium bromide	-5.0285
129-06-6	Warfarin sodium	-5.0285
641571-10-0	Nilotinib	-5.028

141-94-6	Hexetidine	-5.0277
103-90-2	Acetaminophen	-5.0272
61-16-5	Methoxamine hydrochloride	-5.0272
28395-03-1	Bumetanide	-5.0269
405165-61-9	Besifloxacin HCl	-5.0269
129938-20-1	Dapoxetine HCl	-5.0265
153773-82-1	Ertapenem sodium	-5.0259
119478-55-6	Danofloxacin mesylate	-5.0259
150915-40-5	Tirofiban hydrochloride monohydrate	-5.0256
130018-77-8	Levocetirizine	-5.0255
83881-52-1	Cetirizine dihydrochloride	-5.0255
58-14-0	Pyrimethamine	-5.0255
7327-87-9	Dihydralazine sulphate	-5.0254
80-77-3	Chlormezanone	-5.0245
5633-20-5	Oxybutynin	-5.0245
1508-65-2	Oxybutynin hydrochloride	-5.0245
91296-87-6	Sarafloxacin hydrochloride	-5.0242
6533-68-2	Scopolamine HBr trihydrate	-5.0241
72-80-0	Chloquinan	-5.024
6024-85-7	Tetrahydropalmatine hydrochloride	-5.0239
7179-49-9	Lincomycin hydrochloride monohydrate	-5.0238
144-83-2	Sulfapyridine	-5.0237
74011-58-8	Enoxacin	-5.0237
84294-96-2	Enoxacin Sesquihydrate	-5.0237
133-67-5	Trichlormethiazide	-5.0236
163253-35-8	Sitafloxacin Hydrate	-5.0233
26833-87-4	Homoharringtonine	-5.0233
66357-59-3	Ranitidine Hydrochloride	-5.0227
859-18-7	Lincomycin hydrochloride	-5.0226
15622-65-8	Molindone hydrochloride	-5.0223
97-18-7	Bithionol	-5.0222
1490-04-6	DL-Menthol	-5.0215
1135-23-5	Hydroferulic acid	-5.0214
198904-31-3	Atazanavir	-5.0212
229975-97-7	Atazanavir sulfate	-5.0212
59937-28-9	Malotilate	-5.0211
13311-84-7	Flutamide	-5.0207
6160-12-9	(-)-Sparteine sulfate pentahydrate	-5.0204
26833-87-4	Homoharringtonine	-5.0203
946075-13-4	Isavuconazonium sulfate	-5.0202
147127-20-6	Tenofovir	-5.0201
4852-22-6	Proanthocyanidins	-5.0201
67-45-8	Furazolidone	-5.0195

65666-07-1	Silymarin	-5.0195
162011-90-7	Rofecoxib	-5.0191
22252-43-3	Cefalexine EP IMpurity B	-5.0181
435-97-2	Phenprocoumon	-5.0181
475086-01-2	Selexipag	-5.0178
59338-87-3	Alizapride hydrochloride	-5.0176
103890-78-4	Lacidipine	-5.0165
51333-22-3	Budesonide	-5.0164
1197953-54-0	Brigatinib	-5.0164
1405-41-0	Gentamycin sulfate	-5.0163
153559-49-0	Bexarotene	-5.0163
945667-22-1	Saxagliptin hydrate	-5.0162
908-54-3	Diminazene Aceturate	-5.0159
345909-26-4	Taurocholic acid sodium salt hydrate	-5.0154
115256-11-6	Dofetilide	-5.0153
35189-28-7	Norgestimate	-5.0152
1490-04-6	DL-Menthol	-5.0149
38083-17-9	Climbazole	-5.0148
4394-00-7	Niflumic acid	-5.0145
201530-41-8	Deferasirox	-5.0141
161973-10-0	Esomeprazole Magnesium	-5.0139
2398-81-4	Nicotinic acid N-oxide	-5.0134
31282-04-9	Hygromycin B	-5.0133
159989-65-8	Nelfinavir Mesylate	-5.0133
139110-80-8	Zanamivir	-5.0132
2259-96-3	Cyclothiazide	-5.0129
5534-09-8	Beclomethasone dipropionate	-5.0129
1137608-69-5	Telotristat Etiprate	-5.0125
864750-70-9	Revefenacin	-5.0123
138530-94-6	R-(+)-Lansoprazole	-5.0122
103577-45-3	Lansoprazole	-5.0122
141-94-6	Hexetidine	-5.0117
161973-10-0	Esomeprazole Magnesium	-5.0111
3366-95-8	Secnidazole	-5.0105
1405-10-3	Neomycin sulfate	-5.01
1405-41-0	Gentamycin sulfate	-5.0099
164579-32-2	Pantoprazole Sodium Hydrate	-5.009
33005-95-7	Tiaprofenic acid	-5.0087
554-57-4	Methazolamide	-5.0084
182431-12-5	Lomitapide	-5.0082
18883-66-4	Streptozocin	-5.0081

29908-03-0	Ademetionine	-5.0077
81-27-6	Sennoside A	-5.0072
804-30-8	Fursultiamine	-5.0072
50-23-7	Hydrocortisone	-5.0071
122892-31-3	Itopride hydrochloride	-5.0071
32449-92-6	Dicurone	-5.007
58-54-8	Ethacrynic acid	-5.0068
66-76-2	Dicumarol	-5.0067
3306-62-5	2-Aminobenzenesulfonamide	-5.0063
129-06-6	Warfarin sodium	-5.0061
965-52-6	Nifuroxazide	-5.0061
136-40-3	Phenazopyridine hydrochloride	-5.006
84611-23-4	Erdosteine	-5.0056
141-94-6	Hexetidine	-5.0048
59-47-2	Mephenesin	-5.0045
50924-49-7	Mizoribine	-5.0042
138452-21-8	Fexofenadine hydrochloride	-5.0041
673-06-3	D-Phenylalanine	-5.0039
92636-39-0	Cefminox Sodium	-5.0037
4429-63-4	Tabersonine	-5.0031
4852-22-6	Proanthocyanidins	-5.0025
286930-03-8	Fesoterodine fumarate	-5.0025
78613-38-4	Amorolfine hydrochloride	-5.0012
61336-70-7	Amoxicillin trihydrate	-5.001
26787-78-0	Amoxicillin	-5.001
51-05-8	Procaine hydrochloride	-5.001
59-46-1	Procaine	-5.001
22204-24-6	Pyrantel pamoate	-5.0009
94055-76-2	Suplatast tosilate	-5.0007
35554-44-0	Imazalil	-5.0004
364-98-7	Diazoxide	-5.0002
27164-46-1	Cefazolin sodium salt	-4.9998
73-48-3	Bendroflumethiazide	-4.9996
946075-13-4	Isavuconazonium sulfate	-4.999
456-59-7	Cyclandelate	-4.9985
17737-65-4	Clonixin	-4.9982
128607-22-7	Ospemifene	-4.9981
31282-04-9	Hygromycin B	-4.9979
50-99-7	D(+)-Glucose	-4.9977
1229194-11-9	Edoxaban Tosylate Monohydrate	-4.9977
3543-75-7	Bendamustine hydrochloride	-4.9976
33005-95-7	Tiaprofenic acid	-4.9974

85721-33-1	Ciprofloxacin	-4.9973
93107-08-5	Ciprofloxacin hydrochloride	-4.9973
155-41-9	Methscopolamine	-4.9968
2203-97-6	Hydrocortisone 21-hemisuccinate	-4.9967
132810-10-7	Blonanserin	-4.9966
6809-52-5	Teprenone	-4.9965
132-60-5	Cinchophen	-4.9958
54-36-4	Metyrapone	-4.9955
102767-28-2	Levetiracetam	-4.9954
27741-01-1	Geniposidic Acid	-4.9951
926037-48-1	Radotinib	-4.9949
175865-59-5	Valganciclovir hydrochloride	-4.9944
54-71-7	(+)-Pilocarpine hydrochloride	-4.9942
22204-24-6	Pyrantel pamoate	-4.9941
29342-05-0	Ciclopirox	-4.9941
41621-49-2	Ciclopirox ethanolamine	-4.9941
119478-55-6	Danofloxacin mesylate	-4.9935
148-72-1	Pilocarpine nitrate	-4.9933
59338-87-3	Alizapride hydrochloride	-4.9925
1231930-82-7	LY2835219	-4.9922
2259-96-3	Cyclothiazide	-4.992
926037-48-1	Radotinib	-4.9919
141-94-6	Hexetidine	-4.9908
912273-65-5	Edoxaban	-4.9908
577778-58-6	Topiroxostat	-4.9906
485-49-4	(+)-Bicuculline	-4.9901
380843-75-4	Bosutinib	-4.9896
54-85-3	Isoniazid	-4.9888
4852-22-6	Proanthocyanidins	-4.9883
70797-11-4	Cefpiramide acid	-4.9882
157810-81-6	Indinavir sulfate	-4.9879
57-68-1	Sulfamethazine	-4.9872
132-60-5	Cinchophen	-4.9869
151533-22-1	Levomefolate calcium	-4.9869
96847-55-1	Levomilnacipran	-4.9868
161973-10-0	Esomeprazole Magnesium	-4.9866
330808-88-3	Vardenafil hydrochloride trihydrate	-4.9866
122-11-2	Sulfadimethoxine	-4.9865
78967-07-4	Mofezolac	-4.9863
101152-94-7	Milnacipran hydrochloride	-4.9862
446-86-6	Azathioprine	-4.986
123524-52-7	Azelnidipine	-4.9855

119478-55-6	Danofloxacin mesylate	-4.985
1137608-69-5	Telotristat Etiprate	-4.9847
4936-47-4	Nifuratel	-4.9844
23327-57-3	Nefopam hydrochloride	-4.9833
13171-25-0	Trimetazidine dihydrochloride	-4.9829
594839-88-0	Tafamidis	-4.9827
123441-03-2	Rivastigmine	-4.9826
41340-25-4	Etodolac	-4.9818
162011-90-7	Rofecoxib	-4.9817
129101-54-8	Rivastigmine tartrate	-4.9817
515-64-0	Sulfisomidine	-4.9816
3485-62-9	Clidinium bromide	-4.9815
1110813-31-4	Dacomitinib	-4.9814
157810-81-6	Indinavir sulfate	-4.9811
6112-76-1	6-Mercaptopurine monohydrate	-4.9809
50-44-2	Mercaptopurine	-4.9806
61-68-7	Mefenamic acid	-4.9805
50-99-7	D(+)-Glucose	-4.9804
94-16-6	Sodium 4-aminohippurate Hydrate	-4.9802
61-78-9	4-Aminohippuric Acid	-4.9802
51940-44-4	Pipemidic acid	-4.9802
95635-55-5	Ranolazine	-4.9802
95635-56-6	Ranolazine dihydrochloride	-4.9799
98-96-4	Pyrazinamide	-4.9799
22254-24-6	Ipratropium Bromide	-4.9798
66985-17-9	Ipratropium bromide monohydrate	-4.9798
3485-62-9	Clidinium bromide	-4.9798
172152-50-0	Ilaprazole sodium	-4.9795
54527-84-3	Nicardipine hydrochloride	-4.9789
164579-32-2	Pantoprazole Sodium Hydrate	-4.9787
123447-62-1	Prulifloxacin	-4.9786
70458-96-7	Norfloxacin	-4.9784
432-60-0	Allylestrenol	-4.9783
1492-18-8	Calcium folinate	-4.9783
515-64-0	Sulfisomidine	-4.978
526-08-9	Sulfaphenazole	-4.9778
10040-45-6	Sodium Picosulfate	-4.9774
132539-06-1	Olanzapine	-4.9773
41294-56-8	Alfacalcidol	-4.9771
84294-96-2	Enoxacin Sesquihydrate	-4.9765
38083-17-9	Climbazole	-4.9765

157810-81-6	Indinavir sulfate	-4.9763
74011-58-8	Enoxacin	-4.9759
71320-77-9	Moclobemide	-4.9758
149022-22-0	Amikacin Sulfate Salt	-4.9754
68767-14-6	Loxoprofen	-4.975
66-84-2	Glucosamine hydrochloride	-4.9748
41294-56-8	Alfacalcidol	-4.9746
78613-38-4	Amorolfine hydrochloride	-4.9744
1490-04-6	DL-Menthol	-4.9743
1146-99-2	chlophenadione	-4.9742
22161-81-5	(S)-(+)-Ketoprofen	-4.9742
22071-15-4	Ketoprofen	-4.9742
912-60-7	Noscapine hydrochloride	-4.9734
6055-19-2	Cyclophosphamide monohydrate	-4.9732
13710-19-5	Tolfenamic Acid	-4.9731
1051375-19-9	Dolutegravir sodium	-4.973
56-04-2	Methylthiouracil	-4.9723
101831-37-2	Diclazuril	-4.9723
103577-45-3	Lansoprazole	-4.9722
78415-72-2	Milrinone	-4.9721
51037-30-0	Acipimox	-4.9717
56-53-1	Diethylstilbestrol	-4.9714
6055-19-2	Cyclophosphamide monohydrate	-4.9708
81-25-4	Cholic Acid	-4.9708
59338-87-3	Alizapride hydrochloride	-4.9694
16595-80-5	Levamisole hydrochloride	-4.9692
82752-99-6	Nefazodone hydrochloride	-4.9691
3200-06-4	Nafronyl oxalate	-4.9691
446-86-6	Azathioprine	-4.9687
4940-39-0	Chromocarbe	-4.9682
58-95-7	Vitamin E Acetate	-4.9682
12650-69-0	Mupirocin	-4.9679
82956-11-4	Nafamostat mesylate	-4.9677
89-25-8	Edaravone	-4.9674
53-43-0	Dehydroepiandrosterone	-4.9673
60-12-8	Phenethyl alcohol	-4.9672
127-69-5	Sulfisoxazole	-4.9669
23256-30-6	Nifurtimox	-4.9664
554-57-4	Methazolamide	-4.9664
148-72-1	Pilocarpine nitrate	-4.9662
7759-35-5	Nestoron	-4.9661
97-53-0	Eugenol	-4.9658

1300-94-3	Amylmetacresol	-4.9657
131707-23-8	Arbidol hydrochloride	-4.9652
112885-42-4	Mosapride Citrate	-4.9652
54-71-7	(+)-Pilocarpine hydrochloride	-4.965
95789-30-3	Cefotiam Hexetil Hydrochloride	-4.965
20123-80-2	Calcium dobesilate	-4.9649
871038-72-1	Raltegravir potassium	-4.9648
10402-53-6	Eprazinone dihydrochloride	-4.9648
435-97-2	Phenprocoumon	-4.9644
496-67-3	Bromisoval	-4.9641
99755-59-6	Rotigotine	-4.9638
1135-23-5	Hydroferulic acid	-4.9637
30751-05-4	Troxipide	-4.9635
6035-45-6	Folinic Acid Calcium Salt Pentahydrate	-4.9634
2624-44-4	Ethamsylate	-4.9634
912-60-7	Noscapine hydrochloride	-4.9632
58-94-6	Chlorothiazide	-4.963
172152-50-0	Ilaprazole sodium	-4.9627
172152-50-0	Ilaprazole sodium	-4.9626
29908-03-0	Ademetionine	-4.9624
4852-22-6	Proanthocyanidins	-4.9621
51333-22-3	Budesonide	-4.9617
4852-22-6	Proanthocyanidins	-4.9617
518048-05-0	Raltegravir	-4.9616
577778-58-6	Topiroxostat	-4.9613
80-32-0	Sulfachloropyridazine	-4.9612
26833-87-4	Homoharringtonine	-4.9607
56-69-9	5-hydroxytryptophan	-4.9606
122-11-2	Sulfadimethoxine	-4.9605
890-38-0	2'-Deoxyinosine	-4.9603
62613-82-5	Oxiracetam	-4.96
51-15-0	Pralidoxime Chloride	-4.9599
127294-70-6	Balofloxacin	-4.9598
1226781-44-7	Omarigliptin	-4.9598
139110-80-8	Zanamivir	-4.9598
29908-03-0	Ademetionine	-4.9598
17440-83-4	Amiloride hydrochloride dihydrate	-4.9597
127-69-5	Sulfisoxazole	-4.9597
1722-62-9	Mepivacaine hydrochloride	-4.9597
1257044-40-8	ABT199	-4.9596
210353-53-0	Gemifloxacin?mesylate	-4.9595

345909-26-4	Taurocholic acid sodium salt hydrate	-4.9594
641571-10-0	Nilotinib	-4.9589
141-94-6	Hexetidine	-4.9588
58-54-8	Ethacrynic acid	-4.9586
73590-85-9	Omeprazole sulfide	-4.9573
1490-04-6	DL-Menthol	-4.9571
141-94-6	Hexetidine	-4.957
50370-12-2	Cefadroxil	-4.9569
4342-03-4	Dacarbazine	-4.9568
596-51-0	Glycopyrrolate	-4.9566
4852-22-6	Proanthocyanidins	-4.9561
67814-76-0	Isepamicin sulfate	-4.9554
4800-94-6	Carbenicillin disodium	-4.9548
58-71-9	Cephalothin sodium	-4.9547
31677-93-7	Bupropion hydrochloride	-4.9547
29908-03-0	Ademetionine	-4.9545
60-54-8	Tetracycline	-4.9541
443913-73-3	Vandetanib	-4.9537
596-51-0	Glycopyrrolate	-4.9536
129-06-6	Warfarin sodium	-4.9535
537-55-3	N-Acetyl-L-tyrosine	-4.9534
16773-42-5	Ornidazole	-4.9534
124-94-7	Triamcinolone	-4.9532
31430-15-6	Flubendazole	-4.953
139110-80-8	Zanamivir	-4.9529
53797-35-6	Ribostamycin sulfate	-4.9528
7081-44-9	Cloxacillin sodium monohydrate	-4.9525
357-08-4	Naloxone hydrochloride	-4.9524
35554-44-0	Imazalil	-4.952
65666-07-1	Silymarin	-4.9519
58-94-6	Chlorothiazide	-4.9517
23674-86-4	Difluprednate	-4.9513
76-90-4	Mepenzolate Bromide	-4.9513
79902-63-9	Simvastatin	-4.9511
58-14-0	Pyrimethamine	-4.9507
73-48-3	Bendroflumethiazide	-4.9499
65666-07-1	Silymarin	-4.9497
54-11-5	Nicotine	-4.9496
547-44-4	Sulfanilylurea	-4.9495
71320-77-9	Moclobemide	-4.9492
6533-68-2	Scopolamine HBr trihydrate	-4.9491
878143-33-0	Dalasetron Mesylate Hydrate	-4.949
172152-50-0	Ilaprazole sodium	-4.9489

747-36-4	Hydroxychloroquine sulfate	-4.9487
547-44-4	Sulfanilylurea	-4.9485
80-32-0	Sulfachloropyridazine	-4.9483
95789-30-3	Cefotiam Hexetil Hydrochloride	-4.9483
7280-37-7	Estropipate	-4.9482
141-94-6	Hexetidine	-4.9479
64953-12-4	Latamoxef sodium	-4.9476
987-78-0	Citicoline	-4.9469
577-11-7	Docusate sodium	-4.9469
157810-81-6	Indinavir sulfate	-4.9467
65-31-6	Nicotine Ditartrate	-4.9466
3759-92-0	Furaltadone hydrochloride	-4.9464
305-03-3	Chloroambucil	-4.9462
908-54-3	Diminazene Aceturate	-4.9459
81-27-6	Sennoside A	-4.9458
33818-15-4	Citicoline sodium	-4.9457
115939-25-8	Salvianolic acid B	-4.9453
56211-40-6	Torasemide	-4.9451
53-06-5	Cortisone	-4.9449
4852-22-6	Proanthocyanidins	-4.9448
22254-24-6	Ipratropium Bromide	-4.9442
66985-17-9	Ipratropium bromide monohydrate	-4.9442
1354744-91-4	Naloxegol oxalate	-4.944
56391-57-2	Netilmicin sulfate	-4.9436
64485-93-4	Cefotaxime sodium	-4.9436
17696-69-4	Hematoporphyrin dihydrochloride	-4.9433
22888-70-6	Silibinin	-4.9432
366789-02-8	Rivaroxaban	-4.9431
54527-84-3	Nicardipine hydrochloride	-4.9429
50-99-7	D(+)-Glucose	-4.9429
71125-38-7	Meloxicam	-4.9427
946075-13-4	Isavuconazonium sulfate	-4.9423
133-67-5	Trichlormethiazide	-4.9421
54-85-3	Isoniazid	-4.942
4852-22-6	Proanthocyanidins	-4.9417
101477-54-7	Lomerizine hydrochloride	-4.9415
526-18-1	Osalmid	-4.9406
6385-02-0	Meclofenamate Sodium	-4.9403
357-08-4	Naloxone hydrochloride	-4.9403
2152-44-5	Betamethasone 17-valerate	-4.94
6533-68-2	Scopolamine HBr trihydrate	-4.94
21736-83-4	Spectinomycin dihydrochloride	-4.9399

4852-22-6	Proanthocyanidins	-4.9399
469-77-2	Spiculisporic Acid	-4.9398
15622-65-8	Molindone hydrochloride	-4.9397
17560-51-9	Metolazone	-4.9394
84625-61-6	Itraconazole	-4.9393
936727-05-8	Lumacaftor	-4.9392
17696-69-4	Hematoporphyrin dihydrochloride	-4.9389
330784-47-9	Avanafil	-4.9387
71125-38-7	Meloxicam	-4.9386
67-20-9	Nitrofurantoin	-4.9386
69655-05-6	Didanosine	-4.9384
155-41-9	Methscopolamine	-4.9381
90-22-2	Valethamate bromide	-4.9379
98079-52-8	Lomefloxacin hydrochloride	-4.9376
98079-51-7	Lomefloxacin	-4.9376
364782-34-3	Cinacalcet hydrochloride	-4.9374
118292-40-3	Tazarotene	-4.9372
141-94-6	Hexetidine	-4.937
141-94-6	Hexetidine	-4.9364
154598-52-4	Efavirenz	-4.9361
23142-01-0	Carbetapentane citrate	-4.936
149676-40-4	Pefloxacin Mesylate Dihydrate	-4.9353
70458-95-6	Pefloxacin Mesylate	-4.9344
124858-35-1	Nadifloxacin	-4.9343
64953-12-4	Latamoxef sodium	-4.9337
738-70-5	Trimethoprim	-4.9334
577-11-7	Docusate sodium	-4.9327
304-84-7	Ethamivan	-4.9324
68767-14-6	Loxoprofen	-4.9323
1405-10-3	Neomycin sulfate	-4.932
1289023-67-1	BMS927711	-4.9318
2259-96-3	Cyclothiazide	-4.9316
946075-13-4	Isavuconazonium sulfate	-4.9316
111902-57-9	Temocapil	-4.9315
110221-44-8	Temocapril HCl	-4.9315
912-60-7	Noscapine hydrochloride	-4.9313
93106-60-6	Enrofloxacin	-4.9312
10246-75-0	Hydroxyzine Pamoate	-4.9305
12650-69-0	Mupirocin	-4.9303
7083-71-8	(+)-Emetine dihydrochloride hydrate	-4.9302
16595-80-5	Levamisole hydrochloride	-4.93
118288-08-7	Lafutidine	-4.93

1051375-16-6	Dolutegravir	-4.9295
2192-20-3	Hydroxyzine dihydrochloride	-4.9293
210353-53-0	Gemifloxacin?mesylate	-4.9292
501-68-8	Beclamide	-4.9284
596-51-0	Glycopyrrolate	-4.9282
3206-73-3	Thioctamide	-4.9276
99464-64-9	Ampiroxicam	-4.9274
1247-42-3	Meprednisone	-4.927
82419-36-1	Ofloxacin	-4.927
82419-36-1	Ofloxacin	-4.9267
57-66-9	Probenecid	-4.9265
170105-16-5	Imidafenacin	-4.9264
100986-85-4	Levofloxacin	-4.9264
138199-71-0	Levofloxacin Hemihydrate	-4.9264
52205-73-9	Estramustine phosphate sodium	-4.9263
15686-71-2	Cephalexin	-4.926
22254-24-6	Ipratropium Bromide	-4.9258
66985-17-9	Ipratropium bromide monohydrate	-4.9258
552-94-3	Sasapyrine	-4.9257
78415-72-2	Milrinone	-4.9255
112885-42-4	Mosapride Citrate	-4.9253
17696-69-4	Hematoporphyrin dihydrochloride	-4.9253
7083-71-8	(+)-Emetine dihydrochloride hydrate	-4.9242
1811-28-5	Proflavine Hemisulfate	-4.924
853-23-6	Dehydroepiandrosterone acetate	-4.9238
30123-17-2	Tianeptine sodium	-4.9238
24729-96-2	Clindamycin phosphate	-4.9234
57-67-0	Sulfaguanidine	-4.9233
50-44-2	Mercaptopurine	-4.9225
1231929-97-7	Abemaciclib	-4.9225
80-32-0	Sulfachloropyridazine	-4.9221
84680-54-6	Enalaprilat Dihydrate	-4.9219
6112-76-1	6-Mercaptopurine monohydrate	-4.9216
56-47-3	Deoxycorticosterone acetate	-4.9212
29908-03-0	Ademetionine	-4.9211
79660-72-3	Fleroxacin	-4.9211
926037-48-1	Radotinib	-4.9208
596-51-0	Glycopyrrolate	-4.9207
56-69-9	5-hydroxytryptophan	-4.9202
7280-37-7	Estropipate	-4.9201
956697-53-3	NVP-LDE225	-4.92

515-64-0	Sulfisomidine	-4.9198
42540-40-9	Cefamandole nafate	-4.9197
40828-46-4	Suprofen	-4.9194
1490-04-6	DL-Menthol	-4.9193
21462-39-5	Clindamycin HCl	-4.9192
83919-23-7	Mometasone furoate	-4.9192
88678-31-3	Liranaftate	-4.9191
6533-68-2	Scopolamine HBr trihydrate	-4.919
51333-22-3	Budesonide	-4.9187
131707-23-8	Arbidol hydrochloride	-4.9186
89226-50-6	Manidipine	-4.9185
17896-21-8	Citiolone	-4.9181
80-49-9	Homatropine Methylbromide	-4.918
163521-12-8	Vilazodone	-4.9174
141-94-6	Hexetidine	-4.9174
89226-75-5	Manidipine dihydrochloride	-4.9173
17696-69-4	Hematoporphyrin dihydrochloride	-4.9172
6506-37-2	Nimorazole	-4.9172
2259-96-3	Cyclothiazide	-4.917
141-94-6	Hexetidine	-4.9169
2447-57-6	Sulfadoxine	-4.9164
22373-78-0	Monensin sodium salt	-4.9155
94-26-8	Butylparaben	-4.9155
31282-04-9	Hygromycin B	-4.9151
29908-03-0	Ademetionine	-4.9145
133-67-5	Trichlormethiazide	-4.9142
6024-85-7	Tetrahydropalmatine hydrochloride	-4.9136
2135-17-3	Flumethasone	-4.9134
112665-43-7	Seratrodast	-4.9133
78613-38-4	Amorolfine hydrochloride	-4.9115
3200-06-4	Nafronyl oxalate	-4.9113
1098-97-1	Pyrithioxin	-4.911
72432-10-1	Aniracetam	-4.9103
29908-03-0	Ademetionine	-4.9091
6533-68-2	Scopolamine HBr trihydrate	-4.909
2259-96-3	Cyclothiazide	-4.9085
35554-44-0	Imazalil	-4.908
141-94-6	Hexetidine	-4.9079
69659-80-9	Tanshinone IIA sulfonate sodium	-4.9078
95789-30-3	Cefotiam Hexetil Hydrochloride	-4.9072
4852-22-6	Proanthocyanidins	-4.9072
31282-04-9	Hygromycin B	-4.9072
129618-40-2	Nevirapine	-4.907

83-73-8	Iodoquinol	-4.907
90-22-2	Valethamate bromide	-4.9069
723-46-6	Sulfamethoxazole	-4.9064
469-77-2	Spiculisporic Acid	-4.9062
171599-83-0	Sildenafil citrate	-4.9059
80433-71-2	Calcium Levofolinate	-4.9057
36703-88-5	Isoprinosine	-4.9056
58-63-9	Inosine	-4.9056
475-67-2	S-Isocorydine(+)	-4.9052
36703-88-5	Isoprinosine	-4.9047
58-63-9	Inosine	-4.9047
36703-88-5	Isoprinosine	-4.9044
58-63-9	Inosine	-4.9044
29608-49-9	Almitrine dimesylate	-4.9044
304-20-1	Hydralazine hydrochloride	-4.9041
12650-69-0	Mupirocin	-4.9041
22839-47-0	Aspartame	-4.9037
3206-73-3	Thioctamide	-4.9035
54-11-5	Nicotine	-4.9028
23142-01-0	Carbetapentane citrate	-4.9025
65-31-6	Nicotine Ditartrate	-4.9022
1230487-00-9	Siponimod	-4.9018
63-74-1	Sulfanilamide	-4.9017
173352-39-1	Afobazole hydrochloride	-4.9006
89226-50-6	Manidipine	-4.8998
330784-47-9	Avanafil	-4.8997
485-49-4	(+)-Bicuculline	-4.8996
136-40-3	Phenazopyridine hydrochloride	-4.8994
89226-75-5	Manidipine dihydrochloride	-4.8992
127-56-0	Sulfacetamide sodium	-4.8989
6209-17-2	Sulfacetamide sodium salt hydrate	-4.8989
2295-58-1	Flopropione	-4.8987
313-67-7	Aristolochic Acid	-4.8978
138452-21-8	Fexofenadine hydrochloride	-4.8965
24729-96-2	Clindamycin phosphate	-4.8964
198904-31-3	Atazanavir	-4.8961
229975-97-7	Atazanavir sulfate	-4.8961
122892-31-3	Itopride hydrochloride	-4.8959
606143-89-9	Binimetinib	-4.8959
90-22-2	Valethamate bromide	-4.8958
54527-84-3	Nicardipine hydrochloride	-4.8956
59-47-2	Mephenesin	-4.8949

134308-13-7	Tolcapone	-4.8949
122-11-2	Sulfadimethoxine	-4.8942
32449-92-6	Dicurone	-4.8938
141-94-6	Hexetidine	-4.8937
59338-87-3	Alizapride hydrochloride	-4.8933
22839-47-0	Aspartame	-4.8933
6112-76-1	6-Mercaptopurine monohydrate	-4.8926
320-67-2	5-Azacytidine	-4.8923
1490-04-6	DL-Menthol	-4.8918
59-87-0	Furacilin	-4.8917
485-49-4	(+)-Bicuculline	-4.8915
2192-20-3	Hydroxyzine dihydrochloride	-4.8915
132810-10-7	Blonanserin	-4.8913
127294-70-6	Balofloxacin	-4.8912
3759-92-0	Furaltadone hydrochloride	-4.8909
76494-51-4	Ligustrazine hydrochloride	-4.8907
31282-04-9	Hygromycin B	-4.8907
10246-75-0	Hydroxyzine Pamoate	-4.8906
130018-77-8	Levocetirizine	-4.8904
83881-52-1	Cetirizine dihydrochloride	-4.8904
937272-79-2	Pacritinib	-4.8898
4940-39-0	Chromocarbe	-4.8894
10405-02-4	Trospium chloride	-4.889
10605-21-7	Carbendazim	-4.8889
1953-04-4	Galanthamine HBr	-4.8883
305-03-3	Chloroambucil	-4.8881
131707-23-8	Arbidol hydrochloride	-4.8875
143388-64-1	Naratriptan	-4.8874
61-78-9	4-Aminohippuric Acid	-4.8869
68767-14-6	Loxoprofen	-4.8868
61-68-7	Mefenamic acid	-4.8867
94-16-6	Sodium 4-aminohippurate Hydrate	-4.8866
50-44-2	Mercaptopurine	-4.8866
54856-23-4	Betahistine mesylate	-4.8865
5579-84-0	Betahistine dihydrochloride	-4.8865
6620-60-6	Proglumide	-4.8865
175865-59-5	Valganciclovir hydrochloride	-4.8863
5638-76-6	Betahistine	-4.8859
14698-29-4	Oxolinic acid	-4.8858
320-67-2	5-Azacytidine	-4.8855
4852-22-6	Proanthocyanidins	-4.885
99300-78-4	Venlafaxine hydrochloride	-4.8849
242478-38-2	Solifenacin succinate	-4.8848

157810-81-6	Indinavir sulfate	-4.8844
27591-69-1	Tilorone dihydrochloride	-4.8842
34031-32-8	Auranofin	-4.8841
19237-84-4	Prazosin hydrochloride	-4.884
116-43-8	Succinylsulfathiazole	-4.8839
54-96-6	Amifampridine	-4.8837
220620-09-7	Tigecycline	-4.8836
136790-76-6	Lubiprostone	-4.8835
82586-52-5	Moexipril HCl	-4.8832
853-23-6	Dehydroepiandrosterone acetate	-4.883
73590-85-9	Omeprazole sulfide	-4.8829
170105-16-5	Imidafenacin	-4.8828
987-78-0	Citicoline	-4.8826
126-07-8	Griseofulvin	-4.8819
547-44-4	Sulfanilylurea	-4.8813
10402-53-6	Eprazinone dihydrochloride	-4.8812
33818-15-4	Citicoline sodium	-4.8811
T5164	Cabozantinib hydrochloride (849217-68-1(free base))	-4.8811
849217-68-1	Cabozantinib	-4.8811
1140909-48-3	Cabozantinib Malate	-4.8811
6106-81-6	Scopolamine N-oxide HBr	-4.881
68767-14-6	Loxoprofen	-4.881
10402-53-6	Eprazinone dihydrochloride	-4.8808
127-56-0	Sulfacetamide sodium	-4.88
6209-17-2	Sulfacetamide sodium salt hydrate	-4.88
51037-30-0	Acipimox	-4.8799
405165-61-9	Besifloxacin HCl	-4.8796
2398-81-4	Nicotinic acid N-oxide	-4.8792
13171-25-0	Trimetazidine dihydrochloride	-4.879
57-66-9	Probenecid	-4.8788
68767-14-6	Loxoprofen	-4.8786
127-47-9	Retinyl acetate	-4.8785
94-62-2	Piperine	-4.8784
65666-07-1	Silymarin	-4.8781
79559-97-0	Sertraline hydrochloride	-4.8778
141-94-6	Hexetidine	-4.8774
475-67-2	S-Isocorydine(+)	-4.8773
4852-22-6	Proanthocyanidins	-4.8769
122-11-2	Sulfadimethoxine	-4.8766
67-03-8	Thiamine hydrochloride	-4.8766
856867-55-5	Tedizolid Phosphate	-4.8764
23142-01-0	Carbetapentane citrate	-4.8762

29908-03-0	Ademetionine	-4.8758
129497-78-5	Verteporfin	-4.8754
92134-98-0	Fosphenytoin sodium	-4.8753
161715-24-8	Tebipenem Pivoxil	-4.8751
178307-42-1	Revaprazan hydrochloride	-4.8749
1132935-63-7	Dasabuvir	-4.8745
444731-52-6	Pazopanib	-4.8738
54527-84-3	Nicardipine hydrochloride	-4.8736
635702-64-6	Pazopanib HCl	-4.8732
31282-04-9	Hygromycin B	-4.8729
149022-22-0	Amikacin Sulfate Salt	-4.8726
945667-22-1	Saxagliptin hydrate	-4.8725
639089-54-6	Tozasertib	-4.872
102518-79-6	(-)-Huperzine A	-4.8718
90098-04-7	Rebamipide	-4.8718
59-05-2	Methotrexate	-4.871
596-51-0	Glycopyrrolate	-4.8703
4852-22-6	Proanthocyanidins	-4.8695
129722-12-9	Aripiprazole	-4.8695
22881-35-2	Famprofazone	-4.8686
530-78-9	Flufenamic acid	-4.8685
67-45-8	Furazolidone	-4.8685
119478-55-6	Danofloxacin mesylate	-4.8684
110871-86-8	Sparfloxacin	-4.8682
50-65-7	Niclosamide	-4.8682
52432-72-1	Oxeladin citrate	-4.8679
1607799-13-2	bremelanotide	-4.8679
1953-04-4	Galanthamine HBr	-4.8674
16595-80-5	Levamisole hydrochloride	-4.8673
187227-45-8	Oseltamivir acid	-4.8667
1405-37-4	Capastat sulfate	-4.8666
16676-29-2	Naltrexone hydrochloride	-4.8666
4852-22-6	Proanthocyanidins	-4.8655
54029-12-8	Albendazole Oxide	-4.8653
13609-67-1	Hydrocortisone butyrate	-4.8651
94-26-8	Butylparaben	-4.8647
30484-77-6	Flunarizine dihydrochloride	-4.8647
14028-44-5	Amoxapine	-4.8643
23256-30-6	Nifurtimox	-4.8642
66-84-2	Glucosamine hydrochloride	-4.8638
485-49-4	(+)-Bicuculline	-4.8638

4852-22-6	Proanthocyanidins	-4.8637
56-47-3	Deoxycorticosterone acetate	-4.8634
26171-23-3	Tolmetin	-4.8634
439239-90-4	Lasmiditan	-4.8631
51-24-1	Tiratricol	-4.8631
24356-60-3	Cephapirin Sodium	-4.8629
82586-52-5	Moexipril HCl	-4.8628
2447-57-6	Sulfadoxine	-4.8626
106730-54-5	Olprinone	-4.8621
64506-49-6	Sofalcone	-4.8616
1231930-82-7	LY2835219	-4.8614
82419-36-1	Ofloxacin	-4.8613
85721-33-1	Ciprofloxacin	-4.861
93107-08-5	Ciprofloxacin hydrochloride	-4.861
100986-85-4	Levofloxacin	-4.8607
138199-71-0	Levofloxacin Hemihydrate	-4.8607
210353-53-0	Gemifloxacin?mesylate	-4.8601
53797-35-6	Ribostamycin sulfate	-4.8597
27833-64-3	Loxapine succinate salt	-4.8595
83-12-5	Phenylindione	-4.8583
58-15-1	Aminophenazone	-4.8583
3200-06-4	Nafronyl oxalate	-4.8583
147221-93-0	Delavirdine (mesylate)	-4.8577
130-26-7	Clioquinol	-4.8574
33069-62-4	Paclitaxel	-4.8569
25655-41-8	Povidone-iodine	-4.8562
80-77-3	Chlormezanone	-4.8556
73-48-3	Bendroflumethiazide	-4.8552
71675-85-9	Amisulpride	-4.855
2398-81-4	Nicotinic acid N-oxide	-4.8542
864750-70-9	Revefenacin	-4.8541
1009119-65-6	Daclatasvir dihydrochloride	-4.854
83881-52-1	Cetirizine dihydrochloride	-4.8535
144060-53-7	Febuxostat	-4.8531
54-96-6	Amifampridine	-4.853
61336-70-7	Amoxicillin trihydrate	-4.8524
26787-78-0	Amoxicillin	-4.8524
66-84-2	Glucosamine hydrochloride	-4.8522
122320-73-4	Rosiglitazone	-4.8522
141625-93-6	Dronedarone hydrochloride	-4.8521
133-67-5	Trichlormethiazide	-4.852

71-81-8	Isopropamide Iodide	-4.852
110871-86-8	Sparfloxacin	-4.8519
94-26-8	Butylparaben	-4.8517
66-84-2	Glucosamine hydrochloride	-4.8509
51333-22-3	Budesonide	-4.8507
317-34-0	Aminophylline	-4.8503
141626-36-0	Dronedarone	-4.8503
24390-14-5	Doxycycline hyclate	-4.8501
68-26-8	Retinol	-4.85
138-14-7	Deferoxamine Mesylate	-4.8498
142217-69-4	Entecavir	-4.8498
22232-54-8	Carbimazole	-4.8496
130018-77-8	Levocetirizine	-4.8496
119478-55-6	Danofloxacin mesylate	-4.8489
13614-98-7	Minocycline hydrochloride	-4.8488
114-80-7	Neostigmine bromide	-4.8487
382-45-6	Adrenosterone	-4.8483
62-44-2	Phenacetin	-4.8483
3366-95-8	Secnidazole	-4.848
1231930-82-7	LY2835219	-4.848
27833-64-3	Loxapine succinate salt	-4.8479
882-09-7	Clofibric acid	-4.8477
209216-23-9	Entecavir hydrate	-4.8477
57-96-5	Sulfinpyrazone	-4.8476
141-94-6	Hexetidine	-4.8476
64019-93-8	Dipivefrin hydrochloride	-4.8475
51-60-5	Hodostin	-4.8469
6160-12-9	(-)-Sparteine sulfate pentahydrate	-4.8464
53-03-2	Prednisone	-4.8461
167465-36-3	Zosuquidar 3HCl	-4.8459
103628-48-4	Sumatriptan succinate	-4.8458
70374-39-9	Lornoxicam	-4.8458
129101-54-8	Rivastigmine tartrate	-4.8444
747-36-4	Hydroxychloroquine sulfate	-4.8443
163253-35-8	Sitafloxacin Hydrate	-4.8442
58-14-0	Pyrimethamine	-4.8441
1405-10-3	Neomycin sulfate	-4.8441
1672-63-5	4-Hydroxyantipyrine	-4.8439
141-94-6	Hexetidine	-4.8439
912-60-7	Noscapine hydrochloride	-4.8436
52432-72-1	Oxeladin citrate	-4.8434
78755-81-4	Flumazenil	-4.8427

566-48-3	Formestane	-4.8426
97-18-7	Bithionol	-4.8425
172152-50-0	Ilaprazole sodium	-4.8423
53797-35-6	Ribostamycin sulfate	-4.8422
119478-55-6	Danofloxacin mesylate	-4.842
123441-03-2	Rivastigmine	-4.842
706782-28-7	Pimavanserin tartrate	-4.8418
706779-91-1	Pimavanserin	-4.8418
4852-22-6	Proanthocyanidins	-4.8416
69655-05-6	Didanosine	-4.8414
5987-82-6	Oxybuprocaine hydrochloride	-4.8413
52214-84-3	Ciprofibrate	-4.8411
157810-81-6	Indinavir sulfate	-4.8411
749-02-0	Spiperone	-4.8411
16676-29-2	Naltrexone hydrochloride	-4.841
98079-52-8	Lomefloxacin hydrochloride	-4.841
98079-51-7	Lomefloxacin	-4.841
50-02-2	Dexamethasone	-4.8409
882-09-7	Clofibric acid	-4.8407
110871-86-8	Sparfloxacin	-4.8401
108612-45-9	Mizolastine	-4.8387
32854-75-4	Lappaconitine	-4.8386
1137608-69-5	Telotristat Etiprate	-4.8384
168555-66-6	Fosbretabulin Disodium	-4.8382
127-79-7	Sulfamerazine	-4.838
76494-51-4	Ligustrazine hydrochloride	-4.8378
1220-83-3	Sulfamonomethoxine	-4.8378
4146-30-9	Neomycin Sulphate B	-4.8377
60-12-8	Phenethyl alcohol	-4.8372
68-35-9	Sulfadiazine	-4.8365
24729-96-2	Clindamycin phosphate	-4.8359
7083-71-8	(+)-Emetine dihydrochloride hydrate	-4.8358
124832-27-5	Valacyclovir hydrochloride	-4.8357
4342-03-4	Dacarbazine	-4.8353
163680-77-1	Pazufloxacin Mesylate	-4.8348
131707-23-8	Arbidol hydrochloride	-4.8348
469-77-2	Spiculisporic Acid	-4.8347
863031-21-4	Azilsartan Medoxomil	-4.8345
50924-49-7	Mizoribine	-4.8343
56796-39-5	Cefmetazole sodium	-4.8342
1231930-82-7	LY2835219	-4.8341

827022-33-3	Palbociclib Isethionate	-4.8337
827022-32-2	Palbociclib hydrochloride	-4.8337
1177-87-3	Dexamethason acetate	-4.8334
22373-78-0	Monensin sodium salt	-4.8332
571190-30-2	Palbociclib	-4.8331
34140-59-5	Trimebutine maleate	-4.8327
39133-31-8	Trimebutine	-4.8327
120202-66-6	(S)-(+)-Clopidogrel hydrogen sulfate	-4.8326
73816-42-9	Meclocycline sulfosalicylate salt	-4.8321
50-99-7	D(+)-Glucose	-4.8312
4852-22-6	Proanthocyanidins	-4.8304
63675-72-9	Nisoldipine	-4.8294
1220-83-3	Sulfamonomethoxine	-4.8292
856676-23-8	Choline Fenofibrate	-4.8289
27833-64-3	Loxapine succinate salt	-4.8283
58-14-0	Pyrimethamine	-4.8283
98079-52-8	Lomefloxacin hydrochloride	-4.8281
98079-51-7	Lomefloxacin	-4.8281
75507-68-5	Flupirtine maleate	-4.828
51-05-8	Procaine hydrochloride	-4.8278
59-46-1	Procaine	-4.8278
175865-59-5	Valganciclovir hydrochloride	-4.8273
554-92-7	Trimethobenzamide hydrochloride	-4.8273
58-94-6	Chlorothiazide	-4.8272
87233-61-2	Emedastine	-4.8272
56391-57-2	Netilmicin sulfate	-4.8272
4093-35-0	Bromopride	-4.8272
59338-87-3	Alizapride hydrochloride	-4.8268
108929-04-0	Epinastine HCl	-4.8268
144-82-1	Sulfamethizole	-4.8267
566-48-3	Formestane	-4.8267
33342-05-1	Gliquidone	-4.8267
51940-44-4	Pipemidic acid	-4.8262
18010-40-7	Bupivacaine hydrochloride	-4.8256
83-07-8	4-Aminoantipyrine	-4.8255
55079-83-9	Acitretin	-4.8254
98079-52-8	Lomefloxacin hydrochloride	-4.8253
98079-51-7	Lomefloxacin	-4.8253
330784-47-9	Avanafil	-4.8247
965-52-6	Nifuroxazide	-4.8245
84611-23-4	Erdosteine	-4.8243
1247-42-3	Meprednisone	-4.824
204255-11-8	Oseltamivir phosphate	-4.8231

33069-62-4	Paclitaxel	-4.8228
150322-43-3	Prasugrel	-4.8227
856676-23-8	Choline Fenofibrate	-4.8226
1156-19-0	Tolazamide	-4.8222
73816-42-9	Meclocycline sulfosalicylate salt	-4.8218
96847-55-1	Levomilnacipran	-4.8218
95789-30-3	Cefotiam Hexetil Hydrochloride	-4.8212
4852-22-6	Proanthocyanidins	-4.821
81-25-4	Cholic Acid	-4.8207
51-48-9	L-Thyroxine	-4.8197
749-02-0	Spiperone	-4.8197
57-68-1	Sulfamethazine	-4.8196
82956-11-4	Nafamostat mesylate	-4.8192
50-24-8	Prednisolone	-4.8188
31677-93-7	Bupropion hydrochloride	-4.8187
4936-47-4	Nifuratel	-4.8184
89-25-8	Edaravone	-4.8182
108929-04-0	Epinastine HCl	-4.8182
132539-06-1	Olanzapine	-4.818
80-49-9	Homatropine Methylbromide	-4.8172
57-68-1	Sulfamethazine	-4.8171
68767-14-6	Loxoprofen	-4.8164
537-12-2	Diperodon hydrochloride	-4.8164
50-04-4	Cortisone acetate	-4.8161
64485-93-4	Cefotaxime sodium	-4.8161
903576-44-3	Pitolisant hydrochloride	-4.8159
83105-70-8	Sultamicillin Tosylate	-4.8155
220620-09-7	Tigecycline	-4.8154
51333-22-3	Budesonide	-4.8151
6506-37-2	Nimorazole	-4.815
52-86-8	Haloperidol	-4.815
128-13-2	Ursodeoxycholic acid	-4.8146
73-48-3	Bendroflumethiazide	-4.8145
16676-29-2	Naltrexone hydrochloride	-4.8144
595-33-5	Megestrol acetate	-4.8133
2856-75-9	Modaline sulfate	-4.8131
496-67-3	Bromisoval	-4.813
161715-24-8	Tebipenem Pivoxil	-4.813
98079-52-8	Lomefloxacin hydrochloride	-4.8128
98079-51-7	Lomefloxacin	-4.8128
34161-24-5	Fipexide	-4.8128
17896-21-8	Citiolone	-4.8126
164579-32-2	Pantoprazole Sodium Hydrate	-4.8118

61618-27-7	Amfenac Sodium Monohydrate	-4.8115
76494-51-4	Ligustrazine hydrochloride	-4.8112
66309-69-1	Cefotiam hydrochloride	-4.8106
59-30-3	Folic acid	-4.8098
63-74-1	Sulfanilamide	-4.8088
69655-05-6	Didanosine	-4.8086
63968-64-9	Artemisinin	-4.8085
141-94-6	Hexetidine	-4.8084
864750-70-9	Revefenacin	-4.8081
946075-13-4	Isavuconazonium sulfate	-4.8079
673-06-3	D-Phenylalanine	-4.8077
4852-22-6	Proanthocyanidins	-4.8075
59-47-2	Mephenesin	-4.8073
439081-18-2	Afatinib	-4.8073
1722-62-9	Mepivacaine hydrochloride	-4.8072
134308-13-7	Tolcapone	-4.8072
51264-14-3	Amsacrine	-4.8066
52214-84-3	Ciprofibrate	-4.8064
66-76-2	Dicumarol	-4.8063
131707-23-8	Arbidol hydrochloride	-4.8059
138-37-4	Mafenide hydrochloride	-4.8058
136572-09-3	Irinotecan hydrochloride trihydrate	-4.8056
62-44-2	Phenacetin	-4.8055
439081-18-2	Afatinib	-4.8053
23256-30-6	Nifurtimox	-4.8052
4852-22-6	Proanthocyanidins	-4.8051
850140-73-7	Afatinib Dimaleate	-4.8049
65-45-2	Salicylamide	-4.8048
4394-00-7	Niflumic acid	-4.8048
926037-48-1	Radotinib	-4.8048
611-71-2	Mandelic acid	-4.8046
53902-12-8	Tranilast	-4.8045
850140-73-7	Afatinib Dimaleate	-4.8044
485-49-4	(+)-Bicuculline	-4.8041
97682-44-5	Irinotecan	-4.8041
29908-03-0	Ademetionine	-4.804
153773-82-1	Ertapenem sodium	-4.8039
3685-84-5	Meclofenoxate hydrochloride	-4.8037
74536-44-0	Metadoxine	-4.8029
58-56-0	Pyridoxine hydrochloride	-4.8026
65-23-6	Pyridoxine	-4.8026
98079-52-8	Lomefloxacin hydrochloride	-4.8024
98079-51-7	Lomefloxacin	-4.8024

24280-93-1	Mycophenolic acid	-4.802
98-96-4	Pyrazinamide	-4.8019
103980-44-5	Ceftiofur hydrochloride	-4.8014
90098-04-7	Rebamipide	-4.8014
611-75-6	Bromhexine hydrochloride	-4.8013
22839-47-0	Aspartame	-4.8013
220620-09-7	Tigecycline	-4.8013
29868-97-1	Pirenzepine dihydrochloride	-4.8012
17696-69-4	Hematoporphyrin dihydrochloride	-4.801
432-60-0	Allylestrenol	-4.801
68813-55-8	Oxantel Pamoate	-4.8005
864750-70-9	Revefenacin	-4.8003
596-51-0	Glycopyrrolate	-4.8003
1229194-11-9	Edoxaban Tosylate Monohydrate	-4.8
4146-30-9	Neomycin Sulphate B	-4.7999
1146-99-2	chlophenadione	-4.7998
515-64-0	Sulfisomidine	-4.7996
1263-89-4	Paromomycin Sulfate	-4.799
163680-77-1	Pazufloxacin Mesylate	-4.7986
6506-37-2	Nimorazole	-4.7984
473-98-3	Betulin	-4.7982
912273-65-5	Edoxaban	-4.7982
96847-55-1	Levomilnacipran	-4.798
13710-19-5	Tolfenamic Acid	-4.7972
112811-59-3	Gatifloxacin	-4.7971
147221-93-0	Delavirdine (mesylate)	-4.797
747-36-4	Hydroxychloroquine sulfate	-4.7968
136-40-3	Phenazopyridine hydrochloride	-4.7965
66309-69-1	Cefotiam hydrochloride	-4.7964
17696-69-4	Hematoporphyrin dihydrochloride	-4.7963
22373-78-0	Monensin sodium salt	-4.7962
33386-08-2	Buspirone hydrochloride	-4.7962
856867-55-5	Tedizolid Phosphate	-4.7958
302-22-7	Chlormadinone acetate	-4.7957
6385-02-0	Meclofenamate Sodium	-4.7956
4180-23-8	Trans-Anethole	-4.7954
1247-42-3	Meprednisone	-4.7946
500287-72-9	Rilpivirine	-4.7946
5638-76-6	Betahistine	-4.7944
54856-23-4	Betahistine mesylate	-4.7944
5579-84-0	Betahistine dihydrochloride	-4.7944
14882-18-9	Bismuth subsalicylat	-4.7942

53902-12-8	Tranilast	-4.7942
482-36-0	Hyperoside	-4.7942
118288-08-7	Lafutidine	-4.794
112885-42-4	Mosapride Citrate	-4.7921
69-72-7	Salicylic acid	-4.7921
1405-10-3	Neomycin sulfate	-4.7919
24729-96-2	Clindamycin phosphate	-4.7914
25389-94-0	Kanamycin sulfate	-4.7912
68-35-9	Sulfadiazine	-4.7912
459789-99-2	Obeticholic Acid	-4.7911
57-68-1	Sulfamethazine	-4.7908
64-75-5	Tetracycline hydrochloride	-4.79
25389-94-0	Kanamycin sulfate	-4.79
50-24-8	Prednisolone	-4.7899
73816-42-9	Meclocycline sulfosalicylate salt	-4.7897
112887-68-0	Raltitrexed	-4.7895
477600-75-2	Tofacitinib	-4.789
540737-29-9	Tofacitinib Citrate	-4.789
60-54-8	Tetracycline	-4.7884
90-22-2	Valethamate bromide	-4.7884
73816-42-9	Meclocycline sulfosalicylate salt	-4.788
1220-83-3	Sulfamonomethoxine	-4.7867
119-36-8	Methyl salicylate	-4.7865
56391-57-2	Netilmicin sulfate	-4.7865
2259-96-3	Cyclothiazide	-4.7864
4852-22-6	Proanthocyanidins	-4.7862
59338-87-3	Alizapride hydrochloride	-4.7862
248281-84-7	Laquinimod	-4.786
42461-84-7	Flunixin meglumin	-4.786
1137608-69-5	Telotristat Etiprate	-4.786
14698-29-4	Oxolinic acid	-4.7859
97240-79-4	Topiramate	-4.7854
98079-52-8	Lomefloxacin hydrochloride	-4.7851
98079-51-7	Lomefloxacin	-4.7851
153-18-4	Rutin	-4.7847
56-92-8	Histamine 2HCl	-4.7842
51-45-6	Histamine	-4.7842
127-79-7	Sulfamerazine	-4.784
107753-78-6	Zafirlukast	-4.7839
1263-89-4	Paromomycin Sulfate	-4.7834
129497-78-5	Verteporfin	-4.7833
83881-52-1	Cetirizine dihydrochloride	-4.783

78415-72-2	Milrinone	-4.783
89226-50-6	Manidipine	-4.783
548-73-2	Droperidol	-4.783
29908-03-0	Ademetionine	-4.7828
211914-51-1	Dabigatran	-4.7824
58-14-0	Pyrimethamine	-4.7824
59-30-3	Folic acid	-4.7822
54-11-5	Nicotine	-4.782
10605-21-7	Carbendazim	-4.7818
577-11-7	Docusate sodium	-4.7817
89226-75-5	Manidipine dihydrochloride	-4.7812
167465-36-3	Zosuquidar 3HCl	-4.7804
65-31-6	Nicotine Ditartrate	-4.7802
382-45-6	Adrenosterone	-4.7801
1231929-97-7	Abemaciclib	-4.7795
4852-22-6	Proanthocyanidins	-4.7794
79902-63-9	Simvastatin	-4.7791
127-47-9	Retinyl acetate	-4.7788
1786-81-8	Propitocaine hydrochloride	-4.7786
111974-72-2	Quetiapine Fumarate	-4.7786
31282-04-9	Hygromycin B	-4.778
24729-96-2	Clindamycin phosphate	-4.778
721-50-6	Prilocaine	-4.7774
82586-52-5	Moexipril HCl	-4.7773
33342-05-1	Gliquidone	-4.7773
127294-70-6	Balofloxacin	-4.777
59338-87-3	Alizapride hydrochloride	-4.7757
120068-37-3	Fipronil	-4.7756
31677-93-7	Bupropion hydrochloride	-4.7754
126544-47-6	Ciclesonide	-4.7747
52205-73-9	Estramustine phosphate sodium	-4.7745
863127-77-9	Dasatinib monohydrate	-4.7743
168273-06-1	Rimonabant	-4.7742
158681-13-1	Rimonabant hydrochloride	-4.7742
24868-20-0	Dantrolene sodium hemiheptahydrate	-4.7742
443913-73-3	Vandetanib	-4.7734
59338-87-3	Alizapride hydrochloride	-4.7734
1263-89-4	Paromomycin Sulfate	-4.7733
537-55-3	N-Acetyl-L-tyrosine	-4.7731
69558-55-0	Thymopentin	-4.7731
3485-62-9	Clidinium bromide	-4.773
141-94-6	Hexetidine	-4.7728

614-39-1	Procainamide hydrochloride	-4.7727
122320-73-4	Rosiglitazone	-4.7725
4852-22-6	Proanthocyanidins	-4.7722
144-83-2	Sulfapyridine	-4.7718
71125-38-7	Meloxicam	-4.7714
22373-78-0	Monensin sodium salt	-4.7713
26833-87-4	Homoharringtonine	-4.7712
128-13-2	Ursodeoxycholic acid	-4.771
4852-22-6	Proanthocyanidins	-4.7709
130018-77-8	Levocetirizine	-4.7704
126-07-8	Griseofulvin	-4.7702
30751-05-4	Troxipide	-4.7701
912-60-7	Noscapine hydrochloride	-4.7701
81-27-6	Sennoside A	-4.77
123524-52-7	Azelnidipine	-4.7699
504-03-0	Lupetidin	-4.7695
81-25-4	Cholic Acid	-4.7695
364622-82-2	Doripenem Hydrate	-4.769
16773-42-5	Ornidazole	-4.7688
73-24-5	Adenine	-4.7684
65666-07-1	Silymarin	-4.7684
303-25-3	Cyclizine hydrochloride	-4.7682
918504-65-1	Vemurafenib	-4.768
127-79-7	Sulfamerazine	-4.7679
4852-22-6	Proanthocyanidins	-4.7679
864750-70-9	Revefenacin	-4.7675
321-30-2	Adenine sulfate	-4.7672
119478-55-6	Danofloxacin mesylate	-4.7667
985-13-7	Ethaverine hydrochloride	-4.7667
34140-59-5	Trimebutine maleate	-4.7665
39133-31-8	Trimebutine	-4.7665
1490-04-6	DL-Menthol	-4.7661
22373-78-0	Monensin sodium salt	-4.7661
64-72-2	Chlortetracycline hydrochloride	-4.7656
59-26-7	Nikethamide	-4.7645
53-06-5	Cortisone	-4.7645
59338-87-3	Alizapride hydrochloride	-4.7645
6809-52-5	Teprenone	-4.7642
36322-90-4	Piroxicam	-4.764
83915-83-7	Lisinopril dihydrate	-4.764
3093-35-4	Halcinonide	-4.7638
93-14-1	Guaifenesin	-4.7638
58-14-0	Pyrimethamine	-4.7638

440-17-5	Trifluoperazine dihydrochloride	-4.7636
7280-37-7	Estropipate	-4.7633
7554-65-6	4-Methylpyrazole	-4.7632
129-06-6	Warfarin sodium	-4.7631
15622-65-8	Molindone hydrochloride	-4.7613
82956-11-4	Nafamostat mesylate	-4.7613
83881-52-1	Cetirizine dihydrochloride	-4.7611
127294-70-6	Balofloxacin	-4.7606
651-06-9	Sulfameter	-4.7604
150322-43-3	Prasugrel	-4.7604
21187-98-4	Gliclazide	-4.7597
204255-11-8	Oseltamivir phosphate	-4.7597
24729-96-2	Clindamycin phosphate	-4.7593
1405-41-0	Gentamycin sulfate	-4.759
79-57-2	Oxytetracycline	-4.7587
4342-03-4	Dacarbazine	-4.7585
172152-50-0	Ilaprazole sodium	-4.7584
55203-24-2	Dexamethasone Sodium Phosphate	-4.7583
1811-28-5	Proflavine Hemisulfate	-4.7582
171228-49-2	Posaconazole	-4.7581
1405-37-4	Capastat sulfate	-4.7579
1289023-67-1	BMS927711	-4.7579
1229194-11-9	Edoxaban Tosylate Monohydrate	-4.7578
639089-54-6	Tozasertib	-4.7577
97682-44-5	Irinotecan	-4.7576
1137608-69-5	Telotristat Etiprate	-4.7569
738-70-5	Trimethoprim	-4.7567
136572-09-3	Irinotecan hydrochloride trihydrate	-4.7567
94055-76-2	Suplatast tosilate	-4.7565
33386-08-2	Buspirone hydrochloride	-4.7558
3543-75-7	Bendamustine hydrochloride	-4.7555
606143-89-9	Binimetinib	-4.7551
51460-26-5	Carbazochrome sodium sulfonate	-4.7549
645-43-2	Guanethidine Monosulfate	-4.7549
83-12-5	Phenylindione	-4.7544
64-75-5	Tetracycline hydrochloride	-4.7544
1137608-69-5	Telotristat Etiprate	-4.7542
651-06-9	Sulfameter	-4.7541
3093-35-4	Halcinonide	-4.7538
129618-40-2	Nevirapine	-4.7533

69-72-7	Salicylic acid	-4.7531
122892-31-3	Itopride hydrochloride	-4.7529
14882-18-9	Bismuth subsalicylat	-4.7528
66309-69-1	Cefotiam hydrochloride	-4.7527
4936-47-4	Nifuratel	-4.7527
5638-76-6	Betahistine	-4.7525
54856-23-4	Betahistine mesylate	-4.7522
5579-84-0	Betahistine dihydrochloride	-4.7522
97-53-0	Eugenol	-4.7517
38194-50-2	Sulindac	-4.7513
31282-04-9	Hygromycin B	-4.7499
51940-44-4	Pipemidic acid	-4.7499
6160-12-9	(-)-Sparteine sulfate pentahydrate	-4.7498
54-11-5	Nicotine	-4.7489
14919-77-8	Benserazide hydrochloride	-4.7488
53164-05-9	Acemetacin	-4.7484
76824-35-6	Famotidine	-4.7481
89226-75-5	Manidipine dihydrochloride	-4.7471
21829-25-4	Nifedipine	-4.7468
65-31-6	Nicotine Ditartrate	-4.7468
152-47-6	Sulfalen	-4.7464
155148-31-5	Plerixafor 8HCl (AMD3100 8HCl)	-4.7464
110078-46-1	Plerixafor	-4.7464
54029-12-8	Albendazole Oxide	-4.7463
89226-50-6	Manidipine	-4.7462
83-73-8	Iodoquinol	-4.7457
141-94-6	Hexetidine	-4.7454
17560-51-9	Metolazone	-4.7453
84371-65-3	Mifepristone	-4.7449
73816-42-9	Meclocycline sulfosalicylate salt	-4.7449
37517-28-5	Amikacin	-4.7448
57-66-9	Probenecid	-4.7446
170105-16-5	Imidafenacin	-4.7446
53179-09-2	Sisomicin Sulfate	-4.7442
577-11-7	Docusate sodium	-4.7441
4936-47-4	Nifuratel	-4.7441
64506-49-6	Sofalcone	-4.7434
13171-25-0	Trimetazidine dihydrochloride	-4.7433
3380-34-5	Triclosan	-4.7428
6469-93-8	Chlorprothixene hydrochloride	-4.7426
113-59-7	Chlorprothixene	-4.7426
24729-96-2	Clindamycin phosphate	-4.7424
1722-62-9	Mepivacaine hydrochloride	-4.7422

103577-45-3	Lansoprazole	-4.7415
141-94-6	Hexetidine	-4.7412
5987-82-6	Oxybuprocaine hydrochloride	-4.7403
126-27-2	Oxethazaine	-4.7401
60-12-8	Phenethyl alcohol	-4.7398
23256-30-6	Nifurtimox	-4.7397
1156-19-0	Tolazamide	-4.7396
12650-69-0	Mupirocin	-4.7395
2192-20-3	Hydroxyzine dihydrochloride	-4.739
485-49-4	(+)-Bicuculline	-4.7389
68767-14-6	Loxoprofen	-4.7388
473-98-3	Betulin	-4.7386
21736-83-4	Spectinomycin dihydrochloride	-4.7386
66309-69-1	Cefotiam hydrochloride	-4.7386
69659-80-9	Tanshinone IIA sulfonate sodium	-4.7385
10246-75-0	Hydroxyzine Pamoate	-4.7384
4342-03-4	Dacarbazine	-4.7382
23256-30-6	Nifurtimox	-4.7377
84371-65-3	Mifepristone	-4.7376
99464-64-9	Ampiroxicam	-4.7371
4800-94-6	Carbenicillin disodium	-4.7357
141-94-6	Hexetidine	-4.7355
31430-15-6	Flubendazole	-4.734
4852-22-6	Proanthocyanidins	-4.734
839712-12-8	Cariprazine	-4.7339
141-94-6	Hexetidine	-4.7337
475-67-2	S-Isocorydine(+)	-4.7335
444731-52-6	Pazopanib	-4.7331
58947-95-8	DL-trihexyphenidyl hydrochloride	-4.733
117977-21-6	Rabeprazole Related Compound E	-4.7329
1490-04-6	DL-Menthol	-4.7326
144-83-2	Sulfapyridine	-4.7324
459868-92-9	Rucaparib Phosphate	-4.7321
283173-50-2	Rucaparib	-4.7321
58-95-7	Vitamin E Acetate	-4.7315
15622-65-8	Molindone hydrochloride	-4.7314
59338-87-3	Alizapride hydrochloride	-4.7311
635702-64-6	Pazopanib HCl	-4.731
114-80-7	Neostigmine bromide	-4.7307
97-59-6	Allantoin	-4.7306
59338-87-3	Alizapride hydrochloride	-4.73
74103-07-4	Ketorolac tromethamine salt	-4.7299
68-26-8	Retinol	-4.7296

51-60-5	Hodostin	-4.7295
2451-01-6	Terpin Monohydrate	-4.729
1098-97-1	Pyrithioxin	-4.729
141625-93-6	Dronedarone hydrochloride	-4.7289
149022-22-0	Amikacin Sulfate Salt	-4.7288
59338-87-3	Alizapride hydrochloride	-4.7287
738-70-5	Trimethoprim	-4.7286
2135-17-3	Flumethasone	-4.7281
141626-36-0	Dronedarone	-4.728
4697-14-7	Ticarcillin disodium	-4.7276
56391-57-2	Netilmicin sulfate	-4.7272
145040-37-5	Candesartan Cilexetil	-4.7268
138844-81-2	Ibandronate sodium	-4.7264
4852-22-6	Proanthocyanidins	-4.726
53797-35-6	Ribostamycin sulfate	-4.726
74103-07-4	Ketorolac tromethamine salt	-4.7253
63968-64-9	Artemisinin	-4.7251
31677-93-7	Bupropion hydrochloride	-4.7251
618385-01-6	Vorapaxar	-4.7244
2135-17-3	Flumethasone	-4.724
936091-26-8	Fedratinib	-4.7235
1229194-11-9	Edoxaban Tosylate Monohydrate	-4.7232
2353-33-5	Decitabine	-4.7225
248281-84-7	Laquinimod	-4.7223
62-44-2	Phenacetin	-4.7223
17696-69-4	Hematoporphyrin dihydrochloride	-4.7222
102518-79-6	(-)-Huperzine A	-4.7208
58-71-9	Cephalothin sodium	-4.7206
4852-22-6	Proanthocyanidins	-4.7206
10402-53-6	Eprazinone dihydrochloride	-4.7205
1197-18-8	Amstat	-4.7202
2447-57-6	Sulfadoxine	-4.7199
79350-37-1	Cefixime	-4.7191
81840-15-5	Vesnarinone	-4.7191
51460-26-5	Carbazochrome sodium sulfonate	-4.7189
363-24-6	Prostaglandin E2 (PGE2)	-4.7183
97240-79-4	Topiramate	-4.7179
432-60-0	Allylestrenol	-4.7177
76-25-5	Triamcinolone acetonide	-4.7175
1135-24-6	Ferulic Acid	-4.7173
136790-76-6	Lubiprostone	-4.7173
552-94-3	Sasapyrine	-4.717

17896-21-8	Citiolone	-4.7165
4852-22-6	Proanthocyanidins	-4.7157
66-84-2	Glucosamine hydrochloride	-4.7154
960203-27-4	Vortioxetine HBr	-4.715
4852-22-6	Proanthocyanidins	-4.7143
56391-57-2	Netilmicin sulfate	-4.7138
508233-74-7	Vortioxetine	-4.7138
357-08-4	Naloxone hydrochloride	-4.7135
4852-22-6	Proanthocyanidins	-4.7134
36703-88-5	Isoprinosine	-4.7134
58-63-9	Inosine	-4.7134
24390-14-5	Doxycycline hyclate	-4.7128
73590-58-6	Omeprazole	-4.7126
24390-14-5	Doxycycline hyclate	-4.7125
99755-59-6	Rotigotine	-4.7121
382-45-6	Adrenosterone	-4.712
27262-48-2	Levobupivacaine HCl	-4.712
27262-47-1	Levobupivacaine	-4.712
18010-40-7	Bupivacaine hydrochloride	-4.712
132539-06-1	Olanzapine	-4.7111
2856-75-9	Modaline sulfate	-4.7108
22457-89-2	Benfotiamine	-4.7107
723-46-6	Sulfamethoxazole	-4.7105
111406-87-2	Zileuton	-4.7102
1221573-85-8	Paritaprevir	-4.71
138452-21-8	Fexofenadine hydrochloride	-4.7099
330808-88-3	Vardenafil hydrochloride trihydrate	-4.7097
577778-58-6	Topiroxostat	-4.7096
107133-36-8	Perindopril erbumine	-4.7096
119-36-8	Methyl salicylate	-4.7096
34562-97-5	Pikamilone	-4.7095
164579-32-2	Pantoprazole Sodium Hydrate	-4.7095
70356-03-5	Cefaclor monohydrate	-4.7075
34161-24-5	Fipexide	-4.7072
13171-25-0	Trimetazidine dihydrochloride	-4.707
4936-47-4	Nifuratel	-4.7069
26328-04-1	Cinepazide maleate	-4.7068
1231930-82-7	LY2835219	-4.706
120068-37-3	Fipronil	-4.706
66357-59-3	Ranitidine Hydrochloride	-4.7057
4852-22-6	Proanthocyanidins	-4.7053

968-81-0	Acetohexamide	-4.7053
122-11-2	Sulfadimethoxine	-4.7052
66852-54-8	Halobetasol Propionate	-4.7049
638-94-8	Desonide	-4.7049
202189-78-4	Bilastine	-4.7047
23288-49-5	Probucol	-4.7046
1177-87-3	Dexamethason acetate	-4.704
23964-57-0	Articaine HCl	-4.704
138844-81-2	Ibandronate sodium	-4.7036
1135-23-5	Hydroferulic acid	-4.7033
378-44-9	Betamethasone	-4.7033
31282-04-9	Hygromycin B	-4.703
58-54-8	Ethacrynic acid	-4.7028
138530-94-6	R-(+)-Lansoprazole	-4.7025
103577-45-3	Lansoprazole	-4.7025
827022-33-3	Palbociclib Isethionate	-4.7025
827022-32-2	Palbociclib hydrochloride	-4.7025
4852-22-6	Proanthocyanidins	-4.7023
4852-22-6	Proanthocyanidins	-4.7022
946075-13-4	Isavuconazonium sulfate	-4.702
571190-30-2	Palbociclib	-4.7016
6004-24-6	Cetylpyridinium chloride monohydrate	-4.7016
24390-14-5	Doxycycline hyclate	-4.7013
112811-59-3	Gatifloxacin	-4.7006
697761-98-1	Elvitegravir	-4.7004
58895-64-0	Nalmefene hydrochloride	-4.7004
864750-70-9	Revefenacin	-4.7004
154229-19-3	Abiraterone	-4.7003
871038-72-1	Raltegravir potassium	-4.6995
72432-03-2	Miglitol	-4.6993
118288-08-7	Lafutidine	-4.6992
152459-95-5	Imatinib	-4.6988
220127-57-1	Imatinib Mesylate	-4.6988
68-35-9	Sulfadiazine	-4.6981
68767-14-6	Loxoprofen	-4.6979
52432-72-1	Oxeladin citrate	-4.6978
163680-77-1	Pazufloxacin Mesylate	-4.6976
482-36-0	Hyperoside	-4.6973
122-11-2	Sulfadimethoxine	-4.6971
54-36-4	Metyrapone	-4.6971
1490-04-6	DL-Menthol	-4.697
912273-65-5	Edoxaban	-4.6969
74011-58-8	Enoxacin	-4.6967

51-48-9	L-Thyroxine	-4.6966
141-94-6	Hexetidine	-4.6964
1229194-11-9	Edoxaban Tosylate Monohydrate	-4.6957
84294-96-2	Enoxacin Sesquihydrate	-4.6955
31282-04-9	Hygromycin B	-4.6955
3801-06-7	Fluorometholone Acetate	-4.6955
3685-84-5	Meclofenoxate hydrochloride	-4.6951
72432-03-2	Miglitol	-4.6949
60-54-8	Tetracycline	-4.6947
81-25-4	Cholic Acid	-4.6944
40828-46-4	Suprofen	-4.6939
2557-49-5	Diflorasone	-4.6935
54-71-7	(+)-Pilocarpine hydrochloride	-4.6934
24316-19-6	Cephalotaxine	-4.6933
853-23-6	Dehydroepiandrosterone acetate	-4.6932
504-03-0	Lupetidin	-4.6929
148-72-1	Pilocarpine nitrate	-4.6928
518048-05-0	Raltegravir	-4.6928
645-05-6	Altretamine	-4.6922
2447-57-6	Sulfadoxine	-4.6915
93479-97-1	Glimepiride	-4.6914
23541-50-6	Daunorubicin hydrochloride	-4.6911
182349-12-8	Rupatadine Fumarate	-4.6905
5875-06-9	Proparacaine hydrochloride	-4.6901
112811-59-3	Gatifloxacin	-4.69
804-30-8	Fursultiamine	-4.69
161735-79-1	Rasagiline Mesylate	-4.6895
71320-77-9	Moclobemide	-4.6892
123948-87-8	Topotecan	-4.6889
157810-81-6	Indinavir sulfate	-4.688
59338-87-3	Alizapride hydrochloride	-4.6876
136236-51-6	Rasagiline	-4.6871
14484-47-0	Deflazacort	-4.6869
14484-47-0	Deflazacort	-4.6869
22457-89-2	Benfotiamine	-4.6869
1220-83-3	Sulfamonomethoxine	-4.6867
122647-32-9	Ibutilide Fumarate	-4.686
4852-22-6	Proanthocyanidins	-4.6856
4852-22-6	Proanthocyanidins	-4.6849
31282-04-9	Hygromycin B	-4.6847
723-46-6	Sulfamethoxazole	-4.6844
747-36-4	Hydroxychloroquine sulfate	-4.684

120202-66-6	(S)-(+)-Clopidogrel hydrogen sulfate	-4.684
956697-53-3	NVP-LDE225	-4.6839
4852-22-6	Proanthocyanidins	-4.6836
302-22-7	Chlormadinone acetate	-4.6835
147403-03-0	Azilsartan	-4.6831
31677-93-7	Bupropion hydrochloride	-4.6831
167465-36-3	Zosuquidar 3HCl	-4.683
68767-14-6	Loxoprofen	-4.6829
577-11-7	Docusate sodium	-4.6827
747-36-4	Hydroxychloroquine sulfate	-4.6816
645-43-2	Guanethidine Monosulfate	-4.6815
596-51-0	Glycopyrrolate	-4.6811
68767-14-6	Loxoprofen	-4.6806
59277-89-3	Aciclovir	-4.6805
58-56-0	Pyridoxine hydrochloride	-4.6804
65-23-6	Pyridoxine	-4.6804
4852-22-6	Proanthocyanidins	-4.6802
74536-44-0	Metadoxine	-4.6798
59-26-7	Nikethamide	-4.6796
4180-23-8	Trans-Anethole	-4.6781
93479-97-1	Glimepiride	-4.6781
2856-75-9	Modaline sulfate	-4.678
738606-46-7	ETC1002	-4.6779
5593-20-4	Betamethasone dipropionate	-4.6779
22199-08-2	Silver sulfadiazine	-4.6777
164579-32-2	Pantoprazole Sodium Hydrate	-4.677
302-79-4	Tretinoin	-4.6769
93479-97-1	Glimepiride	-4.6763
871700-17-3	Trametinib	-4.6762
850140-73-7	Afatinib Dimaleate	-4.6754
34031-32-8	Auranofin	-4.6751
173352-39-1	Afobazole hydrochloride	-4.6751
439081-18-2	Afatinib	-4.6748
53882-12-5	Lodoxamide	-4.6743
3093-35-4	Halcinonide	-4.6741
73963-72-1	Cilostazol	-4.6736
84057-84-1	Lamotrigine	-4.6732
13311-84-7	Flutamide	-4.6731
61336-70-7	Amoxicillin trihydrate	-4.6727
26787-78-0	Amoxicillin	-4.6727
773-76-2	Capitrol	-4.6723
56-92-8	Histamine 2HCl	-4.672
51-45-6	Histamine	-4.672

29868-97-1	Pirenzepine dihydrochloride	-4.672
52205-73-9	Estramustine phosphate sodium	-4.6719
76-25-5	Triamcinolone acetonide	-4.6714
51460-26-5	Carbazochrome sodium sulfonate	-4.6714
22373-78-0	Monensin sodium salt	-4.6713
61336-70-7	Amoxicillin trihydrate	-4.6712
26787-78-0	Amoxicillin	-4.6712
122320-73-4	Rosiglitazone	-4.6711
298-57-7	Stugeron	-4.6709
32449-92-6	Dicurone	-4.6702
1847-24-1	Flucloxacillin sodium	-4.6702
186826-86-8	Moxifloxacin hydrochloride	-4.67
747-36-4	Hydroxychloroquine sulfate	-4.668
3543-75-7	Bendamustine hydrochloride	-4.6678
73816-42-9	Meclocycline sulfosalicylate salt	-4.6676
30751-05-4	Troxipide	-4.6675
52214-84-3	Ciprofibrate	-4.6673
54-71-7	(+)-Pilocarpine hydrochloride	-4.6673
122-11-2	Sulfadimethoxine	-4.6672
119478-55-6	Danofloxacin mesylate	-4.667
148-72-1	Pilocarpine nitrate	-4.667
2181-04-6	Potassium canrenoate	-4.6667
29908-03-0	Ademetionine	-4.6665
4852-22-6	Proanthocyanidins	-4.6664
59-30-3	Folic acid	-4.6664
1229194-11-9	Edoxaban Tosylate Monohydrate	-4.6659
67392-87-4	Drospirenone	-4.6656
83-88-5	Riboflavin	-4.6653
22232-54-8	Carbimazole	-4.665
52-01-7	Abbolactone	-4.6647
132810-10-7	Blonanserin	-4.6646
89-25-8	Edaravone	-4.6641
912273-65-5	Edoxaban	-4.6641
133-67-5	Trichlormethiazide	-4.6636
4852-22-6	Proanthocyanidins	-4.6636
149003-01-0	Dexrazoxane HCl	-4.6633
141-94-6	Hexetidine	-4.6633
202189-78-4	Bilastine	-4.6632
83-07-8	4-Aminoantipyrine	-4.6631
150322-43-3	Prasugrel	-4.6631
37517-28-5	Amikacin	-4.663
29908-03-0	Ademetionine	-4.6627

890-38-0	2'-Deoxyinosine	-4.6626
120202-66-6	(S)-(+)-Clopidogrel hydrogen sulfate	-4.6625
178307-42-1	Revaprazan hydrochloride	-4.6624
86780-90-7	Aranidipine	-4.6621
140462-76-6	Olopatadine hydrochloride	-4.662
68-35-9	Sulfadiazine	-4.6614
738606-46-7	ETC1002	-4.6608
120638-55-3	Bromfenac sodium	-4.6606
303-25-3	Cyclizine hydrochloride	-4.6605
57-96-5	Sulfinpyrazone	-4.6593
3697-42-5	Chlorhexidine hydrochloride	-4.6593
56-95-1	Chlorhexidine Acetate	-4.6593
84611-23-4	Erdosteine	-4.659
127-79-7	Sulfamerazine	-4.6588
937272-79-2	Pacritinib	-4.6585
119478-55-6	Danofloxacin mesylate	-4.6575
504-03-0	Lupetidin	-4.6575
106685-40-9	Adapalene	-4.6573
163451-81-8	Teriflunomide	-4.6572
108605-62-5	Teriflunomide	-4.6572
3056-17-5	Stavudine	-4.6569
4852-22-6	Proanthocyanidins	-4.6569
52214-84-3	Ciprofibrate	-4.6568
152459-95-5	Imatinib	-4.656
220127-57-1	Imatinib Mesylate	-4.656
55661-38-6	Nimustine Hydrochloride	-4.6558
99464-64-9	Ampiroxicam	-4.6555
54-11-5	Nicotine	-4.6554
747-36-4	Hydroxychloroquine sulfate	-4.6544
56-69-9	5-hydroxytryptophan	-4.6542
65-31-6	Nicotine Ditartrate	-4.6542
93-14-1	Guaifenesin	-4.6542
474-25-9	Chenodeoxycholic acid	-4.6539
132539-06-1	Olanzapine	-4.6534
66-84-2	Glucosamine hydrochloride	-4.6533
130018-77-8	Levocetirizine	-4.6533
83881-52-1	Cetirizine dihydrochloride	-4.6527
378-44-9	Betamethasone	-4.6527
147403-03-0	Azilsartan	-4.6525
13636-18-5	Fendiline hydrochloride	-4.6523
79559-97-0	Sertraline hydrochloride	-4.6522
73816-42-9	Meclocycline sulfosalicylate salt	-4.6513
864750-70-9	Revefenacin	-4.6513

33286-22-5	Diltiazem hydrochloride	-4.6512
31430-15-6	Flubendazole	-4.6511
33342-05-1	Gliquidone	-4.6508
1009119-64-5	Daclatasvir	-4.6506
140462-76-6	Olopatadine hydrochloride	-4.6503
3801-06-7	Fluorometholone Acetate	-4.6501
79-57-2	Oxytetracycline	-4.6501
515-64-0	Sulfisomidine	-4.65
220620-09-7	Tigecycline	-4.65
75330-75-5	Lovastatin	-4.6498
1289023-67-1	BMS927711	-4.6498
50-24-8	Prednisolone	-4.6495
3801-06-7	Fluorometholone Acetate	-4.6494
25655-41-8	Povidone-iodine	-4.6489
71-58-9	Medroxyprogesterone Acetate	-4.6482
501-68-8	Beclamide	-4.6481
22373-78-0	Monensin sodium salt	-4.648
10402-53-6	Eprazinone dihydrochloride	-4.6472
76824-35-6	Famotidine	-4.6471
76824-35-6	Famotidine	-4.647
82586-52-5	Moexipril HCl	-4.647
17896-21-8	Citiolone	-4.6467
76-90-4	Mepenzolate Bromide	-4.6467
124832-27-5	Valacyclovir hydrochloride	-4.6465
122841-12-7	Cefoselis Sulfate	-4.6464
119478-55-6	Danofloxacin mesylate	-4.6463
29908-03-0	Ademetionine	-4.6463
515-64-0	Sulfisomidine	-4.646
73816-42-9	Meclocycline sulfosalicylate salt	-4.646
211914-51-1	Dabigatran	-4.6454
618385-01-6	Vorapaxar	-4.6453
82419-36-1	Ofloxacin	-4.6451
120638-55-3	Bromfenac sodium	-4.6449
57-68-1	Sulfamethazine	-4.6449
102518-79-6	(-)-Huperzine A	-4.6448
82586-52-5	Moexipril HCl	-4.6442
5633-20-5	Oxybutynin	-4.644
1508-65-2	Oxybutynin hydrochloride	-4.644
210353-53-0	Gemifloxacin?mesylate	-4.6436
32986-56-4	Tobramycin	-4.6426
5987-82-6	Oxybuprocaine hydrochloride	-4.6426

86780-90-7	Aranidipine	-4.6417
4008-48-4	Nitroxoline	-4.6415
1173-88-2	Oxacillin sodium	-4.6413
4852-22-6	Proanthocyanidins	-4.641
72432-03-2	Miglitol	-4.641
141-94-6	Hexetidine	-4.6408
61422-45-5	Carmofur	-4.6407
51333-22-3	Budesonide	-4.6401
129722-12-9	Aripiprazole	-4.6401
64-73-3	Demeclocycline hydrochloride	-4.6397
67392-87-4	Drospirenone	-4.6393
141-94-6	Hexetidine	-4.6388
21829-25-4	Nifedipine	-4.6387
1220-83-3	Sulfamonomethoxine	-4.6384
127294-70-6	Balofloxacin	-4.6378
31282-04-9	Hygromycin B	-4.6377
248281-84-7	Laquinimod	-4.6375
3546-41-6	Pyrvinium pamoate	-4.6372
74536-44-0	Metadoxine	-4.6372
603-50-9	Bisacodyl	-4.6372
144060-53-7	Febuxostat	-4.6371
303-25-3	Cyclizine hydrochloride	-4.637
129618-40-2	Nevirapine	-4.6369
84057-84-1	Lamotrigine	-4.6363
22373-78-0	Monensin sodium salt	-4.6356
521-74-4	Broxyquinoline	-4.6355
64953-12-4	Latamoxef sodium	-4.6351
58-95-7	Vitamin E Acetate	-4.6349
444731-52-6	Pazopanib	-4.6348
681492-22-8	Delamanid	-4.6342
737789-87-6	Relugolix	-4.6342
4360-12-7	Ajmaline	-4.6339
554-57-4	Methazolamide	-4.6337
635702-64-6	Pazopanib HCl	-4.6336
136236-51-6	Rasagiline	-4.633
68-35-9	Sulfadiazine	-4.6328
64-73-3	Demeclocycline hydrochloride	-4.6327
248281-84-7	Laquinimod	-4.6327
705260-08-8	Vorapaxar sulfate	-4.6324
58-56-0	Pyridoxine hydrochloride	-4.6318
65-23-6	Pyridoxine	-4.6318
161735-79-1	Rasagiline Mesylate	-4.6315
357-08-4	Naloxone hydrochloride	-4.6314

890-38-0	2'-Deoxyinosine	-4.631
474-25-9	Chenodeoxycholic acid	-4.6309
153504-70-2	Cevimeline hydrochloride hemihydrate	-4.6308
26171-23-3	Tolmetin	-4.6307
863127-77-9	Dasatinib monohydrate	-4.6307
145202-66-0	Rizatriptan benzoate	-4.6306
61270-78-8	Cefonicid sodium	-4.6305
877399-52-5	Crizotinib	-4.6302
330808-88-3	Vardenafil hydrochloride trihydrate	-4.63
93479-97-1	Glimepiride	-4.6299
58-14-0	Pyrimethamine	-4.6296
65666-07-1	Silymarin	-4.6295
871700-17-3	Trametinib	-4.6294
97-18-7	Bithionol	-4.6293
149022-22-0	Amikacin Sulfate Salt	-4.6292
37517-28-5	Amikacin	-4.6291
131707-23-8	Arbidol hydrochloride	-4.6288
51264-14-3	Amsacrine	-4.6286
82419-36-1	Ofloxacin	-4.6279
211914-51-1	Dabigatran	-4.6277
56-94-0	Demecarium bromide	-4.6276
65666-07-1	Silymarin	-4.6271
120068-37-3	Fipronil	-4.6266
459868-92-9	Rucaparib Phosphate	-4.6265
283173-50-2	Rucaparib	-4.6265
248281-84-7	Laquinimod	-4.6263
23288-49-5	Probucol	-4.6261
100986-85-4	Levofloxacin	-4.6261
138199-71-0	Levofloxacin Hemihydrate	-4.6261
51940-44-4	Pipemidic acid	-4.626
872728-81-9	Dabigatran Etexilate Mesylate	-4.6259
695-53-4	5,5-Dimethyloxazolidine-2,4-dione	-4.6256
482-36-0	Hyperoside	-4.6252
53902-12-8	Tranilast	-4.6248
25655-41-8	Povidone-iodine	-4.6244
75438-57-2	Moxonidine	-4.6235
23210-58-4	Ifenprodil Tartrate	-4.6231
79-57-2	Oxytetracycline	-4.622
51264-14-3	Amsacrine	-4.6212
55661-38-6	Nimustine Hydrochloride	-4.6196
182349-12-8	Rupatadine Fumarate	-4.6194
53797-35-6	Ribostamycin sulfate	-4.6193
64-75-5	Tetracycline hydrochloride	-4.6184

53797-35-6	Ribostamycin sulfate	-4.6183
1197-18-8	Amstat	-4.6181
123447-62-1	Prulifloxacin	-4.618
120202-66-6	(S)-(+)-Clopidogrel hydrogen sulfate	-4.6176
141-94-6	Hexetidine	-4.6166
220119-17-5	Selamectin	-4.6162
537-12-2	Diperodon hydrochloride	-4.6159
298-57-7	Stugeron	-4.6158
751-94-0	Sodium fusidate	-4.6156
6990-06-3	Fusidine	-4.6156
141-94-6	Hexetidine	-4.6155
51-48-9	L-Thyroxine	-4.6155
945667-22-1	Saxagliptin hydrate	-4.6152
4852-22-6	Proanthocyanidins	-4.6148
71320-77-9	Moclobemide	-4.6141
4852-22-6	Proanthocyanidins	-4.614
63675-72-9	Nisoldipine	-4.6136
152459-95-5	Imatinib	-4.6136
220127-57-1	Imatinib Mesylate	-4.6136
4852-22-6	Proanthocyanidins	-4.6131
210353-53-0	Gemifloxacin?mesylate	-4.6129
54527-84-3	Nicardipine hydrochloride	-4.6129
35554-44-0	Imazalil	-4.6128
4852-22-6	Proanthocyanidins	-4.6122
554-57-4	Methazolamide	-4.6119
84057-95-4	Ropivacaine	-4.6113
98717-15-8	Ropivacaine hydrochloride	-4.6113
103980-44-5	Ceftiofur hydrochloride	-4.6096
35554-44-0	Imazalil	-4.6095
112665-43-7	Seratrodast	-4.6077
773-76-2	Capitrol	-4.6075
94-09-7	Benzocaine	-4.6075
10246-75-0	Hydroxyzine Pamoate	-4.6071
117977-21-6	Rabeprazole Related Compound E	-4.607
71320-77-9	Moclobemide	-4.6069
2192-20-3	Hydroxyzine dihydrochloride	-4.6065
112811-59-3	Gatifloxacin	-4.606
4852-22-6	Proanthocyanidins	-4.6053
182349-12-8	Rupatadine Fumarate	-4.6053
59-26-7	Nikethamide	-4.6052
3056-17-5	Stavudine	-4.6052
577-11-7	Docusate sodium	-4.6052
17737-65-4	Clonixin	-4.6047

95789-30-3	Cefotiam Hexetil Hydrochloride	-4.6043
16676-29-2	Naltrexone hydrochloride	-4.6042
100986-85-4	Levofloxacin	-4.6038
138199-71-0	Levofloxacin Hemihydrate	-4.6038
82419-36-1	Ofloxacin	-4.6032
58-15-1	Aminophenazone	-4.6031
76824-35-6	Famotidine	-4.603
29908-03-0	Ademetionine	-4.6028
22254-24-6	Ipratropium Bromide	-4.6026
66985-17-9	Ipratropium bromide monohydrate	-4.6026
34562-97-5	Pikamilone	-4.6021
128794-94-5	Mycophenolate Mofetil	-4.6019
872728-81-9	Dabigatran Etexilate Mesylate	-4.6017
36703-88-5	Isoprinosine	-4.6017
58-63-9	Inosine	-4.6017
18699-02-0	Actarit	-4.6016
55203-24-2	Dexamethasone Sodium Phosphate	-4.6015
1025504-45-3	Valbenazine	-4.6013
317-34-0	Aminophylline	-4.601
504-03-0	Lupetidin	-4.6003
389139-89-3	Omadacycline	-4.6002
172152-50-0	Ilaprazole sodium	-4.5996
705260-08-8	Vorapaxar sulfate	-4.5994
145-41-5	Sodium dehydrocholate	-4.5991
1722-62-9	Mepivacaine hydrochloride	-4.599
113852-37-2	Cidofovir	-4.5987
364-62-5	5-Chloro-2-methoxyprocainamide	-4.5985
7232-21-5	Metoclopramide hydrochloride	-4.5985
56391-57-2	Netilmicin sulfate	-4.5972
1110813-31-4	Dacomitinib	-4.5971
7512-17-6	N-Acetyl-D-Glucosamine	-4.597
107133-36-8	Perindopril erbumine	-4.597
1009119-64-5	Daclatasvir	-4.5968
26328-04-1	Cinepazide maleate	-4.5964
290297-26-6	Netupitant	-4.5963
330784-47-9	Avanafil	-4.5963
64485-93-4	Cefotaxime sodium	-4.5954
302-79-4	Tretinoin	-4.595
606-04-2	Pamabrom	-4.5949
4852-22-6	Proanthocyanidins	-4.5944

22881-35-2	Famprofazone	-4.5942
76824-35-6	Famotidine	-4.594
89226-50-6	Manidipine	-4.594
38194-50-2	Sulindac	-4.5937
141-94-6	Hexetidine	-4.5936
67814-76-0	Isepamicin sulfate	-4.5933
144-82-1	Sulfamethizole	-4.5933
59-63-2	Isocarboxazid	-4.5929
4852-22-6	Proanthocyanidins	-4.5925
112665-43-7	Seratrodast	-4.5919
4852-22-6	Proanthocyanidins	-4.5917
149709-62-6	AHU377	-4.591
1369773-39-6	AHU377 calcium salt	-4.591
51460-26-5	Carbazochrome sodium sulfonate	-4.5909
89226-75-5	Manidipine dihydrochloride	-4.5907
81-27-6	Sennoside A	-4.5906
356-12-7	Fluocinonide	-4.59
18010-40-7	Bupivacaine hydrochloride	-4.5898
1263-89-4	Paromomycin Sulfate	-4.5893
4008-48-4	Nitroxoline	-4.5892
1231929-97-7	Abemaciclib	-4.5891
97-53-0	Eugenol	-4.5889
31430-15-6	Flubendazole	-4.5888
32986-56-4	Tobramycin	-4.5882
132539-06-1	Olanzapine	-4.5873
82571-53-7	Ozagrel	-4.5868
577-11-7	Docusate sodium	-4.5868
1230487-00-9	Siponimod	-4.5868
42461-84-7	Flunixin meglumin	-4.5867
31282-04-9	Hygromycin B	-4.5863
68099-86-5	Bepridil hydrochloride	-4.5863
1025687-58-4	R788 Disodium	-4.5862
78712-43-3	Ozagrel hydrochloride	-4.5856
961-29-5	Isoliquiritigenin	-4.5855
1197-18-8	Amstat	-4.5849
901119-35-5	Fostamatinib	-4.5848
83-07-8	4-Aminoantipyrine	-4.5845
1135-24-6	Ferulic Acid	-4.5831
113852-37-2	Cidofovir	-4.5831
2451-01-6	Terpin Monohydrate	-4.5827

4852-22-6	Proanthocyanidins	-4.5824
69304-47-8	Brivudine	-4.5824
23327-57-3	Nefopam hydrochloride	-4.5818
4093-35-0	Bromopride	-4.5817
951395-08-7	tafamidis meglumine	-4.5816
31282-04-9	Hygromycin B	-4.5816
595-33-5	Megestrol acetate	-4.5807
56796-39-5	Cefmetazole sodium	-4.5807
172152-50-0	Ilaprazole sodium	-4.5797
65666-07-1	Silymarin	-4.5796
120202-66-6	(S)-(+)-Clopidogrel hydrogen sulfate	-4.579
163680-77-1	Pazufloxacin Mesylate	-4.5782
34140-59-5	Trimebutine maleate	-4.5776
39133-31-8	Trimebutine	-4.5776
4800-94-6	Carbenicillin disodium	-4.5776
474-25-9	Chenodeoxycholic acid	-4.5775
24316-19-6	Cephalotaxine	-4.577
35554-44-0	Imazalil	-4.5767
4852-22-6	Proanthocyanidins	-4.5766
936091-26-8	Fedratinib	-4.5765
108605-62-5	Teriflunomide	-4.5764
163451-81-8	Teriflunomide	-4.5761
1405-10-3	Neomycin sulfate	-4.5759
13171-25-0	Trimetazidine dihydrochloride	-4.5757
150322-43-3	Prasugrel	-4.5757
132866-11-6	Lercanidipine hydrochloride	-4.5755
19237-84-4	Prazosin hydrochloride	-4.5752
112811-59-3	Gatifloxacin	-4.5749
29342-05-0	Ciclopirox	-4.5747
41621-49-2	Ciclopirox ethanolamine	-4.5747
161715-24-8	Tebipenem Pivoxil	-4.5746
3485-62-9	Clidinium bromide	-4.5746
65-31-6	Nicotine Ditartrate	-4.5742
141-94-6	Hexetidine	-4.574
145202-66-0	Rizatriptan benzoate	-4.5737
747-36-4	Hydroxychloroquine sulfate	-4.5735
149022-22-0	Amikacin Sulfate Salt	-4.5734
54-11-5	Nicotine	-4.573
96847-55-1	Levomilnacipran	-4.5728
100427-26-7	Lercanidipine	-4.5728
751-94-0	Sodium fusidate	-4.5725
6990-06-3	Fusidine	-4.5725
4852-22-6	Proanthocyanidins	-4.5722

59-63-2	Isocarboxazid	-4.5719
68497-62-1	Pramiracetam	-4.5716
24316-19-6	Cephalotaxine	-4.5711
64-72-2	Chlortetracycline hydrochloride	-4.571
59721-29-8	Camostat mesilate	-4.571
138844-81-2	Ibandronate sodium	-4.5709
6035-45-6	Folinic Acid Calcium Salt Pentahydrate	-4.5707
53179-09-2	Sisomicin Sulfate	-4.5707
61270-78-8	Cefonicid sodium	-4.5705
936091-26-8	Fedratinib	-4.5699
518048-05-0	Raltegravir	-4.5698
960203-27-4	Vortioxetine HBr	-4.5694
4800-94-6	Carbenicillin disodium	-4.5687
508233-74-7	Vortioxetine	-4.5685
58895-64-0	Nalmefene hydrochloride	-4.5683
72-80-0	Chloquinan	-4.568
64506-49-6	Sofalcone	-4.5677
83-73-8	Iodoquinol	-4.5673
4146-30-9	Neomycin Sulphate B	-4.5672
76824-35-6	Famotidine	-4.5665
4342-03-4	Dacarbazine	-4.5664
577-11-7	Docusate sodium	-4.5664
210353-53-0	Gemifloxacin?mesylate	-4.5661
73816-42-9	Meclocycline sulfosalicylate salt	-4.566
112665-43-7	Seratrodast	-4.5654
2353-33-5	Decitabine	-4.5639
123524-52-7	Azelnidipine	-4.5638
459868-92-9	Rucaparib Phosphate	-4.5638
283173-50-2	Rucaparib	-4.5638
145040-37-5	Candesartan Cilexetil	-4.5632
111406-87-2	Zileuton	-4.5629
1135-24-6	Ferulic Acid	-4.5626
24729-96-2	Clindamycin phosphate	-4.5626
149709-62-6	AHU377	-4.5619
1369773-39-6	AHU377 calcium salt	-4.5619
73590-58-6	Omeprazole	-4.5617
141-94-6	Hexetidine	-4.5616
16676-29-2	Naltrexone hydrochloride	-4.5616
1220-83-3	Sulfamonomethoxine	-4.5609
2259-96-3	Cyclothiazide	-4.5608
73816-42-9	Meclocycline sulfosalicylate salt	-4.5606
75330-75-5	Lovastatin	-4.5605

103980-44-5	Ceftiofur hydrochloride	-4.5605
102518-79-6	(-)-Huperzine A	-4.5603
33286-22-5	Diltiazem hydrochloride	-4.5599
112887-68-0	Raltitrexed	-4.5594
69004-03-1	Toltrazuril	-4.5593
20123-80-2	Calcium dobesilate	-4.5573
638-94-8	Desonide	-4.5566
863127-77-9	Dasatinib monohydrate	-4.5566
167465-36-3	Zosuquidar 3HCl	-4.5564
122320-73-4	Rosiglitazone	-4.5563
302962-49-8	Dasatinib	-4.5561
82186-77-4	Lumefantrine	-4.5558
1691249-45-2	zanubrutinib	-4.5553
1360460-82-7	Anlotinib Dihydrochloride	-4.5553
1058156-90-3	Anlotinib	-4.5553
286930-03-8	Fesoterodine fumarate	-4.5553
117977-21-6	Rabeprazole Related Compound E	-4.5552
79660-72-3	Fleroxacin	-4.555
302962-49-8	Dasatinib	-4.5548
2624-44-4	Ethamsylate	-4.5547
145040-37-5	Candesartan Cilexetil	-4.5547
1180-71-8	Limonin	-4.5539
122647-32-9	Ibutilide Fumarate	-4.5539
119-36-8	Methyl salicylate	-4.5535
122647-32-9	Ibutilide Fumarate	-4.5532
120410-24-4	Biapenem	-4.5531
22252-43-3	Cefalexine EP IMpurity B	-4.5529
6533-68-2	Scopolamine HBr trihydrate	-4.5529
67-03-8	Thiamine hydrochloride	-4.5512
747-36-4	Hydroxychloroquine sulfate	-4.551
71675-85-9	Amisulpride	-4.5509
66-76-2	Dicumarol	-4.5507
645-05-6	Altretamine	-4.5506
33342-05-1	Gliquidone	-4.5504
58-94-6	Chlorothiazide	-4.5502
210353-53-0	Gemifloxacin?mesylate	-4.55
64019-93-8	Dipivefrin hydrochloride	-4.5497
127-65-1	Chloramine-T	-4.5487
67-03-8	Thiamine hydrochloride	-4.5487
641571-10-0	Nilotinib	-4.5485

504-03-0	Lupetidin	-4.5478
138844-81-2	Ibandronate sodium	-4.5471
66-84-2	Glucosamine hydrochloride	-4.5466
1025687-58-4	R788 Disodium	-4.5462
66-76-2	Dicumarol	-4.5461
138844-81-2	Ibandronate sodium	-4.5459
1051375-16-6	Dolutegravir	-4.5456
71320-77-9	Moclobemide	-4.5455
2451-01-6	Terpin Monohydrate	-4.5454
68813-55-8	Oxantel Pamoate	-4.5453
79-57-2	Oxytetracycline	-4.5453
54-71-7	(+)-Pilocarpine hydrochloride	-4.5451
901119-35-5	Fostamatinib	-4.5451
745-65-3	Alprostadil	-4.5445
606143-52-6	Selumetinib	-4.5445
5534-09-8	Beclomethasone dipropionate	-4.5434
1207456-01-6	Talazoparib	-4.5432
773-76-2	Capitrol	-4.5428
68497-62-1	Pramiracetam	-4.542
4350-09-8	L-5-Hydroxytryptophan	-4.5419
138844-81-2	Ibandronate sodium	-4.5413
303-25-3	Cyclizine hydrochloride	-4.5408
57-96-5	Sulfinpyrazone	-4.5406
1405-10-3	Neomycin sulfate	-4.5405
18010-40-7	Bupivacaine hydrochloride	-4.5399
148-72-1	Pilocarpine nitrate	-4.5394
56391-57-2	Netilmicin sulfate	-4.5393
577-11-7	Docusate sodium	-4.5392
67-73-2	Fluocinolone acetonide	-4.5391
63968-64-9	Artemisinin	-4.5387
25389-94-0	Kanamycin sulfate	-4.5387
618385-01-6	Vorapaxar	-4.5383
94-20-2	Chlorpropamide	-4.5381
4852-22-6	Proanthocyanidins	-4.5367
107753-78-6	Zafirlukast	-4.5361
19237-84-4	Prazosin hydrochloride	-4.536
618385-01-6	Vorapaxar	-4.5356
1405-10-3	Neomycin sulfate	-4.5344
67814-76-0	Isepamicin sulfate	-4.5343
69056-38-8	Sapropterin Hydrochloride	-4.5343

150322-43-3	Prasugrel	-4.534
141-90-2	Deracil	-4.5335
1025504-45-3	Valbenazine	-4.5331
78613-38-4	Amorolfine hydrochloride	-4.5316
145202-66-0	Rizatriptan benzoate	-4.5313
78613-38-4	Amorolfine hydrochloride	-4.5312
141-94-6	Hexetidine	-4.531
4146-30-9	Neomycin Sulphate B	-4.531
61422-45-5	Carmofur	-4.5307
30751-05-4	Troxipide	-4.5305
155-41-9	Methscopolamine	-4.5305
4852-22-6	Proanthocyanidins	-4.5303
1354744-91-4	Naloxegol oxalate	-4.53
23541-50-6	Daunorubicin hydrochloride	-4.5293
1420477-60-6	Acalabrutinib	-4.5293
6160-12-9	(-)-Sparteine sulfate pentahydrate	-4.5292
1257044-40-8	ABT199	-4.5284
526-08-9	Sulfaphenazole	-4.5271
54-36-4	Metyrapone	-4.5262
1405-37-4	Capastat sulfate	-4.5258
1501-84-4	Rimantadine hydrochloride	-4.5257
66-76-2	Dicumarol	-4.5253
138402-11-6	Irbesartan	-4.5253
211914-51-1	Dabigatran	-4.525
25389-94-0	Kanamycin sulfate	-4.5249
2447-57-6	Sulfadoxine	-4.5244
31282-04-9	Hygromycin B	-4.5241
88495-63-0	Artesunate	-4.524
93106-60-6	Enrofloxacin	-4.524
1360460-82-7	Anlotinib Dihydrochloride	-4.5236
1058156-90-3	Anlotinib	-4.5236
70374-39-9	Lornoxicam	-4.5235
22252-43-3	Cefalexine EP IMpurity B	-4.5233
61825-94-3	Oxaliplatin	-4.5233
1492-18-8	Calcium folinate	-4.5232
64-72-2	Chlortetracycline hydrochloride	-4.5228
2624-44-4	Ethamsylate	-4.5222
145202-66-0	Rizatriptan benzoate	-4.5216

22888-70-6	Silibinin	-4.5214
20123-80-2	Calcium dobesilate	-4.5212
220119-17-5	Selamectin	-4.5212
298-57-7	Stugeron	-4.5209
18699-02-0	Actarit	-4.5205
107724-20-9	Eplerenone	-4.5202
1009119-64-5	Daclatasvir	-4.52
97-77-8	Disulfiram	-4.5195
101831-37-2	Diclazuril	-4.5194
84-02-6	Prochlorperazine Maleate	-4.5193
56391-57-2	Netilmicin sulfate	-4.5191
22881-35-2	Famprofazone	-4.5189
67814-76-0	Isepamicin sulfate	-4.5188
1490-04-6	DL-Menthol	-4.5187
70458-95-6	Pefloxacin Mesylate	-4.5184
4852-22-6	Proanthocyanidins	-4.5183
31282-04-9	Hygromycin B	-4.5182
177834-92-3	Eletriptan HBr	-4.5178
515-64-0	Sulfisomidine	-4.5177
81-25-4	Cholic Acid	-4.5176
1051375-19-9	Dolutegravir sodium	-4.5164
149676-40-4	Pefloxacin Mesylate Dihydrate	-4.5163
13412-64-1	Dicloxacillin sodium monohydrate	-4.5161
248281-84-7	Laquinimod	-4.516
122320-73-4	Rosiglitazone	-4.5158
52-01-7	Abbolactone	-4.5151
1231930-82-7	LY2835219	-4.5149
22254-24-6	Ipratropium Bromide	-4.5143
66985-17-9	Ipratropium bromide monohydrate	-4.5143
21462-39-5	Clindamycin HCl	-4.5141
937272-79-2	Pacritinib	-4.5139
58895-64-0	Nalmefene hydrochloride	-4.5133
630420-16-5	Asunaprevir	-4.5132
33069-62-4	Paclitaxel	-4.5129
127-79-7	Sulfamerazine	-4.5129
23210-58-4	Ifenprodil Tartrate	-4.5125
4852-22-6	Proanthocyanidins	-4.5122
71675-85-9	Amisulpride	-4.5119
90729-43-4	Ebastine	-4.5115
50-78-2	Aspirin	-4.5111

504-03-0	Lupetidin	-4.5105
749-02-0	Spiperone	-4.509
51940-44-4	Pipemidic acid	-4.5088
59-66-5	Acetazolamide	-4.5086
70374-39-9	Lornoxicam	-4.5082
25122-46-7	Clobetasol propionate	-4.5078
56391-57-2	Netilmicin sulfate	-4.5078
96847-55-1	Levomilnacipran	-4.5077
23210-58-4	Ifenprodil Tartrate	-4.5076
4852-22-6	Proanthocyanidins	-4.5073
19408-46-9	Kasugamycin hydrochloride	-4.5072
141-94-6	Hexetidine	-4.507
6160-12-9	(-)-Sparteine sulfate pentahydrate	-4.5068
23327-57-3	Nefopam hydrochloride	-4.5052
4852-22-6	Proanthocyanidins	-4.5048
6106-81-6	Scopolamine N-oxide HBr	-4.5046
25122-46-7	Clobetasol propionate	-4.5046
1672-63-5	4-Hydroxyantipyrine	-4.5043
56211-40-6	Torasemide	-4.5042
2353-33-5	Decitabine	-4.504
290297-26-6	Netupitant	-4.504
173903-47-4	Tizoxanide	-4.5036
15622-65-8	Molindone hydrochloride	-4.5028
105462-24-6	Risedronic Acid	-4.5027
115436-72-1	Risedronate Sodium	-4.5024
877399-52-5	Crizotinib	-4.5023
100986-85-4	Levofloxacin	-4.5017
138199-71-0	Levofloxacin Hemihydrate	-4.5017
82419-36-1	Ofloxacin	-4.5011
13412-64-1	Dicloxacillin sodium monohydrate	-4.5009
56-94-0	Demecarium bromide	-4.5004
87239-81-4	Cefpodoxime proxetil	-4.4998
1025687-58-4	R788 Disodium	-4.4997
4852-22-6	Proanthocyanidins	-4.4995
13609-67-1	Hydrocortisone butyrate	-4.4991
59338-87-3	Alizapride hydrochloride	-4.4989
901119-35-5	Fostamatinib	-4.4988
23541-50-6	Daunorubicin hydrochloride	-4.4987
129722-12-9	Aripiprazole	-4.4983
67814-76-0	Isepamicin sulfate	-4.4981
202189-78-4	Bilastine	-4.4977
65141-46-0	Nicorandil	-4.4971

1180-71-8	Limonin	-4.4968
31430-15-6	Flubendazole	-4.4964
577-11-7	Docusate sodium	-4.4957
112811-59-3	Gatifloxacin	-4.4956
68401-81-0	Ceftizoxime	-4.4954
66-76-2	Dicumarol	-4.4954
54527-84-3	Nicardipine hydrochloride	-4.495
111406-87-2	Zileuton	-4.4946
60-56-0	Methimazole	-4.4943
282526-98-1	Cetilistat	-4.4942
61-78-9	4-Aminohippuric Acid	-4.4936
93106-60-6	Enrofloxacin	-4.4926
122320-73-4	Rosiglitazone	-4.4925
94-16-6	Sodium 4-aminohippurate Hydrate	-4.4924
132810-10-7	Blonanserin	-4.4923
95635-55-5	Ranolazine	-4.4923
95635-56-6	Ranolazine dihydrochloride	-4.4923
282526-98-1	Cetilistat	-4.4919
4852-22-6	Proanthocyanidins	-4.4913
132810-10-7	Blonanserin	-4.4902
69655-05-6	Didanosine	-4.4894
53179-09-2	Sisomicin Sulfate	-4.4893
31430-15-6	Flubendazole	-4.4892
1009119-65-6	Daclatasvir dihydrochloride	-4.4886
66357-59-3	Ranitidine Hydrochloride	-4.4882
1360460-82-7	Anlotinib Dihydrochloride	-4.4877
1058156-90-3	Anlotinib	-4.4877
92134-98-0	Fosphenytoin sodium	-4.4876
69056-38-8	Sapropterin Hydrochloride	-4.4864
79902-63-9	Simvastatin	-4.4862
138844-81-2	Ibandronate sodium	-4.486
87-17-2	Salicylanilide	-4.486
850876-88-9	Danoprevir	-4.4859
141-94-6	Hexetidine	-4.4859
537-73-5	Isoferulic acid	-4.485
10402-53-6	Eprazinone dihydrochloride	-4.485
173334-58-2	Aliskiren hemifumarate	-4.4845
1405-10-3	Neomycin sulfate	-4.4844
66-76-2	Dicumarol	-4.4835
53797-35-6	Ribostamycin sulfate	-4.4832

152-95-4	Sophoricoside	-4.483
80474-14-2	Fluticasone propionate	-4.4828
903576-44-3	Pitolisant hydrochloride	-4.4818
521-74-4	Broxyquinoline	-4.4815
115256-11-6	Dofetilide	-4.4814
112885-42-4	Mosapride Citrate	-4.4813
177036-94-1	Ambrisentan	-4.4806
189188-57-6	Tegaserod maleate	-4.4805
23327-57-3	Nefopam hydrochloride	-4.4793
486460-32-6	Sitagliptin	-4.4791
2192-20-3	Hydroxyzine dihydrochloride	-4.4791
65-45-2	Salicylamide	-4.4789
357-08-4	Naloxone hydrochloride	-4.4788
504-03-0	Lupetidin	-4.4782
914462-92-3	Rolapitant hydrochloride	-4.4777
1104-22-9	Meclizine dihydrochloride	-4.477
51333-22-3	Budesonide	-4.4769
537-73-5	Isoferulic acid	-4.4768
577-11-7	Docusate sodium	-4.4762
747-36-4	Hydroxychloroquine sulfate	-4.476
98753-19-6	Cefpirome sulfate	-4.4759
58895-64-0	Nalmefene hydrochloride	-4.4756
10246-75-0	Hydroxyzine Pamoate	-4.4752
364622-82-2	Doripenem Hydrate	-4.4751
72-80-0	Chloquinan	-4.4747
1137608-69-5	Telotristat Etiprate	-4.4741
18699-02-0	Actarit	-4.4731
84057-84-1	Lamotrigine	-4.4724
68302-57-8	Amlexanox	-4.4714
84611-23-4	Erdosteine	-4.4708
459868-92-9	Rucaparib Phosphate	-4.4702
283173-50-2	Rucaparib	-4.4702
1263-89-4	Paromomycin Sulfate	-4.47
4697-14-7	Ticarcillin disodium	-4.4696
4146-30-9	Neomycin Sulphate B	-4.4693
964-52-3	Moxisylyte hydrochloride	-4.4691
22373-78-0	Monensin sodium salt	-4.4685
6024-85-7	Tetrahydropalmatine hydrochloride	-4.4678
67227-57-0	Fenoldopam mesylate	-4.4678
24390-14-5	Doxycycline hyclate	-4.4675
936091-26-8	Fedratinib	-4.4675

1229194-11-9	Edoxaban Tosylate Monohydrate	-4.4669
162359-56-0	Fingolimod hydrochloride	-4.4667
1197953-54-0	Brigatinib	-4.4666
282526-98-1	Cetilistat	-4.4663
4852-22-6	Proanthocyanidins	-4.4663
64019-93-8	Dipivefrin hydrochloride	-4.4662
168555-66-6	Fosbretabulin Disodium	-4.4656
773092-05-0	Acotiamide hydrochloride	-4.4652
951395-08-7	tafamidis meglumine	-4.4643
912273-65-5	Edoxaban	-4.4642
94-20-2	Chlorpropamide	-4.464
64953-12-4	Latamoxef sodium	-4.464
936539-80-9	Betrixaban maleate	-4.4634
T4690	Betrixaban hydrochloride(330942-05-7(free base))	-4.4634
29908-03-0	Ademetionine	-4.4632
154229-18-2	Abiraterone Acetate	-4.463
65666-07-1	Silymarin	-4.462
1490-04-6	DL-Menthol	-4.4613
4852-22-6	Proanthocyanidins	-4.4609
1405-10-3	Neomycin sulfate	-4.4606
67814-76-0	Isepamicin sulfate	-4.4605
25122-46-7	Clobetasol propionate	-4.46
67227-57-0	Fenoldopam mesylate	-4.4596
134308-13-7	Tolcapone	-4.4593
101831-37-2	Diclazuril	-4.4588
57808-66-9	Domperidone	-4.4587
21829-25-4	Nifedipine	-4.4586
115256-11-6	Dofetilide	-4.4584
76824-35-6	Famotidine	-4.4584
827022-33-3	Palbociclib Isethionate	-4.4583
827022-32-2	Palbociclib hydrochloride	-4.4583
571190-30-2	Palbociclib	-4.4583
526-18-1	Osalmid	-4.4568
173903-47-4	Tizoxanide	-4.4568
1132935-63-7	Dasabuvir	-4.4566
130-40-5	Flavin mononucleotide	-4.4565
1257044-40-8	ABT199	-4.4563
554-57-4	Methazolamide	-4.4562
27591-69-1	Tilorone dihydrochloride	-4.4557
149709-62-6	AHU377	-4.4544

1369773-39-6	AHU377 calcium salt	-4.4544
189188-57-6	Tegaserod maleate	-4.4539
59338-87-3	Alizapride hydrochloride	-4.4531
73151-29-8	Fenticonazole Nitrate	-4.4531
69304-47-8	Brivudine	-4.4529
611-71-2	Mandelic acid	-4.4527
79307-93-0	Azelastine hydrochloride	-4.4525
67814-76-0	Isepamicin sulfate	-4.4524
224452-66-8	Retapamulin	-4.4523
155148-31-5	Plerixafor 8HCl (AMD3100 8HCl)	-4.4522
110078-46-1	Plerixafor	-4.4522
34562-97-5	Pikamilone	-4.4521
56-69-9	5-hydroxytryptophan	-4.452
504-03-0	Lupetidin	-4.4518
77-36-1	Chlortalidone	-4.4518
143388-64-1	Naratriptan	-4.4515
82571-53-7	Ozagrel	-4.4512
84680-54-6	Enalaprilat Dihydrate	-4.451
84611-23-4	Erdosteine	-4.4508
154229-18-2	Abiraterone Acetate	-4.4505
496-67-3	Bromisoval	-4.4505
78712-43-3	Ozagrel hydrochloride	-4.4503
956697-53-3	NVP-LDE225	-4.4503
1025504-45-3	Valbenazine	-4.45
1405-86-3	Glycyrrhizin	-4.45
79165-06-3	Diammonium Glycyrrhizinate	-4.45
827022-33-3	Palbociclib Isethionate	-4.4497
827022-32-2	Palbociclib hydrochloride	-4.4497
71-58-9	Medroxyprogesterone Acetate	-4.4495
890-38-0	2'-Deoxyinosine	-4.4494
34140-59-5	Trimebutine maleate	-4.4493
39133-31-8	Trimebutine	-4.4493
571190-30-2	Palbociclib	-4.4491
71675-85-9	Amisulpride	-4.449
107753-78-6	Zafirlukast	-4.4489
59-66-5	Acetazolamide	-4.4485
73-48-3	Bendroflumethiazide	-4.4482
4852-22-6	Proanthocyanidins	-4.448
4146-30-9	Neomycin Sulphate B	-4.4477
1435-55-8	Hydroquinidine	-4.4475
31282-04-9	Hygromycin B	-4.4474

4852-22-6	Proanthocyanidins	-4.4473
483-63-6	Crotamiton	-4.4471
123441-03-2	Rivastigmine	-4.4467
59721-29-8	Camostat mesilate	-4.4467
1231929-97-7	Abemaciclib	-4.4466
330808-88-3	Vardenafil hydrochloride trihydrate	-4.4463
129101-54-8	Rivastigmine tartrate	-4.4461
3697-42-5	Chlorhexidine hydrochloride	-4.4457
56-95-1	Chlorhexidine Acetate	-4.4457
84625-61-6	Itraconazole	-4.4455
138-14-7	Deferoxamine Mesylate	-4.444
618385-01-6	Vorapaxar	-4.4438
951395-08-7	tafamidis meglumine	-4.4435
4800-94-6	Carbenicillin disodium	-4.4433
651-06-9	Sulfameter	-4.443
864750-70-9	Revefenacin	-4.4419
111025-46-8	Pioglitazone	-4.4416
7512-17-6	N-Acetyl-D-Glucosamine	-4.4406
123441-03-2	Rivastigmine	-4.4406
59-66-5	Acetazolamide	-4.4405
84-02-6	Prochlorperazine Maleate	-4.4401
19408-46-9	Kasugamycin hydrochloride	-4.4393
645-05-6	Altretamine	-4.4388
74103-07-4	Ketorolac tromethamine salt	-4.4386
137975-06-5	Mozavaptan	-4.4382
171599-83-0	Sildenafil citrate	-4.438
59338-87-3	Alizapride hydrochloride	-4.4378
1360460-82-7	Anlotinib Dihydrochloride	-4.4377
1058156-90-3	Anlotinib	-4.4377
81-27-6	Sennoside A	-4.4372
129101-54-8	Rivastigmine tartrate	-4.4367
65141-46-0	Nicorandil	-4.4366
1220-83-3	Sulfamonomethoxine	-4.4362
111406-87-2	Zileuton	-4.4361
87-17-2	Salicylanilide	-4.4352
3562-84-3	Benzbromarone	-4.4333
66357-59-3	Ranitidine Hydrochloride	-4.432
745-65-3	Alprostadil	-4.432
220119-17-5	Selamectin	-4.4318
149003-01-0	Dexrazoxane HCl	-4.4313

4008-48-4	Nitroxoline	-4.4306
66215-27-8	Cyromazine	-4.4306
22457-89-2	Benfotiamine	-4.4305
77-36-1	Chlortalidone	-4.4304
42461-84-7	Flunixin meglumin	-4.4303
97-18-7	Bithionol	-4.4303
66357-59-3	Ranitidine Hydrochloride	-4.43
33286-22-5	Diltiazem hydrochloride	-4.4293
138844-81-2	Ibandronate sodium	-4.4289
1229-29-4	Doxepin hydrochloride	-4.4289
210353-53-0	Gemifloxacin?mesylate	-4.4288
75438-57-2	Moxonidine	-4.4281
621-72-7	Bendazol	-4.4266
97-77-8	Disulfiram	-4.4264
107133-36-8	Perindopril erbumine	-4.4262
54-91-1	Pipobroman	-4.4258
123524-52-7	Azelnidipine	-4.4251
4394-00-7	Niflumic acid	-4.425
5560-59-8	Alverine citrate	-4.425
1722-62-9	Mepivacaine hydrochloride	-4.4249
6078-17-7	Berbamine dihydrochloride	-4.4242
103628-48-4	Sumatriptan succinate	-4.4234
59-05-2	Methotrexate	-4.4234
6055-19-2	Cyclophosphamide monohydrate	-4.423
500287-72-9	Rilpivirine	-4.4227
2259-96-3	Cyclothiazide	-4.4222
68099-86-5	Bepridil hydrochloride	-4.422
4852-22-6	Proanthocyanidins	-4.4216
1207456-01-6	Talazoparib	-4.4211
951395-08-7	tafamidis meglumine	-4.4209
890-38-0	2'-Deoxyinosine	-4.4209
122-11-2	Sulfadimethoxine	-4.4204
183133-96-2	Cabazitaxel	-4.4194
72-14-0	Sulfathiazole	-4.4193
1009119-64-5	Daclatasvir	-4.4193
1811-28-5	Proflavine Hemisulfate	-4.4191
66357-59-3	Ranitidine Hydrochloride	-4.4189
1009119-65-6	Daclatasvir dihydrochloride	-4.4176
903576-44-3	Pitolisant hydrochloride	-4.4175
66215-27-8	Cyromazine	-4.417

17696-69-4	Hematoporphyrin dihydrochloride	-4.4166
7179-49-9	Lincomycin hydrochloride monohydrate	-4.4165
859-18-7	Lincomycin hydrochloride	-4.4165
1041434-82-5	Peramivir Trihydrate	-4.4162
66215-27-8	Cyromazine	-4.4157
73590-58-6	Omeprazole	-4.415
141-94-6	Hexetidine	-4.4147
2295-58-1	Flopropione	-4.414
41354-29-4	Cyproheptadine hydrochloride	-4.4137
56-92-8	Histamine 2HCl	-4.4134
51-45-6	Histamine	-4.4134
58895-64-0	Nalmefene hydrochloride	-4.4132
101831-37-2	Diclazuril	-4.4126
4852-22-6	Proanthocyanidins	-4.4125
1403254-99-8	EPZ6438	-4.4124
872728-81-9	Dabigatran Etexilate Mesylate	-4.4123
211915-06-9	Dabigatran etexilate	-4.4123
751-94-0	Sodium fusidate	-4.4122
6990-06-3	Fusidine	-4.4122
4180-23-8	Trans-Anethole	-4.4121
125-02-0	Prednisolone phosphate sodium	-4.4119
132539-06-1	Olanzapine	-4.4114
29908-03-0	Ademetionine	-4.4112
23964-57-0	Articaine HCl	-4.4112
141625-93-6	Dronedarone hydrochloride	-4.4105
951395-08-7	tafamidis meglumine	-4.4101
594839-88-0	Tafamidis	-4.4099
4852-22-6	Proanthocyanidins	-4.4094
126-27-2	Oxethazaine	-4.409
29868-97-1	Pirenzepine dihydrochloride	-4.409
68302-57-8	Amlexanox	-4.4087
74536-44-0	Metadoxine	-4.4083
58-56-0	Pyridoxine hydrochloride	-4.4083
65-23-6	Pyridoxine	-4.4083
596-51-0	Glycopyrrolate	-4.4078
79660-72-3	Fleroxacin	-4.4076
23210-58-4	Ifenprodil Tartrate	-4.407
177036-94-1	Ambrisentan	-4.4063
595-33-5	Megestrol acetate	-4.4063
4852-22-6	Proanthocyanidins	-4.4063
63675-72-9	Nisoldipine	-4.4062

93793-83-0	Roxatidine Acetate hydrochloride	-4.4061
496-67-3	Bromisoval	-4.4055
961-29-5	Isoliquiritigenin	-4.4048
141626-36-0	Dronedarone	-4.4045
1611493-60-7	Bictegravir	-4.4043
93793-83-0	Roxatidine Acetate hydrochloride	-4.4042
23210-58-4	Ifenprodil Tartrate	-4.404
4852-22-6	Proanthocyanidins	-4.4032
102518-79-6	(-)-Huperzine A	-4.403
137975-06-5	Mozavaptan	-4.4028
1405-10-3	Neomycin sulfate	-4.4025
111902-57-9	Temocapil	-4.402
68099-86-5	Bepridil hydrochloride	-4.4019
1405-37-4	Capastat sulfate	-4.4017
25389-94-0	Kanamycin sulfate	-4.4016
705260-08-8	Vorapaxar sulfate	-4.4016
773092-05-0	Acotiamide hydrochloride	-4.4015
121123-17-9	Cefprozil hydrate	-4.4014
1405-37-4	Capastat sulfate	-4.4014
69056-38-8	Sapropterin Hydrochloride	-4.4011
139264-17-8	Zolmitriptan	-4.4008
121123-17-9	Cefprozil hydrate	-4.4007
1110813-31-4	Dacomitinib	-4.4
132866-11-6	Lercanidipine hydrochloride	-4.3998
122647-32-9	Ibutilide Fumarate	-4.3998
469-77-2	Spiculisporic Acid	-4.3994
577-11-7	Docusate sodium	-4.3994
596-51-0	Glycopyrrolate	-4.3993
123524-52-7	Azelnidipine	-4.3986
330808-88-3	Vardenafil hydrochloride trihydrate	-4.3977
53-86-1	Indometacin	-4.3977
7081-44-9	Cloxacillin sodium monohydrate	-4.3975
100427-26-7	Lercanidipine	-4.3971
65666-07-1	Silymarin	-4.3971
163680-77-1	Pazufloxacin Mesylate	-4.397
330808-88-3	Vardenafil hydrochloride trihydrate	-4.3959
117467-28-4	Cefditoren pivoxil	-4.3959
90098-04-7	Rebamipide	-4.3949
611-71-2	Mandelic acid	-4.3945
59338-87-3	Alizapride hydrochloride	-4.3937
918504-65-1	Vemurafenib	-4.3936

427-51-0	Cyproterone acetate	-4.3935
936091-26-8	Fedratinib	-4.3935
51-35-4	L-Hydroxyprolin	-4.3934
4146-30-9	Neomycin Sulphate B	-4.3934
1435-55-8	Hydroquinidine	-4.3933
98079-52-8	Lomefloxacin hydrochloride	-4.3931
98079-51-7	Lomefloxacin	-4.3931
53179-09-2	Sisomicin Sulfate	-4.3929
871038-72-1	Raltegravir potassium	-4.3929
74536-44-0	Metadoxine	-4.3925
58-56-0	Pyridoxine hydrochloride	-4.3925
65-23-6	Pyridoxine	-4.3925
1263-89-4	Paromomycin Sulfate	-4.3921
119478-55-6	Danofloxacin mesylate	-4.3912
24280-93-1	Mycophenolic acid	-4.391
29342-05-0	Ciclopirox	-4.3908
41621-49-2	Ciclopirox ethanolamine	-4.3908
4852-22-6	Proanthocyanidins	-4.3906
153504-70-2	Cevimeline hydrochloride hemihydrate	-4.3904
147526-32-7	Pitavastatin calcium	-4.3902
127294-70-6	Balofloxacin	-4.3899
3963-95-9	Methacycline hydrochloride	-4.3895
25389-94-0	Kanamycin sulfate	-4.3893
554-57-4	Methazolamide	-4.3892
804-30-8	Fursultiamine	-4.389
38194-50-2	Sulindac	-4.3887
4852-22-6	Proanthocyanidins	-4.3877
23674-86-4	Difluprednate	-4.3877
738606-46-7	ETC1002	-4.3871
773092-05-0	Acotiamide hydrochloride	-4.3871
56211-40-6	Torasemide	-4.387
63675-72-9	Nisoldipine	-4.3869
54527-84-3	Nicardipine hydrochloride	-4.3868
54965-21-8	Albendazole	-4.3862
14484-47-0	Deflazacort	-4.3859
84-02-6	Prochlorperazine Maleate	-4.3856
79559-97-0	Sertraline hydrochloride	-4.3854
4697-14-7	Ticarcillin disodium	-4.3851
136790-76-6	Lubiprostone	-4.3846
141-94-6	Hexetidine	-4.3842
55203-24-2	Dexamethasone Sodium Phosphate	-4.384
695-53-4	5,5-Dimethyloxazolidine-2,4-dione	-4.3839
536-43-6	Dyclonine hydrochloride	-4.383

1137608-69-5	Telotristat Etiprate	-4.3825
86780-90-7	Aranidipine	-4.3824
67814-76-0	Isepamicin sulfate	-4.3822
72-80-0	Chloquinan	-4.3816
54-91-1	Pipobroman	-4.3814
123524-52-7	Azelnidipine	-4.3813
147526-32-7	Pitavastatin calcium	-4.3811
56-92-8	Histamine 2HCl	-4.381
51-45-6	Histamine	-4.381
1365970-03-1	Glecaprevir	-4.3809
618385-01-6	Vorapaxar	-4.3806
65-45-2	Salicylamide	-4.3798
83915-83-7	Lisinopril dihydrate	-4.3793
141-94-6	Hexetidine	-4.378
1403254-99-8	EPZ6438	-4.3776
97-59-6	Allantoin	-4.3776
1256580-46-7	Alectinib	-4.3768
66-84-2	Glucosamine hydrochloride	-4.3766
108612-45-9	Mizolastine	-4.3761
4800-94-6	Carbenicillin disodium	-4.376
1263-89-4	Paromomycin Sulfate	-4.3758
54965-21-8	Albendazole	-4.3756
1230487-00-9	Siponimod	-4.3756
144-83-2	Sulfapyridine	-4.3754
90098-04-7	Rebamipide	-4.3747
6024-85-7	Tetrahydropalmatine hydrochloride	-4.3747
6506-37-2	Nimorazole	-4.3746
13636-18-5	Fendiline hydrochloride	-4.3743
56-53-1	Diethylstilbestrol	-4.3731
6078-17-7	Berbamine dihydrochloride	-4.3729
210353-53-0	Gemifloxacin?mesylate	-4.3728
1180-71-8	Limonin	-4.3728
6160-12-9	(-)-Sparteine sulfate pentahydrate	-4.3726
380843-75-4	Bosutinib	-4.371
61825-94-3	Oxaliplatin	-4.3694
483-63-6	Crotamiton	-4.3688
330942-05-7	Betrixaban	-4.3686
1338225-97-0	Doravirine	-4.3684

4852-22-6	Proanthocyanidins	-4.3681
105628-07-7	Fasudil hydrochloride	-4.3679
67-73-2	Fluocinolone acetonide	-4.3679
82159-09-9	Epalrestat	-4.3672
1360460-82-7	Anlotinib Dihydrochloride	-4.3663
1058156-90-3	Anlotinib	-4.3663
6078-17-7	Berbamine dihydrochloride	-4.3661
82186-77-4	Lumefantrine	-4.366
427-51-0	Cyproterone acetate	-4.3656
145040-37-5	Candesartan Cilexetil	-4.3645
303-25-3	Cyclizine hydrochloride	-4.3641
33419-42-0	Etoposide	-4.364
1405-37-4	Capastat sulfate	-4.3639
72432-03-2	Miglitol	-4.3636
6506-37-2	Nimorazole	-4.3633
107868-30-4	Exemestane	-4.3617
859-18-7	Lincomycin hydrochloride	-4.361
53179-09-2	Sisomicin Sulfate	-4.3598
380843-75-4	Bosutinib	-4.3585
1435-55-8	Hydroquinidine	-4.358
1722-62-9	Mepivacaine hydrochloride	-4.358
705260-08-8	Vorapaxar sulfate	-4.3576
124937-52-6	Tolterodine tartrate	-4.3571
24729-96-2	Clindamycin phosphate	-4.3561
138890-62-7	Brinzolamide	-4.3556
27833-64-3	Loxapine succinate salt	-4.3552
951395-08-7	tafamidis meglumine	-4.3548
125317-39-7	Vinorelbine Tartrate	-4.3547
67814-76-0	Isepamicin sulfate	-4.3546
56211-40-6	Torasemide	-4.3544
7179-49-9	Lincomycin hydrochloride monohydrate	-4.3544
2066-89-9	Pasiniazid	-4.3538
80-50-2	Anisotropine Methylbromide	-4.3535
56-69-9	5-hydroxytryptophan	-4.353
31282-04-9	Hygromycin B	-4.353
21736-83-4	Spectinomycin dihydrochloride	-4.3525
55203-24-2	Dexamethasone Sodium Phosphate	-4.3524
1137608-69-5	Telotristat Etiprate	-4.3523
181183-52-8	Almotriptan Malate	-4.3515
138844-81-2	Ibandronate sodium	-4.3511

64-73-3	Demeclocycline hydrochloride	-4.3511
122647-32-9	Ibutilide Fumarate	-4.3511
102518-79-6	(-)-Huperzine A	-4.3509
22232-54-8	Carbimazole	-4.3506
606-04-2	Pamabrom	-4.3505
108605-62-5	Teriflunomide	-4.3502
163451-81-8	Teriflunomide	-4.3499
1722-62-9	Mepivacaine hydrochloride	-4.3496
1226781-44-7	Omarigliptin	-4.3492
537-12-2	Diperodon hydrochloride	-4.3491
54965-24-1	Tamoxifen Z-isomer citrate	-4.3487
181183-52-8	Almotriptan Malate	-4.3486
32986-56-4	Tobramycin	-4.3482
123447-62-1	Prulifloxacin	-4.3481
945667-22-1	Saxagliptin hydrate	-4.348
330942-05-7	Betrixaban	-4.3477
1263-89-4	Paromomycin Sulfate	-4.3472
10540-29-1	Tamoxifen	-4.347
68099-86-5	Bepridil hydrochloride	-4.3469
24729-96-2	Clindamycin phosphate	-4.3469
32986-56-4	Tobramycin	-4.3464
24729-96-2	Clindamycin phosphate	-4.346
3160-91-6	Moroxydine hydrochloride	-4.3458
93106-60-6	Enrofloxacin	-4.3457
93479-97-1	Glimepiride	-4.3454
126-07-8	Griseofulvin	-4.3453
554-57-4	Methazolamide	-4.3446
1257044-40-8	ABT199	-4.344
1226781-44-7	Omarigliptin	-4.3439
2295-58-1	Flopropione	-4.3438
115939-25-8	Salvianolic acid B	-4.3435
71963-77-4	Artemether	-4.3429
88495-63-0	Artesunate	-4.3422
23210-58-4	Ifenprodil Tartrate	-4.3421
747-36-4	Hydroxychloroquine sulfate	-4.3417
59-05-2	Methotrexate	-4.3416
23327-57-3	Nefopam hydrochloride	-4.3415
6160-12-9	(-)-Sparteine sulfate pentahydrate	-4.3409
97-59-6	Allantoin	-4.3406
111406-87-2	Zileuton	-4.3403

29908-03-0	Ademetionine	-4.3398
1229194-11-9	Edoxaban Tosylate Monohydrate	-4.3394
97-18-7	Bithionol	-4.3392
90-22-2	Valethamate bromide	-4.3392
168555-66-6	Fosbretabulin Disodium	-4.3391
1722-62-9	Mepivacaine hydrochloride	-4.3389
71-58-9	Medroxyprogesterone Acetate	-4.3387
303-25-3	Cyclizine hydrochloride	-4.3384
13010-47-4	Lomustine	-4.3383
29868-97-1	Pirenzepine dihydrochloride	-4.3381
77-36-1	Chlortalidone	-4.3374
75438-57-2	Moxonidine	-4.3368
1476-53-5	Novobiocin Sodium	-4.3363
173352-39-1	Afobazole hydrochloride	-4.3363
79660-72-3	Fleroxacin	-4.3361
51-15-0	Pralidoxime Chloride	-4.336
1110813-31-4	Dacomitinib	-4.336
14611-52-0	Selegiline hydrochloride	-4.3359
125317-39-7	Vinorelbine Tartrate	-4.3357
145202-66-0	Rizatriptan benzoate	-4.3355
111974-72-2	Quetiapine Fumarate	-4.335
300-08-3	Arecoline HBr	-4.334
25389-94-0	Kanamycin sulfate	-4.3333
918504-65-1	Vemurafenib	-4.3331
80474-14-2	Fluticasone propionate	-4.333
65-85-0	Benzoic acid	-4.3322
1421373-65-0	Osimertinib	-4.3316
127-79-7	Sulfamerazine	-4.3315
32986-56-4	Tobramycin	-4.3312
22373-78-0	Monensin sodium salt	-4.3299
102518-79-6	(-)-Huperzine A	-4.3294
113852-37-2	Cidofovir	-4.3293
95789-30-3	Cefotiam Hexetil Hydrochloride	-4.3292
4852-22-6	Proanthocyanidins	-4.3288
125-84-8	Aminoglutethimide	-4.3282
125317-39-7	Vinorelbine Tartrate	-4.328
145202-66-0	Rizatriptan benzoate	-4.3278
1421373-66-1	Osimertinib mesylate	-4.3274
70-00-8	Trifluridine	-4.3273
4852-22-6	Proanthocyanidins	-4.3268

50-65-7	Niclosamide	-4.3265
69655-05-6	Didanosine	-4.3261
13636-18-5	Fendiline hydrochloride	-4.3258
635702-64-6	Pazopanib HCl	-4.3258
444731-52-6	Pazopanib	-4.3258
122647-32-9	Ibutilide Fumarate	-4.3257
65141-46-0	Nicorandil	-4.3243
877399-52-5	Crizotinib	-4.3243
1405-10-3	Neomycin sulfate	-4.3238
27591-69-1	Tilorone dihydrochloride	-4.3235
123524-52-7	Azelnidipine	-4.3231
115256-11-6	Dofetilide	-4.3224
56-92-8	Histamine 2HCl	-4.3222
51-45-6	Histamine	-4.3222
71963-77-4	Artemether	-4.3222
4759-48-2	Isotretinoin	-4.3219
75507-68-5	Flupirtine maleate	-4.3218
1421373-65-0	Osimertinib	-4.3217
14611-52-0	Selegiline hydrochloride	-4.3209
64-73-3	Demeclocycline hydrochloride	-4.3204
321-30-2	Adenine sulfate	-4.3202
73-24-5	Adenine	-4.3202
24390-14-5	Doxycycline hyclate	-4.3201
1220-83-3	Sulfamonomethoxine	-4.3193
95635-56-6	Ranolazine dihydrochloride	-4.3193
1421373-66-1	Osimertinib mesylate	-4.3193
536-43-6	Dyclonine hydrochloride	-4.3188
92134-98-0	Fosphenytoin sodium	-4.3188
95635-55-5	Ranolazine	-4.3187
6469-93-8	Chlorprothixene hydrochloride	-4.3186
113-59-7	Chlorprothixene	-4.3186
56-89-3	cystine	-4.318
102-76-1	Triacetin	-4.3176
3380-34-5	Triclosan	-4.3176
163521-08-2	Vilazodone HCl	-4.3174
79350-37-1	Cefixime	-4.317
621-72-7	Bendazol	-4.3167
1611493-60-7	Bictegravir	-4.3165
66357-59-3	Ranitidine Hydrochloride	-4.3162
4146-30-9	Neomycin Sulphate B	-4.3157

302962-49-8	Dasatinib	-4.3154
80-35-3	Sulfamethoxypyridazine	-4.3152
139481-59-7	Candesartan	-4.3142
32986-56-4	Tobramycin	-4.3139
164650-44-6	Efinaconazole	-4.3138
137975-06-5	Mozavaptan	-4.3138
51-15-0	Pralidoxime Chloride	-4.3138
1501-84-4	Rimantadine hydrochloride	-4.3131
82186-77-4	Lumefantrine	-4.3127
4852-22-6	Proanthocyanidins	-4.3124
150322-43-3	Prasugrel	-4.3115
130693-82-2	Dorzolamide hydrochloride	-4.3106
95635-55-5	Ranolazine	-4.3102
153-18-4	Rutin	-4.31
164650-44-6	Efinaconazole	-4.31
13010-47-4	Lomustine	-4.3096
606-04-2	Pamabrom	-4.3092
72-14-0	Sulfathiazole	-4.3083
108612-45-9	Mizolastine	-4.3081
122647-32-9	Ibutilide Fumarate	-4.3081
163451-81-8	Teriflunomide	-4.308
108605-62-5	Teriflunomide	-4.308
330808-88-3	Vardenafil hydrochloride trihydrate	-4.3075
95635-56-6	Ranolazine dihydrochloride	-4.3072
140-87-4	Cyanoacetohydrazide	-4.3068
439081-18-2	Afatinib	-4.306
850140-73-7	Afatinib Dimaleate	-4.3051
132810-10-7	Blonanserin	-4.3049
41100-52-1	Memantine hydrochloride	-4.3042
15622-65-8	Molindone hydrochloride	-4.3041
112665-43-7	Seratrodast	-4.3036
3380-34-5	Triclosan	-4.3034
87-17-2	Salicylanilide	-4.3029
29908-03-0	Ademetionine	-4.3029
145040-37-5	Candesartan Cilexetil	-4.3028
84057-95-4	Ropivacaine	-4.3027
98717-15-8	Ropivacaine hydrochloride	-4.3027
122892-31-3	Itopride hydrochloride	-4.3021
15686-71-2	Cephalexin	-4.3021
19408-46-9	Kasugamycin hydrochloride	-4.302
1403254-99-8	EPZ6438	-4.302
81-23-2	Dehydrocholic acid	-4.3016

T4284	(S)-Amlodipine Besylate (103129-82-4(free base))	-4.3013
88150-42-9	Amlodipine	-4.3013
139264-17-8	Zolmitriptan	-4.3008
122647-32-9	Ibutilide Fumarate	-4.2999
61336-70-7	Amoxicillin trihydrate	-4.299
26787-78-0	Amoxicillin	-4.299
4852-22-6	Proanthocyanidins	-4.2978
22373-78-0	Monensin sodium salt	-4.2975
290297-26-6	Netupitant	-4.2973
108605-62-5	Teriflunomide	-4.2967
163451-81-8	Teriflunomide	-4.2966
446-72-0	Genistein	-4.2965
149022-22-0	Amikacin Sulfate Salt	-4.2964
50-63-5	Chloroquine diphosphate	-4.2962
108605-62-5	Teriflunomide	-4.2957
163451-81-8	Teriflunomide	-4.2946
1009119-65-6	Daclatasvir dihydrochloride	-4.2945
23210-58-4	Ifenprodil Tartrate	-4.2944
4852-22-6	Proanthocyanidins	-4.2941
111025-46-8	Pioglitazone	-4.2937
537-12-2	Diperodon hydrochloride	-4.2937
459789-99-2	Obeticholic Acid	-4.2933
59-30-3	Folic acid	-4.2931
35554-44-0	Imazalil	-4.2926
123447-62-1	Prulifloxacin	-4.2924
6055-19-2	Cyclophosphamide monohydrate	-4.2923
705260-08-8	Vorapaxar sulfate	-4.2918
1405-86-3	Glycyrrhizin	-4.2915
79165-06-3	Diammonium Glycyrrhizinate	-4.2915
115939-25-8	Salvianolic acid B	-4.2913
6055-19-2	Cyclophosphamide monohydrate	-4.2911
936091-26-8	Fedratinib	-4.2909
72-14-0	Sulfathiazole	-4.2908
446-72-0	Genistein	-4.2905
1405-37-4	Capastat sulfate	-4.2905
31282-04-9	Hygromycin B	-4.2904
157810-81-6	Indinavir sulfate	-4.2902
4759-48-2	Isotretinoin	-4.2901
38194-50-2	Sulindac	-4.2897
94-09-7	Benzocaine	-4.2895
4852-22-6	Proanthocyanidins	-4.2893
22881-35-2	Famprofazone	-4.289

606-04-2	Pamabrom	-4.2889
945667-22-1	Saxagliptin hydrate	-4.2889
66532-86-3	Propacetamol Hydrochloride	-4.2889
1421373-66-1	Osimertinib mesylate	-4.2885
1257044-40-8	ABT199	-4.2877
102518-79-6	(-)-Huperzine A	-4.2874
99464-64-9	Ampiroxicam	-4.2873
4852-22-6	Proanthocyanidins	-4.2868
163451-81-8	Teriflunomide	-4.2866
67227-57-0	Fenoldopam mesylate	-4.2866
4852-22-6	Proanthocyanidins	-4.2862
4852-22-6	Proanthocyanidins	-4.2859
108605-62-5	Teriflunomide	-4.2857
74050-98-9	Ketanserin	-4.2854
1421373-65-0	Osimertinib	-4.2849
936539-80-9	Betrixaban maleate	-4.2844
T4690	Betrixaban hydrochloride(330942-05-7(free base))	-4.2844
97-18-7	Bithionol	-4.2843
322-79-2	Triflusal	-4.2839
4852-22-6	Proanthocyanidins	-4.2836
89226-75-5	Manidipine dihydrochloride	-4.2835
3160-91-6	Moroxydine hydrochloride	-4.283
67-43-6	Pentetic Acid	-4.2825
65-45-2	Salicylamide	-4.2823
29608-49-9	Almitrine dimesylate	-4.282
67227-57-0	Fenoldopam mesylate	-4.2819
22199-08-2	Silver sulfadiazine	-4.2814
50-78-2	Aspirin	-4.2804
76824-35-6	Famotidine	-4.2803
23210-58-4	Ifenprodil Tartrate	-4.2802
968-81-0	Acetohexamide	-4.2798
112887-68-0	Raltitrexed	-4.2796
637-32-1	Proguanil hydrochloride	-4.2794
1041434-82-5	Peramivir Trihydrate	-4.2793
34031-32-8	Auranofin	-4.2781
115256-11-6	Dofetilide	-4.2774
89226-50-6	Manidipine	-4.2772
357-08-4	Naloxone hydrochloride	-4.2771
177834-92-3	Eletriptan HBr	-4.277
737789-87-6	Relugolix	-4.2765

85650-56-2	Asenapine Maleate	-4.2765
124832-27-5	Valacyclovir hydrochloride	-4.2761
76824-35-6	Famotidine	-4.2761
7554-65-6	4-Methylpyrazole	-4.2754
125-02-0	Prednisolone phosphate sodium	-4.2751
4852-22-6	Proanthocyanidins	-4.2748
54-35-3	Procaine penicillin G	-4.2738
138844-81-2	Ibandronate sodium	-4.2728
132866-11-6	Lercanidipine hydrochloride	-4.2723
1041434-82-5	Peramivir Trihydrate	-4.2712
100427-26-7	Lercanidipine	-4.2711
705260-08-8	Vorapaxar sulfate	-4.2707
330808-88-3	Vardenafil hydrochloride trihydrate	-4.2704
111025-46-8	Pioglitazone	-4.2702
526-18-1	Osalmid	-4.2701
603-50-9	Bisacodyl	-4.27
21187-98-4	Gliclazide	-4.2699
173352-39-1	Afobazole hydrochloride	-4.2698
107868-30-4	Exemestane	-4.2695
26750-81-2	Alibendol	-4.2694
138890-62-7	Brinzolamide	-4.2693
170729-80-3	Aprepitant	-4.2686
134-03-2	Sodium ascorbate	-4.2683
6078-17-7	Berbamine dihydrochloride	-4.2683
50-81-7	L(+)-Ascorbic acid	-4.2677
29908-03-0	Ademetionine	-4.2675
57808-66-9	Domperidone	-4.2674
577-11-7	Docusate sodium	-4.2673
14882-18-9	Bismuth subsalicylat	-4.2667
69-72-7	Salicylic acid	-4.2664
32449-92-6	Dicurone	-4.266
5934-29-2	L-Histidine monohydrochloride monohydrat	-4.2659
71-00-1	histidine	-4.2659
99464-64-9	Ampiroxicam	-4.2659
91832-40-5	Cefdinir	-4.2654
1173-88-2	Oxacillin sodium	-4.2654
186826-86-8	Moxifloxacin hydrochloride	-4.2648
83905-01-5	Azithromycin	-4.2642
24868-20-0	Dantrolene sodium hemiheptahydrate	-4.2626
167465-36-3	Zosuquidar 3HCl	-4.2626
92134-98-0	Fosphenytoin sodium	-4.2626
1405-10-3	Neomycin sulfate	-4.2622

1617-90-9	Vincamine	-4.262
157810-81-6	Indinavir sulfate	-4.2618
67392-87-4	Drospirenone	-4.2614
107133-36-8	Perindopril erbumine	-4.2602
1435-55-8	Hydroquinidine	-4.2602
15622-65-8	Molindone hydrochloride	-4.2598
32986-56-4	Tobramycin	-4.2598
1156-19-0	Tolazamide	-4.2595
66357-59-3	Ranitidine Hydrochloride	-4.2589
4852-22-6	Proanthocyanidins	-4.2585
127-71-9	Sulfabenzamide	-4.2585
1257044-40-8	ABT199	-4.2583
1421373-66-1	Osimertinib mesylate	-4.2582
69056-38-8	Sapropterin Hydrochloride	-4.2578
1421373-65-0	Osimertinib	-4.257
1611493-60-7	Bictegravir	-4.2567
113-98-4	Benzylpenicillin potassium	-4.2558
89226-75-5	Manidipine dihydrochloride	-4.2558
194798-83-9	Fosfluconazole	-4.2557
968-81-0	Acetohexamide	-4.2557
89226-50-6	Manidipine	-4.2555
2447-57-6	Sulfadoxine	-4.2548
17737-65-4	Clonixin	-4.2547
69-57-8	Penicillin G sodium salt	-4.2546
51333-22-3	Budesonide	-4.2533
105628-07-7	Fasudil hydrochloride	-4.2526
29908-03-0	Ademetionine	-4.2523
23327-57-3	Nefopam hydrochloride	-4.2519
97-77-8	Disulfiram	-4.2516
1449-05-4	Glycyrrhetinic acid	-4.2516
111406-87-2	Zileuton	-4.2511
22373-78-0	Monensin sodium salt	-4.251
548-62-9	Crystal Violet	-4.2506
464-49-3	D-Camphor	-4.2501
1405-37-4	Capastat sulfate	-4.2497
461-06-3	DL-Carnitine	-4.2495
83881-52-1	Cetirizine dihydrochloride	-4.2492
10017-44-4	L-Carnitine hydrochloride	-4.2488
330808-88-3	Vardenafil hydrochloride trihydrate	-4.2477
4852-22-6	Proanthocyanidins	-4.2459

5934-29-2	L-Histidine monohydrochloride monohydrat	-4.2458
71-00-1	histidine	-4.2458
300-08-3	Arecoline HBr	-4.2449
74050-98-9	Ketanserin	-4.2448
154229-19-3	Abiraterone	-4.2447
37091-65-9	Azlocillin sodium	-4.2443
50-63-5	Chloroquine diphosphate	-4.2426
164579-32-2	Pantoprazole Sodium Hydrate	-4.2424
427-51-0	Cyproterone acetate	-4.2423
850140-73-7	Afatinib Dimaleate	-4.2413
131707-23-8	Arbidol hydrochloride	-4.2411
4852-22-6	Proanthocyanidins	-4.2409
439081-18-2	Afatinib	-4.2401
138844-81-2	Ibandronate sodium	-4.24
58-93-5	Hydrochlorothiazide	-4.2399
274901-16-5	Vildagliptin	-4.2396
19428-14-9	Benproperine phosphate	-4.2395
141-94-6	Hexetidine	-4.2391
637-32-1	Proguanil hydrochloride	-4.2389
32795-44-1	Acecainide	-4.2388
554-57-4	Methazolamide	-4.2387
136790-76-6	Lubiprostone	-4.2387
50700-72-6	Vecuronium bromide	-4.2377
65-85-0	Benzoic acid	-4.2366
96-50-4	Aminothiazole	-4.2361
71675-85-9	Amisulpride	-4.2359
111025-46-8	Pioglitazone	-4.2356
130-26-7	Clioquinol	-4.2354
115436-72-1	Risedronate Sodium	-4.2353
105462-24-6	Risedronic Acid	-4.2347
464-49-3	D-Camphor	-4.2344
120410-24-4	Biapenem	-4.2337
469-77-2	Spiculisporic Acid	-4.2334
22199-08-2	Silver sulfadiazine	-4.2334
6202-23-9	Cyclobenzaprine hydrochloride	-4.2334
464-49-3	D-Camphor	-4.2323
83881-52-1	Cetirizine dihydrochloride	-4.2317
72432-03-2	Miglitol	-4.2312
1405-10-3	Neomycin sulfate	-4.2312
504-03-0	Lupetidin	-4.231
78613-38-4	Amorolfine hydrochloride	-4.2304
41354-29-4	Cyproheptadine hydrochloride	-4.2304
83919-23-7	Mometasone furoate	-4.2302

125317-39-7	Vinorelbine Tartrate	-4.23
80474-14-2	Fluticasone propionate	-4.2295
42461-84-7	Flunixin meglumin	-4.2274
78613-38-4	Amorolfine hydrochloride	-4.2268
13614-98-7	Minocycline hydrochloride	-4.2266
306-07-0	Pargyline hydrochloride	-4.2264
555-57-7	Pargyline	-4.2264
60142-95-2	Gabapentin HCl	-4.2261
60142-96-3	Gabapentin	-4.2261
50-63-5	Chloroquine diphosphate	-4.2257
115436-72-1	Risedronate Sodium	-4.2256
4852-22-6	Proanthocyanidins	-4.2256
105462-24-6	Risedronic Acid	-4.2253
13636-18-5	Fendiline hydrochloride	-4.2253
1263-89-4	Paromomycin Sulfate	-4.225
10402-53-6	Eprazinone dihydrochloride	-4.2239
128794-94-5	Mycophenolate Mofetil	-4.2238
32795-44-1	Acecainide	-4.2229
5934-29-2	L-Histidine monohydrochloride monohydrat	-4.2227
71-00-1	histidine	-4.2227
76-25-5	Triamcinolone acetonide	-4.2224
13010-47-4	Lomustine	-4.2222
3562-84-3	Benzbromarone	-4.2216
100643-71-8	Desloratadine	-4.2215
53797-35-6	Ribostamycin sulfate	-4.2208
1108743-60-7	Entrectinib	-4.2203
80-50-2	Anisotropine Methylbromide	-4.2201
52-01-7	Abbolactone	-4.2194
177834-92-3	Eletriptan HBr	-4.2175
26171-23-3	Tolmetin	-4.2173
118072-93-8	Zoledronic Acid	-4.2169
66357-59-3	Ranitidine Hydrochloride	-4.2132
248281-84-7	Laquinimod	-4.2125
87-17-2	Salicylanilide	-4.2124
54063-53-5	Propafenone	-4.212
137975-06-5	Mozavaptan	-4.2114
125-84-8	Aminoglutethimide	-4.211
951395-08-7	tafamidis meglumine	-4.2108
1350514-68-9	Grazoprevir	-4.2105
32986-56-4	Tobramycin	-4.2105
681492-22-8	Delamanid	-4.2105

4852-22-6	Proanthocyanidins	-4.2104
53179-09-2	Sisomicin Sulfate	-4.2102
69558-55-0	Thymopentin	-4.2101
85650-56-2	Asenapine Maleate	-4.2099
37517-28-5	Amikacin	-4.2093
29908-03-0	Ademetionine	-4.209
4852-22-6	Proanthocyanidins	-4.2088
1374356-45-2	(S)-crizotinib	-4.2088
81-23-2	Dehydrocholic acid	-4.2083
137975-06-5	Mozavaptan	-4.2073
121123-17-9	Cefprozil hydrate	-4.2066
96-50-4	Aminothiazole	-4.2064
4852-22-6	Proanthocyanidins	-4.2061
6078-17-7	Berbamine dihydrochloride	-4.2056
7554-65-6	4-Methylpyrazole	-4.2049
577-11-7	Docusate sodium	-4.2042
51-15-0	Pralidoxime Chloride	-4.204
125-84-8	Aminoglutethimide	-4.2033
172152-50-0	Ilaprazole sodium	-4.2033
67-73-2	Fluocinolone acetonide	-4.203
220620-09-7	Tigecycline	-4.203
804-30-8	Fursultiamine	-4.2026
67814-76-0	Isepamicin sulfate	-4.2025
168273-06-1	Rimonabant	-4.2022
158681-13-1	Rimonabant hydrochloride	-4.2022
137975-06-5	Mozavaptan	-4.202
115939-25-8	Salvianolic acid B	-4.2019
29868-97-1	Pirenzepine dihydrochloride	-4.2017
4852-22-6	Proanthocyanidins	-4.2014
50-63-5	Chloroquine diphosphate	-4.2011
4146-30-9	Neomycin Sulphate B	-4.2011
66357-59-3	Ranitidine Hydrochloride	-4.2008
71675-85-9	Amisulpride	-4.2006
163521-08-2	Vilazodone HCl	-4.1995
4852-22-6	Proanthocyanidins	-4.1989
167933-07-5	Flibanserin	-4.1987
82419-36-1	Ofloxacin	-4.1976
1226781-44-7	Omarigliptin	-4.1967
59-30-3	Folic acid	-4.1965
59-52-9	Dithiopropanol	-4.1959
651-06-9	Sulfameter	-4.1958

56391-57-2	Netilmicin sulfate	-4.1943
695-53-4	5,5-Dimethyloxazolidine-2,4-dione	-4.1942
101477-54-7	Lomerizine hydrochloride	-4.1941
90-22-2	Valethamate bromide	-4.194
96-50-4	Aminothiazole	-4.1936
56391-57-2	Netilmicin sulfate	-4.1935
459789-99-2	Obeticholic Acid	-4.1935
71610-00-9	Cephalomannine	-4.1924
4852-22-6	Proanthocyanidins	-4.1923
111025-46-8	Pioglitazone	-4.1921
89226-50-6	Manidipine	-4.1917
147084-10-4	Alcaftadine	-4.1915
1257044-40-8	ABT199	-4.1915
68302-57-8	Amlexanox	-4.191
4852-22-6	Proanthocyanidins	-4.1906
5934-29-2	L-Histidine monohydrochloride monohydrat	-4.1904
71-00-1	histidine	-4.1904
89226-75-5	Manidipine dihydrochloride	-4.1902
138-37-4	Mafenide hydrochloride	-4.1901
129101-54-8	Rivastigmine tartrate	-4.1891
1981-58-4	Sulfamethazine sodium salt	-4.1888
50-55-5	Reserpine	-4.1885
123441-03-2	Rivastigmine	-4.1885
4852-22-6	Proanthocyanidins	-4.1884
6024-85-7	Tetrahydropalmatine hydrochloride	-4.1882
172152-50-0	Ilaprazole sodium	-4.1878
3562-84-3	Benzbromarone	-4.1873
62571-86-2	Captopril	-4.187
138890-62-7	Brinzolamide	-4.187
94-20-2	Chlorpropamide	-4.1869
57-68-1	Sulfamethazine	-4.1864
173334-58-2	Aliskiren hemifumarate	-4.1856
936539-80-9	Betrixaban maleate	-4.1849
T4690	Betrixaban hydrochloride(330942-05-7(free base))	-4.1849
15686-71-2	Cephalexin	-4.1845
95233-18-4	Atovaquone	-4.1845
21736-83-4	Spectinomycin dihydrochloride	-4.1841
330942-05-7	Betrixaban	-4.1834
67814-76-0	Isepamicin sulfate	-4.1833
66532-86-3	Propacetamol Hydrochloride	-4.1833
69056-38-8	Sapropterin Hydrochloride	-4.1829
31282-04-9	Hygromycin B	-4.1828

59-66-5	Acetazolamide	-4.1821
115436-72-1	Risedronate Sodium	-4.1821
69056-38-8	Sapropterin Hydrochloride	-4.1819
115436-72-1	Risedronate Sodium	-4.1818
475086-01-2	Selexipag	-4.1811
300-08-3	Arecoline HBr	-4.1809
6035-45-6	Folinic Acid Calcium Salt Pentahydrate	-4.1808
121123-17-9	Cefprozil hydrate	-4.1807
111902-57-9	Temocapil	-4.1807
105462-24-6	Risedronic Acid	-4.18
153559-49-0	Bexarotene	-4.18
865759-25-7	Trelagliptin	-4.1799
1029877-94-8	Trelagliptin succinate	-4.1799
483-63-6	Crotamiton	-4.1786
36322-90-4	Piroxicam	-4.1784
287714-41-4	Rosuvastatin	-4.1781
105462-24-6	Risedronic Acid	-4.1779
64-73-3	Demeclocycline hydrochloride	-4.1778
183133-96-2	Cabazitaxel	-4.1777
152-47-6	Sulfalen	-4.1769
1405-10-3	Neomycin sulfate	-4.1769
5875-06-9	Proparacaine hydrochloride	-4.1768
15622-65-8	Molindone hydrochloride	-4.1766
50-81-7	L(+)-Ascorbic acid	-4.1762
112811-59-3	Gatifloxacin	-4.1762
32986-56-4	Tobramycin	-4.176
1137608-69-5	Telotristat Etiprate	-4.176
4852-22-6	Proanthocyanidins	-4.1759
62-68-0	Proadifen hydrochloride	-4.1756
951395-08-7	tafamidis meglumine	-4.1751
6151-40-2	Quinidine hydrochloride	-4.1747
10017-44-4	L-Carnitine hydrochloride	-4.1745
23327-57-3	Nefopam hydrochloride	-4.1743
127294-70-6	Balofloxacin	-4.1741
111974-72-2	Quetiapine Fumarate	-4.1737
65666-07-1	Silymarin	-4.1734
23210-58-4	Ifenprodil Tartrate	-4.1731
4852-22-6	Proanthocyanidins	-4.173
134-03-2	Sodium ascorbate	-4.1724
5534-09-8	Beclomethasone dipropionate	-4.1719
461-06-3	DL-Carnitine	-4.1716

12650-69-0	Mupirocin	-4.1715
66-84-2	Glucosamine hydrochloride	-4.1712
33125-97-2	Etomidate	-4.1704
459789-99-2	Obeticholic Acid	-4.1689
1508-75-4	Tropicamide	-4.1686
41100-52-1	Memantine hydrochloride	-4.1683
773092-05-0	Acotiamide hydrochloride	-4.1675
469-77-2	Spiculisporic Acid	-4.1675
2753-45-9	Mebeverine hydrochloride	-4.167
68497-62-1	Pramiracetam	-4.1667
1492-18-8	Calcium folinate	-4.1663
2181-04-6	Potassium canrenoate	-4.1656
93106-60-6	Enrofloxacin	-4.1656
872728-81-9	Dabigatran Etexilate Mesylate	-4.1656
3963-95-9	Methacycline hydrochloride	-4.1655
14611-52-0	Selegiline hydrochloride	-4.1655
621-72-7	Bendazol	-4.1652
66357-59-3	Ranitidine Hydrochloride	-4.1651
330808-88-3	Vardenafil hydrochloride trihydrate	-4.1639
138890-62-7	Brinzolamide	-4.1636
330808-88-3	Vardenafil hydrochloride trihydrate	-4.1636
1365970-03-1	Glecaprevir	-4.1634
72496-41-4	Pirarubicin	-4.1626
123524-52-7	Azelnidipine	-4.1623
1981-58-4	Sulfamethazine sodium salt	-4.161
50370-12-2	Cefadroxil	-4.1609
5986-55-0	Patchouli alcohol	-4.1609
31282-04-9	Hygromycin B	-4.1608
4429-63-4	Tabersonine	-4.1607
140462-76-6	Olopatadine hydrochloride	-4.1605
637-58-1	Pramoxine hydrochloride	-4.1604
211915-06-9	Dabigatran etexilate	-4.1604
94-09-7	Benzocaine	-4.1603
29342-05-0	Ciclopirox	-4.1603
41621-49-2	Ciclopirox ethanolamine	-4.1603
521-74-4	Broxyquinoline	-4.16
5934-29-2	L-Histidine monohydrochloride monohydrat	-4.1593
71-00-1	histidine	-4.1593
170729-80-3	Aprepitant	-4.1586
1374640-70-6	Rociletinib	-4.1577
860-79-7	Cyclovirobuxin D	-4.1573

29908-03-0	Ademetionine	-4.1572
1405-37-4	Capastat sulfate	-4.1567
134-03-2	Sodium ascorbate	-4.1563
119478-55-6	Danofloxacin mesylate	-4.1556
26027-38-3	Nonoxynol	-4.1548
62571-86-2	Captopril	-4.1547
147084-10-4	Alcaftadine	-4.1535
68-35-9	Sulfadiazine	-4.1532
50-81-7	L(+)-Ascorbic acid	-4.1532
138-37-4	Mafenide hydrochloride	-4.1526
62571-86-2	Captopril	-4.1522
1338225-97-0	Doravirine	-4.1521
5986-55-0	Patchouli alcohol	-4.1503
68401-81-0	Ceftizoxime	-4.1501
54965-21-8	Albendazole	-4.1497
508233-74-7	Vortioxetine	-4.1494
132-69-4	Benzydamine hydrochloride	-4.1492
951395-08-7	tafamidis meglumine	-4.149
3963-95-9	Methacycline hydrochloride	-4.1489
69056-38-8	Sapropterin Hydrochloride	-4.1487
951395-08-7	tafamidis meglumine	-4.1487
35554-44-0	Imazalil	-4.1486
960203-27-4	Vortioxetine HBr	-4.1482
300-08-3	Arecoline HBr	-4.1478
747-36-4	Hydroxychloroquine sulfate	-4.1477
123447-62-1	Prulifloxacin	-4.1476
130-26-7	Clioquinol	-4.1475
56391-57-2	Netilmicin sulfate	-4.1473
59-67-6	Nicotinic acid	-4.1467
14611-52-0	Selegiline hydrochloride	-4.1467
53179-09-2	Sisomicin Sulfate	-4.1459
637-32-1	Proguanil hydrochloride	-4.1457
93106-60-6	Enrofloxacin	-4.1456
22254-24-6	Ipratropium Bromide	-4.1455
66985-17-9	Ipratropium bromide monohydrate	-4.1455
27833-64-3	Loxapine succinate salt	-4.1454
30299-08-2	Clinofibrate	-4.1454
122892-31-3	Itopride hydrochloride	-4.1448
79-57-2	Oxytetracycline	-4.1447
964-52-3	Moxisylyte hydrochloride	-4.1438
66357-59-3	Ranitidine Hydrochloride	-4.1433
64-73-3	Demeclocycline hydrochloride	-4.1424

97-59-6	Allantoin	-4.1422
4852-22-6	Proanthocyanidins	-4.1421
112665-43-7	Seratrodast	-4.1421
6202-23-9	Cyclobenzaprine hydrochloride	-4.1421
606-04-2	Pamabrom	-4.1419
30299-08-2	Clinofibrate	-4.1418
100643-71-8	Desloratadine	-4.1415
1405-86-3	Glycyrrhizin	-4.1411
79165-06-3	Diammonium Glycyrrhizinate	-4.1411
141-94-6	Hexetidine	-4.1411
22199-08-2	Silver sulfadiazine	-4.1395
75507-68-5	Flupirtine maleate	-4.139
964-52-3	Moxisylyte hydrochloride	-4.1388
18010-40-7	Bupivacaine hydrochloride	-4.1387
67227-57-0	Fenoldopam mesylate	-4.1385
30299-08-2	Clinofibrate	-4.1384
100643-71-8	Desloratadine	-4.1383
97-59-6	Allantoin	-4.1378
4852-22-6	Proanthocyanidins	-4.1372
204255-11-8	Oseltamivir phosphate	-4.1372
36322-90-4	Piroxicam	-4.1369
22373-78-0	Monensin sodium salt	-4.1368
363-24-6	Prostaglandin E2 (PGE2)	-4.1368
865759-25-7	Trelagliptin	-4.1364
1104-22-9	Meclizine dihydrochloride	-4.136
55661-38-6	Nimustine Hydrochloride	-4.1357
59-30-3	Folic acid	-4.1356
906093-29-6	Teneligliptin hydrobromide	-4.1356
116-43-8	Succinylsulfathiazole	-4.1354
60142-95-2	Gabapentin HCl	-4.1353
60142-96-3	Gabapentin	-4.1353
83919-23-7	Mometasone furoate	-4.1349
1029877-94-8	Trelagliptin succinate	-4.1349
65666-07-1	Silymarin	-4.1346
50-33-9	Phenylbutazone	-4.1342
154598-52-4	Efavirenz	-4.1325
60-93-5	Quinine dihydrochloride	-4.1325
130-95-0	Quinine	-4.1325
15622-65-8	Molindone hydrochloride	-4.132
30299-08-2	Clinofibrate	-4.132
872728-81-9	Dabigatran Etexilate Mesylate	-4.132
2295-58-1	Flopropione	-4.1317

100427-26-7	Lercanidipine	-4.1315
536-43-6	Dyclonine hydrochloride	-4.1313
1374356-45-2	(S)-crizotinib	-4.1311
130-26-7	Clioquinol	-4.1308
58-93-5	Hydrochlorothiazide	-4.1307
81-23-2	Dehydrocholic acid	-4.1305
56391-57-2	Netilmicin sulfate	-4.1305
66357-59-3	Ranitidine Hydrochloride	-4.1304
1156-19-0	Tolazamide	-4.1302
173903-47-4	Tizoxanide	-4.1299
4759-48-2	Isotretinoin	-4.1292
65666-07-1	Silymarin	-4.1282
65666-07-1	Silymarin	-4.1277
132866-11-6	Lercanidipine hydrochloride	-4.1276
773092-05-0	Acotiamide hydrochloride	-4.1272
521-74-4	Broxyquinoline	-4.127
477600-75-2	Tofacitinib	-4.1266
540737-29-9	Tofacitinib Citrate	-4.1266
125317-39-7	Vinorelbine Tartrate	-4.1265
286930-03-8	Fesoterodine fumarate	-4.1263
4429-63-4	Tabersonine	-4.1255
4852-22-6	Proanthocyanidins	-4.1254
5934-29-2	L-Histidine monohydrochloride monohydrat	-4.1253
71-00-1	histidine	-4.1253
4852-22-6	Proanthocyanidins	-4.1251
65391-42-6	Bestatin hydrochloride	-4.1247
58970-76-6	Ubenimex	-4.1247
651-06-9	Sulfameter	-4.1241
99464-64-9	Ampiroxicam	-4.1239
1611493-60-7	Bictegravir	-4.1229
173334-58-2	Aliskiren hemifumarate	-4.1228
543-15-7	Heptaminol hydrochloride	-4.1225
139481-59-7	Candesartan	-4.1225
747-36-4	Hydroxychloroquine sulfate	-4.1217
149676-40-4	Pefloxacin Mesylate Dihydrate	-4.1214
1435-55-8	Hydroquinidine	-4.1213
15622-65-8	Molindone hydrochloride	-4.1206
459789-99-2	Obeticholic Acid	-4.1199
951395-08-7	tafamidis meglumine	-4.1198
102518-79-6	(-)-Huperzine A	-4.1195
58066-85-6	Miltefosine	-4.1191

82419-36-1	Ofloxacin	-4.119
70458-95-6	Pefloxacin Mesylate	-4.119
152-47-6	Sulfalen	-4.1184
808118-40-3	Roxadustat	-4.1182
155148-31-5	Plerixafor 8HCl (AMD3100 8HCl)	-4.1174
110078-46-1	Plerixafor	-4.1174
300-08-3	Arecoline HBr	-4.1169
749-02-0	Spiperone	-4.1169
120410-24-4	Biapenem	-4.1168
4146-30-9	Neomycin Sulphate B	-4.1168
1132935-63-7	Dasabuvir	-4.1166
1492-18-8	Calcium folinate	-4.1163
4852-22-6	Proanthocyanidins	-4.1163
961-29-5	Isoliquiritigenin	-4.1161
100986-85-4	Levofloxacin	-4.116
138199-71-0	Levofloxacin Hemihydrate	-4.116
105462-24-6	Risedronic Acid	-4.1155
4852-22-6	Proanthocyanidins	-4.1153
138844-81-2	Ibandronate sodium	-4.1152
1254053-43-4	Gilteritinib	-4.1151
14984-68-0	Cloperastine hydrochloride	-4.1148
79660-72-3	Fleroxacin	-4.1146
961-29-5	Isoliquiritigenin	-4.1145
58-46-8	Tetrabenazine	-4.1141
26833-87-4	Homoharringtonine	-4.114
3778-73-2	Ifosfamide	-4.1132
140-87-4	Cyanoacetohydrazide	-4.1129
55-56-1	Chlorhexidine	-4.1129
389139-89-3	Omadacycline	-4.1128
10238-21-8	Glyburide	-4.1128
14639-25-9	Chromium picolinate	-4.1123
6078-17-7	Berbamine dihydrochloride	-4.112
115436-72-1	Risedronate Sodium	-4.1119
65-85-0	Benzoic acid	-4.1117
1218779-75-9	Apatinib mesylate	-4.1116
811803-05-1	Apatinib	-4.1116
554-57-4	Methazolamide	-4.1115
14484-47-0	Deflazacort	-4.1114
2753-45-9	Mebeverine hydrochloride	-4.1114
147403-03-0	Azilsartan	-4.1111

1207456-01-6	Talazoparib	-4.1109
1508-75-4	Tropicamide	-4.1107
4697-14-7	Ticarcillin disodium	-4.1103
1435-55-8	Hydroquinidine	-4.1103
951395-08-7	tafamidis meglumine	-4.1102
4852-22-6	Proanthocyanidins	-4.1102
4008-48-4	Nitroxoline	-4.1099
89-25-8	Edaravone	-4.1092
78712-43-3	Ozagrel hydrochloride	-4.1089
3681-99-0	Puerarin	-4.1087
1421373-66-1	Osimertinib mesylate	-4.1084
23964-57-0	Articaine HCl	-4.1078
155148-31-5	Plerixafor 8HCl (AMD3100 8HCl)	-4.1076
110078-46-1	Plerixafor	-4.1076
1421373-65-0	Osimertinib	-4.1072
60-87-7	Promethazine	-4.1071
155148-31-5	Plerixafor 8HCl (AMD3100 8HCl)	-4.1063
110078-46-1	Plerixafor	-4.1063
131707-23-8	Arbidol hydrochloride	-4.1062
102518-79-6	(-)-Huperzine A	-4.106
26833-87-4	Homoharringtonine	-4.1056
864750-70-9	Revefenacin	-4.105
473-98-3	Betulin	-4.1048
839712-12-8	Cariprazine	-4.1044
98769-84-7	Reboxetine mesylate	-4.1042
4852-22-6	Proanthocyanidins	-4.1042
82571-53-7	Ozagrel	-4.1041
115436-72-1	Risedronate Sodium	-4.1041
130693-82-2	Dorzolamide hydrochloride	-4.104
10402-53-6	Eprazinone dihydrochloride	-4.1036
68401-81-0	Ceftizoxime	-4.1036
152459-95-5	Imatinib	-4.1025
220127-57-1	Imatinib Mesylate	-4.1025
119302-91-9	Rocuronium bromide	-4.1021
113852-37-2	Cidofovir	-4.1015
50-63-5	Chloroquine diphosphate	-4.1013
3679-64-9	Salifungin	-4.1013
98769-84-7	Reboxetine mesylate	-4.0999
1108743-60-7	Entrectinib	-4.0999
105462-24-6	Risedronic Acid	-4.0993

1501-84-4	Rimantadine hydrochloride	-4.0993
1360460-82-7	Anlotinib Dihydrochloride	-4.0991
1058156-90-3	Anlotinib	-4.0991
21187-98-4	Gliclazide	-4.0991
138844-81-2	Ibandronate sodium	-4.0988
170729-80-3	Aprepitant	-4.0986
1405-37-4	Capastat sulfate	-4.0972
1405-37-4	Capastat sulfate	-4.0964
302-22-7	Chlormadinone acetate	-4.0963
105462-24-6	Risedronic Acid	-4.0962
7681-93-8	Pimaricin	-4.0958
469-77-2	Spiculisporic Acid	-4.0954
66532-86-3	Propacetamol Hydrochloride	-4.095
168555-66-6	Fosbretabulin Disodium	-4.0949
1374356-45-2	(S)-crizotinib	-4.0943
38194-50-2	Sulindac	-4.094
1108743-60-7	Entrectinib	-4.0939
6398-98-7	Amodiaquine hydrochloride	-4.0939
53179-09-2	Sisomicin Sulfate	-4.0931
115436-72-1	Risedronate Sodium	-4.0923
6469-93-8	Chlorprothixene hydrochloride	-4.092
113-59-7	Chlorprothixene	-4.092
548-62-9	Crystal Violet	-4.091
2430-27-5	Valpramide	-4.0908
183204-72-0	Tipiracil hydrochloride	-4.0907
5875-06-9	Proparacaine hydrochloride	-4.0905
6160-12-9	(-)-Sparteine sulfate pentahydrate	-4.0903
4852-22-6	Proanthocyanidins	-4.0896
80-35-3	Sulfamethoxypyridazine	-4.0893
89-25-8	Edaravone	-4.0887
434-07-1	Oxymetholone	-4.0878
651-06-9	Sulfameter	-4.0874
23210-58-4	Ifenprodil Tartrate	-4.0864
19428-14-9	Benproperine phosphate	-4.0856
79350-37-1	Cefixime	-4.0854
34031-32-8	Auranofin	-4.0854
434-07-1	Oxymetholone	-4.0844
2430-27-5	Valpramide	-4.0841
808118-40-3	Roxadustat	-4.084
111025-46-8	Pioglitazone	-4.0836

108612-45-9	Mizolastine	-4.0835
1435-55-8	Hydroquinidine	-4.0831
808118-40-3	Roxadustat	-4.0829
60-93-5	Quinine dihydrochloride	-4.0824
130-95-0	Quinine	-4.0824
2259-96-3	Cyclothiazide	-4.0823
57-96-5	Sulfinpyrazone	-4.0821
102518-79-6	(-)-Huperzine A	-4.082
31282-04-9	Hygromycin B	-4.0815
75706-12-6	Leflunomide	-4.0812
51773-92-3	Mefloquine hydrochloride	-4.0806
172152-50-0	Ilaprazole sodium	-4.08
1254053-43-4	Gilteritinib	-4.08
24868-20-0	Dantrolene sodium hemiheptahydrate	-4.0799
34580-14-8	Ketotifen fumarate	-4.0797
147-85-3	proline	-4.0797
66852-54-8	Halobetasol Propionate	-4.0796
3963-95-9	Methacycline hydrochloride	-4.0793
82419-36-1	Ofloxacin	-4.079
137975-06-5	Mozavaptan	-4.0787
126-27-2	Oxethazaine	-4.0787
26750-81-2	Alibendol	-4.0786
521-74-4	Broxyquinoline	-4.0774
537-73-5	Isoferulic acid	-4.0769
53882-12-5	Lodoxamide	-4.0769
141-94-6	Hexetidine	-4.0768
79307-93-0	Azelastine hydrochloride	-4.0768
89778-27-8	Toremifene citrate	-4.0766
89778-26-7	Toremifene	-4.0763
153504-70-2	Cevimeline hydrochloride hemihydrate	-4.0761
67227-57-0	Fenoldopam mesylate	-4.0758
123171-59-5	Cefepime Dihydrochloride Monohydrate	-4.0746
85650-56-2	Asenapine Maleate	-4.0745
3160-91-6	Moroxydine hydrochloride	-4.0743
6078-17-7	Berbamine dihydrochloride	-4.0732
162359-56-0	Fingolimod hydrochloride	-4.0731
1405-37-4	Capastat sulfate	-4.073
79660-72-3	Fleroxacin	-4.0726
17737-65-4	Clonixin	-4.0722
1672-63-5	4-Hydroxyantipyrine	-4.0721
6151-40-2	Quinidine hydrochloride	-4.072
477600-75-2	Tofacitinib	-4.0718

540737-29-9	Tofacitinib Citrate	-4.0718
53882-12-5	Lodoxamide	-4.0717
872728-81-9	Dabigatran Etexilate Mesylate	-4.0717
13614-98-7	Minocycline hydrochloride	-4.0714
155148-31-5	Plerixafor 8HCl (AMD3100 8HCl)	-4.0711
110078-46-1	Plerixafor	-4.0711
65666-07-1	Silymarin	-4.0709
50-63-5	Chloroquine diphosphate	-4.0704
440-17-5	Trifluoperazine dihydrochloride	-4.07
163451-81-8	Teriflunomide	-4.0695
108612-45-9	Mizolastine	-4.0695
108605-62-5	Teriflunomide	-4.0689
439081-18-2	Afatinib	-4.0689
57-41-0	Phenytoin	-4.0688
138844-81-2	Ibandronate sodium	-4.068
89778-27-8	Toremifene citrate	-4.068
54063-53-5	Propafenone	-4.068
55661-38-6	Nimustine Hydrochloride	-4.0677
154-93-8	Carmustine	-4.0674
89778-26-7	Toremifene	-4.0671
1642-54-2	Diethylcarbamazine citrate	-4.0668
59721-29-8	Camostat mesilate	-4.0667
116649-85-5	Ramatroban	-4.066
2181-04-6	Potassium canrenoate	-4.0659
2430-27-5	Valpramide	-4.0659
2447-57-6	Sulfadoxine	-4.0658
65391-42-6	Bestatin hydrochloride	-4.0656
58970-76-6	Ubenimex	-4.0656
26328-04-1	Cinepazide maleate	-4.0645
62-68-0	Proadifen hydrochloride	-4.0639
115939-25-8	Salvianolic acid B	-4.0634
41575-94-4	Carboplatin	-4.0634
635702-64-6	Pazopanib HCl	-4.0634
17321-77-6	Clomipramine hydrochloride	-4.0622
363-24-6	Prostaglandin E2 (PGE2)	-4.0619
6398-98-7	Amodiaquine hydrochloride	-4.0616
1435-55-8	Hydroquinidine	-4.061
7083-71-8	(+)-Emetine dihydrochloride hydrate	-4.0608
863031-21-4	Azilsartan Medoxomil	-4.0606
444731-52-6	Pazopanib	-4.0601
125-02-0	Prednisolone phosphate sodium	-4.0601
95635-55-5	Ranolazine	-4.06
1405-37-4	Capastat sulfate	-4.0595

84625-61-6	Itraconazole	-4.0593
3681-99-0	Puerarin	-4.0593
1617-90-9	Vincamine	-4.0591
128-13-2	Ursodeoxycholic acid	-4.0589
80-35-3	Sulfamethoxypyridazine	-4.0589
745-65-3	Alprostadil	-4.0586
56391-57-2	Netilmicin sulfate	-4.0584
951395-08-7	tafamidis meglumine	-4.0578
122320-73-4	Rosiglitazone	-4.0571
95635-56-6	Ranolazine dihydrochloride	-4.057
89226-50-6	Manidipine	-4.0561
10238-21-8	Glyburide	-4.056
850140-73-7	Afatinib Dimaleate	-4.0557
149647-78-9	Vorinostat	-4.0556
6398-98-7	Amodiaquine hydrochloride	-4.0554
115436-72-1	Risedronate Sodium	-4.055
60-87-7	Promethazine	-4.055
537-12-2	Diperodon hydrochloride	-4.055
51-48-9	L-Thyroxine	-4.0543
526-18-1	Osalmid	-4.054
138402-11-6	Irbesartan	-4.0536
6024-85-7	Tetrahydropalmatine hydrochloride	-4.0534
118072-93-8	Zoledronic Acid	-4.0531
41100-52-1	Memantine hydrochloride	-4.0529
151-67-7	Anestan	-4.0529
134308-13-7	Tolcapone	-4.052
4852-22-6	Proanthocyanidins	-4.0516
804-30-8	Fursultiamine	-4.0514
755038-65-4	Volasertib	-4.0513
105462-24-6	Risedronic Acid	-4.0508
95233-18-4	Atovaquone	-4.0508
27591-69-1	Tilorone dihydrochloride	-4.0502
132-69-4	Benzydamine hydrochloride	-4.0493
154-69-8	Tripelennamine hydrochloride	-4.0489
681492-22-8	Delamanid	-4.0486
486460-32-6	Sitagliptin	-4.0482
491833-29-5	Eliglustat	-4.0472
105628-07-7	Fasudil hydrochloride	-4.047
89226-75-5	Manidipine dihydrochloride	-4.0468
56-89-3	cystine	-4.0466
23210-58-4	Ifenprodil Tartrate	-4.0466
1508-75-4	Tropicamide	-4.0465
69056-38-8	Sapropterin Hydrochloride	-4.0464

1104-22-9	Meclizine dihydrochloride	-4.0462
51333-22-3	Budesonide	-4.0459
24868-20-0	Dantrolene sodium hemiheptahydrate	-4.0453
194798-83-9	Fosfluconazole	-4.0448
4852-22-6	Proanthocyanidins	-4.0444
4852-22-6	Proanthocyanidins	-4.0436
98769-84-7	Reboxetine mesylate	-4.0434
951395-08-7	tafamidis meglumine	-4.0431
4852-22-6	Proanthocyanidins	-4.043
637-58-1	Pramoxine hydrochloride	-4.0426
10402-53-6	Eprazinone dihydrochloride	-4.0421
440-17-5	Trifluoperazine dihydrochloride	-4.0415
706782-28-7	Pimavanserin tartrate	-4.0403
706779-91-1	Pimavanserin	-4.0403
543-15-7	Heptaminol hydrochloride	-4.0402
3978-86-7	Azatadine Maleate	-4.0396
75706-12-6	Leflunomide	-4.0396
91374-20-8	Ropinirole hydrochloride	-4.039
219861-08-2	Escitalopram Oxalate	-4.0389
59729-32-7	Citalopram HBr	-4.0389
123171-59-5	Cefepime Dihydrochloride Monohydrate	-4.0384
69056-38-8	Sapropterin Hydrochloride	-4.0384
2259-96-3	Cyclothiazide	-4.0384
434-07-1	Oxymetholone	-4.0383
125-84-8	Aminoglutethimide	-4.037
577-11-7	Docusate sodium	-4.0368
53-60-1	Promazine hydrochloride	-4.0367
155148-31-5	Plerixafor 8HCl (AMD3100 8HCl)	-4.0363
110078-46-1	Plerixafor	-4.0363
127-65-1	Chloramine-T	-4.0362
65666-07-1	Silymarin	-4.0362
115256-11-6	Dofetilide	-4.0358
6170-42-9	Chloropyramine hydrochloride	-4.0351
162359-56-0	Fingolimod hydrochloride	-4.035
1405-41-0	Gentamycin sulfate	-4.0347
54063-53-5	Propafenone	-4.0346
1405-37-4	Capastat sulfate	-4.0345
287714-41-4	Rosuvastatin	-4.0341
56391-57-2	Netilmicin sulfate	-4.0329
59-52-9	Dithiopropanol	-4.0327
56-92-8	Histamine 2HCl	-4.0327
51-45-6	Histamine	-4.0327
57248-88-1	Pamidronate disodium salt	-4.0317

97-59-6	Allantoin	-4.0311
79307-93-0	Azelastine hydrochloride	-4.031
87333-19-5	Ramipril	-4.0308
51-48-9	L-Thyroxine	-4.0305
4852-22-6	Proanthocyanidins	-4.0305
173903-47-4	Tizoxanide	-4.0301
119793-66-7	Propionyl-L-carnitine hydrochloride	-4.0301
68302-57-8	Amlexanox	-4.0298
65666-07-1	Silymarin	-4.0296
4852-22-6	Proanthocyanidins	-4.0295
434-07-1	Oxymetholone	-4.0288
32986-56-4	Tobramycin	-4.0285
3778-73-2	Ifosfamide	-4.0277
1421373-65-0	Osimertinib	-4.0276
53179-09-2	Sisomicin Sulfate	-4.0275
1642-54-2	Diethylcarbamazine citrate	-4.0273
65666-07-1	Silymarin	-4.0271
543-15-7	Heptaminol hydrochloride	-4.0266
72496-41-4	Pirarubicin	-4.0263
914462-92-3	Rolapitant hydrochloride	-4.026
3963-95-9	Methacycline hydrochloride	-4.0253
81-25-4	Cholic Acid	-4.025
65666-07-1	Silymarin	-4.0246
6078-17-7	Berbamine dihydrochloride	-4.0236
1617-90-9	Vincamine	-4.0234
13614-98-7	Minocycline hydrochloride	-4.0233
4852-22-6	Proanthocyanidins	-4.0232
107724-20-9	Eplerenone	-4.0228
101477-54-7	Lomerizine hydrochloride	-4.0228
469-77-2	Spiculisporic Acid	-4.0227
70775-75-6	Octenidine Dihydrochloride	-4.0224
66532-86-3	Propacetamol Hydrochloride	-4.0223
543-15-7	Heptaminol hydrochloride	-4.0221
122320-73-4	Rosiglitazone	-4.0221
35554-44-0	Imazalil	-4.0215
1421373-66-1	Osimertinib mesylate	-4.021
871038-72-1	Raltegravir potassium	-4.0205
68099-86-5	Bepridil hydrochloride	-4.0202
79660-72-3	Fleroxacin	-4.0196
51333-22-3	Budesonide	-4.0195
123948-87-8	Topotecan	-4.0194

132-69-4	Benzydamine hydrochloride	-4.0194
107753-78-6	Zafirlukast	-4.0194
83881-52-1	Cetirizine dihydrochloride	-4.0194
130018-77-8	Levocetirizine	-4.0191
125-84-8	Aminoglutethimide	-4.0191
77-36-1	Chlortalidone	-4.0188
152459-95-5	Imatinib	-4.0187
220127-57-1	Imatinib Mesylate	-4.0187
31282-04-9	Hygromycin B	-4.0185
65666-07-1	Silymarin	-4.0181
68-35-9	Sulfadiazine	-4.0179
168555-66-6	Fosbretabulin Disodium	-4.0164
152-47-6	Sulfalen	-4.0161
125317-39-7	Vinorelbine Tartrate	-4.0159
4852-22-6	Proanthocyanidins	-4.0156
1338225-97-0	Doravirine	-4.0155
1405-41-0	Gentamycin sulfate	-4.0154
147-85-3	proline	-4.0151
70-00-8	Trifluridine	-4.015
55203-24-2	Dexamethasone Sodium Phosphate	-4.0136
26328-04-1	Cinepazide maleate	-4.0127
4852-22-6	Proanthocyanidins	-4.0126
119793-66-7	Propionyl-L-carnitine hydrochloride	-4.0123
123447-62-1	Prulifloxacin	-4.0123
1405-37-4	Capastat sulfate	-4.0121
59-05-2	Methotrexate	-4.0119
1981-58-4	Sulfamethazine sodium salt	-4.0115
56391-57-2	Netilmicin sulfate	-4.0114
149676-40-4	Pefloxacin Mesylate Dihydrate	-4.0113
737789-87-6	Relugolix	-4.0112
25389-94-0	Kanamycin sulfate	-4.0111
70458-95-6	Pefloxacin Mesylate	-4.011
154-69-8	Tripelennamine hydrochloride	-4.0108
638-94-8	Desonide	-4.0106
1492-18-8	Calcium folinate	-4.0105
14611-52-0	Selegiline hydrochloride	-4.0102
155148-31-5	Plerixafor 8HCl (AMD3100 8HCl)	-4.0098
110078-46-1	Plerixafor	-4.0098
34031-32-8	Auranofin	-4.0097
4852-22-6	Proanthocyanidins	-4.0097
2753-45-9	Mebeverine hydrochloride	-4.0095
1508-75-4	Tropicamide	-4.0095

21187-98-4	Gliclazide	-4.0086
111902-57-9	Temocapil	-4.0081
6108-05-0	Lidocaine Hydrochloride hydrate	-4.0074
73-78-9	Lidocaine hydrochloride	-4.0074
1226781-44-7	Omarigliptin	-4.0069
150322-43-3	Prasugrel	-4.0067
137-58-6	Xylocaine	-4.0062
53-60-1	Promazine hydrochloride	-4.0061
10402-53-6	Eprazinone dihydrochloride	-4.006
300-08-3	Arecoline HBr	-4.0056
141-94-6	Hexetidine	-4.0053
74150-27-9	Pimobendan	-4.0051
55079-83-9	Acitretin	-4.0046
149022-22-0	Amikacin Sulfate Salt	-4.0042
951395-08-7	tafamidis meglumine	-4.0039
52-66-4	DL-Penicillamine	-4.0035
138844-81-2	Ibandronate sodium	-4.0031
434-07-1	Oxymetholone	-4.0028
951395-08-7	tafamidis meglumine	-4.0026
389139-89-3	Omadacycline	-4.0024
23828-92-4	Ambroxol hydrochloride	-4.0018
59-67-6	Nicotinic acid	-4.0018
906093-29-6	Teneligliptin hydrobromide	-4.0016
72-19-5	threonine	-4.0013
53797-35-6	Ribostamycin sulfate	-4.001
10402-53-6	Eprazinone dihydrochloride	-4.0008
96847-55-1	Levomilnacipran	-4.0006
116-43-8	Succinylsulfathiazole	-3.9994
951395-08-7	tafamidis meglumine	-3.9993
122647-32-9	Ibutilide Fumarate	-3.999
78613-38-4	Amorolfine hydrochloride	-3.9988
35554-44-0	Imazalil	-3.9986
18683-91-5	Ambroxol	-3.9985
894-71-3	Nortriptyline hydrochloride	-3.9984
101831-37-2	Diclazuril	-3.9982
108612-45-9	Mizolastine	-3.9981
951395-08-7	tafamidis meglumine	-3.998
1197953-54-0	Brigatinib	-3.9978
2192-20-3	Hydroxyzine dihydrochloride	-3.9966
68099-86-5	Bepridil hydrochloride	-3.9963
10246-75-0	Hydroxyzine Pamoate	-3.9963

82419-36-1	Ofloxacin	-3.9959
100986-85-4	Levofloxacin	-3.9953
138199-71-0	Levofloxacin Hemihydrate	-3.9953
70-47-3	L-Asparagine	-3.995
300-08-3	Arecoline HBr	-3.9946
1421373-66-1	Osimertinib mesylate	-3.9939
84625-61-6	Itraconazole	-3.9936
4852-22-6	Proanthocyanidins	-3.9927
82419-36-1	Ofloxacin	-3.992
65666-07-1	Silymarin	-3.9917
1421373-65-0	Osimertinib	-3.9915
951395-08-7	tafamidis meglumine	-3.9914
630-93-3	Phenytoin sodium	-3.9914
1156-19-0	Tolazamide	-3.9905
1254053-43-4	Gilteritinib	-3.9905
101831-37-2	Diclazuril	-3.9904
79307-93-0	Azelastine hydrochloride	-3.9904
865759-25-7	Trelagliptin	-3.9904
1218779-75-9	Apatinib mesylate	-3.9894
811803-05-1	Apatinib	-3.9894
113852-37-2	Cidofovir	-3.9886
1229194-11-9	Edoxaban Tosylate Monohydrate	-3.9886
521-74-4	Broxyquinoline	-3.9884
70-47-3	L-Asparagine	-3.9883
69056-38-8	Sapropterin Hydrochloride	-3.9878
1029877-94-8	Trelagliptin succinate	-3.9871
4852-22-6	Proanthocyanidins	-3.9867
864750-70-9	Revefenacin	-3.9862
951395-08-7	tafamidis meglumine	-3.986
26750-81-2	Alibendol	-3.986
56391-57-2	Netilmicin sulfate	-3.9859
13614-98-7	Minocycline hydrochloride	-3.9856
55-56-1	Chlorhexidine	-3.9856
543-15-7	Heptaminol hydrochloride	-3.9853
68-35-9	Sulfadiazine	-3.9853
3778-73-2	Ifosfamide	-3.9851
147-85-3	proline	-3.9834
12650-69-0	Mupirocin	-3.9831

1405-10-3	Neomycin sulfate	-3.9827
171599-83-0	Sildenafil citrate	-3.9826
69056-38-8	Sapropterin Hydrochloride	-3.9823
76824-35-6	Famotidine	-3.9814
70-47-3	L-Asparagine	-3.981
51333-22-3	Budesonide	-3.9801
1405-37-4	Capastat sulfate	-3.9799
65666-07-1	Silymarin	-3.9798
50-65-7	Niclosamide	-3.9797
22457-89-2	Benfotiamine	-3.978
59-67-6	Nicotinic acid	-3.9769
13189-98-5	Fudosteine	-3.9767
14639-25-9	Chromium picolinate	-3.9766
2259-96-3	Cyclothiazide	-3.9762
105826-92-4	Tropisetron hydrochloride	-3.9759
951395-08-7	tafamidis meglumine	-3.9757
7681-93-8	Pimaricin	-3.9753
108612-45-9	Mizolastine	-3.975
76824-35-6	Famotidine	-3.9749
10402-53-6	Eprazinone dihydrochloride	-3.9747
99-15-0	Acetylleucine	-3.9746
52-67-5	d-penicillamin	-3.9737
130018-77-8	Levocetirizine	-3.9734
25389-94-0	Kanamycin sulfate	-3.9732
52-66-4	DL-Penicillamine	-3.9729
4852-22-6	Proanthocyanidins	-3.9725
83881-52-1	Cetirizine dihydrochloride	-3.9725
23964-57-0	Articaine HCl	-3.9714
56391-57-2	Netilmicin sulfate	-3.9711
537-12-2	Diperodon hydrochloride	-3.9696
144-82-1	Sulfamethizole	-3.9688
4146-30-9	Neomycin Sulphate B	-3.9676
140-87-4	Cyanoacetohydrazide	-3.9674
3778-73-2	Ifosfamide	-3.9674
127-65-1	Chloramine-T	-3.9671
118072-93-8	Zoledronic Acid	-3.9668
1256580-46-7	Alectinib	-3.9665
14484-47-0	Deflazacort	-3.9665
4852-22-6	Proanthocyanidins	-3.9661
1098-60-8	Triflupromazine hydrochloride	-3.9661
2259-96-3	Cyclothiazide	-3.9654

1611493-60-7	Bictegravir	-3.9654
119793-66-7	Propionyl-L-carnitine hydrochloride	-3.9647
4852-22-6	Proanthocyanidins	-3.9643
4852-22-6	Proanthocyanidins	-3.9637
58-46-8	Tetrabenazine	-3.9635
4852-22-6	Proanthocyanidins	-3.9626
10402-53-6	Eprazinone dihydrochloride	-3.9621
65-45-2	Salicylamide	-3.9613
577-11-7	Docusate sodium	-3.9613
1365970-03-1	Glecaprevir	-3.9612
97-59-6	Allantoin	-3.961
3759-92-0	Furaltadone hydrochloride	-3.9609
154229-18-2	Abiraterone Acetate	-3.9606
1263-89-4	Paromomycin Sulfate	-3.9604
738606-46-7	ETC1002	-3.9596
53179-09-2	Sisomicin Sulfate	-3.9583
138890-62-7	Brinzolamide	-3.9583
22252-43-3	Cefalexine EP IMpurity B	-3.9572
182431-12-5	Lomitapide	-3.9568
1254053-43-4	Gilteritinib	-3.9567
4852-22-6	Proanthocyanidins	-3.9566
4852-22-6	Proanthocyanidins	-3.9564
97-59-6	Allantoin	-3.9564
122320-73-4	Rosiglitazone	-3.9557
1449-05-4	Glycyrrhetinic acid	-3.9554
59804-37-4	Tenoxicam	-3.955
4852-22-6	Proanthocyanidins	-3.9548
194798-83-9	Fosfluconazole	-3.9544
4852-22-6	Proanthocyanidins	-3.9544
4852-22-6	Proanthocyanidins	-3.9542
154-93-8	Carmustine	-3.9541
57-37-4	Benactyzine hydrochloride	-3.9539
82640-04-8	Raloxifene hydrochloride	-3.953
6035-45-6	Folinic Acid Calcium Salt Pentahydrate	-3.9526
124937-52-6	Tolterodine tartrate	-3.9526
3978-86-7	Azatadine Maleate	-3.9524
171599-83-0	Sildenafil citrate	-3.9513
167933-07-5	Flibanserin	-3.9511
60-93-5	Quinine dihydrochloride	-3.9505
130-95-0	Quinine	-3.9505

178429-62-4	solriamfetol	-3.9493
1435-55-8	Hydroquinidine	-3.9493
141-94-6	Hexetidine	-3.9491
4852-22-6	Proanthocyanidins	-3.9491
111025-46-8	Pioglitazone	-3.949
6202-23-9	Cyclobenzaprine hydrochloride	-3.9483
7421-40-1	Carbenoxolone disodium	-3.9479
167933-07-5	Flibanserin	-3.9479
66852-54-8	Halobetasol Propionate	-3.9478
151-67-7	Anestan	-3.9475
72496-41-4	Pirarubicin	-3.9474
65391-42-6	Bestatin hydrochloride	-3.947
58970-76-6	Ubenimex	-3.947
10402-53-6	Eprazinone dihydrochloride	-3.9469
1403254-99-8	EPZ6438	-3.9461
130018-77-8	Levocetirizine	-3.946
83881-52-1	Cetirizine dihydrochloride	-3.946
85409-38-7	Rilmenidine Phosphate	-3.9459
55661-38-6	Nimustine Hydrochloride	-3.9456
130693-82-2	Dorzolamide hydrochloride	-3.9448
72-18-4	L-Valine	-3.9446
469-77-2	Spiculisporic Acid	-3.9442
6055-19-2	Cyclophosphamide monohydrate	-3.9432
58-93-5	Hydrochlorothiazide	-3.9429
95233-18-4	Atovaquone	-3.9429
74711-43-6	Zaltoprofen	-3.9425
163680-77-1	Pazufloxacin Mesylate	-3.9412
140-40-9	Nithiamide	-3.9408
30299-08-2	Clinofibrate	-3.9403
804-30-8	Fursultiamine	-3.9396
88495-63-0	Artesunate	-3.9395
77-36-1	Chlortalidone	-3.9394
541-15-1	L(-)-Carnitine	-3.9391
461-06-3	DL-Carnitine	-3.9391
97-59-6	Allantoin	-3.9388
57-68-1	Sulfamethazine	-3.938
145-41-5	Sodium dehydrocholate	-3.938
951395-08-7	tafamidis meglumine	-3.938
30299-08-2	Clinofibrate	-3.9372
19428-14-9	Benproperine phosphate	-3.9365
14611-52-0	Selegiline hydrochloride	-3.9357
906093-29-6	Teneligliptin hydrobromide	-3.9344

105826-92-4	Tropisetron hydrochloride	-3.9336
154229-19-3	Abiraterone	-3.9334
1104-22-9	Meclizine dihydrochloride	-3.9327
21187-98-4	Gliclazide	-3.9323
60-87-7	Promethazine	-3.9318
864750-70-9	Revefenacin	-3.9308
923604-59-5	Simeprevir	-3.9307
31282-04-9	Hygromycin B	-3.9295
10402-53-6	Eprazinone dihydrochloride	-3.9294
59804-37-4	Tenoxicam	-3.9279
56-89-3	cystine	-3.9278
59-52-9	Dithiopropanol	-3.9267
167465-36-3	Zosuquidar 3HCl	-3.9266
32854-75-4	Lappaconitine	-3.9263
173352-39-1	Afobazole hydrochloride	-3.9259
84625-61-6	Itraconazole	-3.9254
839712-12-8	Cariprazine	-3.9253
10540-29-1	Tamoxifen	-3.9247
54965-24-1	Tamoxifen Z-isomer citrate	-3.9247
3717-88-2	Flavoxate hydrochloride	-3.9245
102-76-1	Triacetin	-3.9243
181183-52-8	Almotriptan Malate	-3.9243
95233-18-4	Atovaquone	-3.9243
6024-85-7	Tetrahydropalmatine hydrochloride	-3.9227
1405-10-3	Neomycin sulfate	-3.9226
554-57-4	Methazolamide	-3.9222
55-56-1	Chlorhexidine	-3.9218
54965-21-8	Albendazole	-3.9217
79-55-0	Pempidine	-3.9212
15622-65-8	Molindone hydrochloride	-3.9206
6151-40-2	Quinidine hydrochloride	-3.9204
4852-22-6	Proanthocyanidins	-3.9203
T5164	Cabozantinib hydrochloride (849217-68-1(free base))	-3.9201
849217-68-1	Cabozantinib	-3.9201
1140909-48-3	Cabozantinib Malate	-3.9201
4008-48-4	Nitroxoline	-3.919
23964-57-0	Articaine HCl	-3.9186
50-78-2	Aspirin	-3.9181
1229-29-4	Doxepin hydrochloride	-3.9179
78613-38-4	Amorolfine hydrochloride	-3.9175
26328-04-1	Cinepazide maleate	-3.917
872728-81-9	Dabigatran Etexilate Mesylate	-3.917

4852-22-6	Proanthocyanidins	-3.9167
155148-31-5	Plerixafor 8HCl (AMD3100 8HCl)	-3.9159
110078-46-1	Plerixafor	-3.9159
93106-60-6	Enrofloxacin	-3.9156
4852-22-6	Proanthocyanidins	-3.9152
6506-37-2	Nimorazole	-3.9145
26833-87-4	Homoharringtonine	-3.9143
302-95-4	Sodium Desoxycholate	-3.9134
130693-82-2	Dorzolamide hydrochloride	-3.9132
85409-38-7	Rilmenidine Phosphate	-3.913
147403-03-0	Azilsartan	-3.9126
151-67-7	Anestan	-3.9125
34580-14-8	Ketotifen fumarate	-3.9125
4852-22-6	Proanthocyanidins	-3.9121
443913-73-3	Vandetanib	-3.9121
961-29-5	Isoliquiritigenin	-3.912
72432-03-2	Miglitol	-3.9119
84057-95-4	Ropivacaine	-3.9111
98717-15-8	Ropivacaine hydrochloride	-3.9111
1254053-43-4	Gilteritinib	-3.9109
127373-66-4	Sivelestat	-3.9107
1230487-00-9	Siponimod	-3.9105
65666-07-1	Silymarin	-3.9102
128-13-2	Ursodeoxycholic acid	-3.9102
83-44-3	Deoxycholic acid	-3.9095
41575-94-4	Carboplatin	-3.9084
19428-14-9	Benproperine phosphate	-3.9069
30299-08-2	Clinofibrate	-3.9068
122647-32-9	Ibutilide Fumarate	-3.9068
155148-31-5	Plerixafor 8HCl (AMD3100 8HCl)	-3.9055
110078-46-1	Plerixafor	-3.9055
300-08-3	Arecoline HBr	-3.9054
138844-81-2	Ibandronate sodium	-3.9047
53179-09-2	Sisomicin Sulfate	-3.9047
306-07-0	Pargyline hydrochloride	-3.9042
555-57-7	Pargyline	-3.9042
22373-78-0	Monensin sodium salt	-3.904
65666-07-1	Silymarin	-3.9013
59-52-9	Dithiopropanol	-3.9012
51773-92-3	Mefloquine hydrochloride	-3.9009
13614-98-7	Minocycline hydrochloride	-3.9006

67-43-6	Pentetic Acid	-3.9002
120410-24-4	Biapenem	-3.9
173903-47-4	Tizoxanide	-3.8993
55203-24-2	Dexamethasone Sodium Phosphate	-3.8992
32986-56-4	Tobramycin	-3.8989
167465-36-3	Zosuquidar 3HCl	-3.8984
434-07-1	Oxymetholone	-3.8977
4852-22-6	Proanthocyanidins	-3.8977
141505-33-1	Levosimendan	-3.8976
434-07-1	Oxymetholone	-3.8975
116-43-8	Succinylsulfathiazole	-3.8967
61718-82-9	Fluvoxamine maleate	-3.8963
53179-09-2	Sisomicin Sulfate	-3.8962
13114-29-9	Sodium Demethylcantharidate	-3.8953
4852-22-6	Proanthocyanidins	-3.8951
1221573-85-8	Paritaprevir	-3.895
95-25-0	Chlorzoxazone	-3.8943
57-96-5	Sulfinpyrazone	-3.8938
147084-10-4	Alcaftadine	-3.8934
56391-57-2	Netilmicin sulfate	-3.8928
155148-31-5	Plerixafor 8HCl (AMD3100 8HCl)	-3.8926
110078-46-1	Plerixafor	-3.8926
3778-73-2	Ifosfamide	-3.8924
120410-24-4	Biapenem	-3.8919
57-96-5	Sulfinpyrazone	-3.8918
81-23-2	Dehydrocholic acid	-3.8908
127-79-7	Sulfamerazine	-3.8906
182431-12-5	Lomitapide	-3.8905
72-18-4	L-Valine	-3.8892
51940-44-4	Pipemidic acid	-3.8886
14484-47-0	Deflazacort	-3.8879
1405-37-4	Capastat sulfate	-3.8877
1508-75-4	Tropicamide	-3.8871
145-41-5	Sodium dehydrocholate	-3.887
84371-65-3	Mifepristone	-3.8864
27262-48-2	Levobupivacaine HCl	-3.886
27262-47-1	Levobupivacaine	-3.886
18010-40-7	Bupivacaine hydrochloride	-3.886
4852-22-6	Proanthocyanidins	-3.8851
17896-21-8	Citiolone	-3.885
1260141-27-2	Vonoprazan fumarate	-3.8842

54063-53-5	Propafenone	-3.8837
1446321-46-5	voxelotor	-3.8832
64-72-2	Chlortetracycline hydrochloride	-3.8822
65666-07-1	Silymarin	-3.8817
259793-96-9	Favipiravir	-3.8815
147403-03-0	Azilsartan	-3.8813
56391-57-2	Netilmicin sulfate	-3.8802
77-36-1	Chlortalidone	-3.8802
860-79-7	Cyclovirobuxin D	-3.8799
120638-55-3	Bromfenac sodium	-3.8797
65666-07-1	Silymarin	-3.8793
69558-55-0	Thymopentin	-3.8793
503612-47-3	Apixaban	-3.8786
3978-86-7	Azatadine Maleate	-3.8782
59-05-2	Methotrexate	-3.8778
3681-99-0	Puerarin	-3.8778
19428-14-9	Benproperine phosphate	-3.8777
83905-01-5	Azithromycin	-3.8757
183133-96-2	Cabazitaxel	-3.8757
10402-53-6	Eprazinone dihydrochloride	-3.8752
738606-46-7	ETC1002	-3.875
57808-65-8	Closantel	-3.8743
2259-96-3	Cyclothiazide	-3.8741
543-82-8	2-Amino-6-methylheptane	-3.8733
127-79-7	Sulfamerazine	-3.8733
115256-11-6	Dofetilide	-3.873
62-68-0	Proadifen hydrochloride	-3.873
130-40-5	Flavin mononucleotide	-3.873
79660-72-3	Fleroxacin	-3.8729
84625-61-6	Itraconazole	-3.8723
364622-82-2	Doripenem Hydrate	-3.8716
4350-09-8	L-5-Hydroxytryptophan	-3.8713
127-79-7	Sulfamerazine	-3.8712
55079-83-9	Acitretin	-3.871
4852-22-6	Proanthocyanidins	-3.8709
95233-18-4	Atovaquone	-3.8709
1421373-65-0	Osimertinib	-3.8708
51481-61-9	Cimetidine	-3.8705
4852-22-6	Proanthocyanidins	-3.8701
55-56-1	Chlorhexidine	-3.8698

1421373-66-1	Osimertinib mesylate	-3.8696
179474-81-8	Prucalopride	-3.8694
1642-54-2	Diethylcarbamazine citrate	-3.8685
57-41-0	Phenytoin	-3.8685
53179-09-2	Sisomicin Sulfate	-3.8673
161715-24-8	Tebipenem Pivoxil	-3.8673
24390-14-5	Doxycycline hyclate	-3.8672
14984-68-0	Cloperastine hydrochloride	-3.8667
1221573-85-8	Paritaprevir	-3.8666
1420477-60-6	Acalabrutinib	-3.8662
34580-14-8	Ketotifen fumarate	-3.8658
137862-53-4	Valsartan	-3.8655
82419-36-1	Ofloxacin	-3.8655
100643-71-8	Desloratadine	-3.8653
1254053-43-4	Gilteritinib	-3.865
554-57-4	Methazolamide	-3.8646
71125-38-7	Meloxicam	-3.8645
67-43-6	Pentetic Acid	-3.8639
118072-93-8	Zoledronic Acid	-3.8637
100986-85-4	Levofloxacin	-3.8637
138199-71-0	Levofloxacin Hemihydrate	-3.8637
51-41-2	Norepinephrine	-3.8634
69815-49-2	Noradrenaline bitartrate	-3.8634
95233-18-4	Atovaquone	-3.8631
99291-25-5	Levodropropizine	-3.863
73816-42-9	Meclocycline sulfosalicylate salt	-3.8624
14611-52-0	Selegiline hydrochloride	-3.8623
122647-32-9	Ibutilide Fumarate	-3.8619
13636-18-5	Fendiline hydrochloride	-3.8614
65-45-2	Salicylamide	-3.8609
61-90-5	L-Leucine	-3.8607
4852-22-6	Proanthocyanidins	-3.8605
4852-22-6	Proanthocyanidins	-3.8601
73816-42-9	Meclocycline sulfosalicylate salt	-3.8599
4852-22-6	Proanthocyanidins	-3.8587
59-66-5	Acetazolamide	-3.8586
3759-92-0	Furaltadone hydrochloride	-3.8582
74150-27-9	Pimobendan	-3.8575
102518-79-6	(-)-Huperzine A	-3.8575
76824-35-6	Famotidine	-3.8574

4852-22-6	Proanthocyanidins	-3.8568
3963-95-9	Methacycline hydrochloride	-3.8568
194798-83-9	Fosfluconazole	-3.8552
864750-70-9	Revefenacin	-3.8543
3685-84-5	Meclofenoxate hydrochloride	-3.8535
951395-08-7	tafamidis meglumine	-3.8528
3685-84-5	Meclofenoxate hydrochloride	-3.8521
55981-09-4	Nitazoxanide	-3.8518
630-93-3	Phenytoin sodium	-3.8511
74150-27-9	Pimobendan	-3.851
706782-28-7	Pimavanserin tartrate	-3.8504
706779-91-1	Pimavanserin	-3.8504
211914-51-1	Dabigatran	-3.8502
4852-22-6	Proanthocyanidins	-3.85
53-60-1	Promazine hydrochloride	-3.8497
64019-93-8	Dipivefrin hydrochloride	-3.8487
4146-30-9	Neomycin Sulphate B	-3.8478
3697-42-5	Chlorhexidine hydrochloride	-3.8477
56-95-1	Chlorhexidine Acetate	-3.8477
127373-66-4	Sivelestat	-3.8468
57808-65-8	Closantel	-3.8456
7681-93-8	Pimaricin	-3.8455
4350-09-8	L-5-Hydroxytryptophan	-3.8452
6024-85-7	Tetrahydropalmatine hydrochloride	-3.8451
121808-62-6	Pidotimod	-3.8447
51940-44-4	Pipemidic acid	-3.8446
60-54-8	Tetracycline	-3.8443
6170-42-9	Chloropyramine hydrochloride	-3.8438
183204-72-0	Tipiracil hydrochloride	-3.8435
95233-18-4	Atovaquone	-3.843
74150-27-9	Pimobendan	-3.8429
5633-20-5	Oxybutynin	-3.8427
1508-65-2	Oxybutynin hydrochloride	-3.8427
173903-47-4	Tizoxanide	-3.8425
13114-29-9	Sodium Demethylcantharidate	-3.8422
1211441-98-3	Ribociclib	-3.842
71675-85-9	Amisulpride	-3.8418
74711-43-6	Zaltoprofen	-3.8417
154-93-8	Carmustine	-3.8412
65666-07-1	Silymarin	-3.8412
14639-25-9	Chromium picolinate	-3.8402
124937-52-6	Tolterodine tartrate	-3.8398

4852-22-6	Proanthocyanidins	-3.8397
87333-19-5	Ramipril	-3.8373
630420-16-5	Asunaprevir	-3.8371
182431-12-5	Lomitapide	-3.837
24729-96-2	Clindamycin phosphate	-3.8368
306-07-0	Pargyline hydrochloride	-3.8356
555-57-7	Pargyline	-3.8356
4852-22-6	Proanthocyanidins	-3.8355
491833-29-5	Eliglustat	-3.8349
127-07-1	Hydroxyurea	-3.8346
51-41-2	Norepinephrine	-3.8345
69815-49-2	Noradrenaline bitartrate	-3.8345
33125-97-2	Etomidate	-3.834
91296-87-6	Sarafloxacin hydrochloride	-3.8339
172152-50-0	Ilaprazole sodium	-3.8326
224452-66-8	Retapamulin	-3.8323
951395-08-7	tafamidis meglumine	-3.8317
13189-98-5	Fudosteine	-3.8314
543-82-8	2-Amino-6-methylheptane	-3.8303
78213-16-8	Diclofenac diethylamine	-3.8289
2259-96-3	Cyclothiazide	-3.8287
51940-44-4	Pipemidic acid	-3.8282
15307-86-5	Diclofenac	-3.8277
4852-22-6	Proanthocyanidins	-3.8275
4350-09-8	L-5-Hydroxytryptophan	-3.8271
4852-22-6	Proanthocyanidins	-3.8266
24356-60-3	Cephapirin Sodium	-3.8262
83905-01-5	Azithromycin	-3.8256
4852-22-6	Proanthocyanidins	-3.825
79307-93-0	Azelastine hydrochloride	-3.8249
606143-52-6	Selumetinib	-3.8242
4852-22-6	Proanthocyanidins	-3.8241
306-07-0	Pargyline hydrochloride	-3.8239
555-57-7	Pargyline	-3.8239
1665-48-1	Metaxalone	-3.8238
526-18-1	Osalmid	-3.8235
31282-04-9	Hygromycin B	-3.8233
32986-56-4	Tobramycin	-3.823
79307-93-0	Azelastine hydrochloride	-3.8226
151-67-7	Anestan	-3.8219
1226781-44-7	Omarigliptin	-3.8215
27262-48-2	Levobupivacaine HCl	-3.8212

27262-47-1	Levobupivacaine	-3.8212
18010-40-7	Bupivacaine hydrochloride	-3.8212
1405-10-3	Neomycin sulfate	-3.8207
112887-68-0	Raltitrexed	-3.8202
4330-99-8	Trimeprazine tartrate	-3.82
1665-48-1	Metaxalone	-3.8194
54063-53-5	Propafenone	-3.8192
4852-22-6	Proanthocyanidins	-3.8183
183204-72-0	Tipiracil hydrochloride	-3.8177
71963-77-4	Artemether	-3.8171
503612-47-3	Apixaban	-3.8166
95635-56-6	Ranolazine dihydrochloride	-3.8164
951395-08-7	tafamidis meglumine	-3.8162
17321-77-6	Clomipramine hydrochloride	-3.8162
33125-97-2	Etomidate	-3.8157
859-18-7	Lincomycin hydrochloride	-3.8154
93793-83-0	Roxatidine Acetate hydrochloride	-3.815
4008-48-4	Nitroxoline	-3.8147
60-87-7	Promethazine	-3.8146
7179-49-9	Lincomycin hydrochloride monohydrate	-3.8142
73816-42-9	Meclocycline sulfosalicylate salt	-3.8136
13171-25-0	Trimetazidine dihydrochloride	-3.8136
72-18-4	L-Valine	-3.8135
1665-48-1	Metaxalone	-3.8133
19428-14-9	Benproperine phosphate	-3.8129
107724-20-9	Eplerenone	-3.8126
95635-55-5	Ranolazine	-3.8122
302-95-4	Sodium Desoxycholate	-3.8121
83-44-3	Deoxycholic acid	-3.8121
1104-22-9	Meclizine dihydrochloride	-3.8117
10402-53-6	Eprazinone dihydrochloride	-3.8113
51333-22-3	Budesonide	-3.811
357-08-4	Naloxone hydrochloride	-3.8106
56238-63-2	Cefuroxime sodium	-3.8091
300-08-3	Arecoline HBr	-3.8088
65666-07-1	Silymarin	-3.8077
113852-37-2	Cidofovir	-3.8077
58066-85-6	Miltefosine	-3.8068
274901-16-5	Vildagliptin	-3.8065
69-65-8	D-Mannitol	-3.8064
220620-09-7	Tigecycline	-3.8059
5053-08-7	Fenspiride hydrochloride	-3.8059
4429-63-4	Tabersonine	-3.8058

661-19-8	n-Docosanol	-3.8058
2259-96-3	Cyclothiazide	-3.8057
13114-29-9	Sodium Demethylcantharidate	-3.8054
115256-11-6	Dofetilide	-3.8048
102-76-1	Triacetin	-3.8038
122320-73-4	Rosiglitazone	-3.8038
5633-20-5	Oxybutynin	-3.803
1508-65-2	Oxybutynin hydrochloride	-3.803
57-15-8	Chlorobutanol	-3.8029
80-50-2	Anisotropine Methylbromide	-3.8021
151-67-7	Anestan	-3.8019
5986-55-0	Patchouli alcohol	-3.8014
259793-96-9	Favipiravir	-3.8003
14611-52-0	Selegiline hydrochloride	-3.7998
4852-22-6	Proanthocyanidins	-3.7995
61422-45-5	Carmofur	-3.799
100986-85-4	Levofloxacin	-3.7988
138199-71-0	Levofloxacin Hemihydrate	-3.7988
4852-22-6	Proanthocyanidins	-3.7986
136434-34-9	Duloxetine hydrochloride	-3.7983
82419-36-1	Ofloxacin	-3.7982
59804-37-4	Tenoxicam	-3.7979
65666-07-1	Silymarin	-3.797
90729-43-4	Ebastine	-3.7969
24390-14-5	Doxycycline hyclate	-3.7969
31282-04-9	Hygromycin B	-3.7964
58-46-8	Tetrabenazine	-3.7962
665-66-7	Amantadine hydrochloride	-3.7954
444731-52-6	Pazopanib	-3.7948
894-71-3	Nortriptyline hydrochloride	-3.7933
6035-45-6	Folinic Acid Calcium Salt Pentahydrate	-3.7929
15622-65-8	Molindone hydrochloride	-3.7926
302-95-4	Sodium Desoxycholate	-3.7924
83-44-3	Deoxycholic acid	-3.7921
65666-07-1	Silymarin	-3.792
59-33-6	Pyrilamine maleate	-3.7916
4330-99-8	Trimeprazine tartrate	-3.7914
6990-06-3	Fusidine	-3.7911
635702-64-6	Pazopanib HCl	-3.7909
127-56-0	Sulfacetamide sodium	-3.7906
6209-17-2	Sulfacetamide sodium salt hydrate	-3.7906
154-69-8	Tripelennamine hydrochloride	-3.7904
122320-73-4	Rosiglitazone	-3.7899

60-93-5	Quinine dihydrochloride	-3.7898
130-95-0	Quinine	-3.7898
64019-93-8	Dipivefrin hydrochloride	-3.7895
71125-38-7	Meloxicam	-3.7895
630-93-3	Phenytoin sodium	-3.7894
53179-09-2	Sisomicin Sulfate	-3.789
1405-10-3	Neomycin sulfate	-3.789
82186-77-4	Lumefantrine	-3.7883
1104-22-9	Meclizine dihydrochloride	-3.7882
120014-06-4	Donepezil	-3.7878
82186-77-4	Lumefantrine	-3.7873
951395-08-7	tafamidis meglumine	-3.7872
163451-81-8	Teriflunomide	-3.7869
108605-62-5	Teriflunomide	-3.7869
4330-99-8	Trimeprazine tartrate	-3.7865
138890-62-7	Brinzolamide	-3.7864
56391-57-2	Netilmicin sulfate	-3.786
27848-84-6	Nicergoline	-3.786
13636-18-5	Fendiline hydrochloride	-3.7856
80-50-2	Anisotropine Methylbromide	-3.7854
120011-70-3	Donepezil HCl	-3.7854
71125-38-7	Meloxicam	-3.7847
4852-22-6	Proanthocyanidins	-3.7842
12650-69-0	Mupirocin	-3.7841
75438-57-2	Moxonidine	-3.7836
2753-45-9	Mebeverine hydrochloride	-3.7826
122320-73-4	Rosiglitazone	-3.7824
145040-37-5	Candesartan Cilexetil	-3.7824
1405-41-0	Gentamycin sulfate	-3.7814
1256580-46-7	Alectinib	-3.7809
15622-65-8	Molindone hydrochloride	-3.7809
94-07-5	Synephrine	-3.7806
95233-18-4	Atovaquone	-3.7803
90-22-2	Valethamate bromide	-3.7802
4852-22-6	Proanthocyanidins	-3.7792
94-07-5	Synephrine	-3.7783
56-92-8	Histamine 2HCl	-3.7782
51-45-6	Histamine	-3.7782
630420-16-5	Asunaprevir	-3.7778
1226781-44-7	Omarigliptin	-3.7778
54063-53-5	Propafenone	-3.7775

6078-17-7	Berbamine dihydrochloride	-3.777
121268-17-5	Alendronate sodium hydrate	-3.776
1405-41-0	Gentamycin sulfate	-3.775
21736-83-4	Spectinomycin dihydrochloride	-3.775
149003-01-0	Dexrazoxane HCl	-3.7748
543-82-8	2-Amino-6-methylheptane	-3.7742
77337-73-6	Acamprosate calcium	-3.7736
4852-22-6	Proanthocyanidins	-3.7735
1421373-65-0	Osimertinib	-3.7731
32854-75-4	Lappaconitine	-3.7725
13114-29-9	Sodium Demethylcantharidate	-3.7722
1421373-66-1	Osimertinib mesylate	-3.7722
79660-72-3	Fleroxacin	-3.772
6620-60-6	Proglumide	-3.7716
58066-85-6	Miltefosine	-3.7713
155148-31-5	Plerixafor 8HCl (AMD3100 8HCl)	-3.7712
110078-46-1	Plerixafor	-3.7712
57-41-0	Phenytoin	-3.7711
123948-87-8	Topotecan	-3.771
577-11-7	Docusate sodium	-3.7705
723-46-6	Sulfamethoxazole	-3.7705
13114-29-9	Sodium Demethylcantharidate	-3.7705
496-67-3	Bromisoval	-3.7696
4852-22-6	Proanthocyanidins	-3.7691
10040-45-6	Sodium Picosulfate	-3.7689
4852-22-6	Proanthocyanidins	-3.7684
55-98-1	Busulfan	-3.7683
53179-09-2	Sisomicin Sulfate	-3.7682
1218779-75-9	Apatinib mesylate	-3.7679
811803-05-1	Apatinib	-3.7679
65666-07-1	Silymarin	-3.7678
6035-45-6	Folinic Acid Calcium Salt Pentahydrate	-3.7673
1218779-75-9	Apatinib mesylate	-3.7672
811803-05-1	Apatinib	-3.7672
4852-22-6	Proanthocyanidins	-3.7671
1009119-64-5	Daclatasvir	-3.7671
35554-44-0	Imazalil	-3.7665
121268-17-5	Alendronate sodium hydrate	-3.7661
61422-45-5	Carmofur	-3.7655

1229-29-4	Doxepin hydrochloride	-3.7653
82571-53-7	Ozagrel	-3.7651
1354744-91-4	Naloxegol oxalate	-3.7647
65666-07-1	Silymarin	-3.7646
2259-96-3	Cyclothiazide	-3.764
172152-50-0	Ilaprazole sodium	-3.7635
60-93-5	Quinine dihydrochloride	-3.7632
130-95-0	Quinine	-3.7632
10238-21-8	Glyburide	-3.763
3778-73-2	Ifosfamide	-3.7627
434-07-1	Oxymetholone	-3.7625
59-30-3	Folic acid	-3.7625
4697-14-7	Ticarcillin disodium	-3.7624
1098-60-8	Triflupromazine hydrochloride	-3.7624
55-98-1	Busulfan	-3.7622
194798-83-9	Fosfluconazole	-3.7614
4852-22-6	Proanthocyanidins	-3.7613
341-69-5	Orphenadrine hydrochloride	-3.761
123447-62-1	Prulifloxacin	-3.7608
2192-20-3	Hydroxyzine dihydrochloride	-3.7607
56-92-8	Histamine 2HCl	-3.7606
51-45-6	Histamine	-3.7606
15574-96-6	Pizotifen	-3.7599
5189-11-7	Pizotifen Malate	-3.7599
154229-19-3	Abiraterone	-3.7597
31282-04-9	Hygromycin B	-3.7594
10246-75-0	Hydroxyzine Pamoate	-3.7589
137-58-6	Xylocaine	-3.7584
94-07-5	Synephrine	-3.7581
6108-05-0	Lidocaine Hydrochloride hydrate	-3.7581
73-78-9	Lidocaine hydrochloride	-3.7581
951395-08-7	tafamidis meglumine	-3.7578
74150-27-9	Pimobendan	-3.7566
751-94-0	Sodium fusidate	-3.7565
389139-89-3	Omadacycline	-3.7563
804-30-8	Fursultiamine	-3.7563
951395-08-7	tafamidis meglumine	-3.7562
83-44-3	Deoxycholic acid	-3.7562
1135-24-6	Ferulic Acid	-3.756
55661-38-6	Nimustine Hydrochloride	-3.7557
2259-96-3	Cyclothiazide	-3.7556
56839-43-1	Eperisone hydrochloride	-3.7556

78712-43-3	Ozagrel hydrochloride	-3.7555
127-56-0	Sulfacetamide sodium	-3.7548
6209-17-2	Sulfacetamide sodium salt hydrate	-3.7548
13171-25-0	Trimetazidine dihydrochloride	-3.7542
1009119-65-6	Daclatasvir dihydrochloride	-3.7542
543-82-8	2-Amino-6-methylheptane	-3.754
66357-59-3	Ranitidine Hydrochloride	-3.7537
144689-24-7	Olmesartan	-3.7535
4852-22-6	Proanthocyanidins	-3.7531
78712-43-3	Ozagrel hydrochloride	-3.7527
32986-56-4	Tobramycin	-3.7526
82571-53-7	Ozagrel	-3.7524
357-08-4	Naloxone hydrochloride	-3.7522
4852-22-6	Proanthocyanidins	-3.7514
1009119-65-6	Daclatasvir dihydrochloride	-3.7511
107133-36-8	Perindopril erbumine	-3.7486
41575-94-4	Carboplatin	-3.7484
4146-30-9	Neomycin Sulphate B	-3.7481
2259-96-3	Cyclothiazide	-3.7479
17737-65-4	Clonixin	-3.7462
66357-59-3	Ranitidine Hydrochloride	-3.7458
4852-22-6	Proanthocyanidins	-3.7456
121268-17-5	Alendronate sodium hydrate	-3.7454
721-50-6	Prilocaine	-3.7451
4852-22-6	Proanthocyanidins	-3.7451
1981-58-4	Sulfamethazine sodium salt	-3.7448
85409-38-7	Rilmenidine Phosphate	-3.7443
482-36-0	Hyperoside	-3.7442
69-09-0	Chlorpromazine hydrochloride	-3.7431
59-33-6	Pyrilamine maleate	-3.743
1786-81-8	Propitocaine hydrochloride	-3.7427
1218779-75-9	Apatinib mesylate	-3.7426
811803-05-1	Apatinib	-3.7426
179474-81-8	Prucalopride	-3.7424
3963-95-9	Methacycline hydrochloride	-3.7418
88150-42-9	Amlodipine	-3.7412
64-75-5	Tetracycline hydrochloride	-3.7411
55-98-1	Busulfan	-3.741
118072-93-8	Zoledronic Acid	-3.7408
5633-20-5	Oxybutynin	-3.7388

1508-65-2	Oxybutynin hydrochloride	-3.7388
5633-20-5	Oxybutynin	-3.7388
1508-65-2	Oxybutynin hydrochloride	-3.7388
1231930-82-7	LY2835219	-3.7385
6160-12-9	(-)-Sparteine sulfate pentahydrate	-3.738
577-11-7	Docusate sodium	-3.737
1405-41-0	Gentamycin sulfate	-3.7363
59-66-5	Acetazolamide	-3.7359
57-37-4	Benactyzine hydrochloride	-3.7356
7414-83-7	Sodium etidronate	-3.7355
1642-54-2	Diethylcarbamazine citrate	-3.7348
13114-29-9	Sodium Demethylcantharidate	-3.7345
2062-78-4	Pimozide	-3.7334
7421-40-1	Carbenoxolone disodium	-3.7333
65666-07-1	Silymarin	-3.7327
1350514-68-9	Grazoprevir	-3.7326
122111-03-9	Gemcitabine HCl	-3.7322
95058-81-4	Gemcitabine	-3.7322
116649-85-5	Ramatroban	-3.7317
864750-70-9	Revefenacin	-3.7313
30484-77-6	Flunarizine dihydrochloride	-3.7313
97-59-6	Allantoin	-3.7307
4350-09-8	L-5-Hydroxytryptophan	-3.7295
386750-22-7	Desvenlafaxine succinate hydrate	-3.7285
93413-62-8	Desvenlafaxine	-3.7285
751-94-0	Sodium fusidate	-3.728
61-90-5	L-Leucine	-3.7279
2809-21-4	Etidronate	-3.7277
T4284	(S)-Amlodipine Besylate (103129-82-4(free base))	-3.7274
88150-42-9	Amlodipine	-3.7274
10402-53-6	Eprazinone dihydrochloride	-3.726
1405-41-0	Gentamycin sulfate	-3.7257
681492-22-8	Delamanid	-3.7255
55-86-7	Mechlorethamine hydrochloride	-3.7253
61825-94-3	Oxaliplatin	-3.7248
147403-03-0	Azilsartan	-3.724
4852-22-6	Proanthocyanidins	-3.7216
548-62-9	Crystal Violet	-3.7215
1421373-65-0	Osimertinib	-3.7197
88150-42-9	Amlodipine	-3.7195

386750-22-7	Desvenlafaxine succinate hydrate	-3.7193
93413-62-8	Desvenlafaxine	-3.7193
61422-45-5	Carmofur	-3.7174
721-50-6	Prilocaine	-3.7166
56391-57-2	Netilmicin sulfate	-3.7165
53179-09-2	Sisomicin Sulfate	-3.7163
4852-22-6	Proanthocyanidins	-3.7157
1421373-66-1	Osimertinib mesylate	-3.7149
10163-15-2	Disodium monofluorophosphate	-3.7149
951395-08-7	tafamidis meglumine	-3.7149
1786-81-8	Propitocaine hydrochloride	-3.7148
439239-90-4	Lasmiditan	-3.7135
4852-22-6	Proanthocyanidins	-3.7127
61718-82-9	Fluvoxamine maleate	-3.7125
57808-66-9	Domperidone	-3.7123
56-92-8	Histamine 2HCl	-3.7123
51-45-6	Histamine	-3.7123
1435-55-8	Hydroquinidine	-3.712
3759-92-0	Furaltadone hydrochloride	-3.7117
637-32-1	Proguanil hydrochloride	-3.7115
60-87-7	Promethazine	-3.7109
83905-01-5	Azithromycin	-3.7089
6064-83-1	Fosfosal	-3.7088
65666-07-1	Silymarin	-3.7087
75438-57-2	Moxonidine	-3.7076
65666-07-1	Silymarin	-3.7076
55-86-7	Mechlorethamine hydrochloride	-3.7074
65666-07-1	Silymarin	-3.7074
144-83-2	Sulfapyridine	-3.7071
59-30-3	Folic acid	-3.7068
317-34-0	Aminophylline	-3.7067
21462-39-5	Clindamycin HCl	-3.7059
1508-75-4	Tropicamide	-3.7059
1403254-99-8	EPZ6438	-3.7058
53179-09-2	Sisomicin Sulfate	-3.7035
120-97-8	Dichlorphenamide	-3.7031
914462-92-3	Rolapitant hydrochloride	-3.703
380843-75-4	Bosutinib	-3.7023
951395-08-7	tafamidis meglumine	-3.7015
1446321-46-5	voxelotor	-3.7014

155148-31-5	Plerixafor 8HCl (AMD3100 8HCl)	-3.7011
110078-46-1	Plerixafor	-3.7011
4852-22-6	Proanthocyanidins	-3.7005
118072-93-8	Zoledronic Acid	-3.6999
82419-36-1	Ofloxacin	-3.6997
951395-08-7	tafamidis meglumine	-3.6995
122647-32-9	Ibutilide Fumarate	-3.6994
73-32-5	isoleucine	-3.6992
57808-65-8	Closantel	-3.6991
10402-53-6	Eprazinone dihydrochloride	-3.699
132866-11-6	Lercanidipine hydrochloride	-3.6989
961-29-5	Isoliquiritigenin	-3.6985
100427-26-7	Lercanidipine	-3.6983
364622-82-2	Doripenem Hydrate	-3.697
118072-93-8	Zoledronic Acid	-3.6969
22373-78-0	Monensin sodium salt	-3.6962
341-69-5	Orphenadrine hydrochloride	-3.6954
128-13-2	Ursodeoxycholic acid	-3.6951
82419-36-1	Ofloxacin	-3.695
2259-96-3	Cyclothiazide	-3.6948
3737-09-5	Disopyramide	-3.6946
4852-22-6	Proanthocyanidins	-3.6939
111025-46-8	Pioglitazone	-3.6935
860-79-7	Cyclovirobuxin D	-3.6931
4852-22-6	Proanthocyanidins	-3.6929
91296-87-6	Sarafloxacin hydrochloride	-3.6926
80-50-2	Anisotropine Methylbromide	-3.6924
119478-55-6	Danofloxacin mesylate	-3.6914
4852-22-6	Proanthocyanidins	-3.6913
65666-07-1	Silymarin	-3.6895
380843-75-4	Bosutinib	-3.6892
1405-41-0	Gentamycin sulfate	-3.6884
1260141-27-2	Vonoprazan fumarate	-3.6878
97-59-6	Allantoin	-3.6878
1350514-68-9	Grazoprevir	-3.6876
1260141-27-2	Vonoprazan fumarate	-3.6872
224452-66-8	Retapamulin	-3.6865
95233-18-4	Atovaquone	-3.6857
81-23-2	Dehydrocholic acid	-3.6855
82571-53-7	Ozagrel	-3.6846

951395-08-7	tafamidis meglumine	-3.6845
65666-07-1	Silymarin	-3.6844
57808-65-8	Closantel	-3.6842
57-96-5	Sulfinpyrazone	-3.6837
13114-29-9	Sodium Demethylcantharidate	-3.6837
123447-62-1	Prulifloxacin	-3.6829
78712-43-3	Ozagrel hydrochloride	-3.6828
55-86-7	Mechlorethamine hydrochloride	-3.6818
5053-08-7	Fenspiride hydrochloride	-3.6814
4852-22-6	Proanthocyanidins	-3.6814
668270-12-0	Linagliptin	-3.6805
4350-09-8	L-5-Hydroxytryptophan	-3.6803
211915-06-9	Dabigatran etexilate	-3.6802
6990-06-3	Fusidine	-3.6801
637-32-1	Proguanil hydrochloride	-3.6786
211915-06-9	Dabigatran etexilate	-3.6781
4852-22-6	Proanthocyanidins	-3.6781
721-50-6	Prilocaine	-3.678
543-82-8	2-Amino-6-methylheptane	-3.6779
751-94-0	Sodium fusidate	-3.6774
60-31-1	Acetylcholine chloride	-3.6771
3094-09-5	Doxifluridine	-3.677
56839-43-1	Eperisone hydrochloride	-3.6762
31282-04-9	Hygromycin B	-3.6758
1786-81-8	Propitocaine hydrochloride	-3.6753
50-65-7	Niclosamide	-3.6751
155148-31-5	Plerixafor 8HCl (AMD3100 8HCl)	-3.6747
110078-46-1	Plerixafor	-3.6747
64-65-3	Bemegrid	-3.674
1405-41-0	Gentamycin sulfate	-3.6738
54965-24-1	Tamoxifen Z-isomer citrate	-3.6738
135729-62-3	Palonosetron hydrochloride	-3.6736
10540-29-1	Tamoxifen	-3.6732
4852-22-6	Proanthocyanidins	-3.6728
4146-30-9	Neomycin Sulphate B	-3.6726
60-87-7	Promethazine	-3.6717
122647-32-9	Ibutilide Fumarate	-3.6708
112811-59-3	Gatifloxacin	-3.6707
13114-29-9	Sodium Demethylcantharidate	-3.6702
79-57-2	Oxytetracycline	-3.6699
4852-22-6	Proanthocyanidins	-3.6695
4330-99-8	Trimeprazine tartrate	-3.6683
4852-22-6	Proanthocyanidins	-3.6679

59-52-9	Dithiopropanol	-3.6676
1132935-63-7	Dasabuvir	-3.6676
523-87-5	Dimenhydrinate	-3.667
116649-85-5	Ramatroban	-3.6668
7081-44-9	Cloxacillin sodium monohydrate	-3.6668
843663-66-1	Bedaquiline	-3.6663
4852-22-6	Proanthocyanidins	-3.666
69056-38-8	Sapropterin Hydrochloride	-3.6659
65666-07-1	Silymarin	-3.6659
75438-57-2	Moxonidine	-3.6656
147-24-0	Diphenhydramine hydrochloride	-3.6652
52-66-4	DL-Penicillamine	-3.665
107-35-7	Taurine	-3.6649
53179-09-2	Sisomicin Sulfate	-3.6649
357-08-4	Naloxone hydrochloride	-3.6649
721-50-6	Prilocaine	-3.6647
80433-71-2	Calcium Levofolinate	-3.6645
306-07-0	Pargyline hydrochloride	-3.6634
555-57-7	Pargyline	-3.6634
104376-79-6	Ceftriaxone sodium	-3.6631
41354-29-4	Cyproheptadine hydrochloride	-3.6631
5053-08-7	Fenspiride hydrochloride	-3.663
130-40-5	Flavin mononucleotide	-3.663
1786-81-8	Propitocaine hydrochloride	-3.6629
1211441-98-3	Ribociclib	-3.662
83-73-8	Iodoquinol	-3.6605
543-15-7	Heptaminol hydrochloride	-3.6604
52-90-4	cysteine	-3.6601
4852-22-6	Proanthocyanidins	-3.66
59-52-9	Dithiopropanol	-3.66
24390-14-5	Doxycycline hyclate	-3.6598
79-57-2	Oxytetracycline	-3.6597
721-50-6	Prilocaine	-3.6589
155148-31-5	Plerixafor 8HCl (AMD3100 8HCl)	-3.6575
110078-46-1	Plerixafor	-3.6575
66357-59-3	Ranitidine Hydrochloride	-3.6575
65666-07-1	Silymarin	-3.6572
59-33-6	Pyrilamine maleate	-3.6563
306-07-0	Pargyline hydrochloride	-3.655
555-57-7	Pargyline	-3.655
13189-98-5	Fudosteine	-3.6545

148849-67-6	Ivabradine hydrochloride	-3.6542
1786-81-8	Propitocaine hydrochloride	-3.6538
54965-21-8	Albendazole	-3.6536
63-68-3	L-Methionine	-3.6528
23964-57-0	Articaine HCl	-3.6521
6064-83-1	Fosfosal	-3.6503
315-30-0	Allopurinol	-3.6503
329-63-5	DL-Adrenaline Hydrochloride	-3.6497
548-66-3	Drofenine hydrochloride	-3.6493
665-66-7	Amantadine hydrochloride	-3.6492
53797-35-6	Ribostamycin sulfate	-3.6486
665-66-7	Amantadine hydrochloride	-3.6484
1642-54-2	Diethylcarbamazine citrate	-3.6483
73816-42-9	Meclocycline sulfosalicylate salt	-3.6481
863031-21-4	Azilsartan Medoxomil	-3.648
51-41-2	Norepinephrine	-3.6476
69815-49-2	Noradrenaline bitartrate	-3.6476
155148-31-5	Plerixafor 8HCl (AMD3100 8HCl)	-3.6466
110078-46-1	Plerixafor	-3.6466
123948-87-8	Topotecan	-3.6463
76801-85-9	Azathramycin	-3.6462
1786-81-8	Propitocaine hydrochloride	-3.6461
721-50-6	Prilocaine	-3.6458
23210-58-4	Ifenprodil Tartrate	-3.6456
37517-28-5	Amikacin	-3.6455
81-23-2	Dehydrocholic acid	-3.6443
118072-93-8	Zoledronic Acid	-3.6442
53179-09-2	Sisomicin Sulfate	-3.6439
341-69-5	Orphenadrine hydrochloride	-3.643
548-66-3	Drofenine hydrochloride	-3.6427
32986-56-4	Tobramycin	-3.6427
154598-52-4	Efavirenz	-3.6424
14984-68-0	Cloperastine hydrochloride	-3.6424
130-16-5	Dermofongin	-3.6422
125-02-0	Prednisolone phosphate sodium	-3.6409
329-63-5	DL-Adrenaline Hydrochloride	-3.6408
22839-47-0	Aspartame	-3.6406
65666-07-1	Silymarin	-3.6402
738606-46-7	ETC1002	-3.6399
1256580-46-7	Alectinib	-3.6399
65666-07-1	Silymarin	-3.6397
70775-75-6	Octenidine Dihydrochloride	-3.6392

31282-04-9	Hygromycin B	-3.639
306-07-0	Pargyline hydrochloride	-3.6384
555-57-7	Pargyline	-3.6384
70458-95-6	Pefloxacin Mesylate	-3.6382
155148-31-5	Plerixafor 8HCl (AMD3100 8HCl)	-3.6379
110078-46-1	Plerixafor	-3.6379
380843-75-4	Bosutinib	-3.6375
90729-43-4	Ebastine	-3.6371
151-67-7	Anestan	-3.6368
63-68-3	L-Methionine	-3.6358
149003-01-0	Dexrazoxane HCl	-3.6358
149676-40-4	Pefloxacin Mesylate Dihydrate	-3.6358
56839-43-1	Eperisone hydrochloride	-3.6355
31282-04-9	Hygromycin B	-3.6353
3685-84-5	Meclofenoxate hydrochloride	-3.6351
2259-96-3	Cyclothiazide	-3.6349
57-15-8	Chlorobutanol	-3.6346
4852-22-6	Proanthocyanidins	-3.6337
749-02-0	Spiperone	-3.6336
73-32-5	isoleucine	-3.6324
4852-22-6	Proanthocyanidins	-3.632
469-77-2	Spiculisporic Acid	-3.6318
4852-22-6	Proanthocyanidins	-3.6315
18683-91-5	Ambroxol	-3.6301
747-36-4	Hydroxychloroquine sulfate	-3.6301
1104-22-9	Meclizine dihydrochloride	-3.6296
4852-22-6	Proanthocyanidins	-3.6292
3679-64-9	Salifungin	-3.6277
138844-81-2	Ibandronate sodium	-3.6276
23828-92-4	Ambroxol hydrochloride	-3.6262
4852-22-6	Proanthocyanidins	-3.6256
61-90-5	L-Leucine	-3.6255
936727-05-8	Lumacaftor	-3.6254
1254053-43-4	Gilteritinib	-3.6251
91374-20-8	Ropinirole hydrochloride	-3.6248
65666-07-1	Silymarin	-3.6248
80-50-2	Anisotropine Methylbromide	-3.6247
118072-93-8	Zoledronic Acid	-3.6233
194798-83-9	Fosfluconazole	-3.6226
80433-71-2	Calcium Levofolinate	-3.6202
59-05-2	Methotrexate	-3.62
3717-88-2	Flavoxate hydrochloride	-3.62

67-68-5	Dimethyl sulfoxide	-3.6198
341-69-5	Orphenadrine hydrochloride	-3.6196
127-07-1	Hydroxyurea	-3.6195
87-33-2	Isosorbide dinitrate	-3.6194
951395-08-7	tafamidis meglumine	-3.6186
65-86-1	Orotic acid	-3.6181
120-97-8	Dichlorphenamide	-3.6172
56238-63-2	Cefuroxime sodium	-3.6171
83-73-8	Iodoquinol	-3.617
15622-65-8	Molindone hydrochloride	-3.6169
15574-96-6	Pizotifen	-3.6155
5189-11-7	Pizotifen Malate	-3.6155
30484-77-6	Flunarizine dihydrochloride	-3.6151
153-98-0	Serotonin hydrochloride	-3.6147
287714-41-4	Rosuvastatin	-3.6147
23828-92-4	Ambroxol hydrochloride	-3.6134
1492-18-8	Calcium folinate	-3.6133
111025-46-8	Pioglitazone	-3.6131
152459-95-5	Imatinib	-3.613
220127-57-1	Imatinib Mesylate	-3.613
18683-91-5	Ambroxol	-3.6125
65666-07-1	Silymarin	-3.6118
521-78-8	Trimipramine maleate	-3.6105
79-57-2	Oxytetracycline	-3.6104
95635-55-5	Ranolazine	-3.6101
125-02-0	Prednisolone phosphate sodium	-3.6098
123948-87-8	Topotecan	-3.6093
65666-07-1	Silymarin	-3.6092
3858-89-7	Chloroprocaine HCl	-3.6091
24729-96-2	Clindamycin phosphate	-3.6088
70458-95-6	Pefloxacin Mesylate	-3.6085
111025-46-8	Pioglitazone	-3.6084
95635-56-6	Ranolazine dihydrochloride	-3.6083
637-58-1	Pramoxine hydrochloride	-3.6083
4852-22-6	Proanthocyanidins	-3.6082
107-35-7	Taurine	-3.6076
149676-40-4	Pefloxacin Mesylate Dihydrate	-3.6055
341-69-5	Orphenadrine hydrochloride	-3.6055
155148-31-5	Plerixafor 8HCl (AMD3100 8HCl)	-3.6048
110078-46-1	Plerixafor	-3.6048
66357-59-3	Ranitidine Hydrochloride	-3.6039
459789-99-2	Obeticholic Acid	-3.6034
5080-50-2	O-Acetyl-L-carnitine hydrochloride	-3.6024

2753-45-9	Mebeverine hydrochloride	-3.6023
74711-43-6	Zaltoprofen	-3.6016
4852-22-6	Proanthocyanidins	-3.6013
15574-96-6	Pizotifen	-3.6005
5189-11-7	Pizotifen Malate	-3.6005
53716-49-7	Carprofen	-3.6004
543-82-8	2-Amino-6-methylheptane	-3.6002
773092-05-0	Acotiamide hydrochloride	-3.6002
1405-41-0	Gentamycin sulfate	-3.6002
1405-10-3	Neomycin sulfate	-3.6002
50924-49-7	Mizoribine	-3.6
1256580-46-7	Alectinib	-3.6
496775-62-3	Eltrombopag Olamine	-3.5992
10402-53-6	Eprazinone dihydrochloride	-3.5987
141-94-6	Hexetidine	-3.5986
51481-61-9	Cimetidine	-3.5984
95233-18-4	Atovaquone	-3.5982
79-55-0	Pempidine	-3.5978
7083-71-8	(+)-Emetine dihydrochloride hydrate	-3.5955
4852-22-6	Proanthocyanidins	-3.5952
3543-75-7	Bendamustine hydrochloride	-3.5951
61825-94-3	Oxaliplatin	-3.5941
106685-40-9	Adapalene	-3.5939
79-55-0	Pempidine	-3.5931
4852-22-6	Proanthocyanidins	-3.5931
67-68-5	Dimethyl sulfoxide	-3.5915
115256-11-6	Dofetilide	-3.5905
4852-22-6	Proanthocyanidins	-3.5905
22862-76-6	Anisomycin	-3.5904
13114-29-9	Sodium Demethylcantharidate	-3.5889
1226781-44-7	Omarigliptin	-3.5885
5633-20-5	Oxybutynin	-3.5882
1508-65-2	Oxybutynin hydrochloride	-3.5882
130-16-5	Dermofongin	-3.5877
259793-96-9	Favipiravir	-3.5876
145040-37-5	Candesartan Cilexetil	-3.5874
3978-86-7	Azatadine Maleate	-3.5849
95635-55-5	Ranolazine	-3.5838
79-57-2	Oxytetracycline	-3.5832
315-30-0	Allopurinol	-3.5831
136-47-0	Tetracaine hydrochloride	-3.5831

1009119-64-5	Daclatasvir	-3.5826
10163-15-2	Disodium monofluorophosphate	-3.5821
3759-92-0	Furaltadone hydrochloride	-3.582
95635-56-6	Ranolazine dihydrochloride	-3.5817
25389-94-0	Kanamycin sulfate	-3.5813
31282-04-9	Hygromycin B	-3.5802
144-82-1	Sulfamethizole	-3.5799
4852-22-6	Proanthocyanidins	-3.5796
50-65-7	Niclosamide	-3.5793
89396-94-1	Imidapril HCl	-3.5792
56839-43-1	Eperisone hydrochloride	-3.5789
606143-89-9	Binimetinib	-3.5788
951395-08-7	tafamidis meglumine	-3.5788
54-30-8	Camylofin	-3.5786
1226781-44-7	Omarigliptin	-3.5774
60-31-1	Acetylcholine chloride	-3.5772
5934-29-2	L-Histidine monohydrochloride monohydrat	-3.5762
71-00-1	histidine	-3.5762
624-49-7	Dimethyl fumarate	-3.5761
464-92-6	Asiatic acid	-3.5761
119478-55-6	Danofloxacin mesylate	-3.5759
843663-66-1	Bedaquiline	-3.5751
872728-81-9	Dabigatran Etexilate Mesylate	-3.5744
211915-06-9	Dabigatran etexilate	-3.5744
153-98-0	Serotonin hydrochloride	-3.5739
182349-12-8	Rupatadine Fumarate	-3.5739
459868-92-9	Rucaparib Phosphate	-3.5734
283173-50-2	Rucaparib	-3.5734
54-30-8	Camylofin	-3.5734
546-88-3	Acetohydroxamic acid	-3.5733
520-36-5	Apigenin	-3.5732
13114-29-9	Sodium Demethylcantharidate	-3.5728
6035-45-6	Folinic Acid Calcium Salt Pentahydrate	-3.5727
83-73-8	Iodoquinol	-3.5717
96829-58-2	Orlistat	-3.5716
56-85-9	L-Glutamine	-3.5714
136-47-0	Tetracaine hydrochloride	-3.5714
13114-29-9	Sodium Demethylcantharidate	-3.5714
89796-99-6	Aceclofenac	-3.5708
107133-36-8	Perindopril erbumine	-3.5703
224452-66-8	Retapamulin	-3.5703

136434-34-9	Duloxetine hydrochloride	-3.57
66357-59-3	Ranitidine Hydrochloride	-3.5696
364622-82-2	Doripenem Hydrate	-3.5695
21736-83-4	Spectinomycin dihydrochloride	-3.5681
65666-07-1	Silymarin	-3.5675
24390-14-5	Doxycycline hyclate	-3.5672
120-97-8	Dichlorphenamide	-3.5669
4852-22-6	Proanthocyanidins	-3.5662
864750-70-9	Revefenacin	-3.565
53179-09-2	Sisomicin Sulfate	-3.5642
121268-17-5	Alendronate sodium hydrate	-3.5626
118072-93-8	Zoledronic Acid	-3.5626
107-43-7	Betaine	-3.5623
590-46-5	Betaine hydrochloride	-3.5623
67-68-5	Dimethyl sulfoxide	-3.5615
115939-25-8	Salvianolic acid B	-3.5599
107753-78-6	Zafirlukast	-3.5592
22252-43-3	Cefalexine EP IMpurity B	-3.559
386750-22-7	Desvenlafaxine succinate hydrate	-3.5587
93413-62-8	Desvenlafaxine	-3.5587
51012-33-0	Tiapride hydrochloride	-3.5587
121268-17-5	Alendronate sodium hydrate	-3.5582
668270-12-0	Linagliptin	-3.558
72-19-5	threonine	-3.5562
4852-22-6	Proanthocyanidins	-3.556
31282-04-9	Hygromycin B	-3.5555
60142-95-2	Gabapentin HCl	-3.5552
60142-96-3	Gabapentin	-3.5552
3858-89-7	Chloroprocaine HCl	-3.5552
24356-60-3	Cephapirin Sodium	-3.555
108612-45-9	Mizolastine	-3.5548
82186-77-4	Lumefantrine	-3.5543
53179-09-2	Sisomicin Sulfate	-3.5541
71610-00-9	Cephalomannine	-3.554
137-58-6	Xylocaine	-3.5533
4852-22-6	Proanthocyanidins	-3.5527
56238-63-2	Cefuroxime sodium	-3.5526
120014-06-4	Donepezil	-3.5526
127373-66-4	Sivelestat	-3.5522
50-65-7	Niclosamide	-3.5522
10402-53-6	Eprazinone dihydrochloride	-3.5519
22373-78-0	Monensin sodium salt	-3.5515
1665-48-1	Metaxalone	-3.5514

1230487-00-9	Siponimod	-3.551
1986-47-6	Tranylcypromine (2-PCPA) hydrochloride	-3.5508
120011-70-3	Donepezil HCl	-3.5505
554-57-4	Methazolamide	-3.5505
65-86-1	Orotic acid	-3.5502
951395-08-7	tafamidis meglumine	-3.55
97-59-6	Allantoin	-3.5498
67814-76-0	Isepamicin sulfate	-3.5496
59-66-5	Acetazolamide	-3.549
315-30-0	Allopurinol	-3.5481
18085-97-7	Jaceosidin	-3.5476
87-17-2	Salicylanilide	-3.5473
389139-89-3	Omadacycline	-3.5471
155148-31-5	Plerixafor 8HCl (AMD3100 8HCl)	-3.5467
110078-46-1	Plerixafor	-3.5467
577778-58-6	Topiroxostat	-3.5459
1231929-97-7	Abemaciclib	-3.5459
6108-05-0	Lidocaine Hydrochloride hydrate	-3.5455
73-78-9	Lidocaine hydrochloride	-3.5455
951395-08-7	tafamidis meglumine	-3.5448
138452-21-8	Fexofenadine hydrochloride	-3.5445
4852-22-6	Proanthocyanidins	-3.5434
14984-68-0	Cloperastine hydrochloride	-3.5431
606143-89-9	Binimetinib	-3.5429
3717-88-2	Flavoxate hydrochloride	-3.5421
64-65-3	Bemegrid	-3.5404
50-33-9	Phenylbutazone	-3.5404
100643-71-8	Desloratadine	-3.5403
51012-33-0	Tiapride hydrochloride	-3.5394
4852-22-6	Proanthocyanidins	-3.5388
74050-98-9	Ketanserin	-3.5388
133099-07-7	Darifenacin HBr	-3.5387
1231930-82-7	LY2835219	-3.5387
1135-23-5	Hydroferulic acid	-3.5386
87-67-2	Choline bitartrate	-3.5377
67-48-1	Choline chloride	-3.5377
951395-08-7	tafamidis meglumine	-3.5375
37517-28-5	Amikacin	-3.5371
107-43-7	Betaine	-3.5366
590-46-5	Betaine hydrochloride	-3.5366

1665-48-1	Metaxalone	-3.5364
4852-22-6	Proanthocyanidins	-3.5362
452-35-7	Ethoxzolamide	-3.5358
52-67-5	d-penicillamin	-3.5357
52-66-4	DL-Penicillamine	-3.5352
35554-44-0	Imazalil	-3.5348
5053-08-7	Fenspiride hydrochloride	-3.5335
4852-22-6	Proanthocyanidins	-3.5332
T4284	(S)-Amlodipine Besylate (103129-82-4(free base))	-3.5327
88150-42-9	Amlodipine	-3.5327
56-85-9	L-Glutamine	-3.5327
91374-20-8	Ropinirole hydrochloride	-3.5315
56839-43-1	Eperisone hydrochloride	-3.5312
4852-22-6	Proanthocyanidins	-3.5311
1113-59-3	3-Bromopyruvic acid	-3.531
99-15-0	Acetylleucine	-3.5309
31282-04-9	Hygromycin B	-3.5308
637-32-1	Proguanil hydrochloride	-3.5308
4852-22-6	Proanthocyanidins	-3.5307
4852-22-6	Proanthocyanidins	-3.5293
52-66-4	DL-Penicillamine	-3.5288
6035-45-6	Folinic Acid Calcium Salt Pentahydrate	-3.5288
74050-98-9	Ketanserin	-3.5285
130-16-5	Dermofongin	-3.5284
14611-52-0	Selegiline hydrochloride	-3.527
13614-98-7	Minocycline hydrochloride	-3.5267
74050-98-9	Ketanserin	-3.5262
3644-61-9	Tolperisone hydrochloride	-3.5259
1254053-43-4	Gilteritinib	-3.5248
52-66-4	DL-Penicillamine	-3.5236
16208-51-8	Dimesna	-3.523
15500-66-0	Pancuronium bromide	-3.523
2030-63-9	Clofazimine	-3.523
530-78-9	Flufenamic acid	-3.5229
491-67-8	Baicalein	-3.5226
64-72-2	Chlortetracycline hydrochloride	-3.5224
951395-08-7	tafamidis meglumine	-3.5224
127-69-5	Sulfisoxazole	-3.5219
4852-22-6	Proanthocyanidins	-3.521
69-65-8	D-Mannitol	-3.5207
1405-41-0	Gentamycin sulfate	-3.5207
56391-57-2	Netilmicin sulfate	-3.5204

546-88-3	Acetohydroxamic acid	-3.5203
4852-22-6	Proanthocyanidins	-3.5201
755038-65-4	Volasertib	-3.5201
306-07-0	Pargyline hydrochloride	-3.5196
555-57-7	Pargyline	-3.5196
65666-07-1	Silymarin	-3.5194
554-57-4	Methazolamide	-3.5191
3759-92-0	Furaltadone hydrochloride	-3.5187
76095-16-4	Enalapril Maleate	-3.5187
13114-29-9	Sodium Demethylcantharidate	-3.5184
306-07-0	Pargyline hydrochloride	-3.518
555-57-7	Pargyline	-3.518
13614-98-7	Minocycline hydrochloride	-3.5178
637-58-1	Pramoxine hydrochloride	-3.5178
936727-05-8	Lumacaftor	-3.5174
547-44-4	Sulfanilylurea	-3.5164
274901-16-5	Vildagliptin	-3.5162
65666-07-1	Silymarin	-3.5154
111025-46-8	Pioglitazone	-3.5153
57248-88-1	Pamidronate disodium salt	-3.5152
1446321-46-5	voxelotor	-3.515
59729-32-7	Citalopram HBr	-3.5145
65666-07-1	Silymarin	-3.5144
70-00-8	Trifluridine	-3.5127
50924-49-7	Mizoribine	-3.5124
951395-08-7	tafamidis meglumine	-3.5116
55981-09-4	Nitazoxanide	-3.5115
53179-09-2	Sisomicin Sulfate	-3.5113
67-20-9	Nitrofurantoin	-3.5103
6078-17-7	Berbamine dihydrochloride	-3.5102
4852-22-6	Proanthocyanidins	-3.51
637-58-1	Pramoxine hydrochloride	-3.51
135729-62-3	Palonosetron hydrochloride	-3.5099
547-44-4	Sulfanilylurea	-3.5081
22839-47-0	Aspartame	-3.5078
118072-93-8	Zoledronic Acid	-3.5077
22373-78-0	Monensin sodium salt	-3.5073
52549-17-4	Pranoprofen	-3.5073
65666-07-1	Silymarin	-3.5072
54556-98-8	Propiverine hydrochloride	-3.5066
1135-24-6	Ferulic Acid	-3.5053
144-83-2	Sulfapyridine	-3.5013

4852-22-6	Proanthocyanidins	-3.4983
1263-89-4	Paromomycin Sulfate	-3.4976
2068-78-2	Vincristine sulfate	-3.4973
26027-38-3	Nonoxynol	-3.4968
125-84-8	Aminoglutethimide	-3.4963
111974-72-2	Quetiapine Fumarate	-3.4962
4852-22-6	Proanthocyanidins	-3.496
111025-46-8	Pioglitazone	-3.4956
53179-09-2	Sisomicin Sulfate	-3.4947
5080-50-2	O-Acetyl-L-carnitine hydrochloride	-3.4947
50-42-0	Adiphenine hydrochloride	-3.4947
74150-27-9	Pimobendan	-3.4945
130693-82-2	Dorzolamide hydrochloride	-3.4944
24729-96-2	Clindamycin phosphate	-3.4943
73-32-5	isoleucine	-3.4942
57808-65-8	Closantel	-3.4939
56391-57-2	Netilmicin sulfate	-3.4938
5080-50-2	O-Acetyl-L-carnitine hydrochloride	-3.4914
364622-82-2	Doripenem Hydrate	-3.4914
4852-22-6	Proanthocyanidins	-3.4911
24280-93-1	Mycophenolic acid	-3.4909
1492-18-8	Calcium folinate	-3.4905
127-71-9	Sulfabenzamide	-3.4905
24868-20-0	Dantrolene sodium hemiheptahydrate	-3.4901
56391-57-2	Netilmicin sulfate	-3.489
50-91-9	Floxuridine	-3.4882
637-58-1	Pramoxine hydrochloride	-3.488
155148-31-5	Plerixafor 8HCl (AMD3100 8HCl)	-3.4878
110078-46-1	Plerixafor	-3.4878
606143-52-6	Selumetinib	-3.4874
4852-22-6	Proanthocyanidins	-3.4873
577778-58-6	Topiroxostat	-3.4873
2030-63-9	Clofazimine	-3.4865
6620-60-6	Proglumide	-3.4865
121268-17-5	Alendronate sodium hydrate	-3.4856
122111-03-9	Gemcitabine HCl	-3.4855
95058-81-4	Gemcitabine	-3.4855
4852-22-6	Proanthocyanidins	-3.4853
59729-32-7	Citalopram HBr	-3.4853
864750-70-9	Revefenacin	-3.4851
380843-75-4	Bosutinib	-3.4849
55981-09-4	Nitazoxanide	-3.4846

1197953-54-0	Brigatinib	-3.484
155148-31-5	Plerixafor 8HCl (AMD3100 8HCl)	-3.4839
110078-46-1	Plerixafor	-3.4839
389139-89-3	Omadacycline	-3.4827
57248-88-1	Pamidronate disodium salt	-3.481
56391-57-2	Netilmicin sulfate	-3.4809
127-07-1	Hydroxyurea	-3.4802
496775-62-3	Eltrombopag Olamine	-3.4781
52-67-5	d-penicillamin	-3.4776
63585-09-1	Foscarnet sodium	-3.4773
87-33-2	Isosorbide dinitrate	-3.4772
872728-81-9	Dabigatran Etexilate Mesylate	-3.4772
211915-06-9	Dabigatran etexilate	-3.4772
61-16-5	Methoxamine hydrochloride	-3.4771
2068-78-2	Vincristine sulfate	-3.4769
127-56-0	Sulfacetamide sodium	-3.4765
6209-17-2	Sulfacetamide sodium salt hydrate	-3.4765
3644-61-9	Tolperisone hydrochloride	-3.4764
2068-78-2	Vincristine sulfate	-3.4752
330808-88-3	Vardenafil hydrochloride trihydrate	-3.4746
59-66-5	Acetazolamide	-3.4746
951395-08-7	tafamidis meglumine	-3.4746
1949-20-8	Oxolamine citrate	-3.4742
1370468-36-2	Elbasvir	-3.4742
668270-12-0	Linagliptin	-3.4741
87-33-2	Isosorbide dinitrate	-3.4737
50-70-4	D-Sorbitol	-3.4732
120011-70-3	Donepezil HCl	-3.473
1135-24-6	Ferulic Acid	-3.4728
2753-45-9	Mebeverine hydrochloride	-3.4721
120014-06-4	Donepezil	-3.4721
16208-51-8	Dimesna	-3.4704
54-30-8	Camylofin	-3.4701
59-66-5	Acetazolamide	-3.4697
64-65-3	Bemegrid	-3.4696
491833-29-5	Eliglustat	-3.4695
51481-61-9	Cimetidine	-3.4692
53716-49-7	Carprofen	-3.4691
1405-37-4	Capastat sulfate	-3.4683
94-07-5	Synephrine	-3.4678
386750-22-7	Desvenlafaxine succinate hydrate	-3.4676

93413-62-8	Desvenlafaxine	-3.4676
3978-86-7	Azatadine Maleate	-3.4674
53179-09-2	Sisomicin Sulfate	-3.4672
4852-22-6	Proanthocyanidins	-3.4671
164650-44-6	Efinaconazole	-3.467
67-20-9	Nitrofurantoin	-3.4667
341-69-5	Orphenadrine hydrochloride	-3.4666
2809-21-4	Etidronate	-3.4664
66-22-8	Uracil	-3.4662
386750-22-7	Desvenlafaxine succinate hydrate	-3.4659
93413-62-8	Desvenlafaxine	-3.4659
120011-70-3	Donepezil HCl	-3.4658
14984-68-0	Cloperastine hydrochloride	-3.4657
389139-89-3	Omadacycline	-3.4657
57-15-8	Chlorobutanol	-3.4654
55-97-0	Hexamethonium Bromide	-3.4652
120014-06-4	Donepezil	-3.4646
25389-94-0	Kanamycin sulfate	-3.464
53179-09-2	Sisomicin Sulfate	-3.464
4852-22-6	Proanthocyanidins	-3.4638
4852-22-6	Proanthocyanidins	-3.4624
6990-06-3	Fusidine	-3.4624
178429-62-4	solriamfetol	-3.4623
21736-83-4	Spectinomycin dihydrochloride	-3.4623
7414-83-7	Sodium etidronate	-3.4619
521-78-8	Trimipramine maleate	-3.4611
24280-93-1	Mycophenolic acid	-3.4611
4852-22-6	Proanthocyanidins	-3.4609
389139-89-3	Omadacycline	-3.4608
64-77-7	Tolbutamide	-3.4601
4852-22-6	Proanthocyanidins	-3.4587
65666-07-1	Silymarin	-3.4587
36330-85-5	Fenbufen	-3.4569
6160-12-9	(-)-Sparteine sulfate pentahydrate	-3.4567
4146-30-9	Neomycin Sulphate B	-3.4565
747-36-4	Hydroxychloroquine sulfate	-3.4565
1231930-82-7	LY2835219	-3.4563
74150-27-9	Pimobendan	-3.4546
13171-25-0	Trimetazidine dihydrochloride	-3.4535
138402-11-6	Irbesartan	-3.4534
15622-65-8	Molindone hydrochloride	-3.4534
89796-99-6	Aceclofenac	-3.4533

16208-51-8	Dimesna	-3.453
65666-07-1	Silymarin	-3.4523
51481-61-9	Cimetidine	-3.452
1405-41-0	Gentamycin sulfate	-3.4518
59263-76-2	Meptazinol hydrochloride	-3.4518
2259-96-3	Cyclothiazide	-3.4516
111902-57-9	Temocapil	-3.4514
386750-22-7	Desvenlafaxine succinate hydrate	-3.4511
93413-62-8	Desvenlafaxine	-3.4511
1405-10-3	Neomycin sulfate	-3.4508
4852-22-6	Proanthocyanidins	-3.4498
24390-14-5	Doxycycline hyclate	-3.4495
4852-22-6	Proanthocyanidins	-3.4488
482-36-0	Hyperoside	-3.4487
76801-85-9	Azathramycin	-3.4483
65666-07-1	Silymarin	-3.4479
53179-09-2	Sisomicin Sulfate	-3.4479
523-87-5	Dimenhydrinate	-3.4478
147-24-0	Diphenhydramine hydrochloride	-3.4478
20537-88-6	Amifostine	-3.4475
4852-22-6	Proanthocyanidins	-3.4467
541-15-1	L(-)-Carnitine	-3.4462
461-06-3	DL-Carnitine	-3.4462
1421373-66-1	Osimertinib mesylate	-3.4462
1421373-65-0	Osimertinib	-3.445
1009119-65-6	Daclatasvir dihydrochloride	-3.4449
127-79-7	Sulfamerazine	-3.4446
738606-46-7	ETC1002	-3.4445
59-66-5	Acetazolamide	-3.4432
3737-09-5	Disopyramide	-3.443
149647-78-9	Vorinostat	-3.443
31282-04-9	Hygromycin B	-3.4415
42461-84-7	Flunixin meglumin	-3.4386
92636-39-0	Cefminox Sodium	-3.4381
67-20-9	Nitrofurantoin	-3.4367
112811-59-3	Gatifloxacin	-3.4367
94-20-2	Chlorpropamide	-3.4366
1009119-65-6	Daclatasvir dihydrochloride	-3.4359
1672-63-5	4-Hydroxyantipyrine	-3.4358
1405-37-4	Capastat sulfate	-3.4357

3978-86-7	Azatadine Maleate	-3.435
3858-89-7	Chloroprocaine HCl	-3.4335
6151-40-2	Quinidine hydrochloride	-3.4335
114977-28-5	Docetaxel	-3.4333
179474-81-8	Prucalopride	-3.4325
3963-95-9	Methacycline hydrochloride	-3.432
32986-56-4	Tobramycin	-3.4319
66357-59-3	Ranitidine Hydrochloride	-3.4302
65666-07-1	Silymarin	-3.4299
738606-46-7	ETC1002	-3.4299
125317-39-7	Vinorelbine Tartrate	-3.4297
321-30-2	Adenine sulfate	-3.4295
73-24-5	Adenine	-3.4295
461-06-3	DL-Carnitine	-3.4281
21462-39-5	Clindamycin HCl	-3.4281
4852-22-6	Proanthocyanidins	-3.4279
6138-79-0	Triprolidine hydrochloride	-3.4275
10017-44-4	L-Carnitine hydrochloride	-3.4274
747-36-4	Hydroxychloroquine sulfate	-3.4273
138452-21-8	Fexofenadine hydrochloride	-3.4259
54556-98-8	Propiverine hydrochloride	-3.4243
155148-31-5	Plerixafor 8HCl (AMD3100 8HCl)	-3.4222
110078-46-1	Plerixafor	-3.4222
1405-37-4	Capastat sulfate	-3.422
95233-18-4	Atovaquone	-3.4216
70-26-8	L-Ornithine	-3.4212
115939-25-8	Salvianolic acid B	-3.4208
84625-61-6	Itraconazole	-3.4207
66357-59-3	Ranitidine Hydrochloride	-3.4203
61-16-5	Methoxamine hydrochloride	-3.4203
132866-11-6	Lercanidipine hydrochloride	-3.4202
541-15-1	L(-)-Carnitine	-3.4201
461-06-3	DL-Carnitine	-3.4201
32986-56-4	Tobramycin	-3.4196
4852-22-6	Proanthocyanidins	-3.4191
57248-88-1	Pamidronate disodium salt	-3.4188
1221573-85-8	Paritaprevir	-3.4187
54-11-5	Nicotine	-3.4186
65-31-6	Nicotine Ditartrate	-3.418
110221-44-8	Temocapril HCl	-3.4174
64-77-7	Tolbutamide	-3.4171
32986-56-4	Tobramycin	-3.4171

10402-53-6	Eprazinone dihydrochloride	-3.417
10163-15-2	Disodium monofluorophosphate	-3.4168
125317-39-7	Vinorelbine Tartrate	-3.4167
111902-57-9	Temocapil	-3.4153
4852-22-6	Proanthocyanidins	-3.415
7414-83-7	Sodium etidronate	-3.4128
144-82-1	Sulfamethizole	-3.4113
100427-26-7	Lercanidipine	-3.4109
64-73-3	Demeclocycline hydrochloride	-3.4108
50-33-9	Phenylbutazone	-3.4106
117772-70-0	Azithromycin dihydrate	-3.4102
147816-24-8	Cefcapene Pivoxil Hydrochloride	-3.41
64-72-2	Chlortetracycline hydrochloride	-3.4099
65666-07-1	Silymarin	-3.4095
61-16-5	Methoxamine hydrochloride	-3.4093
10402-53-6	Eprazinone dihydrochloride	-3.4091
63585-09-1	Foscarnet sodium	-3.4086
32854-75-4	Lappaconitine	-3.4084
6078-17-7	Berbamine dihydrochloride	-3.4074
2809-21-4	Etidronate	-3.4059
57808-65-8	Closantel	-3.4057
1405-37-4	Capastat sulfate	-3.4057
91-85-0	Thonzylamine	-3.4057
1135-23-5	Hydroferulic acid	-3.4046
1672-63-5	4-Hydroxyantipyrine	-3.404
95233-18-4	Atovaquone	-3.4039
144675-97-8	Pixantrone dimaleate	-3.4029
4330-99-8	Trimeprazine tartrate	-3.4027
1135-23-5	Hydroferulic acid	-3.4025
3963-95-9	Methacycline hydrochloride	-3.4023
2030-63-9	Clofazimine	-3.4016
13710-19-5	Tolfenamic Acid	-3.4015
65666-07-1	Silymarin	-3.3999
111902-57-9	Temocapil	-3.3994
79-57-2	Oxytetracycline	-3.3992
107-35-7	Taurine	-3.3986
92636-39-0	Cefminox Sodium	-3.3986
24868-20-0	Dantrolene sodium hemiheptahydrate	-3.3975
32986-56-4	Tobramycin	-3.3973
168555-66-6	Fosbretabulin Disodium	-3.3972
22839-47-0	Aspartame	-3.3967
65666-07-1	Silymarin	-3.3965
61-16-5	Methoxamine hydrochloride	-3.3963

63585-09-1	Foscarnet sodium	-3.3962
64439-81-2	Hydroxy Camptothecine	-3.396
69-09-0	Chlorpromazine hydrochloride	-3.3958
951395-08-7	tafamidis meglumine	-3.395
520-36-5	Apigenin	-3.3944
141505-33-1	Levosimendan	-3.3944
118072-93-8	Zoledronic Acid	-3.3941
1257044-40-8	ABT199	-3.3939
3644-61-9	Tolperisone hydrochloride	-3.3933
65666-07-1	Silymarin	-3.3929
31282-04-9	Hygromycin B	-3.3929
89396-94-1	Imidapril HCl	-3.3918
3963-95-9	Methacycline hydrochloride	-3.3917
56-85-9	L-Glutamine	-3.3911
73816-42-9	Meclocycline sulfosalicylate salt	-3.391
50-91-9	Floxuridine	-3.3908
17321-77-6	Clomipramine hydrochloride	-3.3906
155148-31-5	Plerixafor 8HCl (AMD3100 8HCl)	-3.3903
110078-46-1	Plerixafor	-3.3903
70775-75-6	Octenidine Dihydrochloride	-3.3903
74150-27-9	Pimobendan	-3.39
804-30-8	Fursultiamine	-3.3893
22373-78-0	Monensin sodium salt	-3.3889
178429-62-4	solriamfetol	-3.3886
54-30-8	Camylofin	-3.3885
550-24-3	Embelin	-3.3881
570406-98-3	Avatrombopag	-3.3876
138452-21-8	Fexofenadine hydrochloride	-3.3876
6160-12-9	(-)-Sparteine sulfate pentahydrate	-3.387
53179-09-2	Sisomicin Sulfate	-3.387
894-71-3	Nortriptyline hydrochloride	-3.387
645-43-2	Guanethidine Monosulfate	-3.3862
15676-16-1	Sulpiride	-3.3857
65666-07-1	Silymarin	-3.3852
6170-42-9	Chloropyramine hydrochloride	-3.3846
12650-69-0	Mupirocin	-3.3843
443913-73-3	Vandetanib	-3.3839
118072-93-8	Zoledronic Acid	-3.3838
52-24-4	Triethylenethiophosphoramide	-3.3837
1218779-75-9	Apatinib mesylate	-3.3826
811803-05-1	Apatinib	-3.3826

114977-28-5	Docetaxel	-3.382
61718-82-9	Fluvoxamine maleate	-3.3811
577778-58-6	Topiroxostat	-3.3806
6035-45-6	Folinic Acid Calcium Salt Pentahydrate	-3.3805
29608-49-9	Almitrine dimesylate	-3.3802
4852-22-6	Proanthocyanidins	-3.3783
84294-96-2	Enoxacin Sesquihydrate	-3.3782
6064-83-1	Fosfosal	-3.3781
24280-93-1	Mycophenolic acid	-3.3775
74011-58-8	Enoxacin	-3.3773
570406-98-3	Avatrombopag	-3.3773
624-49-7	Dimethyl fumarate	-3.377
10040-45-6	Sodium Picosulfate	-3.3767
389139-89-3	Omadacycline	-3.3756
136790-76-6	Lubiprostone	-3.3756
1435-55-8	Hydroquinidine	-3.3755
50-42-0	Adiphenine hydrochloride	-3.3752
64-73-3	Demeclocycline hydrochloride	-3.3751
550-24-3	Embelin	-3.375
84625-61-6	Itraconazole	-3.3746
1949-20-8	Oxolamine citrate	-3.3745
127-71-9	Sulfabenzamide	-3.3742
15500-66-0	Pancuronium bromide	-3.3732
5053-08-7	Fenspiride hydrochloride	-3.3732
4852-22-6	Proanthocyanidins	-3.372
4852-22-6	Proanthocyanidins	-3.3717
1263-89-4	Paromomycin Sulfate	-3.3716
111902-57-9	Temocapil	-3.3713
145040-37-5	Candesartan Cilexetil	-3.3704
51940-44-4	Pipemidic acid	-3.3696
57-37-4	Benactyzine hydrochloride	-3.3695
50-42-0	Adiphenine hydrochloride	-3.3694
121808-62-6	Pidotimod	-3.3693
486460-32-6	Sitagliptin	-3.3692
13614-98-7	Minocycline hydrochloride	-3.369
4852-22-6	Proanthocyanidins	-3.3688
105826-92-4	Tropisetron hydrochloride	-3.3687
1986-47-6	Tranylcypromine (2-PCPA) hydrochloride	-3.3683
69-09-0	Chlorpromazine hydrochloride	-3.3674
496775-62-3	Eltrombopag Olamine	-3.3661
182349-12-8	Rupatadine Fumarate	-3.366
53179-09-2	Sisomicin Sulfate	-3.3658
530-78-9	Flufenamic acid	-3.3653

61-16-5	Methoxamine hydrochloride	-3.3647
80433-71-2	Calcium Levofolinate	-3.3641
1221573-85-8	Paritaprevir	-3.3639
152459-95-5	Imatinib	-3.3627
220127-57-1	Imatinib Mesylate	-3.3627
121-33-5	Vanillin	-3.3626
87-67-2	Choline bitartrate	-3.3624
67-48-1	Choline chloride	-3.3624
65666-07-1	Silymarin	-3.3621
548-66-3	Drofenine hydrochloride	-3.3621
23979-41-1	(R)-Naproxen	-3.3618
142-47-2	L-Glutamic acid monosodium salt	-3.3616
56-86-0	glutamic acid	-3.3616
2259-96-3	Cyclothiazide	-3.3609
3963-95-9	Methacycline hydrochloride	-3.3608
61-16-5	Methoxamine hydrochloride	-3.36
78246-49-8	Paroxetine hydrochloride	-3.3599
50-41-9	Clomifene citrate	-3.3599
119478-55-6	Danofloxacin mesylate	-3.3592
4852-22-6	Proanthocyanidins	-3.3591
76095-16-4	Enalapril Maleate	-3.3591
27848-84-6	Nicergoline	-3.3582
15622-65-8	Molindone hydrochloride	-3.3579
1113-59-3	3-Bromopyruvic acid	-3.3571
520-36-5	Apigenin	-3.3569
79-57-2	Oxytetracycline	-3.3559
3505-38-2	Carbinoxamine Maleate Salt	-3.3559
64019-93-8	Dipivefrin hydrochloride	-3.3545
53179-09-2	Sisomicin Sulfate	-3.3538
110221-44-8	Temocapril HCl	-3.3533
167465-36-3	Zosuquidar 3HCl	-3.3531
111902-57-9	Temocapil	-3.3524
59-66-5	Acetazolamide	-3.3521
182431-12-5	Lomitapide	-3.3517
64439-81-2	Hydroxy Camptothecine	-3.3513
155148-31-5	Plerixafor 8HCl (AMD3100 8HCl)	-3.3512
110078-46-1	Plerixafor	-3.3512
125317-39-7	Vinorelbine Tartrate	-3.351
24390-14-5	Doxycycline hyclate	-3.3492
20537-88-6	Amifostine	-3.3489
29868-97-1	Pirenzepine dihydrochloride	-3.3489
872728-81-9	Dabigatran Etexilate Mesylate	-3.3488

211915-06-9	Dabigatran etexilate	-3.3488
5987-82-6	Oxybuprocaine hydrochloride	-3.3487
27833-64-3	Loxapine succinate salt	-3.3487
122647-32-9	Ibutilide Fumarate	-3.3481
443913-73-3	Vandetanib	-3.3475
167465-36-3	Zosuquidar 3HCl	-3.3475
3963-95-9	Methacycline hydrochloride	-3.3466
61825-94-3	Oxaliplatin	-3.3461
317-34-0	Aminophylline	-3.346
951395-08-7	tafamidis meglumine	-3.346
469-77-2	Spiculisporic Acid	-3.3457
749-02-0	Spiperone	-3.3448
51940-44-4	Pipemidic acid	-3.3446
951395-08-7	tafamidis meglumine	-3.344
59263-76-2	Meptazinol hydrochloride	-3.344
843663-66-1	Bedaquiline	-3.3438
1435-55-8	Hydroquinidine	-3.3436
4852-22-6	Proanthocyanidins	-3.3424
55-97-0	Hexamethonium Bromide	-3.3421
17696-69-4	Hematoporphyrin dihydrochloride	-3.342
136434-34-9	Duloxetine hydrochloride	-3.3414
89-25-8	Edaravone	-3.3412
1231929-97-7	Abemaciclib	-3.3401
118072-93-8	Zoledronic Acid	-3.3395
66357-59-3	Ranitidine Hydrochloride	-3.3393
1476-53-5	Novobiocin Sodium	-3.3391
119478-55-6	Danofloxacin mesylate	-3.339
3963-95-9	Methacycline hydrochloride	-3.339
4852-22-6	Proanthocyanidins	-3.3388
329-63-5	DL-Adrenaline Hydrochloride	-3.3383
120011-70-3	Donepezil HCl	-3.3379
152-95-4	Sophoricoside	-3.3378
76095-16-4	Enalapril Maleate	-3.3372
1230487-00-9	Siponimod	-3.3364
113-92-8	Chlorpheniramine maleate	-3.3364
31282-04-9	Hygromycin B	-3.3364
6064-83-1	Fosfosal	-3.3359
51-21-8	Fluorouracil	-3.3354
120014-06-4	Donepezil	-3.3352
155148-31-5	Plerixafor 8HCl (AMD3100 8HCl)	-3.335
110078-46-1	Plerixafor	-3.335

65666-07-1	Silymarin	-3.335
4360-12-7	Ajmaline	-3.3348
94-20-2	Chlorpropamide	-3.3344
182431-12-5	Lomitapide	-3.3329
99-15-0	Acetylleucine	-3.3324
76824-35-6	Famotidine	-3.3309
1953-02-2	Tiopronin	-3.3308
317-34-0	Aminophylline	-3.3306
144675-97-8	Pixantrone dimaleate	-3.3303
706782-28-7	Pimavanserin tartrate	-3.3297
706779-91-1	Pimavanserin	-3.3297
1009119-65-6	Daclatasvir dihydrochloride	-3.3294
65666-07-1	Silymarin	-3.3288
827022-33-3	Palbociclib Isethionate	-3.3287
827022-32-2	Palbociclib hydrochloride	-3.3287
571190-30-2	Palbociclib	-3.3284
1231929-97-7	Abemaciclib	-3.3282
23142-01-0	Carbetapentane citrate	-3.3282
482-36-0	Hyperoside	-3.3269
59729-32-7	Citalopram HBr	-3.3268
94-20-2	Chlorpropamide	-3.3253
148849-67-6	Ivabradine hydrochloride	-3.3252
1009119-64-5	Daclatasvir	-3.3252
6064-83-1	Fosfosal	-3.3251
59263-76-2	Meptazinol hydrochloride	-3.3249
4852-22-6	Proanthocyanidins	-3.3244
1098-97-1	Pyrithioxin	-3.3242
118072-93-8	Zoledronic Acid	-3.3237
125317-39-7	Vinorelbine Tartrate	-3.3234
99291-25-5	Levodropropizine	-3.3231
2022-85-7	Fluorocytosine	-3.3226
2259-96-3	Cyclothiazide	-3.322
547-44-4	Sulfanilylurea	-3.3218
303-25-3	Cyclizine hydrochloride	-3.3217
155148-31-5	Plerixafor 8HCl (AMD3100 8HCl)	-3.3216
110078-46-1	Plerixafor	-3.3216
1025504-45-3	Valbenazine	-3.3213
36330-85-5	Fenbufen	-3.3201
4852-22-6	Proanthocyanidins	-3.3199
67656-30-8	10-Hydroxycampothecin	-3.3192

4852-22-6	Proanthocyanidins	-3.3192
113-92-8	Chlorpheniramine maleate	-3.3184
51-24-1	Tiratricol	-3.3181
6078-17-7	Berbamine dihydrochloride	-3.3174
17696-69-4	Hematoporphyrin dihydrochloride	-3.3172
24729-96-2	Clindamycin phosphate	-3.3157
491-67-8	Baicalein	-3.3146
1254053-43-4	Gilteritinib	-3.3141
1113-59-3	3-Bromopyruvic acid	-3.3139
4146-30-9	Neomycin Sulphate B	-3.3128
19767-45-4	Mesna	-3.3124
1611493-60-7	Bictegravir	-3.3117
133099-07-7	Darifenacin HBr	-3.3106
122111-03-9	Gemcitabine HCl	-3.3106
95058-81-4	Gemcitabine	-3.3106
6151-40-2	Quinidine hydrochloride	-3.3104
864750-70-9	Revefenacin	-3.3101
552-41-0	Paeonol	-3.3095
3759-92-0	Furaltadone hydrochloride	-3.309
4852-22-6	Proanthocyanidins	-3.3087
52549-17-4	Pranoprofen	-3.3079
21462-39-5	Clindamycin HCl	-3.3076
14984-68-0	Cloperastine hydrochloride	-3.3076
53179-09-2	Sisomicin Sulfate	-3.3073
80433-71-2	Calcium Levofolinate	-3.3071
120014-06-4	Donepezil	-3.3065
120011-70-3	Donepezil HCl	-3.3062
141-90-2	Deracil	-3.3061
521-78-8	Trimipramine maleate	-3.3059
464-92-6	Asiatic acid	-3.3055
36330-85-5	Fenbufen	-3.3049
66357-59-3	Ranitidine Hydrochloride	-3.3047
1263-89-4	Paromomycin Sulfate	-3.3042
452-35-7	Ethoxzolamide	-3.3033
53179-09-2	Sisomicin Sulfate	-3.3021
1263-89-4	Paromomycin Sulfate	-3.3017
20537-88-6	Amifostine	-3.3016
943-17-9	Etilefrine hydrochloride	-3.3013
74150-27-9	Pimobendan	-3.3006
923604-59-5	Simeprevir	-3.3
137975-06-5	Mozavaptan	-3.2996

3737-09-5	Disopyramide	-3.2996
1254053-43-4	Gilteritinib	-3.2996
3644-61-9	Tolperisone hydrochloride	-3.2991
148849-67-6	Ivabradine hydrochloride	-3.2986
76824-35-6	Famotidine	-3.2984
317-34-0	Aminophylline	-3.2971
4852-22-6	Proanthocyanidins	-3.2966
50-55-5	Reserpine	-3.2958
10402-53-6	Eprazinone dihydrochloride	-3.2954
951395-08-7	tafamidis meglumine	-3.2952
54556-98-8	Propiverine hydrochloride	-3.295
6620-60-6	Proglumide	-3.2939
61-16-5	Methoxamine hydrochloride	-3.2935
1420477-60-6	Acalabrutinib	-3.2931
61-68-7	Mefenamic acid	-3.2929
65666-07-1	Silymarin	-3.2927
61-12-1	Dibucaine hydrochloride	-3.2926
85-79-0	Dibucaine	-3.2926
201530-41-8	Deferasirox	-3.2923
54-30-8	Camylofin	-3.2921
574-84-5	Fraxetin	-3.2919
130693-82-2	Dorzolamide hydrochloride	-3.2902
99291-25-5	Levodropropizine	-3.2901
59-66-5	Acetazolamide	-3.29
61-16-5	Methoxamine hydrochloride	-3.2895
329-63-5	DL-Adrenaline Hydrochloride	-3.2893
5370-01-4	Mexiletine hydrochloride	-3.2893
183204-72-0	Tipiracil hydrochloride	-3.2892
118072-93-8	Zoledronic Acid	-3.2892
83435-67-0	Delapril?Hydrochloride	-3.2878
611-75-6	Bromhexine hydrochloride	-3.2876
152459-95-5	Imatinib	-3.2876
220127-57-1	Imatinib Mesylate	-3.2876
6138-79-0	Triprolidine hydrochloride	-3.2874
364622-82-2	Doripenem Hydrate	-3.2867
1009119-64-5	Daclatasvir	-3.2865
116-38-1	Edrophonium chloride	-3.2863
4852-22-6	Proanthocyanidins	-3.2863
147536-97-8	Bosentan	-3.2861
66357-59-3	Ranitidine Hydrochloride	-3.286

4852-22-6	Proanthocyanidins	-3.2846
469-77-2	Spiculisporic Acid	-3.2846
1009119-65-6	Daclatasvir dihydrochloride	-3.2844
149676-40-4	Pefloxacin Mesylate Dihydrate	-3.2843
76824-35-6	Famotidine	-3.2843
149647-78-9	Vorinostat	-3.2831
70458-95-6	Pefloxacin Mesylate	-3.2831
1009119-64-5	Daclatasvir	-3.282
182349-12-8	Rupatadine Fumarate	-3.2815
611-75-6	Bromhexine hydrochloride	-3.2815
56-84-8	L-Aspartic acid	-3.2813
22204-53-1	Naproxen	-3.2811
54965-21-8	Albendazole	-3.2808
74150-27-9	Pimobendan	-3.2805
523-87-5	Dimenhydrinate	-3.2797
147-24-0	Diphenhydramine hydrochloride	-3.2794
87333-19-5	Ramipril	-3.2792
116-38-1	Edrophonium chloride	-3.2785
1025687-58-4	R788 Disodium	-3.2774
1132935-63-7	Dasabuvir	-3.2767
85721-33-1	Ciprofloxacin	-3.2766
93107-08-5	Ciprofloxacin hydrochloride	-3.2766
1009119-65-6	Daclatasvir dihydrochloride	-3.2763
1354744-91-4	Naloxegol oxalate	-3.276
878672-00-5	Lesinurad	-3.2744
4146-30-9	Neomycin Sulphate B	-3.2742
125317-39-7	Vinorelbine Tartrate	-3.2721
27591-69-1	Tilorone dihydrochloride	-3.2721
108612-45-9	Mizolastine	-3.2716
61-16-5	Methoxamine hydrochloride	-3.2712
544-62-7	Batyl alcohol	-3.2712
3963-95-9	Methacycline hydrochloride	-3.271
137862-53-4	Valsartan	-3.2701
4330-99-8	Trimeprazine tartrate	-3.2689
544-62-7	Batyl alcohol	-3.2689
611-75-6	Bromhexine hydrochloride	-3.2681
23142-01-0	Carbetapentane citrate	-3.2677
37517-28-5	Amikacin	-3.2673

54-30-8	Camylofin	-3.2652
60-31-1	Acetylcholine chloride	-3.2651
552-41-0	Paeonol	-3.2648
27741-01-1	Geniposidic Acid	-3.2643
57644-54-9	Gastrodenol	-3.2642
69-65-8	D-Mannitol	-3.264
1009119-64-5	Daclatasvir	-3.2638
61-16-5	Methoxamine hydrochloride	-3.2636
4852-22-6	Proanthocyanidins	-3.2635
55079-83-9	Acitretin	-3.2633
155148-31-5	Plerixafor 8HCl (AMD3100 8HCl)	-3.2631
110078-46-1	Plerixafor	-3.2631
1986-47-6	Tranylcypromine (2-PCPA) hydrochloride	-3.263
723-46-6	Sulfamethoxazole	-3.2621
723-46-6	Sulfamethoxazole	-3.2617
4852-22-6	Proanthocyanidins	-3.2614
1256580-46-7	Alectinib	-3.2602
4852-22-6	Proanthocyanidins	-3.26
1231929-97-7	Abemaciclib	-3.2596
747-36-4	Hydroxychloroquine sulfate	-3.2593
4852-22-6	Proanthocyanidins	-3.259
5451-09-2	5-Aminolevulinic acid hydrochloride	-3.2588
66532-86-3	Propacetamol Hydrochloride	-3.2578
134308-13-7	Tolcapone	-3.2574
6035-45-6	Folinic Acid Calcium Salt Pentahydrate	-3.2569
56839-43-1	Eperisone hydrochloride	-3.2567
144-83-2	Sulfapyridine	-3.2565
5104-49-4	Flurbiprofen	-3.2562
639089-54-6	Tozasertib	-3.2562
51773-92-3	Mefloquine hydrochloride	-3.2561
206658-92-6	Tacrine hydrochloride hydrate	-3.2561
61-12-1	Dibucaine hydrochloride	-3.256
85-79-0	Dibucaine	-3.256
52-90-4	cysteine	-3.2559
70797-11-4	Cefpiramide acid	-3.2557
65666-07-1	Silymarin	-3.2557
28395-03-1	Bumetanide	-3.2553
99-15-0	Acetylleucine	-3.255
298-57-7	Stugeron	-3.255
125317-39-7	Vinorelbine Tartrate	-3.2546

73816-42-9	Meclocycline sulfosalicylate salt	-3.2543
2295-58-1	Flopropione	-3.254
125317-39-7	Vinorelbine Tartrate	-3.2536
116-38-1	Edrophonium chloride	-3.2532
94-07-5	Synephrine	-3.2526
54-30-8	Camylofin	-3.2524
872728-81-9	Dabigatran Etexilate Mesylate	-3.2522
211915-06-9	Dabigatran etexilate	-3.2522
83-44-3	Deoxycholic acid	-3.2515
149-91-7	Gallic acid	-3.2511
13614-98-7	Minocycline hydrochloride	-3.251
6078-17-7	Berbamine dihydrochloride	-3.2506
681492-22-8	Delamanid	-3.2504
122647-32-9	Ibutilide Fumarate	-3.2502
951395-08-7	tafamidis meglumine	-3.2501
571190-30-2	Palbociclib	-3.2496
61-16-5	Methoxamine hydrochloride	-3.2494
51481-61-9	Cimetidine	-3.2494
87848-99-5	Acrivastine	-3.2482
624-49-7	Dimethyl fumarate	-3.2476
4852-22-6	Proanthocyanidins	-3.2475
827022-33-3	Palbociclib Isethionate	-3.2475
827022-32-2	Palbociclib hydrochloride	-3.2475
27741-01-1	Geniposidic Acid	-3.2471
55-97-0	Hexamethonium Bromide	-3.2469
54-11-5	Nicotine	-3.2469
4852-22-6	Proanthocyanidins	-3.2468
153-98-0	Serotonin hydrochloride	-3.2465
151533-22-1	Levomefolate calcium	-3.2464
65-31-6	Nicotine Ditartrate	-3.2448
59263-76-2	Meptazinol hydrochloride	-3.2444
155148-31-5	Plerixafor 8HCl (AMD3100 8HCl)	-3.2443
110078-46-1	Plerixafor	-3.2443
126784-99-4	Ulipristal acetate	-3.2436
943-17-9	Etilefrine hydrochloride	-3.2434
330808-88-3	Vardenafil hydrochloride trihydrate	-3.242
1098-60-8	Triflupromazine hydrochloride	-3.2414
53797-35-6	Ribostamycin sulfate	-3.2412
1949-20-8	Oxolamine citrate	-3.241
474-25-9	Chenodeoxycholic acid	-3.2401
6160-12-9	(-)-Sparteine sulfate pentahydrate	-3.24
121-33-5	Vanillin	-3.2397
57-96-5	Sulfinpyrazone	-3.2394

473-98-3	Betulin	-3.2389
54-30-8	Camylofin	-3.2376
74711-43-6	Zaltoprofen	-3.2365
65666-07-1	Silymarin	-3.2362
57-67-0	Sulfaguanidine	-3.2358
15307-86-5	Diclofenac	-3.2356
452-35-7	Ethoxzolamide	-3.2354
32986-56-4	Tobramycin	-3.2351
4852-22-6	Proanthocyanidins	-3.2347
78213-16-8	Diclofenac diethylamine	-3.2344
57248-88-1	Pamidronate disodium salt	-3.2339
3644-61-9	Tolperisone hydrochloride	-3.2334
1226781-44-7	Omarigliptin	-3.2325
4852-22-6	Proanthocyanidins	-3.2324
64-77-7	Tolbutamide	-3.2319
220620-09-7	Tigecycline	-3.2316
1665-48-1	Metaxalone	-3.2303
364-98-7	Diazoxide	-3.2302
53179-09-2	Sisomicin Sulfate	-3.2294
32986-56-4	Tobramycin	-3.2293
4146-30-9	Neomycin Sulphate B	-3.2291
65666-07-1	Silymarin	-3.2276
2062-78-4	Pimozide	-3.2276
29608-49-9	Almitrine dimesylate	-3.2275
29216-28-2	Mequitazine	-3.2271
56391-57-2	Netilmicin sulfate	-3.2263
321-30-2	Adenine sulfate	-3.2262
73-24-5	Adenine	-3.2262
965-52-6	Nifuroxazide	-3.2259
439081-18-2	Afatinib	-3.2259
135729-62-3	Palonosetron hydrochloride	-3.2255
951395-08-7	tafamidis meglumine	-3.2252
155148-31-5	Plerixafor 8HCl (AMD3100 8HCl)	-3.2252
110078-46-1	Plerixafor	-3.2252
364622-82-2	Doripenem Hydrate	-3.2244
329-63-5	DL-Adrenaline Hydrochloride	-3.2243
74011-58-8	Enoxacin	-3.2236
84294-96-2	Enoxacin Sesquihydrate	-3.2236
3505-38-2	Carbinoxamine Maleate Salt	-3.2235
96829-58-2	Orlistat	-3.2234
113-92-8	Chlorpheniramine maleate	-3.2229
521-78-8	Trimipramine maleate	-3.2226

5370-01-4	Mexiletine hydrochloride	-3.2223
527-07-1	Sodium gluconate	-3.2218
299-28-5	Calcium gluconate	-3.2218
389139-89-3	Omadacycline	-3.2218
76801-85-9	Azathramycin	-3.2217
5053-08-7	Fenspiride hydrochloride	-3.2213
95635-55-5	Ranolazine	-3.2203
95635-56-6	Ranolazine dihydrochloride	-3.2203
4852-22-6	Proanthocyanidins	-3.2202
1197953-54-0	Brigatinib	-3.2198
51012-33-0	Tiapride hydrochloride	-3.2195
850140-73-7	Afatinib Dimaleate	-3.219
66532-86-3	Propacetamol Hydrochloride	-3.2188
4852-22-6	Proanthocyanidins	-3.2184
2259-96-3	Cyclothiazide	-3.2182
544-62-7	Batyl alcohol	-3.2179
70-26-8	L-Ornithine	-3.2178
550-24-3	Embelin	-3.2172
89-25-8	Edaravone	-3.2167
122647-32-9	Ibutilide Fumarate	-3.216
1476-53-5	Novobiocin Sodium	-3.2155
1009119-64-5	Daclatasvir	-3.2152
66357-59-3	Ranitidine Hydrochloride	-3.2151
4852-22-6	Proanthocyanidins	-3.2149
491-67-8	Baicalein	-3.2149
305-03-3	Chloroambucil	-3.2146
113-92-8	Chlorpheniramine maleate	-3.2144
1405-41-0	Gentamycin sulfate	-3.214
94-07-5	Synephrine	-3.2137
439081-18-2	Afatinib	-3.2134
590-46-5	Betaine hydrochloride	-3.2133
125317-39-7	Vinorelbine Tartrate	-3.2131
123948-87-8	Topotecan	-3.2126
1354744-91-4	Naloxegol oxalate	-3.2126
2809-21-4	Etidronate	-3.2121
50924-49-7	Mizoribine	-3.2119
7414-83-7	Sodium etidronate	-3.2113
107-43-7	Betaine	-3.2112
125317-39-7	Vinorelbine Tartrate	-3.2111
87233-61-2	Emedastine	-3.2105

7414-83-7	Sodium etidronate	-3.21
155148-31-5	Plerixafor 8HCl (AMD3100 8HCl)	-3.2097
110078-46-1	Plerixafor	-3.2097
224452-66-8	Retapamulin	-3.2095
755038-65-4	Volasertib	-3.2092
2259-96-3	Cyclothiazide	-3.2089
29868-97-1	Pirenzepine dihydrochloride	-3.2088
850140-73-7	Afatinib Dimaleate	-3.208
2809-21-4	Etidronate	-3.2077
1009119-65-6	Daclatasvir dihydrochloride	-3.2073
434-07-1	Oxymetholone	-3.2061
125317-39-7	Vinorelbine Tartrate	-3.2059
6035-45-6	Folinic Acid Calcium Salt Pentahydrate	-3.2056
125317-39-7	Vinorelbine Tartrate	-3.2045
2062-78-4	Pimozide	-3.2043
19767-45-4	Mesna	-3.204
79-57-2	Oxytetracycline	-3.2034
70-26-8	L-Ornithine	-3.2032
66357-59-3	Ranitidine Hydrochloride	-3.2028
546-88-3	Acetohydroxamic acid	-3.2028
330808-88-3	Vardenafil hydrochloride trihydrate	-3.2026
73816-42-9	Meclocycline sulfosalicylate salt	-3.2023
4146-30-9	Neomycin Sulphate B	-3.2023
112811-59-3	Gatifloxacin	-3.202
201530-41-8	Deferasirox	-3.202
63-68-3	L-Methionine	-3.202
473-98-3	Betulin	-3.2016
24729-96-2	Clindamycin phosphate	-3.2015
110871-86-8	Sparfloxacin	-3.2013
459789-99-2	Obeticholic Acid	-3.2009
681492-22-8	Delamanid	-3.2003
2259-96-3	Cyclothiazide	-3.1998
121268-17-5	Alendronate sodium hydrate	-3.1993
65-31-6	Nicotine Ditartrate	-3.1992
54-11-5	Nicotine	-3.1983
57248-88-1	Pamidronate disodium salt	-3.1968
755038-65-4	Volasertib	-3.1967
10040-45-6	Sodium Picosulfate	-3.1957
4852-22-6	Proanthocyanidins	-3.1951
305-03-3	Chloroambucil	-3.1948
74150-27-9	Pimobendan	-3.1948
106685-40-9	Adapalene	-3.193

1611493-60-7	Bictegravir	-3.1929
128-13-2	Ursodeoxycholic acid	-3.1926
923604-59-5	Simeprevir	-3.1924
70476-82-3	Mitoxantrone hydrochloride	-3.1916
496775-61-2	Eltrombopag	-3.1907
872728-81-9	Dabigatran Etexilate Mesylate	-3.1906
211915-06-9	Dabigatran etexilate	-3.1906
4852-22-6	Proanthocyanidins	-3.1904
639089-54-6	Tozasertib	-3.1903
60-93-5	Quinine dihydrochloride	-3.1902
130-95-0	Quinine	-3.1902
65666-07-1	Silymarin	-3.1894
65271-80-9	Mitoxantrone	-3.188
118072-93-8	Zoledronic Acid	-3.188
936727-05-8	Lumacaftor	-3.1876
77-67-8	Ethosuximide	-3.1872
64-73-3	Demeclocycline hydrochloride	-3.1867
30299-08-2	Clinofibrate	-3.1866
303-25-3	Cyclizine hydrochloride	-3.1865
25389-94-0	Kanamycin sulfate	-3.1858
87848-99-5	Acrivastine	-3.1852
574-84-5	Fraxetin	-3.1848
2022-85-7	Fluorocytosine	-3.1845
4852-22-6	Proanthocyanidins	-3.1844
52-90-4	cysteine	-3.1832
64-73-3	Demeclocycline hydrochloride	-3.1828
53716-49-7	Carprofen	-3.1825
220620-09-7	Tigecycline	-3.1824
53797-35-6	Ribostamycin sulfate	-3.1817
548-66-3	Drofenine hydrochloride	-3.1816
147536-97-8	Bosentan	-3.1816
1197953-54-0	Brigatinib	-3.1815
548-66-3	Drofenine hydrochloride	-3.1807
4852-22-6	Proanthocyanidins	-3.1789
1098-97-1	Pyrithioxin	-3.1784
965-52-6	Nifuroxazide	-3.1784
6078-17-7	Berbamine dihydrochloride	-3.1769
88150-42-9	Amlodipine	-3.1757
872728-81-9	Dabigatran Etexilate Mesylate	-3.1756
211915-06-9	Dabigatran etexilate	-3.1756
980-71-2	Brompheniramine hydrogen maleate	-3.1755

81919-14-4	Bendazac L-lysine	-3.1753
22161-81-5	(S)-(+)-Ketoprofen	-3.1752
22071-15-4	Ketoprofen	-3.1752
67-43-6	Pentetic Acid	-3.1752
87-99-0	Xylitol	-3.1751
554-57-4	Methazolamide	-3.1749
58-28-6	Desipramine hydrochloride	-3.1746
58-85-5	D-Biotin	-3.1745
4852-22-6	Proanthocyanidins	-3.1741
152-95-4	Sophoricoside	-3.1739
65666-07-1	Silymarin	-3.1735
57644-54-9	Gastrodenol	-3.1721
77-92-9	Citric acid	-3.172
5104-49-4	Flurbiprofen	-3.1714
111902-57-9	Temocapil	-3.1713
6620-60-6	Proglumide	-3.1709
3679-64-9	Salifungin	-3.1701
52-24-4	Triethylenethiophosphoramide	-3.1701
29608-49-9	Almitrine dimesylate	-3.1701
4852-22-6	Proanthocyanidins	-3.1698
496775-61-2	Eltrombopag	-3.1693
3963-95-9	Methacycline hydrochloride	-3.1691
645-43-2	Guanethidine Monosulfate	-3.169
330808-88-3	Vardenafil hydrochloride trihydrate	-3.1681
50-33-9	Phenylbutazone	-3.168
443913-73-3	Vandetanib	-3.1666
552-94-3	Sasapyrine	-3.1661
74150-27-9	Pimobendan	-3.1652
118072-93-8	Zoledronic Acid	-3.1644
122647-32-9	Ibutilide Fumarate	-3.1642
872728-81-9	Dabigatran Etexilate Mesylate	-3.1642
211915-06-9	Dabigatran etexilate	-3.1642
1405-37-4	Capastat sulfate	-3.1641
329-63-5	DL-Adrenaline Hydrochloride	-3.1629
943-17-9	Etilefrine hydrochloride	-3.1629
87848-99-5	Acrivastine	-3.1626
1070-11-7	Ethambutol dihydrochloride	-3.1626
73816-42-9	Meclocycline sulfosalicylate salt	-3.1625
5934-29-2	L-Histidine monohydrochloride monohydrat	-3.1622
71-00-1	histidine	-3.1622
552-41-0	Paeonol	-3.1622
872728-81-9	Dabigatran Etexilate Mesylate	-3.1622
211915-06-9	Dabigatran etexilate	-3.1622

1953-02-2	Tiopronin	-3.162
99-15-0	Acetylleucine	-3.1618
50-33-9	Phenylbutazone	-3.1616
81-25-4	Cholic Acid	-3.1601
681492-22-8	Delamanid	-3.1598
10540-29-1	Tamoxifen	-3.1596
54965-24-1	Tamoxifen Z-isomer citrate	-3.1596
550-24-3	Embelin	-3.1589
1642-54-2	Diethylcarbamazine citrate	-3.1571
91-85-0	Thonzylamine	-3.1569
4852-22-6	Proanthocyanidins	-3.1569
86541-74-4	Benazepril hydrochloride	-3.1565
50-33-9	Phenylbutazone	-3.1564
1211441-98-3	Ribociclib	-3.1562
125317-39-7	Vinorelbine Tartrate	-3.1558
22071-15-4	Ketoprofen	-3.1556
56296-78-7	Fluoxetine hydrochloride	-3.1552
54910-89-3	Fluoxetine	-3.1552
4360-12-7	Ajmaline	-3.1549
980-71-2	Brompheniramine hydrogen maleate	-3.1545
2259-96-3	Cyclothiazide	-3.1541
220991-20-8	Lumiracoxib	-3.1525
4852-22-6	Proanthocyanidins	-3.1519
77-67-8	Ethosuximide	-3.1508
4852-22-6	Proanthocyanidins	-3.1499
65666-07-1	Silymarin	-3.1496
68767-14-6	Loxoprofen	-3.1496
5370-01-4	Mexiletine hydrochloride	-3.1489
52-24-4	Triethylenethiophosphoramide	-3.1473
130929-57-6	Entacapone	-3.1466
330808-88-3	Vardenafil hydrochloride trihydrate	-3.1462
29608-49-9	Almitrine dimesylate	-3.1444
28395-03-1	Bumetanide	-3.1435
459789-99-2	Obeticholic Acid	-3.1431
149003-01-0	Dexrazoxane HCl	-3.143
65666-07-1	Silymarin	-3.1428
57-67-0	Sulfaguanidine	-3.1425
34562-97-5	Pikamilone	-3.1421
4852-22-6	Proanthocyanidins	-3.1408
4852-22-6	Proanthocyanidins	-3.1398
4852-22-6	Proanthocyanidins	-3.1391
73816-42-9	Meclocycline sulfosalicylate salt	-3.1378

74150-27-9	Pimobendan	-3.1377
74711-43-6	Zaltoprofen	-3.1372
56-04-2	Methylthiouracil	-3.1369
79-57-2	Oxytetracycline	-3.1348
53902-12-8	Tranilast	-3.1347
82186-77-4	Lumefantrine	-3.1346
53716-49-7	Carprofen	-3.134
527-07-1	Sodium gluconate	-3.1327
299-28-5	Calcium gluconate	-3.1327
132-18-3	Diphenylpyraline hydrochloride	-3.1315
37517-28-5	Amikacin	-3.1315
4852-22-6	Proanthocyanidins	-3.1297
1254053-43-4	Gilteritinib	-3.1283
5987-82-6	Oxybuprocaine hydrochloride	-3.1281
4852-22-6	Proanthocyanidins	-3.1267
37025-55-1	Carbetocin	-3.1266
6151-40-2	Quinidine hydrochloride	-3.1263
132-18-3	Diphenylpyraline hydrochloride	-3.1257
29908-03-0	Ademetionine	-3.1254
95635-55-5	Ranolazine	-3.1247
95635-56-6	Ranolazine dihydrochloride	-3.1247
1953-02-2	Tiopronin	-3.1246
64439-81-2	Hydroxy Camptothecine	-3.1238
64439-81-2	Hydroxy Camptothecine	-3.1233
53-86-1	Indometacin	-3.1231
878672-00-5	Lesinurad	-3.1229
123948-87-8	Topotecan	-3.1222
72-19-5	threonine	-3.1221
206658-92-6	Tacrine hydrochloride hydrate	-3.122
67656-30-8	10-Hydroxycampothecin	-3.1217
544-62-7	Batyl alcohol	-3.1212
738606-46-7	ETC1002	-3.1211
111902-57-9	Temocapil	-3.121
110221-44-8	Temocapril HCl	-3.121
550-24-3	Embelin	-3.1206
27741-01-1	Geniposidic Acid	-3.1201
1476-53-5	Novobiocin Sodium	-3.1193
50700-72-6	Vecuronium bromide	-3.1186
78246-49-8	Paroxetine hydrochloride	-3.1182
5370-01-4	Mexiletine hydrochloride	-3.118
364622-82-2	Doripenem Hydrate	-3.118
220620-09-7	Tigecycline	-3.1176

64439-81-2	Hydroxy Camptothecine	-3.1175
58895-64-0	Nalmefene hydrochloride	-3.1175
211915-06-9	Dabigatran etexilate	-3.1173
4852-22-6	Proanthocyanidins	-3.1164
53797-35-6	Ribostamycin sulfate	-3.1163
5934-29-2	L-Histidine monohydrochloride monohydrat	-3.1161
71-00-1	histidine	-3.1161
305-03-3	Chloroambucil	-3.1145
1492-18-8	Calcium folinate	-3.1144
74011-58-8	Enoxacin	-3.1138
111902-57-9	Temocapil	-3.1135
84294-96-2	Enoxacin Sesquihydrate	-3.1135
50-70-4	D-Sorbitol	-3.1133
3697-42-5	Chlorhexidine hydrochloride	-3.1131
56-95-1	Chlorhexidine Acetate	-3.1131
74-79-3	L-Arginine	-3.1129
1119-34-2	L-arginine monohydrochloride	-3.1129
90-45-9	Aminacrine	-3.1126
3562-84-3	Benzbromarone	-3.1125
132-20-7	Pheniramine maleate	-3.1123
864750-70-9	Revefenacin	-3.1119
22862-76-6	Anisomycin	-3.1109
23964-57-0	Articaine HCl	-3.1106
330808-88-3	Vardenafil hydrochloride trihydrate	-3.1105
68767-14-6	Loxoprofen	-3.1104
380843-75-4	Bosutinib	-3.1104
67656-30-8	10-Hydroxycampothecin	-3.1102
50-55-5	Reserpine	-3.1093
1953-02-2	Tiopronin	-3.1092
1254053-43-4	Gilteritinib	-3.1089
459868-92-9	Rucaparib Phosphate	-3.1088
283173-50-2	Rucaparib	-3.1088
5728-52-9	4-Biphenylacetic acid	-3.1082
68767-14-6	Loxoprofen	-3.1078
4852-22-6	Proanthocyanidins	-3.1076
6138-79-0	Triprolidine hydrochloride	-3.1068
67656-30-8	10-Hydroxycampothecin	-3.1065
19685-09-7	10-Hydroxycamptothecin	-3.1065
1225-55-4	Protriptyline hydrochloride	-3.1064
53179-09-2	Sisomicin Sulfate	-3.1061
125317-39-7	Vinorelbine Tartrate	-3.1053
59-66-5	Acetazolamide	-3.1049

5104-49-4	Flurbiprofen	-3.1039
57248-88-1	Pamidronate disodium salt	-3.1035
55-56-1	Chlorhexidine	-3.1027
364622-82-2	Doripenem Hydrate	-3.102
3505-38-2	Carbinoxamine Maleate Salt	-3.1016
5633-20-5	Oxybutynin	-3.1012
1508-65-2	Oxybutynin hydrochloride	-3.1012
74150-27-9	Pimobendan	-3.1011
51-24-1	Tiratricol	-3.1011
4852-22-6	Proanthocyanidins	-3.1006
77-67-8	Ethosuximide	-3.1003
30484-77-6	Flunarizine dihydrochloride	-3.1001
872728-81-9	Dabigatran Etexilate Mesylate	-3.0999
211915-06-9	Dabigatran etexilate	-3.0999
95-25-0	Chlorzoxazone	-3.0998
544-62-7	Batyl alcohol	-3.0993
89-25-8	Edaravone	-3.098
56-87-1	lysine	-3.0977
149022-22-0	Amikacin Sulfate Salt	-3.0953
1672-63-5	4-Hydroxyantipyrine	-3.0949
61-16-5	Methoxamine hydrochloride	-3.0944
50-91-9	Floxuridine	-3.0942
22071-15-4	Ketoprofen	-3.0931
1231929-97-7	Abemaciclib	-3.0928
111902-57-9	Temocapil	-3.0925
570406-98-3	Avatrombopag	-3.0923
77337-73-6	Acamprosate calcium	-3.0916
4852-22-6	Proanthocyanidins	-3.0899
220991-20-8	Lumiracoxib	-3.0899
13171-25-0	Trimetazidine dihydrochloride	-3.0896
1009119-65-6	Daclatasvir dihydrochloride	-3.0894
50-70-4	D-Sorbitol	-3.0894
1009119-64-5	Daclatasvir	-3.0888
141-94-6	Hexetidine	-3.0882
56391-57-2	Netilmicin sulfate	-3.0875
153559-49-0	Bexarotene	-3.0871
155148-31-5	Plerixafor 8HCl (AMD3100 8HCl)	-3.0864
110078-46-1	Plerixafor	-3.0864
1132935-63-7	Dasabuvir	-3.0859
201530-41-8	Deferasirox	-3.0859

22368-21-4	Eupatilin	-3.0858
4852-22-6	Proanthocyanidins	-3.0853
29908-03-0	Ademetionine	-3.0849
474-25-9	Chenodeoxycholic acid	-3.0838
4852-22-6	Proanthocyanidins	-3.0833
3505-38-2	Carbinoxamine Maleate Salt	-3.083
98079-52-8	Lomefloxacin hydrochloride	-3.0828
98079-51-7	Lomefloxacin	-3.0828
87239-81-4	Cefpodoxime proxetil	-3.0823
23979-41-1	(R)-Naproxen	-3.0818
303-25-3	Cyclizine hydrochloride	-3.0815
69056-38-8	Sapropterin Hydrochloride	-3.0799
50-55-5	Reserpine	-3.0789
1211441-98-3	Ribociclib	-3.0784
446-86-6	Azathioprine	-3.0783
149-91-7	Gallic acid	-3.0763
132-18-3	Diphenylpyraline hydrochloride	-3.0762
87233-61-2	Emedastine	-3.0754
1173-88-2	Oxacillin sodium	-3.0748
4852-22-6	Proanthocyanidins	-3.0735
1188-38-1	Carbaglu	-3.0733
95734-82-0	Nedaplatin	-3.0731
459868-92-9	Rucaparib Phosphate	-3.073
283173-50-2	Rucaparib	-3.073
52432-72-1	Oxeladin citrate	-3.0722
298-57-7	Stugeron	-3.0708
74150-27-9	Pimobendan	-3.0706
57248-88-1	Pamidronate disodium salt	-3.0698
52432-72-1	Oxeladin citrate	-3.0698
637-32-1	Proguanil hydrochloride	-3.0697
943-17-9	Etilefrine hydrochloride	-3.0686
4852-22-6	Proanthocyanidins	-3.068
95734-82-0	Nedaplatin	-3.0678
3505-38-2	Carbinoxamine Maleate Salt	-3.066
23142-01-0	Carbetapentane citrate	-3.0659
439239-90-4	Lasmiditan	-3.0658
364-62-5	5-Chloro-2-methoxyprocainamide	-3.0657
7232-21-5	Metoclopramide hydrochloride	-3.0657
126784-99-4	Ulipristal acetate	-3.0654
1257044-40-8	ABT199	-3.064
57-08-9	Acexamic Acid	-3.063

5451-09-2	5-Aminolevulinic acid hydrochloride	-3.0621
141-94-6	Hexetidine	-3.0613
65666-07-1	Silymarin	-3.0591
6035-45-6	Folinic Acid Calcium Salt Pentahydrate	-3.0585
59-66-5	Acetazolamide	-3.0581
304-55-2	Succimer	-3.0578
95-25-0	Chlorzoxazone	-3.0575
29216-28-2	Mequitazine	-3.0573
61-12-1	Dibucaine hydrochloride	-3.0572
85-79-0	Dibucaine	-3.0572
56-45-1	serine	-3.0568
59263-76-2	Meptazinol hydrochloride	-3.0568
87-17-2	Salicylanilide	-3.056
51-21-8	Fluorouracil	-3.0556
56-45-1	serine	-3.0522
52549-17-4	Pranoprofen	-3.0514
111902-57-9	Temocapil	-3.0514
82186-77-4	Lumefantrine	-3.0512
64439-81-2	Hydroxy Camptothecine	-3.0512
51-05-8	Procaine hydrochloride	-3.0502
59-46-1	Procaine	-3.0502
4852-22-6	Proanthocyanidins	-3.0497
87848-99-5	Acrivastine	-3.0491
51-30-9	Isoprenaline hydrochloride	-3.0485
92-61-5	Scopoletin	-3.0472
87-99-0	Xylitol	-3.0469
7414-83-7	Sodium etidronate	-3.0461
65-29-2	Gallamine triethiodide	-3.0458
521-78-8	Trimipramine maleate	-3.0452
121-33-5	Vanillin	-3.0451
115939-25-8	Salvianolic acid B	-3.045
77-92-9	Citric acid	-3.0444
128-13-2	Ursodeoxycholic acid	-3.0442
661-19-8	n-Docosanol	-3.044
111902-57-9	Temocapil	-3.0436
54-30-8	Camylofin	-3.0432
2809-21-4	Etidronate	-3.0431
574-84-5	Fraxetin	-3.0429
54-30-8	Camylofin	-3.0413
133099-07-7	Darifenacin HBr	-3.0409
3505-38-2	Carbinoxamine Maleate Salt	-3.0406
55-48-1	Atropine sulfate	-3.04
5908-99-6	Atropine sulfate monohydrate	-3.04

51-55-8	Atropine	-3.04
17696-69-4	Hematoporphyrin dihydrochloride	-3.0398
6064-83-1	Fosfosal	-3.0386
1263-89-4	Paromomycin Sulfate	-3.0386
22862-76-6	Anisomycin	-3.0382
51-30-9	Isoprenaline hydrochloride	-3.0382
3697-42-5	Chlorhexidine hydrochloride	-3.0374
56-95-1	Chlorhexidine Acetate	-3.0374
7414-83-7	Sodium etidronate	-3.037
53716-49-7	Carprofen	-3.0368
15500-66-0	Pancuronium bromide	-3.0361
4852-22-6	Proanthocyanidins	-3.036
59-66-5	Acetazolamide	-3.0358
1211441-98-3	Ribociclib	-3.0357
446-72-0	Genistein	-3.0346
5987-82-6	Oxybuprocaine hydrochloride	-3.0344
7414-83-7	Sodium etidronate	-3.0343
32986-56-4	Tobramycin	-3.0343
32986-56-4	Tobramycin	-3.0334
6160-12-9	(-)-Sparteine sulfate pentahydrate	-3.0331
79-57-2	Oxytetracycline	-3.0331
81-25-4	Cholic Acid	-3.0329
2809-21-4	Etidronate	-3.0322
5104-49-4	Flurbiprofen	-3.0317
34562-97-5	Pikamilone	-3.0316
57644-54-9	Gastrodenol	-3.0316
144675-97-8	Pixantrone dimaleate	-3.0315
4852-22-6	Proanthocyanidins	-3.0314
5370-01-4	Mexiletine hydrochloride	-3.0304
2809-21-4	Etidronate	-3.0295
3543-75-7	Bendamustine hydrochloride	-3.0295
100643-71-8	Desloratadine	-3.0294
6160-12-9	(-)-Sparteine sulfate pentahydrate	-3.0267
91296-87-6	Sarafloxacin hydrochloride	-3.0267
54-30-8	Camylofin	-3.0259
65666-07-1	Silymarin	-3.025
491-67-8	Baicalein	-3.0247
4852-22-6	Proanthocyanidins	-3.0245
1211441-98-3	Ribociclib	-3.0245
95734-82-0	Nedaplatin	-3.0242
4852-22-6	Proanthocyanidins	-3.0234

16676-29-2	Naltrexone hydrochloride	-3.0233
128-13-2	Ursodeoxycholic acid	-3.0232
149-91-7	Gallic acid	-3.0229
3543-75-7	Bendamustine hydrochloride	-3.0229
97-18-7	Bithionol	-3.0227
32986-56-4	Tobramycin	-3.0211
151533-22-1	Levomefolate calcium	-3.0208
119-36-8	Methyl salicylate	-3.0207
42461-84-7	Flunixin meglumin	-3.0206
110-15-6	Succinic acid	-3.0203
4852-22-6	Proanthocyanidins	-3.0202
111902-57-9	Temocapil	-3.0198
64-73-3	Demeclocycline hydrochloride	-3.0196
980-71-2	Brompheniramine hydrogen maleate	-3.0193
30299-08-2	Clinofibrate	-3.0182
22373-78-0	Monensin sodium salt	-3.0172
1231929-97-7	Abemaciclib	-3.0169
59338-87-3	Alizapride hydrochloride	-3.0169
90-45-9	Aminacrine	-3.0157
59338-87-3	Alizapride hydrochloride	-3.0156
3963-95-9	Methacycline hydrochloride	-3.0151
637-32-1	Proguanil hydrochloride	-3.0149
1263-89-4	Paromomycin Sulfate	-3.0148
104376-79-6	Ceftriaxone sodium	-3.0142
224452-66-8	Retapamulin	-3.0139
2022-85-7	Fluorocytosine	-3.0139
574-84-5	Fraxetin	-3.0135
57-67-0	Sulfaguanidine	-3.0125
22494-42-4	Diflunisal	-3.0116
22071-15-4	Ketoprofen	-3.0115
486-35-1	Daphnetin	-3.011
4360-12-7	Ajmaline	-3.0099
139264-17-8	Zolmitriptan	-3.0097
2438-32-6	Dexchlorpheniramine Maleate	-3.0093
56-84-8	L-Aspartic acid	-3.0088
56-87-1	lysine	-3.0074
56-87-1	lysine	-3.0072
59338-87-3	Alizapride hydrochloride	-3.0065
5451-09-2	5-Aminolevulinic acid hydrochloride	-3.0057
65666-07-1	Silymarin	-3.0056
56-45-1	serine	-3.0055
127-69-5	Sulfisoxazole	-3.0054

95-25-0	Chlorzoxazone	-3.0051
121268-17-5	Alendronate sodium hydrate	-3.0048
987-78-0	Citicoline	-3.0047
155148-31-5	Plerixafor 8HCl (AMD3100 8HCl)	-3.0045
110078-46-1	Plerixafor	-3.0045
22888-70-6	Silibinin	-3.004
521-78-8	Trimipramine maleate	-3.0038
5104-49-4	Flurbiprofen	-3.0033
1009119-65-6	Daclatasvir dihydrochloride	-3.0022
2259-96-3	Cyclothiazide	-3.002
79-57-2	Oxytetracycline	-3.0011
99-15-0	Acetylleucine	-3.0006
65666-07-1	Silymarin	-3.0002
125317-39-7	Vinorelbine Tartrate	-3.0001
3562-84-3	Benzbromarone	-2.9996
1025504-45-3	Valbenazine	-2.9994
141-94-6	Hexetidine	-2.9988
4852-22-6	Proanthocyanidins	-2.9981
872728-81-9	Dabigatran Etexilate Mesylate	-2.9978
211915-06-9	Dabigatran etexilate	-2.9978
65666-07-1	Silymarin	-2.9971
544-62-7	Batyl alcohol	-2.9969
59263-76-2	Meptazinol hydrochloride	-2.9968
943-17-9	Etilefrine hydrochloride	-2.9962
83-44-3	Deoxycholic acid	-2.9961
951395-08-7	tafamidis meglumine	-2.9957
827022-33-3	Palbociclib Isethionate	-2.995
827022-32-2	Palbociclib hydrochloride	-2.995
110871-86-8	Sparfloxacin	-2.9944
83-44-3	Deoxycholic acid	-2.9941
111902-57-9	Temocapil	-2.9937
51940-44-4	Pipemidic acid	-2.9929
74711-43-6	Zaltoprofen	-2.9925
81-25-4	Cholic Acid	-2.9924
389139-89-3	Omadacycline	-2.9922
571190-30-2	Palbociclib	-2.992
16676-29-2	Naltrexone hydrochloride	-2.9918
57-96-5	Sulfinpyrazone	-2.9911
22204-53-1	Naproxen	-2.9886
491-67-8	Baicalein	-2.9885
3737-09-5	Disopyramide	-2.9882

4852-22-6	Proanthocyanidins	-2.9875
1231930-82-7	LY2835219	-2.9864
1847-24-1	Flucloxacillin sodium	-2.9864
4852-22-6	Proanthocyanidins	-2.9847
113-92-8	Chlorpheniramine maleate	-2.9843
1421373-65-0	Osimertinib	-2.9839
51-30-9	Isoprenaline hydrochloride	-2.9829
645-43-2	Guanethidine Monosulfate	-2.9829
59338-87-3	Alizapride hydrochloride	-2.9828
74011-58-8	Enoxacin	-2.9827
84294-96-2	Enoxacin Sesquihydrate	-2.9827
67656-30-8	10-Hydroxycampothecin	-2.9825
19685-09-7	10-Hydroxycamptothecin	-2.9825
19767-45-4	Mesna	-2.9824
661-19-8	n-Docosanol	-2.9822
125317-39-7	Vinorelbine Tartrate	-2.982
5104-49-4	Flurbiprofen	-2.9814
1421373-66-1	Osimertinib mesylate	-2.9812
59338-87-3	Alizapride hydrochloride	-2.9804
364-98-7	Diazoxide	-2.9804
111902-57-9	Temocapil	-2.9796
65-85-0	Benzoic acid	-2.9791
1953-02-2	Tiopronin	-2.9788
51-21-8	Fluorouracil	-2.9774
97-18-7	Bithionol	-2.9764
6078-17-7	Berbamine dihydrochloride	-2.9762
4852-22-6	Proanthocyanidins	-2.976
3562-84-3	Benzbromarone	-2.9758
68767-14-6	Loxoprofen	-2.9758
4852-22-6	Proanthocyanidins	-2.9754
439081-18-2	Afatinib	-2.975
53179-09-2	Sisomicin Sulfate	-2.9743
219861-08-2	Escitalopram Oxalate	-2.9738
59729-32-7	Citalopram HBr	-2.9738
87239-81-4	Cefpodoxime proxetil	-2.9725
149003-01-0	Dexrazoxane HCl	-2.972
68497-62-1	Pramiracetam	-2.9719
96829-58-2	Orlistat	-2.9713
87-67-2	Choline bitartrate	-2.971
67-48-1	Choline chloride	-2.971

58895-64-0	Nalmefene hydrochloride	-2.9709
64-73-3	Demeclocycline hydrochloride	-2.9708
58-85-5	D-Biotin	-2.9696
5728-52-9	4-Biphenylacetic acid	-2.9695
537-73-5	Isoferulic acid	-2.9688
17696-69-4	Hematoporphyrin dihydrochloride	-2.9686
69558-55-0	Thymopentin	-2.9686
64-72-2	Chlortetracycline hydrochloride	-2.9683
552-94-3	Sasapyrine	-2.9679
850140-73-7	Afatinib Dimaleate	-2.9666
58895-64-0	Nalmefene hydrochloride	-2.9656
98079-52-8	Lomefloxacin hydrochloride	-2.9649
98079-51-7	Lomefloxacin	-2.9649
81919-14-4	Bendazac L-lysine	-2.9646
77-67-8	Ethosuximide	-2.9643
155148-31-5	Plerixafor 8HCl (AMD3100 8HCl)	-2.9634
110078-46-1	Plerixafor	-2.9634
17696-69-4	Hematoporphyrin dihydrochloride	-2.9633
41575-94-4	Carboplatin	-2.9624
1953-02-2	Tiopronin	-2.9614
108612-45-9	Mizolastine	-2.9614
2438-32-6	Dexchlorpheniramine Maleate	-2.961
6620-60-6	Proglumide	-2.9609
85721-33-1	Ciprofloxacin	-2.9599
93107-08-5	Ciprofloxacin hydrochloride	-2.9599
65666-07-1	Silymarin	-2.958
1009119-65-6	Daclatasvir dihydrochloride	-2.9572
74103-07-4	Ketorolac tromethamine salt	-2.9563
4852-22-6	Proanthocyanidins	-2.9542
83-44-3	Deoxycholic acid	-2.9542
2398-81-4	Nicotinic acid N-oxide	-2.9534
164579-32-2	Pantoprazole Sodium Hydrate	-2.9524
77337-73-6	Acamprosate calcium	-2.9514
111902-57-9	Temocapil	-2.951
144143-96-4	Eprosartan Mesylate	-2.9504
12650-69-0	Mupirocin	-2.949
155148-31-5	Plerixafor 8HCl (AMD3100 8HCl)	-2.9487
110078-46-1	Plerixafor	-2.9487
65666-07-1	Silymarin	-2.9486
122647-32-9	Ibutilide Fumarate	-2.9485
4852-22-6	Proanthocyanidins	-2.9483
83905-01-5	Azithromycin	-2.9477

90-45-9	Aminacrine	-2.9474
134308-13-7	Tolcapone	-2.9474
527-07-1	Sodium gluconate	-2.9435
299-28-5	Calcium gluconate	-2.9435
637-32-1	Proguanil hydrochloride	-2.9427
56391-57-2	Netilmicin sulfate	-2.9426
65666-07-1	Silymarin	-2.9424
17696-69-4	Hematoporphyrin dihydrochloride	-2.9424
637-32-1	Proguanil hydrochloride	-2.9416
148-24-3	Hydroxyquinoline	-2.9415
1492-18-8	Calcium folinate	-2.9415
97-18-7	Bithionol	-2.9406
108612-45-9	Mizolastine	-2.9403
4852-22-6	Proanthocyanidins	-2.9399
53179-09-2	Sisomicin Sulfate	-2.9396
4852-22-6	Proanthocyanidins	-2.9391
965-52-6	Nifuroxazide	-2.9383
878672-00-5	Lesinurad	-2.938
220620-09-7	Tigecycline	-2.9373
68767-14-6	Loxoprofen	-2.9369
147127-20-6	Tenofovir	-2.9365
4852-22-6	Proanthocyanidins	-2.9362
3963-95-9	Methacycline hydrochloride	-2.9362
749-02-0	Spiperone	-2.9355
65666-07-1	Silymarin	-2.9347
29216-28-2	Mequitazine	-2.934
57-08-9	Acexamic Acid	-2.9336
2068-78-2	Vincristine sulfate	-2.9334
16676-29-2	Naltrexone hydrochloride	-2.9325
125317-39-7	Vinorelbine Tartrate	-2.9309
151533-22-1	Levomefolate calcium	-2.9308
51-30-9	Isoprenaline hydrochloride	-2.9305
2068-78-2	Vincristine sulfate	-2.93
28395-03-1	Bumetanide	-2.9298
486-35-1	Daphnetin	-2.9283
4093-35-0	Bromopride	-2.9281
17696-69-4	Hematoporphyrin dihydrochloride	-2.9266
15676-16-1	Sulpiride	-2.9245
3644-61-9	Tolperisone hydrochloride	-2.9241
206658-92-6	Tacrine hydrochloride hydrate	-2.9241
441798-33-0	Macitentan	-2.9241
141505-33-1	Levosimendan	-2.9236
3679-64-9	Salifungin	-2.9232

90-33-5	4-Methylumbelliferone	-2.9229
1009119-64-5	Daclatasvir	-2.9228
1254053-43-4	Gilteritinib	-2.9225
22368-21-4	Eupatilin	-2.9216
56296-78-7	Fluoxetine hydrochloride	-2.9216
54910-89-3	Fluoxetine	-2.9216
131707-23-8	Arbidol hydrochloride	-2.9205
78246-49-8	Paroxetine hydrochloride	-2.9204
1405-41-0	Gentamycin sulfate	-2.9204
55-86-7	Mechlorethamine hydrochloride	-2.9195
99300-78-4	Venlafaxine hydrochloride	-2.9195
81-25-4	Cholic Acid	-2.9193
51-24-1	Tiratricol	-2.9192
6078-17-7	Berbamine dihydrochloride	-2.9191
104206-65-7	Nitisinone	-2.919
90-33-5	4-Methylumbelliferone	-2.9182
99-50-3	Protocatechuic acid	-2.9171
3679-64-9	Salifungin	-2.9163
30484-77-6	Flunarizine dihydrochloride	-2.9155
65-86-1	Orotic acid	-2.9154
64-77-7	Tolbutamide	-2.9149
872728-81-9	Dabigatran Etexilate Mesylate	-2.9146
211915-06-9	Dabigatran etexilate	-2.9146
1225-55-4	Protriptyline hydrochloride	-2.9146
3094-09-5	Doxifluridine	-2.9142
6138-79-0	Triprolidine hydrochloride	-2.9138
1476-53-5	Novobiocin Sodium	-2.9138
119-36-8	Methyl salicylate	-2.9129
81103-11-9	Clarithromycin	-2.9125
5370-01-4	Mexiletine hydrochloride	-2.9125
111902-57-9	Temocapil	-2.9125
4852-22-6	Proanthocyanidins	-2.9118
89396-94-1	Imidapril HCl	-2.9107
1354744-91-4	Naloxegol oxalate	-2.9105
18085-97-7	Jaceosidin	-2.9104
1403254-99-8	EPZ6438	-2.9095
459868-92-9	Rucaparib Phosphate	-2.9091
283173-50-2	Rucaparib	-2.9091
108612-45-9	Mizolastine	-2.9089
80433-71-2	Calcium Levofolinate	-2.9082

57248-88-1	Pamidronate disodium salt	-2.908
1009119-65-6	Daclatasvir dihydrochloride	-2.9078
29216-28-2	Mequitazine	-2.9077
486-35-1	Daphnetin	-2.9076
81919-14-4	Bendazac L-lysine	-2.9075
4852-22-6	Proanthocyanidins	-2.9066
13171-25-0	Trimetazidine dihydrochloride	-2.906
137975-06-5	Mozavaptan	-2.9047
4852-22-6	Proanthocyanidins	-2.9043
65-29-2	Gallamine triethiodide	-2.9039
6078-17-7	Berbamine dihydrochloride	-2.9031
64-75-5	Tetracycline hydrochloride	-2.9027
3737-09-5	Disopyramide	-2.9023
101-31-5	L-Hyoscyamine	-2.9023
55-48-1	Atropine sulfate	-2.902
5908-99-6	Atropine sulfate monohydrate	-2.902
51-55-8	Atropine	-2.902
4852-22-6	Proanthocyanidins	-2.9008
73536-69-3	Bifendatatum	-2.9002
364-62-5	5-Chloro-2-methoxyprocainamide	-2.9002
7232-21-5	Metoclopramide hydrochloride	-2.9002
24729-96-2	Clindamycin phosphate	-2.8997
119-36-8	Methyl salicylate	-2.8994
1231930-82-7	LY2835219	-2.8991
153-18-4	Rutin	-2.8981
4852-22-6	Proanthocyanidins	-2.8968
92-61-5	Scopoletin	-2.8956
99-50-3	Protocatechuic acid	-2.8945
7681-93-8	Pimaricin	-2.8944
1492-18-8	Calcium folinate	-2.8943
3963-95-9	Methacycline hydrochloride	-2.8943
59-67-6	Nicotinic acid	-2.8941
4852-22-6	Proanthocyanidins	-2.8926
6620-60-6	Proglumide	-2.8924
550-99-2	Naphazoline hydrochloride	-2.8905
141505-33-1	Levosimendan	-2.8884
4852-22-6	Proanthocyanidins	-2.8879
59-67-6	Nicotinic acid	-2.8875
69056-38-8	Sapropterin Hydrochloride	-2.8874
4852-22-6	Proanthocyanidins	-2.8872
87-99-0	Xylitol	-2.8862

141505-33-1	Levosimendan	-2.8857
53179-09-2	Sisomicin Sulfate	-2.8853
68767-14-6	Loxoprofen	-2.8847
85721-33-1	Ciprofloxacin	-2.8838
93107-08-5	Ciprofloxacin hydrochloride	-2.8838
3737-09-5	Disopyramide	-2.8816
171599-83-0	Sildenafil citrate	-2.8811
164579-32-2	Pantoprazole Sodium Hydrate	-2.8806
51-30-9	Isoprenaline hydrochloride	-2.8805
52549-17-4	Pranoprofen	-2.8804
68767-14-6	Loxoprofen	-2.8798
17696-69-4	Hematoporphyrin dihydrochloride	-2.8791
639089-54-6	Tozasertib	-2.8791
112811-59-3	Gatifloxacin	-2.8785
56391-57-2	Netilmicin sulfate	-2.8769
74150-27-9	Pimobendan	-2.8758
4852-22-6	Proanthocyanidins	-2.8753
58-85-5	D-Biotin	-2.875
637-32-1	Proguanil hydrochloride	-2.8746
82586-52-5	Moexipril HCl	-2.8739
59338-87-3	Alizapride hydrochloride	-2.8734
136-47-0	Tetracaine hydrochloride	-2.8733
83-44-3	Deoxycholic acid	-2.8728
82586-52-5	Moexipril HCl	-2.8726
113-92-8	Chlorpheniramine maleate	-2.8721
51481-61-9	Cimetidine	-2.872
459868-92-9	Rucaparib Phosphate	-2.8704
283173-50-2	Rucaparib	-2.8704
55-86-7	Mechlorethamine hydrochloride	-2.8701
4852-22-6	Proanthocyanidins	-2.87
4852-22-6	Proanthocyanidins	-2.8696
74-79-3	L-Arginine	-2.8684
1119-34-2	L-arginine monohydrochloride	-2.8684
220620-09-7	Tigecycline	-2.8677
486-35-1	Daphnetin	-2.8676
17696-69-4	Hematoporphyrin dihydrochloride	-2.8675
61-68-7	Mefenamic acid	-2.8673
68767-14-6	Loxoprofen	-2.867
65-29-2	Gallamine triethiodide	-2.8669
486-35-1	Daphnetin	-2.8658
57-08-9	Acexamic Acid	-2.8652
951395-08-7	tafamidis meglumine	-2.8636
4852-22-6	Proanthocyanidins	-2.8635

247257-48-3	Fimasartan	-2.8634
32986-56-4	Tobramycin	-2.8623
304-55-2	Succimer	-2.8612
164579-32-2	Pantoprazole Sodium Hydrate	-2.8611
22204-53-1	Naproxen	-2.8611
18085-97-7	Jaceosidin	-2.8604
980-71-2	Brompheniramine hydrogen maleate	-2.86
872728-81-9	Dabigatran Etexilate Mesylate	-2.8598
211915-06-9	Dabigatran etexilate	-2.8598
121268-17-5	Alendronate sodium hydrate	-2.8597
59-67-6	Nicotinic acid	-2.8597
4394-00-7	Niflumic acid	-2.8596
1009119-65-6	Daclatasvir dihydrochloride	-2.8588
69056-38-8	Sapropterin Hydrochloride	-2.8584
4852-22-6	Proanthocyanidins	-2.8568
464-92-6	Asiatic acid	-2.8544
41575-94-4	Carboplatin	-2.8541
95-25-0	Chlorzoxazone	-2.8526
103766-25-2	Gimeracil	-2.8519
53179-09-2	Sisomicin Sulfate	-2.8514
29908-03-0	Ademetionine	-2.8513
40828-46-4	Suprofen	-2.851
486-35-1	Daphnetin	-2.8509
548-66-3	Drofenine hydrochloride	-2.8506
987-78-0	Citicoline	-2.8496
15676-16-1	Sulpiride	-2.8468
66-22-8	Uracil	-2.8465
23672-07-3	Levosulpiride	-2.8456
2203-97-6	Hydrocortisone 21-hemisuccinate	-2.8453
12650-69-0	Mupirocin	-2.8426
70458-96-7	Norfloxacin	-2.8423
127373-66-4	Sivelestat	-2.8421
74103-07-4	Ketorolac tromethamine salt	-2.8419
69056-38-8	Sapropterin Hydrochloride	-2.8417
144143-96-4	Eprosartan Mesylate	-2.8413
14639-25-9	Chromium picolinate	-2.8408
574-84-5	Fraxetin	-2.8407
4852-22-6	Proanthocyanidins	-2.8403
1449-05-4	Glycyrrhetinic acid	-2.8392
4852-22-6	Proanthocyanidins	-2.8383
16676-29-2	Naltrexone hydrochloride	-2.8374
13710-19-5	Tolfenamic Acid	-2.837

68-35-9	Sulfadiazine	-2.8365
65666-07-1	Silymarin	-2.836
304-55-2	Succimer	-2.8355
65666-07-1	Silymarin	-2.8352
980-71-2	Brompheniramine hydrogen maleate	-2.8336
59338-87-3	Alizapride hydrochloride	-2.8332
550-99-2	Naphazoline hydrochloride	-2.8321
60-54-8	Tetracycline	-2.831
40828-46-4	Suprofen	-2.83
52549-17-4	Pranoprofen	-2.8291
55-48-1	Atropine sulfate	-2.8285
5908-99-6	Atropine sulfate monohydrate	-2.8285
51-55-8	Atropine	-2.8285
4852-22-6	Proanthocyanidins	-2.8282
87233-61-2	Emedastine	-2.8275
4852-22-6	Proanthocyanidins	-2.8273
74150-27-9	Pimobendan	-2.8265
22888-70-6	Silibinin	-2.8263
923604-59-5	Simeprevir	-2.8255
58947-95-8	DL-trihexyphenidyl hydrochloride	-2.8251
101-31-5	L-Hyoscyamine	-2.824
132-20-7	Pheniramine maleate	-2.8238
64-72-2	Chlortetracycline hydrochloride	-2.8235
1420477-60-6	Acalabrutinib	-2.8233
134308-13-7	Tolcapone	-2.8231
56391-57-2	Netilmicin sulfate	-2.8228
4852-22-6	Proanthocyanidins	-2.8224
87239-81-4	Cefpodoxime proxetil	-2.8221
31282-04-9	Hygromycin B	-2.8221
2259-96-3	Cyclothiazide	-2.8217
16676-29-2	Naltrexone hydrochloride	-2.8215
138452-21-8	Fexofenadine hydrochloride	-2.8215
135459-87-9	Strontium ranelate	-2.821
69558-55-0	Thymopentin	-2.8202
23672-07-3	Levosulpiride	-2.8198
41575-94-4	Carboplatin	-2.8194
81-25-4	Cholic Acid	-2.8194
290297-26-6	Netupitant	-2.8194
248281-84-7	Laquinimod	-2.8192
15676-16-1	Sulpiride	-2.8186
2508-72-7	Antazoline hydrochloride	-2.8184
475086-01-2	Selexipag	-2.8177

70797-11-4	Cefpiramide acid	-2.8173
4852-22-6	Proanthocyanidins	-2.8173
4852-22-6	Proanthocyanidins	-2.8141
923604-59-5	Simeprevir	-2.812
15676-16-1	Sulpiride	-2.8114
65666-07-1	Silymarin	-2.8112
4852-22-6	Proanthocyanidins	-2.8108
1221573-85-8	Paritaprevir	-2.8105
16676-29-2	Naltrexone hydrochloride	-2.8099
57-41-0	Phenytoin	-2.8093
630-93-3	Phenytoin sodium	-2.8087
126784-99-4	Ulipristal acetate	-2.808
127294-70-6	Balofloxacin	-2.8068
57-41-0	Phenytoin	-2.8067
6856-31-1	Loxeen	-2.8063
66-22-8	Uracil	-2.8032
630-93-3	Phenytoin sodium	-2.8028
14639-25-9	Chromium picolinate	-2.8025
3505-38-2	Carbinoxamine Maleate Salt	-2.802
155148-31-5	Plerixafor 8HCl (AMD3100 8HCl)	-2.8012
110078-46-1	Plerixafor	-2.8012
459789-99-2	Obeticholic Acid	-2.7992
530-78-9	Flufenamic acid	-2.7985
1098-97-1	Pyrithioxin	-2.7966
1346242-81-6	Erdafitinib	-2.7962
141-94-6	Hexetidine	-2.796
364-62-5	5-Chloro-2-methoxyprocainamide	-2.7954
7232-21-5	Metoclopramide hydrochloride	-2.7954
64-75-5	Tetracycline hydrochloride	-2.7946
755038-65-4	Volasertib	-2.793
17737-65-4	Clonixin	-2.7923
577778-58-6	Topiroxostat	-2.7918
29868-97-1	Pirenzepine dihydrochloride	-2.7902
3094-09-5	Doxifluridine	-2.7892
91-85-0	Thonzylamine	-2.7889
23979-41-1	(R)-Naproxen	-2.7871
155148-31-5	Plerixafor 8HCl (AMD3100 8HCl)	-2.787
110078-46-1	Plerixafor	-2.787
83435-67-0	Delapril?Hydrochloride	-2.7866
464-92-6	Asiatic acid	-2.7862
108612-45-9	Mizolastine	-2.7845

14639-25-9	Chromium picolinate	-2.7842
144143-96-4	Eprosartan Mesylate	-2.7841
6856-31-1	Loxeen	-2.7839
61-68-7	Mefenamic acid	-2.7834
4852-22-6	Proanthocyanidins	-2.7832
125317-39-7	Vinorelbine Tartrate	-2.7828
537-73-5	Isoferulic acid	-2.7814
389139-89-3	Omadacycline	-2.7814
562-10-7	Doxylamine succinate	-2.7813
139264-17-8	Zolmitriptan	-2.7809
330808-88-3	Vardenafil hydrochloride trihydrate	-2.7791
389139-89-3	Omadacycline	-2.7778
56391-57-2	Netilmicin sulfate	-2.7777
34562-97-5	Pikamilone	-2.7776
114798-26-4	Losartan	-2.7776
51-24-1	Tiratricol	-2.7775
439239-90-4	Lasmiditan	-2.7774
74011-58-8	Enoxacin	-2.7763
112811-59-3	Gatifloxacin	-2.7761
63-45-6	Primaquine diphosphate	-2.776
3505-38-2	Carbinoxamine Maleate Salt	-2.7757
84294-96-2	Enoxacin Sesquihydrate	-2.7754
4852-22-6	Proanthocyanidins	-2.7737
53179-09-2	Sisomicin Sulfate	-2.773
155148-31-5	Plerixafor 8HCl (AMD3100 8HCl)	-2.7721
110078-46-1	Plerixafor	-2.7721
127373-66-4	Sivelestat	-2.7696
55-86-7	Mechlorethamine hydrochloride	-2.7694
901119-35-5	Fostamatinib	-2.7687
21736-83-4	Spectinomycin dihydrochloride	-2.7682
1405-41-0	Gentamycin sulfate	-2.768
81-23-2	Dehydrocholic acid	-2.7666
389139-89-3	Omadacycline	-2.7656
81-25-4	Cholic Acid	-2.765
67-43-6	Pentetic Acid	-2.7647
56-89-3	cystine	-2.7641
29608-49-9	Almitrine dimesylate	-2.7628
27591-69-1	Tilorone dihydrochloride	-2.7602
4852-22-6	Proanthocyanidins	-2.7601
76584-70-8	Divalproex Sodium	-2.7601
77-92-9	Citric acid	-2.7592
74-79-3	L-Arginine	-2.7576
1119-34-2	L-arginine monohydrochloride	-2.7576

24356-60-3	Cephapirin Sodium	-2.7573
491-67-8	Baicalein	-2.7558
290297-26-6	Netupitant	-2.7543
4852-22-6	Proanthocyanidins	-2.753
141505-33-1	Levosimendan	-2.7522
934660-93-2	Cobimetinib	-2.752
339-72-0	L-Cycloserine	-2.7509
74150-27-9	Pimobendan	-2.7484
82009-34-5	Cilastatin	-2.7471
22888-70-6	Silibinin	-2.7466
29216-28-2	Mequitazine	-2.7464
98079-52-8	Lomefloxacin hydrochloride	-2.7438
98079-51-7	Lomefloxacin	-2.7438
6138-79-0	Triprolidine hydrochloride	-2.7435
68767-14-6	Loxoprofen	-2.742
13710-19-5	Tolfenamic Acid	-2.7418
3679-64-9	Salifungin	-2.7416
10347-81-6	Maprotiline hydrochloride	-2.7411
58947-95-8	DL-trihexyphenidyl hydrochloride	-2.741
69056-38-8	Sapropterin Hydrochloride	-2.7393
58-28-6	Desipramine hydrochloride	-2.7387
23964-57-0	Articaine HCl	-2.7384
6856-31-1	Loxeen	-2.7378
73816-42-9	Meclocycline sulfosalicylate salt	-2.7375
3505-38-2	Carbinoxamine Maleate Salt	-2.7373
127-69-5	Sulfisoxazole	-2.7363
132-17-2	Benztropine mesylate	-2.7327
1231930-82-7	LY2835219	-2.7323
459868-92-9	Rucaparib Phosphate	-2.7323
283173-50-2	Rucaparib	-2.7323
298-57-7	Stugeron	-2.7322
50700-72-6	Vecuronium bromide	-2.7303
144060-53-7	Febuxostat	-2.7299
56-92-8	Histamine 2HCl	-2.727
51-45-6	Histamine	-2.727
92636-39-0	Cefminox Sodium	-2.7265
1476-53-5	Novobiocin Sodium	-2.7258
22161-81-5	(S)-(+)-Ketoprofen	-2.7257
22071-15-4	Ketoprofen	-2.7257
220620-09-7	Tigecycline	-2.7255
37517-28-5	Amikacin	-2.7254
5728-52-9	4-Biphenylacetic acid	-2.7254

132-20-7	Pheniramine maleate	-2.724
25389-94-0	Kanamycin sulfate	-2.724
171599-83-0	Sildenafil citrate	-2.7236
78213-16-8	Diclofenac diethylamine	-2.7235
37025-55-1	Carbetocin	-2.723
15307-86-5	Diclofenac	-2.7226
112811-59-3	Gatifloxacin	-2.7225
68767-14-6	Loxoprofen	-2.7224
142-47-2	L-Glutamic acid monosodium salt	-2.7219
56-86-0	glutamic acid	-2.7219
4852-22-6	Proanthocyanidins	-2.7214
864750-70-9	Revefenacin	-2.7206
446-86-6	Azathioprine	-2.7205
51-05-8	Procaine hydrochloride	-2.7192
59-46-1	Procaine	-2.7192
29908-03-0	Ademetionine	-2.7189
56-92-8	Histamine 2HCl	-2.7187
51-45-6	Histamine	-2.7187
56-84-8	L-Aspartic acid	-2.7187
86541-74-4	Benazepril hydrochloride	-2.7183
574-84-5	Fraxetin	-2.7178
7421-40-1	Carbenoxolone disodium	-2.7175
4852-22-6	Proanthocyanidins	-2.717
138452-21-8	Fexofenadine hydrochloride	-2.7157
29908-03-0	Ademetionine	-2.7149
1025504-45-3	Valbenazine	-2.7136
53-86-1	Indometacin	-2.7133
1009119-64-5	Daclatasvir	-2.7128
51-30-9	Isoprenaline hydrochloride	-2.7124
32986-56-4	Tobramycin	-2.7123
4852-22-6	Proanthocyanidins	-2.7121
550-24-3	Embelin	-2.7118
5451-09-2	5-Aminolevulinic acid hydrochloride	-2.7113
50-01-1	Guanidine hydrochloride	-2.7112
82186-77-4	Lumefantrine	-2.7106
36322-90-4	Piroxicam	-2.7104
148-24-3	Hydroxyquinoline	-2.7092
2508-72-7	Antazoline hydrochloride	-2.7057
6138-79-0	Triprolidine hydrochloride	-2.7046
138402-11-6	Irbesartan	-2.7043
4852-22-6	Proanthocyanidins	-2.704

3505-38-2	Carbinoxamine Maleate Salt	-2.7038
755038-65-4	Volasertib	-2.7036
4852-22-6	Proanthocyanidins	-2.7028
26171-23-3	Tolmetin	-2.7028
937272-79-2	Pacritinib	-2.7026
6078-17-7	Berbamine dihydrochloride	-2.7014
4852-22-6	Proanthocyanidins	-2.7006
68302-57-8	Amlexanox	-2.6991
1197953-54-0	Brigatinib	-2.6991
83905-01-5	Azithromycin	-2.6989
747-36-4	Hydroxychloroquine sulfate	-2.6984
4852-22-6	Proanthocyanidins	-2.6982
116-43-8	Succinylsulfathiazole	-2.6969
33005-95-7	Tiaprofenic acid	-2.6959
53716-49-7	Carprofen	-2.6956
87848-99-5	Acrivastine	-2.6955
141-94-6	Hexetidine	-2.6951
4852-22-6	Proanthocyanidins	-2.6941
4852-22-6	Proanthocyanidins	-2.694
4852-22-6	Proanthocyanidins	-2.6929
141-94-6	Hexetidine	-2.6927
32795-44-1	Acecainide	-2.6925
7414-83-7	Sodium etidronate	-2.6887
1611493-60-7	Bictegravir	-2.6884
864750-70-9	Revefenacin	-2.6884
2259-96-3	Cyclothiazide	-2.6868
4852-22-6	Proanthocyanidins	-2.6866
2068-78-2	Vincristine sulfate	-2.6864
142-47-2	L-Glutamic acid monosodium salt	-2.6862
56-86-0	glutamic acid	-2.6862
50-55-5	Reserpine	-2.6834
224452-66-8	Retapamulin	-2.6828
4852-22-6	Proanthocyanidins	-2.6826
2809-21-4	Etidronate	-2.6824
2438-32-6	Dexchlorpheniramine Maleate	-2.6796
111902-57-9	Temocapil	-2.6781
55-48-1	Atropine sulfate	-2.6763
5908-99-6	Atropine sulfate monohydrate	-2.6763
51-55-8	Atropine	-2.6763
155148-31-5	Plerixafor 8HCl (AMD3100 8HCl)	-2.6763
110078-46-1	Plerixafor	-2.6763

18699-02-0	Actarit	-2.6751
65666-07-1	Silymarin	-2.6748
1492-18-8	Calcium folinate	-2.6739
943-17-9	Etilefrine hydrochloride	-2.673
637-32-1	Proguanil hydrochloride	-2.6726
111902-57-9	Temocapil	-2.672
562-10-7	Doxylamine succinate	-2.6715
67-43-6	Pentetic Acid	-2.6713
128-13-2	Ursodeoxycholic acid	-2.6709
1038915-73-9	Niraparib tosylate	-2.6707
15676-16-1	Sulpiride	-2.67
126784-99-4	Ulipristal acetate	-2.6695
4146-30-9	Neomycin Sulphate B	-2.6691
81-27-6	Sennoside A	-2.6683
1038915-60-4	Niraparib	-2.6677
562-10-7	Doxylamine succinate	-2.6675
148-24-3	Hydroxyquinoline	-2.6673
23964-57-0	Articaine HCl	-2.6672
139264-17-8	Zolmitriptan	-2.6658
23672-07-3	Levosulpiride	-2.6658
90-33-5	4-Methylumbelliferone	-2.6656
1492-18-8	Calcium folinate	-2.6654
42461-84-7	Flunixin meglumin	-2.6649
934660-93-2	Cobimetinib	-2.6646
3505-38-2	Carbinoxamine Maleate Salt	-2.6645
60-54-8	Tetracycline	-2.6645
90098-04-7	Rebamipide	-2.6629
6035-45-6	Folinic Acid Calcium Salt Pentahydrate	-2.6617
51-05-8	Procaine hydrochloride	-2.6613
59-46-1	Procaine	-2.6613
172152-50-0	Ilaprazole sodium	-2.6609
22494-42-4	Diflunisal	-2.6609
83435-67-0	Delapril?Hydrochloride	-2.6607
936091-26-8	Fedratinib	-2.6597
32986-56-4	Tobramycin	-2.6592
302-79-4	Tretinoin	-2.6583
70458-96-7	Norfloxacin	-2.6582
58947-95-8	DL-trihexyphenidyl hydrochloride	-2.6569
56-89-3	cystine	-2.6569
4852-22-6	Proanthocyanidins	-2.6569
2203-97-6	Hydrocortisone 21-hemisuccinate	-2.6567

172152-50-0	Ilaprazole sodium	-2.6553
1492-18-8	Calcium folinate	-2.6548
550-24-3	Embelin	-2.6546
2508-72-7	Antazoline hydrochloride	-2.6538
562-10-7	Doxylamine succinate	-2.6525
144060-53-7	Febuxostat	-2.6518
29908-03-0	Ademetionine	-2.6517
151533-22-1	Levomefolate calcium	-2.6511
1405-37-4	Capastat sulfate	-2.6502
747-36-4	Hydroxychloroquine sulfate	-2.6501
99300-78-4	Venlafaxine hydrochloride	-2.6498
53902-12-8	Tranilast	-2.6497
132-60-5	Cinchophen	-2.6484
3254-89-5	Diphenidol hydrochloride	-2.6476
1197953-54-0	Brigatinib	-2.6472
65666-07-1	Silymarin	-2.6463
155148-31-5	Plerixafor 8HCl (AMD3100 8HCl)	-2.6458
110078-46-1	Plerixafor	-2.6458
5053-08-7	Fenspiride hydrochloride	-2.6449
73816-42-9	Meclocycline sulfosalicylate salt	-2.6445
923604-59-5	Simeprevir	-2.6431
64-72-2	Chlortetracycline hydrochloride	-2.641
82586-55-8	Quinapril hydrochloride	-2.6392
110-15-6	Succinic acid	-2.6382
98079-52-8	Lomefloxacin hydrochloride	-2.638
98079-51-7	Lomefloxacin	-2.638
111902-57-9	Temocapil	-2.637
52549-17-4	Pranoprofen	-2.6365
168273-06-1	Rimonabant	-2.6362
158681-13-1	Rimonabant hydrochloride	-2.6362
474-25-9	Chenodeoxycholic acid	-2.6354
37517-28-5	Amikacin	-2.6352
1070-11-7	Ethambutol dihydrochloride	-2.635
29908-03-0	Ademetionine	-2.6349
80433-71-2	Calcium Levofolinate	-2.6339
3505-38-2	Carbinoxamine Maleate Salt	-2.6333
70458-96-7	Norfloxacin	-2.633
65666-07-1	Silymarin	-2.6325
2259-96-3	Cyclothiazide	-2.6316
389139-89-3	Omadacycline	-2.631
630-93-3	Phenytoin sodium	-2.6302
562-10-7	Doxylamine succinate	-2.63

78967-07-4	Mofezolac	-2.6294
138452-21-8	Fexofenadine hydrochloride	-2.6292
57-41-0	Phenytoin	-2.629
56-41-7	alanine	-2.6287
96847-55-1	Levomilnacipran	-2.6284
536-43-6	Dyclonine hydrochloride	-2.627
562-10-7	Doxylamine succinate	-2.6269
219861-08-2	Escitalopram Oxalate	-2.6269
59729-32-7	Citalopram HBr	-2.6269
53902-12-8	Tranilast	-2.6268
550-99-2	Naphazoline hydrochloride	-2.6242
1405-41-0	Gentamycin sulfate	-2.6238
562-10-7	Doxylamine succinate	-2.6227
2068-78-2	Vincristine sulfate	-2.6215
62-31-7	Dopamine hydrochloride	-2.6205
68767-14-6	Loxoprofen	-2.62
4852-22-6	Proanthocyanidins	-2.6186
26171-23-3	Tolmetin	-2.6179
65666-07-1	Silymarin	-2.6178
139264-17-8	Zolmitriptan	-2.617
389139-89-3	Omadacycline	-2.6167
126784-99-4	Ulipristal acetate	-2.6167
126784-99-4	Ulipristal acetate	-2.6163
808118-40-3	Roxadustat	-2.6163
65-85-0	Benzoic acid	-2.6162
97682-44-5	Irinotecan	-2.6155
99-50-3	Protocatechuic acid	-2.6141
56-92-8	Histamine 2HCl	-2.6138
51-45-6	Histamine	-2.6138
147127-20-6	Tenofovir	-2.6138
4852-22-6	Proanthocyanidins	-2.6133
95-25-0	Chlorzoxazone	-2.6129
136572-09-3	Irinotecan hydrochloride trihydrate	-2.6128
27848-84-6	Nicergoline	-2.6121
1374356-45-2	(S)-crizotinib	-2.6102
138452-21-8	Fexofenadine hydrochloride	-2.6099
4394-00-7	Niflumic acid	-2.6098
6078-17-7	Berbamine dihydrochloride	-2.6095
96847-55-1	Levomilnacipran	-2.6095
4852-22-6	Proanthocyanidins	-2.6092
2068-78-2	Vincristine sulfate	-2.6081
550-24-3	Embelin	-2.6078

101-31-5	L-Hyoscyamine	-2.6075
121808-62-6	Pidotimod	-2.6071
55-48-1	Atropine sulfate	-2.6057
5908-99-6	Atropine sulfate monohydrate	-2.6057
51-55-8	Atropine	-2.6057
65666-07-1	Silymarin	-2.6047
31282-04-9	Hygromycin B	-2.6046
171599-83-0	Sildenafil citrate	-2.6042
155148-31-5	Plerixafor 8HCl (AMD3100 8HCl)	-2.6036
110078-46-1	Plerixafor	-2.6036
537-73-5	Isoferulic acid	-2.6035
562-10-7	Doxylamine succinate	-2.6031
40828-46-4	Suprofen	-2.6031
83-12-5	Phenylindione	-2.6013
775304-57-9	Ataluren	-2.5994
1225-55-4	Protriptyline hydrochloride	-2.5991
25389-94-0	Kanamycin sulfate	-2.599
183204-72-0	Tipiracil hydrochloride	-2.5983
144060-53-7	Febuxostat	-2.5978
55-48-1	Atropine sulfate	-2.5976
5908-99-6	Atropine sulfate monohydrate	-2.5976
51-55-8	Atropine	-2.5976
82248-59-7	Atomoxetine hydrochloride	-2.5973
114798-26-4	Losartan	-2.5971
74103-07-4	Ketorolac tromethamine salt	-2.5963
62-31-7	Dopamine hydrochloride	-2.5962
155148-31-5	Plerixafor 8HCl (AMD3100 8HCl)	-2.5951
110078-46-1	Plerixafor	-2.5951
135459-87-9	Strontium ranelate	-2.5944
86541-74-4	Benazepril hydrochloride	-2.5939
51481-61-9	Cimetidine	-2.5937
775304-57-9	Ataluren	-2.5927
2068-78-2	Vincristine sulfate	-2.5921
13171-25-0	Trimetazidine dihydrochloride	-2.5911
537-55-3	N-Acetyl-L-tyrosine	-2.5909
3254-89-5	Diphenidol hydrochloride	-2.5893
65666-07-1	Silymarin	-2.5887
59338-87-3	Alizapride hydrochloride	-2.5877
71675-85-9	Amisulpride	-2.5866
2068-78-2	Vincristine sulfate	-2.5865
29908-03-0	Ademetionine	-2.5858
389139-89-3	Omadacycline	-2.5849
64-77-7	Tolbutamide	-2.5845

4852-22-6	Proanthocyanidins	-2.5844
163253-35-8	Sitafloxacin Hydrate	-2.5839
26171-23-3	Tolmetin	-2.5838
380843-75-4	Bosutinib	-2.5824
4852-22-6	Proanthocyanidins	-2.5823
155148-31-5	Plerixafor 8HCl (AMD3100 8HCl)	-2.5818
110078-46-1	Plerixafor	-2.5818
147536-97-8	Bosentan	-2.5807
66357-59-3	Ranitidine Hydrochloride	-2.5807
87848-99-5	Acrivastine	-2.5801
82586-52-5	Moexipril HCl	-2.5798
1350514-68-9	Grazoprevir	-2.5794
980-71-2	Brompheniramine hydrogen maleate	-2.5792
1025967-78-5	Lifitegrast	-2.579
1038915-73-9	Niraparib tosylate	-2.5786
53179-09-2	Sisomicin Sulfate	-2.5785
23964-57-0	Articaine HCl	-2.5773
364-98-7	Diazoxide	-2.5771
29608-49-9	Almitrine dimesylate	-2.5764
934660-93-2	Cobimetinib	-2.5753
1611493-60-7	Bictegravir	-2.575
74103-07-4	Ketorolac tromethamine salt	-2.5746
1405-10-3	Neomycin sulfate	-2.5741
14976-57-9	Clemastine fumarate	-2.574
51-21-8	Fluorouracil	-2.5739
6035-45-6	Folinic Acid Calcium Salt Pentahydrate	-2.5724
132-20-7	Pheniramine maleate	-2.5719
33005-95-7	Tiaprofenic acid	-2.5717
22494-42-4	Diflunisal	-2.5711
1038915-60-4	Niraparib	-2.5708
1197953-54-0	Brigatinib	-2.5701
69056-38-8	Sapropterin Hydrochloride	-2.5689
155148-31-5	Plerixafor 8HCl (AMD3100 8HCl)	-2.5676
110078-46-1	Plerixafor	-2.5676
6035-45-6	Folinic Acid Calcium Salt Pentahydrate	-2.5667
31282-04-9	Hygromycin B	-2.5667
1115-70-4	Metformin hydrochloride	-2.5649
125317-39-7	Vinorelbine Tartrate	-2.5649

1231930-82-7	LY2835219	-2.5637
56296-78-7	Fluoxetine hydrochloride	-2.5634
54910-89-3	Fluoxetine	-2.5634
154-69-8	Tripelennamine hydrochloride	-2.5633
1405-41-0	Gentamycin sulfate	-2.5632
67-43-6	Pentetic Acid	-2.5626
26027-38-3	Nonoxynol	-2.5592
96847-55-1	Levomilnacipran	-2.5583
58947-95-8	DL-trihexyphenidyl hydrochloride	-2.5583
459789-99-2	Obeticholic Acid	-2.5568
536-43-6	Dyclonine hydrochloride	-2.5562
737789-87-6	Relugolix	-2.5555
33005-95-7	Tiaprofenic acid	-2.5553
389139-89-3	Omadacycline	-2.555
23964-57-0	Articaine HCl	-2.5544
6078-17-7	Berbamine dihydrochloride	-2.5543
1254053-43-4	Gilteritinib	-2.5536
872728-81-9	Dabigatran Etexilate Mesylate	-2.5531
211915-06-9	Dabigatran etexilate	-2.5531
32986-56-4	Tobramycin	-2.5525
71675-85-9	Amisulpride	-2.5515
1207456-01-6	Talazoparib	-2.5499
16676-29-2	Naltrexone hydrochloride	-2.5495
59338-87-3	Alizapride hydrochloride	-2.5495
1115-70-4	Metformin hydrochloride	-2.549
53-86-1	Indometacin	-2.5489
51-24-1	Tiratricol	-2.5488
138452-21-8	Fexofenadine hydrochloride	-2.5482
67-43-6	Pentetic Acid	-2.5473
99300-78-4	Venlafaxine hydrochloride	-2.5448
220991-20-8	Lumiracoxib	-2.5443
58947-95-8	DL-trihexyphenidyl hydrochloride	-2.5429
4394-00-7	Niflumic acid	-2.5429
4852-22-6	Proanthocyanidins	-2.5425
51481-61-9	Cimetidine	-2.5419
65666-07-1	Silymarin	-2.541
6170-42-9	Chloropyramine hydrochloride	-2.5388
302-79-4	Tretinoin	-2.5375
446-86-6	Azathioprine	-2.5371
98079-52-8	Lomefloxacin hydrochloride	-2.5369

98079-51-7	Lomefloxacin	-2.5369
1197953-54-0	Brigatinib	-2.5361
51-24-1	Tiratricol	-2.535
149003-01-0	Dexrazoxane HCl	-2.5332
552-94-3	Sasapyrine	-2.5324
29908-03-0	Ademetionine	-2.5316
78967-07-4	Mofezolac	-2.5311
68767-14-6	Loxoprofen	-2.5307
76584-70-8	Divalproex Sodium	-2.5302
17696-69-4	Hematoporphyrin dihydrochloride	-2.53
59-33-6	Pyrilamine maleate	-2.5268
155148-31-5	Plerixafor 8HCl (AMD3100 8HCl)	-2.5258
110078-46-1	Plerixafor	-2.5258
68497-62-1	Pramiracetam	-2.5212
138452-21-8	Fexofenadine hydrochloride	-2.5204
132-60-5	Cinchophen	-2.5201
63-45-6	Primaquine diphosphate	-2.5199
59-33-6	Pyrilamine maleate	-2.5198
172152-50-0	Ilaprazole sodium	-2.5193
22368-21-4	Eupatilin	-2.5189
375815-87-5	Varenicline Tartrate	-2.5185
1981-58-4	Sulfamethazine sodium salt	-2.5167
4852-22-6	Proanthocyanidins	-2.5161
56391-57-2	Netilmicin sulfate	-2.5159
33005-95-7	Tiaprofenic acid	-2.5149
98079-52-8	Lomefloxacin hydrochloride	-2.5141
98079-51-7	Lomefloxacin	-2.5141
145040-37-5	Candesartan Cilexetil	-2.5128
99300-78-4	Venlafaxine hydrochloride	-2.5126
56-69-9	5-hydroxytryptophan	-2.5116
4852-22-6	Proanthocyanidins	-2.5112
550-24-3	Embelin	-2.5101
73816-42-9	Meclocycline sulfosalicylate salt	-2.5094
808118-40-3	Roxadustat	-2.5088
31282-04-9	Hygromycin B	-2.5081
6101-15-1	Succinylcholine Chloride Dihydrate	-2.5062
2203-97-6	Hydrocortisone 21-hemisuccinate	-2.5058
40828-46-4	Suprofen	-2.5046
5451-09-2	5-Aminolevulinic acid hydrochloride	-2.5014
2398-81-4	Nicotinic acid N-oxide	-2.5009
52432-72-1	Oxeladin citrate	-2.5
59338-87-3	Alizapride hydrochloride	-2.4999

4852-22-6	Proanthocyanidins	-2.4969
4852-22-6	Proanthocyanidins	-2.4966
77-67-8	Ethosuximide	-2.4964
17696-69-4	Hematoporphyrin dihydrochloride	-2.4962
58-28-6	Desipramine hydrochloride	-2.4962
7681-93-8	Pimaricin	-2.4956
96847-55-1	Levomilnacipran	-2.4935
101152-94-7	Milnacipran hydrochloride	-2.4932
90098-04-7	Rebamipide	-2.4914
29908-03-0	Ademetionine	-2.4908
56-89-3	cystine	-2.4883
6170-42-9	Chloropyramine hydrochloride	-2.488
172152-50-0	Ilaprazole sodium	-2.4871
58947-95-8	DL-trihexyphenidyl hydrochloride	-2.4867
987-78-0	Citicoline	-2.4864
1197953-54-0	Brigatinib	-2.4837
97682-44-5	Irinotecan	-2.4827
67656-30-8	10-Hydroxycampothecin	-2.481
19685-09-7	10-Hydroxycamptothecin	-2.481
132-17-2	Benztropine mesylate	-2.4806
775304-57-9	Ataluren	-2.4803
136572-09-3	Irinotecan hydrochloride trihydrate	-2.4803
562-10-7	Doxylamine succinate	-2.4782
101152-94-7	Milnacipran hydrochloride	-2.4779
29908-03-0	Ademetionine	-2.4773
459789-99-2	Obeticholic Acid	-2.4759
56391-57-2	Netilmicin sulfate	-2.4757
144689-63-4	Olmesartan Medoxomil	-2.4753
123040-16-4	Azasetron hydrochloride	-2.4738
1476-53-5	Novobiocin Sodium	-2.473
138452-21-8	Fexofenadine hydrochloride	-2.4723
4759-48-2	Isotretinoin	-2.4696
16485-10-2	Panthenol	-2.4635
127294-70-6	Balofloxacin	-2.4615
4852-22-6	Proanthocyanidins	-2.4598
164579-32-2	Pantoprazole Sodium Hydrate	-2.4595
65666-07-1	Silymarin	-2.4595
56391-57-2	Netilmicin sulfate	-2.4584
10347-81-6	Maprotiline hydrochloride	-2.4581
872728-81-9	Dabigatran Etexilate Mesylate	-2.4566
211915-06-9	Dabigatran etexilate	-2.4566
4852-22-6	Proanthocyanidins	-2.4564

23964-57-0	Articaine HCl	-2.4563
4093-35-0	Bromopride	-2.456
70797-11-4	Cefpiramide acid	-2.4557
51-52-5	Propylthiouracil	-2.455
5053-08-7	Fenspiride hydrochloride	-2.4543
4852-22-6	Proanthocyanidins	-2.4542
6035-45-6	Folinic Acid Calcium Salt Pentahydrate	-2.454
96-50-4	Aminothiazole	-2.4539
92-61-5	Scopoletin	-2.4537
29908-03-0	Ademetionine	-2.4535
97682-44-5	Irinotecan	-2.4532
22161-81-5	(S)-(+)-Ketoprofen	-2.4518
22071-15-4	Ketoprofen	-2.4518
302-79-4	Tretinoin	-2.4513
123040-16-4	Azasetron hydrochloride	-2.4512
136572-09-3	Irinotecan hydrochloride trihydrate	-2.4511
56-81-5	Glycerol	-2.4489
62-31-7	Dopamine hydrochloride	-2.4475
74103-07-4	Ketorolac tromethamine salt	-2.447
53179-09-2	Sisomicin Sulfate	-2.4469
747-36-4	Hydroxychloroquine sulfate	-2.4469
96847-55-1	Levomilnacipran	-2.4449
56-92-8	Histamine 2HCl	-2.4434
51-45-6	Histamine	-2.4434
25389-94-0	Kanamycin sulfate	-2.4423
83-12-5	Phenylindione	-2.4409
4852-22-6	Proanthocyanidins	-2.4401
183204-72-0	Tipiracil hydrochloride	-2.4394
537-55-3	N-Acetyl-L-tyrosine	-2.4367
51-52-5	Propylthiouracil	-2.4361
96-50-4	Aminothiazole	-2.4344
116-43-8	Succinylsulfathiazole	-2.4341
552-94-3	Sasapyrine	-2.434
2068-78-2	Vincristine sulfate	-2.4336
1070-11-7	Ethambutol dihydrochloride	-2.4326
4852-22-6	Proanthocyanidins	-2.4324
62013-04-1	Dirithromycin	-2.4324
1009119-64-5	Daclatasvir	-2.431
74103-07-4	Ketorolac tromethamine salt	-2.4307
132-17-2	Benztropine mesylate	-2.4301
191217-81-9	Pramipexole 2HCl Monohydrate	-2.4291
71125-38-7	Meloxicam	-2.4288

5053-08-7	Fenspiride hydrochloride	-2.4266
112887-68-0	Raltitrexed	-2.4265
58895-64-0	Nalmefene hydrochloride	-2.4257
755038-65-4	Volasertib	-2.4255
29908-03-0	Ademetionine	-2.4247
56296-78-7	Fluoxetine hydrochloride	-2.4236
54910-89-3	Fluoxetine	-2.4236
81-23-2	Dehydrocholic acid	-2.4218
1350514-68-9	Grazoprevir	-2.421
172152-50-0	Ilaprazole sodium	-2.4185
1405-41-0	Gentamycin sulfate	-2.4183
4852-22-6	Proanthocyanidins	-2.4172
90098-04-7	Rebamipide	-2.4169
17696-69-4	Hematoporphyrin dihydrochloride	-2.4166
5560-59-8	Alverine citrate	-2.4163
33005-95-7	Tiaprofenic acid	-2.4144
639089-54-6	Tozasertib	-2.4139
6101-15-1	Succinylcholine Chloride Dihydrate	-2.4121
103766-25-2	Gimeracil	-2.4097
112-80-1	Oleic acid	-2.409
220620-09-7	Tigecycline	-2.4084
4852-22-6	Proanthocyanidins	-2.4077
987-78-0	Citicoline	-2.4076
88495-63-0	Artesunate	-2.4068
987-78-0	Citicoline	-2.4067
37025-55-1	Carbetocin	-2.4062
125317-39-7	Vinorelbine Tartrate	-2.406
73536-69-3	Bifendatatum	-2.4051
1405-41-0	Gentamycin sulfate	-2.4046
60-54-8	Tetracycline	-2.4033
4759-48-2	Isotretinoin	-2.4028
115939-25-8	Salvianolic acid B	-2.4025
29908-03-0	Ademetionine	-2.4019
76584-70-8	Divalproex Sodium	-2.4006
111902-57-9	Temocapil	-2.3995
867-81-2	D-Pantothenic acid sodium salt	-2.398
155148-31-5	Plerixafor 8HCl (AMD3100 8HCl)	-2.3978
110078-46-1	Plerixafor	-2.3978
56-92-8	Histamine 2HCl	-2.397
51-45-6	Histamine	-2.397
69558-55-0	Thymopentin	-2.3958
82586-55-8	Quinapril hydrochloride	-2.3957

96847-55-1	Levomilnacipran	-2.3954
78712-43-3	Ozagrel hydrochloride	-2.3954
67-43-6	Pentetic Acid	-2.3952
82571-53-7	Ozagrel	-2.3933
50924-49-7	Mizoribine	-2.3927
94-26-8	Butylparaben	-2.3919
4852-22-6	Proanthocyanidins	-2.3919
1374356-45-2	(S)-crizotinib	-2.3908
1405-37-4	Capastat sulfate	-2.39
614-39-1	Procainamide hydrochloride	-2.3895
749-02-0	Spiperone	-2.3888
70-70-2	Vanatone	-2.3885
54-96-6	Amifampridine	-2.3879
58895-64-0	Nalmefene hydrochloride	-2.3875
29908-03-0	Ademetionine	-2.387
89796-99-6	Aceclofenac	-2.3865
141626-36-0	Dronedarone	-2.3857
56296-78-7	Fluoxetine hydrochloride	-2.3854
54910-89-3	Fluoxetine	-2.3854
51-52-5	Propylthiouracil	-2.3846
639089-54-6	Tozasertib	-2.3845
101152-94-7	Milnacipran hydrochloride	-2.3843
141625-93-6	Dronedarone hydrochloride	-2.383
29868-97-1	Pirenzepine dihydrochloride	-2.3829
1025967-78-5	Lifitegrast	-2.3828
1197953-54-0	Brigatinib	-2.3821
139481-59-7	Candesartan	-2.382
16676-29-2	Naltrexone hydrochloride	-2.3819
132-20-7	Pheniramine maleate	-2.3819
50-01-1	Guanidine hydrochloride	-2.3815
441798-33-0	Macitentan	-2.3797
1405-41-0	Gentamycin sulfate	-2.3795
22199-08-2	Silver sulfadiazine	-2.3794
53179-09-2	Sisomicin Sulfate	-2.3776
147127-20-6	Tenofovir	-2.3765
145040-37-5	Candesartan Cilexetil	-2.3763
1350514-68-9	Grazoprevir	-2.376
2398-81-4	Nicotinic acid N-oxide	-2.3726
562-10-7	Doxylamine succinate	-2.372
141626-36-0	Dronedarone	-2.3718

4759-48-2	Isotretinoin	-2.3707
141625-93-6	Dronedarone hydrochloride	-2.37
65-85-0	Benzoic acid	-2.3698
78712-43-3	Ozagrel hydrochloride	-2.3684
82571-53-7	Ozagrel	-2.3678
50-01-1	Guanidine hydrochloride	-2.3674
987-78-0	Citicoline	-2.3661
151533-22-1	Levomefolate calcium	-2.3659
151533-22-1	Levomefolate calcium	-2.3652
21256-18-8	Oxaprozin	-2.3647
151533-22-1	Levomefolate calcium	-2.3646
4852-22-6	Proanthocyanidins	-2.3636
73816-42-9	Meclocycline sulfosalicylate salt	-2.3631
132-20-7	Pheniramine maleate	-2.3624
62893-19-0	Cefoperazone	-2.3622
4852-22-6	Proanthocyanidins	-2.3619
552-94-3	Sasapyrine	-2.3618
54-30-8	Camylofin	-2.3609
16676-29-2	Naltrexone hydrochloride	-2.3609
135459-87-9	Strontium ranelate	-2.3601
4852-22-6	Proanthocyanidins	-2.3589
73816-42-9	Meclocycline sulfosalicylate salt	-2.3561
88495-63-0	Artesunate	-2.3553
1476-53-5	Novobiocin Sodium	-2.3547
867-81-2	D-Pantothenic acid sodium salt	-2.3512
1197953-54-0	Brigatinib	-2.3505
127294-70-6	Balofloxacin	-2.3504
59804-37-4	Tenoxicam	-2.3491
16485-10-2	Panthenol	-2.3486
65666-07-1	Silymarin	-2.3484
29908-03-0	Ademetionine	-2.3481
737789-87-6	Relugolix	-2.348
4852-22-6	Proanthocyanidins	-2.3479
65666-07-1	Silymarin	-2.3466
22199-08-2	Silver sulfadiazine	-2.3462
1354744-91-4	Naloxegol oxalate	-2.3457
78964-85-9	Fosfomycin Tromethamine	-2.3436
77-86-1	Trometamol	-2.3436
50924-49-7	Mizoribine	-2.3425
441798-33-0	Macitentan	-2.3421
81-13-0	Dexpanthenol	-2.3393

6078-17-7	Berbamine dihydrochloride	-2.3381
63-45-6	Primaquine diphosphate	-2.3378
867-81-2	D-Pantothenic acid sodium salt	-2.337
111902-57-9	Temocapil	-2.3364
1405-37-4	Capastat sulfate	-2.3364
1025967-78-5	Lifitegrast	-2.3355
6078-17-7	Berbamine dihydrochloride	-2.3333
25389-94-0	Kanamycin sulfate	-2.3317
70476-82-3	Mitoxantrone hydrochloride	-2.3316
73816-42-9	Meclocycline sulfosalicylate salt	-2.33
65271-80-9	Mitoxantrone	-2.3289
155148-31-5	Plerixafor 8HCl (AMD3100 8HCl)	-2.3259
110078-46-1	Plerixafor	-2.3259
56-81-5	Glycerol	-2.3237
4852-22-6	Proanthocyanidins	-2.3229
70-70-2	Vanatone	-2.3216
850876-88-9	Danoprevir	-2.3209
56391-57-2	Netilmicin sulfate	-2.3203
4852-22-6	Proanthocyanidins	-2.3176
81-23-2	Dehydrocholic acid	-2.3163
357-08-4	Naloxone hydrochloride	-2.3152
3963-95-9	Methacycline hydrochloride	-2.3148
63-45-6	Primaquine diphosphate	-2.3146
1115-70-4	Metformin hydrochloride	-2.314
78712-43-3	Ozagrel hydrochloride	-2.3116
94-26-8	Butylparaben	-2.3111
112887-68-0	Raltitrexed	-2.3105
72-14-0	Sulfathiazole	-2.3097
155148-31-5	Plerixafor 8HCl (AMD3100 8HCl)	-2.3093
110078-46-1	Plerixafor	-2.3093
14976-57-9	Clemastine fumarate	-2.3086
149-91-7	Gallic acid	-2.3085
737789-87-6	Relugolix	-2.3084
82571-53-7	Ozagrel	-2.3062
1492-18-8	Calcium folinate	-2.3049
58895-64-0	Nalmefene hydrochloride	-2.3047
77-67-8	Ethosuximide	-2.3038
4852-22-6	Proanthocyanidins	-2.3035
164579-32-2	Pantoprazole Sodium Hydrate	-2.3011
357-08-4	Naloxone hydrochloride	-2.3007
1492-18-8	Calcium folinate	-2.3005
537-55-3	N-Acetyl-L-tyrosine	-2.3003

339-72-0	L-Cycloserine	-2.2975
67227-57-0	Fenoldopam mesylate	-2.2967
1405-37-4	Capastat sulfate	-2.2966
357-08-4	Naloxone hydrochloride	-2.2964
951395-08-7	tafamidis meglumine	-2.2955
10347-81-6	Maprotiline hydrochloride	-2.2942
107007-99-8	Granisetron hydrochloride	-2.2934
120-47-8	Ethylparaben	-2.2932
1197953-54-0	Brigatinib	-2.2927
290297-26-6	Netupitant	-2.2925
71675-85-9	Amisulpride	-2.2919
72-14-0	Sulfathiazole	-2.2915
29908-03-0	Ademetionine	-2.2915
4852-22-6	Proanthocyanidins	-2.2904
96-50-4	Aminothiazole	-2.2868
304-84-7	Ethamivan	-2.2858
56-41-7	alanine	-2.2844
51481-61-9	Cimetidine	-2.2836
1346242-81-6	Erdafitinib	-2.2825
73816-42-9	Meclocycline sulfosalicylate salt	-2.2816
50-55-5	Reserpine	-2.2813
2259-96-3	Cyclothiazide	-2.2794
357-08-4	Naloxone hydrochloride	-2.2789
290297-26-6	Netupitant	-2.2788
64-77-7	Tolbutamide	-2.2771
29868-97-1	Pirenzepine dihydrochloride	-2.2765
56-81-5	Glycerol	-2.2758
1374356-45-2	(S)-crizotinib	-2.2757
614-39-1	Procainamide hydrochloride	-2.2754
149-91-7	Gallic acid	-2.2753
15663-27-1	Cisplatin	-2.2753
1197953-54-0	Brigatinib	-2.2721
71675-85-9	Amisulpride	-2.2718
1981-58-4	Sulfamethazine sodium salt	-2.2698
1231930-82-7	LY2835219	-2.2693
131707-23-8	Arbidol hydrochloride	-2.2687
25389-94-0	Kanamycin sulfate	-2.2687
13614-98-7	Minocycline hydrochloride	-2.2678
375815-87-5	Varenicline Tartrate	-2.2672

97240-79-4	Topiramate	-2.2668
13614-98-7	Minocycline hydrochloride	-2.2616
321-30-2	Adenine sulfate	-2.2614
73-24-5	Adenine	-2.2608
114798-26-4	Losartan	-2.2597
154-69-8	Tripelennamine hydrochloride	-2.2592
124750-99-8	Losartan potassium	-2.2583
3963-95-9	Methacycline hydrochloride	-2.2581
62571-86-2	Captopril	-2.2573
36322-90-4	Piroxicam	-2.2562
120-47-8	Ethylparaben	-2.254
141-90-2	Deracil	-2.2514
4093-35-0	Bromopride	-2.2503
59721-29-8	Camostat mesilate	-2.2489
1070-11-7	Ethambutol dihydrochloride	-2.2479
4852-22-6	Proanthocyanidins	-2.2477
147084-10-4	Alcaftadine	-2.2477
56391-57-2	Netilmicin sulfate	-2.2456
29908-03-0	Ademetionine	-2.2456
141-90-2	Deracil	-2.2447
117772-70-0	Azithromycin dihydrate	-2.244
1025687-58-4	R788 Disodium	-2.2439
936091-26-8	Fedratinib	-2.2434
5875-06-9	Proparacaine hydrochloride	-2.243
1405-37-4	Capastat sulfate	-2.242
59338-87-3	Alizapride hydrochloride	-2.2419
155148-31-5	Plerixafor 8HCl (AMD3100 8HCl)	-2.2414
110078-46-1	Plerixafor	-2.2414
29908-03-0	Ademetionine	-2.2408
56-04-2	Methylthiouracil	-2.2394
149-91-7	Gallic acid	-2.2393
138452-21-8	Fexofenadine hydrochloride	-2.2384
1197953-54-0	Brigatinib	-2.2373
6035-45-6	Folinic Acid Calcium Salt Pentahydrate	-2.2359
459789-99-2	Obeticholic Acid	-2.2349
83-12-5	Phenylindione	-2.2337
1231930-82-7	LY2835219	-2.2333
6035-45-6	Folinic Acid Calcium Salt Pentahydrate	-2.2322
808118-40-3	Roxadustat	-2.23
552-94-3	Sasapyrine	-2.2295

59721-29-8	Camostat mesilate	-2.2277
53179-09-2	Sisomicin Sulfate	-2.2247
29908-03-0	Ademetionine	-2.2246
14976-57-9	Clemastine fumarate	-2.2242
69558-55-0	Thymopentin	-2.2217
191217-81-9	Pramipexole 2HCl Monohydrate	-2.2212
67-43-6	Pentetic Acid	-2.221
1405-37-4	Capastat sulfate	-2.2199
82248-59-7	Atomoxetine hydrochloride	-2.2197
50264-69-2	Lonidamine	-2.2197
72-14-0	Sulfathiazole	-2.2191
50-55-5	Reserpine	-2.2157
141-94-6	Hexetidine	-2.2147
21256-18-8	Oxaprozin	-2.2145
56391-57-2	Netilmicin sulfate	-2.2144
1032900-25-6	LDK378	-2.2141
4852-22-6	Proanthocyanidins	-2.2119
357-08-4	Naloxone hydrochloride	-2.2097
210353-53-0	Gemifloxacin?mesylate	-2.2093
1492-18-8	Calcium folinate	-2.209
112887-68-0	Raltitrexed	-2.2084
123040-16-4	Azasetron hydrochloride	-2.2082
4852-22-6	Proanthocyanidins	-2.2081
104376-79-6	Ceftriaxone sodium	-2.2053
81-27-6	Sennoside A	-2.2033
104376-79-6	Ceftriaxone sodium	-2.2019
94-26-8	Butylparaben	-2.2017
90098-04-7	Rebamipide	-2.2013
1492-18-8	Calcium folinate	-2.2006
59-33-6	Pyrilamine maleate	-2.1998
17737-65-4	Clonixin	-2.1996
2068-78-2	Vincristine sulfate	-2.1993
62571-86-2	Captopril	-2.199
4852-22-6	Proanthocyanidins	-2.1986
4852-22-6	Proanthocyanidins	-2.1983
138452-21-8	Fexofenadine hydrochloride	-2.1974
5560-59-8	Alverine citrate	-2.1952
951395-08-7	tafamidis meglumine	-2.1946
71675-85-9	Amisulpride	-2.1945
951395-08-7	tafamidis meglumine	-2.1935
5451-09-2	5-Aminolevulinic acid hydrochloride	-2.1906
54-96-6	Amifampridine	-2.1889

1492-18-8	Calcium folinate	-2.1886
4852-22-6	Proanthocyanidins	-2.1841
951395-08-7	tafamidis meglumine	-2.1835
154-69-8	Tripelennamine hydrochloride	-2.1827
67814-76-0	Isepamicin sulfate	-2.1816
577778-58-6	Topiroxostat	-2.1808
58895-64-0	Nalmefene hydrochloride	-2.1804
6101-15-1	Succinylcholine Chloride Dihydrate	-2.1779
29908-03-0	Ademetionine	-2.1759
951395-08-7	tafamidis meglumine	-2.1758
4852-22-6	Proanthocyanidins	-2.1753
56-04-2	Methylthiouracil	-2.1735
84611-23-4	Erdosteine	-2.1712
99300-78-4	Venlafaxine hydrochloride	-2.1711
151533-22-1	Levomefolate calcium	-2.1704
138402-11-6	Irbesartan	-2.1686
29908-03-0	Ademetionine	-2.1644
76824-35-6	Famotidine	-2.1621
141-90-2	Deracil	-2.1618
57-66-9	Probenecid	-2.1618
123040-16-4	Azasetron hydrochloride	-2.1611
84611-23-4	Erdosteine	-2.161
639089-54-6	Tozasertib	-2.1605
191217-81-9	Pramipexole 2HCl Monohydrate	-2.1584
1476-53-5	Novobiocin Sodium	-2.1579
30751-05-4	Troxipide	-2.1568
5875-06-9	Proparacaine hydrochloride	-2.1563
755038-65-4	Volasertib	-2.156
32795-44-1	Acecainide	-2.1556
65666-07-1	Silymarin	-2.1545
29868-97-1	Pirenzepine dihydrochloride	-2.1538
29908-03-0	Ademetionine	-2.152
639089-54-6	Tozasertib	-2.1516
951395-08-7	tafamidis meglumine	-2.1492
3254-89-5	Diphenidol hydrochloride	-2.1482
104376-79-6	Ceftriaxone sodium	-2.1475
70476-82-3	Mitoxantrone hydrochloride	-2.1472
120-47-8	Ethylparaben	-2.1459
1449-05-4	Glycyrrhetinic acid	-2.1459
56-41-7	alanine	-2.1441
65271-80-9	Mitoxantrone	-2.1436
7681-93-8	Pimaricin	-2.1431
125-33-7	Primidone	-2.1418

128-13-2	Ursodeoxycholic acid	-2.1409
50924-49-7	Mizoribine	-2.1408
50924-49-7	Mizoribine	-2.1406
755038-65-4	Volasertib	-2.1405
115939-25-8	Salvianolic acid B	-2.14
59-67-6	Nicotinic acid	-2.1387
71125-38-7	Meloxicam	-2.1386
577778-58-6	Topiroxostat	-2.1351
190786-44-8	Bepotastine Besilate	-2.1351
562-10-7	Doxylamine succinate	-2.1342
315-30-0	Allopurinol	-2.1332
99300-78-4	Venlafaxine hydrochloride	-2.1322
81-23-2	Dehydrocholic acid	-2.1292
81-27-6	Sennoside A	-2.1288
562-10-7	Doxylamine succinate	-2.1273
145040-37-5	Candesartan Cilexetil	-2.1267
73816-42-9	Meclocycline sulfosalicylate salt	-2.1265
172152-50-0	Ilaprazole sodium	-2.1231
54-31-9	Furosemide	-2.123
29868-97-1	Pirenzepine dihydrochloride	-2.1226
90098-04-7	Rebamipide	-2.1219
29908-03-0	Ademetionine	-2.1218
78964-85-9	Fosfomycin Tromethamine	-2.1195
77-86-1	Trometamol	-2.1195
130929-57-6	Entacapone	-2.1191
58895-64-0	Nalmefene hydrochloride	-2.1189
149003-01-0	Dexrazoxane HCl	-2.1187
125-33-7	Primidone	-2.1146
155148-31-5	Plerixafor 8HCl (AMD3100 8HCl)	-2.1129
110078-46-1	Plerixafor	-2.1129
127373-66-4	Sivelestat	-2.1111
1188-38-1	Carbaglu	-2.1088
62571-86-2	Captopril	-2.1044
83-44-3	Deoxycholic acid	-2.1021
6035-45-6	Folinic Acid Calcium Salt Pentahydrate	-2.0981
23651-95-8	Droxidopa	-2.0963
53179-09-2	Sisomicin Sulfate	-2.0962
473-98-3	Betulin	-2.0959
78964-85-9	Fosfomycin Tromethamine	-2.0941
77-86-1	Trometamol	-2.0941
1354744-91-4	Naloxegol oxalate	-2.0931
153504-70-2	Cevimeline hydrochloride hemihydrate	-2.0925

125-33-7	Primidone	-2.0919
29908-03-0	Ademetionine	-2.0917
71675-85-9	Amisulpride	-2.0913
96847-55-1	Levomilnacipran	-2.0908
773092-05-0	Acotiamide hydrochloride	-2.0876
364622-82-2	Doripenem Hydrate	-2.0863
114798-26-4	Losartan	-2.0843
151533-22-1	Levomefolate calcium	-2.0836
850876-88-9	Danoprevir	-2.0822
5875-06-9	Proparacaine hydrochloride	-2.0802
1449-05-4	Glycyrrhetinic acid	-2.0794
357-08-4	Naloxone hydrochloride	-2.0788
4852-22-6	Proanthocyanidins	-2.0781
81-23-2	Dehydrocholic acid	-2.0732
164579-32-2	Pantoprazole Sodium Hydrate	-2.0721
59-67-6	Nicotinic acid	-2.0711
103766-25-2	Gimeracil	-2.0709
78967-07-4	Mofezolac	-2.0708
6035-45-6	Folinic Acid Calcium Salt Pentahydrate	-2.0699
29908-03-0	Ademetionine	-2.069
115939-25-8	Salvianolic acid B	-2.0678
1231930-82-7	LY2835219	-2.0669
73816-42-9	Meclocycline sulfosalicylate salt	-2.0655
951395-08-7	tafamidis meglumine	-2.0644
140462-76-6	Olopatadine hydrochloride	-2.0638
68-41-7	D-Cycloserine	-2.0635
375815-87-5	Varenicline Tartrate	-2.0578
97240-79-4	Topiramate	-2.0577
464-92-6	Asiatic acid	-2.0575
570406-98-3	Avatrombopag	-2.0568
83881-52-1	Cetirizine dihydrochloride	-2.0542
52-66-4	DL-Penicillamine	-2.0526
52-67-5	d-penicillamin	-2.0517
304-84-7	Ethamivan	-2.0496
224452-66-8	Retapamulin	-2.0494
315-30-0	Allopurinol	-2.0482
1405-41-0	Gentamycin sulfate	-2.0451
16485-10-2	Panthenol	-2.0451
82248-59-7	Atomoxetine hydrochloride	-2.0447
53179-09-2	Sisomicin Sulfate	-2.0422
56-40-6	glycine	-2.0417
14783-68-7	Magnesium glycinate	-2.041

20150-34-9	Ferrous Bisglycinate	-2.041
112887-68-0	Raltitrexed	-2.0399
23651-95-8	Droxidopa	-2.0392
951395-08-7	tafamidis meglumine	-2.0391
79-57-2	Oxytetracycline	-2.0348
639089-54-6	Tozasertib	-2.0345
123040-16-4	Azasetron hydrochloride	-2.0344
80433-71-2	Calcium Levofolinate	-2.0324
1038915-73-9	Niraparib tosylate	-2.032
153559-49-0	Bexarotene	-2.0319
155148-31-5	Plerixafor 8HCl (AMD3100 8HCl)	-2.0317
110078-46-1	Plerixafor	-2.0317
1038915-60-4	Niraparib	-2.0314
6078-17-7	Berbamine dihydrochloride	-2.0309
321-30-2	Adenine sulfate	-2.0295
73-24-5	Adenine	-2.0295
155148-31-5	Plerixafor 8HCl (AMD3100 8HCl)	-2.0291
110078-46-1	Plerixafor	-2.0291
155148-31-5	Plerixafor 8HCl (AMD3100 8HCl)	-2.0266
110078-46-1	Plerixafor	-2.0266
1449-05-4	Glycyrrhetinic acid	-2.0256
32986-56-4	Tobramycin	-2.0199
69304-47-8	Brivudine	-2.0197
67814-76-0	Isepamicin sulfate	-2.0164
6078-17-7	Berbamine dihydrochloride	-2.0146
29908-03-0	Ademetionine	-2.0136
141-01-5	Ferrous fumarate	-2.013
614-39-1	Procainamide hydrochloride	-2.0125
70-70-2	Vanatone	-2.0119
153504-70-2	Cevimeline hydrochloride hemihydrate	-2.0117
110871-86-8	Sparfloxacin	-2.0115
127294-70-6	Balofloxacin	-2.0055
23651-95-8	Droxidopa	-2.004
6170-42-9	Chloropyramine hydrochloride	-2.0021
151533-22-1	Levomefolate calcium	-2.0012
773092-05-0	Acotiamide hydrochloride	-2.001
83905-01-5	Azithromycin	-2.0003
1025687-58-4	R788 Disodium	-1.9994
29908-03-0	Ademetionine	-1.9987
80433-71-2	Calcium Levofolinate	-1.9973

224452-66-8	Retapamulin	-1.996
50-78-2	Aspirin	-1.9923
190786-44-8	Bepotastine Besilate	-1.9912
951395-08-7	tafamidis meglumine	-1.9911
541-22-0	Decamethonium bromide	-1.9879
112-80-1	Oleic acid	-1.9874
69304-47-8	Brivudine	-1.9865
1197953-54-0	Brigatinib	-1.9845
6078-17-7	Berbamine dihydrochloride	-1.9839
755038-65-4	Volasertib	-1.9829
81-13-0	Dexpanthenol	-1.9808
67-43-6	Pentetic Acid	-1.9785
59338-87-3	Alizapride hydrochloride	-1.9785
5560-59-8	Alverine citrate	-1.9784
4852-22-6	Proanthocyanidins	-1.9774
63-45-6	Primaquine diphosphate	-1.9774
149003-01-0	Dexrazoxane HCl	-1.9768
10017-44-4	L-Carnitine hydrochloride	-1.9761
339-72-0	L-Cycloserine	-1.9735
877399-52-5	Crizotinib	-1.9716
901119-35-5	Fostamatinib	-1.9716
951395-08-7	tafamidis meglumine	-1.9688
56296-78-7	Fluoxetine hydrochloride	-1.9678
54910-89-3	Fluoxetine	-1.9678
1146-99-2	chlophenadione	-1.9648
140462-76-6	Olopatadine hydrochloride	-1.9636
56-69-9	5-hydroxytryptophan	-1.963
1070-11-7	Ethambutol dihydrochloride	-1.962
114977-28-5	Docetaxel	-1.9619
67-43-6	Pentetic Acid	-1.9617
1070-11-7	Ethambutol dihydrochloride	-1.9604
79-57-2	Oxytetracycline	-1.9592
29908-03-0	Ademetionine	-1.9585
364622-82-2	Doripenem Hydrate	-1.9583
6078-17-7	Berbamine dihydrochloride	-1.9566
1263-89-4	Paromomycin Sulfate	-1.9565
52-66-4	DL-Penicillamine	-1.9551
52-67-5	d-penicillamin	-1.9545
84611-23-4	Erdosteine	-1.9543
67-43-6	Pentetic Acid	-1.9535
127294-70-6	Balofloxacin	-1.9534
112-80-1	Oleic acid	-1.9498

67-43-6	Pentetic Acid	-1.9481
29908-03-0	Ademetionine	-1.9474
872728-81-9	Dabigatran Etexilate Mesylate	-1.9464
211915-06-9	Dabigatran etexilate	-1.9464
155148-31-5	Plerixafor 8HCl (AMD3100 8HCl)	-1.945
110078-46-1	Plerixafor	-1.945
52-66-4	DL-Penicillamine	-1.9447
52-67-5	d-penicillamin	-1.9438
29908-03-0	Ademetionine	-1.943
903576-44-3	Pitolisant hydrochloride	-1.9425
155148-31-5	Plerixafor 8HCl (AMD3100 8HCl)	-1.9407
110078-46-1	Plerixafor	-1.9407
27591-69-1	Tilorone dihydrochloride	-1.94
79-57-2	Oxytetracycline	-1.9399
6078-17-7	Berbamine dihydrochloride	-1.9391
67227-57-0	Fenoldopam mesylate	-1.9391
29908-03-0	Ademetionine	-1.9374
1207456-01-6	Talazoparib	-1.9366
50-55-5	Reserpine	-1.9354
247257-48-3	Fimasartan	-1.9343
67227-57-0	Fenoldopam mesylate	-1.9341
52-90-4	cysteine	-1.9339
951395-08-7	tafamidis meglumine	-1.9321
50-78-2	Aspirin	-1.9321
464-92-6	Asiatic acid	-1.9312
64-73-3	Demeclocycline hydrochloride	-1.9302
96847-55-1	Levomilnacipran	-1.9296
951395-08-7	tafamidis meglumine	-1.9271
125317-39-7	Vinorelbine Tartrate	-1.9268
68-41-7	D-Cycloserine	-1.9267
53164-05-9	Acemetacin	-1.9262
79-57-2	Oxytetracycline	-1.9249
57-09-0	Cetrimonium bromide	-1.9248
17737-65-4	Clonixin	-1.9237
79-57-2	Oxytetracycline	-1.9222
210353-53-0	Gemifloxacin?mesylate	-1.9213
536-43-6	Dyclonine hydrochloride	-1.9187
25389-94-0	Kanamycin sulfate	-1.9186
140462-76-6	Olopatadine hydrochloride	-1.9181
89778-27-8	Toremifene citrate	-1.9174
141-90-2	Deracil	-1.9173
89778-26-7	Toremifene	-1.9171

872728-81-9	Dabigatran Etexilate Mesylate	-1.916
211915-06-9	Dabigatran etexilate	-1.916
698387-09-6	Neratinib	-1.9145
903576-44-3	Pitolisant hydrochloride	-1.9107
7083-71-8	(+)-Emetine dihydrochloride hydrate	-1.9078
951395-08-7	tafamidis meglumine	-1.9074
773092-05-0	Acotiamide hydrochloride	-1.9061
68302-57-8	Amlexanox	-1.9056
68302-57-8	Amlexanox	-1.9037
76963-41-2	Nizatidine	-1.9031
82586-55-8	Quinapril hydrochloride	-1.9018
56-40-6	glycine	-1.8997
14783-68-7	Magnesium glycinate	-1.899
20150-34-9	Ferrous Bisglycinate	-1.899
1032900-25-6	LDK378	-1.8968
78964-85-9	Fosfomycin Tromethamine	-1.8963
77-86-1	Trometamol	-1.8963
54-96-6	Amifampridine	-1.8963
50-63-5	Chloroquine diphosphate	-1.8933
18699-02-0	Actarit	-1.8929
2068-78-2	Vincristine sulfate	-1.8928
79-57-2	Oxytetracycline	-1.8928
64-72-2	Chlortetracycline hydrochloride	-1.8918
155148-31-5	Plerixafor 8HCl (AMD3100 8HCl)	-1.8915
110078-46-1	Plerixafor	-1.8915
1188-38-1	Carbaglu	-1.8914
29908-03-0	Ademetionine	-1.8906
951395-08-7	tafamidis meglumine	-1.8902
67-43-6	Pentetic Acid	-1.8899
339-72-0	L-Cycloserine	-1.8876
115939-25-8	Salvianolic acid B	-1.8858
29216-28-2	Mequitazine	-1.8851
54-35-3	Procaine penicillin G	-1.8847
3963-95-9	Methacycline hydrochloride	-1.8846
67-43-6	Pentetic Acid	-1.8825
6078-17-7	Berbamine dihydrochloride	-1.8804
79-57-2	Oxytetracycline	-1.8797
81-23-2	Dehydrocholic acid	-1.8795
7083-71-8	(+)-Emetine dihydrochloride hydrate	-1.8793
951395-08-7	tafamidis meglumine	-1.8782
155148-31-5	Plerixafor 8HCl (AMD3100 8HCl)	-1.877
110078-46-1	Plerixafor	-1.877

29908-03-0	Ademetionine	-1.8765
13614-98-7	Minocycline hydrochloride	-1.8763
1263-89-4	Paromomycin Sulfate	-1.876
51-21-8	Fluorouracil	-1.8749
639089-54-6	Tozasertib	-1.8739
3963-95-9	Methacycline hydrochloride	-1.8737
936091-26-8	Fedratinib	-1.8719
4852-22-6	Proanthocyanidins	-1.8718
4852-22-6	Proanthocyanidins	-1.8715
4852-22-6	Proanthocyanidins	-1.8711
6078-17-7	Berbamine dihydrochloride	-1.8705
155148-31-5	Plerixafor 8HCl (AMD3100 8HCl)	-1.8662
110078-46-1	Plerixafor	-1.8662
59-67-6	Nicotinic acid	-1.866
97240-79-4	Topiramate	-1.8638
53797-35-6	Ribostamycin sulfate	-1.8629
189188-57-6	Tegaserod maleate	-1.8623
1207456-01-6	Talazoparib	-1.862
29908-03-0	Ademetionine	-1.8601
475086-01-2	Selexipag	-1.8594
1207456-01-6	Talazoparib	-1.8588
7083-71-8	(+)-Emetine dihydrochloride hydrate	-1.8576
951395-08-7	tafamidis meglumine	-1.8568
611-71-2	Mandelic acid	-1.8538
29908-03-0	Ademetionine	-1.853
202189-78-4	Bilastine	-1.8517
163253-35-8	Sitafloxacin Hydrate	-1.8513
4852-22-6	Proanthocyanidins	-1.8501
1263-89-4	Paromomycin Sulfate	-1.8466
138402-11-6	Irbesartan	-1.8464
220620-09-7	Tigecycline	-1.8451
79-57-2	Oxytetracycline	-1.8439
951395-08-7	tafamidis meglumine	-1.8434
67814-76-0	Isepamicin sulfate	-1.8433
64-73-3	Demeclocycline hydrochloride	-1.8408
147084-10-4	Alcaftadine	-1.8403
58-85-5	D-Biotin	-1.8394
1146-99-2	chlophenadione	-1.839
4852-22-6	Proanthocyanidins	-1.8385
76824-35-6	Famotidine	-1.8377
321-30-2	Adenine sulfate	-1.8369

73-24-5	Adenine	-1.8369
56-69-9	5-hydroxytryptophan	-1.8365
52-66-4	DL-Penicillamine	-1.8362
50264-69-2	Lonidamine	-1.836
50264-69-2	Lonidamine	-1.8358
29608-49-9	Almitrine dimesylate	-1.8356
64-72-2	Chlortetracycline hydrochloride	-1.8355
13189-98-5	Fudosteine	-1.835
147084-10-4	Alcaftadine	-1.8347
4852-22-6	Proanthocyanidins	-1.8346
78964-85-9	Fosfomycin Tromethamine	-1.8342
77-86-1	Trometamol	-1.8342
1207456-01-6	Talazoparib	-1.8333
67-43-6	Pentetic Acid	-1.8313
68497-62-1	Pramiracetam	-1.8299
145040-37-5	Candesartan Cilexetil	-1.8289
96847-55-1	Levomilnacipran	-1.8286
56-40-6	glycine	-1.8282
14783-68-7	Magnesium glycinate	-1.8275
20150-34-9	Ferrous Bisglycinate	-1.8275
1254053-43-4	Gilteritinib	-1.8274
73536-69-3	Bifendatatum	-1.8261
198470-84-7	Parecoxib	-1.8248
6078-17-7	Berbamine dihydrochloride	-1.8239
198470-84-7	Parecoxib	-1.8215
63-45-6	Primaquine diphosphate	-1.8184
81-13-0	Dexpanthenol	-1.8182
50924-49-7	Mizoribine	-1.818
52-66-4	DL-Penicillamine	-1.8174
117772-70-0	Azithromycin dihydrate	-1.8166
14919-77-8	Benserazide hydrochloride	-1.8163
6035-45-6	Folinic Acid Calcium Salt Pentahydrate	-1.8158
58895-64-0	Nalmefene hydrochloride	-1.8155
951395-08-7	tafamidis meglumine	-1.815
186826-86-8	Moxifloxacin hydrochloride	-1.8134
872728-81-9	Dabigatran Etexilate Mesylate	-1.8132
211915-06-9	Dabigatran etexilate	-1.8132
1405-37-4	Capastat sulfate	-1.8113
69304-47-8	Brivudine	-1.8109
290297-26-6	Netupitant	-1.8108
138402-11-6	Irbesartan	-1.8098

56-92-8	Histamine 2HCl	-1.809
51-45-6	Histamine	-1.809
68-41-7	D-Cycloserine	-1.8083
211915-06-9	Dabigatran etexilate	-1.8076
138-14-7	Deferoxamine Mesylate	-1.8067
22839-47-0	Aspartame	-1.8048
76824-35-6	Famotidine	-1.8045
51-21-8	Fluorouracil	-1.8044
59721-29-8	Camostat mesilate	-1.8037
951395-08-7	tafamidis meglumine	-1.7999
198470-84-7	Parecoxib	-1.7997
83905-01-5	Azithromycin	-1.7995
67-43-6	Pentetic Acid	-1.7985
951395-08-7	tafamidis meglumine	-1.7964
79-57-2	Oxytetracycline	-1.7934
639089-54-6	Tozasertib	-1.7928
84611-23-4	Erdosteine	-1.7926
79-57-2	Oxytetracycline	-1.7919
3963-95-9	Methacycline hydrochloride	-1.791
50-63-5	Chloroquine diphosphate	-1.7892
29908-03-0	Ademetionine	-1.7889
6035-45-6	Folinic Acid Calcium Salt Pentahydrate	-1.7868
53797-35-6	Ribostamycin sulfate	-1.7864
153504-70-2	Cevimeline hydrochloride hemihydrate	-1.7851
1254053-43-4	Gilteritinib	-1.7851
611-71-2	Mandelic acid	-1.7847
405165-61-9	Besifloxacin HCl	-1.783
54-31-9	Furosemide	-1.7767
951395-08-7	tafamidis meglumine	-1.7712
29908-03-0	Ademetionine	-1.7707
59-92-7	Levodopa	-1.7702
6078-17-7	Berbamine dihydrochloride	-1.7693
131707-23-8	Arbidol hydrochloride	-1.7673
1492-18-8	Calcium folinate	-1.7661
67227-57-0	Fenoldopam mesylate	-1.7656
83881-52-1	Cetirizine dihydrochloride	-1.765
951395-08-7	tafamidis meglumine	-1.7626
38194-50-2	Sulindac	-1.7584
901119-35-5	Fostamatinib	-1.7572
151533-22-1	Levomefolate calcium	-1.757
64-72-2	Chlortetracycline hydrochloride	-1.7562
50924-49-7	Mizoribine	-1.756

1937-19-5	Aminoguanidine hydrochloride	-1.7559
31282-04-9	Hygromycin B	-1.7555
54-31-9	Furosemide	-1.7546
4852-22-6	Proanthocyanidins	-1.7534
67-43-6	Pentetic Acid	-1.7518
64-72-2	Chlortetracycline hydrochloride	-1.7502
59-92-7	Levodopa	-1.75
50-78-2	Aspirin	-1.7498
4852-22-6	Proanthocyanidins	-1.7479
106685-40-9	Adapalene	-1.7466
315-30-0	Allopurinol	-1.7452
106685-40-9	Adapalene	-1.7417
29908-03-0	Ademetionine	-1.7412
570406-98-3	Avatrombopag	-1.7404
68-41-7	D-Cycloserine	-1.7397
136-47-0	Tetracaine hydrochloride	-1.7383
32795-44-1	Acecainide	-1.738
31282-04-9	Hygromycin B	-1.7358
67227-57-0	Fenoldopam mesylate	-1.7334
171599-83-0	Sildenafil citrate	-1.732
6078-17-7	Berbamine dihydrochloride	-1.732
138-14-7	Deferoxamine Mesylate	-1.7315
364622-82-2	Doripenem Hydrate	-1.7303
153773-82-1	Ertapenem sodium	-1.7295
698387-09-6	Neratinib	-1.729
21256-18-8	Oxaprozin	-1.7289
901119-35-5	Fostamatinib	-1.7287
220620-09-7	Tigecycline	-1.7272
877399-52-5	Crizotinib	-1.7266
639089-54-6	Tozasertib	-1.7246
83905-01-5	Azithromycin	-1.7237
106685-40-9	Adapalene	-1.7232
29908-03-0	Ademetionine	-1.7198
1492-18-8	Calcium folinate	-1.7196
50-63-5	Chloroquine diphosphate	-1.7191
59-05-2	Methotrexate	-1.718
61-78-9	4-Aminohippuric Acid	-1.7175
53179-09-2	Sisomicin Sulfate	-1.7168
304-84-7	Ethamivan	-1.7166
84611-23-4	Erdosteine	-1.7153
1405-37-4	Capastat sulfate	-1.714
76584-70-8	Divalproex Sodium	-1.7138
550-24-3	Embelin	-1.7113

380843-75-4	Bosutinib	-1.7076
117772-70-0	Azithromycin dihydrate	-1.707
951395-08-7	tafamidis meglumine	-1.7067
57-66-9	Probenecid	-1.7062
149003-01-0	Dexrazoxane HCl	-1.7039
68401-81-0	Ceftizoxime	-1.7038
29908-03-0	Ademetionine	-1.6974
4852-22-6	Proanthocyanidins	-1.6966
56391-57-2	Netilmicin sulfate	-1.6965
117772-70-0	Azithromycin dihydrate	-1.6957
3963-95-9	Methacycline hydrochloride	-1.6954
4852-22-6	Proanthocyanidins	-1.6947
872728-81-9	Dabigatran Etexilate Mesylate	-1.6936
211915-06-9	Dabigatran etexilate	-1.6936
51481-61-9	Cimetidine	-1.6928
1098-97-1	Pyrithioxin	-1.6925
155148-31-5	Plerixafor 8HCl (AMD3100 8HCl)	-1.6911
110078-46-1	Plerixafor	-1.6911
877399-52-5	Crizotinib	-1.6902
22373-78-0	Monensin sodium salt	-1.6901
1098-97-1	Pyrithioxin	-1.6896
76963-41-2	Nizatidine	-1.6893
144689-63-4	Olmesartan Medoxomil	-1.6884
4852-22-6	Proanthocyanidins	-1.6882
4852-22-6	Proanthocyanidins	-1.6872
57-66-9	Probenecid	-1.6813
76963-41-2	Nizatidine	-1.6801
405165-61-9	Besifloxacin HCl	-1.6799
66215-27-8	Cyromazine	-1.678
4852-22-6	Proanthocyanidins	-1.6779
78964-85-9	Fosfomycin Tromethamine	-1.6774
77-86-1	Trometamol	-1.6774
13110-96-8	5-Acetylsalicylic acid	-1.6773
96847-55-1	Levomilnacipran	-1.6753
937272-79-2	Pacritinib	-1.6671
67-43-6	Pentetic Acid	-1.6657
30751-05-4	Troxipide	-1.6656
951395-08-7	tafamidis meglumine	-1.6635
84611-23-4	Erdosteine	-1.6614
56-69-9	5-hydroxytryptophan	-1.6602
639089-54-6	Tozasertib	-1.6577
29908-03-0	Ademetionine	-1.6567
550-24-3	Embelin	-1.6546

67-43-6	Pentetic Acid	-1.6535
611-71-2	Mandelic acid	-1.6508
141-94-6	Hexetidine	-1.6502
155148-31-5	Plerixafor 8HCl (AMD3100 8HCl)	-1.65
110078-46-1	Plerixafor	-1.65
4852-22-6	Proanthocyanidins	-1.6497
155148-31-5	Plerixafor 8HCl (AMD3100 8HCl)	-1.6475
110078-46-1	Plerixafor	-1.6475
29908-03-0	Ademetionine	-1.647
88495-63-0	Artesunate	-1.6464
24390-14-5	Doxycycline hyclate	-1.646
951395-08-7	tafamidis meglumine	-1.6378
66215-27-8	Cyromazine	-1.6348
2068-78-2	Vincristine sulfate	-1.6347
52-66-4	DL-Penicillamine	-1.6338
52214-84-3	Ciprofibrate	-1.6334
951395-08-7	tafamidis meglumine	-1.6333
13110-96-8	5-Acetylsalicylic acid	-1.6326
951395-08-7	tafamidis meglumine	-1.6295
29908-03-0	Ademetionine	-1.6293
80433-71-2	Calcium Levofolinate	-1.6281
903576-44-3	Pitolisant hydrochloride	-1.6272
57-09-0	Cetrimonium bromide	-1.6265
951395-08-7	tafamidis meglumine	-1.6251
151533-22-1	Levomefolate calcium	-1.6243
64-73-3	Demeclocycline hydrochloride	-1.6227
59-05-2	Methotrexate	-1.6225
61-78-9	4-Aminohippuric Acid	-1.621
755038-65-4	Volasertib	-1.6209
50370-12-2	Cefadroxil	-1.6187
13189-98-5	Fudosteine	-1.6157
64-73-3	Demeclocycline hydrochloride	-1.6154
90098-04-7	Rebamipide	-1.6145
594839-88-0	Tafamidis	-1.6132
59-92-7	Levodopa	-1.61
141-94-6	Hexetidine	-1.6077
67227-57-0	Fenoldopam mesylate	-1.6073
68-41-7	D-Cycloserine	-1.6067
26750-81-2	Alibendol	-1.6055
210353-53-0	Gemifloxacin?mesylate	-1.6052
6078-17-7	Berbamine dihydrochloride	-1.6039
155148-31-5	Plerixafor 8HCl (AMD3100 8HCl)	-1.6023
110078-46-1	Plerixafor	-1.6023

1405-41-0	Gentamycin sulfate	-1.6018
4852-22-6	Proanthocyanidins	-1.6006
54-35-3	Procaine penicillin G	-1.6005
138-14-7	Deferoxamine Mesylate	-1.5989
951395-08-7	tafamidis meglumine	-1.5981
64-73-3	Demeclocycline hydrochloride	-1.5972
80621-81-4	Rifaximin	-1.597
4852-22-6	Proanthocyanidins	-1.5953
123040-16-4	Azasetron hydrochloride	-1.5952
1937-19-5	Aminoguanidine hydrochloride	-1.5908
4852-22-6	Proanthocyanidins	-1.5895
24390-14-5	Doxycycline hyclate	-1.5894
210353-53-0	Gemifloxacin?mesylate	-1.5888
116-43-8	Succinylsulfathiazole	-1.5844
53797-35-6	Ribostamycin sulfate	-1.5841
637-32-1	Proguanil hydrochloride	-1.5837
155148-31-5	Plerixafor 8HCl (AMD3100 8HCl)	-1.5836
110078-46-1	Plerixafor	-1.5836
64-75-5	Tetracycline hydrochloride	-1.5833
901119-35-5	Fostamatinib	-1.5785
79-57-2	Oxytetracycline	-1.5773
58-54-8	Ethacrynic acid	-1.5772
4852-22-6	Proanthocyanidins	-1.5752
155148-31-5	Plerixafor 8HCl (AMD3100 8HCl)	-1.5728
110078-46-1	Plerixafor	-1.5728
1953-02-2	Tiopronin	-1.5726
110-15-6	Succinic acid	-1.5724
4852-22-6	Proanthocyanidins	-1.5701
58-54-8	Ethacrynic acid	-1.5691
76963-41-2	Nizatidine	-1.5684
1207456-01-6	Talazoparib	-1.5659
52214-84-3	Ciprofibrate	-1.565
951395-08-7	tafamidis meglumine	-1.5648
145040-37-5	Candesartan Cilexetil	-1.5613
149003-01-0	Dexrazoxane HCl	-1.5598
190786-44-8	Bepotastine Besilate	-1.5598
4852-22-6	Proanthocyanidins	-1.5592
62893-19-0	Cefoperazone	-1.5591
541-22-0	Decamethonium bromide	-1.5582
73816-42-9	Meclocycline sulfosalicylate salt	-1.5566
56-69-9	5-hydroxytryptophan	-1.5548
186826-86-8	Moxifloxacin hydrochloride	-1.5541

83915-83-7	Lisinopril dihydrate	-1.5534
4852-22-6	Proanthocyanidins	-1.5523
149003-01-0	Dexrazoxane HCl	-1.5521
6078-17-7	Berbamine dihydrochloride	-1.5517
79-57-2	Oxytetracycline	-1.55
4852-22-6	Proanthocyanidins	-1.5475
4852-22-6	Proanthocyanidins	-1.547
30751-05-4	Troxipide	-1.5462
103766-25-2	Gimeracil	-1.5456
1207456-01-6	Talazoparib	-1.5452
76824-35-6	Famotidine	-1.5446
901119-35-5	Fostamatinib	-1.5446
951395-08-7	tafamidis meglumine	-1.5445
76584-70-8	Divalproex Sodium	-1.5431
4852-22-6	Proanthocyanidins	-1.5426
26750-81-2	Alibendol	-1.5421
136-47-0	Tetracaine hydrochloride	-1.5419
64-72-2	Chlortetracycline hydrochloride	-1.5406
541-22-0	Decamethonium bromide	-1.5405
115939-25-8	Salvianolic acid B	-1.5396
73816-42-9	Meclocycline sulfosalicylate salt	-1.5371
76584-70-8	Divalproex Sodium	-1.5367
30751-05-4	Troxipide	-1.5343
4852-22-6	Proanthocyanidins	-1.5339
1937-19-5	Aminoguanidine hydrochloride	-1.5325
4852-22-6	Proanthocyanidins	-1.5308
4852-22-6	Proanthocyanidins	-1.528
65391-42-6	Bestatin hydrochloride	-1.5278
58970-76-6	Ubenimex	-1.5278
66215-27-8	Cyromazine	-1.527
29908-03-0	Ademetionine	-1.5261
59-05-2	Methotrexate	-1.5254
339-72-0	L-Cycloserine	-1.5253
56-69-9	5-hydroxytryptophan	-1.5241
141-01-5	Ferrous fumarate	-1.5225
210353-53-0	Gemifloxacin?mesylate	-1.522
50-63-5	Chloroquine diphosphate	-1.521
901119-35-5	Fostamatinib	-1.5176
127294-70-6	Balofloxacin	-1.5139
16485-10-2	Panthenol	-1.5131
121123-17-9	Cefprozil hydrate	-1.5126
322-79-2	Triflusal	-1.5124

171599-83-0	Sildenafil citrate	-1.512
4852-22-6	Proanthocyanidins	-1.5088
64-73-3	Demeclocycline hydrochloride	-1.5081
951395-08-7	tafamidis meglumine	-1.5068
32986-56-4	Tobramycin	-1.5022
4852-22-6	Proanthocyanidins	-1.5011
68401-81-0	Ceftizoxime	-1.5008
18699-02-0	Actarit	-1.4992
67-43-6	Pentetic Acid	-1.4969
4852-22-6	Proanthocyanidins	-1.4966
149003-01-0	Dexrazoxane HCl	-1.4942
695-53-4	5,5-Dimethyloxazolidine-2,4-dione	-1.4919
951395-08-7	tafamidis meglumine	-1.4918
138-14-7	Deferoxamine Mesylate	-1.489
1405-37-4	Capastat sulfate	-1.489
24390-14-5	Doxycycline hyclate	-1.4877
22252-43-3	Cefalexine EP IMpurity B	-1.4864
4852-22-6	Proanthocyanidins	-1.4853
52-90-4	cysteine	-1.482
79-57-2	Oxytetracycline	-1.4814
220620-09-7	Tigecycline	-1.4796
1207456-01-6	Talazoparib	-1.4793
149003-01-0	Dexrazoxane HCl	-1.4793
210353-53-0	Gemifloxacin?mesylate	-1.4779
405165-61-9	Besifloxacin HCl	-1.4763
76824-35-6	Famotidine	-1.4761
32986-56-4	Tobramycin	-1.4757
16485-10-2	Panthenol	-1.4741
518048-05-0	Raltegravir	-1.474
594839-88-0	Tafamidis	-1.4729
69-72-7	Salicylic acid	-1.4726
951395-08-7	tafamidis meglumine	-1.4725
6078-17-7	Berbamine dihydrochloride	-1.4706
64-75-5	Tetracycline hydrochloride	-1.469
138402-11-6	Irbesartan	-1.4676
594839-88-0	Tafamidis	-1.4651
163253-35-8	Sitafloxacin Hydrate	-1.4612
1492-18-8	Calcium folinate	-1.4611
52-90-4	cysteine	-1.4607
66-22-8	Uracil	-1.4588
13189-98-5	Fudosteine	-1.4581
115939-25-8	Salvianolic acid B	-1.4514

550-24-3	Embelin	-1.4514
171599-83-0	Sildenafil citrate	-1.4494
50370-12-2	Cefadroxil	-1.4493
22839-47-0	Aspartame	-1.4471
76824-35-6	Famotidine	-1.4466
322-79-2	Triflusal	-1.4461
6402-23-9	Ethacridine lactate monohydrate	-1.4439
4852-22-6	Proanthocyanidins	-1.4436
50-81-7	L(+)-Ascorbic acid	-1.4419
639089-54-6	Tozasertib	-1.4416
951395-08-7	tafamidis meglumine	-1.4415
70797-11-4	Cefpiramide acid	-1.4372
4852-22-6	Proanthocyanidins	-1.435
4852-22-6	Proanthocyanidins	-1.4339
76824-35-6	Famotidine	-1.4338
64-75-5	Tetracycline hydrochloride	-1.4329
141-90-2	Deracil	-1.4323
247257-48-3	Fimasartan	-1.4321
155148-31-5	Plerixafor 8HCl (AMD3100 8HCl)	-1.4279
110078-46-1	Plerixafor	-1.4279
1837-57-6	Ethacridine lactate	-1.4274
121123-17-9	Cefprozil hydrate	-1.4264
30751-05-4	Troxipide	-1.4243
141626-36-0	Dronedarone	-1.4237
141625-93-6	Dronedarone hydrochloride	-1.4219
76963-41-2	Nizatidine	-1.42
1032900-25-6	LDK378	-1.4182
64-75-5	Tetracycline hydrochloride	-1.418
92636-39-0	Cefminox Sodium	-1.4164
4852-22-6	Proanthocyanidins	-1.4142
91374-20-8	Ropinirole hydrochloride	-1.413
50-81-7	L(+)-Ascorbic acid	-1.4123
4852-22-6	Proanthocyanidins	-1.4093
60-54-8	Tetracycline	-1.4084
82009-34-5	Cilastatin	-1.4082
13614-98-7	Minocycline hydrochloride	-1.4072
51481-61-9	Cimetidine	-1.4069
64506-49-6	Sofalcone	-1.4069
60-54-8	Tetracycline	-1.4068
5638-76-6	Betahistine	-1.4057
54856-23-4	Betahistine mesylate	-1.4039
5579-84-0	Betahistine dihydrochloride	-1.4039

155148-31-5	Plerixafor 8HCl (AMD3100 8HCl)	-1.4038
110078-46-1	Plerixafor	-1.4038
32986-56-4	Tobramycin	-1.3975
202189-78-4	Bilastine	-1.3968
54856-23-4	Betahistine mesylate	-1.3962
5579-84-0	Betahistine dihydrochloride	-1.3962
62013-04-1	Dirithromycin	-1.3961
4852-22-6	Proanthocyanidins	-1.3952
76824-35-6	Famotidine	-1.3939
5638-76-6	Betahistine	-1.3932
30751-05-4	Troxipide	-1.3913
96847-55-1	Levomilnacipran	-1.3897
76963-41-2	Nizatidine	-1.3897
62893-19-0	Cefoperazone	-1.3888
31282-04-9	Hygromycin B	-1.3887
6078-17-7	Berbamine dihydrochloride	-1.3868
637-32-1	Proguanil hydrochloride	-1.3866
112887-68-0	Raltitrexed	-1.3807
60-54-8	Tetracycline	-1.3795
22457-89-2	Benfotiamine	-1.3758
83915-83-7	Lisinopril dihydrate	-1.3734
202189-78-4	Bilastine	-1.3727
112887-68-0	Raltitrexed	-1.3721
66309-69-1	Cefotiam hydrochloride	-1.371
50-81-7	L(+)-Ascorbic acid	-1.3701
191217-81-9	Pramipexole 2HCl Monohydrate	-1.3688
6402-23-9	Ethacridine lactate monohydrate	-1.3683
51481-61-9	Cimetidine	-1.3677
96847-55-1	Levomilnacipran	-1.3669
518048-05-0	Raltegravir	-1.3637
51037-30-0	Acipimox	-1.3636
29908-03-0	Ademetionine	-1.3616
52214-84-3	Ciprofibrate	-1.3608
64-72-2	Chlortetracycline hydrochloride	-1.3607
951395-08-7	tafamidis meglumine	-1.3592
75438-57-2	Moxonidine	-1.3573
29908-03-0	Ademetionine	-1.3564
637-32-1	Proguanil hydrochloride	-1.3525
67227-57-0	Fenoldopam mesylate	-1.3489
69-72-7	Salicylic acid	-1.3475
60-54-8	Tetracycline	-1.3443
4852-22-6	Proanthocyanidins	-1.3438
58-85-5	D-Biotin	-1.3427

6402-23-9	Ethacridine lactate monohydrate	-1.3407
54856-23-4	Betahistine mesylate	-1.3369
5579-84-0	Betahistine dihydrochloride	-1.3369
290297-26-6	Netupitant	-1.3353
5638-76-6	Betahistine	-1.3339
83905-01-5	Azithromycin	-1.333
901119-35-5	Fostamatinib	-1.3312
121123-17-9	Cefprozil hydrate	-1.33
882-09-7	Clofibric acid	-1.3297
4852-22-6	Proanthocyanidins	-1.3293
155148-31-5	Plerixafor 8HCl (AMD3100 8HCl)	-1.3292
110078-46-1	Plerixafor	-1.3292
75438-57-2	Moxonidine	-1.3291
1231930-82-7	LY2835219	-1.3277
1837-57-6	Ethacridine lactate	-1.3273
51481-61-9	Cimetidine	-1.3255
149003-01-0	Dexrazoxane HCl	-1.3254
68401-81-0	Ceftizoxime	-1.3212
26750-81-2	Alibendol	-1.3211
1405-37-4	Capastat sulfate	-1.3206
4852-22-6	Proanthocyanidins	-1.3183
4852-22-6	Proanthocyanidins	-1.3165
155148-31-5	Plerixafor 8HCl (AMD3100 8HCl)	-1.3151
110078-46-1	Plerixafor	-1.3151
16485-10-2	Panthenol	-1.3109
153773-82-1	Ertapenem sodium	-1.3108
57-09-0	Cetrimonium bromide	-1.3103
52214-84-3	Ciprofibrate	-1.3094
76963-41-2	Nizatidine	-1.3083
872728-81-9	Dabigatran Etexilate Mesylate	-1.3068
211915-06-9	Dabigatran etexilate	-1.3068
153773-82-1	Ertapenem sodium	-1.306
107133-36-8	Perindopril erbumine	-1.3049
15686-71-2	Cephalexin	-1.3034
475086-01-2	Selexipag	-1.3027
136-47-0	Tetracaine hydrochloride	-1.2995
339-72-0	L-Cycloserine	-1.2992
4852-22-6	Proanthocyanidins	-1.2985
4852-22-6	Proanthocyanidins	-1.2974
1254053-43-4	Gilteritinib	-1.296
22252-43-3	Cefalexine EP IMpurity B	-1.2935

147127-20-6	Tenofovir	-1.2934
4852-22-6	Proanthocyanidins	-1.2926
114798-26-4	Losartan	-1.2907
13614-98-7	Minocycline hydrochloride	-1.2906
83905-01-5	Azithromycin	-1.2897
951395-08-7	tafamidis meglumine	-1.2892
951395-08-7	tafamidis meglumine	-1.2882
1405-41-0	Gentamycin sulfate	-1.2875
951395-08-7	tafamidis meglumine	-1.2872
937272-79-2	Pacritinib	-1.2856
220620-09-7	Tigecycline	-1.2855
114798-26-4	Losartan	-1.2798
64-75-5	Tetracycline hydrochloride	-1.2781
59-52-9	Dithiopropanol	-1.2697
4852-22-6	Proanthocyanidins	-1.2661
1953-02-2	Tiopronin	-1.2649
4852-22-6	Proanthocyanidins	-1.2644
951395-08-7	tafamidis meglumine	-1.2638
322-79-2	Triflusal	-1.2598
22252-43-3	Cefalexine EP IMpurity B	-1.259
4852-22-6	Proanthocyanidins	-1.2589
1231930-82-7	LY2835219	-1.2573
60-54-8	Tetracycline	-1.256
79-57-2	Oxytetracycline	-1.2526
2066-89-9	Pasiniazid	-1.252
51481-61-9	Cimetidine	-1.2511
2066-89-9	Pasiniazid	-1.25
107007-99-8	Granisetron hydrochloride	-1.2484
389139-89-3	Omadacycline	-1.2475
155148-31-5	Plerixafor 8HCl (AMD3100 8HCl)	-1.2473
110078-46-1	Plerixafor	-1.2473
54-35-3	Procaine penicillin G	-1.2449
51481-61-9	Cimetidine	-1.2448
14919-77-8	Benserazide hydrochloride	-1.2436
4852-22-6	Proanthocyanidins	-1.2417
4852-22-6	Proanthocyanidins	-1.2374
64-75-5	Tetracycline hydrochloride	-1.2373
51481-61-9	Cimetidine	-1.2333
63585-09-1	Foscarnet sodium	-1.2318
87848-99-5	Acrivastine	-1.2304
112-80-1	Oleic acid	-1.2281
67-43-6	Pentetic Acid	-1.2267

698387-09-6	Neratinib	-1.2254
951395-08-7	tafamidis meglumine	-1.2247
31282-04-9	Hygromycin B	-1.2222
6078-17-7	Berbamine dihydrochloride	-1.2219
83-12-5	Phenylindione	-1.2214
51037-30-0	Acipimox	-1.2211
15686-71-2	Cephalexin	-1.2197
87848-99-5	Acrivastine	-1.2186
155148-31-5	Plerixafor 8HCl (AMD3100 8HCl)	-1.2169
110078-46-1	Plerixafor	-1.2169
1953-02-2	Tiopronin	-1.2147
951395-08-7	tafamidis meglumine	-1.2126
73816-42-9	Meclocycline sulfosalicylate salt	-1.2119
155148-31-5	Plerixafor 8HCl (AMD3100 8HCl)	-1.2107
110078-46-1	Plerixafor	-1.2107
194798-83-9	Fosfluconazole	-1.2106
24390-14-5	Doxycycline hyclate	-1.2105
189188-57-6	Tegaserod maleate	-1.2098
220620-09-7	Tigecycline	-1.2082
147127-20-6	Tenofovir	-1.2059
695-53-4	5,5-Dimethyloxazolidine-2,4-dione	-1.2055
20537-88-6	Amifostine	-1.2051
50-63-5	Chloroquine diphosphate	-1.203
13110-96-8	5-Acetylsalicylic acid	-1.2017
389139-89-3	Omadacycline	-1.2012
220620-09-7	Tigecycline	-1.1983
59-52-9	Dithiopropanol	-1.1971
22199-08-2	Silver sulfadiazine	-1.196
87848-99-5	Acrivastine	-1.1903
863031-21-4	Azilsartan Medoxomil	-1.1878
52214-84-3	Ciprofibrate	-1.1856
4852-22-6	Proanthocyanidins	-1.1854
119793-66-7	Propionyl-L-carnitine hydrochloride	-1.1842
52214-84-3	Ciprofibrate	-1.1838
145040-37-5	Candesartan Cilexetil	-1.1828
138452-21-8	Fexofenadine hydrochloride	-1.1802
1146-99-2	chlophenadione	-1.176
220620-09-7	Tigecycline	-1.1757
951395-08-7	tafamidis meglumine	-1.1749
24390-14-5	Doxycycline hyclate	-1.1742
951395-08-7	tafamidis meglumine	-1.1738
138-14-7	Deferoxamine Mesylate	-1.1728
755038-65-4	Volasertib	-1.1708

4852-22-6	Proanthocyanidins	-1.1707
1837-57-6	Ethacridine lactate	-1.1703
4852-22-6	Proanthocyanidins	-1.1702
10017-44-4	L-Carnitine hydrochloride	-1.1694
51037-30-0	Acipimox	-1.1682
115939-25-8	Salvianolic acid B	-1.1654
4852-22-6	Proanthocyanidins	-1.1644
1405-37-4	Capastat sulfate	-1.1608
1207456-01-6	Talazoparib	-1.159
82159-09-9	Epalrestat	-1.1583
951395-08-7	tafamidis meglumine	-1.1551
951395-08-7	tafamidis meglumine	-1.1541
84680-54-6	Enalaprilat Dihydrate	-1.1489
14919-77-8	Benserazide hydrochloride	-1.1425
115939-25-8	Salvianolic acid B	-1.141
1405-41-0	Gentamycin sulfate	-1.1402
1405-41-0	Gentamycin sulfate	-1.1357
24390-14-5	Doxycycline hyclate	-1.1351
4852-22-6	Proanthocyanidins	-1.1331
4852-22-6	Proanthocyanidins	-1.1284
191217-81-9	Pramipexole 2HCl Monohydrate	-1.1282
155148-31-5	Plerixafor 8HCl (AMD3100 8HCl)	-1.1265
110078-46-1	Plerixafor	-1.1265
1405-37-4	Capastat sulfate	-1.1261
321-30-2	Adenine sulfate	-1.1239
73-24-5	Adenine	-1.1239
951395-08-7	tafamidis meglumine	-1.1229
1098-97-1	Pyrithioxin	-1.1213
4852-22-6	Proanthocyanidins	-1.1213
10017-44-4	L-Carnitine hydrochloride	-1.1212
99-15-0	Acetylleucine	-1.1211
59-05-2	Methotrexate	-1.1194
136-77-6	Hexylresorcinol	-1.1191
189188-57-6	Tegaserod maleate	-1.119
51481-61-9	Cimetidine	-1.1174
58-54-8	Ethacrynic acid	-1.1169
85409-38-7	Rilmenidine Phosphate	-1.1154
1405-37-4	Capastat sulfate	-1.1093
83881-52-1	Cetirizine dihydrochloride	-1.107
63585-09-1	Foscarnet sodium	-1.104
53164-05-9	Acemetacin	-1.1036
66309-69-1	Cefotiam hydrochloride	-1.1032

62013-04-1	Dirithromycin	-1.1028
4852-22-6	Proanthocyanidins	-1.1023
51481-61-9	Cimetidine	-1.1023
1257044-40-8	ABT199	-1.1006
107133-36-8	Perindopril erbumine	-1.0965
191217-81-9	Pramipexole 2HCl Monohydrate	-1.0964
83881-52-1	Cetirizine dihydrochloride	-1.096
147127-20-6	Tenofovir	-1.0939
130018-77-8	Levocetirizine	-1.0933
738606-46-7	ETC1002	-1.0927
32986-56-4	Tobramycin	-1.0926
14919-77-8	Benserazide hydrochloride	-1.0924
13614-98-7	Minocycline hydrochloride	-1.0914
4852-22-6	Proanthocyanidins	-1.0909
155148-31-5	Plerixafor 8HCl (AMD3100 8HCl)	-1.0873
110078-46-1	Plerixafor	-1.0873
66309-69-1	Cefotiam hydrochloride	-1.0862
73816-42-9	Meclocycline sulfosalicylate salt	-1.0841
53797-35-6	Ribostamycin sulfate	-1.0812
1492-18-8	Calcium folinate	-1.0811
155148-31-5	Plerixafor 8HCl (AMD3100 8HCl)	-1.0809
110078-46-1	Plerixafor	-1.0809
73-22-3	L-Tryptophan	-1.0808
4852-22-6	Proanthocyanidins	-1.0792
69712-56-7	Cefotetan	-1.0782
110-15-6	Succinic acid	-1.0774
17440-83-4	Amiloride hydrochloride dihydrate	-1.0771
4852-22-6	Proanthocyanidins	-1.0767
83-12-5	Phenylindione	-1.0761
177036-94-1	Ambrisentan	-1.0757
4852-22-6	Proanthocyanidins	-1.0752
144689-24-7	Olmesartan	-1.0737
61336-70-7	Amoxicillin trihydrate	-1.0726
26787-78-0	Amoxicillin	-1.0726
882-09-7	Clofibric acid	-1.0716
50-63-5	Chloroquine diphosphate	-1.0711
51481-61-9	Cimetidine	-1.0702
85409-38-7	Rilmenidine Phosphate	-1.066
4852-22-6	Proanthocyanidins	-1.0638
59-52-9	Dithiopropanol	-1.0633
4852-22-6	Proanthocyanidins	-1.0632
155148-31-5	Plerixafor 8HCl (AMD3100 8HCl)	-1.0619

110078-46-1	Plerixafor	-1.0619
4852-22-6	Proanthocyanidins	-1.06
91374-20-8	Ropinirole hydrochloride	-1.0573
20537-88-6	Amifostine	-1.0565
639089-54-6	Tozasertib	-1.0515
59-52-9	Dithiopropanol	-1.0511
155148-31-5	Plerixafor 8HCl (AMD3100 8HCl)	-1.0502
110078-46-1	Plerixafor	-1.0502
76963-41-2	Nizatidine	-1.0478
73816-42-9	Meclocycline sulfosalicylate salt	-1.0476
4852-22-6	Proanthocyanidins	-1.0467
67227-57-0	Fenoldopam mesylate	-1.0459
4852-22-6	Proanthocyanidins	-1.0451
82159-09-9	Epalrestat	-1.0428
79-57-2	Oxytetracycline	-1.0427
6078-17-7	Berbamine dihydrochloride	-1.0426
53910-25-1	Pentostatin	-1.0403
220620-09-7	Tigecycline	-1.0396
141-01-5	Ferrous fumarate	-1.0332
4852-22-6	Proanthocyanidins	-1.0323
29908-03-0	Ademetionine	-1.0319
76963-41-2	Nizatidine	-1.0318
695-53-4	5,5-Dimethyloxazolidine-2,4-dione	-1.0314
10212-25-6	Cyclocytidine hydrochloride	-1.0306
53910-25-1	Pentostatin	-1.0271
139481-59-7	Candesartan	-1.026
107133-36-8	Perindopril erbumine	-1.0237
4852-22-6	Proanthocyanidins	-1.0219
83881-52-1	Cetirizine dihydrochloride	-1.02
4852-22-6	Proanthocyanidins	-1.018
4852-22-6	Proanthocyanidins	-1.017
130018-77-8	Levocetirizine	-1.0167
91374-20-8	Ropinirole hydrochloride	-1.0102
4852-22-6	Proanthocyanidins	-1.0084
518048-05-0	Raltegravir	-1.0074
211915-06-9	Dabigatran etexilate	-1.0072
698387-09-6	Neratinib	-1.0041
4852-22-6	Proanthocyanidins	-1.0041
4852-22-6	Proanthocyanidins	-0.9963
99-15-0	Acetylleucine	-0.9944
439081-18-2	Afatinib	-0.9935
850140-73-7	Afatinib Dimaleate	-0.9929
4852-22-6	Proanthocyanidins	-0.9906

4852-22-6	Proanthocyanidins	-0.9903
63585-09-1	Foscarnet sodium	-0.9889
17440-83-4	Amiloride hydrochloride dihydrate	-0.9876
66309-69-1	Cefotiam hydrochloride	-0.9869
91832-40-5	Cefdinir	-0.9859
69-72-7	Salicylic acid	-0.9853
4852-22-6	Proanthocyanidins	-0.9829
155148-31-5	Plerixafor 8HCl (AMD3100 8HCl)	-0.9826
110078-46-1	Plerixafor	-0.9826
84680-54-6	Enalaprilat Dihydrate	-0.9817
936539-80-9	Betrixaban maleate	-0.9801
T4690	Betrixaban hydrochloride(330942-05-7(free base))	-0.9801
330942-05-7	Betrixaban	-0.9801
136-77-6	Hexylresorcinol	-0.9795
4852-22-6	Proanthocyanidins	-0.9793
22839-47-0	Aspartame	-0.9755
83-12-5	Phenylindione	-0.9743
65391-42-6	Bestatin hydrochloride	-0.9728
58970-76-6	Ubenimex	-0.9728
38821-49-7	Carbidopa Hydrate	-0.9717
6035-45-6	Folinic Acid Calcium Salt Pentahydrate	-0.9703
103766-25-2	Gimeracil	-0.9688
76963-41-2	Nizatidine	-0.9673
119793-66-7	Propionyl-L-carnitine hydrochloride	-0.9669
80621-81-4	Rifaximin	-0.9665
38821-49-7	Carbidopa Hydrate	-0.9634
73816-42-9	Meclocycline sulfosalicylate salt	-0.963
901119-35-5	Fostamatinib	-0.9625
61336-70-7	Amoxicillin trihydrate	-0.9619
26787-78-0	Amoxicillin	-0.9619
4852-22-6	Proanthocyanidins	-0.9609
389139-89-3	Omadacycline	-0.9597
155148-31-5	Plerixafor 8HCl (AMD3100 8HCl)	-0.9582
110078-46-1	Plerixafor	-0.9582
4852-22-6	Proanthocyanidins	-0.9573
1405-37-4	Capastat sulfate	-0.9556
4852-22-6	Proanthocyanidins	-0.9542
76963-41-2	Nizatidine	-0.9529
107007-99-8	Granisetron hydrochloride	-0.9504
70797-11-4	Cefpiramide acid	-0.9497
61336-70-7	Amoxicillin trihydrate	-0.9465
26787-78-0	Amoxicillin	-0.9465
4852-22-6	Proanthocyanidins	-0.9449

80621-81-4	Rifaximin	-0.9441
1346242-81-6	Erdafitinib	-0.9439
114798-26-4	Losartan	-0.9416
2066-89-9	Pasiniazid	-0.94
4852-22-6	Proanthocyanidins	-0.937
194798-83-9	Fosfluconazole	-0.9369
1188-38-1	Carbaglu	-0.9367
4852-22-6	Proanthocyanidins	-0.9346
83905-01-5	Azithromycin	-0.9307
639089-54-6	Tozasertib	-0.93
4852-22-6	Proanthocyanidins	-0.9299
73-24-5	Adenine	-0.9298
51481-61-9	Cimetidine	-0.9298
321-30-2	Adenine sulfate	-0.9295
4852-22-6	Proanthocyanidins	-0.9286
2624-44-4	Ethamsylate	-0.9274
80621-81-4	Rifaximin	-0.9267
4852-22-6	Proanthocyanidins	-0.9215
73816-42-9	Meclocycline sulfosalicylate salt	-0.921
1188-38-1	Carbaglu	-0.9157
58-85-5	D-Biotin	-0.9152
115939-25-8	Salvianolic acid B	-0.9143
6078-17-7	Berbamine dihydrochloride	-0.9138
82159-09-9	Epalrestat	-0.9098
856867-55-5	Tedizolid Phosphate	-0.909
73816-42-9	Meclocycline sulfosalicylate salt	-0.9064
4852-22-6	Proanthocyanidins	-0.9032
10212-25-6	Cyclocytidine hydrochloride	-0.9024
130018-77-8	Levocetirizine	-0.9015
119793-66-7	Propionyl-L-carnitine hydrochloride	-0.9013
4852-22-6	Proanthocyanidins	-0.901
83881-52-1	Cetirizine dihydrochloride	-0.9003
76801-85-9	Azathramycin	-0.9003
76963-41-2	Nizatidine	-0.8999
4852-22-6	Proanthocyanidins	-0.8982
882-09-7	Clofibric acid	-0.8977
67-43-6	Pentetic Acid	-0.8973
4852-22-6	Proanthocyanidins	-0.8968
73-22-3	L-Tryptophan	-0.8964
1405-37-4	Capastat sulfate	-0.8946
4852-22-6	Proanthocyanidins	-0.8905
10212-25-6	Cyclocytidine hydrochloride	-0.8858

936539-80-9	Betrixaban maleate	-0.8832
T4690	Betrixaban hydrochloride(330942-05-7(free base))	-0.8832
330942-05-7	Betrixaban	-0.8832
20537-88-6	Amifostine	-0.8803
53910-25-1	Pentostatin	-0.8776
4852-22-6	Proanthocyanidins	-0.8745
70356-03-5	Cefaclor monohydrate	-0.8744
82009-34-5	Cilastatin	-0.8743
104206-65-7	Nitisinone	-0.867
698387-09-6	Neratinib	-0.8656
115939-25-8	Salvianolic acid B	-0.8653
66309-69-1	Cefotiam hydrochloride	-0.8633
4852-22-6	Proanthocyanidins	-0.8626
64506-49-6	Sofalcone	-0.8603
115939-25-8	Salvianolic acid B	-0.8586
4852-22-6	Proanthocyanidins	-0.8573
122111-03-9	Gemcitabine HCl	-0.8571
95058-81-4	Gemcitabine	-0.8571
38821-49-7	Carbidopa Hydrate	-0.857
4852-22-6	Proanthocyanidins	-0.8526
65666-07-1	Silymarin	-0.8519
31282-04-9	Hygromycin B	-0.8511
99-15-0	Acetylleucine	-0.8454
1188-38-1	Carbaglu	-0.8405
153-18-4	Rutin	-0.838
389139-89-3	Omadacycline	-0.8375
92636-39-0	Cefminox Sodium	-0.8372
53164-05-9	Acemetacin	-0.8335
103980-44-5	Ceftiofur hydrochloride	-0.8335
99-15-0	Acetylleucine	-0.8323
115939-25-8	Salvianolic acid B	-0.8258
99-15-0	Acetylleucine	-0.8235
4852-22-6	Proanthocyanidins	-0.8213
59-05-2	Methotrexate	-0.8207
65391-42-6	Bestatin hydrochloride	-0.8205
58970-76-6	Ubenimex	-0.8205
4852-22-6	Proanthocyanidins	-0.8202
116-43-8	Succinylsulfathiazole	-0.8177
4852-22-6	Proanthocyanidins	-0.8174
32986-56-4	Tobramycin	-0.8151
7481-89-2	Zalcitabine	-0.8145
4852-22-6	Proanthocyanidins	-0.8139
4852-22-6	Proanthocyanidins	-0.8094

73816-42-9	Meclocycline sulfosalicylate salt	-0.8093
155148-31-5	Plerixafor 8HCl (AMD3100 8HCl)	-0.8079
110078-46-1	Plerixafor	-0.8079
134678-17-4	Lamivudine	-0.8077
4852-22-6	Proanthocyanidins	-0.8055
68-41-7	D-Cycloserine	-0.805
84680-54-6	Enalaprilat Dihydrate	-0.8013
73-22-3	L-Tryptophan	-0.8006
115939-25-8	Salvianolic acid B	-0.7993
4852-22-6	Proanthocyanidins	-0.7953
10163-15-2	Disodium monofluorophosphate	-0.7936
60-18-4	Tyrosine	-0.7906
130929-57-6	Entacapone	-0.7803
73816-42-9	Meclocycline sulfosalicylate salt	-0.777
73816-42-9	Meclocycline sulfosalicylate salt	-0.7723
4852-22-6	Proanthocyanidins	-0.7675
116-43-8	Succinylsulfathiazole	-0.7664
116-43-8	Succinylsulfathiazole	-0.7651
50370-12-2	Cefadroxil	-0.7636
4852-22-6	Proanthocyanidins	-0.7589
389139-89-3	Omadacycline	-0.7575
59-52-9	Dithiopropanol	-0.7568
4852-22-6	Proanthocyanidins	-0.7562
4852-22-6	Proanthocyanidins	-0.756
65666-07-1	Silymarin	-0.7487
67227-57-0	Fenoldopam mesylate	-0.7476
4852-22-6	Proanthocyanidins	-0.7463
220620-09-7	Tigecycline	-0.7441
125317-39-7	Vinorelbine Tartrate	-0.7393
145040-37-5	Candesartan Cilexetil	-0.7393
60-18-4	Tyrosine	-0.739
103766-25-2	Gimeracil	-0.738
20537-88-6	Amifostine	-0.7367
4852-22-6	Proanthocyanidins	-0.7347
14919-77-8	Benserazide hydrochloride	-0.7346
65-86-1	Orotic acid	-0.7318
220620-09-7	Tigecycline	-0.7302
15686-71-2	Cephalexin	-0.7262
155148-31-5	Plerixafor 8HCl (AMD3100 8HCl)	-0.723
110078-46-1	Plerixafor	-0.723
4852-22-6	Proanthocyanidins	-0.7229
85409-38-7	Rilmenidine Phosphate	-0.7207
14919-77-8	Benserazide hydrochloride	-0.7194

220620-09-7	Tigecycline	-0.7192
10212-25-6	Cyclocytidine hydrochloride	-0.7189
4852-22-6	Proanthocyanidins	-0.7185
1953-02-2	Tiopronin	-0.7155
4852-22-6	Proanthocyanidins	-0.714
76963-41-2	Nizatidine	-0.7119
4852-22-6	Proanthocyanidins	-0.7109
4852-22-6	Proanthocyanidins	-0.7082
698387-09-6	Neratinib	-0.7076
1405-41-0	Gentamycin sulfate	-0.7075
1953-02-2	Tiopronin	-0.7069
65666-07-1	Silymarin	-0.701
4852-22-6	Proanthocyanidins	-0.7006
4852-22-6	Proanthocyanidins	-0.6989
10163-15-2	Disodium monofluorophosphate	-0.6944
155148-31-5	Plerixafor 8HCl (AMD3100 8HCl)	-0.6912
110078-46-1	Plerixafor	-0.6912
5080-50-2	O-Acetyl-L-carnitine hydrochloride	-0.6902
4852-22-6	Proanthocyanidins	-0.6886
69712-56-7	Cefotetan	-0.6843
66309-69-1	Cefotiam hydrochloride	-0.6842
4852-22-6	Proanthocyanidins	-0.6834
4852-22-6	Proanthocyanidins	-0.6797
1098-97-1	Pyrithioxin	-0.678
138-14-7	Deferoxamine Mesylate	-0.6776
2624-44-4	Ethamsylate	-0.6751
73816-42-9	Meclocycline sulfosalicylate salt	-0.6741
4852-22-6	Proanthocyanidins	-0.6704
59-92-7	Levodopa	-0.6632
153-18-4	Rutin	-0.6629
155148-31-5	Plerixafor 8HCl (AMD3100 8HCl)	-0.6608
110078-46-1	Plerixafor	-0.6608
4852-22-6	Proanthocyanidins	-0.6594
76963-41-2	Nizatidine	-0.6593
20537-88-6	Amifostine	-0.6534
4852-22-6	Proanthocyanidins	-0.6529
183204-72-0	Tipiracil hydrochloride	-0.6523
69712-56-7	Cefotetan	-0.6504
4852-22-6	Proanthocyanidins	-0.6503
122111-03-9	Gemcitabine HCl	-0.642
95058-81-4	Gemcitabine	-0.642
738606-46-7	ETC1002	-0.6417
4852-22-6	Proanthocyanidins	-0.6411

1146-99-2	chlophenadione	-0.6401
4852-22-6	Proanthocyanidins	-0.6394
4852-22-6	Proanthocyanidins	-0.6387
4852-22-6	Proanthocyanidins	-0.6365
76963-41-2	Nizatidine	-0.635
4852-22-6	Proanthocyanidins	-0.6337
856867-55-5	Tedizolid Phosphate	-0.6337
4852-22-6	Proanthocyanidins	-0.6336
4852-22-6	Proanthocyanidins	-0.6322
51481-61-9	Cimetidine	-0.6311
2624-44-4	Ethamsylate	-0.6305
4852-22-6	Proanthocyanidins	-0.6257
69712-56-7	Cefotetan	-0.6231
155148-31-5	Plerixafor 8HCl (AMD3100 8HCl)	-0.6199
110078-46-1	Plerixafor	-0.6199
4852-22-6	Proanthocyanidins	-0.6194
4852-22-6	Proanthocyanidins	-0.6158
110-15-6	Succinic acid	-0.6156
60-18-4	Tyrosine	-0.6147
123-99-9	Azelaic acid	-0.6117
5451-09-2	5-Aminolevulinic acid hydrochloride	-0.6109
4852-22-6	Proanthocyanidins	-0.6096
4852-22-6	Proanthocyanidins	-0.6078
1365970-03-1	Glecaprevir	-0.6037
4940-39-0	Chromocarbe	-0.6004
92636-39-0	Cefminox Sodium	-0.6002
4852-22-6	Proanthocyanidins	-0.5946
4852-22-6	Proanthocyanidins	-0.5913
59-92-7	Levodopa	-0.5908
637-32-1	Proguanil hydrochloride	-0.589
1492-18-8	Calcium folinate	-0.5888
59-92-7	Levodopa	-0.5873
187227-45-8	Oseltamivir acid	-0.5858
70797-11-4	Cefpiramide acid	-0.5856
53797-35-6	Ribostamycin sulfate	-0.5834
69712-56-7	Cefotetan	-0.5833
4852-22-6	Proanthocyanidins	-0.583
504-03-0	Lupetidin	-0.5808
1501-84-4	Rimantadine hydrochloride	-0.5779
4852-22-6	Proanthocyanidins	-0.5773
4852-22-6	Proanthocyanidins	-0.5721
5080-50-2	O-Acetyl-L-carnitine hydrochloride	-0.5718

4852-22-6	Proanthocyanidins	-0.5712
4940-39-0	Chromocarbe	-0.571
10212-25-6	Cyclocytidine hydrochloride	-0.5692
220620-09-7	Tigecycline	-0.5676
504-03-0	Lupetidin	-0.5652
1405-37-4	Capastat sulfate	-0.564
4852-22-6	Proanthocyanidins	-0.5632
65666-07-1	Silymarin	-0.5607
4852-22-6	Proanthocyanidins	-0.5602
755038-65-4	Volasertib	-0.56
4852-22-6	Proanthocyanidins	-0.5581
4852-22-6	Proanthocyanidins	-0.5565
113852-37-2	Cidofovir	-0.5561
155148-31-5	Plerixafor 8HCl (AMD3100 8HCl)	-0.5532
110078-46-1	Plerixafor	-0.5532
4852-22-6	Proanthocyanidins	-0.5516
4852-22-6	Proanthocyanidins	-0.5499
856867-55-5	Tedizolid Phosphate	-0.5493
65666-07-1	Silymarin	-0.5491
1953-02-2	Tiopronin	-0.549
4852-22-6	Proanthocyanidins	-0.5489
220620-09-7	Tigecycline	-0.5483
104206-65-7	Nitisinone	-0.5474
4852-22-6	Proanthocyanidins	-0.5472
76824-35-6	Famotidine	-0.5455
155148-31-5	Plerixafor 8HCl (AMD3100 8HCl)	-0.5446
110078-46-1	Plerixafor	-0.5446
364622-82-2	Doripenem Hydrate	-0.5419
76963-41-2	Nizatidine	-0.5412
91832-40-5	Cefdinir	-0.5401
31282-04-9	Hygromycin B	-0.5362
951395-08-7	tafamidis meglumine	-0.534
65666-07-1	Silymarin	-0.5335
504-03-0	Lupetidin	-0.5329
136-77-6	Hexylresorcinol	-0.5276
4852-22-6	Proanthocyanidins	-0.5255
4852-22-6	Proanthocyanidins	-0.5245
155148-31-5	Plerixafor 8HCl (AMD3100 8HCl)	-0.5238
110078-46-1	Plerixafor	-0.5238
127-07-1	Hydroxyurea	-0.5228
738606-46-7	ETC1002	-0.5199
4852-22-6	Proanthocyanidins	-0.5196
439081-18-2	Afatinib	-0.5192

123-99-9	Azelaic acid	-0.5138
4852-22-6	Proanthocyanidins	-0.511
4852-22-6	Proanthocyanidins	-0.5095
755038-65-4	Volasertib	-0.5046
79-55-0	Pempidine	-0.5045
637-32-1	Proguanil hydrochloride	-0.5041
4852-22-6	Proanthocyanidins	-0.5038
7481-89-2	Zalcitabine	-0.5038
1405-41-0	Gentamycin sulfate	-0.502
543-15-7	Heptaminol hydrochloride	-0.4923
4852-22-6	Proanthocyanidins	-0.4919
122111-03-9	Gemcitabine HCl	-0.4895
95058-81-4	Gemcitabine	-0.4895
4852-22-6	Proanthocyanidins	-0.4866
5451-09-2	5-Aminolevulinic acid hydrochloride	-0.4859
110-15-6	Succinic acid	-0.4793
4852-22-6	Proanthocyanidins	-0.4779
80621-81-4	Rifaximin	-0.4757
4852-22-6	Proanthocyanidins	-0.4748
59-52-9	Dithiopropanol	-0.4712
4852-22-6	Proanthocyanidins	-0.4711
1257044-40-8	ABT199	-0.4702
220620-09-7	Tigecycline	-0.4677
4852-22-6	Proanthocyanidins	-0.4673
4852-22-6	Proanthocyanidins	-0.4672
24390-14-5	Doxycycline hyclate	-0.4666
99-15-0	Acetylleucine	-0.4625
7681-93-8	Pimaricin	-0.4557
4852-22-6	Proanthocyanidins	-0.4548
4852-22-6	Proanthocyanidins	-0.4535
4852-22-6	Proanthocyanidins	-0.451
66-22-8	Uracil	-0.4488
103980-44-5	Ceftiofur hydrochloride	-0.4479
4852-22-6	Proanthocyanidins	-0.4477
1405-37-4	Capastat sulfate	-0.4424
698387-09-6	Neratinib	-0.441
504-03-0	Lupetidin	-0.4406
321-30-2	Adenine sulfate	-0.438
73-24-5	Adenine	-0.438
4852-22-6	Proanthocyanidins	-0.437
4852-22-6	Proanthocyanidins	-0.4369
69308-37-8	(R)-baclofen	-0.436

1134-47-0	Baclofen	-0.436
4852-22-6	Proanthocyanidins	-0.4347
4852-22-6	Proanthocyanidins	-0.4293
75438-57-2	Moxonidine	-0.4264
177036-94-1	Ambrisentan	-0.4262
4852-22-6	Proanthocyanidins	-0.4251
115436-72-1	Risedronate Sodium	-0.425
4852-22-6	Proanthocyanidins	-0.425
115939-25-8	Salvianolic acid B	-0.4239
55-56-1	Chlorhexidine	-0.4208
4852-22-6	Proanthocyanidins	-0.4184
68-35-9	Sulfadiazine	-0.418
64506-49-6	Sofalcone	-0.417
1009119-64-5	Daclatasvir	-0.4167
5080-50-2	O-Acetyl-L-carnitine hydrochloride	-0.4161
103980-44-5	Ceftiofur hydrochloride	-0.4156
570406-98-3	Avatrombopag	-0.415
67227-57-0	Fenoldopam mesylate	-0.4145
65666-07-1	Silymarin	-0.414
504-03-0	Lupetidin	-0.4139
67227-57-0	Fenoldopam mesylate	-0.4112
504-03-0	Lupetidin	-0.4109
66-22-8	Uracil	-0.4101
149003-01-0	Dexrazoxane HCl	-0.4081
4852-22-6	Proanthocyanidins	-0.4075
389139-89-3	Omadacycline	-0.4072
57248-88-1	Pamidronate disodium salt	-0.4048
4852-22-6	Proanthocyanidins	-0.4018
1405-37-4	Capastat sulfate	-0.4015
4852-22-6	Proanthocyanidins	-0.3996
134678-17-4	Lamivudine	-0.3992
155148-31-5	Plerixafor 8HCl (AMD3100 8HCl)	-0.3888
110078-46-1	Plerixafor	-0.3888
4852-22-6	Proanthocyanidins	-0.388
1009119-65-6	Daclatasvir dihydrochloride	-0.3879
4852-22-6	Proanthocyanidins	-0.3853
22888-70-6	Silibinin	-0.3831
144689-63-4	Olmesartan Medoxomil	-0.3805
7327-87-9	Dihydralazine sulphate	-0.3802
194798-83-9	Fosfluconazole	-0.38
138452-21-8	Fexofenadine hydrochloride	-0.3798

4852-22-6	Proanthocyanidins	-0.3759
4852-22-6	Proanthocyanidins	-0.3747
65666-07-1	Silymarin	-0.3741
1405-41-0	Gentamycin sulfate	-0.3709
637-32-1	Proguanil hydrochloride	-0.3676
863031-21-4	Azilsartan Medoxomil	-0.3654
65666-07-1	Silymarin	-0.3635
65666-07-1	Silymarin	-0.3624
80621-81-4	Rifaximin	-0.3597
4852-22-6	Proanthocyanidins	-0.3594
65666-07-1	Silymarin	-0.359
65666-07-1	Silymarin	-0.3588
4852-22-6	Proanthocyanidins	-0.3581
65666-07-1	Silymarin	-0.3506
183204-72-0	Tipiracil hydrochloride	-0.349
4852-22-6	Proanthocyanidins	-0.3487
155148-31-5	Plerixafor 8HCl (AMD3100 8HCl)	-0.3479
110078-46-1	Plerixafor	-0.3479
4852-22-6	Proanthocyanidins	-0.3461
65666-07-1	Silymarin	-0.3437
57248-88-1	Pamidronate disodium salt	-0.3431
138452-21-8	Fexofenadine hydrochloride	-0.3429
65666-07-1	Silymarin	-0.3414
69558-55-0	Thymopentin	-0.3407
504-03-0	Lupetidin	-0.3384
187227-45-8	Oseltamivir acid	-0.3377
65666-07-1	Silymarin	-0.3374
115939-25-8	Salvianolic acid B	-0.3365
4852-22-6	Proanthocyanidins	-0.3351
4852-22-6	Proanthocyanidins	-0.3329
10212-25-6	Cyclocytidine hydrochloride	-0.3301
76963-41-2	Nizatidine	-0.3275
20537-88-6	Amifostine	-0.3258
59-05-2	Methotrexate	-0.3233
65-86-1	Orotic acid	-0.3197
4852-22-6	Proanthocyanidins	-0.3187
65666-07-1	Silymarin	-0.318
4852-22-6	Proanthocyanidins	-0.3164
65666-07-1	Silymarin	-0.3149
4852-22-6	Proanthocyanidins	-0.3105
1113-59-3	3-Bromopyruvic acid	-0.3077
1113-59-3	3-Bromopyruvic acid	-0.3067

1257044-40-8	ABT199	-0.3049
4852-22-6	Proanthocyanidins	-0.3037
4852-22-6	Proanthocyanidins	-0.3034
138402-11-6	Irbesartan	-0.3031
68-35-9	Sulfadiazine	-0.3007
113852-37-2	Cidofovir	-0.2992
83915-83-7	Lisinopril dihydrate	-0.294
52152-93-9	Cefsulodine sodium	-0.2923
543-82-8	2-Amino-6-methylheptane	-0.289
65666-07-1	Silymarin	-0.2848
7327-87-9	Dihydralazine sulphate	-0.2825
4852-22-6	Proanthocyanidins	-0.2809
115939-25-8	Salvianolic acid B	-0.2773
65666-07-1	Silymarin	-0.2756
65666-07-1	Silymarin	-0.2732
127-07-1	Hydroxyurea	-0.2731
183204-72-0	Tipiracil hydrochloride	-0.2671
4852-22-6	Proanthocyanidins	-0.2656
1146-99-2	chlophenadione	-0.2653
504-03-0	Lupetidin	-0.263
4852-22-6	Proanthocyanidins	-0.2627
65666-07-1	Silymarin	-0.2601
65666-07-1	Silymarin	-0.2563
1254053-43-4	Gilteritinib	-0.255
1098-97-1	Pyrithioxin	-0.2542
543-15-7	Heptaminol hydrochloride	-0.2525
104206-65-7	Nitisinone	-0.2493
65666-07-1	Silymarin	-0.2481
123-99-9	Azelaic acid	-0.2469
23651-95-8	Droxidopa	-0.2458
145040-37-5	Candesartan Cilexetil	-0.2433
127-07-1	Hydroxyurea	-0.2432
504-03-0	Lupetidin	-0.2418
4852-22-6	Proanthocyanidins	-0.2379
1953-02-2	Tiopronin	-0.2376
987-78-0	Citicoline	-0.2329
4852-22-6	Proanthocyanidins	-0.2324
33818-15-4	Citicoline sodium	-0.2323
543-15-7	Heptaminol hydrochloride	-0.2315
76963-41-2	Nizatidine	-0.2304
65666-07-1	Silymarin	-0.23

110871-86-8	Sparfloxacin	-0.2297
155148-31-5	Plerixafor 8HCl (AMD3100 8HCl)	-0.2295
110078-46-1	Plerixafor	-0.2295
52152-93-9	Cefsulodine sodium	-0.2291
79-55-0	Pempidine	-0.2272
144689-24-7	Olmesartan	-0.2185
4852-22-6	Proanthocyanidins	-0.2183
1230487-00-9	Siponimod	-0.2142
72559-06-9	Rifabutin	-0.2141
83-44-3	Deoxycholic acid	-0.2097
41100-52-1	Memantine hydrochloride	-0.2091
134678-17-4	Lamivudine	-0.2083
850140-73-7	Afatinib Dimaleate	-0.1997
543-82-8	2-Amino-6-methylheptane	-0.1957
1501-84-4	Rimantadine hydrochloride	-0.1954
65666-07-1	Silymarin	-0.1933
500287-72-9	Rilpivirine	-0.1925
5451-09-2	5-Aminolevulinic acid hydrochloride	-0.1909
65-86-1	Orotic acid	-0.187
543-15-7	Heptaminol hydrochloride	-0.1842
673-06-3	D-Phenylalanine	-0.1827
115939-25-8	Salvianolic acid B	-0.1812
7327-87-9	Dihydralazine sulphate	-0.1761
1953-02-2	Tiopronin	-0.1729
1501-84-4	Rimantadine hydrochloride	-0.1728
187227-45-8	Oseltamivir acid	-0.1716
1113-59-3	3-Bromopyruvic acid	-0.1703
7681-93-8	Pimaricin	-0.1691
68-35-9	Sulfadiazine	-0.1689
1365970-03-1	Glecaprevir	-0.1683
543-82-8	2-Amino-6-methylheptane	-0.1681
1098-97-1	Pyrithioxin	-0.1663
65666-07-1	Silymarin	-0.1607
7481-89-2	Zalcitabine	-0.1596
543-15-7	Heptaminol hydrochloride	-0.1578
4852-22-6	Proanthocyanidins	-0.1572
65666-07-1	Silymarin	-0.156
2022-85-7	Fluorocytosine	-0.1557
65666-07-1	Silymarin	-0.1537
1953-02-2	Tiopronin	-0.1526
550-24-3	Embelin	-0.151

151533-22-1	Levomefolate calcium	-0.149
936539-80-9	Betrixaban maleate	-0.1475
T4690	Betrixaban hydrochloride(330942-05-7(free base))	-0.1475
330942-05-7	Betrixaban	-0.1475
1953-02-2	Tiopronin	-0.1465
138402-11-6	Irbesartan	-0.1426
4852-22-6	Proanthocyanidins	-0.1421
850140-73-7	Afatinib Dimaleate	-0.1413
41100-52-1	Memantine hydrochloride	-0.1411
439081-18-2	Afatinib	-0.141
364622-82-2	Doripenem Hydrate	-0.1389
4852-22-6	Proanthocyanidins	-0.138
65666-07-1	Silymarin	-0.137
177036-94-1	Ambrisentan	-0.1359
1365970-03-1	Glecaprevir	-0.1331
4852-22-6	Proanthocyanidins	-0.1325
4852-22-6	Proanthocyanidins	-0.1319
151533-22-1	Levomefolate calcium	-0.1309
144675-97-8	Pixantrone dimaleate	-0.1299
4852-22-6	Proanthocyanidins	-0.1274
57248-88-1	Pamidronate disodium salt	-0.1274
543-82-8	2-Amino-6-methylheptane	-0.123
65666-07-1	Silymarin	-0.1229
23651-95-8	Droxidopa	-0.1226
69558-55-0	Thymopentin	-0.121
1257044-40-8	ABT199	-0.1142
153-18-4	Rutin	-0.1134
4852-22-6	Proanthocyanidins	-0.1129
557-08-4	Zinc undecylenate	-0.1128
112-38-9	Undecenoic acid	-0.1128
1257044-40-8	ABT199	-0.1111
65666-07-1	Silymarin	-0.1109
4852-22-6	Proanthocyanidins	-0.1104
4852-22-6	Proanthocyanidins	-0.1073
543-15-7	Heptaminol hydrochloride	-0.1052
138452-21-8	Fexofenadine hydrochloride	-0.1047
51-48-9	L-Thyroxine	-0.1029
4852-22-6	Proanthocyanidins	-0.0984
65666-07-1	Silymarin	-0.0944
872728-81-9	Dabigatran Etexilate Mesylate	-0.0911

211915-06-9	Dabigatran etexilate	-0.0911
121808-62-6	Pidotimod	-0.0884
149003-01-0	Dexrazoxane HCl	-0.0875
70356-03-5	Cefaclor monohydrate	-0.0858
557-08-4	Zinc undecylenate	-0.0858
112-38-9	Undecenoic acid	-0.0858
24390-14-5	Doxycycline hyclate	-0.0842
70356-03-5	Cefaclor monohydrate	-0.0807
151533-22-1	Levomefolate calcium	-0.0784
65666-07-1	Silymarin	-0.0765
67227-57-0	Fenoldopam mesylate	-0.0726
543-82-8	2-Amino-6-methylheptane	-0.0704
151533-22-1	Levomefolate calcium	-0.0648
4852-22-6	Proanthocyanidins	-0.0646
4852-22-6	Proanthocyanidins	-0.0604
1953-02-2	Tiopronin	-0.0568
115436-72-1	Risedronate Sodium	-0.0557
871038-72-1	Raltegravir potassium	-0.0544
2022-85-7	Fluorocytosine	-0.0524
3963-95-9	Methacycline hydrochloride	-0.0501
15663-27-1	Cisplatin	-0.0499
1953-02-2	Tiopronin	-0.0494
65666-07-1	Silymarin	-0.0487
69712-56-7	Cefotetan	-0.0475
220620-09-7	Tigecycline	-0.0473
10163-15-2	Disodium monofluorophosphate	-0.0425
151533-22-1	Levomefolate calcium	-0.042
17440-83-4	Amiloride hydrochloride dihydrate	-0.0376
1230487-00-9	Siponimod	-0.0365
7681-93-8	Pimaricin	-0.0303
22888-70-6	Silibinin	-0.0289
65666-07-1	Silymarin	-0.0286
1146-99-2	chlophenadione	-0.0277
151533-22-1	Levomefolate calcium	-0.0262
4852-22-6	Proanthocyanidins	-0.0224
500287-72-9	Rilpivirine	-0.0224
651-06-9	Sulfameter	-0.0222
3697-42-5	Chlorhexidine hydrochloride	-0.0172
56-95-1	Chlorhexidine Acetate	-0.0172
55-56-1	Chlorhexidine	-0.0172
65666-07-1	Silymarin	-0.0121
23651-95-8	Droxidopa	-0.0052

65666-07-1	Silymarin	0.0041
4852-22-6	Proanthocyanidins	0.0055
1115-70-4	Metformin hydrochloride	0.0059
145040-37-5	Candesartan Cilexetil	0.0121
698387-09-6	Neratinib	0.013
65666-07-1	Silymarin	0.0162
138452-21-8	Fexofenadine hydrochloride	0.0175
79-55-0	Pempidine	0.0206
149003-01-0	Dexrazoxane HCl	0.0216
4852-22-6	Proanthocyanidins	0.0238
4852-22-6	Proanthocyanidins	0.0246
112-80-1	Oleic acid	0.028
4852-22-6	Proanthocyanidins	0.0349
550-24-3	Embelin	0.0351
4852-22-6	Proanthocyanidins	0.0365
4852-22-6	Proanthocyanidins	0.0382
115436-72-1	Risedronate Sodium	0.0382
4852-22-6	Proanthocyanidins	0.0406
1115-70-4	Metformin hydrochloride	0.0471
80621-81-4	Rifaximin	0.0491
112-80-1	Oleic acid	0.0517
113852-37-2	Cidofovir	0.0521
3697-42-5	Chlorhexidine hydrochloride	0.0523
56-95-1	Chlorhexidine Acetate	0.0523
2022-85-7	Fluorocytosine	0.0553
65666-07-1	Silymarin	0.0585
1134-47-0	Baclofen	0.0594
65666-07-1	Silymarin	0.0607
673-06-3	D-Phenylalanine	0.0626
4852-22-6	Proanthocyanidins	0.0636
72559-06-9	Rifabutin	0.0646
4852-22-6	Proanthocyanidins	0.0776
4852-22-6	Proanthocyanidins	0.0808
144675-97-8	Pixantrone dimaleate	0.0894
144675-97-8	Pixantrone dimaleate	0.0954
72559-06-9	Rifabutin	0.0991
110871-86-8	Sparfloxacin	0.1036
4852-22-6	Proanthocyanidins	0.1128
3697-42-5	Chlorhexidine hydrochloride	0.1132
56-95-1	Chlorhexidine Acetate	0.1132
183204-72-0	Tipiracil hydrochloride	0.1135
118072-93-8	Zoledronic Acid	0.1142
546-88-3	Acetohydroxamic acid	0.1153

69308-37-8	(R)-baclofen	0.1242
1134-47-0	Baclofen	0.1242
550-24-3	Embelin	0.1282
24390-14-5	Doxycycline hyclate	0.1312
4852-22-6	Proanthocyanidins	0.1343
52152-93-9	Cefsulodine sodium	0.1371
4852-22-6	Proanthocyanidins	0.1391
4852-22-6	Proanthocyanidins	0.1396
81-27-6	Sennoside A	0.1412
65666-07-1	Silymarin	0.143
55-56-1	Chlorhexidine	0.1443
53179-09-2	Sisomicin Sulfate	0.1576
155148-31-5	Plerixafor 8HCl (AMD3100 8HCl)	0.1605
110078-46-1	Plerixafor	0.1605
4852-22-6	Proanthocyanidins	0.1624
557-08-4	Zinc undecylenate	0.1727
112-38-9	Undecenoic acid	0.1727
115436-72-1	Risedronate Sodium	0.1744
118072-93-8	Zoledronic Acid	0.1799
51-48-9	L-Thyroxine	0.1835
80621-81-4	Rifaximin	0.1848
500287-72-9	Rilpivirine	0.1909
55-56-1	Chlorhexidine	0.2003
65666-07-1	Silymarin	0.2005
5934-29-2	L-Histidine monohydrochloride monohydrat	0.2044
71-00-1	histidine	0.2044
673-06-3	D-Phenylalanine	0.2095
118072-93-8	Zoledronic Acid	0.2096
651-06-9	Sulfameter	0.2127
55-56-1	Chlorhexidine	0.2163
15663-27-1	Cisplatin	0.2184
24729-96-2	Clindamycin phosphate	0.2195
3697-42-5	Chlorhexidine hydrochloride	0.2202
56-95-1	Chlorhexidine Acetate	0.2202
151533-22-1	Levomefolate calcium	0.2203
4852-22-6	Proanthocyanidins	0.2215
41100-52-1	Memantine hydrochloride	0.2229
987-78-0	Citicoline	0.2262
4852-22-6	Proanthocyanidins	0.2267
33818-15-4	Citicoline sodium	0.2301
4852-22-6	Proanthocyanidins	0.2312
4852-22-6	Proanthocyanidins	0.2337
24729-96-2	Clindamycin phosphate	0.2346

1115-70-4	Metformin hydrochloride	0.2393
151533-22-1	Levomefolate calcium	0.2447
155148-31-5	Plerixafor 8HCl (AMD3100 8HCl)	0.2492
110078-46-1	Plerixafor	0.2492
4852-22-6	Proanthocyanidins	0.2555
4852-22-6	Proanthocyanidins	0.2561
151533-22-1	Levomefolate calcium	0.2651
4852-22-6	Proanthocyanidins	0.2662
24729-96-2	Clindamycin phosphate	0.268
211914-51-1	Dabigatran	0.272
4852-22-6	Proanthocyanidins	0.2723
1134-47-0	Baclofen	0.2738
4852-22-6	Proanthocyanidins	0.2744
4852-22-6	Proanthocyanidins	0.2773
53179-09-2	Sisomicin Sulfate	0.2853
543-82-8	2-Amino-6-methylheptane	0.286
149022-22-0	Amikacin Sulfate Salt	0.2909
1230487-00-9	Siponimod	0.2913
115939-25-8	Salvianolic acid B	0.296
81-27-6	Sennoside A	0.2978
151533-22-1	Levomefolate calcium	0.2998
4852-22-6	Proanthocyanidins	0.3003
4852-22-6	Proanthocyanidins	0.3011
65666-07-1	Silymarin	0.3018
4852-22-6	Proanthocyanidins	0.3021
4852-22-6	Proanthocyanidins	0.3108
4852-22-6	Proanthocyanidins	0.3117
24729-96-2	Clindamycin phosphate	0.3137
115939-25-8	Salvianolic acid B	0.3162
123-99-9	Azelaic acid	0.3198
3697-42-5	Chlorhexidine hydrochloride	0.3237
56-95-1	Chlorhexidine Acetate	0.3237
55-56-1	Chlorhexidine	0.3237
51-48-9	L-Thyroxine	0.3254
113852-37-2	Cidofovir	0.3268
149022-22-0	Amikacin Sulfate Salt	0.328
4852-22-6	Proanthocyanidins	0.3296
4852-22-6	Proanthocyanidins	0.3301
482-36-0	Hyperoside	0.3307
57-08-9	Acexamic Acid	0.3343
4852-22-6	Proanthocyanidins	0.3479
81-27-6	Sennoside A	0.348

24390-14-5	Doxycycline hyclate	0.3553
15663-27-1	Cisplatin	0.3581
65666-07-1	Silymarin	0.363
151533-22-1	Levomefolate calcium	0.3636
73816-42-9	Meclocycline sulfosalicylate salt	0.3637
65666-07-1	Silymarin	0.3755
118072-93-8	Zoledronic Acid	0.381
4852-22-6	Proanthocyanidins	0.3825
65666-07-1	Silymarin	0.3842
37517-28-5	Amikacin	0.3859
24390-14-5	Doxycycline hyclate	0.3892
211914-51-1	Dabigatran	0.3913
22888-70-6	Silibinin	0.3921
81-27-6	Sennoside A	0.3957
546-88-3	Acetohydroxamic acid	0.3972
149022-22-0	Amikacin Sulfate Salt	0.3983
4852-22-6	Proanthocyanidins	0.4001
138452-21-8	Fexofenadine hydrochloride	0.401
115939-25-8	Salvianolic acid B	0.4039
65666-07-1	Silymarin	0.4043
72559-06-9	Rifabutin	0.4089
4852-22-6	Proanthocyanidins	0.4096
91832-40-5	Cefdinir	0.4116
66357-59-3	Ranitidine Hydrochloride	0.4131
113852-37-2	Cidofovir	0.4162
118072-93-8	Zoledronic Acid	0.4178
37517-28-5	Amikacin	0.4237
389139-89-3	Omadacycline	0.4261
72559-06-9	Rifabutin	0.427
4852-22-6	Proanthocyanidins	0.4337
80621-81-4	Rifaximin	0.4353
112-38-9	Undecenoic acid	0.4353
82009-34-5	Cilastatin	0.4389
69558-55-0	Thymopentin	0.439
665-66-7	Amantadine hydrochloride	0.4449
113852-37-2	Cidofovir	0.4451
151533-22-1	Levomefolate calcium	0.4455
1405-37-4	Capastat sulfate	0.448
4852-22-6	Proanthocyanidins	0.4541
2295-58-1	Flopropione	0.4665
69308-37-8	(R)-baclofen	0.4684
1134-47-0	Baclofen	0.4684
113852-37-2	Cidofovir	0.4734

2295-58-1	Flopropione	0.4806
57-08-9	Acexamic Acid	0.4831
871038-72-1	Raltegravir potassium	0.4841
482-36-0	Hyperoside	0.4842
37517-28-5	Amikacin	0.4847
115436-72-1	Risedronate Sodium	0.4885
112-38-9	Undecenoic acid	0.4958
57-08-9	Acexamic Acid	0.5019
151533-22-1	Levomefolate calcium	0.5024
1134-47-0	Baclofen	0.5026
482-36-0	Hyperoside	0.5137
389139-89-3	Omadacycline	0.5153
123-99-9	Azelaic acid	0.5171
4852-22-6	Proanthocyanidins	0.519
53179-09-2	Sisomicin Sulfate	0.5205
987-78-0	Citicoline	0.5227
14783-68-7	Magnesium glycinate	0.5297
20150-34-9	Ferrous Bisglycinate	0.5297
183204-72-0	Tipiracil hydrochloride	0.5324
33818-15-4	Citicoline sodium	0.5325
4852-22-6	Proanthocyanidins	0.5436
151533-22-1	Levomefolate calcium	0.5459
4852-22-6	Proanthocyanidins	0.5472
871038-72-1	Raltegravir potassium	0.5515
73816-42-9	Meclocycline sulfosalicylate salt	0.5532
113852-37-2	Cidofovir	0.5566
2295-58-1	Flopropione	0.5642
24729-96-2	Clindamycin phosphate	0.5652
73816-42-9	Meclocycline sulfosalicylate salt	0.5778
151533-22-1	Levomefolate calcium	0.5791
73816-42-9	Meclocycline sulfosalicylate salt	0.5819
4852-22-6	Proanthocyanidins	0.5861
151533-22-1	Levomefolate calcium	0.5872
4852-22-6	Proanthocyanidins	0.5937
1110813-31-4	Dacomitinib	0.5944
4852-22-6	Proanthocyanidins	0.5997
151533-22-1	Levomefolate calcium	0.6027
651-06-9	Sulfameter	0.6039
4852-22-6	Proanthocyanidins	0.6069
5934-29-2	L-Histidine monohydrochloride monohydrat	0.6094
71-00-1	histidine	0.6094
113852-37-2	Cidofovir	0.6159

123-99-9	Azelaic acid	0.6166
4852-22-6	Proanthocyanidins	0.6189
81-27-6	Sennoside A	0.6208
698387-09-6	Neratinib	0.6261
546-88-3	Acetohydroxamic acid	0.6303
4852-22-6	Proanthocyanidins	0.6344
4852-22-6	Proanthocyanidins	0.6349
4852-22-6	Proanthocyanidins	0.6395
4852-22-6	Proanthocyanidins	0.648
183204-72-0	Tipiracil hydrochloride	0.6498
4852-22-6	Proanthocyanidins	0.6512
145040-37-5	Candesartan Cilexetil	0.6513
73816-42-9	Meclocycline sulfosalicylate salt	0.6674
115436-72-1	Risedronate Sodium	0.6704
118072-93-8	Zoledronic Acid	0.6807
4852-22-6	Proanthocyanidins	0.6873
51-35-4	L-Hydroxyprolin	0.6961
110871-86-8	Sparfloxacin	0.6968
69558-55-0	Thymopentin	0.6984
121808-62-6	Pidotimod	0.6988
389139-89-3	Omadacycline	0.7034
4852-22-6	Proanthocyanidins	0.7041
79-57-2	Oxytetracycline	0.7081
73816-42-9	Meclocycline sulfosalicylate salt	0.7107
73816-42-9	Meclocycline sulfosalicylate salt	0.7115
73816-42-9	Meclocycline sulfosalicylate salt	0.7132
4852-22-6	Proanthocyanidins	0.7173
4852-22-6	Proanthocyanidins	0.7254
151533-22-1	Levomefolate calcium	0.7263
73816-42-9	Meclocycline sulfosalicylate salt	0.7311
151533-22-1	Levomefolate calcium	0.7434
79-57-2	Oxytetracycline	0.746
79-57-2	Oxytetracycline	0.7649
4852-22-6	Proanthocyanidins	0.7727
81-27-6	Sennoside A	0.7727
139481-59-7	Candesartan	0.7791
151533-22-1	Levomefolate calcium	0.7796
4852-22-6	Proanthocyanidins	0.7845
665-66-7	Amantadine hydrochloride	0.7915
67814-76-0	Isepamicin sulfate	0.7919
444731-52-6	Pazopanib	0.795
3697-42-5	Chlorhexidine hydrochloride	0.7965
56-95-1	Chlorhexidine Acetate	0.7965

55-56-1	Chlorhexidine	0.7965
4852-22-6	Proanthocyanidins	0.799
121808-62-6	Pidotimod	0.8001
151533-22-1	Levomefolate calcium	0.8033
1197-18-8	Amstat	0.8034
112887-68-0	Raltitrexed	0.8078
1197-18-8	Amstat	0.8189
51-35-4	L-Hydroxyprolin	0.8252
4852-22-6	Proanthocyanidins	0.8278
3963-95-9	Methacycline hydrochloride	0.8301
151533-22-1	Levomefolate calcium	0.8325
4852-22-6	Proanthocyanidins	0.8342
4852-22-6	Proanthocyanidins	0.8362
115939-25-8	Salvianolic acid B	0.8375
3963-95-9	Methacycline hydrochloride	0.8378
546-88-3	Acetohydroxamic acid	0.8382
4852-22-6	Proanthocyanidins	0.8394
151533-22-1	Levomefolate calcium	0.8464
364622-82-2	Doripenem Hydrate	0.8475
151533-22-1	Levomefolate calcium	0.8575
4852-22-6	Proanthocyanidins	0.8601
112-38-9	Undecenoic acid	0.8607
4852-22-6	Proanthocyanidins	0.8619
4852-22-6	Proanthocyanidins	0.8629
4852-22-6	Proanthocyanidins	0.8647
24729-96-2	Clindamycin phosphate	0.8697
220620-09-7	Tigecycline	0.8708
908-54-3	Diminazene Aceturate	0.8824
72559-06-9	Rifabutin	0.883
145040-37-5	Candesartan Cilexetil	0.8843
66357-59-3	Ranitidine Hydrochloride	0.8976
1201902-80-8	MLN9708	0.9038
151533-22-1	Levomefolate calcium	0.9044
4852-22-6	Proanthocyanidins	0.9133
4852-22-6	Proanthocyanidins	0.9149
151533-22-1	Levomefolate calcium	0.92
50-01-1	Guanidine hydrochloride	0.9258
112887-68-0	Raltitrexed	0.9276
4852-22-6	Proanthocyanidins	0.9301
66357-59-3	Ranitidine Hydrochloride	0.933
4852-22-6	Proanthocyanidins	0.9391
220620-09-7	Tigecycline	0.9424

63-68-3	L-Methionine	0.9437
59-30-3	Folic acid	0.9454
56391-57-2	Netilmicin sulfate	0.9504
5934-29-2	L-Histidine monohydrochloride monohydrat	0.9507
71-00-1	histidine	0.9507
665-66-7	Amantadine hydrochloride	0.9519
1405-37-4	Capastat sulfate	0.9589
59-30-3	Folic acid	0.9637
1188-38-1	Carbaglu	0.9678
320-67-2	5-Azacytidine	0.9703
1226781-44-7	Omarigliptin	0.9746
1115-70-4	Metformin hydrochloride	0.9748
25389-94-0	Kanamycin sulfate	0.9866
38821-49-7	Carbidopa Hydrate	0.9873
151533-22-1	Levomefolate calcium	0.9884
755038-65-4	Volasertib	0.9909
4852-22-6	Proanthocyanidins	0.9981
14783-68-7	Magnesium glycinate	1.0038
20150-34-9	Ferrous Bisglycinate	1.0038
1115-70-4	Metformin hydrochloride	1.0053
151533-22-1	Levomefolate calcium	1.0102
4852-22-6	Proanthocyanidins	1.0117
59-05-2	Methotrexate	1.0295
67814-76-0	Isepamicin sulfate	1.0301
56391-57-2	Netilmicin sulfate	1.0374
4852-22-6	Proanthocyanidins	1.0439
151533-22-1	Levomefolate calcium	1.0467
151533-22-1	Levomefolate calcium	1.0551
72-18-4	L-Valine	1.056
112887-68-0	Raltitrexed	1.0587
1226781-44-7	Omarigliptin	1.0663
151533-22-1	Levomefolate calcium	1.0699
69558-55-0	Thymopentin	1.0709
56-40-6	glycine	1.0721
4852-22-6	Proanthocyanidins	1.0724
139481-59-7	Candesartan	1.0752
24390-14-5	Doxycycline hyclate	1.0778
59-05-2	Methotrexate	1.0789
4852-22-6	Proanthocyanidins	1.0838
56-40-6	glycine	1.0868

1110813-31-4	Dacomitinib	1.0928
1110813-31-4	Dacomitinib	1.0954
1115-70-4	Metformin hydrochloride	1.0992
51-35-4	L-Hydroxyprolin	1.107
56-41-7	alanine	1.1094
38821-49-7	Carbidopa Hydrate	1.112
73-32-5	isoleucine	1.1214
546-88-3	Acetohydroxamic acid	1.1253
151533-22-1	Levomefolate calcium	1.1306
38821-49-7	Carbidopa Hydrate	1.1306
63-68-3	L-Methionine	1.1324
82956-11-4	Nafamostat mesylate	1.1398
25389-94-0	Kanamycin sulfate	1.1466
151533-22-1	Levomefolate calcium	1.1479
144143-96-4	Eprosartan Mesylate	1.1608
259793-96-9	Favipiravir	1.1667
4852-22-6	Proanthocyanidins	1.1697
151533-22-1	Levomefolate calcium	1.1709
4852-22-6	Proanthocyanidins	1.1716
70-47-3	L-Asparagine	1.1789
22457-89-2	Benfotiamine	1.1844
645-43-2	Guanethidine Monosulfate	1.1876
59-05-2	Methotrexate	1.1969
56391-57-2	Netilmicin sulfate	1.2007
469-77-2	Spiculisporic Acid	1.2072
76824-35-6	Famotidine	1.2147
112887-68-0	Raltitrexed	1.2177
1188-38-1	Carbaglu	1.2189
76824-35-6	Famotidine	1.2193
1197-18-8	Amstat	1.2233
144143-96-4	Eprosartan Mesylate	1.232
56-41-7	alanine	1.233
320-67-2	5-Azacytidine	1.2369
56-85-9	L-Glutamine	1.2381
67814-76-0	Isepamicin sulfate	1.2512
1201902-80-8	MLN9708	1.2546
73-32-5	isoleucine	1.2715
66357-59-3	Ranitidine Hydrochloride	1.2732
50-01-1	Guanidine hydrochloride	1.2739
320-67-2	5-Azacytidine	1.2745

22204-24-6	Pyrantel pamoate	1.2785
3697-42-5	Chlorhexidine hydrochloride	1.2808
56-95-1	Chlorhexidine Acetate	1.2808
55-56-1	Chlorhexidine	1.2808
112887-68-0	Raltitrexed	1.2847
1405-37-4	Capastat sulfate	1.2949
70-47-3	L-Asparagine	1.2992
908-54-3	Diminazene Aceturate	1.3031
1405-37-4	Capastat sulfate	1.3045
55-56-1	Chlorhexidine	1.306
444731-52-6	Pazopanib	1.3086
4852-22-6	Proanthocyanidins	1.3102
14783-68-7	Magnesium glycinate	1.3114
20150-34-9	Ferrous Bisglycinate	1.3114
61-90-5	L-Leucine	1.3211
56-40-6	glycine	1.3222
56-41-7	alanine	1.3309
25389-94-0	Kanamycin sulfate	1.3363
52-66-4	DL-Penicillamine	1.3367
546-88-3	Acetohydroxamic acid	1.3369
76824-35-6	Famotidine	1.3376
1226781-44-7	Omarigliptin	1.3386
52-67-5	d-penicillamin	1.3398
1405-37-4	Capastat sulfate	1.3505
22204-24-6	Pyrantel pamoate	1.3516
1188-38-1	Carbaglu	1.353
59-05-2	Methotrexate	1.3549
72-18-4	L-Valine	1.3624
112887-68-0	Raltitrexed	1.366
5934-29-2	L-Histidine monohydrochloride monohydrat	1.3672
71-00-1	histidine	1.3672
211914-51-1	Dabigatran	1.3676
69558-55-0	Thymopentin	1.3683
635702-64-6	Pazopanib HCl	1.384
52-66-4	DL-Penicillamine	1.3852
66357-59-3	Ranitidine Hydrochloride	1.3873
67-43-6	Pentetic Acid	1.393
142-47-2	L-Glutamic acid monosodium salt	1.3945
52-66-4	DL-Penicillamine	1.395
1405-10-3	Neomycin sulfate	1.3951
59-05-2	Methotrexate	1.4024
142-47-2	L-Glutamic acid monosodium salt	1.4088

70-47-3	L-Asparagine	1.4188
76824-35-6	Famotidine	1.4196
469-77-2	Spiculisporic Acid	1.4202
259793-96-9	Favipiravir	1.4237
1201902-80-8	MLN9708	1.4299
115939-25-8	Salvianolic acid B	1.4312
79-57-2	Oxytetracycline	1.4417
151533-22-1	Levomefolate calcium	1.4443
1231930-82-7	LY2835219	1.4467
469-77-2	Spiculisporic Acid	1.4551
110-15-6	Succinic acid	1.459
52-66-4	DL-Penicillamine	1.4593
389139-89-3	Omadacycline	1.4624
147-85-3	proline	1.464
645-43-2	Guanethidine Monosulfate	1.4661
52-66-4	DL-Penicillamine	1.4675
63-68-3	L-Methionine	1.4724
56-85-9	L-Glutamine	1.4734
444731-52-6	Pazopanib	1.4826
211914-51-1	Dabigatran	1.4866
4146-30-9	Neomycin Sulphate B	1.487
123-99-9	Azelaic acid	1.4914
1405-10-3	Neomycin sulfate	1.4951
3963-95-9	Methacycline hydrochloride	1.497
151533-22-1	Levomefolate calcium	1.5018
123-99-9	Azelaic acid	1.5066
139481-59-7	Candesartan	1.5106
590-46-5	Betaine hydrochloride	1.5165
59-30-3	Folic acid	1.5197
55-56-1	Chlorhexidine	1.527
76824-35-6	Famotidine	1.5278
83915-83-7	Lisinopril dihydrate	1.5282
1405-37-4	Capastat sulfate	1.5304
79350-37-1	Cefixime	1.5321
590-46-5	Betaine hydrochloride	1.5332
110-15-6	Succinic acid	1.5343
76824-35-6	Famotidine	1.5356
908-54-3	Diminazene Aceturate	1.536
139481-59-7	Candesartan	1.5401
181183-52-8	Almotriptan Malate	1.5535
22204-24-6	Pyrantel pamoate	1.556

389139-89-3	Omadacycline	1.566
56-85-9	L-Glutamine	1.5702
72-18-4	L-Valine	1.5702
1405-37-4	Capastat sulfate	1.574
123-99-9	Azelaic acid	1.5745
139110-80-8	Zanamivir	1.575
61-90-5	L-Leucine	1.579
76824-35-6	Famotidine	1.5814
3963-95-9	Methacycline hydrochloride	1.5844
147-85-3	proline	1.5894
151533-22-1	Levomefolate calcium	1.5957
4146-30-9	Neomycin Sulphate B	1.5959
469-77-2	Spiculisporic Acid	1.5992
181183-52-8	Almotriptan Malate	1.6078
144143-96-4	Eprosartan Mesylate	1.6204
68813-55-8	Oxantel Pamoate	1.6382
1231929-97-7	Abemaciclib	1.643
1405-37-4	Capastat sulfate	1.6471
1201902-80-8	MLN9708	1.6516
50-01-1	Guanidine hydrochloride	1.6582
1405-37-4	Capastat sulfate	1.6583
917389-32-3	Letermovir	1.6624
5934-29-2	L-Histidine monohydrochloride monohydrat	1.6633
71-00-1	histidine	1.6633
76824-35-6	Famotidine	1.6661
52-66-4	DL-Penicillamine	1.6724
1405-37-4	Capastat sulfate	1.6763
917389-32-3	Letermovir	1.6764
4852-22-6	Proanthocyanidins	1.6791
115939-25-8	Salvianolic acid B	1.6842
181183-52-8	Almotriptan Malate	1.6845
1201902-80-8	MLN9708	1.6863
645-43-2	Guanethidine Monosulfate	1.6997
469-77-2	Spiculisporic Acid	1.7024
59-30-3	Folic acid	1.7024
469-77-2	Spiculisporic Acid	1.7053
67-43-6	Pentetic Acid	1.7116
389139-89-3	Omadacycline	1.7152
150915-40-5	Tirofiban hydrochloride monohydrate	1.7183
59-30-3	Folic acid	1.7217

7327-87-9	Dihydralazine sulphate	1.7228
24390-14-5	Doxycycline hyclate	1.7234
32986-56-4	Tobramycin	1.7234
139110-80-8	Zanamivir	1.7281
590-46-5	Betaine hydrochloride	1.7298
637-32-1	Proguanil hydrochloride	1.7339
144689-24-7	Olmesartan	1.7347
60142-95-2	Gabapentin HCl	1.7384
60142-96-3	Gabapentin	1.7384
67-43-6	Pentetic Acid	1.7385
24390-14-5	Doxycycline hyclate	1.7401
79-57-2	Oxytetracycline	1.7414
142-47-2	L-Glutamic acid monosodium salt	1.7427
259793-96-9	Favipiravir	1.7478
55-56-1	Chlorhexidine	1.7499
52-67-5	d-penicillamin	1.7512
143388-64-1	Naratriptan	1.7548
139110-80-8	Zanamivir	1.7626
66357-59-3	Ranitidine Hydrochloride	1.7719
5934-29-2	L-Histidine monohydrochloride monohydrat	1.7741
71-00-1	histidine	1.7741
73-32-5	isoleucine	1.7776
1405-37-4	Capastat sulfate	1.7787
211914-51-1	Dabigatran	1.7789
110-15-6	Succinic acid	1.7863
181183-52-8	Almotriptan Malate	1.7886
73816-42-9	Meclocycline sulfosalicylate salt	1.7934
1405-37-4	Capastat sulfate	1.801
143388-64-1	Naratriptan	1.81
76824-35-6	Famotidine	1.8198
1405-37-4	Capastat sulfate	1.827
69056-38-8	Sapropterin Hydrochloride	1.8281
2353-33-5	Decitabine	1.8311
4852-22-6	Proanthocyanidins	1.8366
79-57-2	Oxytetracycline	1.8438
1231929-97-7	Abemaciclib	1.8461
259793-96-9	Favipiravir	1.8478
51481-61-9	Cimetidine	1.8533
69056-38-8	Sapropterin Hydrochloride	1.8585
469-77-2	Spiculisporic Acid	1.8641
79-57-2	Oxytetracycline	1.8642
3697-42-5	Chlorhexidine hydrochloride	1.8747

56-95-1	Chlorhexidine Acetate	1.8747
1405-37-4	Capastat sulfate	1.8754
79-57-2	Oxytetracycline	1.8798
469-77-2	Spiculisporic Acid	1.8829
140-64-7	Pentamidine isethionate	1.8848
469-77-2	Spiculisporic Acid	1.8948
140-64-7	Pentamidine isethionate	1.8982
550-24-3	Embelin	1.8998
211914-51-1	Dabigatran	1.9014
151533-22-1	Levomefolate calcium	1.9073
61825-94-3	Oxaliplatin	1.9083
181183-52-8	Almotriptan Malate	1.9127
4852-22-6	Proanthocyanidins	1.915
52-90-4	cysteine	1.9152
635702-64-6	Pazopanib HCl	1.9255
3963-95-9	Methacycline hydrochloride	1.9266
52-90-4	cysteine	1.9288
469-77-2	Spiculisporic Acid	1.9295
52-90-4	cysteine	1.9319
144689-24-7	Olmesartan	1.9366
151533-22-1	Levomefolate calcium	1.9392
1937-19-5	Aminoguanidine hydrochloride	1.9466
144689-24-7	Olmesartan	1.9528
4852-22-6	Proanthocyanidins	1.9563
4852-22-6	Proanthocyanidins	1.9572
69056-38-8	Sapropterin Hydrochloride	1.9599
82956-11-4	Nafamostat mesylate	1.9614
140-64-7	Pentamidine isethionate	1.9672
61825-94-3	Oxaliplatin	1.9698
3697-42-5	Chlorhexidine hydrochloride	1.9707
56-95-1	Chlorhexidine Acetate	1.9707
60142-95-2	Gabapentin HCl	1.9737
60142-96-3	Gabapentin	1.9737
72-19-5	threonine	1.9784
1201902-80-8	MLN9708	1.9807
83905-01-5	Azithromycin	1.9856
147-85-3	proline	1.9862
150915-40-5	Tirofiban hydrochloride monohydrate	1.9875
14605-22-2	Tauroursodeoxycholic Acid	1.9899
51481-61-9	Cimetidine	1.9904
259793-96-9	Favipiravir	1.9954
143388-64-1	Naratriptan	1.9994

52-67-5	d-penicillamin	2.0032
61-90-5	L-Leucine	2.0063
83915-83-7	Lisinopril dihydrate	2.0073
55-56-1	Chlorhexidine	2.0106
4852-22-6	Proanthocyanidins	2.0138
1231930-82-7	LY2835219	2.0164
469-77-2	Spiculisporic Acid	2.0223
1937-19-5	Aminoguanidine hydrochloride	2.0298
51481-61-9	Cimetidine	2.0315
181183-52-8	Almotriptan Malate	2.0355
1405-37-4	Capastat sulfate	2.0384
79-57-2	Oxytetracycline	2.0409
4852-22-6	Proanthocyanidins	2.0412
74863-84-6	Argatroban	2.0433
73816-42-9	Meclocycline sulfosalicylate salt	2.0458
4852-22-6	Proanthocyanidins	2.0476
69056-38-8	Sapropterin Hydrochloride	2.0495
60142-95-2	Gabapentin HCl	2.053
60142-96-3	Gabapentin	2.053
469-77-2	Spiculisporic Acid	2.0546
1937-19-5	Aminoguanidine hydrochloride	2.0586
41340-25-4	Etodolac	2.0615
32986-56-4	Tobramycin	2.0622
51481-61-9	Cimetidine	2.0715
73816-42-9	Meclocycline sulfosalicylate salt	2.0721
635702-64-6	Pazopanib HCl	2.0802
3963-95-9	Methacycline hydrochloride	2.0804
143388-64-1	Naratriptan	2.0886
13614-98-7	Minocycline hydrochloride	2.0886
139110-80-8	Zanamivir	2.0915
4852-22-6	Proanthocyanidins	2.0945
259793-96-9	Favipiravir	2.0971
74863-84-6	Argatroban	2.1002
220620-09-7	Tigecycline	2.1019
79-57-2	Oxytetracycline	2.1043
637-32-1	Proguanil hydrochloride	2.1095
917389-32-3	Letermovir	2.1109
4852-22-6	Proanthocyanidins	2.1126
30299-08-2	Clinofibrate	2.1161
151533-22-1	Levomefolate calcium	2.1171
144689-24-7	Olmesartan	2.1224
79-57-2	Oxytetracycline	2.126

3963-95-9	Methacycline hydrochloride	2.1333
68813-55-8	Oxantel Pamoate	2.1426
7327-87-9	Dihydralazine sulphate	2.1466
637-32-1	Proguanil hydrochloride	2.1474
41340-25-4	Etodolac	2.1632
4852-22-6	Proanthocyanidins	2.174
79-57-2	Oxytetracycline	2.175
1231929-97-7	Abemaciclib	2.1758
61825-94-3	Oxaliplatin	2.1815
14605-22-2	Tauroursodeoxycholic Acid	2.1918
79-57-2	Oxytetracycline	2.1957
220620-09-7	Tigecycline	2.1961
637-32-1	Proguanil hydrochloride	2.1984
55-56-1	Chlorhexidine	2.2035
41340-25-4	Etodolac	2.2036
151533-22-1	Levomefolate calcium	2.2107
3963-95-9	Methacycline hydrochloride	2.212
79350-37-1	Cefixime	2.2166
1231930-82-7	LY2835219	2.2211
14605-22-2	Tauroursodeoxycholic Acid	2.2234
68813-55-8	Oxantel Pamoate	2.225
4852-22-6	Proanthocyanidins	2.2373
3697-42-5	Chlorhexidine hydrochloride	2.2404
56-95-1	Chlorhexidine Acetate	2.2404
117976-90-6	Rebeprazole sodium	2.2419
41340-25-4	Etodolac	2.2517
59-05-2	Methotrexate	2.2577
4852-22-6	Proanthocyanidins	2.2617
4852-22-6	Proanthocyanidins	2.2636
72-19-5	threonine	2.2655
13614-98-7	Minocycline hydrochloride	2.2745
4852-22-6	Proanthocyanidins	2.279
3697-42-5	Chlorhexidine hydrochloride	2.2989
56-95-1	Chlorhexidine Acetate	2.2989
1405-37-4	Capastat sulfate	2.3014
151533-22-1	Levomefolate calcium	2.3026
1405-37-4	Capastat sulfate	2.307
41340-25-4	Etodolac	2.3079
13614-98-7	Minocycline hydrochloride	2.3109
7327-87-9	Dihydralazine sulphate	2.3122
59-30-3	Folic acid	2.3145

41340-25-4	Etodolac	2.3279
138844-81-2	Ibandronate sodium	2.3327
79-57-2	Oxytetracycline	2.3328
364622-82-2	Doripenem Hydrate	2.3347
220620-09-7	Tigecycline	2.3355
79350-37-1	Cefixime	2.3422
100299-08-9	Pemirolast potassium	2.3469
550-24-3	Embelin	2.3536
139481-59-7	Candesartan	2.3614
389139-89-3	Omadacycline	2.3622
550-24-3	Embelin	2.3628
1110813-31-4	Dacomitinib	2.3661
69056-38-8	Sapropterin Hydrochloride	2.3703
3963-95-9	Methacycline hydrochloride	2.3741
74863-84-6	Argatroban	2.3899
143388-64-1	Naratriptan	2.3935
4852-22-6	Proanthocyanidins	2.3938
3963-95-9	Methacycline hydrochloride	2.399
4852-22-6	Proanthocyanidins	2.405
1405-37-4	Capastat sulfate	2.4238
74863-84-6	Argatroban	2.4265
41340-25-4	Etodolac	2.4278
41340-25-4	Etodolac	2.4286
3963-95-9	Methacycline hydrochloride	2.433
74863-84-6	Argatroban	2.4381
139110-80-8	Zanamivir	2.4397
30299-08-2	Clinofibrate	2.4418
2353-33-5	Decitabine	2.4437
138844-81-2	Ibandronate sodium	2.453
38821-49-7	Carbidopa Hydrate	2.4548
143388-64-1	Naratriptan	2.4607
64-75-5	Tetracycline hydrochloride	2.4769
83915-83-7	Lisinopril dihydrate	2.4791
3963-95-9	Methacycline hydrochloride	2.4818
69056-38-8	Sapropterin Hydrochloride	2.4877
60-54-8	Tetracycline	2.4877
3963-95-9	Methacycline hydrochloride	2.495
4852-22-6	Proanthocyanidins	2.4972
2353-33-5	Decitabine	2.5009
138844-81-2	Ibandronate sodium	2.5013
550-24-3	Embelin	2.5042
64-72-2	Chlortetracycline hydrochloride	2.5138

51481-61-9	Cimetidine	2.5154
138844-81-2	Ibandronate sodium	2.5218
550-24-3	Embelin	2.5221
550-24-3	Embelin	2.5235
151533-22-1	Levomefolate calcium	2.5287
1197953-54-0	Brigatinib	2.5338
1110813-31-4	Dacomitinib	2.5344
4852-22-6	Proanthocyanidins	2.5388
3963-95-9	Methacycline hydrochloride	2.5403
220620-09-7	Tigecycline	2.5498
4852-22-6	Proanthocyanidins	2.5512
389139-89-3	Omadacycline	2.5574
100299-08-9	Pemirolast potassium	2.5717
117976-90-6	Rebeprazole sodium	2.5751
72-19-5	threonine	2.5829
4852-22-6	Proanthocyanidins	2.5844
64-75-5	Tetracycline hydrochloride	2.585
41340-25-4	Etodolac	2.5855
304-55-2	Succimer	2.5931
4852-22-6	Proanthocyanidins	2.5965
64-72-2	Chlortetracycline hydrochloride	2.5987
4852-22-6	Proanthocyanidins	2.6024
4852-22-6	Proanthocyanidins	2.603
1405-10-3	Neomycin sulfate	2.6062
104376-79-6	Ceftriaxone sodium	2.614
4852-22-6	Proanthocyanidins	2.6148
32986-56-4	Tobramycin	2.6222
4852-22-6	Proanthocyanidins	2.6283
389139-89-3	Omadacycline	2.6283
24390-14-5	Doxycycline hyclate	2.6296
41340-25-4	Etodolac	2.6314
13189-98-5	Fudosteine	2.6318
3810-74-0	Streptomycin sulfate	2.6696
13189-98-5	Fudosteine	2.6717
82956-11-4	Nafamostat mesylate	2.6741
4852-22-6	Proanthocyanidins	2.6849
38821-49-7	Carbidopa Hydrate	2.6873
17696-69-4	Hematoporphyrin dihydrochloride	2.6925
150915-40-5	Tirofiban hydrochloride monohydrate	2.6953
4146-30-9	Neomycin Sulphate B	2.6975
364622-82-2	Doripenem Hydrate	2.7059

117976-90-6	Rebeprazole sodium	2.7073
304-55-2	Succimer	2.7114
30299-08-2	Clinofibrate	2.7117
637-32-1	Proguanil hydrochloride	2.7123
80621-81-4	Rifaximin	2.7128
3697-42-5	Chlorhexidine hydrochloride	2.7147
56-95-1	Chlorhexidine Acetate	2.7147
55-56-1	Chlorhexidine	2.7147
138844-81-2	Ibandronate sodium	2.7165
5490-27-7	Dihydrostreptomycin sulfate	2.72
30299-08-2	Clinofibrate	2.724
24390-14-5	Doxycycline hyclate	2.7275
41340-25-4	Etodolac	2.738
80621-81-4	Rifaximin	2.742
38821-49-7	Carbidopa Hydrate	2.7455
67-43-6	Pentetic Acid	2.7522
100299-08-9	Pemirolast potassium	2.7654
74863-84-6	Argatroban	2.7711
860-79-7	Cyclovirobuxin D	2.7764
30299-08-2	Clinofibrate	2.786
41340-25-4	Etodolac	2.7902
4852-22-6	Proanthocyanidins	2.793
155148-31-5	Plerixafor 8HCl (AMD3100 8HCl)	2.7961
110078-46-1	Plerixafor	2.7961
4852-22-6	Proanthocyanidins	2.7971
151533-22-1	Levomefolate calcium	2.8001
53882-12-5	Lodoxamide	2.812
100299-08-9	Pemirolast potassium	2.8134
144689-24-7	Olmesartan	2.8216
4852-22-6	Proanthocyanidins	2.8249
4852-22-6	Proanthocyanidins	2.8351
304-55-2	Succimer	2.8423
4852-22-6	Proanthocyanidins	2.8423
151533-22-1	Levomefolate calcium	2.8476
4852-22-6	Proanthocyanidins	2.848
4342-03-4	Dacarbazine	2.8534
4852-22-6	Proanthocyanidins	2.8536
4852-22-6	Proanthocyanidins	2.8581
117976-90-6	Rebeprazole sodium	2.8586
4852-22-6	Proanthocyanidins	2.8608
80621-81-4	Rifaximin	2.8627
364622-82-2	Doripenem Hydrate	2.8716
4852-22-6	Proanthocyanidins	2.8721

4852-22-6	Proanthocyanidins	2.8724
4852-22-6	Proanthocyanidins	2.8743
4852-22-6	Proanthocyanidins	2.8785
139264-17-8	Zolmitriptan	2.8805
139264-17-8	Zolmitriptan	2.8877
13189-98-5	Fudosteine	2.8927
56-45-1	serine	2.8931
139264-17-8	Zolmitriptan	2.8989
79-57-2	Oxytetracycline	2.8998
4852-22-6	Proanthocyanidins	2.9014
4852-22-6	Proanthocyanidins	2.9026
364622-82-2	Doripenem Hydrate	2.9033
4852-22-6	Proanthocyanidins	2.9076
4852-22-6	Proanthocyanidins	2.9162
4342-03-4	Dacarbazine	2.9331
1110813-31-4	Dacomitinib	2.9383
139264-17-8	Zolmitriptan	2.9454
24390-14-5	Doxycycline hyclate	2.9481
4852-22-6	Proanthocyanidins	2.9488
79-57-2	Oxytetracycline	2.9545
4852-22-6	Proanthocyanidins	2.9612
4852-22-6	Proanthocyanidins	2.9621
139264-17-8	Zolmitriptan	2.9644
51481-61-9	Cimetidine	2.9665
4852-22-6	Proanthocyanidins	2.9694
4852-22-6	Proanthocyanidins	2.9733
60-54-8	Tetracycline	2.9752
100299-08-9	Pemirolast potassium	2.9816
4852-22-6	Proanthocyanidins	2.9859
4852-22-6	Proanthocyanidins	2.9931
1207456-01-6	Talazoparib	2.9979
30299-08-2	Clinofibrate	2.9992
3963-95-9	Methacycline hydrochloride	3.0037
17696-69-4	Hematoporphyrin dihydrochloride	3.0114
1405-37-4	Capastat sulfate	3.0145
4852-22-6	Proanthocyanidins	3.0161
3963-95-9	Methacycline hydrochloride	3.0167
4852-22-6	Proanthocyanidins	3.0171
139264-17-8	Zolmitriptan	3.019
4852-22-6	Proanthocyanidins	3.0226
637-32-1	Proguanil hydrochloride	3.0233

17696-69-4	Hematoporphyrin dihydrochloride	3.0255
4852-22-6	Proanthocyanidins	3.0261
5490-27-7	Dihydrostreptomycin sulfate	3.0377
4852-22-6	Proanthocyanidins	3.044
1207456-01-6	Talazoparib	3.0489
4852-22-6	Proanthocyanidins	3.0651
4852-22-6	Proanthocyanidins	3.0658
147403-03-0	Azilsartan	3.0659
4342-03-4	Dacarbazine	3.0698
4852-22-6	Proanthocyanidins	3.0738
4852-22-6	Proanthocyanidins	3.0772
64-72-2	Chlortetracycline hydrochloride	3.0789
4852-22-6	Proanthocyanidins	3.0846
60925-61-3	Ceforanide	3.0859
1207456-01-6	Talazoparib	3.0908
4852-22-6	Proanthocyanidins	3.0957
1405-37-4	Capastat sulfate	3.0977
4852-22-6	Proanthocyanidins	3.0986
4852-22-6	Proanthocyanidins	3.0987
151533-22-1	Levomefolate calcium	3.1082
4852-22-6	Proanthocyanidins	3.1127
4852-22-6	Proanthocyanidins	3.1179
4852-22-6	Proanthocyanidins	3.12
1197953-54-0	Brigatinib	3.1208
4852-22-6	Proanthocyanidins	3.122
4852-22-6	Proanthocyanidins	3.1229
60-54-8	Tetracycline	3.1271
4852-22-6	Proanthocyanidins	3.139
30299-08-2	Clinofibrate	3.1422
3697-42-5	Chlorhexidine hydrochloride	3.1469
56-95-1	Chlorhexidine Acetate	3.1469
55-56-1	Chlorhexidine	3.1469
5490-27-7	Dihydrostreptomycin sulfate	3.1483
4852-22-6	Proanthocyanidins	3.1497
139110-80-8	Zanamivir	3.1499
3963-95-9	Methacycline hydrochloride	3.15
138844-81-2	Ibandronate sodium	3.1518
4852-22-6	Proanthocyanidins	3.1578
4852-22-6	Proanthocyanidins	3.1614
4852-22-6	Proanthocyanidins	3.1697

4852-22-6	Proanthocyanidins	3.1708
4852-22-6	Proanthocyanidins	3.171
151533-22-1	Levomefolate calcium	3.1718
4852-22-6	Proanthocyanidins	3.172
220620-09-7	Tigecycline	3.1725
1207456-01-6	Talazoparib	3.1728
4852-22-6	Proanthocyanidins	3.1757
3697-42-5	Chlorhexidine hydrochloride	3.1757
56-95-1	Chlorhexidine Acetate	3.1757
55-56-1	Chlorhexidine	3.1757
860-79-7	Cyclovirobuxin D	3.1759
67-43-6	Pentetic Acid	3.1775
4852-22-6	Proanthocyanidins	3.1775
17696-69-4	Hematoporphyrin dihydrochloride	3.1776
3810-74-0	Streptomycin sulfate	3.182
24390-14-5	Doxycycline hyclate	3.1843
4852-22-6	Proanthocyanidins	3.1857
4852-22-6	Proanthocyanidins	3.1885
4852-22-6	Proanthocyanidins	3.19
4852-22-6	Proanthocyanidins	3.2031
4852-22-6	Proanthocyanidins	3.2119
4852-22-6	Proanthocyanidins	3.2121
17696-69-4	Hematoporphyrin dihydrochloride	3.2137
4852-22-6	Proanthocyanidins	3.2173
3697-42-5	Chlorhexidine hydrochloride	3.2196
56-95-1	Chlorhexidine Acetate	3.2196
55-56-1	Chlorhexidine	3.2196
4852-22-6	Proanthocyanidins	3.2216
4852-22-6	Proanthocyanidins	3.2222
30299-08-2	Clinofibrate	3.2236
4852-22-6	Proanthocyanidins	3.2256
1041434-82-5	Peramivir Trihydrate	3.2274
4852-22-6	Proanthocyanidins	3.2279
17696-69-4	Hematoporphyrin dihydrochloride	3.2313
4852-22-6	Proanthocyanidins	3.2322
56-45-1	serine	3.2327
4852-22-6	Proanthocyanidins	3.2404
1405-37-4	Capastat sulfate	3.2432
364622-82-2	Doripenem Hydrate	3.2447
4852-22-6	Proanthocyanidins	3.2452
3810-74-0	Streptomycin sulfate	3.2477

151533-22-1	Levomefolate calcium	3.252
4852-22-6	Proanthocyanidins	3.2545
155148-31-5	Plerixafor 8HCl (AMD3100 8HCl)	3.2587
110078-46-1	Plerixafor	3.2587
4342-03-4	Dacarbazine	3.2593
4852-22-6	Proanthocyanidins	3.261
73816-42-9	Meclocycline sulfosalicylate salt	3.2621
30299-08-2	Clinofibrate	3.2625
4852-22-6	Proanthocyanidins	3.2631
4852-22-6	Proanthocyanidins	3.2667
56-45-1	serine	3.2693
4852-22-6	Proanthocyanidins	3.2708
4852-22-6	Proanthocyanidins	3.2753
4852-22-6	Proanthocyanidins	3.2814
4852-22-6	Proanthocyanidins	3.2843
4852-22-6	Proanthocyanidins	3.2864
4852-22-6	Proanthocyanidins	3.2876
1405-37-4	Capastat sulfate	3.2911
4852-22-6	Proanthocyanidins	3.2914
4852-22-6	Proanthocyanidins	3.2933
4852-22-6	Proanthocyanidins	3.2937
4852-22-6	Proanthocyanidins	3.2998
147403-03-0	Azilsartan	3.3008
4852-22-6	Proanthocyanidins	3.3169
1405-41-0	Gentamycin sulfate	3.3173
4852-22-6	Proanthocyanidins	3.3176
4852-22-6	Proanthocyanidins	3.3259
17696-69-4	Hematoporphyrin dihydrochloride	3.3275
14882-18-9	Bismuth subsalicylat	3.3287
151533-22-1	Levomefolate calcium	3.3311
4852-22-6	Proanthocyanidins	3.3312
137862-53-4	Valsartan	3.3317
60925-61-3	Ceforanide	3.3399
17696-69-4	Hematoporphyrin dihydrochloride	3.3436
4852-22-6	Proanthocyanidins	3.3507
4852-22-6	Proanthocyanidins	3.3512
151533-22-1	Levomefolate calcium	3.354
4342-03-4	Dacarbazine	3.3555
4852-22-6	Proanthocyanidins	3.3586
1405-37-4	Capastat sulfate	3.3603
4852-22-6	Proanthocyanidins	3.3618
4852-22-6	Proanthocyanidins	3.3654
4852-22-6	Proanthocyanidins	3.368

151533-22-1	Levomefolate calcium	3.3688
4852-22-6	Proanthocyanidins	3.3741
4852-22-6	Proanthocyanidins	3.3745
4852-22-6	Proanthocyanidins	3.3752
4852-22-6	Proanthocyanidins	3.3757
4852-22-6	Proanthocyanidins	3.3794
4852-22-6	Proanthocyanidins	3.3863
4852-22-6	Proanthocyanidins	3.3873
4852-22-6	Proanthocyanidins	3.3901
389139-89-3	Omadacycline	3.3914
151533-22-1	Levomefolate calcium	3.3916
155148-31-5	Plerixafor 8HCl (AMD3100 8HCl)	3.3952
110078-46-1	Plerixafor	3.3952
4852-22-6	Proanthocyanidins	3.3965
4852-22-6	Proanthocyanidins	3.3983
4852-22-6	Proanthocyanidins	3.3986
4852-22-6	Proanthocyanidins	3.3992
115939-25-8	Salvianolic acid B	3.4018
73816-42-9	Meclocycline sulfosalicylate salt	3.4031
138-14-7	Deferoxamine Mesylate	3.4094
4852-22-6	Proanthocyanidins	3.4134
4852-22-6	Proanthocyanidins	3.4149
17696-69-4	Hematoporphyrin dihydrochloride	3.4154
4852-22-6	Proanthocyanidins	3.4172
4852-22-6	Proanthocyanidins	3.4232
1207456-01-6	Talazoparib	3.4244
4852-22-6	Proanthocyanidins	3.43
4852-22-6	Proanthocyanidins	3.4368
1025687-58-4	R788 Disodium	3.4369
4852-22-6	Proanthocyanidins	3.4406
60925-61-3	Ceforanide	3.4406
100299-08-9	Pemirolast potassium	3.4436
4852-22-6	Proanthocyanidins	3.4449
4852-22-6	Proanthocyanidins	3.4463
67-43-6	Pentetic Acid	3.4477
4852-22-6	Proanthocyanidins	3.4521
115939-25-8	Salvianolic acid B	3.4533
4852-22-6	Proanthocyanidins	3.4538
117976-90-6	Rebeprazole sodium	3.4542
17696-69-4	Hematoporphyrin dihydrochloride	3.4602
4852-22-6	Proanthocyanidins	3.4622

4852-22-6	Proanthocyanidins	3.4631
4852-22-6	Proanthocyanidins	3.4686
4852-22-6	Proanthocyanidins	3.4714
4852-22-6	Proanthocyanidins	3.4756
4852-22-6	Proanthocyanidins	3.4783
4852-22-6	Proanthocyanidins	3.4804
4852-22-6	Proanthocyanidins	3.4839
4852-22-6	Proanthocyanidins	3.4851
4852-22-6	Proanthocyanidins	3.4895
4852-22-6	Proanthocyanidins	3.4943
4852-22-6	Proanthocyanidins	3.5023
1207456-01-6	Talazoparib	3.5049
4852-22-6	Proanthocyanidins	3.5108
4852-22-6	Proanthocyanidins	3.5187
4852-22-6	Proanthocyanidins	3.5192
4852-22-6	Proanthocyanidins	3.5227
17696-69-4	Hematoporphyrin dihydrochloride	3.5231
4852-22-6	Proanthocyanidins	3.5396
4852-22-6	Proanthocyanidins	3.54
4852-22-6	Proanthocyanidins	3.5408
4852-22-6	Proanthocyanidins	3.5434
137862-53-4	Valsartan	3.5456
4852-22-6	Proanthocyanidins	3.5482
4852-22-6	Proanthocyanidins	3.5497
4852-22-6	Proanthocyanidins	3.5501
4852-22-6	Proanthocyanidins	3.5502
4852-22-6	Proanthocyanidins	3.5506
4342-03-4	Dacarbazine	3.5515
4852-22-6	Proanthocyanidins	3.5549
3697-42-5	Chlorhexidine hydrochloride	3.5559
56-95-1	Chlorhexidine Acetate	3.5559
4852-22-6	Proanthocyanidins	3.5568
4852-22-6	Proanthocyanidins	3.5581
4852-22-6	Proanthocyanidins	3.5612
4852-22-6	Proanthocyanidins	3.5632
304-55-2	Succimer	3.569
4852-22-6	Proanthocyanidins	3.5702
4852-22-6	Proanthocyanidins	3.5712
73816-42-9	Meclocycline sulfosalicylate salt	3.5714
4852-22-6	Proanthocyanidins	3.5767
4852-22-6	Proanthocyanidins	3.5767
4852-22-6	Proanthocyanidins	3.5782

4852-22-6	Proanthocyanidins	3.5788
4852-22-6	Proanthocyanidins	3.5792
1025687-58-4	R788 Disodium	3.5818
4852-22-6	Proanthocyanidins	3.5834
151533-22-1	Levomefolate calcium	3.5877
364622-82-2	Doripenem Hydrate	3.5925
4852-22-6	Proanthocyanidins	3.5962
1197953-54-0	Brigatinib	3.5969
4852-22-6	Proanthocyanidins	3.6003
4852-22-6	Proanthocyanidins	3.6066
3697-42-5	Chlorhexidine hydrochloride	3.6069
56-95-1	Chlorhexidine Acetate	3.6069
4852-22-6	Proanthocyanidins	3.6093
4852-22-6	Proanthocyanidins	3.61
4852-22-6	Proanthocyanidins	3.61
4852-22-6	Proanthocyanidins	3.6104
4852-22-6	Proanthocyanidins	3.6105
4852-22-6	Proanthocyanidins	3.6155
4852-22-6	Proanthocyanidins	3.6157
220620-09-7	Tigecycline	3.6227
4852-22-6	Proanthocyanidins	3.6273
4852-22-6	Proanthocyanidins	3.6283
4852-22-6	Proanthocyanidins	3.6289
4852-22-6	Proanthocyanidins	3.6322
4852-22-6	Proanthocyanidins	3.6332
4852-22-6	Proanthocyanidins	3.6365
4852-22-6	Proanthocyanidins	3.6368
4852-22-6	Proanthocyanidins	3.6436
4852-22-6	Proanthocyanidins	3.6444
4852-22-6	Proanthocyanidins	3.6451
4852-22-6	Proanthocyanidins	3.6458
4852-22-6	Proanthocyanidins	3.646
4852-22-6	Proanthocyanidins	3.6518
4852-22-6	Proanthocyanidins	3.6617
4852-22-6	Proanthocyanidins	3.6667
4852-22-6	Proanthocyanidins	3.6675
4852-22-6	Proanthocyanidins	3.6687
4852-22-6	Proanthocyanidins	3.6688
13614-98-7	Minocycline hydrochloride	3.6719
4852-22-6	Proanthocyanidins	3.6755
151533-22-1	Levomefolate calcium	3.6782

4852-22-6	Proanthocyanidins	3.6789
151533-22-1	Levomefolate calcium	3.6798
4852-22-6	Proanthocyanidins	3.6819
1405-41-0	Gentamycin sulfate	3.6819
4852-22-6	Proanthocyanidins	3.6863
4852-22-6	Proanthocyanidins	3.6895
304-55-2	Succimer	3.6947
4852-22-6	Proanthocyanidins	3.6947
4852-22-6	Proanthocyanidins	3.6975
4852-22-6	Proanthocyanidins	3.6997
4852-22-6	Proanthocyanidins	3.7005
4852-22-6	Proanthocyanidins	3.7047
151533-22-1	Levomefolate calcium	3.7093
4852-22-6	Proanthocyanidins	3.7094
151533-22-1	Levomefolate calcium	3.7124
4852-22-6	Proanthocyanidins	3.7227
389139-89-3	Omadacycline	3.7259
4852-22-6	Proanthocyanidins	3.7294
4852-22-6	Proanthocyanidins	3.7322
4852-22-6	Proanthocyanidins	3.7338
1405-41-0	Gentamycin sulfate	3.736
4852-22-6	Proanthocyanidins	3.7396
4852-22-6	Proanthocyanidins	3.743
4852-22-6	Proanthocyanidins	3.7446
4852-22-6	Proanthocyanidins	3.7456
151533-22-1	Levomefolate calcium	3.7485
151533-22-1	Levomefolate calcium	3.7526
4852-22-6	Proanthocyanidins	3.7538
79-57-2	Oxytetracycline	3.7554
4852-22-6	Proanthocyanidins	3.7573
4852-22-6	Proanthocyanidins	3.7647
4852-22-6	Proanthocyanidins	3.7688
4852-22-6	Proanthocyanidins	3.771
4852-22-6	Proanthocyanidins	3.7724
121268-17-5	Alendronate sodium hydrate	3.7747
13614-98-7	Minocycline hydrochloride	3.7753
4852-22-6	Proanthocyanidins	3.7764
4852-22-6	Proanthocyanidins	3.7787
4852-22-6	Proanthocyanidins	3.7831
151533-22-1	Levomefolate calcium	3.7859
4852-22-6	Proanthocyanidins	3.7873
17696-69-4	Hematoporphyrin dihydrochloride	3.7895
4852-22-6	Proanthocyanidins	3.7904

117976-90-6	Rebeprazole sodium	3.7926
4852-22-6	Proanthocyanidins	3.7959
4852-22-6	Proanthocyanidins	3.7975
151533-22-1	Levomefolate calcium	3.7985
4852-22-6	Proanthocyanidins	3.8031
4852-22-6	Proanthocyanidins	3.8048
4852-22-6	Proanthocyanidins	3.8063
4852-22-6	Proanthocyanidins	3.809
4852-22-6	Proanthocyanidins	3.8116
4852-22-6	Proanthocyanidins	3.8132
151533-22-1	Levomefolate calcium	3.8158
151533-22-1	Levomefolate calcium	3.8178
4852-22-6	Proanthocyanidins	3.8187
4852-22-6	Proanthocyanidins	3.8225
4852-22-6	Proanthocyanidins	3.823
4852-22-6	Proanthocyanidins	3.8248
4852-22-6	Proanthocyanidins	3.825
304-55-2	Succimer	3.8258
138-14-7	Deferoxamine Mesylate	3.8282
151533-22-1	Levomefolate calcium	3.8347
4852-22-6	Proanthocyanidins	3.8373
4852-22-6	Proanthocyanidins	3.8444
4852-22-6	Proanthocyanidins	3.8445
4852-22-6	Proanthocyanidins	3.8483
56-84-8	L-Aspartic acid	3.8592
4852-22-6	Proanthocyanidins	3.8628
64-75-5	Tetracycline hydrochloride	3.87
4852-22-6	Proanthocyanidins	3.8713
151533-22-1	Levomefolate calcium	3.8713
4852-22-6	Proanthocyanidins	3.8742
4852-22-6	Proanthocyanidins	3.8757
1070-11-7	Ethambutol dihydrochloride	3.8761
151533-22-1	Levomefolate calcium	3.879
4852-22-6	Proanthocyanidins	3.8793
4852-22-6	Proanthocyanidins	3.8802
4852-22-6	Proanthocyanidins	3.8813
151533-22-1	Levomefolate calcium	3.886
4852-22-6	Proanthocyanidins	3.8945
4852-22-6	Proanthocyanidins	3.8955
4852-22-6	Proanthocyanidins	3.8989
4852-22-6	Proanthocyanidins	3.8989
1981-58-4	Sulfamethazine sodium salt	3.9079
7280-37-7	Estropipate	3.9111

4852-22-6	Proanthocyanidins	3.9137
14882-18-9	Bismuth subsalicylat	3.9139
4852-22-6	Proanthocyanidins	3.9148
4852-22-6	Proanthocyanidins	3.9155
4852-22-6	Proanthocyanidins	3.9169
4852-22-6	Proanthocyanidins	3.9169
4852-22-6	Proanthocyanidins	3.918
4852-22-6	Proanthocyanidins	3.9187
4852-22-6	Proanthocyanidins	3.9198
151533-22-1	Levomefolate calcium	3.9214
4852-22-6	Proanthocyanidins	3.9218
151533-22-1	Levomefolate calcium	3.9254
4852-22-6	Proanthocyanidins	3.9257
1041434-82-5	Peramivir Trihydrate	3.9354
4852-22-6	Proanthocyanidins	3.936
4852-22-6	Proanthocyanidins	3.9363
151533-22-1	Levomefolate calcium	3.937
4852-22-6	Proanthocyanidins	3.9388
4852-22-6	Proanthocyanidins	3.9411
1025687-58-4	R788 Disodium	3.9422
4852-22-6	Proanthocyanidins	3.943
4852-22-6	Proanthocyanidins	3.9457
4852-22-6	Proanthocyanidins	3.9469
4852-22-6	Proanthocyanidins	3.9539
4852-22-6	Proanthocyanidins	3.9614
4852-22-6	Proanthocyanidins	3.9647
4852-22-6	Proanthocyanidins	3.9674
4852-22-6	Proanthocyanidins	3.9698
860-79-7	Cyclovirobuxin D	3.9722
4852-22-6	Proanthocyanidins	3.9727
4852-22-6	Proanthocyanidins	3.9753
4852-22-6	Proanthocyanidins	3.9757
4852-22-6	Proanthocyanidins	3.9762
4852-22-6	Proanthocyanidins	3.9799
104376-79-6	Ceftriaxone sodium	3.9805
4852-22-6	Proanthocyanidins	3.9817
4852-22-6	Proanthocyanidins	3.9828
4852-22-6	Proanthocyanidins	3.9831
4852-22-6	Proanthocyanidins	3.9831
4852-22-6	Proanthocyanidins	3.9888
4852-22-6	Proanthocyanidins	3.996

4852-22-6	Proanthocyanidins	3.9985
4852-22-6	Proanthocyanidins	3.9987
4852-22-6	Proanthocyanidins	4.0011
4852-22-6	Proanthocyanidins	4.0032
4852-22-6	Proanthocyanidins	4.0037
4852-22-6	Proanthocyanidins	4.0067
4852-22-6	Proanthocyanidins	4.0145
4852-22-6	Proanthocyanidins	4.0175
151533-22-1	Levomefolate calcium	4.02
4852-22-6	Proanthocyanidins	4.0201
4852-22-6	Proanthocyanidins	4.0273
151533-22-1	Levomefolate calcium	4.0296
220620-09-7	Tigecycline	4.0297
4852-22-6	Proanthocyanidins	4.0333
1981-58-4	Sulfamethazine sodium salt	4.0334
4852-22-6	Proanthocyanidins	4.0337
4852-22-6	Proanthocyanidins	4.0355
4852-22-6	Proanthocyanidins	4.036
4852-22-6	Proanthocyanidins	4.0371
151533-22-1	Levomefolate calcium	4.0414
4852-22-6	Proanthocyanidins	4.054
4852-22-6	Proanthocyanidins	4.0544
4852-22-6	Proanthocyanidins	4.0567
4852-22-6	Proanthocyanidins	4.0573
138-14-7	Deferoxamine Mesylate	4.06
4852-22-6	Proanthocyanidins	4.0606
4852-22-6	Proanthocyanidins	4.061
7280-37-7	Estropipate	4.0615
121268-17-5	Alendronate sodium hydrate	4.0748
4852-22-6	Proanthocyanidins	4.0751
4852-22-6	Proanthocyanidins	4.076
901119-35-5	Fostamatinib	4.0849
4852-22-6	Proanthocyanidins	4.0851
138-14-7	Deferoxamine Mesylate	4.0954
13614-98-7	Minocycline hydrochloride	4.098
4852-22-6	Proanthocyanidins	4.0986
1041434-82-5	Peramivir Trihydrate	4.1009
1070-11-7	Ethambutol dihydrochloride	4.1014
151533-22-1	Levomefolate calcium	4.1058
4852-22-6	Proanthocyanidins	4.1059
4852-22-6	Proanthocyanidins	4.1111
4852-22-6	Proanthocyanidins	4.119

56-84-8	L-Aspartic acid	4.1193
389139-89-3	Omadacycline	4.1212
4852-22-6	Proanthocyanidins	4.122
4852-22-6	Proanthocyanidins	4.1236
4852-22-6	Proanthocyanidins	4.1257
4852-22-6	Proanthocyanidins	4.1262
4852-22-6	Proanthocyanidins	4.129
4852-22-6	Proanthocyanidins	4.1292
4852-22-6	Proanthocyanidins	4.1298
4852-22-6	Proanthocyanidins	4.136
4852-22-6	Proanthocyanidins	4.1365
4852-22-6	Proanthocyanidins	4.1376
4852-22-6	Proanthocyanidins	4.1404
1405-86-3	Glycyrrhizin	4.1585
79165-06-3	Diammonium Glycyrrhizinate	4.1585
138-14-7	Deferoxamine Mesylate	4.1586
14882-18-9	Bismuth subsalicylat	4.1611
4852-22-6	Proanthocyanidins	4.1625
4852-22-6	Proanthocyanidins	4.1648
4852-22-6	Proanthocyanidins	4.1655
4852-22-6	Proanthocyanidins	4.167
4852-22-6	Proanthocyanidins	4.1671
4852-22-6	Proanthocyanidins	4.1681
4852-22-6	Proanthocyanidins	4.1692
4852-22-6	Proanthocyanidins	4.1702
4852-22-6	Proanthocyanidins	4.1717
4852-22-6	Proanthocyanidins	4.1729
151533-22-1	Levomefolate calcium	4.1818
4852-22-6	Proanthocyanidins	4.1837
1197953-54-0	Brigatinib	4.1855
4852-22-6	Proanthocyanidins	4.1882
4852-22-6	Proanthocyanidins	4.1908
4852-22-6	Proanthocyanidins	4.1934
4852-22-6	Proanthocyanidins	4.196
4852-22-6	Proanthocyanidins	4.1997
4852-22-6	Proanthocyanidins	4.2033
4852-22-6	Proanthocyanidins	4.2038
4852-22-6	Proanthocyanidins	4.208
4852-22-6	Proanthocyanidins	4.2136
4852-22-6	Proanthocyanidins	4.2155
4852-22-6	Proanthocyanidins	4.2157
220620-09-7	Tigecycline	4.2226

4852-22-6	Proanthocyanidins	4.2249
1070-11-7	Ethambutol dihydrochloride	4.2272
4852-22-6	Proanthocyanidins	4.2301
4852-22-6	Proanthocyanidins	4.2302
4852-22-6	Proanthocyanidins	4.2317
4852-22-6	Proanthocyanidins	4.2323
4852-22-6	Proanthocyanidins	4.2326
4852-22-6	Proanthocyanidins	4.2366
151533-22-1	Levomefolate calcium	4.2389
4852-22-6	Proanthocyanidins	4.2393
4852-22-6	Proanthocyanidins	4.2417
4852-22-6	Proanthocyanidins	4.2429
4852-22-6	Proanthocyanidins	4.2459
121268-17-5	Alendronate sodium hydrate	4.2465
151533-22-1	Levomefolate calcium	4.2503
4852-22-6	Proanthocyanidins	4.2519
4852-22-6	Proanthocyanidins	4.255
4852-22-6	Proanthocyanidins	4.2567
4852-22-6	Proanthocyanidins	4.257
7280-37-7	Estropipate	4.2604
151533-22-1	Levomefolate calcium	4.2639
4852-22-6	Proanthocyanidins	4.2735
4852-22-6	Proanthocyanidins	4.2738
4852-22-6	Proanthocyanidins	4.2808
151533-22-1	Levomefolate calcium	4.2818
4852-22-6	Proanthocyanidins	4.2835
73816-42-9	Meclocycline sulfosalicylate salt	4.2856
121268-17-5	Alendronate sodium hydrate	4.2859
4852-22-6	Proanthocyanidins	4.291
4852-22-6	Proanthocyanidins	4.3004
4852-22-6	Proanthocyanidins	4.3026
4852-22-6	Proanthocyanidins	4.307
73816-42-9	Meclocycline sulfosalicylate salt	4.3115
4852-22-6	Proanthocyanidins	4.3124
4852-22-6	Proanthocyanidins	4.3173
4852-22-6	Proanthocyanidins	4.3214
4852-22-6	Proanthocyanidins	4.3242
4852-22-6	Proanthocyanidins	4.3252
4852-22-6	Proanthocyanidins	4.3347
151533-22-1	Levomefolate calcium	4.3405
4852-22-6	Proanthocyanidins	4.3409
4852-22-6	Proanthocyanidins	4.346
4852-22-6	Proanthocyanidins	4.3529

4852-22-6	Proanthocyanidins	4.3661
4852-22-6	Proanthocyanidins	4.3667
4852-22-6	Proanthocyanidins	4.3688
56-84-8	L-Aspartic acid	4.3709
4852-22-6	Proanthocyanidins	4.3712
4852-22-6	Proanthocyanidins	4.3744
55-56-1	Chlorhexidine	4.3748
4852-22-6	Proanthocyanidins	4.376
4852-22-6	Proanthocyanidins	4.3779
151533-22-1	Levomefolate calcium	4.379
121268-17-5	Alendronate sodium hydrate	4.3797
4852-22-6	Proanthocyanidins	4.3817
4852-22-6	Proanthocyanidins	4.3839
4852-22-6	Proanthocyanidins	4.3845
4852-22-6	Proanthocyanidins	4.3904
3697-42-5	Chlorhexidine hydrochloride	4.3913
56-95-1	Chlorhexidine Acetate	4.3913
121268-17-5	Alendronate sodium hydrate	4.3935
4852-22-6	Proanthocyanidins	4.3973
4852-22-6	Proanthocyanidins	4.4079
4852-22-6	Proanthocyanidins	4.4112
69712-56-7	Cefotetan	4.4141
4852-22-6	Proanthocyanidins	4.4144
4852-22-6	Proanthocyanidins	4.4166
1405-37-4	Capastat sulfate	4.4193
4852-22-6	Proanthocyanidins	4.4212
220620-09-7	Tigecycline	4.4242
4852-22-6	Proanthocyanidins	4.4245
1981-58-4	Sulfamethazine sodium salt	4.4306
4852-22-6	Proanthocyanidins	4.4356
4852-22-6	Proanthocyanidins	4.4359
4852-22-6	Proanthocyanidins	4.4401
4852-22-6	Proanthocyanidins	4.441
4852-22-6	Proanthocyanidins	4.4455
4852-22-6	Proanthocyanidins	4.4463
151533-22-1	Levomefolate calcium	4.4516
151533-22-1	Levomefolate calcium	4.4527
4852-22-6	Proanthocyanidins	4.4552
151533-22-1	Levomefolate calcium	4.4595
29908-03-0	Ademetionine	4.4658
4852-22-6	Proanthocyanidins	4.4724
4852-22-6	Proanthocyanidins	4.4736
4852-22-6	Proanthocyanidins	4.4784

4852-22-6	Proanthocyanidins	4.4889
4852-22-6	Proanthocyanidins	4.4907
73816-42-9	Meclocycline sulfosalicylate salt	4.4922
4852-22-6	Proanthocyanidins	4.4944
4852-22-6	Proanthocyanidins	4.4968
4852-22-6	Proanthocyanidins	4.4981
4852-22-6	Proanthocyanidins	4.499
1197953-54-0	Brigatinib	4.5002
4852-22-6	Proanthocyanidins	4.5002
4852-22-6	Proanthocyanidins	4.5004
4852-22-6	Proanthocyanidins	4.5018
4852-22-6	Proanthocyanidins	4.5076
151533-22-1	Levomefolate calcium	4.5185
4852-22-6	Proanthocyanidins	4.5197
151533-22-1	Levomefolate calcium	4.5197
151533-22-1	Levomefolate calcium	4.5228
151533-22-1	Levomefolate calcium	4.5232
4852-22-6	Proanthocyanidins	4.5235
4852-22-6	Proanthocyanidins	4.5318
4852-22-6	Proanthocyanidins	4.5332
4852-22-6	Proanthocyanidins	4.5333
4852-22-6	Proanthocyanidins	4.5366
4852-22-6	Proanthocyanidins	4.5401
151533-22-1	Levomefolate calcium	4.5425
4852-22-6	Proanthocyanidins	4.5476
55-56-1	Chlorhexidine	4.5505
56-86-0	glutamic acid	4.5587
4852-22-6	Proanthocyanidins	4.5661
151533-22-1	Levomefolate calcium	4.5717
4852-22-6	Proanthocyanidins	4.5791
4852-22-6	Proanthocyanidins	4.5818
4852-22-6	Proanthocyanidins	4.5896
4852-22-6	Proanthocyanidins	4.5964
1197953-54-0	Brigatinib	4.5969
4852-22-6	Proanthocyanidins	4.5975
4852-22-6	Proanthocyanidins	4.598
4852-22-6	Proanthocyanidins	4.5986
4852-22-6	Proanthocyanidins	4.6016
4852-22-6	Proanthocyanidins	4.6086
4852-22-6	Proanthocyanidins	4.6092
4852-22-6	Proanthocyanidins	4.6178

4852-22-6	Proanthocyanidins	4.6208
4852-22-6	Proanthocyanidins	4.6263
4852-22-6	Proanthocyanidins	4.6277
4852-22-6	Proanthocyanidins	4.6287
4852-22-6	Proanthocyanidins	4.6352
4852-22-6	Proanthocyanidins	4.6434
4852-22-6	Proanthocyanidins	4.6446
4852-22-6	Proanthocyanidins	4.6462
4852-22-6	Proanthocyanidins	4.6468
4852-22-6	Proanthocyanidins	4.647
4350-09-8	L-5-Hydroxytryptophan	4.6517
4852-22-6	Proanthocyanidins	4.6545
4852-22-6	Proanthocyanidins	4.6548
4852-22-6	Proanthocyanidins	4.6591
4852-22-6	Proanthocyanidins	4.664
4852-22-6	Proanthocyanidins	4.6654
4852-22-6	Proanthocyanidins	4.6661
4350-09-8	L-5-Hydroxytryptophan	4.6663
138-14-7	Deferoxamine Mesylate	4.6689
151533-22-1	Levomefolate calcium	4.6703
4852-22-6	Proanthocyanidins	4.6741
4852-22-6	Proanthocyanidins	4.6769
4852-22-6	Proanthocyanidins	4.677
4852-22-6	Proanthocyanidins	4.6802
4852-22-6	Proanthocyanidins	4.6819
4852-22-6	Proanthocyanidins	4.6877
4852-22-6	Proanthocyanidins	4.6958
4852-22-6	Proanthocyanidins	4.6967
151533-22-1	Levomefolate calcium	4.6976
4852-22-6	Proanthocyanidins	4.7071
69712-56-7	Cefotetan	4.7168
4852-22-6	Proanthocyanidins	4.7186
4852-22-6	Proanthocyanidins	4.7202
4852-22-6	Proanthocyanidins	4.7302
4852-22-6	Proanthocyanidins	4.7311
3697-42-5	Chlorhexidine hydrochloride	4.7331
56-95-1	Chlorhexidine Acetate	4.7331
4852-22-6	Proanthocyanidins	4.7344
151533-22-1	Levomefolate calcium	4.7348
4852-22-6	Proanthocyanidins	4.7374
4852-22-6	Proanthocyanidins	4.741
4852-22-6	Proanthocyanidins	4.7431
55-56-1	Chlorhexidine	4.7484

4852-22-6	Proanthocyanidins	4.7519
4852-22-6	Proanthocyanidins	4.7658
70-26-8	L-Ornithine	4.7771
4852-22-6	Proanthocyanidins	4.7825
4852-22-6	Proanthocyanidins	4.7857
29908-03-0	Ademetionine	4.7864
4852-22-6	Proanthocyanidins	4.7887
4852-22-6	Proanthocyanidins	4.7888
4852-22-6	Proanthocyanidins	4.7929
4852-22-6	Proanthocyanidins	4.7935
4852-22-6	Proanthocyanidins	4.8039
4852-22-6	Proanthocyanidins	4.8042
4852-22-6	Proanthocyanidins	4.8058
29908-03-0	Ademetionine	4.8083
4852-22-6	Proanthocyanidins	4.8091
4852-22-6	Proanthocyanidins	4.8097
29908-03-0	Ademetionine	4.8188
4852-22-6	Proanthocyanidins	4.8249
4852-22-6	Proanthocyanidins	4.8287
4852-22-6	Proanthocyanidins	4.8287
3697-42-5	Chlorhexidine hydrochloride	4.8297
56-95-1	Chlorhexidine Acetate	4.8297
4852-22-6	Proanthocyanidins	4.8327
4852-22-6	Proanthocyanidins	4.8387
4852-22-6	Proanthocyanidins	4.8404
4852-22-6	Proanthocyanidins	4.8408
4852-22-6	Proanthocyanidins	4.8411
70-26-8	L-Ornithine	4.8414
4852-22-6	Proanthocyanidins	4.8514
38304-91-5	Minoxidil	4.8524
4852-22-6	Proanthocyanidins	4.8555
4852-22-6	Proanthocyanidins	4.8557
4350-09-8	L-5-Hydroxytryptophan	4.8727
4852-22-6	Proanthocyanidins	4.8808
55-56-1	Chlorhexidine	4.8826
4852-22-6	Proanthocyanidins	4.8828
56-86-0	glutamic acid	4.8848
70-26-8	L-Ornithine	4.887
4852-22-6	Proanthocyanidins	4.8894
29342-05-0	Ciclopirox	4.8921
41621-49-2	Ciclopirox ethanolamine	4.8921
4852-22-6	Proanthocyanidins	4.8961
1405-86-3	Glycyrrhizin	4.8972

79165-06-3	Diammonium Glycyrrhizinate	4.8972
151533-22-1	Levomefolate calcium	4.9035
4852-22-6	Proanthocyanidins	4.9128
29908-03-0	Ademetionine	4.9143
29908-03-0	Ademetionine	4.9154
4852-22-6	Proanthocyanidins	4.916
4852-22-6	Proanthocyanidins	4.9249
4852-22-6	Proanthocyanidins	4.9281
56-86-0	glutamic acid	4.9325
4852-22-6	Proanthocyanidins	4.938
4852-22-6	Proanthocyanidins	4.9453
4852-22-6	Proanthocyanidins	4.9472
4852-22-6	Proanthocyanidins	4.9472
4852-22-6	Proanthocyanidins	4.9489
4852-22-6	Proanthocyanidins	4.959
4852-22-6	Proanthocyanidins	4.9599
4852-22-6	Proanthocyanidins	4.9648
4852-22-6	Proanthocyanidins	4.9694
4852-22-6	Proanthocyanidins	4.9759
6064-83-1	Fosfosal	4.9761
4852-22-6	Proanthocyanidins	4.9779
29908-03-0	Ademetionine	4.9801
4852-22-6	Proanthocyanidins	4.9801
4852-22-6	Proanthocyanidins	4.981
6064-83-1	Fosfosal	4.9818
4852-22-6	Proanthocyanidins	4.9836
4852-22-6	Proanthocyanidins	4.9857
4852-22-6	Proanthocyanidins	4.9929
105462-24-6	Risedronic Acid	4.9968
6064-83-1	Fosfosal	5.0097
4852-22-6	Proanthocyanidins	5.0108
4852-22-6	Proanthocyanidins	5.0143
1405-86-3	Glycyrrhizin	5.0155
79165-06-3	Diammonium Glycyrrhizinate	5.0155
4852-22-6	Proanthocyanidins	5.0276
1197953-54-0	Brigatinib	5.0291
4852-22-6	Proanthocyanidins	5.0297
4852-22-6	Proanthocyanidins	5.0317
4852-22-6	Proanthocyanidins	5.0345
150399-23-8	Pemetrexed disodium	5.0409
357166-30-4	Pemetrexed Disodium Hydrate	5.0412
105462-24-6	Risedronic Acid	5.05

4852-22-6	Proanthocyanidins	5.051
29342-05-0	Ciclopirox	5.0569
41621-49-2	Ciclopirox ethanolamine	5.0569
4852-22-6	Proanthocyanidins	5.063
77-92-9	Citric acid	5.0676
550-24-3	Embelin	5.0711
357166-30-4	Pemetrexed Disodium Hydrate	5.0862
4852-22-6	Proanthocyanidins	5.0862
4852-22-6	Proanthocyanidins	5.0878
150399-23-8	Pemetrexed disodium	5.088
4852-22-6	Proanthocyanidins	5.0884
4350-09-8	L-5-Hydroxytryptophan	5.0968
550-24-3	Embelin	5.0975
4852-22-6	Proanthocyanidins	5.1168
1405-37-4	Capastat sulfate	5.1245
29342-05-0	Ciclopirox	5.1293
41621-49-2	Ciclopirox ethanolamine	5.1293
29908-03-0	Ademetionine	5.1306
1115-70-4	Metformin hydrochloride	5.132
67-43-6	Pentetic Acid	5.1379
104376-79-6	Ceftriaxone sodium	5.1448
4852-22-6	Proanthocyanidins	5.1514
4852-22-6	Proanthocyanidins	5.1527
29908-03-0	Ademetionine	5.1532
1405-37-4	Capastat sulfate	5.1576
151533-22-1	Levomefolate calcium	5.1576
4350-09-8	L-5-Hydroxytryptophan	5.1582
4852-22-6	Proanthocyanidins	5.1786
29908-03-0	Ademetionine	5.183
17696-69-4	Hematoporphyrin dihydrochloride	5.1873
550-24-3	Embelin	5.2116
4852-22-6	Proanthocyanidins	5.2221
4852-22-6	Proanthocyanidins	5.2226
29908-03-0	Ademetionine	5.2249
17696-69-4	Hematoporphyrin dihydrochloride	5.2252
29908-03-0	Ademetionine	5.2256
4852-22-6	Proanthocyanidins	5.235
4852-22-6	Proanthocyanidins	5.2527
29908-03-0	Ademetionine	5.2538
29908-03-0	Ademetionine	5.2556
38304-91-5	Minoxidil	5.2589
4852-22-6	Proanthocyanidins	5.2605
151533-22-1	Levomefolate calcium	5.2746

4852-22-6	Proanthocyanidins	5.2781
4852-22-6	Proanthocyanidins	5.2816
315-30-0	Allopurinol	5.2924
22199-08-2	Silver sulfadiazine	5.2931
105462-24-6	Risedronic Acid	5.3064
357166-30-4	Pemetrexed Disodium Hydrate	5.3087
150399-23-8	Pemetrexed disodium	5.309
29908-03-0	Ademetionine	5.3105
4852-22-6	Proanthocyanidins	5.3115
4852-22-6	Proanthocyanidins	5.3256
22199-08-2	Silver sulfadiazine	5.3268
29908-03-0	Ademetionine	5.3336
4852-22-6	Proanthocyanidins	5.3411
1115-70-4	Metformin hydrochloride	5.3449
550-24-3	Embelin	5.3516
29908-03-0	Ademetionine	5.3609
315-30-0	Allopurinol	5.3626
901119-35-5	Fostamatinib	5.3777
550-24-3	Embelin	5.3844
29908-03-0	Ademetionine	5.3917
4852-22-6	Proanthocyanidins	5.4006
4852-22-6	Proanthocyanidins	5.4177
29908-03-0	Ademetionine	5.4179
4350-09-8	L-5-Hydroxytryptophan	5.4286
4852-22-6	Proanthocyanidins	5.44
4852-22-6	Proanthocyanidins	5.4438
364622-82-2	Doripenem Hydrate	5.444
4852-22-6	Proanthocyanidins	5.4446
22199-08-2	Silver sulfadiazine	5.4463
29908-03-0	Ademetionine	5.4597
4852-22-6	Proanthocyanidins	5.4692
77-92-9	Citric acid	5.4702
404950-80-7	Panobinostat	5.4953
404950-80-7	Panobinostat	5.4957
4852-22-6	Proanthocyanidins	5.4961
56-87-1	lysine	5.5004
61379-65-5	Rifapentine	5.5029
901119-35-5	Fostamatinib	5.5152
550-24-3	Embelin	5.5155
550-24-3	Embelin	5.5469
69712-56-7	Cefotetan	5.5475
4852-22-6	Proanthocyanidins	5.5508
82009-34-5	Cilastatin	5.5597

4852-22-6	Proanthocyanidins	5.566
4852-22-6	Proanthocyanidins	5.5794
4852-22-6	Proanthocyanidins	5.5801
4852-22-6	Proanthocyanidins	5.5871
77-92-9	Citric acid	5.589
404950-80-7	Panobinostat	5.5906
56-87-1	lysine	5.5952
29908-03-0	Ademetionine	5.6085
4852-22-6	Proanthocyanidins	5.613
404950-80-7	Panobinostat	5.6148
4852-22-6	Proanthocyanidins	5.6153
29908-03-0	Ademetionine	5.6155
3963-95-9	Methacycline hydrochloride	5.6328
4852-22-6	Proanthocyanidins	5.6438
17696-69-4	Hematoporphyrin dihydrochloride	5.6469
17696-69-4	Hematoporphyrin dihydrochloride	5.6491
3160-91-6	Moroxydine hydrochloride	5.655
29908-03-0	Ademetionine	5.6571
4852-22-6	Proanthocyanidins	5.6591
404950-80-7	Panobinostat	5.6636
404950-80-7	Panobinostat	5.6666
550-24-3	Embelin	5.6707
29908-03-0	Ademetionine	5.6785
29908-03-0	Ademetionine	5.6819
29908-03-0	Ademetionine	5.688
17696-69-4	Hematoporphyrin dihydrochloride	5.6888
38304-91-5	Minoxidil	5.7061
4852-22-6	Proanthocyanidins	5.7252
19408-46-9	Kasugamycin hydrochloride	5.731
404950-80-7	Panobinostat	5.7331
364622-82-2	Doripenem Hydrate	5.7394
138-14-7	Deferoxamine Mesylate	5.7436
315-30-0	Allopurinol	5.7493
29908-03-0	Ademetionine	5.7559
4852-22-6	Proanthocyanidins	5.7688
29908-03-0	Ademetionine	5.7849
1115-70-4	Metformin hydrochloride	5.7981
29908-03-0	Ademetionine	5.7983
4852-22-6	Proanthocyanidins	5.8045
1405-37-4	Capastat sulfate	5.8072
29908-03-0	Ademetionine	5.8084
56-89-3	cystine	5.8092
29908-03-0	Ademetionine	5.8229

901119-35-5	Fostamatinib	5.8357
364622-82-2	Doripenem Hydrate	5.8528
550-24-3	Embelin	5.8589
3963-95-9	Methacycline hydrochloride	5.864
1197953-54-0	Brigatinib	5.874
29908-03-0	Ademetionine	5.8772
4852-22-6	Proanthocyanidins	5.8819
118072-93-8	Zoledronic Acid	5.8841
3160-91-6	Moroxydine hydrochloride	5.8962
67-43-6	Pentetic Acid	5.9035
173352-39-1	Afobazole hydrochloride	5.9216
1197953-54-0	Brigatinib	5.9317
3697-42-5	Chlorhexidine hydrochloride	5.933
56-95-1	Chlorhexidine Acetate	5.933
29908-03-0	Ademetionine	5.9378
901119-35-5	Fostamatinib	5.9441
118072-93-8	Zoledronic Acid	5.9523
29908-03-0	Ademetionine	5.9534
138-14-7	Deferoxamine Mesylate	5.9568
56-87-1	lysine	5.9611
19408-46-9	Kasugamycin hydrochloride	5.9751
29908-03-0	Ademetionine	5.9801
129497-78-5	Verteporfin	5.9822
698387-09-6	Neratinib	5.9863
698387-09-6	Neratinib	5.9898
2809-21-4	Etidronate	6.0015
17696-69-4	Hematoporphyrin dihydrochloride	6.0037
404950-80-7	Panobinostat	6.004
29908-03-0	Ademetionine	6.009
4852-22-6	Proanthocyanidins	6.0177
29908-03-0	Ademetionine	6.0197
29908-03-0	Ademetionine	6.0299
4852-22-6	Proanthocyanidins	6.0329
107-35-7	Taurine	6.0489
29908-03-0	Ademetionine	6.0512
4350-09-8	L-5-Hydroxytryptophan	6.0522
29908-03-0	Ademetionine	6.0523
17696-69-4	Hematoporphyrin dihydrochloride	6.0714
1405-37-4	Capastat sulfate	6.0761
17696-69-4	Hematoporphyrin dihydrochloride	6.08
404950-80-7	Panobinostat	6.1114

4852-22-6	Proanthocyanidins	6.1283
1254053-43-4	Gilteritinib	6.1363
69558-55-0	Thymopentin	6.1446
56-89-3	cystine	6.1552
698387-09-6	Neratinib	6.1667
56-89-3	cystine	6.1674
17696-69-4	Hematoporphyrin dihydrochloride	6.169
1405-37-4	Capastat sulfate	6.1705
55-56-1	Chlorhexidine	6.1806
59-30-3	Folic acid	6.1927
29908-03-0	Ademetionine	6.2027
17696-69-4	Hematoporphyrin dihydrochloride	6.2097
107-35-7	Taurine	6.211
29908-03-0	Ademetionine	6.211
404950-80-7	Panobinostat	6.2166
17696-69-4	Hematoporphyrin dihydrochloride	6.2175
59-30-3	Folic acid	6.2179
3963-95-9	Methacycline hydrochloride	6.2367
4350-09-8	L-5-Hydroxytryptophan	6.2446
1254053-43-4	Gilteritinib	6.2497
29908-03-0	Ademetionine	6.2692
29908-03-0	Ademetionine	6.2943
2809-21-4	Etidronate	6.2997
29908-03-0	Ademetionine	6.3009
129497-78-5	Verteporfin	6.3053
2809-21-4	Etidronate	6.3164
138-14-7	Deferoxamine Mesylate	6.3398
404950-80-7	Panobinostat	6.3466
17696-69-4	Hematoporphyrin dihydrochloride	6.3532
17696-69-4	Hematoporphyrin dihydrochloride	6.3567
3160-91-6	Moroxydine hydrochloride	6.3872
29908-03-0	Ademetionine	6.395
17696-69-4	Hematoporphyrin dihydrochloride	6.3987
17696-69-4	Hematoporphyrin dihydrochloride	6.409
17696-69-4	Hematoporphyrin dihydrochloride	6.4095
67-43-6	Pentetic Acid	6.4146
17696-69-4	Hematoporphyrin dihydrochloride	6.4164
129497-78-5	Verteporfin	6.4222
1254053-43-4	Gilteritinib	6.435
118072-93-8	Zoledronic Acid	6.4452

17696-69-4	Hematoporphyrin dihydrochloride	6.4496
17696-69-4	Hematoporphyrin dihydrochloride	6.4701
173352-39-1	Afobazole hydrochloride	6.4749
901119-35-5	Fostamatinib	6.4802
59-30-3	Folic acid	6.482
107-35-7	Taurine	6.5008
80621-81-4	Rifaximin	6.5039
29908-03-0	Ademetionine	6.5051
17696-69-4	Hematoporphyrin dihydrochloride	6.5352
4852-22-6	Proanthocyanidins	6.5412
67-43-6	Pentetic Acid	6.5419
4852-22-6	Proanthocyanidins	6.5425
4350-09-8	L-5-Hydroxytryptophan	6.5487
17696-69-4	Hematoporphyrin dihydrochloride	6.5676
404950-80-7	Panobinostat	6.5764
389139-89-3	Omadacycline	6.5834
80621-81-4	Rifaximin	6.5856
17696-69-4	Hematoporphyrin dihydrochloride	6.5908
4852-22-6	Proanthocyanidins	6.6009
115939-25-8	Salvianolic acid B	6.6009
69558-55-0	Thymopentin	6.624
17696-69-4	Hematoporphyrin dihydrochloride	6.6374
4852-22-6	Proanthocyanidins	6.6388
173352-39-1	Afobazole hydrochloride	6.6391
170729-80-3	Aprepitant	6.6672
17696-69-4	Hematoporphyrin dihydrochloride	6.6722
67-43-6	Pentetic Acid	6.6778
4852-22-6	Proanthocyanidins	6.6794
4852-22-6	Proanthocyanidins	6.7123
3697-42-5	Chlorhexidine hydrochloride	6.7262
56-95-1	Chlorhexidine Acetate	6.7262
4852-22-6	Proanthocyanidins	6.7322
29908-03-0	Ademetionine	6.7424
17696-69-4	Hematoporphyrin dihydrochloride	6.757
17696-69-4	Hematoporphyrin dihydrochloride	6.7611
69558-55-0	Thymopentin	6.7659
4852-22-6	Proanthocyanidins	6.7699
4852-22-6	Proanthocyanidins	6.7711
4852-22-6	Proanthocyanidins	6.7713
115939-25-8	Salvianolic acid B	6.7735
17696-69-4	Hematoporphyrin dihydrochloride	6.7816
17696-69-4	Hematoporphyrin dihydrochloride	6.7839
170729-80-3	Aprepitant	6.8126

67-43-6	Pentetic Acid	6.8276
4852-22-6	Proanthocyanidins	6.8279
4350-09-8	L-5-Hydroxytryptophan	6.8289
115939-25-8	Salvianolic acid B	6.8324
3963-95-9	Methacycline hydrochloride	6.8532
4852-22-6	Proanthocyanidins	6.8598
4852-22-6	Proanthocyanidins	6.8607
4350-09-8	L-5-Hydroxytryptophan	6.875
4350-09-8	L-5-Hydroxytryptophan	6.8766
4852-22-6	Proanthocyanidins	6.8921
1405-37-4	Capastat sulfate	6.9096
17696-69-4	Hematoporphyrin dihydrochloride	6.9177
115939-25-8	Salvianolic acid B	6.9187
755038-65-4	Volasertib	6.9203
4852-22-6	Proanthocyanidins	6.9244
3963-95-9	Methacycline hydrochloride	6.9246
4852-22-6	Proanthocyanidins	6.9423
17696-69-4	Hematoporphyrin dihydrochloride	6.9463
115939-25-8	Salvianolic acid B	6.9496
4852-22-6	Proanthocyanidins	6.9802
3963-95-9	Methacycline hydrochloride	6.983
55-56-1	Chlorhexidine	6.9835
4852-22-6	Proanthocyanidins	7.0152
4852-22-6	Proanthocyanidins	7.0169
4852-22-6	Proanthocyanidins	7.0188
170729-80-3	Aprepitant	7.0267
755038-65-4	Volasertib	7.0332
4852-22-6	Proanthocyanidins	7.0384
17696-69-4	Hematoporphyrin dihydrochloride	7.0465
4852-22-6	Proanthocyanidins	7.0482
4852-22-6	Proanthocyanidins	7.0597
4852-22-6	Proanthocyanidins	7.0619
4852-22-6	Proanthocyanidins	7.0848
1405-37-4	Capastat sulfate	7.0875
4852-22-6	Proanthocyanidins	7.0918
4852-22-6	Proanthocyanidins	7.0937
4852-22-6	Proanthocyanidins	7.0995
755038-65-4	Volasertib	7.1003
4852-22-6	Proanthocyanidins	7.1032
80621-81-4	Rifaximin	7.1215
4852-22-6	Proanthocyanidins	7.1633
389139-89-3	Omadacycline	7.1668
4852-22-6	Proanthocyanidins	7.1676

4852-22-6	Proanthocyanidins	7.1799
4852-22-6	Proanthocyanidins	7.1924
4852-22-6	Proanthocyanidins	7.2038
17696-69-4	Hematoporphyrin dihydrochloride	7.2304
17696-69-4	Hematoporphyrin dihydrochloride	7.2337
4852-22-6	Proanthocyanidins	7.2357
4852-22-6	Proanthocyanidins	7.2366
4852-22-6	Proanthocyanidins	7.2422
115939-25-8	Salvianolic acid B	7.2433
4852-22-6	Proanthocyanidins	7.2607
4852-22-6	Proanthocyanidins	7.2691
69558-55-0	Thymopentin	7.271
4852-22-6	Proanthocyanidins	7.2732
4852-22-6	Proanthocyanidins	7.2752
147127-20-6	Tenofovir	7.2879
4852-22-6	Proanthocyanidins	7.2895
55-56-1	Chlorhexidine	7.2936
4852-22-6	Proanthocyanidins	7.2971
4852-22-6	Proanthocyanidins	7.3032
1405-37-4	Capastat sulfate	7.3385
4852-22-6	Proanthocyanidins	7.3437
4852-22-6	Proanthocyanidins	7.3553
4852-22-6	Proanthocyanidins	7.3678
4852-22-6	Proanthocyanidins	7.3767
147127-20-6	Tenofovir	7.3836
6160-12-9	(-)-Sparteine sulfate pentahydrate	7.3904
147127-20-6	Tenofovir	7.4107
24390-14-5	Doxycycline hyclate	7.418
4852-22-6	Proanthocyanidins	7.4191
4852-22-6	Proanthocyanidins	7.4292
17696-69-4	Hematoporphyrin dihydrochloride	7.4353
4852-22-6	Proanthocyanidins	7.4415
1405-37-4	Capastat sulfate	7.4628
4852-22-6	Proanthocyanidins	7.4693
95734-82-0	Nedaplatin	7.4701
4852-22-6	Proanthocyanidins	7.4719
4852-22-6	Proanthocyanidins	7.4719
55-56-1	Chlorhexidine	7.4775
4852-22-6	Proanthocyanidins	7.4847
4852-22-6	Proanthocyanidins	7.4848
4852-22-6	Proanthocyanidins	7.4887
1405-37-4	Capastat sulfate	7.4941
4852-22-6	Proanthocyanidins	7.4955

4852-22-6	Proanthocyanidins	7.4973
4852-22-6	Proanthocyanidins	7.5272
4852-22-6	Proanthocyanidins	7.5346
4852-22-6	Proanthocyanidins	7.5567
4852-22-6	Proanthocyanidins	7.5737
1405-37-4	Capastat sulfate	7.5769
4852-22-6	Proanthocyanidins	7.6032
4852-22-6	Proanthocyanidins	7.6045
4852-22-6	Proanthocyanidins	7.6226
4852-22-6	Proanthocyanidins	7.6324
4852-22-6	Proanthocyanidins	7.6328
4852-22-6	Proanthocyanidins	7.6434
4852-22-6	Proanthocyanidins	7.6484
4852-22-6	Proanthocyanidins	7.6488
4852-22-6	Proanthocyanidins	7.6548
4852-22-6	Proanthocyanidins	7.6553
4852-22-6	Proanthocyanidins	7.6636
24390-14-5	Doxycycline hyclate	7.6738
4852-22-6	Proanthocyanidins	7.676
4852-22-6	Proanthocyanidins	7.709
4852-22-6	Proanthocyanidins	7.7186
4852-22-6	Proanthocyanidins	7.7225
4852-22-6	Proanthocyanidins	7.725
4852-22-6	Proanthocyanidins	7.7336
3697-42-5	Chlorhexidine hydrochloride	7.7379
56-95-1	Chlorhexidine Acetate	7.7379
4852-22-6	Proanthocyanidins	7.7408
6160-12-9	(-)-Sparteine sulfate pentahydrate	7.7464
4852-22-6	Proanthocyanidins	7.7568
4852-22-6	Proanthocyanidins	7.7666
95734-82-0	Nedaplatin	7.7692
6160-12-9	(-)-Sparteine sulfate pentahydrate	7.7797
67-43-6	Pentetic Acid	7.7822
4852-22-6	Proanthocyanidins	7.7897
95734-82-0	Nedaplatin	7.7939
24390-14-5	Doxycycline hyclate	7.8002
24390-14-5	Doxycycline hyclate	7.802
4852-22-6	Proanthocyanidins	7.8055
4852-22-6	Proanthocyanidins	7.8172
4852-22-6	Proanthocyanidins	7.8339
17696-69-4	Hematoporphyrin dihydrochloride	7.8413
4852-22-6	Proanthocyanidins	7.8438
4852-22-6	Proanthocyanidins	7.8542

4852-22-6	Proanthocyanidins	7.8548
4852-22-6	Proanthocyanidins	7.8562
4852-22-6	Proanthocyanidins	7.8744
4852-22-6	Proanthocyanidins	7.8763
4852-22-6	Proanthocyanidins	7.8809
4852-22-6	Proanthocyanidins	7.8815
4852-22-6	Proanthocyanidins	7.8877
4852-22-6	Proanthocyanidins	7.8957
4852-22-6	Proanthocyanidins	7.9056
17696-69-4	Hematoporphyrin dihydrochloride	7.9208
4852-22-6	Proanthocyanidins	7.9339
129497-78-5	Verteporfin	7.9343
4852-22-6	Proanthocyanidins	7.9409
4852-22-6	Proanthocyanidins	7.9423
389139-89-3	Omadacycline	7.9426
4852-22-6	Proanthocyanidins	7.9428
55-56-1	Chlorhexidine	7.9481
4852-22-6	Proanthocyanidins	7.9643
129497-78-5	Verteporfin	7.9806
4852-22-6	Proanthocyanidins	7.996
24390-14-5	Doxycycline hyclate	8.0055
4852-22-6	Proanthocyanidins	8.007
4852-22-6	Proanthocyanidins	8.0088
4852-22-6	Proanthocyanidins	8.0109
4852-22-6	Proanthocyanidins	8.0186
4852-22-6	Proanthocyanidins	8.0275
4852-22-6	Proanthocyanidins	8.03
1405-37-4	Capastat sulfate	8.0386
4852-22-6	Proanthocyanidins	8.0525
4852-22-6	Proanthocyanidins	8.0702
4852-22-6	Proanthocyanidins	8.0942
4852-22-6	Proanthocyanidins	8.0946
4852-22-6	Proanthocyanidins	8.1033
4852-22-6	Proanthocyanidins	8.1122
24390-14-5	Doxycycline hyclate	8.1156
4852-22-6	Proanthocyanidins	8.1158
4852-22-6	Proanthocyanidins	8.1173
3697-42-5	Chlorhexidine hydrochloride	8.118
56-95-1	Chlorhexidine Acetate	8.118
4852-22-6	Proanthocyanidins	8.1201
4852-22-6	Proanthocyanidins	8.1274
1405-37-4	Capastat sulfate	8.1392
4852-22-6	Proanthocyanidins	8.1549

4852-22-6	Proanthocyanidins	8.1625
4852-22-6	Proanthocyanidins	8.1659
4852-22-6	Proanthocyanidins	8.1765
67-43-6	Pentetic Acid	8.1787
4852-22-6	Proanthocyanidins	8.183
4852-22-6	Proanthocyanidins	8.2084
129497-78-5	Verteporfin	8.2266
4852-22-6	Proanthocyanidins	8.2314
4852-22-6	Proanthocyanidins	8.2357
4852-22-6	Proanthocyanidins	8.2451
4852-22-6	Proanthocyanidins	8.2646
4852-22-6	Proanthocyanidins	8.271
4852-22-6	Proanthocyanidins	8.2969
4852-22-6	Proanthocyanidins	8.2996
4852-22-6	Proanthocyanidins	8.3008
4852-22-6	Proanthocyanidins	8.3069
69558-55-0	Thymopentin	8.3225
4852-22-6	Proanthocyanidins	8.3284
4852-22-6	Proanthocyanidins	8.3461
4852-22-6	Proanthocyanidins	8.3485
4852-22-6	Proanthocyanidins	8.3549
24390-14-5	Doxycycline hyclate	8.3575
4852-22-6	Proanthocyanidins	8.3621
4852-22-6	Proanthocyanidins	8.3702
4852-22-6	Proanthocyanidins	8.3808
4852-22-6	Proanthocyanidins	8.3918
4852-22-6	Proanthocyanidins	8.4011
4852-22-6	Proanthocyanidins	8.4105
67-43-6	Pentetic Acid	8.414
4852-22-6	Proanthocyanidins	8.4216
4852-22-6	Proanthocyanidins	8.4288
4852-22-6	Proanthocyanidins	8.4319
4852-22-6	Proanthocyanidins	8.4463
1405-37-4	Capastat sulfate	8.4489
4852-22-6	Proanthocyanidins	8.4536
24390-14-5	Doxycycline hyclate	8.4644
389139-89-3	Omadacycline	8.4723
4852-22-6	Proanthocyanidins	8.4733
4852-22-6	Proanthocyanidins	8.4818
24390-14-5	Doxycycline hyclate	8.4865
4852-22-6	Proanthocyanidins	8.4896
1119-34-2	L-arginine monohydrochloride	8.4925
4852-22-6	Proanthocyanidins	8.4928

74-79-3	L-Arginine	8.4931
4852-22-6	Proanthocyanidins	8.5099
1405-37-4	Capastat sulfate	8.5203
4852-22-6	Proanthocyanidins	8.5207
4852-22-6	Proanthocyanidins	8.5537
4852-22-6	Proanthocyanidins	8.5694
4852-22-6	Proanthocyanidins	8.5696
4852-22-6	Proanthocyanidins	8.5859
4852-22-6	Proanthocyanidins	8.5881
4852-22-6	Proanthocyanidins	8.5916
4852-22-6	Proanthocyanidins	8.5942
4852-22-6	Proanthocyanidins	8.5958
4852-22-6	Proanthocyanidins	8.5959
4852-22-6	Proanthocyanidins	8.5991
4852-22-6	Proanthocyanidins	8.6002
4852-22-6	Proanthocyanidins	8.6019
4852-22-6	Proanthocyanidins	8.6088
1405-37-4	Capastat sulfate	8.6116
4852-22-6	Proanthocyanidins	8.6423
4852-22-6	Proanthocyanidins	8.6662
24390-14-5	Doxycycline hyclate	8.6726
389139-89-3	Omadacycline	8.6885
3697-42-5	Chlorhexidine hydrochloride	8.6959
56-95-1	Chlorhexidine Acetate	8.6959
74-79-3	L-Arginine	8.724
1119-34-2	L-arginine monohydrochloride	8.724
4852-22-6	Proanthocyanidins	8.7259
389139-89-3	Omadacycline	8.7539
1405-37-4	Capastat sulfate	8.7564
4852-22-6	Proanthocyanidins	8.764
4852-22-6	Proanthocyanidins	8.7762
4852-22-6	Proanthocyanidins	8.8075
4852-22-6	Proanthocyanidins	8.816
4852-22-6	Proanthocyanidins	8.8461
4852-22-6	Proanthocyanidins	8.8516
4852-22-6	Proanthocyanidins	8.8762
4852-22-6	Proanthocyanidins	8.8873
4852-22-6	Proanthocyanidins	8.9026
1405-37-4	Capastat sulfate	8.9121
4852-22-6	Proanthocyanidins	8.9134
69558-55-0	Thymopentin	8.914
389139-89-3	Omadacycline	8.9186
4852-22-6	Proanthocyanidins	8.9403

4852-22-6	Proanthocyanidins	8.9514
4852-22-6	Proanthocyanidins	8.9624
1405-37-4	Capastat sulfate	9.027
1405-37-4	Capastat sulfate	9.0397
4852-22-6	Proanthocyanidins	9.0462
4852-22-6	Proanthocyanidins	9.0613
24390-14-5	Doxycycline hyclate	9.076
220620-09-7	Tigecycline	9.0851
4852-22-6	Proanthocyanidins	9.1102
1405-37-4	Capastat sulfate	9.1407
1405-37-4	Capastat sulfate	9.1794
74-79-3	L-Arginine	9.1881
1119-34-2	L-arginine monohydrochloride	9.1881
4852-22-6	Proanthocyanidins	9.2185
24390-14-5	Doxycycline hyclate	9.2406
4852-22-6	Proanthocyanidins	9.2603
4852-22-6	Proanthocyanidins	9.2958
220620-09-7	Tigecycline	9.3091
389139-89-3	Omadacycline	9.3987
220620-09-7	Tigecycline	9.4955
1405-37-4	Capastat sulfate	9.5097
389139-89-3	Omadacycline	9.7864
1405-37-4	Capastat sulfate	10.2174
69558-55-0	Thymopentin	11.5584
137281-23-3	Pemetrexed acid	11.6319
151533-22-1	Levomefolate calcium	12.1527
17696-69-4	Hematoporphyrin dihydrochloride	12.2106
137281-23-3	Pemetrexed acid	12.2485
137281-23-3	Pemetrexed acid	12.2776
17696-69-4	Hematoporphyrin dihydrochloride	12.2784
17696-69-4	Hematoporphyrin dihydrochloride	12.2868
17696-69-4	Hematoporphyrin dihydrochloride	12.3903
17696-69-4	Hematoporphyrin dihydrochloride	12.5655
17696-69-4	Hematoporphyrin dihydrochloride	12.5999
17696-69-4	Hematoporphyrin dihydrochloride	12.603
151533-22-1	Levomefolate calcium	12.6199
151533-22-1	Levomefolate calcium	12.6279
67-43-6	Pentetic Acid	12.6634
17696-69-4	Hematoporphyrin dihydrochloride	12.6952
17696-69-4	Hematoporphyrin dihydrochloride	12.7417
151533-22-1	Levomefolate calcium	12.8054
17696-69-4	Hematoporphyrin dihydrochloride	12.8077
151533-22-1	Levomefolate calcium	12.8171

151533-22-1	Levomefolate calcium	12.8339
151533-22-1	Levomefolate calcium	12.8443
151533-22-1	Levomefolate calcium	12.8443
151533-22-1	Levomefolate calcium	12.8731
67-43-6	Pentetic Acid	12.8774
17696-69-4	Hematoporphyrin dihydrochloride	12.9346
151533-22-1	Levomefolate calcium	12.9428
151533-22-1	Levomefolate calcium	12.956
17696-69-4	Hematoporphyrin dihydrochloride	12.9785
151533-22-1	Levomefolate calcium	12.9808
151533-22-1	Levomefolate calcium	12.9965
151533-22-1	Levomefolate calcium	13.0093
151533-22-1	Levomefolate calcium	13.0096
151533-22-1	Levomefolate calcium	13.0272
151533-22-1	Levomefolate calcium	13.0358
151533-22-1	Levomefolate calcium	13.051
161973-10-0	Esomeprazole Magnesium	13.0779
67-43-6	Pentetic Acid	13.0834
161973-10-0	Esomeprazole Magnesium	13.0895
151533-22-1	Levomefolate calcium	13.0946
151533-22-1	Levomefolate calcium	13.0996
151533-22-1	Levomefolate calcium	13.1095
151533-22-1	Levomefolate calcium	13.1231
151533-22-1	Levomefolate calcium	13.2023
151533-22-1	Levomefolate calcium	13.2103
151533-22-1	Levomefolate calcium	13.2319
151533-22-1	Levomefolate calcium	13.232
151533-22-1	Levomefolate calcium	13.2431
161973-10-0	Esomeprazole Magnesium	13.258
151533-22-1	Levomefolate calcium	13.2728
151533-22-1	Levomefolate calcium	13.2783
151533-22-1	Levomefolate calcium	13.3108
151533-22-1	Levomefolate calcium	13.351
151533-22-1	Levomefolate calcium	13.3516
151533-22-1	Levomefolate calcium	13.3518
151533-22-1	Levomefolate calcium	13.3672
69558-55-0	Thymopentin	13.3702
1197953-54-0	Brigatinib	13.3982
151533-22-1	Levomefolate calcium	13.4059
69558-55-0	Thymopentin	13.4159
151533-22-1	Levomefolate calcium	13.4162
151533-22-1	Levomefolate calcium	13.4209

151533-22-1	Levomefolate calcium	13.4689
151533-22-1	Levomefolate calcium	13.4707
151533-22-1	Levomefolate calcium	13.4726
151533-22-1	Levomefolate calcium	13.4834
151533-22-1	Levomefolate calcium	13.4971
151533-22-1	Levomefolate calcium	13.5005
151533-22-1	Levomefolate calcium	13.5145
151533-22-1	Levomefolate calcium	13.5206
151533-22-1	Levomefolate calcium	13.5298
151533-22-1	Levomefolate calcium	13.5312
151533-22-1	Levomefolate calcium	13.5436
151533-22-1	Levomefolate calcium	13.5459
151533-22-1	Levomefolate calcium	13.5525
151533-22-1	Levomefolate calcium	13.5533
151533-22-1	Levomefolate calcium	13.5626
151533-22-1	Levomefolate calcium	13.5641
151533-22-1	Levomefolate calcium	13.6041
151533-22-1	Levomefolate calcium	13.6198
151533-22-1	Levomefolate calcium	13.6252
151533-22-1	Levomefolate calcium	13.6337
151533-22-1	Levomefolate calcium	13.6426
151533-22-1	Levomefolate calcium	13.644
151533-22-1	Levomefolate calcium	13.6444
151533-22-1	Levomefolate calcium	13.6533
151533-22-1	Levomefolate calcium	13.6641
151533-22-1	Levomefolate calcium	13.6679
151533-22-1	Levomefolate calcium	13.7091
151533-22-1	Levomefolate calcium	13.722
151533-22-1	Levomefolate calcium	13.7339
151533-22-1	Levomefolate calcium	13.7572
3697-42-5	Chlorhexidine hydrochloride	13.7605
56-95-1	Chlorhexidine Acetate	13.7605
55-56-1	Chlorhexidine	13.7605
151533-22-1	Levomefolate calcium	13.7641
151533-22-1	Levomefolate calcium	13.7729
3697-42-5	Chlorhexidine hydrochloride	13.779
56-95-1	Chlorhexidine Acetate	13.779
55-56-1	Chlorhexidine	13.779
151533-22-1	Levomefolate calcium	13.7818
151533-22-1	Levomefolate calcium	13.792
151533-22-1	Levomefolate calcium	13.8143
151533-22-1	Levomefolate calcium	13.8304
151533-22-1	Levomefolate calcium	13.8344

151533-22-1	Levomefolate calcium	13.8478
151533-22-1	Levomefolate calcium	13.8552
151533-22-1	Levomefolate calcium	13.8598
151533-22-1	Levomefolate calcium	13.8758
151533-22-1	Levomefolate calcium	13.8764
151533-22-1	Levomefolate calcium	13.8803
151533-22-1	Levomefolate calcium	13.881
151533-22-1	Levomefolate calcium	13.882
151533-22-1	Levomefolate calcium	13.9032
151533-22-1	Levomefolate calcium	13.9283
1197953-54-0	Brigatinib	13.9434
115939-25-8	Salvianolic acid B	13.9519
151533-22-1	Levomefolate calcium	13.9579
115939-25-8	Salvianolic acid B	13.9947
151533-22-1	Levomefolate calcium	14.0004
151533-22-1	Levomefolate calcium	14.0027
151533-22-1	Levomefolate calcium	14.0195
151533-22-1	Levomefolate calcium	14.0243
151533-22-1	Levomefolate calcium	14.0359
151533-22-1	Levomefolate calcium	14.0407
151533-22-1	Levomefolate calcium	14.048
151533-22-1	Levomefolate calcium	14.0805
151533-22-1	Levomefolate calcium	14.0901
151533-22-1	Levomefolate calcium	14.1071
151533-22-1	Levomefolate calcium	14.1145
151533-22-1	Levomefolate calcium	14.1399
151533-22-1	Levomefolate calcium	14.154
151533-22-1	Levomefolate calcium	14.1551
151533-22-1	Levomefolate calcium	14.1708
151533-22-1	Levomefolate calcium	14.1858
151533-22-1	Levomefolate calcium	14.1895
151533-22-1	Levomefolate calcium	14.1918
115939-25-8	Salvianolic acid B	14.1947
151533-22-1	Levomefolate calcium	14.2015
151533-22-1	Levomefolate calcium	14.2045
151533-22-1	Levomefolate calcium	14.2133
129497-78-5	Verteporfin	14.2251
151533-22-1	Levomefolate calcium	14.226
151533-22-1	Levomefolate calcium	14.2304
3697-42-5	Chlorhexidine hydrochloride	14.2445
56-95-1	Chlorhexidine Acetate	14.2445
55-56-1	Chlorhexidine	14.2445

129497-78-5	Verteporfin	14.2762
1197953-54-0	Brigatinib	14.2769
1207456-01-6	Talazoparib	14.3042
1207456-01-6	Talazoparib	14.3099
151533-22-1	Levomefolate calcium	14.3255
151533-22-1	Levomefolate calcium	14.3314
115939-25-8	Salvianolic acid B	14.3439
129497-78-5	Verteporfin	14.361
151533-22-1	Levomefolate calcium	14.4196
220620-09-7	Tigecycline	14.4636
151533-22-1	Levomefolate calcium	14.4693
151533-22-1	Levomefolate calcium	14.4973
151533-22-1	Levomefolate calcium	14.5244
151533-22-1	Levomefolate calcium	14.554
151533-22-1	Levomefolate calcium	14.5842
151533-22-1	Levomefolate calcium	14.5886
1207456-01-6	Talazoparib	14.6078
220620-09-7	Tigecycline	14.616
151533-22-1	Levomefolate calcium	14.62
151533-22-1	Levomefolate calcium	14.6689
151533-22-1	Levomefolate calcium	14.7151
151533-22-1	Levomefolate calcium	14.7309
151533-22-1	Levomefolate calcium	14.7454
151533-22-1	Levomefolate calcium	14.7497
151533-22-1	Levomefolate calcium	14.7514
151533-22-1	Levomefolate calcium	14.8063
151533-22-1	Levomefolate calcium	14.8419
151533-22-1	Levomefolate calcium	14.8425
151533-22-1	Levomefolate calcium	14.8592
115939-25-8	Salvianolic acid B	14.8993
151533-22-1	Levomefolate calcium	14.9297
151533-22-1	Levomefolate calcium	14.9376
151533-22-1	Levomefolate calcium	14.964
151533-22-1	Levomefolate calcium	15.0094
151533-22-1	Levomefolate calcium	15.0152
151533-22-1	Levomefolate calcium	15.0271
151533-22-1	Levomefolate calcium	15.0519
151533-22-1	Levomefolate calcium	15.0595
151533-22-1	Levomefolate calcium	15.1681
151533-22-1	Levomefolate calcium	15.2212

151533-22-1	Levomefolate calcium	15.236
220620-09-7	Tigecycline	15.2441
151533-22-1	Levomefolate calcium	15.2555
151533-22-1	Levomefolate calcium	15.3072
151533-22-1	Levomefolate calcium	15.318
151533-22-1	Levomefolate calcium	15.4254
23651-95-8	Droxidopa	15.4266
23651-95-8	Droxidopa	15.4497
20537-88-6	Amifostine	15.4751
151533-22-1	Levomefolate calcium	15.5021
23651-95-8	Droxidopa	15.5076
20537-88-6	Amifostine	15.511
1405-37-4	Capastat sulfate	15.5273
151533-22-1	Levomefolate calcium	15.5356
151533-22-1	Levomefolate calcium	15.5763
151533-22-1	Levomefolate calcium	15.596
151533-22-1	Levomefolate calcium	15.5984
151533-22-1	Levomefolate calcium	15.6895
151533-22-1	Levomefolate calcium	15.6958
151533-22-1	Levomefolate calcium	15.7006
151533-22-1	Levomefolate calcium	15.7155
20537-88-6	Amifostine	15.7427
151533-22-1	Levomefolate calcium	15.7804
151533-22-1	Levomefolate calcium	15.8054
1405-37-4	Capastat sulfate	15.8823
151533-22-1	Levomefolate calcium	15.9865
151533-22-1	Levomefolate calcium	16.3519
115939-25-8	Salvianolic acid B	16.4895

LIST of VS1 PDB complexes:

PARP1_DSI_VS1_complex

```
HETATM    1  CH3 ACE B 661      54.735  14.649  60.442  1.00  0.00           C
HETATM    2  C   ACE B 661      53.315  14.962  59.985  1.00  0.00           C
HETATM    3  O   ACE B 661      53.143  15.728  59.040  1.00  0.00           O
HETATM    4 1H   ACE B 661      55.322  14.259  59.611  1.00  0.00           H
HETATM    5 2H   ACE B 661      55.219  15.555  60.805  1.00  0.00           H
HETATM    6 3H   ACE B 661      54.746  13.910  61.245  1.00  0.00           H
ATOM      7  N   LYS B 662      52.316  14.361  60.658  1.00 47.37           N
ANISOU    7  N   LYS B 662     4539   8722   4740   1624   -289   1575       N
ATOM      8  CA  LYS B 662      50.884  14.490  60.367  1.00 46.49           C
ANISOU    8  CA  LYS B 662     4699   8174   4790   1530   -270   1414       C
ATOM      9  C   LYS B 662      50.321  15.917  60.490  1.00 46.77           C
ANISOU    9  C   LYS B 662     4758   8118   4896   1171   -365   1309       C
ATOM     10  O   LYS B 662      50.765  16.697  61.334  1.00 46.53           O
ANISOU   10  O   LYS B 662     4632   8257   4790    983   -456   1346       O
ATOM     11  CB  LYS B 662      50.083  13.544  61.287  1.00 49.43           C
ANISOU   11  CB  LYS B 662     5227   8382   5170   1673   -235   1417       C
ATOM     12  CG  LYS B 662      50.124  12.069  60.858  1.00 63.07           C
ANISOU   12  CG  LYS B 662     7116   9959   6889   2005   -113   1442       C
ATOM     13  CD  LYS B 662      49.141  11.211  61.671  1.00 71.72           C
ANISOU   13  CD  LYS B 662     8477  10707   8067   2016    -78   1360       C
ATOM     14  CE  LYS B 662      49.034   9.769  61.152  1.00 84.00           C
ANISOU   14  CE  LYS B 662    10262  12040   9614   2280     39   1358       C
ATOM     15  NZ  LYS B 662      48.051   8.988  61.924  1.00 93.15           N1+
ANISOU   15  NZ  LYS B 662    11688  12847  10859   2216     63   1266       N1+
ATOM     16  H1  LYS B 662      52.546  13.750  61.429  1.00 47.37           H
ATOM     17  HA  LYS B 662      50.751  14.167  59.333  1.00 46.49           H
ATOM     18  HB3 LYS B 662      49.032  13.842  61.267  1.00 49.43           H
ATOM     19  HB2 LYS B 662      50.399  13.657  62.325  1.00 49.43           H
ATOM     20  HG3 LYS B 662      51.137  11.678  60.965  1.00 63.07           H
ATOM     21  HG2 LYS B 662      49.876  11.996  59.798  1.00 63.07           H
ATOM     22  HD3 LYS B 662      48.156  11.681  61.654  1.00 71.72           H
ATOM     23  HD2 LYS B 662      49.458  11.205  62.715  1.00 71.72           H
ATOM     24  HE3 LYS B 662      50.003   9.272  61.206  1.00 84.00           H
ATOM     25  HE2 LYS B 662      48.729   9.768  60.105  1.00 84.00           H
ATOM     26  HZ1 LYS B 662      47.144   9.428  61.860  1.00 93.15           H
ATOM     27  HZ2 LYS B 662      47.996   8.050  61.554  1.00 93.15           H
ATOM     28  HZ3 LYS B 662      48.338   8.950  62.892  1.00 93.15           H
ATOM     29  N   SER B 663      49.302  16.180  59.656  1.00 40.06           N
ANISOU   29  N   SER B 663     4053   6996   4173   1084   -340   1184       N
ATOM     30  CA  SER B 663      48.497  17.396  59.598  1.00 38.57           C
ANISOU   30  CA  SER B 663     3929   6678   4046    795   -406   1081       C
ATOM     31  C   SER B 663      47.720  17.661  60.900  1.00 42.31           C
ANISOU   31  C   SER B 663     4537   6993   4547    689   -441   1028       C
ATOM     32  O   SER B 663      47.110  16.743  61.452  1.00 41.53           O
ANISOU   32  O   SER B 663     4546   6748   4486    838   -390   1022       O
ATOM     33  CB  SER B 663      47.554  17.280  58.380  1.00 39.61           C
ANISOU   33  CB  SER B 663     4185   6566   4299    786   -355    974       C
ATOM     34  OG  SER B 663      46.699  18.396  58.242  1.00 42.62           O
ANISOU   34  OG  SER B 663     4649   6813   4733    546   -406    880       O
ATOM     35  H   SER B 663      49.046  15.474  58.976  1.00 40.06           H
ATOM     36  HA  SER B 663      49.181  18.229  59.434  1.00 38.57           H
ATOM     37  HB3 SER B 663      46.946  16.377  58.444  1.00 39.61           H
```

ATOM 38 HB2 SER B 663 48.139 17.201 57.463 1.00 39.61 H
ATOM 39 HG SER B 663 47.225 19.121 57.882 1.00 42.62 H
ATOM 40 N LYS B 664 47.740 18.934 61.324 1.00 37.56 N
ANISOU 40 N LYS B 664 3943 6417 3910 432 -526 994 N
ATOM 41 CA LYS B 664 47.018 19.450 62.489 1.00 36.33 C
ANISOU 41 CA LYS B 664 3927 6115 3760 319 -560 941 C
ATOM 42 C LYS B 664 45.661 20.081 62.116 1.00 36.72 C
ANISOU 42 C LYS B 664 4162 5871 3917 230 -536 814 C
ATOM 43 O LYS B 664 44.959 20.537 63.019 1.00 36.19 O
ANISOU 43 O LYS B 664 4231 5660 3859 164 -547 764 O
ATOM 44 CB LYS B 664 47.919 20.464 63.225 1.00 39.27 C
ANISOU 44 CB LYS B 664 4231 6698 3991 99 -667 992 C
ATOM 45 CG LYS B 664 49.188 19.835 63.828 1.00 56.08 C
ANISOU 45 CG LYS B 664 6171 9141 5995 180 -698 1130 C
ATOM 46 CD LYS B 664 50.035 20.854 64.604 1.00 68.58 C
ANISOU 46 CD LYS B 664 7692 10943 7421 -96 -817 1179 C
ATOM 47 CE LYS B 664 51.309 20.230 65.194 1.00 82.30 C
ANISOU 47 CE LYS B 664 9165 13099 9007 -59 -861 1341 C
ATOM 48 NZ LYS B 664 52.114 21.221 65.930 1.00 92.80 N1+
ANISOU 48 NZ LYS B 664 10423 14669 10166 -394 -988 1391 N1+
ATOM 49 H LYS B 664 48.266 19.615 60.796 1.00 37.56 H
ATOM 50 HA LYS B 664 46.803 18.630 63.177 1.00 36.33 H
ATOM 51 HB3 LYS B 664 47.358 20.926 64.039 1.00 39.27 H
ATOM 52 HB2 LYS B 664 48.195 21.275 62.548 1.00 39.27 H
ATOM 53 HG3 LYS B 664 49.795 19.394 63.036 1.00 56.08 H
ATOM 54 HG2 LYS B 664 48.908 19.015 64.490 1.00 56.08 H
ATOM 55 HD3 LYS B 664 49.429 21.285 65.404 1.00 68.58 H
ATOM 56 HD2 LYS B 664 50.297 21.680 63.940 1.00 68.58 H
ATOM 57 HE3 LYS B 664 51.922 19.803 64.399 1.00 82.30 H
ATOM 58 HE2 LYS B 664 51.051 19.417 65.874 1.00 82.30 H
ATOM 59 HZ1 LYS B 664 51.569 21.602 66.690 1.00 92.80 H
ATOM 60 HZ2 LYS B 664 52.941 20.775 66.301 1.00 92.80 H
ATOM 61 HZ3 LYS B 664 52.388 21.967 65.306 1.00 92.80 H
ATOM 62 N LEU B 665 45.300 20.090 60.817 1.00 31.05 N
ANISOU 62 N LEU B 665 3445 5082 3269 244 -499 770 N
ATOM 63 CA LEU B 665 44.015 20.576 60.300 1.00 29.43 C
ANISOU 63 CA LEU B 665 3388 4637 3156 186 -470 665 C
ATOM 64 C LEU B 665 42.829 19.737 60.833 1.00 31.11 C
ANISOU 64 C LEU B 665 3719 4648 3453 307 -406 627 C
ATOM 65 O LEU B 665 43.019 18.547 61.094 1.00 29.28 O
ANISOU 65 O LEU B 665 3464 4424 3236 466 -363 670 O
ATOM 66 CB LEU B 665 44.026 20.499 58.754 1.00 29.18 C
ANISOU 66 CB LEU B 665 3315 4593 3180 210 -436 641 C
ATOM 67 CG LEU B 665 44.998 21.468 58.043 1.00 33.89 C
ANISOU 67 CG LEU B 665 3821 5350 3706 55 -493 662 C
ATOM 68 CD1 LEU B 665 45.111 21.124 56.541 1.00 33.93 C
ANISOU 68 CD1 LEU B 665 3764 5362 3765 132 -446 654 C
ATOM 69 CD2 LEU B 665 44.623 22.948 58.275 1.00 35.93 C
ANISOU 69 CD2 LEU B 665 4217 5501 3935 -149 -539 597 C
ATOM 70 H LEU B 665 45.922 19.683 60.133 1.00 31.05 H
ATOM 71 HA LEU B 665 43.933 21.613 60.618 1.00 29.43 H
ATOM 72 HB3 LEU B 665 43.023 20.680 58.363 1.00 29.18 H
ATOM 73 HB2 LEU B 665 44.264 19.475 58.467 1.00 29.18 H
ATOM 74 HG LEU B 665 45.990 21.312 58.468 1.00 33.89 H
ATOM 75 HD11 LEU B 665 46.154 21.005 56.246 1.00 33.93 H
ATOM 76 HD12 LEU B 665 44.600 20.193 56.294 1.00 33.93 H

```
ATOM     77 HD13 LEU B 665      44.679  21.891  55.901  1.00 33.93           H
ATOM     78 HD21 LEU B 665      44.651  23.539  57.360  1.00 35.93           H
ATOM     79 HD22 LEU B 665      43.622  23.057  58.694  1.00 35.93           H
ATOM     80 HD23 LEU B 665      45.319  23.415  58.972  1.00 35.93           H
ATOM     81  N   PRO B 666      41.614  20.335  60.928  1.00 26.82           N
ANISOU   81  N   PRO B 666     3310   3931   2951    243   -394    556       N
ATOM     82  CA  PRO B 666      40.361  19.579  61.151  1.00 26.38           C
ANISOU   82  CA  PRO B 666     3341   3720   2962    338   -335    533       C
ATOM     83  C   PRO B 666      40.140  18.449  60.129  1.00 29.72           C
ANISOU   83  C   PRO B 666     3754   4079   3457    447   -274    530       C
ATOM     84  O   PRO B 666      40.435  18.646  58.952  1.00 28.92           O
ANISOU   84  O   PRO B 666     3613   3995   3381    432   -269    510       O
ATOM     85  CB  PRO B 666      39.260  20.648  61.025  1.00 28.18           C
ANISOU   85  CB  PRO B 666     3679   3824   3203    257   -329    469       C
ATOM     86  CG  PRO B 666      39.948  21.962  61.342  1.00 32.64           C
ANISOU   86  CG  PRO B 666     4267   4452   3682    122   -392    461       C
ATOM     87  CD  PRO B 666      41.344  21.765  60.770  1.00 28.27           C
ANISOU   87  CD  PRO B 666     3580   4057   3103     92   -428    501       C
ATOM     88  HA  PRO B 666      40.386  19.182  62.168  1.00 26.38           H
ATOM     89  HB3 PRO B 666      38.414  20.459  61.688  1.00 28.18           H
ATOM     90  HB2 PRO B 666      38.869  20.691  60.007  1.00 28.18           H
ATOM     91  HG3 PRO B 666      40.013  22.083  62.424  1.00 32.64           H
ATOM     92  HG2 PRO B 666      39.431  22.832  60.935  1.00 32.64           H
ATOM     93  HD2 PRO B 666      41.366  22.017  59.708  1.00 28.27           H
ATOM     94  HD3 PRO B 666      42.051  22.405  61.297  1.00 28.27           H
ATOM     95  N   LYS B 667      39.622  17.299  60.591  1.00 26.37           N
ANISOU   95  N   LYS B 667     3386   3579   3053    547   -230    551       N
ATOM     96  CA  LYS B 667      39.318  16.132  59.753  1.00 26.07           C
ANISOU   96  CA  LYS B 667     3400   3448   3058    635   -172    548       C
ATOM     97  C   LYS B 667      38.403  16.402  58.525  1.00 28.43           C
ANISOU   97  C   LYS B 667     3727   3655   3419    561   -152    484       C
ATOM     98  O   LYS B 667      38.733  15.882  57.459  1.00 28.02           O
ANISOU   98  O   LYS B 667     3664   3602   3382    602   -132    478       O
ATOM     99  CB  LYS B 667      38.848  14.960  60.651  1.00 27.90           C
ANISOU   99  CB  LYS B 667     3736   3582   3282    703   -134    575       C
ATOM    100  CG  LYS B 667      38.416  13.661  59.940  1.00 41.16           C
ANISOU  100  CG  LYS B 667     5528   5132   4979    765    -75    571       C
ATOM    101  CD  LYS B 667      39.561  12.943  59.197  1.00 52.81           C
ANISOU  101  CD  LYS B 667     7013   6649   6404    930    -47    620       C
ATOM    102  CE  LYS B 667      39.123  11.664  58.458  1.00 66.40           C
ANISOU  102  CE  LYS B 667     8882   8220   8127    973     11    600       C
ATOM    103  NZ  LYS B 667      38.679  10.595  59.369  1.00 74.16           N1+
ANISOU  103  NZ  LYS B 667    10047   9032   9098    938     43    599       N1+
ATOM    104  H   LYS B 667      39.409  17.208  61.574  1.00 26.37           H
ATOM    105  HA  LYS B 667      40.283  15.833  59.341  1.00 26.07           H
ATOM    106  HB3 LYS B 667      38.011  15.292  61.266  1.00 27.90           H
ATOM    107  HB2 LYS B 667      39.642  14.719  61.359  1.00 27.90           H
ATOM    108  HG3 LYS B 667      37.597  13.860  59.248  1.00 41.16           H
ATOM    109  HG2 LYS B 667      37.999  12.994  60.694  1.00 41.16           H
ATOM    110  HD3 LYS B 667      40.362  12.710  59.900  1.00 52.81           H
ATOM    111  HD2 LYS B 667      40.001  13.622  58.466  1.00 52.81           H
ATOM    112  HE3 LYS B 667      39.950  11.285  57.857  1.00 66.40           H
ATOM    113  HE2 LYS B 667      38.307  11.884  57.773  1.00 66.40           H
ATOM    114  HZ1 LYS B 667      37.886  10.919  59.902  1.00 74.16           H
ATOM    115  HZ2 LYS B 667      38.412   9.784  58.830  1.00 74.16           H
ATOM    116  HZ3 LYS B 667      39.431  10.350  59.997  1.00 74.16           H
```

```
ATOM    117  N   PRO B 668      37.366  17.275  58.632  1.00 25.33           N
ANISOU  117  N   PRO B 668     3365   3209   3051    468   -157    444       N
ATOM    118  CA  PRO B 668      36.622  17.781  57.459  1.00 24.47           C
ANISOU  118  CA  PRO B 668     3261   3049   2987    414   -139    397       C
ATOM    119  C   PRO B 668      37.469  18.509  56.399  1.00 27.94           C
ANISOU  119  C   PRO B 668     3635   3552   3429    385   -164    376       C
ATOM    120  O   PRO B 668      37.263  18.265  55.211  1.00 27.61           O
ANISOU  120  O   PRO B 668     3593   3475   3423    377   -143    349       O
ATOM    121  CB  PRO B 668      35.564  18.714  58.070  1.00 25.94           C
ANISOU  121  CB  PRO B 668     3479   3209   3169    358   -136    381       C
ATOM    122  CG  PRO B 668      35.327  18.155  59.458  1.00 29.54           C
ANISOU  122  CG  PRO B 668     3969   3663   3592    388   -135    417       C
ATOM    123  CD  PRO B 668      36.726  17.725  59.872  1.00 25.30           C
ANISOU  123  CD  PRO B 668     3395   3196   3022    430   -169    446       C
ATOM    124  HA  PRO B 668      36.122  16.927  56.996  1.00 24.47           H
ATOM    125  HB3 PRO B 668      34.650  18.765  57.481  1.00 25.94           H
ATOM    126  HB2 PRO B 668      35.947  19.731  58.161  1.00 25.94           H
ATOM    127  HG3 PRO B 668      34.674  17.284  59.393  1.00 29.54           H
ATOM    128  HG2 PRO B 668      34.871  18.871  60.142  1.00 29.54           H
ATOM    129  HD2 PRO B 668      37.264  18.586  60.267  1.00 25.30           H
ATOM    130  HD3 PRO B 668      36.681  16.960  60.645  1.00 25.30           H
ATOM    131  N   VAL B 669      38.405  19.368  56.843  1.00 24.96           N
ANISOU  131  N   VAL B 669     3206   3274   3003    354   -210    392       N
ATOM    132  CA  VAL B 669      39.322  20.121  55.979  1.00 25.22           C
ANISOU  132  CA  VAL B 669     3175   3389   3021    301   -239    384       C
ATOM    133  C   VAL B 669      40.354  19.207  55.288  1.00 30.46           C
ANISOU  133  C   VAL B 669     3762   4125   3684    396   -220    420       C
ATOM    134  O   VAL B 669      40.627  19.423  54.111  1.00 30.19           O
ANISOU  134  O   VAL B 669     3701   4099   3673    390   -208    400       O
ATOM    135  CB  VAL B 669      40.059  21.260  56.751  1.00 28.83           C
ANISOU  135  CB  VAL B 669     3617   3937   3398    196   -302    396       C
ATOM    136  CG1 VAL B 669      41.167  21.987  55.955  1.00 28.32           C
ANISOU  136  CG1 VAL B 669     3485   3973   3303    116   -336    400       C
ATOM    137  CG2 VAL B 669      39.058  22.301  57.279  1.00 28.61           C
ANISOU  137  CG2 VAL B 669     3709   3809   3352    134   -307    357       C
ATOM    138  H   VAL B 669      38.533  19.495  57.836  1.00 24.96           H
ATOM    139  HA  VAL B 669      38.720  20.586  55.195  1.00 25.22           H
ATOM    140  HB  VAL B 669      40.545  20.823  57.623  1.00 28.83           H
ATOM    141 HG11 VAL B 669      41.566  22.831  56.518  1.00 28.32           H
ATOM    142 HG12 VAL B 669      42.015  21.337  55.736  1.00 28.32           H
ATOM    143 HG13 VAL B 669      40.787  22.375  55.009  1.00 28.32           H
ATOM    144 HG21 VAL B 669      39.558  23.075  57.863  1.00 28.61           H
ATOM    145 HG22 VAL B 669      38.530  22.791  56.462  1.00 28.61           H
ATOM    146 HG23 VAL B 669      38.314  21.839  57.925  1.00 28.61           H
ATOM    147  N   GLN B 670      40.864  18.179  55.994  1.00 27.83           N
ANISOU  147  N   GLN B 670     3414   3842   3319    503   -208    476       N
ATOM    148  CA  GLN B 670      41.766  17.167  55.432  1.00 27.57           C
ANISOU  148  CA  GLN B 670     3336   3878   3259    645   -174    524       C
ATOM    149  C   GLN B 670      41.125  16.355  54.290  1.00 31.51           C
ANISOU  149  C   GLN B 670     3938   4226   3810    702   -114    484       C
ATOM    150  O   GLN B 670      41.773  16.153  53.264  1.00 32.41           O
ANISOU  150  O   GLN B 670     4023   4379   3911    772    -88    493       O
ATOM    151  CB  GLN B 670      42.293  16.222  56.532  1.00 28.39           C
ANISOU  151  CB  GLN B 670     3437   4044   3306    773   -163    596       C
ATOM    152  CG  GLN B 670      43.206  16.905  57.572  1.00 37.64           C
ANISOU  152  CG  GLN B 670     4499   5395   4406    713   -227    648       C
```

```
ATOM    153  CD  GLN B 670      43.979  15.909  58.441  1.00 43.61           C
ANISOU  153  CD  GLN B 670     5223   6255   5091    874   -212    736       C
ATOM    154  NE2 GLN B 670      44.223  16.263  59.703  1.00 30.39           N
ANISOU  154  NE2 GLN B 670     3527   4646   3373    823   -256    766       N
ATOM    155  OE1 GLN B 670      44.391  14.847  57.978  1.00 37.47           O
ANISOU  155  OE1 GLN B 670     4451   5503   4284   1056   -156    783       O
ATOM    156  H   GLN B 670      40.598  18.061  56.963  1.00 27.83           H
ATOM    157  HA  GLN B 670      42.618  17.699  55.005  1.00 27.57           H
ATOM    158  HB3 GLN B 670      42.844  15.418  56.042  1.00 28.39           H
ATOM    159  HB2 GLN B 670      41.460  15.740  57.045  1.00 28.39           H
ATOM    160  HG3 GLN B 670      42.620  17.566  58.207  1.00 37.64           H
ATOM    161  HG2 GLN B 670      43.939  17.538  57.074  1.00 37.64           H
ATOM    162 HE22 GLN B 670      44.767  15.660  60.303  1.00 30.39           H
ATOM    163 HE21 GLN B 670      43.881  17.142  60.071  1.00 30.39           H
ATOM    164  N   ASP B 671      39.857  15.948  54.479  1.00 26.57           N
ANISOU  164  N   ASP B 671     3429   3440   3226    659    -93    445       N
ATOM    165  CA  ASP B 671      39.042  15.244  53.483  1.00 26.71           C
ANISOU  165  CA  ASP B 671     3561   3314   3274    661    -49    409       C
ATOM    166  C   ASP B 671      38.657  16.125  52.282  1.00 30.03           C
ANISOU  166  C   ASP B 671     3938   3735   3735    574    -58    359       C
ATOM    167  O   ASP B 671      38.536  15.592  51.180  1.00 30.85           O
ANISOU  167  O   ASP B 671     4092   3789   3842    608    -26    342       O
ATOM    168  CB  ASP B 671      37.763  14.609  54.085  1.00 27.88           C
ANISOU  168  CB  ASP B 671     3820   3339   3435    603    -35    399       C
ATOM    169  CG  ASP B 671      37.973  13.601  55.229  1.00 38.78           C
ANISOU  169  CG  ASP B 671     5285   4682   4768    701    -13    449       C
ATOM    170  OD1 ASP B 671      39.074  13.009  55.322  1.00 39.94           O
ANISOU  170  OD1 ASP B 671     5435   4876   4865    848      7    492       O
ATOM    171  OD2 ASP B 671      36.961  13.329  55.913  1.00 42.49           O1-
ANISOU  171  OD2 ASP B 671     5817   5088   5239    643    -12    453       O1-
ATOM    172  H   ASP B 671      39.405  16.139  55.364  1.00 26.57           H
ATOM    173  HA  ASP B 671      39.657  14.437  53.081  1.00 26.71           H
ATOM    174  HB3 ASP B 671      37.207  14.093  53.301  1.00 27.88           H
ATOM    175  HB2 ASP B 671      37.118  15.405  54.462  1.00 27.88           H
ATOM    176  N   LEU B 672      38.493  17.442  52.503  1.00 25.11           N
ANISOU  176  N   LEU B 672     3243   3168   3132    472    -99    337       N
ATOM    177  CA  LEU B 672      38.217  18.428  51.457  1.00 24.46           C
ANISOU  177  CA  LEU B 672     3124   3094   3076    397   -109    296       C
ATOM    178  C   LEU B 672      39.413  18.610  50.502  1.00 28.13           C
ANISOU  178  C   LEU B 672     3513   3656   3521    440   -112    310       C
ATOM    179  O   LEU B 672      39.199  18.672  49.293  1.00 26.70           O
ANISOU  179  O   LEU B 672     3343   3440   3360    440    -91    283       O
ATOM    180  CB  LEU B 672      37.768  19.758  52.110  1.00 23.67           C
ANISOU  180  CB  LEU B 672     3008   3015   2972    305   -145    278       C
ATOM    181  CG  LEU B 672      37.306  20.862  51.130  1.00 27.58           C
ANISOU  181  CG  LEU B 672     3486   3516   3476    236   -156    241       C
ATOM    182  CD1 LEU B 672      36.073  20.423  50.314  1.00 26.85           C
ANISOU  182  CD1 LEU B 672     3430   3348   3423    224   -123    211       C
ATOM    183  CD2 LEU B 672      37.075  22.198  51.869  1.00 27.89           C
ANISOU  183  CD2 LEU B 672     3555   3569   3473    167   -190    234       C
ATOM    184  H   LEU B 672      38.588  17.802  53.443  1.00 25.11           H
ATOM    185  HA  LEU B 672      37.385  18.040  50.867  1.00 24.46           H
ATOM    186  HB3 LEU B 672      38.588  20.148  52.712  1.00 23.67           H
ATOM    187  HB2 LEU B 672      36.957  19.557  52.812  1.00 23.67           H
ATOM    188  HG  LEU B 672      38.114  21.051  50.425  1.00 27.58           H
ATOM    189 HD11 LEU B 672      35.345  21.223  50.183  1.00 26.85           H
```

ATOM 190 HD12 LEU B 672 36.369 20.093 49.318 1.00 26.85 H
ATOM 191 HD13 LEU B 672 35.548 19.591 50.785 1.00 26.85 H
ATOM 192 HD21 LEU B 672 37.708 22.983 51.455 1.00 27.89 H
ATOM 193 HD22 LEU B 672 36.046 22.548 51.799 1.00 27.89 H
ATOM 194 HD23 LEU B 672 37.308 22.123 52.932 1.00 27.89 H
ATOM 195 N ILE B 673 40.642 18.646 51.051 1.00 26.33 N
ANISOU 195 N ILE B 673 3197 3565 3240 475 -137 363 N
ATOM 196 CA ILE B 673 41.890 18.728 50.285 1.00 26.57 C
ANISOU 196 CA ILE B 673 3122 3746 3228 523 -139 402 C
ATOM 197 C ILE B 673 42.168 17.453 49.461 1.00 31.65 C
ANISOU 197 C ILE B 673 3815 4348 3864 680 -74 414 C
ATOM 198 O ILE B 673 42.551 17.577 48.299 1.00 30.73 O
ANISOU 198 O ILE B 673 3665 4265 3745 698 -57 406 O
ATOM 199 CB ILE B 673 43.113 19.051 51.200 1.00 28.94 C
ANISOU 199 CB ILE B 673 3300 4249 3448 523 -183 478 C
ATOM 200 CG1 ILE B 673 42.995 20.443 51.866 1.00 29.99 C
ANISOU 200 CG1 ILE B 673 3432 4402 3561 348 -250 463 C
ATOM 201 CG2 ILE B 673 44.494 18.912 50.520 1.00 27.22 C
ANISOU 201 CG2 ILE B 673 2940 4237 3166 573 -182 540 C
ATOM 202 CD1 ILE B 673 42.955 21.630 50.896 1.00 37.80 C
ANISOU 202 CD1 ILE B 673 4457 5339 4567 194 -278 406 C
ATOM 203 H ILE B 673 40.737 18.597 52.057 1.00 26.33 H
ATOM 204 HA ILE B 673 41.774 19.537 49.562 1.00 26.57 H
ATOM 205 HB ILE B 673 43.101 18.330 52.018 1.00 28.94 H
ATOM 206 HG13 ILE B 673 43.813 20.581 52.573 1.00 29.99 H
ATOM 207 HG12 ILE B 673 42.091 20.489 52.465 1.00 29.99 H
ATOM 208 HG21 ILE B 673 45.287 19.261 51.179 1.00 27.22 H
ATOM 209 HG22 ILE B 673 44.733 17.877 50.272 1.00 27.22 H
ATOM 210 HG23 ILE B 673 44.550 19.495 49.602 1.00 27.22 H
ATOM 211 HD11 ILE B 673 43.023 22.571 51.440 1.00 37.80 H
ATOM 212 HD12 ILE B 673 43.778 21.605 50.183 1.00 37.80 H
ATOM 213 HD13 ILE B 673 42.020 21.656 50.337 1.00 37.80 H
ATOM 214 N LYS B 674 41.921 16.267 50.049 1.00 29.27 N
ANISOU 214 N LYS B 674 3614 3961 3546 794 -36 434 N
ATOM 215 CA LYS B 674 41.999 14.970 49.366 1.00 30.18 C
ANISOU 215 CA LYS B 674 3845 3997 3627 954 32 446 C
ATOM 216 C LYS B 674 41.054 14.869 48.156 1.00 35.07 C
ANISOU 216 C LYS B 674 4567 4467 4292 881 53 375 C
ATOM 217 O LYS B 674 41.495 14.458 47.086 1.00 34.65 O
ANISOU 217 O LYS B 674 4534 4420 4210 962 89 374 O
ATOM 218 CB LYS B 674 41.693 13.830 50.358 1.00 33.04 C
ANISOU 218 CB LYS B 674 4349 4249 3954 1055 66 473 C
ATOM 219 CG LYS B 674 42.836 13.507 51.329 1.00 50.01 C
ANISOU 219 CG LYS B 674 6413 6556 6032 1192 65 561 C
ATOM 220 CD LYS B 674 42.440 12.378 52.291 1.00 65.07 C
ANISOU 220 CD LYS B 674 8487 8325 7911 1271 97 579 C
ATOM 221 CE LYS B 674 43.590 11.948 53.211 1.00 74.98 C
ANISOU 221 CE LYS B 674 9729 9698 9063 1504 135 676 C
ATOM 222 NZ LYS B 674 43.187 10.846 54.102 1.00 84.23 N1+
ANISOU 222 NZ LYS B 674 11101 10704 10200 1581 171 691 N1+
ATOM 223 H LYS B 674 41.618 16.246 51.014 1.00 29.27 H
ATOM 224 HA LYS B 674 43.021 14.852 49.001 1.00 30.18 H
ATOM 225 HB3 LYS B 674 41.487 12.916 49.797 1.00 33.04 H
ATOM 226 HB2 LYS B 674 40.779 14.051 50.909 1.00 33.04 H
ATOM 227 HG3 LYS B 674 43.121 14.396 51.891 1.00 50.01 H
ATOM 228 HG2 LYS B 674 43.718 13.211 50.760 1.00 50.01 H

ATOM 229 HD3 LYS B 674 42.088 11.521 51.714 1.00 65.07 H
ATOM 230 HD2 LYS B 674 41.593 12.710 52.895 1.00 65.07 H
ATOM 231 HE3 LYS B 674 43.908 12.789 53.822 1.00 74.98 H
ATOM 232 HE2 LYS B 674 44.450 11.628 52.622 1.00 74.98 H
ATOM 233 HZ1 LYS B 674 42.908 10.049 53.549 1.00 84.23 H
ATOM 234 HZ2 LYS B 674 43.963 10.591 54.695 1.00 84.23 H
ATOM 235 HZ3 LYS B 674 42.410 11.147 54.674 1.00 84.23 H
ATOM 236 N MET B 675 39.789 15.270 48.365 1.00 33.21 N
ANISOU 236 N MET B 675 4382 4123 4114 731 29 323 N
ATOM 237 CA MET B 675 38.708 15.289 47.378 1.00 33.36 C
ANISOU 237 CA MET B 675 4477 4029 4169 628 35 264 C
ATOM 238 C MET B 675 38.993 16.210 46.175 1.00 36.83 C
ANISOU 238 C MET B 675 4822 4543 4629 593 25 239 C
ATOM 239 O MET B 675 38.709 15.824 45.041 1.00 36.22 O
ANISOU 239 O MET B 675 4817 4404 4539 615 55 215 O
ATOM 240 CB MET B 675 37.406 15.656 48.131 1.00 35.95 C
ANISOU 240 CB MET B 675 4807 4314 4538 488 5 242 C
ATOM 241 CG MET B 675 36.149 15.874 47.277 1.00 40.25 C
ANISOU 241 CG MET B 675 5434 4763 5096 380 13 205 C
ATOM 242 SD MET B 675 34.674 16.282 48.252 1.00 44.37 S
ANISOU 242 SD MET B 675 5909 5306 5643 255 -16 206 S
ATOM 243 CE MET B 675 33.452 16.369 46.919 1.00 40.97 C
ANISOU 243 CE MET B 675 5510 4847 5212 133 -13 178 C
ATOM 244 H MET B 675 39.531 15.586 49.290 1.00 33.21 H
ATOM 245 HA MET B 675 38.600 14.275 46.989 1.00 33.36 H
ATOM 246 HB3 MET B 675 37.573 16.565 48.711 1.00 35.95 H
ATOM 247 HB2 MET B 675 37.193 14.877 48.864 1.00 35.95 H
ATOM 248 HG3 MET B 675 35.944 14.986 46.679 1.00 40.25 H
ATOM 249 HG2 MET B 675 36.311 16.700 46.586 1.00 40.25 H
ATOM 250 HE1 MET B 675 32.523 16.801 47.287 1.00 40.97 H
ATOM 251 HE2 MET B 675 33.817 17.002 46.113 1.00 40.97 H
ATOM 252 HE3 MET B 675 33.247 15.377 46.517 1.00 40.97 H
ATOM 253 N ILE B 676 39.553 17.399 46.452 1.00 33.05 N
ANISOU 253 N ILE B 676 4203 4187 4167 533 -19 247 N
ATOM 254 CA ILE B 676 39.855 18.440 45.469 1.00 34.01 C
ANISOU 254 CA ILE B 676 4241 4380 4302 476 -35 227 C
ATOM 255 C ILE B 676 41.075 18.122 44.574 1.00 36.70 C
ANISOU 255 C ILE B 676 4519 4830 4595 585 -10 264 C
ATOM 256 O ILE B 676 41.013 18.392 43.374 1.00 35.57 O
ANISOU 256 O ILE B 676 4354 4702 4459 560 -3 241 O
ATOM 257 CB ILE B 676 40.013 19.824 46.180 1.00 38.01 C
ANISOU 257 CB ILE B 676 4670 4958 4815 362 -90 227 C
ATOM 258 CG1 ILE B 676 38.616 20.358 46.582 1.00 38.64 C
ANISOU 258 CG1 ILE B 676 4808 4941 4932 272 -99 183 C
ATOM 259 CG2 ILE B 676 40.786 20.899 45.389 1.00 40.04 C
ANISOU 259 CG2 ILE B 676 4826 5346 5042 314 -116 243 C
ATOM 260 CD1 ILE B 676 38.645 21.577 47.514 1.00 47.57 C
ANISOU 260 CD1 ILE B 676 5927 6105 6044 197 -140 187 C
ATOM 261 H ILE B 676 39.749 17.635 47.415 1.00 33.05 H
ATOM 262 HA ILE B 676 38.995 18.509 44.798 1.00 34.01 H
ATOM 263 HB ILE B 676 40.574 19.657 47.101 1.00 38.01 H
ATOM 264 HG13 ILE B 676 38.037 19.572 47.067 1.00 38.64 H
ATOM 265 HG12 ILE B 676 38.058 20.610 45.682 1.00 38.64 H
ATOM 266 HG21 ILE B 676 40.805 21.859 45.902 1.00 40.04 H
ATOM 267 HG22 ILE B 676 41.828 20.623 45.235 1.00 40.04 H
ATOM 268 HG23 ILE B 676 40.325 21.048 44.414 1.00 40.04 H

ATOM 269 HD11 ILE B 676 37.685 21.708 48.014 1.00 47.57 H
ATOM 270 HD12 ILE B 676 39.410 21.474 48.284 1.00 47.57 H
ATOM 271 HD13 ILE B 676 38.848 22.493 46.960 1.00 47.57 H
ATOM 272 N PHE B 677 42.143 17.554 45.161 1.00 33.53 N
ANISOU 272 N PHE B 677 4081 4522 4135 715 7 328 N
ATOM 273 CA PHE B 677 43.398 17.228 44.469 1.00 33.88 C
ANISOU 273 CA PHE B 677 4046 4715 4113 850 40 384 C
ATOM 274 C PHE B 677 43.483 15.784 43.933 1.00 39.92 C
ANISOU 274 C PHE B 677 4959 5383 4828 1049 120 395 C
ATOM 275 O PHE B 677 44.557 15.396 43.468 1.00 40.10 O
ANISOU 275 O PHE B 677 4925 5539 4771 1219 163 459 O
ATOM 276 CB PHE B 677 44.603 17.588 45.369 1.00 35.49 C
ANISOU 276 CB PHE B 677 4079 5155 4252 875 8 472 C
ATOM 277 CG PHE B 677 44.934 19.068 45.415 1.00 36.86 C
ANISOU 277 CG PHE B 677 4117 5460 4427 682 -63 474 C
ATOM 278 CD1 PHE B 677 45.653 19.663 44.355 1.00 39.38 C
ANISOU 278 CD1 PHE B 677 4348 5889 4726 639 -65 483 C
ATOM 279 CD2 PHE B 677 44.418 19.890 46.437 1.00 38.74 C
ANISOU 279 CD2 PHE B 677 4341 5709 4672 541 -125 471 C
ATOM 280 CE1 PHE B 677 45.917 21.026 44.373 1.00 39.83 C
ANISOU 280 CE1 PHE B 677 4315 6056 4764 443 -131 489 C
ATOM 281 CE2 PHE B 677 44.696 21.248 46.441 1.00 41.24 C
ANISOU 281 CE2 PHE B 677 4585 6121 4962 352 -189 473 C
ATOM 282 CZ PHE B 677 45.452 21.811 45.421 1.00 38.87 C
ANISOU 282 CZ PHE B 677 4209 5922 4637 297 -193 483 C
ATOM 283 H PHE B 677 42.119 17.357 46.153 1.00 33.53 H
ATOM 284 HA PHE B 677 43.480 17.848 43.574 1.00 33.88 H
ATOM 285 HB3 PHE B 677 45.511 17.085 45.033 1.00 35.49 H
ATOM 286 HB2 PHE B 677 44.437 17.228 46.385 1.00 35.49 H
ATOM 287 HD1 PHE B 677 46.012 19.058 43.534 1.00 39.38 H
ATOM 288 HD2 PHE B 677 43.824 19.468 47.232 1.00 38.74 H
ATOM 289 HE1 PHE B 677 46.485 21.475 43.571 1.00 39.83 H
ATOM 290 HE2 PHE B 677 44.326 21.866 47.244 1.00 41.24 H
ATOM 291 HZ PHE B 677 45.669 22.867 45.441 1.00 38.87 H
ATOM 292 N ASP B 678 42.376 15.018 43.983 1.00 37.79 N
ANISOU 292 N ASP B 678 4886 4887 4584 1025 142 339 N
ATOM 293 CA ASP B 678 42.307 13.623 43.533 1.00 37.93 C
ANISOU 293 CA ASP B 678 5116 4763 4534 1183 215 340 C
ATOM 294 C ASP B 678 42.551 13.515 42.012 1.00 42.54 C
ANISOU 294 C ASP B 678 5751 5328 5086 1240 258 316 C
ATOM 295 O ASP B 678 41.734 13.996 41.227 1.00 41.43 O
ANISOU 295 O ASP B 678 5640 5103 4997 1088 235 250 O
ATOM 296 CB ASP B 678 40.976 12.954 43.959 1.00 39.70 C
ANISOU 296 CB ASP B 678 5541 4763 4778 1081 212 292 C
ATOM 297 CG ASP B 678 40.978 11.424 43.820 1.00 53.54 C
ANISOU 297 CG ASP B 678 7562 6344 6435 1227 283 302 C
ATOM 298 OD1 ASP B 678 40.546 10.940 42.751 1.00 54.74 O
ANISOU 298 OD1 ASP B 678 7859 6405 6534 1284 328 277 O
ATOM 299 OD2 ASP B 678 41.569 10.774 44.710 1.00 61.97 O1-
ANISOU 299 OD2 ASP B 678 8721 7354 7471 1282 294 334 O1-
ATOM 300 H ASP B 678 41.526 15.401 44.371 1.00 37.79 H
ATOM 301 HA ASP B 678 43.116 13.098 44.047 1.00 37.93 H
ATOM 302 HB3 ASP B 678 40.141 13.382 43.403 1.00 39.70 H
ATOM 303 HB2 ASP B 678 40.756 13.202 44.996 1.00 39.70 H
ATOM 304 N VAL B 679 43.685 12.899 41.642 1.00 41.05 N
ANISOU 304 N VAL B 679 5563 5236 4799 1473 322 380 N

```
ATOM    305  CA  VAL B 679      44.170  12.766  40.265  1.00 41.49           C
ANISOU  305  CA  VAL B 679     5664   5302   4799   1579    376    374       C
ATOM    306  C   VAL B 679      43.294  11.848  39.384  1.00 45.90           C
ANISOU  306  C   VAL B 679     6531   5578   5330   1554    415    299       C
ATOM    307  O   VAL B 679      43.146  12.133  38.195  1.00 45.16           O
ANISOU  307  O   VAL B 679     6464   5446   5247   1487    417    251       O
ATOM    308  CB  VAL B 679      45.650  12.271  40.261  1.00 45.86           C
ANISOU  308  CB  VAL B 679     6141   6056   5229   1872    447    481       C
ATOM    309  CG1 VAL B 679      46.193  11.739  38.918  1.00 45.87           C
ANISOU  309  CG1 VAL B 679     6238   6041   5149   2026    522    480       C
ATOM    310  CG2 VAL B 679      46.588  13.375  40.785  1.00 45.92           C
ANISOU  310  CG2 VAL B 679     5816   6383   5250   1830    393    559       C
ATOM    311  H   VAL B 679      44.299  12.540  42.359  1.00 41.05           H
ATOM    312  HA  VAL B 679      44.140  13.760  39.814  1.00 41.49           H
ATOM    313  HB  VAL B 679      45.721  11.441  40.966  1.00 45.86           H
ATOM    314 HG11 VAL B 679      47.257  11.509  38.987  1.00 45.87           H
ATOM    315 HG12 VAL B 679      45.696  10.818  38.613  1.00 45.87           H
ATOM    316 HG13 VAL B 679      46.067  12.476  38.126  1.00 45.87           H
ATOM    317 HG21 VAL B 679      47.619  13.027  40.840  1.00 45.92           H
ATOM    318 HG22 VAL B 679      46.570  14.250  40.133  1.00 45.92           H
ATOM    319 HG23 VAL B 679      46.305  13.703  41.785  1.00 45.92           H
ATOM    320  N   GLU B 680      42.708  10.793  39.975  1.00 42.48           N
ANISOU  320  N   GLU B 680     6337   4952   4851   1588    440    291       N
ATOM    321  CA  GLU B 680      41.827   9.850  39.281  1.00 42.46           C
ANISOU  321  CA  GLU B 680     6659   4678   4795   1524    468    227       C
ATOM    322  C   GLU B 680      40.435  10.430  38.967  1.00 44.55           C
ANISOU  322  C   GLU B 680     6894   4876   5158   1214    393    149       C
ATOM    323  O   GLU B 680      39.819   9.986  37.999  1.00 44.54           O
ANISOU  323  O   GLU B 680     7060   4744   5120   1131    402     97       O
ATOM    324  CB  GLU B 680      41.728   8.531  40.080  1.00 44.30           C
ANISOU  324  CB  GLU B 680     7175   4723   4934   1617    512    247       C
ATOM    325  CG  GLU B 680      43.084   7.833  40.351  1.00 58.50           C
ANISOU  325  CG  GLU B 680     9083   6552   6592   1975    611    326       C
ATOM    326  CD  GLU B 680      43.888   7.462  39.092  1.00 83.30           C
ANISOU  326  CD  GLU B 680    12321   9700   9630   2177    690    334       C
ATOM    327  OE1 GLU B 680      43.281   6.921  38.140  1.00 78.06           O
ANISOU  327  OE1 GLU B 680    11913   8825   8919   2088    707    264       O
ATOM    328  OE2 GLU B 680      45.110   7.731  39.093  1.00 76.72           O1-
ANISOU  328  OE2 GLU B 680    11301   9099   8748   2422    736    418       O1-
ATOM    329  H   GLU B 680      42.850  10.626  40.962  1.00 42.48           H
ATOM    330  HA  GLU B 680      42.279   9.627  38.314  1.00 42.46           H
ATOM    331  HB3 GLU B 680      41.061   7.838  39.564  1.00 44.30           H
ATOM    332  HB2 GLU B 680      41.249   8.729  41.039  1.00 44.30           H
ATOM    333  HG3 GLU B 680      42.903   6.914  40.909  1.00 58.50           H
ATOM    334  HG2 GLU B 680      43.691   8.460  41.008  1.00 58.50           H
ATOM    335  N   SER B 681      39.985  11.432  39.742  1.00 38.53           N
ANISOU  335  N   SER B 681     5917   4217   4505   1055    321    149       N
ATOM    336  CA  SER B 681      38.757  12.187  39.488  1.00 37.64           C
ANISOU  336  CA  SER B 681     5738   4088   4475    801    256     95       C
ATOM    337  C   SER B 681      38.893  13.136  38.283  1.00 39.61           C
ANISOU  337  C   SER B 681     5855   4434   4760    766    244     68       C
ATOM    338  O   SER B 681      37.924  13.285  37.539  1.00 40.08           O
ANISOU  338  O   SER B 681     5959   4434   4834    608    219     21       O
ATOM    339  CB  SER B 681      38.336  12.958  40.753  1.00 41.18           C
ANISOU  339  CB  SER B 681     6009   4630   5009    695    198    111       C
ATOM    340  OG  SER B 681      37.841  12.066  41.729  1.00 49.31           O
```

```
ANISOU  340  OG  SER B 681     7169   5564   6003    712    208    134       O
ATOM    341  H   SER B 681      40.526  11.715  40.549  1.00 38.53           H
ATOM    342  HA  SER B 681      37.963  11.478  39.246  1.00 37.64           H
ATOM    343  HB3 SER B 681      37.537  13.666  40.525  1.00 41.18           H
ATOM    344  HB2 SER B 681      39.161  13.538  41.165  1.00 41.18           H
ATOM    345  HG  SER B 681      38.585  11.571  42.092  1.00 49.31           H
ATOM    346  N   MET B 682      40.093  13.718  38.086  1.00 33.87           N
ANISOU  346  N   MET B 682     4962   3872   4034    905    260    108       N
ATOM    347  CA  MET B 682      40.446  14.534  36.917  1.00 33.12           C
ANISOU  347  CA  MET B 682     4743   3881   3962    884    254     93       C
ATOM    348  C   MET B 682      40.466  13.710  35.616  1.00 37.55           C
ANISOU  348  C   MET B 682     5508   4315   4443    945    308     60       C
ATOM    349  O   MET B 682      39.985  14.198  34.594  1.00 36.60           O
ANISOU  349  O   MET B 682     5383   4175   4348    823    286     14       O
ATOM    350  CB  MET B 682      41.811  15.222  37.126  1.00 34.97           C
ANISOU  350  CB  MET B 682     4764   4337   4184   1010    262    160       C
ATOM    351  CG  MET B 682      41.899  16.150  38.344  1.00 37.83           C
ANISOU  351  CG  MET B 682     4934   4828   4611    908    198    186       C
ATOM    352  SD  MET B 682      43.554  16.856  38.584  1.00 41.44           S
ANISOU  352  SD  MET B 682     5152   5576   5019   1013    198    279       S
ATOM    353  CE  MET B 682      43.420  17.377  40.313  1.00 38.54           C
ANISOU  353  CE  MET B 682     4690   5275   4679    940    140    316       C
ATOM    354  H   MET B 682      40.838  13.545  38.747  1.00 33.87           H
ATOM    355  HA  MET B 682      39.688  15.312  36.806  1.00 33.12           H
ATOM    356  HB3 MET B 682      42.063  15.796  36.234  1.00 34.97           H
ATOM    357  HB2 MET B 682      42.586  14.465  37.219  1.00 34.97           H
ATOM    358  HG3 MET B 682      41.631  15.606  39.247  1.00 37.83           H
ATOM    359  HG2 MET B 682      41.183  16.967  38.249  1.00 37.83           H
ATOM    360  HE1 MET B 682      44.326  17.898  40.623  1.00 38.54           H
ATOM    361  HE2 MET B 682      42.570  18.046  40.447  1.00 38.54           H
ATOM    362  HE3 MET B 682      43.282  16.511  40.960  1.00 38.54           H
ATOM    363  N   LYS B 683      40.981  12.469  35.692  1.00 35.02           N
ANISOU  363  N   LYS B 683     5390   3901   4013   1141    381     85       N
ATOM    364  CA  LYS B 683      40.970  11.491  34.602  1.00 35.10           C
ANISOU  364  CA  LYS B 683     5667   3755   3915   1225    443     55       C
ATOM    365  C   LYS B 683      39.550  11.011  34.254  1.00 39.79           C
ANISOU  365  C   LYS B 683     6473   4144   4502    990    407    -17       C
ATOM    366  O   LYS B 683      39.239  10.894  33.071  1.00 38.85           O
ANISOU  366  O   LYS B 683     6446   3965   4351    919    409    -62       O
ATOM    367  CB  LYS B 683      41.860  10.289  34.970  1.00 37.53           C
ANISOU  367  CB  LYS B 683     6183   3989   4087   1506    535    105       C
ATOM    368  CG  LYS B 683      43.362  10.609  35.040  1.00 40.87           C
ANISOU  368  CG  LYS B 683     6410   4649   4469   1771    588    192       C
ATOM    369  CD  LYS B 683      44.161   9.408  35.561  1.00 42.86           C
ANISOU  369  CD  LYS B 683     6866   4843   4573   2077    684    257       C
ATOM    370  CE  LYS B 683      45.673   9.656  35.615  1.00 58.67           C
ANISOU  370  CE  LYS B 683     8629   7142   6521   2344    733    367       C
ATOM    371  NZ  LYS B 683      46.380   8.504  36.201  1.00 71.02          N1+
ANISOU  371  NZ  LYS B 683    10352   8686   7947   2651    819    448      N1+
ATOM    372  H   LYS B 683      41.370  12.151  36.569  1.00 35.02           H
ATOM    373  HA  LYS B 683      41.387  11.973  33.715  1.00 35.10           H
ATOM    374  HB3 LYS B 683      41.723   9.495  34.233  1.00 37.53           H
ATOM    375  HB2 LYS B 683      41.530   9.870  35.921  1.00 37.53           H
ATOM    376  HG3 LYS B 683      43.536  11.468  35.688  1.00 40.87           H
ATOM    377  HG2 LYS B 683      43.726  10.895  34.053  1.00 40.87           H
ATOM    378  HD3 LYS B 683      43.950   8.537  34.938  1.00 42.86           H
```

```
ATOM    379  HD2 LYS B 683      43.799   9.164  36.559  1.00 42.86           H
ATOM    380  HE3 LYS B 683      45.888  10.541  36.214  1.00 58.67           H
ATOM    381  HE2 LYS B 683      46.064   9.844  34.614  1.00 58.67           H
ATOM    382  HZ1 LYS B 683      46.022   8.329  37.133  1.00 71.02           H
ATOM    383  HZ2 LYS B 683      46.220   7.683  35.634  1.00 71.02           H
ATOM    384  HZ3 LYS B 683      47.370   8.693  36.252  1.00 71.02           H
ATOM    385  N   LYS B 684      38.713  10.774  35.282  1.00 37.53           N
ANISOU  385  N   LYS B 684     6243   3777   4239    856    368    -20       N
ATOM    386  CA  LYS B 684      37.311  10.365  35.150  1.00 37.53           C
ANISOU  386  CA  LYS B 684     6402   3637   4222    599    323    -65       C
ATOM    387  C   LYS B 684      36.440  11.431  34.459  1.00 41.24           C
ANISOU  387  C   LYS B 684     6669   4220   4780    391    255    -97       C
ATOM    388  O   LYS B 684      35.576  11.063  33.665  1.00 41.06           O
ANISOU  388  O   LYS B 684     6782   4113   4705    224    234   -134       O
ATOM    389  H   LYS B 684      39.056  10.872  36.229  1.00 37.53           H
ATOM    390  CB  LYS B 684      36.759   9.973  36.539  1.00  0.00           C
ATOM    391  CG  LYS B 684      35.317   9.422  36.530  1.00  0.00           C
ATOM    392  CD  LYS B 684      34.836   8.914  37.904  1.00  0.00           C
ATOM    393  CE  LYS B 684      34.606  10.001  38.974  1.00  0.00           C
ATOM    394  NZ  LYS B 684      33.462  10.877  38.659  1.00  0.00           N1+
ATOM    395  HB2 LYS B 684      37.411   9.210  36.967  1.00  0.00           H
ATOM    396  HB3 LYS B 684      36.818  10.831  37.207  1.00  0.00           H
ATOM    397  HG2 LYS B 684      34.623  10.176  36.158  1.00  0.00           H
ATOM    398  HG3 LYS B 684      35.264   8.599  35.817  1.00  0.00           H
ATOM    399  HD2 LYS B 684      33.922   8.334  37.769  1.00  0.00           H
ATOM    400  HD3 LYS B 684      35.569   8.203  38.288  1.00  0.00           H
ATOM    401  HE2 LYS B 684      34.400   9.525  39.933  1.00  0.00           H
ATOM    402  HE3 LYS B 684      35.499  10.610  39.118  1.00  0.00           H
ATOM    403  HZ1 LYS B 684      33.645  11.392  37.810  1.00  0.00           H
ATOM    404  HZ2 LYS B 684      32.630  10.316  38.541  1.00  0.00           H
ATOM    405  HZ3 LYS B 684      33.318  11.522  39.426  1.00  0.00           H
ATOM    406  HA  LYS B 684      37.297   9.473  34.521  1.00 37.53           H
ATOM    407  N   ALA B 685      36.706  12.718  34.745  1.00 36.72           N
ANISOU  407  N   ALA B 685     5794   3837   4323    402    222    -77       N
ATOM    408  CA  ALA B 685      36.052  13.863  34.111  1.00 36.69           C
ANISOU  408  CA  ALA B 685     5607   3943   4392    252    169    -98       C
ATOM    409  C   ALA B 685      36.381  13.993  32.613  1.00 41.99           C
ANISOU  409  C   ALA B 685     6318   4610   5026    278    190   -129       C
ATOM    410  O   ALA B 685      35.474  14.282  31.835  1.00 41.51           O
ANISOU  410  O   ALA B 685     6258   4551   4963    120    156   -157       O
ATOM    411  CB  ALA B 685      36.414  15.147  34.873  1.00 37.24           C
ANISOU  411  CB  ALA B 685     5410   4179   4561    285    141    -69       C
ATOM    412  H   ALA B 685      37.423  12.936  35.425  1.00 36.72           H
ATOM    413  HA  ALA B 685      34.973  13.717  34.200  1.00 36.69           H
ATOM    414  HB1 ALA B 685      35.955  16.023  34.414  1.00 37.24           H
ATOM    415  HB2 ALA B 685      36.061  15.098  35.903  1.00 37.24           H
ATOM    416  HB3 ALA B 685      37.492  15.311  34.899  1.00 37.24           H
ATOM    417  N   MET B 686      37.645  13.733  32.232  1.00 39.73           N
ANISOU  417  N   MET B 686     6065   4332   4698    483    250   -115       N
ATOM    418  CA  MET B 686      38.105  13.709  30.839  1.00 41.00           C
ANISOU  418  CA  MET B 686     6274   4494   4812    543    284   -137       C
ATOM    419  C   MET B 686      37.515  12.535  30.034  1.00 46.10           C
ANISOU  419  C   MET B 686     7233   4942   5340    479    305   -182       C
ATOM    420  O   MET B 686      37.103  12.748  28.895  1.00 45.45           O
ANISOU  420  O   MET B 686     7176   4855   5238    393    294   -217       O
ATOM    421  CB  MET B 686      39.645  13.701  30.792  1.00 43.62           C
```

ANISOU 421 CB MET B 686 6532 4929 5114 794 347 -91 C
ATOM 422 CG MET B 686 40.289 15.021 31.246 1.00 47.91 C
ANISOU 422 CG MET B 686 6765 5689 5751 785 313 -57 C
ATOM 423 SD MET B 686 42.090 14.954 31.455 1.00 53.37 S
ANISOU 423 SD MET B 686 7321 6564 6392 1043 372 28 S
ATOM 424 CE MET B 686 42.622 14.682 29.744 1.00 50.07 C
ANISOU 424 CE MET B 686 6995 6153 5878 1187 444 22 C
ATOM 425 H MET B 686 38.337 13.503 32.933 1.00 39.73 H
ATOM 426 HA MET B 686 37.761 14.627 30.358 1.00 41.00 H
ATOM 427 HB3 MET B 686 39.967 13.501 29.769 1.00 43.62 H
ATOM 428 HB2 MET B 686 40.036 12.881 31.396 1.00 43.62 H
ATOM 429 HG3 MET B 686 39.864 15.340 32.197 1.00 47.91 H
ATOM 430 HG2 MET B 686 40.055 15.811 30.532 1.00 47.91 H
ATOM 431 HE1 MET B 686 43.710 14.707 29.681 1.00 50.07 H
ATOM 432 HE2 MET B 686 42.282 13.714 29.378 1.00 50.07 H
ATOM 433 HE3 MET B 686 42.217 15.462 29.100 1.00 50.07 H
ATOM 434 N VAL B 687 37.434 11.341 30.650 1.00 43.48 N
ANISOU 434 N VAL B 687 7157 4442 4921 503 333 -180 N
ATOM 435 CA VAL B 687 36.780 10.149 30.092 1.00 43.89 C
ANISOU 435 CA VAL B 687 7573 4271 4832 407 348 -220 C
ATOM 436 C VAL B 687 35.258 10.333 29.897 1.00 48.20 C
ANISOU 436 C VAL B 687 8090 4827 5396 78 261 -249 C
ATOM 437 O VAL B 687 34.710 9.797 28.934 1.00 48.17 O
ANISOU 437 O VAL B 687 8280 4727 5296 -53 252 -287 O
ATOM 438 CB VAL B 687 37.059 8.895 30.981 1.00 47.73 C
ANISOU 438 CB VAL B 687 8345 4576 5215 508 397 -201 C
ATOM 439 CG1 VAL B 687 36.136 7.675 30.761 1.00 47.69 C
ANISOU 439 CG1 VAL B 687 8725 4332 5064 306 386 -240 C
ATOM 440 CG2 VAL B 687 38.522 8.441 30.833 1.00 47.51 C
ANISOU 440 CG2 VAL B 687 8412 4529 5111 860 500 -167 C
ATOM 441 H VAL B 687 37.814 11.240 31.582 1.00 43.48 H
ATOM 442 HA VAL B 687 37.211 9.974 29.104 1.00 43.89 H
ATOM 443 HB VAL B 687 36.930 9.194 32.022 1.00 47.73 H
ATOM 444 HG11 VAL B 687 36.471 6.820 31.349 1.00 47.69 H
ATOM 445 HG12 VAL B 687 35.108 7.875 31.065 1.00 47.69 H
ATOM 446 HG13 VAL B 687 36.128 7.370 29.713 1.00 47.69 H
ATOM 447 HG21 VAL B 687 38.751 7.612 31.505 1.00 47.51 H
ATOM 448 HG22 VAL B 687 38.733 8.112 29.815 1.00 47.51 H
ATOM 449 HG23 VAL B 687 39.217 9.244 31.067 1.00 47.51 H
ATOM 450 N GLU B 688 34.616 11.117 30.780 1.00 45.40 N
ANISOU 450 N GLU B 688 7485 4612 5153 -44 200 -223 N
ATOM 451 CA GLU B 688 33.203 11.492 30.706 1.00 45.31 C
ANISOU 451 CA GLU B 688 7371 4682 5161 -318 122 -224 C
ATOM 452 C GLU B 688 32.891 12.498 29.574 1.00 46.42 C
ANISOU 452 C GLU B 688 7354 4944 5338 -362 98 -243 C
ATOM 453 O GLU B 688 31.779 12.470 29.047 1.00 45.79 O
ANISOU 453 O GLU B 688 7293 4896 5209 -575 48 -251 O
ATOM 454 CB GLU B 688 32.752 11.939 32.114 1.00 47.03 C
ANISOU 454 CB GLU B 688 7351 5034 5483 -355 83 -181 C
ATOM 455 CG GLU B 688 31.293 12.437 32.237 1.00 61.04 C
ANISOU 455 CG GLU B 688 9175 6811 7208 -602 30 -159 C
ATOM 456 CD GLU B 688 30.825 12.743 33.671 1.00 93.71 C
ANISOU 456 CD GLU B 688 13108 11066 11432 -600 7 -113 C
ATOM 457 OE1 GLU B 688 29.696 13.269 33.782 1.00 94.99 O
ANISOU 457 OE1 GLU B 688 13056 11398 11636 -706 -37 -85 O
ATOM 458 OE2 GLU B 688 31.582 12.492 34.638 1.00 92.40 O1-

ANISOU 458 OE2 GLU B 688 12999 10828 11282 -475 39 -101 O1-
ATOM 459 H GLU B 688 35.131 11.499 31.562 1.00 45.40 H
ATOM 460 HA GLU B 688 32.641 10.587 30.468 1.00 45.31 H
ATOM 461 HB3 GLU B 688 33.426 12.712 32.483 1.00 47.03 H
ATOM 462 HB2 GLU B 688 32.885 11.086 32.781 1.00 47.03 H
ATOM 463 HG3 GLU B 688 30.619 11.691 31.814 1.00 61.04 H
ATOM 464 HG2 GLU B 688 31.163 13.343 31.645 1.00 61.04 H
ATOM 465 N TYR B 689 33.890 13.304 29.167 1.00 41.16 N
ANISOU 465 N TYR B 689 6536 4360 4744 -168 132 -243 N
ATOM 466 CA TYR B 689 33.866 14.113 27.941 1.00 40.73 C
ANISOU 466 CA TYR B 689 6345 4409 4721 -177 121 -258 C
ATOM 467 C TYR B 689 34.271 13.321 26.676 1.00 45.86 C
ANISOU 467 C TYR B 689 7224 4940 5261 -131 164 -298 C
ATOM 468 O TYR B 689 34.249 13.896 25.588 1.00 46.96 O
ANISOU 468 O TYR B 689 7284 5149 5411 -128 162 -314 O
ATOM 469 CB TYR B 689 34.770 15.356 28.130 1.00 41.10 C
ANISOU 469 CB TYR B 689 6143 4596 4877 -19 135 -233 C
ATOM 470 CG TYR B 689 34.236 16.411 29.085 1.00 42.23 C
ANISOU 470 CG TYR B 689 6066 4863 5115 -92 86 -203 C
ATOM 471 CD1 TYR B 689 32.968 16.989 28.857 1.00 43.61 C
ANISOU 471 CD1 TYR B 689 6154 5116 5300 -255 35 -199 C
ATOM 472 CD2 TYR B 689 35.016 16.858 30.173 1.00 42.65 C
ANISOU 472 CD2 TYR B 689 5998 4974 5235 17 95 -172 C
ATOM 473 CE1 TYR B 689 32.475 17.986 29.718 1.00 43.58 C
ANISOU 473 CE1 TYR B 689 5970 5225 5363 -283 3 -165 C
ATOM 474 CE2 TYR B 689 34.520 17.851 31.041 1.00 43.45 C
ANISOU 474 CE2 TYR B 689 5934 5170 5406 -39 55 -147 C
ATOM 475 CZ TYR B 689 33.248 18.413 30.814 1.00 49.36 C
ANISOU 475 CZ TYR B 689 6619 5978 6159 -175 15 -144 C
ATOM 476 OH TYR B 689 32.763 19.376 31.647 1.00 51.62 O
ANISOU 476 OH TYR B 689 6766 6354 6492 -195 -11 -114 O
ATOM 477 H TYR B 689 34.770 13.284 29.663 1.00 41.16 H
ATOM 478 HA TYR B 689 32.843 14.448 27.766 1.00 40.73 H
ATOM 479 HB3 TYR B 689 34.902 15.865 27.176 1.00 41.10 H
ATOM 480 HB2 TYR B 689 35.771 15.050 28.437 1.00 41.10 H
ATOM 481 HD1 TYR B 689 32.370 16.678 28.012 1.00 43.61 H
ATOM 482 HD2 TYR B 689 35.998 16.443 30.346 1.00 42.65 H
ATOM 483 HE1 TYR B 689 31.504 18.421 29.532 1.00 43.58 H
ATOM 484 HE2 TYR B 689 35.118 18.184 31.876 1.00 43.45 H
ATOM 485 HH TYR B 689 31.938 19.757 31.339 1.00 51.62 H
ATOM 486 N GLU B 690 34.630 12.033 26.837 1.00 41.94 N
ANISOU 486 N GLU B 690 7035 4252 4648 -87 207 -313 N
ATOM 487 CA GLU B 690 35.100 11.097 25.808 1.00 41.99 C
ANISOU 487 CA GLU B 690 7366 4082 4507 -14 264 -349 C
ATOM 488 C GLU B 690 36.407 11.534 25.118 1.00 45.90 C
ANISOU 488 C GLU B 690 7801 4633 5007 257 339 -344 C
ATOM 489 O GLU B 690 36.618 11.223 23.948 1.00 45.62 O
ANISOU 489 O GLU B 690 7944 4519 4872 299 376 -376 O
ATOM 490 CB GLU B 690 33.971 10.688 24.829 1.00 43.31 C
ANISOU 490 CB GLU B 690 7704 4179 4573 -278 213 -389 C
ATOM 491 CG GLU B 690 32.775 10.018 25.540 1.00 54.60 C
ANISOU 491 CG GLU B 690 9405 5452 5889 -483 183 -393 C
ATOM 492 CD GLU B 690 31.807 9.335 24.565 1.00 74.55 C
ANISOU 492 CD GLU B 690 12116 7925 8286 -797 120 -420 C
ATOM 493 OE1 GLU B 690 30.700 9.883 24.365 1.00 75.31 O
ANISOU 493 OE1 GLU B 690 11967 8211 8438 -1006 40 -397 O

```
ATOM    494  OE2 GLU B 690      32.174   8.260  24.039  1.00 58.52           O1-
ANISOU  494  OE2 GLU B 690    10488   5668   6081   -836    150   -457       O1-
ATOM    495  H   GLU B 690      34.591  11.642  27.768  1.00 41.94           H
ATOM    496  HA  GLU B 690      35.382  10.195  26.350  1.00 41.99           H
ATOM    497  HB3 GLU B 690      34.370  10.007  24.076  1.00 43.31           H
ATOM    498  HB2 GLU B 690      33.618  11.560  24.277  1.00 43.31           H
ATOM    499  HG3 GLU B 690      32.241  10.753  26.143  1.00 54.60           H
ATOM    500  HG2 GLU B 690      33.138   9.264  26.241  1.00 54.60           H
ATOM    501  N   ILE B 691      37.277  12.221  25.878  1.00 41.92           N
ANISOU  501  N   ILE B 691     7063   4267   4598    439    363   -296       N
ATOM    502  CA  ILE B 691      38.647  12.559  25.493  1.00 41.75           C
ANISOU  502  CA  ILE B 691     6929   4356   4576    693    431   -265       C
ATOM    503  C   ILE B 691      39.529  11.300  25.614  1.00 47.50           C
ANISOU  503  C   ILE B 691     7944   4944   5159    943    530   -249       C
ATOM    504  O   ILE B 691      39.360  10.531  26.561  1.00 47.43           O
ANISOU  504  O   ILE B 691     8097   4817   5109    967    542   -238       O
ATOM    505  CB  ILE B 691      39.212  13.696  26.397  1.00 44.25           C
ANISOU  505  CB  ILE B 691     6891   4892   5030    738    403   -211       C
ATOM    506  CG1 ILE B 691      38.385  14.994  26.215  1.00 44.05           C
ANISOU  506  CG1 ILE B 691     6634   4983   5118    528    322   -227       C
ATOM    507  CG2 ILE B 691      40.714  13.992  26.194  1.00 44.61           C
ANISOU  507  CG2 ILE B 691     6810   5089   5051    994    472   -154       C
ATOM    508  CD1 ILE B 691      38.660  16.073  27.272  1.00 48.72           C
ANISOU  508  CD1 ILE B 691     6973   5712   5826    488    276   -189       C
ATOM    509  H   ILE B 691      37.029  12.434  26.835  1.00 41.92           H
ATOM    510  HA  ILE B 691      38.646  12.914  24.463  1.00 41.75           H
ATOM    511  HB  ILE B 691      39.103  13.375  27.432  1.00 44.25           H
ATOM    512 HG13 ILE B 691      37.318  14.776  26.240  1.00 44.05           H
ATOM    513 HG12 ILE B 691      38.573  15.406  25.222  1.00 44.05           H
ATOM    514 HG21 ILE B 691      41.035  14.801  26.848  1.00 44.61           H
ATOM    515 HG22 ILE B 691      41.350  13.141  26.436  1.00 44.61           H
ATOM    516 HG23 ILE B 691      40.927  14.291  25.168  1.00 44.61           H
ATOM    517 HD11 ILE B 691      37.801  16.737  27.381  1.00 48.72           H
ATOM    518 HD12 ILE B 691      38.865  15.636  28.250  1.00 48.72           H
ATOM    519 HD13 ILE B 691      39.515  16.687  26.991  1.00 48.72           H
ATOM    520  N   ASP B 692      40.440  11.112  24.648  1.00 45.27           N
ANISOU  520  N   ASP B 692     7743   4672   4785   1140    606   -244       N
ATOM    521  CA  ASP B 692      41.346   9.968  24.571  1.00 46.10           C
ANISOU  521  CA  ASP B 692     8134   4655   4725   1431    717   -219       C
ATOM    522  C   ASP B 692      42.573  10.243  25.461  1.00 52.30           C
ANISOU  522  C   ASP B 692     8699   5636   5539   1691    766   -127       C
ATOM    523  O   ASP B 692      43.425  11.049  25.086  1.00 53.31           O
ANISOU  523  O   ASP B 692     8565   5995   5695   1822    791    -75       O
ATOM    524  CB  ASP B 692      41.722   9.680  23.094  1.00 47.98           C
ANISOU  524  CB  ASP B 692     8524   4861   4846   1560    786   -240       C
ATOM    525  CG  ASP B 692      42.356   8.310  22.786  1.00 58.23           C
ANISOU  525  CG  ASP B 692    10244   5952   5929   1828    904   -236       C
ATOM    526  OD1 ASP B 692      42.870   7.648  23.713  1.00 60.05           O
ANISOU  526  OD1 ASP B 692    10531   6182   6103   2067    969   -175       O
ATOM    527  OD2 ASP B 692      42.380   7.974  21.583  1.00 62.35           O1-
ANISOU  527  OD2 ASP B 692    11055   6311   6324   1811    934   -290       O1-
ATOM    528  H   ASP B 692      40.540  11.802  23.913  1.00 45.27           H
ATOM    529  HA  ASP B 692      40.820   9.091  24.956  1.00 46.10           H
ATOM    530  HB3 ASP B 692      42.384  10.462  22.718  1.00 47.98           H
ATOM    531  HB2 ASP B 692      40.825   9.776  22.486  1.00 47.98           H
ATOM    532  N   LEU B 693      42.634   9.560  26.618  1.00 48.72           N
```

ANISOU 532 N LEU B 693 8347 5102 5063 1754 778 -100 N
ATOM 533 CA LEU B 693 43.729 9.666 27.588 1.00 48.84 C
ANISOU 533 CA LEU B 693 8157 5308 5091 1986 817 -5 C
ATOM 534 C LEU B 693 45.001 8.893 27.184 1.00 53.21 C
ANISOU 534 C LEU B 693 8826 5917 5476 2385 949 68 C
ATOM 535 O LEU B 693 46.037 9.133 27.801 1.00 52.17 O
ANISOU 535 O LEU B 693 8440 6037 5343 2587 982 167 O
ATOM 536 CB LEU B 693 43.250 9.264 29.006 1.00 49.04 C
ANISOU 536 CB LEU B 693 8247 5236 5149 1917 783 2 C
ATOM 537 CG LEU B 693 42.508 10.388 29.768 1.00 53.82 C
ANISOU 537 CG LEU B 693 8523 5985 5941 1664 671 -1 C
ATOM 538 CD1 LEU B 693 41.111 10.680 29.191 1.00 54.27 C
ANISOU 538 CD1 LEU B 693 8649 5905 6065 1328 585 -85 C
ATOM 539 CD2 LEU B 693 42.449 10.103 31.283 1.00 56.55 C
ANISOU 539 CD2 LEU B 693 8834 6349 6304 1728 667 49 C
ATOM 540 H LEU B 693 41.887 8.922 26.854 1.00 48.72 H
ATOM 541 HA LEU B 693 44.031 10.712 27.632 1.00 48.84 H
ATOM 542 HB3 LEU B 693 44.128 9.008 29.600 1.00 49.04 H
ATOM 543 HB2 LEU B 693 42.650 8.353 28.971 1.00 49.04 H
ATOM 544 HG LEU B 693 43.098 11.300 29.660 1.00 53.82 H
ATOM 545 HD11 LEU B 693 40.394 10.971 29.956 1.00 54.27 H
ATOM 546 HD12 LEU B 693 41.154 11.498 28.472 1.00 54.27 H
ATOM 547 HD13 LEU B 693 40.700 9.808 28.683 1.00 54.27 H
ATOM 548 HD21 LEU B 693 42.851 10.947 31.844 1.00 56.55 H
ATOM 549 HD22 LEU B 693 41.433 9.937 31.639 1.00 56.55 H
ATOM 550 HD23 LEU B 693 43.025 9.223 31.566 1.00 56.55 H
ATOM 551 N GLN B 694 44.938 8.015 26.166 1.00 50.65 N
ANISOU 551 N GLN B 694 8878 5376 4990 2499 1027 27 N
ATOM 552 CA GLN B 694 46.119 7.349 25.608 1.00 50.88 C
ANISOU 552 CA GLN B 694 9051 5449 4833 2908 1167 97 C
ATOM 553 C GLN B 694 46.905 8.308 24.692 1.00 55.62 C
ANISOU 553 C GLN B 694 9333 6340 5462 2984 1184 140 C
ATOM 554 O GLN B 694 48.134 8.324 24.762 1.00 56.45 O
ANISOU 554 O GLN B 694 9309 6670 5469 3313 1276 248 O
ATOM 555 H GLN B 694 44.063 7.858 25.681 1.00 50.65 H
ATOM 556 CB GLN B 694 45.693 6.034 24.910 1.00 0.00 C
ATOM 557 CG GLN B 694 46.839 5.079 24.501 1.00 0.00 C
ATOM 558 CD GLN B 694 47.508 5.397 23.158 1.00 0.00 C
ATOM 559 OE1 GLN B 694 46.874 5.894 22.230 1.00 0.00 O
ATOM 560 NE2 GLN B 694 48.800 5.082 23.041 1.00 0.00 N
ATOM 561 HB2 GLN B 694 45.056 5.485 25.606 1.00 0.00 H
ATOM 562 HB3 GLN B 694 45.057 6.241 24.050 1.00 0.00 H
ATOM 563 HG2 GLN B 694 47.586 5.033 25.294 1.00 0.00 H
ATOM 564 HG3 GLN B 694 46.434 4.071 24.409 1.00 0.00 H
ATOM 565 HE22 GLN B 694 49.285 5.264 22.175 1.00 0.00 H
ATOM 566 HE21 GLN B 694 49.299 4.671 23.815 1.00 0.00 H
ATOM 567 HA GLN B 694 46.778 7.073 26.435 1.00 50.88 H
ATOM 568 N LYS B 695 46.185 9.102 23.879 1.00 51.72 N
ANISOU 568 N LYS B 695 8701 5860 5092 2681 1094 68 N
ATOM 569 CA LYS B 695 46.759 10.090 22.961 1.00 51.68 C
ANISOU 569 CA LYS B 695 8404 6107 5126 2690 1093 98 C
ATOM 570 C LYS B 695 47.084 11.430 23.645 1.00 56.69 C
ANISOU 570 C LYS B 695 8568 7040 5931 2535 1004 152 C
ATOM 571 O LYS B 695 48.084 12.049 23.283 1.00 56.46 O
ANISOU 571 O LYS B 695 8257 7307 5890 2656 1032 238 O
ATOM 572 CB LYS B 695 45.803 10.308 21.773 1.00 53.90 C

ANISOU 572 CB LYS B 695 8825 6228 5427 2458 1049 -7 C
ATOM 573 CG LYS B 695 45.675 9.069 20.874 1.00 61.39 C
ANISOU 573 CG LYS B 695 10222 6932 6174 2632 1148 -49 C
ATOM 574 CD LYS B 695 44.711 9.292 19.701 1.00 72.25 C
ANISOU 574 CD LYS B 695 11761 8124 7566 2339 1082 -160 C
ATOM 575 CE LYS B 695 44.431 7.992 18.927 1.00 86.26 C
ANISOU 575 CE LYS B 695 14023 9635 9119 2489 1176 -206 C
ATOM 576 NZ LYS B 695 43.271 8.133 18.032 1.00 95.77 N1+
ANISOU 576 NZ LYS B 695 15418 10648 10322 2162 1098 -313 N1+
ATOM 577 H LYS B 695 45.177 9.020 23.876 1.00 51.72 H
ATOM 578 HA LYS B 695 47.696 9.693 22.567 1.00 51.68 H
ATOM 579 HB3 LYS B 695 46.162 11.137 21.161 1.00 53.90 H
ATOM 580 HB2 LYS B 695 44.820 10.606 22.142 1.00 53.90 H
ATOM 581 HG3 LYS B 695 45.325 8.225 21.470 1.00 61.39 H
ATOM 582 HG2 LYS B 695 46.656 8.784 20.493 1.00 61.39 H
ATOM 583 HD3 LYS B 695 45.138 10.033 19.024 1.00 72.25 H
ATOM 584 HD2 LYS B 695 43.784 9.730 20.074 1.00 72.25 H
ATOM 585 HE3 LYS B 695 44.215 7.177 19.618 1.00 86.26 H
ATOM 586 HE2 LYS B 695 45.308 7.695 18.351 1.00 86.26 H
ATOM 587 HZ1 LYS B 695 43.440 8.870 17.363 1.00 95.77 H
ATOM 588 HZ2 LYS B 695 43.110 7.262 17.547 1.00 95.77 H
ATOM 589 HZ3 LYS B 695 42.457 8.360 18.588 1.00 95.77 H
ATOM 590 N MET B 696 46.254 11.844 24.615 1.00 53.82 N
ANISOU 590 N MET B 696 8134 6604 5710 2264 899 105 N
ATOM 591 CA MET B 696 46.436 13.066 25.394 1.00 53.75 C
ANISOU 591 CA MET B 696 7743 6833 5846 2107 814 148 C
ATOM 592 C MET B 696 46.053 12.791 26.860 1.00 56.90 C
ANISOU 592 C MET B 696 8120 7199 6300 2064 774 166 C
ATOM 593 O MET B 696 44.930 13.112 27.253 1.00 57.21 O
ANISOU 593 O MET B 696 8173 7114 6451 1811 688 101 O
ATOM 594 CB MET B 696 45.639 14.234 24.772 1.00 56.05 C
ANISOU 594 CB MET B 696 7888 7138 6272 1800 719 83 C
ATOM 595 CG MET B 696 45.954 15.606 25.403 1.00 59.84 C
ANISOU 595 CG MET B 696 7995 7896 6844 1701 660 146 C
ATOM 596 SD MET B 696 44.596 16.802 25.327 1.00 64.15 S
ANISOU 596 SD MET B 696 8423 8389 7561 1333 533 73 S
ATOM 597 CE MET B 696 43.591 16.195 26.700 1.00 61.14 C
ANISOU 597 CE MET B 696 8126 7871 7233 1266 492 54 C
ATOM 598 H MET B 696 45.436 11.294 24.843 1.00 53.82 H
ATOM 599 HA MET B 696 47.479 13.379 25.360 1.00 53.75 H
ATOM 600 HB3 MET B 696 44.569 14.027 24.806 1.00 56.05 H
ATOM 601 HB2 MET B 696 45.893 14.297 23.714 1.00 56.05 H
ATOM 602 HG3 MET B 696 46.816 16.043 24.897 1.00 59.84 H
ATOM 603 HG2 MET B 696 46.247 15.513 26.448 1.00 59.84 H
ATOM 604 HE1 MET B 696 42.707 16.818 26.832 1.00 61.14 H
ATOM 605 HE2 MET B 696 43.275 15.171 26.507 1.00 61.14 H
ATOM 606 HE3 MET B 696 44.168 16.204 27.622 1.00 61.14 H
ATOM 607 N PRO B 697 46.986 12.225 27.662 1.00 52.64 N
ANISOU 607 N PRO B 697 7520 6805 5677 2321 836 265 N
ATOM 608 CA PRO B 697 46.802 12.123 29.122 1.00 51.99 C
ANISOU 608 CA PRO B 697 7395 6717 5642 2292 798 291 C
ATOM 609 C PRO B 697 46.769 13.490 29.835 1.00 53.87 C
ANISOU 609 C PRO B 697 7285 7159 6024 2059 693 312 C
ATOM 610 O PRO B 697 47.015 14.530 29.222 1.00 54.10 O
ANISOU 610 O PRO B 697 7096 7365 6093 1961 661 326 O
ATOM 611 CB PRO B 697 47.989 11.254 29.577 1.00 53.90 C

ANISOU	611	CB	PRO	B	697	7656	7095	5728	2664	905	406	C
ATOM	612	CG	PRO	B	697	49.066	11.494	28.535	1.00	58.66		C
ANISOU	612	CG	PRO	B	697	8126	7922	6241	2843	971	472	C
ATOM	613	CD	PRO	B	697	48.270	11.649	27.246	1.00	54.37		C
ANISOU	613	CD	PRO	B	697	7692	7223	5741	2664	948	369	C
ATOM	614	HA	PRO	B	697	45.867	11.605	29.342	1.00	51.99		H
ATOM	615	HB3	PRO	B	697	47.696	10.203	29.564	1.00	53.90		H
ATOM	616	HB2	PRO	B	697	48.342	11.472	30.586	1.00	53.90		H
ATOM	617	HG3	PRO	B	697	49.815	10.703	28.490	1.00	58.66		H
ATOM	618	HG2	PRO	B	697	49.575	12.429	28.766	1.00	58.66		H
ATOM	619	HD2	PRO	B	697	48.829	12.248	26.531	1.00	54.37		H
ATOM	620	HD3	PRO	B	697	48.082	10.675	26.792	1.00	54.37		H
ATOM	621	N	LEU	B	698	46.462	13.440	31.140	1.00	48.23		N
ANISOU	621	N	LEU	B	698	6545	6406	5374	1969	641	313	N
ATOM	622	CA	LEU	B	698	46.327	14.576	32.058	1.00	47.20		C
ANISOU	622	CA	LEU	B	698	6148	6430	5358	1761	545	331	C
ATOM	623	C	LEU	B	698	47.554	15.519	32.123	1.00	49.66		C
ANISOU	623	C	LEU	B	698	6143	7086	5638	1796	537	430	C
ATOM	624	O	LEU	B	698	47.368	16.712	32.364	1.00	49.15		O
ANISOU	624	O	LEU	B	698	5906	7114	5653	1573	463	416	O
ATOM	625	CB	LEU	B	698	45.955	13.977	33.435	1.00	46.87		C
ANISOU	625	CB	LEU	B	698	6148	6340	5321	1798	533	356	C
ATOM	626	CG	LEU	B	698	45.631	14.956	34.582	1.00	50.80		C
ANISOU	626	CG	LEU	B	698	6599	6759	5943	1547	439	307	C
ATOM	627	CD1	LEU	B	698	44.469	15.909	34.260	1.00	50.62		C
ANISOU	627	CD1	LEU	B	698	6631	6584	6017	1302	382	209	C
ATOM	628	CD2	LEU	B	698	45.339	14.167	35.869	1.00	52.72		C
ANISOU	628	CD2	LEU	B	698	6982	6886	6162	1628	454	320	C
ATOM	629	H	LEU	B	698	46.272	12.538	31.550	1.00	48.23		H
ATOM	630	HA	LEU	B	698	45.485	15.167	31.695	1.00	47.20		H
ATOM	631	HB3	LEU	B	698	46.770	13.325	33.754	1.00	46.87		H
ATOM	632	HB2	LEU	B	698	45.090	13.323	33.305	1.00	46.87		H
ATOM	633	HG	LEU	B	698	46.517	15.562	34.771	1.00	50.80		H
ATOM	634	HD11	LEU	B	698	44.223	16.522	35.128	1.00	50.62		H
ATOM	635	HD12	LEU	B	698	44.720	16.592	33.448	1.00	50.62		H
ATOM	636	HD13	LEU	B	698	43.571	15.360	33.978	1.00	50.62		H
ATOM	637	HD21	LEU	B	698	45.531	14.767	36.758	1.00	52.72		H
ATOM	638	HD22	LEU	B	698	44.299	13.846	35.905	1.00	52.72		H
ATOM	639	HD23	LEU	B	698	45.953	13.270	35.938	1.00	52.72		H
ATOM	640	N	GLY	B	699	48.766	14.988	31.874	1.00	45.27		N
ANISOU	640	N	GLY	B	699	5528	6723	4951	2073	617	537	N
ATOM	641	CA	GLY	B	699	50.015	15.754	31.865	1.00	44.75		C
ANISOU	641	CA	GLY	B	699	5150	7036	4818	2122	617	659	C
ATOM	642	C	GLY	B	699	50.358	16.342	30.484	1.00	48.07		C
ANISOU	642	C	GLY	B	699	5468	7570	5228	2060	623	658	C
ATOM	643	O	GLY	B	699	51.246	17.190	30.412	1.00	49.05		O
ANISOU	643	O	GLY	B	699	5315	7997	5324	1976	589	744	O
ATOM	644	H	GLY	B	699	48.833	14.002	31.670	1.00	45.27		H
ATOM	645	HA3	GLY	B	699	50.828	15.099	32.178	1.00	44.75		H
ATOM	646	HA2	GLY	B	699	49.972	16.568	32.589	1.00	44.75		H
ATOM	647	N	LYS	B	700	49.676	15.917	29.404	1.00	42.98		N
ANISOU	647	N	LYS	B	700	5051	6688	4590	2088	665	567	N
ATOM	648	CA	LYS	B	700	49.811	16.468	28.049	1.00	41.99		C
ANISOU	648	CA	LYS	B	700	4871	6628	4454	2040	677	553	C
ATOM	649	C	LYS	B	700	48.765	17.556	27.738	1.00	43.03		C
ANISOU	649	C	LYS	B	700	4985	6634	4731	1700	578	453	C

```
ATOM    650  O   LYS B 700      48.913  18.245  26.729  1.00 41.65           O
ANISOU  650  O   LYS B 700     4741   6529   4555   1628    575    446       O
ATOM    651  CB  LYS B 700      49.739  15.321  27.016  1.00 45.11           C
ANISOU  651  CB  LYS B 700     5530   6865   4745   2287    787    524       C
ATOM    652  CG  LYS B 700      50.967  14.388  26.993  1.00 62.20           C
ANISOU  652  CG  LYS B 700     7688   9218   6727   2677    907    648       C
ATOM    653  CD  LYS B 700      52.279  15.049  26.525  1.00 75.14           C
ANISOU  653  CD  LYS B 700     9012  11262   8274   2768    938    778       C
ATOM    654  CE  LYS B 700      53.252  15.372  27.674  1.00 89.09           C
ANISOU  654  CE  LYS B 700    10497  13384   9969   2868    935    931       C
ATOM    655  NZ  LYS B 700      54.481  16.020  27.184  1.00 98.31           N1+
ANISOU  655  NZ  LYS B 700    11350  14988  11016   2960    970   1078       N1+
ATOM    656  H   LYS B 700      48.945  15.228  29.523  1.00 42.98           H
ATOM    657  HA  LYS B 700      50.778  16.961  27.945  1.00 41.99           H
ATOM    658  HB3 LYS B 700      49.623  15.732  26.012  1.00 45.11           H
ATOM    659  HB2 LYS B 700      48.838  14.733  27.192  1.00 45.11           H
ATOM    660  HG3 LYS B 700      50.748  13.559  26.320  1.00 62.20           H
ATOM    661  HG2 LYS B 700      51.109  13.935  27.974  1.00 62.20           H
ATOM    662  HD3 LYS B 700      52.049  15.963  25.974  1.00 75.14           H
ATOM    663  HD2 LYS B 700      52.767  14.388  25.807  1.00 75.14           H
ATOM    664  HE3 LYS B 700      53.529  14.456  28.199  1.00 89.09           H
ATOM    665  HE2 LYS B 700      52.787  16.028  28.408  1.00 89.09           H
ATOM    666  HZ1 LYS B 700      54.245  16.888  26.724  1.00 98.31           H
ATOM    667  HZ2 LYS B 700      55.095  16.211  27.962  1.00 98.31           H
ATOM    668  HZ3 LYS B 700      54.949  15.410  26.529  1.00 98.31           H
ATOM    669  N   LEU B 701      47.749  17.707  28.606  1.00 38.60           N
ANISOU  669  N   LEU B 701     4483   5902   4280   1512    502    385       N
ATOM    670  CA  LEU B 701      46.711  18.734  28.532  1.00 37.82           C
ANISOU  670  CA  LEU B 701     4374   5692   4304   1225    414    304       C
ATOM    671  C   LEU B 701      47.311  20.100  28.922  1.00 39.97           C
ANISOU  671  C   LEU B 701     4391   6213   4584   1070    355    370       C
ATOM    672  O   LEU B 701      47.774  20.245  30.053  1.00 39.45           O
ANISOU  672  O   LEU B 701     4177   6329   4483   1077    334    450       O
ATOM    673  CB  LEU B 701      45.553  18.280  29.457  1.00 37.82           C
ANISOU  673  CB  LEU B 701     4486   5494   4388   1115    364    243       C
ATOM    674  CG  LEU B 701      44.173  18.995  29.361  1.00 42.19           C
ANISOU  674  CG  LEU B 701     5159   5833   5041    913    310    141       C
ATOM    675  CD1 LEU B 701      43.758  19.683  30.676  1.00 42.06           C
ANISOU  675  CD1 LEU B 701     5092   5784   5104    754    237    128       C
ATOM    676  CD2 LEU B 701      43.974  19.923  28.147  1.00 43.78           C
ANISOU  676  CD2 LEU B 701     5341   6033   5258    813    299    103       C
ATOM    677  H   LEU B 701      47.706  17.103  29.414  1.00 38.60           H
ATOM    678  HA  LEU B 701      46.365  18.762  27.498  1.00 37.82           H
ATOM    679  HB3 LEU B 701      45.901  18.266  30.490  1.00 37.82           H
ATOM    680  HB2 LEU B 701      45.369  17.226  29.244  1.00 37.82           H
ATOM    681  HG  LEU B 701      43.444  18.196  29.217  1.00 42.19           H
ATOM    682 HD11 LEU B 701      43.315  20.660  30.494  1.00 42.06           H
ATOM    683 HD12 LEU B 701      43.009  19.090  31.202  1.00 42.06           H
ATOM    684 HD13 LEU B 701      44.594  19.827  31.360  1.00 42.06           H
ATOM    685 HD21 LEU B 701      42.919  20.176  28.027  1.00 43.78           H
ATOM    686 HD22 LEU B 701      44.518  20.860  28.263  1.00 43.78           H
ATOM    687 HD23 LEU B 701      44.289  19.458  27.213  1.00 43.78           H
ATOM    688  N   SER B 702      47.330  21.046  27.966  1.00 34.30           N
ANISOU  688  N   SER B 702     3632   5512   3888    933    332    343       N
ATOM    689  CA  SER B 702      47.999  22.345  28.100  1.00 33.01           C
ANISOU  689  CA  SER B 702     3272   5558   3713    755    274    403       C
```

ATOM	690	C	SER	B	702	47.039	23.522	27.857	1.00	34.95	C
ANISOU	690	C	SER	B	702	3579 5654 4047	514 204 325				C
ATOM	691	O	SER	B	702	45.988	23.363	27.237	1.00	33.56	O
ANISOU	691	O	SER	B	702	3549 5276 3927	506 212 240				O
ATOM	692	CB	SER	B	702	49.234	22.374	27.170	1.00	34.44	C
ANISOU	692	CB	SER	B	702	3299 6002 3787	843 322 493				C
ATOM	693	OG	SER	B	702	48.900	22.520	25.803	1.00	35.46	O
ANISOU	693	OG	SER	B	702	3462 6079 3933	755 322 449				O
ATOM	694	H	SER	B	702	46.915	20.852	27.065	1.00	34.30	H
ATOM	695	HA	SER	B	702	48.365	22.456	29.123	1.00	33.01	H
ATOM	696	HB3	SER	B	702	49.830	21.469	27.297	1.00	34.44	H
ATOM	697	HB2	SER	B	702	49.881	23.208	27.442	1.00	34.44	H
ATOM	698	HG	SER	B	702	48.546	21.684	25.488	1.00	35.46	H
ATOM	699	N	LYS	B	703	47.445	24.693	28.372	1.00	30.98	N
ANISOU	699	N	LYS	B	703	2974 5261 3537	321 137 363				N
ATOM	700	CA	LYS	B	703	46.684	25.941	28.351	1.00	30.08	C
ANISOU	700	CA	LYS	B	703	2927 5030 3473	104 74 312				C
ATOM	701	C	LYS	B	703	46.550	26.572	26.951	1.00	32.53	C
ANISOU	701	C	LYS	B	703	3256 5327 3775	68 92 289				C
ATOM	702	O	LYS	B	703	45.474	27.073	26.630	1.00	32.03	O
ANISOU	702	O	LYS	B	703	3320 5074 3774	19 81 212				O
ATOM	703	CB	LYS	B	703	47.326	26.897	29.376	1.00	32.80	C
ANISOU	703	CB	LYS	B	703	3171 5533 3760	-83 9 380				C
ATOM	704	CG	LYS	B	703	46.572	28.215	29.605	1.00	57.03	C
ANISOU	704	CG	LYS	B	703	6369 8427 6872	-258 -54 326				C
ATOM	705	CD	LYS	B	703	47.109	28.964	30.839	1.00	65.90	C
ANISOU	705	CD	LYS	B	703	7429 9684 7925	-409 -115 391				C
ATOM	706	CE	LYS	B	703	46.404	30.302	31.119	1.00	79.90	C
ANISOU	706	CE	LYS	B	703	9352 11318 9688	-619 -175 355				C
ATOM	707	NZ	LYS	B	703	46.722	31.326	30.106	1.00	89.51	N1+
ANISOU	707	NZ	LYS	B	703	10575 12608 10827	-796 -199 385				N1+
ATOM	708	H	LYS	B	703	48.316	24.725	28.883	1.00	30.98	H
ATOM	709	HA	LYS	B	703	45.676	25.710	28.701	1.00	30.08	H
ATOM	710	HB3	LYS	B	703	48.358	27.111	29.096	1.00	32.80	H
ATOM	711	HB2	LYS	B	703	47.380	26.375	30.331	1.00	32.80	H
ATOM	712	HG3	LYS	B	703	45.509	28.009	29.739	1.00	57.03	H
ATOM	713	HG2	LYS	B	703	46.655	28.841	28.717	1.00	57.03	H
ATOM	714	HD3	LYS	B	703	48.184	29.121	30.736	1.00	65.90	H
ATOM	715	HD2	LYS	B	703	46.991	28.325	31.715	1.00	65.90	H
ATOM	716	HE3	LYS	B	703	46.715	30.683	32.092	1.00	79.90	H
ATOM	717	HE2	LYS	B	703	45.324	30.159	31.163	1.00	79.90	H
ATOM	718	HZ1	LYS	B	703	46.425	31.003	29.195	1.00	89.51	H
ATOM	719	HZ2	LYS	B	703	46.241	32.185	30.329	1.00	89.51	H
ATOM	720	HZ3	LYS	B	703	47.718	31.491	30.093	1.00	89.51	H
ATOM	721	N	ARG	B	704	47.621	26.516	26.137	1.00	28.52	N
ANISOU	721	N	ARG	B	704	2612 5042 3182	102 123 365				N
ATOM	722	CA	ARG	B	704	47.634	27.034	24.763	1.00	27.06	C
ANISOU	722	CA	ARG	B	704	2430 4875 2976	75 144 356				C
ATOM	723	C	ARG	B	704	46.850	26.150	23.775	1.00	28.17	C
ANISOU	723	C	ARG	B	704	2701 4843 3159	239 203 279				C
ATOM	724	O	ARG	B	704	46.326	26.680	22.796	1.00	25.99	O
ANISOU	724	O	ARG	B	704	2494 4476 2904	182 201 231				O
ATOM	725	CB	ARG	B	704	49.082	27.242	24.278	1.00	27.22	C
ANISOU	725	CB	ARG	B	704	2254 5208 2880	68 164 471				C
ATOM	726	CG	ARG	B	704	49.864	28.284	25.097	1.00	37.94	C
ANISOU	726	CG	ARG	B	704	3518 6720 4178	-191 87 540				C

```
ATOM    727  CD  ARG B 704      51.270  28.540  24.527  1.00 48.21           C
ANISOU  727  CD  ARG B 704     4604   8366   5347   -228    100    668       C
ATOM    728  NE  ARG B 704      51.935  29.687  25.169  1.00 56.88           N
ANISOU  728  NE  ARG B 704     5604   9652   6357   -495     21    752       N
ATOM    729  CZ  ARG B 704      51.846  30.985  24.815  1.00 72.92           C
ANISOU  729  CZ  ARG B 704     7696  11659   8349   -769    -38    753       C
ATOM    730  NH1 ARG B 704      52.530  31.896  25.520  1.00 62.25           N
ANISOU  730  NH1 ARG B 704     6282  10478   6894  -1030   -114    834       N
ATOM    731  NH2 ARG B 704      51.094  31.397  23.781  1.00 56.93           N1+
ANISOU  731  NH2 ARG B 704     5816   9438   6376   -790    -24    677       N1+
ATOM    732  H   ARG B 704      48.469  26.073  26.460  1.00 28.52           H
ATOM    733  HA  ARG B 704      47.147  28.011  24.769  1.00 27.06           H
ATOM    734  HB3 ARG B 704      49.058  27.578  23.240  1.00 27.22           H
ATOM    735  HB2 ARG B 704      49.618  26.292  24.276  1.00 27.22           H
ATOM    736  HG3 ARG B 704      49.982  27.862  26.096  1.00 37.94           H
ATOM    737  HG2 ARG B 704      49.304  29.212  25.228  1.00 37.94           H
ATOM    738  HD3 ARG B 704      51.289  28.575  23.437  1.00 48.21           H
ATOM    739  HD2 ARG B 704      51.897  27.692  24.805  1.00 48.21           H
ATOM    740  HE  ARG B 704      52.494  29.458  25.978  1.00 56.88           H
ATOM    741 HH12 ARG B 704      52.481  32.874  25.275  1.00 62.25           H
ATOM    742 HH11 ARG B 704      53.107  31.613  26.298  1.00 62.25           H
ATOM    743 HH22 ARG B 704      51.043  32.374  23.535  1.00 56.93           H
ATOM    744 HH21 ARG B 704      50.570  30.723  23.243  1.00 56.93           H
ATOM    745  N   GLN B 705      46.749  24.841  24.063  1.00 25.90           N
ANISOU  745  N   GLN B 705     2473   4495   2874    428    250    265       N
ATOM    746  CA  GLN B 705      45.909  23.884  23.341  1.00 26.04           C
ANISOU  746  CA  GLN B 705     2660   4323   2910    552    298    189       C
ATOM    747  C   GLN B 705      44.406  24.169  23.524  1.00 28.88           C
ANISOU  747  C   GLN B 705     3152   4455   3364    414    245     96       C
ATOM    748  O   GLN B 705      43.654  24.067  22.555  1.00 27.16           O
ANISOU  748  O   GLN B 705     3018   4143   3161    382    248     44       O
ATOM    749  CB  GLN B 705      46.308  22.461  23.784  1.00 27.45           C
ANISOU  749  CB  GLN B 705     2911   4472   3048    767    357    201       C
ATOM    750  CG  GLN B 705      45.443  21.309  23.236  1.00 29.68           C
ANISOU  750  CG  GLN B 705     3423   4529   3327    865    400    120       C
ATOM    751  CD  GLN B 705      45.902  19.944  23.754  1.00 46.71           C
ANISOU  751  CD  GLN B 705     5707   6600   5440   1042    448    125       C
ATOM    752  NE2 GLN B 705      45.743  18.903  22.936  1.00 39.41           N
ANISOU  752  NE2 GLN B 705     5005   5506   4461   1151    501     74       N
ATOM    753  OE1 GLN B 705      46.367  19.821  24.887  1.00 42.33           O
ANISOU  753  OE1 GLN B 705     5076   6120   4888   1078    437    174       O
ATOM    754  H   GLN B 705      47.218  24.481  24.883  1.00 25.90           H
ATOM    755  HA  GLN B 705      46.133  23.979  22.276  1.00 26.04           H
ATOM    756  HB3 GLN B 705      46.290  22.421  24.872  1.00 27.45           H
ATOM    757  HB2 GLN B 705      47.345  22.287  23.492  1.00 27.45           H
ATOM    758  HG3 GLN B 705      45.450  21.318  22.145  1.00 29.68           H
ATOM    759  HG2 GLN B 705      44.408  21.433  23.550  1.00 29.68           H
ATOM    760 HE22 GLN B 705      46.011  17.980  23.244  1.00 39.41           H
ATOM    761 HE21 GLN B 705      45.337  19.019  22.015  1.00 39.41           H
ATOM    762  N   ILE B 706      44.012  24.552  24.751  1.00 25.38           N
ANISOU  762  N   ILE B 706     2714   3955   2973    335    197     88       N
ATOM    763  CA  ILE B 706      42.652  24.965  25.106  1.00 24.59           C
ANISOU  763  CA  ILE B 706     2712   3685   2946    227    152     24       C
ATOM    764  C   ILE B 706      42.258  26.308  24.448  1.00 29.68           C
ANISOU  764  C   ILE B 706     3349   4326   3602     93    119     11       C
ATOM    765  O   ILE B 706      41.130  26.424  23.972  1.00 28.60           O
```

ANISOU 765 O ILE B 706 3294 4079 3493 64 111 -39 O
ATOM 766 CB ILE B 706 42.473 25.035 26.655 1.00 26.98 C
ANISOU 766 CB ILE B 706 3008 3960 3284 191 116 34 C
ATOM 767 CG1 ILE B 706 42.554 23.624 27.284 1.00 27.71 C
ANISOU 767 CG1 ILE B 706 3141 4026 3362 334 152 42 C
ATOM 768 CG2 ILE B 706 41.194 25.757 27.138 1.00 24.77 C
ANISOU 768 CG2 ILE B 706 2806 3543 3061 90 74 -15 C
ATOM 769 CD1 ILE B 706 42.869 23.639 28.786 1.00 29.49 C
ANISOU 769 CD1 ILE B 706 3310 4298 3596 328 126 81 C
ATOM 770 H ILE B 706 44.694 24.596 25.495 1.00 25.38 H
ATOM 771 HA ILE B 706 41.968 24.204 24.725 1.00 24.59 H
ATOM 772 HB ILE B 706 43.316 25.603 27.049 1.00 26.98 H
ATOM 773 HG13 ILE B 706 43.318 23.024 26.790 1.00 27.71 H
ATOM 774 HG12 ILE B 706 41.615 23.094 27.115 1.00 27.71 H
ATOM 775 HG21 ILE B 706 41.066 25.687 28.217 1.00 24.77 H
ATOM 776 HG22 ILE B 706 41.223 26.820 26.905 1.00 24.77 H
ATOM 777 HG23 ILE B 706 40.303 25.334 26.678 1.00 24.77 H
ATOM 778 HD11 ILE B 706 43.189 22.658 29.131 1.00 29.49 H
ATOM 779 HD12 ILE B 706 43.664 24.345 29.028 1.00 29.49 H
ATOM 780 HD13 ILE B 706 41.989 23.915 29.364 1.00 29.49 H
ATOM 781 N GLN B 707 43.203 27.265 24.381 1.00 27.93 N
ANISOU 781 N GLN B 707 3033 4235 3343 8 99 65 N
ATOM 782 CA GLN B 707 43.052 28.553 23.690 1.00 28.06 C
ANISOU 782 CA GLN B 707 3067 4249 3347 -122 72 64 C
ATOM 783 C GLN B 707 42.898 28.436 22.164 1.00 30.59 C
ANISOU 783 C GLN B 707 3410 4557 3654 -82 105 40 C
ATOM 784 O GLN B 707 42.119 29.190 21.580 1.00 30.66 O
ANISOU 784 O GLN B 707 3491 4480 3678 -141 89 7 O
ATOM 785 CB GLN B 707 44.208 29.497 24.067 1.00 29.81 C
ANISOU 785 CB GLN B 707 3194 4631 3500 -242 46 138 C
ATOM 786 CG GLN B 707 44.083 30.051 25.498 1.00 55.79 C
ANISOU 786 CG GLN B 707 6515 7891 6790 -349 -6 151 C
ATOM 787 CD GLN B 707 45.276 30.930 25.868 1.00 81.86 C
ANISOU 787 CD GLN B 707 9747 11354 10002 -514 -42 227 C
ATOM 788 NE2 GLN B 707 45.107 32.251 25.774 1.00 77.34 N
ANISOU 788 NE2 GLN B 707 9292 10698 9394 -674 -80 219 N
ATOM 789 OE1 GLN B 707 46.335 30.426 26.239 1.00 77.39 O
ANISOU 789 OE1 GLN B 707 9029 10996 9380 -502 -36 299 O
ATOM 790 H GLN B 707 44.103 27.098 24.811 1.00 27.93 H
ATOM 791 HA GLN B 707 42.133 29.015 24.046 1.00 28.06 H
ATOM 792 HB3 GLN B 707 44.234 30.342 23.376 1.00 29.81 H
ATOM 793 HB2 GLN B 707 45.160 28.979 23.944 1.00 29.81 H
ATOM 794 HG3 GLN B 707 44.019 29.236 26.218 1.00 55.79 H
ATOM 795 HG2 GLN B 707 43.158 30.620 25.600 1.00 55.79 H
ATOM 796 HE22 GLN B 707 45.865 32.880 25.989 1.00 77.34 H
ATOM 797 HE21 GLN B 707 44.231 32.634 25.438 1.00 77.34 H
ATOM 798 N ALA B 708 43.608 27.472 21.555 1.00 26.78 N
ANISOU 798 N ALA B 708 2875 4168 3132 35 156 60 N
ATOM 799 CA ALA B 708 43.494 27.133 20.137 1.00 26.61 C
ANISOU 799 CA ALA B 708 2888 4138 3085 92 195 36 C
ATOM 800 C ALA B 708 42.178 26.406 19.798 1.00 28.77 C
ANISOU 800 C ALA B 708 3300 4232 3401 121 194 -42 C
ATOM 801 O ALA B 708 41.677 26.570 18.687 1.00 28.94 O
ANISOU 801 O ALA B 708 3370 4208 3417 89 193 -73 O
ATOM 802 CB ALA B 708 44.715 26.296 19.729 1.00 27.44 C
ANISOU 802 CB ALA B 708 2927 4380 3119 244 260 82 C

```
ATOM    803  H   ALA B 708      44.248  26.907  22.098  1.00 26.78           H
ATOM    804  HA  ALA B 708      43.518  28.060  19.562  1.00 26.61           H
ATOM    805  HB1 ALA B 708      44.674  26.020  18.675  1.00 27.44           H
ATOM    806  HB2 ALA B 708      45.638  26.856  19.882  1.00 27.44           H
ATOM    807  HB3 ALA B 708      44.789  25.379  20.314  1.00 27.44           H
ATOM    808  N   ALA B 709      41.633  25.640  20.759  1.00 24.06           N
ANISOU  808  N   ALA B 709     2760   3547   2833    166    191    -66       N
ATOM    809  CA  ALA B 709      40.358  24.935  20.637  1.00 23.53           C
ANISOU  809  CA  ALA B 709     2817   3335   2788    157    181   -126       C
ATOM    810  C   ALA B 709      39.151  25.878  20.764  1.00 25.39           C
ANISOU  810  C   ALA B 709     3057   3526   3065     40    130   -143       C
ATOM    811  O   ALA B 709      38.187  25.705  20.019  1.00 22.92           O
ANISOU  811  O   ALA B 709     2800   3165   2742      8    121   -175       O
ATOM    812  CB  ALA B 709      40.314  23.805  21.672  1.00 23.83           C
ANISOU  812  CB  ALA B 709     2914   3306   2835    216    188   -134       C
ATOM    813  H   ALA B 709      42.115  25.536  21.641  1.00 24.06           H
ATOM    814  HA  ALA B 709      40.310  24.478  19.647  1.00 23.53           H
ATOM    815  HB1 ALA B 709      39.393  23.228  21.596  1.00 23.83           H
ATOM    816  HB2 ALA B 709      41.142  23.111  21.526  1.00 23.83           H
ATOM    817  HB3 ALA B 709      40.387  24.194  22.687  1.00 23.83           H
ATOM    818  N   TYR B 710      39.252  26.894  21.644  1.00 21.90           N
ANISOU  818  N   TYR B 710     2562   3109   2650    -18     99   -114       N
ATOM    819  CA  TYR B 710      38.316  28.020  21.736  1.00 21.66           C
ANISOU  819  CA  TYR B 710     2554   3041   2635    -89     64   -115       C
ATOM    820  C   TYR B 710      38.259  28.837  20.431  1.00 25.70           C
ANISOU  820  C   TYR B 710     3072   3579   3114   -118     69   -114       C
ATOM    821  O   TYR B 710      37.167  29.233  20.026  1.00 25.65           O
ANISOU  821  O   TYR B 710     3102   3541   3103   -131     58   -127       O
ATOM    822  CB  TYR B 710      38.725  28.961  22.890  1.00 22.57           C
ANISOU  822  CB  TYR B 710     2654   3168   2755   -139     40    -82       C
ATOM    823  CG  TYR B 710      38.320  28.608  24.311  1.00 23.70           C
ANISOU  823  CG  TYR B 710     2810   3266   2930   -127     23    -83       C
ATOM    824  CD1 TYR B 710      36.975  28.333  24.644  1.00 25.91           C
ANISOU  824  CD1 TYR B 710     3129   3486   3228   -113     13   -101       C
ATOM    825  CD2 TYR B 710      39.284  28.674  25.337  1.00 24.26           C
ANISOU  825  CD2 TYR B 710     2843   3376   2999   -140     14    -55       C
ATOM    826  CE1 TYR B 710      36.602  28.145  25.991  1.00 25.93           C
ANISOU  826  CE1 TYR B 710     3141   3458   3254   -105      0    -95       C
ATOM    827  CE2 TYR B 710      38.916  28.476  26.677  1.00 25.42           C
ANISOU  827  CE2 TYR B 710     3008   3480   3172   -132     -2    -55       C
ATOM    828  CZ  TYR B 710      37.575  28.224  27.007  1.00 30.52           C
ANISOU  828  CZ  TYR B 710     3701   4054   3841   -111     -7    -77       C
ATOM    829  OH  TYR B 710      37.243  28.075  28.319  1.00 27.32           O
ANISOU  829  OH  TYR B 710     3308   3617   3455   -101    -19    -72       O
ATOM    830  H   TYR B 710      40.062  26.942  22.249  1.00 21.90           H
ATOM    831  HA  TYR B 710      37.320  27.614  21.923  1.00 21.66           H
ATOM    832  HB3 TYR B 710      38.264  29.930  22.729  1.00 22.57           H
ATOM    833  HB2 TYR B 710      39.797  29.155  22.849  1.00 22.57           H
ATOM    834  HD1 TYR B 710      36.217  28.300  23.876  1.00 25.91           H
ATOM    835  HD2 TYR B 710      40.309  28.911  25.112  1.00 24.26           H
ATOM    836  HE1 TYR B 710      35.568  27.952  26.242  1.00 25.93           H
ATOM    837  HE2 TYR B 710      39.660  28.545  27.458  1.00 25.42           H
ATOM    838  HH  TYR B 710      36.297  27.968  28.483  1.00 27.32           H
ATOM    839  N   SER B 711      39.429  29.056  19.802  1.00 22.97           N
ANISOU  839  N   SER B 711     2680   3312   2736   -125     89    -89       N
ATOM    840  CA  SER B 711      39.578  29.800  18.548  1.00 23.52           C
```

```
ANISOU  840  CA  SER B 711     2753   3416   2767   -155     98    -82        C
ATOM    841  C   SER B 711      38.908  29.110  17.344  1.00 26.79           C
ANISOU  841  C   SER B 711     3206   3800   3173   -111    115   -122        C
ATOM    842  O   SER B 711      38.383  29.813  16.482  1.00 26.15           O
ANISOU  842  O   SER B 711     3155   3706   3075   -139    105   -127        O
ATOM    843  CB  SER B 711      41.071  30.057  18.267  1.00 26.60           C
ANISOU  843  CB  SER B 711     3065   3929   3114   -170    120    -37        C
ATOM    844  OG  SER B 711      41.595  31.010  19.169  1.00 35.49           O
ANISOU  844  OG  SER B 711     4168   5095   4223   -266     91      7        O
ATOM    845  H   SER B 711      40.282  28.700  20.209  1.00 22.97           H
ATOM    846  HA  SER B 711      39.087  30.766  18.676  1.00 23.52           H
ATOM    847  HB3 SER B 711      41.209  30.452  17.260  1.00 26.60           H
ATOM    848  HB2 SER B 711      41.655  29.141  18.328  1.00 26.60           H
ATOM    849  HG  SER B 711      41.694  30.595  20.031  1.00 35.49           H
ATOM    850  N   ILE B 712      38.898  27.764  17.324  1.00 23.43           N
ANISOU  850  N   ILE B 712     2803   3355   2745    -43    138   -149        N
ATOM    851  CA  ILE B 712      38.205  26.956  16.315  1.00 22.85           C
ANISOU  851  CA  ILE B 712     2805   3235   2642    -28    148   -191        C
ATOM    852  C   ILE B 712      36.670  27.037  16.452  1.00 25.61           C
ANISOU  852  C   ILE B 712     3185   3539   3006    -94    104   -206        C
ATOM    853  O   ILE B 712      35.996  27.144  15.429  1.00 24.77           O
ANISOU  853  O   ILE B 712     3100   3444   2867   -126     93   -217        O
ATOM    854  CB  ILE B 712      38.669  25.466  16.336  1.00 25.84           C
ANISOU  854  CB  ILE B 712     3258   3573   2986     58    188   -216        C
ATOM    855  CG1 ILE B 712      40.133  25.346  15.855  1.00 26.77           C
ANISOU  855  CG1 ILE B 712     3336   3778   3057    159    245   -186        C
ATOM    856  CG2 ILE B 712      37.785  24.488  15.525  1.00 26.08           C
ANISOU  856  CG2 ILE B 712     3413   3527   2969     26    182   -264        C
ATOM    857  CD1 ILE B 712      40.795  24.007  16.212  1.00 40.14           C
ANISOU  857  CD1 ILE B 712     5106   5444   4703    295    298   -190        C
ATOM    858  H   ILE B 712      39.359  27.253  18.065  1.00 23.43           H
ATOM    859  HA  ILE B 712      38.460  27.367  15.335  1.00 22.85           H
ATOM    860  HB  ILE B 712      38.642  25.135  17.375  1.00 25.84           H
ATOM    861 HG13 ILE B 712      40.740  26.147  16.276  1.00 26.77           H
ATOM    862 HG12 ILE B 712      40.173  25.491  14.775  1.00 26.77           H
ATOM    863 HG21 ILE B 712      38.216  23.488  15.503  1.00 26.08           H
ATOM    864 HG22 ILE B 712      36.786  24.380  15.947  1.00 26.08           H
ATOM    865 HG23 ILE B 712      37.678  24.815  14.490  1.00 26.08           H
ATOM    866 HD11 ILE B 712      41.877  24.116  16.292  1.00 40.14           H
ATOM    867 HD12 ILE B 712      40.430  23.617  17.162  1.00 40.14           H
ATOM    868 HD13 ILE B 712      40.597  23.255  15.449  1.00 40.14           H
ATOM    869  N   LEU B 713      36.149  27.034  17.693  1.00 22.24           N
ANISOU  869  N   LEU B 713     2746   3086   2618   -108     80   -197        N
ATOM    870  CA  LEU B 713      34.719  27.209  17.979  1.00 21.67           C
ANISOU  870  CA  LEU B 713     2673   3014   2547   -152     45   -191        C
ATOM    871  C   LEU B 713      34.213  28.631  17.666  1.00 25.75           C
ANISOU  871  C   LEU B 713     3162   3575   3048   -156     33   -160        C
ATOM    872  O   LEU B 713      33.065  28.776  17.247  1.00 24.68           O
ANISOU  872  O   LEU B 713     3018   3482   2876   -175     15   -149        O
ATOM    873  CB  LEU B 713      34.416  26.856  19.449  1.00 21.07           C
ANISOU  873  CB  LEU B 713     2587   2911   2509   -150     30   -181        C
ATOM    874  CG  LEU B 713      34.640  25.382  19.840  1.00 23.97           C
ANISOU  874  CG  LEU B 713     3007   3222   2878   -147     39   -206        C
ATOM    875  CD1 LEU B 713      34.395  25.193  21.348  1.00 24.00           C
ANISOU  875  CD1 LEU B 713     2993   3211   2915   -156     20   -189        C
ATOM    876  CD2 LEU B 713      33.796  24.401  18.998  1.00 22.68           C
```

```
ANISOU  876  CD2 LEU B 713     2927   3034   2658   -204     32   -236       C
ATOM    877  H   LEU B 713     36.760  26.941  18.494  1.00 22.24           H
ATOM    878  HA  LEU B 713     34.165  26.530  17.329  1.00 21.67           H
ATOM    879  HB3 LEU B 713     33.372  27.090  19.655  1.00 21.07           H
ATOM    880  HB2 LEU B 713     35.007  27.503  20.100  1.00 21.07           H
ATOM    881  HG  LEU B 713     35.687  25.147  19.659  1.00 23.97           H
ATOM    882 HD11 LEU B 713     35.066  24.443  21.767  1.00 24.00           H
ATOM    883 HD12 LEU B 713     34.550  26.114  21.911  1.00 24.00           H
ATOM    884 HD13 LEU B 713     33.373  24.872  21.548  1.00 24.00           H
ATOM    885 HD21 LEU B 713     33.393  23.586  19.597  1.00 22.68           H
ATOM    886 HD22 LEU B 713     32.951  24.895  18.517  1.00 22.68           H
ATOM    887 HD23 LEU B 713     34.403  23.947  18.214  1.00 22.68           H
ATOM    888  N   SER B 714     35.088  29.639  17.827  1.00 21.59           N
ANISOU  888  N   SER B 714     2630   3045   2529   -143     44   -139       N
ATOM    889  CA  SER B 714     34.854  31.017  17.399  1.00 21.58           C
ANISOU  889  CA  SER B 714     2653   3054   2494   -138     43   -108       C
ATOM    890  C   SER B 714     34.842  31.182  15.870  1.00 25.55           C
ANISOU  890  C   SER B 714     3158   3594   2958   -145     52   -116       C
ATOM    891  O   SER B 714     34.106  32.033  15.376  1.00 25.50           O
ANISOU  891  O   SER B 714     3168   3611   2910   -126     47    -92       O
ATOM    892  CB  SER B 714     35.892  31.947  18.057  1.00 24.58           C
ANISOU  892  CB  SER B 714     3064   3405   2870   -163     48    -86       C
ATOM    893  OG  SER B 714     35.386  32.447  19.275  1.00 27.48           O
ANISOU  893  OG  SER B 714     3452   3732   3256   -157     36    -74       O
ATOM    894  H   SER B 714     36.001  29.444  18.214  1.00 21.59           H
ATOM    895  HA  SER B 714     33.861  31.315  17.740  1.00 21.58           H
ATOM    896  HB3 SER B 714     36.109  32.810  17.426  1.00 24.58           H
ATOM    897  HB2 SER B 714     36.841  31.440  18.230  1.00 24.58           H
ATOM    898  HG  SER B 714     36.127  32.586  19.874  1.00 27.48           H
ATOM    899  N   GLU B 715     35.612  30.351  15.144  1.00 21.90           N
ANISOU  899  N   GLU B 715     2683   3142   2495   -155     70   -143       N
ATOM    900  CA  GLU B 715     35.633  30.337  13.681  1.00 21.86           C
ANISOU  900  CA  GLU B 715     2690   3169   2449   -159     83   -156       C
ATOM    901  C   GLU B 715     34.382  29.647  13.095  1.00 25.26           C
ANISOU  901  C   GLU B 715     3133   3617   2849   -178     59   -173       C
ATOM    902  O   GLU B 715     33.907  30.083  12.047  1.00 23.62           O
ANISOU  902  O   GLU B 715     2927   3452   2594   -187     53   -165       O
ATOM    903  CB  GLU B 715     36.951  29.691  13.193  1.00 22.97           C
ANISOU  903  CB  GLU B 715     2824   3322   2581   -138    118   -173       C
ATOM    904  CG  GLU B 715     37.277  29.946  11.701  1.00 28.77           C
ANISOU  904  CG  GLU B 715     3568   4097   3266   -137    139   -174       C
ATOM    905  CD  GLU B 715     37.618  31.401  11.333  1.00 53.21           C
ANISOU  905  CD  GLU B 715     6648   7226   6343   -168    142   -133       C
ATOM    906  OE1 GLU B 715     38.109  32.147  12.211  1.00 45.35           O
ANISOU  906  OE1 GLU B 715     5627   6246   5360   -195    144   -101       O
ATOM    907  OE2 GLU B 715     37.396  31.746  10.152  1.00 45.59           O1-
ANISOU  907  OE2 GLU B 715     5708   6274   5341   -177    139   -128       O1-
ATOM    908  H   GLU B 715     36.209  29.682  15.609  1.00 21.90           H
ATOM    909  HA  GLU B 715     35.617  31.376  13.349  1.00 21.86           H
ATOM    910  HB3 GLU B 715     36.896  28.614  13.348  1.00 22.97           H
ATOM    911  HB2 GLU B 715     37.784  30.020  13.815  1.00 22.97           H
ATOM    912  HG3 GLU B 715     36.453  29.602  11.076  1.00 28.77           H
ATOM    913  HG2 GLU B 715     38.139  29.341  11.420  1.00 28.77           H
ATOM    914  N   VAL B 716     33.836  28.635  13.801  1.00 23.18           N
ANISOU  914  N   VAL B 716     2878   3333   2599   -190     42   -190       N
ATOM    915  CA  VAL B 716     32.542  28.011  13.494  1.00 23.81           C
```

ANISOU 915 CA VAL B 716 2966 3451 2630 -262 9 -195 C
ATOM 916 C VAL B 716 31.371 28.994 13.704 1.00 28.15 C
ANISOU 916 C VAL B 716 3448 4093 3155 -250 -15 -139 C
ATOM 917 O VAL B 716 30.485 29.033 12.854 1.00 28.86 O
ANISOU 917 O VAL B 716 3518 4266 3181 -278 -33 -121 O
ATOM 918 CB VAL B 716 32.291 26.712 14.323 1.00 27.87 C
ANISOU 918 CB VAL B 716 3523 3916 3149 -309 -4 -219 C
ATOM 919 CG1 VAL B 716 30.851 26.152 14.259 1.00 28.34 C
ANISOU 919 CG1 VAL B 716 3585 4044 3140 -419 -50 -208 C
ATOM 920 CG2 VAL B 716 33.261 25.596 13.906 1.00 27.12 C
ANISOU 920 CG2 VAL B 716 3527 3729 3048 -283 32 -267 C
ATOM 921 H VAL B 716 34.298 28.314 14.641 1.00 23.18 H
ATOM 922 HA VAL B 716 32.553 27.738 12.437 1.00 23.81 H
ATOM 923 HB VAL B 716 32.498 26.936 15.370 1.00 27.87 H
ATOM 924 HG11 VAL B 716 30.776 25.205 14.793 1.00 28.34 H
ATOM 925 HG12 VAL B 716 30.122 26.821 14.716 1.00 28.34 H
ATOM 926 HG13 VAL B 716 30.541 25.974 13.229 1.00 28.34 H
ATOM 927 HG21 VAL B 716 33.144 24.712 14.534 1.00 27.12 H
ATOM 928 HG22 VAL B 716 33.087 25.292 12.873 1.00 27.12 H
ATOM 929 HG23 VAL B 716 34.298 25.914 13.986 1.00 27.12 H
ATOM 930 N GLN B 717 31.417 29.798 14.785 1.00 23.39 N
ANISOU 930 N GLN B 717 2820 3480 2588 -192 -9 -106 N
ATOM 931 CA GLN B 717 30.484 30.897 15.065 1.00 23.96 C
ANISOU 931 CA GLN B 717 2857 3624 2624 -127 -13 -44 C
ATOM 932 C GLN B 717 30.405 31.926 13.920 1.00 27.57 C
ANISOU 932 C GLN B 717 3329 4119 3027 -83 -1 -19 C
ATOM 933 O GLN B 717 29.300 32.262 13.493 1.00 26.65 O
ANISOU 933 O GLN B 717 3165 4119 2841 -52 -13 30 O
ATOM 934 CB GLN B 717 30.827 31.534 16.438 1.00 25.84 C
ANISOU 934 CB GLN B 717 3126 3791 2902 -64 4 -28 C
ATOM 935 CG GLN B 717 30.167 32.897 16.757 1.00 38.18 C
ANISOU 935 CG GLN B 717 4724 5372 4410 44 22 32 C
ATOM 936 CD GLN B 717 30.767 33.542 18.007 1.00 49.17 C
ANISOU 936 CD GLN B 717 6197 6661 5824 83 38 37 C
ATOM 937 NE2 GLN B 717 31.566 34.596 17.821 1.00 34.00 N
ANISOU 937 NE2 GLN B 717 4245 4752 3923 101 32 47 N
ATOM 938 OE1 GLN B 717 30.504 33.107 19.125 1.00 42.30 O
ANISOU 938 OE1 GLN B 717 5426 5704 4943 83 54 34 O
ATOM 939 H GLN B 717 32.175 29.685 15.444 1.00 23.39 H
ATOM 940 HA GLN B 717 29.492 30.451 15.153 1.00 23.96 H
ATOM 941 HB3 GLN B 717 31.905 31.660 16.500 1.00 25.84 H
ATOM 942 HB2 GLN B 717 30.578 30.823 17.228 1.00 25.84 H
ATOM 943 HG3 GLN B 717 29.093 32.770 16.894 1.00 38.18 H
ATOM 944 HG2 GLN B 717 30.287 33.606 15.938 1.00 38.18 H
ATOM 945 HE22 GLN B 717 31.992 35.059 18.609 1.00 34.00 H
ATOM 946 HE21 GLN B 717 31.753 34.935 16.886 1.00 34.00 H
ATOM 947 N GLN B 718 31.576 32.381 13.437 1.00 25.94 N
ANISOU 947 N GLN B 718 3181 3834 2841 -82 23 -42 N
ATOM 948 CA GLN B 718 31.686 33.332 12.331 1.00 27.31 C
ANISOU 948 CA GLN B 718 3391 4023 2963 -49 38 -19 C
ATOM 949 C GLN B 718 31.228 32.759 10.979 1.00 32.79 C
ANISOU 949 C GLN B 718 4047 4801 3611 -88 23 -29 C
ATOM 950 O GLN B 718 30.558 33.475 10.238 1.00 32.21 O
ANISOU 950 O GLN B 718 3962 4806 3470 -41 20 16 O
ATOM 951 CB GLN B 718 33.119 33.907 12.245 1.00 28.87 C
ANISOU 951 CB GLN B 718 3653 4132 3184 -73 63 -35 C

```
ATOM    952  CG  GLN B 718      33.567  34.818  13.412  1.00 36.30           C
ANISOU  952  CG  GLN B 718     4661   4994   4139    -57     71    -15       C
ATOM    953  CD  GLN B 718      32.574  35.924  13.777  1.00 55.09           C
ANISOU  953  CD  GLN B 718     7121   7359   6450     35     82     38       C
ATOM    954  NE2 GLN B 718      32.633  37.052  13.068  1.00 41.39           N
ANISOU  954  NE2 GLN B 718     5480   5593   4652     53    100     64       N
ATOM    955  OE1 GLN B 718      31.764  35.761  14.688  1.00 54.01           O
ANISOU  955  OE1 GLN B 718     6975   7240   6305    104     79     62       O
ATOM    956  H   GLN B 718      32.444  32.065  13.847  1.00 25.94           H
ATOM    957  HA  GLN B 718      30.997  34.149  12.546  1.00 27.31           H
ATOM    958  HB3 GLN B 718      33.213  34.482  11.322  1.00 28.87           H
ATOM    959  HB2 GLN B 718      33.833  33.087  12.149  1.00 28.87           H
ATOM    960  HG3 GLN B 718      34.536  35.261  13.182  1.00 36.30           H
ATOM    961  HG2 GLN B 718      33.722  34.221  14.307  1.00 36.30           H
ATOM    962 HE22 GLN B 718      31.996  37.809  13.269  1.00 41.39           H
ATOM    963 HE21 GLN B 718      33.308  37.155  12.324  1.00 41.39           H
ATOM    964  N   ALA B 719      31.550  31.483  10.700  1.00 31.21           N
ANISOU  964  N   ALA B 719     3843   4585   3431   -166     14    -83       N
ATOM    965  CA  ALA B 719      31.125  30.768   9.493  1.00 32.05           C
ANISOU  965  CA  ALA B 719     3947   4751   3477   -226     -5   -102       C
ATOM    966  C   ALA B 719      29.602  30.548   9.420  1.00 37.63           C
ANISOU  966  C   ALA B 719     4586   5594   4117   -262    -48    -58       C
ATOM    967  O   ALA B 719      29.029  30.702   8.342  1.00 36.11           O
ANISOU  967  O   ALA B 719     4368   5502   3850   -279    -65    -33       O
ATOM    968  CB  ALA B 719      31.886  29.437   9.399  1.00 32.60           C
ANISOU  968  CB  ALA B 719     4075   4747   3563   -286      2   -168       C
ATOM    969  H   ALA B 719      32.119  30.958  11.351  1.00 31.21           H
ATOM    970  HA  ALA B 719      31.410  31.376   8.632  1.00 32.05           H
ATOM    971  HB1 ALA B 719      31.610  28.888   8.499  1.00 32.60           H
ATOM    972  HB2 ALA B 719      32.963  29.603   9.360  1.00 32.60           H
ATOM    973  HB3 ALA B 719      31.682  28.797  10.257  1.00 32.60           H
ATOM    974  N   VAL B 720      28.975  30.232  10.567  1.00 35.89           N
ANISOU  974  N   VAL B 720     4323   5400   3914   -273    -66    -37       N
ATOM    975  CA  VAL B 720      27.523  30.092  10.721  1.00 36.73           C
ANISOU  975  CA  VAL B 720     4334   5677   3945   -311   -107     25       C
ATOM    976  C   VAL B 720      26.779  31.429  10.520  1.00 44.39           C
ANISOU  976  C   VAL B 720     5239   6773   4855   -181    -94    111       C
ATOM    977  O   VAL B 720      25.759  31.441   9.831  1.00 43.46           O
ANISOU  977  O   VAL B 720     5044   6829   4640   -205   -122    164       O
ATOM    978  CB  VAL B 720      27.158  29.475  12.111  1.00 40.14           C
ANISOU  978  CB  VAL B 720     4733   6111   4406   -343   -121     35       C
ATOM    979  CG1 VAL B 720      25.701  29.672  12.587  1.00 39.64           C
ANISOU  979  CG1 VAL B 720     4537   6269   4253   -350   -153    128       C
ATOM    980  CG2 VAL B 720      27.486  27.972  12.147  1.00 39.67           C
ANISOU  980  CG2 VAL B 720     4756   5952   4366   -480   -139    -36       C
ATOM    981  H   VAL B 720      29.522  30.099  11.408  1.00 35.89           H
ATOM    982  HA  VAL B 720      27.178  29.408   9.943  1.00 36.73           H
ATOM    983  HB  VAL B 720      27.795  29.950  12.859  1.00 40.14           H
ATOM    984 HG11 VAL B 720      25.513  29.121  13.508  1.00 39.64           H
ATOM    985 HG12 VAL B 720      25.475  30.717  12.802  1.00 39.64           H
ATOM    986 HG13 VAL B 720      24.989  29.317  11.841  1.00 39.64           H
ATOM    987 HG21 VAL B 720      27.358  27.563  13.149  1.00 39.67           H
ATOM    988 HG22 VAL B 720      26.834  27.412  11.476  1.00 39.67           H
ATOM    989 HG23 VAL B 720      28.513  27.771  11.846  1.00 39.67           H
ATOM    990  N   SER B 721      27.309  32.520  11.104  1.00 43.52           N
ANISOU  990  N   SER B 721     5176   6574   4784    -43    -50    128       N
```

```
ATOM    991  CA  SER B 721      26.716  33.860  11.038  1.00 44.74           C
ANISOU  991  CA  SER B 721     5330   6800   4868    115    -22    208       C
ATOM    992  C   SER B 721      26.806  34.524   9.648  1.00 52.24           C
ANISOU  992  C   SER B 721     6318   7762   5768    139    -12    214       C
ATOM    993  O   SER B 721      25.910  35.298   9.311  1.00 51.64           O
ANISOU  993  O   SER B 721     6219   7801   5599    261      2    294       O
ATOM    994  CB  SER B 721      27.294  34.740  12.168  1.00 48.41           C
ANISOU  994  CB  SER B 721     5894   7129   5371    230     20    216       C
ATOM    995  OG  SER B 721      28.598  35.213  11.894  1.00 59.13           O
ANISOU  995  OG  SER B 721     7375   8311   6781    208     44    162       O
ATOM    996  H   SER B 721      28.146  32.427  11.662  1.00 43.52           H
ATOM    997  HA  SER B 721      25.654  33.735  11.255  1.00 44.74           H
ATOM    998  HB3 SER B 721      27.297  34.201  13.116  1.00 48.41           H
ATOM    999  HB2 SER B 721      26.655  35.612  12.312  1.00 48.41           H
ATOM   1000  HG  SER B 721      29.195  34.461  11.846  1.00 59.13           H
ATOM   1001  N   GLN B 722      27.851  34.197   8.863  1.00 52.18           N
ANISOU 1001  N   GLN B 722     6368   7647   5810     42    -13    139       N
ATOM   1002  CA  GLN B 722      28.026  34.666   7.483  1.00 53.29           C
ANISOU 1002  CA  GLN B 722     6542   7802   5902     51     -4    142       C
ATOM   1003  C   GLN B 722      27.181  33.830   6.496  1.00 58.96           C
ANISOU 1003  C   GLN B 722     7172   8685   6544    -37    -49    153       C
ATOM   1004  O   GLN B 722      26.777  34.363   5.463  1.00 59.22           O
ANISOU 1004  O   GLN B 722     7198   8798   6504     -4    -49    186       O
ATOM   1005  CB  GLN B 722      29.549  34.747   7.164  1.00 55.08           C
ANISOU 1005  CB  GLN B 722     6864   7867   6195      3     24     73       C
ATOM   1006  CG  GLN B 722      30.197  33.651   6.278  1.00 77.34           C
ANISOU 1006  CG  GLN B 722     9682  10681   9024   -108     10      8       C
ATOM   1007  CD  GLN B 722      30.190  33.951   4.771  1.00106.19           C
ANISOU 1007  CD  GLN B 722    13350  14389  12608   -107     13     18       C
ATOM   1008  NE2 GLN B 722      30.532  32.951   3.958  1.00 99.93           N
ANISOU 1008  NE2 GLN B 722    12566  13611  11793   -197     -2    -31       N
ATOM   1009  OE1 GLN B 722      29.907  35.065   4.336  1.00104.40           O
ANISOU 1009  OE1 GLN B 722    13148  14181  12337    -23     32     70       O
ATOM   1010  H   GLN B 722      28.564  33.573   9.217  1.00 52.18           H
ATOM   1011  HA  GLN B 722      27.643  35.687   7.430  1.00 53.29           H
ATOM   1012  HB3 GLN B 722      30.100  34.770   8.103  1.00 55.08           H
ATOM   1013  HB2 GLN B 722      29.760  35.732   6.745  1.00 55.08           H
ATOM   1014  HG3 GLN B 722      29.762  32.671   6.478  1.00 77.34           H
ATOM   1015  HG2 GLN B 722      31.247  33.565   6.561  1.00 77.34           H
ATOM   1016 HE22 GLN B 722      30.544  33.095   2.960  1.00 99.93           H
ATOM   1017 HE21 GLN B 722      30.734  32.031   4.331  1.00 99.93           H
ATOM   1018  N   GLY B 723      26.903  32.559   6.844  1.00 56.76           N
ANISOU 1018  N   GLY B 723     6841   8454   6269   -160    -90    128       N
ATOM   1019  CA  GLY B 723      26.072  31.630   6.078  1.00 56.78           C
ANISOU 1019  CA  GLY B 723     6786   8605   6182   -298   -145    135       C
ATOM   1020  C   GLY B 723      26.814  31.058   4.859  1.00 60.94           C
ANISOU 1020  C   GLY B 723     7407   9045   6701   -391   -147     60       C
ATOM   1021  O   GLY B 723      27.864  31.555   4.457  1.00 60.87           O
ANISOU 1021  O   GLY B 723     7480   8895   6752   -327   -101     17       O
ATOM   1022  H   GLY B 723      27.282  32.204   7.711  1.00 56.76           H
ATOM   1023  HA3 GLY B 723      25.158  32.126   5.749  1.00 56.78           H
ATOM   1024  HA2 GLY B 723      25.775  30.814   6.738  1.00 56.78           H
ATOM   1025  N   SER B 724      26.243  29.992   4.267  1.00 56.22           N
ANISOU 1025  N   SER B 724     6809   8539   6015   -554   -201     47       N
ATOM   1026  CA  SER B 724      26.718  29.292   3.059  1.00 55.45           C
ANISOU 1026  CA  SER B 724     6824   8369   5874   -654   -207    -23       C
```

```
ATOM   1027  C   SER B 724      28.119  28.640   3.175  1.00 57.36           C
ANISOU 1027  C   SER B 724     7212   8376   6205   -629   -154   -120       C
ATOM   1028  O   SER B 724      28.678  28.225   2.160  1.00 57.77           O
ANISOU 1028  O   SER B 724     7364   8356   6229   -644   -134   -171       O
ATOM   1029  CB  SER B 724      26.604  30.214   1.818  1.00 59.05           C
ANISOU 1029  CB  SER B 724     7249   8917   6268   -593   -201     15       C
ATOM   1030  OG  SER B 724      25.246  30.443   1.502  1.00 67.08           O
ANISOU 1030  OG  SER B 724     8128  10181   7177   -614   -250    112       O
ATOM   1031  H   SER B 724      25.388  29.625   4.660  1.00 56.22           H
ATOM   1032  HA  SER B 724      26.037  28.451   2.913  1.00 55.45           H
ATOM   1033  HB3 SER B 724      27.055  29.753   0.938  1.00 59.05           H
ATOM   1034  HB2 SER B 724      27.113  31.167   1.963  1.00 59.05           H
ATOM   1035  HG  SER B 724      24.872  31.017   2.174  1.00 67.08           H
ATOM   1036  N   SER B 725      28.649  28.540   4.405  1.00 51.07           N
ANISOU 1036  N   SER B 725     6422   7481   5501   -587   -132   -136       N
ATOM   1037  CA  SER B 725      29.973  28.006   4.725  1.00 49.89           C
ANISOU 1037  CA  SER B 725     6381   7153   5421   -548    -82   -206       C
ATOM   1038  C   SER B 725      29.864  26.640   5.428  1.00 50.10           C
ANISOU 1038  C   SER B 725     6500   7106   5430   -652   -103   -250       C
ATOM   1039  O   SER B 725      30.697  26.319   6.271  1.00 49.60           O
ANISOU 1039  O   SER B 725     6488   6922   5435   -596    -67   -283       O
ATOM   1040  CB  SER B 725      30.738  29.057   5.562  1.00 53.98           C
ANISOU 1040  CB  SER B 725     6846   7617   6047   -418    -39   -185       C
ATOM   1041  OG  SER B 725      31.182  30.112   4.735  1.00 59.98           O
ANISOU 1041  OG  SER B 725     7570   8413   6808   -338    -16   -149       O
ATOM   1042  H   SER B 725      28.123  28.900   5.189  1.00 51.07           H
ATOM   1043  HA  SER B 725      30.544  27.817   3.815  1.00 49.89           H
ATOM   1044  HB3 SER B 725      31.626  28.638   6.033  1.00 53.98           H
ATOM   1045  HB2 SER B 725      30.114  29.454   6.360  1.00 53.98           H
ATOM   1046  HG  SER B 725      31.813  29.759   4.103  1.00 59.98           H
ATOM   1047  N   ASP B 726      28.842  25.847   5.061  1.00 44.45           N
ANISOU 1047  N   ASP B 726     5813   6471   4606   -816   -165   -244       N
ATOM   1048  CA  ASP B 726      28.533  24.528   5.632  1.00 42.91           C
ANISOU 1048  CA  ASP B 726     5735   6208   4362   -957   -195   -277       C
ATOM   1049  C   ASP B 726      29.630  23.475   5.386  1.00 41.85           C
ANISOU 1049  C   ASP B 726     5824   5859   4217   -929   -145   -363       C
ATOM   1050  O   ASP B 726      29.842  22.628   6.253  1.00 40.16           O
ANISOU 1050  O   ASP B 726     5690   5534   4036   -921   -128   -388       O
ATOM   1051  CB  ASP B 726      27.149  23.993   5.181  1.00 45.13           C
ANISOU 1051  CB  ASP B 726     6010   6640   4499  -1175   -278   -243       C
ATOM   1052  CG  ASP B 726      25.947  24.933   5.407  1.00 57.70           C
ANISOU 1052  CG  ASP B 726     7373   8468   6083  -1193   -325   -140       C
ATOM   1053  OD1 ASP B 726      26.017  25.806   6.302  1.00 59.39           O
ANISOU 1053  OD1 ASP B 726     7496   8678   6391  -1095   -304   -111       O
ATOM   1054  OD2 ASP B 726      24.914  24.683   4.749  1.00 62.64           O1-
ANISOU 1054  OD2 ASP B 726     7910   9296   6597  -1295   -380    -81       O1-
ATOM   1055  H   ASP B 726      28.186  26.187   4.373  1.00 44.45           H
ATOM   1056  HA  ASP B 726      28.486  24.657   6.713  1.00 42.91           H
ATOM   1057  HB3 ASP B 726      26.931  23.062   5.707  1.00 45.13           H
ATOM   1058  HB2 ASP B 726      27.190  23.753   4.116  1.00 45.13           H
ATOM   1059  N   SER B 727      30.335  23.572   4.245  1.00 36.31           N
ANISOU 1059  N   SER B 727     5221   5108   3466   -888   -112   -401       N
ATOM   1060  CA  SER B 727      31.496  22.738   3.920  1.00 35.63           C
ANISOU 1060  CA  SER B 727     5342   4842   3354   -805    -46   -470       C
ATOM   1061  C   SER B 727      32.733  23.096   4.769  1.00 37.42           C
ANISOU 1061  C   SER B 727     5487   5018   3712   -607     25   -462       C
```

ATOM 1062 O SER B 727 33.450 22.186 5.183 1.00 35.28 O
ANISOU 1062 O SER B 727 5351 4619 3437 -531 74 -497 O
ATOM 1063 CB SER B 727 31.781 22.838 2.407 1.00 39.87 C
ANISOU 1063 CB SER B 727 5969 5374 3808 -785 -22 -497 C
ATOM 1064 OG SER B 727 32.814 21.959 2.005 1.00 53.73 O
ANISOU 1064 OG SER B 727 7916 6973 5525 -660 57 -552 O
ATOM 1065 H SER B 727 30.098 24.292 3.578 1.00 36.31 H
ATOM 1066 HA SER B 727 31.232 21.702 4.141 1.00 35.63 H
ATOM 1067 HB3 SER B 727 32.055 23.857 2.129 1.00 39.87 H
ATOM 1068 HB2 SER B 727 30.885 22.585 1.839 1.00 39.87 H
ATOM 1069 HG SER B 727 33.632 22.217 2.438 1.00 53.73 H
ATOM 1070 N GLN B 728 32.938 24.399 5.045 1.00 34.27 N
ANISOU 1070 N GLN B 728 4884 4723 3412 -527 30 -411 N
ATOM 1071 CA GLN B 728 34.003 24.910 5.916 1.00 34.20 C
ANISOU 1071 CA GLN B 728 4792 4692 3512 -384 83 -393 C
ATOM 1072 C GLN B 728 33.781 24.499 7.383 1.00 36.43 C
ANISOU 1072 C GLN B 728 5065 4925 3850 -392 71 -390 C
ATOM 1073 O GLN B 728 34.744 24.131 8.049 1.00 34.83 O
ANISOU 1073 O GLN B 728 4898 4653 3681 -292 120 -401 O
ATOM 1074 CB GLN B 728 34.094 26.446 5.770 1.00 35.41 C
ANISOU 1074 CB GLN B 728 4777 4947 3730 -337 82 -339 C
ATOM 1075 CG GLN B 728 35.274 27.106 6.524 1.00 44.13 C
ANISOU 1075 CG GLN B 728 5814 6036 4917 -228 131 -319 C
ATOM 1076 CD GLN B 728 35.108 28.617 6.722 1.00 63.63 C
ANISOU 1076 CD GLN B 728 8166 8570 7441 -213 122 -265 C
ATOM 1077 NE2 GLN B 728 34.589 29.331 5.721 1.00 51.94 N
ANISOU 1077 NE2 GLN B 728 6659 7152 5925 -243 96 -240 N
ATOM 1078 OE1 GLN B 728 35.454 29.140 7.778 1.00 59.38 O
ANISOU 1078 OE1 GLN B 728 7577 8023 6963 -174 139 -242 O
ATOM 1079 H GLN B 728 32.290 25.086 4.689 1.00 34.27 H
ATOM 1080 HA GLN B 728 34.947 24.475 5.581 1.00 34.20 H
ATOM 1081 HB3 GLN B 728 33.162 26.883 6.123 1.00 35.41 H
ATOM 1082 HB2 GLN B 728 34.164 26.703 4.712 1.00 35.41 H
ATOM 1083 HG3 GLN B 728 36.209 26.914 5.998 1.00 44.13 H
ATOM 1084 HG2 GLN B 728 35.394 26.676 7.517 1.00 44.13 H
ATOM 1085 HE22 GLN B 728 34.471 30.328 5.826 1.00 51.94 H
ATOM 1086 HE21 GLN B 728 34.327 28.885 4.855 1.00 51.94 H
ATOM 1087 N ILE B 729 32.520 24.543 7.844 1.00 33.80 N
ANISOU 1087 N ILE B 729 4677 4650 3514 -505 8 -365 N
ATOM 1088 CA ILE B 729 32.085 24.138 9.183 1.00 33.92 C
ANISOU 1088 CA ILE B 729 4674 4639 3574 -534 -12 -354 C
ATOM 1089 C ILE B 729 32.327 22.640 9.479 1.00 37.11 C
ANISOU 1089 C ILE B 729 5276 4896 3927 -551 11 -407 C
ATOM 1090 O ILE B 729 32.657 22.305 10.616 1.00 35.84 O
ANISOU 1090 O ILE B 729 5123 4668 3825 -472 40 -409 O
ATOM 1091 CB ILE B 729 30.590 24.523 9.404 1.00 37.34 C
ANISOU 1091 CB ILE B 729 5000 5206 3982 -655 -81 -304 C
ATOM 1092 CG1 ILE B 729 30.450 26.056 9.555 1.00 37.89 C
ANISOU 1092 CG1 ILE B 729 4897 5390 4111 -566 -80 -242 C
ATOM 1093 CG2 ILE B 729 29.875 23.822 10.576 1.00 37.45 C
ANISOU 1093 CG2 ILE B 729 5030 5194 4005 -726 -107 -296 C
ATOM 1094 CD1 ILE B 729 29.049 26.595 9.231 1.00 48.00 C
ANISOU 1094 CD1 ILE B 729 6075 6844 5318 -631 -130 -183 C
ATOM 1095 H ILE B 729 31.789 24.880 7.230 1.00 33.80 H
ATOM 1096 HA ILE B 729 32.685 24.702 9.899 1.00 33.92 H
ATOM 1097 HB ILE B 729 30.052 24.238 8.499 1.00 37.34 H

ATOM 1098 HG13 ILE B 729 31.155 26.563 8.898 1.00 37.89 H
ATOM 1099 HG12 ILE B 729 30.733 26.357 10.565 1.00 37.89 H
ATOM 1100 HG21 ILE B 729 28.849 24.172 10.666 1.00 37.45 H
ATOM 1101 HG22 ILE B 729 29.814 22.741 10.447 1.00 37.45 H
ATOM 1102 HG23 ILE B 729 30.381 24.026 11.519 1.00 37.45 H
ATOM 1103 HD11 ILE B 729 29.036 27.682 9.281 1.00 48.00 H
ATOM 1104 HD12 ILE B 729 28.732 26.318 8.228 1.00 48.00 H
ATOM 1105 HD13 ILE B 729 28.299 26.226 9.928 1.00 48.00 H
ATOM 1106 N LEU B 730 32.221 21.785 8.446 1.00 35.09 N
ANISOU 1106 N LEU B 730 5200 4583 3551 -642 1 -448 N
ATOM 1107 CA LEU B 730 32.585 20.364 8.488 1.00 35.52 C
ANISOU 1107 CA LEU B 730 5515 4466 3516 -651 31 -502 C
ATOM 1108 C LEU B 730 34.102 20.150 8.655 1.00 36.59 C
ANISOU 1108 C LEU B 730 5697 4515 3691 -424 123 -516 C
ATOM 1109 O LEU B 730 34.497 19.266 9.415 1.00 34.92 O
ANISOU 1109 O LEU B 730 5592 4199 3478 -364 154 -527 O
ATOM 1110 CB LEU B 730 32.052 19.656 7.216 1.00 36.36 C
ANISOU 1110 CB LEU B 730 5821 4526 3467 -776 10 -542 C
ATOM 1111 CG LEU B 730 30.791 18.798 7.449 1.00 42.56 C
ANISOU 1111 CG LEU B 730 6789 5257 4123 -1019 -57 -559 C
ATOM 1112 CD1 LEU B 730 29.621 19.593 8.069 1.00 43.60 C
ANISOU 1112 CD1 LEU B 730 6704 5563 4297 -1175 -140 -495 C
ATOM 1113 CD2 LEU B 730 30.381 18.061 6.156 1.00 45.01 C
ANISOU 1113 CD2 LEU B 730 7286 5542 4272 -1161 -85 -594 C
ATOM 1114 H LEU B 730 31.932 22.139 7.544 1.00 35.09 H
ATOM 1115 HA LEU B 730 32.110 19.924 9.367 1.00 35.52 H
ATOM 1116 HB3 LEU B 730 32.823 19.000 6.807 1.00 36.36 H
ATOM 1117 HB2 LEU B 730 31.857 20.385 6.428 1.00 36.36 H
ATOM 1118 HG LEU B 730 31.067 18.026 8.169 1.00 42.56 H
ATOM 1119 HD11 LEU B 730 28.659 19.352 7.617 1.00 43.60 H
ATOM 1120 HD12 LEU B 730 29.536 19.378 9.134 1.00 43.60 H
ATOM 1121 HD13 LEU B 730 29.758 20.668 7.975 1.00 43.60 H
ATOM 1122 HD21 LEU B 730 30.305 16.988 6.329 1.00 45.01 H
ATOM 1123 HD22 LEU B 730 29.419 18.396 5.769 1.00 45.01 H
ATOM 1124 HD23 LEU B 730 31.107 18.206 5.355 1.00 45.01 H
ATOM 1125 N ASP B 731 34.916 20.979 7.976 1.00 33.35 N
ANISOU 1125 N ASP B 731 5192 4171 3309 -300 168 -504 N
ATOM 1126 CA ASP B 731 36.379 20.993 8.096 1.00 33.51 C
ANISOU 1126 CA ASP B 731 5199 4181 3353 -88 254 -494 C
ATOM 1127 C ASP B 731 36.841 21.462 9.486 1.00 34.74 C
ANISOU 1127 C ASP B 731 5187 4386 3627 -18 259 -450 C
ATOM 1128 O ASP B 731 37.742 20.843 10.047 1.00 35.76 O
ANISOU 1128 O ASP B 731 5367 4472 3747 121 317 -444 O
ATOM 1129 CB ASP B 731 37.088 21.824 6.994 1.00 36.00 C
ANISOU 1129 CB ASP B 731 5418 4596 3665 -17 287 -476 C
ATOM 1130 CG ASP B 731 36.739 21.473 5.536 1.00 58.44 C
ANISOU 1130 CG ASP B 731 8446 7383 6376 -33 305 -519 C
ATOM 1131 OD1 ASP B 731 36.356 20.311 5.268 1.00 61.53 O
ANISOU 1131 OD1 ASP B 731 9093 7630 6656 -24 329 -565 O
ATOM 1132 OD2 ASP B 731 37.001 22.345 4.679 1.00 64.59 O1-
ANISOU 1132 OD2 ASP B 731 9138 8252 7153 -54 297 -507 O1-
ATOM 1133 H ASP B 731 34.517 21.665 7.349 1.00 33.35 H
ATOM 1134 HA ASP B 731 36.714 19.959 7.990 1.00 33.51 H
ATOM 1135 HB3 ASP B 731 38.168 21.710 7.097 1.00 36.00 H
ATOM 1136 HB2 ASP B 731 36.871 22.883 7.142 1.00 36.00 H
ATOM 1137 N LEU B 732 36.192 22.509 10.027 1.00 27.93 N

ANISOU 1137 N   LEU B 732   4138   3617   2858   -106   202   -416   N
ATOM   1138 CA LEU B 732   36.432 23.062 11.364 1.00 27.42   C
ANISOU 1138 CA LEU B 732   3929   3594   2895   -68   197   -377   C
ATOM   1139 C   LEU B 732   36.023 22.109 12.501 1.00 32.34   C
ANISOU 1139 C   LEU B 732   4641   4126   3521   -92   184   -391   C
ATOM   1140 O   LEU B 732   36.684 22.111 13.539 1.00 31.20   O
ANISOU 1140 O   LEU B 732   4449   3980   3427   -6   208   -370   O
ATOM   1141 CB LEU B 732   35.684 24.403 11.511 1.00 26.55   C
ANISOU 1141 CB LEU B 732   3657   3578   2853   -147   144   -341   C
ATOM   1142 CG LEU B 732   36.246 25.560 10.660 1.00 30.16   C
ANISOU 1142 CG LEU B 732   4024   4119   3316   -124   156   -315   C
ATOM   1143 CD1 LEU B 732   35.228 26.709 10.563 1.00 30.19   C
ANISOU 1143 CD1 LEU B 732   3939   4185   3346   -196   106   -284   C
ATOM   1144 CD2 LEU B 732   37.611 26.050 11.182 1.00 29.47   C
ANISOU 1144 CD2 LEU B 732   3852   4078   3266   -33   200   -281   C
ATOM   1145 H   LEU B 732   35.476 22.970   9.481 1.00 27.93   H
ATOM   1146 HA LEU B 732   37.504 23.241 11.459 1.00 27.42   H
ATOM   1147 HB3 LEU B 732   35.690 24.716 12.556 1.00 26.55   H
ATOM   1148 HB2 LEU B 732   34.636 24.243 11.256 1.00 26.55   H
ATOM   1149 HG LEU B 732   36.410 25.199   9.646 1.00 30.16   H
ATOM   1150 HD11 LEU B 732   35.696 27.635 10.234 1.00 30.19   H
ATOM   1151 HD12 LEU B 732   34.445 26.478   9.841 1.00 30.19   H
ATOM   1152 HD13 LEU B 732   34.747 26.900 11.522 1.00 30.19   H
ATOM   1153 HD21 LEU B 732   37.568 27.081 11.525 1.00 29.47   H
ATOM   1154 HD22 LEU B 732   37.973 25.458 12.023 1.00 29.47   H
ATOM   1155 HD23 LEU B 732   38.365 25.998 10.398 1.00 29.47   H
ATOM   1156 N   SER B 733   34.972 21.298 12.281 1.00 28.47   N
ANISOU 1156 N   SER B 733   4281   3571   2965   -225   143   -421   N
ATOM   1157 CA SER B 733   34.522 20.251 13.200 1.00 27.44   C
ANISOU 1157 CA SER B 733   4265   3347   2815   -279   127   -434   C
ATOM   1158 C   SER B 733   35.573 19.138 13.356 1.00 30.70   C
ANISOU 1158 C   SER B 733   4865   3632   3169   -129   202   -458   C
ATOM   1159 O   SER B 733   35.890 18.772 14.486 1.00 31.27   O
ANISOU 1159 O   SER B 733   4932   3669   3279   -55   221   -443   O
ATOM   1160 CB SER B 733   33.156 19.706 12.732 1.00 29.06   C
ANISOU 1160 CB SER B 733   4576   3535   2932   -487   63   -452   C
ATOM   1161 OG SER B 733   32.662 18.699 13.596 1.00 34.21   O
ANISOU 1161 OG SER B 733   5347   4098   3552   -569   43   -458   O
ATOM   1162 H   SER B 733   34.459 21.379 11.413 1.00 28.47   H
ATOM   1163 HA SER B 733   34.382 20.713 14.179 1.00 27.44   H
ATOM   1164 HB3 SER B 733   33.228 19.291 11.728 1.00 29.06   H
ATOM   1165 HB2 SER B 733   32.420 20.509 12.686 1.00 29.06   H
ATOM   1166 HG SER B 733   33.213 17.918 13.500 1.00 34.21   H
ATOM   1167 N   ASN B 734   36.124 18.664 12.224 1.00 28.09   N
ANISOU 1167 N   ASN B 734   4690   3243   2738   -57   251   -488   N
ATOM   1168 CA ASN B 734   37.215 17.687 12.172 1.00 28.20   C
ANISOU 1168 CA ASN B 734   4898   3148   2669   136   340   -501   C
ATOM   1169 C   ASN B 734   38.524 18.227 12.786 1.00 31.94   C
ANISOU 1169 C   ASN B 734   5181   3736   3217   343   398   -445   C
ATOM   1170 O   ASN B 734   39.235 17.469 13.443 1.00 31.81   O
ANISOU 1170 O   ASN B 734   5257   3668   3162   502   458   -431   O
ATOM   1171 CB ASN B 734   37.419 17.251 10.702 1.00 30.78   C
ANISOU 1171 CB ASN B 734   5445   3394   2855   179   384   -541   C
ATOM   1172 CG ASN B 734   38.375 16.063 10.539 1.00 55.40   C
ANISOU 1172 CG ASN B 734   8794   6397   5860   416   489   -547   C
ATOM   1173 ND2 ASN B 734   39.384 16.205   9.677 1.00 45.93   N

ANISOU 1173 ND2 ASN B 734   7524   5292   4637   622   567   -517   N
ATOM   1174 OD1 ASN B 734   38.204 15.029 11.180 1.00 54.44   O
ANISOU 1174 OD1 ASN B 734   8915   6110   5660   427   506   -569   O
ATOM   1175 H   ASN B 734   35.805 19.020 11.333 1.00 28.09   H
ATOM   1176 HA ASN B 734   36.918 16.818 12.762 1.00 28.20   H
ATOM   1177 HB3 ASN B 734   37.758 18.094 10.097 1.00 30.78   H
ATOM   1178 HB2 ASN B 734   36.462 16.944 10.279 1.00 30.78   H
ATOM   1179 HD22 ASN B 734   40.038 15.448   9.541 1.00 45.93   H
ATOM   1180 HD21 ASN B 734   39.500 17.061   9.155 1.00 45.93   H
ATOM   1181 N   ARG B 735   38.797 19.526 12.578 1.00 28.68   N
ANISOU 1181 N   ARG B 735   4512   3487   2898   327   377   -407   N
ATOM   1182 CA ARG B 735   39.972 20.244 13.074 1.00 27.84   C
ANISOU 1182 CA ARG B 735   4198   3526   2854   453   411   -344   C
ATOM   1183 C   ARG B 735   39.989 20.397 14.606 1.00 27.94   C
ANISOU 1183 C   ARG B 735   4134   3538   2942   440   384   -320   C
ATOM   1184 O   ARG B 735   41.070 20.393 15.192 1.00 27.34   O
ANISOU 1184 O   ARG B 735   3999   3525   2863   583   430   -275   O
ATOM   1185 CB ARG B 735   40.029 21.600 12.346 1.00 30.83   C
ANISOU 1185 CB ARG B 735   4377   4041   3295   368   376   -319   C
ATOM   1186 CG ARG B 735   41.376 22.345 12.401 1.00 45.29   C
ANISOU 1186 CG ARG B 735   6048   6034   5125   481   425   -256   C
ATOM   1187 CD ARG B 735   41.405 23.579 11.473 1.00 47.33   C
ANISOU 1187 CD ARG B 735   6212   6381   5392   404   407   -246   C
ATOM   1188 NE ARG B 735   41.359 23.205 10.043 1.00 49.93   N
ANISOU 1188 NE ARG B 735   6676   6667   5628   452   446   -281   N
ATOM   1189 CZ ARG B 735   41.107 23.998   8.985 1.00 61.86   C
ANISOU 1189 CZ ARG B 735   8154   8223   7126   388   433   -285   C
ATOM   1190 NH1 ARG B 735   40.921 25.321   9.110 1.00 48.51   N
ANISOU 1190 NH1 ARG B 735   6312   6615   5505   281   387   -254   N
ATOM   1191 NH2 ARG B 735   41.032 23.445   7.767 1.00 52.19   N1+
ANISOU 1191 NH2 ARG B 735   7071   6953   5807   435   470   -319   N1+
ATOM   1192 H   ARG B 735   38.159 20.070 12.012 1.00 28.68   H
ATOM   1193 HA ARG B 735   40.846 19.660 12.782 1.00 27.84   H
ATOM   1194 HB3 ARG B 735   39.239 22.253 12.716 1.00 30.83   H
ATOM   1195 HB2 ARG B 735   39.803 21.416 11.299 1.00 30.83   H
ATOM   1196 HG3 ARG B 735   42.137 21.640 12.063 1.00 45.29   H
ATOM   1197 HG2 ARG B 735   41.666 22.619 13.415 1.00 45.29   H
ATOM   1198 HD3 ARG B 735   42.236 24.244 11.708 1.00 47.33   H
ATOM   1199 HD2 ARG B 735   40.492 24.148 11.652 1.00 47.33   H
ATOM   1200 HE ARG B 735   41.504 22.222   9.861 1.00 49.93   H
ATOM   1201 HH12 ARG B 735   40.727 25.895   8.302 1.00 48.51   H
ATOM   1202 HH11 ARG B 735   40.976 25.753 10.020 1.00 48.51   H
ATOM   1203 HH22 ARG B 735   40.834 24.015   6.957 1.00 52.19   H
ATOM   1204 HH21 ARG B 735   41.158 22.451   7.647 1.00 52.19   H
ATOM   1205 N   PHE B 736   38.796 20.473 15.223 1.00 23.24   N
ANISOU 1205 N   PHE B 736   3542   2885   2401   275   313   -343   N
ATOM   1206 CA PHE B 736   38.596 20.474 16.672 1.00 22.52   C
ANISOU 1206 CA PHE B 736   3391   2787   2380   252   284   -323   C
ATOM   1207 C   PHE B 736   38.945 19.111 17.302 1.00 27.14   C
ANISOU 1207 C   PHE B 736   4160   3254   2899   359   330   -334   C
ATOM   1208 O   PHE B 736   39.632 19.082 18.322 1.00 26.87   O
ANISOU 1208 O   PHE B 736   4054   3258   2896   455   350   -295   O
ATOM   1209 CB PHE B 736   37.140 20.890 16.974 1.00 24.21   C
ANISOU 1209 CB PHE B 736   3563   2990   2644   68   206   -335   C
ATOM   1210 CG PHE B 736   36.805 21.074 18.442 1.00 25.30   C
ANISOU 1210 CG PHE B 736   3623   3135   2855   45   177   -311   C

ATOM 1211 CD1 PHE B 736 36.366 19.985 19.226 1.00 27.78 C
ANISOU 1211 CD1 PHE B 736 4067 3346 3143 30 176 -324 C
ATOM 1212 CD2 PHE B 736 37.089 22.303 19.071 1.00 26.71 C
ANISOU 1212 CD2 PHE B 736 3623 3413 3114 36 154 -273 C
ATOM 1213 CE1 PHE B 736 36.171 20.147 20.590 1.00 28.61 C
ANISOU 1213 CE1 PHE B 736 4096 3462 3311 16 152 -299 C
ATOM 1214 CE2 PHE B 736 36.876 22.446 20.433 1.00 29.46 C
ANISOU 1214 CE2 PHE B 736 3915 3761 3516 22 131 -252 C
ATOM 1215 CZ PHE B 736 36.421 21.375 21.191 1.00 27.67 C
ANISOU 1215 CZ PHE B 736 3793 3448 3274 17 131 -264 C
ATOM 1216 H PHE B 736 37.955 20.485 14.663 1.00 23.24 H
ATOM 1217 HA PHE B 736 39.262 21.226 17.101 1.00 22.52 H
ATOM 1218 HB3 PHE B 736 36.453 20.156 16.557 1.00 24.21 H
ATOM 1219 HB2 PHE B 736 36.912 21.825 16.463 1.00 24.21 H
ATOM 1220 HD1 PHE B 736 36.187 19.022 18.769 1.00 27.78 H
ATOM 1221 HD2 PHE B 736 37.466 23.138 18.498 1.00 26.71 H
ATOM 1222 HE1 PHE B 736 35.830 19.312 21.183 1.00 28.61 H
ATOM 1223 HE2 PHE B 736 37.074 23.395 20.902 1.00 29.46 H
ATOM 1224 HZ PHE B 736 36.268 21.501 22.252 1.00 27.67 H
ATOM 1225 N TYR B 737 38.488 18.015 16.665 1.00 24.23 N
ANISOU 1225 N TYR B 737 4045 2739 2422 343 349 -381 N
ATOM 1226 CA TYR B 737 38.764 16.634 17.079 1.00 25.41 C
ANISOU 1226 CA TYR B 737 4440 2737 2478 450 401 -394 C
ATOM 1227 C TYR B 737 40.195 16.161 16.774 1.00 31.36 C
ANISOU 1227 C TYR B 737 5237 3522 3156 729 504 -360 C
ATOM 1228 O TYR B 737 40.635 15.188 17.385 1.00 30.97 O
ANISOU 1228 O TYR B 737 5352 3379 3034 877 559 -349 O
ATOM 1229 CB TYR B 737 37.717 15.684 16.458 1.00 27.40 C
ANISOU 1229 CB TYR B 737 4990 2805 2616 304 379 -455 C
ATOM 1230 CG TYR B 737 36.321 15.852 17.030 1.00 30.10 C
ANISOU 1230 CG TYR B 737 5277 3148 3010 48 283 -463 C
ATOM 1231 CD1 TYR B 737 36.104 15.641 18.407 1.00 32.54 C
ANISOU 1231 CD1 TYR B 737 5547 3441 3374 21 261 -441 C
ATOM 1232 CD2 TYR B 737 35.240 16.225 16.204 1.00 30.86 C
ANISOU 1232 CD2 TYR B 737 5332 3295 3097 -155 216 -481 C
ATOM 1233 CE1 TYR B 737 34.830 15.840 18.965 1.00 34.32 C
ANISOU 1233 CE1 TYR B 737 5695 3705 3641 -196 179 -435 C
ATOM 1234 CE2 TYR B 737 33.963 16.430 16.761 1.00 31.63 C
ANISOU 1234 CE2 TYR B 737 5345 3446 3227 -368 134 -468 C
ATOM 1235 CZ TYR B 737 33.763 16.246 18.143 1.00 40.84 C
ANISOU 1235 CZ TYR B 737 6469 4601 4446 -385 118 -443 C
ATOM 1236 OH TYR B 737 32.535 16.452 18.692 1.00 43.95 O
ANISOU 1236 OH TYR B 737 6764 5074 4861 -579 44 -419 O
ATOM 1237 H TYR B 737 37.923 18.125 15.834 1.00 24.23 H
ATOM 1238 HA TYR B 737 38.661 16.597 18.164 1.00 25.41 H
ATOM 1239 HB3 TYR B 737 38.002 14.647 16.643 1.00 27.40 H
ATOM 1240 HB2 TYR B 737 37.696 15.802 15.374 1.00 27.40 H
ATOM 1241 HD1 TYR B 737 36.922 15.334 19.041 1.00 32.54 H
ATOM 1242 HD2 TYR B 737 35.388 16.364 15.144 1.00 30.86 H
ATOM 1243 HE1 TYR B 737 34.679 15.683 20.022 1.00 34.32 H
ATOM 1244 HE2 TYR B 737 33.142 16.732 16.126 1.00 31.63 H
ATOM 1245 HH TYR B 737 31.853 16.647 18.038 1.00 43.95 H
ATOM 1246 N THR B 738 40.905 16.861 15.875 1.00 29.10 N
ANISOU 1246 N THR B 738 4793 3387 2878 810 533 -331 N
ATOM 1247 CA THR B 738 42.333 16.667 15.624 1.00 29.97 C
ANISOU 1247 CA THR B 738 4879 3597 2910 1078 631 -275 C

ATOM 1248 C THR B 738 43.186 17.348 16.715 1.00 36.70 C
ANISOU 1248 C THR B 738 5456 4641 3846 1141 626 -195 C
ATOM 1249 O THR B 738 44.201 16.782 17.120 1.00 38.91 O
ANISOU 1249 O THR B 738 5739 4984 4061 1359 696 -137 O
ATOM 1250 CB THR B 738 42.742 17.248 14.242 1.00 33.82 C
ANISOU 1250 CB THR B 738 5323 4175 3351 1117 663 -273 C
ATOM 1251 CG2 THR B 738 44.240 17.158 13.894 1.00 29.01 C
ANISOU 1251 CG2 THR B 738 4701 3691 2632 1414 776 -205 C
ATOM 1252 OG1 THR B 738 42.030 16.567 13.227 1.00 34.55 O
ANISOU 1252 OG1 THR B 738 5669 4088 3369 1015 650 -351 O
ATOM 1253 H THR B 738 40.465 17.629 15.388 1.00 29.10 H
ATOM 1254 HA THR B 738 42.557 15.598 15.632 1.00 29.97 H
ATOM 1255 HB THR B 738 42.443 18.295 14.187 1.00 33.82 H
ATOM 1256 HG21 THR B 738 44.421 17.481 12.868 1.00 29.01 H
ATOM 1257 HG22 THR B 738 44.848 17.793 14.537 1.00 29.01 H
ATOM 1258 HG23 THR B 738 44.606 16.135 13.987 1.00 29.01 H
ATOM 1259 HG1 THR B 738 41.088 16.723 13.353 1.00 34.55 H
ATOM 1260 N LEU B 739 42.747 18.533 17.177 1.00 32.73 N
ANISOU 1260 N LEU B 739 4733 4230 3473 949 542 -189 N
ATOM 1261 CA LEU B 739 43.433 19.340 18.186 1.00 33.24 C
ANISOU 1261 CA LEU B 739 4553 4465 3614 943 516 -121 C
ATOM 1262 C LEU B 739 43.269 18.775 19.610 1.00 36.77 C
ANISOU 1262 C LEU B 739 5049 4839 4085 966 505 -115 C
ATOM 1263 O LEU B 739 44.251 18.739 20.352 1.00 36.38 O
ANISOU 1263 O LEU B 739 4896 4914 4013 1103 538 -46 O
ATOM 1264 CB LEU B 739 42.920 20.793 18.090 1.00 33.75 C
ANISOU 1264 CB LEU B 739 4448 4595 3782 729 435 -128 C
ATOM 1265 CG LEU B 739 43.836 21.835 18.765 1.00 39.59 C
ANISOU 1265 CG LEU B 739 4947 5551 4543 712 426 -54 C
ATOM 1266 CD1 LEU B 739 45.168 22.016 18.006 1.00 39.30 C
ANISOU 1266 CD1 LEU B 739 4857 5645 4429 799 481 -18 C
ATOM 1267 CD2 LEU B 739 43.096 23.167 18.965 1.00 43.98 C
ANISOU 1267 CD2 LEU B 739 5418 6104 5189 507 347 -69 C
ATOM 1268 H LEU B 739 41.899 18.930 16.797 1.00 32.73 H
ATOM 1269 HA LEU B 739 44.496 19.328 17.942 1.00 33.24 H
ATOM 1270 HB3 LEU B 739 41.916 20.836 18.516 1.00 33.75 H
ATOM 1271 HB2 LEU B 739 42.803 21.080 17.044 1.00 33.75 H
ATOM 1272 HG LEU B 739 44.074 21.474 19.767 1.00 39.59 H
ATOM 1273 HD11 LEU B 739 45.395 23.061 17.795 1.00 39.30 H
ATOM 1274 HD12 LEU B 739 45.999 21.623 18.592 1.00 39.30 H
ATOM 1275 HD13 LEU B 739 45.170 21.490 17.051 1.00 39.30 H
ATOM 1276 HD21 LEU B 739 43.427 23.941 18.274 1.00 43.98 H
ATOM 1277 HD22 LEU B 739 42.021 23.063 18.832 1.00 43.98 H
ATOM 1278 HD23 LEU B 739 43.257 23.533 19.977 1.00 43.98 H
ATOM 1279 N ILE B 740 42.047 18.333 19.945 1.00 33.41 N
ANISOU 1279 N ILE B 740 4773 4230 3690 833 458 -179 N
ATOM 1280 CA ILE B 740 41.710 17.658 21.197 1.00 34.63 C
ANISOU 1280 CA ILE B 740 5004 4293 3861 837 446 -178 C
ATOM 1281 C ILE B 740 41.278 16.225 20.802 1.00 39.79 C
ANISOU 1281 C ILE B 740 5984 4734 4399 906 494 -224 C
ATOM 1282 O ILE B 740 40.158 16.065 20.312 1.00 38.53 O
ANISOU 1282 O ILE B 740 5966 4443 4232 732 450 -284 O
ATOM 1283 CB ILE B 740 40.519 18.359 21.933 1.00 38.32 C
ANISOU 1283 CB ILE B 740 5385 4735 4440 615 354 -202 C
ATOM 1284 CG1 ILE B 740 40.718 19.881 22.139 1.00 39.46 C
ANISOU 1284 CG1 ILE B 740 5281 5041 4672 523 307 -172 C

```
ATOM   1285  CG2 ILE B 740      40.178  17.673  23.277  1.00 38.94           C
ANISOU 1285  CG2 ILE B 740     5514   4744   4538    621    343   -192       C
ATOM   1286  CD1 ILE B 740      41.886  20.271  23.056  1.00 48.81           C
ANISOU 1286  CD1 ILE B 740     6278   6389   5880    593    313   -101       C
ATOM   1287  H   ILE B 740      41.291  18.414  19.277  1.00 33.41           H
ATOM   1288  HA  ILE B 740      42.559  17.648  21.877  1.00 34.63           H
ATOM   1289  HB  ILE B 740      39.628  18.281  21.308  1.00 38.32           H
ATOM   1290 HG13 ILE B 740      39.800  20.306  22.548  1.00 39.46           H
ATOM   1291 HG12 ILE B 740      40.848  20.373  21.174  1.00 39.46           H
ATOM   1292 HG21 ILE B 740      39.354  18.181  23.780  1.00 38.94           H
ATOM   1293 HG22 ILE B 740      39.878  16.634  23.147  1.00 38.94           H
ATOM   1294 HG23 ILE B 740      41.031  17.676  23.955  1.00 38.94           H
ATOM   1295 HD11 ILE B 740      42.032  21.351  23.049  1.00 48.81           H
ATOM   1296 HD12 ILE B 740      41.702  19.974  24.088  1.00 48.81           H
ATOM   1297 HD13 ILE B 740      42.818  19.811  22.732  1.00 48.81           H
ATOM   1298  N   PRO B 741      42.169  15.214  20.945  1.00 38.85           N
ANISOU 1298  N   PRO B 741     6008   4585   4168   1152    584   -193       N
ATOM   1299  CA  PRO B 741      41.864  13.841  20.500  1.00 39.08           C
ANISOU 1299  CA  PRO B 741     6415   4374   4061   1210    633   -242       C
ATOM   1300  C   PRO B 741      40.802  13.167  21.385  1.00 43.77           C
ANISOU 1300  C   PRO B 741     7182   4783   4666   1037    579   -283       C
ATOM   1301  O   PRO B 741      40.974  13.110  22.603  1.00 41.86           O
ANISOU 1301  O   PRO B 741     6846   4573   4487   1041    559   -250       O
ATOM   1302  CB  PRO B 741      43.223  13.126  20.552  1.00 40.89           C
ANISOU 1302  CB  PRO B 741     6733   4639   4164   1552    750   -179       C
ATOM   1303  CG  PRO B 741      44.004  13.860  21.627  1.00 45.48           C
ANISOU 1303  CG  PRO B 741     6971   5481   4828   1634    744    -93       C
ATOM   1304  CD  PRO B 741      43.527  15.304  21.489  1.00 40.64           C
ANISOU 1304  CD  PRO B 741     6074   5004   4362   1391    650   -103       C
ATOM   1305  HA  PRO B 741      41.514  13.849  19.467  1.00 39.08           H
ATOM   1306  HB3 PRO B 741      43.728  13.242  19.592  1.00 40.89           H
ATOM   1307  HB2 PRO B 741      43.149  12.057  20.752  1.00 40.89           H
ATOM   1308  HG3 PRO B 741      45.084  13.747  21.527  1.00 45.48           H
ATOM   1309  HG2 PRO B 741      43.715  13.469  22.603  1.00 45.48           H
ATOM   1310  HD2 PRO B 741      43.570  15.807  22.456  1.00 40.64           H
ATOM   1311  HD3 PRO B 741      44.154  15.850  20.784  1.00 40.64           H
ATOM   1312  N   HIS B 742      39.722  12.698  20.744  1.00 41.11           N
ANISOU 1312  N   HIS B 742     7081   4274   4265    855    547   -350       N
ATOM   1313  CA  HIS B 742      38.615  11.989  21.384  1.00 40.94           C
ANISOU 1313  CA  HIS B 742     7236   4093   4227    642    489   -385       C
ATOM   1314  C   HIS B 742      38.582  10.514  20.962  1.00 47.48           C
ANISOU 1314  C   HIS B 742     8522   4657   4861    696    548   -421       C
ATOM   1315  O   HIS B 742      39.059  10.165  19.880  1.00 47.69           O
ANISOU 1315  O   HIS B 742     8739   4612   4769    817    611   -443       O
ATOM   1316  CB  HIS B 742      37.278  12.667  21.027  1.00 40.97           C
ANISOU 1316  CB  HIS B 742     7128   4146   4292    338    390   -418       C
ATOM   1317  CG  HIS B 742      36.968  13.957  21.748  1.00 43.49           C
ANISOU 1317  CG  HIS B 742     7090   4656   4778    239    321   -385       C
ATOM   1318  CD2 HIS B 742      35.949  14.288  22.617  1.00 44.29           C
ANISOU 1318  CD2 HIS B 742     7100   4786   4943     43    247   -378       C
ATOM   1319  ND1 HIS B 742      37.717  15.111  21.608  1.00 44.70           N
ANISOU 1319  ND1 HIS B 742     6965   4991   5027    338    328   -353       N
ATOM   1320  CE1 HIS B 742      37.131  16.058  22.343  1.00 43.53           C
ANISOU 1320  CE1 HIS B 742     6598   4948   4992    212    262   -334       C
ATOM   1321  NE2 HIS B 742      36.050  15.632  22.983  1.00 43.75           N
ANISOU 1321  NE2 HIS B 742     6718   4899   5007     48    215   -346       N
```

```
ATOM   1322  H   HIS B 742      39.666  12.780  19.739  1.00 41.11           H
ATOM   1323  HA  HIS B 742      38.729  12.000  22.466  1.00 40.94           H
ATOM   1324  HB3 HIS B 742      36.463  11.984  21.266  1.00 40.97           H
ATOM   1325  HB2 HIS B 742      37.216  12.844  19.952  1.00 40.97           H
ATOM   1326  HD2 HIS B 742      35.146  13.669  22.989  1.00 44.29           H
ATOM   1327  HD1 HIS B 742      38.551  15.226  21.044  1.00 44.70           H
ATOM   1328  HE1 HIS B 742      37.497  17.072  22.405  1.00 43.53           H
ATOM   1329  N   ASP B 743      37.978   9.693  21.835  1.00 45.47           N
ANISOU 1329  N   ASP B 743     8466   4250   4562    604    531   -427       N
ATOM   1330  CA  ASP B 743      37.733   8.270  21.639  1.00 46.48           C
ANISOU 1330  CA  ASP B 743     9080   4092   4488    622    581   -462       C
ATOM   1331  C   ASP B 743      36.233   8.033  21.863  1.00 51.47           C
ANISOU 1331  C   ASP B 743     9876   4603   5076    241    484   -500       C
ATOM   1332  O   ASP B 743      35.793   7.919  23.007  1.00 50.63           O
ANISOU 1332  O   ASP B 743     9761   4472   5006    136    447   -479       O
ATOM   1333  CB  ASP B 743      38.623   7.407  22.571  1.00 48.48           C
ANISOU 1333  CB  ASP B 743     9482   4259   4678    917    673   -416       C
ATOM   1334  CG  ASP B 743      38.482   5.875  22.461  1.00 61.34           C
ANISOU 1334  CG  ASP B 743    11662   5563   6083    957    734   -445       C
ATOM   1335  OD1 ASP B 743      38.030   5.383  21.401  1.00 60.10           O
ANISOU 1335  OD1 ASP B 743    11828   5234   5774    874    747   -500       O
ATOM   1336  OD2 ASP B 743      38.963   5.207  23.403  1.00 69.40           O1-
ANISOU 1336  OD2 ASP B 743    12811   6490   7067   1069    769   -413       O1-
ATOM   1337  H   ASP B 743      37.593  10.083  22.686  1.00 45.47           H
ATOM   1338  HA  ASP B 743      37.967   8.000  20.607  1.00 46.48           H
ATOM   1339  HB3 ASP B 743      38.481   7.718  23.608  1.00 48.48           H
ATOM   1340  HB2 ASP B 743      39.661   7.640  22.349  1.00 48.48           H
ATOM   1341  N   PHE B 744      35.492   7.957  20.748  1.00 48.43           N
ANISOU 1341  N   PHE B 744     9628   4167   4608     25    440   -548       N
ATOM   1342  CA  PHE B 744      34.073   7.597  20.709  1.00 49.00           C
ANISOU 1342  CA  PHE B 744     9826   4178   4614   -368    340   -571       C
ATOM   1343  C   PHE B 744      33.840   6.096  20.435  1.00 56.20           C
ANISOU 1343  C   PHE B 744    11305   4773   5274   -480    363   -615       C
ATOM   1344  O   PHE B 744      32.683   5.677  20.384  1.00 56.65           O
ANISOU 1344  O   PHE B 744    11507   4786   5230   -837    278   -638       O
ATOM   1345  CB  PHE B 744      33.346   8.498  19.687  1.00 50.61           C
ANISOU 1345  CB  PHE B 744     9772   4577   4882   -586    256   -581       C
ATOM   1346  CG  PHE B 744      33.120   9.925  20.158  1.00 52.01           C
ANISOU 1346  CG  PHE B 744     9445   5039   5280   -588    205   -534       C
ATOM   1347  CD1 PHE B 744      32.017  10.228  20.983  1.00 54.69           C
ANISOU 1347  CD1 PHE B 744     9606   5493   5682   -823    119   -500       C
ATOM   1348  CD2 PHE B 744      34.084  10.926  19.918  1.00 54.20           C
ANISOU 1348  CD2 PHE B 744     9442   5469   5680   -362    244   -520       C
ATOM   1349  CE1 PHE B 744      31.856  11.509  21.493  1.00 55.24           C
ANISOU 1349  CE1 PHE B 744     9254   5805   5932   -802     81   -457       C
ATOM   1350  CE2 PHE B 744      33.889  12.209  20.412  1.00 56.78           C
ANISOU 1350  CE2 PHE B 744     9359   6028   6185   -378    197   -480       C
ATOM   1351  CZ  PHE B 744      32.787  12.497  21.204  1.00 54.56           C
ANISOU 1351  CZ  PHE B 744     8935   5836   5961   -582    121   -451       C
ATOM   1352  H   PHE B 744      35.940   8.080  19.852  1.00 48.43           H
ATOM   1353  HA  PHE B 744      33.623   7.783  21.685  1.00 49.00           H
ATOM   1354  HB3 PHE B 744      32.367   8.087  19.442  1.00 50.61           H
ATOM   1355  HB2 PHE B 744      33.898   8.521  18.746  1.00 50.61           H
ATOM   1356  HD1 PHE B 744      31.294   9.466  21.228  1.00 54.69           H
ATOM   1357  HD2 PHE B 744      34.968  10.703  19.340  1.00 54.20           H
ATOM   1358  HE1 PHE B 744      31.007  11.735  22.121  1.00 55.24           H
```

ATOM 1359 HE2 PHE B 744 34.604 12.986 20.190 1.00 56.78 H
ATOM 1360 HZ PHE B 744 32.653 13.493 21.600 1.00 54.56 H
ATOM 1361 N GLY B 745 34.918 5.306 20.275 1.00 54.01 N
ANISOU 1361 N GLY B 745 11353 4287 4881 -182 477 -619 N
ATOM 1362 CA GLY B 745 34.856 3.880 19.960 1.00 54.12 C
ANISOU 1362 CA GLY B 745 11974 3959 4631 -240 518 -661 C
ATOM 1363 C GLY B 745 34.390 3.694 18.510 1.00 60.51 C
ANISOU 1363 C GLY B 745 13049 4662 5278 -406 501 -721 C
ATOM 1364 O GLY B 745 34.908 4.342 17.600 1.00 59.36 O
ANISOU 1364 O GLY B 745 12841 4574 5140 -181 562 -734 O
ATOM 1365 H GLY B 745 35.842 5.706 20.369 1.00 54.01 H
ATOM 1366 HA3 GLY B 745 34.195 3.365 20.659 1.00 54.12 H
ATOM 1367 HA2 GLY B 745 35.851 3.449 20.074 1.00 54.12 H
ATOM 1368 N MET B 746 33.413 2.795 18.310 1.00 59.72 N
ANISOU 1368 N MET B 746 13249 4421 5021 -818 413 -752 N
ATOM 1369 CA MET B 746 32.771 2.528 17.017 1.00 60.58 C
ANISOU 1369 CA MET B 746 13653 4421 4945 -1051 376 -809 C
ATOM 1370 C MET B 746 31.732 3.594 16.601 1.00 64.98 C
ANISOU 1370 C MET B 746 13797 5276 5616 -1409 237 -796 C
ATOM 1371 O MET B 746 31.250 3.532 15.469 1.00 65.11 O
ANISOU 1371 O MET B 746 14015 5247 5477 -1699 174 -831 O
ATOM 1372 CB MET B 746 32.132 1.122 17.050 1.00 63.00 C
ANISOU 1372 CB MET B 746 14625 4363 4950 -1298 370 -848 C
ATOM 1373 CG MET B 746 33.098 -0.041 17.349 1.00 66.86 C
ANISOU 1373 CG MET B 746 15618 4515 5269 -922 521 -863 C
ATOM 1374 SD MET B 746 34.364 -0.376 16.089 1.00 70.95 S
ANISOU 1374 SD MET B 746 16348 4928 5681 -474 669 -902 S
ATOM 1375 CE MET B 746 35.794 0.507 16.771 1.00 67.72 C
ANISOU 1375 CE MET B 746 15522 4716 5493 105 793 -827 C
ATOM 1376 H MET B 746 33.043 2.285 19.098 1.00 59.72 H
ATOM 1377 HA MET B 746 33.538 2.532 16.241 1.00 60.58 H
ATOM 1378 HB3 MET B 746 31.646 0.920 16.095 1.00 63.00 H
ATOM 1379 HB2 MET B 746 31.332 1.108 17.793 1.00 63.00 H
ATOM 1380 HG3 MET B 746 32.508 -0.953 17.443 1.00 66.86 H
ATOM 1381 HG2 MET B 746 33.581 0.091 18.317 1.00 66.86 H
ATOM 1382 HE1 MET B 746 36.678 0.305 16.167 1.00 67.72 H
ATOM 1383 HE2 MET B 746 35.625 1.582 16.772 1.00 67.72 H
ATOM 1384 HE3 MET B 746 35.994 0.183 17.792 1.00 67.72 H
ATOM 1385 N LYS B 747 31.401 4.542 17.496 1.00 60.96 N
ANISOU 1385 N LYS B 747 12728 5073 5362 -1378 193 -740 N
ATOM 1386 CA LYS B 747 30.455 5.633 17.250 1.00 60.75 C
ANISOU 1386 CA LYS B 747 12262 5362 5460 -1646 76 -708 C
ATOM 1387 C LYS B 747 31.157 6.825 16.574 1.00 65.31 C
ANISOU 1387 C LYS B 747 12461 6149 6204 -1412 104 -705 C
ATOM 1388 O LYS B 747 32.334 7.074 16.837 1.00 65.42 O
ANISOU 1388 O LYS B 747 12374 6162 6322 -1039 201 -701 O
ATOM 1389 CB LYS B 747 29.838 6.091 18.591 1.00 62.59 C
ANISOU 1389 CB LYS B 747 12144 5787 5851 -1745 16 -642 C
ATOM 1390 CG LYS B 747 29.123 4.970 19.372 1.00 68.57 C
ANISOU 1390 CG LYS B 747 13132 6457 6463 -2109 -59 -620 C
ATOM 1391 CD LYS B 747 28.471 5.406 20.701 1.00 76.03 C
ANISOU 1391 CD LYS B 747 13778 7540 7569 -2076 -75 -558 C
ATOM 1392 CE LYS B 747 29.433 5.609 21.894 1.00 77.62 C
ANISOU 1392 CE LYS B 747 14072 7579 7841 -1697 36 -564 C
ATOM 1393 NZ LYS B 747 30.122 6.913 21.881 1.00 80.52 N1+
ANISOU 1393 NZ LYS B 747 13967 8169 8457 -1393 71 -535 N1+

ATOM 1394 H LYS B 747 31.858 4.550 18.397 1.00 60.96 H
ATOM 1395 HA LYS B 747 29.653 5.278 16.600 1.00 60.75 H
ATOM 1396 HB3 LYS B 747 29.126 6.895 18.401 1.00 62.59 H
ATOM 1397 HB2 LYS B 747 30.618 6.528 19.207 1.00 62.59 H
ATOM 1398 HG3 LYS B 747 29.813 4.150 19.576 1.00 68.57 H
ATOM 1399 HG2 LYS B 747 28.349 4.549 18.730 1.00 68.57 H
ATOM 1400 HD3 LYS B 747 27.743 4.644 20.982 1.00 76.03 H
ATOM 1401 HD2 LYS B 747 27.882 6.310 20.537 1.00 76.03 H
ATOM 1402 HE3 LYS B 747 30.168 4.804 21.944 1.00 77.62 H
ATOM 1403 HE2 LYS B 747 28.861 5.568 22.821 1.00 77.62 H
ATOM 1404 HZ1 LYS B 747 29.444 7.660 21.867 1.00 80.52 H
ATOM 1405 HZ2 LYS B 747 30.699 7.012 22.708 1.00 80.52 H
ATOM 1406 HZ3 LYS B 747 30.713 6.973 21.065 1.00 80.52 H
ATOM 1407 N LYS B 748 30.394 7.574 15.761 1.00 60.77 N
ANISOU 1407 N LYS B 748 11654 5783 5652 -1642 16 -695 N
ATOM 1408 CA LYS B 748 30.819 8.850 15.182 1.00 59.92 C
ANISOU 1408 CA LYS B 748 11180 5888 5701 -1464 30 -686 C
ATOM 1409 C LYS B 748 30.771 9.973 16.246 1.00 62.94 C
ANISOU 1409 C LYS B 748 11083 6514 6319 -1368 11 -624 C
ATOM 1410 O LYS B 748 29.775 10.043 16.970 1.00 62.81 O
ANISOU 1410 O LYS B 748 10929 6608 6328 -1587 -65 -581 O
ATOM 1411 CB LYS B 748 29.879 9.210 14.012 1.00 62.36 C
ANISOU 1411 CB LYS B 748 11429 6333 5934 -1737 -56 -692 C
ATOM 1412 CG LYS B 748 30.085 8.336 12.763 1.00 71.31 C
ANISOU 1412 CG LYS B 748 12994 7251 6852 -1780 -27 -759 C
ATOM 1413 CD LYS B 748 29.313 8.864 11.543 1.00 82.34 C
ANISOU 1413 CD LYS B 748 14229 8831 8226 -1946 -95 -758 C
ATOM 1414 CE LYS B 748 29.573 8.033 10.275 1.00 97.89 C
ANISOU 1414 CE LYS B 748 16659 10579 9954 -2043 -80 -826 C
ATOM 1415 NZ LYS B 748 28.875 8.590 9.102 1.00108.02 N1+
ANISOU 1415 NZ LYS B 748 17772 12032 11240 -2094 -115 -830 N1+
ATOM 1416 H LYS B 748 29.432 7.314 15.599 1.00 60.77 H
ATOM 1417 HA LYS B 748 31.817 8.703 14.776 1.00 59.92 H
ATOM 1418 HB3 LYS B 748 30.076 10.244 13.721 1.00 62.36 H
ATOM 1419 HB2 LYS B 748 28.835 9.183 14.329 1.00 62.36 H
ATOM 1420 HG3 LYS B 748 29.779 7.311 12.977 1.00 71.31 H
ATOM 1421 HG2 LYS B 748 31.148 8.294 12.522 1.00 71.31 H
ATOM 1422 HD3 LYS B 748 29.592 9.904 11.367 1.00 82.34 H
ATOM 1423 HD2 LYS B 748 28.246 8.868 11.772 1.00 82.34 H
ATOM 1424 HE3 LYS B 748 29.246 7.004 10.424 1.00 97.89 H
ATOM 1425 HE2 LYS B 748 30.641 8.003 10.058 1.00 97.89 H
ATOM 1426 HZ1 LYS B 748 29.195 9.533 8.933 1.00108.02 H
ATOM 1427 HZ2 LYS B 748 29.070 8.020 8.291 1.00108.02 H
ATOM 1428 HZ3 LYS B 748 27.880 8.599 9.273 1.00108.02 H
ATOM 1429 N PRO B 749 31.802 10.855 16.305 1.00 57.52 N
ANISOU 1429 N PRO B 749 10146 5920 5791 -1055 77 -614 N
ATOM 1430 CA PRO B 749 31.738 12.103 17.099 1.00 56.67 C
ANISOU 1430 CA PRO B 749 9617 6027 5887 -980 56 -558 C
ATOM 1431 C PRO B 749 30.572 13.039 16.687 1.00 57.84 C
ANISOU 1431 C PRO B 749 9457 6423 6097 -1174 -33 -523 C
ATOM 1432 O PRO B 749 30.224 13.062 15.504 1.00 56.02 O
ANISOU 1432 O PRO B 749 9274 6224 5787 -1279 -59 -543 O
ATOM 1433 CB PRO B 749 33.090 12.788 16.809 1.00 58.57 C
ANISOU 1433 CB PRO B 749 9726 6298 6229 -641 145 -558 C
ATOM 1434 CG PRO B 749 34.015 11.690 16.320 1.00 63.19 C
ANISOU 1434 CG PRO B 749 10673 6673 6662 -494 227 -604 C

```
ATOM   1435  CD  PRO B 749      33.078  10.736  15.598  1.00 58.90           C
ANISOU 1435  CD  PRO B 749    10413   6021   5947   -770    173   -645       C
ATOM   1436  HA  PRO B 749      31.683  11.800  18.144  1.00 56.67           H
ATOM   1437  HB3 PRO B 749      33.494  13.299  17.684  1.00 58.57           H
ATOM   1438  HB2 PRO B 749      32.991  13.537  16.020  1.00 58.57           H
ATOM   1439  HG3 PRO B 749      34.455  11.176  17.172  1.00 63.19           H
ATOM   1440  HG2 PRO B 749      34.830  12.055  15.695  1.00 63.19           H
ATOM   1441  HD2 PRO B 749      32.938  11.035  14.558  1.00 58.90           H
ATOM   1442  HD3 PRO B 749      33.497   9.729  15.624  1.00 58.90           H
ATOM   1443  N   PRO B 750      30.011  13.814  17.645  1.00 52.99           N
ANISOU 1443  N   PRO B 750     8532   5990   5612  -1201    -74   -466       N
ATOM   1444  CA  PRO B 750      28.932  14.774  17.355  1.00 52.07           C
ANISOU 1444  CA  PRO B 750     8137   6117   5529  -1346   -147   -420       C
ATOM   1445  C   PRO B 750      29.433  15.975  16.530  1.00 53.40           C
ANISOU 1445  C   PRO B 750     8133   6384   5771  -1195   -122   -426       C
ATOM   1446  O   PRO B 750      30.348  16.676  16.963  1.00 53.24           O
ANISOU 1446  O   PRO B 750     8013   6352   5864   -959    -62   -431       O
ATOM   1447  CB  PRO B 750      28.413  15.168  18.748  1.00 53.98           C
ANISOU 1447  CB  PRO B 750     8136   6494   5879  -1344   -171   -359       C
ATOM   1448  CG  PRO B 750      29.597  14.982  19.682  1.00 58.51           C
ANISOU 1448  CG  PRO B 750     8777   6924   6533  -1129   -103   -379       C
ATOM   1449  CD  PRO B 750      30.357  13.809  19.067  1.00 54.23           C
ANISOU 1449  CD  PRO B 750     8596   6140   5869  -1098    -54   -437       C
ATOM   1450  HA  PRO B 750      28.129  14.276  16.807  1.00 52.07           H
ATOM   1451  HB3 PRO B 750      27.614  14.484  19.037  1.00 53.98           H
ATOM   1452  HB2 PRO B 750      28.004  16.179  18.794  1.00 53.98           H
ATOM   1453  HG3 PRO B 750      29.309  14.810  20.719  1.00 58.51           H
ATOM   1454  HG2 PRO B 750      30.213  15.880  19.659  1.00 58.51           H
ATOM   1455  HD2 PRO B 750      31.429  13.909  19.234  1.00 54.23           H
ATOM   1456  HD3 PRO B 750      30.020  12.869  19.506  1.00 54.23           H
ATOM   1457  N   LEU B 751      28.843  16.153  15.337  1.00 47.49           N
ANISOU 1457  N   LEU B 751     7358   5740   4945  -1346   -171   -421       N
ATOM   1458  CA  LEU B 751      29.201  17.185  14.362  1.00 46.66           C
ANISOU 1458  CA  LEU B 751     7105   5733   4890  -1233   -154   -422       C
ATOM   1459  C   LEU B 751      28.938  18.604  14.905  1.00 48.19           C
ANISOU 1459  C   LEU B 751     6967   6119   5225  -1133   -163   -361       C
ATOM   1460  O   LEU B 751      27.864  18.861  15.452  1.00 47.16           O
ANISOU 1460  O   LEU B 751     6691   6130   5096  -1242   -214   -304       O
ATOM   1461  CB  LEU B 751      28.428  16.893  13.054  1.00 47.00           C
ANISOU 1461  CB  LEU B 751     7222   5843   4792  -1442   -212   -426       C
ATOM   1462  CG  LEU B 751      28.848  17.708  11.808  1.00 52.49           C
ANISOU 1462  CG  LEU B 751     7900   6558   5488  -1338   -183   -452       C
ATOM   1463  CD1 LEU B 751      30.315  17.463  11.391  1.00 53.21           C
ANISOU 1463  CD1 LEU B 751     8221   6433   5564  -1150    -95   -519       C
ATOM   1464  CD2 LEU B 751      27.869  17.465  10.638  1.00 55.52           C
ANISOU 1464  CD2 LEU B 751     8352   7026   5718  -1577   -253   -448       C
ATOM   1465  H   LEU B 751      28.104  15.525  15.057  1.00 47.49           H
ATOM   1466  HA  LEU B 751      30.271  17.078  14.178  1.00 46.66           H
ATOM   1467  HB3 LEU B 751      27.364  17.051  13.243  1.00 47.00           H
ATOM   1468  HB2 LEU B 751      28.528  15.834  12.810  1.00 47.00           H
ATOM   1469  HG  LEU B 751      28.760  18.762  12.070  1.00 52.49           H
ATOM   1470 HD11 LEU B 751      30.411  17.123  10.360  1.00 53.21           H
ATOM   1471 HD12 LEU B 751      30.889  18.385  11.468  1.00 53.21           H
ATOM   1472 HD13 LEU B 751      30.807  16.715  12.012  1.00 53.21           H
ATOM   1473 HD21 LEU B 751      27.560  18.409  10.192  1.00 55.52           H
ATOM   1474 HD22 LEU B 751      28.303  16.861   9.842  1.00 55.52           H
```

```
ATOM   1475 HD23 LEU B 751      26.962  16.953  10.960  1.00 55.52           H
ATOM   1476  N   LEU B 752      29.940  19.485  14.752  1.00 43.94           N
ANISOU 1476  N   LEU B 752     6322   5586   4788   -926   -111   -368       N
ATOM   1477  CA  LEU B 752      29.949  20.847  15.289  1.00 43.54           C
ANISOU 1477  CA  LEU B 752     6015   5679   4849   -830   -114   -316       C
ATOM   1478  C   LEU B 752      29.378  21.833  14.253  1.00 48.46           C
ANISOU 1478  C   LEU B 752     6513   6454   5447   -857   -141   -286       C
ATOM   1479  O   LEU B 752      30.094  22.717  13.784  1.00 48.58           O
ANISOU 1479  O   LEU B 752     6459   6482   5518   -727   -107   -287       O
ATOM   1480  CB  LEU B 752      31.382  21.211  15.758  1.00 43.40           C
ANISOU 1480  CB  LEU B 752     5956   5593   4941   -624    -52   -327       C
ATOM   1481  CG  LEU B 752      32.032  20.206  16.740  1.00 48.16           C
ANISOU 1481  CG  LEU B 752     6699   6047   5555   -552    -11   -355       C
ATOM   1482  CD1 LEU B 752      33.476  20.614  17.085  1.00 47.99           C
ANISOU 1482  CD1 LEU B 752     6599   6017   5617   -366     44   -350       C
ATOM   1483  CD2 LEU B 752      31.196  19.979  18.010  1.00 50.75           C
ANISOU 1483  CD2 LEU B 752     7031   6366   5886   -642    -43   -334       C
ATOM   1484  H   LEU B 752      30.791  19.193  14.289  1.00 43.94           H
ATOM   1485  HA  LEU B 752      29.294  20.902  16.155  1.00 43.54           H
ATOM   1486  HB3 LEU B 752      31.361  22.198  16.223  1.00 43.40           H
ATOM   1487  HB2 LEU B 752      32.032  21.309  14.888  1.00 43.40           H
ATOM   1488  HG  LEU B 752      32.106  19.241  16.238  1.00 48.16           H
ATOM   1489 HD11 LEU B 752      34.162  19.793  16.878  1.00 47.99           H
ATOM   1490 HD12 LEU B 752      33.813  21.473  16.504  1.00 47.99           H
ATOM   1491 HD13 LEU B 752      33.592  20.878  18.136  1.00 47.99           H
ATOM   1492 HD21 LEU B 752      31.785  19.497  18.790  1.00 50.75           H
ATOM   1493 HD22 LEU B 752      30.810  20.917  18.410  1.00 50.75           H
ATOM   1494 HD23 LEU B 752      30.351  19.320  17.808  1.00 50.75           H
ATOM   1495  N   ASN B 753      28.095  21.634  13.909  1.00 46.22           N
ANISOU 1495  N   ASN B 753     6202   6296   5064  -1037   -203   -251       N
ATOM   1496  CA  ASN B 753      27.375  22.356  12.855  1.00 46.67           C
ANISOU 1496  CA  ASN B 753     6142   6523   5068  -1077   -235   -211       C
ATOM   1497  C   ASN B 753      26.139  23.121  13.369  1.00 52.71           C
ANISOU 1497  C   ASN B 753     6684   7521   5824  -1094   -274   -114       C
ATOM   1498  O   ASN B 753      25.330  23.560  12.551  1.00 53.75           O
ANISOU 1498  O   ASN B 753     6709   7828   5884  -1131   -304    -63       O
ATOM   1499  CB  ASN B 753      27.038  21.383  11.690  1.00 44.92           C
ANISOU 1499  CB  ASN B 753     6076   6291   4700  -1270   -276   -241       C
ATOM   1500  CG  ASN B 753      25.971  20.314  11.994  1.00 62.41           C
ANISOU 1500  CG  ASN B 753     8377   8539   6798  -1516   -339   -223       C
ATOM   1501  ND2 ASN B 753      25.231  19.893  10.966  1.00 55.47           N
ANISOU 1501  ND2 ASN B 753     7622   7685   5768  -1724   -389   -234       N
ATOM   1502  OD1 ASN B 753      25.822  19.860  13.127  1.00 56.32           O
ANISOU 1502  OD1 ASN B 753     7571   7770   6057  -1541   -346   -197       O
ATOM   1503  H   ASN B 753      27.588  20.868  14.332  1.00 46.22           H
ATOM   1504  HA  ASN B 753      27.998  23.151  12.452  1.00 46.67           H
ATOM   1505  HB3 ASN B 753      27.946  20.876  11.365  1.00 44.92           H
ATOM   1506  HB2 ASN B 753      26.705  21.965  10.829  1.00 44.92           H
ATOM   1507 HD22 ASN B 753      24.521  19.190  11.111  1.00 55.47           H
ATOM   1508 HD21 ASN B 753      25.368  20.279  10.043  1.00 55.47           H
ATOM   1509  N   ASN B 754      26.007  23.268  14.696  1.00 47.93           N
ANISOU 1509  N   ASN B 754     6008   6927   5276  -1051   -267    -83       N
ATOM   1510  CA  ASN B 754      24.860  23.903  15.346  1.00 46.95           C
ANISOU 1510  CA  ASN B 754     5681   7026   5130  -1033   -290     16       C
ATOM   1511  C   ASN B 754      25.283  24.564  16.669  1.00 48.73           C
ANISOU 1511  C   ASN B 754     5843   7206   5468   -854   -246    33       C
```

```
ATOM   1512  O   ASN B 754      26.350  24.255  17.202  1.00 47.36           O
ANISOU 1512  O   ASN B 754     5771   6839   5384   -784   -210    -30       O
ATOM   1513  CB  ASN B 754      23.699  22.883  15.499  1.00 44.72           C
ANISOU 1513  CB  ASN B 754     5371   6897   4725  -1267   -357     69       C
ATOM   1514  CG  ASN B 754      24.009  21.687  16.407  1.00 62.76           C
ANISOU 1514  CG  ASN B 754     7816   9016   7015  -1383   -362     21       C
ATOM   1515  ND2 ASN B 754      24.370  20.542  15.823  1.00 53.24           N
ANISOU 1515  ND2 ASN B 754     6789   7737   5702  -1597   -401    -19       N
ATOM   1516  OD1 ASN B 754      23.921  21.796  17.627  1.00 56.84           O
ANISOU 1516  OD1 ASN B 754     7050   8194   6352  -1285   -332     20       O
ATOM   1517  H   ASN B 754      26.723  22.914  15.314  1.00 47.93           H
ATOM   1518  HA  ASN B 754      24.510  24.717  14.708  1.00 46.95           H
ATOM   1519  HB3 ASN B 754      23.399  22.515  14.517  1.00 44.72           H
ATOM   1520  HB2 ASN B 754      22.820  23.388  15.901  1.00 44.72           H
ATOM   1521 HD22 ASN B 754      24.576  19.734  16.392  1.00 53.24           H
ATOM   1522 HD21 ASN B 754      24.462  20.475  14.818  1.00 53.24           H
ATOM   1523  N   ALA B 755      24.418  25.465  17.166  1.00 44.02           N
ANISOU 1523  N   ALA B 755     5081   6799   4846   -774   -248    125       N
ATOM   1524  CA  ALA B 755      24.627  26.258  18.380  1.00 42.49           C
ANISOU 1524  CA  ALA B 755     4839   6576   4727   -605   -207    150       C
ATOM   1525  C   ALA B 755      24.768  25.432  19.671  1.00 43.40           C
ANISOU 1525  C   ALA B 755     5003   6599   4888   -664   -210    128       C
ATOM   1526  O   ALA B 755      25.570  25.813  20.523  1.00 43.28           O
ANISOU 1526  O   ALA B 755     5040   6437   4967   -551   -173     92       O
ATOM   1527  CB  ALA B 755      23.487  27.278  18.516  1.00 43.10           C
ANISOU 1527  CB  ALA B 755     4751   6895   4729   -499   -203    265       C
ATOM   1528  H   ALA B 755      23.558  25.648  16.670  1.00 44.02           H
ATOM   1529  HA  ALA B 755      25.558  26.813  18.247  1.00 42.49           H
ATOM   1530  HB1 ALA B 755      23.626  27.911  19.393  1.00 43.10           H
ATOM   1531  HB2 ALA B 755      23.443  27.935  17.646  1.00 43.10           H
ATOM   1532  HB3 ALA B 755      22.518  26.786  18.612  1.00 43.10           H
ATOM   1533  N   ASP B 756      24.023  24.317  19.778  1.00 38.28           N
ANISOU 1533  N   ASP B 756     4347   6037   4160   -856   -257    151       N
ATOM   1534  CA  ASP B 756      24.060  23.394  20.922  1.00 38.18           C
ANISOU 1534  CA  ASP B 756     4394   5944   4170   -935   -264    137       C
ATOM   1535  C   ASP B 756      25.396  22.638  21.018  1.00 38.99           C
ANISOU 1535  C   ASP B 756     4689   5767   4357   -914   -235     33       C
ATOM   1536  O   ASP B 756      25.907  22.467  22.124  1.00 36.18           O
ANISOU 1536  O   ASP B 756     4362   5312   4071   -848   -212     19       O
ATOM   1537  CB  ASP B 756      22.887  22.378  20.951  1.00 41.09           C
ANISOU 1537  CB  ASP B 756     4747   6458   4407  -1191   -327    185       C
ATOM   1538  CG  ASP B 756      21.458  22.943  20.839  1.00 58.70           C
ANISOU 1538  CG  ASP B 756     6751   9010   6541  -1218   -354    316       C
ATOM   1539  OD1 ASP B 756      21.256  24.151  21.102  1.00 59.83           O
ANISOU 1539  OD1 ASP B 756     6761   9253   6717  -1001   -313    372       O
ATOM   1540  OD2 ASP B 756      20.553  22.115  20.594  1.00 65.94           O1-
ANISOU 1540  OD2 ASP B 756     7634  10087   7332  -1456   -413    371       O1-
ATOM   1541  H   ASP B 756      23.382  24.072  19.037  1.00 38.28           H
ATOM   1542  HA  ASP B 756      23.981  24.005  21.824  1.00 38.18           H
ATOM   1543  HB3 ASP B 756      22.928  21.815  21.886  1.00 41.09           H
ATOM   1544  HB2 ASP B 756      23.016  21.654  20.145  1.00 41.09           H
ATOM   1545  N   SER B 757      25.946  22.225  19.863  1.00 36.46           N
ANISOU 1545  N   SER B 757     4498   5338   4017   -963   -235    -31       N
ATOM   1546  CA  SER B 757      27.243  21.555  19.753  1.00 35.19           C
ANISOU 1546  CA  SER B 757     4521   4944   3906   -916   -199   -117       C
ATOM   1547  C   SER B 757      28.429  22.484  20.078  1.00 35.67           C
```

```
ANISOU 1547  C   SER B 757     4541   4929   4082   -710   -147   -137       C
ATOM   1548  O   SER B 757     29.359  22.042  20.750  1.00 35.53           O
ANISOU 1548  O   SER B 757     4601   4779   4119   -644   -117   -171       O
ATOM   1549  CB  SER B 757     27.397  20.942  18.348  1.00 39.24           C
ANISOU 1549  CB  SER B 757     5175   5392   4342  -1002   -206   -167       C
ATOM   1550  OG  SER B 757     26.676  19.734  18.229  1.00 51.59           O
ANISOU 1550  OG  SER B 757     6852   6966   5784  -1226   -255   -163       O
ATOM   1551  H   SER B 757     25.443  22.369  18.997  1.00 36.46           H
ATOM   1552  HA  SER B 757     27.270  20.745  20.484  1.00 35.19           H
ATOM   1553  HB3 SER B 757     28.440  20.694  18.170  1.00 39.24           H
ATOM   1554  HB2 SER B 757     27.092  21.637  17.565  1.00 39.24           H
ATOM   1555  HG  SER B 757     26.885  19.341  17.376  1.00 51.59           H
ATOM   1556  N   VAL B 758     28.361  23.752  19.630  1.00 30.63           N
ANISOU 1556  N   VAL B 758     3791   4381   3466   -618   -138   -110       N
ATOM   1557  CA  VAL B 758     29.345  24.798  19.937  1.00 30.40           C
ANISOU 1557  CA  VAL B 758     3736   4292   3522   -461    -97   -120       C
ATOM   1558  C   VAL B 758     29.334  25.188  21.430  1.00 32.68           C
ANISOU 1558  C   VAL B 758     3980   4573   3862   -403    -91    -93       C
ATOM   1559  O   VAL B 758     30.409  25.343  22.007  1.00 31.87           O
ANISOU 1559  O   VAL B 758     3916   4370   3823   -336    -66   -119       O
ATOM   1560  CB  VAL B 758     29.128  26.065  19.055  1.00 34.26           C
ANISOU 1560  CB  VAL B 758     4154   4866   3998   -394    -91    -91       C
ATOM   1561  CG1 VAL B 758     29.887  27.336  19.502  1.00 33.59           C
ANISOU 1561  CG1 VAL B 758     4056   4731   3975   -267    -59    -84       C
ATOM   1562  CG2 VAL B 758     29.480  25.766  17.586  1.00 34.44           C
ANISOU 1562  CG2 VAL B 758     4235   4866   3984   -431    -88   -129       C
ATOM   1563  H   VAL B 758     27.565  24.040  19.076  1.00 30.63           H
ATOM   1564  HA  VAL B 758     30.335  24.393  19.715  1.00 30.40           H
ATOM   1565  HB  VAL B 758     28.064  26.308  19.080  1.00 34.26           H
ATOM   1566 HG11 VAL B 758     29.770  28.140  18.774  1.00 33.59           H
ATOM   1567 HG12 VAL B 758     29.521  27.726  20.453  1.00 33.59           H
ATOM   1568 HG13 VAL B 758     30.955  27.143  19.604  1.00 33.59           H
ATOM   1569 HG21 VAL B 758     29.232  26.614  16.947  1.00 34.44           H
ATOM   1570 HG22 VAL B 758     30.545  25.563  17.469  1.00 34.44           H
ATOM   1571 HG23 VAL B 758     28.936  24.903  17.204  1.00 34.44           H
ATOM   1572  N   GLN B 759     28.131  25.284  22.026  1.00 27.52           N
ANISOU 1572  N   GLN B 759     3240   4048   3168   -433   -113    -33       N
ATOM   1573  CA  GLN B 759     27.909  25.512  23.455  1.00 26.65           C
ANISOU 1573  CA  GLN B 759     3086   3955   3087   -379   -107      3       C
ATOM   1574  C   GLN B 759     28.489  24.392  24.338  1.00 31.20           C
ANISOU 1574  C   GLN B 759     3744   4409   3702   -427   -106    -36       C
ATOM   1575  O   GLN B 759     29.201  24.698  25.290  1.00 30.01           O
ANISOU 1575  O   GLN B 759     3608   4183   3614   -343    -85    -46       O
ATOM   1576  CB  GLN B 759     26.404  25.763  23.708  1.00 26.95           C
ANISOU 1576  CB  GLN B 759     3005   4189   3044   -410   -129     86       C
ATOM   1577  CG  GLN B 759     25.986  25.808  25.195  1.00 47.56           C
ANISOU 1577  CG  GLN B 759     5561   6841   5668   -305   -109    136       C
ATOM   1578  CD  GLN B 759     24.504  26.113  25.419  1.00 71.89           C
ANISOU 1578  CD  GLN B 759     8513  10145   8657   -344   -127    229       C
ATOM   1579  NE2 GLN B 759     24.063  25.989  26.672  1.00 64.09           N
ANISOU 1579  NE2 GLN B 759     7504   9171   7674   -358   -127    254       N
ATOM   1580  OE1 GLN B 759     23.767  26.456  24.496  1.00 67.11           O
ANISOU 1580  OE1 GLN B 759     7813   9721   7965   -360   -142    288       O
ATOM   1581  H   GLN B 759     27.295  25.162  21.470  1.00 27.52           H
ATOM   1582  HA  GLN B 759     28.439  26.429  23.718  1.00 26.65           H
ATOM   1583  HB3 GLN B 759     25.819  24.991  23.207  1.00 26.95           H
```

```
ATOM   1584  HB2 GLN B 759      26.130  26.705  23.230  1.00 26.95           H
ATOM   1585  HG3 GLN B 759      26.578  26.552  25.729  1.00 47.56           H
ATOM   1586  HG2 GLN B 759      26.186  24.849  25.672  1.00 47.56           H
ATOM   1587 HE22 GLN B 759      23.096  26.173  26.893  1.00 64.09           H
ATOM   1588 HE21 GLN B 759      24.702  25.712  27.408  1.00 64.09           H
ATOM   1589  N   ALA B 760      28.192  23.128  23.990  1.00 27.42           N
ANISOU 1589  N   ALA B 760     3340   3907   3171   -566   -130    -56       N
ATOM   1590  CA  ALA B 760      28.643  21.934  24.710  1.00 26.88           C
ANISOU 1590  CA  ALA B 760     3390   3709   3115   -609   -125    -89       C
ATOM   1591  C   ALA B 760      30.172  21.768  24.751  1.00 30.15           C
ANISOU 1591  C   ALA B 760     3882   3979   3596   -487    -84   -141       C
ATOM   1592  O   ALA B 760      30.698  21.343  25.780  1.00 31.53           O
ANISOU 1592  O   ALA B 760     4075   4090   3816   -428    -68   -143       O
ATOM   1593  CB  ALA B 760      27.980  20.696  24.088  1.00 27.43           C
ANISOU 1593  CB  ALA B 760     3582   3752   3087   -786   -154   -106       C
ATOM   1594  H   ALA B 760      27.582  22.963  23.200  1.00 27.42           H
ATOM   1595  HA  ALA B 760      28.294  22.024  25.740  1.00 26.88           H
ATOM   1596  HB1 ALA B 760      28.289  19.783  24.599  1.00 27.43           H
ATOM   1597  HB2 ALA B 760      26.894  20.757  24.159  1.00 27.43           H
ATOM   1598  HB3 ALA B 760      28.238  20.591  23.033  1.00 27.43           H
ATOM   1599  N   LYS B 761      30.854  22.131  23.651  1.00 26.07           N
ANISOU 1599  N   LYS B 761     3396   3434   3077   -446    -66   -173       N
ATOM   1600  CA  LYS B 761      32.313  22.129  23.563  1.00 24.66           C
ANISOU 1600  CA  LYS B 761     3261   3169   2941   -329    -24   -204       C
ATOM   1601  C   LYS B 761      32.966  23.364  24.206  1.00 26.45           C
ANISOU 1601  C   LYS B 761     3381   3431   3236   -233    -14   -181       C
ATOM   1602  O   LYS B 761      34.081  23.231  24.703  1.00 25.97           O
ANISOU 1602  O   LYS B 761     3334   3328   3207   -160     10   -186       O
ATOM   1603  CB  LYS B 761      32.755  21.963  22.097  1.00 25.96           C
ANISOU 1603  CB  LYS B 761     3485   3313   3068   -315     -5   -236       C
ATOM   1604  CG  LYS B 761      32.442  20.594  21.463  1.00 34.36           C
ANISOU 1604  CG  LYS B 761     4718   4296   4042   -402     -7   -270       C
ATOM   1605  CD  LYS B 761      32.874  19.372  22.292  1.00 38.43           C
ANISOU 1605  CD  LYS B 761     5390   4680   4533   -363     21   -290       C
ATOM   1606  CE  LYS B 761      32.765  18.064  21.496  1.00 55.60           C
ANISOU 1606  CE  LYS B 761     7784   6750   6592   -473     16   -324       C
ATOM   1607  NZ  LYS B 761      33.057  16.884  22.329  1.00 67.57           N1+
ANISOU 1607  NZ  LYS B 761     9485   8122   8065   -454     40   -336       N1+
ATOM   1608  H   LYS B 761      30.353  22.461  22.838  1.00 26.07           H
ATOM   1609  HA  LYS B 761      32.689  21.278  24.131  1.00 24.66           H
ATOM   1610  HB3 LYS B 761      33.832  22.115  22.036  1.00 25.96           H
ATOM   1611  HB2 LYS B 761      32.305  22.748  21.489  1.00 25.96           H
ATOM   1612  HG3 LYS B 761      32.939  20.560  20.494  1.00 34.36           H
ATOM   1613  HG2 LYS B 761      31.376  20.520  21.258  1.00 34.36           H
ATOM   1614  HD3 LYS B 761      32.240  19.299  23.177  1.00 38.43           H
ATOM   1615  HD2 LYS B 761      33.894  19.513  22.650  1.00 38.43           H
ATOM   1616  HE3 LYS B 761      33.457  18.083  20.653  1.00 55.60           H
ATOM   1617  HE2 LYS B 761      31.759  17.955  21.087  1.00 55.60           H
ATOM   1618  HZ1 LYS B 761      32.414  16.847  23.106  1.00 67.57           H
ATOM   1619  HZ2 LYS B 761      32.956  16.047  21.771  1.00 67.57           H
ATOM   1620  HZ3 LYS B 761      34.004  16.933  22.681  1.00 67.57           H
ATOM   1621  N   ALA B 762      32.276  24.518  24.231  1.00 23.50           N
ANISOU 1621  N   ALA B 762     2919   3140   2871   -233    -29   -149       N
ATOM   1622  CA  ALA B 762      32.730  25.717  24.945  1.00 23.60           C
ANISOU 1622  CA  ALA B 762     2881   3162   2922   -164    -22   -128       C
ATOM   1623  C   ALA B 762      32.726  25.510  26.470  1.00 26.83           C
```

ANISOU 1623 C ALA B 762 3286 3547 3362 -147 -26 -113 C
ATOM 1624 O ALA B 762 33.698 25.883 27.123 1.00 26.67 O
ANISOU 1624 O ALA B 762 3262 3502 3370 -106 -19 -110 O
ATOM 1625 CB ALA B 762 31.878 26.929 24.537 1.00 24.45 C
ANISOU 1625 CB ALA B 762 2947 3340 3004 -148 -29 -96 C
ATOM 1626 H ALA B 762 31.367 24.575 23.791 1.00 23.50 H
ATOM 1627 HA ALA B 762 33.758 25.920 24.639 1.00 23.60 H
ATOM 1628 HB1 ALA B 762 32.219 27.836 25.037 1.00 24.45 H
ATOM 1629 HB2 ALA B 762 31.944 27.110 23.464 1.00 24.45 H
ATOM 1630 HB3 ALA B 762 30.826 26.791 24.787 1.00 24.45 H
ATOM 1631 N GLU B 763 31.673 24.844 26.979 1.00 23.38 N
ANISOU 1631 N GLU B 763 2851 3127 2906 -195 -41 -100 N
ATOM 1632 CA GLU B 763 31.528 24.390 28.363 1.00 23.29 C
ANISOU 1632 CA GLU B 763 2840 3095 2914 -190 -45 -84 C
ATOM 1633 C GLU B 763 32.583 23.350 28.783 1.00 27.82 C
ANISOU 1633 C GLU B 763 3482 3578 3512 -168 -30 -112 C
ATOM 1634 O GLU B 763 33.069 23.429 29.909 1.00 27.50 O
ANISOU 1634 O GLU B 763 3429 3517 3502 -122 -26 -102 O
ATOM 1635 CB GLU B 763 30.101 23.841 28.571 1.00 24.77 C
ANISOU 1635 CB GLU B 763 3003 3352 3057 -268 -66 -52 C
ATOM 1636 CG GLU B 763 29.008 24.938 28.559 1.00 38.71 C
ANISOU 1636 CG GLU B 763 4676 5245 4787 -236 -70 2 C
ATOM 1637 CD GLU B 763 27.554 24.431 28.530 1.00 57.85 C
ANISOU 1637 CD GLU B 763 7031 7812 7136 -323 -93 55 C
ATOM 1638 OE1 GLU B 763 27.334 23.201 28.448 1.00 48.60 O
ANISOU 1638 OE1 GLU B 763 5900 6630 5936 -451 -115 46 O
ATOM 1639 OE2 GLU B 763 26.660 25.305 28.586 1.00 47.71 O1-
ANISOU 1639 OE2 GLU B 763 5660 6662 5807 -264 -88 112 O1-
ATOM 1640 H GLU B 763 30.911 24.597 26.361 1.00 23.38 H
ATOM 1641 HA GLU B 763 31.657 25.258 29.011 1.00 23.29 H
ATOM 1642 HB3 GLU B 763 30.044 23.290 29.511 1.00 24.77 H
ATOM 1643 HB2 GLU B 763 29.898 23.113 27.784 1.00 24.77 H
ATOM 1644 HG3 GLU B 763 29.148 25.600 27.705 1.00 38.71 H
ATOM 1645 HG2 GLU B 763 29.134 25.567 29.441 1.00 38.71 H
ATOM 1646 N MET B 764 32.949 22.424 27.875 1.00 26.33 N
ANISOU 1646 N MET B 764 3376 3336 3294 -189 -18 -143 N
ATOM 1647 CA MET B 764 34.013 21.435 28.083 1.00 25.86 C
ANISOU 1647 CA MET B 764 3399 3194 3231 -124 12 -161 C
ATOM 1648 C MET B 764 35.388 22.088 28.320 1.00 25.69 C
ANISOU 1648 C MET B 764 3308 3211 3243 -25 31 -150 C
ATOM 1649 O MET B 764 36.097 21.671 29.234 1.00 23.65 O
ANISOU 1649 O MET B 764 3046 2944 2996 38 43 -134 O
ATOM 1650 CB MET B 764 34.045 20.444 26.896 1.00 29.19 C
ANISOU 1650 CB MET B 764 3959 3543 3589 -144 30 -194 C
ATOM 1651 CG MET B 764 35.148 19.370 26.962 1.00 34.87 C
ANISOU 1651 CG MET B 764 4774 4191 4283 -15 80 -204 C
ATOM 1652 SD MET B 764 35.294 18.342 25.476 1.00 41.91 S
ANISOU 1652 SD MET B 764 5831 5006 5086 18 120 -242 S
ATOM 1653 CE MET B 764 36.240 19.474 24.423 1.00 38.16 C
ANISOU 1653 CE MET B 764 5192 4659 4649 40 117 -236 C
ATOM 1654 H MET B 764 32.493 22.409 26.974 1.00 26.33 H
ATOM 1655 HA MET B 764 33.763 20.870 28.983 1.00 25.86 H
ATOM 1656 HB3 MET B 764 34.160 20.999 25.969 1.00 29.19 H
ATOM 1657 HB2 MET B 764 33.078 19.947 26.824 1.00 29.19 H
ATOM 1658 HG3 MET B 764 34.968 18.721 27.815 1.00 34.87 H
ATOM 1659 HG2 MET B 764 36.127 19.813 27.130 1.00 34.87 H

```
ATOM   1660  HE1 MET B 764      36.433  19.009  23.457  1.00 38.16           H
ATOM   1661  HE2 MET B 764      35.689  20.400  24.260  1.00 38.16           H
ATOM   1662  HE3 MET B 764      37.198  19.714  24.883  1.00 38.16           H
ATOM   1663  N   LEU B 765      35.720  23.103  27.505  1.00 21.70           N
ANISOU 1663  N   LEU B 765     2744   2761   2740    -26     31   -150       N
ATOM   1664  CA  LEU B 765      36.982  23.842  27.570  1.00 21.21           C
ANISOU 1664  CA  LEU B 765     2611   2760   2688     23     41   -130       C
ATOM   1665  C   LEU B 765      37.136  24.701  28.838  1.00 25.24           C
ANISOU 1665  C   LEU B 765     3068   3297   3225      8     18   -102       C
ATOM   1666  O   LEU B 765      38.271  24.869  29.281  1.00 25.91           O
ANISOU 1666  O   LEU B 765     3110   3429   3305     40     22    -76       O
ATOM   1667  CB  LEU B 765      37.148  24.695  26.299  1.00 20.42           C
ANISOU 1667  CB  LEU B 765     2480   2706   2572      2     44   -134       C
ATOM   1668  CG  LEU B 765      37.360  23.881  25.006  1.00 23.99           C
ANISOU 1668  CG  LEU B 765     2990   3140   2986     25     71   -161       C
ATOM   1669  CD1 LEU B 765      37.361  24.826  23.799  1.00 23.06           C
ANISOU 1669  CD1 LEU B 765     2836   3070   2856     -7     68   -161       C
ATOM   1670  CD2 LEU B 765      38.622  23.000  25.040  1.00 25.79           C
ANISOU 1670  CD2 LEU B 765     3243   3375   3180    134    117   -152       C
ATOM   1671  H   LEU B 765      35.074  23.393  26.783  1.00 21.70           H
ATOM   1672  HA  LEU B 765      37.787  23.107  27.601  1.00 21.21           H
ATOM   1673  HB3 LEU B 765      37.990  25.377  26.418  1.00 20.42           H
ATOM   1674  HB2 LEU B 765      36.267  25.329  26.186  1.00 20.42           H
ATOM   1675  HG  LEU B 765      36.521  23.203  24.876  1.00 23.99           H
ATOM   1676 HD11 LEU B 765      37.477  24.276  22.868  1.00 23.06           H
ATOM   1677 HD12 LEU B 765      36.428  25.386  23.735  1.00 23.06           H
ATOM   1678 HD13 LEU B 765      38.177  25.544  23.867  1.00 23.06           H
ATOM   1679 HD21 LEU B 765      39.080  22.904  24.058  1.00 25.79           H
ATOM   1680 HD22 LEU B 765      39.378  23.392  25.719  1.00 25.79           H
ATOM   1681 HD23 LEU B 765      38.374  21.991  25.370  1.00 25.79           H
ATOM   1682  N   ASP B 766      36.018  25.175  29.426  1.00 21.13           N
ANISOU 1682  N   ASP B 766     2552   2759   2715    -36     -6    -99       N
ATOM   1683  CA  ASP B 766      35.984  25.841  30.738  1.00 21.33           C
ANISOU 1683  CA  ASP B 766     2569   2786   2751    -44    -24    -77       C
ATOM   1684  C   ASP B 766      36.462  24.912  31.869  1.00 24.85           C
ANISOU 1684  C   ASP B 766     3013   3216   3212    -13    -23    -68       C
ATOM   1685  O   ASP B 766      37.255  25.336  32.710  1.00 23.63           O
ANISOU 1685  O   ASP B 766     2834   3090   3054    -13    -34    -46       O
ATOM   1686  CB  ASP B 766      34.590  26.398  31.137  1.00 22.95           C
ANISOU 1686  CB  ASP B 766     2789   2983   2947    -54    -33    -68       C
ATOM   1687  CG  ASP B 766      33.878  27.346  30.159  1.00 34.63           C
ANISOU 1687  CG  ASP B 766     4279   4483   4394    -53    -32    -60       C
ATOM   1688  OD1 ASP B 766      34.549  27.970  29.308  1.00 34.54           O
ANISOU 1688  OD1 ASP B 766     4270   4480   4374    -68    -28    -69       O
ATOM   1689  OD2 ASP B 766      32.661  27.535  30.367  1.00 39.86           O1-
ANISOU 1689  OD2 ASP B 766     4948   5166   5032    -26    -31    -36       O1-
ATOM   1690  H   ASP B 766      35.122  25.026  28.982  1.00 21.13           H
ATOM   1691  HA  ASP B 766      36.683  26.679  30.694  1.00 21.33           H
ATOM   1692  HB3 ASP B 766      34.681  26.937  32.083  1.00 22.95           H
ATOM   1693  HB2 ASP B 766      33.911  25.563  31.322  1.00 22.95           H
ATOM   1694  N   ASN B 767      35.974  23.660  31.851  1.00 23.72           N
ANISOU 1694  N   ASN B 767     2910   3030   3071      3    -10    -81       N
ATOM   1695  CA  ASN B 767      36.293  22.623  32.835  1.00 24.15           C
ANISOU 1695  CA  ASN B 767     2993   3054   3129     46     -1    -72       C
ATOM   1696  C   ASN B 767      37.718  22.073  32.665  1.00 29.00           C
ANISOU 1696  C   ASN B 767     3587   3706   3724    128     24    -56       C
```

```
ATOM   1697  O   ASN B 767      38.354  21.802  33.682  1.00 27.56           O
ANISOU 1697  O   ASN B 767     3374   3557   3540    168     21    -27       O
ATOM   1698  CB  ASN B 767      35.224  21.509  32.773  1.00 24.86           C
ANISOU 1698  CB  ASN B 767     3168   3073   3204     19      5    -87       C
ATOM   1699  CG  ASN B 767      33.890  21.937  33.396  1.00 41.61           C
ANISOU 1699  CG  ASN B 767     5272   5209   5331    -50    -19    -74       C
ATOM   1700  ND2 ASN B 767      32.969  22.480  32.601  1.00 35.72           N
ANISOU 1700  ND2 ASN B 767     4516   4492   4563   -112    -29    -78       N
ATOM   1701  OD1 ASN B 767      33.686  21.777  34.595  1.00 36.74           O
ANISOU 1701  OD1 ASN B 767     4641   4593   4728    -41    -27    -54       O
ATOM   1702  H   ASN B 767      35.336  23.395  31.113  1.00 23.72           H
ATOM   1703  HA  ASN B 767      36.225  23.117  33.805  1.00 24.15           H
ATOM   1704  HB3 ASN B 767      35.567  20.642  33.342  1.00 24.86           H
ATOM   1705  HB2 ASN B 767      35.075  21.158  31.751  1.00 24.86           H
ATOM   1706 HD22 ASN B 767      32.087  22.783  32.987  1.00 35.72           H
ATOM   1707 HD21 ASN B 767      33.150  22.629  31.615  1.00 35.72           H
ATOM   1708  N   LEU B 768      38.222  21.973  31.419  1.00 26.19           N
ANISOU 1708  N   LEU B 768     3241   3367   3341    163     50    -67       N
ATOM   1709  CA  LEU B 768      39.617  21.617  31.131  1.00 25.66           C
ANISOU 1709  CA  LEU B 768     3138   3375   3235    268     84    -37       C
ATOM   1710  C   LEU B 768      40.621  22.676  31.619  1.00 29.03           C
ANISOU 1710  C   LEU B 768     3433   3938   3657    246     61     12       C
ATOM   1711  O   LEU B 768      41.737  22.302  31.971  1.00 27.75           O
ANISOU 1711  O   LEU B 768     3216   3866   3461    328     77     59       O
ATOM   1712  CB  LEU B 768      39.829  21.366  29.620  1.00 25.57           C
ANISOU 1712  CB  LEU B 768     3162   3366   3187    310    119    -54       C
ATOM   1713  CG  LEU B 768      39.220  20.058  29.068  1.00 30.34           C
ANISOU 1713  CG  LEU B 768     3934   3838   3757    339    149    -96       C
ATOM   1714  CD1 LEU B 768      39.391  19.990  27.534  1.00 29.82           C
ANISOU 1714  CD1 LEU B 768     3907   3782   3640    390    186   -110       C
ATOM   1715  CD2 LEU B 768      39.773  18.797  29.771  1.00 33.14           C
ANISOU 1715  CD2 LEU B 768     4397   4124   4070    451    185    -82       C
ATOM   1716  H   LEU B 768      37.638  22.194  30.623  1.00 26.19           H
ATOM   1717  HA  LEU B 768      39.838  20.701  31.678  1.00 25.66           H
ATOM   1718  HB3 LEU B 768      40.898  21.342  29.403  1.00 25.57           H
ATOM   1719  HB2 LEU B 768      39.439  22.219  29.064  1.00 25.57           H
ATOM   1720  HG  LEU B 768      38.148  20.085  29.264  1.00 30.34           H
ATOM   1721 HD11 LEU B 768      38.430  19.848  27.043  1.00 29.82           H
ATOM   1722 HD12 LEU B 768      39.823  20.906  27.130  1.00 29.82           H
ATOM   1723 HD13 LEU B 768      40.039  19.175  27.214  1.00 29.82           H
ATOM   1724 HD21 LEU B 768      40.051  18.008  29.073  1.00 33.14           H
ATOM   1725 HD22 LEU B 768      40.660  19.013  30.366  1.00 33.14           H
ATOM   1726 HD23 LEU B 768      39.025  18.374  30.444  1.00 33.14           H
ATOM   1727  N   LEU B 769      40.220  23.959  31.636  1.00 26.79           N
ANISOU 1727  N   LEU B 769     3115   3671   3391    132     23      6       N
ATOM   1728  CA  LEU B 769      41.059  25.084  32.052  1.00 26.85           C
ANISOU 1728  CA  LEU B 769     3045   3781   3375     55    -11     47       C
ATOM   1729  C   LEU B 769      41.330  25.093  33.572  1.00 30.85           C
ANISOU 1729  C   LEU B 769     3535   4307   3881     57    -33     75       C
ATOM   1730  O   LEU B 769      42.454  25.399  33.967  1.00 30.33           O
ANISOU 1730  O   LEU B 769     3378   4378   3767     47    -45    130       O
ATOM   1731  CB  LEU B 769      40.415  26.395  31.542  1.00 27.06           C
ANISOU 1731  CB  LEU B 769     3121   3750   3410    -54    -40     23       C
ATOM   1732  CG  LEU B 769      41.338  27.630  31.502  1.00 33.00           C
ANISOU 1732  CG  LEU B 769     3848   4572   4119   -140    -52     41       C
ATOM   1733  CD1 LEU B 769      42.515  27.468  30.517  1.00 33.15           C
```

```
ANISOU 1733  CD1 LEU B 769     3770   4724   4103    -96    -25     74        C
ATOM   1734  CD2 LEU B 769      40.519  28.894  31.184  1.00 34.35           C
ANISOU 1734  CD2 LEU B 769     4099   4651   4301   -170    -52      5        C
ATOM   1735  H   LEU B 769      39.288  24.192  31.322  1.00 26.79           H
ATOM   1736  HA  LEU B 769      42.023  24.956  31.559  1.00 26.85           H
ATOM   1737  HB3 LEU B 769      39.537  26.618  32.150  1.00 27.06           H
ATOM   1738  HB2 LEU B 769      40.039  26.243  30.531  1.00 27.06           H
ATOM   1739  HG  LEU B 769      41.760  27.771  32.497  1.00 33.00           H
ATOM   1740 HD11 LEU B 769      42.567  28.288  29.800  1.00 33.15           H
ATOM   1741 HD12 LEU B 769      43.464  27.444  31.053  1.00 33.15           H
ATOM   1742 HD13 LEU B 769      42.450  26.548  29.938  1.00 33.15           H
ATOM   1743 HD21 LEU B 769      41.147  29.785  31.191  1.00 34.35           H
ATOM   1744 HD22 LEU B 769      40.044  28.825  30.205  1.00 34.35           H
ATOM   1745 HD23 LEU B 769      39.732  29.047  31.923  1.00 34.35           H
ATOM   1746  N   ASP B 770      40.330  24.703  34.384  1.00 26.88           N
ANISOU 1746  N   ASP B 770     3106   3689   3417     65    -38     46        N
ATOM   1747  CA  ASP B 770      40.471  24.494  35.833  1.00 26.77           C
ANISOU 1747  CA  ASP B 770     3090   3678   3405     72    -56     68        C
ATOM   1748  C   ASP B 770      41.171  23.167  36.183  1.00 31.21           C
ANISOU 1748  C   ASP B 770     3624   4285   3948    193    -25     99        C
ATOM   1749  O   ASP B 770      41.891  23.139  37.181  1.00 30.17           O
ANISOU 1749  O   ASP B 770     3435   4239   3789    206    -41    144        O
ATOM   1750  CB  ASP B 770      39.129  24.610  36.602  1.00 28.21           C
ANISOU 1750  CB  ASP B 770     3354   3741   3625     52    -64     36        C
ATOM   1751  CG  ASP B 770      38.423  25.978  36.538  1.00 37.10           C
ANISOU 1751  CG  ASP B 770     4520   4835   4740    -30    -92     27        C
ATOM   1752  OD1 ASP B 770      39.125  27.008  36.434  1.00 38.74           O
ANISOU 1752  OD1 ASP B 770     4719   5094   4906   -100   -119     48        O
ATOM   1753  OD2 ASP B 770      37.196  25.992  36.776  1.00 40.53           O1-
ANISOU 1753  OD2 ASP B 770     5006   5201   5192    -23    -85      7        O1-
ATOM   1754  H   ASP B 770      39.427  24.482  33.988  1.00 26.88           H
ATOM   1755  HA  ASP B 770      41.131  25.277  36.212  1.00 26.77           H
ATOM   1756  HB3 ASP B 770      39.295  24.393  37.657  1.00 28.21           H
ATOM   1757  HB2 ASP B 770      38.439  23.854  36.223  1.00 28.21           H
ATOM   1758  N   ILE B 771      40.987  22.112  35.366  1.00 29.24           N
ANISOU 1758  N   ILE B 771     3434   3978   3699    287     21     79        N
ATOM   1759  CA  ILE B 771      41.677  20.819  35.505  1.00 29.48           C
ANISOU 1759  CA  ILE B 771     3486   4028   3688    439     66    110        C
ATOM   1760  C   ILE B 771      43.185  20.909  35.185  1.00 33.32           C
ANISOU 1760  C   ILE B 771     3834   4716   4110    508     79    180        C
ATOM   1761  O   ILE B 771      43.977  20.253  35.863  1.00 32.47           O
ANISOU 1761  O   ILE B 771     3671   4709   3958    603     90    240        O
ATOM   1762  CB  ILE B 771      40.999  19.705  34.643  1.00 32.56           C
ANISOU 1762  CB  ILE B 771     4028   4277   4066    510    113     67        C
ATOM   1763  CG1 ILE B 771      39.661  19.276  35.289  1.00 33.17           C
ANISOU 1763  CG1 ILE B 771     4221   4201   4179    432     95     26        C
ATOM   1764  CG2 ILE B 771      41.856  18.447  34.354  1.00 33.01           C
ANISOU 1764  CG2 ILE B 771     4139   4357   4047    706    177    107        C
ATOM   1765  CD1 ILE B 771      38.732  18.485  34.353  1.00 39.91           C
ANISOU 1765  CD1 ILE B 771     5242   4912   5008    415    120    -20        C
ATOM   1766  H   ILE B 771      40.357  22.198  34.579  1.00 29.24           H
ATOM   1767  HA  ILE B 771      41.597  20.522  36.553  1.00 29.48           H
ATOM   1768  HB  ILE B 771      40.766  20.151  33.677  1.00 32.56           H
ATOM   1769 HG13 ILE B 771      39.119  20.149  35.654  1.00 33.17           H
ATOM   1770 HG12 ILE B 771      39.874  18.676  36.173  1.00 33.17           H
ATOM   1771 HG21 ILE B 771      41.294  17.693  33.804  1.00 33.01           H
```

ATOM 1772 HG22 ILE B 771 42.731 18.672 33.744 1.00 33.01 H
ATOM 1773 HG23 ILE B 771 42.201 17.989 35.282 1.00 33.01 H
ATOM 1774 HD11 ILE B 771 37.688 18.637 34.628 1.00 39.91 H
ATOM 1775 HD12 ILE B 771 38.845 18.793 33.313 1.00 39.91 H
ATOM 1776 HD13 ILE B 771 38.935 17.415 34.411 1.00 39.91 H
ATOM 1777 N GLU B 772 43.554 21.743 34.194 1.00 30.35 N
ANISOU 1777 N GLU B 772 3390 4422 3721 452 75 183 N
ATOM 1778 CA GLU B 772 44.941 22.044 33.834 1.00 30.45 C
ANISOU 1778 CA GLU B 772 3248 4663 3658 488 83 262 C
ATOM 1779 C GLU B 772 45.669 22.770 34.975 1.00 33.86 C
ANISOU 1779 C GLU B 772 3558 5248 4060 381 25 323 C
ATOM 1780 O GLU B 772 46.768 22.355 35.334 1.00 30.84 O
ANISOU 1780 O GLU B 772 3047 5072 3600 460 36 410 O
ATOM 1781 CB GLU B 772 44.985 22.818 32.493 1.00 31.71 C
ANISOU 1781 CB GLU B 772 3373 4868 3809 420 86 250 C
ATOM 1782 CG GLU B 772 46.403 23.056 31.907 1.00 38.16 C
ANISOU 1782 CG GLU B 772 4026 5941 4531 479 109 341 C
ATOM 1783 CD GLU B 772 47.177 24.300 32.384 1.00 53.53 C
ANISOU 1783 CD GLU B 772 5812 8104 6423 323 48 418 C
ATOM 1784 OE1 GLU B 772 46.542 25.276 32.843 1.00 38.31 O
ANISOU 1784 OE1 GLU B 772 3929 6099 4529 126 -16 384 O
ATOM 1785 OE2 GLU B 772 48.421 24.262 32.261 1.00 51.14 O1-
ANISOU 1785 OE2 GLU B 772 5348 8058 6026 401 66 520 O1-
ATOM 1786 H GLU B 772 42.843 22.222 33.658 1.00 30.35 H
ATOM 1787 HA GLU B 772 45.443 21.087 33.677 1.00 30.45 H
ATOM 1788 HB3 GLU B 772 44.429 23.753 32.579 1.00 31.71 H
ATOM 1789 HB2 GLU B 772 44.430 22.246 31.753 1.00 31.71 H
ATOM 1790 HG3 GLU B 772 46.307 23.152 30.825 1.00 38.16 H
ATOM 1791 HG2 GLU B 772 47.012 22.164 32.067 1.00 38.16 H
ATOM 1792 N VAL B 773 45.016 23.799 35.547 1.00 32.07 N
ANISOU 1792 N VAL B 773 3382 4926 3877 209 -34 283 N
ATOM 1793 CA VAL B 773 45.496 24.559 36.704 1.00 31.45 C
ANISOU 1793 CA VAL B 773 3241 4949 3758 81 -95 327 C
ATOM 1794 C VAL B 773 45.653 23.690 37.967 1.00 35.63 C
ANISOU 1794 C VAL B 773 3743 5521 4273 197 -85 367 C
ATOM 1795 O VAL B 773 46.652 23.850 38.664 1.00 35.70 O
ANISOU 1795 O VAL B 773 3618 5742 4203 184 -107 451 O
ATOM 1796 CB VAL B 773 44.581 25.788 36.999 1.00 34.96 C
ANISOU 1796 CB VAL B 773 3803 5243 4236 -90 -145 269 C
ATOM 1797 CG1 VAL B 773 44.729 26.429 38.398 1.00 34.98 C
ANISOU 1797 CG1 VAL B 773 3801 5300 4191 -205 -202 303 C
ATOM 1798 CG2 VAL B 773 44.792 26.875 35.932 1.00 34.50 C
ANISOU 1798 CG2 VAL B 773 3765 5185 4157 -213 -159 255 C
ATOM 1799 H VAL B 773 44.121 24.082 35.173 1.00 32.07 H
ATOM 1800 HA VAL B 773 46.490 24.930 36.445 1.00 31.45 H
ATOM 1801 HB VAL B 773 43.545 25.457 36.916 1.00 34.96 H
ATOM 1802 HG11 VAL B 773 44.106 27.319 38.480 1.00 34.98 H
ATOM 1803 HG12 VAL B 773 44.418 25.758 39.199 1.00 34.98 H
ATOM 1804 HG13 VAL B 773 45.760 26.730 38.590 1.00 34.98 H
ATOM 1805 HG21 VAL B 773 44.117 27.716 36.086 1.00 34.50 H
ATOM 1806 HG22 VAL B 773 45.810 27.262 35.962 1.00 34.50 H
ATOM 1807 HG23 VAL B 773 44.621 26.494 34.926 1.00 34.50 H
ATOM 1808 N ALA B 774 44.704 22.768 38.209 1.00 32.74 N
ANISOU 1808 N ALA B 774 3500 4970 3970 301 -54 315 N
ATOM 1809 CA ALA B 774 44.723 21.814 39.321 1.00 32.71 C
ANISOU 1809 CA ALA B 774 3503 4972 3953 418 -39 347 C

ATOM 1810 C ALA B 774 45.942 20.876 39.298 1.00 36.76 C
ANISOU 1810 C ALA B 774 3914 5670 4382 603 9 434 C
ATOM 1811 O ALA B 774 46.552 20.660 40.344 1.00 35.59 O
ANISOU 1811 O ALA B 774 3669 5678 4175 644 -6 509 O
ATOM 1812 CB ALA B 774 43.426 20.990 39.307 1.00 33.33 C
ANISOU 1812 CB ALA B 774 3747 4820 4098 479 -9 280 C
ATOM 1813 H ALA B 774 43.905 22.709 37.591 1.00 32.74 H
ATOM 1814 HA ALA B 774 44.755 22.393 40.246 1.00 32.71 H
ATOM 1815 HB1 ALA B 774 43.345 20.379 40.206 1.00 33.33 H
ATOM 1816 HB2 ALA B 774 42.547 21.632 39.267 1.00 33.33 H
ATOM 1817 HB3 ALA B 774 43.376 20.320 38.450 1.00 33.33 H
ATOM 1818 N TYR B 775 46.277 20.363 38.101 1.00 33.87 N
ANISOU 1818 N TYR B 775 3571 5302 3997 726 69 430 N
ATOM 1819 CA TYR B 775 47.405 19.461 37.883 1.00 34.86 C
ANISOU 1819 CA TYR B 775 3616 5605 4022 947 133 518 C
ATOM 1820 C TYR B 775 48.758 20.197 37.858 1.00 39.79 C
ANISOU 1820 C TYR B 775 3996 6565 4556 891 101 627 C
ATOM 1821 O TYR B 775 49.726 19.675 38.408 1.00 39.02 O
ANISOU 1821 O TYR B 775 3779 6679 4366 1029 120 730 O
ATOM 1822 CB TYR B 775 47.164 18.646 36.597 1.00 36.47 C
ANISOU 1822 CB TYR B 775 3939 5702 4214 1092 209 482 C
ATOM 1823 CG TYR B 775 48.108 17.469 36.434 1.00 39.78 C
ANISOU 1823 CG TYR B 775 4373 6220 4522 1389 298 561 C
ATOM 1824 CD1 TYR B 775 47.902 16.305 37.202 1.00 42.00 C
ANISOU 1824 CD1 TYR B 775 4824 6351 4784 1549 342 555 C
ATOM 1825 CD2 TYR B 775 49.199 17.536 35.542 1.00 41.18 C
ANISOU 1825 CD2 TYR B 775 4399 6652 4597 1522 344 651 C
ATOM 1826 CE1 TYR B 775 48.772 15.207 37.071 1.00 44.10 C
ANISOU 1826 CE1 TYR B 775 5137 6695 4925 1855 435 635 C
ATOM 1827 CE2 TYR B 775 50.080 16.443 35.425 1.00 42.33 C
ANISOU 1827 CE2 TYR B 775 4564 6902 4616 1838 439 736 C
ATOM 1828 CZ TYR B 775 49.864 15.276 36.185 1.00 51.70 C
ANISOU 1828 CZ TYR B 775 5949 7914 5781 2014 487 726 C
ATOM 1829 OH TYR B 775 50.717 14.217 36.072 1.00 56.06 O
ANISOU 1829 OH TYR B 775 6552 8559 6189 2357 590 817 O
ATOM 1830 H TYR B 775 45.719 20.589 37.289 1.00 33.87 H
ATOM 1831 HA TYR B 775 47.432 18.759 38.719 1.00 34.86 H
ATOM 1832 HB3 TYR B 775 47.212 19.291 35.718 1.00 36.47 H
ATOM 1833 HB2 TYR B 775 46.151 18.241 36.609 1.00 36.47 H
ATOM 1834 HD1 TYR B 775 47.072 16.251 37.891 1.00 42.00 H
ATOM 1835 HD2 TYR B 775 49.369 18.427 34.955 1.00 41.18 H
ATOM 1836 HE1 TYR B 775 48.600 14.316 37.656 1.00 44.10 H
ATOM 1837 HE2 TYR B 775 50.923 16.507 34.753 1.00 42.33 H
ATOM 1838 HH TYR B 775 51.378 14.341 35.386 1.00 56.06 H
ATOM 1839 N SER B 776 48.799 21.400 37.259 1.00 37.64 N
ANISOU 1839 N SER B 776 3656 6349 4298 672 47 611 N
ATOM 1840 CA SER B 776 49.981 22.269 37.204 1.00 38.30 C
ANISOU 1840 CA SER B 776 3522 6749 4282 545 1 714 C
ATOM 1841 C SER B 776 50.410 22.804 38.582 1.00 42.86 C
ANISOU 1841 C SER B 776 4006 7469 4809 437 -67 777 C
ATOM 1842 O SER B 776 51.607 22.967 38.812 1.00 42.21 O
ANISOU 1842 O SER B 776 3717 7714 4606 445 -80 903 O
ATOM 1843 CB SER B 776 49.723 23.429 36.220 1.00 42.32 C
ANISOU 1843 CB SER B 776 4041 7224 4813 307 -47 670 C
ATOM 1844 OG SER B 776 49.892 22.981 34.891 1.00 54.90 O
ANISOU 1844 OG SER B 776 5608 8862 6389 407 11 675 O

```
ATOM   1845  H   SER B 776      47.972  21.755  36.795  1.00 37.64           H
ATOM   1846  HA  SER B 776      50.817  21.675  36.829  1.00 38.30           H
ATOM   1847  HB3 SER B 776      50.436  24.239  36.378  1.00 42.32           H
ATOM   1848  HB2 SER B 776      48.729  23.856  36.352  1.00 42.32           H
ATOM   1849  HG  SER B 776      49.490  23.615  34.288  1.00 54.90           H
ATOM   1850  N   LEU B 777      49.433  23.022  39.477  1.00 40.23           N
ANISOU 1850  N   LEU B 777     3820   6907   4557    346   -105    697       N
ATOM   1851  CA  LEU B 777      49.629  23.402  40.875  1.00 40.70           C
ANISOU 1851  CA  LEU B 777     3845   7038   4583    240   -169    734       C
ATOM   1852  C   LEU B 777      50.225  22.248  41.705  1.00 47.22           C
ANISOU 1852  C   LEU B 777     4571   8029   5342    470   -128    827       C
ATOM   1853  O   LEU B 777      51.101  22.490  42.535  1.00 45.95           O
ANISOU 1853  O   LEU B 777     4234   8154   5073    413   -172    936       O
ATOM   1854  CB  LEU B 777      48.265  23.884  41.426  1.00 40.76           C
ANISOU 1854  CB  LEU B 777     4059   6731   4698    150   -195    619       C
ATOM   1855  CG  LEU B 777      48.285  24.558  42.811  1.00 45.68           C
ANISOU 1855  CG  LEU B 777     4723   7339   5292   -100   -283    609       C
ATOM   1856  CD1 LEU B 777      49.101  25.868  42.808  1.00 45.58           C
ANISOU 1856  CD1 LEU B 777     4650   7474   5195   -335   -340    644       C
ATOM   1857  CD2 LEU B 777      46.850  24.777  43.339  1.00 48.85           C
ANISOU 1857  CD2 LEU B 777     5337   7427   5797   -137   -283    495       C
ATOM   1858  H   LEU B 777      48.471  22.892  39.191  1.00 40.23           H
ATOM   1859  HA  LEU B 777      50.337  24.232  40.894  1.00 40.70           H
ATOM   1860  HB3 LEU B 777      47.576  23.037  41.441  1.00 40.76           H
ATOM   1861  HB2 LEU B 777      47.835  24.606  40.732  1.00 40.76           H
ATOM   1862  HG  LEU B 777      48.765  23.859  43.494  1.00 45.68           H
ATOM   1863 HD11 LEU B 777      48.560  26.699  43.261  1.00 45.58           H
ATOM   1864 HD12 LEU B 777      50.026  25.746  43.373  1.00 45.58           H
ATOM   1865 HD13 LEU B 777      49.373  26.183  41.800  1.00 45.58           H
ATOM   1866 HD21 LEU B 777      46.787  24.533  44.398  1.00 48.85           H
ATOM   1867 HD22 LEU B 777      46.520  25.809  43.227  1.00 48.85           H
ATOM   1868 HD23 LEU B 777      46.118  24.156  42.822  1.00 48.85           H
ATOM   1869  N   LEU B 778      49.763  21.015  41.431  1.00 46.19           N
ANISOU 1869  N   LEU B 778     4558   7736   5258    727    -43    793       N
ATOM   1870  CA  LEU B 778      50.192  19.774  42.077  1.00 47.57           C
ANISOU 1870  CA  LEU B 778     4702   8007   5364   1002     20    872       C
ATOM   1871  C   LEU B 778      51.632  19.353  41.708  1.00 55.92           C
ANISOU 1871  C   LEU B 778     5528   9447   6273   1154     56   1021       C
ATOM   1872  O   LEU B 778      52.282  18.689  42.515  1.00 57.40           O
ANISOU 1872  O   LEU B 778     5595   9854   6360   1302     70   1133       O
ATOM   1873  CB  LEU B 778      49.154  18.679  41.726  1.00 47.36           C
ANISOU 1873  CB  LEU B 778     4913   7678   5402   1206    106    790       C
ATOM   1874  CG  LEU B 778      49.161  17.420  42.619  1.00 51.63           C
ANISOU 1874  CG  LEU B 778     5626   8008   5984   1294    122    752       C
ATOM   1875  CD1 LEU B 778      48.833  17.741  44.094  1.00 51.67           C
ANISOU 1875  CD1 LEU B 778     5580   8035   6017   1107     37    752       C
ATOM   1876  CD2 LEU B 778      48.228  16.336  42.036  1.00 52.78           C
ANISOU 1876  CD2 LEU B 778     6022   7808   6225   1289    156    631       C
ATOM   1877  H   LEU B 778      49.049  20.912  40.723  1.00 46.19           H
ATOM   1878  HA  LEU B 778      50.171  19.956  43.153  1.00 47.57           H
ATOM   1879  HB3 LEU B 778      49.296  18.385  40.686  1.00 47.36           H
ATOM   1880  HB2 LEU B 778      48.152  19.105  41.777  1.00 47.36           H
ATOM   1881  HG  LEU B 778      50.168  17.005  42.596  1.00 51.63           H
ATOM   1882 HD11 LEU B 778      48.065  17.087  44.508  1.00 51.67           H
ATOM   1883 HD12 LEU B 778      49.719  17.621  44.718  1.00 51.67           H
ATOM   1884 HD13 LEU B 778      48.481  18.764  44.227  1.00 51.67           H
```

ATOM 1885 HD21 LEU B 778 48.743 15.378 41.969 1.00 52.78 H
ATOM 1886 HD22 LEU B 778 47.331 16.182 42.637 1.00 52.78 H
ATOM 1887 HD23 LEU B 778 47.888 16.583 41.029 1.00 52.78 H
ATOM 1888 N ARG B 779 52.107 19.768 40.520 1.00 53.39 N
ANISOU 1888 N ARG B 779 5131 9228 5927 1120 73 1033 N
ATOM 1889 CA ARG B 779 53.458 19.519 40.004 1.00 53.96 C
ANISOU 1889 CA ARG B 779 4970 9685 5848 1270 115 1181 C
ATOM 1890 C ARG B 779 54.382 20.749 40.122 1.00 58.49 C
ANISOU 1890 C ARG B 779 5284 10611 6330 989 22 1279 C
ATOM 1891 O ARG B 779 55.563 20.629 39.795 1.00 58.93 O
ANISOU 1891 O ARG B 779 5115 11025 6251 1071 50 1412 O
ATOM 1892 CB ARG B 779 53.345 19.055 38.533 1.00 54.86 C
ANISOU 1892 CB ARG B 779 5167 9715 5963 1459 211 1151 C
ATOM 1893 CG ARG B 779 52.512 17.773 38.334 1.00 67.94 C
ANISOU 1893 CG ARG B 779 7118 11012 7683 1699 299 1054 C
ATOM 1894 CD ARG B 779 53.204 16.494 38.837 1.00 83.33 C
ANISOU 1894 CD ARG B 779 9108 13011 9543 2008 369 1134 C
ATOM 1895 NE ARG B 779 52.276 15.349 38.866 1.00 96.52 N
ANISOU 1895 NE ARG B 779 11108 14285 11281 2141 426 1024 N
ATOM 1896 CZ ARG B 779 51.701 14.784 39.944 1.00112.21 C
ANISOU 1896 CZ ARG B 779 13222 16149 13262 2256 443 1027 C
ATOM 1897 NH1 ARG B 779 50.849 13.767 39.759 1.00 98.78 N
ANISOU 1897 NH1 ARG B 779 11836 14089 11607 2339 491 929 N
ATOM 1898 NH2 ARG B 779 51.949 15.202 41.195 1.00100.14 N1+
ANISOU 1898 NH2 ARG B 779 11513 14864 11673 2272 407 1130 N1+
ATOM 1899 H ARG B 779 51.496 20.294 39.909 1.00 53.39 H
ATOM 1900 HA ARG B 779 53.934 18.730 40.586 1.00 53.96 H
ATOM 1901 HB3 ARG B 779 54.341 18.890 38.118 1.00 54.86 H
ATOM 1902 HB2 ARG B 779 52.901 19.856 37.939 1.00 54.86 H
ATOM 1903 HG3 ARG B 779 52.403 17.677 37.252 1.00 67.94 H
ATOM 1904 HG2 ARG B 779 51.495 17.857 38.718 1.00 67.94 H
ATOM 1905 HD3 ARG B 779 53.778 16.636 39.752 1.00 83.33 H
ATOM 1906 HD2 ARG B 779 53.933 16.202 38.081 1.00 83.33 H
ATOM 1907 HE ARG B 779 52.010 14.992 37.958 1.00 96.52 H
ATOM 1908 HH12 ARG B 779 50.402 13.321 40.547 1.00 98.78 H
ATOM 1909 HH11 ARG B 779 50.648 13.438 38.825 1.00 98.78 H
ATOM 1910 HH22 ARG B 779 51.505 14.760 41.987 1.00100.14 H
ATOM 1911 HH21 ARG B 779 52.576 15.979 41.351 1.00100.14 H
ATOM 1912 N GLY B 780 53.849 21.894 40.581 1.00 54.99 N
ANISOU 1912 N GLY B 780 4883 10076 5937 668 -84 1223 N
ATOM 1913 CA GLY B 780 54.581 23.148 40.751 1.00 55.16 C
ANISOU 1913 CA GLY B 780 4725 10375 5858 346 -185 1301 C
ATOM 1914 C GLY B 780 54.948 23.367 42.225 1.00 61.06 C
ANISOU 1914 C GLY B 780 5404 11258 6538 166 -277 1356 C
ATOM 1915 O GLY B 780 54.638 22.551 43.094 1.00 60.17 O
ANISOU 1915 O GLY B 780 5369 11029 6465 305 -262 1336 O
ATOM 1916 H GLY B 780 52.871 21.910 40.830 1.00 54.99 H
ATOM 1917 HA3 GLY B 780 53.939 23.965 40.421 1.00 55.16 H
ATOM 1918 HA2 GLY B 780 55.481 23.181 40.135 1.00 55.16 H
ATOM 1919 N GLY B 781 55.599 24.511 42.495 1.00 59.91 N
ANISOU 1919 N GLY B 781 5124 11360 6277 -160 -374 1429 N
ATOM 1920 CA GLY B 781 55.993 24.949 43.835 1.00 61.15 C
ANISOU 1920 CA GLY B 781 5219 11677 6336 -403 -479 1489 C
ATOM 1921 C GLY B 781 57.241 24.203 44.330 1.00 67.96 C
ANISOU 1921 C GLY B 781 5786 12998 7037 -260 -475 1677 C
ATOM 1922 O GLY B 781 58.042 23.709 43.535 1.00 66.74 O

ANISOU 1922 O   GLY B 781   5391 13197   6771   -122   -428   1811   O
ATOM   1923 H   GLY B 781   55.841 25.127 41.733 1.00 59.91   H
ATOM   1924 HA3 GLY B 781   55.165 24.810 44.533 1.00 61.15   H
ATOM   1925 HA2 GLY B 781   56.211 26.017 43.805 1.00 61.15   H
ATOM   1926 N   SER B 782   57.401 24.162 45.662 1.00 67.69   N
ANISOU 1926 N   SER B 782   5765 12976   6979   -281   -521   1695   N
ATOM   1927 CA SER B 782   58.486 23.465 46.355 1.00 69.07   C
ANISOU 1927 CA SER B 782   5668 13582   6995   -138   -523   1876   C
ATOM   1928 C   SER B 782   57.974 22.141 46.943 1.00 76.97   C
ANISOU 1928 C   SER B 782   6756 14415   8073   264   -429   1853   C
ATOM   1929 O   SER B 782   56.857 22.102 47.462 1.00 77.53   O
ANISOU 1929 O   SER B 782   7105 14046   8306   298   -412   1696   O
ATOM   1930 CB SER B 782   59.014 24.370 47.487 1.00 72.08   C
ANISOU 1930 CB SER B 782   5966 14182   7241   -512   -664   1946   C
ATOM   1931 OG SER B 782   59.808 25.411 46.955 1.00 77.74   O
ANISOU 1931 OG SER B 782   6562 15151   7826   -879   -750   2014   O
ATOM   1932 H   SER B 782   56.703 24.581 46.258 1.00 67.69   H
ATOM   1933 HA SER B 782   59.308 23.242 45.671 1.00 69.07   H
ATOM   1934 HB3 SER B 782   59.643 23.801 48.173 1.00 72.08   H
ATOM   1935 HB2 SER B 782   58.200 24.791 48.079 1.00 72.08   H
ATOM   1936 HG SER B 782   59.245 25.989 46.436 1.00 77.74   H
ATOM   1937 N   ASP B 783   58.829 21.106 46.896 1.00 75.81   N
ANISOU 1937 N   ASP B 783   6378 14630   7796   577   -361   2019   N
ATOM   1938 CA ASP B 783   58.628 19.825 47.579 1.00 76.72   C
ANISOU 1938 CA ASP B 783   6574 14635   7940   993   -259   2028   C
ATOM   1939 C   ASP B 783   59.413 19.801 48.895 1.00 79.58   C
ANISOU 1939 C   ASP B 783   6743 15336   8156   1018   -308   2176   C
ATOM   1940 O   ASP B 783   60.532 20.310 48.963 1.00 78.85   O
ANISOU 1940 O   ASP B 783   6320 15774   7866   994   -338   2371   O
ATOM   1941 CB ASP B 783   59.044 18.605 46.715 1.00 79.64   C
ANISOU 1941 CB ASP B 783   6875 15130   8255   1407   -117   2106   C
ATOM   1942 CG ASP B 783   58.160 18.313 45.492 1.00 97.20   C
ANISOU 1942 CG ASP B 783   9422 16886 10622   1725   2   1969   C
ATOM   1943 OD1 ASP B 783   56.951 18.632 45.537 1.00 99.14   O
ANISOU 1943 OD1 ASP B 783   9835 16911 10922   1849   19   1922   O
ATOM   1944 OD2 ASP B 783   58.671 17.620 44.585 1.00104.89   O1-
ANISOU 1944 OD2 ASP B 783   10486 17727 11640   1842   77   1915   O1-
ATOM   1945 H   ASP B 783   59.729 21.225 46.453 1.00 75.81   H
ATOM   1946 HA ASP B 783   57.573 19.717 47.840 1.00 76.72   H
ATOM   1947 HB3 ASP B 783   59.052 17.700 47.326 1.00 79.64   H
ATOM   1948 HB2 ASP B 783   60.070 18.751 46.368 1.00 79.64   H
ATOM   1949 N   ASP B 784   58.801 19.159 49.900 1.00 75.43   N
ANISOU 1949 N   ASP B 784   6414 14527   7721   1063   -316   2093   N
ATOM   1950 CA ASP B 784   59.368 18.934 51.221 1.00 74.73   C
ANISOU 1950 CA ASP B 784   6194 14683   7516   1100   -360   2209   C
ATOM   1951 C   ASP B 784   58.723 17.652 51.766 1.00 76.72   C
ANISOU 1951 C   ASP B 784   6662 14638   7851   1466   -262   2157   C
ATOM   1952 O   ASP B 784   57.542 17.665 52.118 1.00 76.23   O
ANISOU 1952 O   ASP B 784   6876 14140   7948   1374   -278   1994   O
ATOM   1953 CB ASP B 784   59.187 20.166 52.148 1.00 76.53   C
ANISOU 1953 CB ASP B 784   6441 14899   7736   629   -516   2163   C
ATOM   1954 CG ASP B 784   59.759 20.066 53.574 1.00 84.42   C
ANISOU 1954 CG ASP B 784   7314 16151   8612   598   -582   2275   C
ATOM   1955 OD1 ASP B 784   60.602 19.178 53.837 1.00 83.77   O
ANISOU 1955 OD1 ASP B 784   7046 16374   8410   932   -518   2431   O
ATOM   1956 OD2 ASP B 784   59.409 20.963 54.372 1.00 91.02   O1-

```
ANISOU 1956  OD2 ASP B 784     8248  16880   9457    250   -696   2209       O1-
ATOM   1957  H   ASP B 784      57.882  18.766  49.750  1.00 75.43           H
ATOM   1958  HA  ASP B 784      60.438  18.750  51.101  1.00 74.73           H
ATOM   1959  HB3 ASP B 784      58.127  20.422  52.204  1.00 76.53           H
ATOM   1960  HB2 ASP B 784      59.656  21.028  51.671  1.00 76.53           H
ATOM   1961  N   SER B 785      59.518  16.571  51.806  1.00 71.78           N
ANISOU 1961  N   SER B 785     5910  14267   7097   1884   -159   2308       N
ATOM   1962  CA  SER B 785      59.100  15.234  52.233  1.00 71.24           C
ANISOU 1962  CA  SER B 785     6041  13976   7052   2282    -50   2297       C
ATOM   1963  C   SER B 785      58.810  15.099  53.741  1.00 73.75           C
ANISOU 1963  C   SER B 785     6419  14220   7382   2203   -114   2287       C
ATOM   1964  O   SER B 785      58.152  14.130  54.121  1.00 74.16           O
ANISOU 1964  O   SER B 785     6739  13917   7522   2406    -46   2205       O
ATOM   1965  CB  SER B 785      60.168  14.213  51.792  1.00 76.08           C
ANISOU 1965  CB  SER B 785     6478  14955   7472   2742     70   2494       C
ATOM   1966  OG  SER B 785      60.117  14.017  50.393  1.00 87.30           O
ANISOU 1966  OG  SER B 785     7993  16263   8915   2944    177   2460       O
ATOM   1967  H   SER B 785      60.474  16.651  51.489  1.00 71.78           H
ATOM   1968  HA  SER B 785      58.164  14.995  51.723  1.00 71.24           H
ATOM   1969  HB3 SER B 785      59.999  13.242  52.261  1.00 76.08           H
ATOM   1970  HB2 SER B 785      61.170  14.534  52.082  1.00 76.08           H
ATOM   1971  HG  SER B 785      59.295  13.570  50.175  1.00 87.30           H
ATOM   1972  N   SER B 786      59.288  16.048  54.569  1.00 67.87           N
ANISOU 1972  N   SER B 786     5440  13810   6538   1899   -244   2374       N
ATOM   1973  CA  SER B 786      59.065  16.062  56.019  1.00 66.11           C
ANISOU 1973  CA  SER B 786     5252  13562   6306   1787   -318   2374       C
ATOM   1974  C   SER B 786      57.633  16.472  56.426  1.00 66.54           C
ANISOU 1974  C   SER B 786     5635  13078   6570   1532   -366   2149       C
ATOM   1975  O   SER B 786      57.197  16.092  57.512  1.00 66.10           O
ANISOU 1975  O   SER B 786     5687  12887   6539   1522   -392   2120       O
ATOM   1976  CB  SER B 786      60.155  16.918  56.704  1.00 69.13           C
ANISOU 1976  CB  SER B 786     5302  14466   6500   1501   -450   2532       C
ATOM   1977  OG  SER B 786      59.911  18.310  56.634  1.00 77.32           O
ANISOU 1977  OG  SER B 786     6356  15444   7578   1019   -571   2441       O
ATOM   1978  H   SER B 786      59.807  16.829  54.190  1.00 67.87           H
ATOM   1979  HA  SER B 786      59.208  15.038  56.369  1.00 66.11           H
ATOM   1980  HB3 SER B 786      61.137  16.705  56.282  1.00 69.13           H
ATOM   1981  HB2 SER B 786      60.211  16.653  57.760  1.00 69.13           H
ATOM   1982  HG  SER B 786      60.109  18.621  55.742  1.00 77.32           H
ATOM   1983  N   LYS B 787      56.931  17.213  55.551  1.00 59.93           N
ANISOU 1983  N   LYS B 787     4947  11954   5871   1343   -372   2001       N
ATOM   1984  CA  LYS B 787      55.534  17.610  55.723  1.00 58.52           C
ANISOU 1984  CA  LYS B 787     5060  11300   5875   1125   -406   1801       C
ATOM   1985  C   LYS B 787      54.573  16.532  55.201  1.00 57.67           C
ANISOU 1985  C   LYS B 787     5231  10757   5922   1379   -290   1677       C
ATOM   1986  O   LYS B 787      54.922  15.787  54.283  1.00 57.21           O
ANISOU 1986  O   LYS B 787     5178  10703   5858   1623   -195   1700       O
ATOM   1987  CB  LYS B 787      55.289  18.926  54.956  1.00 61.69           C
ANISOU 1987  CB  LYS B 787     5466  11659   6315    748   -489   1721       C
ATOM   1988  CG  LYS B 787      55.961  20.153  55.590  1.00 80.22           C
ANISOU 1988  CG  LYS B 787     7659  14296   8525    383   -628   1792       C
ATOM   1989  CD  LYS B 787      55.646  21.440  54.811  1.00 92.60           C
ANISOU 1989  CD  LYS B 787     9281  15790  10114     29   -697   1714       C
ATOM   1990  CE  LYS B 787      56.223  22.699  55.476  1.00105.49           C
ANISOU 1990  CE  LYS B 787    10837  17649  11596   -372   -840   1769       C
ATOM   1991  NZ  LYS B 787      55.867  23.917  54.726  1.00114.45           N1+
```

ANISOU 1991 NZ LYS B 787   12073 18682 12732   -720   -904   1693   N1+
ATOM   1992 H   LYS B 787   57.356 17.471 54.671 1.00 59.93   H
ATOM   1993 HA LYS B 787   55.330 17.776 56.783 1.00 58.52   H
ATOM   1994 HB3 LYS B 787   54.216 19.126 54.921 1.00 61.69   H
ATOM   1995 HB2 LYS B 787   55.607 18.820 53.917 1.00 61.69   H
ATOM   1996 HG3 LYS B 787   57.040 20.005 55.635 1.00 80.22   H
ATOM   1997 HG2 LYS B 787   55.621 20.258 56.621 1.00 80.22   H
ATOM   1998 HD3 LYS B 787   54.564 21.540 54.706 1.00 92.60   H
ATOM   1999 HD2 LYS B 787   56.043 21.344 53.799 1.00 92.60   H
ATOM   2000 HE3 LYS B 787   57.309 22.630 55.542 1.00105.49   H
ATOM   2001 HE2 LYS B 787   55.842 22.796 56.493 1.00105.49   H
ATOM   2002 HZ1 LYS B 787   54.860 24.010 54.675 1.00114.45   H
ATOM   2003 HZ2 LYS B 787   56.251 24.729 55.187 1.00114.45   H
ATOM   2004 HZ3 LYS B 787   56.235 23.856 53.788 1.00114.45   H
ATOM   2005 N   ASP B 788   53.347 16.530 55.754 1.00 50.78   N
ANISOU 2005 N   ASP B 788   4600   9518   5177   1301   -301   1548   N
ATOM   2006 CA ASP B 788   52.188 15.804 55.226 1.00 48.68   C
ANISOU 2006 CA ASP B 788   4615   8827   5055   1449   -214   1421   C
ATOM   2007 C   ASP B 788   51.783 16.470 53.886 1.00 48.42   C
ANISOU 2007 C   ASP B 788   4646   8635   5116   1332   -202   1320   C
ATOM   2008 O   ASP B 788   51.593 17.688 53.880 1.00 47.35   O
ANISOU 2008 O   ASP B 788   4472   8512   5007   1037   -284   1270   O
ATOM   2009 CB ASP B 788   51.024 15.845 56.260 1.00 50.50   C
ANISOU 2009 CB ASP B 788   5037   8768   5384   1307   -253   1317   C
ATOM   2010 CG ASP B 788   49.828 14.891 56.042 1.00 59.51   C
ANISOU 2010 CG ASP B 788   6457   9503   6652   1423   -176   1203   C
ATOM   2011 OD1 ASP B 788   49.531 14.521 54.886 1.00 58.68   O
ANISOU 2011 OD1 ASP B 788   6450   9226   6618   1452   -126   1132   O
ATOM   2012 OD2 ASP B 788   49.137 14.619 57.047 1.00 66.16   O1-
ANISOU 2012 OD2 ASP B 788   7417 10205   7515   1460   -172   1187   O1-
ATOM   2013 H   ASP B 788   53.148 17.166 56.513 1.00 50.78   H
ATOM   2014 HA ASP B 788   52.488 14.764 55.107 1.00 48.68   H
ATOM   2015 HB3 ASP B 788   50.639 16.862 56.341 1.00 50.50   H
ATOM   2016 HB2 ASP B 788   51.432 15.632 57.246 1.00 50.50   H
ATOM   2017 N   PRO B 789   51.647 15.688 52.786 1.00 43.39   N
ANISOU 2017 N   PRO B 789   4123   7847   4515   1558   -101   1292   N
ATOM   2018 CA PRO B 789   51.068 16.167 51.512 1.00 42.73   C
ANISOU 2018 CA PRO B 789   4100   7617   4517   1448   -90   1197   C
ATOM   2019 C   PRO B 789   49.740 16.951 51.579 1.00 44.30   C
ANISOU 2019 C   PRO B 789   4461   7510   4859   1181   -142   1046   C
ATOM   2020 O   PRO B 789   49.518 17.786 50.705 1.00 43.74   O
ANISOU 2020 O   PRO B 789   4367   7423   4827   1006   -173   994   O
ATOM   2021 CB PRO B 789   50.919 14.888 50.674 1.00 44.52   C
ANISOU 2021 CB PRO B 789   4484   7686   4747   1753   31   1187   C
ATOM   2022 CG PRO B 789   52.038 13.991 51.170 1.00 48.97   C
ANISOU 2022 CG PRO B 789   4991   8438   5177   2048   86   1321   C
ATOM   2023 CD PRO B 789   52.085 14.294 52.661 1.00 44.61   C
ANISOU 2023 CD PRO B 789   4386   7946   4617   1922   10   1345   C
ATOM   2024 HA PRO B 789   51.825 16.804 51.051 1.00 42.73   H
ATOM   2025 HB3 PRO B 789   50.983 15.077 49.602 1.00 44.52   H
ATOM   2026 HB2 PRO B 789   49.957 14.410 50.871 1.00 44.52   H
ATOM   2027 HG3 PRO B 789   52.976 14.298 50.707 1.00 48.97   H
ATOM   2028 HG2 PRO B 789   51.881 12.935 50.949 1.00 48.97   H
ATOM   2029 HD2 PRO B 789   51.398 13.642 53.198 1.00 44.61   H
ATOM   2030 HD3 PRO B 789   53.091 14.129 53.050 1.00 44.61   H
ATOM   2031 N   ILE B 790   48.909 16.712 52.610 1.00 39.58   N

ANISOU 2031 N ILE B 790 4024 6688 4326 1160 -147 985 N
ATOM 2032 CA ILE B 790 47.677 17.461 52.884 1.00 38.53 C
ANISOU 2032 CA ILE B 790 4037 6295 4308 943 -188 860 C
ATOM 2033 C ILE B 790 47.954 18.935 53.259 1.00 39.79 C
ANISOU 2033 C ILE B 790 4095 6589 4436 667 -286 859 C
ATOM 2034 O ILE B 790 47.262 19.812 52.745 1.00 39.41 O
ANISOU 2034 O ILE B 790 4116 6411 4449 503 -309 776 O
ATOM 2035 CB ILE B 790 46.833 16.797 54.013 1.00 42.13 C
ANISOU 2035 CB ILE B 790 4657 6538 4812 992 -171 822 C
ATOM 2036 CG1 ILE B 790 46.379 15.372 53.615 1.00 43.24 C
ANISOU 2036 CG1 ILE B 790 4967 6480 4981 1206 -76 802 C
ATOM 2037 CG2 ILE B 790 45.603 17.619 54.460 1.00 42.40 C
ANISOU 2037 CG2 ILE B 790 4800 6376 4933 774 -217 721 C
ATOM 2038 CD1 ILE B 790 45.902 14.529 54.808 1.00 54.61 C
ANISOU 2038 CD1 ILE B 790 6553 7770 6425 1292 -50 804 C
ATOM 2039 H ILE B 790 49.151 16.001 53.291 1.00 39.58 H
ATOM 2040 HA ILE B 790 47.082 17.458 51.969 1.00 38.53 H
ATOM 2041 HB ILE B 790 47.484 16.698 54.883 1.00 42.13 H
ATOM 2042 HG13 ILE B 790 47.190 14.830 53.130 1.00 43.24 H
ATOM 2043 HG12 ILE B 790 45.583 15.436 52.872 1.00 43.24 H
ATOM 2044 HG21 ILE B 790 45.007 17.063 55.180 1.00 42.40 H
ATOM 2045 HG22 ILE B 790 45.880 18.553 54.949 1.00 42.40 H
ATOM 2046 HG23 ILE B 790 44.957 17.858 53.615 1.00 42.40 H
ATOM 2047 HD11 ILE B 790 46.119 13.472 54.649 1.00 54.61 H
ATOM 2048 HD12 ILE B 790 46.392 14.823 55.736 1.00 54.61 H
ATOM 2049 HD13 ILE B 790 44.828 14.633 54.956 1.00 54.61 H
ATOM 2050 N ASP B 791 48.979 19.184 54.095 1.00 34.82 N
ANISOU 2050 N ASP B 791 3314 6222 3692 616 -345 957 N
ATOM 2051 CA ASP B 791 49.472 20.527 54.437 1.00 34.33 C
ANISOU 2051 CA ASP B 791 3177 6310 3559 332 -446 972 C
ATOM 2052 C ASP B 791 50.082 21.257 53.232 1.00 39.15 C
ANISOU 2052 C ASP B 791 3670 7077 4126 222 -464 996 C
ATOM 2053 O ASP B 791 49.815 22.444 53.061 1.00 37.93 O
ANISOU 2053 O ASP B 791 3578 6863 3969 -27 -523 942 O
ATOM 2054 CB ASP B 791 50.497 20.531 55.600 1.00 35.03 C
ANISOU 2054 CB ASP B 791 3117 6679 3512 306 -504 1089 C
ATOM 2055 CG ASP B 791 49.988 19.997 56.947 1.00 43.78 C
ANISOU 2055 CG ASP B 791 4348 7644 4644 323 -516 1060 C
ATOM 2056 OD1 ASP B 791 48.786 20.189 57.242 1.00 43.73 O
ANISOU 2056 OD1 ASP B 791 4538 7343 4733 241 -514 946 O
ATOM 2057 OD2 ASP B 791 50.851 19.559 57.738 1.00 47.17 O1-
ANISOU 2057 OD2 ASP B 791 4665 8273 4982 424 -526 1162 O1-
ATOM 2058 H ASP B 791 49.513 18.412 54.468 1.00 34.82 H
ATOM 2059 HA ASP B 791 48.606 21.116 54.746 1.00 34.33 H
ATOM 2060 HB3 ASP B 791 50.857 21.547 55.772 1.00 35.03 H
ATOM 2061 HB2 ASP B 791 51.368 19.940 55.311 1.00 35.03 H
ATOM 2062 N VAL B 792 50.860 20.534 52.408 1.00 36.90 N
ANISOU 2062 N VAL B 792 3234 6993 3795 418 -408 1082 N
ATOM 2063 CA VAL B 792 51.489 21.060 51.193 1.00 37.00 C
ANISOU 2063 CA VAL B 792 3113 7185 3759 353 -410 1120 C
ATOM 2064 C VAL B 792 50.452 21.485 50.128 1.00 40.92 C
ANISOU 2064 C VAL B 792 3787 7375 4385 278 -386 984 C
ATOM 2065 O VAL B 792 50.619 22.541 49.521 1.00 40.56 O
ANISOU 2065 O VAL B 792 3741 7350 4321 42 -441 959 O
ATOM 2066 CB VAL B 792 52.486 20.028 50.584 1.00 41.45 C
ANISOU 2066 CB VAL B 792 3495 8015 4239 641 -335 1245 C

ATOM 2067 CG1 VAL B 792 53.061 20.410 49.202 1.00 41.28 C
ANISOU 2067 CG1 VAL B 792 3347 8161 4175 592 -325 1279 C
ATOM 2068 CG2 VAL B 792 53.646 19.739 51.556 1.00 41.52 C
ANISOU 2068 CG2 VAL B 792 3294 8388 4094 700 -368 1401 C
ATOM 2069 H VAL B 792 51.038 19.563 52.623 1.00 36.90 H
ATOM 2070 HA VAL B 792 52.052 21.952 51.477 1.00 37.00 H
ATOM 2071 HB VAL B 792 51.950 19.089 50.447 1.00 41.45 H
ATOM 2072 HG11 VAL B 792 53.825 19.700 48.886 1.00 41.28 H
ATOM 2073 HG12 VAL B 792 52.297 20.411 48.425 1.00 41.28 H
ATOM 2074 HG13 VAL B 792 53.520 21.399 49.226 1.00 41.28 H
ATOM 2075 HG21 VAL B 792 54.332 18.998 51.146 1.00 41.52 H
ATOM 2076 HG22 VAL B 792 54.220 20.642 51.766 1.00 41.52 H
ATOM 2077 HG23 VAL B 792 53.288 19.347 52.508 1.00 41.52 H
ATOM 2078 N ASN B 793 49.387 20.683 49.955 1.00 36.65 N
ANISOU 2078 N ASN B 793 3412 6553 3962 463 -307 901 N
ATOM 2079 CA ASN B 793 48.271 20.952 49.038 1.00 35.57 C
ANISOU 2079 CA ASN B 793 3433 6142 3940 411 -281 781 C
ATOM 2080 C ASN B 793 47.313 22.038 49.570 1.00 37.94 C
ANISOU 2080 C ASN B 793 3870 6256 4288 179 -341 692 C
ATOM 2081 O ASN B 793 46.709 22.731 48.752 1.00 37.07 O
ANISOU 2081 O ASN B 793 3823 6049 4215 59 -351 631 O
ATOM 2082 CB ASN B 793 47.513 19.634 48.746 1.00 35.56 C
ANISOU 2082 CB ASN B 793 3570 5919 4020 640 -191 731 C
ATOM 2083 CG ASN B 793 48.224 18.614 47.839 1.00 48.62 C
ANISOU 2083 CG ASN B 793 5152 7707 5616 883 -115 803 C
ATOM 2084 ND2 ASN B 793 49.520 18.781 47.560 1.00 34.81 N
ANISOU 2084 ND2 ASN B 793 3549 5785 3892 1092 -39 782 N
ATOM 2085 OD1 ASN B 793 47.594 17.659 47.392 1.00 48.08 O
ANISOU 2085 OD1 ASN B 793 4909 7897 5463 887 -121 883 O
ATOM 2086 H ASN B 793 49.321 19.828 50.492 1.00 36.65 H
ATOM 2087 HA ASN B 793 48.714 21.309 48.107 1.00 35.57 H
ATOM 2088 HB3 ASN B 793 46.576 19.870 48.243 1.00 35.56 H
ATOM 2089 HB2 ASN B 793 47.239 19.141 49.680 1.00 35.56 H
ATOM 2090 HD22 ASN B 793 50.002 18.106 46.985 1.00 34.81 H
ATOM 2091 HD21 ASN B 793 50.030 19.551 47.966 1.00 34.81 H
ATOM 2092 N TYR B 794 47.213 22.199 50.903 1.00 34.93 N
ANISOU 2092 N TYR B 794 3541 5840 3892 127 -379 693 N
ATOM 2093 CA TYR B 794 46.463 23.276 51.558 1.00 34.72 C
ANISOU 2093 CA TYR B 794 3663 5648 3882 -63 -429 620 C
ATOM 2094 C TYR B 794 47.106 24.657 51.319 1.00 37.69 C
ANISOU 2094 C TYR B 794 4016 6137 4167 -311 -501 634 C
ATOM 2095 O TYR B 794 46.397 25.598 50.962 1.00 35.52 O
ANISOU 2095 O TYR B 794 3884 5690 3922 -422 -508 559 O
ATOM 2096 CB TYR B 794 46.296 22.952 53.061 1.00 35.48 C
ANISOU 2096 CB TYR B 794 3800 5730 3949 -63 -456 636 C
ATOM 2097 CG TYR B 794 45.555 23.996 53.880 1.00 36.62 C
ANISOU 2097 CG TYR B 794 4112 5722 4080 -239 -503 573 C
ATOM 2098 CD1 TYR B 794 44.152 23.932 53.992 1.00 38.74 C
ANISOU 2098 CD1 TYR B 794 4545 5733 4440 -193 -461 486 C
ATOM 2099 CD2 TYR B 794 46.258 25.034 54.529 1.00 37.45 C
ANISOU 2099 CD2 TYR B 794 4218 5950 4060 -448 -587 609 C
ATOM 2100 CE1 TYR B 794 43.457 24.909 54.727 1.00 38.60 C
ANISOU 2100 CE1 TYR B 794 4693 5582 4389 -310 -489 437 C
ATOM 2101 CE2 TYR B 794 45.559 26.019 55.256 1.00 38.01 C
ANISOU 2101 CE2 TYR B 794 4489 5857 4095 -590 -621 550 C
ATOM 2102 CZ TYR B 794 44.155 25.960 55.350 1.00 43.26 C

```
ANISOU 2102  CZ  TYR B 794      5320   6263   4853   -500   -566    464       C
ATOM   2103  OH  TYR B 794      43.466  26.911  56.046  1.00 40.83           O
ANISOU 2103  OH  TYR B 794      5220   5800   4494   -599   -585    414       O
ATOM   2104  H   TYR B 794      47.713  21.571  51.518  1.00 34.93           H
ATOM   2105  HA  TYR B 794      45.466  23.301  51.113  1.00 34.72           H
ATOM   2106  HB3 TYR B 794      47.272  22.799  53.521  1.00 35.48           H
ATOM   2107  HB2 TYR B 794      45.769  22.004  53.171  1.00 35.48           H
ATOM   2108  HD1 TYR B 794      43.602  23.131  53.524  1.00 38.74           H
ATOM   2109  HD2 TYR B 794      47.335  25.088  54.455  1.00 37.45           H
ATOM   2110  HE1 TYR B 794      42.387  24.844  54.813  1.00 38.60           H
ATOM   2111  HE2 TYR B 794      46.105  26.816  55.738  1.00 38.01           H
ATOM   2112  HH  TYR B 794      44.031  27.576  56.448  1.00 40.83           H
ATOM   2113  N   GLU B 795      48.439  24.730  51.481  1.00 35.89           N
ANISOU 2113  N   GLU B 795      3615   6209   3813   -398   -555    740       N
ATOM   2114  CA  GLU B 795      49.276  25.906  51.216  1.00 36.44           C
ANISOU 2114  CA  GLU B 795      3657   6427   3763   -676   -635    773       C
ATOM   2115  C   GLU B 795      49.249  26.371  49.746  1.00 40.28           C
ANISOU 2115  C   GLU B 795      4142   6882   4279   -713   -610    744       C
ATOM   2116  O   GLU B 795      49.362  27.571  49.496  1.00 40.33           O
ANISOU 2116  O   GLU B 795      4255   6842   4227   -945   -661    717       O
ATOM   2117  CB  GLU B 795      50.724  25.597  51.655  1.00 38.31           C
ANISOU 2117  CB  GLU B 795      3665   7046   3844   -760   -696    914       C
ATOM   2118  CG  GLU B 795      50.914  25.538  53.190  1.00 56.37           C
ANISOU 2118  CG  GLU B 795      5957   9385   6075   -770   -737    950       C
ATOM   2119  CD  GLU B 795      52.276  25.000  53.674  1.00 89.55           C
ANISOU 2119  CD  GLU B 795      9893  13998  10133   -767   -779   1106       C
ATOM   2120  OE1 GLU B 795      53.037  24.425  52.862  1.00 95.28           O
ANISOU 2120  OE1 GLU B 795     10419  14931  10853   -554   -722   1190       O
ATOM   2121  OE2 GLU B 795      52.549  25.172  54.882  1.00 85.25           O1-
ANISOU 2121  OE2 GLU B 795      9347  13575   9466   -968   -866   1151       O1-
ATOM   2122  H   GLU B 795      48.937  23.908  51.796  1.00 35.89           H
ATOM   2123  HA  GLU B 795      48.897  26.732  51.821  1.00 36.44           H
ATOM   2124  HB3 GLU B 795      51.412  26.336  51.242  1.00 38.31           H
ATOM   2125  HB2 GLU B 795      51.014  24.643  51.211  1.00 38.31           H
ATOM   2126  HG3 GLU B 795      50.136  24.924  53.643  1.00 56.37           H
ATOM   2127  HG2 GLU B 795      50.773  26.540  53.597  1.00 56.37           H
ATOM   2128  N   LYS B 796      49.085  25.418  48.813  1.00 36.52           N
ANISOU 2128  N   LYS B 796      3582   6405   3887   -481   -529    745       N
ATOM   2129  CA  LYS B 796      48.994  25.650  47.371  1.00 36.13           C
ANISOU 2129  CA  LYS B 796      3523   6326   3877   -470   -492    717       C
ATOM   2130  C   LYS B 796      47.683  26.317  46.907  1.00 39.37           C
ANISOU 2130  C   LYS B 796      4156   6424   4378   -533   -479    595       C
ATOM   2131  O   LYS B 796      47.704  26.967  45.862  1.00 39.57           O
ANISOU 2131  O   LYS B 796      4197   6428   4410   -595   -471    573       O
ATOM   2132  CB  LYS B 796      49.247  24.318  46.638  1.00 37.69           C
ANISOU 2132  CB  LYS B 796      3614   6579   4128   -186   -404    745       C
ATOM   2133  CG  LYS B 796      50.738  23.953  46.545  1.00 44.06           C
ANISOU 2133  CG  LYS B 796      4173   7756   4813   -110   -404    887       C
ATOM   2134  CD  LYS B 796      50.979  22.544  45.980  1.00 49.81           C
ANISOU 2134  CD  LYS B 796      4849   8503   5573    203   -303    912       C
ATOM   2135  CE  LYS B 796      52.473  22.266  45.744  1.00 54.60           C
ANISOU 2135  CE  LYS B 796      5202   9505   6037    318   -290   1068       C
ATOM   2136  NZ  LYS B 796      52.706  20.946  45.131  1.00 65.97           N1+
ANISOU 2136  NZ  LYS B 796      6631  10950   7485    643   -180   1093       N1+
ATOM   2137  H   LYS B 796      49.016  24.454  49.109  1.00 36.52           H
ATOM   2138  HA  LYS B 796      49.798  26.339  47.104  1.00 36.13           H
```

ATOM 2139 HB3 LYS B 796 48.872 24.388 45.621 1.00 37.69 H
ATOM 2140 HB2 LYS B 796 48.679 23.516 47.107 1.00 37.69 H
ATOM 2141 HG3 LYS B 796 51.211 24.041 47.522 1.00 44.06 H
ATOM 2142 HG2 LYS B 796 51.236 24.684 45.907 1.00 44.06 H
ATOM 2143 HD3 LYS B 796 50.415 22.425 45.054 1.00 49.81 H
ATOM 2144 HD2 LYS B 796 50.576 21.807 46.675 1.00 49.81 H
ATOM 2145 HE3 LYS B 796 53.021 22.320 46.685 1.00 54.60 H
ATOM 2146 HE2 LYS B 796 52.893 23.027 45.086 1.00 54.60 H
ATOM 2147 HZ1 LYS B 796 52.236 20.905 44.238 1.00 65.97 H
ATOM 2148 HZ2 LYS B 796 53.698 20.818 44.983 1.00 65.97 H
ATOM 2149 HZ3 LYS B 796 52.359 20.215 45.735 1.00 65.97 H
ATOM 2150 N LEU B 797 46.586 26.179 47.676 1.00 34.80 N
ANISOU 2150 N LEU B 797 3741 5622 3860 -504 -473 527 N
ATOM 2151 CA LEU B 797 45.310 26.848 47.390 1.00 33.86 C
ANISOU 2151 CA LEU B 797 3824 5232 3808 -529 -454 427 C
ATOM 2152 C LEU B 797 45.323 28.345 47.728 1.00 38.45 C
ANISOU 2152 C LEU B 797 4566 5749 4294 -763 -516 406 C
ATOM 2153 O LEU B 797 44.591 29.093 47.084 1.00 37.52 O
ANISOU 2153 O LEU B 797 4608 5446 4202 -783 -496 341 O
ATOM 2154 CB LEU B 797 44.162 26.175 48.170 1.00 33.47 C
ANISOU 2154 CB LEU B 797 3867 5003 3848 -379 -412 378 C
ATOM 2155 CG LEU B 797 43.769 24.779 47.657 1.00 37.53 C
ANISOU 2155 CG LEU B 797 4309 5502 4451 -163 -345 380 C
ATOM 2156 CD1 LEU B 797 42.791 24.124 48.640 1.00 37.86 C
ANISOU 2156 CD1 LEU B 797 4442 5397 4547 -74 -320 348 C
ATOM 2157 CD2 LEU B 797 43.182 24.812 46.232 1.00 39.89 C
ANISOU 2157 CD2 LEU B 797 4622 5720 4816 -106 -297 336 C
ATOM 2158 H LEU B 797 46.632 25.634 48.526 1.00 34.80 H
ATOM 2159 HA LEU B 797 45.112 26.767 46.320 1.00 33.86 H
ATOM 2160 HB3 LEU B 797 43.270 26.804 48.137 1.00 33.47 H
ATOM 2161 HB2 LEU B 797 44.436 26.118 49.224 1.00 33.47 H
ATOM 2162 HG LEU B 797 44.668 24.164 47.639 1.00 37.53 H
ATOM 2163 HD11 LEU B 797 42.566 23.097 48.350 1.00 37.86 H
ATOM 2164 HD12 LEU B 797 43.208 24.108 49.646 1.00 37.86 H
ATOM 2165 HD13 LEU B 797 41.849 24.671 48.686 1.00 37.86 H
ATOM 2166 HD21 LEU B 797 42.198 24.342 46.192 1.00 39.89 H
ATOM 2167 HD22 LEU B 797 43.064 25.828 45.855 1.00 39.89 H
ATOM 2168 HD23 LEU B 797 43.822 24.283 45.528 1.00 39.89 H
ATOM 2169 N LYS B 798 46.120 28.746 48.737 1.00 36.35 N
ANISOU 2169 N LYS B 798 4276 5633 3902 -936 -589 466 N
ATOM 2170 CA LYS B 798 46.238 30.118 49.248 1.00 36.76 C
ANISOU 2170 CA LYS B 798 4520 5622 3823 -1189 -657 453 C
ATOM 2171 C LYS B 798 44.880 30.691 49.698 1.00 41.09 C
ANISOU 2171 C LYS B 798 5336 5870 4406 -1133 -625 363 C
ATOM 2172 O LYS B 798 44.466 31.764 49.254 1.00 41.05 O
ANISOU 2172 O LYS B 798 5543 5708 4346 -1231 -629 319 O
ATOM 2173 CB LYS B 798 47.007 31.002 48.239 1.00 40.18 C
ANISOU 2173 CB LYS B 798 4958 6136 4171 -1386 -691 476 C
ATOM 2174 CG LYS B 798 48.455 30.517 48.061 1.00 63.40 C
ANISOU 2174 CG LYS B 798 7605 9398 7086 -1391 -701 574 C
ATOM 2175 CD LYS B 798 49.349 31.510 47.303 1.00 75.21 C
ANISOU 2175 CD LYS B 798 9046 11138 8393 -1706 -796 663 C
ATOM 2176 CE LYS B 798 50.789 31.003 47.095 1.00 85.89 C
ANISOU 2176 CE LYS B 798 10058 12878 9698 -1672 -804 789 C
ATOM 2177 NZ LYS B 798 51.510 30.807 48.368 1.00 93.37 N1+
ANISOU 2177 NZ LYS B 798 10863 14083 10529 -1755 -868 887 N1+

```
ATOM   2178  H   LYS B 798      46.690  28.057  49.207  1.00 36.35           H
ATOM   2179  HA  LYS B 798      46.838  30.052  50.157  1.00 36.76           H
ATOM   2180  HB3 LYS B 798      47.027  32.028  48.602  1.00 40.18           H
ATOM   2181  HB2 LYS B 798      46.499  31.035  47.274  1.00 40.18           H
ATOM   2182  HG3 LYS B 798      48.456  29.561  47.536  1.00 63.40           H
ATOM   2183  HG2 LYS B 798      48.878  30.323  49.048  1.00 63.40           H
ATOM   2184  HD3 LYS B 798      49.366  32.464  47.831  1.00 75.21           H
ATOM   2185  HD2 LYS B 798      48.903  31.718  46.330  1.00 75.21           H
ATOM   2186  HE3 LYS B 798      51.346  31.721  46.493  1.00 85.89           H
ATOM   2187  HE2 LYS B 798      50.782  30.063  46.543  1.00 85.89           H
ATOM   2188  HZ1 LYS B 798      51.023  30.119  48.925  1.00 93.37           H
ATOM   2189  HZ2 LYS B 798      52.450  30.487  48.183  1.00 93.37           H
ATOM   2190  HZ3 LYS B 798      51.543  31.681  48.874  1.00 93.37           H
ATOM   2191  N   THR B 799      44.201  29.901  50.543  1.00 36.80           N
ANISOU 2191  N   THR B 799     4777   5257   3948   -952   -585    344       N
ATOM   2192  CA  THR B 799      42.864  30.155  51.062  1.00 36.50           C
ANISOU 2192  CA  THR B 799     4939   4985   3944   -853   -544    277       C
ATOM   2193  C   THR B 799      42.815  29.660  52.514  1.00 39.61           C
ANISOU 2193  C   THR B 799     5340   5394   4318   -821   -561    295       C
ATOM   2194  O   THR B 799      43.196  28.517  52.776  1.00 39.38           O
ANISOU 2194  O   THR B 799     5124   5499   4339   -731   -556    338       O
ATOM   2195  CB  THR B 799      41.789  29.369  50.255  1.00 40.40           C
ANISOU 2195  CB  THR B 799     5380   5382   4587   -637   -461    237       C
ATOM   2196  CG2 THR B 799      40.341  29.493  50.770  1.00 36.53           C
ANISOU 2196  CG2 THR B 799     5064   4696   4118   -529   -412    186       C
ATOM   2197  OG1 THR B 799      41.785  29.814  48.915  1.00 36.47           O
ANISOU 2197  OG1 THR B 799     4848   4898   4112   -661   -447    227       O
ATOM   2198  H   THR B 799      44.623  29.038  50.854  1.00 36.80           H
ATOM   2199  HA  THR B 799      42.647  31.224  51.038  1.00 36.50           H
ATOM   2200  HB  THR B 799      42.056  28.310  50.234  1.00 40.40           H
ATOM   2201 HG21 THR B 799      39.654  28.931  50.139  1.00 36.53           H
ATOM   2202 HG22 THR B 799      40.224  29.104  51.780  1.00 36.53           H
ATOM   2203 HG23 THR B 799      40.011  30.530  50.772  1.00 36.53           H
ATOM   2204  HG1 THR B 799      42.657  29.673  48.536  1.00 36.47           H
ATOM   2205  N   ASP B 800      42.307  30.514  53.417  1.00 35.19           N
ANISOU 2205  N   ASP B 800     5012   4686   3674   -875   -572    263       N
ATOM   2206  CA  ASP B 800      41.976  30.155  54.794  1.00 35.06           C
ANISOU 2206  CA  ASP B 800     5034   4649   3637   -828   -578    270       C
ATOM   2207  C   ASP B 800      40.629  29.411  54.782  1.00 36.53           C
ANISOU 2207  C   ASP B 800     5216   4713   3951   -590   -492    234       C
ATOM   2208  O   ASP B 800      39.623  30.026  54.429  1.00 36.07           O
ANISOU 2208  O   ASP B 800     5292   4508   3905   -517   -442    191       O
ATOM   2209  CB  ASP B 800      41.927  31.413  55.702  1.00 37.64           C
ANISOU 2209  CB  ASP B 800     5642   4861   3799   -986   -621    250       C
ATOM   2210  CG  ASP B 800      41.629  31.173  57.196  1.00 53.60           C
ANISOU 2210  CG  ASP B 800     7724   6855   5787   -939   -626    255       C
ATOM   2211  OD1 ASP B 800      41.991  30.091  57.712  1.00 54.97           O
ANISOU 2211  OD1 ASP B 800     7723   7203   5962   -971   -669    308       O
ATOM   2212  OD2 ASP B 800      41.175  32.144  57.840  1.00 62.80           O1-
ANISOU 2212  OD2 ASP B 800     9111   7835   6915   -857   -584    212       O1-
ATOM   2213  H   ASP B 800      42.009  31.435  53.119  1.00 35.19           H
ATOM   2214  HA  ASP B 800      42.754  29.488  55.171  1.00 35.06           H
ATOM   2215  HB3 ASP B 800      41.208  32.126  55.296  1.00 37.64           H
ATOM   2216  HB2 ASP B 800      42.888  31.924  55.640  1.00 37.64           H
ATOM   2217  N   ILE B 801      40.642  28.119  55.144  1.00 31.31           N
ANISOU 2217  N   ILE B 801     4392   4131   3373   -473   -474    262       N
```

ATOM 2218 CA ILE B 801 39.450 27.271 55.191 1.00 30.10 C
ANISOU 2218 CA ILE B 801 4216 3894 3327 -287 -403 243 C
ATOM 2219 C ILE B 801 39.243 26.851 56.657 1.00 33.29 C
ANISOU 2219 C ILE B 801 4665 4279 3703 -244 -404 258 C
ATOM 2220 O ILE B 801 40.078 26.139 57.217 1.00 33.41 O
ANISOU 2220 O ILE B 801 4586 4408 3699 -270 -442 301 O
ATOM 2221 CB ILE B 801 39.587 25.981 54.321 1.00 31.94 C
ANISOU 2221 CB ILE B 801 4262 4199 3675 -185 -370 260 C
ATOM 2222 CG1 ILE B 801 40.053 26.284 52.875 1.00 32.03 C
ANISOU 2222 CG1 ILE B 801 4228 4236 3707 -228 -368 247 C
ATOM 2223 CG2 ILE B 801 38.284 25.150 54.298 1.00 30.64 C
ANISOU 2223 CG2 ILE B 801 4098 3947 3597 -42 -306 245 C
ATOM 2224 CD1 ILE B 801 40.399 25.037 52.047 1.00 34.81 C
ANISOU 2224 CD1 ILE B 801 4417 4667 4142 -136 -339 266 C
ATOM 2225 H ILE B 801 41.509 27.686 55.435 1.00 31.31 H
ATOM 2226 HA ILE B 801 38.567 27.816 54.850 1.00 30.10 H
ATOM 2227 HB ILE B 801 40.343 25.345 54.775 1.00 31.94 H
ATOM 2228 HG13 ILE B 801 40.939 26.918 52.887 1.00 32.03 H
ATOM 2229 HG12 ILE B 801 39.285 26.858 52.356 1.00 32.03 H
ATOM 2230 HG21 ILE B 801 38.418 24.215 53.756 1.00 30.64 H
ATOM 2231 HG22 ILE B 801 37.944 24.880 55.297 1.00 30.64 H
ATOM 2232 HG23 ILE B 801 37.479 25.699 53.811 1.00 30.64 H
ATOM 2233 HD11 ILE B 801 41.155 25.269 51.296 1.00 34.81 H
ATOM 2234 HD12 ILE B 801 40.791 24.232 52.669 1.00 34.81 H
ATOM 2235 HD13 ILE B 801 39.522 24.659 51.521 1.00 34.81 H
ATOM 2236 N LYS B 802 38.128 27.312 57.239 1.00 29.39 N
ANISOU 2236 N LYS B 802 4310 3660 3198 -165 -360 231 N
ATOM 2237 CA LYS B 802 37.706 27.018 58.609 1.00 29.15 C
ANISOU 2237 CA LYS B 802 4334 3605 3136 -112 -351 244 C
ATOM 2238 C LYS B 802 36.353 26.304 58.577 1.00 32.66 C
ANISOU 2238 C LYS B 802 4744 4005 3661 44 -278 244 C
ATOM 2239 O LYS B 802 35.498 26.675 57.777 1.00 31.94 O
ANISOU 2239 O LYS B 802 4666 3870 3601 101 -233 226 O
ATOM 2240 CB LYS B 802 37.588 28.345 59.387 1.00 31.97 C
ANISOU 2240 CB LYS B 802 4930 3868 3349 -179 -370 224 C
ATOM 2241 CG LYS B 802 38.950 28.951 59.760 1.00 51.49 C
ANISOU 2241 CG LYS B 802 7458 6398 5707 -382 -458 234 C
ATOM 2242 CD LYS B 802 38.827 30.368 60.336 1.00 67.57 C
ANISOU 2242 CD LYS B 802 9787 8305 7583 -453 -472 209 C
ATOM 2243 CE LYS B 802 40.152 30.876 60.927 1.00 83.24 C
ANISOU 2243 CE LYS B 802 11852 10348 9426 -704 -569 223 C
ATOM 2244 NZ LYS B 802 40.113 32.324 61.192 1.00 94.98 N1+
ANISOU 2244 NZ LYS B 802 13681 11674 10731 -786 -582 192 N1+
ATOM 2245 H LYS B 802 37.500 27.900 56.708 1.00 29.39 H
ATOM 2246 HA LYS B 802 38.429 26.372 59.110 1.00 29.15 H
ATOM 2247 HB3 LYS B 802 37.036 28.181 60.313 1.00 31.97 H
ATOM 2248 HB2 LYS B 802 37.000 29.060 58.808 1.00 31.97 H
ATOM 2249 HG3 LYS B 802 39.598 28.981 58.884 1.00 51.49 H
ATOM 2250 HG2 LYS B 802 39.446 28.299 60.481 1.00 51.49 H
ATOM 2251 HD3 LYS B 802 38.050 30.387 61.102 1.00 67.57 H
ATOM 2252 HD2 LYS B 802 38.491 31.035 59.540 1.00 67.57 H
ATOM 2253 HE3 LYS B 802 40.975 30.682 60.242 1.00 83.24 H
ATOM 2254 HE2 LYS B 802 40.381 30.344 61.852 1.00 83.24 H
ATOM 2255 HZ1 LYS B 802 39.364 32.539 61.833 1.00 94.98 H
ATOM 2256 HZ2 LYS B 802 40.996 32.618 61.584 1.00 94.98 H
ATOM 2257 HZ3 LYS B 802 39.965 32.809 60.316 1.00 94.98 H

```
ATOM   2258  N   VAL B 803      36.168  25.322  59.473  1.00 29.13           N
ANISOU 2258  N   VAL B 803     4250   3582   3234    101   -267    272       N
ATOM   2259  CA  VAL B 803      34.898  24.616  59.648  1.00 28.68           C
ANISOU 2259  CA  VAL B 803     4157   3507   3233    215   -205    286       C
ATOM   2260  C   VAL B 803      33.920  25.497  60.449  1.00 33.51           C
ANISOU 2260  C   VAL B 803     4914   4058   3758    281   -167    283       C
ATOM   2261  O   VAL B 803      34.270  25.955  61.538  1.00 33.58           O
ANISOU 2261  O   VAL B 803     5043   4039   3677    255   -190    282       O
ATOM   2262  CB  VAL B 803      35.086  23.271  60.412  1.00 32.46           C
ANISOU 2262  CB  VAL B 803     4543   4032   3760    245   -206    323       C
ATOM   2263  CG1 VAL B 803      33.768  22.508  60.669  1.00 32.80           C
ANISOU 2263  CG1 VAL B 803     4558   4062   3843    322   -147    344       C
ATOM   2264  CG2 VAL B 803      36.073  22.346  59.682  1.00 32.04           C
ANISOU 2264  CG2 VAL B 803     4373   4036   3764    223   -231    335       C
ATOM   2265  H   VAL B 803      36.906  25.080  60.117  1.00 29.13           H
ATOM   2266  HA  VAL B 803      34.472  24.399  58.665  1.00 28.68           H
ATOM   2267  HB  VAL B 803      35.525  23.486  61.388  1.00 32.46           H
ATOM   2268 HG11 VAL B 803      33.959  21.529  61.108  1.00 32.80           H
ATOM   2269 HG12 VAL B 803      33.113  23.036  61.362  1.00 32.80           H
ATOM   2270 HG13 VAL B 803      33.216  22.350  59.741  1.00 32.80           H
ATOM   2271 HG21 VAL B 803      36.183  21.392  60.200  1.00 32.04           H
ATOM   2272 HG22 VAL B 803      35.736  22.138  58.668  1.00 32.04           H
ATOM   2273 HG23 VAL B 803      37.065  22.793  59.624  1.00 32.04           H
ATOM   2274  N   VAL B 804      32.713  25.693  59.899  1.00 30.53           N
ANISOU 2274  N   VAL B 804     4531   3676   3394    371   -106    289       N
ATOM   2275  CA  VAL B 804      31.605  26.361  60.578  1.00 30.17           C
ANISOU 2275  CA  VAL B 804     4602   3605   3256    483    -50    304       C
ATOM   2276  C   VAL B 804      30.897  25.349  61.496  1.00 36.42           C
ANISOU 2276  C   VAL B 804     5316   4457   4064    541    -20    351       C
ATOM   2277  O   VAL B 804      30.528  24.266  61.037  1.00 35.89           O
ANISOU 2277  O   VAL B 804     5102   4453   4080    531     -7    377       O
ATOM   2278  CB  VAL B 804      30.579  26.931  59.556  1.00 33.32           C
ANISOU 2278  CB  VAL B 804     5008   4014   3640    571      5    309       C
ATOM   2279  CG1 VAL B 804      29.255  27.429  60.164  1.00 32.87           C
ANISOU 2279  CG1 VAL B 804     5050   3970   3471    735     79    347       C
ATOM   2280  CG2 VAL B 804      31.209  28.049  58.715  1.00 33.05           C
ANISOU 2280  CG2 VAL B 804     5078   3904   3575    510    -23    264       C
ATOM   2281  H   VAL B 804      32.490  25.268  59.007  1.00 30.53           H
ATOM   2282  HA  VAL B 804      31.991  27.187  61.178  1.00 30.17           H
ATOM   2283  HB  VAL B 804      30.319  26.132  58.865  1.00 33.32           H
ATOM   2284 HG11 VAL B 804      28.622  27.897  59.409  1.00 32.87           H
ATOM   2285 HG12 VAL B 804      28.682  26.607  60.585  1.00 32.87           H
ATOM   2286 HG13 VAL B 804      29.429  28.159  60.955  1.00 32.87           H
ATOM   2287 HG21 VAL B 804      30.501  28.452  57.989  1.00 33.05           H
ATOM   2288 HG22 VAL B 804      31.543  28.874  59.345  1.00 33.05           H
ATOM   2289 HG23 VAL B 804      32.070  27.680  58.158  1.00 33.05           H
ATOM   2290  N   ASP B 805      30.716  25.739  62.768  1.00 35.02           N
ANISOU 2290  N   ASP B 805     5258   4253   3794    590     -9    363       N
ATOM   2291  CA  ASP B 805      29.998  24.979  63.792  1.00 35.63           C
ANISOU 2291  CA  ASP B 805     5282   4389   3867    648     23    412       C
ATOM   2292  C   ASP B 805      28.515  24.839  63.398  1.00 39.34           C
ANISOU 2292  C   ASP B 805     5655   4957   4336    747     96    464       C
ATOM   2293  O   ASP B 805      27.874  25.845  63.100  1.00 39.06           O
ANISOU 2293  O   ASP B 805     5690   4931   4221    857    146    474       O
ATOM   2294  CB  ASP B 805      30.188  25.648  65.180  1.00 37.92           C
ANISOU 2294  CB  ASP B 805     5749   4625   4033    698     28    410       C
```

ATOM 2295 CG ASP B 805 29.554 24.978 66.417 1.00 49.68 C
ANISOU 2295 CG ASP B 805 7192 6166 5518 729 44 456 C
ATOM 2296 OD1 ASP B 805 29.150 23.796 66.342 1.00 49.62 O
ANISOU 2296 OD1 ASP B 805 7053 6251 5551 775 90 507 O
ATOM 2297 OD2 ASP B 805 29.575 25.649 67.472 1.00 59.27 O1-
ANISOU 2297 OD2 ASP B 805 8506 7337 6676 694 7 444 O1-
ATOM 2298 H ASP B 805 31.054 26.646 63.056 1.00 35.02 H
ATOM 2299 HA ASP B 805 30.448 23.985 63.828 1.00 35.63 H
ATOM 2300 HB3 ASP B 805 29.842 26.682 65.127 1.00 37.92 H
ATOM 2301 HB2 ASP B 805 31.259 25.727 65.374 1.00 37.92 H
ATOM 2302 N ARG B 806 28.006 23.596 63.418 1.00 36.04 N
ANISOU 2302 N ARG B 806 5084 4619 3989 704 102 506 N
ATOM 2303 CA ARG B 806 26.619 23.236 63.109 1.00 36.42 C
ANISOU 2303 CA ARG B 806 5006 4802 4030 743 156 572 C
ATOM 2304 C ARG B 806 25.574 23.969 63.972 1.00 40.08 C
ANISOU 2304 C ARG B 806 5503 5357 4369 899 229 633 C
ATOM 2305 O ARG B 806 24.488 24.251 63.472 1.00 39.10 O
ANISOU 2305 O ARG B 806 5291 5365 4200 972 280 689 O
ATOM 2306 CB ARG B 806 26.439 21.713 63.277 1.00 38.07 C
ANISOU 2306 CB ARG B 806 5104 5054 4307 634 140 607 C
ATOM 2307 CG ARG B 806 27.239 20.848 62.286 1.00 56.04 C
ANISOU 2307 CG ARG B 806 7329 7282 6684 516 97 571 C
ATOM 2308 CD ARG B 806 27.174 19.358 62.662 1.00 69.54 C
ANISOU 2308 CD ARG B 806 9020 8964 8440 423 76 590 C
ATOM 2309 NE ARG B 806 27.662 18.474 61.586 1.00 78.89 N
ANISOU 2309 NE ARG B 806 10178 10105 9693 330 52 565 N
ATOM 2310 CZ ARG B 806 26.965 17.529 60.922 1.00 92.34 C
ANISOU 2310 CZ ARG B 806 11832 11855 11397 235 63 602 C
ATOM 2311 NH1 ARG B 806 27.587 16.805 59.986 1.00 82.85 N
ANISOU 2311 NH1 ARG B 806 10646 10589 10245 159 43 572 N
ATOM 2312 NH2 ARG B 806 25.669 17.281 61.165 1.00 73.39 N1+
ANISOU 2312 NH2 ARG B 806 9374 9574 8935 207 95 674 N1+
ATOM 2313 H ARG B 806 28.604 22.835 63.708 1.00 36.04 H
ATOM 2314 HA ARG B 806 26.433 23.503 62.068 1.00 36.42 H
ATOM 2315 HB3 ARG B 806 25.384 21.461 63.152 1.00 38.07 H
ATOM 2316 HB2 ARG B 806 26.691 21.429 64.300 1.00 38.07 H
ATOM 2317 HG3 ARG B 806 28.282 21.158 62.369 1.00 56.04 H
ATOM 2318 HG2 ARG B 806 26.952 21.029 61.249 1.00 56.04 H
ATOM 2319 HD3 ARG B 806 26.213 19.076 63.092 1.00 69.54 H
ATOM 2320 HD2 ARG B 806 27.901 19.194 63.458 1.00 69.54 H
ATOM 2321 HE ARG B 806 28.632 18.604 61.333 1.00 78.89 H
ATOM 2322 HH12 ARG B 806 27.085 16.101 59.463 1.00 82.85 H
ATOM 2323 HH11 ARG B 806 28.545 17.006 59.733 1.00 82.85 H
ATOM 2324 HH22 ARG B 806 25.174 16.570 60.646 1.00 73.39 H
ATOM 2325 HH21 ARG B 806 25.179 17.809 61.872 1.00 73.39 H
ATOM 2326 N ASP B 807 25.919 24.271 65.233 1.00 38.11 N
ANISOU 2326 N ASP B 807 5374 5054 4051 958 235 630 N
ATOM 2327 CA ASP B 807 25.026 24.893 66.214 1.00 38.51 C
ANISOU 2327 CA ASP B 807 5484 5182 3967 1129 311 689 C
ATOM 2328 C ASP B 807 25.033 26.438 66.165 1.00 40.97 C
ANISOU 2328 C ASP B 807 6009 5403 4156 1279 347 657 C
ATOM 2329 O ASP B 807 24.268 27.048 66.910 1.00 41.16 O
ANISOU 2329 O ASP B 807 6132 5464 4045 1452 418 701 O
ATOM 2330 CB ASP B 807 25.295 24.366 67.650 1.00 41.54 C
ANISOU 2330 CB ASP B 807 5901 5555 4329 1115 304 708 C
ATOM 2331 CG ASP B 807 25.265 22.832 67.834 1.00 58.05 C

ANISOU 2331 CG ASP B 807   7839   7698   6521   970   269   736   C
ATOM   2332 OD1 ASP B 807   24.616 22.132 67.022 1.00 59.28   O
ANISOU 2332 OD1 ASP B 807   7836   7989   6700   928   292   796   O
ATOM   2333 OD2 ASP B 807   25.782 22.389 68.883 1.00 66.42   O1-
ANISOU 2333 OD2 ASP B 807   8949   8666   7622   893   218   705   O1-
ATOM   2334 H   ASP B 807   26.838 24.015 65.572 1.00 38.11   H
ATOM   2335 HA ASP B 807   24.000 24.611 65.967 1.00 38.51   H
ATOM   2336 HB3 ASP B 807   24.559 24.783 68.338 1.00 41.54   H
ATOM   2337 HB2 ASP B 807   26.275 24.721 67.979 1.00 41.54   H
ATOM   2338 N   SER B 808   25.861 27.049 65.294 1.00 35.86   N
ANISOU 2338 N   SER B 808   5451   4634   3541   1217   305   586   N
ATOM   2339 CA SER B 808   25.920 28.501 65.083 1.00 34.53   C
ANISOU 2339 CA SER B 808   5529   4346   3246   1330   334   551   C
ATOM   2340 C   SER B 808   24.733 29.035 64.254 1.00 38.17   C
ANISOU 2340 C   SER B 808   5963   4909   3629   1517   419   607   C
ATOM   2341 O   SER B 808   24.126 28.282 63.492 1.00 37.28   O
ANISOU 2341 O   SER B 808   5613   4967   3586   1506   435   663   O
ATOM   2342 CB SER B 808   27.282 28.871 64.449 1.00 35.94   C
ANISOU 2342 CB SER B 808   5813   4368   3474   1165   250   463   C
ATOM   2343 OG SER B 808   27.345 28.602 63.061 1.00 38.00   O
ANISOU 2343 OG SER B 808   5929   4670   3840   1097   230   452   O
ATOM   2344 H   SER B 808   26.458 26.487 64.704 1.00 35.86   H
ATOM   2345 HA SER B 808   25.881 28.973 66.067 1.00 34.53   H
ATOM   2346 HB3 SER B 808   28.097 28.352 64.954 1.00 35.94   H
ATOM   2347 HB2 SER B 808   27.469 29.937 64.582 1.00 35.94   H
ATOM   2348 HG SER B 808   27.473 27.654 62.944 1.00 38.00   H
ATOM   2349 N   GLU B 809   24.463 30.345 64.391 1.00 34.99   N
ANISOU 2349 N   GLU B 809   5822   4405   3067   1687   476   597   N
ATOM   2350 CA GLU B 809   23.410 31.064 63.667 1.00 34.37   C
ANISOU 2350 CA GLU B 809   5761   4414   2884   1908   566   656   C
ATOM   2351 C   GLU B 809   23.616 31.102 62.140 1.00 36.99   C
ANISOU 2351 C   GLU B 809   5984   4754   3316   1813   530   630   C
ATOM   2352 O   GLU B 809   22.637 30.961 61.408 1.00 34.95   O
ANISOU 2352 O   GLU B 809   5536   4686   3057   1910   578   704   O
ATOM   2353 CB GLU B 809   23.249 32.479 64.266 1.00 36.01   C
ANISOU 2353 CB GLU B 809   6340   4465   2877   2122   638   645   C
ATOM   2354 CG GLU B 809   22.153 33.344 63.591 1.00 46.99   C
ANISOU 2354 CG GLU B 809   7737   5994   4125   2428   759   738   C
ATOM   2355 CD GLU B 809   21.956 34.751 64.178 1.00 60.35   C
ANISOU 2355 CD GLU B 809   9835   7507   5589   2672   843   729   C
ATOM   2356 OE1 GLU B 809   22.860 35.244 64.890 1.00 40.80   O
ANISOU 2356 OE1 GLU B 809   7670   4757   3074   2548   789   629   O
ATOM   2357 OE2 GLU B 809   20.889 35.331 63.880 1.00 50.60   O1-
ANISOU 2357 OE2 GLU B 809   8613   6417   4196   2989   965   830   O1-
ATOM   2358 H   GLU B 809   25.007 30.899 65.038 1.00 34.99   H
ATOM   2359 HA GLU B 809   22.479 30.524 63.848 1.00 34.37   H
ATOM   2360 HB3 GLU B 809   24.210 32.995 64.217 1.00 36.01   H
ATOM   2361 HB2 GLU B 809   23.012 32.384 65.327 1.00 36.01   H
ATOM   2362 HG3 GLU B 809   21.204 32.807 63.624 1.00 46.99   H
ATOM   2363 HG2 GLU B 809   22.387 33.489 62.536 1.00 46.99   H
ATOM   2364 N   GLU B 810   24.872 31.268 61.681 1.00 33.98   N
ANISOU 2364 N   GLU B 810   5712   4191   3009   1620   444   533   N
ATOM   2365 CA GLU B 810   25.215 31.269 60.255 1.00 34.35   C
ANISOU 2365 CA GLU B 810   5668   4234   3148   1524   408   504   C
ATOM   2366 C   GLU B 810   25.006 29.897 59.584 1.00 35.35   C
ANISOU 2366 C   GLU B 810   5457   4537   3439   1401   374   535   C

ATOM 2367 O GLU B 810 24.650 29.868 58.408 1.00 34.26 O
ANISOU 2367 O GLU B 810 5194 4492 3332 1417 388 559 O
ATOM 2368 CB GLU B 810 26.635 31.837 60.027 1.00 36.36 C
ANISOU 2368 CB GLU B 810 6109 4282 3424 1344 327 407 C
ATOM 2369 CG GLU B 810 27.791 31.061 60.709 1.00 49.46 C
ANISOU 2369 CG GLU B 810 7729 5889 5175 1125 234 359 C
ATOM 2370 CD GLU B 810 29.208 31.594 60.421 1.00 81.31 C
ANISOU 2370 CD GLU B 810 11934 9767 9193 939 154 285 C
ATOM 2371 OE1 GLU B 810 29.352 32.579 59.662 1.00 78.14 O
ANISOU 2371 OE1 GLU B 810 11654 9286 8748 931 155 259 O
ATOM 2372 OE2 GLU B 810 30.155 30.994 60.973 1.00 80.69 O1-
ANISOU 2372 OE2 GLU B 810 11860 9662 9137 794 87 261 O1-
ATOM 2373 H GLU B 810 25.638 31.372 62.331 1.00 33.98 H
ATOM 2374 HA GLU B 810 24.526 31.959 59.764 1.00 34.35 H
ATOM 2375 HB3 GLU B 810 26.650 32.876 60.360 1.00 36.36 H
ATOM 2376 HB2 GLU B 810 26.814 31.874 58.951 1.00 36.36 H
ATOM 2377 HG3 GLU B 810 27.762 30.017 60.401 1.00 49.46 H
ATOM 2378 HG2 GLU B 810 27.643 31.069 61.789 1.00 49.46 H
ATOM 2379 N ALA B 811 25.166 28.800 60.347 1.00 30.81 N
ANISOU 2379 N ALA B 811 4756 4005 2945 1287 336 541 N
ATOM 2380 CA ALA B 811 24.868 27.438 59.902 1.00 30.18 C
ANISOU 2380 CA ALA B 811 4411 4066 2990 1166 310 574 C
ATOM 2381 C ALA B 811 23.361 27.177 59.754 1.00 32.88 C
ANISOU 2381 C ALA B 811 4590 4640 3265 1291 383 682 C
ATOM 2382 O ALA B 811 22.967 26.532 58.784 1.00 30.98 O
ANISOU 2382 O ALA B 811 4163 4523 3085 1208 370 712 O
ATOM 2383 CB ALA B 811 25.496 26.436 60.875 1.00 30.82 C
ANISOU 2383 CB ALA B 811 4451 4116 3142 1038 261 560 C
ATOM 2384 H ALA B 811 25.454 28.903 61.310 1.00 30.81 H
ATOM 2385 HA ALA B 811 25.338 27.298 58.926 1.00 30.18 H
ATOM 2386 HB1 ALA B 811 25.448 25.422 60.482 1.00 30.82 H
ATOM 2387 HB2 ALA B 811 26.543 26.670 61.038 1.00 30.82 H
ATOM 2388 HB3 ALA B 811 25.004 26.443 61.848 1.00 30.82 H
ATOM 2389 N GLU B 812 22.549 27.708 60.687 1.00 31.38 N
ANISOU 2389 N GLU B 812 4468 4522 2934 1489 459 746 N
ATOM 2390 CA GLU B 812 21.083 27.662 60.651 1.00 31.66 C
ANISOU 2390 CA GLU B 812 4339 4824 2867 1643 540 871 C
ATOM 2391 C GLU B 812 20.489 28.424 59.453 1.00 36.90 C
ANISOU 2391 C GLU B 812 4965 5578 3476 1759 579 905 C
ATOM 2392 O GLU B 812 19.570 27.911 58.815 1.00 37.00 O
ANISOU 2392 O GLU B 812 4742 5832 3485 1737 593 991 O
ATOM 2393 CB GLU B 812 20.508 28.204 61.976 1.00 33.11 C
ANISOU 2393 CB GLU B 812 4639 5051 2890 1870 624 931 C
ATOM 2394 CG GLU B 812 20.822 27.315 63.201 1.00 45.54 C
ANISOU 2394 CG GLU B 812 6185 6622 4497 1777 601 936 C
ATOM 2395 CD GLU B 812 20.439 27.908 64.569 1.00 67.70 C
ANISOU 2395 CD GLU B 812 9157 9416 7149 1987 674 972 C
ATOM 2396 OE1 GLU B 812 19.971 29.068 64.626 1.00 50.89 O
ANISOU 2396 OE1 GLU B 812 7220 7244 4871 2224 749 983 O
ATOM 2397 OE2 GLU B 812 20.624 27.167 65.559 1.00 64.38 O1-
ANISOU 2397 OE2 GLU B 812 8695 9019 6747 1920 661 989 O1-
ATOM 2398 H GLU B 812 22.955 28.215 61.462 1.00 31.38 H
ATOM 2399 HA GLU B 812 20.785 26.616 60.551 1.00 31.66 H
ATOM 2400 HB3 GLU B 812 19.427 28.320 61.889 1.00 33.11 H
ATOM 2401 HB2 GLU B 812 20.902 29.206 62.143 1.00 33.11 H
ATOM 2402 HG3 GLU B 812 21.883 27.073 63.229 1.00 45.54 H

ATOM 2403 HG2 GLU B 812 20.301 26.364 63.086 1.00 45.54 H
ATOM 2404 N ILE B 813 21.054 29.607 59.152 1.00 34.27 N
ANISOU 2404 N ILE B 813 4875 5059 3089 1867 594 842 N
ATOM 2405 CA ILE B 813 20.720 30.440 57.993 1.00 34.27 C
ANISOU 2405 CA ILE B 813 4887 5102 3032 1987 631 863 C
ATOM 2406 C ILE B 813 21.058 29.753 56.654 1.00 36.70 C
ANISOU 2406 C ILE B 813 5005 5444 3496 1760 554 829 C
ATOM 2407 O ILE B 813 20.242 29.802 55.734 1.00 36.81 O
ANISOU 2407 O ILE B 813 4834 5668 3483 1803 579 905 O
ATOM 2408 CB ILE B 813 21.422 31.833 58.087 1.00 38.04 C
ANISOU 2408 CB ILE B 813 5725 5324 3406 2122 658 794 C
ATOM 2409 CG1 ILE B 813 20.760 32.683 59.198 1.00 38.14 C
ANISOU 2409 CG1 ILE B 813 5935 5347 3210 2420 765 858 C
ATOM 2410 CG2 ILE B 813 21.496 32.640 56.766 1.00 39.89 C
ANISOU 2410 CG2 ILE B 813 5995 5538 3623 2174 669 786 C
ATOM 2411 CD1 ILE B 813 21.583 33.903 59.638 1.00 41.09 C
ANISOU 2411 CD1 ILE B 813 6731 5396 3486 2453 763 764 C
ATOM 2412 H ILE B 813 21.787 29.963 59.752 1.00 34.27 H
ATOM 2413 HA ILE B 813 19.640 30.599 58.008 1.00 34.27 H
ATOM 2414 HB ILE B 813 22.453 31.654 58.396 1.00 38.04 H
ATOM 2415 HG13 ILE B 813 20.578 32.071 60.082 1.00 38.14 H
ATOM 2416 HG12 ILE B 813 19.773 33.008 58.869 1.00 38.14 H
ATOM 2417 HG21 ILE B 813 21.944 33.620 56.917 1.00 39.89 H
ATOM 2418 HG22 ILE B 813 22.109 32.146 56.012 1.00 39.89 H
ATOM 2419 HG23 ILE B 813 20.504 32.793 56.344 1.00 39.89 H
ATOM 2420 HD11 ILE B 813 21.208 34.298 60.583 1.00 41.09 H
ATOM 2421 HD12 ILE B 813 22.632 33.644 59.784 1.00 41.09 H
ATOM 2422 HD13 ILE B 813 21.531 34.710 58.908 1.00 41.09 H
ATOM 2423 N ILE B 814 22.233 29.102 56.586 1.00 32.06 N
ANISOU 2423 N ILE B 814 4457 4670 3055 1528 463 724 N
ATOM 2424 CA ILE B 814 22.706 28.368 55.413 1.00 31.36 C
ANISOU 2424 CA ILE B 814 4228 4580 3109 1320 392 681 C
ATOM 2425 C ILE B 814 21.872 27.105 55.110 1.00 34.80 C
ANISOU 2425 C ILE B 814 4396 5244 3582 1215 384 759 C
ATOM 2426 O ILE B 814 21.537 26.899 53.945 1.00 34.51 O
ANISOU 2426 O ILE B 814 4227 5322 3565 1161 374 784 O
ATOM 2427 CB ILE B 814 24.229 28.047 55.516 1.00 33.72 C
ANISOU 2427 CB ILE B 814 4628 4655 3528 1132 309 569 C
ATOM 2428 CG1 ILE B 814 25.055 29.336 55.289 1.00 33.66 C
ANISOU 2428 CG1 ILE B 814 4864 4453 3470 1180 305 501 C
ATOM 2429 CG2 ILE B 814 24.745 26.934 54.578 1.00 32.78 C
ANISOU 2429 CG2 ILE B 814 4352 4556 3547 935 247 539 C
ATOM 2430 CD1 ILE B 814 26.510 29.247 55.771 1.00 31.44 C
ANISOU 2430 CD1 ILE B 814 4704 3990 3252 1020 231 415 C
ATOM 2431 H ILE B 814 22.856 29.116 57.383 1.00 32.06 H
ATOM 2432 HA ILE B 814 22.574 29.032 54.555 1.00 31.36 H
ATOM 2433 HB ILE B 814 24.418 27.712 56.537 1.00 33.72 H
ATOM 2434 HG13 ILE B 814 24.583 30.182 55.791 1.00 33.66 H
ATOM 2435 HG12 ILE B 814 25.042 29.592 54.230 1.00 33.66 H
ATOM 2436 HG21 ILE B 814 25.824 26.808 54.661 1.00 32.78 H
ATOM 2437 HG22 ILE B 814 24.307 25.963 54.810 1.00 32.78 H
ATOM 2438 HG23 ILE B 814 24.519 27.170 53.538 1.00 32.78 H
ATOM 2439 HD11 ILE B 814 26.891 30.236 56.026 1.00 31.44 H
ATOM 2440 HD12 ILE B 814 26.602 28.626 56.662 1.00 31.44 H
ATOM 2441 HD13 ILE B 814 27.159 28.832 55.000 1.00 31.44 H
ATOM 2442 N ARG B 815 21.500 26.319 56.140 1.00 31.53 N

ANISOU 2442 N   ARG B 815   3916   4904   3159   1180   389   802   N
ATOM   2443 CA ARG B 815   20.604 25.164 55.985 1.00 31.25   C
ANISOU 2443 CA ARG B 815   3657   5086   3132   1052   379   886   C
ATOM   2444 C   ARG B 815   19.163 25.554 55.615 1.00 35.82   C
ANISOU 2444 C   ARG B 815   4064   5965   3581   1176   441   1014   C
ATOM   2445 O   ARG B 815   18.532 24.796 54.881 1.00 35.26   O
ANISOU 2445 O   ARG B 815   3808   6072   3518   1026   414   1069   O
ATOM   2446 CB ARG B 815   20.575 24.290 57.250 1.00 29.84   C
ANISOU 2446 CB ARG B 815   3469   4916   2953   999   377   911   C
ATOM   2447 CG ARG B 815   21.878 23.516 57.525 1.00 35.28   C
ANISOU 2447 CG ARG B 815   4239   5390   3774   814   304   816   C
ATOM   2448 CD ARG B 815   21.706 22.428 58.602 1.00 46.68   C
ANISOU 2448 CD ARG B 815   5645   6872   5219   728   297   857   C
ATOM   2449 NE ARG B 815   21.397 22.991 59.930 1.00 54.36   N
ANISOU 2449 NE ARG B 815   6681   7874   6100   893   350   895   N
ATOM   2450 CZ ARG B 815   22.271 23.375 60.877 1.00 66.60   C
ANISOU 2450 CZ ARG B 815   8399   9240   7666   943   338   829   C
ATOM   2451 NH1 ARG B 815   23.805 23.907 62.013 1.00 53.42   N
ANISOU 2451 NH1 ARG B 815   6798   7603   5895   1096   390   868   N
ATOM   2452 NH2 ARG B 815   23.594 23.242 60.717 1.00 45.78   N1+
ANISOU 2452 NH2 ARG B 815   5861   6401   5133   841   274   730   N1+
ATOM   2453 H   ARG B 815   21.812 26.533 57.079 1.00 31.53   H
ATOM   2454 HA ARG B 815   20.985 24.554 55.164 1.00 31.25   H
ATOM   2455 HB3 ARG B 815   19.778 23.551 57.142 1.00 29.84   H
ATOM   2456 HB2 ARG B 815   20.303 24.900 58.113 1.00 29.84   H
ATOM   2457 HG3 ARG B 815   22.630 24.230 57.855 1.00 35.28   H
ATOM   2458 HG2 ARG B 815   22.286 23.070 56.617 1.00 35.28   H
ATOM   2459 HD3 ARG B 815   22.551 21.738 58.616 1.00 46.68   H
ATOM   2460 HD2 ARG B 815   20.840 21.818 58.345 1.00 46.68   H
ATOM   2461 HE ARG B 815   20.415 23.143 60.112 1.00 54.36   H
ATOM   2462 HH12 ARG B 815   22.453 24.197 62.735 1.00 53.42   H
ATOM   2463 HH11 ARG B 815   20.813 24.022 62.167 1.00 53.42   H
ATOM   2464 HH22 ARG B 815   24.219 23.545 61.452 1.00 45.78   H
ATOM   2465 HH21 ARG B 815   23.970 22.850 59.866 1.00 45.78   H
ATOM   2466 N   LYS B 816   18.672 26.713 56.093 1.00 33.06   N
ANISOU 2466 N   LYS B 816   3790   5678   3095   1454   525   1067   N
ATOM   2467 CA LYS B 816   17.365 27.257 55.713 1.00 32.82   C
ANISOU 2467 CA LYS B 816   3605   5954   2911   1640   599   1204   C
ATOM   2468 C   LYS B 816   17.317 27.665 54.228 1.00 35.34   C
ANISOU 2468 C   LYS B 816   3872   6306   3249   1621   581   1193   C
ATOM   2469 O   LYS B 816   16.290 27.458 53.585 1.00 34.81   O
ANISOU 2469 O   LYS B 816   3574   6538   3115   1600   592   1303   O
ATOM   2470 CB LYS B 816   16.983 28.419 56.656 1.00 35.19   C
ANISOU 2470 CB LYS B 816   4059   6267   3047   1983   705   1254   C
ATOM   2471 CG LYS B 816   15.574 28.995 56.409 1.00 43.49   C
ANISOU 2471 CG LYS B 816   4919   7695   3910   2217   798   1428   C
ATOM   2472 CD LYS B 816   15.173 30.055 57.443 1.00 48.24   C
ANISOU 2472 CD LYS B 816   5692   8309   4328   2581   915   1488   C
ATOM   2473 CE LYS B 816   13.724 30.534 57.255 1.00 62.74   C
ANISOU 2473 CE LYS B 816   7276 10591   5973   2795   1008   1689   C
ATOM   2474 NZ LYS B 816   13.369 31.598 58.211 1.00 69.65   N1+
ANISOU 2474 NZ LYS B 816   8341 11489   6635   3234   1142   1756   N1+
ATOM   2475 H   LYS B 816   19.233 27.280 56.716 1.00 33.06   H
ATOM   2476 HA LYS B 816   16.628 26.463 55.855 1.00 32.82   H
ATOM   2477 HB3 LYS B 816   17.720 29.219 56.582 1.00 35.19   H
ATOM   2478 HB2 LYS B 816   17.030 28.057 57.683 1.00 35.19   H

ATOM 2479 HG3 LYS B 816 14.847 28.182 56.418 1.00 43.49 H
ATOM 2480 HG2 LYS B 816 15.521 29.442 55.415 1.00 43.49 H
ATOM 2481 HD3 LYS B 816 15.859 30.900 57.366 1.00 48.24 H
ATOM 2482 HD2 LYS B 816 15.301 29.646 58.447 1.00 48.24 H
ATOM 2483 HE3 LYS B 816 13.033 29.701 57.384 1.00 62.74 H
ATOM 2484 HE2 LYS B 816 13.576 30.916 56.245 1.00 62.74 H
ATOM 2485 HZ1 LYS B 816 13.981 32.391 58.077 1.00 69.65 H
ATOM 2486 HZ2 LYS B 816 12.412 31.885 58.059 1.00 69.65 H
ATOM 2487 HZ3 LYS B 816 13.470 31.253 59.155 1.00 69.65 H
ATOM 2488 N TYR B 817 18.444 28.183 53.709 1.00 30.56 N
ANISOU 2488 N TYR B 817 3474 5412 2724 1613 551 1067 N
ATOM 2489 CA TYR B 817 18.622 28.547 52.305 1.00 30.25 C
ANISOU 2489 CA TYR B 817 3427 5351 2716 1590 530 1037 C
ATOM 2490 C TYR B 817 18.673 27.315 51.378 1.00 34.19 C
ANISOU 2490 C TYR B 817 3730 5935 3328 1295 449 1028 C
ATOM 2491 O TYR B 817 18.143 27.392 50.273 1.00 33.48 O
ANISOU 2491 O TYR B 817 3498 6019 3203 1277 446 1081 O
ATOM 2492 CB TYR B 817 19.866 29.462 52.184 1.00 31.23 C
ANISOU 2492 CB TYR B 817 3832 5132 2904 1602 507 901 C
ATOM 2493 CG TYR B 817 20.071 30.282 50.913 1.00 32.75 C
ANISOU 2493 CG TYR B 817 4102 5263 3079 1670 514 873 C
ATOM 2494 CD1 TYR B 817 19.044 30.476 49.963 1.00 34.50 C
ANISOU 2494 CD1 TYR B 817 4143 5729 3235 1737 541 964 C
ATOM 2495 CD2 TYR B 817 21.315 30.918 50.713 1.00 33.24 C
ANISOU 2495 CD2 TYR B 817 4411 5036 3183 1646 488 761 C
ATOM 2496 CE1 TYR B 817 19.257 31.288 48.835 1.00 33.42 C
ANISOU 2496 CE1 TYR B 817 4083 5533 3082 1802 548 940 C
ATOM 2497 CE2 TYR B 817 21.529 31.732 49.583 1.00 33.34 C
ANISOU 2497 CE2 TYR B 817 4507 4986 3176 1696 495 737 C
ATOM 2498 CZ TYR B 817 20.501 31.911 48.639 1.00 38.46 C
ANISOU 2498 CZ TYR B 817 4984 5861 3766 1784 527 824 C
ATOM 2499 OH TYR B 817 20.706 32.684 47.535 1.00 40.58 O
ANISOU 2499 OH TYR B 817 5345 6060 4015 1835 533 799 O
ATOM 2500 H TYR B 817 19.244 28.326 54.310 1.00 30.56 H
ATOM 2501 HA TYR B 817 17.738 29.116 52.022 1.00 30.25 H
ATOM 2502 HB3 TYR B 817 20.769 28.878 52.367 1.00 31.23 H
ATOM 2503 HB2 TYR B 817 19.830 30.198 52.988 1.00 31.23 H
ATOM 2504 HD1 TYR B 817 18.078 30.018 50.083 1.00 34.50 H
ATOM 2505 HD2 TYR B 817 22.109 30.793 51.435 1.00 33.24 H
ATOM 2506 HE1 TYR B 817 18.460 31.438 48.123 1.00 33.42 H
ATOM 2507 HE2 TYR B 817 22.486 32.215 49.446 1.00 33.34 H
ATOM 2508 HH TYR B 817 21.515 33.203 47.591 1.00 40.58 H
ATOM 2509 N VAL B 818 19.235 26.187 51.851 1.00 31.40 N
ANISOU 2509 N VAL B 818 3378 5466 3086 1075 387 970 N
ATOM 2510 CA VAL B 818 19.199 24.902 51.140 1.00 30.98 C
ANISOU 2510 CA VAL B 818 3201 5452 3118 795 315 957 C
ATOM 2511 C VAL B 818 17.781 24.285 51.140 1.00 37.77 C
ANISOU 2511 C VAL B 818 3823 6660 3868 727 329 1103 C
ATOM 2512 O VAL B 818 17.322 23.821 50.097 1.00 36.40 O
ANISOU 2512 O VAL B 818 3516 6636 3678 588 296 1141 O
ATOM 2513 CB VAL B 818 20.203 23.867 51.740 1.00 33.18 C
ANISOU 2513 CB VAL B 818 3586 5499 3521 617 258 864 C
ATOM 2514 CG1 VAL B 818 20.088 22.434 51.168 1.00 32.41 C
ANISOU 2514 CG1 VAL B 818 3415 5416 3483 351 195 853 C
ATOM 2515 CG2 VAL B 818 21.655 24.343 51.581 1.00 32.62 C
ANISOU 2515 CG2 VAL B 818 3714 5140 3539 666 239 740 C

```
ATOM   2516  H   VAL B 818      19.667  26.186  52.765  1.00 31.40           H
ATOM   2517  HA  VAL B 818      19.489  25.094  50.107  1.00 30.98           H
ATOM   2518  HB  VAL B 818      20.014  23.794  52.811  1.00 33.18           H
ATOM   2519 HG11 VAL B 818      20.884  21.793  51.547  1.00 32.41           H
ATOM   2520 HG12 VAL B 818      19.149  21.952  51.440  1.00 32.41           H
ATOM   2521 HG13 VAL B 818      20.162  22.436  50.079  1.00 32.41           H
ATOM   2522 HG21 VAL B 818      22.356  23.645  52.034  1.00 32.62           H
ATOM   2523 HG22 VAL B 818      21.928  24.445  50.533  1.00 32.62           H
ATOM   2524 HG23 VAL B 818      21.812  25.306  52.057  1.00 32.62           H
ATOM   2525  N   LYS B 819      17.120  24.293  52.309  1.00 37.35           N
ANISOU 2525  N   LYS B 819     3716   6747   3729    813    374   1188       N
ATOM   2526  CA  LYS B 819      15.831  23.644  52.549  1.00 38.36           C
ANISOU 2526  CA  LYS B 819     3606   7238   3731    745    390   1344       C
ATOM   2527  C   LYS B 819      14.650  24.283  51.791  1.00 44.33           C
ANISOU 2527  C   LYS B 819     4167   8331   4344    874    433   1473       C
ATOM   2528  O   LYS B 819      13.803  23.547  51.287  1.00 44.59           O
ANISOU 2528  O   LYS B 819     3985   8653   4305    688    402   1577       O
ATOM   2529  CB  LYS B 819      15.595  23.587  54.075  1.00 41.22           C
ANISOU 2529  CB  LYS B 819     3973   7665   4024    866    443   1406       C
ATOM   2530  CG  LYS B 819      14.306  22.871  54.515  1.00 58.38           C
ANISOU 2530  CG  LYS B 819     5904  10201   6075    741    449   1565       C
ATOM   2531  CD  LYS B 819      14.222  22.710  56.044  1.00 70.33           C
ANISOU 2531  CD  LYS B 819     7458  11702   7563    787    481   1593       C
ATOM   2532  CE  LYS B 819      12.981  21.939  56.531  1.00 82.66           C
ANISOU 2532  CE  LYS B 819     8772  13644   8990    651    488   1762       C
ATOM   2533  NZ  LYS B 819      11.726  22.678  56.294  1.00 92.12           N1+
ANISOU 2533  NZ  LYS B 819     9766  15240   9994    913    578   1931       N1+
ATOM   2534  H   LYS B 819      17.568  24.693  53.123  1.00 37.35           H
ATOM   2535  HA  LYS B 819      15.922  22.616  52.191  1.00 38.36           H
ATOM   2536  HB3 LYS B 819      15.600  24.598  54.484  1.00 41.22           H
ATOM   2537  HB2 LYS B 819      16.442  23.072  54.530  1.00 41.22           H
ATOM   2538  HG3 LYS B 819      14.259  21.890  54.041  1.00 58.38           H
ATOM   2539  HG2 LYS B 819      13.439  23.428  54.159  1.00 58.38           H
ATOM   2540  HD3 LYS B 819      14.260  23.693  56.516  1.00 70.33           H
ATOM   2541  HD2 LYS B 819      15.112  22.184  56.392  1.00 70.33           H
ATOM   2542  HE3 LYS B 819      13.065  21.751  57.602  1.00 82.66           H
ATOM   2543  HE2 LYS B 819      12.923  20.966  56.042  1.00 82.66           H
ATOM   2544  HZ1 LYS B 819      11.611  22.837  55.304  1.00 92.12           H
ATOM   2545  HZ2 LYS B 819      10.945  22.138  56.640  1.00 92.12           H
ATOM   2546  HZ3 LYS B 819      11.760  23.566  56.774  1.00 92.12           H
ATOM   2547  N   ASN B 820      14.608  25.625  51.739  1.00 41.18           N
ANISOU 2547  N   ASN B 820     3858   7900   3890   1183    502   1471       N
ATOM   2548  CA  ASN B 820      13.483  26.390  51.188  1.00 41.17           C
ANISOU 2548  CA  ASN B 820     3691   8223   3729   1385    563   1607       C
ATOM   2549  C   ASN B 820      13.557  26.590  49.665  1.00 43.45           C
ANISOU 2549  C   ASN B 820     3949   8513   4045   1331    528   1578       C
ATOM   2550  O   ASN B 820      12.509  26.842  49.070  1.00 42.78           O
ANISOU 2550  O   ASN B 820     3686   8751   3818   1468    571   1710       O
ATOM   2551  CB  ASN B 820      13.410  27.766  51.890  1.00 42.35           C
ANISOU 2551  CB  ASN B 820     3984   8347   3761   1793    675   1640       C
ATOM   2552  CG  ASN B 820      12.974  27.726  53.361  1.00 53.45           C
ANISOU 2552  CG  ASN B 820     5317   9957   5033   1957    750   1758       C
ATOM   2553  ND2 ASN B 820      12.868  28.905  53.975  1.00 43.28           N
ANISOU 2553  ND2 ASN B 820     4142   8703   3599   2342    860   1814       N
ATOM   2554  OD1 ASN B 820      12.728  26.669  53.940  1.00 47.74           O
ANISOU 2554  OD1 ASN B 820     4459   9354   4325   1753    714   1801       O
```

ATOM	2555	H	ASN	B	820	15.339	26.164	52.185	1.00	41.18	H
ATOM	2556	HA	ASN	B	820	12.568	25.839	51.416	1.00	41.17	H
ATOM	2557	HB3	ASN	B	820	12.688	28.402	51.375	1.00	42.35	H
ATOM	2558	HB2	ASN	B	820	14.371	28.277	51.809	1.00	42.35	H
ATOM	2559	HD22	ASN	B	820	12.565	28.951	54.935	1.00	43.28	H
ATOM	2560	HD21	ASN	B	820	13.099	29.755	53.476	1.00	43.28	H
ATOM	2561	N	THR	B	821	14.758	26.509	49.066	1.00	38.77	N
ANISOU	2561	N	THR	B	821	3529	7581	3620	1172	461	1417 N
ATOM	2562	CA	THR	B	821	14.992	26.874	47.663	1.00	37.72	C
ANISOU	2562	CA	THR	B	821	3384	7442	3507	1149	436	1388 C
ATOM	2563	C	THR	B	821	15.365	25.671	46.764	1.00	42.35	C
ANISOU	2563	C	THR	B	821	3903	7993	4195	785	334	1333 C
ATOM	2564	O	THR	B	821	15.889	25.877	45.668	1.00	40.30	O
ANISOU	2564	O	THR	B	821	3728	7570	4016	730	299	1243 O
ATOM	2565	CB	THR	B	821	16.032	28.026	47.534	1.00	36.81	C
ANISOU	2565	CB	THR	B	821	3537	6999	3449	1342	465	1269 C
ATOM	2566	CG2	THR	B	821	15.798	29.195	48.505	1.00	32.99	C
ANISOU	2566	CG2	THR	B	821	3167	6544	2823	1718	573	1331 C
ATOM	2567	OG1	THR	B	821	17.381	27.599	47.602	1.00	33.26	O
ANISOU	2567	OG1	THR	B	821	3277	6197	3165	1183	408	1116 O
ATOM	2568	H	THR	B	821	15.577	26.278	49.611	1.00	38.77	H
ATOM	2569	HA	THR	B	821	14.069	27.257	47.224	1.00	37.72	H
ATOM	2570	HB	THR	B	821	15.902	28.454	46.543	1.00	36.81	H
ATOM	2571	HG21	THR	B	821	16.468	30.025	48.283	1.00	32.99	H
ATOM	2572	HG22	THR	B	821	14.779	29.572	48.418	1.00	32.99	H
ATOM	2573	HG23	THR	B	821	15.957	28.906	49.544	1.00	32.99	H
ATOM	2574	HG1	THR	B	821	17.609	27.451	48.526	1.00	33.26	H
ATOM	2575	N	HIE	B	822	15.032	24.441	47.200	1.00	41.21	N
ANISOU	2575	N	HIE	B	822	3620	8010	4026	538	290	1394 N
ATOM	2576	CA	HIE	B	822	15.059	23.248	46.350	1.00	41.72	C
ANISOU	2576	CA	HIE	B	822	3652	8054	4144	189	200	1357 C
ATOM	2577	C	HIE	B	822	13.747	23.171	45.547	1.00	48.03	C
ANISOU	2577	C	HIE	B	822	4190	9273	4785	121	192	1510 C
ATOM	2578	O	HIE	B	822	12.674	23.081	46.145	1.00	47.74	O
ANISOU	2578	O	HIE	B	822	3954	9579	4607	124	216	1663 O
ATOM	2579	CB	HIE	B	822	15.291	21.986	47.209	1.00	42.23	C
ANISOU	2579	CB	HIE	B	822	3761	8035	4250	-67	153	1337 C
ATOM	2580	CG	HIE	B	822	15.718	20.767	46.420	1.00	45.44	C
ANISOU	2580	CG	HIE	B	822	4251	8293	4722	-392	69	1259 C
ATOM	2581	CD2	HIE	B	822	14.961	19.964	45.589	1.00	46.88	C
ANISOU	2581	CD2	HIE	B	822	4340	8634	4838	-618	15	1301 C
ATOM	2582	ND1	HIE	B	822	17.031	20.282	46.431	1.00	47.28	N
ANISOU	2582	ND1	HIE	B	822	4705	8169	5089	-485	38	1125 N
ATOM	2583	CE1	HIE	B	822	17.019	19.237	45.613	1.00	46.41	C
ANISOU	2583	CE1	HIE	B	822	4650	7998	4987	-744	-24	1087 C
ATOM	2584	NE2	HIE	B	822	15.820	19.007	45.079	1.00	46.49	N
ANISOU	2584	NE2	HIE	B	822	4484	8302	4879	-847	-45	1185 N
ATOM	2585	H	HIE	B	822	14.602	24.333	48.107	1.00	41.21	H
ATOM	2586	HA	HIE	B	822	15.904	23.342	45.668	1.00	41.72	H
ATOM	2587	HB3	HIE	B	822	14.398	21.737	47.784	1.00	42.23	H
ATOM	2588	HB2	HIE	B	822	16.072	22.189	47.945	1.00	42.23	H
ATOM	2589	HD2	HIE	B	822	13.916	20.005	45.319	1.00	46.88	H
ATOM	2590	HE1	HIE	B	822	17.888	18.635	45.397	1.00	46.41	H
ATOM	2591	N	ALA	B	823	13.862	23.223	44.211	1.00	46.48	N
ANISOU	2591	N	ALA	B	823	3983	9077	4601	70	160	1480 N
ATOM	2592	CA	ALA	B	823	12.739	23.213	43.274	1.00	46.77	C

```
ANISOU 2592  CA  ALA B 823     3770   9521   4482      1    145   1625       C
ATOM   2593  C   ALA B 823    12.105  21.823  43.110  1.00 52.13           C
ANISOU 2593  C   ALA B 823     4322  10401   5085   -407     61   1695       C
ATOM   2594  O   ALA B 823    12.822  20.822  43.069  1.00 51.77           O
ANISOU 2594  O   ALA B 823     4452  10078   5141   -670     -2   1581       O
ATOM   2595  CB  ALA B 823    13.237  23.708  41.911  1.00 47.55           C
ANISOU 2595  CB  ALA B 823     3924   9517   4624     46    129   1556       C
ATOM   2596  H   ALA B 823    14.783  23.286  43.794  1.00 46.48           H
ATOM   2597  HA  ALA B 823    11.982  23.911  43.637  1.00 46.77           H
ATOM   2598  HB1 ALA B 823    12.431  23.707  41.182  1.00 47.55           H
ATOM   2599  HB2 ALA B 823    13.635  24.721  41.969  1.00 47.55           H
ATOM   2600  HB3 ALA B 823    14.021  23.061  41.522  1.00 47.55           H
ATOM   2601  N   THR B 824    10.771  21.808  42.944  1.00 50.57           N
ANISOU 2601  N   THR B 824     3828  10697   4689   -460     62   1892       N
ATOM   2602  CA  THR B 824     9.960  20.613  42.683  1.00 50.24           C
ANISOU 2602  CA  THR B 824     3640  10924   4524   -880    -23   1992       C
ATOM   2603  C   THR B 824    10.241  19.960  41.309  1.00 53.41           C
ANISOU 2603  C   THR B 824     4147  11184   4962  -1185   -115   1902       C
ATOM   2604  O   THR B 824    10.073  18.749  41.180  1.00 52.12           O
ANISOU 2604  O   THR B 824     4073  10951   4781  -1569   -195   1877       O
ATOM   2605  CB  THR B 824     8.445  20.953  42.763  1.00 59.81           C
ANISOU 2605  CB  THR B 824     4482  12746   5496   -834      5   2241       C
ATOM   2606  CG2 THR B 824     7.476  19.772  42.564  1.00 58.95           C
ANISOU 2606  CG2 THR B 824     4203  12969   5227  -1310    -95   2362       C
ATOM   2607  OG1 THR B 824     8.168  21.517  44.031  1.00 59.28           O
ANISOU 2607  OG1 THR B 824     4359  12769   5395   -605     85   2313       O
ATOM   2608  H   THR B 824    10.256  22.675  43.005  1.00 50.57           H
ATOM   2609  HA  THR B 824    10.197  19.876  43.453  1.00 50.24           H
ATOM   2610  HB  THR B 824     8.210  21.718  42.021  1.00 59.81           H
ATOM   2611 HG21 THR B 824     6.448  20.072  42.767  1.00 58.95           H
ATOM   2612 HG22 THR B 824     7.495  19.395  41.541  1.00 58.95           H
ATOM   2613 HG23 THR B 824     7.717  18.943  43.231  1.00 58.95           H
ATOM   2614  HG1 THR B 824     8.242  20.830  44.697  1.00 59.28           H
ATOM   2615  N   THR B 825    10.705  20.753  40.328  1.00 49.98           N
ANISOU 2615  N   THR B 825     3741  10673   4577  -1009   -101   1845       N
ATOM   2616  CA  THR B 825    11.136  20.282  39.007  1.00 49.97           C
ANISOU 2616  CA  THR B 825     3858  10512   4617  -1247   -177   1748       C
ATOM   2617  C   THR B 825    12.513  19.575  39.024  1.00 53.53           C
ANISOU 2617  C   THR B 825     4653  10428   5257  -1363   -206   1540       C
ATOM   2618  O   THR B 825    12.838  18.903  38.046  1.00 53.77           O
ANISOU 2618  O   THR B 825     4816  10306   5307  -1599   -270   1459       O
ATOM   2619  CB  THR B 825    11.210  21.459  37.994  1.00 58.05           C
ANISOU 2619  CB  THR B 825     4807  11619   5632  -1005   -146   1756       C
ATOM   2620  CG2 THR B 825     9.887  22.228  37.855  1.00 59.26           C
ANISOU 2620  CG2 THR B 825     4614  12330   5572   -884   -117   1977       C
ATOM   2621  OG1 THR B 825    12.252  22.372  38.300  1.00 56.61           O
ANISOU 2621  OG1 THR B 825     4797  11113   5599   -649    -70   1640       O
ATOM   2622  H   THR B 825    10.827  21.740  40.503  1.00 49.98           H
ATOM   2623  HA  THR B 825    10.401  19.561  38.642  1.00 49.97           H
ATOM   2624  HB  THR B 825    11.444  21.049  37.010  1.00 58.05           H
ATOM   2625 HG21 THR B 825     9.970  23.016  37.106  1.00 59.26           H
ATOM   2626 HG22 THR B 825     9.082  21.562  37.542  1.00 59.26           H
ATOM   2627 HG23 THR B 825     9.582  22.694  38.792  1.00 59.26           H
ATOM   2628  HG1 THR B 825    13.093  21.911  38.230  1.00 56.61           H
ATOM   2629  N   HIE B 826    13.283  19.725  40.118  1.00 49.19           N
ANISOU 2629  N   HIE B 826     4247   9610   4831  -1185   -155   1460       N
```

```
ATOM   2630  CA  HIE B 826      14.586  19.087  40.335  1.00 48.63           C
ANISOU 2630  CA  HIE B 826     4477   9074   4926  -1248   -172   1284       C
ATOM   2631  C   HIE B 826      14.479  17.977  41.401  1.00 54.23           C
ANISOU 2631  C   HIE B 826     5260   9734   5610  -1468   -197   1301       C
ATOM   2632  O   HIE B 826      15.380  17.823  42.226  1.00 54.64           O
ANISOU 2632  O   HIE B 826     5482   9500   5778  -1375   -171   1211       O
ATOM   2633  CB  HIE B 826      15.643  20.166  40.674  1.00 48.78           C
ANISOU 2633  CB  HIE B 826     4617   8821   5097   -895   -103   1179       C
ATOM   2634  CG  HIE B 826      15.802  21.266  39.647  1.00 51.59           C
ANISOU 2634  CG  HIE B 826     4950   9174   5477   -695    -78   1152       C
ATOM   2635  CD2 HIE B 826      15.672  21.200  38.273  1.00 52.91           C
ANISOU 2635  CD2 HIE B 826     5094   9392   5617   -798   -117   1141       C
ATOM   2636  ND1 HIE B 826      16.127  22.582  39.996  1.00 52.85           N
ANISOU 2636  ND1 HIE B 826     5131   9270   5680   -356     -6   1137       N
ATOM   2637  CE1 HIE B 826      16.149  23.252  38.852  1.00 52.02           C
ANISOU 2637  CE1 HIE B 826     5019   9172   5576   -265     -2   1118       C
ATOM   2638  NE2 HIE B 826      15.886  22.482  37.797  1.00 52.41           N
ANISOU 2638  NE2 HIE B 826     5027   9301   5587   -516    -67   1122       N
ATOM   2639  H   HIE B 826      12.938  20.280  40.889  1.00 49.19           H
ATOM   2640  HA  HIE B 826      14.915  18.589  39.421  1.00 48.63           H
ATOM   2641  HB3 HIE B 826      16.622  19.705  40.811  1.00 48.78           H
ATOM   2642  HB2 HIE B 826      15.387  20.632  41.628  1.00 48.78           H
ATOM   2643  HD2 HIE B 826      15.437  20.373  37.619  1.00 52.91           H
ATOM   2644  HE1 HIE B 826      16.361  24.308  38.784  1.00 52.02           H
ATOM   2645  N   ASN B 827      13.379  17.203  41.345  1.00 52.14           N
ANISOU 2645  N   ASN B 827     4873   9757   5180  -1780   -252   1422       N
ATOM   2646  CA  ASN B 827      13.053  16.093  42.249  1.00 52.48           C
ANISOU 2646  CA  ASN B 827     4977   9798   5166  -2020   -278   1463       C
ATOM   2647  C   ASN B 827      13.735  14.751  41.893  1.00 55.21           C
ANISOU 2647  C   ASN B 827     5644   9792   5542  -2326   -340   1345       C
ATOM   2648  O   ASN B 827      13.255  13.707  42.335  1.00 55.35           O
ANISOU 2648  O   ASN B 827     5737   9824   5472  -2593   -374   1391       O
ATOM   2649  CB  ASN B 827      11.512  15.963  42.401  1.00 56.04           C
ANISOU 2649  CB  ASN B 827     5134  10760   5400  -2213   -303   1671       C
ATOM   2650  CG  ASN B 827      10.746  15.497  41.150  1.00 88.61           C
ANISOU 2650  CG  ASN B 827     9185  15119   9364  -2572   -389   1743       C
ATOM   2651  ND2 ASN B 827       9.608  14.830  41.354  1.00 87.57           N
ANISOU 2651  ND2 ASN B 827     8907  15326   9041  -2889   -438   1897       N
ATOM   2652  OD1 ASN B 827      11.158  15.743  40.018  1.00 82.47           O
ANISOU 2652  OD1 ASN B 827     8474  14241   8618  -2583   -415   1670       O
ATOM   2653  H   ASN B 827      12.694  17.384  40.624  1.00 52.14           H
ATOM   2654  HA  ASN B 827      13.438  16.342  43.239  1.00 52.48           H
ATOM   2655  HB3 ASN B 827      11.099  16.920  42.718  1.00 56.04           H
ATOM   2656  HB2 ASN B 827      11.292  15.272  43.216  1.00 56.04           H
ATOM   2657 HD22 ASN B 827       9.067  14.510  40.564  1.00 87.57           H
ATOM   2658 HD21 ASN B 827       9.278  14.646  42.289  1.00 87.57           H
ATOM   2659  N   ALA B 828      14.846  14.786  41.137  1.00 50.06           N
ANISOU 2659  N   ALA B 828     5202   8811   5007  -2263   -344   1196       N
ATOM   2660  CA  ALA B 828      15.690  13.623  40.845  1.00 48.97           C
ANISOU 2660  CA  ALA B 828     5405   8304   4898  -2469   -381   1077       C
ATOM   2661  C   ALA B 828      16.548  13.168  42.044  1.00 50.58           C
ANISOU 2661  C   ALA B 828     5797   8218   5203  -2362   -343   1009       C
ATOM   2662  O   ALA B 828      16.976  12.014  42.066  1.00 50.99           O
ANISOU 2662  O   ALA B 828     6112   8041   5219  -2569   -369    964       O
ATOM   2663  CB  ALA B 828      16.584  13.958  39.641  1.00 49.64           C
ANISOU 2663  CB  ALA B 828     5633   8147   5080  -2362   -379    949       C
```

ATOM 2664 H ALA B 828 15.181 15.676 40.798 1.00 50.06 H
ATOM 2665 HA ALA B 828 15.046 12.787 40.566 1.00 48.97 H
ATOM 2666 HB1 ALA B 828 17.240 13.124 39.389 1.00 49.64 H
ATOM 2667 HB2 ALA B 828 15.983 14.178 38.759 1.00 49.64 H
ATOM 2668 HB3 ALA B 828 17.212 14.827 39.839 1.00 49.64 H
ATOM 2669 N TYR B 829 16.775 14.074 43.010 1.00 43.50 N
ANISOU 2669 N TYR B 829 4788 7322 4418 -2039 -280 1003 N
ATOM 2670 CA TYR B 829 17.597 13.855 44.198 1.00 41.92 C
ANISOU 2670 CA TYR B 829 4731 6883 4314 -1909 -244 946 C
ATOM 2671 C TYR B 829 17.146 14.782 45.340 1.00 46.14 C
ANISOU 2671 C TYR B 829 5058 7610 4862 -1688 -192 1025 C
ATOM 2672 O TYR B 829 16.558 15.835 45.086 1.00 45.89 O
ANISOU 2672 O TYR B 829 4800 7840 4797 -1542 -168 1097 O
ATOM 2673 CB TYR B 829 19.093 14.065 43.847 1.00 41.43 C
ANISOU 2673 CB TYR B 829 4867 6462 4412 -1701 -218 795 C
ATOM 2674 CG TYR B 829 19.460 15.450 43.326 1.00 41.04 C
ANISOU 2674 CG TYR B 829 4683 6460 4452 -1431 -184 763 C
ATOM 2675 CD1 TYR B 829 19.777 16.491 44.224 1.00 42.56 C
ANISOU 2675 CD1 TYR B 829 4777 6680 4714 -1162 -134 770 C
ATOM 2676 CD2 TYR B 829 19.463 15.706 41.939 1.00 41.37 C
ANISOU 2676 CD2 TYR B 829 4720 6505 4495 -1451 -203 727 C
ATOM 2677 CE1 TYR B 829 20.069 17.780 43.740 1.00 42.97 C
ANISOU 2677 CE1 TYR B 829 4743 6762 4821 -932 -104 747 C
ATOM 2678 CE2 TYR B 829 19.755 16.997 41.454 1.00 41.98 C
ANISOU 2678 CE2 TYR B 829 4692 6619 4641 -1213 -172 703 C
ATOM 2679 CZ TYR B 829 20.052 18.036 42.357 1.00 49.12 C
ANISOU 2679 CZ TYR B 829 5517 7544 5603 -958 -122 713 C
ATOM 2680 OH TYR B 829 20.323 19.293 41.902 1.00 48.36 O
ANISOU 2680 OH TYR B 829 5362 7459 5554 -737 -91 691 O
ATOM 2681 H TYR B 829 16.384 15.002 42.922 1.00 43.50 H
ATOM 2682 HA TYR B 829 17.445 12.828 44.530 1.00 41.92 H
ATOM 2683 HB3 TYR B 829 19.398 13.329 43.104 1.00 41.43 H
ATOM 2684 HB2 TYR B 829 19.708 13.855 44.724 1.00 41.43 H
ATOM 2685 HD1 TYR B 829 19.784 16.308 45.288 1.00 42.56 H
ATOM 2686 HD2 TYR B 829 19.229 14.915 41.242 1.00 41.37 H
ATOM 2687 HE1 TYR B 829 20.304 18.578 44.427 1.00 42.97 H
ATOM 2688 HE2 TYR B 829 19.745 17.185 40.391 1.00 41.98 H
ATOM 2689 HH TYR B 829 20.234 19.384 40.947 1.00 48.36 H
ATOM 2690 N ASP B 830 17.480 14.372 46.574 1.00 42.63 N
ANISOU 2690 N ASP B 830 4716 7020 4460 -1641 -170 1008 N
ATOM 2691 CA ASP B 830 17.426 15.199 47.786 1.00 42.05 C
ANISOU 2691 CA ASP B 830 4518 7046 4412 -1414 -117 1056 C
ATOM 2692 C ASP B 830 18.847 15.686 48.113 1.00 41.17 C
ANISOU 2692 C ASP B 830 4557 6623 4463 -1155 -83 926 C
ATOM 2693 O ASP B 830 19.814 14.997 47.783 1.00 38.79 O
ANISOU 2693 O ASP B 830 4459 6045 4232 -1201 -102 824 O
ATOM 2694 CB ASP B 830 16.837 14.476 49.030 1.00 45.30 C
ANISOU 2694 CB ASP B 830 4960 7500 4750 -1575 -122 1126 C
ATOM 2695 CG ASP B 830 15.647 13.548 48.751 1.00 67.38 C
ANISOU 2695 CG ASP B 830 7679 10549 7371 -1925 -172 1247 C
ATOM 2696 OD1 ASP B 830 14.530 14.086 48.573 1.00 70.53 O
ANISOU 2696 OD1 ASP B 830 7810 11331 7659 -1938 -168 1377 O
ATOM 2697 OD2 ASP B 830 15.890 12.334 48.577 1.00 76.90 O1-
ANISOU 2697 OD2 ASP B 830 9103 11581 8536 -2187 -213 1221 O1-
ATOM 2698 H ASP B 830 17.921 13.469 46.689 1.00 42.63 H
ATOM 2699 HA ASP B 830 16.807 16.078 47.594 1.00 42.05 H

```
ATOM   2700  HB3 ASP B 830      16.500  15.229  49.745  1.00 45.30           H
ATOM   2701  HB2 ASP B 830      17.605  13.902  49.550  1.00 45.30           H
ATOM   2702  N   LEU B 831      18.947  16.844  48.783  1.00 36.52           N
ANISOU 2702  N   LEU B 831     3877   6086   3913   -891    -33    938       N
ATOM   2703  CA  LEU B 831      20.214  17.406  49.253  1.00 35.33           C
ANISOU 2703  CA  LEU B 831     3854   5681   3891   -672     -8    831       C
ATOM   2704  C   LEU B 831      20.294  17.328  50.783  1.00 39.66           C
ANISOU 2704  C   LEU B 831     4430   6188   4449   -570     22    848       C
ATOM   2705  O   LEU B 831      19.315  17.620  51.470  1.00 38.86           O
ANISOU 2705  O   LEU B 831     4194   6304   4268   -507     53    944       O
ATOM   2706  CB  LEU B 831      20.354  18.871  48.778  1.00 35.08           C
ANISOU 2706  CB  LEU B 831     3754   5684   3889   -451     21    808       C
ATOM   2707  CG  LEU B 831      20.631  19.017  47.265  1.00 39.16           C
ANISOU 2707  CG  LEU B 831     4286   6159   4434   -497     -4    755       C
ATOM   2708  CD1 LEU B 831      20.593  20.497  46.827  1.00 39.21           C
ANISOU 2708  CD1 LEU B 831     4207   6262   4430   -285     32    768       C
ATOM   2709  CD2 LEU B 831      21.942  18.323  46.838  1.00 39.54           C
ANISOU 2709  CD2 LEU B 831     4522   5908   4592   -517    -26    633       C
ATOM   2710  H   LEU B 831      18.113  17.352  49.041  1.00 36.52           H
ATOM   2711  HA  LEU B 831      21.053  16.832  48.859  1.00 35.33           H
ATOM   2712  HB3 LEU B 831      21.168  19.357  49.319  1.00 35.08           H
ATOM   2713  HB2 LEU B 831      19.450  19.422  49.044  1.00 35.08           H
ATOM   2714  HG  LEU B 831      19.815  18.518  46.744  1.00 39.16           H
ATOM   2715 HD11 LEU B 831      19.939  20.632  45.964  1.00 39.21           H
ATOM   2716 HD12 LEU B 831      20.218  21.149  47.617  1.00 39.21           H
ATOM   2717 HD13 LEU B 831      21.577  20.876  46.548  1.00 39.21           H
ATOM   2718 HD21 LEU B 831      22.486  18.889  46.082  1.00 39.54           H
ATOM   2719 HD22 LEU B 831      22.619  18.173  47.679  1.00 39.54           H
ATOM   2720 HD23 LEU B 831      21.739  17.341  46.411  1.00 39.54           H
ATOM   2721  N   GLU B 832      21.494  16.978  51.265  1.00 36.99           N
ANISOU 2721  N   GLU B 832     4264   5588   4204   -533     14    759       N
ATOM   2722  CA  GLU B 832      21.882  16.956  52.670  1.00 36.90           C
ANISOU 2722  CA  GLU B 832     4303   5506   4212   -438     36    760       C
ATOM   2723  C   GLU B 832      23.235  17.665  52.788  1.00 39.00           C
ANISOU 2723  C   GLU B 832     4663   5578   4576   -254     43    665       C
ATOM   2724  O   GLU B 832      24.178  17.263  52.109  1.00 39.34           O
ANISOU 2724  O   GLU B 832     4809   5452   4688   -276     20    588       O
ATOM   2725  CB  GLU B 832      21.943  15.489  53.153  1.00 38.83           C
ANISOU 2725  CB  GLU B 832     4670   5644   4439   -616     13    768       C
ATOM   2726  CG  GLU B 832      22.547  15.278  54.566  1.00 55.93           C
ANISOU 2726  CG  GLU B 832     6865   7784   6602   -543     35    793       C
ATOM   2727  CD  GLU B 832      22.719  13.813  55.003  1.00 87.63           C
ANISOU 2727  CD  GLU B 832    11063  11602  10633   -635     19    766       C
ATOM   2728  OE1 GLU B 832      23.093  13.631  56.183  1.00 86.30           O
ANISOU 2728  OE1 GLU B 832    10924  11392  10473   -550     35    776       O
ATOM   2729  OE2 GLU B 832      22.501  12.898  54.176  1.00 85.72           O1-
ANISOU 2729  OE2 GLU B 832    10950  11238  10382   -779     -6    736       O1-
ATOM   2730  H   GLU B 832      22.229  16.733  50.615  1.00 36.99           H
ATOM   2731  HA  GLU B 832      21.153  17.498  53.276  1.00 36.90           H
ATOM   2732  HB3 GLU B 832      22.500  14.907  52.420  1.00 38.83           H
ATOM   2733  HB2 GLU B 832      20.930  15.082  53.142  1.00 38.83           H
ATOM   2734  HG3 GLU B 832      21.927  15.794  55.301  1.00 55.93           H
ATOM   2735  HG2 GLU B 832      23.537  15.731  54.629  1.00 55.93           H
ATOM   2736  N   VAL B 833      23.318  18.680  53.662  1.00 33.48           N
ANISOU 2736  N   VAL B 833     3939   4913   3869    -79     75    676       N
ATOM   2737  CA  VAL B 833      24.561  19.392  53.959  1.00 32.72           C
```

ANISOU 2737 CA VAL B 833   3939   4652   3840   59   73   596   C
ATOM   2738 C   VAL B 833   25.410 18.549 54.931 1.00 34.72   C
ANISOU 2738 C   VAL B 833   4297   4757   4136   34   55   565   C
ATOM   2739 O   VAL B 833   24.992 18.338 56.071 1.00 34.59   O
ANISOU 2739 O   VAL B 833   4278   4782   4081   39   69   612   O
ATOM   2740 CB VAL B 833   24.283 20.783 54.602 1.00 36.77   C
ANISOU 2740 CB VAL B 833   4438   5230   4304   244   112   614   C
ATOM   2741 CG1 VAL B 833   25.562 21.554 54.988 1.00 36.48   C
ANISOU 2741 CG1 VAL B 833   4525   5020   4316   330   97   533   C
ATOM   2742 CG2 VAL B 833   23.422 21.669 53.684 1.00 36.53   C
ANISOU 2742 CG2 VAL B 833   4316   5344   4218   303   137   651   C
ATOM   2743 H   VAL B 833   22.507 18.951 54.198 1.00 33.48   H
ATOM   2744 HA VAL B 833   25.120 19.547 53.034 1.00 32.72   H
ATOM   2745 HB VAL B 833   23.708 20.634 55.517 1.00 36.77   H
ATOM   2746 HG11 VAL B 833   25.311 22.547 55.360 1.00 36.48   H
ATOM   2747 HG12 VAL B 833   26.128 21.055 55.775 1.00 36.48   H
ATOM   2748 HG13 VAL B 833   26.224 21.681 54.130 1.00 36.48   H
ATOM   2749 HG21 VAL B 833   23.225 22.638 54.145 1.00 36.53   H
ATOM   2750 HG22 VAL B 833   23.921 21.852 52.733 1.00 36.53   H
ATOM   2751 HG23 VAL B 833   22.454 21.215 53.472 1.00 36.53   H
ATOM   2752 N   ILE B 834   26.573 18.076 54.453 1.00 29.52   N
ANISOU 2752 N   ILE B 834   3725   3945   3547   17   27   497   N
ATOM   2753 CA ILE B 834   27.516 17.280 55.239 1.00 28.78   C
ANISOU 2753 CA ILE B 834   3725   3727   3483   26   15   476   C
ATOM   2754 C   ILE B 834   28.396 18.218 56.088 1.00 32.20   C
ANISOU 2754 C   ILE B 834   4180   4125   3930   150   9   447   C
ATOM   2755 O   ILE B 834   28.364 18.122 57.313 1.00 32.97   O
ANISOU 2755 O   ILE B 834   4303   4220   4005   186   14   471   O
ATOM   2756 CB ILE B 834   28.441 16.376 54.360 1.00 31.44   C
ANISOU 2756 CB ILE B 834   4150   3939   3857   -27   -3   436   C
ATOM   2757 CG1 ILE B 834   27.630 15.484 53.390 1.00 31.41   C
ANISOU 2757 CG1 ILE B 834   4171   3948   3816   -178   -3   459   C
ATOM   2758 CG2 ILE B 834   29.394 15.504 55.214 1.00 32.35   C
ANISOU 2758 CG2 ILE B 834   4358   3950   3983   24   -7   432   C
ATOM   2759 CD1 ILE B 834   28.492 14.732 52.359 1.00 36.78   C
ANISOU 2759 CD1 ILE B 834   4919   4534   4523   -197   -12   410   C
ATOM   2760 H   ILE B 834   26.855 18.291 53.506 1.00 29.52   H
ATOM   2761 HA ILE B 834   26.956 16.629 55.914 1.00 28.78   H
ATOM   2762 HB ILE B 834   29.066 17.021 53.741 1.00 31.44   H
ATOM   2763 HG13 ILE B 834   26.907 16.085 52.837 1.00 31.41   H
ATOM   2764 HG12 ILE B 834   27.039 14.767 53.962 1.00 31.41   H
ATOM   2765 HG21 ILE B 834   30.032 14.873 54.597 1.00 32.35   H
ATOM   2766 HG22 ILE B 834   30.065 16.100 55.830 1.00 32.35   H
ATOM   2767 HG23 ILE B 834   28.832 14.848 55.879 1.00 32.35   H
ATOM   2768 HD11 ILE B 834   28.512 13.664 52.573 1.00 36.78   H
ATOM   2769 HD12 ILE B 834   28.102 14.858 51.349 1.00 36.78   H
ATOM   2770 HD13 ILE B 834   29.521 15.083 52.341 1.00 36.78   H
ATOM   2771 N   ASP B 835   29.143 19.109 55.417 1.00 27.55   N
ANISOU 2771 N   ASP B 835   3592   3508   3368   195   -4   399   N
ATOM   2772 CA ASP B 835   30.064 20.073 56.022 1.00 27.79   C
ANISOU 2772 CA ASP B 835   3664   3505   3391   267   -20   372   C
ATOM   2773 C   ASP B 835   29.793 21.460 55.428 1.00 29.86   C
ANISOU 2773 C   ASP B 835   3931   3787   3629   309   -14   350   C
ATOM   2774 O   ASP B 835   29.528 21.572 54.230 1.00 26.59   O
ANISOU 2774 O   ASP B 835   3479   3388   3234   285   -11   336   O
ATOM   2775 CB ASP B 835   31.555 19.698 55.795 1.00 29.90   C

ANISOU 2775 CB ASP B 835 3956 3706 3698 259 -54 340 C
ATOM 2776 CG ASP B 835 32.003 18.332 56.345 1.00 40.28 C
ANISOU 2776 CG ASP B 835 5298 4982 5022 255 -55 363 C
ATOM 2777 OD1 ASP B 835 31.593 17.989 57.476 1.00 41.98 O
ANISOU 2777 OD1 ASP B 835 5537 5205 5209 276 -52 392 O
ATOM 2778 OD2 ASP B 835 32.860 17.701 55.685 1.00 46.03 O1-
ANISOU 2778 OD2 ASP B 835 6044 5669 5777 244 -56 354 O1-
ATOM 2779 H ASP B 835 29.079 19.144 54.410 1.00 27.55 H
ATOM 2780 HA ASP B 835 29.879 20.137 57.096 1.00 27.79 H
ATOM 2781 HB3 ASP B 835 32.200 20.454 56.245 1.00 29.90 H
ATOM 2782 HB2 ASP B 835 31.762 19.712 54.724 1.00 29.90 H
ATOM 2783 N ILE B 836 29.914 22.487 56.281 1.00 26.76 N
ANISOU 2783 N ILE B 836 3609 3378 3180 369 -13 346 N
ATOM 2784 CA ILE B 836 29.913 23.895 55.893 1.00 25.89 C
ANISOU 2784 CA ILE B 836 3567 3250 3018 417 -4 324 C
ATOM 2785 C ILE B 836 31.300 24.441 56.271 1.00 29.44 C
ANISOU 2785 C ILE B 836 4106 3625 3453 374 -52 283 C
ATOM 2786 O ILE B 836 31.695 24.352 57.435 1.00 29.47 O
ANISOU 2786 O ILE B 836 4162 3611 3425 369 -70 289 O
ATOM 2787 CB ILE B 836 28.835 24.732 56.654 1.00 28.80 C
ANISOU 2787 CB ILE B 836 3987 3665 3293 534 50 364 C
ATOM 2788 CG1 ILE B 836 27.410 24.167 56.462 1.00 29.09 C
ANISOU 2788 CG1 ILE B 836 3894 3835 3325 556 91 428 C
ATOM 2789 CG2 ILE B 836 28.861 26.231 56.268 1.00 28.80 C
ANISOU 2789 CG2 ILE B 836 4099 3621 3223 601 67 341 C
ATOM 2790 CD1 ILE B 836 26.355 24.794 57.387 1.00 31.80 C
ANISOU 2790 CD1 ILE B 836 4245 4276 3563 686 152 496 C
ATOM 2791 H ILE B 836 30.142 22.306 57.248 1.00 26.76 H
ATOM 2792 HA ILE B 836 29.758 24.011 54.818 1.00 25.89 H
ATOM 2793 HB ILE B 836 29.052 24.661 57.720 1.00 28.80 H
ATOM 2794 HG13 ILE B 836 27.406 23.092 56.642 1.00 29.09 H
ATOM 2795 HG12 ILE B 836 27.101 24.297 55.425 1.00 29.09 H
ATOM 2796 HG21 ILE B 836 28.160 26.807 56.872 1.00 28.80 H
ATOM 2797 HG22 ILE B 836 29.836 26.692 56.417 1.00 28.80 H
ATOM 2798 HG23 ILE B 836 28.590 26.373 55.222 1.00 28.80 H
ATOM 2799 HD11 ILE B 836 25.491 24.138 57.495 1.00 31.80 H
ATOM 2800 HD12 ILE B 836 26.756 24.979 58.384 1.00 31.80 H
ATOM 2801 HD13 ILE B 836 25.995 25.744 56.991 1.00 31.80 H
ATOM 2802 N PHE B 837 32.002 24.998 55.276 1.00 25.75 N
ANISOU 2802 N PHE B 837 3650 3134 3000 328 -75 249 N
ATOM 2803 CA PHE B 837 33.283 25.677 55.446 1.00 24.79 C
ANISOU 2803 CA PHE B 837 3596 2977 2847 251 -126 223 C
ATOM 2804 C PHE B 837 33.073 27.171 55.180 1.00 30.00 C
ANISOU 2804 C PHE B 837 4406 3574 3418 254 -121 200 C
ATOM 2805 O PHE B 837 32.303 27.521 54.288 1.00 30.18 O
ANISOU 2805 O PHE B 837 4430 3592 3444 302 -87 196 O
ATOM 2806 CB PHE B 837 34.317 25.113 54.448 1.00 25.75 C
ANISOU 2806 CB PHE B 837 3612 3136 3038 182 -160 212 C
ATOM 2807 CG PHE B 837 34.589 23.623 54.562 1.00 25.98 C
ANISOU 2807 CG PHE B 837 3537 3201 3134 199 -157 235 C
ATOM 2808 CD1 PHE B 837 35.423 23.127 55.586 1.00 27.69 C
ANISOU 2808 CD1 PHE B 837 3740 3447 3335 189 -187 257 C
ATOM 2809 CD2 PHE B 837 33.899 22.707 53.738 1.00 27.26 C
ANISOU 2809 CD2 PHE B 837 3636 3365 3357 221 -126 237 C
ATOM 2810 CE1 PHE B 837 35.607 21.758 55.725 1.00 28.59 C
ANISOU 2810 CE1 PHE B 837 3793 3576 3493 229 -175 282 C

```
ATOM   2811  CE2 PHE B 837      34.093  21.343  53.897  1.00 29.48           C
ANISOU 2811  CE2 PHE B 837     3878   3647   3675    235   -119    257       C
ATOM   2812  CZ  PHE B 837      34.946  20.871  54.885  1.00 27.58           C
ANISOU 2812  CZ  PHE B 837     3640   3421   3420    251   -140    280       C
ATOM   2813  H   PHE B 837      31.621  25.016  54.338  1.00 25.75           H
ATOM   2814  HA  PHE B 837      33.658  25.538  56.459  1.00 24.79           H
ATOM   2815  HB3 PHE B 837      35.264  25.631  54.603  1.00 25.75           H
ATOM   2816  HB2 PHE B 837      34.024  25.337  53.422  1.00 25.75           H
ATOM   2817  HD1 PHE B 837      35.925  23.808  56.259  1.00 27.69           H
ATOM   2818  HD2 PHE B 837      33.225  23.058  52.972  1.00 27.26           H
ATOM   2819  HE1 PHE B 837      36.262  21.380  56.494  1.00 28.59           H
ATOM   2820  HE2 PHE B 837      33.573  20.645  53.257  1.00 29.48           H
ATOM   2821  HZ  PHE B 837      35.082  19.807  55.007  1.00 27.58           H
ATOM   2822  N   LYS B 838      33.797  28.013  55.928  1.00 26.15           N
ANISOU 2822  N   LYS B 838     4065   3035   2837    198   -154    189       N
ATOM   2823  CA  LYS B 838      33.902  29.451  55.706  1.00 26.49           C
ANISOU 2823  CA  LYS B 838     4313   2986   2767    170   -157    165       C
ATOM   2824  C   LYS B 838      35.283  29.706  55.096  1.00 31.22           C
ANISOU 2824  C   LYS B 838     4875   3612   3375      4   -223    149       C
ATOM   2825  O   LYS B 838      36.293  29.392  55.729  1.00 31.67           O
ANISOU 2825  O   LYS B 838     4843   3744   3448    -93   -278    163       O
ATOM   2826  CB  LYS B 838      33.681  30.187  57.042  1.00 28.91           C
ANISOU 2826  CB  LYS B 838     4830   3213   2941    176   -160    164       C
ATOM   2827  CG  LYS B 838      33.537  31.713  56.917  1.00 34.46           C
ANISOU 2827  CG  LYS B 838     5814   3783   3498    195   -138    142       C
ATOM   2828  CD  LYS B 838      33.003  32.347  58.214  1.00 50.45           C
ANISOU 2828  CD  LYS B 838     8075   5716   5378    224   -130    141       C
ATOM   2829  CE  LYS B 838      32.629  33.829  58.044  1.00 56.13           C
ANISOU 2829  CE  LYS B 838     9131   6271   5924    283    -89    122       C
ATOM   2830  NZ  LYS B 838      32.011  34.379  59.263  1.00 60.31           N1+
ANISOU 2830  NZ  LYS B 838     9791   6761   6365    518      1    147       N1+
ATOM   2831  H   LYS B 838      34.414  27.635  56.638  1.00 26.15           H
ATOM   2832  HA  LYS B 838      33.133  29.784  55.007  1.00 26.49           H
ATOM   2833  HB3 LYS B 838      34.458  29.933  57.765  1.00 28.91           H
ATOM   2834  HB2 LYS B 838      32.747  29.818  57.460  1.00 28.91           H
ATOM   2835  HG3 LYS B 838      32.854  31.942  56.099  1.00 34.46           H
ATOM   2836  HG2 LYS B 838      34.496  32.160  56.652  1.00 34.46           H
ATOM   2837  HD3 LYS B 838      33.759  32.246  58.994  1.00 50.45           H
ATOM   2838  HD2 LYS B 838      32.134  31.786  58.561  1.00 50.45           H
ATOM   2839  HE3 LYS B 838      31.917  33.942  57.225  1.00 56.13           H
ATOM   2840  HE2 LYS B 838      33.511  34.417  57.789  1.00 56.13           H
ATOM   2841  HZ1 LYS B 838      32.659  34.319  60.035  1.00 60.31           H
ATOM   2842  HZ2 LYS B 838      31.751  35.344  59.111  1.00 60.31           H
ATOM   2843  HZ3 LYS B 838      31.180  33.844  59.485  1.00 60.31           H
ATOM   2844  N   ILE B 839      35.282  30.215  53.855  1.00 28.38           N
ANISOU 2844  N   ILE B 839     4559   3219   3007    -17   -215    130       N
ATOM   2845  CA  ILE B 839      36.474  30.371  53.025  1.00 27.99           C
ANISOU 2845  CA  ILE B 839     4446   3221   2967   -170   -271    125       C
ATOM   2846  C   ILE B 839      36.804  31.854  52.786  1.00 34.99           C
ANISOU 2846  C   ILE B 839     5561   4015   3721   -288   -297    105       C
ATOM   2847  O   ILE B 839      35.907  32.698  52.754  1.00 34.98           O
ANISOU 2847  O   ILE B 839     5755   3889   3645   -203   -250     89       O
ATOM   2848  CB  ILE B 839      36.316  29.642  51.659  1.00 30.26           C
ANISOU 2848  CB  ILE B 839     4538   3579   3380   -123   -249    125       C
ATOM   2849  CG1 ILE B 839      35.103  30.106  50.813  1.00 29.45           C
ANISOU 2849  CG1 ILE B 839     4491   3413   3285     -7   -187    112       C
```

```
ATOM   2850  CG2 ILE B 839      36.303  28.115  51.879  1.00 29.97           C
ANISOU 2850  CG2 ILE B 839     4309   3629   3450    -60   -241    147       C
ATOM   2851  CD1 ILE B 839      35.130  29.632  49.354  1.00 28.31           C
ANISOU 2851  CD1 ILE B 839     4195   3326   3236      5   -174    106       C
ATOM   2852  H   ILE B 839      34.409  30.495  53.429  1.00 28.38           H
ATOM   2853  HA  ILE B 839      37.327  29.926  53.532  1.00 27.99           H
ATOM   2854  HB  ILE B 839      37.212  29.858  51.074  1.00 30.26           H
ATOM   2855 HG13 ILE B 839      35.037  31.193  50.794  1.00 29.45           H
ATOM   2856 HG12 ILE B 839      34.182  29.766  51.284  1.00 29.45           H
ATOM   2857 HG21 ILE B 839      36.386  27.564  50.943  1.00 29.97           H
ATOM   2858 HG22 ILE B 839      37.139  27.803  52.503  1.00 29.97           H
ATOM   2859 HG23 ILE B 839      35.387  27.790  52.374  1.00 29.97           H
ATOM   2860 HD11 ILE B 839      34.162  29.805  48.886  1.00 28.31           H
ATOM   2861 HD12 ILE B 839      35.881  30.173  48.779  1.00 28.31           H
ATOM   2862 HD13 ILE B 839      35.342  28.568  49.259  1.00 28.31           H
ATOM   2863  N   GLU B 840      38.108  32.125  52.633  1.00 33.10           N
ANISOU 2863  N   GLU B 840     5293   3848   3436   -485   -371    117       N
ATOM   2864  CA  GLU B 840      38.673  33.454  52.441  1.00 33.79           C
ANISOU 2864  CA  GLU B 840     5597   3863   3377   -665   -414    107       C
ATOM   2865  C   GLU B 840      39.967  33.317  51.634  1.00 38.04           C
ANISOU 2865  C   GLU B 840     5969   4549   3936   -830   -469    131       C
ATOM   2866  O   GLU B 840      40.995  32.939  52.198  1.00 38.09           O
ANISOU 2866  O   GLU B 840     5847   4709   3917   -966   -534    171       O
ATOM   2867  CB  GLU B 840      38.847  34.149  53.816  1.00 35.60           C
ANISOU 2867  CB  GLU B 840     6029   4040   3457   -789   -462    110       C
ATOM   2868  CG  GLU B 840      39.629  35.487  53.792  1.00 51.32           C
ANISOU 2868  CG  GLU B 840     8379   5863   5257   -928   -482     87       C
ATOM   2869  CD  GLU B 840      39.763  36.198  55.152  1.00 84.95           C
ANISOU 2869  CD  GLU B 840    12850  10075   9353  -1082   -539     90       C
ATOM   2870  OE1 GLU B 840      39.027  35.846  56.102  1.00 68.70           O
ANISOU 2870  OE1 GLU B 840    10912   7921   7270   -932   -495     76       O
ATOM   2871  OE2 GLU B 840      40.613  37.113  55.216  1.00 88.65           O1-
ANISOU 2871  OE2 GLU B 840    13359  10617   9708  -1362   -630    112       O1-
ATOM   2872  H   GLU B 840      38.782  31.375  52.708  1.00 33.10           H
ATOM   2873  HA  GLU B 840      37.979  34.055  51.848  1.00 33.79           H
ATOM   2874  HB3 GLU B 840      39.331  33.461  54.511  1.00 35.60           H
ATOM   2875  HB2 GLU B 840      37.852  34.329  54.224  1.00 35.60           H
ATOM   2876  HG3 GLU B 840      39.143  36.174  53.099  1.00 51.32           H
ATOM   2877  HG2 GLU B 840      40.636  35.325  53.407  1.00 51.32           H
ATOM   2878  N   ARG B 841      39.887  33.614  50.326  1.00 34.61           N
ANISOU 2878  N   ARG B 841     5517   4090   3542   -802   -440    117       N
ATOM   2879  CA  ARG B 841      41.043  33.667  49.430  1.00 34.43           C
ANISOU 2879  CA  ARG B 841     5348   4203   3530   -941   -480    142       C
ATOM   2880  C   ARG B 841      41.916  34.899  49.721  1.00 39.77           C
ANISOU 2880  C   ARG B 841     6216   4870   4023  -1216   -555    158       C
ATOM   2881  O   ARG B 841      41.379  35.968  50.018  1.00 38.52           O
ANISOU 2881  O   ARG B 841     6373   4520   3744  -1256   -546    127       O
ATOM   2882  CB  ARG B 841      40.574  33.725  47.966  1.00 32.13           C
ANISOU 2882  CB  ARG B 841     5041   3855   3310   -839   -425    117       C
ATOM   2883  CG  ARG B 841      39.777  32.498  47.472  1.00 31.62           C
ANISOU 2883  CG  ARG B 841     4754   3847   3413   -637   -369    112       C
ATOM   2884  CD  ARG B 841      39.437  32.523  45.967  1.00 29.16           C
ANISOU 2884  CD  ARG B 841     4397   3528   3154   -595   -336     97       C
ATOM   2885  NE  ARG B 841      40.640  32.719  45.135  1.00 30.20           N
ANISOU 2885  NE  ARG B 841     4430   3785   3258   -752   -381    125       N
ATOM   2886  CZ  ARG B 841      41.056  33.861  44.554  1.00 46.47           C
```

```
ANISOU 2886  CZ  ARG B 841     6628   5809   5221   -900   -406    125       C
ATOM   2887  NH1 ARG B 841      42.280  33.907  44.015  1.00 33.67           N
ANISOU 2887  NH1 ARG B 841     4886   4341   3566  -1053   -449    164       N
ATOM   2888  NH2 ARG B 841      40.293  34.964  44.509  1.00 29.63           N1+
ANISOU 2888  NH2 ARG B 841     4760   3491   3006   -888   -383     94       N1+
ATOM   2889  H   ARG B 841      39.005  33.908  49.932  1.00 34.61           H
ATOM   2890  HA  ARG B 841      41.641  32.764  49.567  1.00 34.43           H
ATOM   2891  HB3 ARG B 841      41.467  33.850  47.353  1.00 32.13           H
ATOM   2892  HB2 ARG B 841      39.975  34.619  47.806  1.00 32.13           H
ATOM   2893  HG3 ARG B 841      38.827  32.532  48.007  1.00 31.62           H
ATOM   2894  HG2 ARG B 841      40.234  31.550  47.753  1.00 31.62           H
ATOM   2895  HD3 ARG B 841      38.598  33.176  45.732  1.00 29.16           H
ATOM   2896  HD2 ARG B 841      39.104  31.522  45.694  1.00 29.16           H
ATOM   2897  HE  ARG B 841      41.271  31.928  45.129  1.00 30.20           H
ATOM   2898 HH12 ARG B 841      42.622  34.755  43.587  1.00 33.67           H
ATOM   2899 HH11 ARG B 841      42.882  33.093  44.035  1.00 33.67           H
ATOM   2900 HH22 ARG B 841      40.638  35.814  44.089  1.00 29.63           H
ATOM   2901 HH21 ARG B 841      39.365  34.951  44.915  1.00 29.63           H
ATOM   2902  N   GLU B 842      43.243  34.734  49.580  1.00 38.71           N
ANISOU 2902  N   GLU B 842     5908   4951   3851  -1405   -626    214       N
ATOM   2903  CA  GLU B 842      44.227  35.812  49.706  1.00 40.22           C
ANISOU 2903  CA  GLU B 842     6254   5179   3851  -1718   -710    245       C
ATOM   2904  C   GLU B 842      44.034  36.899  48.631  1.00 45.52           C
ANISOU 2904  C   GLU B 842     7129   5714   4454  -1806   -698    218       C
ATOM   2905  O   GLU B 842      44.045  36.590  47.439  1.00 45.38           O
ANISOU 2905  O   GLU B 842     6961   5742   4541  -1710   -658    216       O
ATOM   2906  CB  GLU B 842      45.659  35.238  49.652  1.00 41.87           C
ANISOU 2906  CB  GLU B 842     6171   5710   4029  -1888   -784    332       C
ATOM   2907  CG  GLU B 842      46.029  34.359  50.870  1.00 53.62           C
ANISOU 2907  CG  GLU B 842     7579   7331   5464  -1945   -837    376       C
ATOM   2908  CD  GLU B 842      47.447  33.757  50.843  1.00 81.98           C
ANISOU 2908  CD  GLU B 842    10857  11285   9009  -2078   -902    482       C
ATOM   2909  OE1 GLU B 842      47.679  32.845  51.667  1.00 75.00           O
ANISOU 2909  OE1 GLU B 842     9781  10547   8170  -1966   -904    523       O
ATOM   2910  OE2 GLU B 842      48.281  34.189  50.014  1.00 79.12           O1-
ANISOU 2910  OE2 GLU B 842    10437  11074   8553  -2287   -948    533       O1-
ATOM   2911  H   GLU B 842      43.613  33.824  49.337  1.00 38.71           H
ATOM   2912  HA  GLU B 842      44.086  36.270  50.687  1.00 40.22           H
ATOM   2913  HB3 GLU B 842      46.371  36.062  49.571  1.00 41.87           H
ATOM   2914  HB2 GLU B 842      45.775  34.649  48.740  1.00 41.87           H
ATOM   2915  HG3 GLU B 842      45.308  33.548  50.973  1.00 53.62           H
ATOM   2916  HG2 GLU B 842      45.940  34.954  51.780  1.00 53.62           H
ATOM   2917  N   GLY B 843      43.865  38.149  49.092  1.00 43.19           N
ANISOU 2917  N   GLY B 843     7202   5233   3975  -1974   -727    196       N
ATOM   2918  CA  GLY B 843      43.746  39.345  48.256  1.00 43.18           C
ANISOU 2918  CA  GLY B 843     7477   5064   3867  -2074   -717    173       C
ATOM   2919  C   GLY B 843      42.342  39.563  47.663  1.00 48.10           C
ANISOU 2919  C   GLY B 843     8279   5447   4550  -1794   -613    114       C
ATOM   2920  O   GLY B 843      42.137  40.598  47.030  1.00 48.96           O
ANISOU 2920  O   GLY B 843     8676   5383   4542  -1858   -599     95       O
ATOM   2921  H   GLY B 843      43.855  38.298  50.091  1.00 43.19           H
ATOM   2922  HA3 GLY B 843      44.473  39.306  47.443  1.00 43.18           H
ATOM   2923  HA2 GLY B 843      44.004  40.213  48.862  1.00 43.18           H
ATOM   2924  N   GLU B 844      41.382  38.636  47.852  1.00 44.70           N
ANISOU 2924  N   GLU B 844     7683   5016   4286  -1489   -541     92       N
ATOM   2925  CA  GLU B 844      40.026  38.728  47.295  1.00 44.37           C
```

```
ANISOU 2925  CA  GLU B 844     7754  4807  4298 -1211  -442    55       C
ATOM   2926  C   GLU B 844     39.159  39.814  47.958  1.00 49.07           C
ANISOU 2926  C   GLU B 844     8787  5143  4716 -1154  -405    27       C
ATOM   2927  O   GLU B 844     38.379  40.461  47.260  1.00 47.30           O
ANISOU 2927  O   GLU B 844     8787  4762  4425 -1057  -349    12       O
ATOM   2928  CB  GLU B 844     39.355  37.341  47.327  1.00 45.32           C
ANISOU 2928  CB  GLU B 844     7581  5024  4615  -947  -385    53       C
ATOM   2929  CG  GLU B 844     37.930  37.303  46.724  1.00 52.89           C
ANISOU 2929  CG  GLU B 844     8513  5927  5655  -715  -302    37       C
ATOM   2930  CD  GLU B 844     37.350  35.888  46.623  1.00 61.63           C
ANISOU 2930  CD  GLU B 844     9342  7139  6935  -503  -255    41       C
ATOM   2931  OE1 GLU B 844     37.258  35.219  47.674  1.00 38.14           O
ANISOU 2931  OE1 GLU B 844     6407  4127  3957  -367  -222    41       O
ATOM   2932  OE2 GLU B 844     36.995  35.490  45.491  1.00 47.64           O1-
ANISOU 2932  OE2 GLU B 844     7332  5483  5288  -478  -250    46       O1-
ATOM   2933  H   GLU B 844     41.594  37.802  48.384  1.00 44.70           H
ATOM   2934  HA  GLU B 844     40.131  39.003  46.244  1.00 44.37           H
ATOM   2935  HB3 GLU B 844     39.329  36.976  48.355  1.00 45.32           H
ATOM   2936  HB2 GLU B 844     39.991  36.648  46.778  1.00 45.32           H
ATOM   2937  HG3 GLU B 844     37.940  37.778  45.743  1.00 52.89           H
ATOM   2938  HG2 GLU B 844     37.245  37.893  47.333  1.00 52.89           H
ATOM   2939  N   CYS B 845     39.333  40.013  49.275  1.00 48.19           N
ANISOU 2939  N   CYS B 845     8815  4983  4514 -1205  -431    25       N
ATOM   2940  CA  CYS B 845     38.670  41.062  50.054  1.00 49.92           C
ANISOU 2940  CA  CYS B 845     9482  4948  4536 -1151  -395     0       C
ATOM   2941  C   CYS B 845     39.127  42.475  49.630  1.00 52.73           C
ANISOU 2941  C   CYS B 845    10239  5119  4679 -1361  -420   -10       C
ATOM   2942  O   CYS B 845     38.293  43.376  49.538  1.00 51.24           O
ANISOU 2942  O   CYS B 845    10399  4701  4368 -1196  -344   -29       O
ATOM   2943  CB  CYS B 845     38.849  40.814  51.569  1.00 51.83           C
ANISOU 2943  CB  CYS B 845     9781  5200  4713 -1232  -439     1       C
ATOM   2944  SG  CYS B 845     37.973  42.034  52.595  1.00 56.77           S
ANISOU 2944  SG  CYS B 845    11018  5502  5050 -1226  -409   -29       S
ATOM   2945  H   CYS B 845     39.984  39.426  49.778  1.00 48.19           H
ATOM   2946  HA  CYS B 845     37.602  40.991  49.838  1.00 49.92           H
ATOM   2947  HB3 CYS B 845     39.906  40.825  51.839  1.00 51.83           H
ATOM   2948  HB2 CYS B 845     38.469  39.826  51.829  1.00 51.83           H
ATOM   2949  HG  CYS B 845     38.724  43.082  52.246  1.00 56.77           H
ATOM   2950  N   GLN B 846     40.433  42.622  49.343  1.00 49.77           N
ANISOU 2950  N   GLN B 846     9801  4859  4250 -1714  -524    14       N
ATOM   2951  CA  GLN B 846     41.054  43.863  48.877  1.00 50.13           C
ANISOU 2951  CA  GLN B 846    10188  4769  4091 -1987  -567    15       C
ATOM   2952  C   GLN B 846     40.686  44.190  47.415  1.00 52.18           C
ANISOU 2952  C   GLN B 846    10445  4973  4407 -1852  -503    10       C
ATOM   2953  O   GLN B 846     40.515  45.367  47.094  1.00 52.48           O
ANISOU 2953  O   GLN B 846    10904  4772  4266 -1881  -475     -5       O
ATOM   2954  CB  GLN B 846     42.578  43.754  49.092  1.00 52.27           C
ANISOU 2954  CB  GLN B 846    10302  5259  4301 -2406  -699     62       C
ATOM   2955  CG  GLN B 846     43.345  45.074  48.883  1.00 79.62           C
ANISOU 2955  CG  GLN B 846    14239  8548  7463 -2774  -771     65       C
ATOM   2956  CD  GLN B 846     44.827  44.920  49.228  1.00106.89           C
ANISOU 2956  CD  GLN B 846    17517 12266 10831 -3207  -909    129       C
ATOM   2957  NE2 GLN B 846     45.279  45.575  50.300  1.00102.27           N
ANISOU 2957  NE2 GLN B 846    17129 11652 10076 -3417  -980    134       N
ATOM   2958  OE1 GLN B 846     45.560  44.224  48.531  1.00101.96           O
ANISOU 2958  OE1 GLN B 846    16579 11889 10274 -3357  -954    181       O
```

```
ATOM   2959  H   GLN B 846      41.048  41.825  49.424  1.00 49.77           H
ATOM   2960  HA  GLN B 846      40.680  44.676  49.503  1.00 50.13           H
ATOM   2961  HB3 GLN B 846      42.988  42.978  48.443  1.00 52.27           H
ATOM   2962  HB2 GLN B 846      42.758  43.413  50.114  1.00 52.27           H
ATOM   2963  HG3 GLN B 846      42.903  45.865  49.490  1.00 79.62           H
ATOM   2964  HG2 GLN B 846      43.270  45.401  47.845  1.00 79.62           H
ATOM   2965 HE22 GLN B 846      46.252  45.501  50.560  1.00102.27           H
ATOM   2966 HE21 GLN B 846      44.657  46.142  50.857  1.00102.27           H
ATOM   2967  N   ARG B 847      40.523  43.149  46.577  1.00 46.82           N
ANISOU 2967  N   ARG B 847     9319   4504   3966  -1697   -478     24       N
ATOM   2968  CA  ARG B 847      40.023  43.231  45.201  1.00 45.56           C
ANISOU 2968  CA  ARG B 847     9083   4337   3891  -1555   -420     22       C
ATOM   2969  C   ARG B 847      38.549  43.683  45.137  1.00 49.14           C
ANISOU 2969  C   ARG B 847     9786   4577   4310  -1206   -303     -3       C
ATOM   2970  O   ARG B 847      38.193  44.443  44.238  1.00 48.72           O
ANISOU 2970  O   ARG B 847     9932   4395   4185  -1145   -258     -5       O
ATOM   2971  CB  ARG B 847      40.232  41.860  44.512  1.00 42.05           C
ANISOU 2971  CB  ARG B 847     8110   4168   3699  -1459   -420     39       C
ATOM   2972  CG  ARG B 847      39.826  41.796  43.022  1.00 45.21           C
ANISOU 2972  CG  ARG B 847     8377   4605   4195  -1358   -377     41       C
ATOM   2973  CD  ARG B 847      39.913  40.389  42.397  1.00 48.01           C
ANISOU 2973  CD  ARG B 847     8261   5200   4780  -1244   -370     52       C
ATOM   2974  NE  ARG B 847      38.837  39.489  42.858  1.00 57.08           N
ANISOU 2974  NE  ARG B 847     9298   6340   6049   -947   -302     35       N
ATOM   2975  CZ  ARG B 847      38.576  38.240  42.425  1.00 66.13           C
ANISOU 2975  CZ  ARG B 847    10105   7642   7378   -810   -280     39       C
ATOM   2976  NH1 ARG B 847      39.335  37.627  41.504  1.00 50.40           N
ANISOU 2976  NH1 ARG B 847     7856   5818   5475   -906   -313     55       N
ATOM   2977  NH2 ARG B 847      37.522  37.586  42.928  1.00 51.93           N1+
ANISOU 2977  NH2 ARG B 847     8238   5835   5660   -576   -224     31       N1+
ATOM   2978  H   ARG B 847      40.717  42.216  46.916  1.00 46.82           H
ATOM   2979  HA  ARG B 847      40.623  43.975  44.673  1.00 45.56           H
ATOM   2980  HB3 ARG B 847      39.681  41.100  45.063  1.00 42.05           H
ATOM   2981  HB2 ARG B 847      41.283  41.582  44.592  1.00 42.05           H
ATOM   2982  HG3 ARG B 847      40.377  42.518  42.419  1.00 45.21           H
ATOM   2983  HG2 ARG B 847      38.785  42.107  42.956  1.00 45.21           H
ATOM   2984  HD3 ARG B 847      40.832  39.918  42.748  1.00 48.01           H
ATOM   2985  HD2 ARG B 847      39.987  40.441  41.310  1.00 48.01           H
ATOM   2986  HE  ARG B 847      38.223  39.876  43.561  1.00 57.08           H
ATOM   2987 HH12 ARG B 847      39.104  36.699  41.179  1.00 50.40           H
ATOM   2988 HH11 ARG B 847      40.127  38.109  41.105  1.00 50.40           H
ATOM   2989 HH22 ARG B 847      36.912  38.029  43.603  1.00 51.93           H
ATOM   2990 HH21 ARG B 847      37.302  36.648  42.627  1.00 51.93           H
ATOM   2991  N   TYR B 848      37.736  43.207  46.095  1.00 45.43           N
ANISOU 2991  N   TYR B 848     9284   4092   3884   -971   -253    -11       N
ATOM   2992  CA  TYR B 848      36.304  43.485  46.208  1.00 45.53           C
ANISOU 2992  CA  TYR B 848     9467   3970   3861   -609   -139    -14       C
ATOM   2993  C   TYR B 848      35.972  44.865  46.818  1.00 52.28           C
ANISOU 2993  C   TYR B 848    10901   4522   4440   -571    -95    -25       C
ATOM   2994  O   TYR B 848      34.813  45.273  46.751  1.00 51.65           O
ANISOU 2994  O   TYR B 848    11005   4327   4294   -259     11    -14       O
ATOM   2995  CB  TYR B 848      35.649  42.317  46.981  1.00 45.38           C
ANISOU 2995  CB  TYR B 848     9175   4085   3982   -407   -109     -6       C
ATOM   2996  CG  TYR B 848      34.144  42.413  47.154  1.00 44.41           C
ANISOU 2996  CG  TYR B 848     9115   3915   3845    -22      9     12       C
ATOM   2997  CD1 TYR B 848      33.297  42.187  46.049  1.00 45.46           C
```

ANISOU 2997 CD1 TYR B 848 9035 4152 4087 185 69 36 C
ATOM 2998 CD2 TYR B 848 33.586 42.755 48.404 1.00 44.08 C
ANISOU 2998 CD2 TYR B 848 9319 3756 3673 137 60 15 C
ATOM 2999 CE1 TYR B 848 31.906 42.343 46.180 1.00 44.56 C
ANISOU 2999 CE1 TYR B 848 8931 4053 3947 532 174 72 C
ATOM 3000 CE2 TYR B 848 32.192 42.888 48.539 1.00 44.11 C
ANISOU 3000 CE2 TYR B 848 9341 3766 3653 506 173 51 C
ATOM 3001 CZ TYR B 848 31.355 42.699 47.422 1.00 47.45 C
ANISOU 3001 CZ TYR B 848 9527 4322 4180 699 228 84 C
ATOM 3002 OH TYR B 848 30.009 42.862 47.537 1.00 43.90 O
ANISOU 3002 OH TYR B 848 9068 3926 3686 1055 338 138 O
ATOM 3003 H TYR B 848 38.113 42.572 46.785 1.00 45.43 H
ATOM 3004 HA TYR B 848 35.887 43.491 45.202 1.00 45.53 H
ATOM 3005 HB3 TYR B 848 36.111 42.220 47.965 1.00 45.38 H
ATOM 3006 HB2 TYR B 848 35.857 41.379 46.463 1.00 45.38 H
ATOM 3007 HD1 TYR B 848 33.711 41.904 45.096 1.00 45.46 H
ATOM 3008 HD2 TYR B 848 34.226 42.932 49.256 1.00 44.08 H
ATOM 3009 HE1 TYR B 848 31.261 42.193 45.329 1.00 44.56 H
ATOM 3010 HE2 TYR B 848 31.770 43.154 49.497 1.00 44.11 H
ATOM 3011 HH TYR B 848 29.717 42.914 48.454 1.00 43.90 H
ATOM 3012 N LYS B 849 36.972 45.569 47.380 1.00 51.91 N
ANISOU 3012 N LYS B 849 11151 4356 4218 -884 -174 -41 N
ATOM 3013 CA LYS B 849 36.824 46.846 48.088 1.00 53.57 C
ANISOU 3013 CA LYS B 849 11972 4248 4133 -901 -145 -58 C
ATOM 3014 C LYS B 849 36.063 47.987 47.354 1.00 59.51 C
ANISOU 3014 C LYS B 849 13109 4769 4734 -689 -46 -54 C
ATOM 3015 O LYS B 849 35.283 48.659 48.031 1.00 59.87 O
ANISOU 3015 O LYS B 849 13525 4610 4614 -423 49 -54 O
ATOM 3016 H LYS B 849 37.902 45.174 47.375 1.00 51.91 H
ATOM 3017 CB LYS B 849 38.204 47.293 48.625 1.00 0.00 C
ATOM 3018 CG LYS B 849 38.184 48.544 49.529 1.00 0.00 C
ATOM 3019 CD LYS B 849 39.573 48.951 50.060 1.00 0.00 C
ATOM 3020 CE LYS B 849 40.560 49.481 48.998 1.00 0.00 C
ATOM 3021 NZ LYS B 849 40.122 50.757 48.400 1.00 0.00 N1+
ATOM 3022 HB2 LYS B 849 38.636 46.470 49.197 1.00 0.00 H
ATOM 3023 HB3 LYS B 849 38.879 47.458 47.788 1.00 0.00 H
ATOM 3024 HG2 LYS B 849 37.736 49.391 49.010 1.00 0.00 H
ATOM 3025 HG3 LYS B 849 37.531 48.345 50.380 1.00 0.00 H
ATOM 3026 HD2 LYS B 849 39.448 49.695 50.848 1.00 0.00 H
ATOM 3027 HD3 LYS B 849 40.025 48.088 50.551 1.00 0.00 H
ATOM 3028 HE2 LYS B 849 41.533 49.646 49.460 1.00 0.00 H
ATOM 3029 HE3 LYS B 849 40.715 48.748 48.207 1.00 0.00 H
ATOM 3030 HZ1 LYS B 849 40.025 51.455 49.124 1.00 0.00 H
ATOM 3031 HZ2 LYS B 849 40.806 51.066 47.724 1.00 0.00 H
ATOM 3032 HZ3 LYS B 849 39.232 50.626 47.940 1.00 0.00 H
ATOM 3033 HA LYS B 849 36.218 46.605 48.964 1.00 53.57 H
ATOM 3034 N PRO B 850 36.217 48.168 46.016 1.00 57.71 N
ANISOU 3034 N PRO B 850 12816 4566 4544 -755 -50 -43 N
ATOM 3035 CA PRO B 850 35.415 49.154 45.256 1.00 58.11 C
ANISOU 3035 CA PRO B 850 13249 4393 4437 -509 55 -32 C
ATOM 3036 C PRO B 850 33.904 48.855 45.158 1.00 63.59 C
ANISOU 3036 C PRO B 850 13814 5143 5205 0 190 -1 C
ATOM 3037 O PRO B 850 33.133 49.781 44.916 1.00 64.07 O
ANISOU 3037 O PRO B 850 14242 5013 5091 270 295 20 O
ATOM 3038 CB PRO B 850 36.065 49.178 43.859 1.00 59.72 C
ANISOU 3038 CB PRO B 850 13300 4679 4714 -709 8 -23 C

ATOM 3039 CG PRO B 850 37.459 48.611 44.062 1.00 64.07 C
ANISOU 3039 CG PRO B 850 13532 5436 5376 -1120 -129 -29 C
ATOM 3040 CD PRO B 850 37.238 47.576 45.152 1.00 59.70 C
ANISOU 3040 CD PRO B 850 12667 5048 4966 -1031 -142 -34 C
ATOM 3041 HA PRO B 850 35.553 50.129 45.729 1.00 58.11 H
ATOM 3042 HB3 PRO B 850 36.086 50.177 43.423 1.00 59.72 H
ATOM 3043 HB2 PRO B 850 35.519 48.533 43.167 1.00 59.72 H
ATOM 3044 HG3 PRO B 850 38.123 49.394 44.429 1.00 64.07 H
ATOM 3045 HG2 PRO B 850 37.894 48.195 43.152 1.00 64.07 H
ATOM 3046 HD2 PRO B 850 36.834 46.662 44.716 1.00 59.70 H
ATOM 3047 HD3 PRO B 850 38.172 47.335 45.655 1.00 59.70 H
ATOM 3048 N PHE B 851 33.522 47.579 45.335 1.00 60.14 N
ANISOU 3048 N PHE B 851 12867 4978 5008 130 190 11 N
ATOM 3049 CA PHE B 851 32.158 47.070 45.177 1.00 60.15 C
ANISOU 3049 CA PHE B 851 12656 5107 5093 560 300 54 C
ATOM 3050 C PHE B 851 31.478 46.736 46.519 1.00 67.98 C
ANISOU 3050 C PHE B 851 13654 6119 6058 761 347 65 C
ATOM 3051 O PHE B 851 30.314 46.339 46.507 1.00 68.22 O
ANISOU 3051 O PHE B 851 13510 6278 6133 1111 440 114 O
ATOM 3052 CB PHE B 851 32.211 45.818 44.275 1.00 61.31 C
ANISOU 3052 CB PHE B 851 12210 5561 5526 549 267 68 C
ATOM 3053 CG PHE B 851 32.755 46.071 42.881 1.00 61.93 C
ANISOU 3053 CG PHE B 851 12242 5646 5641 398 234 63 C
ATOM 3054 CD1 PHE B 851 31.956 46.717 41.915 1.00 64.63 C
ANISOU 3054 CD1 PHE B 851 12712 5935 5908 644 321 98 C
ATOM 3055 CD2 PHE B 851 34.110 45.806 42.585 1.00 63.04 C
ANISOU 3055 CD2 PHE B 851 12208 5866 5880 24 121 34 C
ATOM 3056 CE1 PHE B 851 32.482 47.017 40.666 1.00 65.27 C
ANISOU 3056 CE1 PHE B 851 12761 6019 6018 502 291 94 C
ATOM 3057 CE2 PHE B 851 34.617 46.116 41.331 1.00 65.75 C
ANISOU 3057 CE2 PHE B 851 12507 6226 6248 -111 95 36 C
ATOM 3058 CZ PHE B 851 33.805 46.710 40.373 1.00 64.00 C
ANISOU 3058 CZ PHE B 851 12427 5931 5959 123 179 61 C
ATOM 3059 H PHE B 851 34.221 46.879 45.545 1.00 60.14 H
ATOM 3060 HA PHE B 851 31.532 47.816 44.685 1.00 60.15 H
ATOM 3061 HB3 PHE B 851 31.208 45.410 44.162 1.00 61.31 H
ATOM 3062 HB2 PHE B 851 32.802 45.032 44.748 1.00 61.31 H
ATOM 3063 HD1 PHE B 851 30.933 46.974 42.139 1.00 64.63 H
ATOM 3064 HD2 PHE B 851 34.754 45.359 43.328 1.00 63.04 H
ATOM 3065 HE1 PHE B 851 31.862 47.493 39.920 1.00 65.27 H
ATOM 3066 HE2 PHE B 851 35.649 45.902 41.099 1.00 65.75 H
ATOM 3067 HZ PHE B 851 34.208 46.946 39.400 1.00 64.00 H
ATOM 3068 N LYS B 852 32.198 46.898 47.644 1.00 66.76 N
ANISOU 3068 N LYS B 852 13684 5859 5822 532 281 28 N
ATOM 3069 CA LYS B 852 31.758 46.530 48.993 1.00 67.78 C
ANISOU 3069 CA LYS B 852 13810 6012 5933 674 310 33 C
ATOM 3070 C LYS B 852 30.585 47.367 49.550 1.00 74.23 C
ANISOU 3070 C LYS B 852 14992 6675 6535 1092 454 71 C
ATOM 3071 O LYS B 852 29.899 46.880 50.448 1.00 74.17 O
ANISOU 3071 O LYS B 852 14845 6777 6560 1309 506 99 O
ATOM 3072 H LYS B 852 33.148 47.234 47.568 1.00 66.76 H
ATOM 3073 CB LYS B 852 32.997 46.530 49.921 1.00 0.00 C
ATOM 3074 CG LYS B 852 32.733 46.054 51.363 1.00 0.00 C
ATOM 3075 CD LYS B 852 34.010 45.963 52.215 1.00 0.00 C
ATOM 3076 CE LYS B 852 33.775 45.423 53.639 1.00 0.00 C
ATOM 3077 NZ LYS B 852 32.947 46.321 54.467 1.00 0.00 N1+

```
ATOM   3078  HB2 LYS B 852      33.754  45.878  49.482  1.00  0.00           H
ATOM   3079  HB3 LYS B 852      33.438  47.528  49.945  1.00  0.00           H
ATOM   3080  HG2 LYS B 852      32.038  46.736  51.851  1.00  0.00           H
ATOM   3081  HG3 LYS B 852      32.243  45.079  51.335  1.00  0.00           H
ATOM   3082  HD2 LYS B 852      34.725  45.312  51.710  1.00  0.00           H
ATOM   3083  HD3 LYS B 852      34.488  46.942  52.262  1.00  0.00           H
ATOM   3084  HE2 LYS B 852      33.303  44.441  53.598  1.00  0.00           H
ATOM   3085  HE3 LYS B 852      34.734  45.290  54.141  1.00  0.00           H
ATOM   3086  HZ1 LYS B 852      32.039  46.433  54.038  1.00  0.00           H
ATOM   3087  HZ2 LYS B 852      32.835  45.925  55.389  1.00  0.00           H
ATOM   3088  HZ3 LYS B 852      33.396  47.223  54.542  1.00  0.00           H
ATOM   3089  HA  LYS B 852      31.391  45.505  48.933  1.00 67.78           H
ATOM   3090  N   GLN B 853      30.362  48.579  49.013  1.00 71.86           N
ANISOU 3090  N   GLN B 853    15163   6134   6009   1216    525     80       N
ATOM   3091  CA  GLN B 853      29.292  49.492  49.432  1.00 72.08           C
ANISOU 3091  CA  GLN B 853    15591   6002   5793   1649    676    126       C
ATOM   3092  C   GLN B 853      28.187  49.653  48.362  1.00 76.02           C
ANISOU 3092  C   GLN B 853    16000   6599   6284   2028    792    199       C
ATOM   3093  O   GLN B 853      27.180  50.301  48.646  1.00 76.12           O
ANISOU 3093  O   GLN B 853    16350   6498   6072   2428    930    254       O
ATOM   3094  H   GLN B 853      30.968  48.909  48.275  1.00 71.86           H
ATOM   3095  CB  GLN B 853      29.946  50.827  49.868  1.00  0.00           C
ATOM   3096  CG  GLN B 853      28.975  51.876  50.463  1.00  0.00           C
ATOM   3097  CD  GLN B 853      29.642  53.047  51.194  1.00  0.00           C
ATOM   3098  OE1 GLN B 853      28.975  53.753  51.946  1.00  0.00           O
ATOM   3099  NE2 GLN B 853      30.940  53.281  50.984  1.00  0.00           N
ATOM   3100  HB2 GLN B 853      30.699  50.587  50.620  1.00  0.00           H
ATOM   3101  HB3 GLN B 853      30.486  51.263  49.026  1.00  0.00           H
ATOM   3102  HG2 GLN B 853      28.349  52.303  49.680  1.00  0.00           H
ATOM   3103  HG3 GLN B 853      28.303  51.388  51.170  1.00  0.00           H
ATOM   3104 HE22 GLN B 853      31.394  54.052  51.452  1.00  0.00           H
ATOM   3105 HE21 GLN B 853      31.471  52.698  50.355  1.00  0.00           H
ATOM   3106  HA  GLN B 853      28.785  49.086  50.309  1.00 72.08           H
ATOM   3107  N   LEU B 854      28.354  49.036  47.175  1.00 71.34           N
ANISOU 3107  N   LEU B 854    14947   6235   5926   1921    739    207       N
ATOM   3108  CA  LEU B 854      27.381  49.029  46.071  1.00 70.06           C
ANISOU 3108  CA  LEU B 854    14625   6214   5781   2235    829    280       C
ATOM   3109  C   LEU B 854      26.034  48.398  46.488  1.00 71.42           C
ANISOU 3109  C   LEU B 854    14522   6640   5973   2648    934    368       C
ATOM   3110  O   LEU B 854      26.024  47.502  47.327  1.00 71.79           O
ANISOU 3110  O   LEU B 854    14303   6834   6138   2598    902    363       O
ATOM   3111  CB  LEU B 854      28.026  48.276  44.878  1.00 70.24           C
ANISOU 3111  CB  LEU B 854    14175   6444   6068   1978    731    260       C
ATOM   3112  CG  LEU B 854      27.280  48.364  43.524  1.00 74.88           C
ANISOU 3112  CG  LEU B 854    14758   7061   6632   2130    782    303       C
ATOM   3113  CD1 LEU B 854      27.223  49.810  42.984  1.00 75.28           C
ANISOU 3113  CD1 LEU B 854    15349   6776   6479   1980    774    265       C
ATOM   3114  CD2 LEU B 854      27.881  47.380  42.495  1.00 77.07           C
ANISOU 3114  CD2 LEU B 854    14484   7606   7193   1932    696    292       C
ATOM   3115  H   LEU B 854      29.185  48.483  47.025  1.00 71.34           H
ATOM   3116  HA  LEU B 854      27.203  50.069  45.795  1.00 70.06           H
ATOM   3117  HB3 LEU B 854      28.127  47.226  45.156  1.00 70.24           H
ATOM   3118  HB2 LEU B 854      29.044  48.640  44.730  1.00 70.24           H
ATOM   3119  HG  LEU B 854      26.253  48.037  43.679  1.00 74.88           H
ATOM   3120 HD11 LEU B 854      27.557  49.896  41.950  1.00 75.28           H
ATOM   3121 HD12 LEU B 854      26.202  50.190  43.014  1.00 75.28           H
```

```
ATOM   3122 HD13 LEU B 854      27.838  50.492  43.571  1.00 75.28           H
ATOM   3123 HD21 LEU B 854      28.128  47.848  41.542  1.00 77.07           H
ATOM   3124 HD22 LEU B 854      28.796  46.920  42.864  1.00 77.07           H
ATOM   3125 HD23 LEU B 854      27.184  46.571  42.280  1.00 77.07           H
ATOM   3126  N   HIE B 855      24.917  48.866  45.905  1.00 65.18           N
ANISOU 3126  N   HIE B 855    13803   5913   5051   3057   1062    460       N
ATOM   3127  CA  HIE B 855      23.581  48.315  46.163  1.00 63.55           C
ANISOU 3127  CA  HIE B 855    13306   6002   4839   3453   1166    568       C
ATOM   3128  C   HIE B 855      23.380  46.922  45.529  1.00 62.61           C
ANISOU 3128  C   HIE B 855    12507   6266   5017   3329   1095    592       C
ATOM   3129  O   HIE B 855      24.123  46.544  44.623  1.00 61.90           O
ANISOU 3129  O   HIE B 855    12232   6183   5106   3007    989    530       O
ATOM   3130  CB  HIE B 855      22.500  49.331  45.728  1.00 64.70           C
ANISOU 3130  CB  HIE B 855    13755   6116   4711   3953   1332    675       C
ATOM   3131  CG  HIE B 855      22.429  49.686  44.260  1.00 68.53           C
ANISOU 3131  CG  HIE B 855    14152   6669   5218   4005   1342    711       C
ATOM   3132  CD2 HIE B 855      21.367  49.533  43.394  1.00 70.49           C
ANISOU 3132  CD2 HIE B 855    14073   7233   5475   4306   1414    827       C
ATOM   3133  ND1 HIE B 855      23.488  50.286  43.568  1.00 70.54           N
ANISOU 3133  ND1 HIE B 855    14687   6654   5463   3722   1274    629       N
ATOM   3134  CE1 HIE B 855      23.033  50.468  42.336  1.00 70.18           C
ANISOU 3134  CE1 HIE B 855    14478   6754   5434   3868   1308    690       C
ATOM   3135  NE2 HIE B 855      21.780  50.043  42.176  1.00 70.46           N
ANISOU 3135  NE2 HIE B 855    14147   7144   5480   4218   1390    810       N
ATOM   3136  H   HIE B 855      24.971  49.597  45.210  1.00 65.18           H
ATOM   3137  HA  HIE B 855      23.480  48.191  47.243  1.00 63.55           H
ATOM   3138  HB3 HIE B 855      22.635  50.259  46.284  1.00 64.70           H
ATOM   3139  HB2 HIE B 855      21.518  48.957  46.023  1.00 64.70           H
ATOM   3140  HD2 HIE B 855      20.377  49.128  43.545  1.00 70.49           H
ATOM   3141  HE1 HIE B 855      23.619  50.910  41.543  1.00 70.18           H
ATOM   3142  N   ASN B 856      22.385  46.177  46.042  1.00 55.94           N
ANISOU 3142  N   ASN B 856    11308   5736   4212   3565   1151    683       N
ATOM   3143  CA  ASN B 856      22.010  44.811  45.635  1.00 54.31           C
ANISOU 3143  CA  ASN B 856    10489   5898   4249   3461   1092    719       C
ATOM   3144  C   ASN B 856      23.157  43.787  45.806  1.00 54.68           C
ANISOU 3144  C   ASN B 856    10291   5930   4555   3003    942    610       C
ATOM   3145  O   ASN B 856      23.375  42.955  44.926  1.00 54.15           O
ANISOU 3145  O   ASN B 856     9887   6004   4683   2793    863    589       O
ATOM   3146  CB  ASN B 856      21.397  44.799  44.204  1.00 55.52           C
ANISOU 3146  CB  ASN B 856    10437   6233   4424   3567   1114    783       C
ATOM   3147  CG  ASN B 856      20.049  45.518  44.094  1.00 88.45           C
ANISOU 3147  CG  ASN B 856    14625  10600   8381   4053   1262    933       C
ATOM   3148  ND2 ASN B 856      19.878  46.348  43.065  1.00 84.51           N
ANISOU 3148  ND2 ASN B 856    14317  10037   7754   4228   1319    971       N
ATOM   3149  OD1 ASN B 856      19.153  45.296  44.906  1.00 81.70           O
ANISOU 3149  OD1 ASN B 856    13601   9975   7464   4284   1329   1027       O
ATOM   3150  H   ASN B 856      21.827  46.567  46.788  1.00 55.94           H
ATOM   3151  HA  ASN B 856      21.233  44.524  46.345  1.00 54.31           H
ATOM   3152  HB3 ASN B 856      21.194  43.772  43.897  1.00 55.52           H
ATOM   3153  HB2 ASN B 856      22.103  45.198  43.474  1.00 55.52           H
ATOM   3154 HD22 ASN B 856      18.999  46.828  42.941  1.00 84.51           H
ATOM   3155 HD21 ASN B 856      20.624  46.494  42.393  1.00 84.51           H
ATOM   3156  N   ARG B 857      23.850  43.838  46.957  1.00 48.74           N
ANISOU 3156  N   ARG B 857     9713   5016   3789   2867    907    549       N
ATOM   3157  CA  ARG B 857      24.795  42.802  47.384  1.00 47.50           C
ANISOU 3157  CA  ARG B 857     9313   4881   3856   2486    777    466       C
```

```
ATOM   3158  C   ARG B 857      24.009  41.617  47.960  1.00 47.91           C
ANISOU 3158  C   ARG B 857     8940   5237   4026   2563    786    528       C
ATOM   3159  O   ARG B 857      23.363  41.772  48.998  1.00 47.22           O
ANISOU 3159  O   ARG B 857     8920   5213   3808   2827    874    595       O
ATOM   3160  CB  ARG B 857      25.783  43.379  48.413  1.00 48.88           C
ANISOU 3160  CB  ARG B 857     9851   4770   3951   2288    728    382       C
ATOM   3161  CG  ARG B 857      26.780  44.365  47.772  1.00 57.44           C
ANISOU 3161  CG  ARG B 857    11274   5587   4965   2063    675    311       C
ATOM   3162  CD  ARG B 857      27.679  45.142  48.753  1.00 71.95           C
ANISOU 3162  CD  ARG B 857    13508   7156   6676   1861    628    243       C
ATOM   3163  NE  ARG B 857      26.915  45.905  49.756  1.00 83.12           N
ANISOU 3163  NE  ARG B 857    15376   8385   7820   2154    739    274       N
ATOM   3164  CZ  ARG B 857      26.650  45.565  51.031  1.00103.85           C
ANISOU 3164  CZ  ARG B 857    18104  10979  10375   2220    759    274       C
ATOM   3165  NH1 ARG B 857      27.116  44.438  51.590  1.00 90.57           N
ANISOU 3165  NH1 ARG B 857    16105   9432   8874   2000    671    244       N
ATOM   3166  NH2 ARG B 857      25.885  46.379  51.767  1.00 97.91           N1+
ANISOU 3166  NH2 ARG B 857    17794  10050   9357   2517    872    306       N1+
ATOM   3167  H   ARG B 857      23.629  44.547  47.641  1.00 48.74           H
ATOM   3168  HA  ARG B 857      25.374  42.463  46.528  1.00 47.50           H
ATOM   3169  HB3 ARG B 857      26.354  42.571  48.876  1.00 48.88           H
ATOM   3170  HB2 ARG B 857      25.220  43.850  49.219  1.00 48.88           H
ATOM   3171  HG3 ARG B 857      26.322  45.024  47.036  1.00 57.44           H
ATOM   3172  HG2 ARG B 857      27.447  43.730  47.189  1.00 57.44           H
ATOM   3173  HD3 ARG B 857      28.158  45.931  48.174  1.00 71.95           H
ATOM   3174  HD2 ARG B 857      28.501  44.554  49.154  1.00 71.95           H
ATOM   3175  HE  ARG B 857      26.504  46.756  49.396  1.00 83.12           H
ATOM   3176 HH12 ARG B 857      26.864  44.189  52.536  1.00 90.57           H
ATOM   3177 HH11 ARG B 857      27.709  43.811  51.057  1.00 90.57           H
ATOM   3178 HH22 ARG B 857      25.635  46.124  52.712  1.00 97.91           H
ATOM   3179 HH21 ARG B 857      25.521  47.235  51.374  1.00 97.91           H
ATOM   3180  N   ARG B 858      24.061  40.476  47.254  1.00 40.95           N
ANISOU 3180  N   ARG B 858     7635   4552   3371   2353    706    515       N
ATOM   3181  CA  ARG B 858      23.313  39.266  47.594  1.00 38.59           C
ANISOU 3181  CA  ARG B 858     6939   4542   3181   2379    705    575       C
ATOM   3182  C   ARG B 858      24.241  38.052  47.681  1.00 37.92           C
ANISOU 3182  C   ARG B 858     6612   4480   3314   2047    591    504       C
ATOM   3183  O   ARG B 858      25.150  37.913  46.863  1.00 36.18           O
ANISOU 3183  O   ARG B 858     6385   4158   3202   1806    512    428       O
ATOM   3184  CB  ARG B 858      22.195  39.008  46.560  1.00 38.08           C
ANISOU 3184  CB  ARG B 858     6584   4761   3125   2530    746    671       C
ATOM   3185  CG  ARG B 858      21.206  40.173  46.407  1.00 57.50           C
ANISOU 3185  CG  ARG B 858     9111   7369   5369   2934    878    799       C
ATOM   3186  CD  ARG B 858      20.014  39.838  45.499  1.00 67.79           C
ANISOU 3186  CD  ARG B 858    10110   8982   6664   3062    909    904       C
ATOM   3187  NE  ARG B 858      19.232  41.046  45.184  1.00 79.04           N
ANISOU 3187  NE  ARG B 858    11770  10391   7871   3407   1019    981       N
ATOM   3188  CZ  ARG B 858      18.170  41.143  44.363  1.00 95.36           C
ANISOU 3188  CZ  ARG B 858    13631  12731   9871   3594   1068   1094       C
ATOM   3189  NH1 ARG B 858      17.670  40.082  43.711  1.00 84.88           N
ANISOU 3189  NH1 ARG B 858    11861  11716   8673   3441   1011   1141       N
ATOM   3190  NH2 ARG B 858      17.595  42.341  44.195  1.00 81.62           N1+
ANISOU 3190  NH2 ARG B 858    12141  10953   7917   3932   1175   1164       N1+
ATOM   3191  H   ARG B 858      24.622  40.430  46.413  1.00 40.95           H
ATOM   3192  HA  ARG B 858      22.849  39.402  48.569  1.00 38.59           H
ATOM   3193  HB3 ARG B 858      21.643  38.110  46.844  1.00 38.08           H
```

ATOM 3194 HB2 ARG B 858 22.640 38.792 45.588 1.00 38.08 H
ATOM 3195 HG3 ARG B 858 21.694 41.098 46.098 1.00 57.50 H
ATOM 3196 HG2 ARG B 858 20.816 40.361 47.408 1.00 57.50 H
ATOM 3197 HD3 ARG B 858 19.327 39.212 46.068 1.00 67.79 H
ATOM 3198 HD2 ARG B 858 20.306 39.262 44.619 1.00 67.79 H
ATOM 3199 HE ARG B 858 19.532 41.883 45.661 1.00 79.04 H
ATOM 3200 HH12 ARG B 858 16.861 40.176 43.118 1.00 84.88 H
ATOM 3201 HH11 ARG B 858 18.096 39.172 43.822 1.00 84.88 H
ATOM 3202 HH22 ARG B 858 16.788 42.446 43.599 1.00 81.62 H
ATOM 3203 HH21 ARG B 858 17.964 43.156 44.667 1.00 81.62 H
ATOM 3204 N LEU B 859 23.945 37.175 48.653 1.00 32.50 N
ANISOU 3204 N LEU B 859 5728 3940 2680 2048 590 536 N
ATOM 3205 CA LEU B 859 24.590 35.880 48.845 1.00 30.85 C
ANISOU 3205 CA LEU B 859 5288 3773 2662 1777 497 485 C
ATOM 3206 C LEU B 859 23.892 34.862 47.928 1.00 31.39 C
ANISOU 3206 C LEU B 859 4992 4089 2844 1727 479 533 C
ATOM 3207 O LEU B 859 22.716 34.574 48.149 1.00 29.71 O
ANISOU 3207 O LEU B 859 4605 4105 2579 1879 533 629 O
ATOM 3208 CB LEU B 859 24.497 35.496 50.343 1.00 30.72 C
ANISOU 3208 CB LEU B 859 5282 3766 2622 1804 507 498 C
ATOM 3209 CG LEU B 859 25.383 34.298 50.752 1.00 34.57 C
ANISOU 3209 CG LEU B 859 5627 4232 3276 1535 413 436 C
ATOM 3210 CD1 LEU B 859 26.886 34.650 50.672 1.00 33.83 C
ANISOU 3210 CD1 LEU B 859 5722 3916 3215 1325 338 340 C
ATOM 3211 CD2 LEU B 859 24.987 33.755 52.141 1.00 33.95 C
ANISOU 3211 CD2 LEU B 859 5531 4203 3166 1594 435 468 C
ATOM 3212 H LEU B 859 23.167 37.374 49.270 1.00 32.50 H
ATOM 3213 HA LEU B 859 25.639 35.970 48.569 1.00 30.85 H
ATOM 3214 HB3 LEU B 859 23.456 35.281 50.590 1.00 30.72 H
ATOM 3215 HB2 LEU B 859 24.767 36.352 50.963 1.00 30.72 H
ATOM 3216 HG LEU B 859 25.203 33.489 50.046 1.00 34.57 H
ATOM 3217 HD11 LEU B 859 27.426 34.429 51.593 1.00 33.83 H
ATOM 3218 HD12 LEU B 859 27.370 34.087 49.876 1.00 33.83 H
ATOM 3219 HD13 LEU B 859 27.052 35.707 50.460 1.00 33.83 H
ATOM 3220 HD21 LEU B 859 25.843 33.415 52.723 1.00 33.95 H
ATOM 3221 HD22 LEU B 859 24.472 34.505 52.740 1.00 33.95 H
ATOM 3222 HD23 LEU B 859 24.316 32.900 52.042 1.00 33.95 H
ATOM 3223 N LEU B 860 24.601 34.392 46.889 1.00 27.03 N
ANISOU 3223 N LEU B 860 4340 3503 2428 1514 406 472 N
ATOM 3224 CA LEU B 860 24.039 33.576 45.806 1.00 26.61 C
ANISOU 3224 CA LEU B 860 3997 3647 2468 1440 383 503 C
ATOM 3225 C LEU B 860 24.872 32.303 45.592 1.00 30.62 C
ANISOU 3225 C LEU B 860 4341 4144 3148 1177 297 442 C
ATOM 3226 O LEU B 860 26.086 32.319 45.804 1.00 31.15 O
ANISOU 3226 O LEU B 860 4516 4045 3276 1043 249 367 O
ATOM 3227 CB LEU B 860 23.997 34.412 44.504 1.00 26.46 C
ANISOU 3227 CB LEU B 860 4029 3614 2409 1495 398 505 C
ATOM 3228 CG LEU B 860 23.096 35.670 44.553 1.00 30.62 C
ANISOU 3228 CG LEU B 860 4725 4164 2746 1793 495 580 C
ATOM 3229 CD1 LEU B 860 23.337 36.578 43.326 1.00 30.28 C
ANISOU 3229 CD1 LEU B 860 4793 4037 2673 1809 498 559 C
ATOM 3230 CD2 LEU B 860 21.606 35.314 44.751 1.00 33.41 C
ANISOU 3230 CD2 LEU B 860 4852 4824 3018 1982 557 708 C
ATOM 3231 H LEU B 860 25.567 34.672 46.774 1.00 27.03 H
ATOM 3232 HA LEU B 860 23.027 33.255 46.055 1.00 26.61 H
ATOM 3233 HB3 LEU B 860 23.652 33.779 43.687 1.00 26.46 H

ATOM 3234 HB2 LEU B 860 25.015 34.711 44.248 1.00 26.46 H
ATOM 3235 HG LEU B 860 23.396 36.264 45.414 1.00 30.62 H
ATOM 3236 HD11 LEU B 860 22.421 36.818 42.789 1.00 30.28 H
ATOM 3237 HD12 LEU B 860 23.775 37.528 43.633 1.00 30.28 H
ATOM 3238 HD13 LEU B 860 24.020 36.126 42.607 1.00 30.28 H
ATOM 3239 HD21 LEU B 860 20.944 35.840 44.063 1.00 33.41 H
ATOM 3240 HD22 LEU B 860 21.418 34.248 44.615 1.00 33.41 H
ATOM 3241 HD23 LEU B 860 21.282 35.571 45.760 1.00 33.41 H
ATOM 3242 N TRP B 861 24.190 31.231 45.151 1.00 27.60 N
ANISOU 3242 N TRP B 861 3711 3948 2827 1106 281 481 N
ATOM 3243 CA TRP B 861 24.774 29.920 44.862 1.00 27.83 C
ANISOU 3243 CA TRP B 861 3603 3975 2995 883 213 435 C
ATOM 3244 C TRP B 861 25.556 29.892 43.540 1.00 29.51 C
ANISOU 3244 C TRP B 861 3813 4114 3286 761 170 373 C
ATOM 3245 O TRP B 861 25.145 30.536 42.575 1.00 27.60 O
ANISOU 3245 O TRP B 861 3576 3910 2999 827 190 390 O
ATOM 3246 CB TRP B 861 23.670 28.841 44.807 1.00 26.88 C
ANISOU 3246 CB TRP B 861 3260 4077 2875 835 213 508 C
ATOM 3247 CG TRP B 861 22.829 28.667 46.033 1.00 28.54 C
ANISOU 3247 CG TRP B 861 3425 4409 3009 933 253 585 C
ATOM 3248 CD1 TRP B 861 21.580 29.155 46.205 1.00 31.63 C
ANISOU 3248 CD1 TRP B 861 3721 5022 3273 1098 313 696 C
ATOM 3249 CD2 TRP B 861 23.175 28.006 47.286 1.00 28.46 C
ANISOU 3249 CD2 TRP B 861 3438 4338 3038 873 238 568 C
ATOM 3250 CE2 TRP B 861 22.071 28.127 48.183 1.00 32.31 C
ANISOU 3250 CE2 TRP B 861 3854 5003 3418 1005 292 667 C
ATOM 3251 CE3 TRP B 861 24.318 27.321 47.766 1.00 29.43 C
ANISOU 3251 CE3 TRP B 861 3633 4286 3263 738 187 487 C
ATOM 3252 NE1 TRP B 861 21.120 28.819 47.460 1.00 30.97 N
ANISOU 3252 NE1 TRP B 861 3605 5017 3145 1145 339 748 N
ATOM 3253 CZ2 TRP B 861 22.105 27.611 49.488 1.00 31.62 C
ANISOU 3253 CZ2 TRP B 861 3773 4908 3333 989 294 679 C
ATOM 3254 CZ3 TRP B 861 24.367 26.806 49.075 1.00 30.58 C
ANISOU 3254 CZ3 TRP B 861 3784 4426 3410 727 188 501 C
ATOM 3255 CH2 TRP B 861 23.268 26.962 49.940 1.00 31.32 C
ANISOU 3255 CH2 TRP B 861 3819 4678 3404 847 241 592 C
ATOM 3256 H TRP B 861 23.201 31.316 44.960 1.00 27.60 H
ATOM 3257 HA TRP B 861 25.449 29.676 45.678 1.00 27.83 H
ATOM 3258 HB3 TRP B 861 24.128 27.871 44.608 1.00 26.88 H
ATOM 3259 HB2 TRP B 861 23.000 29.030 43.968 1.00 26.88 H
ATOM 3260 HD1 TRP B 861 21.038 29.713 45.458 1.00 31.63 H
ATOM 3261 HE3 TRP B 861 25.177 27.214 47.122 1.00 29.43 H
ATOM 3262 HE1 TRP B 861 20.204 29.089 47.792 1.00 30.97 H
ATOM 3263 HZ2 TRP B 861 21.256 27.724 50.144 1.00 31.62 H
ATOM 3264 HZ3 TRP B 861 25.259 26.304 49.423 1.00 30.58 H
ATOM 3265 HH2 TRP B 861 23.320 26.586 50.950 1.00 31.32 H
ATOM 3266 N HIS B 862 26.614 29.068 43.511 1.00 25.45 N
ANISOU 3266 N HIS B 862 3282 3507 2881 596 116 308 N
ATOM 3267 CA HIS B 862 27.352 28.692 42.307 1.00 23.22 C
ANISOU 3267 CA HIS B 862 2974 3175 2675 479 77 255 C
ATOM 3268 C HIS B 862 27.851 27.254 42.491 1.00 24.34 C
ANISOU 3268 C HIS B 862 3030 3312 2905 342 40 230 C
ATOM 3269 O HIS B 862 28.691 27.017 43.359 1.00 21.77 O
ANISOU 3269 O HIS B 862 2750 2904 2616 305 21 202 O
ATOM 3270 CB HIS B 862 28.503 29.689 42.040 1.00 23.93 C
ANISOU 3270 CB HIS B 862 3214 3114 2763 462 62 200 C

```
ATOM   3271  CG  HIS B 862      29.402  29.332  40.877  1.00 27.16           C
ANISOU 3271  CG  HIS B 862     3580   3501   3237    360     32    158       C
ATOM   3272  CD2 HIS B 862      30.476  28.473  40.774  1.00 28.97           C
ANISOU 3272  CD2 HIS B 862     3763   3697   3547    247     -5    117       C
ATOM   3273  ND1 HIS B 862      29.264  29.898  39.623  1.00 28.66           N
ANISOU 3273  ND1 HIS B 862     3774   3718   3396    393     47    164       N
ATOM   3274  CE1 HIS B 862      30.209  29.372  38.842  1.00 28.32           C
ANISOU 3274  CE1 HIS B 862     3688   3653   3421    289     16    123       C
ATOM   3275  NE2 HIS B 862      30.981  28.495  39.472  1.00 28.75           N
ANISOU 3275  NE2 HIS B 862     3711   3673   3538    208    -12     96       N
ATOM   3276  H   HIS B 862      26.888  28.588  44.358  1.00 25.45           H
ATOM   3277  HA  HIS B 862      26.676  28.708  41.449  1.00 23.22           H
ATOM   3278  HB3 HIS B 862      29.126  29.802  42.926  1.00 23.93           H
ATOM   3279  HB2 HIS B 862      28.083  30.674  41.844  1.00 23.93           H
ATOM   3280  HD2 HIS B 862      30.914  27.837  41.529  1.00 28.97           H
ATOM   3281  HD1 HIS B 862      28.573  30.583  39.341  1.00 28.66           H
ATOM   3282  HE1 HIS B 862      30.334  29.634  37.801  1.00 28.32           H
ATOM   3283  N   GLY B 863      27.343  26.327  41.665  1.00 21.74           N
ANISOU 3283  N   GLY B 863     2595   3073   2592    271     30    246       N
ATOM   3284  CA  GLY B 863      27.781  24.933  41.658  1.00 22.13           C
ANISOU 3284  CA  GLY B 863     2606   3101   2700    143      1    223       C
ATOM   3285  C   GLY B 863      28.695  24.693  40.456  1.00 26.47           C
ANISOU 3285  C   GLY B 863     3186   3566   3306     81    -20    164       C
ATOM   3286  O   GLY B 863      28.588  25.354  39.422  1.00 25.11           O
ANISOU 3286  O   GLY B 863     3011   3408   3122     99    -17    154       O
ATOM   3287  H   GLY B 863      26.673  26.599  40.960  1.00 21.74           H
ATOM   3288  HA3 GLY B 863      26.920  24.275  41.597  1.00 22.13           H
ATOM   3289  HA2 GLY B 863      28.304  24.660  42.575  1.00 22.13           H
ATOM   3290  N   SER B 864      29.587  23.708  40.616  1.00 22.85           N
ANISOU 3290  N   SER B 864     2760   3025   2896     26    -37    131       N
ATOM   3291  CA  SER B 864      30.597  23.296  39.647  1.00 22.04           C
ANISOU 3291  CA  SER B 864     2686   2854   2832     -6    -47     84       C
ATOM   3292  C   SER B 864      31.066  21.878  40.018  1.00 24.94           C
ANISOU 3292  C   SER B 864     3097   3160   3220    -46    -51     72       C
ATOM   3293  O   SER B 864      30.863  21.447  41.156  1.00 23.83           O
ANISOU 3293  O   SER B 864     2970   3010   3076    -44    -50     93       O
ATOM   3294  CB  SER B 864      31.749  24.332  39.643  1.00 24.01           C
ANISOU 3294  CB  SER B 864     2966   3060   3097     40    -52     61       C
ATOM   3295  OG  SER B 864      32.751  24.019  38.698  1.00 31.05           O
ANISOU 3295  OG  SER B 864     3857   3935   4006     21    -56     32       O
ATOM   3296  H   SER B 864      29.602  23.191  41.485  1.00 22.85           H
ATOM   3297  HA  SER B 864      30.141  23.254  38.656  1.00 22.04           H
ATOM   3298  HB3 SER B 864      32.211  24.398  40.629  1.00 24.01           H
ATOM   3299  HB2 SER B 864      31.372  25.328  39.408  1.00 24.01           H
ATOM   3300  HG  SER B 864      33.269  23.282  39.032  1.00 31.05           H
ATOM   3301  N   ARG B 865      31.690  21.171  39.059  1.00 22.60           N
ANISOU 3301  N   ARG B 865     2839   2815   2932    -69    -49     41       N
ATOM   3302  CA  ARG B 865      32.308  19.858  39.281  1.00 22.71           C
ANISOU 3302  CA  ARG B 865     2938   2748   2942    -74    -40     32       C
ATOM   3303  C   ARG B 865      33.452  19.931  40.309  1.00 27.22           C
ANISOU 3303  C   ARG B 865     3511   3297   3536     11    -37     38       C
ATOM   3304  O   ARG B 865      34.167  20.931  40.351  1.00 26.60           O
ANISOU 3304  O   ARG B 865     3377   3257   3473     51    -45     38       O
ATOM   3305  CB  ARG B 865      32.842  19.287  37.952  1.00 22.50           C
ANISOU 3305  CB  ARG B 865     2978   2670   2901    -78    -28     -1       C
ATOM   3306  CG  ARG B 865      31.791  19.092  36.847  1.00 33.93           C
```

```
ANISOU 3306  CG  ARG B 865     4452  4131  4307  -188   -37    -7       C
ATOM   3307  CD  ARG B 865      32.431  18.539  35.562  1.00 39.49           C
ANISOU 3307  CD  ARG B 865     5254  4763  4986  -179   -20   -44       C
ATOM   3308  NE  ARG B 865      31.438  18.141  34.550  1.00 52.19           N
ANISOU 3308  NE  ARG B 865     6884  6397  6548  -298   -37   -51       N
ATOM   3309  CZ  ARG B 865      30.918  16.912  34.370  1.00 74.34           C
ANISOU 3309  CZ  ARG B 865     9827  9137  9281  -415   -43   -58       C
ATOM   3310  NH1 ARG B 865      31.234  15.883  35.170  1.00 66.99           N
ANISOU 3310  NH1 ARG B 865     9046  8090  8316  -413   -27   -59       N
ATOM   3311  NH2 ARG B 865      30.065  16.703  33.359  1.00 65.40           N1+
ANISOU 3311  NH2 ARG B 865     8698  8055  8095  -543   -66   -58       N1+
ATOM   3312  H   ARG B 865      31.819  21.577  38.143  1.00 22.60           H
ATOM   3313  HA  ARG B 865      31.540  19.187  39.668  1.00 22.71           H
ATOM   3314  HB3 ARG B 865      33.316  18.324  38.146  1.00 22.50           H
ATOM   3315  HB2 ARG B 865      33.630  19.933  37.570  1.00 22.50           H
ATOM   3316  HG3 ARG B 865      31.391  20.081  36.618  1.00 33.93           H
ATOM   3317  HG2 ARG B 865      30.945  18.491  37.180  1.00 33.93           H
ATOM   3318  HD3 ARG B 865      33.192  17.782  35.756  1.00 39.49           H
ATOM   3319  HD2 ARG B 865      32.963  19.357  35.079  1.00 39.49           H
ATOM   3320  HE  ARG B 865      31.147  18.876  33.922  1.00 52.19           H
ATOM   3321 HH12 ARG B 865      30.847  14.961  34.999  1.00 66.99           H
ATOM   3322 HH11 ARG B 865      31.870  16.015  35.941  1.00 66.99           H
ATOM   3323 HH22 ARG B 865      29.678  15.778  33.214  1.00 65.40           H
ATOM   3324 HH21 ARG B 865      29.800  17.454  32.740  1.00 65.40           H
ATOM   3325  N   THR B 866      33.606  18.855  41.099  1.00 24.62           N
ANISOU 3325  N   THR B 866     3253  2910  3193    25   -27    51       N
ATOM   3326  CA  THR B 866      34.603  18.692  42.167  1.00 24.85           C
ANISOU 3326  CA  THR B 866     3279  2934  3229   109   -24    69       C
ATOM   3327  C   THR B 866      36.068  18.910  41.721  1.00 28.52           C
ANISOU 3327  C   THR B 866     3704  3438  3696   190   -18    66       C
ATOM   3328  O   THR B 866      36.860  19.445  42.498  1.00 29.95           O
ANISOU 3328  O   THR B 866     3814  3685  3881   218   -35    85       O
ATOM   3329  CB  THR B 866      34.487  17.268  42.780  1.00 32.17           C
ANISOU 3329  CB  THR B 866     4317  3779  4126   119    -7    84       C
ATOM   3330  CG2 THR B 866      35.536  16.892  43.841  1.00 30.74           C
ANISOU 3330  CG2 THR B 866     4129  3607  3943   221    -2   112       C
ATOM   3331  OG1 THR B 866      33.200  17.119  43.351  1.00 34.33           O
ANISOU 3331  OG1 THR B 866     4596  4058  4391    21   -20   100       O
ATOM   3332  H   THR B 866      32.961  18.083  41.003  1.00 24.62           H
ATOM   3333  HA  THR B 866      34.377  19.426  42.942  1.00 24.85           H
ATOM   3334  HB  THR B 866      34.552  16.532  41.976  1.00 32.17           H
ATOM   3335 HG21 THR B 866      35.288  15.947  44.325  1.00 30.74           H
ATOM   3336 HG22 THR B 866      36.530  16.769  43.409  1.00 30.74           H
ATOM   3337 HG23 THR B 866      35.603  17.662  44.610  1.00 30.74           H
ATOM   3338  HG1 THR B 866      33.139  16.245  43.747  1.00 34.33           H
ATOM   3339  N   THR B 867      36.383  18.530  40.471  1.00 24.10           N
ANISOU 3339  N   THR B 867     3187  2852  3119   214     5    47       N
ATOM   3340  CA  THR B 867      37.694  18.668  39.831  1.00 23.78           C
ANISOU 3340  CA  THR B 867     3100  2872  3064   297    20    54       C
ATOM   3341  C   THR B 867      38.108  20.127  39.531  1.00 28.85           C
ANISOU 3341  C   THR B 867     3623  3613  3727   248   -8    53       C
ATOM   3342  O   THR B 867      39.296  20.369  39.318  1.00 30.07           O
ANISOU 3342  O   THR B 867     3712  3854  3860   296   -3    74       O
ATOM   3343  CB  THR B 867      37.711  17.891  38.487  1.00 29.12           C
ANISOU 3343  CB  THR B 867     3883  3479  3705   334    58     30       C
ATOM   3344  CG2 THR B 867      37.390  16.400  38.651  1.00 23.88           C
```

ANISOU 3344 CG2 THR B 867   3380   2708   2987   417   96   40   C
ATOM   3345 OG1 THR B 867   36.811 18.437 37.535 1.00 32.17   O
ANISOU 3345 OG1 THR B 867   4300   3822   4102   215   42   -4   O
ATOM   3346 H   THR B 867   35.665 18.124 39.888 1.00 24.10   H
ATOM   3347 HA THR B 867   38.445 18.240 40.498 1.00 23.78   H
ATOM   3348 HB THR B 867   38.711 17.958 38.058 1.00 29.12   H
ATOM   3349 HG21 THR B 867   37.470 15.889 37.693 1.00 23.88   H
ATOM   3350 HG22 THR B 867   38.089 15.925 39.340 1.00 23.88   H
ATOM   3351 HG23 THR B 867   36.381 16.233 39.027 1.00 23.88   H
ATOM   3352 HG1 THR B 867   37.068 19.344 37.343 1.00 32.17   H
ATOM   3353 N   ASN B 868   37.142 21.062 39.523 1.00 25.85   N
ANISOU 3353 N   ASN B 868   3224   3229   3371   161   -35   38   N
ATOM   3354 CA ASN B 868   37.370 22.484 39.255 1.00 25.71   C
ANISOU 3354 CA ASN B 868   3147   3270   3351   114   -59   37   C
ATOM   3355 C   ASN B 868   37.791 23.261 40.512 1.00 27.56   C
ANISOU 3355 C   ASN B 868   3361   3542   3570   89   -88   61   C
ATOM   3356 O   ASN B 868   38.354 24.343 40.359 1.00 25.56   O
ANISOU 3356 O   ASN B 868   3091   3331   3291   34   -111   66   O
ATOM   3357 CB ASN B 868   36.094 23.135 38.677 1.00 30.08   C
ANISOU 3357 CB ASN B 868   3721   3795   3913   61   -62   15   C
ATOM   3358 CG ASN B 868   35.672 22.609 37.301 1.00 55.33   C
ANISOU 3358 CG ASN B 868   6938   6973   7113   52   -44   -8   C
ATOM   3359 ND2 ASN B 868   35.386 23.519 36.371 1.00 47.61   N
ANISOU 3359 ND2 ASN B 868   5935   6024   6129   44   -42   -19   N
ATOM   3360 OD1 ASN B 868   35.576 21.405 37.079 1.00 53.69   O
ANISOU 3360 OD1 ASN B 868   6773   6725   6901   33   -35   -14   O
ATOM   3361 H   ASN B 868   36.183 20.792 39.702 1.00 25.85   H
ATOM   3362 HA ASN B 868   38.162 22.561 38.510 1.00 25.71   H
ATOM   3363 HB3 ASN B 868   36.250 24.210 38.591 1.00 30.08   H
ATOM   3364 HB2 ASN B 868   35.261 23.019 39.368 1.00 30.08   H
ATOM   3365 HD22 ASN B 868   35.044 23.223 35.468 1.00 47.61   H
ATOM   3366 HD21 ASN B 868   35.522 24.505 36.557 1.00 47.61   H
ATOM   3367 N   PHE B 869   37.496 22.733 41.715 1.00 24.45   N
ANISOU 3367 N   PHE B 869   2987   3125   3178   114   -90   77   N
ATOM   3368 CA PHE B 869   37.669 23.434 42.992 1.00 24.33   C
ANISOU 3368 CA PHE B 869   2975   3129   3139   83   -119   95   C
ATOM   3369 C   PHE B 869   39.105 23.793 43.408 1.00 29.38   C
ANISOU 3369 C   PHE B 869   3557   3869   3736   60   -148   129   C
ATOM   3370 O   PHE B 869   39.265 24.705 44.219 1.00 28.48   O
ANISOU 3370 O   PHE B 869   3469   3770   3581   -17   -182   135   O
ATOM   3371 CB PHE B 869   36.864 22.747 44.111 1.00 25.38   C
ANISOU 3371 CB PHE B 869   3146   3215   3283   113   -112   104   C
ATOM   3372 CG PHE B 869   35.421 23.206 44.149 1.00 26.46   C
ANISOU 3372 CG PHE B 869   3323   3307   3426   96   -100   90   C
ATOM   3373 CD1 PHE B 869   34.460 22.622 43.298 1.00 29.28   C
ANISOU 3373 CD1 PHE B 869   3681   3643   3800   91   -79   80   C
ATOM   3374 CD2 PHE B 869   35.075 24.363 44.877 1.00 28.09   C
ANISOU 3374 CD2 PHE B 869   3568   3506   3598   87   -110   96   C
ATOM   3375 CE1 PHE B 869   33.176 23.146 43.240 1.00 29.25   C
ANISOU 3375 CE1 PHE B 869   3680   3651   3784   78   -69   87   C
ATOM   3376 CE2 PHE B 869   33.786 24.868 44.805 1.00 30.29   C
ANISOU 3376 CE2 PHE B 869   3870   3777   3863   107   -88   100   C
ATOM   3377 CZ PHE B 869   32.835 24.259 43.996 1.00 27.94   C
ANISOU 3377 CZ PHE B 869   3534   3497   3585   103   -69   101   C
ATOM   3378 H   PHE B 869   37.051 21.827 41.761 1.00 24.45   H
ATOM   3379 HA PHE B 869   37.201 24.402 42.841 1.00 24.33   H

```
ATOM   3380  HB3 PHE B 869      37.301  22.955  45.089  1.00 25.38           H
ATOM   3381  HB2 PHE B 869      36.892  21.665  43.990  1.00 25.38           H
ATOM   3382  HD1 PHE B 869      34.726  21.771  42.692  1.00 29.28           H
ATOM   3383  HD2 PHE B 869      35.812  24.862  45.489  1.00 28.09           H
ATOM   3384  HE1 PHE B 869      32.440  22.686  42.603  1.00 29.25           H
ATOM   3385  HE2 PHE B 869      33.531  25.741  45.382  1.00 30.29           H
ATOM   3386  HZ  PHE B 869      31.832  24.657  43.947  1.00 27.94           H
ATOM   3387  N   ALA B 870      40.115  23.134  42.818  1.00 27.92           N
ANISOU 3387  N   ALA B 870     3301   3767   3541    120   -135    157       N
ATOM   3388  CA  ALA B 870      41.522  23.517  42.954  1.00 29.34           C
ANISOU 3388  CA  ALA B 870     3387   4100   3659     86   -165    209       C
ATOM   3389  C   ALA B 870      41.844  24.819  42.201  1.00 33.74           C
ANISOU 3389  C   ALA B 870     3932   4700   4186    -32   -191    201       C
ATOM   3390  O   ALA B 870      42.608  25.634  42.717  1.00 34.36           O
ANISOU 3390  O   ALA B 870     3998   4855   4200   -150   -239    229       O
ATOM   3391  CB  ALA B 870      42.416  22.371  42.465  1.00 30.64           C
ANISOU 3391  CB  ALA B 870     3469   4372   3799    214   -132    257       C
ATOM   3392  H   ALA B 870      39.912  22.390  42.166  1.00 27.92           H
ATOM   3393  HA  ALA B 870      41.735  23.677  44.011  1.00 29.34           H
ATOM   3394  HB1 ALA B 870      43.469  22.653  42.467  1.00 30.64           H
ATOM   3395  HB2 ALA B 870      42.317  21.486  43.091  1.00 30.64           H
ATOM   3396  HB3 ALA B 870      42.144  22.082  41.452  1.00 30.64           H
ATOM   3397  N   GLY B 871      41.222  25.004  41.023  1.00 27.99           N
ANISOU 3397  N   GLY B 871     3230   3910   3494    -15   -163    164       N
ATOM   3398  CA  GLY B 871      41.334  26.205  40.199  1.00 27.65           C
ANISOU 3398  CA  GLY B 871     3201   3877   3427   -114   -179    151       C
ATOM   3399  C   GLY B 871      40.476  27.348  40.758  1.00 31.40           C
ANISOU 3399  C   GLY B 871     3799   4250   3882   -204   -205    125       C
ATOM   3400  O   GLY B 871      40.909  28.496  40.702  1.00 31.05           O
ANISOU 3400  O   GLY B 871     3795   4233   3771   -326   -239    136       O
ATOM   3401  H   GLY B 871      40.589  24.290  40.688  1.00 27.99           H
ATOM   3402  HA3 GLY B 871      40.997  25.972  39.188  1.00 27.65           H
ATOM   3403  HA2 GLY B 871      42.374  26.522  40.127  1.00 27.65           H
ATOM   3404  N   ILE B 872      39.290  27.048  41.319  1.00 28.41           N
ANISOU 3404  N   ILE B 872     3496   3758   3542   -143   -185     95       N
ATOM   3405  CA  ILE B 872      38.376  28.033  41.913  1.00 27.60           C
ANISOU 3405  CA  ILE B 872     3523   3560   3405   -174   -192     78       C
ATOM   3406  C   ILE B 872      38.929  28.664  43.206  1.00 32.89           C
ANISOU 3406  C   ILE B 872     4250   4245   4002   -259   -234    100       C
ATOM   3407  O   ILE B 872      38.733  29.859  43.411  1.00 32.07           O
ANISOU 3407  O   ILE B 872     4278   4086   3823   -341   -253     94       O
ATOM   3408  CB  ILE B 872      36.968  27.423  42.195  1.00 30.08           C
ANISOU 3408  CB  ILE B 872     3870   3798   3760    -78   -157     62       C
ATOM   3409  CG1 ILE B 872      36.252  27.063  40.871  1.00 29.35           C
ANISOU 3409  CG1 ILE B 872     3739   3698   3713    -31   -125     44       C
ATOM   3410  CG2 ILE B 872      36.037  28.270  43.095  1.00 31.52           C
ANISOU 3410  CG2 ILE B 872     4188   3907   3883    -70   -154     60       C
ATOM   3411  CD1 ILE B 872      35.030  26.145  41.040  1.00 27.35           C
ANISOU 3411  CD1 ILE B 872     3471   3426   3493     31    -99     47       C
ATOM   3412  H   ILE B 872      38.970  26.089  41.311  1.00 28.41           H
ATOM   3413  HA  ILE B 872      38.250  28.838  41.187  1.00 27.60           H
ATOM   3414  HB  ILE B 872      37.137  26.498  42.738  1.00 30.08           H
ATOM   3415 HG13 ILE B 872      36.943  26.577  40.183  1.00 29.35           H
ATOM   3416 HG12 ILE B 872      35.946  27.978  40.364  1.00 29.35           H
ATOM   3417 HG21 ILE B 872      35.054  27.811  43.186  1.00 31.52           H
ATOM   3418 HG22 ILE B 872      36.414  28.363  44.114  1.00 31.52           H
```

```
ATOM   3419 HG23 ILE B 872      35.902  29.276  42.698  1.00 31.52           H
ATOM   3420 HD11 ILE B 872      34.848  25.574  40.131  1.00 27.35           H
ATOM   3421 HD12 ILE B 872      35.168  25.431  41.853  1.00 27.35           H
ATOM   3422 HD13 ILE B 872      34.128  26.721  41.248  1.00 27.35           H
ATOM   3423  N   LEU B 873      39.614  27.871  44.046  1.00 29.93           N
ANISOU 3423  N   LEU B 873     3803   3933   3635   -239   -248    127       N
ATOM   3424  CA  LEU B 873      40.200  28.356  45.297  1.00 30.04           C
ANISOU 3424  CA  LEU B 873     3868   3975   3572   -330   -293    151       C
ATOM   3425  C   LEU B 873      41.556  29.056  45.091  1.00 34.00           C
ANISOU 3425  C   LEU B 873     4342   4593   3984   -491   -346    186       C
ATOM   3426  O   LEU B 873      41.866  29.950  45.876  1.00 33.80           O
ANISOU 3426  O   LEU B 873     4452   4528   3862   -624   -386    187       O
ATOM   3427  CB  LEU B 873      40.288  27.198  46.315  1.00 30.14           C
ANISOU 3427  CB  LEU B 873     3811   4032   3611   -259   -293    176       C
ATOM   3428  CG  LEU B 873      38.914  26.754  46.875  1.00 35.28           C
ANISOU 3428  CG  LEU B 873     4515   4571   4317   -147   -253    151       C
ATOM   3429  CD1 LEU B 873      39.020  25.423  47.645  1.00 35.62           C
ANISOU 3429  CD1 LEU B 873     4490   4658   4386    -79   -250    179       C
ATOM   3430  CD2 LEU B 873      38.221  27.851  47.713  1.00 37.34           C
ANISOU 3430  CD2 LEU B 873     4937   4729   4522   -170   -255    131       C
ATOM   3431  H   LEU B 873      39.726  26.888  43.841  1.00 29.93           H
ATOM   3432  HA  LEU B 873      39.538  29.115  45.715  1.00 30.04           H
ATOM   3433  HB3 LEU B 873      40.930  27.479  47.152  1.00 30.14           H
ATOM   3434  HB2 LEU B 873      40.779  26.354  45.832  1.00 30.14           H
ATOM   3435  HG  LEU B 873      38.270  26.551  46.018  1.00 35.28           H
ATOM   3436 HD11 LEU B 873      38.147  24.800  47.450  1.00 35.62           H
ATOM   3437 HD12 LEU B 873      39.895  24.848  47.345  1.00 35.62           H
ATOM   3438 HD13 LEU B 873      39.084  25.572  48.723  1.00 35.62           H
ATOM   3439 HD21 LEU B 873      37.889  27.487  48.686  1.00 37.34           H
ATOM   3440 HD22 LEU B 873      38.876  28.702  47.898  1.00 37.34           H
ATOM   3441 HD23 LEU B 873      37.338  28.228  47.197  1.00 37.34           H
ATOM   3442  N   SER B 874      42.319  28.692  44.043  1.00 30.78           N
ANISOU 3442  N   SER B 874     3779   4325   3591   -487   -344    218       N
ATOM   3443  CA  SER B 874      43.609  29.319  43.732  1.00 30.60           C
ANISOU 3443  CA  SER B 874     3700   4458   3470   -654   -394    268       C
ATOM   3444  C   SER B 874      43.501  30.578  42.848  1.00 34.18           C
ANISOU 3444  C   SER B 874     4280   4833   3873   -780   -404    242       C
ATOM   3445  O   SER B 874      44.281  31.504  43.069  1.00 33.88           O
ANISOU 3445  O   SER B 874     4331   4824   3719   -975   -459    265       O
ATOM   3446  CB  SER B 874      44.597  28.269  43.182  1.00 35.06           C
ANISOU 3446  CB  SER B 874     4045   5232   4044   -584   -380    328       C
ATOM   3447  OG  SER B 874      44.282  27.838  41.873  1.00 39.53           O
ANISOU 3447  OG  SER B 874     4578   5759   4683   -473   -326    300       O
ATOM   3448  H   SER B 874      42.014  27.952  43.427  1.00 30.78           H
ATOM   3449  HA  SER B 874      44.050  29.660  44.669  1.00 30.60           H
ATOM   3450  HB3 SER B 874      44.636  27.403  43.843  1.00 35.06           H
ATOM   3451  HB2 SER B 874      45.605  28.685  43.162  1.00 35.06           H
ATOM   3452  HG  SER B 874      43.637  27.126  41.942  1.00 39.53           H
ATOM   3453  N   GLN B 875      42.556  30.611  41.889  1.00 32.01           N
ANISOU 3453  N   GLN B 875     4034   4455   3672   -680   -355    198       N
ATOM   3454  CA  GLN B 875      42.422  31.692  40.897  1.00 32.27           C
ANISOU 3454  CA  GLN B 875     4186   4415   3661   -769   -355    177       C
ATOM   3455  C   GLN B 875      41.118  32.498  41.041  1.00 35.43           C
ANISOU 3455  C   GLN B 875     4812   4600   4050   -724   -330    125       C
ATOM   3456  O   GLN B 875      41.009  33.552  40.413  1.00 36.31           O
ANISOU 3456  O   GLN B 875     5073   4632   4090   -807   -334    114       O
```

ATOM 3457 CB GLN B 875 42.497 31.105 39.465 1.00 33.70 C
ANISOU 3457 CB GLN B 875 4238 4659 3909 -691 -317 175 C
ATOM 3458 CG GLN B 875 43.741 30.252 39.142 1.00 54.61 C
ANISOU 3458 CG GLN B 875 6672 7535 6541 -704 -327 238 C
ATOM 3459 CD GLN B 875 45.067 30.976 39.386 1.00 88.21 C
ANISOU 3459 CD GLN B 875 10905 11942 10667 -926 -389 300 C
ATOM 3460 NE2 GLN B 875 46.001 30.315 40.072 1.00 83.69 N
ANISOU 3460 NE2 GLN B 875 10173 11580 10047 -953 -418 372 N
ATOM 3461 OE1 GLN B 875 45.252 32.108 38.945 1.00 87.75 O
ANISOU 3461 OE1 GLN B 875 10978 11825 10536 -1081 -414 292 O
ATOM 3462 H GLN B 875 41.954 29.810 41.750 1.00 32.01 H
ATOM 3463 HA GLN B 875 43.231 32.415 41.012 1.00 32.27 H
ATOM 3464 HB3 GLN B 875 42.462 31.929 38.753 1.00 33.70 H
ATOM 3465 HB2 GLN B 875 41.606 30.512 39.257 1.00 33.70 H
ATOM 3466 HG3 GLN B 875 43.708 29.945 38.096 1.00 54.61 H
ATOM 3467 HG2 GLN B 875 43.710 29.332 39.725 1.00 54.61 H
ATOM 3468 HE22 GLN B 875 46.895 30.748 40.255 1.00 83.69 H
ATOM 3469 HE21 GLN B 875 45.813 29.388 40.428 1.00 83.69 H
ATOM 3470 N GLY B 876 40.158 32.015 41.848 1.00 29.74 N
ANISOU 3470 N GLY B 876 4113 3798 3386 -584 -299 101 N
ATOM 3471 CA GLY B 876 38.831 32.616 41.997 1.00 28.87 C
ANISOU 3471 CA GLY B 876 4177 3529 3263 -489 -261 69 C
ATOM 3472 C GLY B 876 37.896 32.120 40.884 1.00 30.66 C
ANISOU 3472 C GLY B 876 4345 3738 3566 -363 -211 50 C
ATOM 3473 O GLY B 876 38.280 31.306 40.040 1.00 28.92 O
ANISOU 3473 O GLY B 876 3974 3604 3410 -357 -206 52 O
ATOM 3474 H GLY B 876 40.309 31.139 42.329 1.00 29.74 H
ATOM 3475 HA3 GLY B 876 38.892 33.705 41.967 1.00 28.87 H
ATOM 3476 HA2 GLY B 876 38.414 32.355 42.969 1.00 28.87 H
ATOM 3477 N LEU B 877 36.658 32.642 40.876 1.00 27.03 N
ANISOU 3477 N LEU B 877 4006 3179 3083 -254 -170 37 N
ATOM 3478 CA LEU B 877 35.724 32.485 39.760 1.00 26.46 C
ANISOU 3478 CA LEU B 877 3878 3115 3061 -148 -128 30 C
ATOM 3479 C LEU B 877 36.160 33.403 38.608 1.00 30.59 C
ANISOU 3479 C LEU B 877 4473 3614 3535 -219 -133 25 C
ATOM 3480 O LEU B 877 36.264 34.616 38.802 1.00 31.00 O
ANISOU 3480 O LEU B 877 4721 3578 3480 -287 -144 28 O
ATOM 3481 CB LEU B 877 34.280 32.799 40.213 1.00 26.17 C
ANISOU 3481 CB LEU B 877 3913 3033 2998 6 -80 40 C
ATOM 3482 CG LEU B 877 33.673 31.785 41.210 1.00 30.29 C
ANISOU 3482 CG LEU B 877 4337 3600 3571 80 -68 52 C
ATOM 3483 CD1 LEU B 877 32.235 32.185 41.595 1.00 29.69 C
ANISOU 3483 CD1 LEU B 877 4325 3516 3439 233 -17 80 C
ATOM 3484 CD2 LEU B 877 33.717 30.341 40.680 1.00 32.13 C
ANISOU 3484 CD2 LEU B 877 4375 3923 3911 73 -72 48 C
ATOM 3485 H LEU B 877 36.381 33.291 41.598 1.00 27.03 H
ATOM 3486 HA LEU B 877 35.765 31.454 39.404 1.00 26.46 H
ATOM 3487 HB3 LEU B 877 33.640 32.830 39.330 1.00 26.17 H
ATOM 3488 HB2 LEU B 877 34.243 33.800 40.645 1.00 26.17 H
ATOM 3489 HG LEU B 877 34.269 31.816 42.123 1.00 30.29 H
ATOM 3490 HD11 LEU B 877 32.102 32.194 42.675 1.00 29.69 H
ATOM 3491 HD12 LEU B 877 31.984 33.181 41.238 1.00 29.69 H
ATOM 3492 HD13 LEU B 877 31.491 31.504 41.182 1.00 29.69 H
ATOM 3493 HD21 LEU B 877 32.987 29.705 41.180 1.00 32.13 H
ATOM 3494 HD22 LEU B 877 33.519 30.298 39.608 1.00 32.13 H
ATOM 3495 HD23 LEU B 877 34.694 29.895 40.853 1.00 32.13 H

```
ATOM   3496  N   ARG B 878      36.434  32.788  37.448  1.00 25.98           N
ANISOU 3496  N   ARG B 878     3751   3101   3018   -216   -127     20       N
ATOM   3497  CA  ARG B 878      36.948  33.455  36.254  1.00 25.89           C
ANISOU 3497  CA  ARG B 878     3776   3088   2972   -286   -131     18       C
ATOM   3498  C   ARG B 878      35.892  33.482  35.143  1.00 28.83           C
ANISOU 3498  C   ARG B 878     4126   3458   3370   -179    -91     12       C
ATOM   3499  O   ARG B 878      34.981  32.654  35.116  1.00 28.34           O
ANISOU 3499  O   ARG B 878     3958   3440   3372    -82    -70     10       O
ATOM   3500  CB  ARG B 878      38.214  32.722  35.759  1.00 25.74           C
ANISOU 3500  CB  ARG B 878     3604   3187   2988   -393   -161     28       C
ATOM   3501  CG  ARG B 878      39.380  32.734  36.768  1.00 31.26           C
ANISOU 3501  CG  ARG B 878     4310   3939   3630   -533   -210     54       C
ATOM   3502  CD  ARG B 878      40.699  32.187  36.192  1.00 40.43           C
ANISOU 3502  CD  ARG B 878     5279   5267   4817   -591   -227     82       C
ATOM   3503  NE  ARG B 878      40.686  30.724  35.987  1.00 44.54           N
ANISOU 3503  NE  ARG B 878     5646   5845   5432   -459   -201     78       N
ATOM   3504  CZ  ARG B 878      41.625  29.988  35.363  1.00 59.94           C
ANISOU 3504  CZ  ARG B 878     7446   7914   7413   -422   -188     96       C
ATOM   3505  NH1 ARG B 878      41.455  28.665  35.264  1.00 50.21           N
ANISOU 3505  NH1 ARG B 878     6136   6694   6246   -297   -159     89       N
ATOM   3506  NH2 ARG B 878      42.732  30.531  34.834  1.00 45.48          N1+
ANISOU 3506  NH2 ARG B 878     5559   6189   5534   -507   -198    126       N1+
ATOM   3507  H   ARG B 878      36.305  31.790  37.372  1.00 25.98           H
ATOM   3508  HA  ARG B 878      37.220  34.488  36.484  1.00 25.89           H
ATOM   3509  HB3 ARG B 878      38.555  33.197  34.837  1.00 25.74           H
ATOM   3510  HB2 ARG B 878      37.966  31.693  35.492  1.00 25.74           H
ATOM   3511  HG3 ARG B 878      39.081  32.097  37.601  1.00 31.26           H
ATOM   3512  HG2 ARG B 878      39.540  33.721  37.203  1.00 31.26           H
ATOM   3513  HD3 ARG B 878      41.563  32.532  36.757  1.00 40.43           H
ATOM   3514  HD2 ARG B 878      40.814  32.599  35.189  1.00 40.43           H
ATOM   3515  HE  ARG B 878      39.883  30.238  36.363  1.00 44.54           H
ATOM   3516 HH12 ARG B 878      42.141  28.085  34.801  1.00 50.21           H
ATOM   3517 HH11 ARG B 878      40.631  28.219  35.656  1.00 50.21           H
ATOM   3518 HH22 ARG B 878      43.420  29.952  34.377  1.00 45.48           H
ATOM   3519 HH21 ARG B 878      42.886  31.526  34.904  1.00 45.48           H
ATOM   3520  N   ILE B 879      36.083  34.439  34.227  1.00 26.45           N
ANISOU 3520  N   ILE B 879     3931   3114   3006   -214    -86     14       N
ATOM   3521  CA  ILE B 879      35.293  34.627  33.015  1.00 27.06           C
ANISOU 3521  CA  ILE B 879     3993   3201   3090   -127    -53     14       C
ATOM   3522  C   ILE B 879      35.999  33.928  31.833  1.00 29.08           C
ANISOU 3522  C   ILE B 879     4082   3550   3416   -188    -65      2       C
ATOM   3523  O   ILE B 879      37.231  33.917  31.785  1.00 26.94           O
ANISOU 3523  O   ILE B 879     3771   3320   3144   -305    -93      5       O
ATOM   3524  CB  ILE B 879      35.142  36.153  32.730  1.00 30.64           C
ANISOU 3524  CB  ILE B 879     4683   3540   3420   -127    -38     26       C
ATOM   3525  CG1 ILE B 879      34.299  36.814  33.846  1.00 31.24           C
ANISOU 3525  CG1 ILE B 879     4917   3535   3419     25      1     45       C
ATOM   3526  CG2 ILE B 879      34.553  36.494  31.345  1.00 31.97           C
ANISOU 3526  CG2 ILE B 879     4844   3724   3579   -105    -19     29       C
ATOM   3527  CD1 ILE B 879      34.302  38.346  33.831  1.00 45.70           C
ANISOU 3527  CD1 ILE B 879     7047   5210   5107    -11      5     50       C
ATOM   3528  H   ILE B 879      36.872  35.061  34.326  1.00 26.45           H
ATOM   3529  HA  ILE B 879      34.300  34.191  33.143  1.00 27.06           H
ATOM   3530  HB  ILE B 879      36.137  36.600  32.769  1.00 30.64           H
ATOM   3531 HG13 ILE B 879      34.652  36.500  34.828  1.00 31.24           H
ATOM   3532 HG12 ILE B 879      33.273  36.454  33.777  1.00 31.24           H
```

```
ATOM   3533 HG21 ILE B 879      34.346  37.558  31.237  1.00 31.97           H
ATOM   3534 HG22 ILE B 879      35.235  36.237  30.538  1.00 31.97           H
ATOM   3535 HG23 ILE B 879      33.621  35.954  31.183  1.00 31.97           H
ATOM   3536 HD11 ILE B 879      34.078  38.740  34.823  1.00 45.70           H
ATOM   3537 HD12 ILE B 879      35.269  38.748  33.527  1.00 45.70           H
ATOM   3538 HD13 ILE B 879      33.547  38.730  33.145  1.00 45.70           H
ATOM   3539  N   ALA B 880      35.202  33.371  30.902  1.00 25.90           N
ANISOU 3539  N   ALA B 880     3587   3196   3059   -109    -42     -4       N
ATOM   3540  CA  ALA B 880      35.655  32.718  29.666  1.00 25.63           C
ANISOU 3540  CA  ALA B 880     3433   3232   3073   -149    -45    -18       C
ATOM   3541  C   ALA B 880      36.509  33.671  28.793  1.00 30.09           C
ANISOU 3541  C   ALA B 880     4065   3786   3581   -244    -54    -10       C
ATOM   3542  O   ALA B 880      36.091  34.813  28.594  1.00 29.75           O
ANISOU 3542  O   ALA B 880     4180   3664   3460   -247    -48      2       O
ATOM   3543  CB  ALA B 880      34.407  32.266  28.894  1.00 26.28           C
ANISOU 3543  CB  ALA B 880     3456   3352   3178    -70    -24    -23       C
ATOM   3544  H   ALA B 880      34.201  33.417  31.026  1.00 25.90           H
ATOM   3545  HA  ALA B 880      36.212  31.834  29.971  1.00 25.63           H
ATOM   3546  HB1 ALA B 880      34.678  31.803  27.944  1.00 26.28           H
ATOM   3547  HB2 ALA B 880      33.839  31.529  29.462  1.00 26.28           H
ATOM   3548  HB3 ALA B 880      33.742  33.102  28.680  1.00 26.28           H
ATOM   3549  N   PRO B 881      37.705  33.224  28.340  1.00 26.60           N
ANISOU 3549  N   PRO B 881     3521   3426   3159   -320    -65     -9       N
ATOM   3550  CA  PRO B 881      38.727  34.123  27.762  1.00 26.19           C
ANISOU 3550  CA  PRO B 881     3521   3392   3039   -439    -79     12       C
ATOM   3551  C   PRO B 881      38.345  34.738  26.391  1.00 30.59           C
ANISOU 3551  C   PRO B 881     4141   3919   3564   -423    -59     10       C
ATOM   3552  O   PRO B 881      37.325  34.344  25.824  1.00 28.75           O
ANISOU 3552  O   PRO B 881     3873   3680   3369   -314    -35     -8       O
ATOM   3553  CB  PRO B 881      39.974  33.223  27.697  1.00 27.93           C
ANISOU 3553  CB  PRO B 881     3576   3752   3285   -486    -87     29       C
ATOM   3554  CG  PRO B 881      39.425  31.820  27.512  1.00 31.84           C
ANISOU 3554  CG  PRO B 881     3969   4266   3862   -359    -61      2       C
ATOM   3555  CD  PRO B 881      38.179  31.839  28.388  1.00 27.46           C
ANISOU 3555  CD  PRO B 881     3479   3620   3333   -293    -62    -16       C
ATOM   3556  HA  PRO B 881      38.895  34.935  28.470  1.00 26.19           H
ATOM   3557  HB3 PRO B 881      40.513  33.284  28.643  1.00 27.93           H
ATOM   3558  HB2 PRO B 881      40.678  33.485  26.912  1.00 27.93           H
ATOM   3559  HG3 PRO B 881      40.134  31.038  27.783  1.00 31.84           H
ATOM   3560  HG2 PRO B 881      39.141  31.674  26.469  1.00 31.84           H
ATOM   3561  HD2 PRO B 881      37.434  31.125  28.034  1.00 27.46           H
ATOM   3562  HD3 PRO B 881      38.442  31.586  29.417  1.00 27.46           H
ATOM   3563  N   PRO B 882      39.164  35.687  25.866  1.00 29.63           N
ANISOU 3563  N   PRO B 882     4108   3789   3362   -543    -71     32       N
ATOM   3564  CA  PRO B 882      38.886  36.377  24.586  1.00 30.40           C
ANISOU 3564  CA  PRO B 882     4274   3852   3423   -520    -49     32       C
ATOM   3565  C   PRO B 882      38.683  35.485  23.347  1.00 36.42           C
ANISOU 3565  C   PRO B 882     4881   4704   4253   -449    -27     16       C
ATOM   3566  O   PRO B 882      37.883  35.850  22.485  1.00 36.67           O
ANISOU 3566  O   PRO B 882     4954   4706   4275   -376     -6      9       O
ATOM   3567  CB  PRO B 882      40.082  37.325  24.402  1.00 32.03           C
ANISOU 3567  CB  PRO B 882     4584   4057   3529   -703    -73     65       C
ATOM   3568  CG  PRO B 882      40.559  37.602  25.815  1.00 36.33           C
ANISOU 3568  CG  PRO B 882     5200   4576   4027   -809   -108     80       C
ATOM   3569  CD  PRO B 882      40.349  36.263  26.508  1.00 31.78           C
ANISOU 3569  CD  PRO B 882     4434   4086   4086   -723   -108     65       C
```

```
ATOM   3570  HA  PRO B 882      37.987  36.975  24.740  1.00 30.40           H
ATOM   3571  HB3 PRO B 882      39.819  38.239  23.867  1.00 32.03           H
ATOM   3572  HB2 PRO B 882      40.882  36.834  23.844  1.00 32.03           H
ATOM   3573  HG3 PRO B 882      39.918  38.356  26.275  1.00 36.33           H
ATOM   3574  HG2 PRO B 882      41.588  37.958  25.864  1.00 36.33           H
ATOM   3575  HD2 PRO B 882      41.205  35.613  26.321  1.00 31.78           H
ATOM   3576  HD3 PRO B 882      40.254  36.403  27.584  1.00 31.78           H
ATOM   3577  N   GLU B 883      39.372  34.332  23.303  1.00 33.48           N
ANISOU 3577  N   GLU B 883     4345   4442   3934   -458    -29     13       N
ATOM   3578  CA  GLU B 883      39.273  33.334  22.237  1.00 32.97           C
ANISOU 3578  CA  GLU B 883     4164   4448   3917   -388     -4     -6       C
ATOM   3579  C   GLU B 883      37.919  32.601  22.185  1.00 33.21           C
ANISOU 3579  C   GLU B 883     4186   4434   4000   -275      7    -40       C
ATOM   3580  O   GLU B 883      37.561  32.118  21.113  1.00 32.58           O
ANISOU 3580  O   GLU B 883     4069   4380   3932   -234     23    -59       O
ATOM   3581  CB  GLU B 883      40.421  32.301  22.361  1.00 34.63           C
ANISOU 3581  CB  GLU B 883     4236   4775   4147   -390      1      8       C
ATOM   3582  CG  GLU B 883      41.853  32.884  22.242  1.00 48.07           C
ANISOU 3582  CG  GLU B 883     5888   6594   5782   -509    -11     61       C
ATOM   3583  CD  GLU B 883      42.508  33.431  23.525  1.00 67.19           C
ANISOU 3583  CD  GLU B 883     8347   9025   8156   -632    -51     96       C
ATOM   3584  OE1 GLU B 883      43.554  34.098  23.377  1.00 62.89           O
ANISOU 3584  OE1 GLU B 883     7778   8584   7532   -776    -71    149       O
ATOM   3585  OE2 GLU B 883      41.990  33.176  24.635  1.00 51.94           O1-
ANISOU 3585  OE2 GLU B 883     6474   7007   6256   -600    -64     76       O1-
ATOM   3586  H   GLU B 883      40.027  34.113  24.042  1.00 33.48           H
ATOM   3587  HA  GLU B 883      39.390  33.855  21.284  1.00 32.97           H
ATOM   3588  HB3 GLU B 883      40.302  31.561  21.569  1.00 34.63           H
ATOM   3589  HB2 GLU B 883      40.309  31.725  23.281  1.00 34.63           H
ATOM   3590  HG3 GLU B 883      41.867  33.657  21.472  1.00 48.07           H
ATOM   3591  HG2 GLU B 883      42.509  32.094  21.876  1.00 48.07           H
ATOM   3592  N   ALA B 884      37.213  32.501  23.327  1.00 26.60           N
ANISOU 3592  N   ALA B 884     3378   3547   3184   -240     -3    -44       N
ATOM   3593  CA  ALA B 884      35.972  31.736  23.487  1.00 26.27           C
ANISOU 3593  CA  ALA B 884     3313   3493   3175   -161      2    -61       C
ATOM   3594  C   ALA B 884      34.776  32.372  22.741  1.00 29.46           C
ANISOU 3594  C   ALA B 884     3757   3895   3542   -112     11    -52       C
ATOM   3595  O   ALA B 884      34.666  33.600  22.743  1.00 28.93           O
ANISOU 3595  O   ALA B 884     3787   3786   3417   -105     17    -29       O
ATOM   3596  CB  ALA B 884      35.671  31.629  24.990  1.00 26.80           C
ANISOU 3596  CB  ALA B 884     3400   3525   3258   -140     -8    -54       C
ATOM   3597  H   ALA B 884      37.564  32.947  24.163  1.00 26.60           H
ATOM   3598  HA  ALA B 884      36.182  30.739  23.106  1.00 26.27           H
ATOM   3599  HB1 ALA B 884      34.854  30.933  25.176  1.00 26.80           H
ATOM   3600  HB2 ALA B 884      36.537  31.263  25.543  1.00 26.80           H
ATOM   3601  HB3 ALA B 884      35.389  32.590  25.420  1.00 26.80           H
ATOM   3602  N   PRO B 885      33.901  31.542  22.122  1.00 26.77           N
ANISOU 3602  N   PRO B 885     3356   3602   3212    -81     13    -62       N
ATOM   3603  CA  PRO B 885      32.711  32.033  21.398  1.00 27.00           C
ANISOU 3603  CA  PRO B 885     3399   3671   3190    -30     19    -36       C
ATOM   3604  C   PRO B 885      31.651  32.618  22.350  1.00 33.57           C
ANISOU 3604  C   PRO B 885     4257   4513   3985     53     24      5       C
ATOM   3605  O   PRO B 885      31.133  31.888  23.192  1.00 33.69           O
ANISOU 3605  O   PRO B 885     4217   4561   4023     58     15      9       O
ATOM   3606  CB  PRO B 885      32.198  30.777  20.669  1.00 27.97           C
ANISOU 3606  CB  PRO B 885     3445   3860   3321    -59      9    -56       C
```

ATOM   3607 CG PRO B 885   32.625 29.618 21.552 1.00 32.26   C
ANISOU 3607 CG PRO B 885   3964   4380   3915   -96   0   -84   C
ATOM   3608 CD PRO B 885   33.976 30.078 22.085 1.00 28.47   C
ANISOU 3608 CD PRO B 885   3510   3841   3467   -100   7   -90   C
ATOM   3609 HA PRO B 885   32.992 32.789 20.663 1.00 27.00   H
ATOM   3610 HB3 PRO B 885   32.688 30.697 19.699 1.00 27.97   H
ATOM   3611 HB2 PRO B 885   31.122 30.780 20.487 1.00 27.97   H
ATOM   3612 HG3 PRO B 885   32.662 28.665 21.031 1.00 32.26   H
ATOM   3613 HG2 PRO B 885   31.923 29.508 22.380 1.00 32.26   H
ATOM   3614 HD2 PRO B 885   34.173 29.633 23.061 1.00 28.47   H
ATOM   3615 HD3 PRO B 885   34.773 29.785 21.400 1.00 28.47   H
ATOM   3616 N   VAL B 886   31.345 33.919 22.195 1.00 30.55   N
ANISOU 3616 N   VAL B 886   3973   4104   3531   128   45   41   N
ATOM   3617 CA VAL B 886   30.351 34.639 23.003 1.00 29.94   C
ANISOU 3617 CA VAL B 886   3947   4039   3388   254   67   92   C
ATOM   3618 C   VAL B 886   28.908 34.091 22.865 1.00 31.20   C
ANISOU 3618 C   VAL B 886   3968   4362   3525   314   64   135   C
ATOM   3619 O   VAL B 886   28.150 34.160 23.832 1.00 28.86   O
ANISOU 3619 O   VAL B 886   3653   4113   3201   391   76   174   O
ATOM   3620 CB VAL B 886   30.373 36.166 22.692 1.00 34.21   C
ANISOU 3620 CB VAL B 886   4665   4503   3830   344   99   125   C
ATOM   3621 CG1 VAL B 886   29.837 36.538 21.293 1.00 34.58   C
ANISOU 3621 CG1 VAL B 886   4685   4627   3828   392   111   151   C
ATOM   3622 CG2 VAL B 886   29.662 37.009 23.770 1.00 34.06   C
ANISOU 3622 CG2 VAL B 886   4752   4461   3728   497   134   175   C
ATOM   3623 H   VAL B 886   31.808 34.455 21.476 1.00 30.55   H
ATOM   3624 HA VAL B 886   30.643 34.506 24.045 1.00 29.94   H
ATOM   3625 HB VAL B 886   31.420 36.469 22.711 1.00 34.21   H
ATOM   3626 HG11 VAL B 886   30.006 37.594 21.082 1.00 34.58   H
ATOM   3627 HG12 VAL B 886   30.335 35.966 20.510 1.00 34.58   H
ATOM   3628 HG13 VAL B 886   28.765 36.363 21.204 1.00 34.58   H
ATOM   3629 HG21 VAL B 886   29.784 38.076 23.577 1.00 34.06   H
ATOM   3630 HG22 VAL B 886   28.592 36.810 23.806 1.00 34.06   H
ATOM   3631 HG23 VAL B 886   30.070 36.810 24.761 1.00 34.06   H
ATOM   3632 N   THR B 887   28.582 33.510 21.695 1.00 27.40   N
ANISOU 3632 N   THR B 887   3390   3979   3043   263   47   132   N
ATOM   3633 CA THR B 887   27.305 32.845 21.413 1.00 27.03   C
ANISOU 3633 CA THR B 887   3204   4115   2953   269   33   180   C
ATOM   3634 C   THR B 887   27.173 31.445 22.058 1.00 31.33   C
ANISOU 3634 C   THR B 887   3669   4684   3551   160   2   157   C
ATOM   3635 O   THR B 887   26.055 30.936 22.130 1.00 31.36   O
ANISOU 3635 O   THR B 887   3563   4846   3507   140   -14   207   O
ATOM   3636 CB THR B 887   27.051 32.712 19.887 1.00 34.53   C
ANISOU 3636 CB THR B 887   4098   5161   3862   233   19   189   C
ATOM   3637 CG2 THR B 887   27.139 34.052 19.137 1.00 34.53   C
ANISOU 3637 CG2 THR B 887   4185   5142   3795   355   52   222   C
ATOM   3638 OG1 THR B 887   27.901 31.760 19.270 1.00 35.72   O
ANISOU 3638 OG1 THR B 887   4253   5238   4081   91   -6   113   O
ATOM   3639 H   THR B 887   29.268 33.481 20.953 1.00 27.40   H
ATOM   3640 HA THR B 887   26.512 33.461 21.829 1.00 27.03   H
ATOM   3641 HB THR B 887   26.034 32.341 19.747 1.00 34.53   H
ATOM   3642 HG21 THR B 887   26.909 33.923 18.078 1.00 34.53   H
ATOM   3643 HG22 THR B 887   26.426 34.770 19.543 1.00 34.53   H
ATOM   3644 HG23 THR B 887   28.130 34.499 19.206 1.00 34.53   H
ATOM   3645 HG1 THR B 887   28.792 32.121 19.233 1.00 35.72   H
ATOM   3646 N   GLY B 888   28.284 30.877 22.561 1.00 28.64   N

ANISOU 3646 N GLY B 888 3385 4204 3295 90 -4 92 N
ATOM 3647 CA GLY B 888 28.312 29.624 23.319 1.00 28.13 C
ANISOU 3647 CA GLY B 888 3284 4128 3277 3 -25 67 C
ATOM 3648 C GLY B 888 27.875 29.810 24.787 1.00 31.29 C
ANISOU 3648 C GLY B 888 3670 4548 3670 64 -16 106 C
ATOM 3649 O GLY B 888 27.889 28.835 25.534 1.00 30.45 O
ANISOU 3649 O GLY B 888 3536 4441 3594 -4 -33 95 O
ATOM 3650 H GLY B 888 29.165 31.367 22.478 1.00 28.64 H
ATOM 3651 HA3 GLY B 888 29.330 29.234 23.305 1.00 28.13 H
ATOM 3652 HA2 GLY B 888 27.679 28.874 22.841 1.00 28.13 H
ATOM 3653 N TYR B 889 27.511 31.039 25.200 1.00 27.40 N
ANISOU 3653 N TYR B 889 3219 4066 3125 202 15 153 N
ATOM 3654 CA TYR B 889 27.073 31.403 26.549 1.00 26.43 C
ANISOU 3654 CA TYR B 889 3115 3950 2977 296 36 193 C
ATOM 3655 C TYR B 889 25.818 32.276 26.415 1.00 30.54 C
ANISOU 3655 C TYR B 889 3602 4620 3383 455 70 285 C
ATOM 3656 O TYR B 889 25.858 33.252 25.666 1.00 30.21 O
ANISOU 3656 O TYR B 889 3640 4553 3286 553 96 302 O
ATOM 3657 CB TYR B 889 28.202 32.176 27.268 1.00 26.52 C
ANISOU 3657 CB TYR B 889 3278 3780 3020 322 50 153 C
ATOM 3658 CG TYR B 889 29.556 31.489 27.245 1.00 27.67 C
ANISOU 3658 CG TYR B 889 3437 3819 3256 190 23 81 C
ATOM 3659 CD1 TYR B 889 30.534 31.893 26.313 1.00 29.16 C
ANISOU 3659 CD1 TYR B 889 3661 3958 3462 137 18 46 C
ATOM 3660 CD2 TYR B 889 29.825 30.416 28.117 1.00 27.73 C
ANISOU 3660 CD2 TYR B 889 3420 3793 3321 135 8 58 C
ATOM 3661 CE1 TYR B 889 31.773 31.227 26.249 1.00 28.69 C
ANISOU 3661 CE1 TYR B 889 3593 3841 3466 45 2 -2 C
ATOM 3662 CE2 TYR B 889 31.062 29.746 28.047 1.00 28.01 C
ANISOU 3662 CE2 TYR B 889 3461 3760 3422 49 -9 7 C
ATOM 3663 CZ TYR B 889 32.038 30.150 27.115 1.00 33.06 C
ANISOU 3663 CZ TYR B 889 4120 4373 4070 12 -9 -19 C
ATOM 3664 OH TYR B 889 33.239 29.505 27.047 1.00 26.90 O
ANISOU 3664 OH TYR B 889 3329 3558 3335 -44 -16 -52 O
ATOM 3665 H TYR B 889 27.524 31.799 24.535 1.00 27.40 H
ATOM 3666 HA TYR B 889 26.823 30.511 27.127 1.00 26.43 H
ATOM 3667 HB3 TYR B 889 27.922 32.359 28.305 1.00 26.52 H
ATOM 3668 HB2 TYR B 889 28.325 33.158 26.810 1.00 26.52 H
ATOM 3669 HD1 TYR B 889 30.330 32.707 25.635 1.00 29.16 H
ATOM 3670 HD2 TYR B 889 29.078 30.089 28.825 1.00 27.73 H
ATOM 3671 HE1 TYR B 889 32.514 31.541 25.529 1.00 28.69 H
ATOM 3672 HE2 TYR B 889 31.249 28.913 28.705 1.00 28.01 H
ATOM 3673 HH TYR B 889 33.372 28.862 27.753 1.00 26.90 H
ATOM 3674 N MET B 890 24.734 31.914 27.129 1.00 26.90 N
ANISOU 3674 N MET B 890 3020 4329 2873 487 71 354 N
ATOM 3675 CA MET B 890 23.389 32.499 27.000 1.00 27.18 C
ANISOU 3675 CA MET B 890 2973 4576 2779 653 107 467 C
ATOM 3676 C MET B 890 23.328 34.033 27.120 1.00 30.10 C
ANISOU 3676 C MET B 890 3503 4869 3064 885 171 501 C
ATOM 3677 O MET B 890 22.730 34.675 26.258 1.00 29.70 O
ANISOU 3677 O MET B 890 3430 4946 2909 1025 202 574 O
ATOM 3678 CB MET B 890 22.418 31.809 27.987 1.00 30.04 C
ANISOU 3678 CB MET B 890 3200 5112 3103 654 105 536 C
ATOM 3679 CG MET B 890 20.960 32.298 27.880 1.00 34.41 C
ANISOU 3679 CG MET B 890 3607 5961 3505 808 137 673 C
ATOM 3680 SD MET B 890 19.775 31.449 28.952 1.00 39.38 S

ANISOU 3680 SD MET B 890   4034   6860   4070   770   129   774   S
ATOM   3681 CE MET B 890   18.266 32.310 28.437 1.00 36.56   C
ANISOU 3681 CE MET B 890   3491   6889   3509   985   172   952   C
ATOM   3682 H   MET B 890   24.800 31.111 27.740 1.00 26.90   H
ATOM   3683 HA MET B 890   23.053 32.251 25.992 1.00 27.18   H
ATOM   3684 HB3 MET B 890   22.766 31.960 29.010 1.00 30.04   H
ATOM   3685 HB2 MET B 890   22.440 30.732 27.820 1.00 30.04   H
ATOM   3686 HG3 MET B 890   20.621 32.182 26.852 1.00 34.41   H
ATOM   3687 HG2 MET B 890   20.891 33.360 28.115 1.00 34.41   H
ATOM   3688 HE1 MET B 890   17.416 31.965 29.022 1.00 36.56   H
ATOM   3689 HE2 MET B 890   18.368 33.385 28.582 1.00 36.56   H
ATOM   3690 HE3 MET B 890   18.060 32.123 27.383 1.00 36.56   H
ATOM   3691 N   PHE B 891   23.962 34.575 28.169 1.00 25.91   N
ANISOU 3691 N   PHE B 891   3150   4133   2561   925   192   456   N
ATOM   3692 CA PHE B 891   24.036 36.008 28.455 1.00 25.21   C
ANISOU 3692 CA PHE B 891   3283   3923   2375   1122   252   479   C
ATOM   3693 C   PHE B 891   25.439 36.590 28.190 1.00 27.74   C
ANISOU 3693 C   PHE B 891   3814   3978   2750   1018   237   386   C
ATOM   3694 O   PHE B 891   25.757 37.659 28.707 1.00 27.65   O
ANISOU 3694 O   PHE B 891   4034   3803   2668   1109   272   382   O
ATOM   3695 CB PHE B 891   23.524 36.274 29.887 1.00 27.05   C
ANISOU 3695 CB PHE B 891   3571   4180   2527   1293   301   536   C
ATOM   3696 CG PHE B 891   22.036 36.024 30.061 1.00 28.30   C
ANISOU 3696 CG PHE B 891   3512   4642   2598   1416   325   653   C
ATOM   3697 CD1 PHE B 891   21.104 36.902 29.471 1.00 31.66   C
ANISOU 3697 CD1 PHE B 891   3897   5252   2882   1626   376   758   C
ATOM   3698 CD2 PHE B 891   21.566 34.863 30.710 1.00 29.43   C
ANISOU 3698 CD2 PHE B 891   3488   4907   2786   1322   299   669   C
ATOM   3699 CE1 PHE B 891   19.744 36.654 29.593 1.00 32.62   C
ANISOU 3699 CE1 PHE B 891   3785   5706   2903   1733   396   886   C
ATOM   3700 CE2 PHE B 891   20.201 34.634 30.819 1.00 32.72   C
ANISOU 3700 CE2 PHE B 891   3691   5639   3104   1413   318   791   C
ATOM   3701 CZ PHE B 891   19.293 35.529 30.270 1.00 30.96   C
ANISOU 3701 CZ PHE B 891   3402   5625   2735   1615   365   903   C
ATOM   3702 H   PHE B 891   24.444 33.972 28.825 1.00 25.91   H
ATOM   3703 HA PHE B 891   23.372 36.547 27.783 1.00 25.21   H
ATOM   3704 HB3 PHE B 891   23.699 37.313 30.165 1.00 27.05   H
ATOM   3705 HB2 PHE B 891   24.080 35.672 30.608 1.00 27.05   H
ATOM   3706 HD1 PHE B 891   21.448 37.770 28.929 1.00 31.66   H
ATOM   3707 HD2 PHE B 891   22.263 34.157 31.134 1.00 29.43   H
ATOM   3708 HE1 PHE B 891   19.034 37.337 29.155 1.00 32.62   H
ATOM   3709 HE2 PHE B 891   19.843 33.750 31.327 1.00 32.72   H
ATOM   3710 HZ PHE B 891   18.233 35.343 30.356 1.00 30.96   H
ATOM   3711 N   GLY B 892   26.255 35.894 27.381 1.00 23.65   N
ANISOU 3711 N   GLY B 892   3223   3429   2333   830   187   322   N
ATOM   3712 CA GLY B 892   27.628 36.284 27.073 1.00 22.74   C
ANISOU 3712 CA GLY B 892   3257   3119   2263   711   168   249   C
ATOM   3713 C   GLY B 892   28.591 35.799 28.167 1.00 27.03   C
ANISOU 3713 C   GLY B 892   3821   3559   2891   579   135   191   C
ATOM   3714 O   GLY B 892   28.228 35.011 29.043 1.00 25.53   O
ANISOU 3714 O   GLY B 892   3528   3430   2740   573   126   197   O
ATOM   3715 H   GLY B 892   25.927 35.025 26.981 1.00 23.65   H
ATOM   3716 HA3 GLY B 892   27.705 37.364 26.950 1.00 22.74   H
ATOM   3717 HA2 GLY B 892   27.911 35.830 26.123 1.00 22.74   H
ATOM   3718 N   LYS B 893   29.850 36.252 28.075 1.00 23.16   N
ANISOU 3718 N   LYS B 893   3462   2923   2414   469   118   144   N

ATOM	3719	CA	LYS B 893	30.962	35.793	28.907	1.00	22.94			C
ANISOU	3719	CA	LYS B 893	3435	2827	2456	329	82	98		C
ATOM	3720	C	LYS B 893	31.020	36.571	30.234	1.00	28.01			C
ANISOU	3720	C	LYS B 893	4258	3356	3029	367	94	107		C
ATOM	3721	O	LYS B 893	31.597	37.657	30.284	1.00	29.19			O
ANISOU	3721	O	LYS B 893	4612	3373	3104	314	93	98		O
ATOM	3722	CB	LYS B 893	32.266	35.889	28.082	1.00	24.48			C
ANISOU	3722	CB	LYS B 893	3629	2982	2690	171	51	59		C
ATOM	3723	CG	LYS B 893	32.247	34.934	26.870	1.00	27.39			C
ANISOU	3723	CG	LYS B 893	3828	3456	3124	142	42	45		C
ATOM	3724	CD	LYS B 893	33.532	34.897	26.030	1.00	32.98			C
ANISOU	3724	CD	LYS B 893	4517	4153	3862	13	21	16		C
ATOM	3725	CE	LYS B 893	33.864	36.230	25.342	1.00	31.54			C
ANISOU	3725	CE	LYS B 893	4452	3927	3607	6	35	29		C
ATOM	3726	NZ	LYS B 893	35.051	36.111	24.480	1.00	34.42			N1+
ANISOU	3726	NZ	LYS B 893	4769	4314	3996	-124	16	11		N1+
ATOM	3727	H	LYS B 893	30.086	36.914	27.350	1.00	23.16			H
ATOM	3728	HA	LYS B 893	30.813	34.737	29.147	1.00	22.94			H
ATOM	3729	HB3	LYS B 893	33.117	35.634	28.714	1.00	24.48			H
ATOM	3730	HB2	LYS B 893	32.423	36.917	27.753	1.00	24.48			H
ATOM	3731	HG3	LYS B 893	31.411	35.178	26.216	1.00	27.39			H
ATOM	3732	HG2	LYS B 893	32.053	33.929	27.237	1.00	27.39			H
ATOM	3733	HD3	LYS B 893	33.417	34.115	25.277	1.00	32.98			H
ATOM	3734	HD2	LYS B 893	34.364	34.582	26.658	1.00	32.98			H
ATOM	3735	HE3	LYS B 893	34.054	37.008	26.082	1.00	31.54			H
ATOM	3736	HE2	LYS B 893	33.022	36.559	24.732	1.00	31.54			H
ATOM	3737	HZ1	LYS B 893	34.883	35.413	23.767	1.00	34.42			H
ATOM	3738	HZ2	LYS B 893	35.254	36.998	24.042	1.00	34.42			H
ATOM	3739	HZ3	LYS B 893	35.843	35.819	25.038	1.00	34.42			H
ATOM	3740	N	GLY B 894	30.417	35.982	31.278	1.00	23.98			N
ANISOU	3740	N	GLY B 894	3681	2900	2530	447	105	126		N
ATOM	3741	CA	GLY B 894	30.420	36.487	32.650	1.00	22.95			C
ANISOU	3741	CA	GLY B 894	3702	2680	2338	498	120	135		C
ATOM	3742	C	GLY B 894	30.452	35.293	33.621	1.00	26.47			C
ANISOU	3742	C	GLY B 894	4006	3182	2871	446	94	122		C
ATOM	3743	O	GLY B 894	30.612	34.144	33.204	1.00	25.90			O
ANISOU	3743	O	GLY B 894	3754	3185	2904	347	62	98		O
ATOM	3744	H	GLY B 894	29.966	35.088	31.139	1.00	23.98			H
ATOM	3745	HA3	GLY B 894	29.523	37.083	32.816	1.00	22.95			H
ATOM	3746	HA2	GLY B 894	31.278	37.132	32.838	1.00	22.95			H
ATOM	3747	N	ILE B 895	30.302	35.578	34.926	1.00	23.36			N
ANISOU	3747	N	ILE B 895	3713	2742	2421	522	113	139		N
ATOM	3748	CA	ILE B 895	30.247	34.581	36.003	1.00	21.90			C
ANISOU	3748	CA	ILE B 895	3416	2603	2303	490	95	134		C
ATOM	3749	C	ILE B 895	28.781	34.445	36.456	1.00	26.23			C
ANISOU	3749	C	ILE B 895	3890	3271	2805	663	141	196		C
ATOM	3750	O	ILE B 895	28.205	35.417	36.944	1.00	25.93			O
ANISOU	3750	O	ILE B 895	3999	3205	2647	829	193	238		O
ATOM	3751	CB	ILE B 895	31.091	34.990	37.248	1.00	23.76			C
ANISOU	3751	CB	ILE B 895	3803	2712	2511	406	70	106		C
ATOM	3752	CG1	ILE B 895	32.561	35.290	36.883	1.00	23.26			C
ANISOU	3752	CG1	ILE B 895	3790	2583	2465	222	23	67		C
ATOM	3753	CG2	ILE B 895	31.042	33.929	38.374	1.00	24.95			C
ANISOU	3753	CG2	ILE B 895	3821	2921	2738	376	51	103		C
ATOM	3754	CD1	ILE B 895	33.326	36.038	37.981	1.00	28.17			C
ANISOU	3754	CD1	ILE B 895	4627	3078	2998	122	-2	54		C

ATOM 3755 H ILE B 895 30.180 36.542 35.211 1.00 23.36 H
ATOM 3756 HA ILE B 895 30.609 33.613 35.650 1.00 21.90 H
ATOM 3757 HB ILE B 895 30.671 35.913 37.652 1.00 23.76 H
ATOM 3758 HG13 ILE B 895 32.609 35.908 35.989 1.00 23.26 H
ATOM 3759 HG12 ILE B 895 33.083 34.365 36.634 1.00 23.26 H
ATOM 3760 HG21 ILE B 895 31.558 34.281 39.265 1.00 24.95 H
ATOM 3761 HG22 ILE B 895 30.029 33.683 38.688 1.00 24.95 H
ATOM 3762 HG23 ILE B 895 31.515 32.999 38.060 1.00 24.95 H
ATOM 3763 HD11 ILE B 895 34.304 36.338 37.616 1.00 28.17 H
ATOM 3764 HD12 ILE B 895 32.797 36.940 38.293 1.00 28.17 H
ATOM 3765 HD13 ILE B 895 33.489 35.418 38.861 1.00 28.17 H
ATOM 3766 N TYR B 896 28.223 33.240 36.277 1.00 23.54 N
ANISOU 3766 N TYR B 896 3331 3072 2543 624 125 209 N
ATOM 3767 CA TYR B 896 26.813 32.917 36.498 1.00 23.36 C
ANISOU 3767 CA TYR B 896 3179 3223 2475 742 158 282 C
ATOM 3768 C TYR B 896 26.555 32.455 37.943 1.00 26.72 C
ANISOU 3768 C TYR B 896 3573 3678 2900 765 165 304 C
ATOM 3769 O TYR B 896 27.389 31.765 38.528 1.00 26.55 O
ANISOU 3769 O TYR B 896 3508 3611 2969 633 125 261 O
ATOM 3770 CB TYR B 896 26.396 31.825 35.487 1.00 23.85 C
ANISOU 3770 CB TYR B 896 3038 3431 2595 646 130 291 C
ATOM 3771 CG TYR B 896 26.309 32.278 34.033 1.00 24.56 C
ANISOU 3771 CG TYR B 896 3129 3545 2658 662 134 294 C
ATOM 3772 CD1 TYR B 896 27.471 32.619 33.305 1.00 26.09 C
ANISOU 3772 CD1 TYR B 896 3425 3596 2894 580 114 229 C
ATOM 3773 CD2 TYR B 896 25.054 32.342 33.393 1.00 25.38 C
ANISOU 3773 CD2 TYR B 896 3111 3841 2691 742 155 368 C
ATOM 3774 CE1 TYR B 896 27.376 33.051 31.968 1.00 25.66 C
ANISOU 3774 CE1 TYR B 896 3373 3562 2815 590 118 232 C
ATOM 3775 CE2 TYR B 896 24.959 32.748 32.048 1.00 26.31 C
ANISOU 3775 CE2 TYR B 896 3226 3987 2782 757 156 372 C
ATOM 3776 CZ TYR B 896 26.118 33.114 31.339 1.00 29.81 C
ANISOU 3776 CZ TYR B 896 3792 4261 3275 683 140 299 C
ATOM 3777 OH TYR B 896 26.023 33.526 30.043 1.00 25.66 O
ANISOU 3777 OH TYR B 896 3271 3758 2721 697 143 304 O
ATOM 3778 H TYR B 896 28.785 32.491 35.898 1.00 23.54 H
ATOM 3779 HA TYR B 896 26.213 33.808 36.301 1.00 23.36 H
ATOM 3780 HB3 TYR B 896 25.416 31.435 35.767 1.00 23.85 H
ATOM 3781 HB2 TYR B 896 27.075 30.973 35.547 1.00 23.85 H
ATOM 3782 HD1 TYR B 896 28.446 32.555 33.763 1.00 26.09 H
ATOM 3783 HD2 TYR B 896 24.155 32.076 33.930 1.00 25.38 H
ATOM 3784 HE1 TYR B 896 28.271 33.322 31.428 1.00 25.66 H
ATOM 3785 HE2 TYR B 896 23.994 32.784 31.567 1.00 26.31 H
ATOM 3786 HH TYR B 896 26.853 33.878 29.703 1.00 25.66 H
ATOM 3787 N PHE B 897 25.382 32.833 38.471 1.00 23.91 N
ANISOU 3787 N PHE B 897 3232 3419 2435 949 221 381 N
ATOM 3788 CA PHE B 897 24.882 32.522 39.814 1.00 23.66 C
ANISOU 3788 CA PHE B 897 3171 3440 2377 1004 239 418 C
ATOM 3789 C PHE B 897 23.369 32.237 39.753 1.00 30.27 C
ANISOU 3789 C PHE B 897 3833 4537 3130 1128 280 527 C
ATOM 3790 O PHE B 897 22.734 32.544 38.745 1.00 30.66 O
ANISOU 3790 O PHE B 897 3838 4710 3103 1238 311 585 O
ATOM 3791 CB PHE B 897 25.143 33.719 40.759 1.00 24.71 C
ANISOU 3791 CB PHE B 897 3558 3412 2418 1135 279 409 C
ATOM 3792 CG PHE B 897 26.591 34.046 41.073 1.00 25.45 C
ANISOU 3792 CG PHE B 897 3827 3279 2563 991 234 318 C

ATOM	3793	CD1	PHE	B	897	27.271	33.351	42.095	1.00	27.75		C
ANISOU	3793	CD1	PHE	B	897	4109	3508	2927	866	192	276	C
ATOM	3794	CD2	PHE	B	897	27.307	34.960	40.270	1.00	26.15		C
ANISOU	3794	CD2	PHE	B	897	4085	3237	2612	975	231	284	C
ATOM	3795	CE1	PHE	B	897	28.613	33.611	42.338	1.00	28.69		C
ANISOU	3795	CE1	PHE	B	897	4357	3468	3075	722	145	209	C
ATOM	3796	CE2	PHE	B	897	28.645	35.213	40.536	1.00	29.34		C
ANISOU	3796	CE2	PHE	B	897	4631	3472	3043	813	183	215	C
ATOM	3797	CZ	PHE	B	897	29.293	34.544	41.566	1.00	27.52		C
ANISOU	3797	CZ	PHE	B	897	4362	3212	2881	687	139	182	C
ATOM	3798	H	PHE	B	897	24.766	33.412	37.916	1.00	23.91		H
ATOM	3799	HA	PHE	B	897	25.379	31.629	40.196	1.00	23.66		H
ATOM	3800	HB3	PHE	B	897	24.651	33.538	41.713	1.00	24.71		H
ATOM	3801	HB2	PHE	B	897	24.675	34.615	40.354	1.00	24.71		H
ATOM	3802	HD1	PHE	B	897	26.750	32.625	42.701	1.00	27.75		H
ATOM	3803	HD2	PHE	B	897	26.816	35.471	39.455	1.00	26.15		H
ATOM	3804	HE1	PHE	B	897	29.128	33.088	43.130	1.00	28.69		H
ATOM	3805	HE2	PHE	B	897	29.188	35.924	39.932	1.00	29.34		H
ATOM	3806	HZ	PHE	B	897	30.334	34.747	41.765	1.00	27.52		H
ATOM	3807	N	ALA	B	898	22.810	31.696	40.849	1.00	29.12		N
ANISOU	3807	N	ALA	B	898	3587	4493	2984	1118	283	567	N
ATOM	3808	CA	ALA	B	898	21.370	31.485	41.032	1.00	29.62		C
ANISOU	3808	CA	ALA	B	898	3463	4843	2948	1221	321	689	C
ATOM	3809	C	ALA	B	898	20.990	31.588	42.518	1.00	33.44		C
ANISOU	3809	C	ALA	B	898	4010	5349	3349	1382	376	740	C
ATOM	3810	O	ALA	B	898	21.842	31.378	43.380	1.00	32.14		O
ANISOU	3810	O	ALA	B	898	3993	4983	3237	1337	361	668	O
ATOM	3811	CB	ALA	B	898	20.961	30.120	40.449	1.00	30.54		C
ANISOU	3811	CB	ALA	B	898	3360	5104	3138	989	261	701	C
ATOM	3812	H	ALA	B	898	23.392	31.457	41.642	1.00	29.12		H
ATOM	3813	HA	ALA	B	898	20.824	32.271	40.507	1.00	29.62		H
ATOM	3814	HB1	ALA	B	898	19.888	29.951	40.551	1.00	30.54		H
ATOM	3815	HB2	ALA	B	898	21.196	30.058	39.386	1.00	30.54		H
ATOM	3816	HB3	ALA	B	898	21.471	29.298	40.952	1.00	30.54		H
ATOM	3817	N	ASP	B	899	19.708	31.893	42.787	1.00	31.71		N
ANISOU	3817	N	ASP	B	899	3669	5393	2987	1574	440	871	N
ATOM	3818	CA	ASP	B	899	19.131	31.960	44.139	1.00	31.72		C
ANISOU	3818	CA	ASP	B	899	3696	5465	2892	1741	500	938	C
ATOM	3819	C	ASP	B	899	18.494	30.625	44.587	1.00	34.90		C
ANISOU	3819	C	ASP	B	899	3840	6106	3316	1590	469	1006	C
ATOM	3820	O	ASP	B	899	18.283	30.446	45.785	1.00	34.88		O
ANISOU	3820	O	ASP	B	899	3829	6171	3251	1683	509	1058	O
ATOM	3821	CB	ASP	B	899	18.156	33.154	44.339	1.00	33.53		C
ANISOU	3821	CB	ASP	B	899	4009	5812	2918	2105	609	1046	C
ATOM	3822	CG	ASP	B	899	17.097	33.425	43.251	1.00	41.11		C
ANISOU	3822	CG	ASP	B	899	4749	7110	3761	2231	645	1180	C
ATOM	3823	OD1	ASP	B	899	16.708	32.485	42.521	1.00	42.33		O
ANISOU	3823	OD1	ASP	B	899	4626	7495	3963	2028	587	1224	O
ATOM	3824	OD2	ASP	B	899	16.600	34.572	43.239	1.00	41.75		O1-
ANISOU	3824	OD2	ASP	B	899	4948	7230	3685	2538	733	1249	O1-
ATOM	3825	H	ASP	B	899	19.059	32.056	42.029	1.00	31.71		H
ATOM	3826	HA	ASP	B	899	19.947	32.134	44.843	1.00	31.72		H
ATOM	3827	HB3	ASP	B	899	18.757	34.057	44.451	1.00	33.53		H
ATOM	3828	HB2	ASP	B	899	17.633	33.044	45.290	1.00	33.53		H
ATOM	3829	N	MET	B	900	18.232	29.707	43.640	1.00	31.23		N
ANISOU	3829	N	MET	B	900	3190	5747	2928	1341	398	1000	N

```
ATOM   3830  CA  MET B 900      17.774  28.345  43.914  1.00 31.49           C
ANISOU 3830  CA  MET B 900     3014   5977   2974   1134    356   1057       C
ATOM   3831  C   MET B 900      18.992  27.419  44.041  1.00 33.15           C
ANISOU 3831  C   MET B 900     3314   5928   3352    872    279    926       C
ATOM   3832  O   MET B 900      19.813  27.379  43.121  1.00 31.29           O
ANISOU 3832  O   MET B 900     3140   5543   3208    753    233    839       O
ATOM   3833  CB  MET B 900      16.818  27.866  42.799  1.00 34.91           C
ANISOU 3833  CB  MET B 900     3201   6730   3332   1030    331   1158       C
ATOM   3834  CG  MET B 900      15.553  28.728  42.619  1.00 40.08           C
ANISOU 3834  CG  MET B 900     3715   7718   3797   1303    410   1318       C
ATOM   3835  SD  MET B 900      14.399  28.779  44.020  1.00 46.57           S
ANISOU 3835  SD  MET B 900     4373   8861   4462   1451    477   1482       S
ATOM   3836  CE  MET B 900      13.511  27.219  43.768  1.00 44.24           C
ANISOU 3836  CE  MET B 900     3757   8918   4136   1073    390   1581       C
ATOM   3837  H   MET B 900      18.422  29.929  42.674  1.00 31.23           H
ATOM   3838  HA  MET B 900      17.229  28.323  44.853  1.00 31.49           H
ATOM   3839  HB3 MET B 900      16.519  26.835  42.995  1.00 34.91           H
ATOM   3840  HB2 MET B 900      17.349  27.842  41.847  1.00 34.91           H
ATOM   3841  HG3 MET B 900      15.000  28.364  41.755  1.00 40.08           H
ATOM   3842  HG2 MET B 900      15.834  29.753  42.382  1.00 40.08           H
ATOM   3843  HE1 MET B 900      12.821  27.042  44.593  1.00 44.24           H
ATOM   3844  HE2 MET B 900      12.939  27.253  42.841  1.00 44.24           H
ATOM   3845  HE3 MET B 900      14.204  26.381  43.714  1.00 44.24           H
ATOM   3846  N   VAL B 901      19.083  26.690  45.169  1.00 29.27           N
ANISOU 3846  N   VAL B 901     2840   5387   2893    802    270    918       N
ATOM   3847  CA  VAL B 901      20.177  25.754  45.453  1.00 28.61           C
ANISOU 3847  CA  VAL B 901     2854   5068   2950    596    209    809       C
ATOM   3848  C   VAL B 901      20.224  24.571  44.468  1.00 33.02           C
ANISOU 3848  C   VAL B 901     3337   5648   3561    335    142    785       C
ATOM   3849  O   VAL B 901      21.317  24.187  44.061  1.00 31.57           O
ANISOU 3849  O   VAL B 901     3268   5240   3486    231    102    679       O
ATOM   3850  CB  VAL B 901      20.136  25.199  46.910  1.00 31.69           C
ANISOU 3850  CB  VAL B 901     3265   5435   3342    584    217    824       C
ATOM   3851  CG1 VAL B 901      18.976  24.228  47.211  1.00 31.41           C
ANISOU 3851  CG1 VAL B 901     3038   5666   3229    456    210    936       C
ATOM   3852  CG2 VAL B 901      21.463  24.531  47.319  1.00 31.28           C
ANISOU 3852  CG2 VAL B 901     3359   5103   3422    459    171    706       C
ATOM   3853  H   VAL B 901      18.379  26.793  45.890  1.00 29.27           H
ATOM   3854  HA  VAL B 901      21.107  26.313  45.334  1.00 28.61           H
ATOM   3855  HB  VAL B 901      20.002  26.055  47.572  1.00 31.69           H
ATOM   3856 HG11 VAL B 901      19.004  23.911  48.249  1.00 31.41           H
ATOM   3857 HG12 VAL B 901      18.011  24.696  47.033  1.00 31.41           H
ATOM   3858 HG13 VAL B 901      19.023  23.311  46.625  1.00 31.41           H
ATOM   3859 HG21 VAL B 901      21.435  24.217  48.360  1.00 31.28           H
ATOM   3860 HG22 VAL B 901      21.679  23.643  46.724  1.00 31.28           H
ATOM   3861 HG23 VAL B 901      22.299  25.219  47.208  1.00 31.28           H
ATOM   3862  N   SER B 902      19.045  24.049  44.088  1.00 31.33           N
ANISOU 3862  N   SER B 902     2939   5711   3253    231    131    889       N
ATOM   3863  CA  SER B 902      18.887  22.904  43.195  1.00 31.82           C
ANISOU 3863  CA  SER B 902     2957   5803   3329    -39     66    876       C
ATOM   3864  C   SER B 902      19.260  23.207  41.736  1.00 34.19           C
ANISOU 3864  C   SER B 902     3290   6035   3665    -45     47    818       C
ATOM   3865  O   SER B 902      19.756  22.305  41.065  1.00 33.49           O
ANISOU 3865  O   SER B 902     3273   5817   3635   -236     -3    748       O
ATOM   3866  CB  SER B 902      17.464  22.343  43.341  1.00 35.40           C
ANISOU 3866  CB  SER B 902     3207   6596   3647   -183     52   1015       C
```

```
ATOM   3867  OG  SER B 902      16.490  23.248  42.862  1.00 45.36           O
ANISOU 3867  OG  SER B 902     4292   8159   4783    -16     95   1134       O
ATOM   3868  H   SER B 902      18.188  24.442  44.453  1.00 31.33           H
ATOM   3869  HA  SER B 902      19.562  22.126  43.551  1.00 31.82           H
ATOM   3870  HB3 SER B 902      17.259  22.104  44.382  1.00 35.40           H
ATOM   3871  HB2 SER B 902      17.373  21.408  42.789  1.00 35.40           H
ATOM   3872  HG  SER B 902      16.456  23.175  41.901  1.00 45.36           H
ATOM   3873  N   LYS B 903      19.062  24.462  41.286  1.00 29.96           N
ANISOU 3873  N   LYS B 903     2730   5568   3087    174     92    846       N
ATOM   3874  CA  LYS B 903      19.492  24.935  39.970  1.00 29.28           C
ANISOU 3874  CA  LYS B 903     2681   5413   3029    184     79    793       C
ATOM   3875  C   LYS B 903      21.023  24.935  39.834  1.00 33.17           C
ANISOU 3875  C   LYS B 903     3374   5564   3665    158     59    648       C
ATOM   3876  O   LYS B 903      21.531  24.414  38.842  1.00 32.64           O
ANISOU 3876  O   LYS B 903     3346   5403   3651     28     21    584       O
ATOM   3877  CB  LYS B 903      18.867  26.314  39.663  1.00 30.56           C
ANISOU 3877  CB  LYS B 903     2797   5718   3095    444    139    865       C
ATOM   3878  CG  LYS B 903      19.349  26.937  38.338  1.00 35.53           C
ANISOU 3878  CG  LYS B 903     3486   6261   3754    469    130    810       C
ATOM   3879  CD  LYS B 903      18.499  28.126  37.878  1.00 37.90           C
ANISOU 3879  CD  LYS B 903     3690   6791   3918    681    181    918       C
ATOM   3880  CE  LYS B 903      19.084  28.792  36.624  1.00 47.14           C
ANISOU 3880  CE  LYS B 903     4946   7842   5123    708    174    855       C
ATOM   3881  NZ  LYS B 903      18.220  29.871  36.124  1.00 50.93           N1+
ANISOU 3881  NZ  LYS B 903     5380   8505   5467    958    235    955       N1+
ATOM   3882  H   LYS B 903      18.660  25.152  41.905  1.00 29.96           H
ATOM   3883  HA  LYS B 903      19.101  24.230  39.234  1.00 29.28           H
ATOM   3884  HB3 LYS B 903      19.088  27.011  40.472  1.00 30.56           H
ATOM   3885  HB2 LYS B 903      17.782  26.206  39.641  1.00 30.56           H
ATOM   3886  HG3 LYS B 903      19.350  26.177  37.556  1.00 35.53           H
ATOM   3887  HG2 LYS B 903      20.383  27.269  38.445  1.00 35.53           H
ATOM   3888  HD3 LYS B 903      18.410  28.853  38.688  1.00 37.90           H
ATOM   3889  HD2 LYS B 903      17.492  27.766  37.667  1.00 37.90           H
ATOM   3890  HE3 LYS B 903      19.211  28.058  35.827  1.00 47.14           H
ATOM   3891  HE2 LYS B 903      20.070  29.205  36.839  1.00 47.14           H
ATOM   3892  HZ1 LYS B 903      18.115  30.582  36.836  1.00 50.93           H
ATOM   3893  HZ2 LYS B 903      18.649  30.287  35.304  1.00 50.93           H
ATOM   3894  HZ3 LYS B 903      17.312  29.508  35.876  1.00 50.93           H
ATOM   3895  N   SER B 904      21.716  25.484  40.846  1.00 28.85           N
ANISOU 3895  N   SER B 904     2946   4851   3164    273     85    605       N
ATOM   3896  CA  SER B 904      23.175  25.489  40.931  1.00 27.57           C
ANISOU 3896  CA  SER B 904     2943   4415   3117    244     65    489       C
ATOM   3897  C   SER B 904      23.770  24.084  41.150  1.00 31.23           C
ANISOU 3897  C   SER B 904     3434   4781   3649     57     22    444       C
ATOM   3898  O   SER B 904      24.780  23.767  40.527  1.00 30.03           O
ANISOU 3898  O   SER B 904     3364   4473   3571    -10     -2    365       O
ATOM   3899  CB  SER B 904      23.631  26.491  42.008  1.00 28.96           C
ANISOU 3899  CB  SER B 904     3238   4471   3294    404     99    469       C
ATOM   3900  OG  SER B 904      23.716  27.788  41.453  1.00 29.20           O
ANISOU 3900  OG  SER B 904     3329   4501   3264    570    137    482       O
ATOM   3901  H   SER B 904      21.221  25.901  41.622  1.00 28.85           H
ATOM   3902  HA  SER B 904      23.564  25.839  39.975  1.00 27.57           H
ATOM   3903  HB3 SER B 904      24.626  26.242  42.377  1.00 28.96           H
ATOM   3904  HB2 SER B 904      22.959  26.492  42.867  1.00 28.96           H
ATOM   3905  HG  SER B 904      23.975  28.407  42.139  1.00 29.20           H
ATOM   3906  N   ALA B 905      23.108  23.253  41.975  1.00 28.66           N
```

ANISOU 3906 N ALA B 905 3051 4550 3287 -17 18 499 N
ATOM 3907 CA ALA B 905 23.510 21.880 42.294 1.00 28.13 C
ANISOU 3907 CA ALA B 905 3047 4379 3262 -186 -15 465 C
ATOM 3908 C ALA B 905 23.450 20.892 41.111 1.00 31.33 C
ANISOU 3908 C ALA B 905 3473 4768 3664 -366 -53 440 C
ATOM 3909 O ALA B 905 24.140 19.875 41.168 1.00 29.91 O
ANISOU 3909 O ALA B 905 3415 4418 3530 -453 -72 379 O
ATOM 3910 CB ALA B 905 22.677 21.372 43.480 1.00 28.96 C
ANISOU 3910 CB ALA B 905 3091 4606 3308 -238 -10 542 C
ATOM 3911 H ALA B 905 22.281 23.587 42.454 1.00 28.66 H
ATOM 3912 HA ALA B 905 24.549 21.922 42.621 1.00 28.13 H
ATOM 3913 HB1 ALA B 905 22.977 20.370 43.781 1.00 28.96 H
ATOM 3914 HB2 ALA B 905 22.799 22.013 44.353 1.00 28.96 H
ATOM 3915 HB3 ALA B 905 21.619 21.335 43.232 1.00 28.96 H
ATOM 3916 N ASN B 906 22.687 21.211 40.047 1.00 29.30 N
ANISOU 3916 N ASN B 906 3111 4684 3339 -407 -62 487 N
ATOM 3917 CA ASN B 906 22.682 20.460 38.781 1.00 29.96 C
ANISOU 3917 CA ASN B 906 3224 4760 3399 -585 -101 464 C
ATOM 3918 C ASN B 906 24.023 20.568 38.026 1.00 34.05 C
ANISOU 3918 C ASN B 906 3874 5052 4009 -536 -101 357 C
ATOM 3919 O ASN B 906 24.416 19.600 37.376 1.00 33.65 O
ANISOU 3919 O ASN B 906 3934 4891 3961 -666 -124 309 O
ATOM 3920 CB ASN B 906 21.532 20.944 37.865 1.00 31.49 C
ANISOU 3920 CB ASN B 906 3256 5215 3493 -615 -109 546 C
ATOM 3921 CG ASN B 906 20.120 20.652 38.387 1.00 53.90 C
ANISOU 3921 CG ASN B 906 5936 8336 6207 -722 -120 672 C
ATOM 3922 ND2 ASN B 906 19.159 21.501 38.022 1.00 43.91 N
ANISOU 3922 ND2 ASN B 906 4719 7043 4921 -878 -140 686 N
ATOM 3923 OD1 ASN B 906 19.890 19.671 39.091 1.00 54.10 O
ANISOU 3923 OD1 ASN B 906 5788 8628 6140 -665 -109 767 O
ATOM 3924 H ASN B 906 22.127 22.051 40.073 1.00 29.30 H
ATOM 3925 HA ASN B 906 22.516 19.410 39.035 1.00 29.96 H
ATOM 3926 HB3 ASN B 906 21.605 20.446 36.897 1.00 31.49 H
ATOM 3927 HB2 ASN B 906 21.637 22.011 37.665 1.00 31.49 H
ATOM 3928 HD22 ASN B 906 18.212 21.360 38.345 1.00 43.91 H
ATOM 3929 HD21 ASN B 906 19.374 22.297 37.440 1.00 43.91 H
ATOM 3930 N TYR B 907 24.712 21.719 38.150 1.00 30.19 N
ANISOU 3930 N TYR B 907 3393 4496 3581 -351 -72 324 N
ATOM 3931 CA TYR B 907 26.028 21.975 37.553 1.00 29.83 C
ANISOU 3931 CA TYR B 907 3444 4277 3612 -297 -70 239 C
ATOM 3932 C TYR B 907 27.198 21.300 38.294 1.00 32.23 C
ANISOU 3932 C TYR B 907 3864 4401 3982 -292 -69 184 C
ATOM 3933 O TYR B 907 28.320 21.354 37.789 1.00 30.38 O
ANISOU 3933 O TYR B 907 3693 4053 3797 -250 -66 127 O
ATOM 3934 CB TYR B 907 26.256 23.493 37.401 1.00 30.77 C
ANISOU 3934 CB TYR B 907 3539 4408 3743 -137 -45 237 C
ATOM 3935 CG TYR B 907 25.278 24.169 36.457 1.00 31.59 C
ANISOU 3935 CG TYR B 907 3552 4668 3781 -119 -41 284 C
ATOM 3936 CD1 TYR B 907 25.467 24.084 35.062 1.00 33.05 C
ANISOU 3936 CD1 TYR B 907 3747 4839 3970 -183 -58 251 C
ATOM 3937 CD2 TYR B 907 24.171 24.874 36.967 1.00 32.01 C
ANISOU 3937 CD2 TYR B 907 3511 4898 3756 -21 -15 369 C
ATOM 3938 CE1 TYR B 907 24.552 24.699 34.185 1.00 33.14 C
ANISOU 3938 CE1 TYR B 907 3670 5006 3914 -161 -55 299 C
ATOM 3939 CE2 TYR B 907 23.254 25.488 36.093 1.00 32.95 C
ANISOU 3939 CE2 TYR B 907 3534 5189 3797 18 -7 428 C

```
ATOM   3940  CZ  TYR B 907      23.445  25.402  34.700  1.00 40.29           C
ANISOU 3940  CZ  TYR B 907     4471   6099   4738    -56    -30    391       C
ATOM   3941  OH  TYR B 907      22.556  25.998  33.854  1.00 42.26           O
ANISOU 3941  OH  TYR B 907     4622   6532   4905    -13    -24    455       O
ATOM   3942  H   TYR B 907      24.334  22.464  38.719  1.00 30.19           H
ATOM   3943  HA  TYR B 907      26.018  21.549  36.549  1.00 29.83           H
ATOM   3944  HB3 TYR B 907      27.258  23.684  37.016  1.00 30.77           H
ATOM   3945  HB2 TYR B 907      26.221  23.983  38.374  1.00 30.77           H
ATOM   3946  HD1 TYR B 907      26.312  23.543  34.661  1.00 33.05           H
ATOM   3947  HD2 TYR B 907      24.019  24.936  38.032  1.00 32.01           H
ATOM   3948  HE1 TYR B 907      24.705  24.628  33.118  1.00 33.14           H
ATOM   3949  HE2 TYR B 907      22.405  26.022  36.494  1.00 32.95           H
ATOM   3950  HH  TYR B 907      22.790  25.897  32.929  1.00 42.26           H
ATOM   3951  N   CYS B 908      26.929  20.635  39.433  1.00 29.47           N
ANISOU 3951  N   CYS B 908     3535   4042   3620   -331    -70    209       N
ATOM   3952  CA  CYS B 908      27.891  19.770  40.121  1.00 29.62           C
ANISOU 3952  CA  CYS B 908     3665   3906   3684   -318    -67    170       C
ATOM   3953  C   CYS B 908      28.167  18.466  39.345  1.00 36.43           C
ANISOU 3953  C   CYS B 908     4649   4669   4523   -427    -77    137       C
ATOM   3954  O   CYS B 908      29.267  17.931  39.469  1.00 36.67           O
ANISOU 3954  O   CYS B 908     4787   4564   4581   -375    -65     98       O
ATOM   3955  CB  CYS B 908      27.468  19.445  41.567  1.00 29.13           C
ANISOU 3955  CB  CYS B 908     3596   3865   3606   -321    -63    212       C
ATOM   3956  SG  CYS B 908      27.362  20.964  42.555  1.00 32.55           S
ANISOU 3956  SG  CYS B 908     3946   4373   4049   -168    -42    243       S
ATOM   3957  H   CYS B 908      25.990  20.642  39.805  1.00 29.47           H
ATOM   3958  HA  CYS B 908      28.834  20.304  40.170  1.00 29.62           H
ATOM   3959  HB3 CYS B 908      28.201  18.791  42.041  1.00 29.13           H
ATOM   3960  HB2 CYS B 908      26.512  18.924  41.594  1.00 29.13           H
ATOM   3961  HG  CYS B 908      28.678  21.197  42.605  1.00 32.55           H
ATOM   3962  N   HIS B 909      27.176  17.996  38.560  1.00 35.20           N
ANISOU 3962  N   HIS B 909     4487   4591   4298   -574    -98    160       N
ATOM   3963  CA  HIS B 909      27.204  16.786  37.726  1.00 36.26           C
ANISOU 3963  CA  HIS B 909     4772   4629   4374   -713   -111    133       C
ATOM   3964  C   HIS B 909      27.586  15.500  38.490  1.00 39.55           C
ANISOU 3964  C   HIS B 909     5360   4901   4765   -756   -104    127       C
ATOM   3965  O   HIS B 909      28.322  14.661  37.971  1.00 37.96           O
ANISOU 3965  O   HIS B 909     5343   4532   4548   -739    -87     82       O
ATOM   3966  CB  HIS B 909      28.043  17.001  36.446  1.00 37.62           C
ANISOU 3966  CB  HIS B 909     5004   4715   4574   -648   -100     71       C
ATOM   3967  CG  HIS B 909      27.496  18.035  35.494  1.00 41.87           C
ANISOU 3967  CG  HIS B 909     5412   5389   5108   -655   -112     82       C
ATOM   3968  CD2 HIS B 909      26.605  17.924  34.449  1.00 44.01           C
ANISOU 3968  CD2 HIS B 909     5573   5713   5436   -521    -99     76       C
ATOM   3969  ND1 HIS B 909      27.889  19.361  35.508  1.00 43.98           N
ANISOU 3969  ND1 HIS B 909     5666   5755   5290   -822   -143    109       N
ATOM   3970  CE1 HIS B 909      27.237  19.976  34.518  1.00 43.53           C
ANISOU 3970  CE1 HIS B 909     5479   5813   5246   -760   -144    119       C
ATOM   3971  NE2 HIS B 909      26.441  19.166  33.832  1.00 43.89           N
ANISOU 3971  NE2 HIS B 909     5477   5822   5375   -579   -115     98       N
ATOM   3972  H   HIS B 909      26.314  18.522  38.512  1.00 35.20           H
ATOM   3973  HA  HIS B 909      26.173  16.622  37.408  1.00 36.26           H
ATOM   3974  HB3 HIS B 909      28.113  16.073  35.877  1.00 37.62           H
ATOM   3975  HB2 HIS B 909      29.066  17.269  36.708  1.00 37.62           H
ATOM   3976  HD2 HIS B 909      26.076  17.053  34.092  1.00 44.01           H
ATOM   3977  HD1 HIS B 909      28.530  19.791  36.161  1.00 43.98           H
```

```
ATOM   3978  HE1 HIS B 909      27.345  21.029  34.302  1.00 43.53           H
ATOM   3979  N   THR B 910      27.053  15.371  39.715  1.00 36.87           N
ANISOU 3979  N   THR B 910     4965   4631   4411   -793   -109    180       N
ATOM   3980  CA  THR B 910      27.148  14.172  40.546  1.00 37.41           C
ANISOU 3980  CA  THR B 910     5190   4577   4447   -840   -102    186       C
ATOM   3981  C   THR B 910      26.252  13.045  39.992  1.00 44.71           C
ANISOU 3981  C   THR B 910     6273   5466   5250  -1083   -129    200       C
ATOM   3982  O   THR B 910      25.326  13.301  39.220  1.00 43.93           O
ANISOU 3982  O   THR B 910     6088   5512   5090  -1233   -162    231       O
ATOM   3983  CB  THR B 910      26.711  14.490  41.998  1.00 41.51           C
ANISOU 3983  CB  THR B 910     5590   5197   4987   -802    -99    241       C
ATOM   3984  CG2 THR B 910      27.615  15.527  42.677  1.00 35.05           C
ANISOU 3984  CG2 THR B 910     4684   4368   4266   -586    -76    219       C
ATOM   3985  OG1 THR B 910      25.373  14.941  42.047  1.00 47.89           O
ANISOU 3985  OG1 THR B 910     6223   6226   5745   -898   -119    307       O
ATOM   3986  H   THR B 910      26.452  16.098  40.073  1.00 36.87           H
ATOM   3987  HA  THR B 910      28.184  13.828  40.552  1.00 37.41           H
ATOM   3988  HB  THR B 910      26.755  13.577  42.594  1.00 41.51           H
ATOM   3989 HG21 THR B 910      27.361  15.635  43.729  1.00 35.05           H
ATOM   3990 HG22 THR B 910      28.662  15.223  42.627  1.00 35.05           H
ATOM   3991 HG23 THR B 910      27.531  16.506  42.207  1.00 35.05           H
ATOM   3992  HG1 THR B 910      25.187  15.270  42.931  1.00 47.89           H
ATOM   3993  N   SER B 911      26.584  11.808  40.382  1.00 44.91           N
ANISOU 3993  N   SER B 911     6544   5295   5223  -1121   -116    180       N
ATOM   3994  CA  SER B 911      25.990  10.585  39.847  1.00 46.09           C
ANISOU 3994  CA  SER B 911     6930   5350   5233  -1363   -139    185       C
ATOM   3995  C   SER B 911      25.722   9.563  40.960  1.00 50.62           C
ANISOU 3995  C   SER B 911     7662   5833   5737  -1464   -138    222       C
ATOM   3996  O   SER B 911      26.029   9.814  42.125  1.00 49.71           O
ANISOU 3996  O   SER B 911     7466   5729   5695  -1319   -115    239       O
ATOM   3997  CB  SER B 911      26.918  10.046  38.731  1.00 50.77           C
ANISOU 3997  CB  SER B 911     7773   5724   5795  -1293   -111    111       C
ATOM   3998  OG  SER B 911      28.133   9.528  39.244  1.00 60.35           O
ANISOU 3998  OG  SER B 911     9081   6781   7067  -1043    -56     79       O
ATOM   3999  H   SER B 911      27.327  11.678  41.056  1.00 44.91           H
ATOM   4000  HA  SER B 911      25.018  10.813  39.406  1.00 46.09           H
ATOM   4001  HB3 SER B 911      27.143  10.827  38.004  1.00 50.77           H
ATOM   4002  HB2 SER B 911      26.419   9.249  38.178  1.00 50.77           H
ATOM   4003  HG  SER B 911      28.691  10.265  39.503  1.00 60.35           H
ATOM   4004  N   GLN B 912      25.166   8.408  40.563  1.00 47.85           N
ANISOU 4004  N   GLN B 912     7558   5388   5234  -1724   -164    234       N
ATOM   4005  CA  GLN B 912      24.950   7.239  41.421  1.00 48.04           C
ANISOU 4005  CA  GLN B 912     7804   5288   5162  -1855   -163    266       C
ATOM   4006  C   GLN B 912      26.264   6.647  41.976  1.00 50.97           C
ANISOU 4006  C   GLN B 912     8415   5389   5564  -1605    -99    217       C
ATOM   4007  O   GLN B 912      26.267   6.152  43.102  1.00 49.61           O
ANISOU 4007  O   GLN B 912     8309   5159   5382  -1581    -83    248       O
ATOM   4008  CB  GLN B 912      24.190   6.157  40.619  1.00 49.96           C
ANISOU 4008  CB  GLN B 912     8297   5474   5212  -2216   -210    286       C
ATOM   4009  CG  GLN B 912      22.796   6.562  40.085  1.00 72.64           C
ANISOU 4009  CG  GLN B 912    10922   8648   8029  -2468   -277    347       C
ATOM   4010  CD  GLN B 912      21.696   6.659  41.148  1.00 97.07           C
ANISOU 4010  CD  GLN B 912    13874  11969  11039  -2717   -321    456       C
ATOM   4011  NE2 GLN B 912      20.463   6.911  40.703  1.00 90.95           N
ANISOU 4011  NE2 GLN B 912    12907  11477  10172  -2965   -380    529       N
ATOM   4012  OE1 GLN B 912      21.935   6.502  42.343  1.00 94.35           O
```

ANISOU 4012 OE1 GLN B 912 13581 11570 10696 -2695 -302 486 O
ATOM 4013 H GLN B 912 24.925 8.285 39.590 1.00 47.85 H
ATOM 4014 HA GLN B 912 24.346 7.556 42.273 1.00 48.04 H
ATOM 4015 HB3 GLN B 912 24.091 5.252 41.221 1.00 49.96 H
ATOM 4016 HB2 GLN B 912 24.803 5.866 39.764 1.00 49.96 H
ATOM 4017 HG3 GLN B 912 22.478 5.814 39.359 1.00 72.64 H
ATOM 4018 HG2 GLN B 912 22.849 7.505 39.541 1.00 72.64 H
ATOM 4019 HE22 GLN B 912 19.695 6.973 41.356 1.00 90.95 H
ATOM 4020 HE21 GLN B 912 20.288 7.035 39.717 1.00 90.95 H
ATOM 4021 N GLY B 913 27.347 6.725 41.180 1.00 48.12 N
ANISOU 4021 N GLY B 913 8159 4893 5233 -1410 -60 150 N
ATOM 4022 CA GLY B 913 28.673 6.212 41.525 1.00 48.93 C
ANISOU 4022 CA GLY B 913 8454 4787 5350 -1132 9 116 C
ATOM 4023 C GLY B 913 29.568 7.276 42.182 1.00 54.90 C
ANISOU 4023 C GLY B 913 8940 5655 6265 -847 35 121 C
ATOM 4024 O GLY B 913 30.654 6.926 42.641 1.00 55.15 O
ANISOU 4024 O GLY B 913 9081 5572 6302 -630 85 121 O
ATOM 4025 H GLY B 913 27.257 7.167 40.277 1.00 48.12 H
ATOM 4026 HA3 GLY B 913 29.159 5.872 40.610 1.00 48.93 H
ATOM 4027 HA2 GLY B 913 28.599 5.343 42.181 1.00 48.93 H
ATOM 4028 N ASP B 914 29.135 8.549 42.239 1.00 51.08 N
ANISOU 4028 N ASP B 914 8114 5402 5893 -848 2 132 N
ATOM 4029 CA ASP B 914 29.868 9.649 42.870 1.00 50.44 C
ANISOU 4029 CA ASP B 914 7777 5442 5947 -631 15 138 C
ATOM 4030 C ASP B 914 28.847 10.680 43.408 1.00 50.44 C
ANISOU 4030 C ASP B 914 7500 5665 5999 -740 -27 179 C
ATOM 4031 O ASP B 914 28.687 11.737 42.795 1.00 50.35 O
ANISOU 4031 O ASP B 914 7302 5790 6039 -734 -44 170 O
ATOM 4032 H ASP B 914 28.230 8.781 41.855 1.00 51.08 H
ATOM 4033 CB ASP B 914 30.947 10.245 41.921 1.00 0.00 C
ATOM 4034 CG ASP B 914 31.926 11.274 42.520 1.00 0.00 C
ATOM 4035 OD1 ASP B 914 32.029 11.370 43.764 1.00 0.00 O
ATOM 4036 OD2 ASP B 914 32.643 11.891 41.700 1.00 0.00 O1-
ATOM 4037 HB2 ASP B 914 31.547 9.428 41.519 1.00 0.00 H
ATOM 4038 HB3 ASP B 914 30.459 10.678 41.046 1.00 0.00 H
ATOM 4039 HA ASP B 914 30.399 9.245 43.733 1.00 50.44 H
ATOM 4040 N PRO B 915 28.125 10.335 44.503 1.00 43.16 N
ANISOU 4040 N PRO B 915 6569 4783 5047 -834 -39 232 N
ATOM 4041 CA PRO B 915 27.002 11.148 45.010 1.00 41.40 C
ANISOU 4041 CA PRO B 915 6099 4787 4843 -925 -69 286 C
ATOM 4042 C PRO B 915 27.401 12.322 45.933 1.00 41.73 C
ANISOU 4042 C PRO B 915 5944 4928 4984 -739 -56 298 C
ATOM 4043 O PRO B 915 26.548 12.775 46.692 1.00 42.29 O
ANISOU 4043 O PRO B 915 5887 5137 5045 -782 -64 354 O
ATOM 4044 CB PRO B 915 26.147 10.097 45.742 1.00 43.23 C
ANISOU 4044 CB PRO B 915 6445 5019 4963 -1155 -88 344 C
ATOM 4045 CG PRO B 915 27.170 9.146 46.337 1.00 48.10 C
ANISOU 4045 CG PRO B 915 7346 5387 5542 -1095 -57 319 C
ATOM 4046 CD PRO B 915 28.229 9.071 45.244 1.00 44.19 C
ANISOU 4046 CD PRO B 915 6926 4759 5107 -865 -22 252 C
ATOM 4047 HA PRO B 915 26.421 11.570 44.189 1.00 41.40 H
ATOM 4048 HB3 PRO B 915 25.526 9.568 45.018 1.00 43.23 H
ATOM 4049 HB2 PRO B 915 25.477 10.500 46.501 1.00 43.23 H
ATOM 4050 HG3 PRO B 915 26.756 8.173 46.603 1.00 48.10 H
ATOM 4051 HG2 PRO B 915 27.600 9.588 47.236 1.00 48.10 H
ATOM 4052 HD2 PRO B 915 29.222 8.910 45.666 1.00 44.19 H

ATOM 4053 HD3 PRO B 915 27.992 8.237 44.583 1.00 44.19 H
ATOM 4054 N ILE B 916 28.657 12.797 45.878 1.00 34.39 N
ANISOU 4054 N ILE B 916 4997 3937 4134 -539 -36 252 N
ATOM 4055 CA ILE B 916 29.136 13.911 46.702 1.00 33.10 C
ANISOU 4055 CA ILE B 916 4683 3846 4048 -380 -30 255 C
ATOM 4056 C ILE B 916 29.629 15.029 45.764 1.00 34.20 C
ANISOU 4056 C ILE B 916 4707 4046 4241 -302 -33 219 C
ATOM 4057 O ILE B 916 30.422 14.759 44.861 1.00 33.88 O
ANISOU 4057 O ILE B 916 4736 3931 4208 -268 -26 179 O
ATOM 4058 CB ILE B 916 30.314 13.499 47.639 1.00 36.03 C
ANISOU 4058 CB ILE B 916 5143 4108 4439 -236 -9 248 C
ATOM 4059 CG1 ILE B 916 30.077 12.165 48.391 1.00 36.62 C
ANISOU 4059 CG1 ILE B 916 5388 4082 4446 -306 1 280 C
ATOM 4060 CG2 ILE B 916 30.684 14.609 48.648 1.00 36.84 C
ANISOU 4060 CG2 ILE B 916 5099 4296 4601 -113 -14 255 C
ATOM 4061 CD1 ILE B 916 28.888 12.159 49.366 1.00 46.19 C
ANISOU 4061 CD1 ILE B 916 6535 5392 5623 -437 -14 337 C
ATOM 4062 H ILE B 916 29.322 12.411 45.221 1.00 34.39 H
ATOM 4063 HA ILE B 916 28.331 14.314 47.312 1.00 33.10 H
ATOM 4064 HB ILE B 916 31.197 13.336 47.017 1.00 36.03 H
ATOM 4065 HG13 ILE B 916 30.983 11.912 48.944 1.00 36.62 H
ATOM 4066 HG12 ILE B 916 29.955 11.352 47.674 1.00 36.62 H
ATOM 4067 HG21 ILE B 916 31.565 14.333 49.229 1.00 36.84 H
ATOM 4068 HG22 ILE B 916 30.905 15.555 48.161 1.00 36.84 H
ATOM 4069 HG23 ILE B 916 29.873 14.800 49.351 1.00 36.84 H
ATOM 4070 HD11 ILE B 916 28.293 11.254 49.241 1.00 46.19 H
ATOM 4071 HD12 ILE B 916 29.230 12.188 50.400 1.00 46.19 H
ATOM 4072 HD13 ILE B 916 28.223 13.008 49.220 1.00 46.19 H
ATOM 4073 N GLY B 917 29.137 16.257 45.995 1.00 29.12 N
ANISOU 4073 N GLY B 917 3910 3531 3623 -268 -40 238 N
ATOM 4074 CA GLY B 917 29.471 17.437 45.197 1.00 28.30 C
ANISOU 4074 CA GLY B 917 3716 3478 3558 -199 -43 210 C
ATOM 4075 C GLY B 917 29.804 18.617 46.115 1.00 29.84 C
ANISOU 4075 C GLY B 917 3837 3717 3782 -88 -40 214 C
ATOM 4076 O GLY B 917 29.606 18.558 47.331 1.00 27.84 O
ANISOU 4076 O GLY B 917 3581 3478 3519 -63 -36 243 O
ATOM 4077 H GLY B 917 28.515 16.401 46.780 1.00 29.12 H
ATOM 4078 HA3 GLY B 917 28.621 17.700 44.567 1.00 28.30 H
ATOM 4079 HA2 GLY B 917 30.319 17.257 44.534 1.00 28.30 H
ATOM 4080 N LEU B 918 30.306 19.699 45.498 1.00 24.44 N
ANISOU 4080 N LEU B 918 3114 3049 3124 -30 -43 186 N
ATOM 4081 CA LEU B 918 30.713 20.941 46.157 1.00 23.25 C
ANISOU 4081 CA LEU B 918 2938 2919 2979 51 -45 184 C
ATOM 4082 C LEU B 918 29.987 22.129 45.505 1.00 27.14 C
ANISOU 4082 C LEU B 918 3386 3481 3445 75 -36 191 C
ATOM 4083 O LEU B 918 29.888 22.183 44.278 1.00 26.83 O
ANISOU 4083 O LEU B 918 3324 3461 3409 43 -37 178 O
ATOM 4084 CB LEU B 918 32.246 21.107 46.031 1.00 22.85 C
ANISOU 4084 CB LEU B 918 2912 2814 2957 91 -59 152 C
ATOM 4085 CG LEU B 918 33.088 20.057 46.790 1.00 26.41 C
ANISOU 4085 CG LEU B 918 3402 3215 3418 113 -61 156 C
ATOM 4086 CD1 LEU B 918 34.592 20.226 46.497 1.00 25.11 C
ANISOU 4086 CD1 LEU B 918 3220 3060 3262 156 -73 143 C
ATOM 4087 CD2 LEU B 918 32.801 20.024 48.303 1.00 29.40 C
ANISOU 4087 CD2 LEU B 918 3795 3596 3781 133 -63 186 C
ATOM 4088 H LEU B 918 30.428 19.685 44.495 1.00 24.44 H

```
ATOM   4089  HA  LEU B 918      30.435  20.916  47.209  1.00 23.25           H
ATOM   4090  HB3 LEU B 918      32.533  22.099  46.379  1.00 22.85           H
ATOM   4091  HB2 LEU B 918      32.512  21.073  44.974  1.00 22.85           H
ATOM   4092  HG  LEU B 918      32.810  19.086  46.384  1.00 26.41           H
ATOM   4093 HD11 LEU B 918      35.085  19.256  46.421  1.00 25.11           H
ATOM   4094 HD12 LEU B 918      34.768  20.748  45.558  1.00 25.11           H
ATOM   4095 HD13 LEU B 918      35.100  20.792  47.278  1.00 25.11           H
ATOM   4096 HD21 LEU B 918      33.710  19.919  48.893  1.00 29.40           H
ATOM   4097 HD22 LEU B 918      32.295  20.926  48.648  1.00 29.40           H
ATOM   4098 HD23 LEU B 918      32.169  19.172  48.551  1.00 29.40           H
ATOM   4099  N   ILE B 919      29.517  23.067  46.343  1.00 23.53           N
ANISOU 4099  N   ILE B 919     2931   3061   2948    144    -22    217       N
ATOM   4100  CA  ILE B 919      28.769  24.257  45.932  1.00 23.06           C
ANISOU 4100  CA  ILE B 919     2858   3064   2838    210     -1    235       C
ATOM   4101  C   ILE B 919      29.220  25.462  46.786  1.00 26.67           C
ANISOU 4101  C   ILE B 919     3413   3462   3257    292      6    223       C
ATOM   4102  O   ILE B 919      29.477  25.306  47.979  1.00 26.37           O
ANISOU 4102  O   ILE B 919     3423   3387   3210    307      1    225       O
ATOM   4103  CB  ILE B 919      27.230  24.020  46.082  1.00 26.46           C
ANISOU 4103  CB  ILE B 919     3204   3638   3211    221     25    305       C
ATOM   4104  CG1 ILE B 919      26.383  25.035  45.285  1.00 27.18           C
ANISOU 4104  CG1 ILE B 919     3251   3832   3246    288     50    336       C
ATOM   4105  CG2 ILE B 919      26.739  23.939  47.544  1.00 27.81           C
ANISOU 4105  CG2 ILE B 919     3385   3844   3337    286     47    348       C
ATOM   4106  CD1 ILE B 919      24.898  24.651  45.185  1.00 37.61           C
ANISOU 4106  CD1 ILE B 919     4435   5358   4497    268     67    422       C
ATOM   4107  H   ILE B 919      29.652  22.958  47.341  1.00 23.53           H
ATOM   4108  HA  ILE B 919      29.001  24.487  44.891  1.00 23.06           H
ATOM   4109  HB  ILE B 919      27.032  23.043  45.638  1.00 26.46           H
ATOM   4110 HG13 ILE B 919      26.774  25.118  44.275  1.00 27.18           H
ATOM   4111 HG12 ILE B 919      26.476  26.029  45.722  1.00 27.18           H
ATOM   4112 HG21 ILE B 919      25.693  23.644  47.606  1.00 27.81           H
ATOM   4113 HG22 ILE B 919      27.311  23.200  48.104  1.00 27.81           H
ATOM   4114 HG23 ILE B 919      26.817  24.899  48.054  1.00 27.81           H
ATOM   4115 HD11 ILE B 919      24.392  25.233  44.419  1.00 37.61           H
ATOM   4116 HD12 ILE B 919      24.771  23.598  44.934  1.00 37.61           H
ATOM   4117 HD13 ILE B 919      24.375  24.838  46.122  1.00 37.61           H
ATOM   4118  N   LEU B 920      29.313  26.639  46.153  1.00 23.23           N
ANISOU 4118  N   LEU B 920     3030   3008   2787    335     15    210       N
ATOM   4119  CA  LEU B 920      29.732  27.898  46.774  1.00 23.34           C
ANISOU 4119  CA  LEU B 920     3189   2942   2737    391     20    198       C
ATOM   4120  C   LEU B 920      28.521  28.730  47.215  1.00 26.61           C
ANISOU 4120  C   LEU B 920     3667   3395   3048    537     73    244       C
ATOM   4121  O   LEU B 920      27.449  28.622  46.622  1.00 26.66           O
ANISOU 4121  O   LEU B 920     3590   3509   3028    601    105    286       O
ATOM   4122  CB  LEU B 920      30.533  28.724  45.738  1.00 23.27           C
ANISOU 4122  CB  LEU B 920     3251   2861   2730    339     -3    156       C
ATOM   4123  CG  LEU B 920      31.920  28.147  45.395  1.00 26.16           C
ANISOU 4123  CG  LEU B 920     3568   3205   3167    222    -50    121       C
ATOM   4124  CD1 LEU B 920      32.459  28.733  44.072  1.00 25.52           C
ANISOU 4124  CD1 LEU B 920     3491   3111   3093    177    -60     96       C
ATOM   4125  CD2 LEU B 920      32.911  28.315  46.570  1.00 25.29           C
ANISOU 4125  CD2 LEU B 920     3531   3047   3030    174    -82    112       C
ATOM   4126  H   LEU B 920      29.079  26.696  45.169  1.00 23.23           H
ATOM   4127  HA  LEU B 920      30.359  27.698  47.644  1.00 23.34           H
ATOM   4128  HB3 LEU B 920      30.666  29.751  46.082  1.00 23.27           H
```

ATOM 4129 HB2 LEU B 920 29.934 28.802 44.832 1.00 23.27 H
ATOM 4130 HG LEU B 920 31.786 27.081 45.220 1.00 26.16 H
ATOM 4131 HD11 LEU B 920 32.616 27.941 43.339 1.00 25.52 H
ATOM 4132 HD12 LEU B 920 31.769 29.449 43.623 1.00 25.52 H
ATOM 4133 HD13 LEU B 920 33.409 29.251 44.191 1.00 25.52 H
ATOM 4134 HD21 LEU B 920 33.155 27.353 47.017 1.00 25.29 H
ATOM 4135 HD22 LEU B 920 33.853 28.775 46.273 1.00 25.29 H
ATOM 4136 HD23 LEU B 920 32.496 28.936 47.363 1.00 25.29 H
ATOM 4137 N LEU B 921 28.776 29.619 48.185 1.00 23.13 N
ANISOU 4137 N LEU B 921 3384 2872 2531 597 85 241 N
ATOM 4138 CA LEU B 921 27.993 30.821 48.454 1.00 23.80 C
ANISOU 4138 CA LEU B 921 3604 2952 2486 768 146 279 C
ATOM 4139 C LEU B 921 28.977 31.993 48.409 1.00 30.10 C
ANISOU 4139 C LEU B 921 4635 3586 3216 742 131 232 C
ATOM 4140 O LEU B 921 29.944 31.995 49.172 1.00 30.08 O
ANISOU 4140 O LEU B 921 4729 3487 3213 631 86 194 O
ATOM 4141 CB LEU B 921 27.318 30.740 49.840 1.00 23.41 C
ANISOU 4141 CB LEU B 921 3584 2933 2376 862 178 319 C
ATOM 4142 CG LEU B 921 26.043 29.880 49.880 1.00 27.68 C
ANISOU 4142 CG LEU B 921 3943 3668 2907 950 221 399 C
ATOM 4143 CD1 LEU B 921 25.554 29.678 51.329 1.00 28.29 C
ANISOU 4143 CD1 LEU B 921 4046 3768 2933 1017 246 433 C
ATOM 4144 CD2 LEU B 921 24.936 30.463 48.981 1.00 30.87 C
ANISOU 4144 CD2 LEU B 921 4338 4186 3207 1129 287 464 C
ATOM 4145 H LEU B 921 29.683 29.596 48.635 1.00 23.13 H
ATOM 4146 HA LEU B 921 27.242 30.971 47.679 1.00 23.80 H
ATOM 4147 HB3 LEU B 921 27.058 31.740 50.188 1.00 23.41 H
ATOM 4148 HB2 LEU B 921 28.044 30.364 50.555 1.00 23.41 H
ATOM 4149 HG LEU B 921 26.303 28.894 49.495 1.00 27.68 H
ATOM 4150 HD11 LEU B 921 25.378 28.623 51.535 1.00 28.29 H
ATOM 4151 HD12 LEU B 921 26.279 30.033 52.062 1.00 28.29 H
ATOM 4152 HD13 LEU B 921 24.621 30.205 51.528 1.00 28.29 H
ATOM 4153 HD21 LEU B 921 23.941 30.248 49.365 1.00 30.87 H
ATOM 4154 HD22 LEU B 921 25.024 31.542 48.863 1.00 30.87 H
ATOM 4155 HD23 LEU B 921 24.991 30.034 47.982 1.00 30.87 H
ATOM 4156 N GLY B 922 28.712 32.960 47.519 1.00 28.87 N
ANISOU 4156 N GLY B 922 4563 3409 2999 819 162 240 N
ATOM 4157 CA GLY B 922 29.531 34.158 47.371 1.00 29.39 C
ANISOU 4157 CA GLY B 922 4879 3309 2980 776 150 200 C
ATOM 4158 C GLY B 922 28.627 35.388 47.432 1.00 33.98 C
ANISOU 4158 C GLY B 922 5706 3815 3389 980 225 233 C
ATOM 4159 O GLY B 922 27.505 35.372 46.924 1.00 32.04 O
ANISOU 4159 O GLY B 922 5382 3689 3102 1173 291 295 O
ATOM 4160 H GLY B 922 27.894 32.888 46.928 1.00 28.87 H
ATOM 4161 HA3 GLY B 922 30.041 34.132 46.408 1.00 29.39 H
ATOM 4162 HA2 GLY B 922 30.296 34.236 48.144 1.00 29.39 H
ATOM 4163 N GLU B 923 29.167 36.470 48.015 1.00 32.57 N
ANISOU 4163 N GLU B 923 5840 3445 3090 937 216 198 N
ATOM 4164 CA GLU B 923 28.612 37.819 47.981 1.00 33.06 C
ANISOU 4164 CA GLU B 923 6223 3381 2959 1130 291 221 C
ATOM 4165 C GLU B 923 28.884 38.425 46.594 1.00 37.30 C
ANISOU 4165 C GLU B 923 6827 3866 3478 1103 291 210 C
ATOM 4166 O GLU B 923 30.049 38.596 46.232 1.00 37.03 O
ANISOU 4166 O GLU B 923 6851 3743 3476 873 221 158 O
ATOM 4167 CB GLU B 923 29.254 38.653 49.113 1.00 34.48 C
ANISOU 4167 CB GLU B 923 6753 3351 2996 1061 276 185 C

```
ATOM   4168  CG  GLU B 923      28.748  40.116  49.193  1.00 43.50           C
ANISOU 4168  CG  GLU B 923     8300   4321   3909   1271    361    205       C
ATOM   4169  CD  GLU B 923      29.489  41.035  50.181  1.00 61.21           C
ANISOU 4169  CD  GLU B 923    10969   6316   5971   1181    345    164       C
ATOM   4170  OE1 GLU B 923      30.498  40.615  50.789  1.00 48.07           O
ANISOU 4170  OE1 GLU B 923     9275   4642   4346    994    277    131       O
ATOM   4171  OE2 GLU B 923      29.039  42.196  50.296  1.00 55.30           O1-
ANISOU 4171  OE2 GLU B 923    10608   5380   5025   1303    405    168       O1-
ATOM   4172  H   GLU B 923      30.109  36.406  48.378  1.00 32.57           H
ATOM   4173  HA  GLU B 923      27.536  37.766  48.159  1.00 33.06           H
ATOM   4174  HB3 GLU B 923      30.337  38.643  48.983  1.00 34.48           H
ATOM   4175  HB2 GLU B 923      29.063  38.162  50.069  1.00 34.48           H
ATOM   4176  HG3 GLU B 923      27.689  40.113  49.450  1.00 43.50           H
ATOM   4177  HG2 GLU B 923      28.813  40.589  48.213  1.00 43.50           H
ATOM   4178  N   VAL B 924      27.807  38.720  45.853  1.00 33.38           N
ANISOU 4178  N   VAL B 924     6301   3453   2927   1334    368    268       N
ATOM   4179  CA  VAL B 924      27.867  39.261  44.499  1.00 32.85           C
ANISOU 4179  CA  VAL B 924     6288   3353   2841   1333    374    265       C
ATOM   4180  C   VAL B 924      27.240  40.663  44.516  1.00 37.68           C
ANISOU 4180  C   VAL B 924     7286   3805   3225   1563    462    297       C
ATOM   4181  O   VAL B 924      26.056  40.791  44.832  1.00 35.12           O
ANISOU 4181  O   VAL B 924     6997   3556   2792   1853    552    368       O
ATOM   4182  CB  VAL B 924      27.069  38.382  43.493  1.00 35.76           C
ANISOU 4182  CB  VAL B 924     6291   3960   3335   1381    379    307       C
ATOM   4183  CG1 VAL B 924      27.187  38.884  42.040  1.00 35.28           C
ANISOU 4183  CG1 VAL B 924     6262   3863   3280   1328    367    292       C
ATOM   4184  CG2 VAL B 924      27.495  36.907  43.552  1.00 35.33           C
ANISOU 4184  CG2 VAL B 924     5908   4043   3472   1193    309    283       C
ATOM   4185  H   VAL B 924      26.880  38.536  46.217  1.00 33.38           H
ATOM   4186  HA  VAL B 924      28.898  39.330  44.149  1.00 32.85           H
ATOM   4187  HB  VAL B 924      26.011  38.408  43.761  1.00 35.76           H
ATOM   4188 HG11 VAL B 924      26.625  38.247  41.357  1.00 35.28           H
ATOM   4189 HG12 VAL B 924      26.806  39.898  41.921  1.00 35.28           H
ATOM   4190 HG13 VAL B 924      28.227  38.882  41.710  1.00 35.28           H
ATOM   4191 HG21 VAL B 924      26.948  36.317  42.819  1.00 35.33           H
ATOM   4192 HG22 VAL B 924      28.561  36.793  43.354  1.00 35.33           H
ATOM   4193 HG23 VAL B 924      27.285  36.464  44.525  1.00 35.33           H
ATOM   4194  N   ALA B 925      28.045  41.679  44.168  1.00 37.50           N
ANISOU 4194  N   ALA B 925     7560   3573   3117   1439    439    253       N
ATOM   4195  CA  ALA B 925      27.626  43.075  44.053  1.00 38.67           C
ANISOU 4195  CA  ALA B 925     8141   3524   3030   1638    521    276       C
ATOM   4196  C   ALA B 925      26.960  43.318  42.691  1.00 42.53           C
ANISOU 4196  C   ALA B 925     8530   4118   3510   1816    574    330       C
ATOM   4197  O   ALA B 925      27.654  43.601  41.714  1.00 42.94           O
ANISOU 4197  O   ALA B 925     8582   4123   3609   1650    529    297       O
ATOM   4198  CB  ALA B 925      28.856  43.972  44.253  1.00 39.50           C
ANISOU 4198  CB  ALA B 925     8626   3352   3030   1379    463    208       C
ATOM   4199  H   ALA B 925      29.007  41.484  43.922  1.00 37.50           H
ATOM   4200  HA  ALA B 925      26.906  43.310  44.839  1.00 38.67           H
ATOM   4201  HB1 ALA B 925      28.592  45.024  44.161  1.00 39.50           H
ATOM   4202  HB2 ALA B 925      29.280  43.839  45.246  1.00 39.50           H
ATOM   4203  HB3 ALA B 925      29.640  43.752  43.527  1.00 39.50           H
ATOM   4204  N   LEU B 926      25.626  43.162  42.649  1.00 39.24           N
ANISOU 4204  N   LEU B 926     7992   3881   3036   2149    668    420       N
ATOM   4205  CA  LEU B 926      24.831  43.205  41.420  1.00 39.41           C
ANISOU 4205  CA  LEU B 926     7858   4072   3043   2346    721    492       C
```

```
ATOM   4206  C   LEU B 926      24.607  44.625  40.879  1.00 45.95           C
ANISOU 4206  C   LEU B 926     9101   4714   3643   2570    808    525       C
ATOM   4207  O   LEU B 926      24.504  44.772  39.662  1.00 45.34           O
ANISOU 4207  O   LEU B 926     8965   4688   3576   2595    813    546       O
ATOM   4208  CB  LEU B 926      23.472  42.511  41.651  1.00 38.95           C
ANISOU 4208  CB  LEU B 926     7452   4343   3004   2586    778    595       C
ATOM   4209  CG  LEU B 926      23.559  41.024  42.058  1.00 42.95           C
ANISOU 4209  CG  LEU B 926     7547   5047   3725   2373    699    575       C
ATOM   4210  CD1 LEU B 926      22.227  40.515  42.638  1.00 42.90           C
ANISOU 4210  CD1 LEU B 926     7315   5315   3672   2607    764    682       C
ATOM   4211  CD2 LEU B 926      24.029  40.143  40.892  1.00 44.52           C
ANISOU 4211  CD2 LEU B 926     7433   5366   4117   2133    614    540       C
ATOM   4212  H   LEU B 926      25.125  42.916  43.493  1.00 39.24           H
ATOM   4213  HA  LEU B 926      25.375  42.660  40.648  1.00 39.41           H
ATOM   4214  HB3 LEU B 926      22.858  42.587  40.755  1.00 38.95           H
ATOM   4215  HB2 LEU B 926      22.932  43.066  42.416  1.00 38.95           H
ATOM   4216  HG  LEU B 926      24.296  40.922  42.854  1.00 42.95           H
ATOM   4217 HD11 LEU B 926      21.881  39.610  42.142  1.00 42.90           H
ATOM   4218 HD12 LEU B 926      22.345  40.277  43.694  1.00 42.90           H
ATOM   4219 HD13 LEU B 926      21.429  41.255  42.560  1.00 42.90           H
ATOM   4220 HD21 LEU B 926      24.135  39.107  41.213  1.00 44.52           H
ATOM   4221 HD22 LEU B 926      23.319  40.178  40.066  1.00 44.52           H
ATOM   4222 HD23 LEU B 926      24.996  40.457  40.502  1.00 44.52           H
ATOM   4223  N   GLY B 927      24.531  45.634  41.766  1.00 44.24           N
ANISOU 4223  N   GLY B 927     9317   4278   3212   2738    878    532       N
ATOM   4224  CA  GLY B 927      24.298  47.039  41.419  1.00 44.37           C
ANISOU 4224  CA  GLY B 927     9808   4078   2973   2983    975    566       C
ATOM   4225  C   GLY B 927      22.976  47.215  40.658  1.00 48.02           C
ANISOU 4225  C   GLY B 927    10113   4773   3359   3354   1075    685       C
ATOM   4226  O   GLY B 927      21.957  46.625  41.017  1.00 48.74           O
ANISOU 4226  O   GLY B 927     9880   5167   3471   3575   1124    776       O
ATOM   4227  H   GLY B 927      24.620  45.428  42.752  1.00 44.24           H
ATOM   4228  HA3 GLY B 927      25.139  47.413  40.835  1.00 44.37           H
ATOM   4229  HA2 GLY B 927      24.257  47.641  42.324  1.00 44.37           H
ATOM   4230  N   ASN B 928      23.015  48.050  39.612  1.00 42.35           N
ANISOU 4230  N   ASN B 928     9596   3940   2556   3397   1097    693       N
ATOM   4231  CA  ASN B 928      21.921  48.283  38.675  1.00 42.47           C
ANISOU 4231  CA  ASN B 928     9490   4163   2483   3732   1186    807       C
ATOM   4232  C   ASN B 928      21.823  47.094  37.696  1.00 47.01           C
ANISOU 4232  C   ASN B 928     9482   5069   3312   3545   1100    815       C
ATOM   4233  O   ASN B 928      22.699  46.931  36.845  1.00 44.94           O
ANISOU 4233  O   ASN B 928     9158   4730   3186   3242   1010    738       O
ATOM   4234  CB  ASN B 928      22.199  49.629  37.967  1.00 44.37           C
ANISOU 4234  CB  ASN B 928    10219   4113   2527   3824   1238    803       C
ATOM   4235  CG  ASN B 928      21.032  50.215  37.166  1.00 68.81           C
ANISOU 4235  CG  ASN B 928    13292   7385   5469   4242   1353    935       C
ATOM   4236  ND2 ASN B 928      21.305  51.296  36.433  1.00 65.81           N
ANISOU 4236  ND2 ASN B 928    13275   6775   4953   4281   1384    928       N
ATOM   4237  OD1 ASN B 928      19.909  49.715  37.197  1.00 59.43           O
ANISOU 4237  OD1 ASN B 928    11764   6550   4266   4529   1416   1052       O
ATOM   4238  H   ASN B 928      23.891  48.501  39.377  1.00 42.35           H
ATOM   4239  HA  ASN B 928      20.976  48.357  39.219  1.00 42.47           H
ATOM   4240  HB3 ASN B 928      23.083  49.551  37.335  1.00 44.37           H
ATOM   4241  HB2 ASN B 928      22.442  50.378  38.723  1.00 44.37           H
ATOM   4242 HD22 ASN B 928      20.579  51.753  35.901  1.00 65.81           H
ATOM   4243 HD21 ASN B 928      22.246  51.667  36.409  1.00 65.81           H
```

ATOM	4244	N	MET	B	929	20.780	46.266	37.869	1.00	44.16		N
ANISOU	4244	N	MET	B	929	8703	5079	2995	3718	1129	912	N
ATOM	4245	CA	MET	B	929	20.572	45.026	37.115	1.00	43.36		C
ANISOU	4245	CA	MET	B	929	8063	5304	3108	3538	1050	927	C
ATOM	4246	C	MET	B	929	19.882	45.292	35.768	1.00	47.85		C
ANISOU	4246	C	MET	B	929	8494	6063	3625	3694	1083	1011	C
ATOM	4247	O	MET	B	929	18.878	46.005	35.728	1.00	48.88		O
ANISOU	4247	O	MET	B	929	8738	6296	3539	4090	1199	1134	O
ATOM	4248	CB	MET	B	929	19.736	44.036	37.955	1.00	45.27		C
ANISOU	4248	CB	MET	B	929	7928	5867	3407	3598	1054	998	C
ATOM	4249	CG	MET	B	929	20.306	43.763	39.358	1.00	48.40		C
ANISOU	4249	CG	MET	B	929	8407	6109	3874	3430	1014	918	C
ATOM	4250	SD	MET	B	929	19.562	42.359	40.233	1.00	52.27		S
ANISOU	4250	SD	MET	B	929	8451	6973	4437	3462	1011	1000	S
ATOM	4251	CE	MET	B	929	17.875	42.980	40.466	1.00	49.11		C
ANISOU	4251	CE	MET	B	929	8160	6763	3735	4012	1181	1177	C
ATOM	4252	H	MET	B	929	20.099	46.466	38.587	1.00	44.16		H
ATOM	4253	HA	MET	B	929	21.545	44.566	36.932	1.00	43.36		H
ATOM	4254	HB3	MET	B	929	19.645	43.091	37.417	1.00	45.27		H
ATOM	4255	HB2	MET	B	929	18.720	44.419	38.059	1.00	45.27		H
ATOM	4256	HG3	MET	B	929	20.201	44.646	39.990	1.00	48.40		H
ATOM	4257	HG2	MET	B	929	21.375	43.572	39.280	1.00	48.40		H
ATOM	4258	HE1	MET	B	929	17.293	42.269	41.052	1.00	49.11		H
ATOM	4259	HE2	MET	B	929	17.889	43.935	40.992	1.00	49.11		H
ATOM	4260	HE3	MET	B	929	17.378	43.114	39.506	1.00	49.11		H
ATOM	4261	N	TYR	B	930	20.419	44.683	34.699	1.00	42.95		N
ANISOU	4261	N	TYR	B	930	7620	5506	3193	3398	983	953	N
ATOM	4262	CA	TYR	B	930	19.859	44.699	33.349	1.00	41.84		C
ANISOU	4262	CA	TYR	B	930	7294	5570	3032	3487	993	1024	C
ATOM	4263	C	TYR	B	930	18.980	43.452	33.166	1.00	45.19		C
ANISOU	4263	C	TYR	B	930	7193	6415	3561	3438	954	1098	C
ATOM	4264	O	TYR	B	930	19.487	42.383	32.826	1.00	45.00		O
ANISOU	4264	O	TYR	B	930	6921	6430	3745	3104	850	1018	O
ATOM	4265	CB	TYR	B	930	21.012	44.795	32.321	1.00	42.32		C
ANISOU	4265	CB	TYR	B	930	7439	5426	3215	3187	911	914	C
ATOM	4266	CG	TYR	B	930	20.628	44.825	30.846	1.00	43.46		C
ANISOU	4266	CG	TYR	B	930	7401	5758	3355	3237	909	971	C
ATOM	4267	CD1	TYR	B	930	19.462	45.491	30.404	1.00	45.43		C
ANISOU	4267	CD1	TYR	B	930	7687	6177	3397	3624	1014	1111	C
ATOM	4268	CD2	TYR	B	930	21.461	44.196	29.896	1.00	43.67		C
ANISOU	4268	CD2	TYR	B	930	7245	5782	3566	2912	809	888	C
ATOM	4269	CE1	TYR	B	930	19.122	45.503	29.038	1.00	46.65		C
ANISOU	4269	CE1	TYR	B	930	7673	6513	3538	3666	1009	1167	C
ATOM	4270	CE2	TYR	B	930	21.120	44.204	28.529	1.00	44.06		C
ANISOU	4270	CE2	TYR	B	930	7146	5991	3603	2949	806	936	C
ATOM	4271	CZ	TYR	B	930	19.949	44.856	28.100	1.00	50.28		C
ANISOU	4271	CZ	TYR	B	930	7951	6960	4192	3317	902	1075	C
ATOM	4272	OH	TYR	B	930	19.622	44.865	26.776	1.00	49.23		O
ANISOU	4272	OH	TYR	B	930	7663	7001	4041	3351	894	1128	O
ATOM	4273	H	TYR	B	930	21.266	44.141	34.814	1.00	42.95		H
ATOM	4274	HA	TYR	B	930	19.234	45.586	33.238	1.00	41.84		H
ATOM	4275	HB3	TYR	B	930	21.701	43.964	32.476	1.00	42.32		H
ATOM	4276	HB2	TYR	B	930	21.597	45.689	32.523	1.00	42.32		H
ATOM	4277	HD1	TYR	B	930	18.813	45.995	31.103	1.00	45.43		H
ATOM	4278	HD2	TYR	B	930	22.373	43.709	30.206	1.00	43.67		H
ATOM	4279	HE1	TYR	B	930	18.224	46.008	28.715	1.00	46.65		H

ATOM 4280 HE2 TYR B 930 21.762 43.713 27.812 1.00 44.06 H
ATOM 4281 HH TYR B 930 18.802 45.329 26.591 1.00 49.23 H
ATOM 4282 N GLU B 931 17.680 43.615 33.448 1.00 41.57 N
ANISOU 4282 N GLU B 931 6586 6272 2935 3778 1043 1258 N
ATOM 4283 CA GLU B 931 16.688 42.543 33.452 1.00 41.07 C
ANISOU 4283 CA GLU B 931 6046 6645 2915 3751 1016 1357 C
ATOM 4284 C GLU B 931 16.318 42.085 32.029 1.00 44.41 C
ANISOU 4284 C GLU B 931 6143 7339 3393 3631 959 1401 C
ATOM 4285 O GLU B 931 15.891 42.908 31.219 1.00 45.29 O
ANISOU 4285 O GLU B 931 6296 7552 3358 3869 1018 1491 O
ATOM 4286 CB GLU B 931 15.466 42.994 34.272 1.00 42.08 C
ANISOU 4286 CB GLU B 931 6142 7027 2819 4161 1137 1523 C
ATOM 4287 CG GLU B 931 15.816 43.238 35.761 1.00 50.41 C
ANISOU 4287 CG GLU B 931 7503 7824 3828 4250 1186 1473 C
ATOM 4288 CD GLU B 931 14.636 43.564 36.688 1.00 70.08 C
ANISOU 4288 CD GLU B 931 10024 10517 6088 4674 1317 1626 C
ATOM 4289 OE1 GLU B 931 13.514 43.803 36.187 1.00 61.32 O
ANISOU 4289 OE1 GLU B 931 8726 9768 4806 4983 1395 1799 O
ATOM 4290 OE2 GLU B 931 14.883 43.559 37.915 1.00 71.30 O1-
ANISOU 4290 OE2 GLU B 931 10390 10479 6221 4706 1344 1579 O1-
ATOM 4291 H GLU B 931 17.343 44.523 33.734 1.00 41.57 H
ATOM 4292 HA GLU B 931 17.128 41.698 33.979 1.00 41.07 H
ATOM 4293 HB3 GLU B 931 14.683 42.242 34.197 1.00 42.08 H
ATOM 4294 HB2 GLU B 931 15.052 43.907 33.840 1.00 42.08 H
ATOM 4295 HG3 GLU B 931 16.539 44.050 35.848 1.00 50.41 H
ATOM 4296 HG2 GLU B 931 16.308 42.350 36.155 1.00 50.41 H
ATOM 4297 N LEU B 932 16.513 40.783 31.764 1.00 39.33 N
ANISOU 4297 N LEU B 932 5197 6802 2947 3265 845 1339 N
ATOM 4298 CA LEU B 932 16.331 40.136 30.462 1.00 38.69 C
ANISOU 4298 CA LEU B 932 4820 6942 2937 3069 770 1355 C
ATOM 4299 C LEU B 932 15.551 38.821 30.641 1.00 42.83 C
ANISOU 4299 C LEU B 932 4932 7846 3496 2908 714 1431 C
ATOM 4300 O LEU B 932 15.745 38.133 31.641 1.00 41.20 O
ANISOU 4300 O LEU B 932 4666 7624 3363 2784 688 1400 O
ATOM 4301 CB LEU B 932 17.720 39.850 29.839 1.00 38.16 C
ANISOU 4301 CB LEU B 932 4861 6569 3069 2732 677 1183 C
ATOM 4302 CG LEU B 932 18.532 41.101 29.425 1.00 41.96 C
ANISOU 4302 CG LEU B 932 5732 6691 3520 2809 712 1106 C
ATOM 4303 CD1 LEU B 932 19.987 40.734 29.068 1.00 41.51 C
ANISOU 4303 CD1 LEU B 932 5745 6365 3664 2453 619 944 C
ATOM 4304 CD2 LEU B 932 17.836 41.906 28.307 1.00 44.22 C
ANISOU 4304 CD2 LEU B 932 6049 7098 3654 3043 768 1202 C
ATOM 4305 H LEU B 932 16.866 40.180 32.498 1.00 39.33 H
ATOM 4306 HA LEU B 932 15.748 40.779 29.801 1.00 38.69 H
ATOM 4307 HB3 LEU B 932 17.603 39.212 28.961 1.00 38.16 H
ATOM 4308 HB2 LEU B 932 18.303 39.267 30.556 1.00 38.16 H
ATOM 4309 HG LEU B 932 18.601 41.755 30.294 1.00 41.96 H
ATOM 4310 HD11 LEU B 932 20.241 40.953 28.031 1.00 41.51 H
ATOM 4311 HD12 LEU B 932 20.686 41.291 29.691 1.00 41.51 H
ATOM 4312 HD13 LEU B 932 20.192 39.678 29.228 1.00 41.51 H
ATOM 4313 HD21 LEU B 932 18.505 42.154 27.482 1.00 44.22 H
ATOM 4314 HD22 LEU B 932 16.992 41.367 27.877 1.00 44.22 H
ATOM 4315 HD23 LEU B 932 17.454 42.849 28.698 1.00 44.22 H
ATOM 4316 N LYS B 933 14.701 38.487 29.656 1.00 41.14 N
ANISOU 4316 N LYS B 933 4441 7972 3218 2890 689 1532 N
ATOM 4317 CA LYS B 933 13.853 37.286 29.660 1.00 40.81 C

```
ANISOU 4317  CA  LYS B 933     4004   8337   3166   2711    629   1627        C
ATOM   4318  C   LYS B 933     14.395  36.153  28.769  1.00 44.61           C
ANISOU 4318  C   LYS B 933     4346   8793   3811   2289    504   1527        C
ATOM   4319  O   LYS B 933     13.991  35.006  28.961  1.00 44.91           O
ANISOU 4319  O   LYS B 933     4149   9030   3884   2043    436   1550        O
ATOM   4320  CB  LYS B 933     12.440  37.669  29.174  1.00 43.00           C
ANISOU 4320  CB  LYS B 933     4047   9083   3208   2991    691   1843        C
ATOM   4321  CG  LYS B 933     11.702  38.665  30.081  1.00 53.35           C
ANISOU 4321  CG  LYS B 933     5430  10523   4318   3431    824   1982        C
ATOM   4322  CD  LYS B 933     10.252  38.882  29.622  1.00 66.17           C
ANISOU 4322  CD  LYS B 933     6775  12667   5698   3707    882   2213        C
ATOM   4323  CE  LYS B 933      9.577  40.073  30.320  1.00 76.28           C
ANISOU 4323  CE  LYS B 933     8118  14113   6752   4187   1027   2371        C
ATOM   4324  NZ  LYS B 933      8.165  40.206  29.921  1.00 83.37           N1+
ANISOU 4324  NZ  LYS B 933     8683  15593   7399   4452   1081   2621        N1+
ATOM   4325  H   LYS B 933     14.596  39.102  28.862  1.00 41.14           H
ATOM   4326  HA  LYS B 933     13.765  36.888  30.673  1.00 40.81           H
ATOM   4327  HB3 LYS B 933     11.832  36.769  29.101  1.00 43.00           H
ATOM   4328  HB2 LYS B 933     12.495  38.076  28.163  1.00 43.00           H
ATOM   4329  HG3 LYS B 933     12.232  39.618  30.088  1.00 53.35           H
ATOM   4330  HG2 LYS B 933     11.707  38.299  31.108  1.00 53.35           H
ATOM   4331  HD3 LYS B 933      9.686  37.970  29.820  1.00 66.17           H
ATOM   4332  HD2 LYS B 933     10.228  39.025  28.540  1.00 66.17           H
ATOM   4333  HE3 LYS B 933     10.099  40.998  30.076  1.00 76.28           H
ATOM   4334  HE2 LYS B 933      9.627  39.959  31.402  1.00 76.28           H
ATOM   4335  HZ1 LYS B 933      7.671  39.351  30.142  1.00 83.37           H
ATOM   4336  HZ2 LYS B 933      7.742  40.979  30.414  1.00 83.37           H
ATOM   4337  HZ3 LYS B 933      8.108  40.370  28.926  1.00 83.37           H
ATOM   4338  N   HIE B 934     15.256  36.490  27.795  1.00 39.96           N
ANISOU 4338  N   HIE B 934     3912   7968   3303   2211    477   1424        N
ATOM   4339  CA  HIE B 934     15.776  35.581  26.769  1.00 38.75           C
ANISOU 4339  CA  HIE B 934     3670   7771   3284   1858    372   1328        C
ATOM   4340  C   HIE B 934     17.265  35.861  26.518  1.00 39.19           C
ANISOU 4340  C   HIE B 934     4007   7387   3498   1750    353   1152        C
ATOM   4341  O   HIE B 934     17.779  36.889  26.964  1.00 39.30           O
ANISOU 4341  O   HIE B 934     4282   7152   3499   1940    416   1115        O
ATOM   4342  CB  HIE B 934     14.953  35.738  25.469  1.00 39.98           C
ANISOU 4342  CB  HIE B 934     3630   8242   3319   1880    356   1435        C
ATOM   4343  CG  HIE B 934     13.491  35.385  25.596  1.00 43.91           C
ANISOU 4343  CG  HIE B 934     3805   9232   3645   1941    360   1624        C
ATOM   4344  CD2 HIE B 934     12.408  36.226  25.752  1.00 46.31           C
ANISOU 4344  CD2 HIE B 934     4014   9833   3747   2294    451   1802        C
ATOM   4345  ND1 HIE B 934     13.036  34.063  25.595  1.00 45.96           N
ANISOU 4345  ND1 HIE B 934     3815   9728   3920   1610    267   1649        N
ATOM   4346  CE1 HIE B 934     11.725  34.152  25.771  1.00 45.65           C
ANISOU 4346  CE1 HIE B 934     3510  10144   3690   1744    295   1844        C
ATOM   4347  NE2 HIE B 934     11.298  35.410  25.873  1.00 46.18           N
ANISOU 4347  NE2 HIE B 934     3648  10278   3621   2174    409   1946        N
ATOM   4348  H   HIE B 934     15.572  37.446  27.722  1.00 39.96           H
ATOM   4349  HA  HIE B 934     15.702  34.548  27.117  1.00 38.75           H
ATOM   4350  HB3 HIE B 934     15.371  35.111  24.680  1.00 39.98           H
ATOM   4351  HB2 HIE B 934     15.024  36.765  25.106  1.00 39.98           H
ATOM   4352  HD2 HIE B 934     12.343  37.303  25.806  1.00 46.31           H
ATOM   4353  HE1 HIE B 934     11.073  33.293  25.838  1.00 45.65           H
ATOM   4354  N   ALA B 935     17.920  34.933  25.795  1.00 33.00           N
ANISOU 4354  N   ALA B 935     3180   6514   2846   1443   267   1049        N
```

ATOM 4355 CA ALA B 935 19.339 34.966 25.429 1.00 32.16 C
ANISOU 4355 CA ALA B 935 3292 6041 2887 1313 243 893 C
ATOM 4356 C ALA B 935 19.769 36.269 24.731 1.00 35.92 C
ANISOU 4356 C ALA B 935 3983 6354 3314 1504 297 883 C
ATOM 4357 O ALA B 935 19.057 36.763 23.856 1.00 36.37 O
ANISOU 4357 O ALA B 935 3984 6588 3246 1660 327 976 O
ATOM 4358 CB ALA B 935 19.645 33.757 24.531 1.00 32.80 C
ANISOU 4358 CB ALA B 935 3276 6115 3072 1004 156 815 C
ATOM 4359 H ALA B 935 17.407 34.129 25.463 1.00 33.00 H
ATOM 4360 HA ALA B 935 19.906 34.860 26.357 1.00 32.16 H
ATOM 4361 HB1 ALA B 935 20.713 33.683 24.321 1.00 32.80 H
ATOM 4362 HB2 ALA B 935 19.340 32.824 25.004 1.00 32.80 H
ATOM 4363 HB3 ALA B 935 19.122 33.826 23.576 1.00 32.80 H
ATOM 4364 N SER B 936 20.932 36.789 25.146 1.00 31.81 N
ANISOU 4364 N SER B 936 3711 5503 2872 1489 310 780 N
ATOM 4365 CA SER B 936 21.488 38.058 24.693 1.00 31.72 C
ANISOU 4365 CA SER B 936 3960 5276 2815 1617 355 754 C
ATOM 4366 C SER B 936 23.009 37.983 24.875 1.00 33.13 C
ANISOU 4366 C SER B 936 4300 5148 3139 1402 314 613 C
ATOM 4367 O SER B 936 23.504 38.249 25.969 1.00 31.59 O
ANISOU 4367 O SER B 936 4224 4798 2980 1384 320 570 O
ATOM 4368 CB SER B 936 20.818 39.215 25.472 1.00 37.55 C
ANISOU 4368 CB SER B 936 4883 5986 3398 1932 448 835 C
ATOM 4369 OG SER B 936 21.283 40.478 25.039 1.00 50.91 O
ANISOU 4369 OG SER B 936 6874 7446 5025 2038 491 810 O
ATOM 4370 H SER B 936 21.476 36.295 25.841 1.00 31.81 H
ATOM 4371 HA SER B 936 21.277 38.183 23.629 1.00 31.72 H
ATOM 4372 HB3 SER B 936 21.002 39.119 26.543 1.00 37.55 H
ATOM 4373 HB2 SER B 936 19.735 39.191 25.339 1.00 37.55 H
ATOM 4374 HG SER B 936 20.903 40.665 24.177 1.00 50.91 H
ATOM 4375 N HIS B 937 23.707 37.572 23.803 1.00 28.68 N
ANISOU 4375 N HIS B 937 3714 4529 2653 1232 269 548 N
ATOM 4376 CA HIS B 937 25.144 37.273 23.757 1.00 28.86 C
ANISOU 4376 CA HIS B 937 3835 4325 2804 1024 228 432 C
ATOM 4377 C HIS B 937 26.021 38.545 23.748 1.00 33.39 C
ANISOU 4377 C HIS B 937 4695 4669 3324 1083 263 408 C
ATOM 4378 O HIS B 937 26.757 38.779 22.788 1.00 33.03 O
ANISOU 4378 O HIS B 937 4718 4550 3281 1020 255 380 O
ATOM 4379 CB HIS B 937 25.414 36.372 22.529 1.00 29.41 C
ANISOU 4379 CB HIS B 937 3759 4454 2962 832 173 382 C
ATOM 4380 CG HIS B 937 24.512 35.169 22.393 1.00 32.59 C
ANISOU 4380 CG HIS B 937 3925 5082 3376 759 137 416 C
ATOM 4381 CD2 HIS B 937 23.690 34.754 21.367 1.00 33.85 C
ANISOU 4381 CD2 HIS B 937 3933 5448 3480 744 118 467 C
ATOM 4382 ND1 HIS B 937 24.372 34.216 23.386 1.00 34.55 N
ANISOU 4382 ND1 HIS B 937 4088 5356 3684 669 112 400 N
ATOM 4383 CE1 HIS B 937 23.515 33.297 22.937 1.00 33.37 C
ANISOU 4383 CE1 HIS B 937 3753 5418 3506 591 79 442 C
ATOM 4384 NE2 HIS B 937 23.058 33.560 21.718 1.00 33.57 N
ANISOU 4384 NE2 HIS B 937 3729 5566 3462 624 78 484 N
ATOM 4385 H HIS B 937 23.205 37.371 22.950 1.00 28.68 H
ATOM 4386 HA HIS B 937 25.398 36.705 24.654 1.00 28.86 H
ATOM 4387 HB3 HIS B 937 26.444 36.017 22.550 1.00 29.41 H
ATOM 4388 HB2 HIS B 937 25.312 36.951 21.609 1.00 29.41 H
ATOM 4389 HD2 HIS B 937 23.510 35.217 20.409 1.00 33.85 H
ATOM 4390 HD1 HIS B 937 24.851 34.194 24.278 1.00 34.55 H

```
ATOM   4391  HE1 HIS B 937      23.228  32.425  23.505  1.00 33.37           H
ATOM   4392  N   ILE B 938      25.911  39.356  24.812  1.00 31.11           N
ANISOU 4392  N   ILE B 938     4588   4262   2968   1195    302    423       N
ATOM   4393  CA  ILE B 938      26.582  40.646  24.947  1.00 31.53           C
ANISOU 4393  CA  ILE B 938     4968   4083   2928   1251    338    410       C
ATOM   4394  C   ILE B 938      28.096  40.521  25.207  1.00 36.86           C
ANISOU 4394  C   ILE B 938     5750   4564   3689   1007    289    318       C
ATOM   4395  O   ILE B 938      28.537  39.625  25.927  1.00 34.34           O
ANISOU 4395  O   ILE B 938     5317   4254   3478    871    248    273       O
ATOM   4396  CB  ILE B 938      25.950  41.512  26.075  1.00 34.51           C
ANISOU 4396  CB  ILE B 938     5546   4408   3156   1498    410    474       C
ATOM   4397  CG1 ILE B 938      25.911  40.847  27.475  1.00 34.30           C
ANISOU 4397  CG1 ILE B 938     5420   4426   3187   1479    398    466       C
ATOM   4398  CG2 ILE B 938      24.551  42.010  25.665  1.00 36.63           C
ANISOU 4398  CG2 ILE B 938     5785   4852   3280   1789    479    587       C
ATOM   4399  CD1 ILE B 938      25.782  41.852  28.630  1.00 38.13           C
ANISOU 4399  CD1 ILE B 938     6202   4744   3541   1636    456    485       C
ATOM   4400  H   ILE B 938      25.278  39.103  25.560  1.00 31.11           H
ATOM   4401  HA  ILE B 938      26.450  41.177  24.001  1.00 31.53           H
ATOM   4402  HB  ILE B 938      26.570  42.406  26.171  1.00 34.51           H
ATOM   4403 HG13 ILE B 938      26.811  40.264  27.657  1.00 34.30           H
ATOM   4404 HG12 ILE B 938      25.092  40.129  27.520  1.00 34.30           H
ATOM   4405 HG21 ILE B 938      24.133  42.698  26.400  1.00 36.63           H
ATOM   4406 HG22 ILE B 938      24.582  42.536  24.711  1.00 36.63           H
ATOM   4407 HG23 ILE B 938      23.854  41.178  25.562  1.00 36.63           H
ATOM   4408 HD11 ILE B 938      25.028  41.525  29.345  1.00 38.13           H
ATOM   4409 HD12 ILE B 938      26.726  41.948  29.168  1.00 38.13           H
ATOM   4410 HD13 ILE B 938      25.502  42.849  28.291  1.00 38.13           H
ATOM   4411  N   SER B 939      28.852  41.457  24.616  1.00 36.33           N
ANISOU 4411  N   SER B 939     5913   4336   3557    956    297    302       N
ATOM   4412  CA  SER B 939      30.285  41.639  24.853  1.00 37.66           C
ANISOU 4412  CA  SER B 939     6212   4340   3759    720    254    238       C
ATOM   4413  C   SER B 939      30.553  42.637  25.996  1.00 44.61           C
ANISOU 4413  C   SER B 939     7410   5022   4520    746    277    240       C
ATOM   4414  O   SER B 939      31.646  42.614  26.560  1.00 44.84           O
ANISOU 4414  O   SER B 939     7480   4970   4586    556    233    196       O
ATOM   4415  CB  SER B 939      30.945  42.134  23.552  1.00 41.14           C
ANISOU 4415  CB  SER B 939     6740   4725   4166    632    248    231       C
ATOM   4416  OG  SER B 939      31.032  41.081  22.613  1.00 53.40           O
ANISOU 4416  OG  SER B 939     8018   6438   5831    565    219    215       O
ATOM   4417  H   SER B 939      28.418  42.130  24.001  1.00 36.33           H
ATOM   4418  HA  SER B 939      30.744  40.691  25.142  1.00 37.66           H
ATOM   4419  HB3 SER B 939      31.960  42.487  23.741  1.00 41.14           H
ATOM   4420  HB2 SER B 939      30.394  42.969  23.117  1.00 41.14           H
ATOM   4421  HG  SER B 939      31.677  40.443  22.926  1.00 53.40           H
ATOM   4422  N   LYS B 940      29.564  43.492  26.306  1.00 42.76           N
ANISOU 4422  N   LYS B 940     7407   4715   4124    989    349    297       N
ATOM   4423  CA  LYS B 940      29.641  44.555  27.303  1.00 43.45           C
ANISOU 4423  CA  LYS B 940     7864   4586   4058   1051    385    304       C
ATOM   4424  C   LYS B 940      28.215  44.976  27.696  1.00 46.92           C
ANISOU 4424  C   LYS B 940     8369   5080   4379   1400    471    379       C
ATOM   4425  O   LYS B 940      27.315  44.959  26.855  1.00 47.51           O
ANISOU 4425  O   LYS B 940     8271   5337   4443   1596    508    441       O
ATOM   4426  CB  LYS B 940      30.477  45.728  26.732  1.00 47.00           C
ANISOU 4426  CB  LYS B 940     8670   4820   4366    976    397    301       C
ATOM   4427  CG  LYS B 940      30.692  46.910  27.696  1.00 67.97           C
```

ANISOU 4427 CG LYS B 940 11749 7206 6869 880 400 280 C
ATOM 4428 CD LYS B 940 31.708 47.949 27.190 1.00 80.77 C
ANISOU 4428 CD LYS B 940 13658 8643 8389 656 374 263 C
ATOM 4429 CE LYS B 940 31.262 48.686 25.913 1.00 95.52 C
ANISOU 4429 CE LYS B 940 15780 10412 10102 853 446 314 C
ATOM 4430 NZ LYS B 940 32.256 49.687 25.486 1.00107.15 N1+
ANISOU 4430 NZ LYS B 940 17508 11719 11486 590 412 296 N1+
ATOM 4431 H LYS B 940 28.684 43.433 25.812 1.00 42.76 H
ATOM 4432 HA LYS B 940 30.136 44.156 28.191 1.00 43.45 H
ATOM 4433 HB3 LYS B 940 30.009 46.084 25.814 1.00 47.00 H
ATOM 4434 HB2 LYS B 940 31.459 45.351 26.442 1.00 47.00 H
ATOM 4435 HG3 LYS B 940 31.031 46.528 28.659 1.00 67.97 H
ATOM 4436 HG2 LYS B 940 29.745 47.412 27.887 1.00 67.97 H
ATOM 4437 HD3 LYS B 940 32.665 47.453 27.017 1.00 80.77 H
ATOM 4438 HD2 LYS B 940 31.885 48.672 27.988 1.00 80.77 H
ATOM 4439 HE3 LYS B 940 30.309 49.188 26.082 1.00 95.52 H
ATOM 4440 HE2 LYS B 940 31.116 47.984 25.092 1.00 95.52 H
ATOM 4441 HZ1 LYS B 940 33.136 49.229 25.295 1.00107.15 H
ATOM 4442 HZ2 LYS B 940 31.931 50.151 24.650 1.00107.15 H
ATOM 4443 HZ3 LYS B 940 32.386 50.369 26.220 1.00107.15 H
ATOM 4444 N LEU B 941 28.052 45.366 28.969 1.00 41.90 N
ANISOU 4444 N LEU B 941 7978 4304 3639 1480 503 382 N
ATOM 4445 CA LEU B 941 26.805 45.876 29.547 1.00 40.68 C
ANISOU 4445 CA LEU B 941 7911 4201 3346 1836 596 463 C
ATOM 4446 C LEU B 941 26.413 47.256 28.968 1.00 44.97 C
ANISOU 4446 C LEU B 941 8842 4584 3660 2077 685 521 C
ATOM 4447 O LEU B 941 27.310 48.034 28.639 1.00 43.15 O
ANISOU 4447 O LEU B 941 8940 4104 3351 1917 669 478 O
ATOM 4448 CB LEU B 941 27.009 46.004 31.074 1.00 40.26 C
ANISOU 4448 CB LEU B 941 8003 4040 3255 1836 601 442 C
ATOM 4449 CG LEU B 941 27.057 44.652 31.813 1.00 43.74 C
ANISOU 4449 CG LEU B 941 8065 4690 3864 1773 559 431 C
ATOM 4450 CD1 LEU B 941 27.634 44.813 33.233 1.00 43.15 C
ANISOU 4450 CD1 LEU B 941 8168 4458 3767 1676 540 386 C
ATOM 4451 CD2 LEU B 941 25.676 43.963 31.811 1.00 44.34 C
ANISOU 4451 CD2 LEU B 941 7913 5030 3905 2081 625 530 C
ATOM 4452 H LEU B 941 28.845 45.352 29.593 1.00 41.90 H
ATOM 4453 HA LEU B 941 26.040 45.133 29.336 1.00 40.68 H
ATOM 4454 HB3 LEU B 941 26.218 46.611 31.516 1.00 40.26 H
ATOM 4455 HB2 LEU B 941 27.932 46.557 31.259 1.00 40.26 H
ATOM 4456 HG LEU B 941 27.744 43.994 31.279 1.00 43.74 H
ATOM 4457 HD11 LEU B 941 27.062 44.270 33.981 1.00 43.15 H
ATOM 4458 HD12 LEU B 941 28.653 44.431 33.280 1.00 43.15 H
ATOM 4459 HD13 LEU B 941 27.668 45.856 33.547 1.00 43.15 H
ATOM 4460 HD21 LEU B 941 25.347 43.668 32.805 1.00 44.34 H
ATOM 4461 HD22 LEU B 941 24.892 44.597 31.399 1.00 44.34 H
ATOM 4462 HD23 LEU B 941 25.711 43.060 31.205 1.00 44.34 H
ATOM 4463 N PRO B 942 25.094 47.579 28.937 1.00 43.78 N
ANISOU 4463 N PRO B 942 8685 4572 3376 2462 782 626 N
ATOM 4464 CA PRO B 942 24.616 48.970 28.776 1.00 44.39 C
ANISOU 4464 CA PRO B 942 9189 4471 3206 2731 880 687 C
ATOM 4465 C PRO B 942 25.194 49.918 29.842 1.00 48.00 C
ANISOU 4465 C PRO B 942 10166 4582 3492 2732 915 651 C
ATOM 4466 O PRO B 942 25.315 49.518 30.999 1.00 46.59 O
ANISOU 4466 O PRO B 942 9960 4391 3351 2678 897 621 O
ATOM 4467 CB PRO B 942 23.086 48.861 28.902 1.00 46.33 C

ANISOU 4467 CB PRO B 942 9262 4996 3347 3160 978 820 C
ATOM 4468 CG PRO B 942 22.764 47.411 28.595 1.00 50.54 C
ANISOU 4468 CG PRO B 942 9224 5879 4099 3031 906 823 C
ATOM 4469 CD PRO B 942 23.971 46.664 29.143 1.00 45.72 C
ANISOU 4469 CD PRO B 942 8565 5146 3662 2676 812 706 C
ATOM 4470 HA PRO B 942 24.886 49.304 27.772 1.00 44.39 H
ATOM 4471 HB3 PRO B 942 22.561 49.546 28.235 1.00 46.33 H
ATOM 4472 HB2 PRO B 942 22.758 49.089 29.918 1.00 46.33 H
ATOM 4473 HG3 PRO B 942 22.712 47.272 27.515 1.00 50.54 H
ATOM 4474 HG2 PRO B 942 21.818 47.083 29.024 1.00 50.54 H
ATOM 4475 HD2 PRO B 942 23.856 46.475 30.212 1.00 45.72 H
ATOM 4476 HD3 PRO B 942 24.083 45.710 28.627 1.00 45.72 H
ATOM 4477 N LYS B 943 25.574 51.136 29.426 1.00 46.05 N
ANISOU 4477 N LYS B 943 10402 4044 3051 2761 957 650 N
ATOM 4478 CA LYS B 943 26.237 52.127 30.275 1.00 46.64 C
ANISOU 4478 CA LYS B 943 11042 3750 2928 2716 984 612 C
ATOM 4479 C LYS B 943 25.374 52.527 31.491 1.00 51.32 C
ANISOU 4479 C LYS B 943 11791 4332 3375 3054 1078 665 C
ATOM 4480 O LYS B 943 24.261 53.021 31.312 1.00 51.25 O
ANISOU 4480 O LYS B 943 11692 4511 3271 3474 1182 772 O
ATOM 4481 CB LYS B 943 26.648 53.328 29.398 1.00 50.68 C
ANISOU 4481 CB LYS B 943 12073 3964 3219 2745 1031 623 C
ATOM 4482 CG LYS B 943 27.524 54.369 30.122 1.00 70.11 C
ANISOU 4482 CG LYS B 943 14875 6116 5647 2296 943 526 C
ATOM 4483 CD LYS B 943 28.031 55.504 29.214 1.00 79.52 C
ANISOU 4483 CD LYS B 943 16526 7044 6642 2266 974 539 C
ATOM 4484 CE LYS B 943 29.039 55.049 28.141 1.00 82.83 C
ANISOU 4484 CE LYS B 943 16724 7520 7229 1840 859 484 C
ATOM 4485 NZ LYS B 943 29.563 56.190 27.367 1.00 88.90 N1+
ANISOU 4485 NZ LYS B 943 17975 8010 7791 1760 883 494 N1+
ATOM 4486 H LYS B 943 25.439 51.396 28.460 1.00 46.05 H
ATOM 4487 HA LYS B 943 27.153 51.660 30.641 1.00 46.64 H
ATOM 4488 HB3 LYS B 943 25.761 53.813 28.987 1.00 50.68 H
ATOM 4489 HB2 LYS B 943 27.203 52.939 28.545 1.00 50.68 H
ATOM 4490 HG3 LYS B 943 28.376 53.871 30.586 1.00 70.11 H
ATOM 4491 HG2 LYS B 943 26.957 54.814 30.940 1.00 70.11 H
ATOM 4492 HD3 LYS B 943 28.484 56.270 29.845 1.00 79.52 H
ATOM 4493 HD2 LYS B 943 27.174 55.981 28.735 1.00 79.52 H
ATOM 4494 HE3 LYS B 943 28.579 54.350 27.443 1.00 82.83 H
ATOM 4495 HE2 LYS B 943 29.879 54.534 28.608 1.00 82.83 H
ATOM 4496 HZ1 LYS B 943 30.029 56.834 27.990 1.00 88.90 H
ATOM 4497 HZ2 LYS B 943 30.221 55.858 26.676 1.00 88.90 H
ATOM 4498 HZ3 LYS B 943 28.801 56.664 26.905 1.00 88.90 H
ATOM 4499 N GLY B 944 25.913 52.272 32.695 1.00 47.80 N
ANISOU 4499 N GLY B 944 11523 3717 2924 2861 1037 596 N
ATOM 4500 CA GLY B 944 25.248 52.507 33.977 1.00 47.33 C
ANISOU 4500 CA GLY B 944 11619 3632 2733 3140 1118 634 C
ATOM 4501 C GLY B 944 24.756 51.201 34.627 1.00 51.04 C
ANISOU 4501 C GLY B 944 11552 4429 3412 3143 1082 644 C
ATOM 4502 O GLY B 944 24.385 51.242 35.800 1.00 52.52 O
ANISOU 4502 O GLY B 944 11843 4599 3513 3313 1133 664 O
ATOM 4503 H GLY B 944 26.832 51.855 32.738 1.00 47.80 H
ATOM 4504 HA3 GLY B 944 24.408 53.195 33.877 1.00 47.33 H
ATOM 4505 HA2 GLY B 944 25.963 52.982 34.649 1.00 47.33 H
ATOM 4506 N LYS B 945 24.764 50.060 33.910 1.00 46.09 N
ANISOU 4506 N LYS B 945 10371 4095 3047 2956 997 631 N

ATOM 4507 CA LYS B 945 24.446 48.731 34.445 1.00 44.37 C
ANISOU 4507 CA LYS B 945 9640 4185 3034 2914 952 637 C
ATOM 4508 C LYS B 945 25.716 48.048 34.975 1.00 46.74 C
ANISOU 4508 C LYS B 945 9821 4415 3524 2482 827 525 C
ATOM 4509 O LYS B 945 26.798 48.260 34.425 1.00 45.71 O
ANISOU 4509 O LYS B 945 9756 4153 3460 2164 747 453 O
ATOM 4510 CB LYS B 945 23.786 47.859 33.354 1.00 45.40 C
ANISOU 4510 CB LYS B 945 9268 4673 3309 2972 937 696 C
ATOM 4511 CG LYS B 945 22.460 48.412 32.797 1.00 42.26 C
ANISOU 4511 CG LYS B 945 8880 4449 2729 3422 1059 834 C
ATOM 4512 CD LYS B 945 21.328 48.423 33.834 1.00 46.04 C
ANISOU 4512 CD LYS B 945 9333 5081 3080 3770 1154 930 C
ATOM 4513 CE LYS B 945 19.967 48.797 33.231 1.00 47.50 C
ANISOU 4513 CE LYS B 945 9526 5467 3055 4241 1282 1085 C
ATOM 4514 NZ LYS B 945 18.898 48.781 34.246 1.00 48.11 N1+
ANISOU 4514 NZ LYS B 945 9465 5783 3029 4559 1366 1194 N1+
ATOM 4515 H LYS B 945 25.074 50.080 32.947 1.00 46.09 H
ATOM 4516 HA LYS B 945 23.747 48.832 35.274 1.00 44.37 H
ATOM 4517 HB3 LYS B 945 23.602 46.858 33.750 1.00 45.40 H
ATOM 4518 HB2 LYS B 945 24.480 47.713 32.529 1.00 45.40 H
ATOM 4519 HG3 LYS B 945 22.163 47.812 31.936 1.00 42.26 H
ATOM 4520 HG2 LYS B 945 22.610 49.420 32.409 1.00 42.26 H
ATOM 4521 HD3 LYS B 945 21.580 49.138 34.614 1.00 46.04 H
ATOM 4522 HD2 LYS B 945 21.273 47.449 34.320 1.00 46.04 H
ATOM 4523 HE3 LYS B 945 19.702 48.098 32.437 1.00 47.50 H
ATOM 4524 HE2 LYS B 945 20.013 49.791 32.785 1.00 47.50 H
ATOM 4525 HZ1 LYS B 945 19.120 49.431 34.988 1.00 48.11 H
ATOM 4526 HZ2 LYS B 945 18.018 49.040 33.823 1.00 48.11 H
ATOM 4527 HZ3 LYS B 945 18.819 47.853 34.643 1.00 48.11 H
ATOM 4528 N HIE B 946 25.545 47.233 36.028 1.00 43.25 N
ANISOU 4528 N HIE B 946 9193 4082 3160 2477 812 519 N
ATOM 4529 CA HIE B 946 26.623 46.528 36.731 1.00 42.95 C
ANISOU 4529 CA HIE B 946 9040 3995 3283 2108 702 426 C
ATOM 4530 C HIE B 946 26.483 44.996 36.657 1.00 43.17 C
ANISOU 4530 C HIE B 946 8523 4321 3561 1984 635 419 C
ATOM 4531 O HIE B 946 27.466 44.303 36.909 1.00 42.66 O
ANISOU 4531 O HIE B 946 8313 4247 3649 1675 540 347 O
ATOM 4532 CB HIE B 946 26.667 46.997 38.199 1.00 44.49 C
ANISOU 4532 CB HIE B 946 9556 4003 3347 2142 726 407 C
ATOM 4533 CG HIE B 946 26.793 48.488 38.396 1.00 48.68 C
ANISOU 4533 CG HIE B 946 10687 4206 3604 2255 793 409 C
ATOM 4534 CD2 HIE B 946 27.924 49.274 38.334 1.00 50.77 C
ANISOU 4534 CD2 HIE B 946 11328 4191 3773 1999 747 350 C
ATOM 4535 ND1 HIE B 946 25.691 49.303 38.683 1.00 51.11 N
ANISOU 4535 ND1 HIE B 946 11268 4463 3686 2675 926 488 N
ATOM 4536 CE1 HIE B 946 26.188 50.529 38.771 1.00 50.72 C
ANISOU 4536 CE1 HIE B 946 11788 4073 3410 2670 960 466 C
ATOM 4537 NE2 HIE B 946 27.506 50.572 38.573 1.00 50.86 N
ANISOU 4537 NE2 HIE B 946 11887 3943 3496 2248 849 382 N
ATOM 4538 H HIE B 946 24.617 47.119 36.416 1.00 43.25 H
ATOM 4539 HA HIE B 946 27.585 46.772 36.277 1.00 42.95 H
ATOM 4540 HB3 HIE B 946 27.488 46.513 38.730 1.00 44.49 H
ATOM 4541 HB2 HIE B 946 25.754 46.688 38.699 1.00 44.49 H
ATOM 4542 HD2 HIE B 946 28.956 49.024 38.138 1.00 50.77 H
ATOM 4543 HE1 HIE B 946 25.587 51.401 38.981 1.00 50.72 H
ATOM 4544 N SER B 947 25.299 44.484 36.283 1.00 37.21 N

```
ANISOU 4544  N   SER B 947     7475   3836   2827   2224    686    503       N
ATOM   4545  CA  SER B 947      25.034  43.057  36.090  1.00 36.22           C
ANISOU 4545  CA  SER B 947     6868   3991   2903   2119    631    508       C
ATOM   4546  C   SER B 947      23.848  42.859  35.135  1.00 38.35           C
ANISOU 4546  C   SER B 947     6861   4548   3162   2322    674    605       C
ATOM   4547  O   SER B 947      23.151  43.821  34.812  1.00 37.25           O
ANISOU 4547  O   SER B 947     6889   4410   2854   2590    757    678       O
ATOM   4548  CB  SER B 947      24.820  42.360  37.459  1.00 38.49           C
ANISOU 4548  CB  SER B 947     7080   4338   3205   2159    638    518       C
ATOM   4549  OG  SER B 947      23.558  42.648  38.025  1.00 44.46           O
ANISOU 4549  OG  SER B 947     7966   5155   3772   2523    749    616       O
ATOM   4550  H   SER B 947      24.527  45.101  36.070  1.00 37.21           H
ATOM   4551  HA  SER B 947      25.908  42.613  35.607  1.00 36.22           H
ATOM   4552  HB3 SER B 947      25.600  42.638  38.168  1.00 38.49           H
ATOM   4553  HB2 SER B 947      24.884  41.279  37.352  1.00 38.49           H
ATOM   4554  HG  SER B 947      23.612  43.518  38.435  1.00 44.46           H
ATOM   4555  N   VAL B 948      23.630  41.597  34.741  1.00 33.93           N
ANISOU 4555  N   VAL B 948     5889   4236   2766   2193    618    611       N
ATOM   4556  CA  VAL B 948      22.395  41.115  34.128  1.00 33.44           C
ANISOU 4556  CA  VAL B 948     5519   4493   2695   2329    641    708       C
ATOM   4557  C   VAL B 948      21.662  40.235  35.148  1.00 36.38           C
ANISOU 4557  C   VAL B 948     5646   5098   3080   2393    652    772       C
ATOM   4558  O   VAL B 948      22.306  39.470  35.866  1.00 34.70           O
ANISOU 4558  O   VAL B 948     5342   4846   2997   2187    593    708       O
ATOM   4559  CB  VAL B 948      22.679  40.278  32.840  1.00 36.57           C
ANISOU 4559  CB  VAL B 948     5666   4986   3243   2093    562    667       C
ATOM   4560  CG1 VAL B 948      21.554  39.330  32.361  1.00 35.86           C
ANISOU 4560  CG1 VAL B 948     5198   5256   3172   2130    555    757       C
ATOM   4561  CG2 VAL B 948      23.042  41.213  31.682  1.00 36.12           C
ANISOU 4561  CG2 VAL B 948     5808   4792   3122   2124    577    653       C
ATOM   4562  H   VAL B 948      24.273  40.873  35.038  1.00 33.93           H
ATOM   4563  HA  VAL B 948      21.752  41.956  33.872  1.00 33.44           H
ATOM   4564  HB  VAL B 948      23.552  39.655  33.032  1.00 36.57           H
ATOM   4565 HG11 VAL B 948      21.821  38.865  31.413  1.00 35.86           H
ATOM   4566 HG12 VAL B 948      21.368  38.515  33.059  1.00 35.86           H
ATOM   4567 HG13 VAL B 948      20.616  39.865  32.208  1.00 35.86           H
ATOM   4568 HG21 VAL B 948      23.301  40.655  30.783  1.00 36.12           H
ATOM   4569 HG22 VAL B 948      22.199  41.857  31.439  1.00 36.12           H
ATOM   4570 HG23 VAL B 948      23.887  41.848  31.935  1.00 36.12           H
ATOM   4571  N   LYS B 949      20.325  40.331  35.142  1.00 33.61           N
ANISOU 4571  N   LYS B 949     5177   5009   2584   2680    729    907       N
ATOM   4572  CA  LYS B 949      19.444  39.371  35.789  1.00 33.37           C
ANISOU 4572  CA  LYS B 949     4860   5264   2556   2719    736    988       C
ATOM   4573  C   LYS B 949      18.636  38.670  34.690  1.00 38.84           C
ANISOU 4573  C   LYS B 949     5182   6314   3262   2679    708   1075       C
ATOM   4574  O   LYS B 949      17.869  39.323  33.985  1.00 39.03           O
ANISOU 4574  O   LYS B 949     5184   6501   3143   2909    767   1177       O
ATOM   4575  CB  LYS B 949      18.548  40.083  36.824  1.00 34.56           C
ANISOU 4575  CB  LYS B 949     5151   5476   2506   3075    849   1092       C
ATOM   4576  CG  LYS B 949      17.541  39.146  37.521  1.00 42.35           C
ANISOU 4576  CG  LYS B 949     5819   6772   3498   3080    849   1178       C
ATOM   4577  CD  LYS B 949      16.643  39.866  38.535  1.00 46.13           C
ANISOU 4577  CD  LYS B 949     6414   7342   3771   3448    967   1291       C
ATOM   4578  CE  LYS B 949      15.618  38.908  39.163  1.00 54.91           C
ANISOU 4578  CE  LYS B 949     7155   8843   4867   3459    970   1407       C
ATOM   4579  NZ  LYS B 949      14.688  39.613  40.062  1.00 71.83           N1+
```

```
ANISOU 4579  NZ  LYS B 949     9411  11074   6808   3822   1090   1516       N1+
ATOM   4580  H   LYS B 949     19.878  40.982  34.510  1.00 33.61           H
ATOM   4581  HA  LYS B 949     20.026  38.620  36.325  1.00 33.37           H
ATOM   4582  HB3 LYS B 949     18.008  40.893  36.334  1.00 34.56           H
ATOM   4583  HB2 LYS B 949     19.179  40.553  37.579  1.00 34.56           H
ATOM   4584  HG3 LYS B 949     18.082  38.340  38.018  1.00 42.35           H
ATOM   4585  HG2 LYS B 949     16.899  38.667  36.782  1.00 42.35           H
ATOM   4586  HD3 LYS B 949     16.133  40.695  38.041  1.00 46.13           H
ATOM   4587  HD2 LYS B 949     17.263  40.313  39.312  1.00 46.13           H
ATOM   4588  HE3 LYS B 949     16.126  38.123  39.722  1.00 54.91           H
ATOM   4589  HE2 LYS B 949     15.034  38.417  38.384  1.00 54.91           H
ATOM   4590  HZ1 LYS B 949     15.205  40.047  40.812  1.00 71.83           H
ATOM   4591  HZ2 LYS B 949     14.193  40.324  39.541  1.00 71.83           H
ATOM   4592  HZ3 LYS B 949     14.026  38.953  40.444  1.00 71.83           H
ATOM   4593  N   GLY B 950     18.782  37.340  34.609  1.00 36.54           N
ANISOU 4593  N   GLY B 950     4627   6126   3130   2378    616   1033       N
ATOM   4594  CA  GLY B 950     17.875  36.476  33.866  1.00 37.21           C
ANISOU 4594  CA  GLY B 950     4368   6552   3220   2278    575   1113       C
ATOM   4595  C   GLY B 950     16.615  36.337  34.726  1.00 44.41           C
ANISOU 4595  C   GLY B 950     5070   7805   3999   2445    625   1264       C
ATOM   4596  O   GLY B 950     16.691  35.827  35.843  1.00 43.22           O
ANISOU 4596  O   GLY B 950     4879   7646   3896   2362    612   1249       O
ATOM   4597  H   GLY B 950     19.426  36.880  35.241  1.00 36.54           H
ATOM   4598  HA3 GLY B 950     18.341  35.501  33.735  1.00 37.21           H
ATOM   4599  HA2 GLY B 950     17.664  36.874  32.873  1.00 37.21           H
ATOM   4600  N   LEU B 951     15.474  36.818  34.210  1.00 44.44           N
ANISOU 4600  N   LEU B 951     4952   8114   3819   2707    692   1418       N
ATOM   4601  CA  LEU B 951     14.200  36.872  34.923  1.00 45.18           C
ANISOU 4601  CA  LEU B 951     4836   8584   3747   2920    756   1591       C
ATOM   4602  C   LEU B 951     13.528  35.494  34.929  1.00 49.16           C
ANISOU 4602  C   LEU B 951     4951   9441   4286   2645    677   1660       C
ATOM   4603  O   LEU B 951     13.108  35.018  33.875  1.00 47.70           O
ANISOU 4603  O   LEU B 951     4549   9482   4093   2494    620   1705       O
ATOM   4604  CB  LEU B 951     13.271  37.916  34.264  1.00 45.19           C
ANISOU 4604  CB  LEU B 951     4842   8820   3510   3337    867   1754       C
ATOM   4605  CG  LEU B 951     13.753  39.381  34.332  1.00 50.15           C
ANISOU 4605  CG  LEU B 951     5903   9104   4047   3649    963   1709       C
ATOM   4606  CD1 LEU B 951     12.844  40.287  33.470  1.00 50.86           C
ANISOU 4606  CD1 LEU B 951     5980   9443   3903   4042   1064   1874       C
ATOM   4607  CD2 LEU B 951     13.891  39.890  35.783  1.00 51.88           C
ANISOU 4607  CD2 LEU B 951     6382   9105   4223   3807   1029   1679       C
ATOM   4608  H   LEU B 951     15.484  37.232  33.285  1.00 44.44           H
ATOM   4609  HA  LEU B 951     14.392  37.167  35.956  1.00 45.18           H
ATOM   4610  HB3 LEU B 951     12.289  37.860  34.735  1.00 45.19           H
ATOM   4611  HB2 LEU B 951     13.119  37.643  33.218  1.00 45.19           H
ATOM   4612  HG  LEU B 951     14.747  39.426  33.884  1.00 50.15           H
ATOM   4613 HD11 LEU B 951     12.441  41.138  34.020  1.00 50.86           H
ATOM   4614 HD12 LEU B 951     13.397  40.688  32.621  1.00 50.86           H
ATOM   4615 HD13 LEU B 951     11.988  39.745  33.067  1.00 50.86           H
ATOM   4616 HD21 LEU B 951     13.368  40.830  35.956  1.00 51.88           H
ATOM   4617 HD22 LEU B 951     13.498  39.182  36.511  1.00 51.88           H
ATOM   4618 HD23 LEU B 951     14.939  40.064  36.024  1.00 51.88           H
ATOM   4619  N   GLY B 952     13.421  34.896  36.123  1.00 46.37           N
ANISOU 4619  N   GLY B 952     4535   9123   3960   2570    671   1667       N
ATOM   4620  CA  GLY B 952     12.742  33.626  36.342  1.00 46.68           C
ANISOU 4620  CA  GLY B 952     4247   9480   4011   2300    600   1737       C
```

```
ATOM   4621  C   GLY B 952      11.224  33.811  36.331  1.00 51.62           C
ANISOU 4621  C   GLY B 952     4583  10624   4406   2519    665   1968       C
ATOM   4622  O   GLY B 952      10.713  34.888  36.636  1.00 49.55           O
ANISOU 4622  O   GLY B 952     4407  10438   3984   2924    782   2065       O
ATOM   4623  H   GLY B 952      13.791  35.355  36.945  1.00 46.37           H
ATOM   4624  HA3 GLY B 952      13.013  33.261  37.330  1.00 46.68           H
ATOM   4625  HA2 GLY B 952      13.059  32.882  35.609  1.00 46.68           H
ATOM   4626  N   LYS B 953      10.516  32.716  36.025  1.00 50.68           N
ANISOU 4626  N   LYS B 953     4130  10873   4253   2245    589   2062       N
ATOM   4627  CA  LYS B 953       9.061  32.583  36.121  1.00 52.03           C
ANISOU 4627  CA  LYS B 953     3946  11619   4202   2345    622   2298       C
ATOM   4628  C   LYS B 953       8.602  32.369  37.582  1.00 57.02           C
ANISOU 4628  C   LYS B 953     4567  12318   4780   2480    685   2359       C
ATOM   4629  O   LYS B 953       7.471  32.718  37.922  1.00 56.17           O
ANISOU 4629  O   LYS B 953     4279  12605   4457   2780    776   2556       O
ATOM   4630  CB  LYS B 953       8.662  31.419  35.192  1.00 55.62           C
ANISOU 4630  CB  LYS B 953     4111  12354   4667   1893    495   2338       C
ATOM   4631  CG  LYS B 953       7.157  31.284  34.908  1.00 78.42           C
ANISOU 4631  CG  LYS B 953     6642  15829   7323   1956    499   2565       C
ATOM   4632  CD  LYS B 953       6.830  30.183  33.880  1.00 91.33           C
ANISOU 4632  CD  LYS B 953     8078  17642   8979   1462    358   2565       C
ATOM   4633  CE  LYS B 953       7.396  30.450  32.470  1.00 99.42           C
ANISOU 4633  CE  LYS B 953     9151  18595  10028   1425    319   2511       C
ATOM   4634  NZ  LYS B 953       6.988  29.411  31.508  1.00106.92           N1+
ANISOU 4634  NZ  LYS B 953     9927  19728  10968    945    183   2518       N1+
ATOM   4635  H   LYS B 953      11.010  31.878  35.752  1.00 50.68           H
ATOM   4636  HA  LYS B 953       8.598  33.500  35.751  1.00 52.03           H
ATOM   4637  HB3 LYS B 953       9.052  30.479  35.583  1.00 55.62           H
ATOM   4638  HB2 LYS B 953       9.169  31.584  34.243  1.00 55.62           H
ATOM   4639  HG3 LYS B 953       6.764  32.238  34.555  1.00 78.42           H
ATOM   4640  HG2 LYS B 953       6.629  31.064  35.836  1.00 78.42           H
ATOM   4641  HD3 LYS B 953       5.746  30.075  33.828  1.00 91.33           H
ATOM   4642  HD2 LYS B 953       7.208  29.229  34.254  1.00 91.33           H
ATOM   4643  HE3 LYS B 953       8.486  30.475  32.479  1.00 99.42           H
ATOM   4644  HE2 LYS B 953       7.055  31.418  32.103  1.00 99.42           H
ATOM   4645  HZ1 LYS B 953       5.980  29.388  31.441  1.00106.92           H
ATOM   4646  HZ2 LYS B 953       7.380  29.622  30.601  1.00106.92           H
ATOM   4647  HZ3 LYS B 953       7.325  28.511  31.817  1.00106.92           H
ATOM   4648  N   THR B 954       9.515  31.844  38.415  1.00 54.38           N
ANISOU 4648  N   THR B 954     4422  11606   4633   2267    640   2194       N
ATOM   4649  CA  THR B 954       9.409  31.703  39.864  1.00 54.63           C
ANISOU 4649  CA  THR B 954     4496  11606   4657   2341    685   2209       C
ATOM   4650  C   THR B 954      10.599  32.440  40.511  1.00 59.44           C
ANISOU 4650  C   THR B 954     5513  11690   5381   2519    734   2039       C
ATOM   4651  O   THR B 954      11.695  32.459  39.946  1.00 57.39           O
ANISOU 4651  O   THR B 954     5467  11041   5297   2351    675   1861       O
ATOM   4652  CB  THR B 954       9.478  30.202  40.263  1.00 60.24           C
ANISOU 4652  CB  THR B 954     5052  12374   5464   1898    579   2182       C
ATOM   4653  CG2 THR B 954       9.590  29.888  41.768  1.00 56.64           C
ANISOU 4653  CG2 THR B 954     4660  11837   5024   1941    615   2176       C
ATOM   4654  OG1 THR B 954       8.317  29.556  39.779  1.00 59.21           O
ANISOU 4654  OG1 THR B 954     4551  12767   5179   1737    538   2364       O
ATOM   4655  H   THR B 954      10.411  31.572  38.033  1.00 54.38           H
ATOM   4656  HA  THR B 954       8.476  32.142  40.217  1.00 54.63           H
ATOM   4657  HB  THR B 954      10.325  29.739  39.757  1.00 60.24           H
ATOM   4658 HG21 THR B 954       9.522  28.815  41.948  1.00 56.64           H
```

ATOM 4659 HG22 THR B 954 10.542 30.216 42.187 1.00 56.64 H
ATOM 4660 HG23 THR B 954 8.790 30.365 42.331 1.00 56.64 H
ATOM 4661 HG1 THR B 954 8.305 29.637 38.822 1.00 59.21 H
ATOM 4662 N THR B 955 10.351 33.028 41.691 1.00 58.55 N
ANISOU 4662 N THR B 955 5510 11591 5147 2867 846 2106 N
ATOM 4663 CA THR B 955 11.310 33.803 42.476 1.00 59.82 C
ANISOU 4663 CA THR B 955 6069 11305 5355 3069 905 1977 C
ATOM 4664 C THR B 955 11.076 33.533 43.983 1.00 66.80 C
ANISOU 4664 C THR B 955 6972 12207 6201 3144 951 2009 C
ATOM 4665 O THR B 955 9.919 33.373 44.375 1.00 66.25 O
ANISOU 4665 O THR B 955 6623 12568 5980 3238 992 2187 O
ATOM 4666 CB THR B 955 11.127 35.325 42.179 1.00 72.66 C
ANISOU 4666 CB THR B 955 7906 12887 6814 3504 1020 2030 C
ATOM 4667 CG2 THR B 955 9.754 35.912 42.568 1.00 70.94 C
ANISOU 4667 CG2 THR B 955 7571 13061 6323 3938 1157 2248 C
ATOM 4668 OG1 THR B 955 12.093 36.103 42.862 1.00 75.16 O
ANISOU 4668 OG1 THR B 955 8658 12698 7202 3583 1043 1867 O
ATOM 4669 H THR B 955 9.423 32.955 42.093 1.00 58.55 H
ATOM 4670 HA THR B 955 12.315 33.505 42.183 1.00 59.82 H
ATOM 4671 HB THR B 955 11.282 35.486 41.112 1.00 72.66 H
ATOM 4672 HG21 THR B 955 9.650 36.932 42.197 1.00 70.94 H
ATOM 4673 HG22 THR B 955 8.938 35.327 42.147 1.00 70.94 H
ATOM 4674 HG23 THR B 955 9.607 35.947 43.648 1.00 70.94 H
ATOM 4675 HG1 THR B 955 11.870 36.123 43.798 1.00 75.16 H
ATOM 4676 N PRO B 956 12.145 33.522 44.818 1.00 65.82 N
ANISOU 4676 N PRO B 956 7161 11653 6193 3111 945 1853 N
ATOM 4677 CA PRO B 956 12.022 33.614 46.291 1.00 66.55 C
ANISOU 4677 CA PRO B 956 7281 11769 6237 3201 993 1888 C
ATOM 4678 C PRO B 956 11.158 34.789 46.780 1.00 74.07 C
ANISOU 4678 C PRO B 956 8280 12935 6927 3689 1145 2049 C
ATOM 4679 O PRO B 956 11.287 35.891 46.243 1.00 74.25 O
ANISOU 4679 O PRO B 956 8473 12906 6831 3982 1220 2075 O
ATOM 4680 CB PRO B 956 13.475 33.778 46.766 1.00 68.11 C
ANISOU 4680 CB PRO B 956 7843 11445 6591 3094 957 1685 C
ATOM 4681 CG PRO B 956 14.297 33.082 45.702 1.00 72.58 C
ANISOU 4681 CG PRO B 956 8481 11771 7325 2842 864 1547 C
ATOM 4682 CD PRO B 956 13.551 33.432 44.421 1.00 67.79 C
ANISOU 4682 CD PRO B 956 7743 11408 6605 2991 896 1651 C
ATOM 4683 HA PRO B 956 11.618 32.663 46.647 1.00 66.55 H
ATOM 4684 HB3 PRO B 956 13.648 33.363 47.759 1.00 68.11 H
ATOM 4685 HB2 PRO B 956 13.761 34.831 46.803 1.00 68.11 H
ATOM 4686 HG3 PRO B 956 14.266 32.003 45.864 1.00 72.58 H
ATOM 4687 HG2 PRO B 956 15.341 33.391 45.698 1.00 72.58 H
ATOM 4688 HD2 PRO B 956 13.878 34.399 44.040 1.00 67.79 H
ATOM 4689 HD3 PRO B 956 13.735 32.676 43.657 1.00 67.79 H
ATOM 4690 N ASP B 957 10.299 34.512 47.776 1.00 72.80 N
ANISOU 4690 N ASP B 957 7979 13019 6664 3789 1197 2162 N
ATOM 4691 CA ASP B 957 9.395 35.457 48.440 1.00 73.83 C
ANISOU 4691 CA ASP B 957 8147 13376 6527 4278 1354 2328 C
ATOM 4692 C ASP B 957 10.178 36.678 49.001 1.00 79.11 C
ANISOU 4692 C ASP B 957 9335 13568 7155 4550 1432 2207 C
ATOM 4693 O ASP B 957 10.989 36.477 49.908 1.00 78.80 O
ANISOU 4693 O ASP B 957 9513 13198 7228 4407 1397 2072 O
ATOM 4694 CB ASP B 957 8.600 34.706 49.543 1.00 76.14 C
ANISOU 4694 CB ASP B 957 8183 14008 6737 4279 1383 2461 C
ATOM 4695 CG ASP B 957 7.499 35.476 50.303 1.00 88.67 C

ANISOU 4695 CG ASP B 957 9669 16002 8019 4775 1544 2690 C
ATOM 4696 OD1 ASP B 957 7.203 36.645 49.962 1.00 89.09 O
ANISOU 4696 OD1 ASP B 957 10041 15889 7921 5198 1663 2697 O
ATOM 4697 OD2 ASP B 957 6.927 34.849 51.219 1.00 96.31 O1-
ANISOU 4697 OD2 ASP B 957 10252 17456 8883 4743 1555 2869 O1-
ATOM 4698 H ASP B 957 10.246 33.561 48.114 1.00 72.80 H
ATOM 4699 HA ASP B 957 8.665 35.750 47.692 1.00 73.83 H
ATOM 4700 HB3 ASP B 957 9.297 34.282 50.268 1.00 76.14 H
ATOM 4701 HB2 ASP B 957 8.123 33.839 49.084 1.00 76.14 H
ATOM 4702 N PRO B 958 9.951 37.900 48.449 1.00 76.55 N
ANISOU 4702 N PRO B 958 9232 13189 6663 4917 1531 2250 N
ATOM 4703 CA PRO B 958 10.622 39.138 48.905 1.00 76.35 C
ANISOU 4703 CA PRO B 958 9755 12674 6580 5132 1597 2127 C
ATOM 4704 C PRO B 958 10.522 39.471 50.405 1.00 79.91 C
ANISOU 4704 C PRO B 958 10436 13041 6887 5393 1698 2154 C
ATOM 4705 O PRO B 958 11.493 39.986 50.958 1.00 79.99 O
ANISOU 4705 O PRO B 958 10883 12591 6919 5384 1699 2005 O
ATOM 4706 CB PRO B 958 9.985 40.249 48.055 1.00 78.28 C
ANISOU 4706 CB PRO B 958 10149 12961 6633 5499 1696 2211 C
ATOM 4707 CG PRO B 958 9.542 39.547 46.787 1.00 82.92 C
ANISOU 4707 CG PRO B 958 10272 13971 7262 5355 1636 2318 C
ATOM 4708 CD PRO B 958 9.092 38.185 47.295 1.00 78.38 C
ANISOU 4708 CD PRO B 958 9253 13788 6741 5138 1584 2411 C
ATOM 4709 HA PRO B 958 11.672 39.047 48.628 1.00 76.35 H
ATOM 4710 HB3 PRO B 958 10.671 41.072 47.854 1.00 78.28 H
ATOM 4711 HB2 PRO B 958 9.108 40.667 48.554 1.00 78.28 H
ATOM 4712 HG3 PRO B 958 10.400 39.422 46.125 1.00 82.92 H
ATOM 4713 HG2 PRO B 958 8.767 40.083 46.239 1.00 82.92 H
ATOM 4714 HD2 PRO B 958 8.053 38.220 47.627 1.00 78.38 H
ATOM 4715 HD3 PRO B 958 9.172 37.455 46.490 1.00 78.38 H
ATOM 4716 N SER B 959 9.375 39.155 51.033 1.00 75.53 N
ANISOU 4716 N SER B 959 9592 12934 6173 5615 1782 2345 N
ATOM 4717 CA SER B 959 9.123 39.386 52.460 1.00 74.79 C
ANISOU 4717 CA SER B 959 9685 12803 5928 5880 1885 2386 C
ATOM 4718 C SER B 959 9.928 38.464 53.402 1.00 77.43 C
ANISOU 4718 C SER B 959 10014 12928 6477 5487 1776 2247 C
ATOM 4719 O SER B 959 10.077 38.801 54.576 1.00 76.35 O
ANISOU 4719 O SER B 959 10129 12624 6256 5637 1837 2223 O
ATOM 4720 CB SER B 959 7.601 39.322 52.720 1.00 78.36 C
ANISOU 4720 CB SER B 959 9802 13840 6129 6249 2013 2651 C
ATOM 4721 OG SER B 959 7.094 38.001 52.771 1.00 87.27 O
ANISOU 4721 OG SER B 959 10370 15433 7355 5944 1923 2755 O
ATOM 4722 H SER B 959 8.627 38.714 50.516 1.00 75.53 H
ATOM 4723 HA SER B 959 9.440 40.408 52.674 1.00 74.79 H
ATOM 4724 HB3 SER B 959 7.054 39.886 51.963 1.00 78.36 H
ATOM 4725 HB2 SER B 959 7.376 39.794 53.676 1.00 78.36 H
ATOM 4726 HG SER B 959 7.098 37.620 51.885 1.00 87.27 H
ATOM 4727 N ALA B 960 10.433 37.337 52.871 1.00 73.17 N
ANISOU 4727 N ALA B 960 9215 12385 6203 4996 1616 2155 N
ATOM 4728 CA ALA B 960 11.194 36.326 53.601 1.00 72.30 C
ANISOU 4728 CA ALA B 960 9079 12086 6305 4606 1504 2026 C
ATOM 4729 C ALA B 960 12.724 36.488 53.484 1.00 75.12 C
ANISOU 4729 C ALA B 960 9793 11899 6849 4350 1409 1793 C
ATOM 4730 O ALA B 960 13.438 35.647 54.029 1.00 74.37 O
ANISOU 4730 O ALA B 960 9697 11627 6934 4033 1312 1680 O
ATOM 4731 CB ALA B 960 10.733 34.945 53.112 1.00 72.89 C

```
ANISOU 4731  CB  ALA B 960     8673  12501   6520   4245   1399   2084       C
ATOM   4732  H   ALA B 960      10.271  37.141  51.893  1.00 73.17           H
ATOM   4733  HA  ALA B 960      10.956  36.389  54.664  1.00 72.30           H
ATOM   4734  HB1 ALA B 960      11.254  34.136  53.625  1.00 72.89           H
ATOM   4735  HB2 ALA B 960       9.665  34.806  53.286  1.00 72.89           H
ATOM   4736  HB3 ALA B 960      10.903  34.832  52.043  1.00 72.89           H
ATOM   4737  N   ASN B 961      13.215  37.553  52.819  1.00 70.99           N
ANISOU 4737  N   ASN B 961     9585  11123   6265   4501   1441   1734       N
ATOM   4738  CA  ASN B 961      14.641  37.915  52.775  1.00 70.44           C
ANISOU 4738  CA  ASN B 961     9870  10567   6327   4282   1361   1535       C
ATOM   4739  C   ASN B 961      15.138  38.351  54.164  1.00 74.06           C
ANISOU 4739  C   ASN B 961    10679  10721   6740   4314   1381   1450       C
ATOM   4740  O   ASN B 961      14.461  39.119  54.848  1.00 73.58           O
ANISOU 4740  O   ASN B 961    10826  10679   6452   4680   1508   1530       O
ATOM   4741  CB  ASN B 961      14.865  39.045  51.741  1.00 70.52           C
ANISOU 4741  CB  ASN B 961    10162  10411   6221   4488   1415   1523       C
ATOM   4742  CG  ASN B 961      15.041  38.542  50.306  1.00 92.58           C
ANISOU 4742  CG  ASN B 961    12841  13137   9199   4192   1306   1439       C
ATOM   4743  ND2 ASN B 961      13.982  38.005  49.698  1.00 77.95           N
ANISOU 4743  ND2 ASN B 961    10591  11651   7377   4161   1290   1543       N
ATOM   4744  OD1 ASN B 961      16.134  38.635  49.749  1.00 91.41           O
ANISOU 4744  OD1 ASN B 961    12960  12621   9150   3987   1236   1287       O
ATOM   4745  H   ASN B 961      12.571  38.220  52.416  1.00 70.99           H
ATOM   4746  HA  ASN B 961      15.151  37.009  52.439  1.00 70.44           H
ATOM   4747  HB3 ASN B 961      15.790  39.575  51.981  1.00 70.52           H
ATOM   4748  HB2 ASN B 961      14.076  39.797  51.794  1.00 70.52           H
ATOM   4749 HD22 ASN B 961      14.063  37.662  48.752  1.00 77.95           H
ATOM   4750 HD21 ASN B 961      13.094  37.932  50.175  1.00 77.95           H
ATOM   4751  N   ILE B 962      16.331  37.856  54.522  1.00 70.29           N
ANISOU 4751  N   ILE B 962    10260   9985   6462   3945   1259   1298       N
ATOM   4752  CA  ILE B 962      17.035  38.155  55.771  1.00 69.88           C
ANISOU 4752  CA  ILE B 962    10522   9649   6379   3917   1257   1211       C
ATOM   4753  C   ILE B 962      18.492  38.541  55.455  1.00 72.70           C
ANISOU 4753  C   ILE B 962    11236   9581   6807   3692   1175   1043       C
ATOM   4754  O   ILE B 962      19.005  38.165  54.402  1.00 72.72           O
ANISOU 4754  O   ILE B 962    11174   9534   6922   3522   1109    992       O
ATOM   4755  CB  ILE B 962      17.026  36.932  56.742  1.00 73.05           C
ANISOU 4755  CB  ILE B 962    10656  10194   6905   3722   1202   1219       C
ATOM   4756  CG1 ILE B 962      17.777  35.673  56.231  1.00 73.43           C
ANISOU 4756  CG1 ILE B 962    10435  10237   7227   3286   1054   1132       C
ATOM   4757  CG2 ILE B 962      15.583  36.573  57.142  1.00 73.65           C
ANISOU 4757  CG2 ILE B 962    10430  10693   6859   3962   1296   1400       C
ATOM   4758  CD1 ILE B 962      19.234  35.551  56.711  1.00 78.91           C
ANISOU 4758  CD1 ILE B 962    11348  10599   8036   3031    962    982       C
ATOM   4759  H   ILE B 962      16.825  37.247  53.883  1.00 70.29           H
ATOM   4760  HA  ILE B 962      16.573  39.010  56.268  1.00 69.88           H
ATOM   4761  HB  ILE B 962      17.508  37.252  57.667  1.00 73.05           H
ATOM   4762 HG13 ILE B 962      17.730  35.609  55.145  1.00 73.43           H
ATOM   4763 HG12 ILE B 962      17.256  34.782  56.584  1.00 73.43           H
ATOM   4764 HG21 ILE B 962      15.570  35.831  57.941  1.00 73.65           H
ATOM   4765 HG22 ILE B 962      15.044  37.449  57.504  1.00 73.65           H
ATOM   4766 HG23 ILE B 962      15.022  36.161  56.303  1.00 73.65           H
ATOM   4767 HD11 ILE B 962      19.885  35.196  55.914  1.00 78.91           H
ATOM   4768 HD12 ILE B 962      19.641  36.496  57.067  1.00 78.91           H
ATOM   4769 HD13 ILE B 962      19.309  34.843  57.534  1.00 78.91           H
ATOM   4770  N   SER B 963      19.131  39.284  56.373  1.00 67.81           N
```

ANISOU 4770 N   SER B 963   10990   8671   6105   3688   1178   965   N
ATOM   4771 CA SER B 963   20.507 39.762 56.223 1.00 67.06   C
ANISOU 4771 CA SER B 963   11240   8197   6043   3449   1096   820   C
ATOM   4772 C   SER B 963   21.487 38.898 57.039 1.00 69.55   C
ANISOU 4772 C   SER B 963   11504   8406   6517   3109   980   723   C
ATOM   4773 O   SER B 963   21.164 38.499 58.159 1.00 68.93   O
ANISOU 4773 O   SER B 963   11440   8361   6388   3176   1007   746   O
ATOM   4774 CB SER B 963   20.555 41.243 56.650 1.00 70.31   C
ANISOU 4774 CB SER B 963   12206   8329   6181   3706   1192   809   C
ATOM   4775 OG SER B 963   21.823 41.815 56.402 1.00 78.99   O
ANISOU 4775 OG SER B 963   13640   9079   7291   3435   1104   677   O
ATOM   4776 H   SER B 963   18.661 39.542 57.229 1.00 67.81   H
ATOM   4777 HA SER B 963   20.793 39.719 55.172 1.00 67.06   H
ATOM   4778 HB3 SER B 963   20.318 41.352 57.710 1.00 70.31   H
ATOM   4779 HB2 SER B 963   19.813 41.819 56.097 1.00 70.31   H
ATOM   4780 HG SER B 963   21.909 41.959 55.456 1.00 78.99   H
ATOM   4781 N   LEU B 964   22.676 38.660 56.462 1.00 65.20   N
ANISOU 4781 N   LEU B 964   10878   7746   6149   2760   855   624   N
ATOM   4782 CA LEU B 964   23.791 37.935 57.066 1.00 64.71   C
ANISOU 4782 CA LEU B 964   10767   7588   6234   2431   738   536   C
ATOM   4783 C   LEU B 964   25.069 38.734 56.768 1.00 69.55   C
ANISOU 4783 C   LEU B 964   11711   7904   6811   2220   667   431   C
ATOM   4784 O   LEU B 964   25.542 38.708 55.632 1.00 68.67   O
ANISOU 4784 O   LEU B 964   11533   7768   6789   2073   615   396   O
ATOM   4785 CB LEU B 964   23.810 36.490 56.498 1.00 64.47   C
ANISOU 4785 CB LEU B 964   10272   7770   6453   2209   656   538   C
ATOM   4786 CG LEU B 964   24.950 35.570 57.000 1.00 68.90   C
ANISOU 4786 CG LEU B 964   10761   8249   7170   1885   536   455   C
ATOM   4787 CD1 LEU B 964   24.939 35.399 58.535 1.00 69.14   C
ANISOU 4787 CD1 LEU B 964   10825   8286   7159   1913   546   468   C
ATOM   4788 CD2 LEU B 964   24.931 34.212 56.262 1.00 69.95   C
ANISOU 4788 CD2 LEU B 964   10517   8541   7519   1693   467   452   C
ATOM   4789 H   LEU B 964   22.847 39.016 55.530 1.00 65.20   H
ATOM   4790 HA LEU B 964   23.661 37.894 58.148 1.00 64.71   H
ATOM   4791 HB3 LEU B 964   23.866 36.549 55.410 1.00 64.47   H
ATOM   4792 HB2 LEU B 964   22.853 36.014 56.717 1.00 64.47   H
ATOM   4793 HG LEU B 964   25.898 36.042 56.737 1.00 68.90   H
ATOM   4794 HD11 LEU B 964   25.754 35.965 58.988 1.00 69.14   H
ATOM   4795 HD12 LEU B 964   24.010 35.754 58.980 1.00 69.14   H
ATOM   4796 HD13 LEU B 964   25.063 34.362 58.847 1.00 69.14   H
ATOM   4797 HD21 LEU B 964   24.700 33.374 56.920 1.00 69.95   H
ATOM   4798 HD22 LEU B 964   24.191 34.192 55.461 1.00 69.95   H
ATOM   4799 HD23 LEU B 964   25.899 34.005 55.806 1.00 69.95   H
ATOM   4800 N   ASP B 965   25.570 39.468 57.781 1.00 67.27   N
ANISOU 4800 N   ASP B 965   11793   7394   6371   2201   666   387   N
ATOM   4801 CA ASP B 965   26.712 40.398 57.697 1.00 67.61   C
ANISOU 4801 CA ASP B 965   12210   7153   6327   1978   598   298   C
ATOM   4802 C   ASP B 965   26.486 41.557 56.699 1.00 70.70   C
ANISOU 4802 C   ASP B 965   12888   7399   6577   2098   653   299   C
ATOM   4803 O   ASP B 965   27.413 41.956 55.993 1.00 70.63   O
ANISOU 4803 O   ASP B 965   12953   7277   6606   1856   576   242   O
ATOM   4804 CB ASP B 965   28.090 39.695 57.499 1.00 70.06   C
ANISOU 4804 CB ASP B 965   12294   7487   6837   1579   451   230   C
ATOM   4805 CG ASP B 965   28.532 38.751 58.632 1.00 84.08   C
ANISOU 4805 CG ASP B 965   14232   9148   8567   1363   373   179   C
ATOM   4806 OD1 ASP B 965   28.187 39.028 59.802 1.00 85.18   O

```
ANISOU 4806  OD1 ASP B 965     14814   9049   8499   1315    372    142       O
ATOM   4807  OD2 ASP B 965      29.350  37.851  58.336  1.00 91.29           O1-
ANISOU 4807  OD2 ASP B 965     14842  10205   9637   1235    313    180       O1-
ATOM   4808  H   ASP B 965      25.122  39.418  58.685  1.00 67.27           H
ATOM   4809  HA  ASP B 965      26.734  40.902  58.664  1.00 67.61           H
ATOM   4810  HB3 ASP B 965      28.883  40.436  57.398  1.00 70.06           H
ATOM   4811  HB2 ASP B 965      28.074  39.130  56.565  1.00 70.06           H
ATOM   4812  N   GLY B 966      25.250  42.086  56.666  1.00 66.21           N
ANISOU 4812  N   GLY B 966     12460   6855   5840   2483    790    375       N
ATOM   4813  CA  GLY B 966      24.859  43.209  55.810  1.00 65.43           C
ANISOU 4813  CA  GLY B 966     12656   6628   5577   2678    868    396       C
ATOM   4814  C   GLY B 966      24.584  42.793  54.354  1.00 67.30           C
ANISOU 4814  C   GLY B 966     12566   7051   5954   2697    865    433       C
ATOM   4815  O   GLY B 966      24.391  43.675  53.518  1.00 67.82           O
ANISOU 4815  O   GLY B 966     12857   7023   5889   2862    929    455       O
ATOM   4816  H   GLY B 966      24.533  41.703  57.267  1.00 66.21           H
ATOM   4817  HA3 GLY B 966      25.629  43.982  55.826  1.00 65.43           H
ATOM   4818  HA2 GLY B 966      23.956  43.663  56.219  1.00 65.43           H
ATOM   4819  N   VAL B 967      24.580  41.483  54.049  1.00 60.85           N
ANISOU 4819  N   VAL B 967     11238   6492   5392   2536    795    442       N
ATOM   4820  CA  VAL B 967      24.348  40.923  52.717  1.00 58.68           C
ANISOU 4820  CA  VAL B 967     10623   6409   5262   2517    780    473       C
ATOM   4821  C   VAL B 967      22.998  40.188  52.730  1.00 58.92           C
ANISOU 4821  C   VAL B 967     10294   6775   5319   2772    857    589       C
ATOM   4822  O   VAL B 967      22.780  39.345  53.601  1.00 57.81           O
ANISOU 4822  O   VAL B 967      9949   6775   5242   2755    849    614       O
ATOM   4823  CB  VAL B 967      25.446  39.885  52.344  1.00 61.83           C
ANISOU 4823  CB  VAL B 967     10753   6835   5906   2121    639    395       C
ATOM   4824  CG1 VAL B 967      25.276  39.305  50.928  1.00 61.10           C
ANISOU 4824  CG1 VAL B 967     10385   6893   5939   2090    623    415       C
ATOM   4825  CG2 VAL B 967      26.858  40.472  52.490  1.00 61.72           C
ANISOU 4825  CG2 VAL B 967     11059   6545   5847   1848    557    299       C
ATOM   4826  H   VAL B 967      24.763  40.807  54.777  1.00 60.85           H
ATOM   4827  HA  VAL B 967      24.328  41.707  51.956  1.00 58.68           H
ATOM   4828  HB  VAL B 967      25.388  39.041  53.033  1.00 61.83           H
ATOM   4829 HG11 VAL B 967      26.065  38.588  50.695  1.00 61.10           H
ATOM   4830 HG12 VAL B 967      24.330  38.780  50.816  1.00 61.10           H
ATOM   4831 HG13 VAL B 967      25.312  40.091  50.175  1.00 61.10           H
ATOM   4832 HG21 VAL B 967      27.618  39.761  52.163  1.00 61.72           H
ATOM   4833 HG22 VAL B 967      26.961  41.378  51.895  1.00 61.72           H
ATOM   4834 HG23 VAL B 967      27.087  40.724  53.525  1.00 61.72           H
ATOM   4835  N   ASP B 968      22.129  40.513  51.761  1.00 53.88           N
ANISOU 4835  N   ASP B 968      9568   6280   4624   2989    926    667       N
ATOM   4836  CA  ASP B 968      20.791  39.930  51.611  1.00 52.98           C
ANISOU 4836  CA  ASP B 968      9093   6529   4509   3212    995    797       C
ATOM   4837  C   ASP B 968      20.857  38.463  51.157  1.00 53.76           C
ANISOU 4837  C   ASP B 968      8712   6863   4853   2943    899    794       C
ATOM   4838  O   ASP B 968      21.588  38.140  50.222  1.00 51.74           O
ANISOU 4838  O   ASP B 968      8354   6554   4749   2693    811    725       O
ATOM   4839  CB  ASP B 968      19.897  40.748  50.646  1.00 55.43           C
ANISOU 4839  CB  ASP B 968      9462   6937   4662   3525   1095    890       C
ATOM   4840  CG  ASP B 968      19.702  42.234  51.004  1.00 74.43           C
ANISOU 4840  CG  ASP B 968     12345   9154   6780   3883   1223    926       C
ATOM   4841  OD1 ASP B 968      19.761  42.577  52.207  1.00 75.44           O
ANISOU 4841  OD1 ASP B 968     12552   9329   6784   4088   1297    979       O
ATOM   4842  OD2 ASP B 968      19.359  42.991  50.070  1.00 83.57           O1-
```

ANISOU 4842 OD2 ASP B 968   13819 10111   7825   3965   1253   903   O1-
ATOM   4843 H   ASP B 968   22.387 41.207 51.071 1.00 53.88   H
ATOM   4844 HA ASP B 968   20.320 39.947 52.597 1.00 52.98   H
ATOM   4845 HB3 ASP B 968   18.907 40.293 50.583 1.00 55.43   H
ATOM   4846 HB2 ASP B 968   20.330 40.704 49.646 1.00 55.43   H
ATOM   4847 N   VAL B 969   20.078 37.607 51.828 1.00 49.07   N
ANISOU 4847 N   VAL B 969   7845   6517   4282   2994   919   870   N
ATOM   4848 CA VAL B 969   20.001 36.173 51.575 1.00 47.67   C
ANISOU 4848 CA VAL B 969   7247   6568   4298   2758   841   884   C
ATOM   4849 C   VAL B 969   18.540 35.864 51.168 1.00 50.30   C
ANISOU 4849 C   VAL B 969   7268   7290   4554   2953   909   1039   C
ATOM   4850 O   VAL B 969   17.662 35.912 52.035 1.00 48.33   O
ANISOU 4850 O   VAL B 969   6966   7229   4169   3181   992   1147   O
ATOM   4851 CB VAL B 969   20.342 35.361 52.861 1.00 51.06   C
ANISOU 4851 CB VAL B 969   7622   6968   4810   2601   795   849   C
ATOM   4852 CG1 VAL B 969   20.349 33.840 52.611 1.00 50.30   C
ANISOU 4852 CG1 VAL B 969   7165   7031   4914   2320   705   841   C
ATOM   4853 CG2 VAL B 969   21.687 35.785 53.479 1.00 50.91   C
ANISOU 4853 CG2 VAL B 969   7934   6605   4804   2465   745   724   C
ATOM   4854 H   VAL B 969   19.519 37.944 52.603 1.00 49.07   H
ATOM   4855 HA VAL B 969   20.714 35.871 50.810 1.00 47.67   H
ATOM   4856 HB VAL B 969   19.587 35.555 53.622 1.00 51.06   H
ATOM   4857 HG11 VAL B 969   20.597 33.290 53.519 1.00 50.30   H
ATOM   4858 HG12 VAL B 969   19.383 33.473 52.269 1.00 50.30   H
ATOM   4859 HG13 VAL B 969   21.090 33.578 51.856 1.00 50.30   H
ATOM   4860 HG21 VAL B 969   21.964 35.136 54.310 1.00 50.91   H
ATOM   4861 HG22 VAL B 969   22.489 35.748 52.743 1.00 50.91   H
ATOM   4862 HG23 VAL B 969   21.644 36.801 53.871 1.00 50.91   H
ATOM   4863 N   PRO B 970   18.281 35.589 49.866 1.00 47.14   N
ANISOU 4863 N   PRO B 970   6657   7033   4222   2867   875   1060   N
ATOM   4864 CA PRO B 970   16.929 35.263 49.374 1.00 47.08   C
ANISOU 4864 CA PRO B 970   6334   7430   4123   3024   929   1219   C
ATOM   4865 C   PRO B 970   16.530 33.793 49.637 1.00 51.66   C
ANISOU 4865 C   PRO B 970   6548   8267   4811   2791   867   1265   C
ATOM   4866 O   PRO B 970   16.277 33.041 48.695 1.00 50.46   O
ANISOU 4866 O   PRO B 970   6143   8276   4754   2585   802   1279   O
ATOM   4867 CB PRO B 970   17.012 35.647 47.885 1.00 48.60   C
ANISOU 4867 CB PRO B 970   6499   7629   4337   3000   908   1209   C
ATOM   4868 CG PRO B 970   18.445 35.327 47.500 1.00 52.51   C
ANISOU 4868 CG PRO B 970   7127   7800   5026   2682   801   1040   C
ATOM   4869 CD PRO B 970   19.234 35.690 48.755 1.00 48.06   C
ANISOU 4869 CD PRO B 970   6801   6970   4487   2619   785   950   C
ATOM   4870 HA PRO B 970   16.176 35.885 49.856 1.00 47.08   H
ATOM   4871 HB3 PRO B 970   16.840 36.719 47.782 1.00 48.60   H
ATOM   4872 HB2 PRO B 970   16.284 35.146 47.247 1.00 48.60   H
ATOM   4873 HG3 PRO B 970   18.786 35.851 46.606 1.00 52.51   H
ATOM   4874 HG2 PRO B 970   18.530 34.256 47.311 1.00 52.51   H
ATOM   4875 HD2 PRO B 970   20.092 35.029 48.875 1.00 48.06   H
ATOM   4876 HD3 PRO B 970   19.595 36.718 48.690 1.00 48.06   H
ATOM   4877 N   LEU B 971   16.462 33.420 50.927 1.00 49.55   N
ANISOU 4877 N   LEU B 971   6278   8032   4517   2818   889   1291   N
ATOM   4878 CA LEU B 971   16.056 32.099 51.418 1.00 50.52   C
ANISOU 4878 CA LEU B 971   6105   8360   4731   2584   832   1330   C
ATOM   4879 C   LEU B 971   14.532 31.906 51.536 1.00 56.92   C
ANISOU 4879 C   LEU B 971   6604   9635   5390   2725   895   1525   C
ATOM   4880 O   LEU B 971   14.102 30.933 52.156 1.00 57.33   O

```
ANISOU 4880  O   LEU B 971     6441   9869   5472   2557    863   1577       O
ATOM   4881  CB  LEU B 971      16.846  31.737  52.702  1.00 50.62           C
ANISOU 4881  CB  LEU B 971     6256   8158   4821   2472    802   1246       C
ATOM   4882  CG  LEU B 971      16.785  32.714  53.901  1.00 54.99           C
ANISOU 4882  CG  LEU B 971     7091   8570   5231   2747    890   1249       C
ATOM   4883  CD1 LEU B 971      15.401  32.769  54.569  1.00 54.77           C
ANISOU 4883  CD1 LEU B 971     6920   8884   5005   3037   1001   1424       C
ATOM   4884  CD2 LEU B 971      17.879  32.357  54.928  1.00 56.66           C
ANISOU 4884  CD2 LEU B 971     7433   8543   5554   2561    830   1142       C
ATOM   4885  H   LEU B 971      16.687  34.102  51.640  1.00 49.55           H
ATOM   4886  HA  LEU B 971      16.352  31.367  50.667  1.00 50.52           H
ATOM   4887  HB3 LEU B 971      17.889  31.645  52.402  1.00 50.62           H
ATOM   4888  HB2 LEU B 971      16.559  30.743  53.047  1.00 50.62           H
ATOM   4889  HG  LEU B 971      17.014  33.718  53.543  1.00 54.99           H
ATOM   4890 HD11 LEU B 971      15.454  33.078  55.613  1.00 54.77           H
ATOM   4891 HD12 LEU B 971      14.760  33.484  54.057  1.00 54.77           H
ATOM   4892 HD13 LEU B 971      14.905  31.801  54.538  1.00 54.77           H
ATOM   4893 HD21 LEU B 971      17.483  32.173  55.927  1.00 56.66           H
ATOM   4894 HD22 LEU B 971      18.434  31.462  54.645  1.00 56.66           H
ATOM   4895 HD23 LEU B 971      18.603  33.166  55.011  1.00 56.66           H
ATOM   4896  N   GLY B 972      13.738  32.799  50.921  1.00 54.83           N
ANISOU 4896  N   GLY B 972     6314   9569   4951   3026    984   1638       N
ATOM   4897  CA  GLY B 972      12.289  32.665  50.802  1.00 54.97           C
ANISOU 4897  CA  GLY B 972     6012  10079   4795   3186   1050   1846       C
ATOM   4898  C   GLY B 972      11.934  31.499  49.869  1.00 59.81           C
ANISOU 4898  C   GLY B 972     6270  10973   5484   2864    956   1898       C
ATOM   4899  O   GLY B 972      12.652  31.220  48.908  1.00 59.85           O
ANISOU 4899  O   GLY B 972     6307  10790   5645   2613    865   1782       O
ATOM   4900  H   GLY B 972      14.166  33.566  50.423  1.00 54.83           H
ATOM   4901  HA3 GLY B 972      11.885  33.584  50.377  1.00 54.97           H
ATOM   4902  HA2 GLY B 972      11.841  32.523  51.786  1.00 54.97           H
ATOM   4903  N   THR B 973      10.797  30.844  50.145  1.00 56.42           N
ANISOU 4903  N   THR B 973     5506  10999   4932   2853    976   2076       N
ATOM   4904  CA  THR B 973      10.237  29.785  49.303  1.00 56.32           C
ANISOU 4904  CA  THR B 973     5158  11293   4947   2524    886   2147       C
ATOM   4905  C   THR B 973       9.699  30.362  47.977  1.00 61.76           C
ANISOU 4905  C   THR B 973     5729  12197   5541   2645    903   2227       C
ATOM   4906  O   THR B 973       9.264  31.516  47.938  1.00 60.20           O
ANISOU 4906  O   THR B 973     5584  12118   5172   3052   1015   2322       O
ATOM   4907  CB  THR B 973       9.085  29.045  50.032  1.00 60.31           C
ANISOU 4907  CB  THR B 973     5358  12212   5346   2415    888   2311       C
ATOM   4908  CG2 THR B 973       9.545  28.350  51.323  1.00 58.34           C
ANISOU 4908  CG2 THR B 973     5179  11730   5257   2088    809   2201       C
ATOM   4909  OG1 THR B 973       8.004  29.908  50.338  1.00 57.20           O
ANISOU 4909  OG1 THR B 973     4933  12058   4740   2831   1024   2461       O
ATOM   4910  H   THR B 973      10.229  31.123  50.931  1.00 56.42           H
ATOM   4911  HA  THR B 973      11.030  29.069  49.079  1.00 56.32           H
ATOM   4912  HB  THR B 973       8.692  28.273  49.368  1.00 60.31           H
ATOM   4913 HG21 THR B 973       8.721  27.809  51.789  1.00 58.34           H
ATOM   4914 HG22 THR B 973      10.334  27.626  51.117  1.00 58.34           H
ATOM   4915 HG23 THR B 973       9.929  29.061  52.055  1.00 58.34           H
ATOM   4916  HG1 THR B 973       7.661  30.278  49.520  1.00 57.20           H
ATOM   4917  N   GLY B 974       9.774  29.543  46.914  1.00 61.20           N
ANISOU 4917  N   GLY B 974     5531  12147   5576   2298    793   2182       N
ATOM   4918  CA  GLY B 974       9.444  29.929  45.544  1.00 62.03           C
ANISOU 4918  CA  GLY B 974     5520  12425   5625   2323    780   2234       C
```

```
ATOM   4919  C   GLY B 974       7.957  30.279  45.399  1.00 68.55           C
ANISOU 4919  C   GLY B 974     6017  13832   6195   2539    850   2484       C
ATOM   4920  O   GLY B 974       7.086  29.484  45.752  1.00 68.69           O
ANISOU 4920  O   GLY B 974     5735  14250   6116   2381    828   2630       O
ATOM   4921  H   GLY B 974      10.138  28.610  47.042  1.00 61.20           H
ATOM   4922  HA3 GLY B 974       9.675  29.096  44.880  1.00 62.03           H
ATOM   4923  HA2 GLY B 974      10.078  30.762  45.237  1.00 62.03           H
ATOM   4924  N   ILE B 975       7.702  31.468  44.840  1.00 66.30           N
ANISOU 4924  N   ILE B 975     5797  13605   5787   2909    937   2542       N
ATOM   4925  CA  ILE B 975       6.391  31.994  44.465  1.00 66.94           C
ANISOU 4925  CA  ILE B 975     5591  14238   5604   3202   1020   2788       C
ATOM   4926  C   ILE B 975       6.470  32.530  43.023  1.00 71.35           C
ANISOU 4926  C   ILE B 975     6149  14806   6155   3236    999   2778       C
ATOM   4927  O   ILE B 975       7.569  32.782  42.528  1.00 70.07           O
ANISOU 4927  O   ILE B 975     6268  14180   6174   3127    950   2580       O
ATOM   4928  CB  ILE B 975       5.949  33.153  45.411  1.00 70.48           C
ANISOU 4928  CB  ILE B 975     6153  14779   5847   3753   1191   2906       C
ATOM   4929  CG1 ILE B 975       6.905  34.377  45.449  1.00 71.36           C
ANISOU 4929  CG1 ILE B 975     6751  14359   6002   4067   1266   2748       C
ATOM   4930  CG2 ILE B 975       5.667  32.627  46.831  1.00 71.35           C
ANISOU 4930  CG2 ILE B 975     6191  14987   5930   3719   1214   2955       C
ATOM   4931  CD1 ILE B 975       6.287  35.619  46.107  1.00 78.70           C
ANISOU 4931  CD1 ILE B 975     7820  15417   6665   4675   1448   2892       C
ATOM   4932  H   ILE B 975       8.480  32.068  44.595  1.00 66.30           H
ATOM   4933  HA  ILE B 975       5.644  31.199  44.483  1.00 66.94           H
ATOM   4934  HB  ILE B 975       4.991  33.514  45.034  1.00 70.48           H
ATOM   4935 HG13 ILE B 975       7.224  34.667  44.449  1.00 71.36           H
ATOM   4936 HG12 ILE B 975       7.817  34.107  45.982  1.00 71.36           H
ATOM   4937 HG21 ILE B 975       5.208  33.391  47.459  1.00 71.35           H
ATOM   4938 HG22 ILE B 975       4.986  31.776  46.807  1.00 71.35           H
ATOM   4939 HG23 ILE B 975       6.584  32.304  47.326  1.00 71.35           H
ATOM   4940 HD11 ILE B 975       6.859  36.512  45.855  1.00 78.70           H
ATOM   4941 HD12 ILE B 975       5.262  35.780  45.775  1.00 78.70           H
ATOM   4942 HD13 ILE B 975       6.279  35.530  47.193  1.00 78.70           H
ATOM   4943  N   SER B 976       5.308  32.713  42.371  1.00 68.59           N
ANISOU 4943  N   SER B 976     5478  14997   5584   3394   1037   3000       N
ATOM   4944  CA  SER B 976       5.221  33.287  41.025  1.00 68.58           C
ANISOU 4944  CA  SER B 976     5463  15043   5551   3460   1025   3011       C
ATOM   4945  C   SER B 976       5.721  34.745  41.004  1.00 71.58           C
ANISOU 4945  C   SER B 976     6243  15046   5909   3907   1140   2930       C
ATOM   4946  O   SER B 976       5.270  35.556  41.813  1.00 71.75           O
ANISOU 4946  O   SER B 976     6388  15103   5771   4338   1276   3016       O
ATOM   4947  CB  SER B 976       3.775  33.167  40.504  1.00 73.24           C
ANISOU 4947  CB  SER B 976     5614  16337   5878   3575   1052   3290       C
ATOM   4948  OG  SER B 976       3.662  33.632  39.172  1.00 86.35           O
ANISOU 4948  OG  SER B 976     7275  18030   7502   3657   1044   3299       O
ATOM   4949  H   SER B 976       4.434  32.502  42.828  1.00 68.59           H
ATOM   4950  HA  SER B 976       5.861  32.687  40.374  1.00 68.58           H
ATOM   4951  HB3 SER B 976       3.087  33.735  41.134  1.00 73.24           H
ATOM   4952  HB2 SER B 976       3.447  32.127  40.533  1.00 73.24           H
ATOM   4953  HG  SER B 976       4.083  32.996  38.588  1.00 86.35           H
ATOM   4954  N   SER B 977       6.648  35.031  40.078  1.00 66.78           N
ANISOU 4954  N   SER B 977     5860  14065   5449   3795   1085   2766       N
ATOM   4955  CA  SER B 977       7.259  36.347  39.889  1.00 66.33           C
ANISOU 4955  CA  SER B 977     6213  13615   5374   4147   1176   2676       C
ATOM   4956  C   SER B 977       6.346  37.368  39.181  1.00 70.63           C
```

```
ANISOU 4956  C   SER B 977     6679  14502   5654   4602   1292   2872       C
ATOM   4957  O   SER B 977      6.615  38.565  39.271  1.00 69.76           O
ANISOU 4957  O   SER B 977     6911  14159   5435   5018   1412   2863       O
ATOM   4958  CB  SER B 977      8.582  36.143  39.125  1.00 68.51           C
ANISOU 4958  CB  SER B 977     6723  13416   5890   3842   1073   2447       C
ATOM   4959  OG  SER B 977      8.341  35.778  37.782  1.00 72.05           O
ANISOU 4959  OG  SER B 977     6920  14109   6346   3636    994   2491       O
ATOM   4960  H   SER B 977      6.961  34.309  39.442  1.00 66.78           H
ATOM   4961  HA  SER B 977      7.494  36.755  40.873  1.00 66.33           H
ATOM   4962  HB3 SER B 977      9.195  35.374  39.598  1.00 68.51           H
ATOM   4963  HB2 SER B 977      9.169  37.062  39.132  1.00 68.51           H
ATOM   4964  HG  SER B 977      9.185  35.558  37.368  1.00 72.05           H
ATOM   4965  N   GLY B 978      5.318  36.878  38.466  1.00 67.57           N
ANISOU 4965  N   GLY B 978     5865  14651   5156   4508   1252   3046       N
ATOM   4966  CA  GLY B 978      4.443  37.680  37.612  1.00 68.26           C
ANISOU 4966  CA  GLY B 978     5815  15132   4990   4907   1347   3250       C
ATOM   4967  C   GLY B 978      5.069  37.969  36.234  1.00 74.70           C
ANISOU 4967  C   GLY B 978     6749  15744   5889   4814   1292   3153       C
ATOM   4968  O   GLY B 978      4.417  38.620  35.418  1.00 74.76           O
ANISOU 4968  O   GLY B 978     6644  16057   5705   5103   1353   3308       O
ATOM   4969  H   GLY B 978      5.163  35.880  38.462  1.00 67.57           H
ATOM   4970  HA3 GLY B 978      4.185  38.620  38.101  1.00 68.26           H
ATOM   4971  HA2 GLY B 978      3.511  37.134  37.465  1.00 68.26           H
ATOM   4972  N   VAL B 979      6.303  37.499  35.962  1.00 72.89           N
ANISOU 4972  N   VAL B 979     6744  15011   5941   4420   1178   2902       N
ATOM   4973  CA  VAL B 979      6.993  37.656  34.682  1.00 73.62           C
ANISOU 4973  CA  VAL B 979     6968  14855   6150   4268   1112   2778       C
ATOM   4974  C   VAL B 979      6.431  36.647  33.665  1.00 79.45           C
ANISOU 4974  C   VAL B 979     7284  16013   6890   3910    991   2862       C
ATOM   4975  O   VAL B 979      6.601  35.439  33.839  1.00 79.63           O
ANISOU 4975  O   VAL B 979     7108  16125   7024   3488    882   2827       O
ATOM   4976  CB  VAL B 979      8.531  37.440  34.812  1.00 76.98           C
ANISOU 4976  CB  VAL B 979     7776  14619   6854   3996   1043   2495       C
ATOM   4977  CG1 VAL B 979      9.285  37.485  33.465  1.00 76.80           C
ANISOU 4977  CG1 VAL B 979     7844  14383   6955   3798    966   2376       C
ATOM   4978  CG2 VAL B 979      9.159  38.462  35.777  1.00 76.72           C
ANISOU 4978  CG2 VAL B 979     8183  14182   6785   4334   1156   2422       C
ATOM   4979  H   VAL B 979      6.797  36.967  36.667  1.00 72.89           H
ATOM   4980  HA  VAL B 979      6.824  38.672  34.316  1.00 73.62           H
ATOM   4981  HB  VAL B 979      8.704  36.449  35.234  1.00 76.98           H
ATOM   4982 HG11 VAL B 979     10.356  37.355  33.607  1.00 76.80           H
ATOM   4983 HG12 VAL B 979      8.969  36.701  32.778  1.00 76.80           H
ATOM   4984 HG13 VAL B 979      9.128  38.440  32.969  1.00 76.80           H
ATOM   4985 HG21 VAL B 979     10.227  38.282  35.901  1.00 76.72           H
ATOM   4986 HG22 VAL B 979      9.036  39.481  35.409  1.00 76.72           H
ATOM   4987 HG23 VAL B 979      8.711  38.413  36.769  1.00 76.72           H
ATOM   4988  N   ASN B 980      5.776  37.182  32.626  1.00 76.11           N
ANISOU 4988  N   ASN B 980     6751  15838   6330   4076   1012   2972       N
ATOM   4989  CA  ASN B 980      5.197  36.413  31.524  1.00 76.09           C
ANISOU 4989  CA  ASN B 980     6368  16251   6290   3765    903   3065       C
ATOM   4990  C   ASN B 980      6.177  36.376  30.342  1.00 79.20           C
ANISOU 4990  C   ASN B 980     6923  15809   6861   3499    809   2887       C
ATOM   4991  O   ASN B 980      6.919  37.339  30.136  1.00 79.14           O
ANISOU 4991  O   ASN B 980     7263  15900   6906   3715    866   2777       O
ATOM   4992  CB  ASN B 980      3.858  37.047  31.076  1.00 77.86           C
ANISOU 4992  CB  ASN B 980     6281  17100   6201   4133    987   3353       C
```

```
ATOM   4993  CG  ASN B 980       2.840  37.208  32.210  1.00112.88           C
ANISOU 4993  CG  ASN B 980    10508  21960  10422   4431   1089   3566       C
ATOM   4994  ND2 ASN B 980       2.312  36.096  32.725  1.00106.74           N
ANISOU 4994  ND2 ASN B 980     9822  21316   9417   5022   1253   3714       N
ATOM   4995  OD1 ASN B 980       2.535  38.326  32.617  1.00110.49           O
ANISOU 4995  OD1 ASN B 980     9971  21869  10139   4147   1028   3605       O
ATOM   4996  H   ASN B 980       5.663  38.184  32.572  1.00 76.11           H
ATOM   4997  HA  ASN B 980       5.009  35.381  31.829  1.00 76.09           H
ATOM   4998  HB3 ASN B 980       3.402  36.444  30.290  1.00 77.86           H
ATOM   4999  HB2 ASN B 980       4.037  38.030  30.636  1.00 77.86           H
ATOM   5000 HD22 ASN B 980       1.639  36.161  33.475  1.00106.74           H
ATOM   5001 HD21 ASN B 980       2.574  35.188  32.371  1.00106.74           H
ATOM   5002  N   ASP B 981       6.122  35.277  29.570  1.00 74.30           N
ANISOU 5002  N   ASP B 981     6057  15863   6310   3026    668   2870       N
ATOM   5003  CA  ASP B 981       6.849  35.072  28.309  1.00 72.99           C
ANISOU 5003  CA  ASP B 981     5976  15470   6289   2724    566   2726       C
ATOM   5004  C   ASP B 981       8.381  35.145  28.477  1.00 72.66           C
ANISOU 5004  C   ASP B 981     6353  14743   6511   2615    549   2454       C
ATOM   5005  O   ASP B 981       9.040  35.941  27.808  1.00 71.99           O
ANISOU 5005  O   ASP B 981     6500  14369   6484   2719    567   2358       O
ATOM   5006  CB  ASP B 981       6.334  35.958  27.139  1.00 75.25           C
ANISOU 5006  CB  ASP B 981     6172  16013   6405   2982    607   2854       C
ATOM   5007  CG  ASP B 981       4.819  35.852  26.904  1.00 91.16           C
ANISOU 5007  CG  ASP B 981     7737  18760   8139   3086    619   3142       C
ATOM   5008  OD1 ASP B 981       4.082  36.705  27.447  1.00 92.29           O
ANISOU 5008  OD1 ASP B 981     7766  19188   8112   3415    721   3308       O
ATOM   5009  OD2 ASP B 981       4.410  34.832  26.305  1.00 98.66           O1-
ANISOU 5009  OD2 ASP B 981     8446  20015   9025   2842    527   3209       O1-
ATOM   5010  H   ASP B 981       5.463  34.549  29.808  1.00 74.30           H
ATOM   5011  HA  ASP B 981       6.643  34.037  28.029  1.00 72.99           H
ATOM   5012  HB3 ASP B 981       6.838  35.679  26.212  1.00 75.25           H
ATOM   5013  HB2 ASP B 981       6.581  37.005  27.322  1.00 75.25           H
ATOM   5014  N   THR B 982       8.905  34.303  29.382  1.00 65.47           N
ANISOU 5014  N   THR B 982     5528  13597   5749   2399    513   2340       N
ATOM   5015  CA  THR B 982      10.332  34.135  29.647  1.00 62.87           C
ANISOU 5015  CA  THR B 982     5549  12679   5662   2258    487   2099       C
ATOM   5016  C   THR B 982      10.730  32.655  29.485  1.00 64.10           C
ANISOU 5016  C   THR B 982     5643  12732   5982   1754    353   1978       C
ATOM   5017  O   THR B 982       9.946  31.766  29.825  1.00 65.33           O
ANISOU 5017  O   THR B 982     5534  13221   6068   1523    294   2074       O
ATOM   5018  CB  THR B 982      10.710  34.643  31.071  1.00 63.27           C
ANISOU 5018  CB  THR B 982     5823  12477   5738   2490    573   2057       C
ATOM   5019  CG2 THR B 982       9.939  33.993  32.239  1.00 60.45           C
ANISOU 5019  CG2 THR B 982     5243  12414   5310   2443    577   2168       C
ATOM   5020  OG1 THR B 982      12.087  34.440  31.324  1.00 58.35           O
ANISOU 5020  OG1 THR B 982     5497  11332   5344   2298    529   1833       O
ATOM   5021  H   THR B 982       8.293  33.684  29.894  1.00 65.47           H
ATOM   5022  HA  THR B 982      10.912  34.701  28.917  1.00 62.87           H
ATOM   5023  HB  THR B 982      10.540  35.720  31.105  1.00 63.27           H
ATOM   5024 HG21 THR B 982      10.257  34.408  33.196  1.00 60.45           H
ATOM   5025 HG22 THR B 982       8.867  34.169  32.154  1.00 60.45           H
ATOM   5026 HG23 THR B 982      10.097  32.916  32.286  1.00 60.45           H
ATOM   5027  HG1 THR B 982      12.278  34.667  32.241  1.00 58.35           H
ATOM   5028  N   SER B 983      11.950  32.431  28.967  1.00 57.22           N
ANISOU 5028  N   SER B 983     5025  11407   5307   1587    309   1776       N
ATOM   5029  CA  SER B 983      12.547  31.108  28.763  1.00 55.69           C
```

```
ANISOU 5029  CA  SER B 983     4835  11061   5265   1153    197   1650       C
ATOM   5030  C   SER B 983     13.079  30.445  30.049  1.00 56.31           C
ANISOU 5030  C   SER B 983     5011  10920   5465   1016    183   1562       C
ATOM   5031  O   SER B 983     13.361  29.247  30.015  1.00 55.55           O
ANISOU 5031  O   SER B 983     4925  10713   5470    675     99   1473       O
ATOM   5032  CB  SER B 983     13.643  31.221  27.682  1.00 58.53           C
ANISOU 5032  CB  SER B 983     5403  11074   5761   1061    164   1491       C
ATOM   5033  OG  SER B 983     14.765  31.964  28.120  1.00 68.05           O
ANISOU 5033  OG  SER B 983     6894  11900   7060   1275    229   1385       O
ATOM   5034  H   SER B 983     12.544  33.216  28.732  1.00 57.22           H
ATOM   5035  HA  SER B 983     11.768  30.452  28.368  1.00 55.69           H
ATOM   5036  HB3 SER B 983     13.242  31.669  26.774  1.00 58.53           H
ATOM   5037  HB2 SER B 983     13.989  30.226  27.399  1.00 58.53           H
ATOM   5038  HG  SER B 983     15.240  31.445  28.775  1.00 68.05           H
ATOM   5039  N   LEU B 984     13.226  31.217  31.139  1.00 49.76           N
ANISOU 5039  N   LEU B 984     4269  10026   4610   1290    268   1590       N
ATOM   5040  CA  LEU B 984     13.815  30.763  32.398  1.00 48.31           C
ANISOU 5040  CA  LEU B 984     4194   9623   4539   1193    261   1506       C
ATOM   5041  C   LEU B 984     12.712  30.455  33.420  1.00 51.36           C
ANISOU 5041  C   LEU B 984     4377  10332   4803   1234    287   1650       C
ATOM   5042  O   LEU B 984     11.819  31.279  33.619  1.00 49.80           O
ANISOU 5042  O   LEU B 984     4079  10403   4440   1547    371   1800       O
ATOM   5043  CB  LEU B 984     14.757  31.862  32.934  1.00 47.96           C
ANISOU 5043  CB  LEU B 984     4466   9166   4591   1405    323   1385       C
ATOM   5044  CG  LEU B 984     15.932  32.220  31.996  1.00 52.53           C
ANISOU 5044  CG  LEU B 984     5262   9400   5297   1344    297   1235       C
ATOM   5045  CD1 LEU B 984     16.717  33.426  32.545  1.00 52.84           C
ANISOU 5045  CD1 LEU B 984     5563   9201   5313   1643    380   1202       C
ATOM   5046  CD2 LEU B 984     16.846  31.008  31.722  1.00 53.96           C
ANISOU 5046  CD2 LEU B 984     5536   9301   5665   1032    219   1080       C
ATOM   5047  H   LEU B 984     12.955  32.191  31.099  1.00 49.76           H
ATOM   5048  HA  LEU B 984     14.398  29.856  32.236  1.00 48.31           H
ATOM   5049  HB3 LEU B 984     15.158  31.554  33.899  1.00 47.96           H
ATOM   5050  HB2 LEU B 984     14.175  32.763  33.127  1.00 47.96           H
ATOM   5051  HG  LEU B 984     15.524  32.539  31.036  1.00 52.53           H
ATOM   5052 HD11 LEU B 984     17.787  33.237  32.619  1.00 52.84           H
ATOM   5053 HD12 LEU B 984     16.587  34.299  31.904  1.00 52.84           H
ATOM   5054 HD13 LEU B 984     16.377  33.704  33.543  1.00 52.84           H
ATOM   5055 HD21 LEU B 984     17.888  31.293  31.581  1.00 53.96           H
ATOM   5056 HD22 LEU B 984     16.814  30.284  32.536  1.00 53.96           H
ATOM   5057 HD23 LEU B 984     16.535  30.492  30.813  1.00 53.96           H
ATOM   5058  N   LEU B 985     12.822  29.287  34.074  1.00 48.72           N
ANISOU 5058  N   LEU B 985     4002   9965   4545    932    220   1608       N
ATOM   5059  CA  LEU B 985     11.933  28.864  35.162  1.00 48.20           C
ANISOU 5059  CA  LEU B 985     3756  10178   4378    921    237   1734       C
ATOM   5060  C   LEU B 985     12.267  29.557  36.494  1.00 48.46           C
ANISOU 5060  C   LEU B 985     3970  10000   4444   1203    324   1700       C
ATOM   5061  O   LEU B 985     11.351  29.817  37.270  1.00 46.65           O
ANISOU 5061  O   LEU B 985     3617  10039   4071   1424    395   1843       O
ATOM   5062  CB  LEU B 985     12.016  27.331  35.345  1.00 48.68           C
ANISOU 5062  CB  LEU B 985     3769  10220   4506    492    137   1688       C
ATOM   5063  CG  LEU B 985     11.448  26.499  34.175  1.00 54.06           C
ANISOU 5063  CG  LEU B 985     4283  11141   5118    150     41   1737       C
ATOM   5064  CD1 LEU B 985     11.763  24.999  34.368  1.00 54.62           C
ANISOU 5064  CD1 LEU B 985     4438  11042   5274   -254    -48   1647       C
ATOM   5065  CD2 LEU B 985      9.941  26.757  33.950  1.00 55.76           C
```

ANISOU 5065 CD2 LEU B 985 4147 11947 5092 189 51 1975 C
ATOM 5066 H LEU B 985 13.572 28.654 33.840 1.00 48.72 H
ATOM 5067 HA LEU B 985 10.910 29.138 34.901 1.00 48.20 H
ATOM 5068 HB3 LEU B 985 11.487 27.042 36.255 1.00 48.68 H
ATOM 5069 HB2 LEU B 985 13.059 27.055 35.511 1.00 48.68 H
ATOM 5070 HG LEU B 985 11.968 26.809 33.267 1.00 54.06 H
ATOM 5071 HD11 LEU B 985 12.294 24.601 33.503 1.00 54.62 H
ATOM 5072 HD12 LEU B 985 12.393 24.821 35.240 1.00 54.62 H
ATOM 5073 HD13 LEU B 985 10.867 24.393 34.505 1.00 54.62 H
ATOM 5074 HD21 LEU B 985 9.377 25.842 33.772 1.00 55.76 H
ATOM 5075 HD22 LEU B 985 9.479 27.254 34.804 1.00 55.76 H
ATOM 5076 HD23 LEU B 985 9.788 27.397 33.080 1.00 55.76 H
ATOM 5077 N TYR B 986 13.562 29.825 36.733 1.00 43.44 N
ANISOU 5077 N TYR B 986 3626 8897 3981 1196 319 1518 N
ATOM 5078 CA TYR B 986 14.101 30.405 37.966 1.00 42.05 C
ANISOU 5078 CA TYR B 986 3665 8469 3845 1412 386 1462 C
ATOM 5079 C TYR B 986 15.117 31.495 37.606 1.00 41.78 C
ANISOU 5079 C TYR B 986 3917 8087 3871 1590 423 1349 C
ATOM 5080 O TYR B 986 15.606 31.520 36.475 1.00 41.27 O
ANISOU 5080 O TYR B 986 3897 7911 3873 1484 380 1278 O
ATOM 5081 CB TYR B 986 14.765 29.299 38.820 1.00 43.58 C
ANISOU 5081 CB TYR B 986 3918 8471 4171 1171 335 1370 C
ATOM 5082 CG TYR B 986 13.883 28.084 39.053 1.00 45.53 C
ANISOU 5082 CG TYR B 986 3909 9035 4355 925 283 1472 C
ATOM 5083 CD1 TYR B 986 12.750 28.189 39.885 1.00 47.19 C
ANISOU 5083 CD1 TYR B 986 3920 9608 4402 1059 336 1643 C
ATOM 5084 CD2 TYR B 986 14.143 26.876 38.371 1.00 46.27 C
ANISOU 5084 CD2 TYR B 986 3959 9099 4521 560 186 1416 C
ATOM 5085 CE1 TYR B 986 11.847 27.114 39.985 1.00 46.74 C
ANISOU 5085 CE1 TYR B 986 3614 9882 4263 802 283 1754 C
ATOM 5086 CE2 TYR B 986 13.243 25.798 38.477 1.00 47.00 C
ANISOU 5086 CE2 TYR B 986 3845 9485 4527 300 133 1517 C
ATOM 5087 CZ TYR B 986 12.082 25.928 39.264 1.00 54.09 C
ANISOU 5087 CZ TYR B 986 4529 10756 5266 406 177 1690 C
ATOM 5088 OH TYR B 986 11.177 24.910 39.321 1.00 56.46 O
ANISOU 5088 OH TYR B 986 4621 11366 5464 113 118 1799 O
ATOM 5089 H TYR B 986 14.245 29.609 36.022 1.00 43.44 H
ATOM 5090 HA TYR B 986 13.303 30.874 38.543 1.00 42.05 H
ATOM 5091 HB3 TYR B 986 15.068 29.708 39.784 1.00 43.58 H
ATOM 5092 HB2 TYR B 986 15.683 28.967 38.337 1.00 43.58 H
ATOM 5093 HD1 TYR B 986 12.549 29.110 40.413 1.00 47.19 H
ATOM 5094 HD2 TYR B 986 15.008 26.785 37.731 1.00 46.27 H
ATOM 5095 HE1 TYR B 986 10.962 27.210 40.596 1.00 46.74 H
ATOM 5096 HE2 TYR B 986 13.431 24.888 37.928 1.00 47.00 H
ATOM 5097 HH TYR B 986 11.465 24.123 38.845 1.00 56.46 H
ATOM 5098 N ASN B 987 15.410 32.391 38.564 1.00 36.09 N
ANISOU 5098 N ASN B 987 3397 7207 3107 1857 503 1340 N
ATOM 5099 CA ASN B 987 16.335 33.510 38.351 1.00 34.62 C
ANISOU 5099 CA ASN B 987 3524 6682 2948 2015 540 1242 C
ATOM 5100 C ASN B 987 17.792 33.038 38.201 1.00 36.82 C
ANISOU 5100 C ASN B 987 3966 6597 3427 1748 461 1061 C
ATOM 5101 O ASN B 987 18.178 32.005 38.752 1.00 35.94 O
ANISOU 5101 O ASN B 987 3788 6434 3435 1503 397 1002 O
ATOM 5102 CB ASN B 987 16.265 34.536 39.506 1.00 33.61 C
ANISOU 5102 CB ASN B 987 3610 6448 2713 2317 636 1269 C
ATOM 5103 CG ASN B 987 14.881 35.139 39.762 1.00 49.62 C

```
ANISOU 5103  CG  ASN B 987      5548   8795   4512   2678    743   1450       C
ATOM   5104  ND2 ASN B 987      14.450  35.135  41.024  1.00 40.27           N
ANISOU 5104  ND2 ASN B 987      4412   7654   3234   2865    811   1508       N
ATOM   5105  OD1 ASN B 987      14.230  35.637  38.847  1.00 47.74           O
ANISOU 5105  OD1 ASN B 987      5204   8770   4165   2814    772   1544       O
ATOM   5106  H   ASN B 987      14.985  32.323  39.478  1.00 36.09           H
ATOM   5107  HA  ASN B 987      16.039  34.005  37.427  1.00 34.62           H
ATOM   5108  HB3 ASN B 987      16.936  35.371  39.296  1.00 33.61           H
ATOM   5109  HB2 ASN B 987      16.639  34.083  40.420  1.00 33.61           H
ATOM   5110 HD22 ASN B 987      13.555  35.536  41.269  1.00 40.27           H
ATOM   5111 HD21 ASN B 987      15.029  34.742  41.758  1.00 40.27           H
ATOM   5112  N   GLU B 988      18.575  33.853  37.485  1.00 31.92           N
ANISOU 5112  N   GLU B 988      3564   5737   2829   1807    470    984       N
ATOM   5113  CA  GLU B 988      20.029  33.770  37.425  1.00 30.88           C
ANISOU 5113  CA  GLU B 988      3612   5271   2852   1612    413    829       C
ATOM   5114  C   GLU B 988      20.611  35.182  37.331  1.00 34.68           C
ANISOU 5114  C   GLU B 988      4396   5511   3270   1785    463    789       C
ATOM   5115  O   GLU B 988      19.914  36.108  36.921  1.00 34.90           O
ANISOU 5115  O   GLU B 988      4482   5624   3153   2028    531    870       O
ATOM   5116  CB  GLU B 988      20.499  32.835  36.290  1.00 31.74           C
ANISOU 5116  CB  GLU B 988      3597   5371   3091   1335    328    761       C
ATOM   5117  CG  GLU B 988      19.964  33.170  34.883  1.00 36.60           C
ANISOU 5117  CG  GLU B 988      4123   6127   3656   1359    326    804       C
ATOM   5118  CD  GLU B 988      20.473  32.165  33.845  1.00 46.25           C
ANISOU 5118  CD  GLU B 988      5225   7352   4996   1072    243    740       C
ATOM   5119  OE1 GLU B 988      21.519  32.455  33.227  1.00 36.54           O
ANISOU 5119  OE1 GLU B 988      4100   5949   3836    998    219    656       O
ATOM   5120  OE2 GLU B 988      19.812  31.114  33.692  1.00 34.85           O1-
ANISOU 5120  OE2 GLU B 988      3602   6076   3562    916    203    775       O1-
ATOM   5121  H   GLU B 988      18.173  34.676  37.054  1.00 31.92           H
ATOM   5122  HA  GLU B 988      20.374  33.361  38.375  1.00 30.88           H
ATOM   5123  HB3 GLU B 988      20.245  31.810  36.557  1.00 31.74           H
ATOM   5124  HB2 GLU B 988      21.590  32.850  36.256  1.00 31.74           H
ATOM   5125  HG3 GLU B 988      20.264  34.178  34.593  1.00 36.60           H
ATOM   5126  HG2 GLU B 988      18.873  33.166  34.880  1.00 36.60           H
ATOM   5127  N   TYR B 989      21.868  35.322  37.769  1.00 30.24           N
ANISOU 5127  N   TYR B 989      4040   4658   2793   1670    433    677       N
ATOM   5128  CA  TYR B 989      22.546  36.605  37.930  1.00 29.85           C
ANISOU 5128  CA  TYR B 989      4321   4348   2672   1773    468    631       C
ATOM   5129  C   TYR B 989      23.960  36.484  37.357  1.00 32.35           C
ANISOU 5129  C   TYR B 989      4720   4455   3116   1528    396    515       C
ATOM   5130  O   TYR B 989      24.652  35.523  37.687  1.00 31.36           O
ANISOU 5130  O   TYR B 989      4482   4303   3129   1309    330    452       O
ATOM   5131  CB  TYR B 989      22.589  36.981  39.425  1.00 31.18           C
ANISOU 5131  CB  TYR B 989      4683   4393   2772   1877    507    628       C
ATOM   5132  CG  TYR B 989      21.239  37.034  40.121  1.00 33.57           C
ANISOU 5132  CG  TYR B 989      4891   4922   2943   2131    585    750       C
ATOM   5133  CD1 TYR B 989      20.731  35.884  40.763  1.00 34.80           C
ANISOU 5133  CD1 TYR B 989      4768   5295   3157   2051    561    795       C
ATOM   5134  CD2 TYR B 989      20.485  38.226  40.122  1.00 35.15           C
ANISOU 5134  CD2 TYR B 989      5292   5123   2941   2458    687    829       C
ATOM   5135  CE1 TYR B 989      19.475  35.923  41.397  1.00 35.94           C
ANISOU 5135  CE1 TYR B 989      4802   5686   3169   2276    633    921       C
ATOM   5136  CE2 TYR B 989      19.236  38.268  40.771  1.00 36.52           C
ANISOU 5136  CE2 TYR B 989      5364   5536   2975   2721    768    957       C
ATOM   5137  CZ  TYR B 989      18.727  37.116  41.403  1.00 45.30           C
```

ANISOU 5137 CZ TYR B 989 6165 6892 4154 2621 739 1005 C
ATOM 5138 OH TYR B 989 17.510 37.160 42.019 1.00 48.66 O
ANISOU 5138 OH TYR B 989 6465 7591 4431 2870 819 1144 O
ATOM 5139 H TYR B 989 22.381 34.506 38.079 1.00 30.24 H
ATOM 5140 HA TYR B 989 22.017 37.389 37.388 1.00 29.85 H
ATOM 5141 HB3 TYR B 989 23.070 37.950 39.526 1.00 31.18 H
ATOM 5142 HB2 TYR B 989 23.217 36.281 39.975 1.00 31.18 H
ATOM 5143 HD1 TYR B 989 21.300 34.966 40.764 1.00 34.80 H
ATOM 5144 HD2 TYR B 989 20.864 39.111 39.632 1.00 35.15 H
ATOM 5145 HE1 TYR B 989 19.095 35.035 41.878 1.00 35.94 H
ATOM 5146 HE2 TYR B 989 18.670 39.187 40.779 1.00 36.52 H
ATOM 5147 HH TYR B 989 17.254 36.319 42.417 1.00 48.66 H
ATOM 5148 N ILE B 990 24.356 37.443 36.505 1.00 28.49 N
ANISOU 5148 N ILE B 990 4429 3831 2567 1573 413 496 N
ATOM 5149 CA ILE B 990 25.617 37.397 35.770 1.00 28.23 C
ANISOU 5149 CA ILE B 990 4462 3632 2631 1347 350 402 C
ATOM 5150 C ILE B 990 26.349 38.741 35.930 1.00 32.28 C
ANISOU 5150 C ILE B 990 5333 3886 3043 1362 368 364 C
ATOM 5151 O ILE B 990 25.817 39.769 35.512 1.00 31.64 O
ANISOU 5151 O ILE B 990 5459 3756 2808 1571 437 413 O
ATOM 5152 CB ILE B 990 25.421 37.111 34.245 1.00 30.76 C
ANISOU 5152 CB ILE B 990 4607 4074 3008 1284 326 408 C
ATOM 5153 CG1 ILE B 990 24.552 35.858 33.973 1.00 30.50 C
ANISOU 5153 CG1 ILE B 990 4249 4295 3044 1236 302 449 C
ATOM 5154 CG2 ILE B 990 26.779 36.939 33.529 1.00 31.41 C
ANISOU 5154 CG2 ILE B 990 4731 4012 3191 1054 265 318 C
ATOM 5155 CD1 ILE B 990 23.042 36.129 33.870 1.00 34.70 C
ANISOU 5155 CD1 ILE B 990 4655 5080 3449 1458 360 572 C
ATOM 5156 H ILE B 990 23.736 38.213 36.286 1.00 28.49 H
ATOM 5157 HA ILE B 990 26.254 36.615 36.172 1.00 28.23 H
ATOM 5158 HB ILE B 990 24.922 37.963 33.781 1.00 30.76 H
ATOM 5159 HG13 ILE B 990 24.751 35.086 34.717 1.00 30.50 H
ATOM 5160 HG12 ILE B 990 24.848 35.418 33.021 1.00 30.50 H
ATOM 5161 HG21 ILE B 990 26.644 36.747 32.464 1.00 31.41 H
ATOM 5162 HG22 ILE B 990 27.405 37.827 33.605 1.00 31.41 H
ATOM 5163 HG23 ILE B 990 27.338 36.100 33.945 1.00 31.41 H
ATOM 5164 HD11 ILE B 990 22.473 35.224 34.080 1.00 34.70 H
ATOM 5165 HD12 ILE B 990 22.700 36.901 34.557 1.00 34.70 H
ATOM 5166 HD13 ILE B 990 22.780 36.456 32.865 1.00 34.70 H
ATOM 5167 N VAL B 991 27.564 38.694 36.501 1.00 29.77 N
ANISOU 5167 N VAL B 991 5097 3414 2798 1134 306 284 N
ATOM 5168 CA VAL B 991 28.496 39.824 36.580 1.00 29.56 C
ANISOU 5168 CA VAL B 991 5402 3151 2678 1053 299 243 C
ATOM 5169 C VAL B 991 29.657 39.617 35.590 1.00 34.07 C
ANISOU 5169 C VAL B 991 5911 3694 3341 816 234 191 C
ATOM 5170 O VAL B 991 30.052 38.479 35.330 1.00 33.42 O
ANISOU 5170 O VAL B 991 5558 3731 3408 684 185 166 O
ATOM 5171 CB VAL B 991 29.084 40.006 38.008 1.00 31.83 C
ANISOU 5171 CB VAL B 991 5899 3291 2906 1001 289 216 C
ATOM 5172 CG1 VAL B 991 28.014 40.509 38.990 1.00 31.29 C
ANISOU 5172 CG1 VAL B 991 5988 3211 2691 1282 374 276 C
ATOM 5173 CG2 VAL B 991 29.829 38.780 38.573 1.00 31.46 C
ANISOU 5173 CG2 VAL B 991 5622 3320 3013 812 219 177 C
ATOM 5174 H VAL B 991 27.930 37.803 36.810 1.00 29.77 H
ATOM 5175 HA VAL B 991 27.985 40.749 36.305 1.00 29.56 H
ATOM 5176 HB VAL B 991 29.825 40.805 37.954 1.00 31.83 H

```
ATOM   5177 HG11 VAL B 991      28.441  40.701  39.974  1.00 31.29           H
ATOM   5178 HG12 VAL B 991      27.576  41.445  38.644  1.00 31.29           H
ATOM   5179 HG13 VAL B 991      27.207  39.786  39.110  1.00 31.29           H
ATOM   5180 HG21 VAL B 991      30.166  38.972  39.592  1.00 31.46           H
ATOM   5181 HG22 VAL B 991      29.185  37.901  38.603  1.00 31.46           H
ATOM   5182 HG23 VAL B 991      30.714  38.531  37.988  1.00 31.46           H
ATOM   5183  N   TYR B 992      30.154  40.735  35.040  1.00 31.58           N
ANISOU 5183  N   TYR B 992     5856   3222   2920    775    240    180       N
ATOM   5184  CA  TYR B 992      31.096  40.772  33.913  1.00 32.19           C
ANISOU 5184  CA  TYR B 992     5887   3290   3056    572    190    146       C
ATOM   5185  C   TYR B 992      32.475  41.330  34.311  1.00 38.97           C
ANISOU 5185  C   TYR B 992     6938   4002   3868    319    133    106       C
ATOM   5186  O   TYR B 992      33.293  41.595  33.430  1.00 39.78           O
ANISOU 5186  O   TYR B 992     7020   4103   3992    141     94     89       O
ATOM   5187  CB  TYR B 992      30.455  41.561  32.750  1.00 33.17           C
ANISOU 5187  CB  TYR B 992     6101   3402   3101    705    238    180       C
ATOM   5188  CG  TYR B 992      29.152  40.955  32.254  1.00 34.27           C
ANISOU 5188  CG  TYR B 992     6004   3739   3279    919    281    232       C
ATOM   5189  CD1 TYR B 992      27.936  41.277  32.891  1.00 35.82           C
ANISOU 5189  CD1 TYR B 992     6267   3975   3369   1186    352    295       C
ATOM   5190  CD2 TYR B 992      29.157  40.034  31.186  1.00 34.33           C
ANISOU 5190  CD2 TYR B 992     5718   3913   3415    849    250    225       C
ATOM   5191  CE1 TYR B 992      26.739  40.668  32.480  1.00 35.47           C
ANISOU 5191  CE1 TYR B 992     5970   4162   3344   1355    384    359       C
ATOM   5192  CE2 TYR B 992      27.956  39.430  30.767  1.00 34.47           C
ANISOU 5192  CE2 TYR B 992     5516   4130   3451   1002    277    279       C
ATOM   5193  CZ  TYR B 992      26.747  39.744  31.420  1.00 38.43           C
ANISOU 5193  CZ  TYR B 992     6059   4699   3843   1246    341    351       C
ATOM   5194  OH  TYR B 992      25.582  39.153  31.037  1.00 31.63           O
ANISOU 5194  OH  TYR B 992     4958   4080   2978   1379    364    421       O
ATOM   5195  H   TYR B 992      29.791  41.631  35.334  1.00 31.58           H
ATOM   5196  HA  TYR B 992      31.282  39.759  33.552  1.00 32.19           H
ATOM   5197  HB3 TYR B 992      31.144  41.615  31.905  1.00 33.17           H
ATOM   5198  HB2 TYR B 992      30.270  42.592  33.053  1.00 33.17           H
ATOM   5199  HD1 TYR B 992      27.921  41.972  33.717  1.00 35.82           H
ATOM   5200  HD2 TYR B 992      30.083  39.778  30.693  1.00 34.33           H
ATOM   5201  HE1 TYR B 992      25.816  40.907  32.985  1.00 35.47           H
ATOM   5202  HE2 TYR B 992      27.969  38.720  29.953  1.00 34.47           H
ATOM   5203  HH  TYR B 992      25.683  38.593  30.259  1.00 31.63           H
ATOM   5204  N   ASP B 993      32.724  41.444  35.624  1.00 35.56           N
ANISOU 5204  N   ASP B 993     6666   3475   3370    288    125     97       N
ATOM   5205  CA  ASP B 993      34.016  41.758  36.222  1.00 35.14           C
ANISOU 5205  CA  ASP B 993     6785   3313   3254     28     62     69       C
ATOM   5206  C   ASP B 993      34.201  40.766  37.380  1.00 37.04           C
ANISOU 5206  C   ASP B 993     6852   3638   3583    -22     26     56       C
ATOM   5207  O   ASP B 993      33.288  40.600  38.192  1.00 36.33           O
ANISOU 5207  O   ASP B 993     6804   3530   3468    154     68     69       O
ATOM   5208  CB  ASP B 993      34.081  43.244  36.667  1.00 37.23           C
ANISOU 5208  CB  ASP B 993     7523   3332   3292     53     95     75       C
ATOM   5209  CG  ASP B 993      35.400  43.776  37.262  1.00 53.04           C
ANISOU 5209  CG  ASP B 993     9767   5207   5179   -262     24     54       C
ATOM   5210  OD1 ASP B 993      36.439  43.081  37.188  1.00 52.87           O
ANISOU 5210  OD1 ASP B 993     9533   5308   5248   -476    -51     41       O
ATOM   5211  OD2 ASP B 993      35.361  44.934  37.732  1.00 62.16           O1-
ANISOU 5211  OD2 ASP B 993    11336   6146   6135   -296     43     57       O1-
ATOM   5212  H   ASP B 993      31.995  41.208  36.283  1.00 35.56           H
```

ATOM 5213 HA ASP B 993 34.806 41.576 35.489 1.00 35.14 H
ATOM 5214 HB3 ASP B 993 33.282 43.437 37.381 1.00 37.23 H
ATOM 5215 HB2 ASP B 993 33.837 43.869 35.806 1.00 37.23 H
ATOM 5216 N ILE B 994 35.384 40.131 37.433 1.00 34.00 N
ANISOU 5216 N ILE B 994 6264 3361 3294 -245 -46 40 N
ATOM 5217 CA ILE B 994 35.776 39.172 38.470 1.00 34.21 C
ANISOU 5217 CA ILE B 994 6102 3486 3409 -303 -85 33 C
ATOM 5218 C ILE B 994 35.853 39.789 39.886 1.00 37.12 C
ANISOU 5218 C ILE B 994 6725 3726 3651 -341 -96 30 C
ATOM 5219 O ILE B 994 35.639 39.070 40.860 1.00 36.21 O
ANISOU 5219 O ILE B 994 6500 3665 3592 -285 -98 29 O
ATOM 5220 CB ILE B 994 37.138 38.487 38.137 1.00 37.95 C
ANISOU 5220 CB ILE B 994 6325 4115 3978 -512 -153 31 C
ATOM 5221 CG1 ILE B 994 38.368 39.434 38.170 1.00 38.94 C
ANISOU 5221 CG1 ILE B 994 6621 4198 3975 -783 -212 41 C
ATOM 5222 CG2 ILE B 994 37.059 37.722 36.798 1.00 39.01 C
ANISOU 5222 CG2 ILE B 994 6206 4373 4243 -438 -133 30 C
ATOM 5223 CD1 ILE B 994 39.711 38.722 37.954 1.00 47.48 C
ANISOU 5223 CD1 ILE B 994 7487 5457 5095 -992 -284 61 C
ATOM 5224 H ILE B 994 36.087 40.346 36.741 1.00 34.00 H
ATOM 5225 HA ILE B 994 35.004 38.402 38.505 1.00 34.21 H
ATOM 5226 HB ILE B 994 37.302 37.732 38.908 1.00 37.95 H
ATOM 5227 HG13 ILE B 994 38.432 39.961 39.121 1.00 38.94 H
ATOM 5228 HG12 ILE B 994 38.250 40.207 37.409 1.00 38.94 H
ATOM 5229 HG21 ILE B 994 37.948 37.114 36.629 1.00 39.01 H
ATOM 5230 HG22 ILE B 994 36.210 37.043 36.775 1.00 39.01 H
ATOM 5231 HG23 ILE B 994 36.960 38.401 35.951 1.00 39.01 H
ATOM 5232 HD11 ILE B 994 40.523 39.271 38.432 1.00 47.48 H
ATOM 5233 HD12 ILE B 994 39.706 37.714 38.370 1.00 47.48 H
ATOM 5234 HD13 ILE B 994 39.948 38.645 36.893 1.00 47.48 H
ATOM 5235 N ALA B 995 36.110 41.108 39.964 1.00 34.08 N
ANISOU 5235 N ALA B 995 6706 3161 3083 -435 -99 28 N
ATOM 5236 CA ALA B 995 36.195 41.885 41.199 1.00 34.37 C
ANISOU 5236 CA ALA B 995 7063 3037 2959 -489 -108 21 C
ATOM 5237 C ALA B 995 34.846 42.150 41.894 1.00 37.80 C
ANISOU 5237 C ALA B 995 7649 3376 3337 -184 -21 29 C
ATOM 5238 O ALA B 995 34.867 42.590 43.040 1.00 37.39 O
ANISOU 5238 O ALA B 995 7825 3211 3170 -194 -22 22 O
ATOM 5239 CB ALA B 995 36.930 43.200 40.901 1.00 35.19 C
ANISOU 5239 CB ALA B 995 7547 2962 2863 -699 -136 18 C
ATOM 5240 H ALA B 995 36.251 41.630 39.109 1.00 34.08 H
ATOM 5241 HA ALA B 995 36.799 41.317 41.906 1.00 34.37 H
ATOM 5242 HB1 ALA B 995 37.050 43.804 41.801 1.00 35.19 H
ATOM 5243 HB2 ALA B 995 37.926 43.009 40.502 1.00 35.19 H
ATOM 5244 HB3 ALA B 995 36.390 43.800 40.169 1.00 35.19 H
ATOM 5245 N GLN B 996 33.714 41.861 41.227 1.00 34.08 N
ANISOU 5245 N GLN B 996 7043 2973 2933 85 53 51 N
ATOM 5246 CA GLN B 996 32.363 41.939 41.798 1.00 33.10 C
ANISOU 5246 CA GLN B 996 7008 2820 2747 400 144 81 C
ATOM 5247 C GLN B 996 31.972 40.729 42.672 1.00 36.32 C
ANISOU 5247 C GLN B 996 7122 3391 3287 479 142 90 C
ATOM 5248 O GLN B 996 30.887 40.757 43.252 1.00 36.70 O
ANISOU 5248 O GLN B 996 7192 3463 3290 730 215 126 O
ATOM 5249 CB GLN B 996 31.338 42.146 40.664 1.00 34.23 C
ANISOU 5249 CB GLN B 996 7122 3006 2877 642 221 119 C
ATOM 5250 CG GLN B 996 31.423 43.541 40.025 1.00 43.39 C

ANISOU 5250 CG GLN B 996   8709   3948   3830   702   267   127   C
ATOM   5251 CD GLN B 996   30.412 43.705 38.892 1.00 52.13   C
ANISOU 5251 CD GLN B 996   9756   5122   4928   929   335   172   C
ATOM   5252 NE2 GLN B 996   29.196 44.137 39.223 1.00 36.33   N
ANISOU 5252 NE2 GLN B 996   7721   3204   2877   1257   425   231   N
ATOM   5253 OE1 GLN B 996   30.716 43.435 37.733 1.00 51.28   O
ANISOU 5253 OE1 GLN B 996   9625   5011   4848   813   310   161   O
ATOM   5254 H   GLN B 996   33.774 41.510 40.281 1.00 34.08   H
ATOM   5255 HA GLN B 996   32.315 42.812 42.452 1.00 33.10   H
ATOM   5256 HB3 GLN B 996   30.325 42.014 41.049 1.00 34.23   H
ATOM   5257 HB2 GLN B 996   31.466 41.374 39.904 1.00 34.23   H
ATOM   5258 HG3 GLN B 996   32.424 43.724 39.633 1.00 43.39   H
ATOM   5259 HG2 GLN B 996   31.241 44.302 40.782 1.00 43.39   H
ATOM   5260 HE22 GLN B 996   28.496 44.250 38.499 1.00 36.33   H
ATOM   5261 HE21 GLN B 996   28.945 44.308 40.188 1.00 36.33   H
ATOM   5262 N   VAL B 997   32.835 39.701 42.750 1.00 30.90   N
ANISOU 5262 N   VAL B 997   6167   2825   2750   275   65   67   N
ATOM   5263 CA VAL B 997   32.602 38.467 43.501 1.00 29.70   C
ANISOU 5263 CA VAL B 997   5750   2814   2723   321   57   74   C
ATOM   5264 C   VAL B 997   33.572 38.387 44.696 1.00 33.48   C
ANISOU 5264 C   VAL B 997   6291   3253   3177   151   -3   53   C
ATOM   5265 O   VAL B 997   34.761 38.679 44.547 1.00 32.95   O
ANISOU 5265 O   VAL B 997   6231   3182   3108   -91   -75   34   O
ATOM   5266 CB VAL B 997   32.832 37.217 42.598 1.00 32.54   C
ANISOU 5266 CB VAL B 997   5728   3362   3274   283   33   74   C
ATOM   5267 CG1 VAL B 997   32.688 35.864 43.331 1.00 31.64   C
ANISOU 5267 CG1 VAL B 997   5386   3369   3267   330   30   85   C
ATOM   5268 CG2 VAL B 997   31.898 37.235 41.373 1.00 32.03   C
ANISOU 5268 CG2 VAL B 997   5589   3355   3226   429   83   98   C
ATOM   5269 H   VAL B 997   33.715 39.755 42.254 1.00 30.90   H
ATOM   5270 HA VAL B 997   31.578 38.434 43.877 1.00 29.70   H
ATOM   5271 HB VAL B 997   33.852 37.256 42.210 1.00 32.54   H
ATOM   5272 HG11 VAL B 997   32.785 35.030 42.636 1.00 31.64   H
ATOM   5273 HG12 VAL B 997   33.457 35.719 44.091 1.00 31.64   H
ATOM   5274 HG13 VAL B 997   31.715 35.779 43.817 1.00 31.64   H
ATOM   5275 HG21 VAL B 997   32.017 36.337 40.768 1.00 32.03   H
ATOM   5276 HG22 VAL B 997   30.851 37.299 41.673 1.00 32.03   H
ATOM   5277 HG23 VAL B 997   32.111 38.084 40.723 1.00 32.03   H
ATOM   5278 N   ASN B 998   33.035 37.949 45.845 1.00 29.58   N
ANISOU 5278 N   ASN B 998   5817   2761   2663   276   27   66   N
ATOM   5279 CA ASN B 998   33.776 37.670 47.071 1.00 28.76   C
ANISOU 5279 CA ASN B 998   5732   2648   2549   139   -27   52   C
ATOM   5280 C   ASN B 998   33.183 36.394 47.684 1.00 30.24   C
ANISOU 5280 C   ASN B 998   5628   2987   2876   237   -16   71   C
ATOM   5281 O   ASN B 998   32.075 36.441 48.217 1.00 29.88   O
ANISOU 5281 O   ASN B 998   5609   2948   2796   439   49   98   O
ATOM   5282 CB ASN B 998   33.675 38.890 48.019 1.00 28.44   C
ANISOU 5282 CB ASN B 998   6082   2416   2306   164   -7   46   C
ATOM   5283 CG ASN B 998   34.650 38.896 49.203 1.00 50.80   C
ANISOU 5283 CG ASN B 998   8979   5227   5096   -63   -87   27   C
ATOM   5284 ND2 ASN B 998   34.529 39.908 50.064 1.00 41.02   N
ANISOU 5284 ND2 ASN B 998   7489   4127   3968   -276   -169   24   N
ATOM   5285 OD1 ASN B 998   35.503 38.024 49.347 1.00 44.01   O
ANISOU 5285 OD1 ASN B 998   8398   4235   4088   -44   -75   21   O
ATOM   5286 H   ASN B 998   32.042 37.762 45.887 1.00 29.58   H
ATOM   5287 HA ASN B 998   34.825 37.463 46.853 1.00 28.76   H

ATOM 5288 HB3 ASN B 998 32.654 39.034 48.377 1.00 28.44 H
ATOM 5289 HB2 ASN B 998 33.916 39.777 47.439 1.00 28.44 H
ATOM 5290 HD22 ASN B 998 35.164 39.984 50.845 1.00 41.02 H
ATOM 5291 HD21 ASN B 998 33.823 40.616 49.919 1.00 41.02 H
ATOM 5292 N LEU B 999 33.918 35.275 47.570 1.00 26.31 N
ANISOU 5292 N LEU B 999 4857 2618 2521 104 -72 64 N
ATOM 5293 CA LEU B 999 33.520 33.957 48.071 1.00 26.56 C
ANISOU 5293 CA LEU B 999 4634 2778 2681 163 -68 80 C
ATOM 5294 C LEU B 999 33.522 33.925 49.610 1.00 31.04 C
ANISOU 5294 C LEU B 999 5290 3312 3191 174 -75 85 C
ATOM 5295 O LEU B 999 34.494 34.371 50.220 1.00 32.24 O
ANISOU 5295 O LEU B 999 5575 3405 3268 24 -127 70 O
ATOM 5296 CB LEU B 999 34.461 32.878 47.490 1.00 26.32 C
ANISOU 5296 CB LEU B 999 4356 2863 2781 29 -122 72 C
ATOM 5297 CG LEU B 999 34.420 32.741 45.950 1.00 29.86 C
ANISOU 5297 CG LEU B 999 4693 3357 3295 19 -115 65 C
ATOM 5298 CD1 LEU B 999 35.543 31.810 45.440 1.00 29.93 C
ANISOU 5298 CD1 LEU B 999 4479 3478 3416 -72 -154 64 C
ATOM 5299 CD2 LEU B 999 33.025 32.315 45.443 1.00 28.06 C
ANISOU 5299 CD2 LEU B 999 4403 3162 3096 192 -52 83 C
ATOM 5300 H LEU B 999 34.835 35.322 47.146 1.00 26.31 H
ATOM 5301 HA LEU B 999 32.505 33.758 47.726 1.00 26.56 H
ATOM 5302 HB3 LEU B 999 34.209 31.913 47.928 1.00 26.32 H
ATOM 5303 HB2 LEU B 999 35.482 33.090 47.809 1.00 26.32 H
ATOM 5304 HG LEU B 999 34.630 33.723 45.524 1.00 29.86 H
ATOM 5305 HD11 LEU B 999 36.169 32.325 44.711 1.00 29.93 H
ATOM 5306 HD12 LEU B 999 36.201 31.483 46.247 1.00 29.93 H
ATOM 5307 HD13 LEU B 999 35.165 30.909 44.960 1.00 29.93 H
ATOM 5308 HD21 LEU B 999 33.078 31.542 44.680 1.00 28.06 H
ATOM 5309 HD22 LEU B 999 32.398 31.923 46.244 1.00 28.06 H
ATOM 5310 HD23 LEU B 999 32.500 33.163 45.003 1.00 28.06 H
ATOM 5311 N LYS B1000 32.425 33.416 50.196 1.00 27.03 N
ANISOU 5311 N LYS B1000 4720 2850 2702 342 -21 113 N
ATOM 5312 CA LYS B1000 32.176 33.440 51.640 1.00 26.08 C
ANISOU 5312 CA LYS B1000 4693 2698 2518 387 -13 124 C
ATOM 5313 C LYS B1000 32.168 32.040 52.257 1.00 29.64 C
ANISOU 5313 C LYS B1000 4910 3265 3086 375 -30 141 C
ATOM 5314 O LYS B1000 32.784 31.860 53.307 1.00 29.57 O
ANISOU 5314 O LYS B1000 4934 3242 3060 296 -69 135 O
ATOM 5315 CB LYS B1000 30.832 34.146 51.925 1.00 27.92 C
ANISOU 5315 CB LYS B1000 5103 2879 2625 613 74 154 C
ATOM 5316 CG LYS B1000 30.800 35.640 51.568 1.00 40.21 C
ANISOU 5316 CG LYS B1000 6982 4273 4022 646 99 138 C
ATOM 5317 CD LYS B1000 31.827 36.476 52.347 1.00 48.85 C
ANISOU 5317 CD LYS B1000 8367 5210 4983 508 55 105 C
ATOM 5318 CE LYS B1000 31.666 37.972 52.074 1.00 60.43 C
ANISOU 5318 CE LYS B1000 10196 6489 6277 497 72 86 C
ATOM 5319 NZ LYS B1000 32.601 38.783 52.872 1.00 64.22 N1+
ANISOU 5319 NZ LYS B1000 10988 6811 6601 333 23 55 N1+
ATOM 5320 H LYS B1000 31.676 33.048 49.626 1.00 27.03 H
ATOM 5321 HA LYS B1000 32.972 33.980 52.152 1.00 26.08 H
ATOM 5322 HB3 LYS B1000 30.595 34.054 52.986 1.00 27.92 H
ATOM 5323 HB2 LYS B1000 30.025 33.638 51.395 1.00 27.92 H
ATOM 5324 HG3 LYS B1000 29.798 36.022 51.767 1.00 40.21 H
ATOM 5325 HG2 LYS B1000 30.955 35.767 50.497 1.00 40.21 H
ATOM 5326 HD3 LYS B1000 32.837 36.180 52.060 1.00 48.85 H

ATOM 5327 HD2 LYS B1000 31.729 36.270 53.414 1.00 48.85 H
ATOM 5328 HE3 LYS B1000 30.649 38.287 52.309 1.00 60.43 H
ATOM 5329 HE2 LYS B1000 31.838 38.176 51.016 1.00 60.43 H
ATOM 5330 HZ1 LYS B1000 33.551 38.525 52.646 1.00 64.22 H
ATOM 5331 HZ2 LYS B1000 32.452 39.758 52.651 1.00 64.22 H
ATOM 5332 HZ3 LYS B1000 32.430 38.630 53.855 1.00 64.22 H
ATOM 5333 N TYR B1001 31.465 31.089 51.621 1.00 26.01 N
ANISOU 5333 N TYR B1001 4235 2919 2729 447 -1 165 N
ATOM 5334 CA TYR B1001 31.333 29.720 52.117 1.00 25.36 C
ANISOU 5334 CA TYR B1001 3966 2929 2742 433 -11 185 C
ATOM 5335 C TYR B1001 31.468 28.714 50.971 1.00 28.79 C
ANISOU 5335 C TYR B1001 4201 3441 3298 374 -27 181 C
ATOM 5336 O TYR B1001 31.228 29.045 49.810 1.00 28.12 O
ANISOU 5336 O TYR B1001 4084 3374 3228 387 -13 177 O
ATOM 5337 CB TYR B1001 29.988 29.508 52.856 1.00 26.25 C
ANISOU 5337 CB TYR B1001 4054 3104 2815 583 51 235 C
ATOM 5338 CG TYR B1001 29.643 30.516 53.937 1.00 28.36 C
ANISOU 5338 CG TYR B1001 4537 3294 2946 683 83 244 C
ATOM 5339 CD1 TYR B1001 30.103 30.332 55.258 1.00 30.13 C
ANISOU 5339 CD1 TYR B1001 4821 3481 3145 642 58 238 C
ATOM 5340 CD2 TYR B1001 28.850 31.640 53.623 1.00 29.03 C
ANISOU 5340 CD2 TYR B1001 4787 3334 2909 829 143 259 C
ATOM 5341 CE1 TYR B1001 29.775 31.271 56.257 1.00 31.68 C
ANISOU 5341 CE1 TYR B1001 5248 3589 3200 730 89 241 C
ATOM 5342 CE2 TYR B1001 28.531 32.582 54.618 1.00 29.58 C
ANISOU 5342 CE2 TYR B1001 5106 3304 2828 936 180 264 C
ATOM 5343 CZ TYR B1001 28.992 32.398 55.936 1.00 36.64 C
ANISOU 5343 CZ TYR B1001 6064 4156 3700 880 152 253 C
ATOM 5344 OH TYR B1001 28.667 33.312 56.894 1.00 38.58 O
ANISOU 5344 OH TYR B1001 6580 4296 3784 988 192 257 O
ATOM 5345 H TYR B1001 30.984 31.301 50.757 1.00 26.01 H
ATOM 5346 HA TYR B1001 32.150 29.517 52.808 1.00 25.36 H
ATOM 5347 HB3 TYR B1001 29.971 28.515 53.309 1.00 26.25 H
ATOM 5348 HB2 TYR B1001 29.169 29.497 52.142 1.00 26.25 H
ATOM 5349 HD1 TYR B1001 30.703 29.471 55.511 1.00 30.13 H
ATOM 5350 HD2 TYR B1001 28.493 31.789 52.614 1.00 29.03 H
ATOM 5351 HE1 TYR B1001 30.126 31.120 57.267 1.00 31.68 H
ATOM 5352 HE2 TYR B1001 27.930 33.444 54.369 1.00 29.58 H
ATOM 5353 HH TYR B1001 28.935 33.053 57.785 1.00 38.58 H
ATOM 5354 N LEU B1002 31.819 27.483 51.360 1.00 25.14 N
ANISOU 5354 N LEU B1002 3623 3020 2910 322 -51 187 N
ATOM 5355 CA LEU B1002 31.872 26.299 50.518 1.00 25.13 C
ANISOU 5355 CA LEU B1002 3474 3070 3005 277 -60 186 C
ATOM 5356 C LEU B1002 31.166 25.180 51.290 1.00 29.76 C
ANISOU 5356 C LEU B1002 3988 3703 3616 300 -42 221 C
ATOM 5357 O LEU B1002 31.574 24.868 52.407 1.00 30.13 O
ANISOU 5357 O LEU B1002 4065 3736 3648 305 -51 232 O
ATOM 5358 CB LEU B1002 33.352 25.996 50.176 1.00 25.10 C
ANISOU 5358 CB LEU B1002 3440 3055 3040 187 -107 161 C
ATOM 5359 CG LEU B1002 33.649 24.638 49.496 1.00 30.62 C
ANISOU 5359 CG LEU B1002 4029 3786 3818 163 -111 160 C
ATOM 5360 CD1 LEU B1002 32.801 24.394 48.231 1.00 31.99 C
ANISOU 5360 CD1 LEU B1002 4165 3973 4018 165 -90 149 C
ATOM 5361 CD2 LEU B1002 35.161 24.492 49.213 1.00 32.15 C
ANISOU 5361 CD2 LEU B1002 4193 4003 4020 113 -148 153 C
ATOM 5362 H LEU B1002 31.995 27.317 52.344 1.00 25.14 H

```
ATOM   5363  HA  LEU B1002      31.323  26.481  49.593  1.00 25.13           H
ATOM   5364  HB3 LEU B1002      33.943  26.051  51.091  1.00 25.10           H
ATOM   5365  HB2 LEU B1002      33.724  26.798  49.537  1.00 25.10           H
ATOM   5366  HG  LEU B1002      33.398  23.851  50.209  1.00 30.62           H
ATOM   5367 HD11 LEU B1002      33.390  23.978  47.417  1.00 31.99           H
ATOM   5368 HD12 LEU B1002      32.001  23.681  48.434  1.00 31.99           H
ATOM   5369 HD13 LEU B1002      32.337  25.306  47.859  1.00 31.99           H
ATOM   5370 HD21 LEU B1002      35.530  23.527  49.556  1.00 32.15           H
ATOM   5371 HD22 LEU B1002      35.397  24.569  48.151  1.00 32.15           H
ATOM   5372 HD23 LEU B1002      35.749  25.258  49.719  1.00 32.15           H
ATOM   5373  N   LEU B1003      30.111  24.616  50.689  1.00 25.08           N
ANISOU 5373  N   LEU B1003     3310   3174   3047    303    -17    244       N
ATOM   5374  CA  LEU B1003      29.327  23.521  51.251  1.00 26.21           C
ANISOU 5374  CA  LEU B1003     3392   3373   3195    291     -2    286       C
ATOM   5375  C   LEU B1003      29.657  22.231  50.494  1.00 28.92           C
ANISOU 5375  C   LEU B1003     3691   3698   3599    202    -20    274       C
ATOM   5376  O   LEU B1003      29.742  22.240  49.267  1.00 27.49           O
ANISOU 5376  O   LEU B1003     3486   3516   3442    166    -25    253       O
ATOM   5377  CB  LEU B1003      27.817  23.817  51.127  1.00 26.66           C
ANISOU 5377  CB  LEU B1003     3388   3547   3193    340     38    340       C
ATOM   5378  CG  LEU B1003      27.241  24.773  52.191  1.00 33.22           C
ANISOU 5378  CG  LEU B1003     4264   4425   3934    468     79    381       C
ATOM   5379  CD1 LEU B1003      27.722  26.230  52.018  1.00 33.50           C
ANISOU 5379  CD1 LEU B1003     4424   4384   3920    551     88    348       C
ATOM   5380  CD2 LEU B1003      25.700  24.669  52.222  1.00 35.32           C
ANISOU 5380  CD2 LEU B1003     4417   4866   4136    514    121    460       C
ATOM   5381  H   LEU B1003      29.842  24.918  49.760  1.00 25.08           H
ATOM   5382  HA  LEU B1003      29.567  23.377  52.306  1.00 26.21           H
ATOM   5383  HB3 LEU B1003      27.286  22.869  51.224  1.00 26.66           H
ATOM   5384  HB2 LEU B1003      27.581  24.190  50.134  1.00 26.66           H
ATOM   5385  HG  LEU B1003      27.595  24.427  53.162  1.00 33.22           H
ATOM   5386 HD11 LEU B1003      26.950  26.958  52.263  1.00 33.50           H
ATOM   5387 HD12 LEU B1003      28.567  26.435  52.676  1.00 33.50           H
ATOM   5388 HD13 LEU B1003      28.045  26.436  50.997  1.00 33.50           H
ATOM   5389 HD21 LEU B1003      25.215  25.604  51.951  1.00 35.32           H
ATOM   5390 HD22 LEU B1003      25.317  23.916  51.532  1.00 35.32           H
ATOM   5391 HD23 LEU B1003      25.352  24.392  53.217  1.00 35.32           H
ATOM   5392  N   LYS B1004      29.778  21.143  51.263  1.00 25.34           N
ANISOU 5392  N   LYS B1004     3249   3222   3156    174    -24    291       N
ATOM   5393  CA  LYS B1004      29.931  19.777  50.785  1.00 24.70           C
ANISOU 5393  CA  LYS B1004     3181   3102   3104    102    -30    289       C
ATOM   5394  C   LYS B1004      28.580  19.076  50.974  1.00 28.72           C
ANISOU 5394  C   LYS B1004     3657   3686   3570     33    -14    341       C
ATOM   5395  O   LYS B1004      28.142  18.908  52.112  1.00 26.76           O
ANISOU 5395  O   LYS B1004     3398   3481   3288     52     -2    382       O
ATOM   5396  CB  LYS B1004      31.089  19.145  51.582  1.00 26.38           C
ANISOU 5396  CB  LYS B1004     3450   3239   3333    129    -42    282       C
ATOM   5397  CG  LYS B1004      31.359  17.652  51.334  1.00 32.53           C
ANISOU 5397  CG  LYS B1004     4294   3947   4118     87    -37    282       C
ATOM   5398  CD  LYS B1004      32.640  17.214  52.063  1.00 41.06           C
ANISOU 5398  CD  LYS B1004     5425   4972   5204    161    -41    282       C
ATOM   5399  CE  LYS B1004      32.751  15.697  52.275  1.00 53.27           C
ANISOU 5399  CE  LYS B1004     7073   6445   6721    146    -24    310       C
ATOM   5400  NZ  LYS B1004      33.928  15.357  53.098  1.00 62.07           N1+
ANISOU 5400  NZ  LYS B1004     8235   7522   7828    254    -21    321       N1+
ATOM   5401  H   LYS B1004      29.678  21.241  52.266  1.00 25.34           H
```

ATOM 5402 HA LYS B1004 30.194 19.768 49.726 1.00 24.70 H
ATOM 5403 HB3 LYS B1004 30.900 19.280 52.647 1.00 26.38 H
ATOM 5404 HB2 LYS B1004 31.998 19.708 51.365 1.00 26.38 H
ATOM 5405 HG3 LYS B1004 31.448 17.451 50.266 1.00 32.53 H
ATOM 5406 HG2 LYS B1004 30.512 17.069 51.691 1.00 32.53 H
ATOM 5407 HD3 LYS B1004 32.702 17.731 53.019 1.00 41.06 H
ATOM 5408 HD2 LYS B1004 33.499 17.558 51.485 1.00 41.06 H
ATOM 5409 HE3 LYS B1004 32.815 15.179 51.318 1.00 53.27 H
ATOM 5410 HE2 LYS B1004 31.865 15.325 52.789 1.00 53.27 H
ATOM 5411 HZ1 LYS B1004 33.854 15.828 53.991 1.00 62.07 H
ATOM 5412 HZ2 LYS B1004 33.966 14.359 53.249 1.00 62.07 H
ATOM 5413 HZ3 LYS B1004 34.769 15.661 52.631 1.00 62.07 H
ATOM 5414 N LEU B1005 27.933 18.731 49.851 1.00 27.06 N
ANISOU 5414 N LEU B1005 3422 3511 3347 -54 -16 346 N
ATOM 5415 CA LEU B1005 26.583 18.168 49.811 1.00 28.05 C
ANISOU 5415 CA LEU B1005 3497 3749 3411 -162 -10 408 C
ATOM 5416 C LEU B1005 26.626 16.670 49.486 1.00 32.27 C
ANISOU 5416 C LEU B1005 4125 4209 3927 -311 -25 408 C
ATOM 5417 O LEU B1005 27.480 16.241 48.710 1.00 31.72 O
ANISOU 5417 O LEU B1005 4142 4025 3884 -327 -34 357 O
ATOM 5418 CB LEU B1005 25.762 18.871 48.707 1.00 28.49 C
ANISOU 5418 CB LEU B1005 3447 3947 3432 -174 -5 432 C
ATOM 5419 CG LEU B1005 25.545 20.389 48.875 1.00 33.86 C
ANISOU 5419 CG LEU B1005 4071 4691 4104 -22 17 435 C
ATOM 5420 CD1 LEU B1005 24.813 20.947 47.635 1.00 34.56 C
ANISOU 5420 CD1 LEU B1005 4078 4893 4160 -38 20 453 C
ATOM 5421 CD2 LEU B1005 24.820 20.737 50.192 1.00 35.50 C
ANISOU 5421 CD2 LEU B1005 4230 5008 4249 66 49 500 C
ATOM 5422 H LEU B1005 28.378 18.875 48.954 1.00 27.06 H
ATOM 5423 HA LEU B1005 26.090 18.306 50.772 1.00 28.05 H
ATOM 5424 HB3 LEU B1005 24.785 18.390 48.631 1.00 28.49 H
ATOM 5425 HB2 LEU B1005 26.251 18.703 47.747 1.00 28.49 H
ATOM 5426 HG LEU B1005 26.522 20.875 48.908 1.00 33.86 H
ATOM 5427 HD11 LEU B1005 24.048 21.684 47.882 1.00 34.56 H
ATOM 5428 HD12 LEU B1005 25.518 21.425 46.955 1.00 34.56 H
ATOM 5429 HD13 LEU B1005 24.322 20.160 47.063 1.00 34.56 H
ATOM 5430 HD21 LEU B1005 24.058 21.506 50.069 1.00 35.50 H
ATOM 5431 HD22 LEU B1005 24.323 19.869 50.626 1.00 35.50 H
ATOM 5432 HD23 LEU B1005 25.530 21.108 50.932 1.00 35.50 H
ATOM 5433 N LYS B1006 25.640 15.934 50.022 1.00 29.42 N
ANISOU 5433 N LYS B1006 3759 3918 3500 -426 -25 471 N
ATOM 5434 CA LYS B1006 25.328 14.560 49.644 1.00 29.68 C
ANISOU 5434 CA LYS B1006 3914 3882 3480 -607 -41 481 C
ATOM 5435 C LYS B1006 24.045 14.570 48.801 1.00 33.40 C
ANISOU 5435 C LYS B1006 4289 4528 3873 -772 -58 533 C
ATOM 5436 O LYS B1006 23.014 15.067 49.255 1.00 31.76 O
ANISOU 5436 O LYS B1006 3920 4533 3616 -781 -51 609 O
ATOM 5437 CB LYS B1006 25.169 13.688 50.905 1.00 31.91 C
ANISOU 5437 CB LYS B1006 4278 4125 3721 -666 -35 524 C
ATOM 5438 CG LYS B1006 25.165 12.184 50.594 1.00 41.00 C
ANISOU 5438 CG LYS B1006 5638 5133 4808 -841 -48 519 C
ATOM 5439 CD LYS B1006 25.099 11.313 51.860 1.00 53.72 C
ANISOU 5439 CD LYS B1006 7358 6686 6366 -907 -42 563 C
ATOM 5440 CE LYS B1006 25.556 9.859 51.643 1.00 63.79 C
ANISOU 5440 CE LYS B1006 8906 7708 7624 -897 -31 522 C
ATOM 5441 NZ LYS B1006 24.734 9.147 50.647 1.00 73.06 N1+

ANISOU 5441 NZ LYS B1006 10257 8768 8736 -1033 -43 491 N1+
ATOM 5442 H LYS B1006 24.987 16.372 50.661 1.00 29.42 H
ATOM 5443 HA LYS B1006 26.141 14.146 49.045 1.00 29.68 H
ATOM 5444 HB3 LYS B1006 24.272 13.963 51.458 1.00 31.91 H
ATOM 5445 HB2 LYS B1006 26.001 13.884 51.578 1.00 31.91 H
ATOM 5446 HG3 LYS B1006 26.066 11.939 50.032 1.00 41.00 H
ATOM 5447 HG2 LYS B1006 24.325 11.943 49.941 1.00 41.00 H
ATOM 5448 HD3 LYS B1006 24.077 11.321 52.244 1.00 53.72 H
ATOM 5449 HD2 LYS B1006 25.709 11.760 52.646 1.00 53.72 H
ATOM 5450 HE3 LYS B1006 25.507 9.314 52.586 1.00 63.79 H
ATOM 5451 HE2 LYS B1006 26.597 9.838 51.319 1.00 63.79 H
ATOM 5452 HZ1 LYS B1006 24.798 9.619 49.757 1.00 73.06 H
ATOM 5453 HZ2 LYS B1006 25.062 8.196 50.551 1.00 73.06 H
ATOM 5454 HZ3 LYS B1006 23.769 9.143 50.949 1.00 73.06 H
ATOM 5455 N PHE B1007 24.153 14.011 47.589 1.00 32.34 N
ANISOU 5455 N PHE B1007 4250 4318 3719 -886 -78 497 N
ATOM 5456 CA PHE B1007 23.090 13.896 46.599 1.00 33.66 C
ANISOU 5456 CA PHE B1007 4341 4647 3800 -1072 -105 544 C
ATOM 5457 C PHE B1007 22.441 12.515 46.728 1.00 39.58 C
ANISOU 5457 C PHE B1007 5219 5384 4434 -1338 -132 591 C
ATOM 5458 O PHE B1007 23.042 11.514 46.339 1.00 39.58 O
ANISOU 5458 O PHE B1007 5464 5157 4419 -1406 -136 540 O
ATOM 5459 CB PHE B1007 23.678 14.105 45.190 1.00 34.98 C
ANISOU 5459 CB PHE B1007 4551 4741 3998 -1059 -116 477 C
ATOM 5460 CG PHE B1007 24.099 15.527 44.866 1.00 36.05 C
ANISOU 5460 CG PHE B1007 4545 4938 4213 -855 -97 449 C
ATOM 5461 CD1 PHE B1007 25.240 16.105 45.464 1.00 37.83 C
ANISOU 5461 CD1 PHE B1007 4796 5042 4535 -651 -73 395 C
ATOM 5462 CD2 PHE B1007 23.311 16.310 43.998 1.00 37.31 C
ANISOU 5462 CD2 PHE B1007 4562 5276 4339 -879 -107 480 C
ATOM 5463 CE1 PHE B1007 25.594 17.413 45.160 1.00 38.24 C
ANISOU 5463 CE1 PHE B1007 4755 5135 4641 -496 -60 371 C
ATOM 5464 CE2 PHE B1007 23.691 17.608 43.695 1.00 39.45 C
ANISOU 5464 CE2 PHE B1007 4743 5576 4669 -692 -87 454 C
ATOM 5465 CZ PHE B1007 24.829 18.158 44.272 1.00 37.15 C
ANISOU 5465 CZ PHE B1007 4503 5146 4469 -512 -64 397 C
ATOM 5466 H PHE B1007 25.042 13.612 47.311 1.00 32.34 H
ATOM 5467 HA PHE B1007 22.332 14.665 46.766 1.00 33.66 H
ATOM 5468 HB3 PHE B1007 22.941 13.798 44.446 1.00 34.98 H
ATOM 5469 HB2 PHE B1007 24.538 13.453 45.037 1.00 34.98 H
ATOM 5470 HD1 PHE B1007 25.854 15.534 46.145 1.00 37.83 H
ATOM 5471 HD2 PHE B1007 22.427 15.893 43.539 1.00 37.31 H
ATOM 5472 HE1 PHE B1007 26.475 17.849 45.606 1.00 38.24 H
ATOM 5473 HE2 PHE B1007 23.096 18.189 43.007 1.00 39.45 H
ATOM 5474 HZ PHE B1007 25.119 19.170 44.031 1.00 37.15 H
ATOM 5475 N ASN B1008 21.230 12.497 47.297 1.00 37.77 N
ANISOU 5475 N ASN B1008 4837 5403 4109 -1481 -146 697 N
ATOM 5476 CA ASN B1008 20.459 11.293 47.593 1.00 38.79 C
ANISOU 5476 CA ASN B1008 5068 5567 4104 -1780 -179 762 C
ATOM 5477 C ASN B1008 19.487 11.032 46.432 1.00 44.63 C
ANISOU 5477 C ASN B1008 5753 6477 4726 -2032 -227 808 C
ATOM 5478 O ASN B1008 18.323 11.423 46.507 1.00 45.09 O
ANISOU 5478 O ASN B1008 5561 6864 4706 -2104 -239 913 O
ATOM 5479 CB ASN B1008 19.739 11.487 48.949 1.00 41.69 C
ANISOU 5479 CB ASN B1008 5294 6127 4421 -1799 -166 864 C
ATOM 5480 CG ASN B1008 20.704 11.679 50.128 1.00 75.09 C

ANISOU 5480 CG ASN B1008   9529 10240   8760 -1535   -120   829   C
ATOM   5481 ND2 ASN B1008   20.491 12.722 50.934 1.00 71.46   N
ANISOU 5481 ND2 ASN B1008   8847   9971   8332 -1340   -89   870   N
ATOM   5482 OD1 ASN B1008   21.630 10.891 50.314 1.00 65.33   O
ANISOU 5482 OD1 ASN B1008   8508   8750   7566 -1498   -111   770   O
ATOM   5483 H   ASN B1008   20.805 13.373 47.580 1.00 37.77   H
ATOM   5484 HA ASN B1008   21.126 10.432 47.678 1.00 38.79   H
ATOM   5485 HB3 ASN B1008   19.118 10.618 49.169 1.00 41.69   H
ATOM   5486 HB2 ASN B1008   19.063 12.339 48.892 1.00 41.69   H
ATOM   5487 HD22 ASN B1008   21.094 12.875 51.731 1.00 71.46   H
ATOM   5488 HD21 ASN B1008   19.725 13.357 50.764 1.00 71.46   H
ATOM   5489 N   PHE B1009   20.008 10.408 45.361 1.00 42.61   N
ANISOU 5489 N   PHE B1009   5732   6011   4445 -2153   -250   734   N
ATOM   5490 CA PHE B1009   19.270 10.071 44.141 1.00 43.94   C
ANISOU 5490 CA PHE B1009   5902   6298   4497 -2409   -302   761   C
ATOM   5491 C   PHE B1009   18.117   9.083 44.381 1.00 51.77   C
ANISOU 5491 C   PHE B1009   6912   7459   5298 -2787   -354   871   C
ATOM   5492 O   PHE B1009   18.307   8.082 45.073 1.00 52.13   O
ANISOU 5492 O   PHE B1009   7175   7342   5290 -2912   -355   874   O
ATOM   5493 CB PHE B1009   20.237   9.518 43.077 1.00 45.46   C
ANISOU 5493 CB PHE B1009   6384   6183   4706 -2414   -304   645   C
ATOM   5494 CG PHE B1009   21.180 10.547 42.486 1.00 46.43   C
ANISOU 5494 CG PHE B1009   6427   6231   4984 -2100   -267   560   C
ATOM   5495 CD1 PHE B1009   20.713 11.434 41.493 1.00 49.60   C
ANISOU 5495 CD1 PHE B1009   6646   6807   5393 -2082   -282   567   C
ATOM   5496 CD2 PHE B1009   22.470 10.736 43.025 1.00 48.15   C
ANISOU 5496 CD2 PHE B1009   6743   6223   5328 -1832   -217   483   C
ATOM   5497 CE1 PHE B1009   21.539 12.444 41.021 1.00 50.31   C
ANISOU 5497 CE1 PHE B1009   6673   6827   5615 -1810   -249   494   C
ATOM   5498 CE2 PHE B1009   23.283 11.749 42.534 1.00 50.86   C
ANISOU 5498 CE2 PHE B1009   6999   6527   5799 -1575   -189   418   C
ATOM   5499 CZ PHE B1009   22.819 12.596 41.536 1.00 48.82   C
ANISOU 5499 CZ PHE B1009   6578   6424   5547 -1570   -204   422   C
ATOM   5500 H   PHE B1009   20.983 10.144 45.373 1.00 42.61   H
ATOM   5501 HA PHE B1009   18.840 10.996 43.757 1.00 43.94   H
ATOM   5502 HB3 PHE B1009   19.664   9.091 42.253 1.00 45.46   H
ATOM   5503 HB2 PHE B1009   20.823   8.699 43.495 1.00 45.46   H
ATOM   5504 HD1 PHE B1009   19.710 11.337 41.104 1.00 49.60   H
ATOM   5505 HD2 PHE B1009   22.832 10.096 43.816 1.00 48.15   H
ATOM   5506 HE1 PHE B1009   21.183 13.120 40.258 1.00 50.31   H
ATOM   5507 HE2 PHE B1009   24.276 11.886 42.937 1.00 50.86   H
ATOM   5508 HZ PHE B1009   23.451 13.387 41.163 1.00 48.82   H
ATOM   5509 N   LYS B1010   16.956   9.390 43.785 1.00 51.04   N
ANISOU 5509 N   LYS B1010   6588   7711   5093 -2973   -397   971   N
ATOM   5510 CA LYS B1010   15.721   8.619 43.915 1.00 52.28   C
ANISOU 5510 CA LYS B1010   6716   8103   5046 -3366   -455   1099   C
ATOM   5511 C   LYS B1010   15.459   7.773 42.660 1.00 58.62   C
ANISOU 5511 C   LYS B1010   7738   8840   5694 -3730   -526   1085   C
ATOM   5512 O   LYS B1010   15.773   8.196 41.547 1.00 59.38   O
ANISOU 5512 O   LYS B1010   7829   8911   5821 -3676   -536   1027   O
ATOM   5513 CB LYS B1010   14.562   9.594 44.192 1.00 55.32   C
ANISOU 5513 CB LYS B1010   6667   8985   5369 -3345   -457   1255   C
ATOM   5514 CG LYS B1010   14.572 10.109 45.645 1.00 70.15   C
ANISOU 5514 CG LYS B1010   8378 10931   7346 -3047   -389   1289   C
ATOM   5515 CD LYS B1010   13.630 11.295 45.890 1.00 79.54   C
ANISOU 5515 CD LYS B1010   9155 12581   8487 -2906   -365   1427   C

```
ATOM   5516  CE  LYS B1010      14.207  12.609  45.344  1.00 88.86           C
ANISOU 5516  CE  LYS B1010    10226  13716   9821  -2499   -308   1357       C
ATOM   5517  NZ  LYS B1010      13.329  13.758  45.618  1.00 95.54           N1+
ANISOU 5517  NZ  LYS B1010    10710  15002  10590  -2350   -280   1496       N1+
ATOM   5518  H   LYS B1010      16.898  10.217  43.205  1.00 51.04           H
ATOM   5519  HA  LYS B1010      15.800   7.939  44.766  1.00 52.28           H
ATOM   5520  HB3 LYS B1010      13.605   9.096  44.027  1.00 55.32           H
ATOM   5521  HB2 LYS B1010      14.599  10.415  43.476  1.00 55.32           H
ATOM   5522  HG3 LYS B1010      15.584  10.389  45.944  1.00 70.15           H
ATOM   5523  HG2 LYS B1010      14.288   9.291  46.307  1.00 70.15           H
ATOM   5524  HD3 LYS B1010      13.447  11.385  46.962  1.00 79.54           H
ATOM   5525  HD2 LYS B1010      12.662  11.091  45.430  1.00 79.54           H
ATOM   5526  HE3 LYS B1010      14.383  12.538  44.273  1.00 88.86           H
ATOM   5527  HE2 LYS B1010      15.173  12.798  45.806  1.00 88.86           H
ATOM   5528  HZ1 LYS B1010      13.239  13.862  46.621  1.00 95.54           H
ATOM   5529  HZ2 LYS B1010      13.745  14.595  45.237  1.00 95.54           H
ATOM   5530  HZ3 LYS B1010      12.422  13.604  45.202  1.00 95.54           H
ATOM   5531  N   THR B1011      14.877   6.589  42.894  1.00 56.23           N
ANISOU 5531  N   THR B1011     7654   8496   5213  -4113   -575   1136       N
ATOM   5532  CA  THR B1011      14.535   5.578  41.893  1.00 94.12           C
ANISOU 5532  CA  THR B1011    12723  13218   9821  -4525   -650   1131       C
ATOM   5533  C   THR B1011      13.156   4.962  42.232  1.00129.01           C
ANISOU 5533  C   THR B1011    17005  18014  14000  -4984   -729   1301       C
ATOM   5534  O   THR B1011      12.514   5.379  43.199  1.00 92.15           O
ANISOU 5534  O   THR B1011    11903  13806   9304  -4965   -738   1435       O
ATOM   5535  CB  THR B1011      15.617   4.454  41.844  1.00102.24           C
ANISOU 5535  CB  THR B1011    14290  13724  10832  -4568   -633   1003       C
ATOM   5536  CG2 THR B1011      17.048   4.962  41.597  1.00100.88           C
ANISOU 5536  CG2 THR B1011    14263  13227  10839  -4191   -571    847       C
ATOM   5537  OG1 THR B1011      15.617   3.639  43.004  1.00102.00           O
ANISOU 5537  OG1 THR B1011    14378  13565  10811  -4538   -600   1023       O
ATOM   5538  H   THR B1011      14.640   6.338  43.843  1.00 56.23           H
ATOM   5539  HA  THR B1011      14.448   6.043  40.909  1.00 94.12           H
ATOM   5540  HB  THR B1011      15.381   3.795  41.008  1.00102.24           H
ATOM   5541 HG21 THR B1011      17.737   4.128  41.454  1.00100.88           H
ATOM   5542 HG22 THR B1011      17.095   5.584  40.703  1.00100.88           H
ATOM   5543 HG23 THR B1011      17.421   5.552  42.435  1.00100.88           H
ATOM   5544  HG1 THR B1011      14.775   3.180  43.061  1.00102.00           H
TER
HETATM 5545  H   NMA B1011A     13.276   3.696  40.633  1.00  0.00           H
HETATM 5546  N   NMA B1011A     12.718   3.983  41.425  1.00  0.00           N
HETATM 5547  CA  NMA B1011A     11.466   3.275  41.646  1.00  0.00           C
HETATM 5548 1HA  NMA B1011A     10.635   3.971  41.767  1.00  0.00           H
HETATM 5549 2HA  NMA B1011A     11.247   2.631  40.794  1.00  0.00           H
HETATM 5550 3HA  NMA B1011A     11.528   2.649  42.537  1.00  0.00           H
ATOM   5551  HE2 HIE B 822      15.589  18.273  44.423  1.00 46.49           H
ATOM   5552  HE2 HIE B 826      15.852  22.778  36.831  1.00 52.41           H
ATOM   5553  HE2 HIE B 855      21.237  50.083  41.325  1.00 70.46           H
ATOM   5554  HE2 HIE B 934      10.346  35.708  26.036  1.00 46.18           H
ATOM   5555  HE2 HIE B 946      28.088  51.396  38.591  1.00 50.86           H
TER
HETATM 5556  C1       900      32.945  25.390  34.561  1.00  0.00           C
HETATM 5557  C2       900      31.931  25.819  35.631  1.00  0.00           C
HETATM 5558  C3       900      30.587  25.048  35.625  1.00  0.00           C
HETATM 5559  C4       900      30.679  23.527  35.905  1.00  0.00           C
HETATM 5560  O1       900      31.063  22.774  34.773  1.00  0.00           O
```

```
HETATM 5561  N1        900      29.613  25.690  36.521  1.00  0.00           N
HETATM 5562  C5        900      28.774  26.672  36.171  1.00  0.00           C
HETATM 5563  O2        900      28.807  27.231  35.077  1.00  0.00           O
HETATM 5564  N2        900      27.875  26.908  37.136  1.00  0.00           N
HETATM 5565  C6        900      26.692  27.658  37.053  1.00  0.00           C
HETATM 5566  C7        900      26.130  28.091  38.255  1.00  0.00           C
HETATM 5567  C8        900      24.919  28.782  38.252  1.00  0.00           C
HETATM 5568  C9        900      24.274  29.034  37.043  1.00  0.00           C
HETATM 5569 CL1        900      22.766  29.861  37.019  1.00  0.00          Cl
HETATM 5570  C10       900      24.830  28.613  35.840  1.00  0.00           C
HETATM 5571  C11       900      26.036  27.924  35.842  1.00  0.00           C
HETATM 5572  H1        900      33.815  26.047  34.562  1.00  0.00           H
HETATM 5573  H2        900      32.510  25.420  33.561  1.00  0.00           H
HETATM 5574  H3        900      33.302  24.383  34.749  1.00  0.00           H
HETATM 5575  H4        900      32.389  25.758  36.620  1.00  0.00           H
HETATM 5576  H5        900      31.725  26.881  35.476  1.00  0.00           H
HETATM 5577  H6        900      30.168  25.126  34.615  1.00  0.00           H
HETATM 5578  H7        900      31.361  23.315  36.724  1.00  0.00           H
HETATM 5579  H8        900      29.700  23.143  36.199  1.00  0.00           H
HETATM 5580  H9        900      31.988  22.491  34.897  1.00  0.00           H
HETATM 5581  H10       900      29.611  25.413  37.492  1.00  0.00           H
HETATM 5582  H11       900      28.083  26.491  38.036  1.00  0.00           H
HETATM 5583  H12       900      26.630  27.892  39.190  1.00  0.00           H
HETATM 5584  H13       900      24.475  29.111  39.181  1.00  0.00           H
HETATM 5585  H14       900      24.315  28.800  34.909  1.00  0.00           H
HETATM 5586  H15       900      26.430  27.573  34.900  1.00  0.00           H
CONECT    1    2    4    5    6
CONECT    2    1    3    7
CONECT    3    2
CONECT    4    1
CONECT    5    1
CONECT    6    1
CONECT    7    2
CONECT 5533 5546
CONECT 5545 5546
CONECT 5546 5533 5545 5547
CONECT 5547 5546 5548 5549 5550
CONECT 5548 5547
CONECT 5549 5547
CONECT 5550 5547
CONECT 5556 5557 5572 5573 5574
CONECT 5557 5556 5558 5575 5576
CONECT 5558 5557 5559 5561 5577
CONECT 5559 5558 5560 5578 5579
CONECT 5560 5559 5580
CONECT 5561 5558 5562 5581
CONECT 5562 5561 5563 5564
CONECT 5563 5562
CONECT 5564 5562 5565 5582
CONECT 5565 5564 5566 5571
CONECT 5566 5565 5567 5583
CONECT 5567 5566 5568 5584
CONECT 5568 5567 5569 5570
CONECT 5569 5568
CONECT 5570 5568 5571 5585
CONECT 5571 5565 5570 5586
```

CONECT 5572 5556
CONECT 5573 5556
CONECT 5574 5556
CONECT 5575 5557
CONECT 5576 5557
CONECT 5577 5558
CONECT 5578 5559
CONECT 5579 5559
CONECT 5580 5560
CONECT 5581 5561
CONECT 5582 5564
CONECT 5583 5566
CONECT 5584 5567
CONECT 5585 5570
CONECT 5586 5571
END

PARP1_PDB_FRAG_VS1_complex

HETATM 1 CH3 ACE B 661 54.735 14.649 60.442 1.00 0.00 C
HETATM 2 C ACE B 661 53.315 14.962 59.985 1.00 0.00 C
HETATM 3 O ACE B 661 53.143 15.728 59.040 1.00 0.00 O
HETATM 4 1H ACE B 661 55.322 14.259 59.611 1.00 0.00 H
HETATM 5 2H ACE B 661 55.219 15.555 60.805 1.00 0.00 H
HETATM 6 3H ACE B 661 54.746 13.910 61.245 1.00 0.00 H
ATOM 7 N LYS B 662 52.316 14.361 60.658 1.00 47.37 N
ANISOU 7 N LYS B 662 4539 8722 4740 1624 -289 1575 N
ATOM 8 CA LYS B 662 50.884 14.490 60.367 1.00 46.49 C
ANISOU 8 CA LYS B 662 4699 8174 4790 1530 -270 1414 C
ATOM 9 C LYS B 662 50.321 15.917 60.490 1.00 46.77 C
ANISOU 9 C LYS B 662 4758 8118 4896 1171 -365 1309 C
ATOM 10 O LYS B 662 50.765 16.697 61.334 1.00 46.53 O
ANISOU 10 O LYS B 662 4632 8257 4790 983 -456 1346 O
ATOM 11 CB LYS B 662 50.083 13.544 61.287 1.00 49.43 C
ANISOU 11 CB LYS B 662 5227 8382 5170 1673 -235 1417 C
ATOM 12 CG LYS B 662 50.124 12.069 60.858 1.00 63.07 C
ANISOU 12 CG LYS B 662 7116 9959 6889 2005 -113 1442 C
ATOM 13 CD LYS B 662 49.141 11.211 61.671 1.00 71.72 C
ANISOU 13 CD LYS B 662 8477 10707 8067 2016 -78 1360 C
ATOM 14 CE LYS B 662 49.034 9.769 61.152 1.00 84.00 C
ANISOU 14 CE LYS B 662 10262 12040 9614 2280 39 1358 C
ATOM 15 NZ LYS B 662 48.051 8.988 61.924 1.00 93.15 N1+
ANISOU 15 NZ LYS B 662 11688 12847 10859 2216 63 1266 N1+
ATOM 16 H1 LYS B 662 52.546 13.750 61.429 1.00 47.37 H
ATOM 17 HA LYS B 662 50.751 14.167 59.333 1.00 46.49 H
ATOM 18 HB3 LYS B 662 49.032 13.842 61.267 1.00 49.43 H
ATOM 19 HB2 LYS B 662 50.399 13.657 62.325 1.00 49.43 H
ATOM 20 HG3 LYS B 662 51.137 11.678 60.965 1.00 63.07 H
ATOM 21 HG2 LYS B 662 49.876 11.996 59.798 1.00 63.07 H
ATOM 22 HD3 LYS B 662 48.156 11.681 61.654 1.00 71.72 H
ATOM 23 HD2 LYS B 662 49.458 11.205 62.715 1.00 71.72 H
ATOM 24 HE3 LYS B 662 50.003 9.272 61.206 1.00 84.00 H
ATOM 25 HE2 LYS B 662 48.729 9.768 60.105 1.00 84.00 H
ATOM 26 HZ1 LYS B 662 47.144 9.428 61.860 1.00 93.15 H
ATOM 27 HZ2 LYS B 662 47.996 8.050 61.554 1.00 93.15 H
ATOM 28 HZ3 LYS B 662 48.338 8.950 62.892 1.00 93.15 H

ATOM 29 N SER B 663 49.302 16.180 59.656 1.00 40.06 N
ANISOU 29 N SER B 663 4053 6996 4173 1084 -340 1184 N
ATOM 30 CA SER B 663 48.497 17.396 59.598 1.00 38.57 C
ANISOU 30 CA SER B 663 3929 6678 4046 795 -406 1081 C
ATOM 31 C SER B 663 47.720 17.661 60.900 1.00 42.31 C
ANISOU 31 C SER B 663 4537 6993 4547 689 -441 1028 C
ATOM 32 O SER B 663 47.110 16.743 61.452 1.00 41.53 O
ANISOU 32 O SER B 663 4546 6748 4486 838 -390 1022 O
ATOM 33 CB SER B 663 47.554 17.280 58.380 1.00 39.61 C
ANISOU 33 CB SER B 663 4185 6566 4299 786 -355 974 C
ATOM 34 OG SER B 663 46.699 18.396 58.242 1.00 42.62 O
ANISOU 34 OG SER B 663 4649 6813 4733 546 -406 880 O
ATOM 35 H SER B 663 49.046 15.474 58.976 1.00 40.06 H
ATOM 36 HA SER B 663 49.181 18.229 59.434 1.00 38.57 H
ATOM 37 HB3 SER B 663 46.946 16.377 58.444 1.00 39.61 H
ATOM 38 HB2 SER B 663 48.139 17.201 57.463 1.00 39.61 H
ATOM 39 HG SER B 663 47.225 19.121 57.882 1.00 42.62 H
ATOM 40 N LYS B 664 47.740 18.934 61.324 1.00 37.56 N
ANISOU 40 N LYS B 664 3943 6417 3910 432 -526 994 N
ATOM 41 CA LYS B 664 47.018 19.450 62.489 1.00 36.33 C
ANISOU 41 CA LYS B 664 3927 6115 3760 319 -560 941 C
ATOM 42 C LYS B 664 45.661 20.081 62.116 1.00 36.72 C
ANISOU 42 C LYS B 664 4162 5871 3917 230 -536 814 C
ATOM 43 O LYS B 664 44.959 20.537 63.019 1.00 36.19 O
ANISOU 43 O LYS B 664 4231 5660 3859 164 -547 764 O
ATOM 44 CB LYS B 664 47.919 20.464 63.225 1.00 39.27 C
ANISOU 44 CB LYS B 664 4231 6698 3991 99 -667 992 C
ATOM 45 CG LYS B 664 49.188 19.835 63.828 1.00 56.08 C
ANISOU 45 CG LYS B 664 6171 9141 5995 180 -698 1130 C
ATOM 46 CD LYS B 664 50.035 20.854 64.604 1.00 68.58 C
ANISOU 46 CD LYS B 664 7692 10943 7421 -96 -817 1179 C
ATOM 47 CE LYS B 664 51.309 20.230 65.194 1.00 82.30 C
ANISOU 47 CE LYS B 664 9165 13099 9007 -59 -861 1341 C
ATOM 48 NZ LYS B 664 52.114 21.221 65.930 1.00 92.80 N1+
ANISOU 48 NZ LYS B 664 10423 14669 10166 -394 -988 1391 N1+
ATOM 49 H LYS B 664 48.266 19.615 60.796 1.00 37.56 H
ATOM 50 HA LYS B 664 46.803 18.630 63.177 1.00 36.33 H
ATOM 51 HB3 LYS B 664 47.358 20.926 64.039 1.00 39.27 H
ATOM 52 HB2 LYS B 664 48.195 21.275 62.548 1.00 39.27 H
ATOM 53 HG3 LYS B 664 49.795 19.394 63.036 1.00 56.08 H
ATOM 54 HG2 LYS B 664 48.908 19.015 64.490 1.00 56.08 H
ATOM 55 HD3 LYS B 664 49.429 21.285 65.404 1.00 68.58 H
ATOM 56 HD2 LYS B 664 50.297 21.680 63.940 1.00 68.58 H
ATOM 57 HE3 LYS B 664 51.922 19.803 64.399 1.00 82.30 H
ATOM 58 HE2 LYS B 664 51.051 19.417 65.874 1.00 82.30 H
ATOM 59 HZ1 LYS B 664 51.569 21.602 66.690 1.00 92.80 H
ATOM 60 HZ2 LYS B 664 52.941 20.775 66.301 1.00 92.80 H
ATOM 61 HZ3 LYS B 664 52.388 21.967 65.306 1.00 92.80 H
ATOM 62 N LEU B 665 45.300 20.090 60.817 1.00 31.05 N
ANISOU 62 N LEU B 665 3445 5082 3269 244 -499 770 N
ATOM 63 CA LEU B 665 44.015 20.576 60.300 1.00 29.43 C
ANISOU 63 CA LEU B 665 3388 4637 3156 186 -470 665 C
ATOM 64 C LEU B 665 42.829 19.737 60.833 1.00 31.11 C
ANISOU 64 C LEU B 665 3719 4648 3453 307 -406 627 C
ATOM 65 O LEU B 665 43.019 18.547 61.094 1.00 29.28 O
ANISOU 65 O LEU B 665 3464 4424 3236 466 -363 670 O

ATOM	66	CB	LEU	B	665	44.026	20.499	58.754	1.00	29.18		C
ANISOU	66	CB	LEU	B	665	3315	4593	3180	210	-436	641	C
ATOM	67	CG	LEU	B	665	44.998	21.468	58.043	1.00	33.89		C
ANISOU	67	CG	LEU	B	665	3821	5350	3706	55	-493	662	C
ATOM	68	CD1	LEU	B	665	45.111	21.124	56.541	1.00	33.93		C
ANISOU	68	CD1	LEU	B	665	3764	5362	3765	132	-446	654	C
ATOM	69	CD2	LEU	B	665	44.623	22.948	58.275	1.00	35.93		C
ANISOU	69	CD2	LEU	B	665	4217	5501	3935	-149	-539	597	C
ATOM	70	H	LEU	B	665	45.922	19.683	60.133	1.00	31.05		H
ATOM	71	HA	LEU	B	665	43.933	21.613	60.618	1.00	29.43		H
ATOM	72	HB3	LEU	B	665	43.023	20.680	58.363	1.00	29.18		H
ATOM	73	HB2	LEU	B	665	44.264	19.475	58.467	1.00	29.18		H
ATOM	74	HG	LEU	B	665	45.990	21.312	58.468	1.00	33.89		H
ATOM	75	HD11	LEU	B	665	46.154	21.005	56.246	1.00	33.93		H
ATOM	76	HD12	LEU	B	665	44.600	20.193	56.294	1.00	33.93		H
ATOM	77	HD13	LEU	B	665	44.679	21.891	55.901	1.00	33.93		H
ATOM	78	HD21	LEU	B	665	44.651	23.539	57.360	1.00	35.93		H
ATOM	79	HD22	LEU	B	665	43.622	23.057	58.694	1.00	35.93		H
ATOM	80	HD23	LEU	B	665	45.319	23.415	58.972	1.00	35.93		H
ATOM	81	N	PRO	B	666	41.614	20.335	60.928	1.00	26.82		N
ANISOU	81	N	PRO	B	666	3310	3931	2951	243	-394	556	N
ATOM	82	CA	PRO	B	666	40.361	19.579	61.151	1.00	26.38		C
ANISOU	82	CA	PRO	B	666	3341	3720	2962	338	-335	533	C
ATOM	83	C	PRO	B	666	40.140	18.449	60.129	1.00	29.72		C
ANISOU	83	C	PRO	B	666	3754	4079	3457	447	-274	530	C
ATOM	84	O	PRO	B	666	40.435	18.646	58.952	1.00	28.92		O
ANISOU	84	O	PRO	B	666	3613	3995	3381	432	-269	510	O
ATOM	85	CB	PRO	B	666	39.260	20.648	61.025	1.00	28.18		C
ANISOU	85	CB	PRO	B	666	3679	3824	3203	257	-329	469	C
ATOM	86	CG	PRO	B	666	39.948	21.962	61.342	1.00	32.64		C
ANISOU	86	CG	PRO	B	666	4267	4452	3682	122	-392	461	C
ATOM	87	CD	PRO	B	666	41.344	21.765	60.770	1.00	28.27		C
ANISOU	87	CD	PRO	B	666	3580	4057	3103	92	-428	501	C
ATOM	88	HA	PRO	B	666	40.386	19.182	62.168	1.00	26.38		H
ATOM	89	HB3	PRO	B	666	38.414	20.459	61.688	1.00	28.18		H
ATOM	90	HB2	PRO	B	666	38.869	20.691	60.007	1.00	28.18		H
ATOM	91	HG3	PRO	B	666	40.013	22.083	62.424	1.00	32.64		H
ATOM	92	HG2	PRO	B	666	39.431	22.832	60.935	1.00	32.64		H
ATOM	93	HD2	PRO	B	666	41.366	22.017	59.708	1.00	28.27		H
ATOM	94	HD3	PRO	B	666	42.051	22.405	61.297	1.00	28.27		H
ATOM	95	N	LYS	B	667	39.622	17.299	60.591	1.00	26.37		N
ANISOU	95	N	LYS	B	667	3386	3579	3053	547	-230	551	N
ATOM	96	CA	LYS	B	667	39.318	16.132	59.753	1.00	26.07		C
ANISOU	96	CA	LYS	B	667	3400	3448	3058	635	-172	548	C
ATOM	97	C	LYS	B	667	38.403	16.402	58.525	1.00	28.43		C
ANISOU	97	C	LYS	B	667	3727	3655	3419	561	-152	484	C
ATOM	98	O	LYS	B	667	38.733	15.882	57.459	1.00	28.02		O
ANISOU	98	O	LYS	B	667	3664	3602	3382	602	-132	478	O
ATOM	99	CB	LYS	B	667	38.848	14.960	60.651	1.00	27.90		C
ANISOU	99	CB	LYS	B	667	3736	3582	3282	703	-134	575	C
ATOM	100	CG	LYS	B	667	38.416	13.661	59.940	1.00	41.16		C
ANISOU	100	CG	LYS	B	667	5528	5132	4979	765	-75	571	C
ATOM	101	CD	LYS	B	667	39.561	12.943	59.197	1.00	52.81		C
ANISOU	101	CD	LYS	B	667	7013	6649	6404	930	-47	620	C
ATOM	102	CE	LYS	B	667	39.123	11.664	58.458	1.00	66.40		C
ANISOU	102	CE	LYS	B	667	8882	8220	8127	973	11	600	C

```
ATOM    103  NZ  LYS B 667      38.679  10.595  59.369  1.00 74.16           N1+
ANISOU  103  NZ  LYS B 667    10047   9032   9098    938     43    599       N1+
ATOM    104  H   LYS B 667      39.409  17.208  61.574  1.00 26.37           H
ATOM    105  HA  LYS B 667      40.283  15.833  59.341  1.00 26.07           H
ATOM    106  HB3 LYS B 667      38.011  15.292  61.266  1.00 27.90           H
ATOM    107  HB2 LYS B 667      39.642  14.719  61.359  1.00 27.90           H
ATOM    108  HG3 LYS B 667      37.597  13.860  59.248  1.00 41.16           H
ATOM    109  HG2 LYS B 667      37.999  12.994  60.694  1.00 41.16           H
ATOM    110  HD3 LYS B 667      40.362  12.710  59.900  1.00 52.81           H
ATOM    111  HD2 LYS B 667      40.001  13.622  58.466  1.00 52.81           H
ATOM    112  HE3 LYS B 667      39.950  11.285  57.857  1.00 66.40           H
ATOM    113  HE2 LYS B 667      38.307  11.884  57.773  1.00 66.40           H
ATOM    114  HZ1 LYS B 667      37.886  10.919  59.902  1.00 74.16           H
ATOM    115  HZ2 LYS B 667      38.412   9.784  58.830  1.00 74.16           H
ATOM    116  HZ3 LYS B 667      39.431  10.350  59.997  1.00 74.16           H
ATOM    117  N   PRO B 668      37.366  17.275  58.632  1.00 25.33           N
ANISOU  117  N   PRO B 668     3365   3209   3051    468   -157    444       N
ATOM    118  CA  PRO B 668      36.622  17.781  57.459  1.00 24.47           C
ANISOU  118  CA  PRO B 668     3261   3049   2987    414   -139    397       C
ATOM    119  C   PRO B 668      37.469  18.509  56.399  1.00 27.94           C
ANISOU  119  C   PRO B 668     3635   3552   3429    385   -164    376       C
ATOM    120  O   PRO B 668      37.263  18.265  55.211  1.00 27.61           O
ANISOU  120  O   PRO B 668     3593   3475   3423    377   -143    349       O
ATOM    121  CB  PRO B 668      35.564  18.714  58.070  1.00 25.94           C
ANISOU  121  CB  PRO B 668     3479   3209   3169    358   -136    381       C
ATOM    122  CG  PRO B 668      35.327  18.155  59.458  1.00 29.54           C
ANISOU  122  CG  PRO B 668     3969   3663   3592    388   -135    417       C
ATOM    123  CD  PRO B 668      36.726  17.725  59.872  1.00 25.30           C
ANISOU  123  CD  PRO B 668     3395   3196   3022    430   -169    446       C
ATOM    124  HA  PRO B 668      36.122  16.927  56.996  1.00 24.47           H
ATOM    125  HB3 PRO B 668      34.650  18.765  57.481  1.00 25.94           H
ATOM    126  HB2 PRO B 668      35.947  19.731  58.161  1.00 25.94           H
ATOM    127  HG3 PRO B 668      34.674  17.284  59.393  1.00 29.54           H
ATOM    128  HG2 PRO B 668      34.871  18.871  60.142  1.00 29.54           H
ATOM    129  HD2 PRO B 668      37.264  18.586  60.267  1.00 25.30           H
ATOM    130  HD3 PRO B 668      36.681  16.960  60.645  1.00 25.30           H
ATOM    131  N   VAL B 669      38.405  19.368  56.843  1.00 24.96           N
ANISOU  131  N   VAL B 669     3206   3274   3003    354   -210    392       N
ATOM    132  CA  VAL B 669      39.322  20.121  55.979  1.00 25.22           C
ANISOU  132  CA  VAL B 669     3175   3389   3021    301   -239    384       C
ATOM    133  C   VAL B 669      40.354  19.207  55.288  1.00 30.46           C
ANISOU  133  C   VAL B 669     3762   4125   3684    396   -220    420       C
ATOM    134  O   VAL B 669      40.627  19.423  54.111  1.00 30.19           O
ANISOU  134  O   VAL B 669     3701   4099   3673    390   -208    400       O
ATOM    135  CB  VAL B 669      40.059  21.260  56.751  1.00 28.83           C
ANISOU  135  CB  VAL B 669     3617   3937   3398    196   -302    396       C
ATOM    136  CG1 VAL B 669      41.167  21.987  55.955  1.00 28.32           C
ANISOU  136  CG1 VAL B 669     3485   3973   3303    116   -336    400       C
ATOM    137  CG2 VAL B 669      39.058  22.301  57.279  1.00 28.61           C
ANISOU  137  CG2 VAL B 669     3709   3809   3352    134   -307    357       C
ATOM    138  H   VAL B 669      38.533  19.495  57.836  1.00 24.96           H
ATOM    139  HA  VAL B 669      38.720  20.586  55.195  1.00 25.22           H
ATOM    140  HB  VAL B 669      40.545  20.823  57.623  1.00 28.83           H
ATOM    141 HG11 VAL B 669      41.566  22.831  56.518  1.00 28.32           H
ATOM    142 HG12 VAL B 669      42.015  21.337  55.736  1.00 28.32           H
ATOM    143 HG13 VAL B 669      40.787  22.375  55.009  1.00 28.32           H
```

```
ATOM    144 HG21 VAL B 669      39.558  23.075  57.863  1.00 28.61           H
ATOM    145 HG22 VAL B 669      38.530  22.791  56.462  1.00 28.61           H
ATOM    146 HG23 VAL B 669      38.314  21.839  57.925  1.00 28.61           H
ATOM    147  N   GLN B 670      40.864  18.179  55.994  1.00 27.83           N
ANISOU  147  N   GLN B 670     3414   3842   3319    503   -208    476       N
ATOM    148  CA  GLN B 670      41.766  17.167  55.432  1.00 27.57           C
ANISOU  148  CA  GLN B 670     3336   3878   3259    645   -174    524       C
ATOM    149  C   GLN B 670      41.125  16.355  54.290  1.00 31.51           C
ANISOU  149  C   GLN B 670     3938   4226   3810    702   -114    484       C
ATOM    150  O   GLN B 670      41.773  16.153  53.264  1.00 32.41           O
ANISOU  150  O   GLN B 670     4023   4379   3911    772    -88    493       O
ATOM    151  CB  GLN B 670      42.293  16.222  56.532  1.00 28.39           C
ANISOU  151  CB  GLN B 670     3437   4044   3306    773   -163    596       C
ATOM    152  CG  GLN B 670      43.206  16.905  57.572  1.00 37.64           C
ANISOU  152  CG  GLN B 670     4499   5395   4406    713   -227    648       C
ATOM    153  CD  GLN B 670      43.979  15.909  58.441  1.00 43.61           C
ANISOU  153  CD  GLN B 670     5223   6255   5091    874   -212    736       C
ATOM    154  NE2 GLN B 670      44.223  16.263  59.703  1.00 30.39           N
ANISOU  154  NE2 GLN B 670     3527   4646   3373    823   -256    766       N
ATOM    155  OE1 GLN B 670      44.391  14.847  57.978  1.00 37.47           O
ANISOU  155  OE1 GLN B 670     4451   5503   4284   1056   -156    783       O
ATOM    156  H   GLN B 670      40.598  18.061  56.963  1.00 27.83           H
ATOM    157  HA  GLN B 670      42.618  17.699  55.005  1.00 27.57           H
ATOM    158  HB3 GLN B 670      42.844  15.418  56.042  1.00 28.39           H
ATOM    159  HB2 GLN B 670      41.460  15.740  57.045  1.00 28.39           H
ATOM    160  HG3 GLN B 670      42.620  17.566  58.207  1.00 37.64           H
ATOM    161  HG2 GLN B 670      43.939  17.538  57.074  1.00 37.64           H
ATOM    162 HE22 GLN B 670      44.767  15.660  60.303  1.00 30.39           H
ATOM    163 HE21 GLN B 670      43.881  17.142  60.071  1.00 30.39           H
ATOM    164  N   ASP B 671      39.857  15.948  54.479  1.00 26.57           N
ANISOU  164  N   ASP B 671     3429   3440   3226    659    -93    445       N
ATOM    165  CA  ASP B 671      39.042  15.244  53.483  1.00 26.71           C
ANISOU  165  CA  ASP B 671     3561   3314   3274    661    -49    409       C
ATOM    166  C   ASP B 671      38.657  16.125  52.282  1.00 30.03           C
ANISOU  166  C   ASP B 671     3938   3735   3735    574    -58    359       C
ATOM    167  O   ASP B 671      38.536  15.592  51.180  1.00 30.85           O
ANISOU  167  O   ASP B 671     4092   3789   3842    608    -26    342       O
ATOM    168  CB  ASP B 671      37.763  14.609  54.085  1.00 27.88           C
ANISOU  168  CB  ASP B 671     3820   3339   3435    603    -35    399       C
ATOM    169  CG  ASP B 671      37.973  13.601  55.229  1.00 38.78           C
ANISOU  169  CG  ASP B 671     5285   4682   4768    701    -13    449       C
ATOM    170  OD1 ASP B 671      39.074  13.009  55.322  1.00 39.94           O
ANISOU  170  OD1 ASP B 671     5435   4876   4865    848      7    492       O
ATOM    171  OD2 ASP B 671      36.961  13.329  55.913  1.00 42.49           O1-
ANISOU  171  OD2 ASP B 671     5817   5088   5239    643    -12    453       O1-
ATOM    172  H   ASP B 671      39.405  16.139  55.364  1.00 26.57           H
ATOM    173  HA  ASP B 671      39.657  14.437  53.081  1.00 26.71           H
ATOM    174  HB3 ASP B 671      37.207  14.093  53.301  1.00 27.88           H
ATOM    175  HB2 ASP B 671      37.118  15.405  54.462  1.00 27.88           H
ATOM    176  N   LEU B 672      38.493  17.442  52.503  1.00 25.11           N
ANISOU  176  N   LEU B 672     3243   3168   3132    472    -99    337       N
ATOM    177  CA  LEU B 672      38.217  18.428  51.457  1.00 24.46           C
ANISOU  177  CA  LEU B 672     3124   3094   3076    397   -109    296       C
ATOM    178  C   LEU B 672      39.413  18.610  50.502  1.00 28.13           C
ANISOU  178  C   LEU B 672     3513   3656   3521    440   -112    310       C
ATOM    179  O   LEU B 672      39.199  18.672  49.293  1.00 26.70           O
```

ANISOU 179 O LEU B 672 3343 3440 3360 440 -91 283 O
ATOM 180 CB LEU B 672 37.768 19.758 52.110 1.00 23.67 C
ANISOU 180 CB LEU B 672 3008 3015 2972 305 -145 278 C
ATOM 181 CG LEU B 672 37.306 20.862 51.130 1.00 27.58 C
ANISOU 181 CG LEU B 672 3486 3516 3476 236 -156 241 C
ATOM 182 CD1 LEU B 672 36.073 20.423 50.314 1.00 26.85 C
ANISOU 182 CD1 LEU B 672 3430 3348 3423 224 -123 211 C
ATOM 183 CD2 LEU B 672 37.075 22.198 51.869 1.00 27.89 C
ANISOU 183 CD2 LEU B 672 3555 3569 3473 167 -190 234 C
ATOM 184 H LEU B 672 38.588 17.802 53.443 1.00 25.11 H
ATOM 185 HA LEU B 672 37.385 18.040 50.867 1.00 24.46 H
ATOM 186 HB3 LEU B 672 38.588 20.148 52.712 1.00 23.67 H
ATOM 187 HB2 LEU B 672 36.957 19.557 52.812 1.00 23.67 H
ATOM 188 HG LEU B 672 38.114 21.051 50.425 1.00 27.58 H
ATOM 189 HD11 LEU B 672 35.345 21.223 50.183 1.00 26.85 H
ATOM 190 HD12 LEU B 672 36.369 20.093 49.318 1.00 26.85 H
ATOM 191 HD13 LEU B 672 35.548 19.591 50.785 1.00 26.85 H
ATOM 192 HD21 LEU B 672 37.708 22.983 51.455 1.00 27.89 H
ATOM 193 HD22 LEU B 672 36.046 22.548 51.799 1.00 27.89 H
ATOM 194 HD23 LEU B 672 37.308 22.123 52.932 1.00 27.89 H
ATOM 195 N ILE B 673 40.642 18.646 51.051 1.00 26.33 N
ANISOU 195 N ILE B 673 3197 3565 3240 475 -137 363 N
ATOM 196 CA ILE B 673 41.890 18.728 50.285 1.00 26.57 C
ANISOU 196 CA ILE B 673 3122 3746 3228 523 -139 402 C
ATOM 197 C ILE B 673 42.168 17.453 49.461 1.00 31.65 C
ANISOU 197 C ILE B 673 3815 4348 3864 680 -74 414 C
ATOM 198 O ILE B 673 42.551 17.577 48.299 1.00 30.73 O
ANISOU 198 O ILE B 673 3665 4265 3745 698 -57 406 O
ATOM 199 CB ILE B 673 43.113 19.051 51.200 1.00 28.94 C
ANISOU 199 CB ILE B 673 3300 4249 3448 523 -183 478 C
ATOM 200 CG1 ILE B 673 42.995 20.443 51.866 1.00 29.99 C
ANISOU 200 CG1 ILE B 673 3432 4402 3561 348 -250 463 C
ATOM 201 CG2 ILE B 673 44.494 18.912 50.520 1.00 27.22 C
ANISOU 201 CG2 ILE B 673 2940 4237 3166 573 -182 540 C
ATOM 202 CD1 ILE B 673 42.955 21.630 50.896 1.00 37.80 C
ANISOU 202 CD1 ILE B 673 4457 5339 4567 194 -278 406 C
ATOM 203 H ILE B 673 40.737 18.597 52.057 1.00 26.33 H
ATOM 204 HA ILE B 673 41.774 19.537 49.562 1.00 26.57 H
ATOM 205 HB ILE B 673 43.101 18.330 52.018 1.00 28.94 H
ATOM 206 HG13 ILE B 673 43.813 20.581 52.573 1.00 29.99 H
ATOM 207 HG12 ILE B 673 42.091 20.489 52.465 1.00 29.99 H
ATOM 208 HG21 ILE B 673 45.287 19.261 51.179 1.00 27.22 H
ATOM 209 HG22 ILE B 673 44.733 17.877 50.272 1.00 27.22 H
ATOM 210 HG23 ILE B 673 44.550 19.495 49.602 1.00 27.22 H
ATOM 211 HD11 ILE B 673 43.023 22.571 51.440 1.00 37.80 H
ATOM 212 HD12 ILE B 673 43.778 21.605 50.183 1.00 37.80 H
ATOM 213 HD13 ILE B 673 42.020 21.656 50.337 1.00 37.80 H
ATOM 214 N LYS B 674 41.921 16.267 50.049 1.00 29.27 N
ANISOU 214 N LYS B 674 3614 3961 3546 794 -36 434 N
ATOM 215 CA LYS B 674 41.999 14.970 49.366 1.00 30.18 C
ANISOU 215 CA LYS B 674 3845 3997 3627 954 32 446 C
ATOM 216 C LYS B 674 41.054 14.869 48.156 1.00 35.07 C
ANISOU 216 C LYS B 674 4567 4467 4292 881 53 375 C
ATOM 217 O LYS B 674 41.495 14.458 47.086 1.00 34.65 O
ANISOU 217 O LYS B 674 4534 4420 4210 962 89 374 O
ATOM 218 CB LYS B 674 41.693 13.830 50.358 1.00 33.04 C

```
ANISOU  218  CB  LYS B 674     4349   4249   3954   1055     66    473       C
ATOM    219  CG  LYS B 674     42.836  13.507  51.329  1.00 50.01           C
ANISOU  219  CG  LYS B 674     6413   6556   6032   1192     65    561       C
ATOM    220  CD  LYS B 674     42.440  12.378  52.291  1.00 65.07           C
ANISOU  220  CD  LYS B 674     8487   8325   7911   1271     97    579       C
ATOM    221  CE  LYS B 674     43.590  11.948  53.211  1.00 74.98           C
ANISOU  221  CE  LYS B 674     9729   9698   9063   1504    135    676       C
ATOM    222  NZ  LYS B 674     43.187  10.846  54.102  1.00 84.23           N1+
ANISOU  222  NZ  LYS B 674    11101  10704  10200   1581    171    691       N1+
ATOM    223  H   LYS B 674     41.618  16.246  51.014  1.00 29.27           H
ATOM    224  HA  LYS B 674     43.021  14.852  49.001  1.00 30.18           H
ATOM    225  HB3 LYS B 674     41.487  12.916  49.797  1.00 33.04           H
ATOM    226  HB2 LYS B 674     40.779  14.051  50.909  1.00 33.04           H
ATOM    227  HG3 LYS B 674     43.121  14.396  51.891  1.00 50.01           H
ATOM    228  HG2 LYS B 674     43.718  13.211  50.760  1.00 50.01           H
ATOM    229  HD3 LYS B 674     42.088  11.521  51.714  1.00 65.07           H
ATOM    230  HD2 LYS B 674     41.593  12.710  52.895  1.00 65.07           H
ATOM    231  HE3 LYS B 674     43.908  12.789  53.822  1.00 74.98           H
ATOM    232  HE2 LYS B 674     44.450  11.628  52.622  1.00 74.98           H
ATOM    233  HZ1 LYS B 674     42.908  10.049  53.549  1.00 84.23           H
ATOM    234  HZ2 LYS B 674     43.963  10.591  54.695  1.00 84.23           H
ATOM    235  HZ3 LYS B 674     42.410  11.147  54.674  1.00 84.23           H
ATOM    236  N   MET B 675     39.789  15.270  48.365  1.00 33.21           N
ANISOU  236  N   MET B 675     4382   4123   4114    731     29    323       N
ATOM    237  CA  MET B 675     38.708  15.289  47.378  1.00 33.36           C
ANISOU  237  CA  MET B 675     4477   4029   4169    628     35    264       C
ATOM    238  C   MET B 675     38.993  16.210  46.175  1.00 36.83           C
ANISOU  238  C   MET B 675     4822   4543   4629    593     25    239       C
ATOM    239  O   MET B 675     38.709  15.824  45.041  1.00 36.22           O
ANISOU  239  O   MET B 675     4817   4404   4539    615     55    215       O
ATOM    240  CB  MET B 675     37.406  15.656  48.131  1.00 35.95           C
ANISOU  240  CB  MET B 675     4807   4314   4538    488      5    242       C
ATOM    241  CG  MET B 675     36.149  15.874  47.277  1.00 40.25           C
ANISOU  241  CG  MET B 675     5434   4763   5096    380     13    205       C
ATOM    242  SD  MET B 675     34.674  16.282  48.252  1.00 44.37           S
ANISOU  242  SD  MET B 675     5909   5306   5643    255    -16    206       S
ATOM    243  CE  MET B 675     33.452  16.369  46.919  1.00 40.97           C
ANISOU  243  CE  MET B 675     5510   4847   5212    133    -13    178       C
ATOM    244  H   MET B 675     39.531  15.586  49.290  1.00 33.21           H
ATOM    245  HA  MET B 675     38.600  14.275  46.989  1.00 33.36           H
ATOM    246  HB3 MET B 675     37.573  16.565  48.711  1.00 35.95           H
ATOM    247  HB2 MET B 675     37.193  14.877  48.864  1.00 35.95           H
ATOM    248  HG3 MET B 675     35.944  14.986  46.679  1.00 40.25           H
ATOM    249  HG2 MET B 675     36.311  16.700  46.586  1.00 40.25           H
ATOM    250  HE1 MET B 675     32.523  16.801  47.287  1.00 40.97           H
ATOM    251  HE2 MET B 675     33.817  17.002  46.113  1.00 40.97           H
ATOM    252  HE3 MET B 675     33.247  15.377  46.517  1.00 40.97           H
ATOM    253  N   ILE B 676     39.553  17.399  46.452  1.00 33.05           N
ANISOU  253  N   ILE B 676     4203   4187   4167    533    -19    247       N
ATOM    254  CA  ILE B 676     39.855  18.440  45.469  1.00 34.01           C
ANISOU  254  CA  ILE B 676     4241   4380   4302    476    -35    227       C
ATOM    255  C   ILE B 676     41.075  18.122  44.574  1.00 36.70           C
ANISOU  255  C   ILE B 676     4519   4830   4595    585    -10    264       C
ATOM    256  O   ILE B 676     41.013  18.392  43.374  1.00 35.57           O
ANISOU  256  O   ILE B 676     4354   4702   4459    560     -3    241       O
ATOM    257  CB  ILE B 676     40.013  19.824  46.180  1.00 38.01           C
```

ANISOU 257 CB ILE B 676 4670 4958 4815 362 -90 227 C
ATOM 258 CG1 ILE B 676 38.616 20.358 46.582 1.00 38.64 C
ANISOU 258 CG1 ILE B 676 4808 4941 4932 272 -99 183 C
ATOM 259 CG2 ILE B 676 40.786 20.899 45.389 1.00 40.04 C
ANISOU 259 CG2 ILE B 676 4826 5346 5042 314 -116 243 C
ATOM 260 CD1 ILE B 676 38.645 21.577 47.514 1.00 47.57 C
ANISOU 260 CD1 ILE B 676 5927 6105 6044 197 -140 187 C
ATOM 261 H ILE B 676 39.749 17.635 47.415 1.00 33.05 H
ATOM 262 HA ILE B 676 38.995 18.509 44.798 1.00 34.01 H
ATOM 263 HB ILE B 676 40.574 19.657 47.101 1.00 38.01 H
ATOM 264 HG13 ILE B 676 38.037 19.572 47.067 1.00 38.64 H
ATOM 265 HG12 ILE B 676 38.058 20.610 45.682 1.00 38.64 H
ATOM 266 HG21 ILE B 676 40.805 21.859 45.902 1.00 40.04 H
ATOM 267 HG22 ILE B 676 41.828 20.623 45.235 1.00 40.04 H
ATOM 268 HG23 ILE B 676 40.325 21.048 44.414 1.00 40.04 H
ATOM 269 HD11 ILE B 676 37.685 21.708 48.014 1.00 47.57 H
ATOM 270 HD12 ILE B 676 39.410 21.474 48.284 1.00 47.57 H
ATOM 271 HD13 ILE B 676 38.848 22.493 46.960 1.00 47.57 H
ATOM 272 N PHE B 677 42.143 17.554 45.161 1.00 33.53 N
ANISOU 272 N PHE B 677 4081 4522 4135 715 7 328 N
ATOM 273 CA PHE B 677 43.398 17.228 44.469 1.00 33.88 C
ANISOU 273 CA PHE B 677 4046 4715 4113 850 40 384 C
ATOM 274 C PHE B 677 43.483 15.784 43.933 1.00 39.92 C
ANISOU 274 C PHE B 677 4959 5383 4828 1049 120 395 C
ATOM 275 O PHE B 677 44.557 15.396 43.468 1.00 40.10 O
ANISOU 275 O PHE B 677 4925 5539 4771 1219 163 459 O
ATOM 276 CB PHE B 677 44.603 17.588 45.369 1.00 35.49 C
ANISOU 276 CB PHE B 677 4079 5155 4252 875 8 472 C
ATOM 277 CG PHE B 677 44.934 19.068 45.415 1.00 36.86 C
ANISOU 277 CG PHE B 677 4117 5460 4427 682 -63 474 C
ATOM 278 CD1 PHE B 677 45.653 19.663 44.355 1.00 39.38 C
ANISOU 278 CD1 PHE B 677 4348 5889 4726 639 -65 483 C
ATOM 279 CD2 PHE B 677 44.418 19.890 46.437 1.00 38.74 C
ANISOU 279 CD2 PHE B 677 4341 5709 4672 541 -125 471 C
ATOM 280 CE1 PHE B 677 45.917 21.026 44.373 1.00 39.83 C
ANISOU 280 CE1 PHE B 677 4315 6056 4764 443 -131 489 C
ATOM 281 CE2 PHE B 677 44.696 21.248 46.441 1.00 41.24 C
ANISOU 281 CE2 PHE B 677 4585 6121 4962 352 -189 473 C
ATOM 282 CZ PHE B 677 45.452 21.811 45.421 1.00 38.87 C
ANISOU 282 CZ PHE B 677 4209 5922 4637 297 -193 483 C
ATOM 283 H PHE B 677 42.119 17.357 46.153 1.00 33.53 H
ATOM 284 HA PHE B 677 43.480 17.848 43.574 1.00 33.88 H
ATOM 285 HB3 PHE B 677 45.511 17.085 45.033 1.00 35.49 H
ATOM 286 HB2 PHE B 677 44.437 17.228 46.385 1.00 35.49 H
ATOM 287 HD1 PHE B 677 46.012 19.058 43.534 1.00 39.38 H
ATOM 288 HD2 PHE B 677 43.824 19.468 47.232 1.00 38.74 H
ATOM 289 HE1 PHE B 677 46.485 21.475 43.571 1.00 39.83 H
ATOM 290 HE2 PHE B 677 44.326 21.866 47.244 1.00 41.24 H
ATOM 291 HZ PHE B 677 45.669 22.867 45.441 1.00 38.87 H
ATOM 292 N ASP B 678 42.376 15.018 43.983 1.00 37.79 N
ANISOU 292 N ASP B 678 4886 4887 4584 1025 142 339 N
ATOM 293 CA ASP B 678 42.307 13.623 43.533 1.00 37.93 C
ANISOU 293 CA ASP B 678 5116 4763 4534 1183 215 340 C
ATOM 294 C ASP B 678 42.551 13.515 42.012 1.00 42.54 C
ANISOU 294 C ASP B 678 5751 5328 5086 1240 258 316 C
ATOM 295 O ASP B 678 41.734 13.996 41.227 1.00 41.43 O

```
ANISOU  295  O   ASP B 678     5640   5103   4997   1088    235    250       O
ATOM    296  CB  ASP B 678      40.976  12.954  43.959  1.00 39.70           C
ANISOU  296  CB  ASP B 678     5541   4763   4778   1081    212    292       C
ATOM    297  CG  ASP B 678      40.978  11.424  43.820  1.00 53.54           C
ANISOU  297  CG  ASP B 678     7562   6344   6435   1227    283    302       C
ATOM    298  OD1 ASP B 678      40.546  10.940  42.751  1.00 54.74           O
ANISOU  298  OD1 ASP B 678     7859   6405   6534   1284    328    277       O
ATOM    299  OD2 ASP B 678      41.569  10.774  44.710  1.00 61.97           O1-
ANISOU  299  OD2 ASP B 678     8721   7354   7471   1282    294    334       O1-
ATOM    300  H   ASP B 678      41.526  15.401  44.371  1.00 37.79           H
ATOM    301  HA  ASP B 678      43.116  13.098  44.047  1.00 37.93           H
ATOM    302  HB3 ASP B 678      40.141  13.382  43.403  1.00 39.70           H
ATOM    303  HB2 ASP B 678      40.756  13.202  44.996  1.00 39.70           H
ATOM    304  N   VAL B 679      43.685  12.899  41.642  1.00 41.05           N
ANISOU  304  N   VAL B 679     5563   5236   4799   1473    322    380       N
ATOM    305  CA  VAL B 679      44.170  12.766  40.265  1.00 41.49           C
ANISOU  305  CA  VAL B 679     5664   5302   4799   1579    376    374       C
ATOM    306  C   VAL B 679      43.294  11.848  39.384  1.00 45.90           C
ANISOU  306  C   VAL B 679     6531   5578   5330   1554    415    299       C
ATOM    307  O   VAL B 679      43.146  12.133  38.195  1.00 45.16           O
ANISOU  307  O   VAL B 679     6464   5446   5247   1487    417    251       O
ATOM    308  CB  VAL B 679      45.650  12.271  40.261  1.00 45.86           C
ANISOU  308  CB  VAL B 679     6141   6056   5229   1872    447    481       C
ATOM    309  CG1 VAL B 679      46.193  11.739  38.918  1.00 45.87           C
ANISOU  309  CG1 VAL B 679     6238   6041   5149   2026    522    480       C
ATOM    310  CG2 VAL B 679      46.588  13.375  40.785  1.00 45.92           C
ANISOU  310  CG2 VAL B 679     5816   6383   5250   1830    393    559       C
ATOM    311  H   VAL B 679      44.299  12.540  42.359  1.00 41.05           H
ATOM    312  HA  VAL B 679      44.140  13.760  39.814  1.00 41.49           H
ATOM    313  HB  VAL B 679      45.721  11.441  40.966  1.00 45.86           H
ATOM    314 HG11 VAL B 679      47.257  11.509  38.987  1.00 45.87           H
ATOM    315 HG12 VAL B 679      45.696  10.818  38.613  1.00 45.87           H
ATOM    316 HG13 VAL B 679      46.067  12.476  38.126  1.00 45.87           H
ATOM    317 HG21 VAL B 679      47.619  13.027  40.840  1.00 45.92           H
ATOM    318 HG22 VAL B 679      46.570  14.250  40.133  1.00 45.92           H
ATOM    319 HG23 VAL B 679      46.305  13.703  41.785  1.00 45.92           H
ATOM    320  N   GLU B 680      42.708  10.793  39.975  1.00 42.48           N
ANISOU  320  N   GLU B 680     6337   4952   4851   1588    440    291       N
ATOM    321  CA  GLU B 680      41.827   9.850  39.281  1.00 42.46           C
ANISOU  321  CA  GLU B 680     6659   4678   4795   1524    468    227       C
ATOM    322  C   GLU B 680      40.435  10.430  38.967  1.00 44.55           C
ANISOU  322  C   GLU B 680     6894   4876   5158   1214    393    149       C
ATOM    323  O   GLU B 680      39.819   9.986  37.999  1.00 44.54           O
ANISOU  323  O   GLU B 680     7060   4744   5120   1131    402     97       O
ATOM    324  CB  GLU B 680      41.728   8.531  40.080  1.00 44.30           C
ANISOU  324  CB  GLU B 680     7175   4723   4934   1617    512    247       C
ATOM    325  CG  GLU B 680      43.084   7.833  40.351  1.00 58.50           C
ANISOU  325  CG  GLU B 680     9083   6552   6592   1975    611    326       C
ATOM    326  CD  GLU B 680      43.888   7.462  39.092  1.00 83.30           C
ANISOU  326  CD  GLU B 680    12321   9700   9630   2177    690    334       C
ATOM    327  OE1 GLU B 680      43.281   6.921  38.140  1.00 78.06           O
ANISOU  327  OE1 GLU B 680    11913   8825   8919   2088    707    264       O
ATOM    328  OE2 GLU B 680      45.110   7.731  39.093  1.00 76.72           O1-
ANISOU  328  OE2 GLU B 680    11301   9099   8748   2422    736    418       O1-
ATOM    329  H   GLU B 680      42.850  10.626  40.962  1.00 42.48           H
ATOM    330  HA  GLU B 680      42.279   9.627  38.314  1.00 42.46           H
```

ATOM	331	HB3	GLU	B	680	41.061	7.838	39.564	1.00	44.30	H
ATOM	332	HB2	GLU	B	680	41.249	8.729	41.039	1.00	44.30	H
ATOM	333	HG3	GLU	B	680	42.903	6.914	40.909	1.00	58.50	H
ATOM	334	HG2	GLU	B	680	43.691	8.460	41.008	1.00	58.50	H
ATOM	335	N	SER	B	681	39.985	11.432	39.742	1.00	38.53	N
ANISOU	335	N	SER	B	681	5917	4217	4505	1055	321	149	N
ATOM	336	CA	SER	B	681	38.757	12.187	39.488	1.00	37.64	C
ANISOU	336	CA	SER	B	681	5738	4088	4475	801	256	95	C
ATOM	337	C	SER	B	681	38.893	13.136	38.283	1.00	39.61	C
ANISOU	337	C	SER	B	681	5855	4434	4760	766	244	68	C
ATOM	338	O	SER	B	681	37.924	13.285	37.539	1.00	40.08	O
ANISOU	338	O	SER	B	681	5959	4434	4834	608	219	21	O
ATOM	339	CB	SER	B	681	38.336	12.958	40.753	1.00	41.18	C
ANISOU	339	CB	SER	B	681	6009	4630	5009	695	198	111	C
ATOM	340	OG	SER	B	681	37.841	12.066	41.729	1.00	49.31	O
ANISOU	340	OG	SER	B	681	7169	5564	6003	712	208	134	O
ATOM	341	H	SER	B	681	40.526	11.715	40.549	1.00	38.53	H
ATOM	342	HA	SER	B	681	37.963	11.478	39.246	1.00	37.64	H
ATOM	343	HB3	SER	B	681	37.537	13.666	40.525	1.00	41.18	H
ATOM	344	HB2	SER	B	681	39.161	13.538	41.165	1.00	41.18	H
ATOM	345	HG	SER	B	681	38.585	11.571	42.092	1.00	49.31	H
ATOM	346	N	MET	B	682	40.093	13.718	38.086	1.00	33.87	N
ANISOU	346	N	MET	B	682	4962	3872	4034	905	260	108	N
ATOM	347	CA	MET	B	682	40.446	14.534	36.917	1.00	33.12	C
ANISOU	347	CA	MET	B	682	4743	3881	3962	884	254	93	C
ATOM	348	C	MET	B	682	40.466	13.710	35.616	1.00	37.55	C
ANISOU	348	C	MET	B	682	5508	4315	4443	945	308	60	C
ATOM	349	O	MET	B	682	39.985	14.198	34.594	1.00	36.60	O
ANISOU	349	O	MET	B	682	5383	4175	4348	823	286	14	O
ATOM	350	CB	MET	B	682	41.811	15.222	37.126	1.00	34.97	C
ANISOU	350	CB	MET	B	682	4764	4337	4184	1010	262	160	C
ATOM	351	CG	MET	B	682	41.899	16.150	38.344	1.00	37.83	C
ANISOU	351	CG	MET	B	682	4934	4828	4611	908	198	186	C
ATOM	352	SD	MET	B	682	43.554	16.856	38.584	1.00	41.44	S
ANISOU	352	SD	MET	B	682	5152	5576	5019	1013	198	279	S
ATOM	353	CE	MET	B	682	43.420	17.377	40.313	1.00	38.54	C
ANISOU	353	CE	MET	B	682	4690	5275	4679	940	140	316	C
ATOM	354	H	MET	B	682	40.838	13.545	38.747	1.00	33.87	H
ATOM	355	HA	MET	B	682	39.688	15.312	36.806	1.00	33.12	H
ATOM	356	HB3	MET	B	682	42.063	15.796	36.234	1.00	34.97	H
ATOM	357	HB2	MET	B	682	42.586	14.465	37.219	1.00	34.97	H
ATOM	358	HG3	MET	B	682	41.631	15.606	39.247	1.00	37.83	H
ATOM	359	HG2	MET	B	682	41.183	16.967	38.249	1.00	37.83	H
ATOM	360	HE1	MET	B	682	44.326	17.898	40.623	1.00	38.54	H
ATOM	361	HE2	MET	B	682	42.570	18.046	40.447	1.00	38.54	H
ATOM	362	HE3	MET	B	682	43.282	16.511	40.960	1.00	38.54	H
ATOM	363	N	LYS	B	683	40.981	12.469	35.692	1.00	35.02	N
ANISOU	363	N	LYS	B	683	5390	3901	4013	1141	381	85	N
ATOM	364	CA	LYS	B	683	40.970	11.491	34.602	1.00	35.10	C
ANISOU	364	CA	LYS	B	683	5667	3755	3915	1225	443	55	C
ATOM	365	C	LYS	B	683	39.550	11.011	34.254	1.00	39.79	C
ANISOU	365	C	LYS	B	683	6473	4144	4502	990	407	-17	C
ATOM	366	O	LYS	B	683	39.239	10.894	33.071	1.00	38.85	O
ANISOU	366	O	LYS	B	683	6446	3965	4351	919	409	-62	O
ATOM	367	CB	LYS	B	683	41.860	10.289	34.970	1.00	37.53	C
ANISOU	367	CB	LYS	B	683	6183	3989	4087	1506	535	105	C

ATOM 368 CG LYS B 683 43.362 10.609 35.040 1.00 40.87 C
ANISOU 368 CG LYS B 683 6410 4649 4469 1771 588 192 C
ATOM 369 CD LYS B 683 44.161 9.408 35.561 1.00 42.86 C
ANISOU 369 CD LYS B 683 6866 4843 4573 2077 684 257 C
ATOM 370 CE LYS B 683 45.673 9.656 35.615 1.00 58.67 C
ANISOU 370 CE LYS B 683 8629 7142 6521 2344 733 367 C
ATOM 371 NZ LYS B 683 46.380 8.504 36.201 1.00 71.02 N1+
ANISOU 371 NZ LYS B 683 10352 8686 7947 2651 819 448 N1+
ATOM 372 H LYS B 683 41.370 12.151 36.569 1.00 35.02 H
ATOM 373 HA LYS B 683 41.387 11.973 33.715 1.00 35.10 H
ATOM 374 HB3 LYS B 683 41.723 9.495 34.233 1.00 37.53 H
ATOM 375 HB2 LYS B 683 41.530 9.870 35.921 1.00 37.53 H
ATOM 376 HG3 LYS B 683 43.536 11.468 35.688 1.00 40.87 H
ATOM 377 HG2 LYS B 683 43.726 10.895 34.053 1.00 40.87 H
ATOM 378 HD3 LYS B 683 43.950 8.537 34.938 1.00 42.86 H
ATOM 379 HD2 LYS B 683 43.799 9.164 36.559 1.00 42.86 H
ATOM 380 HE3 LYS B 683 45.888 10.541 36.214 1.00 58.67 H
ATOM 381 HE2 LYS B 683 46.064 9.844 34.614 1.00 58.67 H
ATOM 382 HZ1 LYS B 683 46.022 8.329 37.133 1.00 71.02 H
ATOM 383 HZ2 LYS B 683 46.220 7.683 35.634 1.00 71.02 H
ATOM 384 HZ3 LYS B 683 47.370 8.693 36.252 1.00 71.02 H
ATOM 385 N LYS B 684 38.713 10.774 35.282 1.00 37.53 N
ANISOU 385 N LYS B 684 6243 3777 4239 856 368 -20 N
ATOM 386 CA LYS B 684 37.311 10.365 35.150 1.00 37.53 C
ANISOU 386 CA LYS B 684 6402 3637 4222 599 323 -65 C
ATOM 387 C LYS B 684 36.440 11.431 34.459 1.00 41.24 C
ANISOU 387 C LYS B 684 6669 4220 4780 391 255 -97 C
ATOM 388 O LYS B 684 35.576 11.063 33.665 1.00 41.06 O
ANISOU 388 O LYS B 684 6782 4113 4705 224 234 -134 O
ATOM 389 H LYS B 684 39.056 10.872 36.229 1.00 37.53 H
ATOM 390 CB LYS B 684 36.759 9.973 36.539 1.00 0.00 C
ATOM 391 CG LYS B 684 35.317 9.422 36.530 1.00 0.00 C
ATOM 392 CD LYS B 684 34.836 8.914 37.904 1.00 0.00 C
ATOM 393 CE LYS B 684 34.606 10.001 38.974 1.00 0.00 C
ATOM 394 NZ LYS B 684 33.462 10.877 38.659 1.00 0.00 N1+
ATOM 395 HB2 LYS B 684 37.411 9.210 36.967 1.00 0.00 H
ATOM 396 HB3 LYS B 684 36.818 10.831 37.207 1.00 0.00 H
ATOM 397 HG2 LYS B 684 34.623 10.176 36.158 1.00 0.00 H
ATOM 398 HG3 LYS B 684 35.264 8.599 35.817 1.00 0.00 H
ATOM 399 HD2 LYS B 684 33.922 8.334 37.769 1.00 0.00 H
ATOM 400 HD3 LYS B 684 35.569 8.203 38.288 1.00 0.00 H
ATOM 401 HE2 LYS B 684 34.400 9.525 39.933 1.00 0.00 H
ATOM 402 HE3 LYS B 684 35.499 10.610 39.118 1.00 0.00 H
ATOM 403 HZ1 LYS B 684 33.645 11.392 37.810 1.00 0.00 H
ATOM 404 HZ2 LYS B 684 32.630 10.316 38.541 1.00 0.00 H
ATOM 405 HZ3 LYS B 684 33.318 11.522 39.426 1.00 0.00 H
ATOM 406 HA LYS B 684 37.297 9.473 34.521 1.00 37.53 H
ATOM 407 N ALA B 685 36.706 12.718 34.745 1.00 36.72 N
ANISOU 407 N ALA B 685 5794 3837 4323 402 222 -77 N
ATOM 408 CA ALA B 685 36.052 13.863 34.111 1.00 36.69 C
ANISOU 408 CA ALA B 685 5607 3943 4392 252 169 -98 C
ATOM 409 C ALA B 685 36.381 13.993 32.613 1.00 41.99 C
ANISOU 409 C ALA B 685 6318 4610 5026 278 190 -129 C
ATOM 410 O ALA B 685 35.474 14.282 31.835 1.00 41.51 O
ANISOU 410 O ALA B 685 6258 4551 4963 120 156 -157 O
ATOM 411 CB ALA B 685 36.414 15.147 34.873 1.00 37.24 C

ANISOU 411 CB ALA B 685 5410 4179 4561 285 141 -69 C
ATOM 412 H ALA B 685 37.423 12.936 35.425 1.00 36.72 H
ATOM 413 HA ALA B 685 34.973 13.717 34.200 1.00 36.69 H
ATOM 414 HB1 ALA B 685 35.955 16.023 34.414 1.00 37.24 H
ATOM 415 HB2 ALA B 685 36.061 15.098 35.903 1.00 37.24 H
ATOM 416 HB3 ALA B 685 37.492 15.311 34.899 1.00 37.24 H
ATOM 417 N MET B 686 37.645 13.733 32.232 1.00 39.73 N
ANISOU 417 N MET B 686 6065 4332 4698 483 250 -115 N
ATOM 418 CA MET B 686 38.105 13.709 30.839 1.00 41.00 C
ANISOU 418 CA MET B 686 6274 4494 4812 543 284 -137 C
ATOM 419 C MET B 686 37.515 12.535 30.034 1.00 46.10 C
ANISOU 419 C MET B 686 7233 4942 5340 479 305 -182 C
ATOM 420 O MET B 686 37.103 12.748 28.895 1.00 45.45 O
ANISOU 420 O MET B 686 7176 4855 5238 393 294 -217 O
ATOM 421 CB MET B 686 39.645 13.701 30.792 1.00 43.62 C
ANISOU 421 CB MET B 686 6532 4929 5114 794 347 -91 C
ATOM 422 CG MET B 686 40.289 15.021 31.246 1.00 47.91 C
ANISOU 422 CG MET B 686 6765 5689 5751 785 313 -57 C
ATOM 423 SD MET B 686 42.090 14.954 31.455 1.00 53.37 S
ANISOU 423 SD MET B 686 7321 6564 6392 1043 372 28 S
ATOM 424 CE MET B 686 42.622 14.682 29.744 1.00 50.07 C
ANISOU 424 CE MET B 686 6995 6153 5878 1187 444 22 C
ATOM 425 H MET B 686 38.337 13.503 32.933 1.00 39.73 H
ATOM 426 HA MET B 686 37.761 14.627 30.358 1.00 41.00 H
ATOM 427 HB3 MET B 686 39.967 13.501 29.769 1.00 43.62 H
ATOM 428 HB2 MET B 686 40.036 12.881 31.396 1.00 43.62 H
ATOM 429 HG3 MET B 686 39.864 15.340 32.197 1.00 47.91 H
ATOM 430 HG2 MET B 686 40.055 15.811 30.532 1.00 47.91 H
ATOM 431 HE1 MET B 686 43.710 14.707 29.681 1.00 50.07 H
ATOM 432 HE2 MET B 686 42.282 13.714 29.378 1.00 50.07 H
ATOM 433 HE3 MET B 686 42.217 15.462 29.100 1.00 50.07 H
ATOM 434 N VAL B 687 37.434 11.341 30.650 1.00 43.48 N
ANISOU 434 N VAL B 687 7157 4442 4921 503 333 -180 N
ATOM 435 CA VAL B 687 36.780 10.149 30.092 1.00 43.89 C
ANISOU 435 CA VAL B 687 7573 4271 4832 407 348 -220 C
ATOM 436 C VAL B 687 35.258 10.333 29.897 1.00 48.20 C
ANISOU 436 C VAL B 687 8090 4827 5396 78 261 -249 C
ATOM 437 O VAL B 687 34.710 9.797 28.934 1.00 48.17 O
ANISOU 437 O VAL B 687 8280 4727 5296 -53 252 -287 O
ATOM 438 CB VAL B 687 37.059 8.895 30.981 1.00 47.73 C
ANISOU 438 CB VAL B 687 8345 4576 5215 508 397 -201 C
ATOM 439 CG1 VAL B 687 36.136 7.675 30.761 1.00 47.69 C
ANISOU 439 CG1 VAL B 687 8725 4332 5064 306 386 -240 C
ATOM 440 CG2 VAL B 687 38.522 8.441 30.833 1.00 47.51 C
ANISOU 440 CG2 VAL B 687 8412 4529 5111 860 500 -167 C
ATOM 441 H VAL B 687 37.814 11.240 31.582 1.00 43.48 H
ATOM 442 HA VAL B 687 37.211 9.974 29.104 1.00 43.89 H
ATOM 443 HB VAL B 687 36.930 9.194 32.022 1.00 47.73 H
ATOM 444 HG11 VAL B 687 36.471 6.820 31.349 1.00 47.69 H
ATOM 445 HG12 VAL B 687 35.108 7.875 31.065 1.00 47.69 H
ATOM 446 HG13 VAL B 687 36.128 7.370 29.713 1.00 47.69 H
ATOM 447 HG21 VAL B 687 38.751 7.612 31.505 1.00 47.51 H
ATOM 448 HG22 VAL B 687 38.733 8.112 29.815 1.00 47.51 H
ATOM 449 HG23 VAL B 687 39.217 9.244 31.067 1.00 47.51 H
ATOM 450 N GLU B 688 34.616 11.117 30.780 1.00 45.40 N
ANISOU 450 N GLU B 688 7485 4612 5153 -44 200 -223 N

```
ATOM    451  CA  GLU B 688      33.203  11.492  30.706  1.00 45.31           C
ANISOU  451  CA  GLU B 688     7371   4682   5161   -318    122   -224       C
ATOM    452  C   GLU B 688      32.891  12.498  29.574  1.00 46.42           C
ANISOU  452  C   GLU B 688     7354   4944   5338   -362     98   -243       C
ATOM    453  O   GLU B 688      31.779  12.470  29.047  1.00 45.79           O
ANISOU  453  O   GLU B 688     7293   4896   5209   -575     48   -251       O
ATOM    454  CB  GLU B 688      32.752  11.939  32.114  1.00 47.03           C
ANISOU  454  CB  GLU B 688     7351   5034   5483   -355     83   -181       C
ATOM    455  CG  GLU B 688      31.293  12.437  32.237  1.00 61.04           C
ANISOU  455  CG  GLU B 688     9175   6811   7208   -602     30   -159       C
ATOM    456  CD  GLU B 688      30.825  12.743  33.671  1.00 93.71           C
ANISOU  456  CD  GLU B 688    13108  11066  11432   -600      7   -113       C
ATOM    457  OE1 GLU B 688      29.696  13.269  33.782  1.00 94.99           O
ANISOU  457  OE1 GLU B 688    13056  11398  11636   -706    -37    -85       O
ATOM    458  OE2 GLU B 688      31.582  12.492  34.638  1.00 92.40           O1-
ANISOU  458  OE2 GLU B 688    12999  10828  11282   -475     39   -101       O1-
ATOM    459  H   GLU B 688      35.131  11.499  31.562  1.00 45.40           H
ATOM    460  HA  GLU B 688      32.641  10.587  30.468  1.00 45.31           H
ATOM    461  HB3 GLU B 688      33.426  12.712  32.483  1.00 47.03           H
ATOM    462  HB2 GLU B 688      32.885  11.086  32.781  1.00 47.03           H
ATOM    463  HG3 GLU B 688      30.619  11.691  31.814  1.00 61.04           H
ATOM    464  HG2 GLU B 688      31.163  13.343  31.645  1.00 61.04           H
ATOM    465  N   TYR B 689      33.890  13.304  29.167  1.00 41.16           N
ANISOU  465  N   TYR B 689     6536   4360   4744   -168    132   -243       N
ATOM    466  CA  TYR B 689      33.866  14.113  27.941  1.00 40.73           C
ANISOU  466  CA  TYR B 689     6345   4409   4721   -177    121   -258       C
ATOM    467  C   TYR B 689      34.271  13.321  26.676  1.00 45.86           C
ANISOU  467  C   TYR B 689     7224   4940   5261   -131    164   -298       C
ATOM    468  O   TYR B 689      34.249  13.896  25.588  1.00 46.96           O
ANISOU  468  O   TYR B 689     7284   5149   5411   -128    162   -314       O
ATOM    469  CB  TYR B 689      34.770  15.356  28.130  1.00 41.10           C
ANISOU  469  CB  TYR B 689     6143   4596   4877    -19    135   -233       C
ATOM    470  CG  TYR B 689      34.236  16.411  29.085  1.00 42.23           C
ANISOU  470  CG  TYR B 689     6066   4863   5115    -92     86   -203       C
ATOM    471  CD1 TYR B 689      32.968  16.989  28.857  1.00 43.61           C
ANISOU  471  CD1 TYR B 689     6154   5116   5300   -255     35   -199       C
ATOM    472  CD2 TYR B 689      35.016  16.858  30.173  1.00 42.65           C
ANISOU  472  CD2 TYR B 689     5998   4974   5235     17     95   -172       C
ATOM    473  CE1 TYR B 689      32.475  17.986  29.718  1.00 43.58           C
ANISOU  473  CE1 TYR B 689     5970   5225   5363   -283      3   -165       C
ATOM    474  CE2 TYR B 689      34.520  17.851  31.041  1.00 43.45           C
ANISOU  474  CE2 TYR B 689     5934   5170   5406    -39     55   -147       C
ATOM    475  CZ  TYR B 689      33.248  18.413  30.814  1.00 49.36           C
ANISOU  475  CZ  TYR B 689     6619   5978   6159   -175     15   -144       C
ATOM    476  OH  TYR B 689      32.763  19.376  31.647  1.00 51.62           O
ANISOU  476  OH  TYR B 689     6766   6354   6492   -195    -11   -114       O
ATOM    477  H   TYR B 689      34.770  13.284  29.663  1.00 41.16           H
ATOM    478  HA  TYR B 689      32.843  14.448  27.766  1.00 40.73           H
ATOM    479  HB3 TYR B 689      34.902  15.865  27.176  1.00 41.10           H
ATOM    480  HB2 TYR B 689      35.771  15.050  28.437  1.00 41.10           H
ATOM    481  HD1 TYR B 689      32.370  16.678  28.012  1.00 43.61           H
ATOM    482  HD2 TYR B 689      35.998  16.443  30.346  1.00 42.65           H
ATOM    483  HE1 TYR B 689      31.504  18.421  29.532  1.00 43.58           H
ATOM    484  HE2 TYR B 689      35.118  18.184  31.876  1.00 43.45           H
ATOM    485  HH  TYR B 689      31.938  19.757  31.339  1.00 51.62           H
ATOM    486  N   GLU B 690      34.630  12.033  26.837  1.00 41.94           N
```

```
ANISOU  486  N   GLU B 690     7035   4252   4648    -87    207   -313        N
ATOM    487  CA  GLU B 690      35.100  11.097  25.808  1.00 41.99           C
ANISOU  487  CA  GLU B 690     7366   4082   4507    -14    264   -349        C
ATOM    488  C   GLU B 690      36.407  11.534  25.118  1.00 45.90           C
ANISOU  488  C   GLU B 690     7801   4633   5007    257    339   -344        C
ATOM    489  O   GLU B 690      36.618  11.223  23.948  1.00 45.62           O
ANISOU  489  O   GLU B 690     7944   4519   4872    299    376   -376        O
ATOM    490  CB  GLU B 690      33.971  10.688  24.829  1.00 43.31           C
ANISOU  490  CB  GLU B 690     7704   4179   4573   -278    213   -389        C
ATOM    491  CG  GLU B 690      32.775  10.018  25.540  1.00 54.60           C
ANISOU  491  CG  GLU B 690     9405   5452   5889   -483    183   -393        C
ATOM    492  CD  GLU B 690      31.807   9.335  24.565  1.00 74.55           C
ANISOU  492  CD  GLU B 690    12116   7925   8286   -797    120   -420        C
ATOM    493  OE1 GLU B 690      30.700   9.883  24.365  1.00 75.31           O
ANISOU  493  OE1 GLU B 690    11967   8211   8438  -1006     40   -397        O
ATOM    494  OE2 GLU B 690      32.174   8.260  24.039  1.00 58.52           O1-
ANISOU  494  OE2 GLU B 690    10488   5668   6081   -836    150   -457        O1-
ATOM    495  H   GLU B 690      34.591  11.642  27.768  1.00 41.94           H
ATOM    496  HA  GLU B 690      35.382  10.195  26.350  1.00 41.99           H
ATOM    497  HB3 GLU B 690      34.370  10.007  24.076  1.00 43.31           H
ATOM    498  HB2 GLU B 690      33.618  11.560  24.277  1.00 43.31           H
ATOM    499  HG3 GLU B 690      32.241  10.753  26.143  1.00 54.60           H
ATOM    500  HG2 GLU B 690      33.138   9.264  26.241  1.00 54.60           H
ATOM    501  N   ILE B 691      37.277  12.221  25.878  1.00 41.92           N
ANISOU  501  N   ILE B 691     7063   4267   4598    439    363   -296        N
ATOM    502  CA  ILE B 691      38.647  12.559  25.493  1.00 41.75           C
ANISOU  502  CA  ILE B 691     6929   4356   4576    693    431   -265        C
ATOM    503  C   ILE B 691      39.529  11.300  25.614  1.00 47.50           C
ANISOU  503  C   ILE B 691     7944   4944   5159    943    530   -249        C
ATOM    504  O   ILE B 691      39.360  10.531  26.561  1.00 47.43           O
ANISOU  504  O   ILE B 691     8097   4817   5109    967    542   -238        O
ATOM    505  CB  ILE B 691      39.212  13.696  26.397  1.00 44.25           C
ANISOU  505  CB  ILE B 691     6891   4892   5030    738    403   -211        C
ATOM    506  CG1 ILE B 691      38.385  14.994  26.215  1.00 44.05           C
ANISOU  506  CG1 ILE B 691     6634   4983   5118    528    322   -227        C
ATOM    507  CG2 ILE B 691      40.714  13.992  26.194  1.00 44.61           C
ANISOU  507  CG2 ILE B 691     6810   5089   5051    994    472   -154        C
ATOM    508  CD1 ILE B 691      38.660  16.073  27.272  1.00 48.72           C
ANISOU  508  CD1 ILE B 691     6973   5712   5826    488    276   -189        C
ATOM    509  H   ILE B 691      37.029  12.434  26.835  1.00 41.92           H
ATOM    510  HA  ILE B 691      38.646  12.914  24.463  1.00 41.75           H
ATOM    511  HB  ILE B 691      39.103  13.375  27.432  1.00 44.25           H
ATOM    512 HG13 ILE B 691      37.318  14.776  26.240  1.00 44.05           H
ATOM    513 HG12 ILE B 691      38.573  15.406  25.222  1.00 44.05           H
ATOM    514 HG21 ILE B 691      41.035  14.801  26.848  1.00 44.61           H
ATOM    515 HG22 ILE B 691      41.350  13.141  26.436  1.00 44.61           H
ATOM    516 HG23 ILE B 691      40.927  14.291  25.168  1.00 44.61           H
ATOM    517 HD11 ILE B 691      37.801  16.737  27.381  1.00 48.72           H
ATOM    518 HD12 ILE B 691      38.865  15.636  28.250  1.00 48.72           H
ATOM    519 HD13 ILE B 691      39.515  16.687  26.991  1.00 48.72           H
ATOM    520  N   ASP B 692      40.440  11.112  24.648  1.00 45.27           N
ANISOU  520  N   ASP B 692     7743   4672   4785   1140    606   -244        N
ATOM    521  CA  ASP B 692      41.346   9.968  24.571  1.00 46.10           C
ANISOU  521  CA  ASP B 692     8134   4655   4725   1431    717   -219        C
ATOM    522  C   ASP B 692      42.573  10.243  25.461  1.00 52.30           C
ANISOU  522  C   ASP B 692     8699   5636   5539   1691    766   -127        C
```

```
ATOM    523  O   ASP B 692      43.425  11.049  25.086  1.00 53.31           O
ANISOU  523  O   ASP B 692     8565   5995   5695   1822    791    -75       O
ATOM    524  CB  ASP B 692      41.722   9.680  23.094  1.00 47.98           C
ANISOU  524  CB  ASP B 692     8524   4861   4846   1560    786   -240       C
ATOM    525  CG  ASP B 692      42.356   8.310  22.786  1.00 58.23           C
ANISOU  525  CG  ASP B 692    10244   5952   5929   1828    904   -236       C
ATOM    526  OD1 ASP B 692      42.870   7.648  23.713  1.00 60.05           O
ANISOU  526  OD1 ASP B 692    10531   6182   6103   2067    969   -175       O
ATOM    527  OD2 ASP B 692      42.380   7.974  21.583  1.00 62.35           O1-
ANISOU  527  OD2 ASP B 692    11055   6311   6324   1811    934   -290       O1-
ATOM    528  H   ASP B 692      40.540  11.802  23.913  1.00 45.27           H
ATOM    529  HA  ASP B 692      40.820   9.091  24.956  1.00 46.10           H
ATOM    530  HB3 ASP B 692      42.384  10.462  22.718  1.00 47.98           H
ATOM    531  HB2 ASP B 692      40.825   9.776  22.486  1.00 47.98           H
ATOM    532  N   LEU B 693      42.634   9.560  26.618  1.00 48.72           N
ANISOU  532  N   LEU B 693     8347   5102   5063   1754    778   -100       N
ATOM    533  CA  LEU B 693      43.729   9.666  27.588  1.00 48.84           C
ANISOU  533  CA  LEU B 693     8157   5308   5091   1986    817     -5       C
ATOM    534  C   LEU B 693      45.001   8.893  27.184  1.00 53.21           C
ANISOU  534  C   LEU B 693     8826   5917   5476   2385    949     68       C
ATOM    535  O   LEU B 693      46.037   9.133  27.801  1.00 52.17           O
ANISOU  535  O   LEU B 693     8440   6037   5343   2587    982    167       O
ATOM    536  CB  LEU B 693      43.250   9.264  29.006  1.00 49.04           C
ANISOU  536  CB  LEU B 693     8247   5236   5149   1917    783      2       C
ATOM    537  CG  LEU B 693      42.508  10.388  29.768  1.00 53.82           C
ANISOU  537  CG  LEU B 693     8523   5985   5941   1664    671     -1       C
ATOM    538  CD1 LEU B 693      41.111  10.680  29.191  1.00 54.27           C
ANISOU  538  CD1 LEU B 693     8649   5905   6065   1328    585    -85       C
ATOM    539  CD2 LEU B 693      42.449  10.103  31.283  1.00 56.55           C
ANISOU  539  CD2 LEU B 693     8834   6349   6304   1728    667     49       C
ATOM    540  H   LEU B 693      41.887   8.922  26.854  1.00 48.72           H
ATOM    541  HA  LEU B 693      44.031  10.712  27.632  1.00 48.84           H
ATOM    542  HB3 LEU B 693      44.128   9.008  29.600  1.00 49.04           H
ATOM    543  HB2 LEU B 693      42.650   8.353  28.971  1.00 49.04           H
ATOM    544  HG  LEU B 693      43.098  11.300  29.660  1.00 53.82           H
ATOM    545 HD11 LEU B 693      40.394  10.971  29.956  1.00 54.27           H
ATOM    546 HD12 LEU B 693      41.154  11.498  28.472  1.00 54.27           H
ATOM    547 HD13 LEU B 693      40.700   9.808  28.683  1.00 54.27           H
ATOM    548 HD21 LEU B 693      42.851  10.947  31.844  1.00 56.55           H
ATOM    549 HD22 LEU B 693      41.433   9.937  31.639  1.00 56.55           H
ATOM    550 HD23 LEU B 693      43.025   9.223  31.566  1.00 56.55           H
ATOM    551  N   GLN B 694      44.938   8.015  26.166  1.00 50.65           N
ANISOU  551  N   GLN B 694     8878   5376   4990   2499   1027     27       N
ATOM    552  CA  GLN B 694      46.119   7.349  25.608  1.00 50.88           C
ANISOU  552  CA  GLN B 694     9051   5449   4833   2908   1167     97       C
ATOM    553  C   GLN B 694      46.905   8.308  24.692  1.00 55.62           C
ANISOU  553  C   GLN B 694     9333   6340   5462   2984   1184    140       C
ATOM    554  O   GLN B 694      48.134   8.324  24.762  1.00 56.45           O
ANISOU  554  O   GLN B 694     9309   6670   5469   3313   1276    248       O
ATOM    555  H   GLN B 694      44.063   7.858  25.681  1.00 50.65           H
ATOM    556  CB  GLN B 694      45.693   6.034  24.910  1.00  0.00           C
ATOM    557  CG  GLN B 694      46.839   5.079  24.501  1.00  0.00           C
ATOM    558  CD  GLN B 694      47.508   5.397  23.158  1.00  0.00           C
ATOM    559  OE1 GLN B 694      46.874   5.894  22.230  1.00  0.00           O
ATOM    560  NE2 GLN B 694      48.800   5.082  23.041  1.00  0.00           N
ATOM    561  HB2 GLN B 694      45.056   5.485  25.606  1.00  0.00           H
```

```
ATOM    562  HB3 GLN B 694      45.057   6.241  24.050  1.00  0.00           H
ATOM    563  HG2 GLN B 694      47.586   5.033  25.294  1.00  0.00           H
ATOM    564  HG3 GLN B 694      46.434   4.071  24.409  1.00  0.00           H
ATOM    565 HE22 GLN B 694      49.285   5.264  22.175  1.00  0.00           H
ATOM    566 HE21 GLN B 694      49.299   4.671  23.815  1.00  0.00           H
ATOM    567  HA  GLN B 694      46.778   7.073  26.435  1.00 50.88           H
ATOM    568  N   LYS B 695      46.185   9.102  23.879  1.00 51.72           N
ANISOU  568  N   LYS B 695     8701   5860   5092   2681   1094     68       N
ATOM    569  CA  LYS B 695      46.759  10.090  22.961  1.00 51.68           C
ANISOU  569  CA  LYS B 695     8404   6107   5126   2690   1093     98       C
ATOM    570  C   LYS B 695      47.084  11.430  23.645  1.00 56.69           C
ANISOU  570  C   LYS B 695     8568   7040   5931   2535   1004    152       C
ATOM    571  O   LYS B 695      48.084  12.049  23.283  1.00 56.46           O
ANISOU  571  O   LYS B 695     8257   7307   5890   2656   1032    238       O
ATOM    572  CB  LYS B 695      45.803  10.308  21.773  1.00 53.90           C
ANISOU  572  CB  LYS B 695     8825   6228   5427   2458   1049     -7       C
ATOM    573  CG  LYS B 695      45.675   9.069  20.874  1.00 61.39           C
ANISOU  573  CG  LYS B 695    10222   6932   6174   2632   1148    -49       C
ATOM    574  CD  LYS B 695      44.711   9.292  19.701  1.00 72.25           C
ANISOU  574  CD  LYS B 695    11761   8124   7566   2339   1082   -160       C
ATOM    575  CE  LYS B 695      44.431   7.992  18.927  1.00 86.26           C
ANISOU  575  CE  LYS B 695    14023   9635   9119   2489   1176   -206       C
ATOM    576  NZ  LYS B 695      43.271   8.133  18.032  1.00 95.77           N1+
ANISOU  576  NZ  LYS B 695    15418  10648  10322   2162   1098   -313       N1+
ATOM    577  H   LYS B 695      45.177   9.020  23.876  1.00 51.72           H
ATOM    578  HA  LYS B 695      47.696   9.693  22.567  1.00 51.68           H
ATOM    579  HB3 LYS B 695      46.162  11.137  21.161  1.00 53.90           H
ATOM    580  HB2 LYS B 695      44.820  10.606  22.142  1.00 53.90           H
ATOM    581  HG3 LYS B 695      45.325   8.225  21.470  1.00 61.39           H
ATOM    582  HG2 LYS B 695      46.656   8.784  20.493  1.00 61.39           H
ATOM    583  HD3 LYS B 695      45.138  10.033  19.024  1.00 72.25           H
ATOM    584  HD2 LYS B 695      43.784   9.730  20.074  1.00 72.25           H
ATOM    585  HE3 LYS B 695      44.215   7.177  19.618  1.00 86.26           H
ATOM    586  HE2 LYS B 695      45.308   7.695  18.351  1.00 86.26           H
ATOM    587  HZ1 LYS B 695      43.440   8.870  17.363  1.00 95.77           H
ATOM    588  HZ2 LYS B 695      43.110   7.262  17.547  1.00 95.77           H
ATOM    589  HZ3 LYS B 695      42.457   8.360  18.588  1.00 95.77           H
ATOM    590  N   MET B 696      46.254  11.844  24.615  1.00 53.82           N
ANISOU  590  N   MET B 696     8134   6604   5710   2264    899    105       N
ATOM    591  CA  MET B 696      46.436  13.066  25.394  1.00 53.75           C
ANISOU  591  CA  MET B 696     7743   6833   5846   2107    814    148       C
ATOM    592  C   MET B 696      46.053  12.791  26.860  1.00 56.90           C
ANISOU  592  C   MET B 696     8120   7199   6300   2064    774    166       C
ATOM    593  O   MET B 696      44.930  13.112  27.253  1.00 57.21           O
ANISOU  593  O   MET B 696     8173   7114   6451   1811    688    101       O
ATOM    594  CB  MET B 696      45.639  14.234  24.772  1.00 56.05           C
ANISOU  594  CB  MET B 696     7888   7138   6272   1800    719     83       C
ATOM    595  CG  MET B 696      45.954  15.606  25.403  1.00 59.84           C
ANISOU  595  CG  MET B 696     7995   7896   6844   1701    660    146       C
ATOM    596  SD  MET B 696      44.596  16.802  25.327  1.00 64.15           S
ANISOU  596  SD  MET B 696     8423   8389   7561   1333    533     73       S
ATOM    597  CE  MET B 696      43.591  16.195  26.700  1.00 61.14           C
ANISOU  597  CE  MET B 696     8126   7871   7233   1266    492     54       C
ATOM    598  H   MET B 696      45.436  11.294  24.843  1.00 53.82           H
ATOM    599  HA  MET B 696      47.479  13.379  25.360  1.00 53.75           H
ATOM    600  HB3 MET B 696      44.569  14.027  24.806  1.00 56.05           H
```

```
ATOM    601  HB2 MET B 696      45.893  14.297  23.714  1.00 56.05           H
ATOM    602  HG3 MET B 696      46.816  16.043  24.897  1.00 59.84           H
ATOM    603  HG2 MET B 696      46.247  15.513  26.448  1.00 59.84           H
ATOM    604  HE1 MET B 696      42.707  16.818  26.832  1.00 61.14           H
ATOM    605  HE2 MET B 696      43.275  15.171  26.507  1.00 61.14           H
ATOM    606  HE3 MET B 696      44.168  16.204  27.622  1.00 61.14           H
ATOM    607  N   PRO B 697      46.986  12.225  27.662  1.00 52.64           N
ANISOU  607  N   PRO B 697     7520   6805   5677   2321    836    265       N
ATOM    608  CA  PRO B 697      46.802  12.123  29.122  1.00 51.99           C
ANISOU  608  CA  PRO B 697     7395   6717   5642   2292    798    291       C
ATOM    609  C   PRO B 697      46.769  13.490  29.835  1.00 53.87           C
ANISOU  609  C   PRO B 697     7285   7159   6024   2059    693    312       C
ATOM    610  O   PRO B 697      47.015  14.530  29.222  1.00 54.10           O
ANISOU  610  O   PRO B 697     7096   7365   6093   1961    661    326       O
ATOM    611  CB  PRO B 697      47.989  11.254  29.577  1.00 53.90           C
ANISOU  611  CB  PRO B 697     7656   7095   5728   2664    905    406       C
ATOM    612  CG  PRO B 697      49.066  11.494  28.535  1.00 58.66           C
ANISOU  612  CG  PRO B 697     8126   7922   6241   2843    971    472       C
ATOM    613  CD  PRO B 697      48.270  11.649  27.246  1.00 54.37           C
ANISOU  613  CD  PRO B 697     7692   7223   5741   2664    948    369       C
ATOM    614  HA  PRO B 697      45.867  11.605  29.342  1.00 51.99           H
ATOM    615  HB3 PRO B 697      47.696  10.203  29.564  1.00 53.90           H
ATOM    616  HB2 PRO B 697      48.342  11.472  30.586  1.00 53.90           H
ATOM    617  HG3 PRO B 697      49.815  10.703  28.490  1.00 58.66           H
ATOM    618  HG2 PRO B 697      49.575  12.429  28.766  1.00 58.66           H
ATOM    619  HD2 PRO B 697      48.829  12.248  26.531  1.00 54.37           H
ATOM    620  HD3 PRO B 697      48.082  10.675  26.792  1.00 54.37           H
ATOM    621  N   LEU B 698      46.462  13.440  31.140  1.00 48.23           N
ANISOU  621  N   LEU B 698     6545   6406   5374   1969    641    313       N
ATOM    622  CA  LEU B 698      46.327  14.576  32.058  1.00 47.20           C
ANISOU  622  CA  LEU B 698     6148   6430   5358   1761    545    331       C
ATOM    623  C   LEU B 698      47.554  15.519  32.123  1.00 49.66           C
ANISOU  623  C   LEU B 698     6143   7086   5638   1796    537    430       C
ATOM    624  O   LEU B 698      47.368  16.712  32.364  1.00 49.15           O
ANISOU  624  O   LEU B 698     5906   7114   5653   1573    463    416       O
ATOM    625  CB  LEU B 698      45.955  13.977  33.435  1.00 46.87           C
ANISOU  625  CB  LEU B 698     6148   6340   5321   1798    533    356       C
ATOM    626  CG  LEU B 698      45.631  14.956  34.582  1.00 50.80           C
ANISOU  626  CG  LEU B 698     6599   6759   5943   1547    439    307       C
ATOM    627  CD1 LEU B 698      44.469  15.909  34.260  1.00 50.62           C
ANISOU  627  CD1 LEU B 698     6631   6584   6017   1302    382    209       C
ATOM    628  CD2 LEU B 698      45.339  14.167  35.869  1.00 52.72           C
ANISOU  628  CD2 LEU B 698     6982   6886   6162   1628    454    320       C
ATOM    629  H   LEU B 698      46.272  12.538  31.550  1.00 48.23           H
ATOM    630  HA  LEU B 698      45.485  15.167  31.695  1.00 47.20           H
ATOM    631  HB3 LEU B 698      46.770  13.325  33.754  1.00 46.87           H
ATOM    632  HB2 LEU B 698      45.090  13.323  33.305  1.00 46.87           H
ATOM    633  HG  LEU B 698      46.517  15.562  34.771  1.00 50.80           H
ATOM    634 HD11 LEU B 698      44.223  16.522  35.128  1.00 50.62           H
ATOM    635 HD12 LEU B 698      44.720  16.592  33.448  1.00 50.62           H
ATOM    636 HD13 LEU B 698      43.571  15.360  33.978  1.00 50.62           H
ATOM    637 HD21 LEU B 698      45.531  14.767  36.758  1.00 52.72           H
ATOM    638 HD22 LEU B 698      44.299  13.846  35.905  1.00 52.72           H
ATOM    639 HD23 LEU B 698      45.953  13.270  35.938  1.00 52.72           H
ATOM    640  N   GLY B 699      48.766  14.988  31.874  1.00 45.27           N
ANISOU  640  N   GLY B 699     5528   6723   4951   2073    617    537       N
```

ATOM	641	CA	GLY	B	699	50.015	15.754	31.865	1.00	44.75	C
ANISOU	641	CA	GLY	B	699	5150	7036	4818	2122	617	659	C
ATOM	642	C	GLY	B	699	50.358	16.342	30.484	1.00	48.07	C
ANISOU	642	C	GLY	B	699	5468	7570	5228	2060	623	658	C
ATOM	643	O	GLY	B	699	51.246	17.190	30.412	1.00	49.05	O
ANISOU	643	O	GLY	B	699	5315	7997	5324	1976	589	744	O
ATOM	644	H	GLY	B	699	48.833	14.002	31.670	1.00	45.27	H
ATOM	645	HA3	GLY	B	699	50.828	15.099	32.178	1.00	44.75	H
ATOM	646	HA2	GLY	B	699	49.972	16.568	32.589	1.00	44.75	H
ATOM	647	N	LYS	B	700	49.676	15.917	29.404	1.00	42.98	N
ANISOU	647	N	LYS	B	700	5051	6688	4590	2088	665	567	N
ATOM	648	CA	LYS	B	700	49.811	16.468	28.049	1.00	41.99	C
ANISOU	648	CA	LYS	B	700	4871	6628	4454	2040	677	553	C
ATOM	649	C	LYS	B	700	48.765	17.556	27.738	1.00	43.03	C
ANISOU	649	C	LYS	B	700	4985	6634	4731	1700	578	453	C
ATOM	650	O	LYS	B	700	48.913	18.245	26.729	1.00	41.65	O
ANISOU	650	O	LYS	B	700	4741	6529	4555	1628	575	446	O
ATOM	651	CB	LYS	B	700	49.739	15.321	27.016	1.00	45.11	C
ANISOU	651	CB	LYS	B	700	5530	6865	4745	2287	787	524	C
ATOM	652	CG	LYS	B	700	50.967	14.388	26.993	1.00	62.20	C
ANISOU	652	CG	LYS	B	700	7688	9218	6727	2677	907	648	C
ATOM	653	CD	LYS	B	700	52.279	15.049	26.525	1.00	75.14	C
ANISOU	653	CD	LYS	B	700	9012	11262	8274	2768	938	778	C
ATOM	654	CE	LYS	B	700	53.252	15.372	27.674	1.00	89.09	C
ANISOU	654	CE	LYS	B	700	10497	13384	9969	2868	935	931	C
ATOM	655	NZ	LYS	B	700	54.481	16.020	27.184	1.00	98.31	N1+
ANISOU	655	NZ	LYS	B	700	11350	14988	11016	2960	970	1078	N1+
ATOM	656	H	LYS	B	700	48.945	15.228	29.523	1.00	42.98	H
ATOM	657	HA	LYS	B	700	50.778	16.961	27.945	1.00	41.99	H
ATOM	658	HB3	LYS	B	700	49.623	15.732	26.012	1.00	45.11	H
ATOM	659	HB2	LYS	B	700	48.838	14.733	27.192	1.00	45.11	H
ATOM	660	HG3	LYS	B	700	50.748	13.559	26.320	1.00	62.20	H
ATOM	661	HG2	LYS	B	700	51.109	13.935	27.974	1.00	62.20	H
ATOM	662	HD3	LYS	B	700	52.049	15.963	25.974	1.00	75.14	H
ATOM	663	HD2	LYS	B	700	52.767	14.388	25.807	1.00	75.14	H
ATOM	664	HE3	LYS	B	700	53.529	14.456	28.199	1.00	89.09	H
ATOM	665	HE2	LYS	B	700	52.787	16.028	28.408	1.00	89.09	H
ATOM	666	HZ1	LYS	B	700	54.245	16.888	26.724	1.00	98.31	H
ATOM	667	HZ2	LYS	B	700	55.095	16.211	27.962	1.00	98.31	H
ATOM	668	HZ3	LYS	B	700	54.949	15.410	26.529	1.00	98.31	H
ATOM	669	N	LEU	B	701	47.749	17.707	28.606	1.00	38.60	N
ANISOU	669	N	LEU	B	701	4483	5902	4280	1512	502	385	N
ATOM	670	CA	LEU	B	701	46.711	18.734	28.532	1.00	37.82	C
ANISOU	670	CA	LEU	B	701	4374	5692	4304	1225	414	304	C
ATOM	671	C	LEU	B	701	47.311	20.100	28.922	1.00	39.97	C
ANISOU	671	C	LEU	B	701	4391	6213	4584	1070	355	370	C
ATOM	672	O	LEU	B	701	47.774	20.245	30.053	1.00	39.45	O
ANISOU	672	O	LEU	B	701	4177	6329	4483	1077	334	450	O
ATOM	673	CB	LEU	B	701	45.553	18.280	29.457	1.00	37.82	C
ANISOU	673	CB	LEU	B	701	4486	5494	4388	1115	364	243	C
ATOM	674	CG	LEU	B	701	44.173	18.995	29.361	1.00	42.19	C
ANISOU	674	CG	LEU	B	701	5159	5833	5041	913	310	141	C
ATOM	675	CD1	LEU	B	701	43.758	19.683	30.676	1.00	42.06	C
ANISOU	675	CD1	LEU	B	701	5092	5784	5104	754	237	128	C
ATOM	676	CD2	LEU	B	701	43.974	19.923	28.147	1.00	43.78	C
ANISOU	676	CD2	LEU	B	701	5341	6033	5258	813	299	103	C

```
ATOM    677  H   LEU B 701      47.706  17.103  29.414  1.00 38.60           H
ATOM    678  HA  LEU B 701      46.365  18.762  27.498  1.00 37.82           H
ATOM    679  HB3 LEU B 701      45.901  18.266  30.490  1.00 37.82           H
ATOM    680  HB2 LEU B 701      45.369  17.226  29.244  1.00 37.82           H
ATOM    681  HG  LEU B 701      43.444  18.196  29.217  1.00 42.19           H
ATOM    682 HD11 LEU B 701      43.315  20.660  30.494  1.00 42.06           H
ATOM    683 HD12 LEU B 701      43.009  19.090  31.202  1.00 42.06           H
ATOM    684 HD13 LEU B 701      44.594  19.827  31.360  1.00 42.06           H
ATOM    685 HD21 LEU B 701      42.919  20.176  28.027  1.00 43.78           H
ATOM    686 HD22 LEU B 701      44.518  20.860  28.263  1.00 43.78           H
ATOM    687 HD23 LEU B 701      44.289  19.458  27.213  1.00 43.78           H
ATOM    688  N   SER B 702      47.330  21.046  27.966  1.00 34.30           N
ANISOU  688  N   SER B 702     3632   5512   3888    933    332    343       N
ATOM    689  CA  SER B 702      47.999  22.345  28.100  1.00 33.01           C
ANISOU  689  CA  SER B 702     3272   5558   3713    755    274    403       C
ATOM    690  C   SER B 702      47.039  23.522  27.857  1.00 34.95           C
ANISOU  690  C   SER B 702     3579   5654   4047    514    204    325       C
ATOM    691  O   SER B 702      45.988  23.363  27.237  1.00 33.56           O
ANISOU  691  O   SER B 702     3549   5276   3927    506    212    240       O
ATOM    692  CB  SER B 702      49.234  22.374  27.170  1.00 34.44           C
ANISOU  692  CB  SER B 702     3299   6002   3787    843    322    493       C
ATOM    693  OG  SER B 702      48.900  22.520  25.803  1.00 35.46           O
ANISOU  693  OG  SER B 702     3462   6079   3933    755    322    449       O
ATOM    694  H   SER B 702      46.915  20.852  27.065  1.00 34.30           H
ATOM    695  HA  SER B 702      48.365  22.456  29.123  1.00 33.01           H
ATOM    696  HB3 SER B 702      49.830  21.469  27.297  1.00 34.44           H
ATOM    697  HB2 SER B 702      49.881  23.208  27.442  1.00 34.44           H
ATOM    698  HG  SER B 702      48.546  21.684  25.488  1.00 35.46           H
ATOM    699  N   LYS B 703      47.445  24.693  28.372  1.00 30.98           N
ANISOU  699  N   LYS B 703     2974   5261   3537    321    137    363       N
ATOM    700  CA  LYS B 703      46.684  25.941  28.351  1.00 30.08           C
ANISOU  700  CA  LYS B 703     2927   5030   3473    104     74    312       C
ATOM    701  C   LYS B 703      46.550  26.572  26.951  1.00 32.53           C
ANISOU  701  C   LYS B 703     3256   5327   3775     68     92    289       C
ATOM    702  O   LYS B 703      45.474  27.073  26.630  1.00 32.03           O
ANISOU  702  O   LYS B 703     3320   5074   3774     19     81    212       O
ATOM    703  CB  LYS B 703      47.326  26.897  29.376  1.00 32.80           C
ANISOU  703  CB  LYS B 703     3171   5533   3760    -83      9    380       C
ATOM    704  CG  LYS B 703      46.572  28.215  29.605  1.00 57.03           C
ANISOU  704  CG  LYS B 703     6369   8427   6872   -258    -54    326       C
ATOM    705  CD  LYS B 703      47.109  28.964  30.839  1.00 65.90           C
ANISOU  705  CD  LYS B 703     7429   9684   7925   -409   -115    391       C
ATOM    706  CE  LYS B 703      46.404  30.302  31.119  1.00 79.90           C
ANISOU  706  CE  LYS B 703     9352  11318   9688   -619   -175    355       C
ATOM    707  NZ  LYS B 703      46.722  31.326  30.106  1.00 89.51          N1+
ANISOU  707  NZ  LYS B 703    10575  12608  10827   -796   -199    385       N1+
ATOM    708  H   LYS B 703      48.316  24.725  28.883  1.00 30.98           H
ATOM    709  HA  LYS B 703      45.676  25.710  28.701  1.00 30.08           H
ATOM    710  HB3 LYS B 703      48.358  27.111  29.096  1.00 32.80           H
ATOM    711  HB2 LYS B 703      47.380  26.375  30.331  1.00 32.80           H
ATOM    712  HG3 LYS B 703      45.509  28.009  29.739  1.00 57.03           H
ATOM    713  HG2 LYS B 703      46.655  28.841  28.717  1.00 57.03           H
ATOM    714  HD3 LYS B 703      48.184  29.121  30.736  1.00 65.90           H
ATOM    715  HD2 LYS B 703      46.991  28.325  31.715  1.00 65.90           H
ATOM    716  HE3 LYS B 703      46.715  30.683  32.092  1.00 79.90           H
ATOM    717  HE2 LYS B 703      45.324  30.159  31.163  1.00 79.90           H
```

```
ATOM    718  HZ1 LYS B 703      46.425  31.003  29.195  1.00 89.51           H
ATOM    719  HZ2 LYS B 703      46.241  32.185  30.329  1.00 89.51           H
ATOM    720  HZ3 LYS B 703      47.718  31.491  30.093  1.00 89.51           H
ATOM    721  N   ARG B 704      47.621  26.516  26.137  1.00 28.52           N
ANISOU  721  N   ARG B 704     2612   5042   3182    102    123    365       N
ATOM    722  CA  ARG B 704      47.634  27.034  24.763  1.00 27.06           C
ANISOU  722  CA  ARG B 704     2430   4875   2976     75    144    356       C
ATOM    723  C   ARG B 704      46.850  26.150  23.775  1.00 28.17           C
ANISOU  723  C   ARG B 704     2701   4843   3159    239    203    279       C
ATOM    724  O   ARG B 704      46.326  26.680  22.796  1.00 25.99           O
ANISOU  724  O   ARG B 704     2494   4476   2904    182    201    231       O
ATOM    725  CB  ARG B 704      49.082  27.242  24.278  1.00 27.22           C
ANISOU  725  CB  ARG B 704     2254   5208   2880     68    164    471       C
ATOM    726  CG  ARG B 704      49.864  28.284  25.097  1.00 37.94           C
ANISOU  726  CG  ARG B 704     3518   6720   4178   -191     87    540       C
ATOM    727  CD  ARG B 704      51.270  28.540  24.527  1.00 48.21           C
ANISOU  727  CD  ARG B 704     4604   8366   5347   -228    100    668       C
ATOM    728  NE  ARG B 704      51.935  29.687  25.169  1.00 56.88           N
ANISOU  728  NE  ARG B 704     5604   9652   6357   -495     21    752       N
ATOM    729  CZ  ARG B 704      51.846  30.985  24.815  1.00 72.92           C
ANISOU  729  CZ  ARG B 704     7696  11659   8349   -769    -38    753       C
ATOM    730  NH1 ARG B 704      52.530  31.896  25.520  1.00 62.25           N
ANISOU  730  NH1 ARG B 704     6282  10478   6894  -1030   -114    834       N
ATOM    731  NH2 ARG B 704      51.094  31.397  23.781  1.00 56.93           N1+
ANISOU  731  NH2 ARG B 704     5816   9438   6376   -790    -24    677       N1+
ATOM    732  H   ARG B 704      48.469  26.073  26.460  1.00 28.52           H
ATOM    733  HA  ARG B 704      47.147  28.011  24.769  1.00 27.06           H
ATOM    734  HB3 ARG B 704      49.058  27.578  23.240  1.00 27.22           H
ATOM    735  HB2 ARG B 704      49.618  26.292  24.276  1.00 27.22           H
ATOM    736  HG3 ARG B 704      49.982  27.862  26.096  1.00 37.94           H
ATOM    737  HG2 ARG B 704      49.304  29.212  25.228  1.00 37.94           H
ATOM    738  HD3 ARG B 704      51.289  28.575  23.437  1.00 48.21           H
ATOM    739  HD2 ARG B 704      51.897  27.692  24.805  1.00 48.21           H
ATOM    740  HE  ARG B 704      52.494  29.458  25.978  1.00 56.88           H
ATOM    741 HH12 ARG B 704      52.481  32.874  25.275  1.00 62.25           H
ATOM    742 HH11 ARG B 704      53.107  31.613  26.298  1.00 62.25           H
ATOM    743 HH22 ARG B 704      51.043  32.374  23.535  1.00 56.93           H
ATOM    744 HH21 ARG B 704      50.570  30.723  23.243  1.00 56.93           H
ATOM    745  N   GLN B 705      46.749  24.841  24.063  1.00 25.90           N
ANISOU  745  N   GLN B 705     2473   4495   2874    428    250    265       N
ATOM    746  CA  GLN B 705      45.909  23.884  23.341  1.00 26.04           C
ANISOU  746  CA  GLN B 705     2660   4323   2910    552    298    189       C
ATOM    747  C   GLN B 705      44.406  24.169  23.524  1.00 28.88           C
ANISOU  747  C   GLN B 705     3152   4455   3364    414    245     96       C
ATOM    748  O   GLN B 705      43.654  24.067  22.555  1.00 27.16           O
ANISOU  748  O   GLN B 705     3018   4143   3161    382    248     44       O
ATOM    749  CB  GLN B 705      46.308  22.461  23.784  1.00 27.45           C
ANISOU  749  CB  GLN B 705     2911   4472   3048    767    357    201       C
ATOM    750  CG  GLN B 705      45.443  21.309  23.236  1.00 29.68           C
ANISOU  750  CG  GLN B 705     3423   4529   3327    865    400    120       C
ATOM    751  CD  GLN B 705      45.902  19.944  23.754  1.00 46.71           C
ANISOU  751  CD  GLN B 705     5707   6600   5440   1042    448    125       C
ATOM    752  NE2 GLN B 705      45.743  18.903  22.936  1.00 39.41           N
ANISOU  752  NE2 GLN B 705     5005   5506   4461   1151    501     74       N
ATOM    753  OE1 GLN B 705      46.367  19.821  24.887  1.00 42.33           O
ANISOU  753  OE1 GLN B 705     5076   6120   4888   1078    437    174       O
```

```
ATOM    754  H   GLN B 705      47.218  24.481  24.883  1.00 25.90           H
ATOM    755  HA  GLN B 705      46.133  23.979  22.276  1.00 26.04           H
ATOM    756  HB3 GLN B 705      46.290  22.421  24.872  1.00 27.45           H
ATOM    757  HB2 GLN B 705      47.345  22.287  23.492  1.00 27.45           H
ATOM    758  HG3 GLN B 705      45.450  21.318  22.145  1.00 29.68           H
ATOM    759  HG2 GLN B 705      44.408  21.433  23.550  1.00 29.68           H
ATOM    760 HE22 GLN B 705      46.011  17.980  23.244  1.00 39.41           H
ATOM    761 HE21 GLN B 705      45.337  19.019  22.015  1.00 39.41           H
ATOM    762  N   ILE B 706      44.012  24.552  24.751  1.00 25.38           N
ANISOU  762  N   ILE B 706     2714   3955   2973    335    197     88       N
ATOM    763  CA  ILE B 706      42.652  24.965  25.106  1.00 24.59           C
ANISOU  763  CA  ILE B 706     2712   3685   2946    227    152     24       C
ATOM    764  C   ILE B 706      42.258  26.308  24.448  1.00 29.68           C
ANISOU  764  C   ILE B 706     3349   4326   3602     93    119     11       C
ATOM    765  O   ILE B 706      41.130  26.424  23.972  1.00 28.60           O
ANISOU  765  O   ILE B 706     3294   4079   3493     64    111    -39       O
ATOM    766  CB  ILE B 706      42.473  25.035  26.655  1.00 26.98           C
ANISOU  766  CB  ILE B 706     3008   3960   3284    191    116     34       C
ATOM    767  CG1 ILE B 706      42.554  23.624  27.284  1.00 27.71           C
ANISOU  767  CG1 ILE B 706     3141   4026   3362    334    152     42       C
ATOM    768  CG2 ILE B 706      41.194  25.757  27.138  1.00 24.77           C
ANISOU  768  CG2 ILE B 706     2806   3543   3061     90     74    -15       C
ATOM    769  CD1 ILE B 706      42.869  23.639  28.786  1.00 29.49           C
ANISOU  769  CD1 ILE B 706     3310   4298   3596    328    126     81       C
ATOM    770  H   ILE B 706      44.694  24.596  25.495  1.00 25.38           H
ATOM    771  HA  ILE B 706      41.968  24.204  24.725  1.00 24.59           H
ATOM    772  HB  ILE B 706      43.316  25.603  27.049  1.00 26.98           H
ATOM    773 HG13 ILE B 706      43.318  23.024  26.790  1.00 27.71           H
ATOM    774 HG12 ILE B 706      41.615  23.094  27.115  1.00 27.71           H
ATOM    775 HG21 ILE B 706      41.066  25.687  28.217  1.00 24.77           H
ATOM    776 HG22 ILE B 706      41.223  26.820  26.905  1.00 24.77           H
ATOM    777 HG23 ILE B 706      40.303  25.334  26.678  1.00 24.77           H
ATOM    778 HD11 ILE B 706      43.189  22.658  29.131  1.00 29.49           H
ATOM    779 HD12 ILE B 706      43.664  24.345  29.028  1.00 29.49           H
ATOM    780 HD13 ILE B 706      41.989  23.915  29.364  1.00 29.49           H
ATOM    781  N   GLN B 707      43.203  27.265  24.381  1.00 27.93           N
ANISOU  781  N   GLN B 707     3033   4235   3343      8     99     65       N
ATOM    782  CA  GLN B 707      43.052  28.553  23.690  1.00 28.06           C
ANISOU  782  CA  GLN B 707     3067   4249   3347   -122     72     64       C
ATOM    783  C   GLN B 707      42.898  28.436  22.164  1.00 30.59           C
ANISOU  783  C   GLN B 707     3410   4557   3654    -82    105     40       C
ATOM    784  O   GLN B 707      42.119  29.190  21.580  1.00 30.66           O
ANISOU  784  O   GLN B 707     3491   4480   3678   -141     89      7       O
ATOM    785  CB  GLN B 707      44.208  29.497  24.067  1.00 29.81           C
ANISOU  785  CB  GLN B 707     3194   4631   3500   -242     46    138       C
ATOM    786  CG  GLN B 707      44.083  30.051  25.498  1.00 55.79           C
ANISOU  786  CG  GLN B 707     6515   7891   6790   -349     -6    151       C
ATOM    787  CD  GLN B 707      45.276  30.930  25.868  1.00 81.86           C
ANISOU  787  CD  GLN B 707     9747  11354  10002   -514    -42    227       C
ATOM    788  NE2 GLN B 707      45.107  32.251  25.774  1.00 77.34           N
ANISOU  788  NE2 GLN B 707     9292  10698   9394   -674    -80    219       N
ATOM    789  OE1 GLN B 707      46.335  30.426  26.239  1.00 77.39           O
ANISOU  789  OE1 GLN B 707     9029  10996   9380   -502    -36    299       O
ATOM    790  H   GLN B 707      44.103  27.098  24.811  1.00 27.93           H
ATOM    791  HA  GLN B 707      42.133  29.015  24.046  1.00 28.06           H
ATOM    792  HB3 GLN B 707      44.234  30.342  23.376  1.00 29.81           H
```

ATOM 793 HB2 GLN B 707 45.160 28.979 23.944 1.00 29.81 H
ATOM 794 HG3 GLN B 707 44.019 29.236 26.218 1.00 55.79 H
ATOM 795 HG2 GLN B 707 43.158 30.620 25.600 1.00 55.79 H
ATOM 796 HE22 GLN B 707 45.865 32.880 25.989 1.00 77.34 H
ATOM 797 HE21 GLN B 707 44.231 32.634 25.438 1.00 77.34 H
ATOM 798 N ALA B 708 43.608 27.472 21.555 1.00 26.78 N
ANISOU 798 N ALA B 708 2875 4168 3132 35 156 60 N
ATOM 799 CA ALA B 708 43.494 27.133 20.137 1.00 26.61 C
ANISOU 799 CA ALA B 708 2888 4138 3085 92 195 36 C
ATOM 800 C ALA B 708 42.178 26.406 19.798 1.00 28.77 C
ANISOU 800 C ALA B 708 3300 4232 3401 121 194 -42 C
ATOM 801 O ALA B 708 41.677 26.570 18.687 1.00 28.94 O
ANISOU 801 O ALA B 708 3370 4208 3417 89 193 -73 O
ATOM 802 CB ALA B 708 44.715 26.296 19.729 1.00 27.44 C
ANISOU 802 CB ALA B 708 2927 4380 3119 244 260 82 C
ATOM 803 H ALA B 708 44.248 26.907 22.098 1.00 26.78 H
ATOM 804 HA ALA B 708 43.518 28.060 19.562 1.00 26.61 H
ATOM 805 HB1 ALA B 708 44.674 26.020 18.675 1.00 27.44 H
ATOM 806 HB2 ALA B 708 45.638 26.856 19.882 1.00 27.44 H
ATOM 807 HB3 ALA B 708 44.789 25.379 20.314 1.00 27.44 H
ATOM 808 N ALA B 709 41.633 25.640 20.759 1.00 24.06 N
ANISOU 808 N ALA B 709 2760 3547 2833 166 191 -66 N
ATOM 809 CA ALA B 709 40.358 24.935 20.637 1.00 23.53 C
ANISOU 809 CA ALA B 709 2817 3335 2788 157 181 -126 C
ATOM 810 C ALA B 709 39.151 25.878 20.764 1.00 25.39 C
ANISOU 810 C ALA B 709 3057 3526 3065 40 130 -143 C
ATOM 811 O ALA B 709 38.187 25.705 20.019 1.00 22.92 O
ANISOU 811 O ALA B 709 2800 3165 2742 8 121 -175 O
ATOM 812 CB ALA B 709 40.314 23.805 21.672 1.00 23.83 C
ANISOU 812 CB ALA B 709 2914 3306 2835 216 188 -134 C
ATOM 813 H ALA B 709 42.115 25.536 21.641 1.00 24.06 H
ATOM 814 HA ALA B 709 40.310 24.478 19.647 1.00 23.53 H
ATOM 815 HB1 ALA B 709 39.393 23.228 21.596 1.00 23.83 H
ATOM 816 HB2 ALA B 709 41.142 23.111 21.526 1.00 23.83 H
ATOM 817 HB3 ALA B 709 40.387 24.194 22.687 1.00 23.83 H
ATOM 818 N TYR B 710 39.252 26.894 21.644 1.00 21.90 N
ANISOU 818 N TYR B 710 2562 3109 2650 -18 99 -114 N
ATOM 819 CA TYR B 710 38.316 28.020 21.736 1.00 21.66 C
ANISOU 819 CA TYR B 710 2554 3041 2635 -89 64 -115 C
ATOM 820 C TYR B 710 38.259 28.837 20.431 1.00 25.70 C
ANISOU 820 C TYR B 710 3072 3579 3114 -118 69 -114 C
ATOM 821 O TYR B 710 37.167 29.233 20.026 1.00 25.65 O
ANISOU 821 O TYR B 710 3102 3541 3103 -131 58 -127 O
ATOM 822 CB TYR B 710 38.725 28.961 22.890 1.00 22.57 C
ANISOU 822 CB TYR B 710 2654 3168 2755 -139 40 -82 C
ATOM 823 CG TYR B 710 38.320 28.608 24.311 1.00 23.70 C
ANISOU 823 CG TYR B 710 2810 3266 2930 -127 23 -83 C
ATOM 824 CD1 TYR B 710 36.975 28.333 24.644 1.00 25.91 C
ANISOU 824 CD1 TYR B 710 3129 3486 3228 -113 13 -101 C
ATOM 825 CD2 TYR B 710 39.284 28.674 25.337 1.00 24.26 C
ANISOU 825 CD2 TYR B 710 2843 3376 2999 -140 14 -55 C
ATOM 826 CE1 TYR B 710 36.602 28.145 25.991 1.00 25.93 C
ANISOU 826 CE1 TYR B 710 3141 3458 3254 -105 0 -95 C
ATOM 827 CE2 TYR B 710 38.916 28.476 26.677 1.00 25.42 C
ANISOU 827 CE2 TYR B 710 3008 3480 3172 -132 -2 -55 C
ATOM 828 CZ TYR B 710 37.575 28.224 27.007 1.00 30.52 C

ANISOU	828	CZ	TYR B 710	3701	4054	3841	-111	-7	-77	C
ATOM	829	OH	TYR B 710	37.243	28.075	28.319	1.00	27.32		O
ANISOU	829	OH	TYR B 710	3308	3617	3455	-101	-19	-72	O
ATOM	830	H	TYR B 710	40.062	26.942	22.249	1.00	21.90		H
ATOM	831	HA	TYR B 710	37.320	27.614	21.923	1.00	21.66		H
ATOM	832	HB3	TYR B 710	38.264	29.930	22.729	1.00	22.57		H
ATOM	833	HB2	TYR B 710	39.797	29.155	22.849	1.00	22.57		H
ATOM	834	HD1	TYR B 710	36.217	28.300	23.876	1.00	25.91		H
ATOM	835	HD2	TYR B 710	40.309	28.911	25.112	1.00	24.26		H
ATOM	836	HE1	TYR B 710	35.568	27.952	26.242	1.00	25.93		H
ATOM	837	HE2	TYR B 710	39.660	28.545	27.458	1.00	25.42		H
ATOM	838	HH	TYR B 710	36.297	27.968	28.483	1.00	27.32		H
ATOM	839	N	SER B 711	39.429	29.056	19.802	1.00	22.97		N
ANISOU	839	N	SER B 711	2680	3312	2736	-125	89	-89	N
ATOM	840	CA	SER B 711	39.578	29.800	18.548	1.00	23.52		C
ANISOU	840	CA	SER B 711	2753	3416	2767	-155	98	-82	C
ATOM	841	C	SER B 711	38.908	29.110	17.344	1.00	26.79		C
ANISOU	841	C	SER B 711	3206	3800	3173	-111	115	-122	C
ATOM	842	O	SER B 711	38.383	29.813	16.482	1.00	26.15		O
ANISOU	842	O	SER B 711	3155	3706	3075	-139	105	-127	O
ATOM	843	CB	SER B 711	41.071	30.057	18.267	1.00	26.60		C
ANISOU	843	CB	SER B 711	3065	3929	3114	-170	120	-37	C
ATOM	844	OG	SER B 711	41.595	31.010	19.169	1.00	35.49		O
ANISOU	844	OG	SER B 711	4168	5095	4223	-266	91	7	O
ATOM	845	H	SER B 711	40.282	28.700	20.209	1.00	22.97		H
ATOM	846	HA	SER B 711	39.087	30.766	18.676	1.00	23.52		H
ATOM	847	HB3	SER B 711	41.209	30.452	17.260	1.00	26.60		H
ATOM	848	HB2	SER B 711	41.655	29.141	18.328	1.00	26.60		H
ATOM	849	HG	SER B 711	41.694	30.595	20.031	1.00	35.49		H
ATOM	850	N	ILE B 712	38.898	27.764	17.324	1.00	23.43		N
ANISOU	850	N	ILE B 712	2803	3355	2745	-43	138	-149	N
ATOM	851	CA	ILE B 712	38.205	26.956	16.315	1.00	22.85		C
ANISOU	851	CA	ILE B 712	2805	3235	2642	-28	148	-191	C
ATOM	852	C	ILE B 712	36.670	27.037	16.452	1.00	25.61		C
ANISOU	852	C	ILE B 712	3185	3539	3006	-94	104	-206	C
ATOM	853	O	ILE B 712	35.996	27.144	15.429	1.00	24.77		O
ANISOU	853	O	ILE B 712	3100	3444	2867	-126	93	-217	O
ATOM	854	CB	ILE B 712	38.669	25.466	16.336	1.00	25.84		C
ANISOU	854	CB	ILE B 712	3258	3573	2986	58	188	-216	C
ATOM	855	CG1	ILE B 712	40.133	25.346	15.855	1.00	26.77		C
ANISOU	855	CG1	ILE B 712	3336	3778	3057	159	245	-186	C
ATOM	856	CG2	ILE B 712	37.785	24.488	15.525	1.00	26.08		C
ANISOU	856	CG2	ILE B 712	3413	3527	2969	26	182	-264	C
ATOM	857	CD1	ILE B 712	40.795	24.007	16.212	1.00	40.14		C
ANISOU	857	CD1	ILE B 712	5106	5444	4703	295	298	-190	C
ATOM	858	H	ILE B 712	39.359	27.253	18.065	1.00	23.43		H
ATOM	859	HA	ILE B 712	38.460	27.367	15.335	1.00	22.85		H
ATOM	860	HB	ILE B 712	38.642	25.135	17.375	1.00	25.84		H
ATOM	861	HG13	ILE B 712	40.740	26.147	16.276	1.00	26.77		H
ATOM	862	HG12	ILE B 712	40.173	25.491	14.775	1.00	26.77		H
ATOM	863	HG21	ILE B 712	38.216	23.488	15.503	1.00	26.08		H
ATOM	864	HG22	ILE B 712	36.786	24.380	15.947	1.00	26.08		H
ATOM	865	HG23	ILE B 712	37.678	24.815	14.490	1.00	26.08		H
ATOM	866	HD11	ILE B 712	41.877	24.116	16.292	1.00	40.14		H
ATOM	867	HD12	ILE B 712	40.430	23.617	17.162	1.00	40.14		H
ATOM	868	HD13	ILE B 712	40.597	23.255	15.449	1.00	40.14		H

```
ATOM    869  N   LEU B 713      36.149  27.034  17.693  1.00 22.24           N
ANISOU  869  N   LEU B 713     2746   3086   2618   -108     80   -197       N
ATOM    870  CA  LEU B 713      34.719  27.209  17.979  1.00 21.67           C
ANISOU  870  CA  LEU B 713     2673   3014   2547   -152     45   -191       C
ATOM    871  C   LEU B 713      34.213  28.631  17.666  1.00 25.75           C
ANISOU  871  C   LEU B 713     3162   3575   3048   -156     33   -160       C
ATOM    872  O   LEU B 713      33.065  28.776  17.247  1.00 24.68           O
ANISOU  872  O   LEU B 713     3018   3482   2876   -175     15   -149       O
ATOM    873  CB  LEU B 713      34.416  26.856  19.449  1.00 21.07           C
ANISOU  873  CB  LEU B 713     2587   2911   2509   -150     30   -181       C
ATOM    874  CG  LEU B 713      34.640  25.382  19.840  1.00 23.97           C
ANISOU  874  CG  LEU B 713     3007   3222   2878   -147     39   -206       C
ATOM    875  CD1 LEU B 713      34.395  25.193  21.348  1.00 24.00           C
ANISOU  875  CD1 LEU B 713     2993   3211   2915   -156     20   -189       C
ATOM    876  CD2 LEU B 713      33.796  24.401  18.998  1.00 22.68           C
ANISOU  876  CD2 LEU B 713     2927   3034   2658   -204     32   -236       C
ATOM    877  H   LEU B 713      36.760  26.941  18.494  1.00 22.24           H
ATOM    878  HA  LEU B 713      34.165  26.530  17.329  1.00 21.67           H
ATOM    879  HB3 LEU B 713      33.372  27.090  19.655  1.00 21.07           H
ATOM    880  HB2 LEU B 713      35.007  27.503  20.100  1.00 21.07           H
ATOM    881  HG  LEU B 713      35.687  25.147  19.659  1.00 23.97           H
ATOM    882 HD11 LEU B 713      35.066  24.443  21.767  1.00 24.00           H
ATOM    883 HD12 LEU B 713      34.550  26.114  21.911  1.00 24.00           H
ATOM    884 HD13 LEU B 713      33.373  24.872  21.548  1.00 24.00           H
ATOM    885 HD21 LEU B 713      33.393  23.586  19.597  1.00 22.68           H
ATOM    886 HD22 LEU B 713      32.951  24.895  18.517  1.00 22.68           H
ATOM    887 HD23 LEU B 713      34.403  23.947  18.214  1.00 22.68           H
ATOM    888  N   SER B 714      35.088  29.639  17.827  1.00 21.59           N
ANISOU  888  N   SER B 714     2630   3045   2529   -143     44   -139       N
ATOM    889  CA  SER B 714      34.854  31.017  17.399  1.00 21.58           C
ANISOU  889  CA  SER B 714     2653   3054   2494   -138     43   -108       C
ATOM    890  C   SER B 714      34.842  31.182  15.870  1.00 25.55           C
ANISOU  890  C   SER B 714     3158   3594   2958   -145     52   -116       C
ATOM    891  O   SER B 714      34.106  32.033  15.376  1.00 25.50           O
ANISOU  891  O   SER B 714     3168   3611   2910   -126     47    -92       O
ATOM    892  CB  SER B 714      35.892  31.947  18.057  1.00 24.58           C
ANISOU  892  CB  SER B 714     3064   3405   2870   -163     48    -86       C
ATOM    893  OG  SER B 714      35.386  32.447  19.275  1.00 27.48           O
ANISOU  893  OG  SER B 714     3452   3732   3256   -157     36    -74       O
ATOM    894  H   SER B 714      36.001  29.444  18.214  1.00 21.59           H
ATOM    895  HA  SER B 714      33.861  31.315  17.740  1.00 21.58           H
ATOM    896  HB3 SER B 714      36.109  32.810  17.426  1.00 24.58           H
ATOM    897  HB2 SER B 714      36.841  31.440  18.230  1.00 24.58           H
ATOM    898  HG  SER B 714      36.127  32.586  19.874  1.00 27.48           H
ATOM    899  N   GLU B 715      35.612  30.351  15.144  1.00 21.90           N
ANISOU  899  N   GLU B 715     2683   3142   2495   -155     70   -143       N
ATOM    900  CA  GLU B 715      35.633  30.337  13.681  1.00 21.86           C
ANISOU  900  CA  GLU B 715     2690   3169   2449   -159     83   -156       C
ATOM    901  C   GLU B 715      34.382  29.647  13.095  1.00 25.26           C
ANISOU  901  C   GLU B 715     3133   3617   2849   -178     59   -173       C
ATOM    902  O   GLU B 715      33.907  30.083  12.047  1.00 23.62           O
ANISOU  902  O   GLU B 715     2927   3452   2594   -187     53   -165       O
ATOM    903  CB  GLU B 715      36.951  29.691  13.193  1.00 22.97           C
ANISOU  903  CB  GLU B 715     2824   3322   2581   -138    118   -173       C
ATOM    904  CG  GLU B 715      37.277  29.946  11.701  1.00 28.77           C
ANISOU  904  CG  GLU B 715     3568   4097   3266   -137    139   -174       C
```

```
ATOM    905  CD  GLU B 715      37.618  31.401  11.333  1.00 53.21           C
ANISOU  905  CD  GLU B 715     6648   7226   6343   -168    142   -133       C
ATOM    906  OE1 GLU B 715      38.109  32.147  12.211  1.00 45.35           O
ANISOU  906  OE1 GLU B 715     5627   6246   5360   -195    144   -101       O
ATOM    907  OE2 GLU B 715      37.396  31.746  10.152  1.00 45.59           O1-
ANISOU  907  OE2 GLU B 715     5708   6274   5341   -177    139   -128       O1-
ATOM    908  H   GLU B 715      36.209  29.682  15.609  1.00 21.90           H
ATOM    909  HA  GLU B 715      35.617  31.376  13.349  1.00 21.86           H
ATOM    910  HB3 GLU B 715      36.896  28.614  13.348  1.00 22.97           H
ATOM    911  HB2 GLU B 715      37.784  30.020  13.815  1.00 22.97           H
ATOM    912  HG3 GLU B 715      36.453  29.602  11.076  1.00 28.77           H
ATOM    913  HG2 GLU B 715      38.139  29.341  11.420  1.00 28.77           H
ATOM    914  N   VAL B 716      33.836  28.635  13.801  1.00 23.18           N
ANISOU  914  N   VAL B 716     2878   3333   2599   -199     42   -190       N
ATOM    915  CA  VAL B 716      32.542  28.011  13.494  1.00 23.81           C
ANISOU  915  CA  VAL B 716     2966   3451   2630   -262      9   -195       C
ATOM    916  C   VAL B 716      31.371  28.994  13.704  1.00 28.15           C
ANISOU  916  C   VAL B 716     3448   4093   3155   -250    -15   -139       C
ATOM    917  O   VAL B 716      30.485  29.033  12.854  1.00 28.86           O
ANISOU  917  O   VAL B 716     3518   4266   3181   -278    -33   -121       O
ATOM    918  CB  VAL B 716      32.291  26.712  14.323  1.00 27.87           C
ANISOU  918  CB  VAL B 716     3523   3916   3149   -309     -4   -219       C
ATOM    919  CG1 VAL B 716      30.851  26.152  14.259  1.00 28.34           C
ANISOU  919  CG1 VAL B 716     3585   4044   3140   -419    -50   -208       C
ATOM    920  CG2 VAL B 716      33.261  25.596  13.906  1.00 27.12           C
ANISOU  920  CG2 VAL B 716     3527   3729   3048   -283     32   -267       C
ATOM    921  H   VAL B 716      34.298  28.314  14.641  1.00 23.18           H
ATOM    922  HA  VAL B 716      32.553  27.738  12.437  1.00 23.81           H
ATOM    923  HB  VAL B 716      32.498  26.936  15.370  1.00 27.87           H
ATOM    924 HG11 VAL B 716      30.776  25.205  14.793  1.00 28.34           H
ATOM    925 HG12 VAL B 716      30.122  26.821  14.716  1.00 28.34           H
ATOM    926 HG13 VAL B 716      30.541  25.974  13.229  1.00 28.34           H
ATOM    927 HG21 VAL B 716      33.144  24.712  14.534  1.00 27.12           H
ATOM    928 HG22 VAL B 716      33.087  25.292  12.873  1.00 27.12           H
ATOM    929 HG23 VAL B 716      34.298  25.914  13.986  1.00 27.12           H
ATOM    930  N   GLN B 717      31.417  29.798  14.785  1.00 23.39           N
ANISOU  930  N   GLN B 717     2820   3480   2588   -192     -9   -106       N
ATOM    931  CA  GLN B 717      30.484  30.897  15.065  1.00 23.96           C
ANISOU  931  CA  GLN B 717     2857   3624   2624   -127    -13    -44       C
ATOM    932  C   GLN B 717      30.405  31.926  13.920  1.00 27.57           C
ANISOU  932  C   GLN B 717     3329   4119   3027    -83     -1    -19       C
ATOM    933  O   GLN B 717      29.300  32.262  13.493  1.00 26.65           O
ANISOU  933  O   GLN B 717     3165   4119   2841    -52    -13     30       O
ATOM    934  CB  GLN B 717      30.827  31.534  16.438  1.00 25.84           C
ANISOU  934  CB  GLN B 717     3126   3791   2902    -64      4    -28       C
ATOM    935  CG  GLN B 717      30.167  32.897  16.757  1.00 38.18           C
ANISOU  935  CG  GLN B 717     4724   5372   4410     44     22     32       C
ATOM    936  CD  GLN B 717      30.767  33.542  18.007  1.00 49.17           C
ANISOU  936  CD  GLN B 717     6197   6661   5824     83     38     37       C
ATOM    937  NE2 GLN B 717      31.566  34.596  17.821  1.00 34.00           N
ANISOU  937  NE2 GLN B 717     4245   4752   3923     101     32     47       N
ATOM    938  OE1 GLN B 717      30.504  33.107  19.125  1.00 42.30           O
ANISOU  938  OE1 GLN B 717     5426   5704   4943     83     54     34       O
ATOM    939  H   GLN B 717      32.175  29.685  15.444  1.00 23.39           H
ATOM    940  HA  GLN B 717      29.492  30.451  15.153  1.00 23.96           H
ATOM    941  HB3 GLN B 717      31.905  31.660  16.500  1.00 25.84           H
```

ATOM 942 HB2 GLN B 717 30.578 30.823 17.228 1.00 25.84 H
ATOM 943 HG3 GLN B 717 29.093 32.770 16.894 1.00 38.18 H
ATOM 944 HG2 GLN B 717 30.287 33.606 15.938 1.00 38.18 H
ATOM 945 HE22 GLN B 717 31.992 35.059 18.609 1.00 34.00 H
ATOM 946 HE21 GLN B 717 31.753 34.935 16.886 1.00 34.00 H
ATOM 947 N GLN B 718 31.576 32.381 13.437 1.00 25.94 N
ANISOU 947 N GLN B 718 3181 3834 2841 -82 23 -42 N
ATOM 948 CA GLN B 718 31.686 33.332 12.331 1.00 27.31 C
ANISOU 948 CA GLN B 718 3391 4023 2963 -49 38 -19 C
ATOM 949 C GLN B 718 31.228 32.759 10.979 1.00 32.79 C
ANISOU 949 C GLN B 718 4047 4801 3611 -88 23 -29 C
ATOM 950 O GLN B 718 30.558 33.475 10.238 1.00 32.21 O
ANISOU 950 O GLN B 718 3962 4806 3470 -41 20 16 O
ATOM 951 CB GLN B 718 33.119 33.907 12.245 1.00 28.87 C
ANISOU 951 CB GLN B 718 3653 4132 3184 -73 63 -35 C
ATOM 952 CG GLN B 718 33.567 34.818 13.412 1.00 36.30 C
ANISOU 952 CG GLN B 718 4661 4994 4139 -57 71 -15 C
ATOM 953 CD GLN B 718 32.574 35.924 13.777 1.00 55.09 C
ANISOU 953 CD GLN B 718 7121 7359 6450 35 82 38 C
ATOM 954 NE2 GLN B 718 32.633 37.052 13.068 1.00 41.39 N
ANISOU 954 NE2 GLN B 718 5480 5593 4652 53 100 64 N
ATOM 955 OE1 GLN B 718 31.764 35.761 14.688 1.00 54.01 O
ANISOU 955 OE1 GLN B 718 6975 7240 6305 104 79 62 O
ATOM 956 H GLN B 718 32.444 32.065 13.847 1.00 25.94 H
ATOM 957 HA GLN B 718 30.997 34.149 12.546 1.00 27.31 H
ATOM 958 HB3 GLN B 718 33.213 34.482 11.322 1.00 28.87 H
ATOM 959 HB2 GLN B 718 33.833 33.087 12.149 1.00 28.87 H
ATOM 960 HG3 GLN B 718 34.536 35.261 13.182 1.00 36.30 H
ATOM 961 HG2 GLN B 718 33.722 34.221 14.307 1.00 36.30 H
ATOM 962 HE22 GLN B 718 31.996 37.809 13.269 1.00 41.39 H
ATOM 963 HE21 GLN B 718 33.308 37.155 12.324 1.00 41.39 H
ATOM 964 N ALA B 719 31.550 31.483 10.700 1.00 31.21 N
ANISOU 964 N ALA B 719 3843 4585 3431 -166 14 -83 N
ATOM 965 CA ALA B 719 31.125 30.768 9.493 1.00 32.05 C
ANISOU 965 CA ALA B 719 3947 4751 3477 -226 -5 -102 C
ATOM 966 C ALA B 719 29.602 30.548 9.420 1.00 37.63 C
ANISOU 966 C ALA B 719 4586 5594 4117 -262 -48 -58 C
ATOM 967 O ALA B 719 29.029 30.702 8.342 1.00 36.11 O
ANISOU 967 O ALA B 719 4368 5502 3850 -279 -65 -33 O
ATOM 968 CB ALA B 719 31.886 29.437 9.399 1.00 32.60 C
ANISOU 968 CB ALA B 719 4075 4747 3563 -286 2 -168 C
ATOM 969 H ALA B 719 32.119 30.958 11.351 1.00 31.21 H
ATOM 970 HA ALA B 719 31.410 31.376 8.632 1.00 32.05 H
ATOM 971 HB1 ALA B 719 31.610 28.888 8.499 1.00 32.60 H
ATOM 972 HB2 ALA B 719 32.963 29.603 9.360 1.00 32.60 H
ATOM 973 HB3 ALA B 719 31.682 28.797 10.257 1.00 32.60 H
ATOM 974 N VAL B 720 28.975 30.232 10.567 1.00 35.89 N
ANISOU 974 N VAL B 720 4323 5400 3914 -273 -66 -37 N
ATOM 975 CA VAL B 720 27.523 30.092 10.721 1.00 36.73 C
ANISOU 975 CA VAL B 720 4334 5677 3945 -311 -107 25 C
ATOM 976 C VAL B 720 26.779 31.429 10.520 1.00 44.39 C
ANISOU 976 C VAL B 720 5239 6773 4855 -181 -94 111 C
ATOM 977 O VAL B 720 25.759 31.441 9.831 1.00 43.46 O
ANISOU 977 O VAL B 720 5044 6829 4640 -205 -122 164 O
ATOM 978 CB VAL B 720 27.158 29.475 12.111 1.00 40.14 C
ANISOU 978 CB VAL B 720 4733 6111 4406 -343 -121 35 C

```
ATOM    979  CG1 VAL B 720      25.701  29.672  12.587  1.00 39.64           C
ANISOU  979  CG1 VAL B 720     4537   6269   4253   -350   -153    128       C
ATOM    980  CG2 VAL B 720      27.486  27.972  12.147  1.00 39.67           C
ANISOU  980  CG2 VAL B 720     4756   5952   4366   -480   -139    -36       C
ATOM    981  H   VAL B 720      29.522  30.099  11.408  1.00 35.89           H
ATOM    982  HA  VAL B 720      27.178  29.408   9.943  1.00 36.73           H
ATOM    983  HB  VAL B 720      27.795  29.950  12.859  1.00 40.14           H
ATOM    984 HG11 VAL B 720      25.513  29.121  13.508  1.00 39.64           H
ATOM    985 HG12 VAL B 720      25.475  30.717  12.802  1.00 39.64           H
ATOM    986 HG13 VAL B 720      24.989  29.317  11.841  1.00 39.64           H
ATOM    987 HG21 VAL B 720      27.358  27.563  13.149  1.00 39.67           H
ATOM    988 HG22 VAL B 720      26.834  27.412  11.476  1.00 39.67           H
ATOM    989 HG23 VAL B 720      28.513  27.771  11.846  1.00 39.67           H
ATOM    990  N   SER B 721      27.309  32.520  11.104  1.00 43.52           N
ANISOU  990  N   SER B 721     5176   6574   4784    -43    -50    128       N
ATOM    991  CA  SER B 721      26.716  33.860  11.038  1.00 44.74           C
ANISOU  991  CA  SER B 721     5330   6800   4868    115    -22    208       C
ATOM    992  C   SER B 721      26.806  34.524   9.648  1.00 52.24           C
ANISOU  992  C   SER B 721     6318   7762   5768    139    -12    214       C
ATOM    993  O   SER B 721      25.910  35.298   9.311  1.00 51.64           O
ANISOU  993  O   SER B 721     6219   7801   5599    261      2    294       O
ATOM    994  CB  SER B 721      27.294  34.740  12.168  1.00 48.41           C
ANISOU  994  CB  SER B 721     5894   7129   5371    230     20    216       C
ATOM    995  OG  SER B 721      28.598  35.213  11.894  1.00 59.13           O
ANISOU  995  OG  SER B 721     7375   8311   6781    208     44    162       O
ATOM    996  H   SER B 721      28.146  32.427  11.662  1.00 43.52           H
ATOM    997  HA  SER B 721      25.654  33.735  11.255  1.00 44.74           H
ATOM    998  HB3 SER B 721      27.297  34.201  13.116  1.00 48.41           H
ATOM    999  HB2 SER B 721      26.655  35.612  12.312  1.00 48.41           H
ATOM   1000  HG  SER B 721      29.195  34.461  11.846  1.00 59.13           H
ATOM   1001  N   GLN B 722      27.851  34.197   8.863  1.00 52.18           N
ANISOU 1001  N   GLN B 722     6368   7647   5810     42    -13    139       N
ATOM   1002  CA  GLN B 722      28.026  34.666   7.483  1.00 53.29           C
ANISOU 1002  CA  GLN B 722     6542   7802   5902     51     -4    142       C
ATOM   1003  C   GLN B 722      27.181  33.830   6.496  1.00 58.96           C
ANISOU 1003  C   GLN B 722     7172   8685   6544    -37    -49    153       C
ATOM   1004  O   GLN B 722      26.777  34.363   5.463  1.00 59.22           O
ANISOU 1004  O   GLN B 722     7198   8798   6504     -4    -49    186       O
ATOM   1005  CB  GLN B 722      29.549  34.747   7.164  1.00 55.08           C
ANISOU 1005  CB  GLN B 722     6864   7867   6195      3     24     73       C
ATOM   1006  CG  GLN B 722      30.197  33.651   6.278  1.00 77.34           C
ANISOU 1006  CG  GLN B 722     9682  10681   9024   -108     10      8       C
ATOM   1007  CD  GLN B 722      30.190  33.951   4.771  1.00106.19           C
ANISOU 1007  CD  GLN B 722    13350  14389  12608   -107     13     18       C
ATOM   1008  NE2 GLN B 722      30.532  32.951   3.958  1.00 99.93           N
ANISOU 1008  NE2 GLN B 722    12566  13611  11793   -197     -2    -31       N
ATOM   1009  OE1 GLN B 722      29.907  35.065   4.336  1.00104.40           O
ANISOU 1009  OE1 GLN B 722    13148  14181  12337    -23     32     70       O
ATOM   1010  H   GLN B 722      28.564  33.573   9.217  1.00 52.18           H
ATOM   1011  HA  GLN B 722      27.643  35.687   7.430  1.00 53.29           H
ATOM   1012  HB3 GLN B 722      30.100  34.770   8.103  1.00 55.08           H
ATOM   1013  HB2 GLN B 722      29.760  35.732   6.745  1.00 55.08           H
ATOM   1014  HG3 GLN B 722      29.762  32.671   6.478  1.00 77.34           H
ATOM   1015  HG2 GLN B 722      31.247  33.565   6.561  1.00 77.34           H
ATOM   1016 HE22 GLN B 722      30.544  33.095   2.960  1.00 99.93           H
ATOM   1017 HE21 GLN B 722      30.734  32.031   4.331  1.00 99.93           H
```

ATOM 1018 N GLY B 723 26.903 32.559 6.844 1.00 56.76 N
ANISOU 1018 N GLY B 723 6841 8454 6269 -160 -90 128 N
ATOM 1019 CA GLY B 723 26.072 31.630 6.078 1.00 56.78 C
ANISOU 1019 CA GLY B 723 6786 8605 6182 -298 -145 135 C
ATOM 1020 C GLY B 723 26.814 31.058 4.859 1.00 60.94 C
ANISOU 1020 C GLY B 723 7407 9045 6701 -391 -147 60 C
ATOM 1021 O GLY B 723 27.864 31.555 4.457 1.00 60.87 O
ANISOU 1021 O GLY B 723 7480 8895 6752 -327 -101 17 O
ATOM 1022 H GLY B 723 27.282 32.204 7.711 1.00 56.76 H
ATOM 1023 HA3 GLY B 723 25.158 32.126 5.749 1.00 56.78 H
ATOM 1024 HA2 GLY B 723 25.775 30.814 6.738 1.00 56.78 H
ATOM 1025 N SER B 724 26.243 29.992 4.267 1.00 56.22 N
ANISOU 1025 N SER B 724 6809 8539 6015 -554 -201 47 N
ATOM 1026 CA SER B 724 26.718 29.292 3.059 1.00 55.45 C
ANISOU 1026 CA SER B 724 6824 8369 5874 -654 -207 -23 C
ATOM 1027 C SER B 724 28.119 28.640 3.175 1.00 57.36 C
ANISOU 1027 C SER B 724 7212 8376 6205 -629 -154 -120 C
ATOM 1028 O SER B 724 28.678 28.225 2.160 1.00 57.77 O
ANISOU 1028 O SER B 724 7364 8356 6229 -644 -134 -171 O
ATOM 1029 CB SER B 724 26.604 30.214 1.818 1.00 59.05 C
ANISOU 1029 CB SER B 724 7249 8917 6268 -593 -201 15 C
ATOM 1030 OG SER B 724 25.246 30.443 1.502 1.00 67.08 O
ANISOU 1030 OG SER B 724 8128 10181 7177 -614 -250 112 O
ATOM 1031 H SER B 724 25.388 29.625 4.660 1.00 56.22 H
ATOM 1032 HA SER B 724 26.037 28.451 2.913 1.00 55.45 H
ATOM 1033 HB3 SER B 724 27.055 29.753 0.938 1.00 59.05 H
ATOM 1034 HB2 SER B 724 27.113 31.167 1.963 1.00 59.05 H
ATOM 1035 HG SER B 724 24.872 31.017 2.174 1.00 67.08 H
ATOM 1036 N SER B 725 28.649 28.540 4.405 1.00 51.07 N
ANISOU 1036 N SER B 725 6422 7481 5501 -587 -132 -136 N
ATOM 1037 CA SER B 725 29.973 28.006 4.725 1.00 49.89 C
ANISOU 1037 CA SER B 725 6381 7153 5421 -548 -82 -206 C
ATOM 1038 C SER B 725 29.864 26.640 5.428 1.00 50.10 C
ANISOU 1038 C SER B 725 6500 7106 5430 -652 -103 -250 C
ATOM 1039 O SER B 725 30.697 26.319 6.271 1.00 49.60 O
ANISOU 1039 O SER B 725 6488 6922 5435 -596 -67 -283 O
ATOM 1040 CB SER B 725 30.738 29.057 5.562 1.00 53.98 C
ANISOU 1040 CB SER B 725 6846 7617 6047 -418 -39 -185 C
ATOM 1041 OG SER B 725 31.182 30.112 4.735 1.00 59.98 O
ANISOU 1041 OG SER B 725 7570 8413 6808 -338 -16 -149 O
ATOM 1042 H SER B 725 28.123 28.900 5.189 1.00 51.07 H
ATOM 1043 HA SER B 725 30.544 27.817 3.815 1.00 49.89 H
ATOM 1044 HB3 SER B 725 31.626 28.638 6.033 1.00 53.98 H
ATOM 1045 HB2 SER B 725 30.114 29.454 6.360 1.00 53.98 H
ATOM 1046 HG SER B 725 31.813 29.759 4.103 1.00 59.98 H
ATOM 1047 N ASP B 726 28.842 25.847 5.061 1.00 44.45 N
ANISOU 1047 N ASP B 726 5813 6471 4606 -816 -165 -244 N
ATOM 1048 CA ASP B 726 28.533 24.528 5.632 1.00 42.91 C
ANISOU 1048 CA ASP B 726 5735 6208 4362 -957 -195 -277 C
ATOM 1049 C ASP B 726 29.630 23.475 5.386 1.00 41.85 C
ANISOU 1049 C ASP B 726 5824 5859 4217 -929 -145 -363 C
ATOM 1050 O ASP B 726 29.842 22.628 6.253 1.00 40.16 O
ANISOU 1050 O ASP B 726 5690 5534 4036 -921 -128 -388 O
ATOM 1051 CB ASP B 726 27.149 23.993 5.181 1.00 45.13 C
ANISOU 1051 CB ASP B 726 6010 6640 4499 -1175 -278 -243 C
ATOM 1052 CG ASP B 726 25.947 24.933 5.407 1.00 57.70 C

```
ANISOU 1052  CG  ASP B 726     7373   8468   6083  -1193   -325   -140       C
ATOM   1053  OD1 ASP B 726      26.017  25.806   6.302  1.00 59.39           O
ANISOU 1053  OD1 ASP B 726     7496   8678   6391  -1095   -304   -111       O
ATOM   1054  OD2 ASP B 726      24.914  24.683   4.749  1.00 62.64           O1-
ANISOU 1054  OD2 ASP B 726     7910   9296   6597  -1295   -380    -81       O1-
ATOM   1055  H   ASP B 726      28.186  26.187   4.373  1.00 44.45           H
ATOM   1056  HA  ASP B 726      28.486  24.657   6.713  1.00 42.91           H
ATOM   1057  HB3 ASP B 726      26.931  23.062   5.707  1.00 45.13           H
ATOM   1058  HB2 ASP B 726      27.190  23.753   4.116  1.00 45.13           H
ATOM   1059  N   SER B 727      30.335  23.572   4.245  1.00 36.31           N
ANISOU 1059  N   SER B 727     5221   5108   3466   -888   -112   -401       N
ATOM   1060  CA  SER B 727      31.496  22.738   3.920  1.00 35.63           C
ANISOU 1060  CA  SER B 727     5342   4842   3354   -805    -46   -470       C
ATOM   1061  C   SER B 727      32.733  23.096   4.769  1.00 37.42           C
ANISOU 1061  C   SER B 727     5487   5018   3712   -607     25   -462       C
ATOM   1062  O   SER B 727      33.450  22.186   5.183  1.00 35.28           O
ANISOU 1062  O   SER B 727     5351   4619   3437   -531     74   -497       O
ATOM   1063  CB  SER B 727      31.781  22.838   2.407  1.00 39.87           C
ANISOU 1063  CB  SER B 727     5969   5374   3808   -785    -22   -497       C
ATOM   1064  OG  SER B 727      32.814  21.959   2.005  1.00 53.73           O
ANISOU 1064  OG  SER B 727     7916   6973   5525   -660     57   -552       O
ATOM   1065  H   SER B 727      30.098  24.292   3.578  1.00 36.31           H
ATOM   1066  HA  SER B 727      31.232  21.702   4.141  1.00 35.63           H
ATOM   1067  HB3 SER B 727      32.055  23.857   2.129  1.00 39.87           H
ATOM   1068  HB2 SER B 727      30.885  22.585   1.839  1.00 39.87           H
ATOM   1069  HG  SER B 727      33.632  22.217   2.438  1.00 53.73           H
ATOM   1070  N   GLN B 728      32.938  24.399   5.045  1.00 34.27           N
ANISOU 1070  N   GLN B 728     4884   4723   3412   -527     30   -411       N
ATOM   1071  CA  GLN B 728      34.003  24.910   5.916  1.00 34.20           C
ANISOU 1071  CA  GLN B 728     4792   4692   3512   -384     83   -393       C
ATOM   1072  C   GLN B 728      33.781  24.499   7.383  1.00 36.43           C
ANISOU 1072  C   GLN B 728     5065   4925   3850   -392     71   -390       C
ATOM   1073  O   GLN B 728      34.744  24.131   8.049  1.00 34.83           O
ANISOU 1073  O   GLN B 728     4898   4653   3681   -292    120   -401       O
ATOM   1074  CB  GLN B 728      34.094  26.446   5.770  1.00 35.41           C
ANISOU 1074  CB  GLN B 728     4777   4947   3730   -337     82   -339       C
ATOM   1075  CG  GLN B 728      35.274  27.106   6.524  1.00 44.13           C
ANISOU 1075  CG  GLN B 728     5814   6036   4917   -228    131   -319       C
ATOM   1076  CD  GLN B 728      35.108  28.617   6.722  1.00 63.63           C
ANISOU 1076  CD  GLN B 728     8166   8570   7441   -213    122   -265       C
ATOM   1077  NE2 GLN B 728      34.589  29.331   5.721  1.00 51.94           N
ANISOU 1077  NE2 GLN B 728     6659   7152   5925   -243     96   -240       N
ATOM   1078  OE1 GLN B 728      35.454  29.140   7.778  1.00 59.38           O
ANISOU 1078  OE1 GLN B 728     7577   8023   6963   -174    139   -242       O
ATOM   1079  H   GLN B 728      32.290  25.086   4.689  1.00 34.27           H
ATOM   1080  HA  GLN B 728      34.947  24.475   5.581  1.00 34.20           H
ATOM   1081  HB3 GLN B 728      33.162  26.883   6.123  1.00 35.41           H
ATOM   1082  HB2 GLN B 728      34.164  26.703   4.712  1.00 35.41           H
ATOM   1083  HG3 GLN B 728      36.209  26.914   5.998  1.00 44.13           H
ATOM   1084  HG2 GLN B 728      35.394  26.676   7.517  1.00 44.13           H
ATOM   1085 HE22 GLN B 728      34.471  30.328   5.826  1.00 51.94           H
ATOM   1086 HE21 GLN B 728      34.327  28.885   4.855  1.00 51.94           H
ATOM   1087  N   ILE B 729      32.520  24.543   7.844  1.00 33.80           N
ANISOU 1087  N   ILE B 729     4677   4650   3514   -505      8   -365       N
ATOM   1088  CA  ILE B 729      32.085  24.138   9.183  1.00 33.92           C
ANISOU 1088  CA  ILE B 729     4674   4639   3574   -534    -12   -354       C
```

ATOM 1089 C ILE B 729 32.327 22.640 9.479 1.00 37.11 C
ANISOU 1089 C ILE B 729 5276 4896 3927 -551 11 -407 C
ATOM 1090 O ILE B 729 32.657 22.305 10.616 1.00 35.84 O
ANISOU 1090 O ILE B 729 5123 4668 3825 -472 40 -409 O
ATOM 1091 CB ILE B 729 30.590 24.523 9.404 1.00 37.34 C
ANISOU 1091 CB ILE B 729 5000 5206 3982 -655 -81 -304 C
ATOM 1092 CG1 ILE B 729 30.450 26.056 9.555 1.00 37.89 C
ANISOU 1092 CG1 ILE B 729 4897 5390 4111 -566 -80 -242 C
ATOM 1093 CG2 ILE B 729 29.875 23.822 10.576 1.00 37.45 C
ANISOU 1093 CG2 ILE B 729 5030 5194 4005 -726 -107 -296 C
ATOM 1094 CD1 ILE B 729 29.049 26.595 9.231 1.00 48.00 C
ANISOU 1094 CD1 ILE B 729 6075 6844 5318 -631 -130 -183 C
ATOM 1095 H ILE B 729 31.789 24.880 7.230 1.00 33.80 H
ATOM 1096 HA ILE B 729 32.685 24.702 9.899 1.00 33.92 H
ATOM 1097 HB ILE B 729 30.052 24.238 8.499 1.00 37.34 H
ATOM 1098 HG13 ILE B 729 31.155 26.563 8.898 1.00 37.89 H
ATOM 1099 HG12 ILE B 729 30.733 26.357 10.565 1.00 37.89 H
ATOM 1100 HG21 ILE B 729 28.849 24.172 10.666 1.00 37.45 H
ATOM 1101 HG22 ILE B 729 29.814 22.741 10.447 1.00 37.45 H
ATOM 1102 HG23 ILE B 729 30.381 24.026 11.519 1.00 37.45 H
ATOM 1103 HD11 ILE B 729 29.036 27.682 9.281 1.00 48.00 H
ATOM 1104 HD12 ILE B 729 28.732 26.318 8.228 1.00 48.00 H
ATOM 1105 HD13 ILE B 729 28.299 26.226 9.928 1.00 48.00 H
ATOM 1106 N LEU B 730 32.221 21.785 8.446 1.00 35.09 N
ANISOU 1106 N LEU B 730 5200 4583 3551 -642 1 -448 N
ATOM 1107 CA LEU B 730 32.585 20.364 8.488 1.00 35.52 C
ANISOU 1107 CA LEU B 730 5515 4466 3516 -651 31 -502 C
ATOM 1108 C LEU B 730 34.102 20.150 8.655 1.00 36.59 C
ANISOU 1108 C LEU B 730 5697 4515 3691 -424 123 -516 C
ATOM 1109 O LEU B 730 34.497 19.266 9.415 1.00 34.92 O
ANISOU 1109 O LEU B 730 5592 4199 3478 -364 154 -527 O
ATOM 1110 CB LEU B 730 32.052 19.656 7.216 1.00 36.36 C
ANISOU 1110 CB LEU B 730 5821 4526 3467 -776 10 -542 C
ATOM 1111 CG LEU B 730 30.791 18.798 7.449 1.00 42.56 C
ANISOU 1111 CG LEU B 730 6789 5257 4123 -1019 -57 -559 C
ATOM 1112 CD1 LEU B 730 29.621 19.593 8.069 1.00 43.60 C
ANISOU 1112 CD1 LEU B 730 6704 5563 4297 -1175 -140 -495 C
ATOM 1113 CD2 LEU B 730 30.381 18.061 6.156 1.00 45.01 C
ANISOU 1113 CD2 LEU B 730 7286 5542 4272 -1161 -85 -594 C
ATOM 1114 H LEU B 730 31.932 22.139 7.544 1.00 35.09 H
ATOM 1115 HA LEU B 730 32.110 19.924 9.367 1.00 35.52 H
ATOM 1116 HB3 LEU B 730 32.823 19.000 6.807 1.00 36.36 H
ATOM 1117 HB2 LEU B 730 31.857 20.385 6.428 1.00 36.36 H
ATOM 1118 HG LEU B 730 31.067 18.026 8.169 1.00 42.56 H
ATOM 1119 HD11 LEU B 730 28.659 19.352 7.617 1.00 43.60 H
ATOM 1120 HD12 LEU B 730 29.536 19.378 9.134 1.00 43.60 H
ATOM 1121 HD13 LEU B 730 29.758 20.668 7.975 1.00 43.60 H
ATOM 1122 HD21 LEU B 730 30.305 16.988 6.329 1.00 45.01 H
ATOM 1123 HD22 LEU B 730 29.419 18.396 5.769 1.00 45.01 H
ATOM 1124 HD23 LEU B 730 31.107 18.206 5.355 1.00 45.01 H
ATOM 1125 N ASP B 731 34.916 20.979 7.976 1.00 33.35 N
ANISOU 1125 N ASP B 731 5192 4171 3309 -300 168 -504 N
ATOM 1126 CA ASP B 731 36.379 20.993 8.096 1.00 33.51 C
ANISOU 1126 CA ASP B 731 5199 4181 3353 -88 254 -494 C
ATOM 1127 C ASP B 731 36.841 21.462 9.486 1.00 34.74 C
ANISOU 1127 C ASP B 731 5187 4386 3627 -18 259 -450 C

```
ATOM   1128  O   ASP B 731      37.742  20.843  10.047  1.00 35.76           O
ANISOU 1128  O   ASP B 731     5367   4472   3747    121    317   -444       O
ATOM   1129  CB  ASP B 731      37.088  21.824   6.994  1.00 36.00           C
ANISOU 1129  CB  ASP B 731     5418   4596   3665    -17    287   -476       C
ATOM   1130  CG  ASP B 731      36.739  21.473   5.536  1.00 58.44           C
ANISOU 1130  CG  ASP B 731     8446   7383   6376    -33    305   -519       C
ATOM   1131  OD1 ASP B 731      36.356  20.311   5.268  1.00 61.53           O
ANISOU 1131  OD1 ASP B 731     9093   7630   6656    -24    329   -565       O
ATOM   1132  OD2 ASP B 731      37.001  22.345   4.679  1.00 64.59           O1-
ANISOU 1132  OD2 ASP B 731     9138   8252   7153    -54    297   -507       O1-
ATOM   1133  H   ASP B 731      34.517  21.665   7.349  1.00 33.35           H
ATOM   1134  HA  ASP B 731      36.714  19.959   7.990  1.00 33.51           H
ATOM   1135  HB3 ASP B 731      38.168  21.710   7.097  1.00 36.00           H
ATOM   1136  HB2 ASP B 731      36.871  22.883   7.142  1.00 36.00           H
ATOM   1137  N   LEU B 732      36.192  22.509  10.027  1.00 27.93           N
ANISOU 1137  N   LEU B 732     4138   3617   2858   -106    202   -416       N
ATOM   1138  CA  LEU B 732      36.432  23.062  11.364  1.00 27.42           C
ANISOU 1138  CA  LEU B 732     3929   3594   2895    -68    197   -377       C
ATOM   1139  C   LEU B 732      36.023  22.109  12.501  1.00 32.34           C
ANISOU 1139  C   LEU B 732     4641   4126   3521    -92    184   -391       C
ATOM   1140  O   LEU B 732      36.684  22.111  13.539  1.00 31.20           O
ANISOU 1140  O   LEU B 732     4449   3980   3427     -6    208   -370       O
ATOM   1141  CB  LEU B 732      35.684  24.403  11.511  1.00 26.55           C
ANISOU 1141  CB  LEU B 732     3657   3578   2853   -147    144   -341       C
ATOM   1142  CG  LEU B 732      36.246  25.560  10.660  1.00 30.16           C
ANISOU 1142  CG  LEU B 732     4024   4119   3316   -124    156   -315       C
ATOM   1143  CD1 LEU B 732      35.228  26.709  10.563  1.00 30.19           C
ANISOU 1143  CD1 LEU B 732     3939   4185   3346   -196    106   -284       C
ATOM   1144  CD2 LEU B 732      37.611  26.050  11.182  1.00 29.47           C
ANISOU 1144  CD2 LEU B 732     3852   4078   3266    -33    200   -281       C
ATOM   1145  H   LEU B 732      35.476  22.970   9.481  1.00 27.93           H
ATOM   1146  HA  LEU B 732      37.504  23.241  11.459  1.00 27.42           H
ATOM   1147  HB3 LEU B 732      35.690  24.716  12.556  1.00 26.55           H
ATOM   1148  HB2 LEU B 732      34.636  24.243  11.256  1.00 26.55           H
ATOM   1149  HG  LEU B 732      36.410  25.199   9.646  1.00 30.16           H
ATOM   1150 HD11 LEU B 732      35.696  27.635  10.234  1.00 30.19           H
ATOM   1151 HD12 LEU B 732      34.445  26.478   9.841  1.00 30.19           H
ATOM   1152 HD13 LEU B 732      34.747  26.900  11.522  1.00 30.19           H
ATOM   1153 HD21 LEU B 732      37.568  27.081  11.525  1.00 29.47           H
ATOM   1154 HD22 LEU B 732      37.973  25.458  12.023  1.00 29.47           H
ATOM   1155 HD23 LEU B 732      38.365  25.998  10.398  1.00 29.47           H
ATOM   1156  N   SER B 733      34.972  21.298  12.281  1.00 28.47           N
ANISOU 1156  N   SER B 733     4281   3571   2965   -225    143   -421       N
ATOM   1157  CA  SER B 733      34.522  20.251  13.200  1.00 27.44           C
ANISOU 1157  CA  SER B 733     4265   3347   2815   -279    127   -434       C
ATOM   1158  C   SER B 733      35.573  19.138  13.356  1.00 30.70           C
ANISOU 1158  C   SER B 733     4865   3632   3169   -129    202   -458       C
ATOM   1159  O   SER B 733      35.890  18.772  14.486  1.00 31.27           O
ANISOU 1159  O   SER B 733     4932   3669   3279    -55    221   -443       O
ATOM   1160  CB  SER B 733      33.156  19.706  12.732  1.00 29.06           C
ANISOU 1160  CB  SER B 733     4576   3535   2932   -487     63   -452       C
ATOM   1161  OG  SER B 733      32.662  18.699  13.596  1.00 34.21           O
ANISOU 1161  OG  SER B 733     5347   4098   3552   -569     43   -458       O
ATOM   1162  H   SER B 733      34.459  21.379  11.413  1.00 28.47           H
ATOM   1163  HA  SER B 733      34.382  20.713  14.179  1.00 27.44           H
ATOM   1164  HB3 SER B 733      33.228  19.291  11.728  1.00 29.06           H
```

ATOM 1165 HB2 SER B 733 32.420 20.509 12.686 1.00 29.06 H
ATOM 1166 HG SER B 733 33.213 17.918 13.500 1.00 34.21 H
ATOM 1167 N ASN B 734 36.124 18.664 12.224 1.00 28.09 N
ANISOU 1167 N ASN B 734 4690 3243 2738 -57 251 -488 N
ATOM 1168 CA ASN B 734 37.215 17.687 12.172 1.00 28.20 C
ANISOU 1168 CA ASN B 734 4898 3148 2669 136 340 -501 C
ATOM 1169 C ASN B 734 38.524 18.227 12.786 1.00 31.94 C
ANISOU 1169 C ASN B 734 5181 3736 3217 343 398 -445 C
ATOM 1170 O ASN B 734 39.235 17.469 13.443 1.00 31.81 O
ANISOU 1170 O ASN B 734 5257 3668 3162 502 458 -431 O
ATOM 1171 CB ASN B 734 37.419 17.251 10.702 1.00 30.78 C
ANISOU 1171 CB ASN B 734 5445 3394 2855 179 384 -541 C
ATOM 1172 CG ASN B 734 38.375 16.063 10.539 1.00 55.40 C
ANISOU 1172 CG ASN B 734 8794 6397 5860 416 489 -547 C
ATOM 1173 ND2 ASN B 734 39.384 16.205 9.677 1.00 45.93 N
ANISOU 1173 ND2 ASN B 734 7524 5292 4637 622 567 -517 N
ATOM 1174 OD1 ASN B 734 38.204 15.029 11.180 1.00 54.44 O
ANISOU 1174 OD1 ASN B 734 8915 6110 5660 427 506 -569 O
ATOM 1175 H ASN B 734 35.805 19.020 11.333 1.00 28.09 H
ATOM 1176 HA ASN B 734 36.918 16.818 12.762 1.00 28.20 H
ATOM 1177 HB3 ASN B 734 37.758 18.094 10.097 1.00 30.78 H
ATOM 1178 HB2 ASN B 734 36.462 16.944 10.279 1.00 30.78 H
ATOM 1179 HD22 ASN B 734 40.038 15.448 9.541 1.00 45.93 H
ATOM 1180 HD21 ASN B 734 39.500 17.061 9.155 1.00 45.93 H
ATOM 1181 N ARG B 735 38.797 19.526 12.578 1.00 28.68 N
ANISOU 1181 N ARG B 735 4512 3487 2898 327 377 -407 N
ATOM 1182 CA ARG B 735 39.972 20.244 13.074 1.00 27.84 C
ANISOU 1182 CA ARG B 735 4198 3526 2854 453 411 -344 C
ATOM 1183 C ARG B 735 39.989 20.397 14.606 1.00 27.94 C
ANISOU 1183 C ARG B 735 4134 3538 2942 440 384 -320 C
ATOM 1184 O ARG B 735 41.070 20.393 15.192 1.00 27.34 O
ANISOU 1184 O ARG B 735 3999 3525 2863 583 430 -275 O
ATOM 1185 CB ARG B 735 40.029 21.600 12.346 1.00 30.83 C
ANISOU 1185 CB ARG B 735 4377 4041 3295 368 376 -319 C
ATOM 1186 CG ARG B 735 41.376 22.345 12.401 1.00 45.29 C
ANISOU 1186 CG ARG B 735 6048 6034 5125 481 425 -256 C
ATOM 1187 CD ARG B 735 41.405 23.579 11.473 1.00 47.33 C
ANISOU 1187 CD ARG B 735 6212 6381 5392 404 407 -246 C
ATOM 1188 NE ARG B 735 41.359 23.205 10.043 1.00 49.93 N
ANISOU 1188 NE ARG B 735 6676 6667 5628 452 446 -281 N
ATOM 1189 CZ ARG B 735 41.107 23.998 8.985 1.00 61.86 C
ANISOU 1189 CZ ARG B 735 8154 8223 7126 388 433 -285 C
ATOM 1190 NH1 ARG B 735 40.921 25.321 9.110 1.00 48.51 N
ANISOU 1190 NH1 ARG B 735 6312 6615 5505 281 387 -254 N
ATOM 1191 NH2 ARG B 735 41.032 23.445 7.767 1.00 52.19 N1+
ANISOU 1191 NH2 ARG B 735 7071 6953 5807 435 470 -319 N1+
ATOM 1192 H ARG B 735 38.159 20.070 12.012 1.00 28.68 H
ATOM 1193 HA ARG B 735 40.846 19.660 12.782 1.00 27.84 H
ATOM 1194 HB3 ARG B 735 39.239 22.253 12.716 1.00 30.83 H
ATOM 1195 HB2 ARG B 735 39.803 21.416 11.299 1.00 30.83 H
ATOM 1196 HG3 ARG B 735 42.137 21.640 12.063 1.00 45.29 H
ATOM 1197 HG2 ARG B 735 41.666 22.619 13.415 1.00 45.29 H
ATOM 1198 HD3 ARG B 735 42.236 24.244 11.708 1.00 47.33 H
ATOM 1199 HD2 ARG B 735 40.492 24.148 11.652 1.00 47.33 H
ATOM 1200 HE ARG B 735 41.504 22.222 9.861 1.00 49.93 H
ATOM 1201 HH12 ARG B 735 40.727 25.895 8.302 1.00 48.51 H

ATOM 1202 HH11 ARG B 735 40.976 25.753 10.020 1.00 48.51 H
ATOM 1203 HH22 ARG B 735 40.834 24.015 6.957 1.00 52.19 H
ATOM 1204 HH21 ARG B 735 41.158 22.451 7.647 1.00 52.19 H
ATOM 1205 N PHE B 736 38.796 20.473 15.223 1.00 23.24 N
ANISOU 1205 N PHE B 736 3542 2885 2401 275 313 -343 N
ATOM 1206 CA PHE B 736 38.596 20.474 16.672 1.00 22.52 C
ANISOU 1206 CA PHE B 736 3391 2787 2380 252 284 -323 C
ATOM 1207 C PHE B 736 38.945 19.111 17.302 1.00 27.14 C
ANISOU 1207 C PHE B 736 4160 3254 2899 359 330 -334 C
ATOM 1208 O PHE B 736 39.632 19.082 18.322 1.00 26.87 O
ANISOU 1208 O PHE B 736 4054 3258 2896 455 350 -295 O
ATOM 1209 CB PHE B 736 37.140 20.890 16.974 1.00 24.21 C
ANISOU 1209 CB PHE B 736 3563 2990 2644 68 206 -335 C
ATOM 1210 CG PHE B 736 36.805 21.074 18.442 1.00 25.30 C
ANISOU 1210 CG PHE B 736 3623 3135 2855 45 177 -311 C
ATOM 1211 CD1 PHE B 736 36.366 19.985 19.226 1.00 27.78 C
ANISOU 1211 CD1 PHE B 736 4067 3346 3143 30 176 -324 C
ATOM 1212 CD2 PHE B 736 37.089 22.303 19.071 1.00 26.71 C
ANISOU 1212 CD2 PHE B 736 3623 3413 3114 36 154 -273 C
ATOM 1213 CE1 PHE B 736 36.171 20.147 20.590 1.00 28.61 C
ANISOU 1213 CE1 PHE B 736 4096 3462 3311 16 152 -299 C
ATOM 1214 CE2 PHE B 736 36.876 22.446 20.433 1.00 29.46 C
ANISOU 1214 CE2 PHE B 736 3915 3761 3516 22 131 -252 C
ATOM 1215 CZ PHE B 736 36.421 21.375 21.191 1.00 27.67 C
ANISOU 1215 CZ PHE B 736 3793 3448 3274 17 131 -264 C
ATOM 1216 H PHE B 736 37.955 20.485 14.663 1.00 23.24 H
ATOM 1217 HA PHE B 736 39.262 21.226 17.101 1.00 22.52 H
ATOM 1218 HB3 PHE B 736 36.453 20.156 16.557 1.00 24.21 H
ATOM 1219 HB2 PHE B 736 36.912 21.825 16.463 1.00 24.21 H
ATOM 1220 HD1 PHE B 736 36.187 19.022 18.769 1.00 27.78 H
ATOM 1221 HD2 PHE B 736 37.466 23.138 18.498 1.00 26.71 H
ATOM 1222 HE1 PHE B 736 35.830 19.312 21.183 1.00 28.61 H
ATOM 1223 HE2 PHE B 736 37.074 23.395 20.902 1.00 29.46 H
ATOM 1224 HZ PHE B 736 36.268 21.501 22.252 1.00 27.67 H
ATOM 1225 N TYR B 737 38.488 18.015 16.665 1.00 24.23 N
ANISOU 1225 N TYR B 737 4045 2739 2422 343 349 -381 N
ATOM 1226 CA TYR B 737 38.764 16.634 17.079 1.00 25.41 C
ANISOU 1226 CA TYR B 737 4440 2737 2478 450 401 -394 C
ATOM 1227 C TYR B 737 40.195 16.161 16.774 1.00 31.36 C
ANISOU 1227 C TYR B 737 5237 3522 3156 729 504 -360 C
ATOM 1228 O TYR B 737 40.635 15.188 17.385 1.00 30.97 O
ANISOU 1228 O TYR B 737 5352 3379 3034 877 559 -349 O
ATOM 1229 CB TYR B 737 37.717 15.684 16.458 1.00 27.40 C
ANISOU 1229 CB TYR B 737 4990 2805 2616 304 379 -455 C
ATOM 1230 CG TYR B 737 36.321 15.852 17.030 1.00 30.10 C
ANISOU 1230 CG TYR B 737 5277 3148 3010 48 283 -463 C
ATOM 1231 CD1 TYR B 737 36.104 15.641 18.407 1.00 32.54 C
ANISOU 1231 CD1 TYR B 737 5547 3441 3374 21 261 -441 C
ATOM 1232 CD2 TYR B 737 35.240 16.225 16.204 1.00 30.86 C
ANISOU 1232 CD2 TYR B 737 5332 3295 3097 -155 216 -481 C
ATOM 1233 CE1 TYR B 737 34.830 15.840 18.965 1.00 34.32 C
ANISOU 1233 CE1 TYR B 737 5695 3705 3641 -196 179 -435 C
ATOM 1234 CE2 TYR B 737 33.963 16.430 16.761 1.00 31.63 C
ANISOU 1234 CE2 TYR B 737 5345 3446 3227 -368 134 -468 C
ATOM 1235 CZ TYR B 737 33.763 16.246 18.143 1.00 40.84 C
ANISOU 1235 CZ TYR B 737 6469 4601 4446 -385 118 -443 C

ATOM 1236 OH TYR B 737 32.535 16.452 18.692 1.00 43.95 O
ANISOU 1236 OH TYR B 737 6764 5074 4861 -579 44 -419 O
ATOM 1237 H TYR B 737 37.923 18.125 15.834 1.00 24.23 H
ATOM 1238 HA TYR B 737 38.661 16.597 18.164 1.00 25.41 H
ATOM 1239 HB3 TYR B 737 38.002 14.647 16.643 1.00 27.40 H
ATOM 1240 HB2 TYR B 737 37.696 15.802 15.374 1.00 27.40 H
ATOM 1241 HD1 TYR B 737 36.922 15.334 19.041 1.00 32.54 H
ATOM 1242 HD2 TYR B 737 35.388 16.364 15.144 1.00 30.86 H
ATOM 1243 HE1 TYR B 737 34.679 15.683 20.022 1.00 34.32 H
ATOM 1244 HE2 TYR B 737 33.142 16.732 16.126 1.00 31.63 H
ATOM 1245 HH TYR B 737 31.853 16.647 18.038 1.00 43.95 H
ATOM 1246 N THR B 738 40.905 16.861 15.875 1.00 29.10 N
ANISOU 1246 N THR B 738 4793 3387 2878 810 533 -331 N
ATOM 1247 CA THR B 738 42.333 16.667 15.624 1.00 29.97 C
ANISOU 1247 CA THR B 738 4879 3597 2910 1078 631 -275 C
ATOM 1248 C THR B 738 43.186 17.348 16.715 1.00 36.70 C
ANISOU 1248 C THR B 738 5456 4641 3846 1141 626 -195 C
ATOM 1249 O THR B 738 44.201 16.782 17.120 1.00 38.91 O
ANISOU 1249 O THR B 738 5739 4984 4061 1359 696 -137 O
ATOM 1250 CB THR B 738 42.742 17.248 14.242 1.00 33.82 C
ANISOU 1250 CB THR B 738 5323 4175 3351 1117 663 -273 C
ATOM 1251 CG2 THR B 738 44.240 17.158 13.894 1.00 29.01 C
ANISOU 1251 CG2 THR B 738 4701 3691 2632 1414 776 -205 C
ATOM 1252 OG1 THR B 738 42.030 16.567 13.227 1.00 34.55 O
ANISOU 1252 OG1 THR B 738 5669 4088 3369 1015 650 -351 O
ATOM 1253 H THR B 738 40.465 17.629 15.388 1.00 29.10 H
ATOM 1254 HA THR B 738 42.557 15.598 15.632 1.00 29.97 H
ATOM 1255 HB THR B 738 42.443 18.295 14.187 1.00 33.82 H
ATOM 1256 HG21 THR B 738 44.421 17.481 12.868 1.00 29.01 H
ATOM 1257 HG22 THR B 738 44.848 17.793 14.537 1.00 29.01 H
ATOM 1258 HG23 THR B 738 44.606 16.135 13.987 1.00 29.01 H
ATOM 1259 HG1 THR B 738 41.088 16.723 13.353 1.00 34.55 H
ATOM 1260 N LEU B 739 42.747 18.533 17.177 1.00 32.73 N
ANISOU 1260 N LEU B 739 4733 4230 3473 949 542 -189 N
ATOM 1261 CA LEU B 739 43.433 19.340 18.186 1.00 33.24 C
ANISOU 1261 CA LEU B 739 4553 4465 3614 943 516 -121 C
ATOM 1262 C LEU B 739 43.269 18.775 19.610 1.00 36.77 C
ANISOU 1262 C LEU B 739 5049 4839 4085 966 505 -115 C
ATOM 1263 O LEU B 739 44.251 18.739 20.352 1.00 36.38 O
ANISOU 1263 O LEU B 739 4896 4914 4013 1103 538 -46 O
ATOM 1264 CB LEU B 739 42.920 20.793 18.090 1.00 33.75 C
ANISOU 1264 CB LEU B 739 4448 4595 3782 729 435 -128 C
ATOM 1265 CG LEU B 739 43.836 21.835 18.765 1.00 39.59 C
ANISOU 1265 CG LEU B 739 4947 5551 4543 712 426 -54 C
ATOM 1266 CD1 LEU B 739 45.168 22.016 18.006 1.00 39.30 C
ANISOU 1266 CD1 LEU B 739 4857 5645 4429 799 481 -18 C
ATOM 1267 CD2 LEU B 739 43.096 23.167 18.965 1.00 43.98 C
ANISOU 1267 CD2 LEU B 739 5418 6104 5189 507 347 -69 C
ATOM 1268 H LEU B 739 41.899 18.930 16.797 1.00 32.73 H
ATOM 1269 HA LEU B 739 44.496 19.328 17.942 1.00 33.24 H
ATOM 1270 HB3 LEU B 739 41.916 20.836 18.516 1.00 33.75 H
ATOM 1271 HB2 LEU B 739 42.803 21.080 17.044 1.00 33.75 H
ATOM 1272 HG LEU B 739 44.074 21.474 19.767 1.00 39.59 H
ATOM 1273 HD11 LEU B 739 45.395 23.061 17.795 1.00 39.30 H
ATOM 1274 HD12 LEU B 739 45.999 21.623 18.592 1.00 39.30 H
ATOM 1275 HD13 LEU B 739 45.170 21.490 17.051 1.00 39.30 H

```
ATOM   1276 HD21 LEU B 739      43.427  23.941  18.274  1.00 43.98           H
ATOM   1277 HD22 LEU B 739      42.021  23.063  18.832  1.00 43.98           H
ATOM   1278 HD23 LEU B 739      43.257  23.533  19.977  1.00 43.98           H
ATOM   1279  N   ILE B 740      42.047  18.333  19.945  1.00 33.41           N
ANISOU 1279  N   ILE B 740     4773   4230   3690    833    458   -179       N
ATOM   1280  CA  ILE B 740      41.710  17.658  21.197  1.00 34.63           C
ANISOU 1280  CA  ILE B 740     5004   4293   3861    837    446   -178       C
ATOM   1281  C   ILE B 740      41.278  16.225  20.802  1.00 39.79           C
ANISOU 1281  C   ILE B 740     5984   4734   4399    906    494   -224       C
ATOM   1282  O   ILE B 740      40.158  16.065  20.312  1.00 38.53           O
ANISOU 1282  O   ILE B 740     5966   4443   4232    732    450   -284       O
ATOM   1283  CB  ILE B 740      40.519  18.359  21.933  1.00 38.32           C
ANISOU 1283  CB  ILE B 740     5385   4735   4440    615    354   -202       C
ATOM   1284  CG1 ILE B 740      40.718  19.881  22.139  1.00 39.46           C
ANISOU 1284  CG1 ILE B 740     5281   5041   4672    523    307   -172       C
ATOM   1285  CG2 ILE B 740      40.178  17.673  23.277  1.00 38.94           C
ANISOU 1285  CG2 ILE B 740     5514   4744   4538    621    343   -192       C
ATOM   1286  CD1 ILE B 740      41.886  20.271  23.056  1.00 48.81           C
ANISOU 1286  CD1 ILE B 740     6278   6389   5880    593    313   -101       C
ATOM   1287  H   ILE B 740      41.291  18.414  19.277  1.00 33.41           H
ATOM   1288  HA  ILE B 740      42.559  17.648  21.877  1.00 34.63           H
ATOM   1289  HB  ILE B 740      39.628  18.281  21.308  1.00 38.32           H
ATOM   1290 HG13 ILE B 740      39.800  20.306  22.548  1.00 39.46           H
ATOM   1291 HG12 ILE B 740      40.848  20.373  21.174  1.00 39.46           H
ATOM   1292 HG21 ILE B 740      39.354  18.181  23.780  1.00 38.94           H
ATOM   1293 HG22 ILE B 740      39.878  16.634  23.147  1.00 38.94           H
ATOM   1294 HG23 ILE B 740      41.031  17.676  23.955  1.00 38.94           H
ATOM   1295 HD11 ILE B 740      42.032  21.351  23.049  1.00 48.81           H
ATOM   1296 HD12 ILE B 740      41.702  19.974  24.088  1.00 48.81           H
ATOM   1297 HD13 ILE B 740      42.818  19.811  22.732  1.00 48.81           H
ATOM   1298  N   PRO B 741      42.169  15.214  20.945  1.00 38.85           N
ANISOU 1298  N   PRO B 741     6008   4585   4168   1152    584   -193       N
ATOM   1299  CA  PRO B 741      41.864  13.841  20.500  1.00 39.08           C
ANISOU 1299  CA  PRO B 741     6415   4374   4061   1210    633   -242       C
ATOM   1300  C   PRO B 741      40.802  13.167  21.385  1.00 43.77           C
ANISOU 1300  C   PRO B 741     7182   4783   4666   1037    579   -283       C
ATOM   1301  O   PRO B 741      40.974  13.110  22.603  1.00 41.86           O
ANISOU 1301  O   PRO B 741     6846   4573   4487   1041    559   -250       O
ATOM   1302  CB  PRO B 741      43.223  13.126  20.552  1.00 40.89           C
ANISOU 1302  CB  PRO B 741     6733   4639   4164   1552    750   -179       C
ATOM   1303  CG  PRO B 741      44.004  13.860  21.627  1.00 45.48           C
ANISOU 1303  CG  PRO B 741     6971   5481   4828   1634    744    -93       C
ATOM   1304  CD  PRO B 741      43.527  15.304  21.489  1.00 40.64           C
ANISOU 1304  CD  PRO B 741     6074   5004   4362   1391    650   -103       C
ATOM   1305  HA  PRO B 741      41.514  13.849  19.467  1.00 39.08           H
ATOM   1306  HB3 PRO B 741      43.728  13.242  19.592  1.00 40.89           H
ATOM   1307  HB2 PRO B 741      43.149  12.057  20.752  1.00 40.89           H
ATOM   1308  HG3 PRO B 741      45.084  13.747  21.527  1.00 45.48           H
ATOM   1309  HG2 PRO B 741      43.715  13.469  22.603  1.00 45.48           H
ATOM   1310  HD2 PRO B 741      43.570  15.807  22.456  1.00 40.64           H
ATOM   1311  HD3 PRO B 741      44.154  15.850  20.784  1.00 40.64           H
ATOM   1312  N   HIS B 742      39.722  12.698  20.744  1.00 41.11           N
ANISOU 1312  N   HIS B 742     7081   4274   4265    855    547   -350       N
ATOM   1313  CA  HIS B 742      38.615  11.989  21.384  1.00 40.94           C
ANISOU 1313  CA  HIS B 742     7236   4093   4227    642    489   -385       C
ATOM   1314  C   HIS B 742      38.582  10.514  20.962  1.00 47.48           C
```

```
ANISOU 1314  C   HIS B 742     8522   4657   4861    696    548   -421       C
ATOM   1315  O   HIS B 742     39.059  10.165  19.880  1.00 47.69           O
ANISOU 1315  O   HIS B 742     8739   4612   4769    817    611   -443       O
ATOM   1316  CB  HIS B 742     37.278  12.667  21.027  1.00 40.97           C
ANISOU 1316  CB  HIS B 742     7128   4146   4292    338    390   -418       C
ATOM   1317  CG  HIS B 742     36.968  13.957  21.748  1.00 43.49           C
ANISOU 1317  CG  HIS B 742     7090   4656   4778    239    321   -385       C
ATOM   1318  CD2 HIS B 742     35.949  14.288  22.617  1.00 44.29           C
ANISOU 1318  CD2 HIS B 742     7100   4786   4943     43    247   -378       C
ATOM   1319  ND1 HIS B 742     37.717  15.111  21.608  1.00 44.70           N
ANISOU 1319  ND1 HIS B 742     6965   4991   5027    338    328   -353       N
ATOM   1320  CE1 HIS B 742     37.131  16.058  22.343  1.00 43.53           C
ANISOU 1320  CE1 HIS B 742     6598   4948   4992    212    262   -334       C
ATOM   1321  NE2 HIS B 742     36.050  15.632  22.983  1.00 43.75           N
ANISOU 1321  NE2 HIS B 742     6718   4899   5007     48    215   -346       N
ATOM   1322  H   HIS B 742     39.666  12.780  19.739  1.00 41.11           H
ATOM   1323  HA  HIS B 742     38.729  12.000  22.466  1.00 40.94           H
ATOM   1324  HB3 HIS B 742     36.463  11.984  21.266  1.00 40.97           H
ATOM   1325  HB2 HIS B 742     37.216  12.844  19.952  1.00 40.97           H
ATOM   1326  HD2 HIS B 742     35.146  13.669  22.989  1.00 44.29           H
ATOM   1327  HD1 HIS B 742     38.551  15.226  21.044  1.00 44.70           H
ATOM   1328  HE1 HIS B 742     37.497  17.072  22.405  1.00 43.53           H
ATOM   1329  N   ASP B 743     37.978   9.693  21.835  1.00 45.47           N
ANISOU 1329  N   ASP B 743     8466   4250   4562    604    531   -427       N
ATOM   1330  CA  ASP B 743     37.733   8.270  21.639  1.00 46.48           C
ANISOU 1330  CA  ASP B 743     9080   4092   4488    622    581   -462       C
ATOM   1331  C   ASP B 743     36.233   8.033  21.863  1.00 51.47           C
ANISOU 1331  C   ASP B 743     9876   4603   5076    241    484   -500       C
ATOM   1332  O   ASP B 743     35.793   7.919  23.007  1.00 50.63           O
ANISOU 1332  O   ASP B 743     9761   4472   5006    136    447   -479       O
ATOM   1333  CB  ASP B 743     38.623   7.407  22.571  1.00 48.48           C
ANISOU 1333  CB  ASP B 743     9482   4259   4678    917    673   -416       C
ATOM   1334  CG  ASP B 743     38.482   5.875  22.461  1.00 61.34           C
ANISOU 1334  CG  ASP B 743    11662   5563   6083    957    734   -445       C
ATOM   1335  OD1 ASP B 743     38.030   5.383  21.401  1.00 60.10           O
ANISOU 1335  OD1 ASP B 743    11828   5234   5774    874    747   -500       O
ATOM   1336  OD2 ASP B 743     38.963   5.207  23.403  1.00 69.40           O1-
ANISOU 1336  OD2 ASP B 743    12811   6490   7067   1069    769   -413       O1-
ATOM   1337  H   ASP B 743     37.593  10.083  22.686  1.00 45.47           H
ATOM   1338  HA  ASP B 743     37.967   8.000  20.607  1.00 46.48           H
ATOM   1339  HB3 ASP B 743     38.481   7.718  23.608  1.00 48.48           H
ATOM   1340  HB2 ASP B 743     39.661   7.640  22.349  1.00 48.48           H
ATOM   1341  N   PHE B 744     35.492   7.957  20.748  1.00 48.43           N
ANISOU 1341  N   PHE B 744     9628   4167   4608     25    440   -548       N
ATOM   1342  CA  PHE B 744     34.073   7.597  20.709  1.00 49.00           C
ANISOU 1342  CA  PHE B 744     9826   4178   4614   -368    340   -571       C
ATOM   1343  C   PHE B 744     33.840   6.096  20.435  1.00 56.20           C
ANISOU 1343  C   PHE B 744    11305   4773   5274   -480    363   -615       C
ATOM   1344  O   PHE B 744     32.683   5.677  20.384  1.00 56.65           O
ANISOU 1344  O   PHE B 744    11507   4786   5230   -837    278   -638       O
ATOM   1345  CB  PHE B 744     33.346   8.498  19.687  1.00 50.61           C
ANISOU 1345  CB  PHE B 744     9772   4577   4882   -586    256   -581       C
ATOM   1346  CG  PHE B 744     33.120   9.925  20.158  1.00 52.01           C
ANISOU 1346  CG  PHE B 744     9445   5039   5280   -588    205   -534       C
ATOM   1347  CD1 PHE B 744     32.017  10.228  20.983  1.00 54.69           C
ANISOU 1347  CD1 PHE B 744     9606   5493   5682   -823    119   -500       C
```

```
ATOM   1348  CD2 PHE B 744      34.084  10.926  19.918  1.00 54.20           C
ANISOU 1348  CD2 PHE B 744     9442   5469   5680   -362    244   -520       C
ATOM   1349  CE1 PHE B 744      31.856  11.509  21.493  1.00 55.24           C
ANISOU 1349  CE1 PHE B 744     9254   5805   5932   -802     81   -457       C
ATOM   1350  CE2 PHE B 744      33.889  12.209  20.412  1.00 56.78           C
ANISOU 1350  CE2 PHE B 744     9359   6028   6185   -378    197   -480       C
ATOM   1351  CZ  PHE B 744      32.787  12.497  21.204  1.00 54.56           C
ANISOU 1351  CZ  PHE B 744     8935   5836   5961   -582    121   -451       C
ATOM   1352  H   PHE B 744      35.940   8.080  19.852  1.00 48.43           H
ATOM   1353  HA  PHE B 744      33.623   7.783  21.685  1.00 49.00           H
ATOM   1354  HB3 PHE B 744      32.367   8.087  19.442  1.00 50.61           H
ATOM   1355  HB2 PHE B 744      33.898   8.521  18.746  1.00 50.61           H
ATOM   1356  HD1 PHE B 744      31.294   9.466  21.228  1.00 54.69           H
ATOM   1357  HD2 PHE B 744      34.968  10.703  19.340  1.00 54.20           H
ATOM   1358  HE1 PHE B 744      31.007  11.735  22.121  1.00 55.24           H
ATOM   1359  HE2 PHE B 744      34.604  12.986  20.190  1.00 56.78           H
ATOM   1360  HZ  PHE B 744      32.653  13.493  21.600  1.00 54.56           H
ATOM   1361  N   GLY B 745      34.918   5.306  20.275  1.00 54.01           N
ANISOU 1361  N   GLY B 745    11353   4287   4881   -182    477   -619       N
ATOM   1362  CA  GLY B 745      34.856   3.880  19.960  1.00 54.12           C
ANISOU 1362  CA  GLY B 745    11974   3959   4631   -240    518   -661       C
ATOM   1363  C   GLY B 745      34.390   3.694  18.510  1.00 60.51           C
ANISOU 1363  C   GLY B 745    13049   4662   5278   -406    501   -721       C
ATOM   1364  O   GLY B 745      34.908   4.342  17.600  1.00 59.36           O
ANISOU 1364  O   GLY B 745    12841   4574   5140   -181    562   -734       O
ATOM   1365  H   GLY B 745      35.842   5.706  20.369  1.00 54.01           H
ATOM   1366  HA3 GLY B 745      34.195   3.365  20.659  1.00 54.12           H
ATOM   1367  HA2 GLY B 745      35.851   3.449  20.074  1.00 54.12           H
ATOM   1368  N   MET B 746      33.413   2.795  18.310  1.00 59.72           N
ANISOU 1368  N   MET B 746    13249   4421   5021   -818    413   -752       N
ATOM   1369  CA  MET B 746      32.771   2.528  17.017  1.00 60.58           C
ANISOU 1369  CA  MET B 746    13653   4421   4945  -1051    376   -809       C
ATOM   1370  C   MET B 746      31.732   3.594  16.601  1.00 64.98           C
ANISOU 1370  C   MET B 746    13797   5276   5616  -1409    237   -796       C
ATOM   1371  O   MET B 746      31.250   3.532  15.469  1.00 65.11           O
ANISOU 1371  O   MET B 746    14015   5247   5477  -1699    174   -831       O
ATOM   1372  CB  MET B 746      32.132   1.122  17.050  1.00 63.00           C
ANISOU 1372  CB  MET B 746    14625   4363   4950  -1298    370   -848       C
ATOM   1373  CG  MET B 746      33.098  -0.041  17.349  1.00 66.86           C
ANISOU 1373  CG  MET B 746    15618   4515   5269   -922    521   -863       C
ATOM   1374  SD  MET B 746      34.364  -0.376  16.089  1.00 70.95           S
ANISOU 1374  SD  MET B 746    16348   4928   5681   -474    669   -902       S
ATOM   1375  CE  MET B 746      35.794   0.507  16.771  1.00 67.72           C
ANISOU 1375  CE  MET B 746    15522   4716   5493    105    793   -827       C
ATOM   1376  H   MET B 746      33.043   2.285  19.098  1.00 59.72           H
ATOM   1377  HA  MET B 746      33.538   2.532  16.241  1.00 60.58           H
ATOM   1378  HB3 MET B 746      31.646   0.920  16.095  1.00 63.00           H
ATOM   1379  HB2 MET B 746      31.332   1.108  17.793  1.00 63.00           H
ATOM   1380  HG3 MET B 746      32.508  -0.953  17.443  1.00 66.86           H
ATOM   1381  HG2 MET B 746      33.581   0.091  18.317  1.00 66.86           H
ATOM   1382  HE1 MET B 746      36.678   0.305  16.167  1.00 67.72           H
ATOM   1383  HE2 MET B 746      35.625   1.582  16.772  1.00 67.72           H
ATOM   1384  HE3 MET B 746      35.994   0.183  17.792  1.00 67.72           H
ATOM   1385  N   LYS B 747      31.401   4.542  17.496  1.00 60.96           N
ANISOU 1385  N   LYS B 747    12728   5073   5362  -1378    193   -740       N
ATOM   1386  CA  LYS B 747      30.455   5.633  17.250  1.00 60.75           C
```

```
ANISOU 1386  CA  LYS B 747    12262   5362   5460  -1646     76   -708       C
ATOM   1387  C   LYS B 747     31.157   6.825  16.574  1.00 65.31           C
ANISOU 1387  C   LYS B 747    12461   6149   6204  -1412    104   -705       C
ATOM   1388  O   LYS B 747     32.334   7.074  16.837  1.00 65.42           O
ANISOU 1388  O   LYS B 747    12374   6162   6322  -1039    201   -701       O
ATOM   1389  CB  LYS B 747     29.838   6.091  18.591  1.00 62.59           C
ANISOU 1389  CB  LYS B 747    12144   5787   5851  -1745     16   -642       C
ATOM   1390  CG  LYS B 747     29.123   4.970  19.372  1.00 68.57           C
ANISOU 1390  CG  LYS B 747    13132   6457   6463  -2109    -59   -620       C
ATOM   1391  CD  LYS B 747     28.471   5.406  20.701  1.00 76.03           C
ANISOU 1391  CD  LYS B 747    13778   7540   7569  -2076    -75   -558       C
ATOM   1392  CE  LYS B 747     29.433   5.609  21.894  1.00 77.62           C
ANISOU 1392  CE  LYS B 747    14072   7579   7841  -1697     36   -564       C
ATOM   1393  NZ  LYS B 747     30.122   6.913  21.881  1.00 80.52           N1+
ANISOU 1393  NZ  LYS B 747    13967   8169   8457  -1393     71   -535       N1+
ATOM   1394  H   LYS B 747     31.858   4.550  18.397  1.00 60.96           H
ATOM   1395  HA  LYS B 747     29.653   5.278  16.600  1.00 60.75           H
ATOM   1396  HB3 LYS B 747     29.126   6.895  18.401  1.00 62.59           H
ATOM   1397  HB2 LYS B 747     30.618   6.528  19.207  1.00 62.59           H
ATOM   1398  HG3 LYS B 747     29.813   4.150  19.576  1.00 68.57           H
ATOM   1399  HG2 LYS B 747     28.349   4.549  18.730  1.00 68.57           H
ATOM   1400  HD3 LYS B 747     27.743   4.644  20.982  1.00 76.03           H
ATOM   1401  HD2 LYS B 747     27.882   6.310  20.537  1.00 76.03           H
ATOM   1402  HE3 LYS B 747     30.168   4.804  21.944  1.00 77.62           H
ATOM   1403  HE2 LYS B 747     28.861   5.568  22.821  1.00 77.62           H
ATOM   1404  HZ1 LYS B 747     29.444   7.660  21.867  1.00 80.52           H
ATOM   1405  HZ2 LYS B 747     30.699   7.012  22.708  1.00 80.52           H
ATOM   1406  HZ3 LYS B 747     30.713   6.973  21.065  1.00 80.52           H
ATOM   1407  N   LYS B 748     30.394   7.574  15.761  1.00 60.77           N
ANISOU 1407  N   LYS B 748    11654   5783   5652  -1642     16   -695       N
ATOM   1408  CA  LYS B 748     30.819   8.850  15.182  1.00 59.92           C
ANISOU 1408  CA  LYS B 748    11180   5888   5701  -1464     30   -686       C
ATOM   1409  C   LYS B 748     30.771   9.973  16.246  1.00 62.94           C
ANISOU 1409  C   LYS B 748    11083   6514   6319  -1368     11   -624       C
ATOM   1410  O   LYS B 748     29.775  10.043  16.970  1.00 62.81           O
ANISOU 1410  O   LYS B 748    10929   6608   6328  -1587    -65   -581       O
ATOM   1411  CB  LYS B 748     29.879   9.210  14.012  1.00 62.36           C
ANISOU 1411  CB  LYS B 748    11429   6333   5934  -1737    -56   -692       C
ATOM   1412  CG  LYS B 748     30.085   8.336  12.763  1.00 71.31           C
ANISOU 1412  CG  LYS B 748    12994   7251   6852  -1780    -27   -759       C
ATOM   1413  CD  LYS B 748     29.313   8.864  11.543  1.00 82.34           C
ANISOU 1413  CD  LYS B 748    14229   8831   8226  -1946    -95   -758       C
ATOM   1414  CE  LYS B 748     29.573   8.033  10.275  1.00 97.89           C
ANISOU 1414  CE  LYS B 748    16659  10579   9954  -2043    -80   -826       C
ATOM   1415  NZ  LYS B 748     28.875   8.590   9.102  1.00108.02           N1+
ANISOU 1415  NZ  LYS B 748    17772  12032  11240  -2094   -115   -830       N1+
ATOM   1416  H   LYS B 748     29.432   7.314  15.599  1.00 60.77           H
ATOM   1417  HA  LYS B 748     31.817   8.703  14.776  1.00 59.92           H
ATOM   1418  HB3 LYS B 748     30.076  10.244  13.721  1.00 62.36           H
ATOM   1419  HB2 LYS B 748     28.835   9.183  14.329  1.00 62.36           H
ATOM   1420  HG3 LYS B 748     29.779   7.311  12.977  1.00 71.31           H
ATOM   1421  HG2 LYS B 748     31.148   8.294  12.522  1.00 71.31           H
ATOM   1422  HD3 LYS B 748     29.592   9.904  11.367  1.00 82.34           H
ATOM   1423  HD2 LYS B 748     28.246   8.868  11.772  1.00 82.34           H
ATOM   1424  HE3 LYS B 748     29.246   7.004  10.424  1.00 97.89           H
ATOM   1425  HE2 LYS B 748     30.641   8.003  10.058  1.00 97.89           H
```

ATOM 1426 HZ1 LYS B 748 29.195 9.533 8.933 1.00108.02 H
ATOM 1427 HZ2 LYS B 748 29.070 8.020 8.291 1.00108.02 H
ATOM 1428 HZ3 LYS B 748 27.880 8.599 9.273 1.00108.02 H
ATOM 1429 N PRO B 749 31.802 10.855 16.305 1.00 57.52 N
ANISOU 1429 N PRO B 749 10146 5920 5791 -1055 77 -614 N
ATOM 1430 CA PRO B 749 31.738 12.103 17.099 1.00 56.67 C
ANISOU 1430 CA PRO B 749 9617 6027 5887 -980 56 -558 C
ATOM 1431 C PRO B 749 30.572 13.039 16.687 1.00 57.84 C
ANISOU 1431 C PRO B 749 9457 6423 6097 -1174 -33 -523 C
ATOM 1432 O PRO B 749 30.224 13.062 15.504 1.00 56.02 O
ANISOU 1432 O PRO B 749 9274 6224 5787 -1279 -59 -543 O
ATOM 1433 CB PRO B 749 33.090 12.788 16.809 1.00 58.57 C
ANISOU 1433 CB PRO B 749 9726 6298 6229 -641 145 -558 C
ATOM 1434 CG PRO B 749 34.015 11.690 16.320 1.00 63.19 C
ANISOU 1434 CG PRO B 749 10673 6673 6662 -494 227 -604 C
ATOM 1435 CD PRO B 749 33.078 10.736 15.598 1.00 58.90 C
ANISOU 1435 CD PRO B 749 10413 6021 5947 -770 173 -645 C
ATOM 1436 HA PRO B 749 31.683 11.800 18.144 1.00 56.67 H
ATOM 1437 HB3 PRO B 749 33.494 13.299 17.684 1.00 58.57 H
ATOM 1438 HB2 PRO B 749 32.991 13.537 16.020 1.00 58.57 H
ATOM 1439 HG3 PRO B 749 34.455 11.176 17.172 1.00 63.19 H
ATOM 1440 HG2 PRO B 749 34.830 12.055 15.695 1.00 63.19 H
ATOM 1441 HD2 PRO B 749 32.938 11.035 14.558 1.00 58.90 H
ATOM 1442 HD3 PRO B 749 33.497 9.729 15.624 1.00 58.90 H
ATOM 1443 N PRO B 750 30.011 13.814 17.645 1.00 52.99 N
ANISOU 1443 N PRO B 750 8532 5990 5612 -1201 -74 -466 N
ATOM 1444 CA PRO B 750 28.932 14.774 17.355 1.00 52.07 C
ANISOU 1444 CA PRO B 750 8137 6117 5529 -1346 -147 -420 C
ATOM 1445 C PRO B 750 29.433 15.975 16.530 1.00 53.40 C
ANISOU 1445 C PRO B 750 8133 6384 5771 -1195 -122 -426 C
ATOM 1446 O PRO B 750 30.348 16.676 16.963 1.00 53.24 O
ANISOU 1446 O PRO B 750 8013 6352 5864 -959 -62 -431 O
ATOM 1447 CB PRO B 750 28.413 15.168 18.748 1.00 53.98 C
ANISOU 1447 CB PRO B 750 8136 6494 5879 -1344 -171 -359 C
ATOM 1448 CG PRO B 750 29.597 14.982 19.682 1.00 58.51 C
ANISOU 1448 CG PRO B 750 8777 6924 6533 -1129 -103 -379 C
ATOM 1449 CD PRO B 750 30.357 13.809 19.067 1.00 54.23 C
ANISOU 1449 CD PRO B 750 8596 6140 5869 -1098 -54 -437 C
ATOM 1450 HA PRO B 750 28.129 14.276 16.807 1.00 52.07 H
ATOM 1451 HB3 PRO B 750 27.614 14.484 19.037 1.00 53.98 H
ATOM 1452 HB2 PRO B 750 28.004 16.179 18.794 1.00 53.98 H
ATOM 1453 HG3 PRO B 750 29.309 14.810 20.719 1.00 58.51 H
ATOM 1454 HG2 PRO B 750 30.213 15.880 19.659 1.00 58.51 H
ATOM 1455 HD2 PRO B 750 31.429 13.909 19.234 1.00 54.23 H
ATOM 1456 HD3 PRO B 750 30.020 12.869 19.506 1.00 54.23 H
ATOM 1457 N LEU B 751 28.843 16.153 15.337 1.00 47.49 N
ANISOU 1457 N LEU B 751 7358 5740 4945 -1346 -171 -421 N
ATOM 1458 CA LEU B 751 29.201 17.185 14.362 1.00 46.66 C
ANISOU 1458 CA LEU B 751 7105 5733 4890 -1233 -154 -422 C
ATOM 1459 C LEU B 751 28.938 18.604 14.905 1.00 48.19 C
ANISOU 1459 C LEU B 751 6967 6119 5225 -1133 -163 -361 C
ATOM 1460 O LEU B 751 27.864 18.861 15.452 1.00 47.16 O
ANISOU 1460 O LEU B 751 6691 6130 5096 -1242 -214 -304 O
ATOM 1461 CB LEU B 751 28.428 16.893 13.054 1.00 47.00 C
ANISOU 1461 CB LEU B 751 7222 5843 4792 -1442 -212 -426 C
ATOM 1462 CG LEU B 751 28.848 17.708 11.808 1.00 52.49 C

ANISOU 1462 CG LEU B 751 7900 6558 5488 -1338 -183 -452 C
ATOM 1463 CD1 LEU B 751 30.315 17.463 11.391 1.00 53.21 C
ANISOU 1463 CD1 LEU B 751 8221 6433 5564 -1150 -95 -519 C
ATOM 1464 CD2 LEU B 751 27.869 17.465 10.638 1.00 55.52 C
ANISOU 1464 CD2 LEU B 751 8352 7026 5718 -1577 -253 -448 C
ATOM 1465 H LEU B 751 28.104 15.525 15.057 1.00 47.49 H
ATOM 1466 HA LEU B 751 30.271 17.078 14.178 1.00 46.66 H
ATOM 1467 HB3 LEU B 751 27.364 17.051 13.243 1.00 47.00 H
ATOM 1468 HB2 LEU B 751 28.528 15.834 12.810 1.00 47.00 H
ATOM 1469 HG LEU B 751 28.760 18.762 12.070 1.00 52.49 H
ATOM 1470 HD11 LEU B 751 30.411 17.123 10.360 1.00 53.21 H
ATOM 1471 HD12 LEU B 751 30.889 18.385 11.468 1.00 53.21 H
ATOM 1472 HD13 LEU B 751 30.807 16.715 12.012 1.00 53.21 H
ATOM 1473 HD21 LEU B 751 27.560 18.409 10.192 1.00 55.52 H
ATOM 1474 HD22 LEU B 751 28.303 16.861 9.842 1.00 55.52 H
ATOM 1475 HD23 LEU B 751 26.962 16.953 10.960 1.00 55.52 H
ATOM 1476 N LEU B 752 29.940 19.485 14.752 1.00 43.94 N
ANISOU 1476 N LEU B 752 6322 5586 4788 -926 -111 -368 N
ATOM 1477 CA LEU B 752 29.949 20.847 15.289 1.00 43.54 C
ANISOU 1477 CA LEU B 752 6015 5679 4849 -830 -114 -316 C
ATOM 1478 C LEU B 752 29.378 21.833 14.253 1.00 48.46 C
ANISOU 1478 C LEU B 752 6513 6454 5447 -857 -141 -286 C
ATOM 1479 O LEU B 752 30.094 22.717 13.784 1.00 48.58 O
ANISOU 1479 O LEU B 752 6459 6482 5518 -727 -107 -287 O
ATOM 1480 CB LEU B 752 31.382 21.211 15.758 1.00 43.40 C
ANISOU 1480 CB LEU B 752 5956 5593 4941 -624 -52 -327 C
ATOM 1481 CG LEU B 752 32.032 20.206 16.740 1.00 48.16 C
ANISOU 1481 CG LEU B 752 6699 6047 5555 -552 -11 -355 C
ATOM 1482 CD1 LEU B 752 33.476 20.614 17.085 1.00 47.99 C
ANISOU 1482 CD1 LEU B 752 6599 6017 5617 -366 44 -350 C
ATOM 1483 CD2 LEU B 752 31.196 19.979 18.010 1.00 50.75 C
ANISOU 1483 CD2 LEU B 752 7031 6366 5886 -642 -43 -334 C
ATOM 1484 H LEU B 752 30.791 19.193 14.289 1.00 43.94 H
ATOM 1485 HA LEU B 752 29.294 20.902 16.155 1.00 43.54 H
ATOM 1486 HB3 LEU B 752 31.361 22.198 16.223 1.00 43.40 H
ATOM 1487 HB2 LEU B 752 32.032 21.309 14.888 1.00 43.40 H
ATOM 1488 HG LEU B 752 32.106 19.241 16.238 1.00 48.16 H
ATOM 1489 HD11 LEU B 752 34.162 19.793 16.878 1.00 47.99 H
ATOM 1490 HD12 LEU B 752 33.813 21.473 16.504 1.00 47.99 H
ATOM 1491 HD13 LEU B 752 33.592 20.878 18.136 1.00 47.99 H
ATOM 1492 HD21 LEU B 752 31.785 19.497 18.790 1.00 50.75 H
ATOM 1493 HD22 LEU B 752 30.810 20.917 18.410 1.00 50.75 H
ATOM 1494 HD23 LEU B 752 30.351 19.320 17.808 1.00 50.75 H
ATOM 1495 N ASN B 753 28.095 21.634 13.909 1.00 46.22 N
ANISOU 1495 N ASN B 753 6202 6296 5064 -1037 -203 -251 N
ATOM 1496 CA ASN B 753 27.375 22.356 12.855 1.00 46.67 C
ANISOU 1496 CA ASN B 753 6142 6523 5068 -1077 -235 -211 C
ATOM 1497 C ASN B 753 26.139 23.121 13.369 1.00 52.71 C
ANISOU 1497 C ASN B 753 6684 7521 5824 -1094 -274 -114 C
ATOM 1498 O ASN B 753 25.330 23.560 12.551 1.00 53.75 O
ANISOU 1498 O ASN B 753 6709 7828 5884 -1131 -304 -63 O
ATOM 1499 CB ASN B 753 27.038 21.383 11.690 1.00 44.92 C
ANISOU 1499 CB ASN B 753 6076 6291 4700 -1270 -276 -241 C
ATOM 1500 CG ASN B 753 25.971 20.314 11.994 1.00 62.41 C
ANISOU 1500 CG ASN B 753 8377 8539 6798 -1516 -339 -223 C
ATOM 1501 ND2 ASN B 753 25.231 19.893 10.966 1.00 55.47 N

```
ANISOU 1501  ND2 ASN B 753     7622   7685   5768  -1724   -389   -234       N
ATOM   1502  OD1 ASN B 753      25.822  19.860  13.127  1.00 56.32           O
ANISOU 1502  OD1 ASN B 753     7571   7770   6057  -1541   -346   -197       O
ATOM   1503  H   ASN B 753      27.588  20.868  14.332  1.00 46.22           H
ATOM   1504  HA  ASN B 753      27.998  23.151  12.452  1.00 46.67           H
ATOM   1505  HB3 ASN B 753      27.946  20.876  11.365  1.00 44.92           H
ATOM   1506  HB2 ASN B 753      26.705  21.965  10.829  1.00 44.92           H
ATOM   1507 HD22 ASN B 753      24.521  19.190  11.111  1.00 55.47           H
ATOM   1508 HD21 ASN B 753      25.368  20.279  10.043  1.00 55.47           H
ATOM   1509  N   ASN B 754      26.007  23.268  14.696  1.00 47.93           N
ANISOU 1509  N   ASN B 754     6008   6927   5276  -1051   -267    -83       N
ATOM   1510  CA  ASN B 754      24.860  23.903  15.346  1.00 46.95           C
ANISOU 1510  CA  ASN B 754     5681   7026   5130  -1033   -290     16       C
ATOM   1511  C   ASN B 754      25.283  24.564  16.669  1.00 48.73           C
ANISOU 1511  C   ASN B 754     5843   7206   5468   -854   -246     33       C
ATOM   1512  O   ASN B 754      26.350  24.255  17.202  1.00 47.36           O
ANISOU 1512  O   ASN B 754     5771   6839   5384   -784   -210    -30       O
ATOM   1513  CB  ASN B 754      23.699  22.883  15.499  1.00 44.72           C
ANISOU 1513  CB  ASN B 754     5371   6897   4725  -1267   -357     69       C
ATOM   1514  CG  ASN B 754      24.009  21.687  16.407  1.00 62.76           C
ANISOU 1514  CG  ASN B 754     7816   9016   7015  -1383   -362     21       C
ATOM   1515  ND2 ASN B 754      24.370  20.542  15.823  1.00 53.24           N
ANISOU 1515  ND2 ASN B 754     6789   7737   5702  -1597   -401    -19       N
ATOM   1516  OD1 ASN B 754      23.921  21.796  17.627  1.00 56.84           O
ANISOU 1516  OD1 ASN B 754     7050   8194   6352  -1285   -332     20       O
ATOM   1517  H   ASN B 754      26.723  22.914  15.314  1.00 47.93           H
ATOM   1518  HA  ASN B 754      24.510  24.717  14.708  1.00 46.95           H
ATOM   1519  HB3 ASN B 754      23.399  22.515  14.517  1.00 44.72           H
ATOM   1520  HB2 ASN B 754      22.820  23.388  15.901  1.00 44.72           H
ATOM   1521 HD22 ASN B 754      24.576  19.734  16.392  1.00 53.24           H
ATOM   1522 HD21 ASN B 754      24.462  20.475  14.818  1.00 53.24           H
ATOM   1523  N   ALA B 755      24.418  25.465  17.166  1.00 44.02           N
ANISOU 1523  N   ALA B 755     5081   6799   4846   -774   -248    125       N
ATOM   1524  CA  ALA B 755      24.627  26.258  18.380  1.00 42.49           C
ANISOU 1524  CA  ALA B 755     4839   6576   4727   -605   -207    150       C
ATOM   1525  C   ALA B 755      24.768  25.432  19.671  1.00 43.40           C
ANISOU 1525  C   ALA B 755     5003   6599   4888   -664   -210    128       C
ATOM   1526  O   ALA B 755      25.570  25.813  20.523  1.00 43.28           O
ANISOU 1526  O   ALA B 755     5040   6437   4967   -551   -173     92       O
ATOM   1527  CB  ALA B 755      23.487  27.278  18.516  1.00 43.10           C
ANISOU 1527  CB  ALA B 755     4751   6895   4729   -499   -203    265       C
ATOM   1528  H   ALA B 755      23.558  25.648  16.670  1.00 44.02           H
ATOM   1529  HA  ALA B 755      25.558  26.813  18.247  1.00 42.49           H
ATOM   1530  HB1 ALA B 755      23.626  27.911  19.393  1.00 43.10           H
ATOM   1531  HB2 ALA B 755      23.443  27.935  17.646  1.00 43.10           H
ATOM   1532  HB3 ALA B 755      22.518  26.786  18.612  1.00 43.10           H
ATOM   1533  N   ASP B 756      24.023  24.317  19.778  1.00 38.28           N
ANISOU 1533  N   ASP B 756     4347   6037   4160   -856   -257    151       N
ATOM   1534  CA  ASP B 756      24.060  23.394  20.922  1.00 38.18           C
ANISOU 1534  CA  ASP B 756     4394   5944   4170   -935   -264    137       C
ATOM   1535  C   ASP B 756      25.396  22.638  21.018  1.00 38.99           C
ANISOU 1535  C   ASP B 756     4689   5767   4357   -914   -235     33       C
ATOM   1536  O   ASP B 756      25.907  22.467  22.124  1.00 36.18           O
ANISOU 1536  O   ASP B 756     4362   5312   4071   -848   -212     19       O
ATOM   1537  CB  ASP B 756      22.887  22.378  20.951  1.00 41.09           C
ANISOU 1537  CB  ASP B 756     4747   6458   4407  -1191   -327    185       C
```

ATOM 1538 CG ASP B 756 21.458 22.943 20.839 1.00 58.70 C
ANISOU 1538 CG ASP B 756 6751 9010 6541 -1218 -354 316 C
ATOM 1539 OD1 ASP B 756 21.256 24.151 21.102 1.00 59.83 O
ANISOU 1539 OD1 ASP B 756 6761 9253 6717 -1001 -313 372 O
ATOM 1540 OD2 ASP B 756 20.553 22.115 20.594 1.00 65.94 O1-
ANISOU 1540 OD2 ASP B 756 7634 10087 7332 -1456 -413 371 O1-
ATOM 1541 H ASP B 756 23.382 24.072 19.037 1.00 38.28 H
ATOM 1542 HA ASP B 756 23.981 24.005 21.824 1.00 38.18 H
ATOM 1543 HB3 ASP B 756 22.928 21.815 21.886 1.00 41.09 H
ATOM 1544 HB2 ASP B 756 23.016 21.654 20.145 1.00 41.09 H
ATOM 1545 N SER B 757 25.946 22.225 19.863 1.00 36.46 N
ANISOU 1545 N SER B 757 4498 5338 4017 -963 -235 -31 N
ATOM 1546 CA SER B 757 27.243 21.555 19.753 1.00 35.19 C
ANISOU 1546 CA SER B 757 4521 4944 3906 -916 -199 -117 C
ATOM 1547 C SER B 757 28.429 22.484 20.078 1.00 35.67 C
ANISOU 1547 C SER B 757 4541 4929 4082 -710 -147 -137 C
ATOM 1548 O SER B 757 29.359 22.042 20.750 1.00 35.53 O
ANISOU 1548 O SER B 757 4601 4779 4119 -644 -117 -171 O
ATOM 1549 CB SER B 757 27.397 20.942 18.348 1.00 39.24 C
ANISOU 1549 CB SER B 757 5175 5392 4342 -1002 -206 -167 C
ATOM 1550 OG SER B 757 26.676 19.734 18.229 1.00 51.59 O
ANISOU 1550 OG SER B 757 6852 6966 5784 -1226 -255 -163 O
ATOM 1551 H SER B 757 25.443 22.369 18.997 1.00 36.46 H
ATOM 1552 HA SER B 757 27.270 20.745 20.484 1.00 35.19 H
ATOM 1553 HB3 SER B 757 28.440 20.694 18.170 1.00 39.24 H
ATOM 1554 HB2 SER B 757 27.092 21.637 17.565 1.00 39.24 H
ATOM 1555 HG SER B 757 26.885 19.341 17.376 1.00 51.59 H
ATOM 1556 N VAL B 758 28.361 23.752 19.630 1.00 30.63 N
ANISOU 1556 N VAL B 758 3791 4381 3466 -618 -138 -110 N
ATOM 1557 CA VAL B 758 29.345 24.798 19.937 1.00 30.40 C
ANISOU 1557 CA VAL B 758 3736 4292 3522 -461 -97 -120 C
ATOM 1558 C VAL B 758 29.334 25.188 21.430 1.00 32.68 C
ANISOU 1558 C VAL B 758 3980 4573 3862 -403 -91 -93 C
ATOM 1559 O VAL B 758 30.409 25.343 22.007 1.00 31.87 O
ANISOU 1559 O VAL B 758 3916 4370 3823 -336 -66 -119 O
ATOM 1560 CB VAL B 758 29.128 26.065 19.055 1.00 34.26 C
ANISOU 1560 CB VAL B 758 4154 4866 3998 -394 -91 -91 C
ATOM 1561 CG1 VAL B 758 29.887 27.336 19.502 1.00 33.59 C
ANISOU 1561 CG1 VAL B 758 4056 4731 3975 -267 -59 -84 C
ATOM 1562 CG2 VAL B 758 29.480 25.766 17.586 1.00 34.44 C
ANISOU 1562 CG2 VAL B 758 4235 4866 3984 -431 -88 -129 C
ATOM 1563 H VAL B 758 27.565 24.040 19.076 1.00 30.63 H
ATOM 1564 HA VAL B 758 30.335 24.393 19.715 1.00 30.40 H
ATOM 1565 HB VAL B 758 28.064 26.308 19.080 1.00 34.26 H
ATOM 1566 HG11 VAL B 758 29.770 28.140 18.774 1.00 33.59 H
ATOM 1567 HG12 VAL B 758 29.521 27.726 20.453 1.00 33.59 H
ATOM 1568 HG13 VAL B 758 30.955 27.143 19.604 1.00 33.59 H
ATOM 1569 HG21 VAL B 758 29.232 26.614 16.947 1.00 34.44 H
ATOM 1570 HG22 VAL B 758 30.545 25.563 17.469 1.00 34.44 H
ATOM 1571 HG23 VAL B 758 28.936 24.903 17.204 1.00 34.44 H
ATOM 1572 N GLN B 759 28.131 25.284 22.026 1.00 27.52 N
ANISOU 1572 N GLN B 759 3240 4048 3168 -433 -113 -33 N
ATOM 1573 CA GLN B 759 27.909 25.512 23.455 1.00 26.65 C
ANISOU 1573 CA GLN B 759 3086 3955 3087 -379 -107 3 C
ATOM 1574 C GLN B 759 28.489 24.392 24.338 1.00 31.20 C
ANISOU 1574 C GLN B 759 3744 4409 3702 -427 -106 -36 C

```
ATOM   1575  O   GLN B 759      29.201  24.698  25.290  1.00 30.01           O
ANISOU 1575  O   GLN B 759     3608   4183   3614   -343    -85    -46       O
ATOM   1576  CB  GLN B 759      26.404  25.763  23.708  1.00 26.95           C
ANISOU 1576  CB  GLN B 759     3005   4189   3044   -410   -129     86       C
ATOM   1577  CG  GLN B 759      25.986  25.808  25.195  1.00 47.56           C
ANISOU 1577  CG  GLN B 759     5561   6841   5668   -305   -109    136       C
ATOM   1578  CD  GLN B 759      24.504  26.113  25.419  1.00 71.89           C
ANISOU 1578  CD  GLN B 759     8513  10145   8657   -344   -127    229       C
ATOM   1579  NE2 GLN B 759      24.063  25.989  26.672  1.00 64.09           N
ANISOU 1579  NE2 GLN B 759     7504   9171   7674   -358   -127    254       N
ATOM   1580  OE1 GLN B 759      23.767  26.456  24.496  1.00 67.11           O
ANISOU 1580  OE1 GLN B 759     7813   9721   7965   -360   -142    288       O
ATOM   1581  H   GLN B 759      27.295  25.162  21.470  1.00 27.52           H
ATOM   1582  HA  GLN B 759      28.439  26.429  23.718  1.00 26.65           H
ATOM   1583  HB3 GLN B 759      25.819  24.991  23.207  1.00 26.95           H
ATOM   1584  HB2 GLN B 759      26.130  26.705  23.230  1.00 26.95           H
ATOM   1585  HG3 GLN B 759      26.578  26.552  25.729  1.00 47.56           H
ATOM   1586  HG2 GLN B 759      26.186  24.849  25.672  1.00 47.56           H
ATOM   1587 HE22 GLN B 759      23.096  26.173  26.893  1.00 64.09           H
ATOM   1588 HE21 GLN B 759      24.702  25.712  27.408  1.00 64.09           H
ATOM   1589  N   ALA B 760      28.192  23.128  23.990  1.00 27.42           N
ANISOU 1589  N   ALA B 760     3340   3907   3171   -566   -130    -56       N
ATOM   1590  CA  ALA B 760      28.643  21.934  24.710  1.00 26.88           C
ANISOU 1590  CA  ALA B 760     3390   3709   3115   -609   -125    -89       C
ATOM   1591  C   ALA B 760      30.172  21.768  24.751  1.00 30.15           C
ANISOU 1591  C   ALA B 760     3882   3979   3596   -487    -84   -141       C
ATOM   1592  O   ALA B 760      30.698  21.343  25.780  1.00 31.53           O
ANISOU 1592  O   ALA B 760     4075   4090   3816   -428    -68   -143       O
ATOM   1593  CB  ALA B 760      27.980  20.696  24.088  1.00 27.43           C
ANISOU 1593  CB  ALA B 760     3582   3752   3087   -786   -154   -106       C
ATOM   1594  H   ALA B 760      27.582  22.963  23.200  1.00 27.42           H
ATOM   1595  HA  ALA B 760      28.294  22.024  25.740  1.00 26.88           H
ATOM   1596  HB1 ALA B 760      28.289  19.783  24.599  1.00 27.43           H
ATOM   1597  HB2 ALA B 760      26.894  20.757  24.159  1.00 27.43           H
ATOM   1598  HB3 ALA B 760      28.238  20.591  23.033  1.00 27.43           H
ATOM   1599  N   LYS B 761      30.854  22.131  23.651  1.00 26.07           N
ANISOU 1599  N   LYS B 761     3396   3434   3077   -446    -66   -173       N
ATOM   1600  CA  LYS B 761      32.313  22.129  23.563  1.00 24.66           C
ANISOU 1600  CA  LYS B 761     3261   3169   2941   -329    -24   -204       C
ATOM   1601  C   LYS B 761      32.966  23.364  24.206  1.00 26.45           C
ANISOU 1601  C   LYS B 761     3381   3431   3236   -233    -14   -181       C
ATOM   1602  O   LYS B 761      34.081  23.231  24.703  1.00 25.97           O
ANISOU 1602  O   LYS B 761     3334   3328   3207   -160     10   -186       O
ATOM   1603  CB  LYS B 761      32.755  21.963  22.097  1.00 25.96           C
ANISOU 1603  CB  LYS B 761     3485   3313   3068   -315     -5   -236       C
ATOM   1604  CG  LYS B 761      32.442  20.594  21.463  1.00 34.36           C
ANISOU 1604  CG  LYS B 761     4718   4296   4042   -402     -7   -270       C
ATOM   1605  CD  LYS B 761      32.874  19.372  22.292  1.00 38.43           C
ANISOU 1605  CD  LYS B 761     5390   4680   4533   -363     21   -290       C
ATOM   1606  CE  LYS B 761      32.765  18.064  21.496  1.00 55.60           C
ANISOU 1606  CE  LYS B 761     7784   6750   6592   -473     16   -324       C
ATOM   1607  NZ  LYS B 761      33.057  16.884  22.329  1.00 67.57           N1+
ANISOU 1607  NZ  LYS B 761     9485   8122   8065   -454     40   -336       N1+
ATOM   1608  H   LYS B 761      30.353  22.461  22.838  1.00 26.07           H
ATOM   1609  HA  LYS B 761      32.689  21.278  24.131  1.00 24.66           H
ATOM   1610  HB3 LYS B 761      33.832  22.115  22.036  1.00 25.96           H
```

ATOM 1611 HB2 LYS B 761 32.305 22.748 21.489 1.00 25.96 H
ATOM 1612 HG3 LYS B 761 32.939 20.560 20.494 1.00 34.36 H
ATOM 1613 HG2 LYS B 761 31.376 20.520 21.258 1.00 34.36 H
ATOM 1614 HD3 LYS B 761 32.240 19.299 23.177 1.00 38.43 H
ATOM 1615 HD2 LYS B 761 33.894 19.513 22.650 1.00 38.43 H
ATOM 1616 HE3 LYS B 761 33.457 18.083 20.653 1.00 55.60 H
ATOM 1617 HE2 LYS B 761 31.759 17.955 21.087 1.00 55.60 H
ATOM 1618 HZ1 LYS B 761 32.414 16.847 23.106 1.00 67.57 H
ATOM 1619 HZ2 LYS B 761 32.956 16.047 21.771 1.00 67.57 H
ATOM 1620 HZ3 LYS B 761 34.004 16.933 22.681 1.00 67.57 H
ATOM 1621 N ALA B 762 32.276 24.518 24.231 1.00 23.50 N
ANISOU 1621 N ALA B 762 2919 3140 2871 -233 -29 -149 N
ATOM 1622 CA ALA B 762 32.730 25.717 24.945 1.00 23.60 C
ANISOU 1622 CA ALA B 762 2881 3162 2922 -164 -22 -128 C
ATOM 1623 C ALA B 762 32.726 25.510 26.470 1.00 26.83 C
ANISOU 1623 C ALA B 762 3286 3547 3362 -147 -26 -113 C
ATOM 1624 O ALA B 762 33.698 25.883 27.123 1.00 26.67 O
ANISOU 1624 O ALA B 762 3262 3502 3370 -106 -19 -110 O
ATOM 1625 CB ALA B 762 31.878 26.929 24.537 1.00 24.45 C
ANISOU 1625 CB ALA B 762 2947 3340 3004 -148 -29 -96 C
ATOM 1626 H ALA B 762 31.367 24.575 23.791 1.00 23.50 H
ATOM 1627 HA ALA B 762 33.758 25.920 24.639 1.00 23.60 H
ATOM 1628 HB1 ALA B 762 32.219 27.836 25.037 1.00 24.45 H
ATOM 1629 HB2 ALA B 762 31.944 27.110 23.464 1.00 24.45 H
ATOM 1630 HB3 ALA B 762 30.826 26.791 24.787 1.00 24.45 H
ATOM 1631 N GLU B 763 31.673 24.844 26.979 1.00 23.38 N
ANISOU 1631 N GLU B 763 2851 3127 2906 -195 -41 -100 N
ATOM 1632 CA GLU B 763 31.528 24.390 28.363 1.00 23.29 C
ANISOU 1632 CA GLU B 763 2840 3095 2914 -190 -45 -84 C
ATOM 1633 C GLU B 763 32.583 23.350 28.783 1.00 27.82 C
ANISOU 1633 C GLU B 763 3482 3578 3512 -168 -30 -112 C
ATOM 1634 O GLU B 763 33.069 23.429 29.909 1.00 27.50 O
ANISOU 1634 O GLU B 763 3429 3517 3502 -122 -26 -102 O
ATOM 1635 CB GLU B 763 30.101 23.841 28.571 1.00 24.77 C
ANISOU 1635 CB GLU B 763 3003 3352 3057 -268 -66 -52 C
ATOM 1636 CG GLU B 763 29.008 24.938 28.559 1.00 38.71 C
ANISOU 1636 CG GLU B 763 4676 5245 4787 -236 -70 2 C
ATOM 1637 CD GLU B 763 27.554 24.431 28.530 1.00 57.85 C
ANISOU 1637 CD GLU B 763 7031 7812 7136 -323 -93 55 C
ATOM 1638 OE1 GLU B 763 27.334 23.201 28.448 1.00 48.60 O
ANISOU 1638 OE1 GLU B 763 5900 6630 5936 -451 -115 46 O
ATOM 1639 OE2 GLU B 763 26.660 25.305 28.586 1.00 47.71 O1-
ANISOU 1639 OE2 GLU B 763 5660 6662 5807 -264 -88 112 O1-
ATOM 1640 H GLU B 763 30.911 24.597 26.361 1.00 23.38 H
ATOM 1641 HA GLU B 763 31.657 25.258 29.011 1.00 23.29 H
ATOM 1642 HB3 GLU B 763 30.044 23.290 29.511 1.00 24.77 H
ATOM 1643 HB2 GLU B 763 29.898 23.113 27.784 1.00 24.77 H
ATOM 1644 HG3 GLU B 763 29.148 25.600 27.705 1.00 38.71 H
ATOM 1645 HG2 GLU B 763 29.134 25.567 29.441 1.00 38.71 H
ATOM 1646 N MET B 764 32.949 22.424 27.875 1.00 26.33 N
ANISOU 1646 N MET B 764 3376 3336 3294 -189 -18 -143 N
ATOM 1647 CA MET B 764 34.013 21.435 28.083 1.00 25.86 C
ANISOU 1647 CA MET B 764 3399 3194 3231 -124 12 -161 C
ATOM 1648 C MET B 764 35.388 22.088 28.320 1.00 25.69 C
ANISOU 1648 C MET B 764 3308 3211 3243 -25 31 -150 C
ATOM 1649 O MET B 764 36.097 21.671 29.234 1.00 23.65 O

```
ANISOU 1649  O   MET B 764     3046   2944   2996     38     43   -134       O
ATOM   1650  CB  MET B 764      34.045  20.444  26.896  1.00 29.19           C
ANISOU 1650  CB  MET B 764     3959   3543   3589   -144     30   -194       C
ATOM   1651  CG  MET B 764      35.148  19.370  26.962  1.00 34.87           C
ANISOU 1651  CG  MET B 764     4774   4191   4283    -15     80   -204       C
ATOM   1652  SD  MET B 764      35.294  18.342  25.476  1.00 41.91           S
ANISOU 1652  SD  MET B 764     5831   5006   5086     18    120   -242       S
ATOM   1653  CE  MET B 764      36.240  19.474  24.423  1.00 38.16           C
ANISOU 1653  CE  MET B 764     5192   4659   4649     40    117   -236       C
ATOM   1654  H   MET B 764      32.493  22.409  26.974  1.00 26.33           H
ATOM   1655  HA  MET B 764      33.763  20.870  28.983  1.00 25.86           H
ATOM   1656  HB3 MET B 764      34.160  20.999  25.969  1.00 29.19           H
ATOM   1657  HB2 MET B 764      33.078  19.947  26.824  1.00 29.19           H
ATOM   1658  HG3 MET B 764      34.968  18.721  27.815  1.00 34.87           H
ATOM   1659  HG2 MET B 764      36.127  19.813  27.130  1.00 34.87           H
ATOM   1660  HE1 MET B 764      36.433  19.009  23.457  1.00 38.16           H
ATOM   1661  HE2 MET B 764      35.689  20.400  24.260  1.00 38.16           H
ATOM   1662  HE3 MET B 764      37.198  19.714  24.883  1.00 38.16           H
ATOM   1663  N   LEU B 765      35.720  23.103  27.505  1.00 21.70           N
ANISOU 1663  N   LEU B 765     2744   2761   2740    -26     31   -150       N
ATOM   1664  CA  LEU B 765      36.982  23.842  27.570  1.00 21.21           C
ANISOU 1664  CA  LEU B 765     2611   2760   2688     23     41   -130       C
ATOM   1665  C   LEU B 765      37.136  24.701  28.838  1.00 25.24           C
ANISOU 1665  C   LEU B 765     3068   3297   3225      8     18   -102       C
ATOM   1666  O   LEU B 765      38.271  24.869  29.281  1.00 25.91           O
ANISOU 1666  O   LEU B 765     3110   3429   3305     40     22    -76       O
ATOM   1667  CB  LEU B 765      37.148  24.695  26.299  1.00 20.42           C
ANISOU 1667  CB  LEU B 765     2480   2706   2572      2     44   -134       C
ATOM   1668  CG  LEU B 765      37.360  23.881  25.006  1.00 23.99           C
ANISOU 1668  CG  LEU B 765     2990   3140   2986     25     71   -161       C
ATOM   1669  CD1 LEU B 765      37.361  24.826  23.799  1.00 23.06           C
ANISOU 1669  CD1 LEU B 765     2836   3070   2856     -7     68   -161       C
ATOM   1670  CD2 LEU B 765      38.622  23.000  25.040  1.00 25.79           C
ANISOU 1670  CD2 LEU B 765     3243   3375   3180    134    117   -152       C
ATOM   1671  H   LEU B 765      35.074  23.393  26.783  1.00 21.70           H
ATOM   1672  HA  LEU B 765      37.787  23.107  27.601  1.00 21.21           H
ATOM   1673  HB3 LEU B 765      37.990  25.377  26.418  1.00 20.42           H
ATOM   1674  HB2 LEU B 765      36.267  25.329  26.186  1.00 20.42           H
ATOM   1675  HG  LEU B 765      36.521  23.203  24.876  1.00 23.99           H
ATOM   1676 HD11 LEU B 765      37.477  24.276  22.868  1.00 23.06           H
ATOM   1677 HD12 LEU B 765      36.428  25.386  23.735  1.00 23.06           H
ATOM   1678 HD13 LEU B 765      38.177  25.544  23.867  1.00 23.06           H
ATOM   1679 HD21 LEU B 765      39.080  22.904  24.058  1.00 25.79           H
ATOM   1680 HD22 LEU B 765      39.378  23.392  25.719  1.00 25.79           H
ATOM   1681 HD23 LEU B 765      38.374  21.991  25.370  1.00 25.79           H
ATOM   1682  N   ASP B 766      36.018  25.175  29.426  1.00 21.13           N
ANISOU 1682  N   ASP B 766     2552   2759   2715    -36     -6    -99       N
ATOM   1683  CA  ASP B 766      35.984  25.841  30.738  1.00 21.33           C
ANISOU 1683  CA  ASP B 766     2569   2786   2751    -44    -24    -77       C
ATOM   1684  C   ASP B 766      36.462  24.912  31.869  1.00 24.85           C
ANISOU 1684  C   ASP B 766     3013   3216   3212    -13    -23    -68       C
ATOM   1685  O   ASP B 766      37.255  25.336  32.710  1.00 23.63           O
ANISOU 1685  O   ASP B 766     2834   3090   3054    -13    -34    -46       O
ATOM   1686  CB  ASP B 766      34.590  26.398  31.137  1.00 22.95           C
ANISOU 1686  CB  ASP B 766     2789   2983   2947    -54    -33    -68       C
ATOM   1687  CG  ASP B 766      33.878  27.346  30.159  1.00 34.63           C
```

ANISOU 1687 CG ASP B 766 4279 4483 4394 -53 -32 -60 C
ATOM 1688 OD1 ASP B 766 34.549 27.970 29.308 1.00 34.54 O
ANISOU 1688 OD1 ASP B 766 4270 4480 4374 -68 -28 -69 O
ATOM 1689 OD2 ASP B 766 32.661 27.535 30.367 1.00 39.86 O1-
ANISOU 1689 OD2 ASP B 766 4948 5166 5032 -26 -31 -36 O1-
ATOM 1690 H ASP B 766 35.122 25.026 28.982 1.00 21.13 H
ATOM 1691 HA ASP B 766 36.683 26.679 30.694 1.00 21.33 H
ATOM 1692 HB3 ASP B 766 34.681 26.937 32.083 1.00 22.95 H
ATOM 1693 HB2 ASP B 766 33.911 25.563 31.322 1.00 22.95 H
ATOM 1694 N ASN B 767 35.974 23.660 31.851 1.00 23.72 N
ANISOU 1694 N ASN B 767 2910 3030 3071 3 -10 -81 N
ATOM 1695 CA ASN B 767 36.293 22.623 32.835 1.00 24.15 C
ANISOU 1695 CA ASN B 767 2993 3054 3129 46 -1 -72 C
ATOM 1696 C ASN B 767 37.718 22.073 32.665 1.00 29.00 C
ANISOU 1696 C ASN B 767 3587 3706 3724 128 24 -56 C
ATOM 1697 O ASN B 767 38.354 21.802 33.682 1.00 27.56 O
ANISOU 1697 O ASN B 767 3374 3557 3540 168 21 -27 O
ATOM 1698 CB ASN B 767 35.224 21.509 32.773 1.00 24.86 C
ANISOU 1698 CB ASN B 767 3168 3073 3204 19 5 -87 C
ATOM 1699 CG ASN B 767 33.890 21.937 33.396 1.00 41.61 C
ANISOU 1699 CG ASN B 767 5272 5209 5331 -50 -19 -74 C
ATOM 1700 ND2 ASN B 767 32.969 22.480 32.601 1.00 35.72 N
ANISOU 1700 ND2 ASN B 767 4516 4492 4563 -112 -29 -78 N
ATOM 1701 OD1 ASN B 767 33.686 21.777 34.595 1.00 36.74 O
ANISOU 1701 OD1 ASN B 767 4641 4593 4728 -41 -27 -54 O
ATOM 1702 H ASN B 767 35.336 23.395 31.113 1.00 23.72 H
ATOM 1703 HA ASN B 767 36.225 23.117 33.805 1.00 24.15 H
ATOM 1704 HB3 ASN B 767 35.567 20.642 33.342 1.00 24.86 H
ATOM 1705 HB2 ASN B 767 35.075 21.158 31.751 1.00 24.86 H
ATOM 1706 HD22 ASN B 767 32.087 22.783 32.987 1.00 35.72 H
ATOM 1707 HD21 ASN B 767 33.150 22.629 31.615 1.00 35.72 H
ATOM 1708 N LEU B 768 38.222 21.973 31.419 1.00 26.19 N
ANISOU 1708 N LEU B 768 3241 3367 3341 163 50 -67 N
ATOM 1709 CA LEU B 768 39.617 21.617 31.131 1.00 25.66 C
ANISOU 1709 CA LEU B 768 3138 3375 3235 268 84 -37 C
ATOM 1710 C LEU B 768 40.621 22.676 31.619 1.00 29.03 C
ANISOU 1710 C LEU B 768 3433 3938 3657 246 61 12 C
ATOM 1711 O LEU B 768 41.737 22.302 31.971 1.00 27.75 O
ANISOU 1711 O LEU B 768 3216 3866 3461 328 77 59 O
ATOM 1712 CB LEU B 768 39.829 21.366 29.620 1.00 25.57 C
ANISOU 1712 CB LEU B 768 3162 3366 3187 310 119 -54 C
ATOM 1713 CG LEU B 768 39.220 20.058 29.068 1.00 30.34 C
ANISOU 1713 CG LEU B 768 3934 3838 3757 339 149 -96 C
ATOM 1714 CD1 LEU B 768 39.391 19.990 27.534 1.00 29.82 C
ANISOU 1714 CD1 LEU B 768 3907 3782 3640 390 186 -110 C
ATOM 1715 CD2 LEU B 768 39.773 18.797 29.771 1.00 33.14 C
ANISOU 1715 CD2 LEU B 768 4397 4124 4070 451 185 -82 C
ATOM 1716 H LEU B 768 37.638 22.194 30.623 1.00 26.19 H
ATOM 1717 HA LEU B 768 39.838 20.701 31.678 1.00 25.66 H
ATOM 1718 HB3 LEU B 768 40.898 21.342 29.403 1.00 25.57 H
ATOM 1719 HB2 LEU B 768 39.439 22.219 29.064 1.00 25.57 H
ATOM 1720 HG LEU B 768 38.148 20.085 29.264 1.00 30.34 H
ATOM 1721 HD11 LEU B 768 38.430 19.848 27.043 1.00 29.82 H
ATOM 1722 HD12 LEU B 768 39.823 20.906 27.130 1.00 29.82 H
ATOM 1723 HD13 LEU B 768 40.039 19.175 27.214 1.00 29.82 H
ATOM 1724 HD21 LEU B 768 40.051 18.008 29.073 1.00 33.14 H

ATOM 1725 HD22 LEU B 768 40.660 19.013 30.366 1.00 33.14 H
ATOM 1726 HD23 LEU B 768 39.025 18.374 30.444 1.00 33.14 H
ATOM 1727 N LEU B 769 40.220 23.959 31.636 1.00 26.79 N
ANISOU 1727 N LEU B 769 3115 3671 3391 132 23 6 N
ATOM 1728 CA LEU B 769 41.059 25.084 32.052 1.00 26.85 C
ANISOU 1728 CA LEU B 769 3045 3781 3375 55 -11 47 C
ATOM 1729 C LEU B 769 41.330 25.093 33.572 1.00 30.85 C
ANISOU 1729 C LEU B 769 3535 4307 3881 57 -33 75 C
ATOM 1730 O LEU B 769 42.454 25.399 33.967 1.00 30.33 O
ANISOU 1730 O LEU B 769 3378 4378 3767 47 -45 130 O
ATOM 1731 CB LEU B 769 40.415 26.395 31.542 1.00 27.06 C
ANISOU 1731 CB LEU B 769 3121 3750 3410 -54 -40 23 C
ATOM 1732 CG LEU B 769 41.338 27.630 31.502 1.00 33.00 C
ANISOU 1732 CG LEU B 769 3848 4572 4119 -140 -52 41 C
ATOM 1733 CD1 LEU B 769 42.515 27.468 30.517 1.00 33.15 C
ANISOU 1733 CD1 LEU B 769 3770 4724 4103 -96 -25 74 C
ATOM 1734 CD2 LEU B 769 40.519 28.894 31.184 1.00 34.35 C
ANISOU 1734 CD2 LEU B 769 4099 4651 4301 -170 -52 5 C
ATOM 1735 H LEU B 769 39.288 24.192 31.322 1.00 26.79 H
ATOM 1736 HA LEU B 769 42.023 24.956 31.559 1.00 26.85 H
ATOM 1737 HB3 LEU B 769 39.537 26.618 32.150 1.00 27.06 H
ATOM 1738 HB2 LEU B 769 40.039 26.243 30.531 1.00 27.06 H
ATOM 1739 HG LEU B 769 41.760 27.771 32.497 1.00 33.00 H
ATOM 1740 HD11 LEU B 769 42.567 28.288 29.800 1.00 33.15 H
ATOM 1741 HD12 LEU B 769 43.464 27.444 31.053 1.00 33.15 H
ATOM 1742 HD13 LEU B 769 42.450 26.548 29.938 1.00 33.15 H
ATOM 1743 HD21 LEU B 769 41.147 29.785 31.191 1.00 34.35 H
ATOM 1744 HD22 LEU B 769 40.044 28.825 30.205 1.00 34.35 H
ATOM 1745 HD23 LEU B 769 39.732 29.047 31.923 1.00 34.35 H
ATOM 1746 N ASP B 770 40.330 24.703 34.384 1.00 26.88 N
ANISOU 1746 N ASP B 770 3106 3689 3417 65 -38 46 N
ATOM 1747 CA ASP B 770 40.471 24.494 35.833 1.00 26.77 C
ANISOU 1747 CA ASP B 770 3090 3678 3405 72 -56 68 C
ATOM 1748 C ASP B 770 41.171 23.167 36.183 1.00 31.21 C
ANISOU 1748 C ASP B 770 3624 4285 3948 193 -25 99 C
ATOM 1749 O ASP B 770 41.891 23.139 37.181 1.00 30.17 O
ANISOU 1749 O ASP B 770 3435 4239 3789 206 -41 144 O
ATOM 1750 CB ASP B 770 39.129 24.610 36.602 1.00 28.21 C
ANISOU 1750 CB ASP B 770 3354 3741 3625 52 -64 36 C
ATOM 1751 CG ASP B 770 38.423 25.978 36.538 1.00 37.10 C
ANISOU 1751 CG ASP B 770 4520 4835 4740 -30 -92 27 C
ATOM 1752 OD1 ASP B 770 39.125 27.008 36.434 1.00 38.74 O
ANISOU 1752 OD1 ASP B 770 4719 5094 4906 -100 -119 48 O
ATOM 1753 OD2 ASP B 770 37.196 25.992 36.776 1.00 40.53 O1-
ANISOU 1753 OD2 ASP B 770 5006 5201 5192 -23 -85 7 O1-
ATOM 1754 H ASP B 770 39.427 24.482 33.988 1.00 26.88 H
ATOM 1755 HA ASP B 770 41.131 25.277 36.212 1.00 26.77 H
ATOM 1756 HB3 ASP B 770 39.295 24.393 37.657 1.00 28.21 H
ATOM 1757 HB2 ASP B 770 38.439 23.854 36.223 1.00 28.21 H
ATOM 1758 N ILE B 771 40.987 22.112 35.366 1.00 29.24 N
ANISOU 1758 N ILE B 771 3434 3978 3699 287 21 79 N
ATOM 1759 CA ILE B 771 41.677 20.819 35.505 1.00 29.48 C
ANISOU 1759 CA ILE B 771 3486 4028 3688 439 66 110 C
ATOM 1760 C ILE B 771 43.185 20.909 35.185 1.00 33.32 C
ANISOU 1760 C ILE B 771 3834 4716 4110 508 79 180 C
ATOM 1761 O ILE B 771 43.977 20.253 35.863 1.00 32.47 O

ANISOU 1761 O   ILE B 771   3671   4709   3958   603   90   240   O
[truncated: 490,337 more chars]
